# Regional Variation of the CD4 and CD8 T Cell Epitopes Conserved in Circulating Dengue Viruses and Shared with Potential Vaccine Candidates

**DOI:** 10.3390/v16050730

**Published:** 2024-05-05

**Authors:** Yadya M. Chawla, Prashant Bajpai, Keshav Saini, Elluri Seetharami Reddy, Ashok Kumar Patel, Kaja Murali-Krishna, Anmol Chandele

**Affiliations:** 1 ICGEB-Emory Vaccine Center, International Centre for Genetic Engineering and Biotechnology, Aruna Asaf Ali Marg, New Delhi 110067, India; yadyachawla@gmail.com (Y.M.C.); prashantbajpai112@gmail.com (P.B.); sainikeshav6@gmail.com (K.S.); rammku.rr@gmail.com (E.S.R.); 2 Kusuma School of Biological Sciences, Indian Institute of Technology Delhi, New Delhi 110016, India; ashok.kumar.patel@bioschool.iitd.ac.in; 3 Department of Pediatrics, Emory University School of Medicine, Emory University, Atlanta, GA 30322, USA; 4 Emory Vaccine Center, Emory University, Atlanta, GA 30317, USA

**Keywords:** dengue, T cell epitopes, vaccines, India

## Abstract

As dengue expands globally and many vaccines are under trials, there is a growing recognition of the need for assessing T cell immunity in addition to assessing the functions of neutralizing antibodies during these endeavors. While several dengue-specific experimentally validated T cell epitopes are known, less is understood about which of these epitopes are conserved among circulating dengue viruses and also shared by potential vaccine candidates. As India emerges as the epicenter of the dengue disease burden and vaccine trials commence in this region, we have here aligned known dengue specific T cell epitopes, reported from other parts of the world with published polyprotein sequences of 107 dengue virus isolates available from India. Of the 1305 CD4 and 584 CD8 epitopes, we found that 24% and 41%, respectively, were conserved universally, whereas 27% and 13% were absent in any viral isolates. With these data, we catalogued epitopes conserved in circulating dengue viruses from India and matched them with each of the six vaccine candidates under consideration (TV003, TDEN, DPIV, CYD-TDV, DENVax and TVDV). Similar analyses with viruses from Thailand, Brazil and Mexico revealed regional overlaps and variations in these patterns. Thus, our study provides detailed and nuanced insights into regional variation that should be considered for itemization of T cell responses during dengue natural infection and vaccine design, testing and evaluation.

## 1. Introduction

Dengue, a mosquito-borne viral disease caused by any of the four dengue virus serotypes (DENV1–4), is an emerging global epidemic, with an estimated 390 million annual infections and 100 million cases of clinical disease [1]. Several vaccines are under consideration [2,3]. While many of the past efforts have focused mainly on neutralizing antibody responses, in the recent past, there has been a growing recognition of the need to evaluate T cell responses, in addition to the study of antibodies, during dengue natural infection and vaccine trials [4,5,6,7,8,9,10,11,12,13,14,15,16,17,18]. Progress in these directions is critically dependent on a detailed understanding of dengue-specific CD8 and CD4 T cell epitopes conserved among circulating dengue viruses and determining which of these are shared by vaccine candidates.

As India emerges as the epicenter of dengue disease burden, contributing nearly a third of the global dengue disease burden, and vaccine trials commence in this region, a detailed understanding of T cell responses to vaccines and natural infection in this region is important [19,20,21,22,23,24,25]. However, many knowledge gaps exist. We do not know which of the dengue-specific experimentally validated CD8 and CD4 T cell epitopes reported from other parts of the world remain conserved in the circulating dengue viruses from India. While many vaccines are under consideration, we do not know which of the epitopes conserved in these circulating dengue viruses from India are also shared with each of the vaccine candidates.

Hence, in this study we first focused on addressing this question by compiling known dengue-specific CD8 and CD4 T cell epitopes worldwide [10,11,13,15,20,26,27,28,29,30,31,32,33,34,35,36,37,38,39,40,41,42,43,44,45,46,47,48,49,50,51,52,53,54,55,56,57,58,59,60,61,62,63,64,65,66,67,68,69,70,71,72,73,74,75,76,77] and aligning these with 107 published full-length polyprotein sequences of dengue virus isolates available from India. Of the experimentally validated globally reported dengue-specific 1305 CD4 and 584 CD8 epitopes compiled, we found 24% and 41%, respectively, were conserved universally, whereas 17% and 13% were absent in any viral isolates from India. Concurrently, by aligning these epitopes with the six dengue vaccines under consideration (TV003, TDEN, DPIV, CYD-TDV, DENVax and TVDV), we catalogued epitopes conserved in circulating dengue viruses from India and also matched with each of the six vaccine candidates. 

Conversely, we performed similar analysis using published polyprotein sequences of circulating dengue viruses from Thailand, Brazil and Mexico to determine how different the conserved epitopes are from the circulating dengue viruses from distinct geographical regions of the world. We found that these epitopes that we found to be conserved among the circulating dengue viruses from India and also shared by the potential vaccine candidates are not necessarily uniform between distinct geographical regions, thus highlighting the importance of selecting and choosing region-specific customized peptide pools for evaluating T cell responses to natural dengue infection as well as vaccine design, testing and evaluation.

## 2. Materials and Methods

### 2.1. Compilation of Experimentally Characterized Dengue-Specific CD8 and CD4 T Cell Epitopes

We first compiled the list of the experimentally validated dengue-specific CD4 and CD8 epitopes worldwide from the literature. For this, epitope metadata was curated from the Immune Epitope Database (IEDB, https://www.iedb.org/, accessed on 12 February 2022). The query was performed using the following parameters—epitope as a linear peptide, epitope source organism as dengue virus, host as human, assay as positive for T cell, MHC restriction as any, and disease as any. This approach resulted in identification of the dengue-specific T cell epitopes that are experimentally validated within any of the T cell assays. A list of 1364 CD4 and 832 CD8 dengue-specific T cell epitopes were obtained. Sequences whose protein source could not be annotated or the ones that did not contain serotype information were excluded. The epitopes were then aligned with each of the 10 dengue protein sequences to accurately annotate the protein distribution for each one of them. After the dengue serotypes and the protein annotation, a total of 1305 dengue-specific CD4 T cell epitopes and 584 dengue-specific CD8 T cell epitopes were filtered and subsequently used for further analysis.

### 2.2. Retrieval of Published Full-Length Dengue Virus Sequences

To determine the CD4 and CD8 T cell epitopes present in the circulating dengue viruses from India, published full-length dengue polyprotein sequences were downloaded from Virus Pathogen Resources (ViPR, https://www.viprbrc.org/brc/home.spg?decorator=flavi, accessed on 12 February 2022). Full-length viral sequences available from the year 2005, the year in which a major dengue outbreak occurred in India, up until the date of this analysis were retrieved. Only those sequences for which full-length viral polyprotein sequences could be deduced were then selected. A total 107 viral isolate sequences were compiled, of which 29 were DENV-1, 49 were DENV-2, 20 were DENV-3 and 9 were DENV-4. To determine how different the conserved epitopes are from circulating dengue viruses from India, and as compared to distinct geographical regions of the world, we selected full-length viral polyprotein sequences from three distinct geographical regions worldwide, including Thailand, Brazil and Mexico. For these sequences we used the same window of time that we used for the Indian sequences above—i.e., from 2005 until the date of this analysis. A ViPR API was used to query the sequences using the following criteria: datatype as protein, family as *flaviviradae* and species as dengue virus; we then selected the ones having complete sequences. If only partial polyprotein sequences were available, they were excluded. To restrict the analysis to a particular geographical region, country name was used as one of the parameters for the API call.

### 2.3. Retrieval of the Sequences of Dengue Vaccine Constructs

We focused on six dengue vaccines that are under consideration for clinical trials in India or elsewhere. These include the NIH tetravalent live attenuated dengue vaccine (TV003), the Walter Reed Army Institute of Research (WRAIR)/GlaxoSmithKline (GSK) tetravalent live attenuated dengue vaccine (TDEN), the WRAIR/GSK/Oswaldo Cruz Foundation (FIOCRUZ) tetravalent dengue purified inactivated vaccine (DPIV), the Sanofi chimeric yellow fever dengue-tetravalent dengue vaccine (Dengvaxia/CYD-TDV), the Centers for Disease Control and Prevention (CDC)/Takeda/Inviragen chimeric tetravalent dengue vaccine (DENVax) and the WRAIR/Naval Medical Center tetravalent DNA vaccine (TVDV). The reference strains and construct information for each of these vaccine constructs were obtained from the literature [78,79,80,81,82,83] and sequence information for each of the corresponding reference strains was downloaded from the ViPR database. Each reference sequence was altered to account for the modifications in the corresponding vaccine construct.

### 2.4. Alignment of the Dengue-Specific CD4 and CD8 T Cell Epitopes with Sequences of the Dengue Viruses and/or Vaccine Constructs

We then segregated the globally reported T cell epitopes into eight separate categories, DENV-1-, DENV-2-, DENV-3- and DENV-4-specific CD4 and CD8 T cell epitopes. Each serotype-specific epitope sequence was then aligned with circulating viral sequences corresponding to that particular serotype in each geographical region. After the alignment query, an epitope was considered to be present if the epitope aligned with 100% identity. if not, then it was considered absent. The epitope was categorized based on the proportion of viral sequences in which it was identified, with the following bins established—for all viral isolates (100%), for 81 –< 100%, for 51–80%, for <0–50%, and for those absent in any of the viral isolates.

To determine vaccine-carried T cell epitopes, polyprotein sequences of each of the six vaccine constructs (TV003, DPIV, TDEN, Dengvaxia, DENVax and TVDV) were also aligned with sequences of the global CD4 and CD8 epitope sequences compiled, as explained above.

To identify epitopes shared by a vaccine and the circulating viral isolates, the epitopes that were present in each of the vaccines were then aligned with full-length sequences of dengue virus isolates retrieved from India or from each of the other three countries mentioned above.

### 2.5. Statistical Analysis

Statistical analysis was performed using method described in the following link, available online—https://www.medcalc.org/calc/comparison_of_proportions.php, accessed on 28 September 2022. A chi-squared (χ2) test was used to compare the proportions of epitopes present in varying percentages of the viral isolates’ categories. The *p* values are indicated as * *p* ≤ 0.05, ** *p* ≤ 0.01 and *** *p* ≤ 0.001.

### 2.6. Code Availability

All R and python scripts required for reproducing the data can be found on the project’s GitHub repository (https://github.com/prashantbajpai/Dengue_T_Cell_Epitope_Analysis, accessed on 7 October 2022). Raw data used for generating the figures are available at the GitHub page and in the Supplementary Files.

## 3. Results

### 3.1. Cataloging CD4/CD8 T Cell Epitopes among the Circulating Dengue Viruses from India

Our initial step involved a compilation of experimentally validated CD4 T cell epitopes and CD8 T cell epitopes specific to dengue, as sourced from the literature, across various global regions. In total, we retrieved 1889 epitopes. Of these, 1305 were CD4 T cell epitopes and 584 were CD8 T cell epitopes. Of the 1305 CD4 T cell epitopes—354, 395, 294 and 262 were specific to DENV-1, DENV-2, DENV-3 and DENV-4, respectively (Table 1). Of the 584 CD8 T cell epitopes—156, 264, 74 and 90 were specific to DENV-1, DENV-2, DENV-3 and DENV-4, respectively (Table 2). The detailed information for the individual epitopes is provided in Table S1.

**Figure 1:**
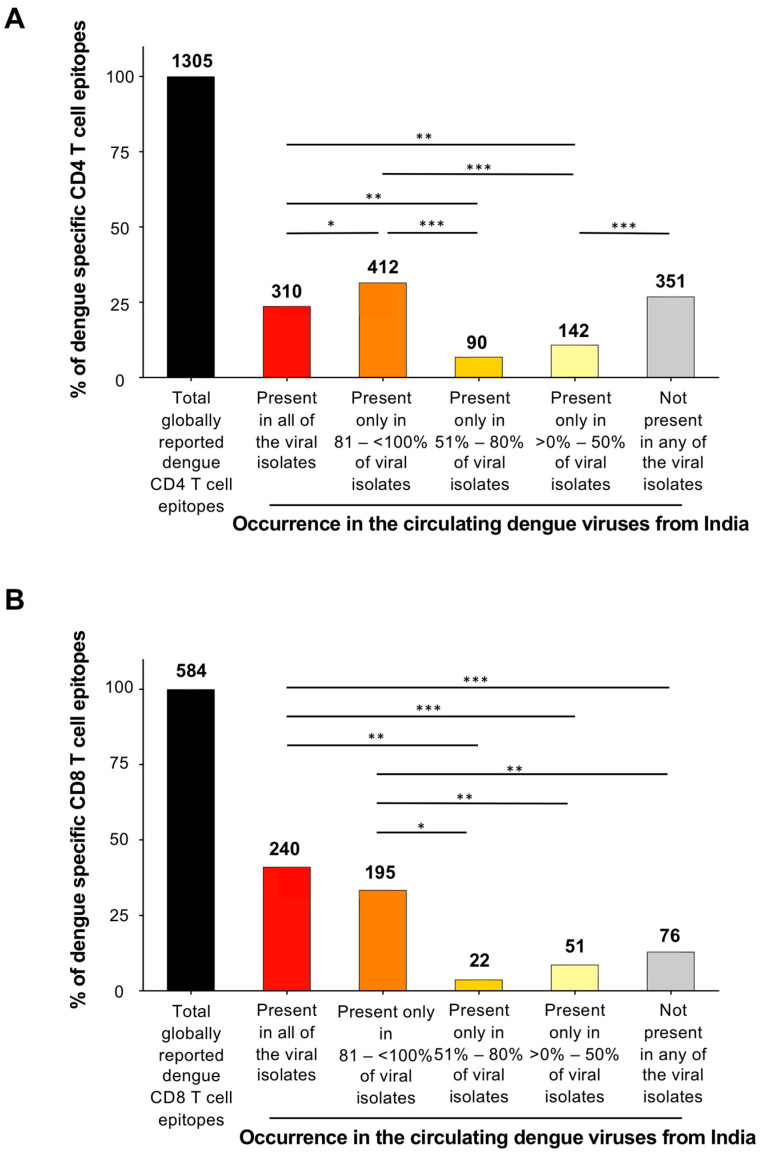
Inspection of the globally reported dengue-specific CD4 and CD8 T cell epitopes for their presence or absence in circulating dengue viruses from India. Bar graphs show the percentages of globally reported dengue-specific (**A**) CD4 T cell epitopes and (**B**) CD8 T cell epitopes in circulating dengue virus isolates from India. In both A and B, the proportion of these epitopes not present in any of the viral isolates; epitopes present in all of the viral isolates; epitopes present in 81 –< 100%; in 51–80%; or in only >0–50% of viral isolates are shown relative to the total epitopes. Numbers of the epitopes present in each category are shown on top of the bars. The significance values associated with the proportions of epitopes in each category are indicated in the coordinates as inset. Significance was calculated by χ2 test. The *p* values are indicated as * *p* ≤ 0.05; ** *p* ≤ 0.01; and *** *p* ≤ 0.001. Detailed information on each of the individual epitopes is shown in Table S3.

To determine which of these experimentally validated globally reported dengue-specific CD4 and CD8 T cell epitopes are conserved among the dengue viruses circulating in India, we first curated a total of 107 published complete polyprotein sequences of dengue viral isolates reported from India, beginning in 2005 (when a major dengue outbreak occurred) and continuing to the date of this analysis, as outlined in the methods section. The detailed information about these dengue polyprotein sequences is provided in Table S2. Of the 1305 dengue-specific CD4 T cell epitopes compiled, we found that 24% of the epitopes were conserved in all the circulating dengue viruses reported from India (Figure 1A). Conversely, we found 27% of the CD4 epitopes were absent in all circulating dengue viruses reported from India. The remainder were found only in a subset of the viruses. A similar trend was even observed for the CD8 T cell epitopes (Figure 1B). Of the 584 CD8 epitopes, 41% were identical in all the circulating dengue viruses reported from India, whereas 13% were absent in any of the dengue virus sequences reported from India, and the remainder only in a subset of the viruses.

Since at least some of these CD8 epitopes have been used for tracking the dengue-specific CD8 T cells using MHC tetramers—the gold standard for enumerating the antigen-specific responses—we then determined the distribution of these known MHC tetramer T cell epitopes among the circulating dengue viruses from India. For this, we compiled a total of 84 known CD8 T cell epitopes that have been successfully used in various studies from different parts of the world to track the dengue-specific CD8 T cell responses using the MHC class I tetramer strategy [84,85]. Interestingly, of these 84 tetramer epitopes, we found that ~11% are absent in any of the circulating dengue viruses from India (namely, E 161–170 HLA A*24:02 SWMIRILIGF, E 737–745 HLA B*58:01 ILIGVIITW, NS3 1608–1617 HLA A*11:01 GTSGSPIVDR, NS3 1608–1617 HLA A*11:01 GTSGSPIADK, NS3 1608–1617 HLA A*11:01GTSGSPIINK, NS4B 2286–2294 HLA B*35:01 VATTFVTPM, NS4B 2436–2444 HLA B*58:01 ATGPVLTLW, NS5 2821–2829 HLA B*55:02 KPWDIIPMV and NS5 3080–3088 HLA A*11:01 TVMDIISRR).

**Figure 2:**
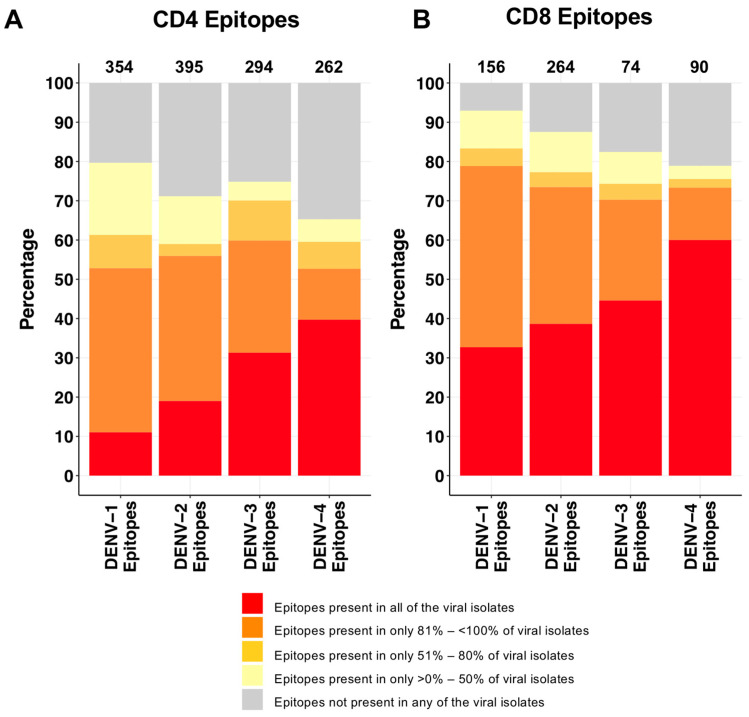
Relative occurrence of the globally reported dengue-specific CD4 and CD8 T cell epitopes in circulating dengue virus isolates from India. Distribution of globally reported dengue-specific (**A**) CD4 T cell epitopes and (**B**) CD8 T cell epitopes in circulating dengue virus isolates from India. In both A and B, distribution of the globally reported epitopes is shown separately for each dengue serotype. Numbers of the globally reported epitopes for each of the serotypes are shown on top of the bars. Stacked bars show the relative distributions of these epitopes in different categories based on their percentage of occurrence in the circulating dengue viruses, as defined earlier. Detailed information on each of the individual epitopes is shown in Table S3.

Of the CD4 T cell epitopes, the order of epitopes, from minimum to maximum, that are conserved in all the circulating dengue viruses from India is DENV1, followed by DENV2, then DENV3 and then the maximum for DENV4 (Figure 2A). Similar trends were observed for CD8 T cell epitopes (Figure 2B).

**Figure 3:**
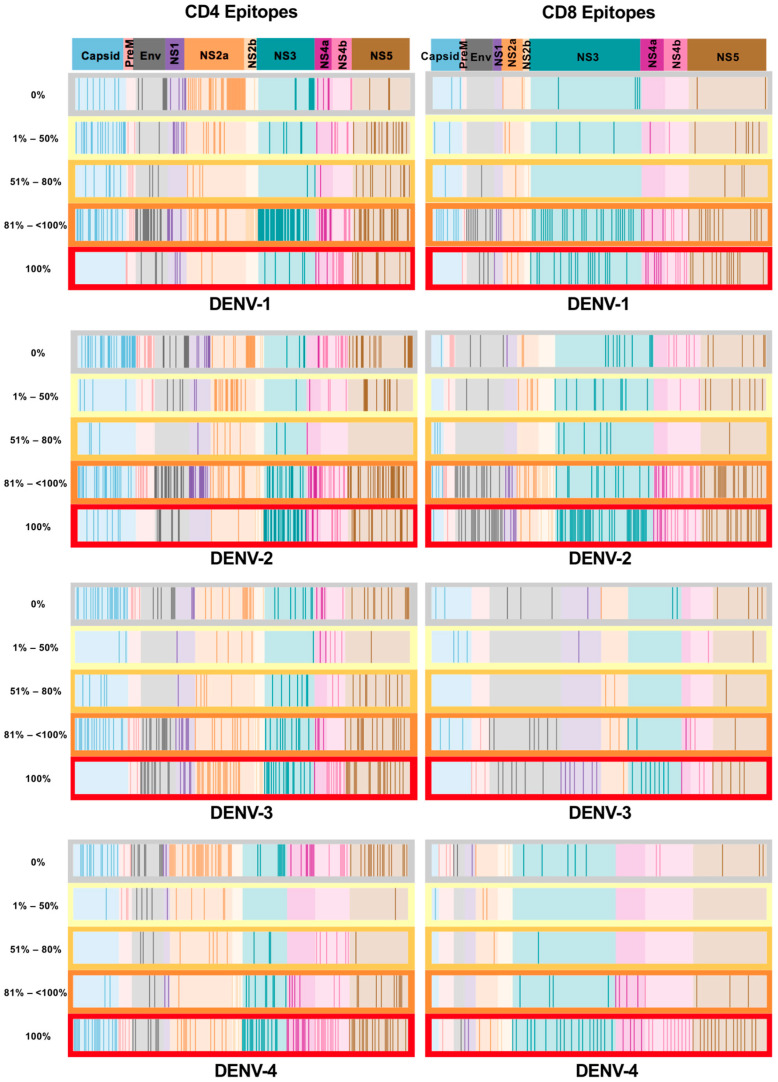
Distribution of the epitopes in the dengue polyprotein in each epitope bin for DENV-1, -2, -3 and -4 serotypes. Bar codes show the protein distributions of epitopes that were binned into the 5 categories based on the percentage of the presence of the epitopes in circulating dengue isolates from India. The sections of the barcode represent each protein of the dengue polyprotein. The length of each section represents the proportion of epitopes in that protein. Each solid line represents the presence of a particular epitope in that category. Protein distributions of CD4 (**left** panel) and CD8 epitopes (**right** panel) are shown separately.

Additionally, we also investigated whether the epitopes present in all the circulating dengue viruses from India were distributed uniformly across the viral proteome or exhibited a bias towards one or more viral proteins. Alignment of all the epitopes in different categories to the dengue virus proteome showed a distribution across all proteins (Figure 3). Comprehensive information on each of the individual epitope sequences that fall into each of these categories is provided in Table S3.

### 3.2. Identification of the CD4 and CD8 T Cell Epitopes within Various Dengue Vaccine Candidates

Many recent studies from India have reported the diversity and evolution of the dengue virus genotype and lineages [86,87,88,89,90,91,92]. Given that the parent strains for all six candidate vaccines under consideration were isolated more than three decades ago [93,94,95,96], and that none of these vaccine strains originated from India [97,98,99], we investigated how well the epitopes conserved in the circulating dengue viruses from India match with the epitopes present in these vaccine strains. For this, we implemented a strategy similar to the above studies by aligning the 1305 dengue-specific global CD4 epitopes and 584 global CD8 T cell epitopes to each of the polyprotein sequences of each of the six dengue vaccine candidates. The details of each of the vaccine construct analyzed are provided in Table 3.

**Figure 4:**
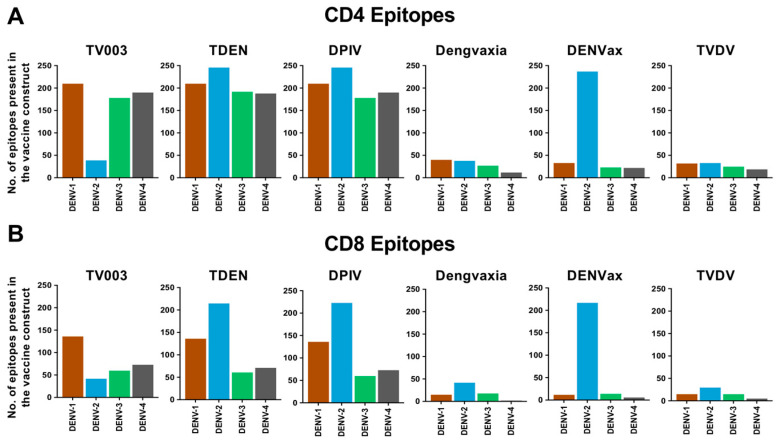

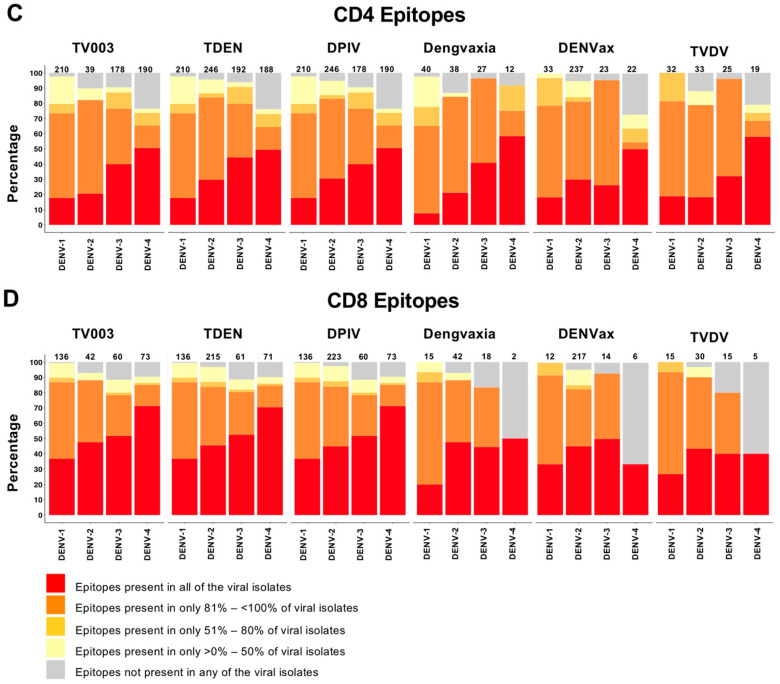
Summary of the distribution of the globally reported dengue-specific global CD4 and CD8 T cell epitopes in individual dengue vaccine candidates and in the circulating dengue viruses from India. Bar graphs show the number of globally reported DENV-1-, DENV-2-, DENV-3- or DENV-4-specific (**A**) CD4 T cell epitopes and (**B**) CD8 T cell epitopes in each of the six indicated dengue vaccine candidate constructs. Distribution of the (**C**) CD4 T cell epitopes and (**D**) CD8 T cell epitopes carried by each of the vaccines among circulating dengue viruses from India. In both (**C**,**D**), numbers on the top of each bar represent the number of epitopes found in the indicated vaccine and for the indicated serotype. Stacked bars show percentages of these epitopes in different categories, as described in the legend.

We found that almost half of the known CD4 T cell epitopes were represented in the whole virus vaccines TV003, TDEN and DPIV, in the cases of DENV-1, DENV-3 and DENV-4 (Figure 4A, Supplementary Table S4). The proportion was even less than a tenth for the chimeric vaccine vectors Dengvaxia, DENVax and TVDV. In the case of DENV2, CD4 epitopes were maximal for TDEN, DPIV and DENVax, and minimal for the rest, possibly reflecting the fact that the non-structural components of DENV-2 are replaced by those of DENV-4 in TV003, and the DENV-2 backbone is utilized for all four chimeras in DENVax [82]. A comparable pattern was observed for CD8 T cell epitopes (Figure 4B, Supplementary Table S4).

Taken together, these findings underscore the importance of considering both serotype specificity and the chimeric nature of the vaccine when assessing, understanding, evaluating and interpreting T cell responses against each of these specific vaccines.

### 3.3. CD4 and CD8 T Cell Epitopes Conserved among the Circulating Dengue Viruses and Shared by the Vaccine Candidates

**Figure 5:**
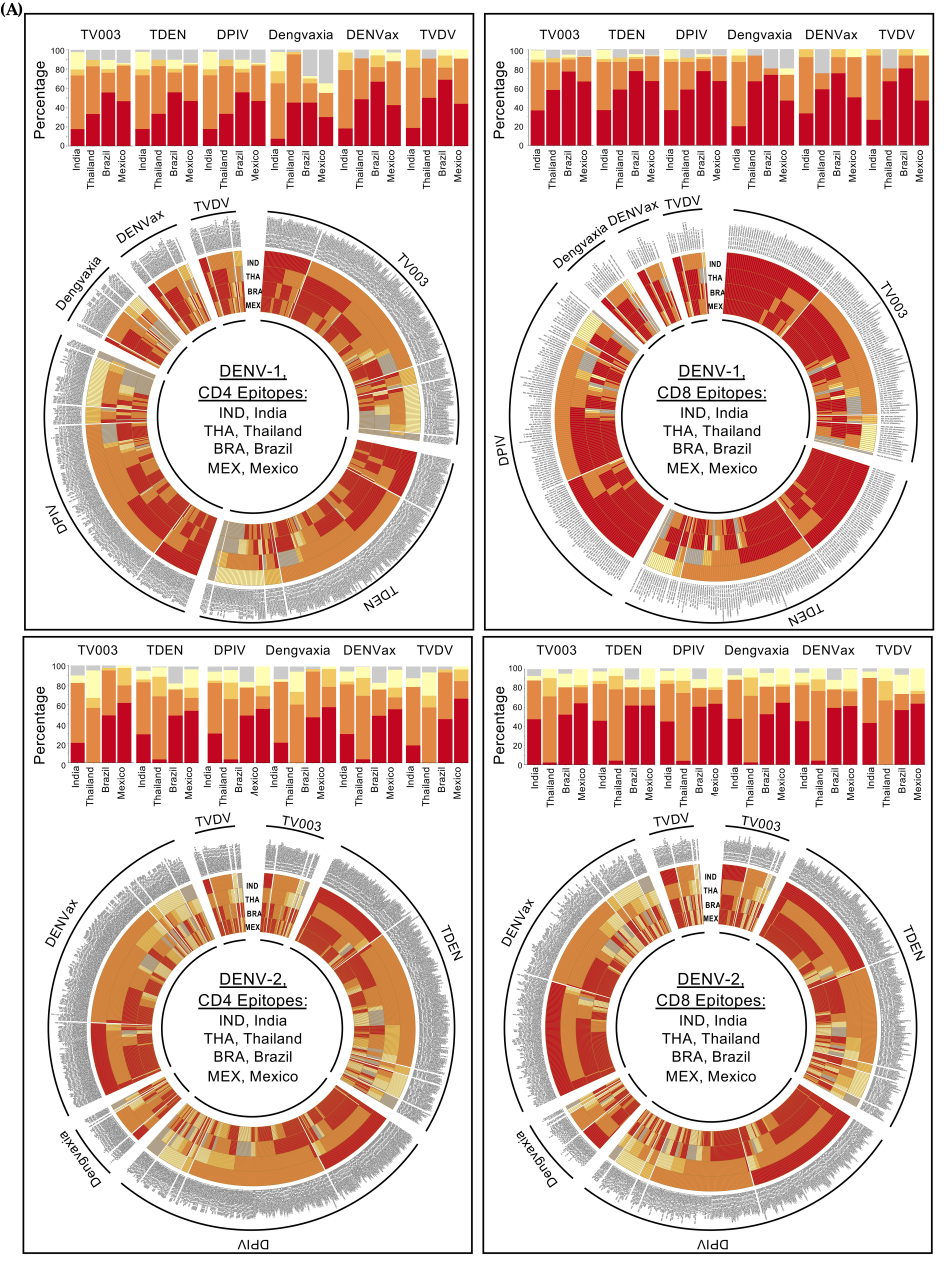

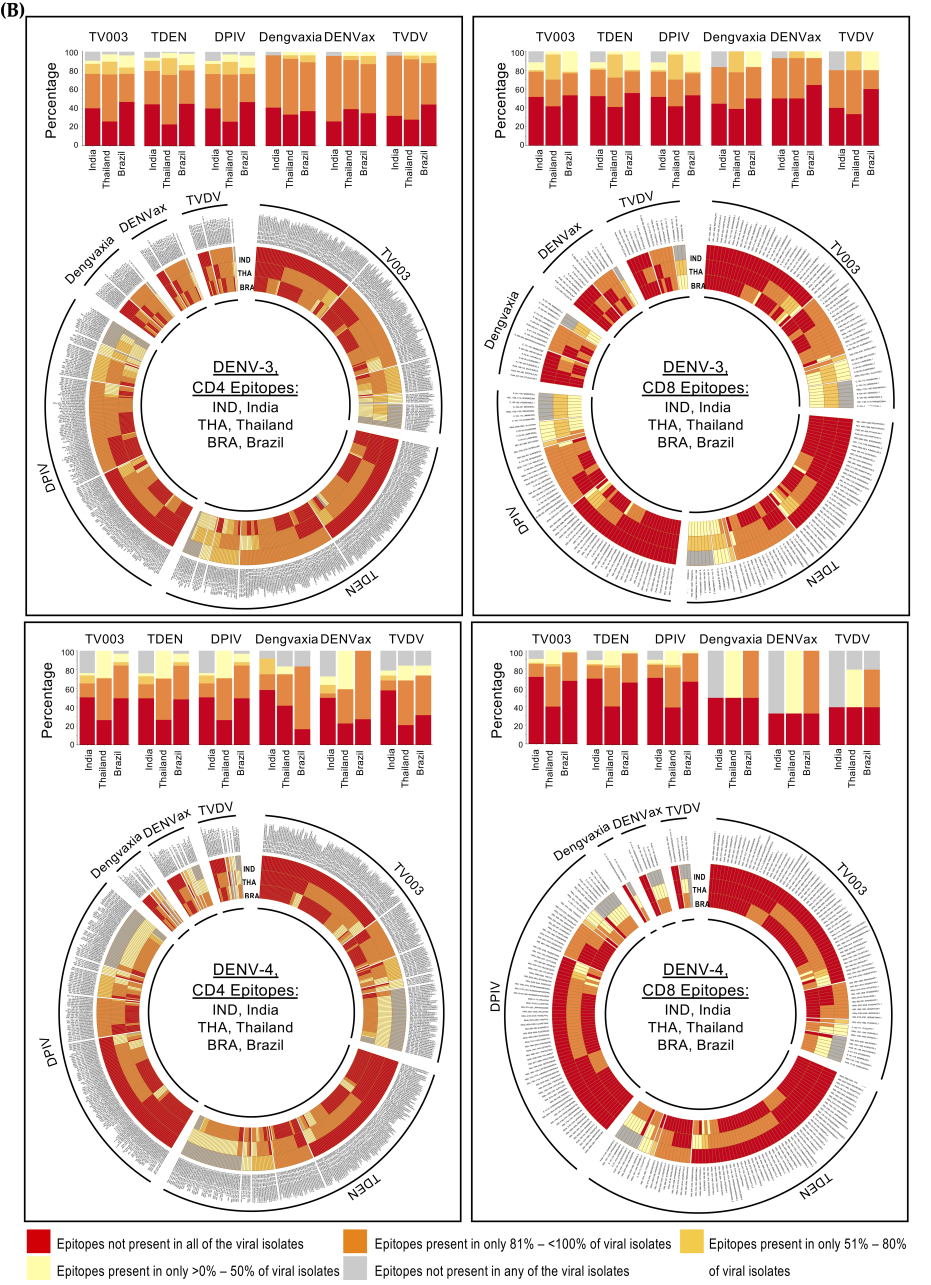
Distribution of the vaccine-carried epitopes in circulating viruses from India compared to circulating viruses from Thailand, Brazil and Mexico. (**A**) DENV-1-specific (top panel) and DENV-2-specific (bottom panel) CD4 epitopes (**left**) and CD8 epitopes (**right**) carried by each of the six indicated vaccines. (**B**) DENV-3-specific (top panel) and DENV-4-specific (bottom panel) CD4 epitopes (**left**) and CD8 epitopes (**right**) carried by each of the six indicated vaccines. In each of the panels, stacked bar graphs show the proportions of the epitopes carried by each of the six indicated vaccines in each of the four indicated serotypes from each of the indicated countries. For DENV-1 and DENV-2, the analysis is performed for India, Thailand, Brazil and Mexico. For DENV-3 and DENV-4, due to there being only two DENV-3 and zero DENV-4 full-length sequences from Mexico, the analysis is restricted to India, Thailand and Brazil only. Stacked bars show percentages of the epitopes in different categories, as indicated.

We next asked which of the epitopes that are conserved among the circulating dengue viruses match with each of the six vaccine candidates. We observed varied proportions across the six vaccine candidates (see Figure 4C for CD4 T cell epitopes and Figure 4D for CD8 T cell epitopes, as well as detailed information on these in Supplementary Tables S3 and S4), with overall presence of epitopes in circulating viruses being least for DENV-1. To determine how different these vaccine-shared conserved epitopes are in distinct geographical regions of the world, we performed a similar analysis using published viral sequences from three geographically distinct regions of the world—Thailand, from Southeast Asia; Brazil from South America; and Mexico from North America. A comparative distribution of the global CD4 and CD8 epitopes carried by each of the six vaccine candidates and conservation among the circulating dengue viruses from each of the geographical regions (India, Thailand, Brazil and Mexico) is summarized for DENV-1 and DENV-2 in Figure 5A and for DENV-3 and DENV-4 in Figure 5B. Detailed information for the individual epitopes both found among the circulating viruses and also shared by individual vaccine candidates, for each of these regions, is provided in Table S4 (for India), Table S6 (for Thailand), Table S7 (for Brazil) and Table S8 (for Mexico). These results, considered together, highlight the fact that the epitopes conserved in circulating viruses and shared by a vaccine candidate in one region need not be uniformly identical in a different region.

## 4. Discussion

Our study provides detailed and nuanced insights on regional variations of the CD4 and CD8 T cell epitopes conserved among circulating dengue viruses, and how these can vary in potential vaccine candidates depending upon the geographical region. This knowledge has important implications for evaluation of T cell responses, not only during dengue natural infection but also for vaccine design, testing, and evaluation efforts.

Our study addresses the important question of which of the globally reported dengue-specific CD4 and CD8 T cell epitopes are present among the dengue viruses, how different these are in distinct geographical regions; which of these epitopes are present in each of the vaccine candidates under consideration; which of these epitopes are shared by both the vaccine candidates and circulating dengue viruses; and how uniform these shared epitopes are when compared to distinct geographical regions of the world. It is surprising that a substantial portion of the CD4 and CD8 T cell epitopes are absent within the circulating dengue viruses from India. Conversely, a substantial portion of the CD4 and CD8 T cell epitopes are absent within the circulating dengue viruses in each of the distinct parts of the world considered. More importantly, the spectrum of the epitopes that are absent from the circulating dengue viruses from India are not necessarily identical to the epitopes absent from the circulating dengue viruses in distinct geographical regions of the world. This may perhaps, indicate that there could have been differences in the T cell mediated host evolutionary pressure on the regional viral evolution that could have been influenced by region specific differences in HLA composition. Alternatively, this could have been a stochastic process. Whether the conservation pattern of certain T cell epitopes in the circulating viruses from a given geographical region impacts antibody responses in any way in that region needs to be determined.

Regardless of the mechanisms, our observation has important implications for interrogation of the region-dependent T cell response data. For example, based on our data, we expect that 27% of the global CD4 epitopes, and 13% of the global CD8 epitopes, will not function, based on the analysis of dengue-specific T cell responses in India. Thus, it is important to design a customized pool that can be used depending upon the geographical region.

It is important to note that at least some of these epitopes that are completely absent in all of the circulating dengue viruses represent well characterized epitopes that have been used for tracking dengue-specific T cells using MHC tetramers. For example, we found that, of the 84 published CD8 epitopes reported for enumeration of dengue-specific CD8 T cells using MHC tetramers, ~11%, are absent in all of the circulating dengue viruses from India. Our findings suggest that these specific MHC tetramers that are absent in the circulating dengue viruses in this certain region are unlikely to work in tracking the dengue-specific T cell responses in that region, thus substantiating the need for the consideration of customized peptides in evaluating T cell responses, depending upon the geographical region of the world.

Our results are of particular relevance to India, given that India is facing the highest dengue burden worldwide [1], contributing nearly a third of the dengue disease burden, with intense efforts taking place to evaluate and deploy vaccines [19,20,21].

In summary, the comprehensive compendium of the dengue-specific CD4 and CD8 T cell peptide epitopes provided here will serve as a fundamental foundation and resource for future efforts aimed at evaluating, assessing and comprehending dengue-specific CD4 and CD8 T cell responses. It also provides a basis for the design, testing and evaluation of vaccines that will be utilized in India and globally.

## Figures and Tables

**Table 1:** Summary of the dengue-specific global CD4 T cell epitopes.

	DENV-1	DENV-2	DENV-3	DENV-4
Capsid	53	68	46	35
PreM	10	22	11	10
Env	34	41	31	25
NS1	20	25	17	5
NS2a	63	53	52	49
NS2b	13	10	9	8
NS3	61	50	44	35
NS4a	18	17	11	22
NS4b	21	32	16	27
NS5	61	77	57	46
All proteins	354	395	294	262
All proteins and all serotypes	1305			

**Table 2:** Summary of dengue-specific global CD8 T cell epitopes.

	DENV-1	DENV-2	DENV-3	DENV-4
Capsid	13	9	8	1
PreM	2	9	4	4
Env	13	39	16	3
NS1	4	10	9	3
NS2a	10	17	6	6
NS2b	3	13	0	4
NS3	52	78	12	28
NS4a	11	11	2	8
NS4b	11	26	5	13
NS5	37	52	12	20
All proteins	156	264	74	90
All proteins and all serotypes	584			

**Table 3:** Details of the dengue tetravalent vaccine constructs analyzed in this study for presence or absence of the known CD4 and CD8 T cell epitopes.

Vaccine	Developer	Clinical Stage	Dengue Serotype	Strain	Accession	Backbone	References
**Whole virus as antigen**							
**TV003**	NIH/NIAID	Phase 3	DENV-1	Nauru/West Pac/1974	NP_059433.1		[71]
(Live attenuated tetravalent DENV with Delta-30 mutation)			DENV-2 (non-structural regions of DENV-4)	New Guinea C	QBK46951.1		
			DENV-3	Sleman 78	AAT69740.1		
			DENV-4	814669	AAK01233.1		
**TDEN**	WRAIR/GSK	Phase 2	DENV-1	Nauru/West Pac/1974	NP_059433.1		[72]
(Live attenuated tetravalent DENV with serial passage in PDK cells)			DENV-2	S16803	ADA00411.1		
			DENV-3	CH53489	AAB69126.2		
			DENV-4	341750	ADA00410.1		
**DPIV**	WRAIR/GSK/FIOCRUZ	Phase 1	DENV-1	Nauru/West Pac/1974	NP_059433.1		[73]
Purified inactivated tetravalent DENV)			DENV-2	New Guinea C	QBK46951.1		
			DENV-3	Sleman 78	AAT69740.1		
			DENV-4	814669	AAK01233.1		
**Pre-membrane and Envelope as antigen**							
**Dengvaxia/CYD-TDV**	Sanofi Pasteur	Licensed	DENV-1	PUO359	AAP80422.1	Yellow fever virus (YFV)	[74]
(Live attenuated chimeric tetravalent DENV)			DENV-2	NewGuineaC_PUO-218hybrid	AAC59274.1		
			DENV-3	PaH881_88	AAA68512.1		
			DENV-4	1228	AER00190.1		
**DENVax/TAK-003**	CDC/Takeda/Inviragen	Phase 3	DENV-1	16007	AAF59976.1	DENV-2 PDK53	[75]
(Live attenuated chimeric tetravalent DENV)			DENV-2	16681	AAA73185.1		
			DENV-3	16562	AAA68508.1		
			DENV-4	1036	AAO83387.1		
**TVDV**	WRAIR/NMRC	Phase 1	DENV-1	Nauru/West Pac/1974	NP_059433.1	plasmid VR1012	[76]
(Tetravalent DNA vaccine with E80 modification in DENV-2)			DENV-2	New Guinea C	QBK46951.1		
			DENV-3	H87	AAA99437.1		
			DENV-4	H241	AAX48017.1		

## Data Availability

All R and python scripts required for reproducing the data can be found on GitHub repository (https://github.com/prashantbajpai/Dengue_T_Cell_Epitope_Analysis accessed on 7 October 2022).

## Supplementary Materials

The following supporting information can be downloaded at: https://www.mdpi.com/article/10.3390/v16050730/s1. Table S1. Details of the individual CD4 and CD8 T cell epitopes published in the literature. Table S2. Dengue polyprotein sequences of circulating viruses from India. Table S3. Alignment of CD4 and CD8 epitopes in circulating viruses from India. Table S4. Globally reported dengue specific CD4 and CD8 T cell epitopes that are present in each of the six different dengue vaccine candidates. Table S5. Number of the complete polyprotein sequences of the dengue viral isolates from Thailand, Brazil and Mexico four each of the four dengue serotypes. Table S6. Alignment of CD4 and CD8 epitopes in circulating dengue viruses from Thailand and six vaccine candidates. Table S7. Alignment of CD4 and CD8 epitopes in circulating dengue viruses from Brazil and six vaccine candidates. Table S8. Alignment of CD4 and CD8 epitopes in circulating dengue viruses from Mexico and six vaccine candidates.
