# Supplementary material for: Regional Variation of the CD4 and CD8 T Cell Epitopes Conserved in Circulating Dengue Viruses and Shared with Potential Vaccine Candidates
Source: Viruses. 2024 May 5;16(5):730. doi: 10.3390/v16050730 (PMC11126086; doi:10.3390/v16050730)
Supplement: Supplementary file 1 [file viruses-16-00730-s001.zip › viruses-2874011-supplementary.pdf]

Supplementary material for

Regional variation of the CD4 and CD8 T cell epitopes conserved in circulating dengue viruses and shared with potential vaccine candidates.

Yadya M. Chawla[1†], Prashant Bajpai[1†], Keshav Saini[1], Elluri Seetharami Reddy[1,2],Ashok Kumar Patel[2] Kaja Murali-Krishna[1,3,4,*] Anmol Chandele[1*]

Supplementary Table S1. Details of the individual CD4 and CD8 T cell epitopes published in the literature. *Detailed information for individual experimentally characterized dengue specific CD4 T cell epitopes and CD8 T cell epitopes that are retrieved from literature. Epitopes are organized into eight categories: DENV-1 specific CD4 epitopes, DENV-2 specific CD4 epitopes, DENV-3 specific CD4 epitopes, DENV-4 specific CD4 epitopes, DENV-1 specific CD8 epitopes, DENV-2 specific CD8 epitopes, DENV-3 specific CD8 epitopes, DENV-4 specific CD8 epitopes. For each epitope, individual columns represent sequence ID, sequence, dengue protein to which the epitope belongs to, start and end amino acid positions of the epitope sequence in the dengue polyprotein, HLA restriction, and functional assays such as IFN-γ Enzyme Linked ImmunoSpot assay (ELISPOT), intracellular IFN-γ cytokine staining assay (ICC) or MHC tetramer assays used to characterize the epitopes.*

CD4 DENV-1 Epitopes

Epitope_ID	AminoAcid_Seq	Protein_Name	Start	End	Allele	Serotype	Method/Technique	Assay Group	Qualitative Measure
1067569	LARWGSFKKNGAIKV	C	66	80	HLA-DRB1*07:01	Dengue virus 1	ELISPOT	IFNg release	Positive-Low
195113	AFLRFLAIPPTAGIL	C	30	44	HLA-DRB1*01:01	Dengue virus 1	ELISPOT	IFNg release	Positive
195117	AGILARWSSFKKNGA	C	51	65	HLA-DRB1*15:01	Dengue virus 1	ELISPOT	IFNg release	Positive
195246	EISNMLNIMNRRKRT	C	87	101	HLA-DRB1*13:01	Dengue virus 1	ELISPOT	IFNg release	Positive
195299	FIAFLRFLAIPPTAG	C	28	42	HLA-DRB1*01:01	Dengue virus 1	ELISPOT	IFNg release	Positive
195314	FNMLKRARNRVSTGS	C	1	15	HLA-DRB1*08:02	Dengue virus 1	ELISPOT	IFNg release	Positive
195315	FNMLKRARNRVSTVS	C	13	27	HLA-DRB1*08:02	Dengue virus 1	ELISPOT	IFNg release	Positive
195468	IAFLRFLAIPPTAGI	C	29	43	HLA-DRB1*01:01	Dengue virus 1	ELISPOT	IFNg release	Positive
195498	IKVLRGFKKEISNML	C	78	92	HLA-DRB1*04:01	Dengue virus 1	ELISPOT	IFNg release	Positive
195535	ISNMLNIMNRRKRSV	C	88	102	HLA-DRB1*08:02	Dengue virus 1	ELISPOT	IFNg release	Positive
195540	ISSMLNIMNRRKRSV	C	76	90	HLA-DRB1*14:02	Dengue virus 1	ELISPOT	IFNg release	Positive
195557	KEISNMLNIMNRRKR	C	86	100	HLA-DRB1*13:01	Dengue virus 1	ELISPOT	IFNg release	Positive
195560	KEVSNMLNIMNRRKR	C	86	100	HLA-DRB1*13:01	Dengue virus 1	ELISPOT	IFNg release	Positive
195586	KRSVTMLLMLLPTAL	C	87	101	HLA-DRB1*01:01	Dengue virus 1	ELISPOT	IFNg release	Positive
195681	LMLLPTALAFHLTTR	C	94	108	HLA-DRB1*04:01	Dengue virus 1	ELISPOT	IFNg release	Positive
195761	MLNIMNRRKRSVTML	C	69	83	HLA-DRB1*08:02	Dengue virus 1	ELISPOT	IFNg release	Positive
195805	NMLNIMNRRKRSVTM	C	90	104	HLA-DRB1*11:01	Dengue virus 1	ELISPOT	IFNg release	Positive
195883	RGFKKEISNMLNIMN	C	82	96	HLA-DRB1*04:01	Dengue virus 1	ELISPOT	IFNg release	Positive
195884	RGFKKEISNMLSIMN	C	82	96	HLA-DRB1*04:01	Dengue virus 1	ELISPOT	IFNg release	Positive
195885	RGFKKEISSMLNIMN	C	60	74	HLA-DRB1*04:01	Dengue virus 1	ELISPOT	IFNg release	Positive
195904	RPSFNMLKRARNRVS	C	10	24	HLA-DRB1*11:01	Dengue virus 1	ELISPOT	IFNg release	Positive
195944	SFNMLKRARNRVSTG	C	12	26	HLA-DRB1*11:01	Dengue virus 1	ELISPOT	IFNg release	Positive
196043	TGRPSFNMLKRARNR	C	8	22	HLA-DRB1*11:01	Dengue virus 1	ELISPOT	IFNg release	Positive
196146	VLRGFKKEISNMLNI	C	80	94	HLA-DRB1*04:01	Dengue virus 1	ELISPOT	IFNg release	Positive
196151	VMAFIAFLRFLAIPP	C	25	39	HLA-DRB1*11:01	Dengue virus 1	ELISPOT	IFNg release	Positive
196188	VSVLFMLLPTALAFH	C	102	116	HLA-DRB1*01:01	Dengue virus 1	ELISPOT	IFNg release	Positive
196195	VTMLFMLLPTALAFH	C	102	116	HLA-DRB1*04:01	Dengue virus 1	ELISPOT	IFNg release	Positive
196196	VTMLLMLLPTALAFH	C	90	104	HLA-DRB1*04:01	Dengue virus 1	ELISPOT	IFNg release	Positive
39096	LRGFKKEISNML	C	81	92	HLA-DPw4	Dengue virus 1	ICS	TNF release	Positive
539043	AFIAFLRFLAIPPTA	C	27	41	HLA-DRB1*12:02	Dengue virus 1	ELISPOT	IFNg release	Positive
539048	AFMAFLRFLAIPPTA	C	27	41	HLA-DRB1*15:02	Dengue virus 1	ELISPOT	IFNg release	Positive
539257	FKKEISNMLNIMNRR	C	84	98	HLA-DRB1*04:03	Dengue virus 1	ELISPOT	IFNg release	Positive
539258	FKKEISSMLNIMNRR	C	62	76	HLA-DRB1*10:01	Dengue virus 1	ELISPOT	IFNg release	Positive
539259	FKKNGAIKVLRGFKK	C	50	64	HLA-DRB1*08:03	Dengue virus 1	ELISPOT	IFNg release	Positive
539278	GAIKVLRGFKKEISN	C	76	90	HLA-DRB1*12:02	Dengue virus 1	ELISPOT	IFNg release	Positive
539459	KEISSMLNIMNRRKR	C	64	78	HLA-DRB1*14:04	Dengue virus 1	ELISPOT	IFNg release	Positive
539475	KKEISSMLNIMNRRK	C	63	77	HLA-DRB1*14:04	Dengue virus 1	ELISPOT	IFNg release	Positive
539495	KNGAIKVLRGFKKEI	C	52	66	HLA-DRB1*12:02	Dengue virus 1	ELISPOT	IFNg release	Positive
539613	LRFLAIPPTAGILAR	C	32	46	HLA-DRB1*04:03	Dengue virus 1	ELISPOT	IFNg release	Positive
539616	LRGFKKEISNMLNIM	C	81	95	HLA-DRB1*10:01	Dengue virus 1	ELISPOT	IFNg release	Positive
539617	LRGFKKEISSMLNIM	C	59	73	HLA-DRB1*13:01	Dengue virus 1	ELISPOT	IFNg release	Positive
539681	MLNIRNRRKRSVTML	C	91	105	HLA-DRB1*13:01	Dengue virus 1	ELISPOT	IFNg release	Positive
539711	NGAIKVLRGFKKEIS	C	53	67	HLA-DRB1*13:01	Dengue virus 1	ELISPOT	IFNg release	Positive
539780	PMKLVMAFIAFLRFL	C	21	35	HLA-DRB1*13:01	Dengue virus 1	ELISPOT	IFNg release	Positive
539785	PSFNMLKRARNRVST	C	11	25	HLA-DRB1*10:01	Dengue virus 1	ELISPOT	IFNg release	Positive
539816	QPSFNMLKRARNRVS	C	10	24	HLA-DRB1*08:03	Dengue virus 1	ELISPOT	IFNg release	Positive
539866	RPSFNMLKRVRNRVS	C	10	24	HLA-DRB1*08:03	Dengue virus 1	ELISPOT	IFNg release	Positive
540059	VLRGFKKEISSMLNI	C	68	82	HLA-DRB1*04:07	Dengue virus 1	ELISPOT	IFNg release	Positive
540117	WGSFKKNGAIKVLRG	C	47	61	HLA-DRB1*09:01	Dengue virus 1	ELISPOT	IFNg release	Positive
738927	FLRFLAIPPTAGILA	C	41	55	HLA-DRB1*14:02	Dengue virus 1	ELISPOT	IFNg release	Positive
740331	SNMLNIMNRRKRSVT	C	89	103	HLA-DRB1*14:02	Dengue virus 1	ELISPOT	IFNg release	Positive
867523	KVLRGFKKEISNMLN	C	79	93	HLA-DRB3*02:02	Dengue virus 1	ELISPOT	IFNg release	Positive
95107	KLVMAFIAFLRFL	C	45	57	HLA-DPw4	Dengue virus 1	ELISPOT	IFNg release	Positive
195368	GIIFILLMLVTPSMA	PreM	266	280	HLA-DRB1*01:01	Dengue virus 1	ELISPOT	IFNg release	Positive
195562	KGIIFILLMLVTPSM	PreM	265	279	HLA-DRB1*08:02	Dengue virus 1	ELISPOT	IFNg release	Positive
195589	KSLLFKTSVGVNMCT	PreM	135	149	HLA-DRB1*07:01	Dengue virus 1	ELISPOT	IFNg release	Positive
195831	PGFTVIALFLAHAIG	PreM	245	259	HLA-DRB1*15:01	Dengue virus 1	ELISPOT	IFNg release	Positive
195887	RGKSLLFKTSAGVNM	PreM	111	125	HLA-DRB1*07:01	Dengue virus 1	ELISPOT	IFNg release	Positive
196085	TVIALFLAHAIGTSI	PreM	248	262	HLA-DRB1*07:01	Dengue virus 1	ELISPOT	IFNg release	Positive
539516	KSLLFKTSAGVNMCT	PreM	123	137	HLA-DRB1*09:01	Dengue virus 1	ELISPOT	IFNg release	Positive
867083	HPGFTVIALFLAHAI	PreM	244	258	HLA-DPB1*02:01	Dengue virus 1	ELISPOT	IFNg release	Positive
867912	LRHPGFTVIALFLAH	PreM	242	256	HLA-DPB1*02:01	Dengue virus 1	ELISPOT	IFNg release	Positive
869062	SVGVNMCTLIAMDLG	PreM	142	156	HLA-DQA1*01:02/DQB1*06:02	Dengue virus 1	ELISPOT	IFNg release	Positive
195145	AKFKCVTKLEGKIVQ	E	117	131	HLA-DRB1*11:01	Dengue virus 1	ELISPOT	IFNg release	Positive
195216	DIELLKTEVTNPAVL	E	37	51	HLA-DRB1*04:01	Dengue virus 1	ELISPOT	IFNg release	Positive
195260	ENLKYSVIVTVHTGD	E	128	142	HLA-DRB1*07:01	Dengue virus 1	ELISPOT	IFNg release	Positive
195359	GGLFTSVGKLIHQVF	E	706	720	HLA-DRB1*11:01	Dengue virus 1	ELISPOT	IFNg release	Positive
195361	GGVFTSVGKLVHQIF	E	426	440	HLA-DRB1*11:01	Dengue virus 1	ELISPOT	IFNg release	Positive
195362	GGVFTSVGKLVHQVF	E	426	440	HLA-DRB1*11:01	Dengue virus 1	ELISPOT	IFNg release	Positive
195477	IELLKTEVTNPAVLR	E	38	52	HLA-DRB1*04:01	Dengue virus 1	ELISPOT	IFNg release	Positive
195484	IGGLFTSVGKLIHQV	E	705	719	HLA-DRB1*07:01	Dengue virus 1	ELISPOT	IFNg release	Positive
195547	IVQYENLKYSVIVTV	E	124	138	HLA-DRB1*15:02	Dengue virus 1	ELISPOT	IFNg release	Positive
195623	LDIELLKTEVTNPAV	E	36	50	HLA-DRB1*04:01	Dengue virus 1	ELISPOT	IFNg release	Positive
195646	LITANPIVTDKEKPV	E	346	360	HLA-DRB1*03:01	Dengue virus 1	ELISPOT	IFNg release	Positive
195814	NRQDLLVTFKTAHAK	E	227	241	HLA-DRB1*04:01	Dengue virus 1	ELISPOT	IFNg release	Positive
195874	QYENLKYSVIVTVHT	E	126	140	HLA-DRB1*07:01	Dengue virus 1	ELISPOT	IFNg release	Positive
196053	TLDIELLKTEVTNPA	E	35	49	HLA-DRB1*04:01	Dengue virus 1	ELISPOT	IFNg release	Positive
539169	DLLVTFKTAHAKKQE	E	230	244	HLA-DRB1*14:04	Dengue virus 1	ELISPOT	IFNg release	Positive
539369	GVSWTMKIGIGILLT	E	445	459	HLA-DRB1*15:02	Dengue virus 1	ELISPOT	IFNg release	Positive
539378	HAKKQEVVVLGSQEGAMH	E	239	256	HLA class II	Dengue virus 1	ICS	IFNg release	Positive
539593	LLVTFKTAHAKKQEV	E	236	250	HLA-DRB1*14:02	Dengue virus 1	ELISPOT	IFNg release	Positive
539670	MKIGIGILLTWLGLN	E	450	464	HLA-DRB1*15:06	Dengue virus 1	ELISPOT	IFNg release	Positive
539800	QDLLVTFKTAHAKKQ	E	229	243	HLA-DRB1*09:01	Dengue virus 1	ELISPOT	IFNg release	Positive
539840	REDLLVTFKTAHAKK	E	513	527	HLA-DRB1*04:03	Dengue virus 1	ELISPOT	IFNg release	Positive
539854	RKDLLVTFKTAHAKK	E	513	527	HLA-DRB1*04:03	Dengue virus 1	ELISPOT	IFNg release	Positive
539869	RQDLLVTFKTAHAKK	E	228	242	HLA-DRB1*08:03	Dengue virus 1	ELISPOT	IFNg release	Positive

540052	VLGSQEGAMHTALTGA	E	247	262	HLA class II	Dengue virus 1	ICS	IFNg release	Positive
540104	VVVLGSQEGAMHTALTG	E	245	261	HLA class II	Dengue virus 1	ICS	IFNg release	Positive
741745	MRCVGIGNRDFVEGLSGATW	E	281	300	HLA class II	Dengue virus 1	ELISPOT	IFNg release	Positive
867045	HAKKQEVAVLGSQEG	E	244	258	HLA-DQA1*03:01/DQB1*03:02	Dengue virus 1	ELISPOT	IFNg release	Positive
867101	HTALTGATEIQTSGT	E	541	555	HLA-DQA1*01:02/DQB1*06:02	Dengue virus 1	ELISPOT	IFNg release	Positive
867378	KCRLKMDKLTLKGMS	E	564	578	HLA-DRB3*02:02	Dengue virus 1	ELISPOT	IFNg release	Positive
868013	LVHKQWFLDLPLPWT	E	487	501	HLA-DQA1*01:01/DQB1*05:01	Dengue virus 1	ELISPOT	IFNg release	Positive
869085	SYIVIGAGKKALKLS	E	376	390	HLA-DRB5*01:01	Dengue virus 1	ELISPOT	IFNg release	Positive
869690	WLAHKQWFLDLPLPW	E	206	220	HLA-DQA1*01:01/DQB1*05:01	Dengue virus 1	ELISPOT	IFNg release	Positive
869691	WLVHKQWFLDLPLPW	E	486	500	HLA-DQA1*01:01/DQB1*05:01	Dengue virus 1	ELISPOT	IFNg release	Positive
95154	SSIGKMFEATARG	E	396	408	HLA-DQ8	Dengue virus 1	ELISPOT	IFNg release	Positive
132583	QYKFQADSPKRLSAA	NS1	31	45	HLA-DRB1*03:01	Dengue virus 1	ELISPOT	IFNg release	Positive
195579	KNETWKLARASFIEV	NS1	206	220	HLA-DRB1*07:01	Dengue virus 1	ELISPOT	IFNg release	Positive
195684	LNHILLENDIKFTVV	NS1	850	864	HLA-DRB1*03:01	Dengue virus 1	ELISPOT	IFNg release	Positive
195685	LNHILLENDMKFTVV	NS1	75	89	HLA-DRB1*03:01	Dengue virus 1	ELISPOT	IFNg release	Positive
195690	LNYILLENDMKFTVV	NS1	850	864	HLA-DRB1*03:01	Dengue virus 1	ELISPOT	IFNg release	Positive
195784	NETWKLARASFIEVK	NS1	207	221	HLA-DRB1*07:01	Dengue virus 1	ELISPOT	IFNg release	Positive
195785	NETWKLTRASFIEVK	NS1	982	996	HLA-DRB1*07:01	Dengue virus 1	ELISPOT	IFNg release	Positive
539401	IESEKNETWKLARASFI	NS1	202	218	HLA class II	Dengue virus 1	ICS	IFNg release	Positive
591686	KFTVVVGDVSGILAQ	NS1	860	874	HLA-DRB1*03:01	Dengue virus 1	ELISPOT	IFNg release	Positive
738503	ANGILAQGKKMIRPQ	NS1	868	882	HLA-DRB1*14:06	Dengue virus 1	ELISPOT	IFNg release	Positive
740562	TVVVGDANGILAQGK	NS1	862	876	HLA-DRB1*01:02	Dengue virus 1	ELISPOT	IFNg release	Positive
866284	DDQRAWNIWEVEDYG	NS1	181	195	HLA-DQA1*01:01/DQB1*05:01	Dengue virus 1	ELISPOT	IFNg release	Positive
866693	FGIFTTNIWLKLRDS	NS1	196	210	HLA-DPB1*04:01	Dengue virus 1	ELISPOT	IFNg release	Positive
867041	HADMGYWIESEKNET	NS1	231	245	HLA-DQA1*01:01/DQB1*05:01	Dengue virus 1	ELISPOT	IFNg release	Positive
867405	KIIGADIQNTTFIID	NS1	897	911	HLA-DPB1*04:02	Dengue virus 1	ELISPOT	IFNg release	Positive
868492	QISNELNYILLENDM	NS1	845	859	HLA-DPB1*01:01	Dengue virus 1	ELISPOT	IFNg release	Positive
868574	QRAWNIWEVEDYGFG	NS1	183	197	HLA-DQA1*01:01/DQB1*05:01	Dengue virus 1	ELISPOT	IFNg release	Positive
868630	QYKFQADSPKKLSAA	NS1	806	820	HLA-DRB3*02:02	Dengue virus 1	ELISPOT	IFNg release	Positive
869135	TEQYKFQADSPKRLS	NS1	65	79	HLA-DRB5*01:01	Dengue virus 1	ELISPOT	IFNg release	Positive
869721	YGFGIFTTNIWLKLR	NS1	194	208	HLA-DPB1*02:01	Dengue virus 1	ELISPOT	IFNg release	Positive
167816	SLVASVELPNSLEELGDGLAMGIMI	NS2a	109	133	HLA class II	Dengue virus 1	ELISPOT	IFNg release	Positive
195162	AMVLSIVSLFPLCLS	NS2a	1296	1310	HLA-DRB1*15:01	Dengue virus 1	ELISPOT	IFNg release	Positive
195196	AWKTMAMALSIVSLF	NS2a	1291	1305	HLA-DRB1*08:02	Dengue virus 1	ELISPOT	IFNg release	Positive
195285	FAVGLLFRRLTSREI	NS2a	1214	1228	HLA-DRB1*11:01	Dengue virus 1	ELISPOT	IFNg release	Positive
195286	FAVGLLFRRLTSREV	NS2a	1214	1228	HLA-DRB1*08:03	Dengue virus 1	ELISPOT	IFNg release	Positive
195323	FQSHQLWATLLSLTF	NS2a	1267	1281	HLA-DPB1*04:02	Dengue virus 1	ELISPOT	IFNg release	Positive
195389	GLLFRKLTSREVLLL	NS2a	1217	1231	HLA-DRB1*11:01	Dengue virus 1	ELISPOT	IFNg release	Positive
195390	GLLFRRLTSREILLL	NS2a	1217	1231	HLA-DRB1*07:01	Dengue virus 1	ELISPOT	IFNg release	Positive
195391	GLLFRRLTSREVLLL	NS2a	1217	1231	HLA-DRB1*08:03	Dengue virus 1	ELISPOT	IFNg release	Positive
195392	GLLFRRLTSREVPLL	NS2a	1217	1231	HLA-DRB1*10:01	Dengue virus 1	ELISPOT	IFNg release	Positive
195422	GTLAVFLLLIMGQLT	NS2a	1162	1176	HLA-DRB1*01:01	Dengue virus 1	ELISPOT	IFNg release	Positive
195423	GTLVVFFLLIMGQLT	NS2a	1162	1176	HLA-DRB1*15:01	Dengue virus 1	ELISPOT	IFNg release	Positive
195595	KTMAMVLSIVSLFPL	NS2a	1293	1307	HLA-DRB1*03:01	Dengue virus 1	ELISPOT	IFNg release	Positive
195613	LAVFLLLIMGQLTWN	NS2a	1164	1178	HLA-DRB1*15:06	Dengue virus 1	ELISPOT	IFNg release	Positive
195614	LAVGLLFRRLTSREV	NS2a	1214	1228	HLA-DRB1*11:01	Dengue virus 1	ELISPOT	IFNg release	Positive
195665	LLLTIGLSLVASVEL	NS2a	1229	1243	HLA-DRB1*07:01	Dengue virus 1	ELISPOT	IFNg release	Positive
195670	LLSLTFIKTTFSLHY	NS2a	1276	1290	HLA-DRB1*07:01	Dengue virus 1	ELISPOT	IFNg release	Positive
195674	LLTIGLSLVASVELP	NS2a	1230	1244	HLA-DRB1*07:01	Dengue virus 1	ELISPOT	IFNg release	Positive
195708	LSIVSLFPLCLSTTS	NS2a	1299	1313	HLA-DRB1*01:01	Dengue virus 1	ELISPOT	IFNg release	Positive
195713	LSLTFIRTSLSLDYA	NS2a	1277	1291	HLA-DRB1*03:01	Dengue virus 1	ELISPOT	IFNg release	Positive
195745	MAMVLSIVSLFPLCL	NS2a	1295	1309	HLA-DRB1*07:01	Dengue virus 1	ELISPOT	IFNg release	Positive
195756	MGLLFRRLTSREVLL	NS2a	1216	1230	HLA-DRB1*11:01	Dengue virus 1	ELISPOT	IFNg release	Positive
195881	REVLLLTVGLSLVAC	NS2a	1226	1240	HLA-DRB1*07:01	Dengue virus 1	ELISPOT	IFNg release	Positive
195959	SILIEEVMRSRWSRK	NS2a	1143	1157	HLA-DRB1*11:04	Dengue virus 1	ELISPOT	IFNg release	Positive
195983	SREVLLLTIGLSLVA	NS2a	1225	1239	HLA-DRB1*15:01	Dengue virus 1	ELISPOT	IFNg release	Positive
196216	WKTMAMVLSIVSLFP	NS2a	1292	1306	HLA-DRB1*08:02	Dengue virus 1	ELISPOT	IFNg release	Positive
539086	AMGIMILKLLTDFQS	NS2a	1255	1269	HLA-DRB1*04:03	Dengue virus 1	ELISPOT	IFNg release	Positive
539094	ATFKMRPMFAVGLLF	NS2a	1206	1220	HLA-DRB1*01:02	Dengue virus 1	ELISPOT	IFNg release	Positive
539205	EEVMRSRWSRKMLMT	NS2a	1147	1161	HLA-DRB1*13:01	Dengue virus 1	ELISPOT	IFNg release	Positive
539272	FSLHYAWKTMAMVLS	NS2a	1286	1300	HLA-DRB1*09:01	Dengue virus 1	ELISPOT	IFNg release	Positive
539398	IEEVMRSRWSRKMLM	NS2a	1146	1160	HLA-DRB1*13:01	Dengue virus 1	ELISPOT	IFNg release	Positive
539517	KTTFSLHYAWKTMAM	NS2a	1283	1297	HLA-DRB1*09:01	Dengue virus 1	ELISPOT	IFNg release	Positive
539624	LSLTFIKTTLSLHYA	NS2a	1277	1291	HLA-DRB1*14:04	Dengue virus 1	ELISPOT	IFNg release	Positive
539625	LSLTFIKTTYSLHYA	NS2a	1277	1291	HLA-DRB1*14:04	Dengue virus 1	ELISPOT	IFNg release	Positive
539626	LSLTFIRSTIPLVMA	NS2a	1275	1289	HLA-DRB1*14:04	Dengue virus 1	ELISPOT	IFNg release	Positive
539651	LVVFLLLIMGQLTWS	NS2a	1164	1178	HLA-DRB1*15:06	Dengue virus 1	ELISPOT	IFNg release	Positive
539657	MALSIVSLFPLCLST	NS2a	1297	1311	HLA-DRB1*15:06	Dengue virus 1	ELISPOT	IFNg release	Positive
539679	MLMTGTLAVFLLLIM	NS2a	1158	1172	HLA-DRB1*15:06	Dengue virus 1	ELISPOT	IFNg release	Positive
539680	MLMTGTLVVFFLLIM	NS2a	1158	1172	HLA-DRB1*15:06	Dengue virus 1	ELISPOT	IFNg release	Positive
539888	RWSRKMLMTGTLAVF	NS2a	1153	1167	HLA-DRB1*04:03	Dengue virus 1	ELISPOT	IFNg release	Positive
539912	SLDYAWKTMAMALSI	NS2a	1287	1301	HLA-DRB1*09:01	Dengue virus 1	ELISPOT	IFNg release	Positive
539913	SLHYAWKTMAMVLSI	NS2a	1287	1301	HLA-DRB1*09:01	Dengue virus 1	ELISPOT	IFNg release	Positive
539918	SLTFIKTTLSLHHAW	NS2a	1278	1292	HLA-DRB1*14:04	Dengue virus 1	ELISPOT	IFNg release	Positive
539970	TFSLHYAWKTMAMVL	NS2a	1285	1299	HLA-DRB1*14:06	Dengue virus 1	ELISPOT	IFNg release	Positive
539974	TGTLVVFLLLTLGQL	NS2a	1161	1175	HLA-DRB1*15:06	Dengue virus 1	ELISPOT	IFNg release	Positive
540006	TTLLSLTFIRTSLSL	NS2a	1274	1288	HLA-DRB1*12:02	Dengue virus 1	ELISPOT	IFNg release	Positive
540038	VGLLFRRLTSREVLL	NS2a	1216	1230	HLA-DRB1*08:03	Dengue virus 1	ELISPOT	IFNg release	Positive
540155	YLALMATFKMRPMFA	NS2a	1201	1215	HLA-DRB1*13:01	Dengue virus 1	ELISPOT	IFNg release	Positive
591779	TLAVFLLLTMGQLTW	NS2a	36	50	HLA-DRB1*11:01	Dengue virus 1	ELISPOT	IFNg release	Positive
738553	AVGLLFRRLTSREVL	NS2a	1215	1229	HLA-DRB1*01:02	Dengue virus 1	ELISPOT	IFNg release	Positive
738679	DLIRLCIMVGANASD	NS2a	1179	1193	HLA-DRB1*11:04	Dengue virus 1	ELISPOT	IFNg release	Positive
739424	LAVFLLLVMGQLTWN	NS2a	1164	1178	HLA-DRB1*11:04	Dengue virus 1	ELISPOT	IFNg release	Positive
739508	LLCISIMIEEVMRSR	NS2a	1139	1153	HLA-DRB1*11:04	Dengue virus 1	ELISPOT	IFNg release	Positive
739578	LSLTFIKTTLSLHHA	NS2a	1277	1291	HLA-DRB1*04:07	Dengue virus 1	ELISPOT	IFNg release	Positive
866118	AMALSIVSLFPLCLS	NS2a	1296	1310	HLA-DPB1*04:01	Dengue virus 1	ELISPOT	IFNg release	Positive
866767	FQSYQLWATLLSLTF	NS2a	1267	1281	HLA-DPB1*04:02	Dengue virus 1	ELISPOT	IFNg release	Positive
866873	GILAVFFLLIMGQLT	NS2a	1162	1176	HLA-DPB1*04:02	Dengue virus 1	ELISPOT	IFNg release	Positive
867232	ILKLLTDFQSHQLWA	NS2a	1260	1274	HLA-DPB1*01:01	Dengue virus 1	ELISPOT	IFNg release	Positive
867997	LTSREILLLTVGLSL	NS2a	1223	1237	HLA-DPB1*04:02	Dengue virus 1	ELISPOT	IFNg release	Positive

868098	MGMGTTYLALMATFK	NS2a	456	470	HLA-DRB5*01:01	Dengue virus 1	ELISPOT	IFNg release	Positive
868100	MGQLTWNDLIRLCIM	NS2a	433	447	HLA-DPB1*04:02	Dengue virus 1	ELISPOT	IFNg release	Positive
868553	QPHQLWATLLSLTFV	NS2a	1268	1282	HLA-DPB1*04:02	Dengue virus 1	ELISPOT	IFNg release	Positive
869103	TDFQPHQLWTTLLSL	NS2a	1265	1279	HLA-DPB1*04:02	Dengue virus 1	ELISPOT	IFNg release	Positive
195191	AVGIVSILLSSLLKN	NS2b	1355	1369	HLA-DRB1*08:02	Dengue virus 1	ELISPOT	IFNg release	Positive
195228	DTLTILLKATLLAVS	NS2b	1438	1452	HLA-DRB1*08:02	Dengue virus 1	ELISPOT	IFNg release	Positive
195508	ILVEVQDDGTMKIKD	NS2b	68	82	HLA-DRB1*03:01	Dengue virus 1	ELISPOT	IFNg release	Positive
195510	IMAVGIVSILLSSLL	NS2b	1353	1367	HLA-DRB1*07:01	Dengue virus 1	ELISPOT	IFNg release	Positive
195715	LSSLLKNDVPLAGPL	NS2b	1363	1377	HLA-DRB1*03:01	Dengue virus 1	ELISPOT	IFNg release	Positive
195724	LTILLKATLLAVSGV	NS2b	1440	1454	HLA-DRB1*08:02	Dengue virus 1	ELISPOT	IFNg release	Positive
195878	RDDTLTILLKATLLA	NS2b	1436	1450	HLA-DRB1*12:02	Dengue virus 1	ELISPOT	IFNg release	Positive
195993	SSLLKNDVPLAGPLI	NS2b	1364	1378	HLA-DRB1*03:01	Dengue virus 1	ELISPOT	IFNg release	Positive
196111	VEVQDDGTMKIKDEE	NS2b	1421	1435	HLA-DRB1*03:01	Dengue virus 1	ELISPOT	IFNg release	Positive
866200	ATLFLWYFWQKKKQR	NS2b	1461	1475	HLA-DPB1*01:01	Dengue virus 1	ELISPOT	IFNg release	Positive
866201	ATLFVWYFWQKKKQR	NS2b	1461	1475	HLA-DPB1*01:01	Dengue virus 1	ELISPOT	IFNg release	Positive
867359	IVSILLSSLLKNDVP	NS2b	1358	1372	HLA-DPB1*04:02	Dengue virus 1	ELISPOT	IFNg release	Positive
867544	LAGPLIAGGMLIACY	NS2b	1373	1387	HLA-DQA1*01:02/DQB1*06:02	Dengue virus 1	ELISPOT	IFNg release	Positive
1392180	GKEIVDLMCHATFTM	NS3	1730	1744	HLA class II	Dengue virus 1	biological activity	activation	Positive
167826	TSGTYVSAIAQAKASQE	NS3	157	173	HLA class II	Dengue virus 1	ELISPOT	IFNg release	Positive
195143	AIVREAIKRKLRTLI	NS3	1682	1696	HLA-DRB1*11:01	Dengue virus 1	ELISPOT	IFNg release	Positive
195163	ANCLRKNGKRVIQLS	NS3	1848	1862	HLA-DRB1*13:01	Dengue virus 1	ELISPOT	IFNg release	Positive
195165	ANFRADRVIDPRRCL	NS3	1891	1905	HLA-DRB1*03:01	Dengue virus 1	ELISPOT	IFNg release	Positive
195201	CHATFTMRLLSPVRV	NS3	1737	1751	HLA-DRB1*01:01	Dengue virus 1	ELISPOT	IFNg release	Positive
195242	EGKIVGLYGNGVVTT	NS3	1618	1632	HLA-DRB1*15:01	Dengue virus 1	ELISPOT	IFNg release	Positive
195296	FHTMWHVTRGAVLMY	NS3	1521	1535	HLA-DRB1*07:01	Dengue virus 1	ELISPOT	IFNg release	Positive
195338	FTMRLLSPVRVPNYN	NS3	1741	1755	HLA-DRB1*01:01	Dengue virus 1	ELISPOT	IFNg release	Positive
195377	GIYRILQRGLLGRSQ	NS3	1496	1510	HLA-DRB1*11:01	Dengue virus 1	ELISPOT	IFNg release	Positive
195379	GKIVGLYGNGVVTTS	NS3	1619	1633	HLA-DRB1*15:01	Dengue virus 1	ELISPOT	IFNg release	Positive
195549	IYRILQRGLLGRSQV	NS3	1497	1511	HLA-DRB1*15:01	Dengue virus 1	ELISPOT	IFNg release	Positive
195585	KRKLRTLILAPTRVV	NS3	1689	1703	HLA-DRB1*10:01	Dengue virus 1	ELISPOT	IFNg release	Positive
195622	LDDGIYRIMQRGLLG	NS3	1493	1507	HLA-DRB1*11:01	Dengue virus 1	ELISPOT	IFNg release	Positive
195705	LRTLILAPTRVVASE	NS3	1692	1706	HLA-DRB1*08:02	Dengue virus 1	ELISPOT	IFNg release	Positive
195777	NCLRKNGKRVIQLSR	NS3	1849	1863	HLA-DRB1*11:01	Dengue virus 1	ELISPOT	IFNg release	Positive
195778	NDIANCLRKNGKRVI	NS3	1845	1859	HLA-DRB1*11:01	Dengue virus 1	ELISPOT	IFNg release	Positive
195896	RKLRTLILAPTRVVA	NS3	1690	1704	HLA-DRB1*01:01	Dengue virus 1	ELISPOT	IFNg release	Positive
195914	RTLILAPTRVVASEM	NS3	1693	1707	HLA-DRB1*10:01	Dengue virus 1	ELISPOT	IFNg release	Positive
196130	VILKDGPERVILAGP	NS3	1908	1922	HLA-DRB1*03:01	Dengue virus 1	ELISPOT	IFNg release	Positive
196138	VLDDGIYRILQRGLL	NS3	1492	1506	HLA-DRB1*11:01	Dengue virus 1	ELISPOT	IFNg release	Positive
196197	VTRGAVLMYQGKRLE	NS3	1527	1541	HLA-DRB1*15:01	Dengue virus 1	ELISPOT	IFNg release	Positive
24915	HTGKEIVDLMCHATE	NS3	251	265	HLA-DPw2	Dengue virus 1	ICS	IFNg release	Positive
30397	KEGERKKLRPRWLDA	NS3	585	599	HLA class II	Dengue virus 1	ICS	IFNg release	Positive
45669	NREGKIVGLYGNGVV	NS3	141	155	HLA-DRB1*15:01	Dengue virus 1	ICS	IFNg release	Positive
49768	PTRVVASEMAEALKG	NS3	224	238	HLA-DRB1*15:01	Dengue virus 1	ICS	TNF release	Positive
539022	AAAIFMTATPPGSVE	NS3	1785	1799	HLA-DRB1*04:03	Dengue virus 1	ELISPOT	IFNg release	Positive
539027	AAIFMTATPPGSVEA	NS3	1786	1800	HLA-DRB1*10:01	Dengue virus 1	ELISPOT	IFNg release	Positive
539155	DGIYRILQRGLLGRS	NS3	1495	1509	HLA-DRB1*12:02	Dengue virus 1	ELISPOT	IFNg release	Positive
539156	DGIYRIMQRGLLGRS	NS3	1495	1509	HLA-DRB1*12:02	Dengue virus 1	ELISPOT	IFNg release	Positive
539171	DLMCHATFTMRLLSP	NS3	1734	1748	HLA-DRB1*13:01	Dengue virus 1	ELISPOT	IFNg release	Positive
539288	GEAAAIFMTATPPGS	NS3	1783	1797	HLA-DRB1*04:03	Dengue virus 1	ELISPOT	IFNg release	Positive
539309	GIYRIMQRGLLGRSQ	NS3	1496	1510	HLA-DRB1*14:04	Dengue virus 1	ELISPOT	IFNg release	Positive
539485	KLRTLILAPTRVVAS	NS3	1691	1705	HLA-DRB1*04:03	Dengue virus 1	ELISPOT	IFNg release	Positive
539543	LDDGIYRILQRGLLG	NS3	1493	1507	HLA-DRB1*12:02	Dengue virus 1	ELISPOT	IFNg release	Positive
539579	LKGMPIRYQTTAVKS	NS3	1711	1725	HLA-DRB1*04:03	Dengue virus 1	ELISPOT	IFNg release	Positive
539715	NGKRVIQLSRKTFDT	NS3	1854	1868	HLA-DRB1*12:02	Dengue virus 1	ELISPOT	IFNg release	Positive
539839	REAIRRKLRTLILAP	NS3	1685	1699	HLA-DRB1*14:04	Dengue virus 1	ELISPOT	IFNg release	Positive
539902	SGTYVSAIAQAKASQ	NS3	1633	1647	HLA-DRB1*10:01	Dengue virus 1	ELISPOT	IFNg release	Positive
539986	TLILAPTRVVASEMA	NS3	1694	1708	HLA-DRB1*13:01	Dengue virus 1	ELISPOT	IFNg release	Positive
539993	TNCLRKNGKRVIQLS	NS3	1848	1862	HLA-DRB1*13:01	Dengue virus 1	ELISPOT	IFNg release	Positive
540040	VGLYGNGVVTTSGTY	NS3	1622	1636	HLA-DRB1*15:06	Dengue virus 1	ELISPOT	IFNg release	Positive
540071	VREAIKKKLRTLILA	NS3	1684	1698	HLA-DRB1*12:02	Dengue virus 1	ELISPOT	IFNg release	Positive
540074	VREAIKRKLRTLILA	NS3	1684	1698	HLA-DRB1*12:02	Dengue virus 1	ELISPOT	IFNg release	Positive
540075	VREAIKRKLRTLVLA	NS3	1684	1698	HLA-DRB1*12:02	Dengue virus 1	ELISPOT	IFNg release	Positive
54391	RKLTIMDLHPGSGKT	NS3	1662	1676	HLA class II	Dengue virus 1	ICS	IFNg release	Positive
738417	AAAIFMTATPPGSAE	NS3	1785	1799	HLA-DRB1*04:07	Dengue virus 1	ELISPOT	IFNg release	Positive
738418	AAAIFMTATPPGSME	NS3	1785	1799	HLA-DRB1*04:07	Dengue virus 1	ELISPOT	IFNg release	Positive
739269	IVGLYGNGVVTTSGT	NS3	132	146	HLA-DRB1*01:02	Dengue virus 1	ELISPOT	IFNg release	Positive
739540	LPAIVREAIKRKLRT	NS3	1680	1694	HLA-DRB1*14:02	Dengue virus 1	ELISPOT	IFNg release	Positive
866181	ASQEGPLPEIEDEVF	NS3	1645	1659	HLA-DQA1*05:01/DQB1*02:01	Dengue virus 1	ELISPOT	IFNg release	Positive
866469	EAIKRKLRTLILAPT	NS3	1686	1700	HLA-DPB1*01:01	Dengue virus 1	ELISPOT	IFNg release	Positive
866721	FKTPEGEVGAIALDF	NS3	1591	1605	HLA-DQA1*03:01/DQB1*03:02	Dengue virus 1	ELISPOT	IFNg release	Positive
867025	GVFQENVFHTMWHVT	NS3	1514	1528	HLA-DPB1*04:01	Dengue virus 1	ELISPOT	IFNg release	Positive
867396	KGMPIRYQTTAVKSE	NS3	1712	1726	HLA-DRB3*02:02	Dengue virus 1	ELISPOT	IFNg release	Positive
868113	MIIMDEAHFTDPASI	NS3	1756	1770	HLA-DQA1*01:01/DQB1*05:01	Dengue virus 1	ELISPOT	IFNg release	Positive
868343	PNYNMIIMDEAHFTD	NS3	1752	1766	HLA-DQA1*05:01/DQB1*02:01	Dengue virus 1	ELISPOT	IFNg release	Positive
869341	TTSGTYVSAIAQAKA	NS3	1631	1645	HLA-DQA1*01:02/DQB1*06:02	Dengue virus 1	ELISPOT	IFNg release	Positive
869688	WHVTRGAVLMYQGKR	NS3	1525	1539	HLA-DQA1*01:02/DQB1*06:02	Dengue virus 1	ELISPOT	IFNg release	Positive
869696	WNTGEEVQVIAVEPG	NS3	1564	1578	HLA-DQA1*03:01/DQB1*03:02	Dengue virus 1	ELISPOT	IFNg release	Positive
95169	YRILQRGLLGRSQ	NS3	23	35	HLA-DR1	Dengue virus 1	ELISPOT	IFNg release	Positive
195478	IETLMLLALIAVLTG	NS4a	2146	2160	HLA-DRB1*15:01	Dengue virus 1	ELISPOT	IFNg release	Positive
195485	IGLLCVMASSALLWM	NS4a	2177	2191	HLA-DRB1*07:01	Dengue virus 1	ELISPOT	IFNg release	Positive
195596	KTSIGLLCVTASSAL	NS4a	2174	2188	HLA-DRB1*07:01	Dengue virus 1	ELISPOT	IFNg release	Positive
195679	LMLLALIAVLTGGVT	NS4a	2149	2163	HLA-DRB1*08:02	Dengue virus 1	ELISPOT	IFNg release	Positive
195680	LMLLALTAVLTGGVT	NS4a	2149	2163	HLA-DRB1*01:01	Dengue virus 1	ELISPOT	IFNg release	Positive
196046	TIETLMLLALIAALT	NS4a	2145	2159	HLA-DRB1*01:01	Dengue virus 1	ELISPOT	IFNg release	Positive
196214	WIAASIILEFFLMVL	NS4a	2198	2212	HLA-DRB1*15:01	Dengue virus 1	ELISPOT	IFNg release	Positive
196255	YVVIGLLFMILTVAA	NS4a	2230	2244	HLA-DRB1*11:01	Dengue virus 1	ELISPOT	IFNg release	Positive
539383	HLTLRAQNALDNLVM	NS4a	2110	2124	HLA-DRB3*02:02	Dengue virus 1	ELISPOT	IFNg release	Positive
539744	NQLAYVVIGLLFMIL	NS4a	2226	2240	HLA-DRB1*15:02	Dengue virus 1	ELISPOT	IFNg release	Positive
738677	DLILEIGKLPQHLTL	NS4a	2099	2113	HLA-DRB1*11:04	Dengue virus 1	ELISPOT	IFNg release	Positive
739034	GLGKTSIGLLCVMAS	NS4a	2171	2185	HLA-DRB1*11:04	Dengue virus 1	ELISPOT	IFNg release	Positive

739085	GVTLFFLSGRGLGKT	NS4a	2161	2175	HLA-DRB1*01:02	Dengue virus 1	ELISPOT	IFNg release	Positive
867109	HWIAASIILEFFLMV	NS4a	2197	2211	HLA-DPB1*04:01	Dengue virus 1	ELISPOT	IFNg release	Positive
867112	IAASIILEFFLMVLL	NS4a	2199	2213	HLA-DQA1*01:01/DQB1*05:01	Dengue virus 1	ELISPOT	IFNg release	Positive
867567	LCVMASSALLWMANV	NS4a	2180	2194	HLA-DQA1*01:02/DQB1*06:02	Dengue virus 1	ELISPOT	IFNg release	Positive
867760	LLCVMASSVLLWMAS	NS4a	2179	2193	HLA-DQA1*01:02/DQB1*06:02	Dengue virus 1	ELISPOT	IFNg release	Positive
868132	MLLALIAVLTGGVTL	NS4a	2150	2164	HLA-DPB1*04:02	Dengue virus 1	ELISPOT	IFNg release	Positive
195114	AGAGLAFSLMKSLGG	NS4b	2476	2490	HLA-DRB1*04:01	Dengue virus 1	ELISPOT	IFNg release	Positive
195193	AVLMLVAHYAIIGPG	NS4b	2355	2369	HLA-DRB1*01:01	Dengue virus 1	ELISPOT	IFNg release	Positive
195308	FMLVAHYAIIGPGLQ	NS4b	2357	2371	HLA-DRB1*07:01	Dengue virus 1	ELISPOT	IFNg release	Positive
195441	HATMLDVDLRPASAW	NS4b	2269	2283	HLA-DRB1*03:01	Dengue virus 1	ELISPOT	IFNg release	Positive
195455	HHHATMLDVDLRPAS	NS4b	2267	2281	HLA-DRB1*03:01	Dengue virus 1	ELISPOT	IFNg release	Positive
195476	IDLDPVVYDAKFEKQ	NS4b	2398	2412	HLA-DRB1*03:01	Dengue virus 1	ELISPOT	IFNg release	Positive
195561	KFWNTTIAVSMANIF	NS4b	2456	2470	HLA-DRB1*07:01	Dengue virus 1	ELISPOT	IFNg release	Positive
195620	LCTSQILLMRTTWAL	NS4b	2421	2435	HLA-DRB1*04:01	Dengue virus 1	ELISPOT	IFNg release	Positive
195637	LGQIMLLILCTSQIL	NS4b	2413	2427	HLA-DRB1*08:02	Dengue virus 1	ELISPOT	IFNg release	Positive
195682	LMLVAHYAIIGPGLQ	NS4b	2357	2371	HLA-DRB1*07:01	Dengue virus 1	ELISPOT	IFNg release	Positive
195760	MLLILCTSQILLMRT	NS4b	2417	2431	HLA-DRB1*07:01	Dengue virus 1	ELISPOT	IFNg release	Positive
195848	QIMLLILCTSQILLM	NS4b	2415	2429	HLA-DRB1*07:01	Dengue virus 1	ELISPOT	IFNg release	Positive
196143	VLMLVAHYAIIGPGL	NS4b	2356	2370	HLA-DRB1*01:01	Dengue virus 1	ELISPOT	IFNg release	Positive
539322	GLAFSLMKSLGGGRR	NS4b	2479	2493	HLA-DRB1*10:01	Dengue virus 1	ELISPOT	IFNg release	Positive
539725	NISLTAIANQAAILM	NS4b	2307	2321	HLA-DRB1*04:03	Dengue virus 1	ELISPOT	IFNg release	Positive
738560	AWTLYAVATTIITPM	NS4b	2282	2296	HLA-DRB1*04:07	Dengue virus 1	ELISPOT	IFNg release	Positive
738686	DLRPASAWTLYAVAT	NS4b	2276	2290	HLA-DRB1*01:02	Dengue virus 1	ELISPOT	IFNg release	Positive
740732	WTLYAVATTVITPMM	NS4b	2283	2297	HLA-DRB1*04:07	Dengue virus 1	ELISPOT	IFNg release	Positive
866002	AIANQAAILMGLDKG	NS4b	2312	2326	HLA-DQA1*01:02/DQB1*06:02	Dengue virus 1	ELISPOT	IFNg release	Positive
868336	PLTLTAAVLMLAAHY	NS4b	2349	2363	HLA-DQA1*01:02/DQB1*06:02	Dengue virus 1	ELISPOT	IFNg release	Positive
869401	VDGIVAIDLDPVVYD	NS4b	2392	2406	HLA-DQA1*01:01/DQB1*05:01	Dengue virus 1	ELISPOT	IFNg release	Positive
1066544	ACLGKSYAQMWQLMY	NS5	3244	3258	HLA-DRB1*04:01	Dengue virus 1	ELISPOT	IFNg release	Positive-Low
195147	AKNGTVMDVISRRDQ	NS5	3076	3090	HLA-DRB1*03:01	Dengue virus 1	ELISPOT	IFNg release	Positive
195150	AKWLWGFLSRNKKPR	NS5	2870	2884	HLA-DRB1*15:01	Dengue virus 1	ELISPOT	IFNg release	Positive
195168	AQMWQLMYFHRRDLR	NS5	3251	3265	HLA-DRB1*13:01	Dengue virus 1	ELISPOT	IFNg release	Positive
195186	ATNIQVAINQVRRLI	NS5	3352	3366	HLA-DRB1*03:01	Dengue virus 1	ELISPOT	IFNg release	Positive
195208	CVYNMMGKREKKLGE	NS5	2942	2956	HLA-DRB1*11:01	Dengue virus 1	ELISPOT	IFNg release	Positive
195212	DDRFATALTALNDMG	NS5	3163	3177	HLA-DRB1*04:01	Dengue virus 1	ELISPOT	IFNg release	Positive
195223	DMGKVRKDIPQWEPS	NS5	3175	3189	HLA-DRB1*03:01	Dengue virus 1	ELISPOT	IFNg release	Positive
195236	EEFTRKVRSNAAIGA	NS5	2889	2903	HLA-DRB1*08:02	Dengue virus 1	ELISPOT	IFNg release	Positive
195354	GEKWKRQLNQLSKSE	NS5	2503	2517	HLA-DRB1*04:01	Dengue virus 1	ELISPOT	IFNg release	Positive
195360	GGMLVRNPLSRNSTH	NS5	2694	2708	HLA-DRB1*08:02	Dengue virus 1	ELISPOT	IFNg release	Positive
195385	GLHKLGYILRDISKI	NS5	3003	3017	HLA-DRB1*08:02	Dengue virus 1	ELISPOT	IFNg release	Positive
195421	GTGNIVSAVNMTSRM	NS5	2716	2730	HLA-DRB1*08:02	Dengue virus 1	ELISPOT	IFNg release	Positive
195438	GYILRDISKIPGGNM	NS5	3008	3022	HLA-DRB1*08:02	Dengue virus 1	ELISPOT	IFNg release	Positive
195464	HWFSRENSLSGVEGE	NS5	2988	3002	HLA-DRB1*04:01	Dengue virus 1	ELISPOT	IFNg release	Positive
195582	KPIDDRFATALTALN	NS5	3160	3174	HLA-DRB1*04:01	Dengue virus 1	ELISPOT	IFNg release	Positive
195655	LLATSIFKLTYQNKV	NS5	3055	3069	HLA-DRB1*07:01	Dengue virus 1	ELISPOT	IFNg release	Positive
195755	MGKVRKDIPQWEPSK	NS5	3176	3190	HLA-DRB1*03:01	Dengue virus 1	ELISPOT	IFNg release	Positive
195781	NEDHWFSRGNSLSGV	NS5	2985	2999	HLA-DRB1*04:01	Dengue virus 1	ELISPOT	IFNg release	Positive
195783	NENYLDYMTSMKRFK	NS5	3368	3382	HLA-DRB1*11:01	Dengue virus 1	ELISPOT	IFNg release	Positive
195786	NGTVMDVISRRDQRG	NS5	3078	3092	HLA-DRB1*03:01	Dengue virus 1	ELISPOT	IFNg release	Positive
195824	NYLDYMTSMKRFKNE	NS5	3370	3384	HLA-DRB1*14:02	Dengue virus 1	ELISPOT	IFNg release	Positive
195852	QLIMKDGREIVVPCR	NS5	3207	3221	HLA-DRB1*03:01	Dengue virus 1	ELISPOT	IFNg release	Positive
195903	RPAKNGTVMDVISRR	NS5	3074	3088	HLA-DRB1*03:01	Dengue virus 1	ELISPOT	IFNg release	Positive
195923	RWLWGFLSRNKKPRI	NS5	2871	2885	HLA-DRB1*15:01	Dengue virus 1	ELISPOT	IFNg release	Positive
195956	SIFKLTYQNKVVRVQ	NS5	3059	3073	HLA-DRB1*09:01	Dengue virus 1	ELISPOT	IFNg release	Positive
196031	TCVYNMMGKREKKLG	NS5	2941	2955	HLA-DRB1*11:01	Dengue virus 1	ELISPOT	IFNg release	Positive
196039	TGNIVSAVNMTSRML	NS5	2717	2731	HLA-DRB1*04:01	Dengue virus 1	ELISPOT	IFNg release	Positive
196191	VTARWLWGFLSRNKK	NS5	2868	2882	HLA-DRB1*15:01	Dengue virus 1	ELISPOT	IFNg release	Positive
196211	WATNIQVAINQVRRL	NS5	3351	3365	HLA-DRB1*03:01	Dengue virus 1	ELISPOT	IFNg release	Positive
196220	WLWGFLSRNKKPRIC	NS5	2872	2886	HLA-DRB1*11:01	Dengue virus 1	ELISPOT	IFNg release	Positive
196241	YQNKVVRVQRPAKNG	NS5	3065	3079	HLA-DRB1*08:02	Dengue virus 1	ELISPOT	IFNg release	Positive
196242	YQNKVVRVQRPAKSG	NS5	3065	3079	HLA-DRB1*08:02	Dengue virus 1	ELISPOT	IFNg release	Positive
539096	ATSIFKLTYQNKVVR	NS5	3057	3071	HLA-DRB1*14:04	Dengue virus 1	ELISPOT	IFNg release	Positive
539107	AVNMTSRMLLNRFTM	NS5	2723	2737	HLA-DRB1*15:02	Dengue virus 1	ELISPOT	IFNg release	Positive
539350	GTAKLRWFVERNLVK	NS5	2551	2565	HLA-DRB1*09:01	Dengue virus 1	ELISPOT	IFNg release	Positive
539396	IDDRFATALTALNDM	NS5	3162	3176	HLA-DPB1*01:01	Dengue virus 1	ELISPOT	IFNg release	Positive
539435	ISGDDCVVKPIDDRFAT	NS5	3152	3168	HLA class II	Dengue virus 1	ICS	IFNg release	Positive
539509	KSEFNIYKRSGIMEV	NS5	2515	2529	HLA-DRB1*15:02	Dengue virus 1	ELISPOT	IFNg release	Positive
539537	LAKAIFKLTYQNKVV	NS5	3056	3070	HLA-DRB1*14:04	Dengue virus 1	ELISPOT	IFNg release	Positive
539691	MTSRMLLNRFTMAHR	NS5	17	31	HLA-DRB1*14:06	Dengue virus 1	ELISPOT	IFNg release	Positive
539700	MVNGVVRLLTKPWDV	NS5	103	117	HLA-DRB1*12:02	Dengue virus 1	ELISPOT	IFNg release	Positive
539702	MVTQIAMTDTTPFGQQR	NS5	2829	2845	HLA class II	Dengue virus 1	ICS	IFNg release	Positive
539862	RMLLNRFTMAHRKPT	NS5	20	34	HLA-DRB1*14:02	Dengue virus 1	ELISPOT	IFNg release	Positive
539905	SHHFHQLIMKDGREI	NS5	3202	3216	HLA-DRB1*13:01	Dengue virus 1	ELISPOT	IFNg release	Positive
540001	TSIFKLTYQNKVVRV	NS5	3058	3072	HLA-DRB1*09:01	Dengue virus 1	ELISPOT	IFNg release	Positive
540018	TYGLNTFTNMEAQLI	NS5	3098	3112	HLA-DRB1*15:06	Dengue virus 1	ELISPOT	IFNg release	Positive
540020	TYGLNTFTNMEVQLI	NS5	3098	3112	HLA-DRB1*04:03	Dengue virus 1	ELISPOT	IFNg release	Positive
540083	VRVQRPAKSGTVMDV	NS5	96	110	HLA-DRB1*13:01	Dengue virus 1	ELISPOT	IFNg release	Positive
540100	VVKPIDDRFATALTALN	NS5	3157	3173	HLA class II	Dengue virus 1	ICS	IFNg release	Positive
540138	WQLMYFHRRDLRLAA	NS5	3254	3268	HLA-DRB1*14:04	Dengue virus 1	ELISPOT	IFNg release	Positive
738814	ENYLDYMTSMKRFKN	NS5	3369	3383	HLA-DRB1*14:02	Dengue virus 1	ELISPOT	IFNg release	Positive
740024	QNKVVRVQRPAKNGT	NS5	3066	3080	HLA-DRB1*14:06	Dengue virus 1	ELISPOT	IFNg release	Positive
740668	VQLIRQMESEGIFLP	NS5	3109	3123	HLA-DRB1*11:04	Dengue virus 1	ELISPOT	IFNg release	Positive
740786	YLDYMTSMKRFKNES	NS5	3371	3385	HLA-DRB1*14:02	Dengue virus 1	ELISPOT	IFNg release	Positive
866481	EAVEDERFWDLVHRE	NS5	2916	2930	HLA-DQA1*01:01/DQB1*05:01	Dengue virus 1	ELISPOT	IFNg release	Positive
867505	KSYAQMWQLMYFHRR	NS5	3248	3262	HLA-DPB1*01:01	Dengue virus 1	ELISPOT	IFNg release	Positive
868240	NKVVRVQRPAKNGTV	NS5	3067	3081	HLA-DRB4*01:01	Dengue virus 1	ELISPOT	IFNg release	Positive
868296	PEHALLATSIFKLTY	NS5	3051	3065	HLA-DPB1*02:01	Dengue virus 1	ELISPOT	IFNg release	Positive
868641	RFLEFEALGFMNEDH	NS5	2974	2988	HLA-DPB1*04:01	Dengue virus 1	ELISPOT	IFNg release	Positive
869091	TAKLRWFVERNLVKP	NS5	2552	2566	HLA-DQA1*01:01/DQB1*05:01	Dengue virus 1	ELISPOT	IFNg release	Positive

CD4 DENV-2 Epitopes

Epitope_ID	AminoAcid_Seq	Protein_Name	Start	End	Allele	Serotype	Method/Technique	Assay Group	Qualitative Measure
1066603	ARNTPFNMLKRERNR	C	8	22	HLA-DRB1*11:01	Dengue virus 2	ELISPOT	IFNg release	Positive-Low
150105	AFLRFLTIPPTAGIL	C	52	66	HLA-DRB1*01:01	Dengue virus 2	ELISPOT	IFNg release	Positive
150247	GMIIMLIPTVMAFHL	C	103	117	HLA-DRB1*01:01	Dengue virus 2	ELISPOT	IFNg release	Positive
150369	LKLFMALVAFLRFLT	C	44	58	HLA-DRB1*01:01	Dengue virus 2	ELISPOT	IFNg release	Positive
150404	LRLFMALVAFLRFLT	C	44	58	HLA-DRB1*11:01	Dengue virus 2	ELISPOT	IFNg release	Positive
180597	LFMALVAFLRFLTIP	C	46	60	HLA class II	Dengue virus 2	ELISPOT	IFNg release	Positive
180768	TIKKSKAINVLRGFR	C	71	85	HLA class II	Dengue virus 2	ELISPOT	IFNg release	Positive
195120	AGMIIMLIPTVMAFH	C	102	116	HLA-DRB1*08:02	Dengue virus 2	ELISPOT	IFNg release	Positive
195121	AGMIIMLIPTVVAFH	C	102	116	HLA-DRB1*08:02	Dengue virus 2	ELISPOT	IFNg release	Positive
195307	FMALVAFLRFLTIPP	C	47	61	HLA-DRB1*11:01	Dengue virus 2	ELISPOT	IFNg release	Positive
195326	FRKEIGRMLNILNRR	C	84	98	HLA-DRB1*04:01	Dengue virus 2	ELISPOT	IFNg release	Positive
195369	GIIIMIIPTVMAFHL	C	103	117	HLA-DRB1*08:02	Dengue virus 2	ELISPOT	IFNg release	Positive
195413	GPLRLFMALVAFLRF	C	42	56	HLA-DRB1*04:01	Dengue virus 2	ELISPOT	IFNg release	Positive
195417	GRMLNILNRRRRTAG	C	89	103	HLA-DRB1*11:01	Dengue virus 2	ELISPOT	IFNg release	Positive
195432	GVIIMMIPTVMAFHL	C	103	117	HLA-DRB1*08:02	Dengue virus 2	ELISPOT	IFNg release	Positive
195489	IGRMLNILNRRRRTA	C	88	102	HLA-DRB1*08:02	Dengue virus 2	ELISPOT	IFNg release	Positive
195490	IGRMLNILSRRRRTA	C	88	102	HLA-DRB1*11:01	Dengue virus 2	ELISPOT	IFNg release	Positive
195496	IIMLIPTVMAFHLTT	C	105	119	HLA-DRB1*01:02	Dengue virus 2	ELISPOT	IFNg release	Positive
195556	KEIGRMLNILNRRRR	C	86	100	HLA-DRB1*08:02	Dengue virus 2	ELISPOT	IFNg release	Positive
195573	KLFMALVAFLRFLTI	C	45	59	HLA-DRB1*15:01	Dengue virus 2	ELISPOT	IFNg release	Positive
195654	LKLYMALVAFLRFLT	C	44	58	HLA-DRB1*15:01	Dengue virus 2	ELISPOT	IFNg release	Positive
195730	LVAFLRFLTIPPTAG	C	50	64	HLA-DRB1*04:01	Dengue virus 2	ELISPOT	IFNg release	Positive
195744	MALVAFLRFLTIPPT	C	48	62	HLA-DRB1*04:01	Dengue virus 2	ELISPOT	IFNg release	Positive
195886	RGFRKEIGRMLNILN	C	82	96	HLA-DRB1*04:01	Dengue virus 2	ELISPOT	IFNg release	Positive
195889	RGPLKLFMALVAFLR	C	41	55	HLA-DRB1*04:01	Dengue virus 2	ELISPOT	IFNg release	Positive
195899	RMLNILNRRRRTAGV	C	71	85	HLA-DRB1*08:02	Dengue virus 2	ELISPOT	IFNg release	Positive
195910	RTAGVIIMMIPTVVA	C	100	114	HLA-DRB1*08:02	Dengue virus 2	ELISPOT	IFNg release	Positive
195911	RTASVIVMLIPTVMA	C	100	114	HLA-DRB1*08:02	Dengue virus 2	ELISPOT	IFNg release	Positive
196024	TAGVIVMLIPTVMAF	C	101	115	HLA-DRB1*08:02	Dengue virus 2	ELISPOT	IFNg release	Positive
196064	TPFNMLKRERNRVST	C	11	25	HLA-DRB1*08:02	Dengue virus 2	ELISPOT	IFNg release	Positive
196084	TVGVIIMLIPTAMAF	C	101	115	HLA-DRB1*08:02	Dengue virus 2	ELISPOT	IFNg release	Positive
196093	VAFLRFLTIPPTAGI	C	51	65	HLA-DRB1*04:01	Dengue virus 2	ELISPOT	IFNg release	Positive
196213	WGTIKKSKAINVLRG	C	69	83	HLA-DRB1*07:01	Dengue virus 2	ELISPOT	IFNg release	Positive
26836	IKKSKAINVLRGFRKEIGRM	C	72	91	HLA class II	Dengue virus 2	ELISPOT	IFNg release	Positive
39098	LRGFRKEIGRML	C	81	92	HLA-DPw4	Dengue virus 2	ICS	IFNg release	Positive
53867	RGFRKEIGRMLNILNRRRS	C	17	35	HLA class II	Dengue virus 2	ELISPOT	IFNg release	Positive
539046	AFLRFLTIPPTVGIL	C	52	66	HLA-DRB1*10:01	Dengue virus 2	ELISPOT	IFNg release	Positive
539051	AFVAFLRFLTIPPTA	C	9	23	HLA-DRB1*15:02	Dengue virus 2	ELISPOT	IFNg release	Positive
539083	ALVAFLRFLTIPPTA	C	49	63	HLA-DRB1*12:02	Dengue virus 2	ELISPOT	IFNg release	Positive
539213	EIGRMLNILNKRRRT	C	87	101	HLA-DRB1*13:01	Dengue virus 2	ELISPOT	IFNg release	Positive
539214	EIGRMLNILNRRRRT	C	87	101	HLA-DRB1*12:02	Dengue virus 2	ELISPOT	IFNg release	Positive
539264	FLRFLTIPPTAGILK	C	53	67	HLA-DRB1*14:02	Dengue virus 2	ELISPOT	IFNg release	Positive
539343	GRMLNIMNRRRRTAG	C	89	103	HLA-DRB1*13:01	Dengue virus 2	ELISPOT	IFNg release	Positive
539410	IKKSKAINVLRGFRK	C	72	86	HLA-DRB1*14:04	Dengue virus 2	ELISPOT	IFNg release	Positive
539514	KSKAINVLRGFRKEI	C	74	88	HLA-DRB1*12:02	Dengue virus 2	ELISPOT	IFNg release	Positive
539549	LFTALVAFLRFLTIP	C	46	60	HLA-DRB1*15:06	Dengue virus 2	ELISPOT	IFNg release	Positive
539612	LQGRGPLKLFMALVA	C	38	52	HLA-DRB1*15:02	Dengue virus 2	ELISPOT	IFNg release	Positive
539618	LRGFRKEIGRMLNIL	C	81	95	HLA-DRB1*09:01	Dengue virus 2	ELISPOT	IFNg release	Positive
539887	RWGTIKKSKAINVLR	C	68	82	HLA-DRB1*08:03	Dengue virus 2	ELISPOT	IFNg release	Positive
540060	VLRGFRKEIGRMLNI	C	80	94	HLA-DRB1*09:01	Dengue virus 2	ELISPOT	IFNg release	Positive
540069	VQQLTKRFSLGMLQG	C	26	40	HLA-DRB1*13:01	Dengue virus 2	ELISPOT	IFNg release	Positive
62853	TAGILKRWGTIKKSKANVL	C	62	81	HLA class II	Dengue virus 2	ELISPOT	IFNg release	Positive
739176	IGRMLNILNKRRRTA	C	88	102	HLA-DRB1*14:02	Dengue virus 2	ELISPOT	IFNg release	Positive
739177	IGRMLNILNRRRRSA	C	88	102	HLA-DRB1*14:02	Dengue virus 2	ELISPOT	IFNg release	Positive
739179	IGRMLNILNRRRRSV	C	88	102	HLA-DRB1*14:02	Dengue virus 2	ELISPOT	IFNg release	Positive
739180	IGRMLNILNRRRRTT	C	88	102	HLA-DRB1*14:02	Dengue virus 2	ELISPOT	IFNg release	Positive
739181	IGRMLNILNRRRRTV	C	42	56	HLA-DRB1*14:02	Dengue virus 2	ELISPOT	IFNg release	Positive
739182	IGRMLNIMNRRRRTA	C	88	102	HLA-DRB1*14:02	Dengue virus 2	ELISPOT	IFNg release	Positive
739183	IGRMMNILNRRRRTA	C	88	102	HLA-DRB1*14:02	Dengue virus 2	ELISPOT	IFNg release	Positive
739879	PFNMLKRERNRVSTV	C	12	26	HLA-DRB1*14:02	Dengue virus 2	ELISPOT	IFNg release	Positive
740399	STPFNMLKRERNRVS	C	10	24	HLA-DRB1*14:02	Dengue virus 2	ELISPOT	IFNg release	Positive
740579	VAFLRFLTISPTAGI	C	51	65	HLA-DRB1*04:07	Dengue virus 2	ELISPOT	IFNg release	Positive
857784	LKRWGTIKKSKAINV	C	66	80	HLA-DRB1*07:01	Dengue virus 2	ELISPOT	IFNg release	Positive-Low
866992	GPLKLYMALVAFLRF	C	42	56	HLA-DRB5*01:01	Dengue virus 2	ELISPOT	IFNg release	Positive
867734	LKGFRKEIGRMLNIL	C	81	95	HLA-DRB3*02:02	Dengue virus 2	ELISPOT	IFNg release	Positive
867910	LRGFRKEIGRMLNIM	C	81	95	HLA-DRB3*02:02	Dengue virus 2	ELISPOT	IFNg release	Positive
867911	LRGFRREIGRMLNIL	C	81	95	HLA-DRB3*02:02	Dengue virus 2	ELISPOT	IFNg release	Positive
868674	RLFMALVAFLRFLTI	C	45	59	HLA-DPB1*04:02	Dengue virus 2	ELISPOT	IFNg release	Positive
150280	HPGFTLMAAILAYTI	PreM	244	258	HLA-DRB1*01:01	Dengue virus 2	ELISPOT	IFNg release	Positive
150490	PGFTIMAAILAYTIG	PreM	245	259	HLA-DRB1*01:01	Dengue virus 2	ELISPOT	IFNg release	Positive
180708	QRIETWILRHPGFTM	PreM	121	135	HLA class II	Dengue virus 2	ELISPOT	IFNg release	Positive-Low
195318	FQKILIFILLTAVAP	PreM	263	277	HLA-DRB1*07:01	Dengue virus 2	ELISPOT	IFNg release	Positive
195320	FQRTLIFILLTAVAP	PreM	263	277	HLA-DRB1*07:01	Dengue virus 2	ELISPOT	IFNg release	Positive
195322	FQRVLIFILLTAVAP	PreM	263	277	HLA-DRB1*07:01	Dengue virus 2	ELISPOT	IFNg release	Positive
195448	HFQRALIFILLTAVA	PreM	262	276	HLA-DRB1*07:01	Dengue virus 2	ELISPOT	IFNg release	Positive
195503	ILRHPGFTIMAAILA	PreM	127	141	HLA-DRB1*07:01	Dengue virus 2	ELISPOT	IFNg release	Positive
195504	ILRHPGFTLMAAILA	PreM	241	255	HLA-DRB1*15:01	Dengue virus 2	ELISPOT	IFNg release	Positive
195702	LRHPGFTIMAAILAY	PreM	242	256	HLA-DRB1*11:01	Dengue virus 2	ELISPOT	IFNg release	Positive
195862	QRALIFILLTAVAPS	PreM	264	278	HLA-DRB1*15:01	Dengue virus 2	ELISPOT	IFNg release	Positive
195863	QRVLIFILLTAVAPS	PreM	264	278	HLA-DRB1*08:02	Dengue virus 2	ELISPOT	IFNg release	Positive
195892	RIETWILRHPGFTIM	PreM	122	136	HLA-DRB1*15:01	Dengue virus 2	ELISPOT	IFNg release	Positive
196045	THFQRALIFILLTAV	PreM	261	275	HLA-DRB1*07:01	Dengue virus 2	ELISPOT	IFNg release	Positive
196141	VLIFILLTAIAPSMT	PreM	266	280	HLA-DRB1*08:02	Dengue virus 2	ELISPOT	IFNg release	Positive
196232	YFQRVLIFILLTAVT	PreM	262	276	HLA-DRB1*07:01	Dengue virus 2	ELISPOT	IFNg release	Positive
539881	RVLIFILLTAIAPSM	PreM	265	279	HLA-DRB1*15:06	Dengue virus 2	ELISPOT	IFNg release	Positive

539943	SVALVPHVGMGLETR	PreM	206	220	HLA-DRB1*15:02	Dengue virus 2	ELISPOT	IFNg release	Positive
540130	WILRHPGFTIMAAIL	PreM	126	140	HLA-DRB1*15:06	Dengue virus 2	ELISPOT	IFNg release	Positive
739425	LAYTIGTTHFQRALI	PreM	254	268	HLA-DRB1*04:07	Dengue virus 2	ELISPOT	IFNg release	Positive
866485	EDGVNMCTLMAIDLG	PreM	142	156	HLA-DQA1*01:02/DQB1*06:02	Dengue virus 2	ELISPOT	IFNg release	Positive
868650	RHPGFTLMAAILAYT	PreM	243	257	HLA-DQA1*01:02/DQB1*06:02	Dengue virus 2	ELISPOT	IFNg release	Positive
144940	RHVLGRLITVNPIVT	E	345	359	HLA-DRB1*08:02	Dengue virus 2	ELISPOT	IFNg release	Positive
180564	KKQDVVVLGSQEGAM	E	526	540	HLA class II	Dengue virus 2	ELISPOT	IFNg release	Positive
180568	KNPHAKKQDVVVLGS	E	521	535	HLA class II	Dengue virus 2	ELISPOT	IFNg release	Positive
180590	LDFELIKTEAKQPAT	E	41	55	HLA class II	Dengue virus 2	ELISPOT	IFNg release	Positive
180820	WLVHRQWFLDLPLPW	E	196	210	HLA class II	Dengue virus 2	ELISPOT	IFNg release	Positive
195351	GATEIQMSSGNLLFT	E	266	280	HLA-DRB1*03:01	Dengue virus 2	ELISPOT	IFNg release	Positive
195366	GHLKCRLRMDKLQLK	E	281	295	HLA-DRB1*03:01	Dengue virus 2	ELISPOT	IFNg release	Positive
195407	GMNSRSTSLSVSLVL	E	467	481	HLA-DRB1*07:01	Dengue virus 2	ELISPOT	IFNg release	Positive
195638	LGRLITVNPIVTEKD	E	628	642	HLA-DRB1*08:02	Dengue virus 2	ELISPOT	IFNg release	Positive
195716	LSVSLVLVGVVTLYL	E	755	769	HLA-DRB1*15:01	Dengue virus 2	ELISPOT	IFNg release	Positive
195793	NLEYTIMITPHSGEE	E	134	148	HLA-DRB1*04:01	Dengue virus 2	ELISPOT	IFNg release	Positive
195952	SGVSWTMKILIGVII	E	449	463	HLA-DRB1*15:01	Dengue virus 2	ELISPOT	IFNg release	Positive
196022	SWTMKILIGVIITWI	E	452	466	HLA-DRB1*08:02	Dengue virus 2	ELISPOT	IFNg release	Positive
196077	TSLSVSLVLVGVVTL	E	753	767	HLA-DRB1*07:01	Dengue virus 2	ELISPOT	IFNg release	Positive
196140	VLGRLITVNPIVTEK	E	627	641	HLA-DRB1*08:02	Dengue virus 2	ELISPOT	IFNg release	Positive
196215	WIQKETLVTFKNPHA	E	231	245	HLA-DRB1*08:02	Dengue virus 2	ELISPOT	IFNg release	Positive
539236	ENLEYTIVITPHSGE	E	133	147	HLA-DRB1*04:07	Dengue virus 2	ELISPOT	IFNg release	Positive
539252	FETTMRGAKRMAILG	E	402	416	HLA-DRB1*15:02	Dengue virus 2	ELISPOT	IFNg release	Positive
539377	HAKKQDVVVLGSQEGAMH	E	244	261	HLA class II	Dengue virus 2	ICS	IFNg release	Positive
539427	IQKKTLVTFKNPHAK	E	512	526	HLA-DRB1*04:03	Dengue virus 2	ELISPOT	IFNg release	Positive
539462	KEMLVTFKNPHAKRQ	E	514	528	HLA-DRB1*04:03	Dengue virus 2	ELISPOT	IFNg release	Positive
539464	KETLVTFKNPHAKKQ	E	234	248	HLA-DRB1*10:01	Dengue virus 2	ELISPOT	IFNg release	Positive
539479	KKTLVTFKNPHAKKQ	E	514	528	HLA-DRB1*14:04	Dengue virus 2	ELISPOT	IFNg release	Positive
539629	LSVTLVLVGFVTLYL	E	475	489	HLA-DRB1*15:06	Dengue virus 2	ELISPOT	IFNg release	Positive
539648	LVTFKNPHAKKQDVV	E	237	251	HLA-DRB1*15:02	Dengue virus 2	ELISPOT	IFNg release	Positive
539689	MRGAKRMAILGDTAW	E	406	420	HLA-DRB1*15:06	Dengue virus 2	ELISPOT	IFNg release	Positive
539733	NLEYTIVITPHSGEE	E	134	148	HLA-DRB1*04:07	Dengue virus 2	ELISPOT	IFNg release	Positive
539806	QKETLVTFKNPHAKK	E	513	527	HLA-DRB1*04:03	Dengue virus 2	ELISPOT	IFNg release	Positive
65140	TLVTFKNPHAKKQDV	E	236	250	HLA class II	Dengue virus 2	ELISPOT	IFNg release	Positive
738866	EYTIVITPHSGEEHA	E	136	150	HLA-DRB1*04:07	Dengue virus 2	ELISPOT	IFNg release	Positive
739010	GGVFTSIGKALHQVF	E	706	720	HLA-DRB1*14:02	Dengue virus 2	ELISPOT	IFNg release	Positive
740315	SLSVSLVLVGVVTLY	E	754	768	HLA-DRB1*01:02	Dengue virus 2	ELISPOT	IFNg release	Positive
741744	MRCIGISNRDFVEGVSGGSW	E	281	300	HLA class II	Dengue virus 2	ELISPOT	IFNg release	Positive
866193	ATEIQMSSGNLLFTG	E	257	271	HLA-DRB3*02:02	Dengue virus 2	ELISPOT	IFNg release	Positive
866243	AWMVHRQWFLDLPLP	E	205	219	HLA-DQA1*01:01/DQB1*05:01	Dengue virus 2	ELISPOT	IFNg release	Positive
867375	KAWLVHRQWFLDLPF	E	204	218	HLA-DQA1*01:01/DQB1*05:01	Dengue virus 2	ELISPOT	IFNg release	Positive
868347	PPFGDSYVIIGVEPG	E	371	385	HLA-DQA1*03:01/DQB1*03:02	Dengue virus 2	ELISPOT	IFNg release	Positive
868713	RTGLDFNEMVLLQME	E	178	192	HLA-DQA1*01:02/DQB1*06:02	Dengue virus 2	ELISPOT	IFNg release	Positive
869123	TEKDSPVNIEAEPPF	E	349	363	HLA-DQA1*03:01/DQB1*03:02	Dengue virus 2	ELISPOT	IFNg release	Positive
869152	TGLDFNEMVLLQMED	E	189	203	HLA-DQA1*03:01/DQB1*03:02	Dengue virus 2	ELISPOT	IFNg release	Positive
869153	TGLDFNEMVLLQMEN	E	179	193	HLA-DQA1*03:01/DQB1*03:02	Dengue virus 2	ELISPOT	IFNg release	Positive
167759	IMTGDIKGIMQA	NS1	88	99	HLA-DQ6	Dengue virus 2	ELISPOT	IFNg release	Positive
180396	DGCWYGMEIRPLKEKEENLVNS	NS1	1102	1123	HLA class II	Dengue virus 2	ICS	IFNg release	Positive
180603	LKYSWKTWGKAKMLS	NS1	111	125	HLA class II	Dengue virus 2	ELISPOT	IFNg release	Positive
180612	LNDTWKIEKASFIEV	NS1	981	995	HLA class II	Dengue virus 2	ELISPOT	IFNg release	Positive
195458	HNQTFLIDGPETAEC	NS1	904	918	HLA-DRB1*04:01	Dengue virus 2	ELISPOT	IFNg release	Positive
195576	KLTIMTGDIKGIMQA	NS1	85	99	HLA-DRB1*03:01	Dengue virus 2	ELISPOT	IFNg release	Positive
195583	KQDVFCDSKLMSAAI	NS1	949	963	HLA-DRB1*03:01	Dengue virus 2	ELISPOT	IFNg release	Positive
195845	QDVFCDSKLMSAAIK	NS1	175	189	HLA-DRB1*03:01	Dengue virus 2	ELISPOT	IFNg release	Positive
195880	REKQDVFCDSKLMSA	NS1	947	961	HLA-DRB1*03:01	Dengue virus 2	ELISPOT	IFNg release	Positive
195954	SHNQTFLIDGPETAE	NS1	903	917	HLA-DRB1*04:01	Dengue virus 2	ELISPOT	IFNg release	Positive
196133	VKLTIMTGDIKGIMQ	NS1	84	98	HLA-DRB1*03:01	Dengue virus 2	ELISPOT	IFNg release	Positive
539708	NDTWKMEKASFIEVK	NS1	982	996	HLA-DRB1*09:01	Dengue virus 2	ELISPOT	IFNg release	Positive
539828	QYKFQPESPSKLASA	NS1	806	820	HLA-DRB3*02:02	Dengue virus 2	ELISPOT	IFNg release	Positive
738586	CGIRSVTRLENLMWK	NS1	55	69	HLA-DRB1*11:04	Dengue virus 2	ELISPOT	IFNg release	Positive
738766	EGICGIRSVTRLENL	NS1	52	66	HLA-DRB1*11:04	Dengue virus 2	ELISPOT	IFNg release	Positive
739191	IKGIMHAGKRSLRPQ	NS1	868	882	HLA-DRB1*14:02	Dengue virus 2	ELISPOT	IFNg release	Positive
739192	IKGIMQAGKRSLRPQ	NS1	93	107	HLA-DRB1*14:06	Dengue virus 2	ELISPOT	IFNg release	Positive
739296	KGIMQAGKRSLRPQP	NS1	94	108	HLA-DRB1*14:06	Dengue virus 2	ELISPOT	IFNg release	Positive
866460	DYGFGVFTTNIWLKL	NS1	932	946	HLA-DPB1*02:01	Dengue virus 2	ELISPOT	IFNg release	Positive
866699	FGVFTTNIWLKLREK	NS1	935	949	HLA-DPB1*04:01	Dengue virus 2	ELISPOT	IFNg release	Positive
866829	GFGFFTTNIWLKLRE	NS1	934	948	HLA-DPB1*01:01	Dengue virus 2	ELISPOT	IFNg release	Positive
866830	GFGVFTTNIWLKLKE	NS1	934	948	HLA-DPB1*04:01	Dengue virus 2	ELISPOT	IFNg release	Positive
866831	GFGVFTTNIWLKLRE	NS1	934	948	HLA-DPB1*04:01	Dengue virus 2	ELISPOT	IFNg release	Positive
867040	HADMGYWIESALNDT	NS1	970	984	HLA-DQA1*01:01/DQB1*05:01	Dengue virus 2	ELISPOT	IFNg release	Positive
869722	YGFGVFTTNIWLKLR	NS1	933	947	HLA-DPB1*04:01	Dengue virus 2	ELISPOT	IFNg release	Positive
150131	ALTIKGLNPTAIFLT	NS2a	1308	1322	HLA-DRB1*09:01	Dengue virus 2	ELISPOT	IFNg release	Positive
150245	GMGVTYLALLAAYKV	NS2a	1196	1210	HLA-DRB1*01:01	Dengue virus 2	ELISPOT	IFNg release	Positive
150273	GVTYLALLAAFKVRP	NS2a	1198	1212	HLA-DRB1*01:01	Dengue virus 2	ELISPOT	IFNg release	Positive
167819	SSQQKTDWIPLALTIKGLNP	NS2a	184	203	HLA-DP	Dengue virus 2	ELISPOT	IFNg release	Positive
195153	ALLAAFKVRPTFAAG	NS2a	1203	1217	HLA-DRB1*08:02	Dengue virus 2	ELISPOT	IFNg release	Positive
195207	CTILAVVSVSPLLLT	NS2a	1144	1158	HLA-DRB1*07:01	Dengue virus 2	ELISPOT	IFNg release	Positive
195269	ERYQLAVTITAILCV	NS2a	1268	1282	HLA-DRB1*07:01	Dengue virus 2	ELISPOT	IFNg release	Positive
195406	GMMVLKIVRNMEKYQ	NS2a	1257	1271	HLA-DRB1*08:02	Dengue virus 2	ELISPOT	IFNg release	Positive
195523	IPLVLIIKGLNPTAI	NS2a	1319	1333	HLA-DRB1*08:02	Dengue virus 2	ELISPOT	IFNg release	Positive
195567	KHAILLVAVSFVTLI	NS2a	1157	1171	HLA-DRB1*07:01	Dengue virus 2	ELISPOT	IFNg release	Positive
195610	LALGMMVLKIVRNME	NS2a	1254	1268	HLA-DRB1*13:01	Dengue virus 2	ELISPOT	IFNg release	Positive
195615	LAVVSVSPLLLTSSQ	NS2a	1186	1200	HLA-DRB1*07:01	Dengue virus 2	ELISPOT	IFNg release	Positive
195650	LKIVRNMEKYQLAVT	NS2a	1261	1275	HLA-DRB1*13:01	Dengue virus 2	ELISPOT	IFNg release	Positive
195688	LNPTAIFLTTLSRTS	NS2a	1328	1342	HLA-DRB1*07:01	Dengue virus 2	ELISPOT	IFNg release	Positive
195712	LSLTFIRSTTPLIMA	NS2a	1275	1289	HLA-DRB1*07:01	Dengue virus 2	ELISPOT	IFNg release	Positive
195752	MEKYQLAVTIMAILC	NS2a	1154	1168	HLA-DRB1*04:07	Dengue virus 2	ELISPOT	IFNg release	Positive
195753	MEKYQLAVTIMAISC	NS2a	1267	1281	HLA-DRB1*10:01	Dengue virus 2	ELISPOT	IFNg release	Positive

195763	MMVLKIVRKMERYQL	NS2a	1258	1272	HLA-DRB1*03:01	Dengue virus 2	ELISPOT	IFNg release	Positive
195798	NMEKYQLAVTIMAIL	NS2a	1266	1280	HLA-DRB1*04:01	Dengue virus 2	ELISPOT	IFNg release	Positive
195799	NMEKYQLAVTIMAIS	NS2a	1266	1280	HLA-DRB1*04:01	Dengue virus 2	ELISPOT	IFNg release	Positive
196174	VRNMEKYQLAVTIMA	NS2a	1264	1278	HLA-DRB1*13:01	Dengue virus 2	ELISPOT	IFNg release	Positive
196217	WKVSCTILAAVSVSP	NS2a	1292	1306	HLA-DRB1*04:01	Dengue virus 2	ELISPOT	IFNg release	Positive
196218	WKVSCTILAVVSVSP	NS2a	1292	1306	HLA-DRB1*04:01	Dengue virus 2	ELISPOT	IFNg release	Positive
196237	YQLAVTIMAISCVPN	NS2a	1270	1284	HLA-DRB1*08:02	Dengue virus 2	ELISPOT	IFNg release	Positive
539077	ALGIMVLKMVRSMEK	NS2a	1255	1269	HLA-DRB1*12:02	Dengue virus 2	ELISPOT	IFNg release	Positive
539082	ALTIKGLNPTAILLT	NS2a	1322	1336	HLA-DRB1*09:01	Dengue virus 2	ELISPOT	IFNg release	Positive
539108	AVSFVTLITGNMSFR	NS2a	1164	1178	HLA-DRB1*10:01	Dengue virus 2	ELISPOT	IFNg release	Positive
539110	AWALGMMVLKIVRNM	NS2a	1253	1267	HLA-DRB1*13:01	Dengue virus 2	ELISPOT	IFNg release	Positive
539231	EMLRTRVGTKHAILL	NS2a	1148	1162	HLA-DRB1*08:03	Dengue virus 2	ELISPOT	IFNg release	Positive
539392	IALGIMVLKMVRSME	NS2a	1254	1268	HLA-DRB1*13:01	Dengue virus 2	ELISPOT	IFNg release	Positive
539474	KIVRNMEKYQLAVTI	NS2a	1262	1276	HLA-DRB1*13:01	Dengue virus 2	ELISPOT	IFNg release	Positive
539540	LALLAAFKVRPTFAA	NS2a	1202	1216	HLA-DRB1*14:04	Dengue virus 2	ELISPOT	IFNg release	Positive
539556	LGIMILKLVRKMEKY	NS2a	1256	1270	HLA-DRB1*12:02	Dengue virus 2	ELISPOT	IFNg release	Positive
539557	LGIMVLKIVRSMEKY	NS2a	1256	1270	HLA-DRB1*12:02	Dengue virus 2	ELISPOT	IFNg release	Positive
539561	LGMMILKIVRNMEKY	NS2a	1256	1270	HLA-DRB1*13:01	Dengue virus 2	ELISPOT	IFNg release	Positive
539562	LGMMVLKIVRNMEKY	NS2a	1256	1270	HLA-DRB1*12:02	Dengue virus 2	ELISPOT	IFNg release	Positive
539686	MMVLKIVRNMEKYQL	NS2a	1244	1258	HLA-DRB1*14:06	Dengue virus 2	ELISPOT	IFNg release	Positive
539699	MVLKMVRNMEKYQLA	NS2a	1259	1273	HLA-DRB1*13:01	Dengue virus 2	ELISPOT	IFNg release	Positive
539706	NAWKVSCTILAAVSV	NS2a	1290	1304	HLA-DRB1*09:01	Dengue virus 2	ELISPOT	IFNg release	Positive
539789	PTAIFLTTLSRTSKK	NS2a	1316	1330	HLA-DRB1*04:03	Dengue virus 2	ELISPOT	IFNg release	Positive
539893	SCTILAAVSVSPLLL	NS2a	1295	1309	HLA-DRB1*10:01	Dengue virus 2	ELISPOT	IFNg release	Positive
539959	TAIFLTTLSRTSKKR	NS2a	1317	1331	HLA-DRB1*14:02	Dengue virus 2	ELISPOT	IFNg release	Positive
540026	VAVSFVTLITGNMSF	NS2a	1163	1177	HLA-DRB1*10:01	Dengue virus 2	ELISPOT	IFNg release	Positive
540086	VSFVTLITGNMSFRD	NS2a	1165	1179	HLA-DRB1*10:01	Dengue virus 2	ELISPOT	IFNg release	Positive
540094	VTYLALLAAFKVRPT	NS2a	1199	1213	HLA-DRB1*12:02	Dengue virus 2	ELISPOT	IFNg release	Positive
540154	YLALLAAFKVRPTFA	NS2a	1201	1215	HLA-DRB1*14:04	Dengue virus 2	ELISPOT	IFNg release	Positive
740700	VVSVSPLLLTSSQQK	NS2a	1188	1202	HLA-DRB1*01:02	Dengue virus 2	ELISPOT	IFNg release	Positive
866586	EKYQLAVTIMAISCV	NS2a	1268	1282	HLA-DQA1*01:02/DQB1*06:02	Dengue virus 2	ELISPOT	IFNg release	Positive
866978	GMALFLEEMLRTRVG	NS2a	1141	1155	HLA-DQA1*01:01/DQB1*05:01	Dengue virus 2	ELISPOT	IFNg release	Positive
867216	ILCVPNAVILQNAWK	NS2a	1279	1293	HLA-DQA1*01:02/DQB1*06:02	Dengue virus 2	ELISPOT	IFNg release	Positive
867571	LCVPNAVILQNAWKV	NS2a	1280	1294	HLA-DRB4*01:01	Dengue virus 2	ELISPOT	IFNg release	Positive
868918	SLGVLGMALFLEEML	NS2a	1136	1150	HLA-DQA1*05:01/DQB1*02:01	Dengue virus 2	ELISPOT	IFNg release	Positive
869200	TKHAILLVAVSFVTL	NS2a	1156	1170	HLA-DPB1*04:01	Dengue virus 2	ELISPOT	IFNg release	Positive
150433	MAVGMVSILASSLLK	NS2b	1354	1368	HLA-DRB1*01:01	Dengue virus 2	ELISPOT	IFNg release	Positive
167732	DQAEISGSSPILSITISEDG	NS2b	63	82	HLA-DP	Dengue virus 2	ELISPOT	IFNg release	Positive
195239	EEQTLTILIRTGLLV	NS2b	91	105	HLA-DRB1*15:01	Dengue virus 2	ELISPOT	IFNg release	Positive
195512	IMAVGMVSILASSLL	NS2b	8	22	HLA-DRB1*07:01	Dengue virus 2	ELISPOT	IFNg release	Positive
195723	LTILIRTGLLVISGL	NS2b	1440	1454	HLA-DRB1*15:06	Dengue virus 2	ELISPOT	IFNg release	Positive
195869	QTLTILIRTGLLVIS	NS2b	1438	1452	HLA-DRB1*15:01	Dengue virus 2	ELISPOT	IFNg release	Positive
195913	RTGLLVISGVFPVSI	NS2b	1445	1459	HLA-DRB1*08:02	Dengue virus 2	ELISPOT	IFNg release	Positive
195992	SSLLKNDIPMTGPLV	NS2b	19	33	HLA-DRB1*03:01	Dengue virus 2	ELISPOT	IFNg release	Positive
539574	LIRTGLLVISGVFPV	NS2b	1443	1457	HLA-DRB1*13:01	Dengue virus 2	ELISPOT	IFNg release	Positive
866986	GMVSILASSLLKNDI	NS2b	1357	1371	HLA-DPB1*01:01	Dengue virus 2	ELISPOT	IFNg release	Positive
1067121	GKVVGLYGNGVVTRS	NS3	1619	1633	HLA-DRB1*15:01	Dengue virus 2	ELISPOT	IFNg release	Positive
12372	EHTGREIVDLMCHAT	NS3	250	264	HLA class II	Dengue virus 2	ELISPOT	IFNg release	Positive
12606	EIVDLMCHATFTMRL	NS3	255	269	HLA class II	Dengue virus 2	ELISPOT	IFNg release	Positive
1392191	GREIVDLMCHATFTM	NS3	1728	1742		Dengue virus 2	biological activity	activation	Positive
139758	GKTKRYLPAIVREAI	NS3	1673	1687	HLA-DRB1*09:01	Dengue virus 2	ELISPOT	IFNg release	Positive
139762	GLPIRYQTPAIRAEH	NS3	1712	1726	HLA-DRB1*04:03	Dengue virus 2	ELISPOT	IFNg release	Positive
180698	PNYNLIIMDEAHFTD	NS3	1751	1765	HLA class II	Dengue virus 2	ELISPOT	IFNg release	Positive
180807	VTDFKGKTVWFVPSI	NS3	1826	1840	HLA class II	Dengue virus 2	ELISPOT	IFNg release	Positive-Low
195154	ALRGLPIRYQTPAIR	NS3	1709	1723	HLA-DRB1*08:02	Dengue virus 2	ELISPOT	IFNg release	Positive
195295	FHTMWHVTRGAVLMH	NS3	1521	1535	HLA-DRB1*07:01	Dengue virus 2	ELISPOT	IFNg release	Positive
195420	GTFHTMWHVTRGAVL	NS3	1519	1533	HLA-DRB1*07:01	Dengue virus 2	ELISPOT	IFNg release	Positive
195564	KGKVVGLYGNGVVTR	NS3	1618	1632	HLA-DRB1*15:01	Dengue virus 2	ELISPOT	IFNg release	Positive
195765	MRLLSPVRVPNYNLI	NS3	1742	1756	HLA-DRB1*13:01	Dengue virus 2	ELISPOT	IFNg release	Positive
195888	RGLRTLILAPTRVVA	NS3	1689	1703	HLA-DRB1*08:02	Dengue virus 2	ELISPOT	IFNg release	Positive
21115	GLRTLILAPTRVVAA	NS3	1690	1704	HLA-DRB1*04:03	Dengue virus 2	ELISPOT	IFNg release	Positive
24916	HTGREIVDLMCHATE	NS3	251	265	HLA-DPw2	Dengue virus 2	ICS	IFNg release	Positive
26965	ILAPTRVVAAEMEEA	NS3	1695	1709	HLA-DQA1*03:01/DQB1*03:02	Dengue virus 2	ELISPOT	IFNg release	Positive
30396	KEGERKKLKPRWLDA	NS3	584	598	HLA class II	Dengue virus 2	ICS	IFNg release	Positive
31726	KKVIQLSRKTFDSEY	NS3	1855	1869	HLA-DRB1*12:02	Dengue virus 2	ELISPOT	IFNg release	Positive
34231	KVVGLYGNGVVTRSG	NS3	145	159	HLA class II	Dengue virus 2	ELISPOT	IFNg release	Positive
36560	LIIMDEAHFTDPASI	NS3	1755	1769	HLA-DQA1*01:01/DQB1*05:01	Dengue virus 2	ELISPOT	IFNg release	Positive
39103	LRGLPIRYQTPAIRA	NS3	1696	1710	HLA-DRB1*14:02	Dengue virus 2	ELISPOT	IFNg release	Positive
39225	LRTLILAPTRVVAAE	NS3	1691	1705	HLA-DRB1*12:02	Dengue virus 2	ELISPOT	IFNg release	Positive
4152	ARGYISTRVEMGEAA	NS3	1757	1771	HLA-DRB1*04:07	Dengue virus 2	ELISPOT	IFNg release	Positive
49766	PTRVVAAEMEEALRG	NS3	223	237	HLA-DRB1*15:01	Dengue virus 2	ICS	IFNg release	Positive
539025	AAGIFMTATPPGSRD	NS3	1784	1798	HLA-DRB1*04:03	Dengue virus 2	ELISPOT	IFNg release	Positive
539041	AEMEEALRGLPIRYQ	NS3	1704	1718	HLA-DRB1*13:01	Dengue virus 2	ELISPOT	IFNg release	Positive
539200	EDDIFRKKRLTIMDL	NS3	1654	1668	HLA-DRB1*13:01	Dengue virus 2	ELISPOT	IFNg release	Positive
539203	EERVILAGPMPVTHS	NS3	1899	1913	HLA-DRB1*09:01	Dengue virus 2	ELISPOT	IFNg release	Positive
539283	GAYRIKQRGILGYSQ	NS3	1496	1510	HLA-DRB1*08:03	Dengue virus 2	ELISPOT	IFNg release	Positive
539289	GEAAGIFMTATPPGS	NS3	1782	1796	HLA-DRB1*04:03	Dengue virus 2	ELISPOT	IFNg release	Positive
539448	IVREAIKRGLRTLIL	NS3	1682	1696	HLA-DRB1*12:02	Dengue virus 2	ELISPOT	IFNg release	Positive
539467	KGKTVWFVPSIKAGN	NS3	1830	1844	HLA-DRB1*08:03	Dengue virus 2	ELISPOT	IFNg release	Positive
539573	LILAPTRVVAAEMEE	NS3	1694	1708	HLA-DRB1*13:01	Dengue virus 2	ELISPOT	IFNg release	Positive
539713	NGKKVIQLSRKTFDS	NS3	1853	1867	HLA-DRB1*12:02	Dengue virus 2	ELISPOT	IFNg release	Positive
539850	RGLPIRYQTPAIRAE	NS3	1711	1725	HLA-DRB1*13:01	Dengue virus 2	ELISPOT	IFNg release	Positive
539857	RKNGKKVIQLSRKTF	NS3	1851	1865	HLA-DRB1*08:03	Dengue virus 2	ELISPOT	IFNg release	Positive
540056	VLMHKGKRIEPSWAD	NS3	1518	1532	HLA-DRB1*13:01	Dengue virus 2	ELISPOT	IFNg release	Positive
54390	RKLTIMDLHPGAGKT	NS3	618	632	HLA class II	Dengue virus 2	ICS	IFNg release	Positive
63672	TFHTMWHVTRGAVLM	NS3	45	59	HLA class II	Dengue virus 2	ELISPOT	IFNg release	Positive
64666	TKRYLPAIVREAIKR	NS3	200	214	HLA class II	Dengue virus 2	ELISPOT	IFNg release	Positive
739148	IDKKGKVVGLYGNGV	NS3	1615	1629	HLA-DRB1*01:02	Dengue virus 2	ELISPOT	IFNg release	Positive

740081	REAIKRGLRTLILAP	NS3	1670	1684	HLA-DRB1*14:06	Dengue virus 2	ELISPOT	IFNg release	Positive
740193	RTLILAPTRVVAAEM	NS3	1678	1692	HLA-DRB1*14:06	Dengue virus 2	ELISPOT	IFNg release	Positive
866477	EALRGLPIRYQTPAI	NS3	1708	1722	HLA-DRB4*01:01	Dengue virus 2	ELISPOT	IFNg release	Positive
868183	MWHVTRGAVLMHRGK	NS3	1524	1538	HLA-DRB3*02:02	Dengue virus 2	ELISPOT	IFNg release	Positive
868724	RTNDWDFVVTTDISE	NS3	1873	1887	HLA-DQA1*03:01/DQB1*03:02	Dengue virus 2	ELISPOT	IFNg release	Positive
868734	RYLPAIVREAIKRGL	NS3	1677	1691	HLA-DRB4*01:01	Dengue virus 2	ELISPOT	IFNg release	Positive
869224	TLILAPTRVVAAEME	NS3	1693	1707	HLA-DQA1*01:02/DQB1*06:02	Dengue virus 2	ELISPOT	IFNg release	Positive
8911	DKKGKVVGLYGNGVV	NS3	141	155	HLA-DRB1*15:01	Dengue virus 2	ICS	IFNg release	Positive
150192	ETLLLLTLLAAVTGG	NS4a	2146	2160	HLA-DRB1*01:01	Dengue virus 2	ELISPOT	IFNg release	Positive
150362	LETLLLLTLLATVTG	NS4a	2145	2159	HLA-DRB1*01:01	Dengue virus 2	ELISPOT	IFNg release	Positive
167780	LTLNLITEMGRLPTFMTQKA	NS4a	2	21	HLA class II	Dengue virus 2	ELISPOT	IFNg release	Positive
195107	AASIILEFFLIVLLI	NS4a	2185	2199	HLA-DRB1*15:01	Dengue virus 2	ELISPOT	IFNg release	Positive
195355	GGIFLFLMSGKGIGK	NS4a	2145	2159	HLA-DRB1*11:01	Dengue virus 2	ELISPOT	IFNg release	Positive
195356	GGIFLFLMSGRGIGK	NS4a	2159	2173	HLA-DRB1*11:01	Dengue virus 2	ELISPOT	IFNg release	Positive
195414	GRAYNHALSELPETL	NS4a	2131	2145	HLA-DRB1*04:01	Dengue virus 2	ELISPOT	IFNg release	Positive
195635	LGMCCIITASILLWY	NS4a	2176	2190	HLA-DRB1*07:01	Dengue virus 2	ELISPOT	IFNg release	Positive
195767	MTLGMCCIITASILL	NS4a	2160	2174	HLA-DRB1*07:01	Dengue virus 2	ELISPOT	IFNg release	Positive
195810	NQLTYVVIAILTLVA	NS4a	2225	2239	HLA-DRB1*07:01	Dengue virus 2	ELISPOT	IFNg release	Positive
196054	TLETLLLLTLLATVT	NS4a	2144	2158	HLA-DRB1*15:01	Dengue virus 2	ELISPOT	IFNg release	Positive
196089	TVTGGIFLFLMSGKG	NS4a	2142	2156	HLA-DRB1*11:01	Dengue virus 2	ELISPOT	IFNg release	Positive
539609	LPTFMTQKARNALDN	NS4a	2092	2106	HLA-DRB1*10:01	Dengue virus 2	ELISPOT	IFNg release	Positive
539801	QDNQLTYVVIAILTV	NS4a	2223	2237	HLA-DRB1*15:02	Dengue virus 2	ELISPOT	IFNg release	Positive
539804	QIQPHWIAASIILEF	NS4a	2192	2206	HLA-DRB1*13:01	Dengue virus 2	ELISPOT	IFNg release	Positive
540148	YAQIQPHWIAASIIL	NS4a	2176	2190	HLA-DRB1*13:01	Dengue virus 2	ELISPOT	IFNg release	Positive
738567	AYNHALSELPETLET	NS4a	2119	2133	HLA-DRB1*04:07	Dengue virus 2	ELISPOT	IFNg release	Positive
150611	TAALLLLVAHYAIIG	NS4b	2351	2365	HLA-DRB1*01:01	Dengue virus 2	ELISPOT	IFNg release	Positive
150636	TLTAALLLLVAHYAI	NS4b	2335	2349	HLA-DRB1*01:01	Dengue virus 2	ELISPOT	IFNg release	Positive
195115	AGAGLLFSIMKNTTN	NS4b	2474	2488	HLA-DRB1*11:01	Dengue virus 2	ELISPOT	IFNg release	Positive
195118	AGLLFSIMRNTTSAR	NS4b	2476	2490	HLA-DRB1*04:01	Dengue virus 2	ELISPOT	IFNg release	Positive
195270	ESESNILDIDLRPAS	NS4b	2265	2279	HLA-DRB1*03:01	Dengue virus 2	ELISPOT	IFNg release	Positive
195347	GAGLLFSIMKNTTNT	NS4b	2475	2489	HLA-DRB1*11:01	Dengue virus 2	ELISPOT	IFNg release	Positive
195387	GLLFFIMKNTTNTRR	NS4b	2477	2491	HLA-DRB1*04:01	Dengue virus 2	ELISPOT	IFNg release	Positive
195393	GLLFSIMKNTANTRR	NS4b	2463	2477	HLA-DRB1*04:01	Dengue virus 2	ELISPOT	IFNg release	Positive
195394	GLLFSIMKNTTNARR	NS4b	2477	2491	HLA-DRB1*04:01	Dengue virus 2	ELISPOT	IFNg release	Positive
195395	GLLFSIMKNTTNSRR	NS4b	2477	2491	HLA-DRB1*04:01	Dengue virus 2	ELISPOT	IFNg release	Positive
195396	GLLFSIMKNTTSARR	NS4b	2477	2491	HLA-DRB1*04:01	Dengue virus 2	ELISPOT	IFNg release	Positive
195397	GLLFSIMKNTTSTRR	NS4b	2477	2491	HLA-DRB1*04:01	Dengue virus 2	ELISPOT	IFNg release	Positive
195398	GLLFSVMKNTTNTRR	NS4b	2477	2491	HLA-DRB1*04:01	Dengue virus 2	ELISPOT	IFNg release	Positive
195621	LCVTQVLMMRTTWAL	NS4b	2405	2419	HLA-DRB1*07:01	Dengue virus 2	ELISPOT	IFNg release	Positive
195629	LFSIMKNTTNTRRGT	NS4b	2479	2493	HLA-DRB1*03:01	Dengue virus 2	ELISPOT	IFNg release	Positive
195658	LLFSIMKNTTNTRRG	NS4b	2478	2492	HLA-DRB1*04:01	Dengue virus 2	ELISPOT	IFNg release	Positive
195659	LLFSIMRNTTTARRG	NS4b	2478	2492	HLA-DRB1*04:01	Dengue virus 2	ELISPOT	IFNg release	Positive
195718	LTAALFLLVAHYAII	NS4b	2350	2364	HLA-DRB1*07:01	Dengue virus 2	ELISPOT	IFNg release	Positive
195827	PESNILDIDLRPASA	NS4b	2266	2280	HLA-DRB1*03:01	Dengue virus 2	ELISPOT	IFNg release	Positive
195882	RFWNTTIAVSMANIF	NS4b	2454	2468	HLA-DRB1*07:01	Dengue virus 2	ELISPOT	IFNg release	Positive
195974	SNILDIDLRPASAWT	NS4b	2155	2169	HLA-DRB1*03:01	Dengue virus 2	ELISPOT	IFNg release	Positive
195980	SQVNPITLTAALLLL	NS4b	2343	2357	HLA-DQA1*01:02/DQB1*06:02	Dengue virus 2	ELISPOT	IFNg release	Positive
539327	GLLFSIMKNTTNTRR	NS4b	2477	2491	HLA-DRB1*14:02	Dengue virus 2	ELISPOT	IFNg release	Positive
539724	NIFRGSYLAGAGLLF	NS4b	2452	2466	HLA-DRB1*09:01	Dengue virus 2	ELISPOT	IFNg release	Positive
539755	PASAWTLYAVATTFV	NS4b	2277	2291	HLA-DRB1*09:01	Dengue virus 2	ELISPOT	IFNg release	Positive
739437	LDIDLRPASAWTLYA	NS4b	2257	2271	HLA-DRB1*01:02	Dengue virus 2	ELISPOT	IFNg release	Positive
866802	FWNTTIAVSMANIFR	NS4b	2455	2469	HLA-DQA1*01:02/DQB1*06:02	Dengue virus 2	ELISPOT	IFNg release	Positive
867335	ITLTAALLLLIAHYA	NS4b	2348	2362	HLA-DPB1*04:02	Dengue virus 2	ELISPOT	IFNg release	Positive
867709	LILCVTQVLMMRTTW	NS4b	2417	2431	HLA-DPB1*04:02	Dengue virus 2	ELISPOT	IFNg release	Positive
867860	LLVLCVTQVLMMRTT	NS4b	2416	2430	HLA-DPB1*04:02	Dengue virus 2	ELISPOT	IFNg release	Positive
869089	TAIANQATVLMGLGK	NS4b	2309	2323	HLA-DQA1*01:02/DQB1*06:02	Dengue virus 2	ELISPOT	IFNg release	Positive
869090	TAIANQATVLMGLGR	NS4b	2309	2323	HLA-DQA1*01:02/DQB1*06:02	Dengue virus 2	ELISPOT	IFNg release	Positive
1066545	ACLGKSYAQMWSLMY	NS5	3243	3257	HLA-DRB1*04:01	Dengue virus 2	ELISPOT	IFNg release	Positive-Low
150597	SRAIWYMWLGARFLE	NS5	2962	2976	HLA-DRB1*01:01	Dengue virus 2	ELISPOT	IFNg release	Positive
167817	SRLNALGKSEFQI	NS5	15	27	HLA class II	Dengue virus 2	ELISPOT	IFNg release	Positive
180579	KSYAQMWSLMYFHRR	NS5	3247	3261	HLA-DRB5*01:01	Dengue virus 2	ELISPOT	IFNg release	Positive
180582	KTWAYHGSYETKQTG	NS5	2792	2806	HLA class II	Dengue virus 2	ICS	IFNg release	Positive
190862	MVTQMAMTDTTPFGQQR	NS5	2828	2844	HLA class II	Dengue virus 2	ICS	IFNg release	Positive
195111	ACLGKSYAQMWTLMY	NS5	3243	3257	HLA-DRB1*04:01	Dengue virus 2	ELISPOT	IFNg release	Positive
195200	CETCVYNMMGKREKK	NS5	2924	2938	HLA-DRB1*11:01	Dengue virus 2	ELISPOT	IFNg release	Positive
195213	DELIGRARISQGAGW	NS5	3223	3237	HLA-DRB1*08:02	Dengue virus 2	ELISPOT	IFNg release	Positive
195233	EDHWFSRENSLSGVE	NS5	2985	2999	HLA-DRB1*04:01	Dengue virus 2	ELISPOT	IFNg release	Positive
195241	EGEGVFKSIQHLTVT	NS5	3101	3115	HLA-DRB1*07:01	Dengue virus 2	ELISPOT	IFNg release	Positive
195250	ELIMKDGRKLVVPCR	NS5	3206	3220	HLA-DRB1*03:01	Dengue virus 2	ELISPOT	IFNg release	Positive
195251	ELIMKDGRVLVVPCR	NS5	3206	3220	HLA-DRB1*03:01	Dengue virus 2	ELISPOT	IFNg release	Positive
195380	GKSYAQMWSLMYFHR	NS5	3232	3246	HLA-DRB1*04:01	Dengue virus 2	ELISPOT	IFNg release	Positive
195384	GLFKSIQHLTSQEEI	NS5	3118	3132	HLA-DRB1*11:01	Dengue virus 2	ELISPOT	IFNg release	Positive
195409	GNIVSSVNMISRMLI	NS5	2717	2731	HLA-DRB1*07:01	Dengue virus 2	ELISPOT	IFNg release	Positive
195428	GTVMDIISRRDQRGS	NS5	3078	3092	HLA-DRB1*03:01	Dengue virus 2	ELISPOT	IFNg release	Positive
195445	HFHELIMKDGRILVV	NS5	3203	3217	HLA-DRB1*03:01	Dengue virus 2	ELISPOT	IFNg release	Positive
195446	HFHELVMKDGRVLVV	NS5	3203	3217	HLA-DRB1*13:01	Dengue virus 2	ELISPOT	IFNg release	Positive
195460	HRLGYILRDVSRKEG	NS5	3004	3018	HLA-DRB1*08:02	Dengue virus 2	ELISPOT	IFNg release	Positive
195513	IMKDGRKLVVPCRNQ	NS5	3207	3221	HLA-DRB1*03:01	Dengue virus 2	ELISPOT	IFNg release	Positive
195640	LGYILRDVSKKEGGA	NS5	2992	3006	HLA-DRB1*03:01	Dengue virus 2	ELISPOT	IFNg release	Positive
195642	LHKLGYILRDVSKKE	NS5	3003	3017	HLA-DRB1*08:02	Dengue virus 2	ELISPOT	IFNg release	Positive
195750	MEGEGIFKSIQHLTA	NS5	3114	3128	HLA-DRB1*11:01	Dengue virus 2	ELISPOT	IFNg release	Positive
195759	MLINRFTMKHKKATY	NS5	2729	2743	HLA-DRB1*11:01	Dengue virus 2	ELISPOT	IFNg release	Positive
195800	NMISRMLINRFTMKH	NS5	2724	2738	HLA-DRB1*08:02	Dengue virus 2	ELISPOT	IFNg release	Positive
195801	NMISRMLINRFTMRH	NS5	6	20	HLA-DRB1*08:02	Dengue virus 2	ELISPOT	IFNg release	Positive
195948	SGNIVSSVNMISRML	NS5	2716	2730	HLA-DRB1*04:01	Dengue virus 2	ELISPOT	IFNg release	Positive
196040	TGNIVSSVNMISRML	NS5	2716	2730	HLA-DRB1*04:01	Dengue virus 2	ELISPOT	IFNg release	Positive
196161	VNMISRMLINRFTMR	NS5	2723	2737	HLA-DRB1*10:01	Dengue virus 2	ELISPOT	IFNg release	Positive
196187	VSSVNMISRMLINRF	NS5	2720	2734	HLA-DRB1*15:01	Dengue virus 2	ELISPOT	IFNg release	Positive

196210	WAKNIQTAINQVRSL	NS5	3336	3350	HLA-DRB1*03:01	Dengue virus 2	ELISPOT	IFNg release	Positive
196223	WSLMYFHRRDLRLAA	NS5	3253	3267	HLA-DRB1*03:01	Dengue virus 2	ELISPOT	IFNg release	Positive
196225	WTLMYFHRRDLRLAA	NS5	3253	3267	HLA-DRB1*03:01	Dengue virus 2	ELISPOT	IFNg release	Positive
196243	YQNKVVRVQRPTPRG	NS5	3064	3078	HLA-DRB1*08:02	Dengue virus 2	ELISPOT	IFNg release	Positive
32939	KPWDIIPMV	NS5	2821	2829	HLA class II	Dengue virus 2	ELISA	IL-2 release	Positive
32941	KPWDVLPMV	NS5	2821	2829	HLA class II	Dengue virus 2	ELISA	IL-2 release	Positive
32942	KPWDVLPTV	NS5	2821	2829	HLA class II	Dengue virus 2	ELISA	IL-2 release	Positive
32943	KPWDVVPMV	NS5	2821	2829	HLA class II	Dengue virus 2	ELISA	IL-2 release	Positive
539144	CVVKPLDDRFASALTAL	NS5	3156	3172	HLA class II	Dengue virus 2	ICS	IFNg release	Positive
539150	DDRFASALTALNDMG	NS5	3162	3176	HLA-DRB1*04:03	Dengue virus 2	ELISPOT	IFNg release	Positive
539161	DHWFSRGNSLSGVEG	NS5	2986	3000	HLA-DRB1*09:01	Dengue virus 2	ELISPOT	IFNg release	Positive
539195	EAIFKLTYQNKVVRV	NS5	3057	3071	HLA-DRB1*09:01	Dengue virus 2	ELISPOT	IFNg release	Positive
539348	GSAKLRWFVERNMVT	NS5	2549	2563	HLA-DRB1*09:01	Dengue virus 2	ELISPOT	IFNg release	Positive
539436	ISGDDCVVKPLDDRFA	NS5	3151	3166	HLA class II	Dengue virus 2	ICS	IFNg release	Positive
539438	ISRMLINRFTMRHKK	NS5	8	22	HLA-DRB1*14:02	Dengue virus 2	ELISPOT	IFNg release	Positive
539560	LGKNEFQIYKKSGIQ	NS5	2511	2525	HLA-DRB1*15:02	Dengue virus 2	ELISPOT	IFNg release	Positive
539599	LMYFHRRDLRLAANA	NS5	3255	3269	HLA-DRB1*13:01	Dengue virus 2	ELISPOT	IFNg release	Positive
539603	LNTFTNMEAQLIRQM	NS5	3100	3114	HLA-DRB1*10:01	Dengue virus 2	ELISPOT	IFNg release	Positive
539668	MISRMLINRFTMKHK	NS5	2725	2739	HLA-DRB1*14:04	Dengue virus 2	ELISPOT	IFNg release	Positive
539669	MISRMLINRFTMRHK	NS5	7	21	HLA-DRB1*14:06	Dengue virus 2	ELISPOT	IFNg release	Positive
539871	RRDLRLAANAICSAV	NS5	3260	3274	HLA-DRB3*02:02	Dengue virus 2	ELISPOT	IFNg release	Positive
539932	SRMLINRFTMKHKKA	NS5	2727	2741	HLA-DRB1*08:03	Dengue virus 2	ELISPOT	IFNg release	Positive
540064	VNGVVRLLTKPWDVI	NS5	2812	2826	HLA-DRB1*12:02	Dengue virus 2	ELISPOT	IFNg release	Positive
540065	VNGVVRLLTKPWDVV	NS5	2812	2826	HLA-DRB1*12:02	Dengue virus 2	ELISPOT	IFNg release	Positive
540066	VNMISRMLINRFTMK	NS5	2723	2737	HLA-DRB1*12:02	Dengue virus 2	ELISPOT	IFNg release	Positive
738754	EEFTRKVRSNAALGA	NS5	2874	2888	HLA-DRB1*11:04	Dengue virus 2	ELISPOT	IFNg release	Positive
739166	IFKLTYQNKVVRVQR	NS5	3045	3059	HLA-DRB1*14:06	Dengue virus 2	ELISPOT	IFNg release	Positive
739791	NLVRLQSGVDVFFTP	NS5	2599	2613	HLA-DRB1*01:02	Dengue virus 2	ELISPOT	IFNg release	Positive
740675	VRSNAALGAIFTDEN	NS5	2880	2894	HLA-DRB1*11:04	Dengue virus 2	ELISPOT	IFNg release	Positive
866015	AIWYMWLGARFLEFE	NS5	2964	2978	HLA-DPB1*01:01	Dengue virus 2	ELISPOT	IFNg release	Positive
866172	ASGNIVSSVNMISRM	NS5	2715	2729	HLA-DRB3*02:02	Dengue virus 2	ELISPOT	IFNg release	Positive
866566	EHKKLAEAIFKLTYQ	NS5	3051	3065	HLA-DPB1*01:01	Dengue virus 2	ELISPOT	IFNg release	Positive
866648	ETCVYNMMGKREKKL	NS5	2939	2953	HLA-DRB5*01:01	Dengue virus 2	ELISPOT	IFNg release	Positive
867419	KKLAEAIFKLTYQNK	NS5	3053	3067	HLA-DPB1*02:01	Dengue virus 2	ELISPOT	IFNg release	Positive
867433	KLAEAIFKLTYQNKV	NS5	3054	3068	HLA-DPB1*02:01	Dengue virus 2	ELISPOT	IFNg release	Positive
867514	KTPVESWEEIPYLGK	NS5	3318	3332	HLA-DQA1*01:01/DQB1*05:01	Dengue virus 2	ELISPOT	IFNg release	Positive
867600	LEFEALGFLNEDHWF	NS5	2975	2989	HLA-DPB1*04:01	Dengue virus 2	ELISPOT	IFNg release	Positive
868637	RDLRLAANAICSAVP	NS5	3261	3275	HLA-DQA1*05:01/DQB1*02:01	Dengue virus 2	ELISPOT	IFNg release	Positive
868640	RFLEFEALGFLNEDH	NS5	2973	2987	HLA-DPB1*01:01	Dengue virus 2	ELISPOT	IFNg release	Positive
868668	RKVRSNAALGAIFTD	NS5	2892	2906	HLA-DRB3*02:02	Dengue virus 2	ELISPOT	IFNg release	Positive
869082	SYAQMWSLMYFHRRD	NS5	3248	3262	HLA-DPB1*04:01	Dengue virus 2	ELISPOT	IFNg release	Positive
869083	SYAQMWTLMYFHRRD	NS5	3248	3262	HLA-DPB1*04:01	Dengue virus 2	ELISPOT	IFNg release	Positive
869148	TFVESWEDIPYLGKR	NS5	3319	3333	HLA-DQA1*01:01/DQB1*05:01	Dengue virus 2	ELISPOT	IFNg release	Positive
869292	TPVESWEEVPYLGKR	NS5	3319	3333	HLA-DQA1*01:01/DQB1*05:01	Dengue virus 2	ELISPOT	IFNg release	Positive
869307	TRKVRSNAALGAIFT	NS5	2891	2905	HLA-DRB3*02:02	Dengue virus 2	ELISPOT	IFNg release	Positive
869426	VESWEEIPYLGKRED	NS5	3321	3335	HLA-DQA1*01:01/DQB1*05:01	Dengue virus 2	ELISPOT	IFNg release	Positive

CD4 DENV-3 Epitopes

Epitope_ID	AminoAcid_Seq	Protein_Name	Start	End	Allele	Serotype	Method/Technique	Assay Group	Qualitative Measure
1068391	TGKPSINMLKRVRNR	C	8	22	HLA-DRB1*11:01	Dengue virus 3	ELISPOT	IFNg release	Positive-Low
195205	CLMMMLPATLAFHLT	C	104	118	HLA-DRB1*15:01	Dengue virus 3	ELISPOT	IFNg release	Positive
195289	FCLMMMLPATLAFHL	C	103	117	HLA-DRB1*01:01	Dengue virus 3	ELISPOT	IFNg release	Positive
195303	FKKEISNMLSIINKR	C	37	51	HLA-DRB1*04:01	Dengue virus 3	ELISPOT	IFNg release	Positive
195306	FLRFLAIPPTAGVLA	C	6	20	HLA-DRB1*01:01	Dengue virus 3	ELISPOT	IFNg release	Positive
195469	IAFLRFLAIPPTAGV	C	4	18	HLA-DRB1*04:01	Dengue virus 3	ELISPOT	IFNg release	Positive
195497	IKVLKGFKKEISNML	C	31	45	HLA-DRB1*04:01	Dengue virus 3	ELISPOT	IFNg release	Positive
195517	INMLKRVRNRVSTGP	C	13	27	HLA-DRB1*08:02	Dengue virus 3	ELISPOT	IFNg release	Positive
195518	INMLKRVRNRVSTGS	C	13	27	HLA-DRB1*08:02	Dengue virus 3	ELISPOT	IFNg release	Positive
195519	INMLKRVRNRVSTGT	C	13	27	HLA-DRB1*08:02	Dengue virus 3	ELISPOT	IFNg release	Positive
195536	ISNMLSIINKRKKTS	C	41	55	HLA-DRB1*11:01	Dengue virus 3	ELISPOT	IFNg release	Positive
195559	KEISNMLSIINKRKK	C	39	53	HLA-DRB1*04:01	Dengue virus 3	ELISPOT	IFNg release	Positive
195597	KTSLCLMMILPAALA	C	53	67	HLA-DRB1*01:01	Dengue virus 3	ELISPOT	IFNg release	Positive
195598	KTSLCLVMILPAALA	C	100	114	HLA-DRB1*01:01	Dengue virus 3	ELISPOT	IFNg release	Positive
195611	LARWGTFKKSGAIKV	C	19	33	HLA-DRB1*07:01	Dengue virus 3	ELISPOT	IFNg release	Positive
195618	LCLMMIMPAALAFHL	C	103	117	HLA-DRB1*01:01	Dengue virus 3	ELISPOT	IFNg release	Positive
195649	LKGFKKEISNMLSII	C	34	48	HLA-DRB1*07:01	Dengue virus 3	ELISPOT	IFNg release	Positive
195803	NMLKRVRNRVSTGPQ	C	14	28	HLA-DRB1*11:01	Dengue virus 3	ELISPOT	IFNg release	Positive
195961	SINMLKRVRNRVSTG	C	12	26	HLA-DRB1*08:02	Dengue virus 3	ELISPOT	IFNg release	Positive
195966	SLCLMMIMPAALAFH	C	102	116	HLA-DRB1*08:02	Dengue virus 3	ELISPOT	IFNg release	Positive
195967	SLCLMMMLPATLAFH	C	102	116	HLA-DRB1*08:02	Dengue virus 3	ELISPOT	IFNg release	Positive
36858	LKGFKKEISNML	C	81	92	HLA-DPw4	Dengue virus 3	ICS	IFNg release	Positive
539277	GAIKVLKGFKKEISN	C	29	43	HLA-DRB1*12:02	Dengue virus 3	ELISPOT	IFNg release	Positive
539447	IVNRRKKTSLCLMMM	C	94	108	HLA-DRB1*13:01	Dengue virus 3	ELISPOT	IFNg release	Positive
539502	KPSINMLKRVRNRVS	C	10	24	HLA-DRB1*08:03	Dengue virus 3	ELISPOT	IFNg release	Positive
539512	KSGAIKVLKGFKKEI	C	27	41	HLA-DRB1*12:02	Dengue virus 3	ELISPOT	IFNg release	Positive
539787	PSINMLKRVRNRVST	C	11	25	HLA-DRB1*14:04	Dengue virus 3	ELISPOT	IFNg release	Positive
539897	SGAIKVLKGFKKEIS	C	28	42	HLA-DRB1*14:04	Dengue virus 3	ELISPOT	IFNg release	Positive
539906	SIINRRKRTSLCLMM	C	93	107	HLA-DRB1*13:01	Dengue virus 3	ELISPOT	IFNg release	Positive
539926	SNMLSIINKRKKTSL	C	42	56	HLA-DRB1*12:02	Dengue virus 3	ELISPOT	IFNg release	Positive
540118	WGSFKKSGAVKVLRG	C	69	83	HLA-DRB1*09:01	Dengue virus 3	ELISPOT	IFNg release	Positive
540119	WGTFKKLGAIKVLKG	C	69	83	HLA-DRB1*09:01	Dengue virus 3	ELISPOT	IFNg release	Positive
540120	WGTFKKLGAIKVLRG	C	69	83	HLA-DRB1*09:01	Dengue virus 3	ELISPOT	IFNg release	Positive
540121	WGTFKKSGAIKVLKG	C	22	36	HLA-DRB1*09:01	Dengue virus 3	ELISPOT	IFNg release	Positive
540122	WGTFKKSGAIKVLKS	C	69	83	HLA-DRB1*09:01	Dengue virus 3	ELISPOT	IFNg release	Positive
540123	WGTFKKSGAIKVLRG	C	69	83	HLA-DRB1*09:01	Dengue virus 3	ELISPOT	IFNg release	Positive
540124	WGTFKKSGAIRVLRG	C	69	83	HLA-DRB1*09:01	Dengue virus 3	ELISPOT	IFNg release	Positive
591709	LRGFKKEISNMLSII	C	81	95	HLA-DRB1*13:02	Dengue virus 3	ELISPOT	IFNg release	Positive
591723	NMLSIINRRKKTSLC	C	90	104	HLA-DRB1*11:01	Dengue virus 3	ELISPOT	IFNg release	Positive
591746	RGFKKEISNMLSIIN	C	82	96	HLA-DRB1*04:01	Dengue virus 3	ELISPOT	IFNg release	Positive
738443	AFLRFLAIPPTAGVL	C	52	66	HLA-DRB1*04:07	Dengue virus 3	ELISPOT	IFNg release	Positive
740207	RWGTFKKSGAIKVLK	C	68	82	HLA-DRB1*14:02	Dengue virus 3	ELISPOT	IFNg release	Positive
867744	LKSFKKEISNMLSII	C	81	95	HLA-DRB3*02:02	Dengue virus 3	ELISPOT	IFNg release	Positive
867909	LRGFKREISNMLSII	C	64	78	HLA-DRB3*02:02	Dengue virus 3	ELISPOT	IFNg release	Positive
868645	RGLLNGQGPMKLVMA	C	15	29	HLA-DRB3*02:02	Dengue virus 3	ELISPOT	IFNg release	Positive
868733	RWGTFKKSGAIKVLR	C	48	62	HLA-DRB3*02:02	Dengue virus 3	ELISPOT	IFNg release	Positive
195602	KVVIFILLMLVTPSM	PreM	265	279	HLA-DRB1*01:01	Dengue virus 3	ELISPOT	IFNg release	Positive
195726	LTQKVVIFILLMLVT	PreM	262	276	HLA-DRB1*11:01	Dengue virus 3	ELISPOT	IFNg release	Positive
195830	PGFTILALFLAHYIG	PreM	245	259	HLA-DRB1*15:06	Dengue virus 3	ELISPOT	IFNg release	Positive
195968	SLIQKVVIFILLMLV	PreM	261	275	HLA-DRB1*15:06	Dengue virus 3	ELISPOT	IFNg release	Positive
196067	TQKVVIFVLLMLVTP	PreM	263	277	HLA-DRB1*15:06	Dengue virus 3	ELISPOT	IFNg release	Positive
196203	VVIFILLMLVTPSMT	PreM	266	280	HLA-DRB1*07:01	Dengue virus 3	ELISPOT	IFNg release	Positive
539529	KVVIFILLILVTPSM	PreM	265	279	HLA-DRB1*15:06	Dengue virus 3	ELISPOT	IFNg release	Positive
539808	QKVVIFTLLMLVTPS	PreM	264	278	HLA-DRB1*15:06	Dengue virus 3	ELISPOT	IFNg release	Positive
540004	TSLTQKVVIFILLML	PreM	260	274	HLA-DPB1*04:02	Dengue virus 3	ELISPOT	IFNg release	Positive
867082	HPGFTILALFLAHYI	PreM	244	258	HLA-DPB1*02:01	Dengue virus 3	ELISPOT	IFNg release	Positive
869302	TQKVVIFILLMLVTP	PreM	263	277	HLA-DPB1*04:02	Dengue virus 3	ELISPOT	IFNg release	Positive
119134	EGKVVQYENLKYTVI	E	126	140	HLA-DR2	Dengue virus 3	ELISPOT	IFNg release	Positive
119289	NGRLITANPVVTKKE	E	626	640	HLA-DRB1*08:02	Dengue virus 3	ELISPOT	IFNg release	Positive
119327	RKELLVTFKNAHAKK	E	231	245	HLA-DR2	Dengue virus 3	ELISPOT	IFNg release	Positive
13376	EMILLTMKNKAWMVH	E	473	487	HLA-DRB1*13:01	Dengue virus 3	ELISPOT	IFNg release	Positive
195215	DFNEMILLTMKNKAW	E	470	484	HLA-DRB1*14:04	Dengue virus 3	ELISPOT	IFNg release	Positive
195252	ELLVTFKNAHAKKQE	E	513	527	HLA-DRB1*04:01	Dengue virus 3	ELISPOT	IFNg release	Positive
195341	FVLKKEVSETQHGTI	E	584	598	HLA-DRB1*04:01	Dengue virus 3	ELISPOT	IFNg release	Positive
195365	GHLKCRLKMDKLELK	E	559	573	HLA-DRB1*03:01	Dengue virus 3	ELISPOT	IFNg release	Positive
195416	GRLITANPVVTKKEE	E	627	641	HLA-DRB1*08:02	Dengue virus 3	ELISPOT	IFNg release	Positive
195813	NRKELLVTFKNAHAK	E	510	524	HLA-DRB1*04:01	Dengue virus 3	ELISPOT	IFNg release	Positive
196033	TFVLKKEVSETQHGT	E	583	597	HLA-DRB1*04:01	Dengue virus 3	ELISPOT	IFNg release	Positive
196170	VQHENLKYTVIITVH	E	130	144	HLA-DRB1*07:01	Dengue virus 3	ELISPOT	IFNg release	Positive
196172	VQYENLKYTVIITVH	E	410	424	HLA-DRB1*07:01	Dengue virus 3	ELISPOT	IFNg release	Positive
539400	IEGKVVQYENLKYTV	E	405	419	HLA-DRB1*15:02	Dengue virus 3	ELISPOT	IFNg release	Positive
539460	KELLVTFKNAHAKKQ	E	512	526	HLA-DRB1*08:03	Dengue virus 3	ELISPOT	IFNg release	Positive
539476	KKELLVTFKNAHAKK	E	511	525	HLA-DRB1*14:04	Dengue virus 3	ELISPOT	IFNg release	Positive
539559	LGKMVHQIFGSAYTA	E	710	724	HLA-DRB1*04:03	Dengue virus 3	ELISPOT	IFNg release	Positive
539710	NEMILLTMKNKAWMV	E	472	486	HLA-DRB1*04:03	Dengue virus 3	ELISPOT	IFNg release	Positive
539856	RKELLTTFKNAHAKK	E	511	525	HLA-DRB1*04:03	Dengue virus 3	ELISPOT	IFNg release	Positive
591605	AFVLKKEVSETQHGT	E	303	317	HLA-DRB1*04:01	Dengue virus 3	ELISPOT	IFNg release	Positive
591719	NEMILLTMKNRAWMV	E	474	488	HLA-DRB1*11:01	Dengue virus 3	ELISPOT	IFNg release	Positive
739011	GGVLNSLGKMVHQIF	E	704	718	HLA-DRB1*11:04	Dengue virus 3	ELISPOT	IFNg release	Positive
739041	GMSYAMCTNTFVLKK	E	574	588	HLA-DRB1*04:07	Dengue virus 3	ELISPOT	IFNg release	Positive
739344	KMVHQIFGSAYTALF	E	31	45	HLA-DRB3*02:02	Dengue virus 3	ELISPOT	IFNg release	Positive
740409	SVGGVLNSLGKMVHQ	E	702	716	HLA-DRB1*11:04	Dengue virus 3	ELISPOT	IFNg release	Positive
741746	MRCVGVGNRDFVEGLSGATW	E	281	300	HLA class II	Dengue virus 3	ELISPOT	IFNg release	Positive
866574	EIQNSGGTSIFAGHL	E	547	561	HLA-DQA1*01:02/DQB1*06:02	Dengue virus 3	ELISPOT	IFNg release	Positive
867374	KAWIVHRQWFFDLPL	E	482	496	HLA-DQA1*01:01/DQB1*05:01	Dengue virus 3	ELISPOT	IFNg release	Positive
867377	KCRLKMDKLELKGMS	E	562	576	HLA-DRB3*02:02	Dengue virus 3	ELISPOT	IFNg release	Positive
868175	MVHRQWFFDLPLPWT	E	485	499	HLA-DQA1*01:01/DQB1*05:01	Dengue virus 3	ELISPOT	IFNg release	Positive
869693	WMVHRQWLFDLPLPW	E	87	101	HLA-DQA1*01:01/DQB1*05:01	Dengue virus 3	ELISPOT	IFNg release	Positive

190705	AKIVTAETQNSSFII	NS1	894	908	HLA-DRB1*04:01	Dengue virus 3	ELISPOT	IFNg release	Positive
190823	KLTVVVGDIIGVLEQ	NS1	85	99	HLA-DR2	Dengue virus 3	ELISPOT	IFNg release	Positive
190825	KQIANELNYILWENN	NS1	69	83	HLA-DR2	Dengue virus 3	ELISPOT	IFNg release	Positive
190996	TEQYKFQADSPKRLA	NS1	802	816	HLA-DRB5*01:01	Dengue virus 3	ELISPOT	IFNg release	Positive
195164	ANELNYILWENNIKL	NS1	845	859	HLA-DRB1*03:01	Dengue virus 3	ELISPOT	IFNg release	Positive
195419	GSWKLEKASLIEVKT	NS1	981	995	HLA-DRB1*01:01	Dengue virus 3	ELISPOT	IFNg release	Positive
195577	KLTVVVGDTIGVLEQ	NS1	858	872	HLA-DRB1*03:01	Dengue virus 3	ELISPOT	IFNg release	Positive
195692	LNYILWENNIKLTVV	NS1	848	862	HLA-DRB1*03:01	Dengue virus 3	ELISPOT	IFNg release	Positive
195782	NELNYILWENNIKLT	NS1	846	860	HLA-DRB1*03:01	Dengue virus 3	ELISPOT	IFNg release	Positive
196235	YKFQADSPKRLATAI	NS1	805	819	HLA-DRB1*03:01	Dengue virus 3	ELISPOT	IFNg release	Positive
539070	AKIVTAETRNSSFII	NS1	121	135	HLA-DRB1*13:01	Dengue virus 3	ELISPOT	IFNg release	Positive
539457	KAKIVIAETQNSSFI	NS1	893	907	HLA-DRB1*13:01	Dengue virus 3	ELISPOT	IFNg release	Positive
539717	NGSWKLEKASLIEVK	NS1	980	994	HLA-DRB1*09:01	Dengue virus 3	ELISPOT	IFNg release	Positive
539825	QYKFQADSPKRLATA	NS1	804	818	HLA-DRB3*02:02	Dengue virus 3	ELISPOT	IFNg release	Positive
866698	FGVFTTNIWLKLRDV	NS1	933	947	HLA-DPB1*04:01	Dengue virus 3	ELISPOT	IFNg release	Positive
866700	FGVFTTNIWLKLREV	NS1	933	947	HLA-DPB1*01:01	Dengue virus 3	ELISPOT	IFNg release	Positive
867042	HADMGYWIESQKNGS	NS1	968	982	HLA-DQA1*01:01/DQB1*05:01	Dengue virus 3	ELISPOT	IFNg release	Positive
167810	RENLLLGVGLAMATTLQLPE	NS2a	1224	1243	HLA-DP	Dengue virus 3	ELISPOT	IFNg release	Positive
195123	AGVFFTFVLLLSGQI	NS2a	1159	1173	HLA-DRB1*07:01	Dengue virus 3	ELISPOT	IFNg release	Positive
195125	AGVIFTFVLLLSGQI	NS2a	1159	1173	HLA-DRB1*07:01	Dengue virus 3	ELISPOT	IFNg release	Positive
195128	AGVLFMFVLLLSGQI	NS2a	1159	1173	HLA-DRB1*07:01	Dengue virus 3	ELISPOT	IFNg release	Positive
195133	AHTFIMIGSNASDRM	NS2a	1179	1193	HLA-DRB1*04:01	Dengue virus 3	ELISPOT	IFNg release	Positive
195134	AHTLIMIGSNASDRM	NS2a	1179	1193	HLA-DRB1*04:01	Dengue virus 3	ELISPOT	IFNg release	Positive
195166	ANTFIMIGSNASDRM	NS2a	1179	1193	HLA-DRB1*04:01	Dengue virus 3	ELISPOT	IFNg release	Positive
195197	AWRTATLILAGVSLL	NS2a	1289	1303	HLA-DRB1*01:01	Dengue virus 3	ELISPOT	IFNg release	Positive
195222	DMAHTLIMIGSNASD	NS2a	1177	1191	HLA-DRB1*04:01	Dengue virus 3	ELISPOT	IFNg release	Positive
195335	FTFVLLLSGQITWRD	NS2a	1163	1177	HLA-DRB1*15:02	Dengue virus 3	ELISPOT	IFNg release	Positive
195337	FTLTVAWRTATLILA	NS2a	1284	1298	HLA-DRB1*07:01	Dengue virus 3	ELISPOT	IFNg release	Positive
195342	FVLLLSGQITWRDMA	NS2a	1165	1179	HLA-DRB1*01:01	Dengue virus 3	ELISPOT	IFNg release	Positive
195429	GVFFTFVLLLSGQIT	NS2a	1160	1174	HLA-DRB1*04:01	Dengue virus 3	ELISPOT	IFNg release	Positive
195433	GVLFTFVLLLSGQIT	NS2a	1160	1174	HLA-DRB1*15:06	Dengue virus 3	ELISPOT	IFNg release	Positive
195456	HMIAGILFMFVLLLS	NS2a	1156	1170	HLA-DRB1*11:01	Dengue virus 3	ELISPOT	IFNg release	Positive
195457	HMIAGVFFTFVLLLS	NS2a	1156	1170	HLA-DRB1*11:01	Dengue virus 3	ELISPOT	IFNg release	Positive
195462	HTLIMIGSNASDRMG	NS2a	1180	1194	HLA-DRB1*04:01	Dengue virus 3	ELISPOT	IFNg release	Positive
195481	IFTLTVAWRTATLIL	NS2a	1283	1297	HLA-DRB1*07:01	Dengue virus 3	ELISPOT	IFNg release	Positive
195609	LALGFFLRKLTSREN	NS2a	1212	1226	HLA-DRB1*11:01	Dengue virus 3	ELISPOT	IFNg release	Positive
195630	LFTFVLLLSGQITWR	NS2a	1162	1176	HLA-DRB1*07:01	Dengue virus 3	ELISPOT	IFNg release	Positive
195632	LGFFLRKLTSRENLL	NS2a	1214	1228	HLA-DRB1*11:01	Dengue virus 3	ELISPOT	IFNg release	Positive
195644	LIMIGSNASDRMGMG	NS2a	1182	1196	HLA-DRB1*04:01	Dengue virus 3	ELISPOT	IFNg release	Positive
195645	LISLTCSNTIFTLTV	NS2a	1274	1288	HLA-DRB1*07:01	Dengue virus 3	ELISPOT	IFNg release	Positive
195729	LTVAWRTATLILAGV	NS2a	1286	1300	HLA-DPB1*04:02	Dengue virus 3	ELISPOT	IFNg release	Positive
195734	LVSLTCSNTILTLTV	NS2a	1274	1288	HLA-DRB1*07:01	Dengue virus 3	ELISPOT	IFNg release	Positive
195740	LWTALVSLTCSNTIF	NS2a	1270	1284	HLA-DRB1*07:01	Dengue virus 3	ELISPOT	IFNg release	Positive
195743	MAHTLIMIGSNASDR	NS2a	1178	1192	HLA-DRB1*04:01	Dengue virus 3	ELISPOT	IFNg release	Positive
195757	MGVTYLALIATFKIQ	NS2a	1195	1209	HLA-DRB1*07:01	Dengue virus 3	ELISPOT	IFNg release	Positive
195819	NTIFTLTVAWRTATL	NS2a	1281	1295	HLA-DRB1*07:01	Dengue virus 3	ELISPOT	IFNg release	Positive
195836	PLFIFSLKDTLKRRS	NS2a	1330	1344	HLA-DRB1*11:01	Dengue virus 3	ELISPOT	IFNg release	Positive
195912	RTATLILAGVSLLPV	NS2a	1291	1305	HLA-DRB1*03:01	Dengue virus 3	ELISPOT	IFNg release	Positive
196123	VGVLFTFVLLLSGQI	NS2a	1159	1173	HLA-DRB1*07:01	Dengue virus 3	ELISPOT	IFNg release	Positive
539075	ALGFFLRKLTSRENL	NS2a	1213	1227	HLA-DRB1*12:02	Dengue virus 3	ELISPOT	IFNg release	Positive
539370	GVTYLALIATFKIQP	NS2a	1196	1210	HLA-DRB1*10:01	Dengue virus 3	ELISPOT	IFNg release	Positive
539429	IQPFLALGFFLRKLT	NS2a	1208	1222	HLA-DRB1*15:06	Dengue virus 3	ELISPOT	IFNg release	Positive
539568	LIATFKIQPFLALGF	NS2a	1202	1216	HLA-DRB1*15:06	Dengue virus 3	ELISPOT	IFNg release	Positive
539802	QFETYQLWTALVSLT	NS2a	1264	1278	HLA-DRB1*15:02	Dengue virus 3	ELISPOT	IFNg release	Positive
540048	VLFTFVLLLSGQITW	NS2a	1161	1175	HLA-DRB1*09:01	Dengue virus 3	ELISPOT	IFNg release	Positive
540093	VTYLALIATFKIQPF	NS2a	1197	1211	HLA-DRB1*04:03	Dengue virus 3	ELISPOT	IFNg release	Positive
865959	AGALFTFVLLLSGQI	NS2a	1159	1173	HLA-DPB1*04:02	Dengue virus 3	ELISPOT	IFNg release	Positive
867116	IAGVFFTFVLLLSGQ	NS2a	1158	1172	HLA-DPB1*01:01	Dengue virus 3	ELISPOT	IFNg release	Positive
867442	KLITQFETYQLWTAL	NS2a	1260	1274	HLA-DPB1*02:01	Dengue virus 3	ELISPOT	IFNg release	Positive
867551	LALIATFKIQPFLAL	NS2a	1200	1214	HLA-DPB1*04:01	Dengue virus 3	ELISPOT	IFNg release	Positive
867702	LIATFKIQPFLTLGF	NS2a	1202	1216	HLA-DPB1*01:01	Dengue virus 3	ELISPOT	IFNg release	Positive
868046	MALKLITQFETYQLW	NS2a	1257	1271	HLA-DPB1*01:01	Dengue virus 3	ELISPOT	IFNg release	Positive
868109	MIAGVIFTFVLLLSG	NS2a	1157	1171	HLA-DPB1*04:02	Dengue virus 3	ELISPOT	IFNg release	Positive
868303	PFLALGFFLRKLTSR	NS2a	1210	1224	HLA-DPB1*01:01	Dengue virus 3	ELISPOT	IFNg release	Positive
868329	PLPLFIFSLKDTLKR	NS2a	1328	1342	HLA-DPB1*01:01	Dengue virus 3	ELISPOT	IFNg release	Positive
868551	QPFLALGFFLRKLTS	NS2a	1209	1223	HLA-DPB1*02:01	Dengue virus 3	ELISPOT	IFNg release	Positive
869256	TLTVAWRTATLILAG	NS2a	1285	1299	HLA-DPB1*04:02	Dengue virus 3	ELISPOT	IFNg release	Positive
869298	TQFETYQLWTALISL	NS2a	1263	1277	HLA-DPB1*04:02	Dengue virus 3	ELISPOT	IFNg release	Positive
869592	VQPFLALGFFLRKLT	NS2a	1208	1222	HLA-DPB1*01:01	Dengue virus 3	ELISPOT	IFNg release	Positive
167823	TMRIKDDETENILTVLLKTA	NS2b	83	102	HLA-DRB1*15:01	Dengue virus 3	ELISPOT	IFNg release	Positive
195180	ASSLLRNDVPMAGPL	NS2b	1361	1375	HLA-DRB1*03:01	Dengue virus 3	ELISPOT	IFNg release	Positive
195403	GLVSILASSLLRNDV	NS2b	1355	1369	HLA-DRB1*01:01	Dengue virus 3	ELISPOT	IFNg release	Positive
195789	NILTVLLKTALLIVS	NS2b	1436	1450	HLA-DRB1*01:01	Dengue virus 3	ELISPOT	IFNg release	Positive
195994	SSLLRNDVPMAGPLV	NS2b	1362	1376	HLA-DRB1*03:01	Dengue virus 3	ELISPOT	IFNg release	Positive
196152	VMAVGLVSILASSLL	NS2b	1351	1365	HLA-DRB1*07:01	Dengue virus 3	ELISPOT	IFNg release	Positive
539100	AVGLVSILASSLLRN	NS2b	1353	1367	HLA-DRB1*10:01	Dengue virus 3	ELISPOT	IFNg release	Positive
540087	VSGIFPYSIPATLLV	NS2b	1449	1463	HLA-DRB1*09:01	Dengue virus 3	ELISPOT	IFNg release	Positive
739173	IGLVSILASSLLRND	NS2b	1354	1368	HLA-DRB1*11:04	Dengue virus 3	ELISPOT	IFNg release	Positive
1067120	GKVVGLYGNGVVTKN	NS3	1617	1631	HLA-DRB1*15:01	Dengue virus 3	ELISPOT	IFNg release	Positive
150544	RRLRTLILAPTRVVA	NS3	1688	1702	HLA-DRB1*01:01	Dengue virus 3	ELISPOT	IFNg release	Positive
150617	TFTMRLLSPVRVPNY	NS3	1738	1752	HLA-DRB1*01:01	Dengue virus 3	ELISPOT	IFNg release	Positive
190307	REIVDLMCHATF	NS3	255	266	HLA-DPA1*01:03/DPB1*02:01	Dengue virus 3	ELISA	IL-2 release	Positive
190710	ANCLRKNGKKVIQLS	NS3	1846	1860	HLA-DRB1*13:01	Dengue virus 3	ELISPOT	IFNg release	Positive
190732	EAAAIFMTATPPGTA	NS3	1782	1796	HLA-DRB1*04:03	Dengue virus 3	ELISPOT	IFNg release	Positive
190774	GKTVWFVPSIKAGND	NS3	357	371	HLA-DR2	Dengue virus 3	ELISPOT	IFNg release	Positive
195144	AIVREAIKRRLRTLI	NS3	1680	1694	HLA-DRB1*08:02	Dengue virus 3	ELISPOT	IFNg release	Positive
195243	EGKVVGLYGNGVVTK	NS3	5	19	HLA-DRB1*15:01	Dengue virus 3	ELISPOT	IFNg release	Positive
195297	FHTMWHVTRGAVLTY	NS3	1519	1533	HLA-DRB1*07:01	Dengue virus 3	ELISPOT	IFNg release	Positive
195430	GVFHTMWHVTRGAVL	NS3	1517	1531	HLA-DRB1*07:01	Dengue virus 3	ELISPOT	IFNg release	Positive
195898	RLRTLILAPTRVVAA	NS3	1689	1703	HLA-DRB1*04:03	Dengue virus 3	ELISPOT	IFNg release	Positive
21420	GNEWITDFVGKTVWF	NS3	31	45	HLA-DRB1*15:01	Dengue virus 3	ICS	IFNg release	Positive

28422	IRYQTTATK	NS3	241	249	HLA-DR15	Dengue virus 3	51 chromium	cytotoxicity	Positive
30395	KEGEKKKLRPRWLDA	NS3	585	599	HLA class II	Dengue virus 3	ICS	IFNg release	Positive
38401	LPAIVREAIKRRLRT	NS3	1678	1692	HLA-DRB3*02:02	Dengue virus 3	ELISPOT	IFNg release	Positive
39	AAAIFMTATPPGTAD	NS3	1783	1797	HLA-DRB1*04:03	Dengue virus 3	ELISPOT	IFNg release	Positive
45670	NREGKVVGLYGNGVV	NS3	141	155	HLA-DRB1*15:01	Dengue virus 3	multimer/tetramer	qualitative binding	Positive
49767	PTRVVAAEMEEAMKG	NS3	1697	1711	HLA-DRB1*15:01	Dengue virus 3	ICS	IFNg release	Positive
539028	AAIFMTATPPGTADA	NS3	1784	1798	HLA-DRB1*10:01	Dengue virus 3	ELISPOT	IFNg release	Positive
539052	AGKTVWFVPSIKAGN	NS3	1829	1843	HLA-DRB1*08:03	Dengue virus 3	ELISPOT	IFNg release	Positive
539432	IREAIKRRLRTLILA	NS3	1682	1696	HLA-DRB1*08:03	Dengue virus 3	ELISPOT	IFNg release	Positive
539578	LKGLPIRYQTTATKS	NS3	1709	1723	HLA-DRB1*13:01	Dengue virus 3	ELISPOT	IFNg release	Positive
539607	LPIRYQTTATKSEHT	NS3	1712	1726	HLA-DRB1*13:01	Dengue virus 3	ELISPOT	IFNg release	Positive
539714	NGKKVIQLSRKTFDT	NS3	1852	1866	HLA-DRB1*12:02	Dengue virus 3	ELISPOT	IFNg release	Positive
539777	PKNFQTMPGIFQTTT	NS3	1579	1593	HLA-DRB1*04:03	Dengue virus 3	ELISPOT	IFNg release	Positive
539838	REAIKRRLRTLILAP	NS3	1683	1697	HLA-DRB1*14:04	Dengue virus 3	ELISPOT	IFNg release	Positive
539844	REGKVVGLYGNGVVT	NS3	1615	1629	HLA-DRB1*01:02	Dengue virus 3	ELISPOT	IFNg release	Positive
539991	TMRLLSPVRVPNYNL	NS3	1740	1754	HLA-DRB1*13:01	Dengue virus 3	ELISPOT	IFNg release	Positive
540037	VGKTVWFVPSIKAGN	NS3	1829	1843	HLA-DRB1*08:03	Dengue virus 3	ELISPOT	IFNg release	Positive
540039	VGLYGNGVVTKNGGY	NS3	9	23	HLA-DRB1*15:06	Dengue virus 3	ELISPOT	IFNg release	Positive
54455	RKYLPAIVRE	NS3	202	211	HLA-DR15	Dengue virus 3	51 chromium	cytotoxicity	Positive
55024	RNLTIMDLHPGSGKT	NS3	187	201	HLA class II	Dengue virus 3	ICS	IFNg release	Positive
66127	TRVVAAEMEEA	NS3	225	235	HLA-DR15	Dengue virus 3	51 chromium	cytotoxicity	Positive
68015	VDLMCHATFT	NS3	258	267	HLA-DPw2	Dengue virus 3	51 chromium	cytotoxicity	Positive
70708	VREAIKRRLRTLILA	NS3	1682	1696	HLA-DRB1*12:02	Dengue virus 3	ELISPOT	IFNg release	Positive
72654	WITDFVGKTVW	NS3	1824	1834	HLA-DR15	Dengue virus 3	51 chromium	cytotoxicity	Positive
866771	FQTTTGEIGAIALDF	NS3	1589	1603	HLA-DQA1*03:01/DQB1*03:02	Dengue virus 3	ELISPOT	IFNg release	Positive
867106	HTMWHVTRGAVLTYN	NS3	1520	1534	HLA-DQA1*05:01/DQB1*02:01	Dengue virus 3	ELISPOT	IFNg release	Positive
867358	IVREAIKRRLRTLIL	NS3	1681	1695	HLA-DRB3*02:02	Dengue virus 3	ELISPOT	IFNg release	Positive
867534	KYLPAIVREAIKRRL	NS3	1676	1690	HLA-DRB4*01:01	Dengue virus 3	ELISPOT	IFNg release	Positive
867875	LNDWDFVVTTDISEM	NS3	1873	1887	HLA-DQA1*03:01/DQB1*03:02	Dengue virus 3	ELISPOT	IFNg release	Positive
869278	TMWHVTRGAVLTHNG	NS3	1521	1535	HLA-DQA1*05:01/DQB1*02:01	Dengue virus 3	ELISPOT	IFNg release	Positive
869689	WHVTRGAVLTYNGKR	NS3	1523	1537	HLA-DQA1*05:01/DQB1*02:01	Dengue virus 3	ELISPOT	IFNg release	Positive
167755	IALDLVTEIGRVPSHLAHRT	NS4a	2	21	HLA class II	Dengue virus 3	ELISPOT	IFNg release	Positive
195199	AYVVIGILTLAAIVA	NS4a	2227	2241	HLA-DRB1*01:01	Dengue virus 3	ELISPOT	IFNg release	Positive
195522	IPLQWIASAIVLEFF	NS4a	2192	2206	HLA-DRB1*01:01	Dengue virus 3	ELISPOT	IFNg release	Positive
195742	MADVPLQWIASAIVL	NS4a	2189	2203	HLA-DRB1*07:01	Dengue virus 3	ELISPOT	IFNg release	Positive
195828	PETMETLLLLGLMIL	NS4a	2141	2155	HLA-DRB1*15:06	Dengue virus 3	ELISPOT	IFNg release	Positive
196056	TLLLLGLMILLTGGA	NS4a	2146	2160	HLA-DRB1*15:06	Dengue virus 3	ELISPOT	IFNg release	Positive
196168	VPLQWIASAIVLEFF	NS4a	2192	2206	HLA-DRB1*01:01	Dengue virus 3	ELISPOT	IFNg release	Positive
539900	SGMLWMADVPLQWIA	NS4a	2184	2198	HLA-DRB1*04:03	Dengue virus 3	ELISPOT	IFNg release	Positive
739020	GIGKTSIGLICVIVS	NS4a	2169	2183	HLA-DRB1*11:04	Dengue virus 3	ELISPOT	IFNg release	Positive
739464	LFLISGKGIGKTSIG	NS4a	2162	2176	HLA-DRB1*01:02	Dengue virus 3	ELISPOT	IFNg release	Positive
869474	VIGILTLAAIIAANE	NS4a	2230	2244	HLA-DQA1*01:02/DQB1*06:02	Dengue virus 3	ELISPOT	IFNg release	Positive
195346	GAGLAFSIMKSVGTG	NS4b	2474	2488	HLA-DRB1*04:01	Dengue virus 3	ELISPOT	IFNg release	Positive
195607	LAFSIMKSVGTGKRG	NS4b	2477	2491	HLA-DRB1*04:01	Dengue virus 3	ELISPOT	IFNg release	Positive
195617	LCAVQLLLMRTSWAL	NS4b	2418	2432	HLA-DRB1*15:01	Dengue virus 3	ELISPOT	IFNg release	Positive
195624	LDPVIYDSKFEKQLG	NS4b	2397	2411	HLA-DRB1*03:01	Dengue virus 3	ELISPOT	IFNg release	Positive
195663	LLLITHYAIIGPGLQ	NS4b	2354	2368	HLA-DRB1*07:01	Dengue virus 3	ELISPOT	IFNg release	Positive
195664	LLLMRTSWALCEALT	NS4b	2423	2437	HLA-DRB1*04:01	Dengue virus 3	ELISPOT	IFNg release	Positive
195807	NPLTLTAAVLLLITH	NS4b	2345	2359	HLA-DRB1*07:01	Dengue virus 3	ELISPOT	IFNg release	Positive
195871	QVMLLVLCAVQLLLM	NS4b	2412	2426	HLA-DRB1*01:01	Dengue virus 3	ELISPOT	IFNg release	Positive
196023	TAAVLLLITHYAIIG	NS4b	2350	2364	HLA-DRB1*15:01	Dengue virus 3	ELISPOT	IFNg release	Positive
196137	VLCAVQLLLMRTSWA	NS4b	2417	2431	HLA-DRB1*12:02	Dengue virus 3	ELISPOT	IFNg release	Positive
196155	VMLLVLCAVQLLLMR	NS4b	2413	2427	HLA-DRB1*03:01	Dengue virus 3	ELISPOT	IFNg release	Positive
196163	VNPLTLTAAVLLLIT	NS4b	2344	2358	HLA-DRB1*01:01	Dengue virus 3	ELISPOT	IFNg release	Positive
738453	AGLAFSIMKSVGTGK	NS4b	2475	2489	HLA-DRB1*04:07	Dengue virus 3	ELISPOT	IFNg release	Positive
867986	LTLTAAVLLLATHYA	NS4b	2347	2361	HLA-DQA1*01:02/DQB1*06:02	Dengue virus 3	ELISPOT	IFNg release	Positive
868878	SLAAIANQAVVLMGL	NS4b	2306	2320	HLA-DQA1*01:02/DQB1*06:02	Dengue virus 3	ELISPOT	IFNg release	Positive
869526	VLLLVTHYAIIGPGL	NS4b	2353	2367	HLA-DPB1*02:01	Dengue virus 3	ELISPOT	IFNg release	Positive
167753	HVNAEPETPNMDVIGERIKR	NS5	263	282	HLA class II	Dengue virus 3	ELISPOT	IFNg release	Positive
167769	KKLNQLSRKEFDL	NS5	15	27	HLA-DP	Dengue virus 3	ELISPOT	IFNg release	Positive
195136	AIFKLTYQNKVVKVQ	NS5	3056	3070	HLA-DRB1*09:01	Dengue virus 3	ELISPOT	IFNg release	Positive
195204	CLGKAYAQMWSLMYF	NS5	3243	3257	HLA-DRB1*04:01	Dengue virus 3	ELISPOT	IFNg release	Positive
195273	ETKGVERLKRMAISG	NS5	3138	3152	HLA-DRB1*11:01	Dengue virus 3	ELISPOT	IFNg release	Positive
195382	GKVRKDIPQWQPSKG	NS5	3175	3189	HLA-DRB1*03:01	Dengue virus 3	ELISPOT	IFNg release	Positive
195408	GNIVASVNMVSRLLL	NS5	3	17	HLA-DRB1*03:01	Dengue virus 3	ELISPOT	IFNg release	Positive
195410	GNIVSSVNMVSRLLL	NS5	2715	2729	HLA-DRB1*03:01	Dengue virus 3	ELISPOT	IFNg release	Positive
195437	GYILRDISKIPGGAM	NS5	43	57	HLA-DRB1*08:02	Dengue virus 3	ELISPOT	IFNg release	Positive
195568	KHGGMLVRNPLSRNS	NS5	2689	2703	HLA-DRB1*08:02	Dengue virus 3	ELISPOT	IFNg release	Positive
195641	LHKLGYILRDISKIP	NS5	39	53	HLA-DRB1*03:01	Dengue virus 3	ELISPOT	IFNg release	Positive
195668	LLNRFTMTHRRPTIE	NS5	16	30	HLA-DRB1*11:01	Dengue virus 3	ELISPOT	IFNg release	Positive
195779	NDMGKVRKDIPQWQP	NS5	3172	3186	HLA-DRB1*03:01	Dengue virus 3	ELISPOT	IFNg release	Positive
195780	NEDHWFSRDNSYSGV	NS5	2982	2996	HLA-DRB1*04:01	Dengue virus 3	ELISPOT	IFNg release	Positive
195806	NMVSRLLLNRFTMTH	NS5	2722	2736	HLA-DRB1*08:02	Dengue virus 3	ELISPOT	IFNg release	Positive
195812	NRFTMTHRRPTIEKD	NS5	18	32	HLA-DRB1*11:01	Dengue virus 3	ELISPOT	IFNg release	Positive
195875	RAIWYMWLGARYLEF	NS5	2961	2975	HLA-DRB1*01:01	Dengue virus 3	ELISPOT	IFNg release	Positive
195939	SCVYNMMGKREKKLG	NS5	2938	2952	HLA-DRB1*11:01	Dengue virus 3	ELISPOT	IFNg release	Positive
196038	TGNIVASVNMVSRLL	NS5	2	16	HLA-DRB1*04:01	Dengue virus 3	ELISPOT	IFNg release	Positive
196041	TGNIVSSVNMVSRLL	NS5	2714	2728	HLA-DRB1*04:01	Dengue virus 3	ELISPOT	IFNg release	Positive
196162	VNMVSRLLLNRFTMT	NS5	9	23	HLA-DRB1*11:01	Dengue virus 3	ELISPOT	IFNg release	Positive
196221	WLWRTLGRNKKPRLC	NS5	157	171	HLA-DRB1*14:02	Dengue virus 3	ELISPOT	IFNg release	Positive
196222	WNIVKLMSGKDVFYL	NS5	2611	2625	HLA-DRB1*15:01	Dengue virus 3	ELISPOT	IFNg release	Positive
196224	WSLMYFHRRDLRLAS	NS5	3252	3266	HLA-DRB1*03:01	Dengue virus 3	ELISPOT	IFNg release	Positive
196239	YQNKVVKVQRPTPKG	NS5	3062	3076	HLA-DRB1*08:02	Dengue virus 3	ELISPOT	IFNg release	Positive
231420	RYLEFEALGFLNEDH	NS5	2971	2985	HLA-DPB1*04:01	Dengue virus 3	ELISPOT	IFNg release	Positive
32944	KPWDVVPTV	NS5	2819	2827	HLA class II	Dengue virus 3	ELISA	IL-2 release	Positive
539444	IVASVNMVSRLLLNR	NS5	2717	2731	HLA-DRB1*12:02	Dengue virus 3	ELISPOT	IFNg release	Positive
539449	IVSSVNMVSRLLLNR	NS5	2717	2731	HLA-DRB1*11:04	Dengue virus 3	ELISPOT	IFNg release	Positive
539458	KCGSCVYNMMGKREK	NS5	2935	2949	HLA-DRB1*13:01	Dengue virus 3	ELISPOT	IFNg release	Positive
539600	LMYFHRRDLRLASNA	NS5	3254	3268	HLA-DRB1*13:01	Dengue virus 3	ELISPOT	IFNg release	Positive
539604	LNTFTNMEAQLVRQM	NS5	3098	3112	HLA-DRB1*10:01	Dengue virus 3	ELISPOT	IFNg release	Positive
539704	NAIFKLTYQNKVVKV	NS5	3055	3069	HLA-DRB1*09:01	Dengue virus 3	ELISPOT	IFNg release	Positive

539726	NIVASVNMVSRLLLN	NS5	2716	2730	HLA-DRB1*15:06	Dengue virus 3	ELISPOT	IFNg release	Positive
539739	NMEVQLVRQMEGEGV	NS5	3103	3117	HLA-DRB1*13:01	Dengue virus 3	ELISPOT	IFNg release	Positive
539831	RATWAQNIPTAIQQV	NS5	3346	3360	HLA-DRB1*09:01	Dengue virus 3	ELISPOT	IFNg release	Positive
539872	RRDLRLASNAICSAV	NS5	3259	3273	HLA-DRB1*04:03	Dengue virus 3	ELISPOT	IFNg release	Positive
539916	SLMYFHRRDLRLASN	NS5	3253	3267	HLA-DRB1*13:01	Dengue virus 3	ELISPOT	IFNg release	Positive
539945	SVNMVSRLLLNRFTM	NS5	2720	2734	HLA-DRB1*12:02	Dengue virus 3	ELISPOT	IFNg release	Positive
540019	TYGLNTFTNMEAQLV	NS5	3095	3109	HLA-DRB1*04:03	Dengue virus 3	ELISPOT	IFNg release	Positive
540025	VASVNMVSRLLLNRF	NS5	2718	2732	HLA-DRB1*14:04	Dengue virus 3	ELISPOT	IFNg release	Positive
540090	VSSVNMVSRLLLNRF	NS5	2718	2732	HLA-DRB1*14:04	Dengue virus 3	ELISPOT	IFNg release	Positive
540152	YKTWAYHGSYEVKAT	NS5	2789	2803	HLA-DRB1*09:01	Dengue virus 3	ELISPOT	IFNg release	Positive
591602	ACLGKAYAQMWTLMY	NS5	3242	3256	HLA-DRB1*04:01	Dengue virus 3	ELISPOT	IFNg release	Positive
738537	ASVNMVSRLLLNRFT	NS5	7	21	HLA-DRB1*14:06	Dengue virus 3	ELISPOT	IFNg release	Positive
738753	EEFLDYMPSMKRFRK	NS5	3367	3381	HLA-DRB1*14:02	Dengue virus 3	ELISPOT	IFNg release	Positive
738759	EFLDYMPSMKRFRKE	NS5	3368	3382	HLA-DRB1*14:06	Dengue virus 3	ELISPOT	IFNg release	Positive
738829	ERELHKLGKCGSCVY	NS5	2927	2941	HLA-DRB1*11:04	Dengue virus 3	ELISPOT	IFNg release	Positive
740771	YGLNTFTNMEVQLVR	NS5	3096	3110	HLA-DRB1*04:07	Dengue virus 3	ELISPOT	IFNg release	Positive
857703	IWYMWLGARYLEFEA	NS5	2963	2977	HLA-DPB1*04:01	Dengue virus 3	ELISPOT	IFNg release	Positive
866168	ARYLEFEALGFLNED	NS5	2970	2984	HLA-DQA1*03:01/DQB1*03:02	Dengue virus 3	ELISPOT	IFNg release	Positive
867015	GSCVYNMMGKREKKL	NS5	2937	2951	HLA-DRB5*01:01	Dengue virus 3	ELISPOT	IFNg release	Positive
867376	KAYAQMWSLMYFHRR	NS5	3246	3260	HLA-DRB5*01:01	Dengue virus 3	ELISPOT	IFNg release	Positive
868338	PMSTYGWNIVKLMSG	NS5	2605	2619	HLA-DQA1*01:01/DQB1*05:01	Dengue virus 3	ELISPOT	IFNg release	Positive
868748	SAKLQWFVERNMVIP	NS5	2549	2563	HLA-DQA1*01:01/DQB1*05:01	Dengue virus 3	ELISPOT	IFNg release	Positive
869489	VKPIDDRFANALLAL	NS5	3157	3171	HLA-DPB1*01:01	Dengue virus 3	ELISPOT	IFNg release	Positive
869705	WYMWLGARYLEFEAL	NS5	2964	2978	HLA-DQA1*05:01/DQB1*02:01	Dengue virus 3	ELISPOT	IFNg release	Positive

CD4 DENV-4 Epitopes

Epitope_ID	AminoAcid_Seq	Protein_Name	Start	End	Allele	Serotype	Method/Technique	Assay Group	Qualitative Measure
1067635	LKRWGQLKKNKAIKI	C	65	79	HLA-DRB1*07:01	Dengue virus 4	ELISPOT	IFNg release	Positive-Low
1068575	VVRPPFNMLKRERNR	C	7	21	HLA-DRB1*11:01	Dengue virus 4	ELISPOT	IFNg release	Positive-Low
17605	FRKEIGRML	C	83	91	HLA-DPw4	Dengue virus 4	51 chromium	cytotoxicity	Positive
195316	FNMLKRERNRVSTPQ	C	12	26	HLA-DRB1*04:01	Dengue virus 4	ELISPOT	IFNg release	Positive
195325	FRKEIGRMLNILNGR	C	19	33	HLA-DRB1*04:01	Dengue virus 4	ELISPOT	IFNg release	Positive
195483	IGFRKEIGRMLNILN	C	81	95	HLA-DRB1*04:01	Dengue virus 4	ELISPOT	IFNg release	Positive
195488	IGRMLNILNRRKRST	C	87	101	HLA-DRB1*14:02	Dengue virus 4	ELISPOT	IFNg release	Positive
195580	KNKAIKILIGFRKEI	C	53	67	HLA-DRB1*15:01	Dengue virus 4	ELISPOT	IFNg release	Positive
195587	KRWGQLKKNKAIKIL	C	46	60	HLA-DRB1*08:03	Dengue virus 4	ELISPOT	IFNg release	Positive
195651	LKKNKAIKILTGFRK	C	51	65	HLA-DRB1*14:04	Dengue virus 4	ELISPOT	IFNg release	Positive
195768	MTLLCLIPTAMAFHL	C	102	116	HLA-DRB1*01:01	Dengue virus 4	ELISPOT	IFNg release	Positive
195900	RMVLAFITFLRVLSI	C	44	58	HLA-DRB1*11:01	Dengue virus 4	ELISPOT	IFNg release	Positive
195922	RWGQLKKTKAIKILT	C	67	81	HLA-DRB1*07:01	Dengue virus 4	ELISPOT	IFNg release	Positive
196065	TPQGLVKRFSSGLFS	C	24	38	HLA-DRB1*15:01	Dengue virus 4	ELISPOT	IFNg release	Positive
196134	VLAFITFLRVLSIPP	C	26	40	HLA-DRB1*04:01	Dengue virus 4	ELISPOT	IFNg release	Positive
36533	LIGFRKEIGRML	C	80	91	HLA-DPw4	Dengue virus 4	ICS	IFNg release	Positive
539044	AFITFLRVLSIPPTA	C	28	42	HLA-DRB1*04:03	Dengue virus 4	ELISPOT	IFNg release	Positive
539255	FITFLRVLSIPPTAG	C	49	63	HLA-DRB1*04:07	Dengue virus 4	ELISPOT	IFNg release	Positive
539294	GFRKEIGRMLNILNG	C	54	68	HLA-DRB3*02:02	Dengue virus 4	ELISPOT	IFNg release	Positive
539496	KNKAIKILTGFRKEI	C	53	67	HLA-DRB1*12:02	Dengue virus 4	ELISPOT	IFNg release	Positive
539536	LAFITFLRVLSIPPT	C	27	41	HLA-DRB1*12:02	Dengue virus 4	ELISPOT	IFNg release	Positive
539571	LIGFRKEIGRMLNIL	C	80	94	HLA-DRB1*09:01	Dengue virus 4	ELISPOT	IFNg release	Positive
539581	LKKNKAIKILIGFRK	C	51	65	HLA-DRB1*14:04	Dengue virus 4	ELISPOT	IFNg release	Positive
539632	LTGFRKEIGRMLNIL	C	52	66	HLA-DRB3*02:02	Dengue virus 4	ELISPOT	IFNg release	Positive
539898	SGKGPLRMVLAFITF	C	18	32	HLA-DRB1*15:06	Dengue virus 4	ELISPOT	IFNg release	Positive
539987	TLLCLIPTVMAFHLS	C	103	117	HLA-DRB1*04:03	Dengue virus 4	ELISPOT	IFNg release	Positive
540116	WGQLKKNKAIKILIG	C	68	82	HLA-DRB1*09:01	Dengue virus 4	ELISPOT	IFNg release	Positive
69367	VLAFITFLR	C	46	54	HLA-DPw4	Dengue virus 4	51 chromium	cytotoxicity	Positive
739059	GRMLNILNGRKRSTI	C	88	102	HLA-DRB1*14:06	Dengue virus 4	ELISPOT	IFNg release	Positive
739060	GRMLNILNGRKRSTM	C	88	102	HLA-DRB1*14:02	Dengue virus 4	ELISPOT	IFNg release	Positive
739175	IGRMLNILNGRKRST	C	87	101	HLA-DRB1*14:02	Dengue virus 4	ELISPOT	IFNg release	Positive
739178	IGRMLNILNRRRRST	C	87	101	HLA-DRB1*14:02	Dengue virus 4	ELISPOT	IFNg release	Positive
740156	RPPFNMLKRERNRVS	C	9	23	HLA-DRB1*14:02	Dengue virus 4	ELISPOT	IFNg release	Positive
868517	QLKKNKAIKILTGFR	C	42	56	HLA-DQA1*01:02/DQB1*06:02	Dengue virus 4	ELISPOT	IFNg release	Positive
868732	RWGQLKKNKAIKILI	C	39	53	HLA-DRB3*02:02	Dengue virus 4	ELISPOT	IFNg release	Positive
195283	FALLAGFMAYMIGQT	PreM	246	260	HLA-DRB1*01:01	Dengue virus 4	ELISPOT	IFNg release	Positive
195505	ILRNPGFALLAGFMA	PreM	240	254	HLA-DRB1*01:01	Dengue virus 4	ELISPOT	IFNg release	Positive
195526	IQRTVFFILMMLVAP	PreM	262	276	HLA-DRB1*07:01	Dengue virus 4	ELISPOT	IFNg release	Positive
195901	RNPGFALLAGFMAYM	PreM	242	256	HLA-DRB1*15:01	Dengue virus 4	ELISPOT	IFNg release	Positive
195916	RTVFFVLMMLVAPSY	PreM	264	278	HLA-DRB1*01:01	Dengue virus 4	ELISPOT	IFNg release	Positive
539395	IDCWCNLTSAWVMYG	PreM	117	131	HLA-DQA1*01:02/DQB1*06:02	Dengue virus 4	ELISPOT	IFNg release	Positive
539820	QRTVFFILMMLVAPS	PreM	263	277	HLA-DRB1*13:01	Dengue virus 4	ELISPOT	IFNg release	Positive
867174	IGQTGIQRTVFFILM	PreM	257	271	HLA-DPB1*02:01	Dengue virus 4	ELISPOT	IFNg release	Positive
869119	TEGINKCTLIAMDLG	PreM	113	127	HLA-DQA1*01:02/DQB1*06:02	Dengue virus 4	ELISPOT	IFNg release	Positive
869670	WCNLTSTWVMYGTCT	PreM	180	194	HLA-DQA1*01:02/DQB1*06:02	Dengue virus 4	ELISPOT	IFNg release	Positive
167808	RDVNKEKVVGRVISSTPLAE	E	340	359	HLA class II	Dengue virus 4	ELISPOT	IFNg release	Positive
195202	CIAVGGITLFLGFTV	E	758	772	HLA-DRB1*15:01	Dengue virus 4	ELISPOT	IFNg release	Positive
195227	DSYIVIGVGNSALTL	E	654	668	HLA-DRB1*15:06	Dengue virus 4	ELISPOT	IFNg release	Positive
195272	ESTYRGAKRMAILGE	E	682	696	HLA-DRB1*15:06	Dengue virus 4	ELISPOT	IFNg release	Positive
195358	GGLFTSLGKAVHQVF	E	705	719	HLA-DRB1*11:01	Dengue virus 4	ELISPOT	IFNg release	Positive
195364	GGVSWMIRILIGILV	E	449	463	HLA-DRB1*15:06	Dengue virus 4	ELISPOT	IFNg release	Positive
195434	GVSWMIRILIGFLVL	E	729	743	HLA-DRB1*08:02	Dengue virus 4	ELISPOT	IFNg release	Positive
195435	GVSWMIRILIGLLVL	E	729	743	HLA-DRB1*15:06	Dengue virus 4	ELISPOT	IFNg release	Positive
195436	GVSWMVRILIGFLVL	E	450	464	HLA-DRB1*08:02	Dengue virus 4	ELISPOT	IFNg release	Positive
195472	IAVGGITLFLGFTVH	E	480	494	HLA-DRB1*15:01	Dengue virus 4	ELISPOT	IFNg release	Positive
195473	IAVGGITLFLGFTVQ	E	759	773	HLA-DRB1*15:01	Dengue virus 4	ELISPOT	IFNg release	Positive
195625	LESTYRGAKRMAILG	E	681	695	HLA-DRB1*14:02	Dengue virus 4	ELISPOT	IFNg release	Positive
196036	TGIQRTVFFVLMMLV	E	44	58	HLA-DRB1*07:01	Dengue virus 4	ELISPOT	IFNg release	Positive
196189	VSWIIRILIGLLVLW	E	451	465	HLA-DRB1*15:06	Dengue virus 4	ELISPOT	IFNg release	Positive
539230	EMILMKMKKKTWLVH	E	474	488	HLA-DRB1*12:02	Dengue virus 4	ELISPOT	IFNg release	Positive
539298	GGVSWMVRILIGLLV	E	449	463	HLA-DRB1*15:06	Dengue virus 4	ELISPOT	IFNg release	Positive
539379	HAKRQDVTVLGSQEGAMH	E	523	540	HLA class II	Dengue virus 4	ICS	IFNg release	Positive
539463	KERMVTFKVPHAKRQ	E	513	527	HLA-DRB1*04:03	Dengue virus 4	ELISPOT	IFNg release	Positive
539734	NLEYTVVVTVHNGDT	E	197	211	HLA-DRB1*04:07	Dengue virus 4	ELISPOT	IFNg release	Positive
540036	VGGLFTSLGKAVHQV	E	704	718	HLA-DRB1*09:01	Dengue virus 4	ELISPOT	IFNg release	Positive
540051	VLGSQEGAMHSALAGA	E	531	546	HLA class II	Dengue virus 4	ICS	IFNg release	Positive
540080	VRILIGFLVLWIGTN	E	455	469	HLA-DRB1*15:06	Dengue virus 4	ELISPOT	IFNg release	Positive
738809	ENLEYTVVVTVHNGD	E	196	210	HLA-DRB1*04:07	Dengue virus 4	ELISPOT	IFNg release	Positive
738886	FESTYRGAKRMAILG	E	681	695	HLA-DRB1*14:02	Dengue virus 4	ELISPOT	IFNg release	Positive
741747	MRCVGVGNRDFVEGVSGGAW	E	280	299	HLA class II	Dengue virus 4	ELISPOT	IFNg release	Positive
195791	NIMWKQITNELNYVL	NS1	839	853	HLA-DRB1*04:07	Dengue virus 4	ELISPOT	IFNg release	Positive
195822	NVMWKQITNELNYVL	NS1	1	15	HLA-DRB1*04:07	Dengue virus 4	ELISPOT	IFNg release	Positive
539829	QYQFQPESPARLASA	NS1	805	819	HLA-DRB1*09:01	Dengue virus 4	ELISPOT	IFNg release	Positive
866696	FGMFTTNIWMKFREG	NS1	934	948	HLA-DPB1*04:01	Dengue virus 4	ELISPOT	IFNg release	Positive
867043	HADMGYWIESSKNQT	NS1	969	983	HLA-DQA1*01:01/DQB1*05:01	Dengue virus 4	ELISPOT	IFNg release	Positive
167725	AMTTTLSIPHDLMELIDGIS	NS2a	50	69	HLA class II	Dengue virus 4	ELISPOT	IFNg release	Positive
195156	ALSLTFIRSTMPLVM	NS2a	1274	1288	HLA-DRB1*07:01	Dengue virus 4	ELISPOT	IFNg release	Positive
195157	ALSLTFIRSTMSLVM	NS2a	1274	1288	HLA-DRB1*04:01	Dengue virus 4	ELISPOT	IFNg release	Positive
195158	ALSLTFIRSTTPLVM	NS2a	1274	1288	HLA-DRB1*07:01	Dengue virus 4	ELISPOT	IFNg release	Positive
195190	AVFKMSPGYVLGVFL	NS2a	1204	1218	HLA-DRB1*01:01	Dengue virus 4	ELISPOT	IFNg release	Positive
195220	DLMEFIDGISLGLIL	NS2a	1244	1258	HLA-DRB1*07:01	Dengue virus 4	ELISPOT	IFNg release	Positive
195221	DLMELISGISLGLIL	NS2a	1243	1257	HLA-DRB1*07:01	Dengue virus 4	ELISPOT	IFNg release	Positive
195541	ITALILGAQALPVYL	NS2a	1320	1334	HLA-DRB1*01:01	Dengue virus 4	ELISPOT	IFNg release	Positive
195571	KHMILVVVTTLCAII	NS2a	1156	1170	HLA-DRB1*08:02	Dengue virus 4	ELISPOT	IFNg release	Positive
195608	LAIMAVFKMSPGYVL	NS2a	1200	1214	HLA-DRB1*13:01	Dengue virus 4	ELISPOT	IFNg release	Positive

195699	LQKQSHWVEITALIL	NS2a	1311	1325	HLA-DRB1*07:01	Dengue virus 4	ELISPOT	IFNg release	Positive
195746	MAVFKMSPGYVLGVF	NS2a	1203	1217	HLA-DRB1*07:01	Dengue virus 4	ELISPOT	IFNg release	Positive
195837	PLVMAWRTIMAVLFV	NS2a	1285	1299	HLA-DRB1*01:01	Dengue virus 4	ELISPOT	IFNg release	Positive
195864	QSHWVEITALILGAQ	NS2a	1314	1328	HLA-DRB1*07:01	Dengue virus 4	ELISPOT	IFNg release	Positive
196060	TMPLVMAWRTIMAVL	NS2a	1283	1297	HLA-DRB1*03:01	Dengue virus 4	ELISPOT	IFNg release	Positive
196061	TMSLVMAWRTIMAVL	NS2a	1283	1297	HLA-DRB1*03:01	Dengue virus 4	ELISPOT	IFNg release	Positive
196070	TRKHMILAVVITLCA	NS2a	1154	1168	HLA-DRB1*08:02	Dengue virus 4	ELISPOT	IFNg release	Positive
196227	WVEITALILGAQALP	NS2a	1317	1331	HLA-DRB1*01:01	Dengue virus 4	ELISPOT	IFNg release	Positive
196252	YVLGIFLRKLTSRET	NS2a	1212	1226	HLA-DRB1*11:01	Dengue virus 4	ELISPOT	IFNg release	Positive
196253	YVLGVFLRKLTSRET	NS2a	1212	1226	HLA-DRB1*11:01	Dengue virus 4	ELISPOT	IFNg release	Positive
539130	CLRRRVTRKHMILAV	NS2a	1148	1162	HLA-DRB1*08:03	Dengue virus 4	ELISPOT	IFNg release	Positive
539131	CLRRRVTRKHMILVV	NS2a	1148	1162	HLA-DRB1*08:03	Dengue virus 4	ELISPOT	IFNg release	Positive
539198	ECLRRRVTRKHMILA	NS2a	1147	1161	HLA-DRB1*13:01	Dengue virus 4	ELISPOT	IFNg release	Positive
539360	GVFLRKLTSRETALM	NS2a	1215	1229	HLA-DRB1*10:01	Dengue virus 4	ELISPOT	IFNg release	Positive
539406	IGGQIHLAIMAVFKM	NS2a	1194	1208	HLA-DRB1*13:01	Dengue virus 4	ELISPOT	IFNg release	Positive
539407	IGGQTHLAIMAVFKM	NS2a	1194	1208	HLA-DRB1*13:01	Dengue virus 4	ELISPOT	IFNg release	Positive
539433	IRSTMPLVMAWRTIM	NS2a	1280	1294	HLA-DRB1*13:01	Dengue virus 4	ELISPOT	IFNg release	Positive
539434	IRSTMSLVMAWRTIM	NS2a	1280	1294	HLA-DRB1*13:01	Dengue virus 4	ELISPOT	IFNg release	Positive
539555	LGIFLRKLTSRETAL	NS2a	1214	1228	HLA-DRB1*08:03	Dengue virus 4	ELISPOT	IFNg release	Positive
539565	LGVFLRKLTSRETAL	NS2a	1214	1228	HLA-DRB1*08:03	Dengue virus 4	ELISPOT	IFNg release	Positive
539627	LSLTFIRSTMPLVMA	NS2a	1275	1289	HLA-DRB1*10:01	Dengue virus 4	ELISPOT	IFNg release	Positive
539628	LSLTFIRSTTPLVMA	NS2a	1275	1289	HLA-DRB1*08:03	Dengue virus 4	ELISPOT	IFNg release	Positive
539664	MGGQIHLAIMAVFKM	NS2a	1194	1208	HLA-DRB1*13:01	Dengue virus 4	ELISPOT	IFNg release	Positive
539687	MPLVMAWRTIMAVLF	NS2a	1284	1298	HLA-DRB1*12:02	Dengue virus 4	ELISPOT	IFNg release	Positive
539919	SLTFIRSTMPLVMAW	NS2a	1276	1290	HLA-DRB1*09:01	Dengue virus 4	ELISPOT	IFNg release	Positive
539921	SLTFIRSTTPLIMAW	NS2a	1276	1290	HLA-DRB1*04:03	Dengue virus 4	ELISPOT	IFNg release	Positive
539930	SRETALMVIGMAMTT	NS2a	1223	1237	HLA-DRB1*15:06	Dengue virus 4	ELISPOT	IFNg release	Positive
539937	STMPLVMAWRTIMAV	NS2a	1282	1296	HLA-DRB1*13:01	Dengue virus 4	ELISPOT	IFNg release	Positive
539961	TALMVIGMAMTTTLS	NS2a	1226	1240	HLA-DRB1*01:02	Dengue virus 4	ELISPOT	IFNg release	Positive
539962	TALMVIGMAMTTVLS	NS2a	1226	1240	HLA-DRB1*15:06	Dengue virus 4	ELISPOT	IFNg release	Positive
540033	VFKMSPGYVLGVFLR	NS2a	1205	1219	HLA-DRB1*09:01	Dengue virus 4	ELISPOT	IFNg release	Positive
540053	VLGVFLRKLTSRETA	NS2a	1213	1227	HLA-DRB1*14:04	Dengue virus 4	ELISPOT	IFNg release	Positive
738559	AVVITLCAIILGGLT	NS2a	1161	1175	HLA-DRB1*01:02	Dengue virus 4	ELISPOT	IFNg release	Positive
738742	ECLRRRVTRKHMILV	NS2a	1147	1161	HLA-DRB1*14:02	Dengue virus 4	ELISPOT	IFNg release	Positive
740701	VVTLIPLCRTSCLQK	NS2a	1299	1313	HLA-DRB1*11:04	Dengue virus 4	ELISPOT	IFNg release	Positive
867048	HDLMEFIDGISLGLI	NS2a	1243	1257	HLA-DQA1*01:01/DQB1*05:01	Dengue virus 4	ELISPOT	IFNg release	Positive
867110	HWVEITALILGAQAL	NS2a	1316	1330	HLA-DQA1*03:01/DQB1*03:02	Dengue virus 4	ELISPOT	IFNg release	Positive
867874	LMVIGMAMTTVLSIP	NS2a	1228	1242	HLA-DQA1*01:02/DQB1*06:02	Dengue virus 4	ELISPOT	IFNg release	Positive
867969	LTFIRSTTPLVMAWR	NS2a	1277	1291	HLA-DRB3*02:02	Dengue virus 4	ELISPOT	IFNg release	Positive
195404	GLVSLLGSALLKNDV	NS2b	9	23	HLA-DRB1*01:02	Dengue virus 4	ELISPOT	IFNg release	Positive
195511	IMAVGLVSLLGSALL	NS2b	1352	1366	HLA-DRB1*01:01	Dengue virus 4	ELISPOT	IFNg release	Positive
195662	LLLAAYVMSGSSADL	NS2b	1381	1395	HLA-DRB1*04:01	Dengue virus 4	ELISPOT	IFNg release	Positive
195748	MAVGLVSLLGSALLK	NS2b	1353	1367	HLA-DRB1*01:01	Dengue virus 4	ELISPOT	IFNg release	Positive
195931	SALLKNDVPLAGPMV	NS2b	1363	1377	HLA-DRB1*03:01	Dengue virus 4	ELISPOT	IFNg release	Positive
196062	TNMITLLVKLALITV	NS2b	1436	1450	HLA-DRB1*08:02	Dengue virus 4	ELISPOT	IFNg release	Positive
539101	AVGLVSLLGSALLKN	NS2b	1354	1368	HLA-DRB1*10:01	Dengue virus 4	ELISPOT	IFNg release	Positive
869635	VTMTLWYMWQVKTQR	NS2b	1460	1474	HLA-DPB1*01:01	Dengue virus 4	ELISPOT	IFNg release	Positive
1067119	GKVIGLYGNGVVTKS	NS3	1618	1632	HLA-DRB1*15:01	Dengue virus 4	ELISPOT	IFNg release	Positive
12605	EIVDLMCHAT	NS3	255	264	HLA-DPw2	Dengue virus 4	51 chromium	cytotoxicity	Positive
1392192	GREIVDLMCHATFTT	NS3	1727	1741		Dengue virus 4	biological activity	activation	Positive
156711	LAPTRVVAAEMEEAL	NS3	1695	1709	HLA class II	Dengue virus 4	51 chromium	cytotoxicity	Positive
195244	EGVYRIMQRGLFGKT	NS3	1494	1508	HLA-DRB1*11:01	Dengue virus 4	ELISPOT	IFNg release	Positive
195298	FHTMWHVTRGSVICH	NS3	1520	1534	HLA-DRB1*07:01	Dengue virus 4	ELISPOT	IFNg release	Positive
195353	GEIGAVTLDFKPGTS	NS3	1595	1609	HLA-DRB1*03:01	Dengue virus 4	ELISPOT	IFNg release	Positive
195563	KGKVIGLYGNGVVTK	NS3	5	19	HLA-DRB1*15:01	Dengue virus 4	ELISPOT	IFNg release	Positive
195695	LPVWLSYKVASAGIS	NS3	2016	2030	HLA-DRB1*08:02	Dengue virus 4	ELISPOT	IFNg release	Positive
195838	PNYNLIVMDEAHFTD	NS3	1750	1764	HLA-DRB1*03:01	Dengue virus 4	ELISPOT	IFNg release	Positive
195962	SIVREALKRRLRTLI	NS3	1680	1694	HLA-DRB1*11:01	Dengue virus 4	ELISPOT	IFNg release	Positive
196032	TEGVYRIMQRGLFGK	NS3	146	160	HLA-DRB1*11:01	Dengue virus 4	ELISPOT	IFNg release	Positive
33192	KRLTIMDLHPGAGKT	NS3	186	200	HLA class II	Dengue virus 4	ICS	IFNg release	Positive
45689	NRKGKIVGLYGNGVV	NS3	141	155	HLA-DRB1*15:01	Dengue virus 4	ICS	IFNg release	Positive
53505	REGEKKKLRPRWLDR	NS3	585	599	HLA class II	Dengue virus 4	ICS	IFNg release	Positive
539020	AAAIFMTATPPGATD	NS3	1783	1797	HLA-DRB1*10:01	Dengue virus 4	ELISPOT	IFNg release	Positive
539021	AAAIFMTATPPGSID	NS3	1783	1797	HLA-DRB1*04:07	Dengue virus 4	ELISPOT	IFNg release	Positive
539023	AAAIFMTATPPGTID	NS3	1783	1797	HLA-DRB1*04:03	Dengue virus 4	ELISPOT	IFNg release	Positive
539024	AAAIFMTATPPGTTD	NS3	1783	1797	HLA-DRB1*04:03	Dengue virus 4	ELISPOT	IFNg release	Positive
539026	AAIFMTATPPGATDP	NS3	1784	1798	HLA-DRB1*09:01	Dengue virus 4	ELISPOT	IFNg release	Positive
539080	ALRGLPIRYQTPAVK	NS3	96	110	HLA-DRB1*13:01	Dengue virus 4	ELISPOT	IFNg release	Positive
539359	GVFHTMWHVTRGSVI	NS3	1518	1532	HLA-DRB1*08:03	Dengue virus 4	ELISPOT	IFNg release	Positive
539520	KVIGLYGNGVVTKSG	NS3	7	21	HLA-DRB1*15:06	Dengue virus 4	ELISPOT	IFNg release	Positive
539547	LFKTLTGEIGAVTLD	NS3	1589	1603	HLA-DRB1*10:01	Dengue virus 4	ELISPOT	IFNg release	Positive
539767	PGLFKTLTGEIGAVT	NS3	1587	1601	HLA-DRB1*10:01	Dengue virus 4	ELISPOT	IFNg release	Positive
539803	QGKTVWFVPSIKAGN	NS3	1829	1843	HLA-DRB1*08:03	Dengue virus 4	ELISPOT	IFNg release	Positive
539859	RKSGKKVIQLSRKTF	NS3	1850	1864	HLA-DRB1*08:03	Dengue virus 4	ELISPOT	IFNg release	Positive
539899	SGKKVIQLSRKTFDT	NS3	1852	1866	HLA-DRB1*12:02	Dengue virus 4	ELISPOT	IFNg release	Positive
540077	VREAIKRRLRTLVLA	NS3	1682	1696	HLA-DRB1*08:03	Dengue virus 4	ELISPOT	IFNg release	Positive
68977	VIGLYGNGV	NS3	146	154	HLA-DRB1*15:01	Dengue virus 4	51 chromium	cytotoxicity	Positive
739036	GLPIRYQTPAVKSEH	NS3	1711	1725	HLA-DRB1*14:06	Dengue virus 4	ELISPOT	IFNg release	Positive
739563	LRGLPIRYQTPAVKS	NS3	1709	1723	HLA-DRB1*14:06	Dengue virus 4	ELISPOT	IFNg release	Positive
863140	TRVEMGEAAAIFMTA	NS3	1776	1790	HLA-DQA1*03:01/DQB1*03:02	Dengue virus 4	ELISPOT	IFNg release	Positive
867453	KLTDWDFVVTTDISE	NS3	1872	1886	HLA-DQA1*03:01/DQB1*03:02	Dengue virus 4	ELISPOT	IFNg release	Positive
868371	PRWLDARVYADPMAL	NS3	2067	2081	HLA-DQA1*03:01/DQB1*03:02	Dengue virus 4	ELISPOT	IFNg release	Positive
167758	ILTEIASLPTYLSSRAKL	NS4a	6	23	HLA-DP	Dengue virus 4	ELISPOT	IFNg release	Positive
195176	ASLPTYLSSRAKLAL	NS4a	2103	2117	HLA-DRB1*07:01	Dengue virus 4	ELISPOT	IFNg release	Positive
195224	DNQLIYVILTILTII	NS4a	2223	2237	HLA-DRB1*15:01	Dengue virus 4	ELISPOT	IFNg release	Positive
195274	ETLMLVALLGAMTAG	NS4a	2145	2159	HLA-DRB1*08:02	Dengue virus 4	ELISPOT	IFNg release	Positive
195386	GLITIAVASGLLWVA	NS4a	2176	2190	HLA-DRB1*07:01	Dengue virus 4	ELISPOT	IFNg release	Positive
195551	IYVILTILTIIALVA	NS4a	2227	2241	HLA-DRB1*01:01	Dengue virus 4	ELISPOT	IFNg release	Positive

195575	KLSMGLIAIAVASGL	NS4a	2172	2186	HLA-DRB1*08:02	Dengue virus 4	ELISPOT	IFNg release	Positive
195648	LIYVILTILTIIALV	NS4a	2226	2240	HLA-DRB1*07:01	Dengue virus 4	ELISPOT	IFNg release	Positive
195809	NQLIYVILTILTIIG	NS4a	2224	2238	HLA-DRB1*08:02	Dengue virus 4	ELISPOT	IFNg release	Positive
195853	QLIYVILTILAIIGL	NS4a	2225	2239	HLA-DRB1*15:02	Dengue virus 4	ELISPOT	IFNg release	Positive
195876	RAKLALDNIVMLHTT	NS4a	2112	2126	HLA-DRB1*03:01	Dengue virus 4	ELISPOT	IFNg release	Positive
196058	TLMLVALLGAMTAGI	NS4a	2146	2160	HLA-DRB1*01:01	Dengue virus 4	ELISPOT	IFNg release	Positive
196250	YVILAILTIIGLVAA	NS4a	2228	2242	HLA-DRB1*08:02	Dengue virus 4	ELISPOT	IFNg release	Positive
196251	YVILALLTIISLIAA	NS4a	2228	2242	HLA-DRB1*15:02	Dengue virus 4	ELISPOT	IFNg release	Positive
539165	DILTEIASLPTYLSS	NS4a	2097	2111	HLA-DRB1*04:07	Dengue virus 4	ELISPOT	IFNg release	Positive
539313	GKAYQHALNELPESL	NS4a	2130	2144	HLA-DRB1*10:01	Dengue virus 4	ELISPOT	IFNg release	Positive
539631	LTEIASLPTYLSSRA	NS4a	2099	2113	HLA-DRB1*04:03	Dengue virus 4	ELISPOT	IFNg release	Positive
539638	LVALLGAMTAGIFLF	NS4a	2149	2163	HLA-DRB1*09:01	Dengue virus 4	ELISPOT	IFNg release	Positive
539666	MGLITIAVASGLLWV	NS4a	2175	2189	HLA-DRB1*09:01	Dengue virus 4	ELISPOT	IFNg release	Positive
738568	AYQHALNELPESLET	NS4a	2132	2146	HLA-DRB1*04:07	Dengue virus 4	ELISPOT	IFNg release	Positive
867726	LIYVILTILTIIGLI	NS4a	2226	2240	HLA-DPB1*04:02	Dengue virus 4	ELISPOT	IFNg release	Positive
868951	SLPTYLSSRAKLALD	NS4a	2104	2118	HLA-DRB3*02:02	Dengue virus 4	ELISPOT	IFNg release	Positive
195177	ASLVMLLVHYAIIGP	NS4b	2348	2362	HLA-DRB1*13:01	Dengue virus 4	ELISPOT	IFNg release	Positive
195214	DFGFYQVKTETTILD	NS4b	2254	2268	HLA-DRB1*04:01	Dengue virus 4	ELISPOT	IFNg release	Positive
195219	DLEPISYDPKFEKQL	NS4b	2393	2407	HLA-DRB1*03:01	Dengue virus 4	ELISPOT	IFNg release	Positive
195276	ETTILDVDLRPASAW	NS4b	2263	2277	HLA-DRB1*03:01	Dengue virus 4	ELISPOT	IFNg release	Positive
195383	GLAFSLIKNAQTPRR	NS4b	2473	2487	HLA-DRB1*04:01	Dengue virus 4	ELISPOT	IFNg release	Positive
195592	KTETTILDVDLRPAS	NS4b	2261	2275	HLA-DRB1*03:01	Dengue virus 4	ELISPOT	IFNg release	Positive
195616	LCAGQLLLMRTTWAF	NS4b	2415	2429	HLA-DRB1*04:01	Dengue virus 4	ELISPOT	IFNg release	Positive
195719	LTASLAMLLVHYAII	NS4b	2346	2360	HLA-DRB1*07:01	Dengue virus 4	ELISPOT	IFNg release	Positive
195720	LTASLVMLLVHYAII	NS4b	2346	2360	HLA-DRB1*12:02	Dengue virus 4	ELISPOT	IFNg release	Positive
196048	TILDVDLRPASAWTL	NS4b	2265	2279	HLA-DRB1*03:01	Dengue virus 4	ELISPOT	IFNg release	Positive
196052	TKTDFGFYQVKTETT	NS4b	2251	2265	HLA-DRB1*04:01	Dengue virus 4	ELISPOT	IFNg release	Positive
196055	TLIASLVMLLVHYAI	NS4b	2345	2359	HLA-DRB1*01:01	Dengue virus 4	ELISPOT	IFNg release	Positive
196082	TTLIASLVMLLVHYA	NS4b	2344	2358	HLA-DRB1*13:01	Dengue virus 4	ELISPOT	IFNg release	Positive
196208	WAFCEVLTLATGPIL	NS4b	2427	2441	HLA-DRB1*07:01	Dengue virus 4	ELISPOT	IFNg release	Positive
196209	WAFCEVLTLATGPVL	NS4b	2427	2441	HLA-DRB1*07:01	Dengue virus 4	ELISPOT	IFNg release	Positive
196226	WTLYAVATTILTPML	NS4b	2277	2291	HLA-DRB1*07:01	Dengue virus 4	ELISPOT	IFNg release	Positive
539057	AGQLLLMRTTWAFCE	NS4b	2417	2431	HLA-DRB1*13:01	Dengue virus 4	ELISPOT	IFNg release	Positive
539185	DVDLRPASAWTLYAV	NS4b	2268	2282	HLA-DRB1*09:01	Dengue virus 4	ELISPOT	IFNg release	Positive
539584	LLAMGCYSQVNPTTL	NS4b	2332	2346	HLA-DRB1*09:01	Dengue virus 4	ELISPOT	IFNg release	Positive
539639	LVHYAIIGPGLQAKA	NS4b	2354	2368	HLA-DQA1*05:01/DQB1*02:01	Dengue virus 4	ELISPOT	IFNg release	Positive
539661	MGCYSQVNPTTLIAS	NS4b	2335	2349	HLA-DRB1*09:01	Dengue virus 4	ELISPOT	IFNg release	Positive
738447	AFSLIKNAQTPRRGT	NS4b	2475	2489	HLA-DRB1*14:06	Dengue virus 4	ELISPOT	IFNg release	Positive
738500	AMGCYSQVNPTTLIA	NS4b	2334	2348	HLA-DRB1*04:07	Dengue virus 4	ELISPOT	IFNg release	Positive
738897	FGFYQVKTETTILDV	NS4b	2255	2269	HLA-DRB1*04:07	Dengue virus 4	ELISPOT	IFNg release	Positive
739202	ILDVDLRPASAWTLY	NS4b	2266	2280	HLA-DRB1*01:02	Dengue virus 4	ELISPOT	IFNg release	Positive
739417	LAFSLIKNAQTPRRG	NS4b	2474	2488	HLA-DRB1*14:02	Dengue virus 4	ELISPOT	IFNg release	Positive
867009	GRFWNTTIAVSTANI	NS4b	2449	2463	HLA-DQA1*01:02/DQB1*06:02	Dengue virus 4	ELISPOT	IFNg release	Positive
1066543	ACLGKAYAQMWSLMY	NS5	3240	3254	HLA-DRB1*04:01	Dengue virus 4	ELISPOT	IFNg release	Positive-Low
195230	DYMPVMKRYSAPSES	NS5	3369	3383	HLA-DRB1*11:01	Dengue virus 4	ELISPOT	IFNg release	Positive
195235	EEFISKVRSNAAIGA	NS5	2884	2898	HLA-DRB1*08:02	Dengue virus 4	ELISPOT	IFNg release	Positive
195240	EEYVDYMPVMKRYSA	NS5	3365	3379	HLA-DRB1*11:01	Dengue virus 4	ELISPOT	IFNg release	Positive
195279	EYVDYMPVMKRYSAP	NS5	3366	3380	HLA-DRB1*11:01	Dengue virus 4	ELISPOT	IFNg release	Positive
195411	GNIVSSVNTTSKMLL	NS5	2713	2727	HLA-DRB1*04:01	Dengue virus 4	ELISPOT	IFNg release	Positive
195454	HHFHKIFMKDGRSLV	NS5	3199	3213	HLA-DRB1*03:01	Dengue virus 4	ELISPOT	IFNg release	Positive
195499	ILAKAIFKLTYQNKV	NS5	3050	3064	HLA-DRB1*07:01	Dengue virus 4	ELISPOT	IFNg release	Positive
195593	KTFMKDGRSLVVPCR	NS5	3202	3216	HLA-DRB1*03:01	Dengue virus 4	ELISPOT	IFNg release	Positive
195737	LWCGSLIGLSSRATW	NS5	3333	3347	HLA-DRB1*11:01	Dengue virus 4	ELISPOT	IFNg release	Positive
195764	MPVMKRYSAPSESEG	NS5	3371	3385	HLA-DRB1*08:02	Dengue virus 4	ELISPOT	IFNg release	Positive
195815	NRVWIEDNPNMTDKT	NS5	3302	3316	HLA-DRB1*03:01	Dengue virus 4	ELISPOT	IFNg release	Positive
195823	NWLWALLGKKKNPRL	NS5	2866	2880	HLA-DRB1*14:02	Dengue virus 4	ELISPOT	IFNg release	Positive
195835	PKPGTRMVMTTTANW	NS5	2853	2867	HLA-DRB1*04:01	Dengue virus 4	ELISPOT	IFNg release	Positive
195858	QMWSLMYFHRRDLRL	NS5	3248	3262	HLA-DRB1*15:01	Dengue virus 4	ELISPOT	IFNg release	Positive
195879	REEFISKVRSNAAIG	NS5	2883	2897	HLA-DRB1*04:01	Dengue virus 4	ELISPOT	IFNg release	Positive
195919	RVWIEDNPNMIDKTP	NS5	3303	3317	HLA-DRB1*03:01	Dengue virus 4	ELISPOT	IFNg release	Positive
196071	TRMVMTTTANWLWAL	NS5	2857	2871	HLA-DRB1*04:01	Dengue virus 4	ELISPOT	IFNg release	Positive
196092	TYQNKVVKVLRPTPR	NS5	3059	3073	HLA-DRB1*08:02	Dengue virus 4	ELISPOT	IFNg release	Positive
196105	VDYMPVMKRYSAPFE	NS5	3368	3382	HLA-DRB1*11:01	Dengue virus 4	ELISPOT	IFNg release	Positive
196106	VDYMPVMKRYSAPSE	NS5	3368	3382	HLA-DRB1*11:01	Dengue virus 4	ELISPOT	IFNg release	Positive
196230	YAQMWSLMYFHRRDL	NS5	3246	3260	HLA-DRB1*15:01	Dengue virus 4	ELISPOT	IFNg release	Positive
539149	DCVVKPLDERFSTSLLF	NS5	3152	3168	HLA class II	Dengue virus 4	ICS	IFNg release	Positive
539222	EKPDMTIIGRRLQRL	NS5	2757	2771	HLA-DRB1*12:02	Dengue virus 4	ELISPOT	IFNg release	Positive
539251	FEEYKRSGILEVDRT	NS5	2512	2526	HLA-DRB1*09:01	Dengue virus 4	ELISPOT	IFNg release	Positive
539354	GTRMVMTTTANWLWA	NS5	2856	2870	HLA-DRB1*04:03	Dengue virus 4	ELISPOT	IFNg release	Positive
539456	KAIFKLTYQNKVVKV	NS5	3053	3067	HLA-DRB1*09:01	Dengue virus 4	ELISPOT	IFNg release	Positive
539493	KMLLNRFTTRHRKPT	NS5	2724	2738	HLA-DRB1*13:01	Dengue virus 4	ELISPOT	IFNg release	Positive
539498	KPDMTIIGRRLQRLQ	NS5	2758	2772	HLA-DRB1*08:03	Dengue virus 4	ELISPOT	IFNg release	Positive
539553	LGEFGRAKGSRAIWY	NS5	2949	2963	HLA-DRB1*09:01	Dengue virus 4	ELISPOT	IFNg release	Positive
539656	MAISGDDCVVKPLDERF	NS5	3146	3162	HLA class II	Dengue virus 4	ICS	IFNg release	Positive
539703	MVTQLAMTDTTPFGQQR	NS5	2824	2840	HLA class II	Dengue virus 4	ICS	IFNg release	Positive
539769	PGTRMVMTTTANWLW	NS5	2855	2869	HLA-DRB1*04:03	Dengue virus 4	ELISPOT	IFNg release	Positive
539770	PHHKILAKAIFKLTY	NS5	3046	3060	HLA-DRB1*12:02	Dengue virus 4	ELISPOT	IFNg release	Positive
540007	TTSKMLLNRFTTRHR	NS5	2721	2735	HLA-DRB1*13:01	Dengue virus 4	ELISPOT	IFNg release	Positive
540041	VGTYGLNTFTNMEVQ	NS5	3091	3105	HLA-DRB1*15:06	Dengue virus 4	ELISPOT	IFNg release	Positive
739405	KVRSNAAIGAVFQEE	NS5	2889	2903	HLA-DRB1*01:02	Dengue virus 4	ELISPOT	IFNg release	Positive
740013	QLIRQMEAEGVITQD	NS5	3105	3119	HLA-DRB1*01:02	Dengue virus 4	ELISPOT	IFNg release	Positive
866364	DERFSTSLLFLNDMG	NS5	3159	3173	HLA-DPB1*01:01	Dengue virus 4	ELISPOT	IFNg release	Positive
866633	ESCVYNMMGKREKKL	NS5	2935	2949	HLA-DRB5*01:01	Dengue virus 4	ELISPOT	IFNg release	Positive
867070	HKIFMKDGRSLVVPC	NS5	3202	3216	HLA-DRB3*02:02	Dengue virus 4	ELISPOT	IFNg release	Positive
867315	ISKVRSNAAIGAVFQ	NS5	2887	2901	HLA-DRB3*02:02	Dengue virus 4	ELISPOT	IFNg release	Positive
867411	KILAKAIFKLTYQNK	NS5	3049	3063	HLA-DPB1*04:02	Dengue virus 4	ELISPOT	IFNg release	Positive
867473	KPLDERFGTSLLFLN	NS5	3156	3170	HLA-DPB1*01:01	Dengue virus 4	ELISPOT	IFNg release	Positive
867575	LDERFGTSLLFLNDM	NS5	3158	3172	HLA-DPB1*04:01	Dengue virus 4	ELISPOT	IFNg release	Positive

868638	RDLRLASMAICSAVP	NS5	3258	3272	HLA-DQA1*01:02/DQB1*06:02	Dengue virus 4	ELISPOT	IFNg release	Positive

CD8 DENV-1 Epitopes

Epitope_ID	AminoAcid_Seq	Protein_Name	Start	End	Allele	Serotype	Method/Technique	Assay Group	Qualitative Measure
150255	GPMKLVMAFI	C	43	52	HLA-B*07:02	Dengue virus 1	ELISPOT	IFNg release	Positive
180637	MAFIAFLRF	C	48	56	HLA-B*35:01	Dengue virus 1	ELISPOT	IFNg release	Positive
183175	FIAFLRFLA	C	50	58	HLA-A*68:02	Dengue virus 1	ELISPOT	IFNg release	Positive
183220	FNMLKRARNR	C	14	23	HLA-A*33:01	Dengue virus 1	ELISPOT	IFNg release	Positive
183800	IMNRRKRSV	C	94	102	HLA-B*08:01	Dengue virus 1	ELISPOT	IFNg release	Positive
184003	KEISSMLNIM	C	86	95	HLA-B*40:01	Dengue virus 1	ELISPOT	IFNg release	Positive
184458	LLMLLPTAL	C	83	91	HLA-B*08:01	Dengue virus 1	ELISPOT	IFNg release	Positive
184835	MKLVMAFIAF	C	44	53	HLA-B*35:01	Dengue virus 1	ELISPOT	IFNg release	Positive
184844	MLKRERNRV	C	15	23	HLA-B*08:01	Dengue virus 1	ELISPOT	IFNg release	Positive
184880	MNRRKRSVT	C	95	103	HLA-B*08:01	Dengue virus 1	ELISPOT	IFNg release	Positive
185102	NRRKRSVTM	C	96	104	HLA-B*08:01	Dengue virus 1	ELISPOT	IFNg release	Positive
185492	RRKRSVTMLL	C	97	106	HLA-B*08:01	Dengue virus 1	ELISPOT	IFNg release	Positive
240082	MVLALITFL	C	24	32	HLA-A*02:01	Dengue virus 1	ELISPOT	IFNg release	Positive
150378	LLMLVTPSM	PreM	271	279	HLA-B*08:01	Dengue virus 1	ELISPOT	IFNg release	Positive
183596	HPGFTVIALF	PreM	244	253	HLA-B*35:01	Dengue virus 1	ELISPOT	IFNg release	Positive
16554	FLDLPLPWT	E	213	221	HLA-A2	Dengue virus 1	ICS	IFNg release	Positive
183421	GLNSRSTSL	E	747	755	HLA-B*08:01	Dengue virus 1	ELISPOT	IFNg release	Positive
183654	IAVGMVTLY	E	760	768	HLA-B*35:01	Dengue virus 1	ELISPOT	IFNg release	Positive
184377	LGLNSRSTSL	E	746	755	HLA-B*08:01	Dengue virus 1	ELISPOT	IFNg release	Positive
184626	LTDYGALTL	E	456	464	HLA-A*01:01	Dengue virus 1	ELISPOT	IFNg release	Positive
185212	PTSEIQLTDY	E	449	458	HLA-A*01:01	Dengue virus 1	ELISPOT	IFNg release	Positive
185893	TEVTNPAVL	E	329	337	HLA-B*40:01	Dengue virus 1	ELISPOT	IFNg release	Positive
186524	WTMKIGIGIL	E	733	742	HLA-B*08:01	Dengue virus 1	ELISPOT	IFNg release	Positive
186551	YENLKYSVIV	E	412	421	HLA-B*40:01	Dengue virus 1	ELISPOT	IFNg release	Positive
419306	PTLDIELLK	E	319	327	HLA-A*11:01	Dengue virus 1	ELISPOT	IFNg release	Positive
50596	QEGAMHTAL	E	536	544	HLA-A*02:01	Dengue virus 1	ELISPOT	IFNg release	Positive
588005	RDFVEGLSGATWVDVVL	E	289	305	HLA class I	Dengue virus 1	ICS	IFNg release	Positive
590597	VEGLSGATWVDVVLEHG	E	292	308	HLA class I	Dengue virus 1	ICS	IFNg release	Positive-Intermediate
150258	GPWHLGKLEL	NS1	1041	1050	HLA-B*07:02	Dengue virus 1	ELISPOT	IFNg release	Positive
180836	YGGPISQHNY	NS1	1022	1031	HLA-B*35:01	Dengue virus 1	ELISPOT	IFNg release	Positive
183619	HTWTEQYKF	NS1	801	809	HLA-B57	Dengue virus 1	51 chromium	cytotoxicity	Positive
60104	SPKRLSAAI	NS1	813	821	HLA-B*08:01	Dengue virus 1	ELISPOT	IFNg release	Positive
180641	MALSIVSLF	NS2a	1297	1305	HLA-B*51:01	Dengue virus 1	ELISPOT	IFNg release	Positive
183245	FQSHQLWATL	NS2a	1267	1276	HLA-B*15:01	Dengue virus 1	ELISPOT	IFNg release	Positive
183246	FRRLTSREVL	NS2a	1220	1229	HLA-B*08:01	Dengue virus 1	ELISPOT	IFNg release	Positive
183252	FSLHYAWKTM	NS2a	1286	1295	HLA-B*35:01	Dengue virus 1	ELISPOT	IFNg release	Positive
184274	LALMATFKM	NS2a	1202	1210	HLA-B*35:01	Dengue virus 1	ELISPOT	IFNg release	Positive
184623	LTDFQSHQL	NS2a	1264	1272	HLA-A*01:01	Dengue virus 1	ELISPOT	IFNg release	Positive
184736	MAMVLSIVS	NS2a	1295	1303	HLA-B*35:01	Dengue virus 1	ELISPOT	IFNg release	Positive
184856	MLMTGTLAVF	NS2a	1158	1167	HLA-B*35:01	Dengue virus 1	ELISPOT	IFNg release	Positive
185468	RPMFAVGLLF	NS2a	1211	1220	HLA-B*35:01	Dengue virus 1	ELISPOT	IFNg release	Positive
419037	RPMFAVGILF	NS2a	1211	1220	HLA-B*35:01	Dengue virus 1	ELISPOT	IFNg release	Positive
150254	GPLVAGGLL	NS2b	1375	1383	HLA-B*07:02	Dengue virus 1	ELISPOT	IFNg release	Positive
185651	SILLSSLLK	NS2b	1360	1368	HLA-A*11:01	Dengue virus 1	ELISPOT	IFNg release	Positive
419005	LLAVSGVYPM	NS2b	1448	1457	HLA-B*15:01	Dengue virus 1	ELISPOT	IFNg release	Positive
13346	EMAEALKGMPIRYQT	NS3	231	245	HLA class I	Dengue virus 1	ELISPOT	IFNg release	Positive
133501	AIVREAIKR	NS3	1682	1690	HLA-A*02:01	Dengue virus 1	ELISPOT	IFNg release	Positive
150169	DPRRCLKPV	NS3	1900	1908	HLA-B*07:02	Dengue virus 1	ELISPOT	IFNg release	Positive
150341	KPGTSGSPI	NS3	1606	1614	HLA-B*07:02	Dengue virus 1	ELISPOT	IFNg release	Positive
150538	RPRWLDART	NS3	2068	2076	HLA-B*07:02	Dengue virus 1	ELISPOT	IFNg release	Positive
150556	RVIDPRRCLK	NS3	1897	1906	HLA-A*03:01	Dengue virus 1	ELISPOT	IFNg release	Positive
150643	TPEGIIPALF	NS3	1976	1985	HLA-B*35:01	Dengue virus 1	ELISPOT	IFNg release	Positive
167826	TSGTYVSAIAQAKASQE	NS3	157	173	HLA class I	Dengue virus 1	ELISPOT	IFNg release	Positive
180373	APTRVVASEM	NS3	1698	1707	HLA class I	Dengue virus 1	ELISPOT	IFNg release	Positive
180408	DPASIAARGY	NS3	1766	1775	HLA-B*35:01	Dengue virus 1	ELISPOT	IFNg release	Positive
180423	EERDIPERSW	NS3	1811	1820	HLA-B*44:02	Dengue virus 1	ELISPOT	IFNg release	Positive
180472	GEARKTFVEL	NS3	2003	2012	HLA-B*40:01	Dengue virus 1	ELISPOT	IFNg release	Positive
180670	MPVTVASAAQ	NS3	1923	1932	HLA-B*35:01	Dengue virus 1	ELISPOT	IFNg release	Positive
180682	NPEIEDDIF	NS3	1650	1658	HLA-B*35:01	Dengue virus 1	ELISPOT	IFNg release	Positive
180804	VPNYNMIIM	NS3	1751	1759	HLA-B*51:01	Dengue virus 1	ELISPOT	IFNg release	Positive
182519	AIKRKLRTL	NS3	1687	1695	HLA-B*08:01	Dengue virus 1	ELISPOT	IFNg release	Positive
182772	DISEMGANF	NS3	1885	1893	HLA-A*26:01	Dengue virus 1	ELISPOT	IFNg release	Positive
183223	FPGKTVWFVP	NS3	1830	1839	HLA-B*35:01	Dengue virus 1	ELISPOT	IFNg release	Positive
183229	FPQSNAVIQD	NS3	1801	1810	HLA-B*35:01	Dengue virus 1	ELISPOT	IFNg release	Positive
183598	HPGSGKTRRY	NS3	1670	1679	HLA-B*35:01	Dengue virus 1	ELISPOT	IFNg release	Positive
183929	IYRILQRGLL	NS3	1497	1506	HLA-B*08:01	Dengue virus 1	ELISPOT	IFNg release	Positive
184316	LEENMDVEIW	NS3	2049	2058	HLA-B*44:03	Dengue virus 1	ELISPOT	IFNg release	Positive
184884	MPIRYQTTAV	NS3	1714	1723	HLA-B*35:01	Dengue virus 1	ELISPOT	IFNg release	Positive
184896	MPVTVASAA	NS3	1923	1931	HLA-B*35:01	Dengue virus 1	ELISPOT	IFNg release	Positive
185124	NTPEGIIPA	NS3	1975	1983	HLA-A*68:02	Dengue virus 1	ELISPOT	IFNg release	Positive
185125	NTPEGIIPAL	NS3	1975	1984	HLA-A*68:02	Dengue virus 1	ELISPOT	IFNg release	Positive
185327	QYIYMGQPL	NS3	1947	1955	HLA-A*24:02	Dengue virus 1	ELISPOT	IFNg release	Positive
185741	SPVRVPNYNM	NS3	1747	1756	HLA-B*35:01	Dengue virus 1	ELISPOT	IFNg release	Positive
185827	SYKVASEGF	NS3	2023	2031	HLA-A*24:02	Dengue virus 1	ELISPOT	IFNg release	Positive
186284	VLDDGIYRI	NS3	1492	1500	HLA-A*02:01	Dengue virus 1	ELISPOT	IFNg release	Positive
186559	YKVASEGFQY	NS3	2024	2033	HLA-B*35:01	Dengue virus 1	ELISPOT	IFNg release	Positive
186603	YSDPLALREF	NS3	2077	2086	HLA-B*35:01	Dengue virus 1	ELISPOT	IFNg release	Positive
22825	GTSGSPIVNR	NS3	133	142	HLA-B*57:01	Dengue virus 1	51 chromium	cytotoxicity	Positive
32147	KLRTLVLAPTRVVAS	NS3	216	230	HLA class I	Dengue virus 1	ELISPOT	IFNg release	Positive

35237	LDNINTPEGIIPALFEPERE	NS3	496	515	HLA class I	Dengue virus 1	ELISPOT	IFNg release	Positive
38400	LPAIVREAI	NS3	1680	1688	HLA-B*53:01	Dengue virus 1	ELISPOT	IFNg release	Positive
38766	LPVWLSYKV	NS3	2018	2026	HLA-B*51:01	Dengue virus 1	ELISPOT	IFNg release	Positive
39095	LRGEARKTFVELMRR	NS3	526	540	HLA class I	Dengue virus 1	ELISPOT	IFNg release	Positive
419024	NMIIMDEAHF	NS3	1755	1764	HLA-B*15:01	Dengue virus 1	ELISPOT	IFNg release	Positive
419124	AVEPGKNPK	NS3	1574	1582	HLA-A*11:01	Dengue virus 1	ELISPOT	IFNg release	Positive
419125	AVKSEHTGR	NS3	1722	1730	HLA-A*11:01	Dengue virus 1	ELISPOT	IFNg release	Positive
419229	IYRILQRGL	NS3	1497	1505	HLA-A*24:02	Dengue virus 1	ELISPOT	IFNg release	Positive
50894	QGKRLEPSWASVKKDLISYG	NS3	1536	1555	HLA class I	Dengue virus 1	ELISPOT	IFNg release	Positive
539170	DLMCHATF	NS3	1734	1741	HLA-B*08:01	Dengue virus 1	ICS	IFNg release	Positive
539663	MGEAAAIFMTATPPGSV	NS3	1782	1798	HLA class I	Dengue virus 1	ICS	IFNg release	Positive
539750	NVRTLILAPTRVVASEM	NS3	1690	1706	HLA class I	Dengue virus 1	ICS	IFNg release	Positive
539753	NYNMIIMDEAHFTDPA	NS3	1753	1768	HLA class I	Dengue virus 1	ICS	IFNg release	Positive
56309	RVIDPRRCL	NS3	1897	1905	HLA-B*07:02	Dengue virus 1	ELISPOT	IFNg release	Positive
585710	LAPTRVVASEM	NS3	83	93	HLA class I	Dengue virus 1	ICS	IFNg release	Positive-High
62184	SVKKDLISYGGGWRF	NS3	71	85	HLA class I	Dengue virus 1	ELISPOT	IFNg release	Positive
65546	TPEGIIPAL	NS3	1976	1984	HLA class I	Dengue virus 1	51 chromium	cytotoxicity	Positive
65549	TPEGIIPSM	NS3	1975	1983	HLA-B*53:01	Dengue virus 1	51 chromium	cytotoxicity	Positive
150649	TPQDNQLAYV	NS4a	2222	2231	HLA-B*07:02	Dengue virus 1	ELISPOT	IFNg release	Positive
180780	TPQDNQLAY	NS4a	2222	2230	HLA-B*35:01	Dengue virus 1	ELISPOT	IFNg release	Positive
182912	EFFLMVLLI	NS4a	2206	2214	HLA-A*24:02	Dengue virus 1	ELISPOT	IFNg release	Positive
183162	FFLMVLLIPE	NS4a	2207	2216	HLA-A*33:01	Dengue virus 1	ELISPOT	IFNg release	Positive
183813	IPEPDRQRTP	NS4a	2214	2223	HLA-B*35:01	Dengue virus 1	ELISPOT	IFNg release	Positive
184522	LPDTIETLM	NS4a	2142	2150	HLA-B*35:01	Dengue virus 1	ELISPOT	IFNg release	Positive
184523	LPDTIETLML	NS4a	2142	2151	HLA-B*35:01	Dengue virus 1	ELISPOT	IFNg release	Positive
184554	LPQHLTQRAQ	NS4a	2107	2116	HLA-B*35:01	Dengue virus 1	ELISPOT	IFNg release	Positive
184739	MASSVLLWM	NS4a	2183	2191	HLA-B*35:01	Dengue virus 1	ELISPOT	IFNg release	Positive
419128	AYRHAMEEL	NS4a	2134	2142	HLA-A*24:02	Dengue virus 1	ELISPOT	IFNg release	Positive-High
41986	MLLALIAVL	NS4a	2150	2158	HLA-A2	Dengue virus 1	ICS	IFNg release	Positive
133716	TLYAVATTI	NS4b	2284	2292	HLA-A*02:01	Dengue virus 1	ELISPOT	IFNg release	Positive
150474	NPLTLTAAV	NS4b	2348	2356	HLA-B*07:02	Dengue virus 1	ELISPOT	IFNg release	Positive
150579	SPGKFWNTTI	NS4b	2453	2462	HLA-B*07:02	Dengue virus 1	ELISPOT	IFNg release	Positive
180382	ATGPLTTLW	NS4b	2442	2450	HLA-B*58:01	Dengue virus 1	ELISPOT	IFNg release	Positive
183594	HPASAWTLYA	NS4b	2278	2287	HLA-B*35:01	Dengue virus 1	ELISPOT	IFNg release	Positive
186427	VVYDAKFEK	NS4b	2403	2411	HLA-A*11:01	Dengue virus 1	ELISPOT	IFNg release	Positive
27180	ILLMRTTWA	NS4b	2426	2434	HLA-A2	Dengue virus 1	ICS	IFNg release	Positive
41093	MANIFRGSY	NS4b	2466	2474	HLA-B*35:01	Dengue virus 1	ELISPOT	IFNg release	Positive
64286	TIENTTANISLTAIA	NS4b	2300	2314	HLA class I	Dengue virus 1	ELISPOT	IFNg release	Positive
69652	VLMLVAHYA	NS4b	2356	2364	HLA-A2	Dengue virus 1	ICS	IFNg release	Positive
74558	YLAGAGLAF	NS4b	2474	2482	HLA-B*07:02	Dengue virus 1	ELISPOT	IFNg release	Positive
1068332	SSWEDVPYL	NS5	3323	3331	HLA-A*02:01	Dengue virus 1	ELISPOT	IFNg release	Positive
133702	SRNSTHEMY	NS5	2703	2711	HLA-A*02:01	Dengue virus 1	ELISPOT	IFNg release	Positive
14325	ETACLGKSY	NS5	3242	3250	HLA-A*26:01	Dengue virus 1	ELISPOT	IFNg release	Positive
150308	IPMATYGWNL	NS5	2607	2616	HLA-B*07:02	Dengue virus 1	ELISPOT	IFNg release	Positive
150674	TVMDIISRR	NS5	3080	3088	HLA-A*11:01	Dengue virus 1	multimer/tetramer	qualitative binding	Positive
180376	ASSMVNGVVR	NS5	2809	2818	HLA-B*57:01	Dengue virus 1	ELISPOT	IFNg release	Positive
180411	DTTPFGQQR	NS5	2837	2845	HLA-A*68:01	Dengue virus 1	ELISPOT	IFNg release	Positive
180548	KAKGSRAIW	NS5	2959	2967	HLA-B*57:01	Dengue virus 1	ELISPOT	IFNg release	Positive
180571	KPRICTREEF	NS5	2882	2891	HLA-B*07:02	Dengue virus 1	ELISPOT	IFNg release	Positive
180723	RFLEFEALGF	NS5	2974	2983	HLA-A*23:01	Dengue virus 1	ELISPOT	IFNg release	Positive
182554	ALLATSIFK	NS5	3054	3062	HLA-A*11:01	Dengue virus 1	ELISPOT	IFNg release	Positive
182599	AQMWQLMYF	NS5	3251	3259	HLA-B*15:01	Dengue virus 1	ELISPOT	IFNg release	Positive
182836	DYMTSMKRF	NS5	3373	3381	HLA-A*24:02	Dengue virus 1	ELISPOT	IFNg release	Positive
183147	FCSHHFHQL	NS5	3200	3208	HLA-B*08:01	Dengue virus 1	ELISPOT	IFNg release	Positive
183275	FTNMEVQLIR	NS5	3104	3113	HLA-B*35:01	Dengue virus 1	ELISPOT	IFNg release	Positive
183828	IPMVTQIAM	NS5	2827	2835	HLA-B*07:02	Dengue virus 1	ELISPOT	IFNg release	Positive
184212	KVRKDIPQW	NS5	3178	3186	HLA-B*57:01	Dengue virus 1	ELISPOT	IFNg release	Positive
184787	MEVQLVRQM	NS5	3107	3115	HLA-B*40:01	Dengue virus 1	ELISPOT	IFNg release	Positive
184849	MLLNRFTMA	NS5	24	32	HLA-B*08:01	Dengue virus 1	ELISPOT	IFNg release	Positive
184898	MQRKHGGML	NS5	2689	2697	HLA-B*08:01	Dengue virus 1	ELISPOT	IFNg release	Positive
184956	MTTEDMLSVW	NS5	3296	3305	HLA-B*58:01	Dengue virus 1	ICS	IFNg release	Positive
185279	QMQRKHGGML	NS5	2688	2697	HLA-B*08:01	Dengue virus 1	ELISPOT	IFNg release	Positive
185354	REIVVPCRNQ	NS5	3214	3223	HLA-B*40:01	Dengue virus 1	ELISPOT	IFNg release	Positive
185485	RQMESEGIFL	NS5	3113	3122	HLA-B*15:01	Dengue virus 1	ELISPOT	IFNg release	Positive
186150	TVMDVISRR	NS5	3080	3088	HLA-A*68:01	Dengue virus 1	ELISPOT	IFNg release	Positive
186176	TWSIHAHHQW	NS5	3286	3295	HLA-A*23:01	Dengue virus 1	ELISPOT	IFNg release	Positive
186217	VEDERFWDL	NS5	2918	2926	HLA-B*40:01	Dengue virus 1	ELISPOT	IFNg release	Positive
419208	GVEGEGLHK	NS5	2998	3006	HLA-A*11:01	Dengue virus 1	ELISPOT	IFNg release	Positive-High
5223	ATYGWNLVK	NS5	2610	2618	HLA-A*03:01	Dengue virus 1	ELISPOT	IFNg release	Positive
539435	ISGDDCVVKPIDDRFAT	NS5	3152	3168	HLA class I	Dengue virus 1	ICS	IFNg release	Positive
539702	MVTQIAMTDTTPFGQQR	NS5	2829	2845	HLA class I	Dengue virus 1	ICS	IFNg release	Positive
540100	VVKPIDDRFATALTALN	NS5	3157	3173	HLA class I	Dengue virus 1	ICS	IFNg release	Positive
62903	TAKWLWGFLSRNKKPRICTR	NS5	2869	2888	HLA class I	Dengue virus 1	ELISPOT	IFNg release	Positive
65567	TPFGQQRVF	NS5	2839	2847	HLA class I	Dengue virus 1	ELISPOT	IFNg release	Positive
67362	TYGWNLVKL	NS5	2611	2619	HLA-A*24:02	Dengue virus 1	ELISPOT	IFNg release	Positive
72563	WHYDEDNPYKTWAYHGSYEV	NS5	2784	2803	HLA class I	Dengue virus 1	ELISPOT	IFNg release	Positive
73076	WSIHAHHQW	NS5	3287	3295	HLA-B*57:01	Dengue virus 1	ELISPOT	IFNg release	Positive

CD8 DENV-2 Epitopes

Epitope_ID	AminoAcid_Seq	Protein_Name	Start	End	Allele	Serotype	Method/Technique	Assay Group	Qualitative Measure
110018	VAFLRFLTI	C	51	59	HLA-B*08:01	Dengue virus 2	ELISPOT	IFNg release	Positive
150252	GPLKLFMAL	C	42	50	HLA-B*08:01	Dengue virus 2	ELISPOT	IFNg release	Positive
183766	ILNRRRRTA	C	94	102	HLA-B*08:01	Dengue virus 2	ELISPOT	IFNg release	Positive
184378	LGMLQGRGPL	C	35	44	HLA-B*08:01	Dengue virus 2	ELISPOT	IFNg release	Positive
184518	LNRRRRTAG	C	95	103	HLA-B*08:01	Dengue virus 2	ELISPOT	IFNg release	Positive
184643	LTIPPTAGIL	C	21	30	HLA-B*08:01	Dengue virus 2	ELISPOT	IFNg release	Positive
185104	NRRRRTAGV	C	96	104	HLA-B*08:01	Dengue virus 2	ELISPOT	IFNg release	Positive
185399	RKKARSTPF	C	5	13	HLA-B*08:01	Dengue virus 2	ELISPOT	IFNg release	Positive
186354	VQQLTKRFSL	C	26	35	HLA-B*08:01	Dengue virus 2	ELISPOT	IFNg release	Positive
150224	FTIMAAILAY	PreM	247	256	HLA-B*35:01	Dengue virus 2	ELISPOT	IFNg release	Positive
150299	ILLTAVAPSM	PreM	270	279	HLA-B*08:01	Dengue virus 2	ELISPOT	IFNg release	Positive
150571	SLLFKTEDGV	PreM	136	145	HLA-A*02:01	Dengue virus 2	ELISPOT	IFNg release	Positive
150634	TLMAMDLGEL	PreM	149	158	HLA-A*02:01	Dengue virus 2	ELISPOT	IFNg release	Positive
180588	LAYTIGTTHF	PreM	254	263	HLA-B*35:01	Dengue virus 2	ELISPOT	IFNg release	Positive
180818	WILRHPGFTMMAAIL	PreM	126	140	HLA class I	Dengue virus 2	ICS	IFNg release	Positive
183577	HFQRALIFIL	PreM	262	271	HLA-B*08:01	Dengue virus 2	ELISPOT	IFNg release	Positive
183621	HVQRIETWIL	PreM	233	242	HLA-B*08:01	Dengue virus 2	ELISPOT	IFNg release	Positive
186264	VGRQEKGKSL	PreM	128	137	HLA-B*08:01	Dengue virus 2	ELISPOT	IFNg release	Positive
150115	AIYGAAFSGV	E	722	731	HLA-A*02:01	Dengue virus 2	ELISPOT	IFNg release	Positive
150319	ITEAELTGY	E	450	458	HLA-A*01:01	Dengue virus 2	ELISPOT	IFNg release	Positive
150399	LQMENKAWLV	E	231	240	HLA-B*08:01	Dengue virus 2	ELISPOT	IFNg release	Positive
150456	MSYSMCTGKF	E	577	586	HLA-B*35:01	Dengue virus 2	ELISPOT	IFNg release	Positive
150529	RLITVNPIV	E	630	638	HLA-A*02:01	Dengue virus 2	ELISPOT	IFNg release	Positive
150531	RLRMDKLQL	E	566	574	HLA-B*08:01	Dengue virus 2	ELISPOT	IFNg release	Positive
150578	SPCKIPFEIM	E	611	620	HLA-B*35:01	Dengue virus 2	ELISPOT	IFNg release	Positive
150725	WLVHRQWFL	E	486	494	HLA-B*08:01	Dengue virus 2	ELISPOT	IFNg release	Positive
16553	FLDLPLPWL	E	213	221	HLA-A2	Dengue virus 2	ICS	TNF release	Positive
180433	EPGQLKLNWF	E	663	672	HLA-B*35:01	Dengue virus 2	ELISPOT	IFNg release	Positive
180453	FGAIYGAAF	E	720	728	HLA-B*35:01	Dengue virus 2	ELISPOT	IFNg release	Positive
180528	IPFEIMDLEK	E	615	624	HLA-B*35:01	Dengue virus 2	ELISPOT	IFNg release	Positive
180563	KILIGVIITWIGMNS	E	456	470	HLA class I	Dengue virus 2	ICS	IFNg release	Positive
180618	LPLPWLPGAD	E	496	505	HLA-B*35:01	Dengue virus 2	ELISPOT	IFNg release	Positive
180726	RMAILGDTAWDFGSL	E	691	705	HLA-B7	Dengue virus 2	ICS	IFNg release	Positive
180816	WGNGCGLFGKGGIVT	E	381	395	HLA class I	Dengue virus 2	ICS	IFNg release	Positive
182846	EAKQPATLR	E	329	337	HLA-A*68:01	Dengue virus 2	ELISPOT	IFNg release	Positive
183310	GAAFSGVSW	E	725	733	HLA-B*58:01	Dengue virus 2	ELISPOT	IFNg release	Positive
183437	GMNSRSTSL	E	747	755	HLA-B*08:01	Dengue virus 2	ELISPOT	IFNg release	Positive
183708	IGMNSRSTSL	E	746	755	HLA-B*08:01	Dengue virus 2	ELISPOT	IFNg release	Positive
183747	ILIGVIITW	E	737	745	HLA-B*58:01	Dengue virus 2	ELISPOT	IFNg release	Positive
183748	ILIGVVITW	E	737	745	HLA-B*58:01	Dengue virus 2	ELISPOT	IFNg release	Positive
183781	IMDLEKRHVL	E	619	628	HLA-B*08:01	Dengue virus 2	ELISPOT	IFNg release	Positive
183840	IQKETLVTF	E	512	520	HLA-B*15:01	Dengue virus 2	ELISPOT	IFNg release	Positive
183841	IQMSSGNLLF	E	550	559	HLA-A*23:01	Dengue virus 2	ELISPOT	IFNg release	Positive
184300	LATLRKYCI	E	53	61	HLA-B*08:01	Dengue virus 2	ELISPOT	IFNg release	Positive
184337	LEKRHVLGRL	E	622	631	HLA-B*08:01	Dengue virus 2	ELISPOT	IFNg release	Positive
184467	LLQMEDKAWL	E	478	487	HLA-B*08:01	Dengue virus 2	ELISPOT	IFNg release	Positive
184571	LQLKGMSYSM	E	572	581	HLA-B*08:01	Dengue virus 2	ELISPOT	IFNg release	Positive
184755	MDLEKRHVL	E	620	628	HLA-B*08:01	Dengue virus 2	ELISPOT	IFNg release	Positive
184899	MRGAKRMAI	E	686	694	HLA-B*08:01	Dengue virus 2	ELISPOT	IFNg release	Positive
184900	MRGAKRMAIL	E	686	695	HLA-B*08:01	Dengue virus 2	ELISPOT	IFNg release	Positive
184920	MSSGNLLFTG	E	552	561	HLA-B*58:01	Dengue virus 2	ELISPOT	IFNg release	Positive
185758	SRSTSLSVSL	E	750	759	HLA-B*08:01	Dengue virus 2	ELISPOT	IFNg release	Positive
185831	SYSMCTGKF	E	578	586	HLA-A*23:01	Dengue virus 2	ELISPOT	IFNg release	Positive
27061	ILGDTAWDFG	E	96	105	HLA class I	Dengue virus 2	ELISPOT	IFNg release	Positive
41407	MENKAWLVHRQWFLD	E	201	215	HLA-B*37:01	Dengue virus 2	ICS	IFNg release	Positive
51388	QLKGMSYSM	E	573	581	HLA-B*08:01	Dengue virus 2	ELISPOT	IFNg release	Positive
583702	GVSGGSWVDIVLEHGSCV	E	14	31	HLA class I	Dengue virus 2	ICS	IFNg release	Positive-Low
150259	GPWHLGKLEM	NS1	1041	1050	HLA-B*35:01	Dengue virus 2	ELISPOT	IFNg release	Positive
180450	FAGPVSQHNY	NS1	1022	1031	HLA-B*35:01	Dengue virus 2	ELISPOT	IFNg release	Positive
180496	GVFTTNIWLKLKEKQ	NS1	936	950	HLA class I	Dengue virus 2	ICS	IFNg release	Positive
180689	NRAVHADMGYWIESA	NS1	966	980	HLA class I	Dengue virus 2	ICS	IFNg release	Positive
182938	EKEENLVNSL	NS1	1115	1124	HLA-B*08:01	Dengue virus 2	ELISPOT	IFNg release	Positive
184512	LMWKQITPEL	NS1	841	850	HLA-B*08:01	Dengue virus 2	ELISPOT	IFNg release	Positive
185342	RAVHADMGY	NS1	192	200	HLA-B*58:01	Dengue virus 2	ELISPOT	IFNg release	Positive
185465	RPLKEKEENL	NS1	1111	1120	HLA-B*08:01	Dengue virus 2	ELISPOT	IFNg release	Positive
186474	WKTWGKAKML	NS1	890	899	HLA-B*08:01	Dengue virus 2	ELISPOT	IFNg release	Positive
19225	GEDGCWYGM	NS1	1100	1108	HLA-B*40:01	Dengue virus 2	ELISPOT	IFNg release	Positive
150206	FLEEMLRTRV	NS2a	1131	1140	HLA-A*02:01	Dengue virus 2	ELISPOT	IFNg release	Positive
150300	ILLVAVSFV	NS2a	1146	1154	HLA-A*02:01	Dengue virus 2	ELISPOT	IFNg release	Positive
150391	LMMTTIGIVL	NS2a	1228	1237	HLA-B*08:01	Dengue virus 2	ELISPOT	IFNg release	Positive
150415	LTDALALGM	NS2a	1250	1258	HLA-A*01:01	Dengue virus 2	ELISPOT	IFNg release	Positive
150448	MMATIGIAL	NS2a	1229	1237	HLA-B*35:01	Dengue virus 2	ELISPOT	IFNg release	Positive
150449	MMATIGIALL	NS2a	1229	1238	HLA-A*02:01	Dengue virus 2	ELISPOT	IFNg release	Positive
150457	MTDDIGMGV	NS2a	1191	1199	HLA-A*01:01	Dengue virus 2	ELISPOT	IFNg release	Positive
150540	RPTFAAGLLL	NS2a	1211	1220	HLA-B*07:02	Dengue virus 2	ELISPOT	IFNg release	Positive
150640	TMTDDIGMGV	NS2a	1176	1185	HLA-A*02:01	Dengue virus 2	ELISPOT	IFNg release	Positive
150741	YQLAVTIMA	NS2a	1157	1165	HLA-A*02:01	Dengue virus 2	ELISPOT	IFNg release	Positive
180671	MSFRDLGRVM	NS2a	1175	1184	HLA-B*35:01	Dengue virus 2	ELISPOT	IFNg release	Positive

180717	RDLGRVMVMVGATMT	NS2a	51	65	HLA class I	Dengue virus 2	ICS	IFNg release	Positive
180735	RVGTKHAILLVAVSF	NS2a	1153	1167	HLA class I	Dengue virus 2	ICS	IFNg release	Positive
183741	ILCVPNAVIL	NS2a	1279	1288	HLA-B*08:01	Dengue virus 2	ELISPOT	IFNg release	Positive
183825	IPLALTIKGL	NS2a	1319	1328	HLA-B*08:01	Dengue virus 2	ELISPOT	IFNg release	Positive
184864	MLRTRVGTK	NS2a	1149	1157	HLA-B*08:01	Dengue virus 2	ELISPOT	IFNg release	Positive
186041	TRVGTKHAIL	NS2a	1152	1161	HLA-B*08:01	Dengue virus 2	ELISPOT	IFNg release	Positive
150101	AAAWYLWEV	NS2b	1463	1471	HLA-A*02:01	Dengue virus 2	ELISPOT	IFNg release	Positive
150243	GLLTVCYVL	NS2b	1381	1389	HLA-A*02:01	Dengue virus 2	ELISPOT	IFNg release	Positive
150304	IMAVGMVSI	NS2b	1353	1361	HLA-A*02:01	Dengue virus 2	ELISPOT	IFNg release	Positive
150381	LLVISGLFPV	NS2b	1448	1457	HLA-A*02:01	Dengue virus 2	ELISPOT	IFNg release	Positive
150425	LVISGLFPV	NS2b	1449	1457	HLA-A*02:01	Dengue virus 2	ELISPOT	IFNg release	Positive
150584	SPILSITISE	NS2b	1416	1425	HLA-B*35:01	Dengue virus 2	ELISPOT	IFNg release	Positive
150610	TAAAWYLWEV	NS2b	117	126	HLA-A*02:01	Dengue virus 2	ELISPOT	IFNg release	Positive
180529	IPITAAAWY	NS2b	1459	1467	HLA-B*35:01	Dengue virus 2	ELISPOT	IFNg release	Positive
180531	IPMTGPLVAG	NS2b	1371	1380	HLA-B*35:01	Dengue virus 2	ELISPOT	IFNg release	Positive
180538	ISGLFPVSIPITAAA	NS2b	106	120	HLA class I	Dengue virus 2	ICS	IFNg release	Positive
180646	MAVGMVSIL	NS2b	1354	1362	HLA-B*35:01	Dengue virus 2	ELISPOT	IFNg release	Positive
183779	IMAVGMVSIL	NS2b	1353	1362	HLA-B*08:01	Dengue virus 2	ELISPOT	IFNg release	Positive
183819	IPITAAAWYL	NS2b	1459	1468	HLA-B*08:01	Dengue virus 2	ELISPOT	IFNg release	Positive
10643	DVKKDLISY	NS3	71	79	HLA-B62	Dengue virus 2	51 chromium	cytotoxicity	Positive
11063	EAHFTDPASIAARGY	NS3	285	299	HLA class I	Dengue virus 2	ELISPOT	IFNg release	Positive
11125	EALRGLPIR	NS3	233	241	HLA class I	Dengue virus 2	ELISPOT	IFNg release	Positive
124391	KTFVDLMRR	NS3	2006	2014	HLA-A*11:01	Dengue virus 2	ELISPOT	IFNg release	Positive
13358	EMEEALRGLPIRYQT	NS3	230	244	HLA class I	Dengue virus 2	ELISPOT	IFNg release	Positive
136890	TPPGSRDPF	NS3	1792	1800	HLA-B*35:01	Dengue virus 2	ELISPOT	IFNg release	Positive
150137	APTRVVAAEM	NS3	222	231	HLA-B7	Dengue virus 2	multimer/tetramer	qualitative binding	Positive
150167	DLMRRGDLPV	NS3	2010	2019	HLA-A*02:01	Dengue virus 2	ELISPOT	IFNg release	Positive
150217	FPQSNAPIM	NS3	1800	1808	HLA-B*35:01	Dengue virus 2	ELISPOT	IFNg release	Positive
150346	KTFDSEYVK	NS3	1863	1871	HLA-A*11:01	Dengue virus 2	multimer/tetramer	qualitative binding	Positive
150450	MPVTHSSAA	NS3	1922	1930	HLA-B*08:01	Dengue virus 2	ELISPOT	IFNg release	Positive
150451	MPVTHSSAAQ	NS3	1922	1931	HLA-B*35:01	Dengue virus 2	ELISPOT	IFNg release	Positive
150526	RIYSDPLALK	NS3	2074	2083	HLA-A*11:01	Dengue virus 2	ELISPOT	IFNg release	Positive
150595	SQIGAGVYK	NS3	1509	1517	HLA-A*11:01	Dengue virus 2	ELISPOT	IFNg release	Positive
150644	TPEGIIPSMF	NS3	1975	1984	HLA-B*53:01	Dengue virus 2	ELISPOT	IFNg release	Positive
150693	VPNYNLIIM	NS3	1750	1758	HLA-B*07:02	Dengue virus 2	ELISPOT	IFNg release	Positive
150740	YLPAIVREA	NS3	1678	1686	HLA-A*02:01	Dengue virus 2	ELISPOT	IFNg release	Positive
17802	FSPGTSGSPIIDKKG	NS3	130	144	HLA class I	Dengue virus 2	ELISPOT	IFNg release	Positive
180360	AAEGINYADRRWCFD	NS3	2026	2040	HLA-A24	Dengue virus 2	ELISPOT	IFNg release	Positive
180407	DNINTPEGIIPSMFE	NS3	496	510	HLA-B35	Dengue virus 2	ELISPOT	IFNg release	Positive
180428	EGEWKEGEEVQVLAL	NS3	1561	1575	HLA class I	Dengue virus 2	ICS	IFNg release	Positive
180440	EREIPERSWNSGHEW	NS3	336	350	HLA-B*57:01	Dengue virus 2	ELISPOT	IFNg release	Positive
180460	FPQSNAPIMD	NS3	1800	1809	HLA-B*35:01	Dengue virus 2	ELISPOT	IFNg release	Positive
180471	GEARKTFVDL	NS3	2002	2011	HLA-B*40:01	Dengue virus 2	ELISPOT	IFNg release	Positive
180503	HPGAGKTKRY	NS3	1669	1678	HLA-B*35:01	Dengue virus 2	ELISPOT	IFNg release	Positive
180507	HTGREIVDLMCHATF	NS3	1726	1740	HLA class I	Dengue virus 2	ELISPOT	IFNg release	Positive
180575	KRYLPAIVREAIKRG	NS3	201	215	HLA-A*31:01	Dengue virus 2	ELISPOT	IFNg release	Positive
180589	LDARIYSDPLALKEF	NS3	596	610	HLA class I	Dengue virus 2	ELISPOT	IFNg release	Positive
180605	LMCHATFTM	NS3	1734	1742	HLA-B*15:01	Dengue virus 2	ELISPOT	IFNg release	Positive
180611	LMRRGDLPVWLAYRV	NS3	2011	2025	HLA-B*55:02	Dengue virus 2	ICS	IFNg release	Positive
180617	LPIRYQTPAI	NS3	1713	1722	HLA-B*35:01	Dengue virus 2	ELISPOT	IFNg release	Positive
180623	LPVWLAYKV	NS3	2017	2025	HLA-B*51:01	Dengue virus 2	ELISPOT	IFNg release	Positive
180624	LPVWLAYKVA	NS3	2017	2026	HLA-B*35:01	Dengue virus 2	ELISPOT	IFNg release	Positive
180625	LPVWLAYRV	NS3	2017	2025	HLA-B*51:01	Dengue virus 2	ELISPOT	IFNg release	Positive
180626	LPVWLAYRVA	NS3	2017	2026	HLA-B*51:01	Dengue virus 2	ELISPOT	IFNg release	Positive
180647	MEEALRGLPIRYQTP	NS3	1706	1720	HLA class I	Dengue virus 2	ELISPOT	IFNg release	Positive
180724	RGEARKTFVDLMRRG	NS3	2001	2015	HLA class I	Dengue virus 2	ELISPOT	IFNg release	Positive
180734	RSWNSGHEW	NS3	342	350	HLA-B*58:01	Dengue virus 2	ICS	IFNg release	Positive
180750	SPGTSGSPIIDKKGK	NS3	131	145	HLA-A*11:01	Dengue virus 2	ELISPOT	IFNg release	Positive
180756	STRVEMGEAAGIFMT	NS3	301	315	HLA-B*40:06	Dengue virus 2	ELISPOT	IFNg release	Positive-Low
182687	AYRIKQRGIL	NS3	1497	1506	HLA-B*08:01	Dengue virus 2	ELISPOT	IFNg release	Positive
182848	EARKTFVDL	NS3	2003	2011	HLA-B*08:01	Dengue virus 2	ELISPOT	IFNg release	Positive
182892	EENMEVEIW	NS3	2049	2057	HLA-B*44:02	Dengue virus 2	ELISPOT	IFNg release	Positive
182898	EEREIPERSW	NS3	1810	1819	HLA-B*44:03	Dengue virus 2	ELISPOT	IFNg release	Positive
183347	GERKKLKPRW	NS3	2061	2070	HLA-B*44:03	Dengue virus 2	ELISPOT	IFNg release	Positive
183724	IKRGLRTLIL	NS3	1687	1696	HLA-B*08:01	Dengue virus 2	ELISPOT	IFNg release	Positive
184318	LEENMEVEIW	NS3	2048	2057	HLA-B*44:02	Dengue virus 2	ELISPOT	IFNg release	Positive
185200	PRRCMKPVIL	NS3	1900	1909	HLA-B*08:01	Dengue virus 2	ELISPOT	IFNg release	Positive
185388	RIKQKGILGY	NS3	1499	1508	HLA-B*15:01	Dengue virus 2	ELISPOT	IFNg release	Positive
186211	VDRKGKVVGL	NS3	1615	1624	HLA-B*08:01	Dengue virus 2	ELISPOT	IFNg release	Positive
186242	VEMGEAAGIF	NS3	1779	1788	HLA-B*44:03	Dengue virus 2	ELISPOT	IFNg release	Positive
186301	VLMHRGKRI	NS3	1532	1540	HLA-B*08:01	Dengue virus 2	ELISPOT	IFNg release	Positive
22816	GTSGSPIADK	NS3	1608	1617	HLA-A*11:01	Dengue virus 2	multimer/tetramer	qualitative binding	Positive
22817	GTSGSPIADKK	NS3	1608	1618	HLA-A11	Dengue virus 2	ELISPOT	IFNg release	Positive
22818	GTSGSPIIDK	NS3	133	142	HLA-A*11:01	Dengue virus 2	ELISPOT	IFNg release	Positive
22819	GTSGSPIIDKK	NS3	133	143	HLA-A11	Dengue virus 2	51 chromium	cytotoxicity	Positive
22823	GTSGSPIVDK	NS3	133	142	HLA-A11	Dengue virus 2	51 chromium	cytotoxicity	Positive
22824	GTSGSPIVDR	NS3	1608	1617	HLA-A*11:01	Dengue virus 2	51 chromium	cytotoxicity	Positive
25929	IEPSWADVKKDLISY	NS3	65	79	HLA class I	Dengue virus 2	ELISPOT	IFNg release	Positive
26965	ILAPTRVVAAEMEEA	NS3	220	234	HLA class I	Dengue virus 2	ELISPOT	IFNg release	Positive
27770	INYADRRWCF	NS3	555	564	HLA-A24	Dengue virus 2	ELISPOT	IFNg release	Positive
2859	ALRGLPIRY	NS3	234	242	HLA-A*03:01	Dengue virus 2	51 chromium	cytotoxicity	Positive

35238	LDNINTPEGIIPSMF	NS3	495	509	HLA class I	Dengue virus 2	ELISPOT	IFNg release	Positive
46575	NYADRKWCF	NS3	2031	2039	HLA-A24	Dengue virus 2	51 chromium	cytotoxicity	Positive
46576	NYADRRWCF	NS3	556	564	HLA-A24	Dengue virus 2	ELISPOT	IFNg release	Positive
539620	LRTLILAPTRVVAAEM	NS3	1691	1706	HLA class I	Dengue virus 2	ICS	IFNg release	Positive
539751	NYNLIIMDEAHFTDPA	NS3	1752	1767	HLA class I	Dengue virus 2	ICS	IFNg release	Positive
539752	NYNLIIMDEAHFTDPASI	NS3	1752	1769	HLA class I	Dengue virus 2	ICS	IFNg release	Positive
539985	TLILAPTRVVAAEMEEA	NS3	1693	1709	HLA class I	Dengue virus 2	ICS	IFNg release	Positive
54176	RIKQKGIL	NS3	1499	1506	HLA-B8	Dengue virus 2	ELISPOT	IFNg release	Positive
5542	AVSLDFSPGTSGSPI	NS3	125	139	HLA class I	Dengue virus 2	ELISPOT	IFNg release	Positive
56310	RVIDPRRCMK	NS3	1896	1905	HLA-A*31:01	Dengue virus 2	ELISPOT	IFNg release	Positive
57554	SEMGANFKA	NS3	1872	1880	HLA-B*44:03	Dengue virus 2	ELISPOT	IFNg release	Positive
65548	TPEGIIPSL	NS3	1975	1983	HLA-B35	Dengue virus 2	51 chromium	cytotoxicity	Positive
65550	TPEGIIPSMFEPERE	NS3	500	514	HLA class I	Dengue virus 2	ELISPOT	IFNg release	Positive
67210	TVWFVPSIK	NS3	358	366	HLA class I	Dengue virus 2	ELISPOT	IFNg release	Positive
790	ADVKKDLISYGGGWK	NS3	70	84	HLA class I	Dengue virus 2	ELISPOT	IFNg release	Positive
8910	DKKGKVVGL	NS3	1616	1624	HLA-B8	Dengue virus 2	ELISPOT	IFNg release	Positive
150289	IILEFFLIV	NS4a	2202	2210	HLA-A*02:01	Dengue virus 2	ELISPOT	IFNg release	Positive
150461	MTQKARNAL	NS4a	2110	2118	HLA-B*07:02	Dengue virus 2	ELISPOT	IFNg release	Positive
150612	TAEAGGRAY	NS4a	2126	2134	HLA-B*35:01	Dengue virus 2	ELISPOT	IFNg release	Positive
150752	YVVIAILTV	NS4a	2229	2237	HLA-A*02:01	Dengue virus 2	ELISPOT	IFNg release	Positive
150753	YVVIAILTVV	NS4a	2229	2238	HLA-A*02:01	Dengue virus 2	ELISPOT	IFNg release	Positive
180620	LPTFMTQKAR	NS4a	2106	2115	HLA-B*35:01	Dengue virus 2	ELISPOT	IFNg release	Positive
180781	TPQDNQLTY	NS4a	2221	2229	HLA-B*35:01	Dengue virus 2	ELISPOT	IFNg release	Positive
185447	RNALDNLAVL	NS4a	2115	2124	HLA-B*08:01	Dengue virus 2	ELISPOT	IFNg release	Positive
2877	ALSELPETL	NS4a	2137	2145	HLA-A*02:01	Dengue virus 2	ELISPOT	IFNg release	Positive
37557	LLLTLLATV	NS4a	56	64	HLA-A2	Dengue virus 2	ICS	IFNg release	Positive
51562	QLTYVVIAIL	NS4a	2226	2235	HLA-B*08:01	Dengue virus 2	ELISPOT	IFNg release	Positive
150147	ATVLMGLGK	NS4b	2315	2323	HLA-A*11:01	Dengue virus 2	multimer/tetramer	qualitative binding	Positive
150373	LLILCVTQV	NS4b	2416	2424	HLA-A*02:01	Dengue virus 2	ELISPOT	IFNg release	Positive
150377	LLLVAHYAI	NS4b	2355	2363	HLA-A*02:01	Dengue virus 2	ELISPOT	IFNg release	Positive
150387	LMGLGKGWPL	NS4b	2318	2327	HLA-A*02:01	Dengue virus 2	ELISPOT	IFNg release	Positive
150389	LMMRTTWAL	NS4b	2425	2433	HLA-B*08:01	Dengue virus 2	ELISPOT	IFNg release	Positive
150445	MLLILCVTQV	NS4b	2415	2424	HLA-A*02:01	Dengue virus 2	ELISPOT	IFNg release	Positive
150534	RPASAWTLY	NS4b	2276	2284	HLA-B*07:02	Dengue virus 2	ELISPOT	IFNg release	Positive
150639	TLYAVATTFV	NS4b	2282	2291	HLA-A*02:01	Dengue virus 2	ELISPOT	IFNg release	Positive
150679	VIDLDPIPY	NS4b	2395	2403	HLA-A*01:01	Dengue virus 2	ELISPOT	IFNg release	Positive
150686	VLMMRTTWA	NS4b	2424	2432	HLA-B*08:01	Dengue virus 2	ELISPOT	IFNg release	Positive
16757	FLLVAHYAI	NS4b	112	120	HLA-A2	Dengue virus 2	ICS	TNF release	Positive
180380	ATGPISTLW	NS4b	2440	2448	HLA-B*58:01	Dengue virus 2	ELISPOT	IFNg release	Positive
180387	CEALTLATGPISTLW	NS4b	191	205	HLA class I	Dengue virus 2	ELISPOT	IFNg release	Positive
180512	IANQATVLM	NS4b	2311	2319	HLA-B*35:01	Dengue virus 2	ELISPOT	IFNg release	Positive
180514	IAVSMANIF	NS4b	2460	2468	HLA-B*35:01	Dengue virus 2	ELISPOT	IFNg release	Positive
180601	LILCVTQVLM	NS4b	2417	2426	HLA-B*35:01	Dengue virus 2	ELISPOT	IFNg release	Positive
180789	TVIDLDPIPYDPKFE	NS4b	2394	2408	HLA class I	Dengue virus 2	ELISPOT	IFNg release	Positive
180794	VATTFVTPM	NS4b	2286	2294	HLA-B*35:01	Dengue virus 2	ELISPOT	IFNg release	Positive
180802	VPLLAIGCY	NS4b	2334	2342	HLA-B*35:01	Dengue virus 2	ELISPOT	IFNg release	Positive
182747	CYSQVNPITL	NS4b	2341	2350	HLA-A*24:02	Dengue virus 2	ELISPOT	IFNg release	Positive
183670	IENSSVNVSL	NS4b	2299	2308	HLA-B*08:01	Dengue virus 2	ELISPOT	IFNg release	Positive
184439	LLILCVTQVL	NS4b	2416	2425	HLA-B*08:01	Dengue virus 2	ELISPOT	IFNg release	Positive
184814	MGLGKGWPL	NS4b	2319	2327	HLA-B*08:01	Dengue virus 2	ELISPOT	IFNg release	Positive
186002	TPMLRHSIE	NS4b	2292	2300	HLA-B*08:01	Dengue virus 2	ELISPOT	IFNg release	Positive
240933	TPMLRHSI	NS4b	2292	2299	HLA-B*07:02	Dengue virus 2	ELISPOT	IFNg release	Positive
35568	LEKTKKDL	NS4b	2249	2256	HLA-B8	Dengue virus 2	ELISPOT	IFNg release	Positive
10587	DVFFTPPEK	NS5	2622	2630	HLA class I	Dengue virus 2	ELISPOT	IFNg release	Positive
150227	FTMRHKKATY	NS5	2734	2743	HLA-B*35:01	Dengue virus 2	ELISPOT	IFNg release	Positive
150309	IPMSTYGWNL	NS5	2605	2614	HLA-B*07:02	Dengue virus 2	ELISPOT	IFNg release	Positive
150541	RPTPRGTVM	NS5	3073	3081	HLA-B*07:02	Dengue virus 2	ELISPOT	IFNg release	Positive
150554	RTTWSIHAK	NS5	3283	3291	HLA-A*11:01	Dengue virus 2	ELISPOT	IFNg release	Positive
150655	TPRMCTREEF	NS5	2881	2890	HLA-B*35:01	Dengue virus 2	ELISPOT	IFNg release	Positive
150698	VPYLGKREDQ	NS5	3327	3336	HLA-B*07:02	Dengue virus 2	ELISPOT	IFNg release	Positive
150750	YTDYMPSMK	NS5	3370	3378	HLA-A*01:01	Dengue virus 2	ELISPOT	IFNg release	Positive
161186	RLLTKPWDVVPMVTQ	NS5	326	340	HLA-B*55:02	Dengue virus 2	ICS	IFNg release	Positive
163027	NIQTAINQV	NS5	3353	3361	HLA-A*02:01	Dengue virus 2	ELISPOT	IFNg release	Positive-Low
163620	TITEEIAVQ	NS5	3127	3135	HLA-A*02:01	Dengue virus 2	ELISPOT	IFNg release	Positive
174066	CGGLKNVREVKGLTKGG	NS5	2582	2598	HLA-A2	Dengue virus 2	ICS	IFNg release	Positive
180437	EPKEGTKKLM	NS5	2856	2865	HLA-B*35:01	Dengue virus 2	ELISPOT	IFNg release	Positive
180443	ETACLGKSYA	NS5	3241	3250	HLA-A*26:01	Dengue virus 2	ELISPOT	IFNg release	Positive
180483	GKKKTPRMCTREEFT	NS5	2877	2891	HLA class I	Dengue virus 2	ELISPOT	IFNg release	Positive
180488	GPGHEEPIPM	NS5	2598	2607	HLA-B*35:01	Dengue virus 2	ELISPOT	IFNg release	Positive
180500	HKKLAEAIFKLTYQN	NS5	3052	3066	HLA class I	Dengue virus 2	ELISPOT	IFNg release	Positive
180579	KSYAQMWSLMYFHRR	NS5	3247	3261	HLA class I	Dengue virus 2	ELISPOT	IFNg release	Positive
180582	KTWAYHGSYETKQTG	NS5	2792	2806	HLA class I	Dengue virus 2	ICS	IFNg release	Positive
180584	KVRKDIQQW	NS5	3177	3185	HLA-B*57:01	Dengue virus 2	ELISPOT	IFNg release	Positive
180606	LMKITAEWLW	NS5	2864	2873	HLA-B*53:01	Dengue virus 2	ELISPOT	IFNg release	Positive
180643	MAMTDTTPFGQQRVF	NS5	2832	2846	HLA class I	Dengue virus 2	ELISPOT	IFNg release	Positive
180667	MPSMKRFRRE	NS5	3374	3383	HLA-B*53:01	Dengue virus 2	ELISPOT	IFNg release	Positive
180668	MPSVIEKMET	NS5	2678	2687	HLA-B*53:01	Dengue virus 2	ELISPOT	IFNg release	Positive
180675	MSTYGWNLVRLQSGV	NS5	2607	2621	HLA class I	Dengue virus 2	ELISPOT	IFNg release	Positive
180681	NEEYTDYMPSMKRFR	NS5	3367	3381	HLA class I	Dengue virus 2	ELISPOT	IFNg release	Positive
180758	SWHYDQDHPYKTWAY	NS5	291	305	HLA class I	Dengue virus 2	ELISPOT	IFNg release	Positive
180774	TLRVLNLVENWLNNN	NS5	2652	2666	HLA class I	Dengue virus 2	ELISPOT	IFNg release	Positive

180814	WAYHGSYET	NS5	2794	2802	HLA-B*35:01	Dengue virus 2	ELISPOT	IFNg release	Positive
180817	WHYDQDHPY	NS5	2783	2791	HLA-B*35:01	Dengue virus 2	ELISPOT	IFNg release	Positive
180828	YAQMWSLMYF	NS5	3249	3258	HLA-B*35:01	Dengue virus 2	ELISPOT	IFNg release	Positive
182855	EAVEDGRFWE	NS5	2915	2924	HLA-B*58:01	Dengue virus 2	ELISPOT	IFNg release	Positive
184131	KREDQWCGSL	NS5	3332	3341	HLA-B*08:01	Dengue virus 2	ELISPOT	IFNg release	Positive
184234	KWKSRLNAL	NS5	2503	2511	HLA-B*08:01	Dengue virus 2	ELISPOT	IFNg release	Positive
184373	LGKKKTPRM	NS5	2876	2884	HLA-B*08:01	Dengue virus 2	ELISPOT	IFNg release	Positive
184840	MLINRFTMR	NS5	2729	2737	HLA-B*08:01	Dengue virus 2	ELISPOT	IFNg release	Positive
184957	MTTEDMLTVW	NS5	3295	3304	HLA-B*58:01	Dengue virus 2	ICS	IFNg release	Positive
185491	RREEEEAGVL	NS5	3381	3390	HLA-B*08:01	Dengue virus 2	ELISPOT	IFNg release	Positive
185931	TKQTGSASSM	NS5	2802	2811	HLA-B*08:01	Dengue virus 2	ELISPOT	IFNg release	Positive
186219	VEDGRFWEL	NS5	2917	2925	HLA-B*40:01	Dengue virus 2	ELISPOT	IFNg release	Positive
190862	MVTQMAMTDTTPFGQQR	NS5	2828	2844	HLA class I	Dengue virus 2	ICS	IFNg release	Positive
31747	KLAEAIFKL	NS5	3054	3062	HLA-A*02:01	Dengue virus 2	ELISPOT	IFNg release	Positive-Low
32939	KPWDIIPMV	NS5	2821	2829	HLA-B*55:02	Dengue virus 2	51 chromium	cytotoxicity	Positive
32941	KPWDVLPMV	NS5	2821	2829	HLA class I	Dengue virus 2	51 chromium	cytotoxicity	Positive
32942	KPWDVLPTV	NS5	2821	2829	HLA class I	Dengue virus 2	51 chromium	cytotoxicity	Positive
32943	KPWDVVPMV	NS5	2821	2829	HLA-B*51:01	Dengue virus 2	51 chromium	cytotoxicity	Positive
53476	REDQWCGSL	NS5	3333	3341	HLA-B*40:01	Dengue virus 2	ELISPOT	IFNg release	Positive
539144	CVVKPLDDRFASALTAL	NS5	3156	3172	HLA class I	Dengue virus 2	ICS	IFNg release	Positive
539436	ISGDDCVVKPLDDRFA	NS5	3151	3166	HLA class I	Dengue virus 2	ICS	IFNg release	Positive
69671	VLNPYMPSV	NS5	2673	2681	HLA-A*02:01	Dengue virus 2	ELISPOT	IFNg release	Positive
73428	YAQMWSLMY	NS5	3249	3257	HLA-B*15:01	Dengue virus 2	ELISPOT	IFNg release	Positive
74305	YILRDVSKK	NS5	517	525	HLA class I	Dengue virus 2	ELISPOT	IFNg release	Positive

CD8 DENV-3 Epitopes

Epitope_ID	AminoAcid_Seq	Protein_Name	Start	End	Allele	Serotype	Method/Technique	Assay Group	Qualitative Measure
180390	CLMMMLPATL	C	104	113	HLA-A*02:01	Dengue virus 3	ELISPOT	IFNg release	Positive
180577	KSGAIKVLK	C	74	82	HLA-A*11:01	Dengue virus 3	ELISPOT	IFNg release	Positive
180609	LMMMLPATL	C	105	113	HLA-A*02:01	Dengue virus 3	ELISPOT	IFNg release	Positive
184260	LAIPPTAGVL	C	57	66	HLA-B*08:01	Dengue virus 3	ELISPOT	IFNg release	Positive
184264	LAKRFSRGL	C	29	37	HLA-B*08:01	Dengue virus 3	ELISPOT	IFNg release	Positive
184265	LAKRFSRGLL	C	29	38	HLA-B*08:01	Dengue virus 3	ELISPOT	IFNg release	Positive
184845	MLKRVRNRV	C	15	23	HLA-B*08:01	Dengue virus 3	ELISPOT	IFNg release	Positive
185260	QLAKRFSRG	C	28	36	HLA-B*08:01	Dengue virus 3	ELISPOT	IFNg release	Positive
180464	FTILALFLAH	PreM	247	256	HLA-B*35:01	Dengue virus 3	ELISPOT	IFNg release	Positive
180504	HPGFTILALF	PreM	244	253	HLA-B*35:01	Dengue virus 3	ELISPOT	IFNg release	Positive
184658	LTQKVVIFIL	PreM	262	271	HLA-B*08:01	Dengue virus 3	ELISPOT	IFNg release	Positive
185685	SLLFKTASG	PreM	136	144	HLA-B*08:01	Dengue virus 3	ELISPOT	IFNg release	Positive
13119	ELKGMSYAM	E	155	163	HLA-B*08:01	Dengue virus 3	ELISPOT	IFNg release	Positive
15796	FFDLPLPWT	E	211	219	HLA-A2	Dengue virus 3	ICS	IFNg release	Positive
180613	LPEEQDQNY	E	82	90	HLA-B*35:01	Dengue virus 3	ELISPOT	IFNg release	Positive
180657	MLVTPSMTM	E	273	281	HLA-B*35:01	Dengue virus 3	ELISPOT	IFNg release	Positive
183793	IMKIGIGVLL	E	452	461	HLA-B*08:01	Dengue virus 3	ELISPOT	IFNg release	Positive
183843	IQNSGGTSIF	E	131	140	HLA-B*15:01	Dengue virus 3	ELISPOT	IFNg release	Positive
184299	LATLRKLCI	E	53	61	HLA-B*08:01	Dengue virus 3	ELISPOT	IFNg release	Positive
184412	LKGMSYAMCL	E	156	165	HLA-B*08:01	Dengue virus 3	ELISPOT	IFNg release	Positive
184536	LPEYGTLGLE	E	453	462	HLA-B*35:01	Dengue virus 3	ELISPOT	IFNg release	Positive
184568	LPWTSGATT	E	496	504	HLA-B*35:01	Dengue virus 3	ELISPOT	IFNg release	Positive
184924	MSYAMCTNTF	E	575	584	HLA-B*35:01	Dengue virus 3	ELISPOT	IFNg release	Positive
184965	MVHQIFGSAY	E	713	722	HLA-B*15:01	Dengue virus 3	ELISPOT	IFNg release	Positive
185419	RLKMDKLEL	E	148	156	HLA-B*08:01	Dengue virus 3	ELISPOT	IFNg release	Positive
186029	TPTWNRKEL	E	226	234	HLA-B*07:02	Dengue virus 3	ELISPOT	IFNg release	Positive-Low
186470	WIMKIGIGVL	E	451	460	HLA-B*08:01	Dengue virus 3	ELISPOT	IFNg release	Positive
589817	SGATWVDVVLEHGGCV	E	268	283	HLA class I	Dengue virus 3	ICS	IFNg release	Positive-High
150537	RPGYHTQTA	NS1	1030	1038	HLA-B*07:02	Dengue virus 3	ELISPOT	IFNg release	Positive
150546	RSCTLPPLRY	NS1	1087	1096	HLA-A*01:01	Dengue virus 3	ELISPOT	IFNg release	Positive
180738	RYMGEDGCWY	NS1	1095	1104	HLA-A*24:02	Dengue virus 3	ELISPOT	IFNg release	Positive
182939	EKEENMVKSL	NS1	1113	1122	HLA-B*08:01	Dengue virus 3	ELISPOT	IFNg release	Positive
183723	IKLTVVVGDI	NS1	857	866	HLA-B*08:01	Dengue virus 3	ELISPOT	IFNg release	Positive
184585	LRTTTVSGKL	NS1	64	73	HLA-B*08:01	Dengue virus 3	ELISPOT	IFNg release	Positive
185464	RPINEKEENM	NS1	1109	1118	HLA-B*35:01	Dengue virus 3	ELISPOT	IFNg release	Positive
185573	RYMGEDGCW	NS1	1095	1103	HLA-A*24:02	Dengue virus 3	ELISPOT	IFNg release	Positive
185734	SPKRLATAI	NS1	811	819	HLA-B*08:01	Dengue virus 3	ELISPOT	IFNg release	Positive
183395	GKKHMIAGVL	NS2a	1153	1162	HLA-B*08:01	Dengue virus 3	ELISPOT	IFNg release	Positive
183587	HMIAGVFFTF	NS2a	368	377	HLA-A*24:02	Dengue virus 3	ELISPOT	IFNg release	Positive
184254	LAILFEEVM	NS2a	1140	1148	HLA-B*35:01	Dengue virus 3	ELISPOT	IFNg release	Positive
184819	MIAGVFFTF	NS2a	369	377	HLA-B*35:01	Dengue virus 3	ELISPOT	IFNg release	Positive
185978	TMGVLCLAIL	NS2a	1134	1143	HLA-B*08:01	Dengue virus 3	ELISPOT	IFNg release	Positive
186175	TWRDMAHTLI	NS2a	1174	1183	HLA-A*24:02	Dengue virus 3	ELISPOT	IFNg release	Positive
180622	LPVWLAHKVA	NS3	226	235	HLA-B*35:01	Dengue virus 3	ELISPOT	IFNg release	Positive
182917	EFKDFAAGR	NS3	2083	2091	HLA-A*33:01	Dengue virus 3	ELISPOT	IFNg release	Positive
184065	KLNDWDFVV	NS3	399	407	HLA-A*02:01	Dengue virus 3	ELISPOT	IFNg release	Positive-High
186234	VEIWTKEGEK	NS3	2053	2062	HLA-B*40:01	Dengue virus 3	ELISPOT	IFNg release	Positive
19434	GESRKTFVE	NS3	2001	2009	HLA-B7	Dengue virus 3	51 chromium	cytotoxicity	Positive
19435	GESRKTFVEL	NS3	211	220	HLA-B*40:01	Dengue virus 3	ICS	IFNg release	Positive
22820	GTSGSPIINR	NS3	133	142	HLA-A*11:01	Dengue virus 3	51 chromium	cytotoxicity	Positive
22821	GTSGSPIINRE	NS3	133	143	HLA-A11	Dengue virus 3	ICS	IFNg release	Positive
3124	AMKGLPIRY	NS3	1708	1716	HLA-B62	Dengue virus 3	51 chromium	cytotoxicity	Positive
34605	KYTDRKWCF	NS3	240	248	HLA-A24	Dengue virus 3	ELISPOT	IFNg release	Positive
590435	TRVVAAEMEEAMKGLPI	NS3	1698	1714	HLA class I	Dengue virus 3	ICS	IFNg release	Positive-Low
62183	SVKKDLISY	NS3	71	79	HLA-B62	Dengue virus 3	51 chromium	cytotoxicity	Positive
183165	FFMMVLLIPE	NS4a	2205	2214	HLA-A*33:01	Dengue virus 3	ELISPOT	IFNg release	Positive
37494	LLLGLMILL	NS4a	2148	2156	HLA-A2	Dengue virus 3	ICS	IFNg release	Positive
180381	ATGPITTLW	NS4b	2439	2447	HLA-B*58:01	Dengue virus 3	ELISPOT	IFNg release	Positive
180788	TTKRDLGMSK	NS4b	2250	2259	HLA-A*11:01	Dengue virus 3	ELISPOT	IFNg release	Positive
183796	IMKSVGTGK	NS4b	2481	2489	HLA-A*03:01	Dengue virus 3	ELISPOT	IFNg release	Positive
37534	LLLMRTSWA	NS4b	2423	2431	HLA-A2	Dengue virus 3	biological activity	proliferation	Positive
69612	VLLLVTHYA	NS4b	2353	2361	HLA-A2	Dengue virus 3	ICS	IFNg release	Positive
1067501	KPIDDRFAN	NS5	3158	3166	HLA-B*07:02	Dengue virus 3	ELISPOT	IFNg release	Positive
180572	KPRLCTREEF	NS5	2879	2888	HLA-B*07:02	Dengue virus 3	ELISPOT	IFNg release	Positive
180650	MEITAEWLW	NS5	2863	2871	HLA-B*58:01	Dengue virus 3	ELISPOT	IFNg release	Positive
180678	MVSRLLLNR	NS5	2723	2731	HLA-A*11:01	Dengue virus 3	ELISPOT	IFNg release	Positive
180728	RPRLCTREEF	NS5	2879	2888	HLA-B*07:02	Dengue virus 3	ELISPOT	IFNg release	Positive
180737	RYLEFEALGF	NS5	2971	2980	HLA-A*24:02	Dengue virus 3	ELISPOT	IFNg release	Positive
180801	VMGITAEWLW	NS5	2862	2871	HLA-B*53:01	Dengue virus 3	ELISPOT	IFNg release	Positive
182553	ALLALNDMGK	NS5	3167	3176	HLA-A*03:01	Dengue virus 3	ELISPOT	IFNg release	Positive
185710	SMINGVVKL	NS5	318	326	HLA-A*02:01	Dengue virus 3	ELISPOT	IFNg release	Positive
186127	TTWEDVPYL	NS5	3321	3329	HLA-A*02:01	Dengue virus 3	ELISPOT	IFNg release	Positive
186215	VEDEDFWKL	NS5	2915	2923	HLA-B*40:01	Dengue virus 3	ELISPOT	IFNg release	Positive

32944	KPWDVVPTV	NS5	2819	2827	HLA class I	Dengue virus 3	51 chromium	cytotoxicity	Positive

CD8 DENV-4 Epitopes

Epitope_ID	AminoAcid_Seq	Protein_Name	Start	End	Allele	Serotype	Method/Technique	Assay Group	Qualitative Measure
180541	ITLLCLIPTV	C	102	111	HLA-A*02:01	Dengue virus 4	ELISPOT	IFNg release	Positive
180810	VTYECPLLV	PreM	162	170	HLA-A*02:01	Dengue virus 4	ELISPOT	IFNg release	Positive
182707	CNLTSTWVMY	PreM	181	190	HLA-A*30:02	Dengue virus 4	ELISPOT	IFNg release	Positive
182971	EMCEDTVTY	PreM	156	164	HLA-B*35:01	Dengue virus 4	ELISPOT	IFNg release	Positive
186303	VLMMLVAPSY	PreM	17	26	HLA-B*15:01	Dengue virus 4	ELISPOT	IFNg release	Positive
185147	NYKERMVTF	E	511	519	HLA-A*24:02	Dengue virus 4	ELISPOT	IFNg release	Positive
185817	SWMIRILIGF	E	161	170	HLA-A*24:02	Dengue virus 4	ELISPOT	IFNg release	Positive
583701	GVSGGAWVDLVLEHGGCV	E	14	31	HLA class I	Dengue virus 4	ICS	IFNg release	Positive-Low
183667	IEKASLIEV	NS1	986	994	HLA-B*40:01	Dengue virus 4	ELISPOT	IFNg release	Positive
185050	NFLEVEDYGF	NS1	925	934	HLA-A*24:02	Dengue virus 4	ELISPOT	IFNg release	Positive
240130	PPASDLKYSW	NS1	880	889	HLA-B*53:01	Dengue virus 4	ELISPOT	IFNg release	Positive
167725	AMTTTLSIPHDLMELIDGIS	NS2a	50	69	HLA class I	Dengue virus 4	ELISPOT	IFNg release	Positive
182562	ALPVYLMTL	NS2a	1329	1337	HLA-A*02:01	Dengue virus 4	ELISPOT	IFNg release	Positive
183818	IPHDLMELI	NS2a	1241	1249	HLA-B*53:01	Dengue virus 4	ELISPOT	IFNg release	Positive
184390	LIDGISLGL	NS2a	1248	1256	HLA-A*01:01	Dengue virus 4	ELISPOT	IFNg release	Positive
184566	LPVYLMTLMK	NS2a	1330	1339	HLA-B*35:01	Dengue virus 4	ELISPOT	IFNg release	Positive
184591	LSIPHDLMEF	NS2a	1239	1248	HLA-A*01:01	Dengue virus 4	ELISPOT	IFNg release	Positive
182901	EETNMITLL	NS2b	1434	1442	HLA-B*40:01	Dengue virus 4	ELISPOT	IFNg release	Positive
186584	YPLAIPVTM	NS2b	1454	1462	HLA-B*53:01	Dengue virus 4	ELISPOT	IFNg release	Positive
239411	ALWYVWQVK	NS2b	1463	1471	HLA-A*03:01	Dengue virus 4	ELISPOT	IFNg release	Positive
418991	IPVTMALWY	NS2b	1458	1466	HLA-B*35:01	Dengue virus 4	ELISPOT	IFNg release	Positive
156711	LAPTRVVAAEMEEAL	NS3	1695	1709	HLA-B7	Dengue virus 4	51 chromium	cytotoxicity	Positive
180473	GEFRLRGEQR	NS3	1995	2004	HLA-B*40:01	Dengue virus 4	ELISPOT	IFNg release	Positive
180475	GEQRKTFVEL	NS3	2001	2010	HLA-B*40:01	Dengue virus 4	ELISPOT	IFNg release	Positive
180640	MALKDFKEF	NS3	2079	2087	HLA-B*35:01	Dengue virus 4	ELISPOT	IFNg release	Positive
180648	MEGVFHTMW	NS3	1516	1524	HLA-B*44:03	Dengue virus 4	ELISPOT	IFNg release	Positive
180778	TPEGIIPTLF	NS3	1974	1983	HLA-B*07:02	Dengue virus 4	ELISPOT	IFNg release	Positive
180843	YPKTKLTDWD	NS3	1868	1877	HLA-B*35:01	Dengue virus 4	ELISPOT	IFNg release	Positive
180850	YTPEGIIPTL	NS3	1973	1982	HLA-A*02:06	Dengue virus 4	ELISPOT	IFNg release	Positive
182563	ALSEGVYRI	NS3	1491	1499	HLA-A*02:01	Dengue virus 4	ELISPOT	IFNg release	Positive
182919	EFRLRGEQR	NS3	1996	2004	HLA-A*33:01	Dengue virus 4	ELISPOT	IFNg release	Positive
183231	FPQSNSPIED	NS3	1799	1808	HLA-B*35:01	Dengue virus 4	ELISPOT	IFNg release	Positive
183338	GEIGAVTLDF	NS3	1595	1604	HLA-B*44:03	Dengue virus 4	ELISPOT	IFNg release	Positive
183407	GLFGKTQVGV	NS3	1503	1512	HLA-A*02:01	Dengue virus 4	ELISPOT	IFNg release	Positive
183427	GLYGNGVVTK	NS3	1622	1631	HLA-A*03:01	Dengue virus 4	ELISPOT	IFNg release	Positive
184079	KLTDWDFVV	NS3	398	406	HLA-A*02:01	Dengue virus 4	ELISPOT	IFNg release	Positive
186008	TPPGATDPF	NS3	1791	1799	HLA-B*35:01	Dengue virus 4	ELISPOT	IFNg release	Positive
186347	VPNYNLIVM	NS3	1749	1757	HLA-B*35:01	Dengue virus 4	ELISPOT	IFNg release	Positive
186602	YSDPLALRE	NS3	2077	2085	HLA-A*01:01	Dengue virus 4	ELISPOT	IFNg release	Positive
195838	PNYNLIVMDEAHFTD	NS3	1750	1764	HLA class I	Dengue virus 4	ICS	IFNg release	Positive
22822	GTSGSPIINRK	NS3	133	143	HLA-A11	Dengue virus 4	51 chromium	cytotoxicity	Positive
239768	GTSGSPIINK	NS3	1607	1616	HLA-A*03:01	Dengue virus 4	ELISPOT	IFNg release	Positive
34918	LAPTRVVAAEME	NS3	221	232	HLA-B7	Dengue virus 4	51 chromium	cytotoxicity	Positive
539058	AHFTDPSSVAARGYIST	NS3	1760	1776	HLA class I	Dengue virus 4	ICS	IFNg release	Positive
539576	LIVMDEAHFTDPSSVAA	NS3	1754	1770	HLA class I	Dengue virus 4	ICS	IFNg release	Positive
540029	VEMGEAAAIFMTATPPG	NS3	1778	1794	HLA class I	Dengue virus 4	ICS	IFNg release	Positive
581940	APTRVVAAEMEEALRGL	NS3	1558	1574	HLA class I	Dengue virus 4	ICS	IFNg release	Positive-Low
62616	SYKDREWCF	NS3	389	397	HLA-A24	Dengue virus 4	ELISPOT	IFNg release	Positive
65552	TPEGIIPTL	NS3	500	508	HLA-B*35:01	Dengue virus 4	51 chromium	cytotoxicity	Positive
180596	LETLMLVALL	NS4a	2144	2153	HLA-B*40:01	Dengue virus 4	ELISPOT	IFNg release	Positive
182508	AIAVASGLLW	NS4a	2179	2188	HLA-B*44:02	Dengue virus 4	ELISPOT	IFNg release	Positive
182926	EIASLPTYL	NS4a	2101	2109	HLA-A*68:02	Dengue virus 4	ELISPOT	IFNg release	Positive
183650	IAVASGLLW	NS4a	2180	2188	HLA-B*57:01	Dengue virus 4	ELISPOT	IFNg release	Positive
184531	LPESLETLML	NS4a	2140	2149	HLA-B*35:01	Dengue virus 4	ELISPOT	IFNg release	Positive
185881	TEIASLPTYL	NS4a	2100	2109	HLA-B*40:01	Dengue virus 4	ELISPOT	IFNg release	Positive
186620	YVILTILTI	NS4a	2228	2236	HLA-A*24:02	Dengue virus 4	ELISPOT	IFNg release	Positive
42083	MLVALLGAM	NS4a	56	64	HLA-A2	Dengue virus 4	ICS	IFNg release	Positive
136826	CYSQVNPTTL	NS4b	2337	2346	HLA-A*24:02	Dengue virus 4	ELISPOT	IFNg release	Positive
150647	TPMLRHTIEN	NS4b	2288	2297	HLA-B*07:02	Dengue virus 4	ELISPOT	IFNg release	Positive
180379	ATGPILTLW	NS4b	2436	2444	HLA-B*58:01	Dengue virus 4	ELISPOT	IFNg release	Positive
180383	ATGPVLTLW	NS4b	2436	2444	HLA-B*58:01	Dengue virus 4	ELISPOT	IFNg release	Positive
180587	LATGPVLTLW	NS4b	2435	2444	HLA-B*53:01	Dengue virus 4	ELISPOT	IFNg release	Positive
180764	TETTILDVDL	NS4b	2262	2271	HLA-B*40:01	Dengue virus 4	ELISPOT	IFNg release	Positive
180803	VPLLAMGCY	NS4b	2330	2338	HLA-B*35:01	Dengue virus 4	ELISPOT	IFNg release	Positive
183672	IENTSANLSL	NS4b	2295	2304	HLA-B*40:01	Dengue virus 4	ELISPOT	IFNg release	Positive
184453	LLLMRTTWAF	NS4b	2420	2429	HLA-A*24:02	Dengue virus 4	ELISPOT	IFNg release	Positive
184713	LYAVATTIL	NS4b	2279	2287	HLA-A*24:02	Dengue virus 4	ELISPOT	IFNg release	Positive
186330	VMLLVHYAI	NS4b	2351	2359	HLA-A*24:02	Dengue virus 4	ELISPOT	IFNg release	Positive
37535	LLLMRTTWA	NS4b	76	84	HLA-A2	Dengue virus 4	ICS	TNF release	Positive
40472	LVMLLVHYA	NS4b	6	14	HLA-A2	Dengue virus 4	ICS	IFNg release	Positive
1067215	HSWEDIPYL	NS5	3319	3327	HLA-A*02:01	Dengue virus 4	ELISPOT	IFNg release	Positive
150548	RSNAAIGAVF	NS5	2891	2900	HLA-B*15:01	Dengue virus 4	ELISPOT	IFNg release	Positive
180684	NPRLCTREEF	NS5	2877	2886	HLA-B*07:02	Dengue virus 4	ELISPOT	IFNg release	Positive
180718	REDLWCGSL	NS5	3330	3338	HLA-B*40:01	Dengue virus 4	ELISPOT	IFNg release	Positive

183353	GESSSNPTI	NS5	2635	2643	HLA-B*44:02	Dengue virus 4	ELISPOT	IFNg release	Positive
183570	HEMYWVSGV	NS5	2703	2711	HLA-B*44:02	Dengue virus 4	ELISPOT	IFNg release	Positive
184051	KLGEFGRAK	NS5	2948	2956	HLA-A*03:01	Dengue virus 4	ELISPOT	IFNg release	Positive
184130	KREDLWCGSL	NS5	3329	3338	HLA-B*08:01	Dengue virus 4	ELISPOT	IFNg release	Positive
184375	LGKSYAQMW	NS5	3246	3254	HLA-B*57:01	Dengue virus 4	ELISPOT	IFNg release	Positive
184954	MTTEDMLKVW	NS5	3292	3301	HLA class I	Dengue virus 4	ICS	IFNg release	Positive
185232	QEEHKETWHY	NS5	2772	2781	HLA-B*44:02	Dengue virus 4	ELISPOT	IFNg release	Positive
186453	WEPSKGWKNW	NS5	3182	3191	HLA-B*44:02	Dengue virus 4	ELISPOT	IFNg release	Positive
186550	YDQENPYRTW	NS5	2781	2790	HLA-B*44:02	Dengue virus 4	ELISPOT	IFNg release	Positive
186640	YYMATLKNV	NS5	2576	2584	HLA-A*24:02	Dengue virus 4	ELISPOT	IFNg release	Positive
186641	YYMATLKNVT	NS5	2576	2585	HLA-A*24:02	Dengue virus 4	ELISPOT	IFNg release	Positive
419017	MPAMKRYSAP	NS5	3371	3380	HLA-B*35:01	Dengue virus 4	ELISPOT	IFNg release	Positive
419048	SPRLCTREEF	NS5	2877	2886	HLA-B*07:02	Dengue virus 4	ELISPOT	IFNg release	Positive
539149	DCVVKPLDERFSTSLLF	NS5	3152	3168	HLA class I	Dengue virus 4	ICS	IFNg release	Positive
539656	MAISGDDCVVKPLDERF	NS5	3146	3162	HLA class I	Dengue virus 4	ICS	IFNg release	Positive
539703	MVTQLAMTDTTPFGQQR	NS5	2824	2840	HLA class I	Dengue virus 4	ICS	IFNg release	Positive

***Supplementary Table S2. Dengue polyprotein sequences of circulating viruses from India*.**

Details of the 107 full length sequences of circulating dengue viruses from India are shown.

NCBI_ID	Strain_Name	Host	Date	Continent	Country	Prot_Name	Virus_Type
UBI73847.1	FT_MD_169_R4_DENV1D_III_India	Human	2018-01-01	Asia	India	polyprotein	Dengue virus 1
ASD49619.1	3083	Human	2014-01-01	Asia	India	polyprotein	Dengue virus 1
AFZ40118.1	DENV-1/IND/55290/2005	Human	2005-01-01	Asia	India	polyprotein	Dengue virus 1
ANC57581.1	DENV1-16687	Human	2011-01-01	Asia	India	polyprotein	Dengue virus 1
QCZ25006.1	FT_DNS1_295_S13_R3	Human	2016-10-06	Asia	India	polyprotein	Dengue virus 1
QCZ25005.1	FT_DNS1_311_S14_R3	Human	2016-10-05	Asia	India	polyprotein	Dengue virus 1
QCZ25004.1	FT_DNS1_323_S15_R3	Human	2016-10-07	Asia	India	polyprotein	Dengue virus 1
QCZ25002.1	FT_DNS1_442_S18_R3	Human	2016-10-20	Asia	India	polyprotein	Dengue virus 1
QCZ25000.1	FT_DNS1_449_S21_R3	Human	2016-08-10	Asia	India	polyprotein	Dengue virus 1
QCZ24998.1	FT_DNS1_456_S24_R3	Human	2016-10-24	Asia	India	polyprotein	Dengue virus 1
QCZ24995.1	FT_DNS1_492_S91_R3	Human	2016-04-09	Asia	India	polyprotein	Dengue virus 1
QCZ24994.1	FT_DNS1_495_S92_R3_	Human	2016-10-27	Asia	India	polyprotein	Dengue virus 1
QBA57534.1	MCVR0543AC/2017	Human	2017-04-26	Asia	India	polyprotein	Dengue virus 1
QBA57538.1	MCVR1715AD/2017	Human	2017-10-22	Asia	India	polyprotein	Dengue virus 1
AJP08954.1	NIV_K130706770_India	Human	2013-01-07	Asia	India	polyprotein	Dengue virus 1
QCE20686.1	R1_BC03_SP9_22042017	Human	2017-04-22	Asia	India	polyprotein	Dengue virus 1
AXX75607.1	R1_J10	Human	2016-01-01	Asia	India	polyprotein	Dengue virus 1
AXX75606.1	R1_J47	Human	2016-01-01	Asia	India	polyprotein	Dengue virus 1
AFN54940.1	RGCB294	Human	2007-07-26	Asia	India	polyprotein	Dengue virus 1
AFN54941.1	RGCB419	Human	2008-10-23	Asia	India	polyprotein	Dengue virus 1
AFN54942.1	RGCB585	Human	2009-04-05	Asia	India	polyprotein	Dengue virus 1
AFN54943.1	RGCB592	Human	2009-04-06	Asia	India	polyprotein	Dengue virus 1
AHZ44537.1	RR107	Human	2011-01-01	Asia	India	polyprotein	Dengue virus 1
AFZ40223.1	RR121	Human	2010-01-01	Asia	India	polyprotein	Dengue virus 1
AFZ40224.1	RR57	Human	2009-01-01	Asia	India	polyprotein	Dengue virus 1
QCE20685.1	SJ290	Human	2016-01-09	Asia	India	polyprotein	Dengue virus 1
AXX75608.1	T24_S68	Human	2012-01-01	Asia	India	polyprotein	Dengue virus 1
AXX75609.1	T28_S69	Human	2012-01-01	Asia	India	polyprotein	Dengue virus 1
ASD49618.1	UOH_21749	Human	2014-01-01	Asia	India	polyprotein	Dengue virus 1
AFZ40227.1	1392	Human	2009-01-01	Asia	India	polyprotein	Dengue virus 2
AYJ72763.1	Cosmopolitan_1	Human	2012-11-08	Asia	India	polyprotein	Dengue virus 2
AYJ72772.1	Cosmopolitan_10	Human	2012-01-01	Asia	India	polyprotein	Dengue virus 2
AYJ72773.1	Cosmopolitan_11	Human	2014-10-28	Asia	India	polyprotein	Dengue virus 2
AYJ72774.1	Cosmopolitan_12	Human	2013-08-19	Asia	India	polyprotein	Dengue virus 2
AYJ72775.1	Cosmopolitan_13	Human	2013-09-04	Asia	India	polyprotein	Dengue virus 2
AYJ72776.1	Cosmopolitan_14	Human	2014-09-16	Asia	India	polyprotein	Dengue virus 2
AYJ72777.1	Cosmopolitan_15	Human	2015-09-09	Asia	India	polyprotein	Dengue virus 2
AYJ72778.1	Cosmopolitan_16	Human	2015-08-31	Asia	India	polyprotein	Dengue virus 2
AYJ72779.1	Cosmopolitan_17	Human	2014-10-25	Asia	India	polyprotein	Dengue virus 2
AYJ72780.1	Cosmopolitan_18	Human	2013-09-09	Asia	India	polyprotein	Dengue virus 2
AYJ72764.1	Cosmopolitan_2	Human	2014-01-01	Asia	India	polyprotein	Dengue virus 2
AYJ72765.1	Cosmopolitan_3	Human	2013-08-30	Asia	India	polyprotein	Dengue virus 2
AYJ72766.1	Cosmopolitan_4	Human	2014-09-22	Asia	India	polyprotein	Dengue virus 2
AYJ72767.1	Cosmopolitan_5	Human	2014-11-07	Asia	India	polyprotein	Dengue virus 2
AYJ72768.1	Cosmopolitan_6	Human	2012-10-08	Asia	India	polyprotein	Dengue virus 2
AYJ72769.1	Cosmopolitan_7	Human	2012-10-19	Asia	India	polyprotein	Dengue virus 2
AYJ72770.1	Cosmopolitan_8	Human	2012-10-19	Asia	India	polyprotein	Dengue virus 2
AYJ72771.1	Cosmopolitan_9	Human	2012-09-24	Asia	India	polyprotein	Dengue virus 2
ACQ44493.1	DENV-2/IN/BID-V2961/2006	Human	2006-01-01	Asia	India	polyprotein	Dengue virus 2
AFZ40121.1	DENV-2/IND/053598/2005	Human	2005-01-01	Asia	India	polyprotein	Dengue virus 2
ANC57597.1	DENV2-2627	Human	2006-01-01	Asia	India	polyprotein	Dengue virus 2
QCZ24979.1	FT_AJ_27_S45_R3	Human	2017-10-05	Asia	India	polyprotein	Dengue virus 2
QCZ24978.1	FT_DNS1_101_S4_R3	Human	2016-09-08	Asia	India	polyprotein	Dengue virus 2
QCZ24977.1	FT_DNS1_105_S5_R3	Human	2016-09-08	Asia	India	polyprotein	Dengue virus 2
QCZ24976.1	FT_DNS1_251_S9_R3	Human	2016-09-11	Asia	India	polyprotein	Dengue virus 2
QCZ24974.1	FT_DNS1_440_S17_R3	Human	2016-10-19	Asia	India	polyprotein	Dengue virus 2
QCZ24973.1	FT_DNS1_443_S19_R3	Human	2016-10-20	Asia	India	polyprotein	Dengue virus 2
QCZ24971.1	FT_DNS1_468_S30_R3	Human	2016-10-05	Asia	India	polyprotein	Dengue virus 2
QCZ24970.1	FT_DNS1_469_S31_R3	Human	2016-10-13	Asia	India	polyprotein	Dengue virus 2
QCZ24969.1	FT_DNS1_472_S32_R3	Human	2016-10-13	Asia	India	polyprotein	Dengue virus 2
QCZ24966.1	FT_J221_S36_R2	Human	2016-08-26	Asia	India	polyprotein	Dengue virus 2
QCZ24965.1	FT_J250_S45_R2	Human	2016-08-31	Asia	India	polyprotein	Dengue virus 2
QBA57539.1	MCVR1233AD/2017	Human	2017-10-12	Asia	India	polyprotein	Dengue virus 2
QBA57535.1	MCVR2188AB/2016	Human	2016-10-26	Asia	India	polyprotein	Dengue virus 2
QBA57537.1	MCVR7291AD/2018	Human	2018-04-23	Asia	India	polyprotein	Dengue virus 2

AFZ40226.1	Od2112	Human	2011-01-01	Asia	India	polyprotein	Dengue virus 2
AII99332.1	P23085 INDI-60	Human	2007-04-01	Asia	India	polyprotein	Dengue virus 2
AYJ72757.1	R1_J17_DENV2	Human	2016-01-01	Asia	India	flavivirus polyprotein	Dengue virus 2
AYJ72758.1	R1_J22_DENV2	Human	2016-01-01	Asia	India	flavivirus polyprotein	Dengue virus 2
AYJ72759.1	R1_J30_DENV2	Human	2016-01-01	Asia	India	flavivirus polyprotein	Dengue virus 2
AYJ72760.1	R1_J36_DENV2	Human	2016-01-01	Asia	India	flavivirus polyprotein	Dengue virus 2
AYJ72761.1	R1_J45_DENV2	Human	2016-01-01	Asia	India	flavivirus polyprotein	Dengue virus 2
AYJ72762.1	R1_J46_DENV2	Human	2016-01-01	Asia	India	flavivirus polyprotein	Dengue virus 2
ARO52692.1	RGCB880/2010	Human	2010-09-01	Asia	India	polyprotein	Dengue virus 2
ARO52693.1	RGCB921/2011	Human	2011-01-03	Asia	India	polyprotein	Dengue virus 2
AFZ40225.1	RR44	Human	2009-01-01	Asia	India	polyprotein	Dengue virus 2
AYE66920.1	UoH_583351	Human	2014-01-01	Asia	India	polyprotein	Dengue virus 2
AYE66919.1	UoH_58620	Human	2014-01-01	Asia	India	polyprotein	Dengue virus 2
ADM63678.1	DEL-72	Human	2008-01-01	Asia	India	polyprotein	Dengue virus 3
AFZ40126.1	DENV-3/IND/58760/2005	Human	2005-01-01	Asia	India	polyprotein	Dengue virus 3
AFZ40127.1	DENV-3/IND/59826/2005	Human	2005-01-01	Asia	India	polyprotein	Dengue virus 3
ANC57607.1	DENV3-2994	Human	2009-01-01	Asia	India	polyprotein	Dengue virus 3
ANC57612.1	DENV3-9468	Human	2011-01-01	Asia	India	polyprotein	Dengue virus 3
QCZ25021.1	FT_AJ_39_S47_R3	Human	2017-10-12	Asia	India	polyprotein	Dengue virus 3
QCZ25020.1	FT_DNS1_473_S33_R3	Human	2016-10-14	Asia	India	polyprotein	Dengue virus 3
QCZ25019.1	FT_DNS1_494_S54_R3	Human	2016-08-24	Asia	India	polyprotein	Dengue virus 3
QCZ25016.1	FT_J226_S40_R2	Human	2016-08-26	Asia	India	polyprotein	Dengue virus 3
QCZ25017.1	FT_J92_S12_R2	Human	2016-08-10	Asia	India	polyprotein	Dengue virus 3
QBA57533.1	MCVR7048AB/2017	Human	2017-02-21	Asia	India	polyprotein	Dengue virus 3
QEP41653.1	MD_33_R4_DENV3_III_India	Human	2017-10-27	Asia	India	polyprotein	Dengue virus 3
ACV04798.1	ND143	Human	2007-01-01	Asia	India	polyprotein	Dengue virus 3
AXX75610.1	R1_J18	Human	2016-01-01	Asia	India	polyprotein	Dengue virus 3
AXX75612.1	R1_J29	Human	2016-01-01	Asia	India	polyprotein	Dengue virus 3
AXX75611.1	R1_J51	Human	2016-01-01	Asia	India	polyprotein	Dengue virus 3
AML29863.1	Rajasthan.India/Balotra 87-s/2013	Human	2013-11-11	Asia	India	polyprotein	Dengue virus 3
AXX75613.1	T38_S72	Human	2013-01-01	Asia	India	polyprotein	Dengue virus 3
QEP41661	MD_177_R4_DENV3_III_India	Human	2018-10-13	Asia	India	polyprotein	Dengue virus 3
QEP41655	MD_137_R4_DENV3_III_India	Human	2018-09-06	Asia	India	polyprotein	Dengue virus 3
AVW85694.1	D4/IND/PUNE/IRSHA-FG-01 (1028)	Human	2016-01-01	Asia	India	polyprotein	Dengue virus 4
AVW85695.1	D4/IND/PUNE/IRSHA-FG-02 (S-41)	Human	2016-01-01	Asia	India	polyprotein	Dengue virus 4
AVW85696.1	D4/IND/PUNE/IRSHA-FG-03 (S-49)	Human	2016-01-01	Asia	India	polyprotein	Dengue virus 4
AFZ40130.1	DENV-4/IND/0952326/2009	Human	2009-01-01	Asia	India	polyprotein	Dengue virus 4
ANC57613.1	DENV4-3274	Human	2009-01-01	Asia	India	polyprotein	Dengue virus 4
QBA57536.1	MCVR4454AC/2017	Human	2017-07-08	Asia	India	polyprotein	Dengue virus 4
QCE20687.1	R1_BC09_SP50_Blr_IND_04062017	Human	2017-06-04	Asia	India	polyprotein	Dengue virus 4
ARB18129.1	UOH_23916	Human	2015-01-01	Asia	India	polyprotein	Dengue virus 4
QDZ58859	FT_MD_139_R4	Human	2018-11-09	Asia	India	polyprotein	Dengue virus 4

Supplementary Table S3. Alignment of CD4 and CD8 epitopes in circulating viruses from India. *Detailed information on the globally reported CD8/CD4 T cell epitopes that are not present in any of the viral isolates, present in all of the viral isolates, present in only 81% – <100% or 51% – 80% or in only >0% – 50% of viral isolates for each of the epitopes for all the four dengue serotypes. The globally reported CD4/CD8 T cell epitopes are organized into eight categories: DENV-1 specific CD4 epitopes, DENV-2 specific CD4 epitopes, DENV-3 specific CD4 epitopes, DENV-4 specific CD4 epitopes, DENV-1 specific CD8 epitopes, DENV-2 specific CD8 epitopes, DENV-3 specific CD8 epitopes and DENV-4 specific CD8 epitopes. In each category, identity in individual viral isolates is detailed. Green color shade and number 1 indicates 100% identity in the given viral isolate. Pink color shade and number zero indicates not identical in the given viral isolate.*

CD4 DENV-1 Epitopes

											NCBI IDs of DENV-1 circulating strains from India, (100% identity=1,green ; else 0, red)																												
Epitope_ID	AA_Seq	Protein_Name	Start	End	Allele	step	Serotype	Method/Technique	Assay Group	Qualitative Measure	UBI73847.1	ASD49619.1	AFZ40118.1	ANC57581.1	QCZ25006.1	QCZ25005.1	QCZ25004.1	QCZ25002.1	QCZ25000.1	QCZ24998.1	QCZ24995.1	QCZ24994.1	QBA57534.1	QBA57538.1	AJP08954.1	QCE20686.1	AXX75607.1	AXX75606.1	AFN54940.1	AFN54941.1	AFN54942.1	AFN54943.1	AHZ44537.1	AFZ40223.1	AFZ40224.1	QCE20685.1	AXX75608.1	AXX75609.1	ASD49618.1
195246	EISNMLNIMNRRKRT	C	87	101	HLA-DRB1*13:01	0%	Dengue virus 1	ELISPOT	IFNg release	Positive	0	0	0	0	0	0	0	0	0	0	0	0	0	0	0	0	0	0	0	0	0	0	0	0	0	0	0	0	0
195560	KEVSNMLNIMNRRKR	C	86	100	HLA-DRB1*13:01	0%	Dengue virus 1	ELISPOT	IFNg release	Positive	0	0	0	0	0	0	0	0	0	0	0	0	0	0	0	0	0	0	0	0	0	0	0	0	0	0	0	0	0
195884	RGFKKEISNMLSIMN	C	82	96	HLA-DRB1*04:01	0%	Dengue virus 1	ELISPOT	IFNg release	Positive	0	0	0	0	0	0	0	0	0	0	0	0	0	0	0	0	0	0	0	0	0	0	0	0	0	0	0	0	0
196188	VSVLFMLLPTALAFH	C	102	116	HLA-DRB1*01:01	0%	Dengue virus 1	ELISPOT	IFNg release	Positive	0	0	0	0	0	0	0	0	0	0	0	0	0	0	0	0	0	0	0	0	0	0	0	0	0	0	0	0	0
539048	AFMAFLRFLAIPPTA	C	27	41	HLA-DRB1*15:02	0%	Dengue virus 1	ELISPOT	IFNg release	Positive	0	0	0	0	0	0	0	0	0	0	0	0	0	0	0	0	0	0	0	0	0	0	0	0	0	0	0	0	0
539681	MLNIRNRRKRSVTML	C	91	105	HLA-DRB1*13:01	0%	Dengue virus 1	ELISPOT	IFNg release	Positive	0	0	0	0	0	0	0	0	0	0	0	0	0	0	0	0	0	0	0	0	0	0	0	0	0	0	0	0	0
539816	QPSFNMLKRARNRVS	C	10	24	HLA-DRB1*08:03	0%	Dengue virus 1	ELISPOT	IFNg release	Positive	0	0	0	0	0	0	0	0	0	0	0	0	0	0	0	0	0	0	0	0	0	0	0	0	0	0	0	0	0
195359	GGLFTSVGKLIHQVF	E	706	720	HLA-DRB1*11:01	0%	Dengue virus 1	ELISPOT	IFNg release	Positive	0	0	0	0	0	0	0	0	0	0	0	0	0	0	0	0	0	0	0	0	0	0	0	0	0	0	0	0	0
195484	IGGLFTSVGKLIHQV	E	705	719	HLA-DRB1*07:01	0%	Dengue virus 1	ELISPOT	IFNg release	Positive	0	0	0	0	0	0	0	0	0	0	0	0	0	0	0	0	0	0	0	0	0	0	0	0	0	0	0	0	0
539840	REDLLVTFKTAHAKK	E	513	527	HLA-DRB1*04:03	0%	Dengue virus 1	ELISPOT	IFNg release	Positive	0	0	0	0	0	0	0	0	0	0	0	0	0	0	0	0	0	0	0	0	0	0	0	0	0	0	0	0	0
539854	RKDLLVTFKTAHAKK	E	513	527	HLA-DRB1*04:03	0%	Dengue virus 1	ELISPOT	IFNg release	Positive	0	0	0	0	0	0	0	0	0	0	0	0	0	0	0	0	0	0	0	0	0	0	0	0	0	0	0	0	0
867045	HAKKQEVAVLGSQEG	E	244	258	HLA-DQA1*03:01/DQB1*03:02	0%	Dengue virus 1	ELISPOT	IFNg release	Positive	0	0	0	0	0	0	0	0	0	0	0	0	0	0	0	0	0	0	0	0	0	0	0	0	0	0	0	0	0
869085	SYIVIGAGKKALKLS	E	376	390	HLA-DRB5*01:01	0%	Dengue virus 1	ELISPOT	IFNg release	Positive	0	0	0	0	0	0	0	0	0	0	0	0	0	0	0	0	0	0	0	0	0	0	0	0	0	0	0	0	0
869690	WLAHKQWFLDLPLPW	E	206	220	HLA-DQA1*01:01/DQB1*05:01	0%	Dengue virus 1	ELISPOT	IFNg release	Positive	0	0	0	0	0	0	0	0	0	0	0	0	0	0	0	0	0	0	0	0	0	0	0	0	0	0	0	0	0
195684	LNHILLENDIKFTVV	NS1	850	864	HLA-DRB1*03:01	0%	Dengue virus 1	ELISPOT	IFNg release	Positive	0	0	0	0	0	0	0	0	0	0	0	0	0	0	0	0	0	0	0	0	0	0	0	0	0	0	0	0	0
195690	LNYILLENDMKFTVV	NS1	850	864	HLA-DRB1*03:01	0%	Dengue virus 1	ELISPOT	IFNg release	Positive	0	0	0	0	0	0	0	0	0	0	0	0	0	0	0	0	0	0	0	0	0	0	0	0	0	0	0	0	0
591686	KFTVVVGDVSGILAQ	NS1	860	874	HLA-DRB1*03:01	0%	Dengue virus 1	ELISPOT	IFNg release	Positive	0	0	0	0	0	0	0	0	0	0	0	0	0	0	0	0	0	0	0	0	0	0	0	0	0	0	0	0	0
868492	QISNELNYILLENDM	NS1	845	859	HLA-DPB1*01:01	0%	Dengue virus 1	ELISPOT	IFNg release	Positive	0	0	0	0	0	0	0	0	0	0	0	0	0	0	0	0	0	0	0	0	0	0	0	0	0	0	0	0	0
868630	QYKFQADSPKKLSAA	NS1	806	820	HLA-DRB3*02:02	0%	Dengue virus 1	ELISPOT	IFNg release	Positive	0	0	0	0	0	0	0	0	0	0	0	0	0	0	0	0	0	0	0	0	0	0	0	0	0	0	0	0	0
195196	AWKTMAMALSIVSLF	NS2a	1291	1305	HLA-DRB1*08:02	0%	Dengue virus 1	ELISPOT	IFNg release	Positive	0	0	0	0	0	0	0	0	0	0	0	0	0	0	0	0	0	0	0	0	0	0	0	0	0	0	0	0	0
195285	FAVGLLFRRLTSREI	NS2a	1214	1228	HLA-DRB1*11:01	0%	Dengue virus 1	ELISPOT	IFNg release	Positive	0	0	0	0	0	0	0	0	0	0	0	0	0	0	0	0	0	0	0	0	0	0	0	0	0	0	0	0	0
195323	FQSHQLWATLLSLTF	NS2a	1267	1281	HLA-DPB1*04:02	0%	Dengue virus 1	ELISPOT	IFNg release	Positive	0	0	0	0	0	0	0	0	0	0	0	0	0	0	0	0	0	0	0	0	0	0	0	0	0	0	0	0	0
195389	GLLFRKLTSREVLLL	NS2a	1217	1231	HLA-DRB1*11:01	0%	Dengue virus 1	ELISPOT	IFNg release	Positive	0	0	0	0	0	0	0	0	0	0	0	0	0	0	0	0	0	0	0	0	0	0	0	0	0	0	0	0	0
195390	GLLFRRLTSREILLL	NS2a	1217	1231	HLA-DRB1*07:01	0%	Dengue virus 1	ELISPOT	IFNg release	Positive	0	0	0	0	0	0	0	0	0	0	0	0	0	0	0	0	0	0	0	0	0	0	0	0	0	0	0	0	0
195392	GLLFRRLTSREVPLL	NS2a	1217	1231	HLA-DRB1*10:01	0%	Dengue virus 1	ELISPOT	IFNg release	Positive	0	0	0	0	0	0	0	0	0	0	0	0	0	0	0	0	0	0	0	0	0	0	0	0	0	0	0	0	0
195422	GTLAVFLLLIMGQLT	NS2a	1162	1176	HLA-DRB1*01:01	0%	Dengue virus 1	ELISPOT	IFNg release	Positive	0	0	0	0	0	0	0	0	0	0	0	0	0	0	0	0	0	0	0	0	0	0	0	0	0	0	0	0	0
195423	GTLVVFFLLIMGQLT	NS2a	1162	1176	HLA-DRB1*15:01	0%	Dengue virus 1	ELISPOT	IFNg release	Positive	0	0	0	0	0	0	0	0	0	0	0	0	0	0	0	0	0	0	0	0	0	0	0	0	0	0	0	0	0
195613	LAVFLLLIMGQLTWN	NS2a	1164	1178	HLA-DRB1*15:06	0%	Dengue virus 1	ELISPOT	IFNg release	Positive	0	0	0	0	0	0	0	0	0	0	0	0	0	0	0	0	0	0	0	0	0	0	0	0	0	0	0	0	0
195614	LAVGLLFRRLTSREV	NS2a	1214	1228	HLA-DRB1*11:01	0%	Dengue virus 1	ELISPOT	IFNg release	Positive	0	0	0	0	0	0	0	0	0	0	0	0	0	0	0	0	0	0	0	0	0	0	0	0	0	0	0	0	0
195670	LLSLTFIKTTFSLHY	NS2a	1276	1290	HLA-DRB1*07:01	0%	Dengue virus 1	ELISPOT	IFNg release	Positive	0	0	0	0	0	0	0	0	0	0	0	0	0	0	0	0	0	0	0	0	0	0	0	0	0	0	0	0	0
195713	LSLTFIRTSLSLDYA	NS2a	1277	1291	HLA-DRB1*03:01	0%	Dengue virus 1	ELISPOT	IFNg release	Positive	0	0	0	0	0	0	0	0	0	0	0	0	0	0	0	0	0	0	0	0	0	0	0	0	0	0	0	0	0
195756	MGLLFRRLTSREVLL	NS2a	1216	1230	HLA-DRB1*11:01	0%	Dengue virus 1	ELISPOT	IFNg release	Positive	0	0	0	0	0	0	0	0	0	0	0	0	0	0	0	0	0	0	0	0	0	0	0	0	0	0	0	0	0
195881	REVLLLTVGLSLVAC	NS2a	1226	1240	HLA-DRB1*07:01	0%	Dengue virus 1	ELISPOT	IFNg release	Positive	0	0	0	0	0	0	0	0	0	0	0	0	0	0	0	0	0	0	0	0	0	0	0	0	0	0	0	0	0
195959	SILIEEVMRSRWSRK	NS2a	1143	1157	HLA-DRB1*11:04	0%	Dengue virus 1	ELISPOT	IFNg release	Positive	0	0	0	0	0	0	0	0	0	0	0	0	0	0	0	0	0	0	0	0	0	0	0	0	0	0	0	0	0
539624	LSLTFIKTTLSLHYA	NS2a	1277	1291	HLA-DRB1*14:04	0%	Dengue virus 1	ELISPOT	IFNg release	Positive	0	0	0	0	0	0	0	0	0	0	0	0	0	0	0	0	0	0	0	0	0	0	0	0	0	0	0	0	0
539625	LSLTFIKTTYSLHYA	NS2a	1277	1291	HLA-DRB1*14:04	0%	Dengue virus 1	ELISPOT	IFNg release	Positive	0	0	0	0	0	0	0	0	0	0	0	0	0	0	0	0	0	0	0	0	0	0	0	0	0	0	0	0	0
539626	LSLTFIRSTIPLVMA	NS2a	1275	1289	HLA-DRB1*14:04	0%	Dengue virus 1	ELISPOT	IFNg release	Positive	0	0	0	0	0	0	0	0	0	0	0	0	0	0	0	0	0	0	0	0	0	0	0	0	0	0	0	0	0
539651	LVVFLLLIMGQLTWS	NS2a	1164	1178	HLA-DRB1*15:06	0%	Dengue virus 1	ELISPOT	IFNg release	Positive	0	0	0	0	0	0	0	0	0	0	0	0	0	0	0	0	0	0	0	0	0	0	0	0	0	0	0	0	0
539657	MALSIVSLFPLCLST	NS2a	1297	1311	HLA-DRB1*15:06	0%	Dengue virus 1	ELISPOT	IFNg release	Positive	0	0	0	0	0	0	0	0	0	0	0	0	0	0	0	0	0	0	0	0	0	0	0	0	0	0	0	0	0
539679	MLMTGTLAVFLLLIM	NS2a	1158	1172	HLA-DRB1*15:06	0%	Dengue virus 1	ELISPOT	IFNg release	Positive	0	0	0	0	0	0	0	0	0	0	0	0	0	0	0	0	0	0	0	0	0	0	0	0	0	0	0	0	0
539680	MLMTGTLVVFFLLIM	NS2a	1158	1172	HLA-DRB1*15:06	0%	Dengue virus 1	ELISPOT	IFNg release	Positive	0	0	0	0	0	0	0	0	0	0	0	0	0	0	0	0	0	0	0	0	0	0	0	0	0	0	0	0	0
539912	SLDYAWKTMAMALSI	NS2a	1287	1301	HLA-DRB1*09:01	0%	Dengue virus 1	ELISPOT	IFNg release	Positive	0	0	0	0	0	0	0	0	0	0	0	0	0	0	0	0	0	0	0	0	0	0	0	0	0	0	0	0	0
539918	SLTFIKTTLSLHHAW	NS2a	1278	1292	HLA-DRB1*14:04	0%	Dengue virus 1	ELISPOT	IFNg release	Positive	0	0	0	0	0	0	0	0	0	0	0	0	0	0	0	0	0	0	0	0	0	0	0	0	0	0	0	0	0
539974	TGTLVVFLLLTLGQL	NS2a	1161	1175	HLA-DRB1*15:06	0%	Dengue virus 1	ELISPOT	IFNg release	Positive	0	0	0	0	0	0	0	0	0	0	0	0	0	0	0	0	0	0	0	0	0	0	0	0	0	0	0	0	0
540006	TTLLSLTFIRTSLSL	NS2a	1274	1288	HLA-DRB1*12:02	0%	Dengue virus 1	ELISPOT	IFNg release	Positive	0	0	0	0	0	0	0	0	0	0	0	0	0	0	0	0	0	0	0	0	0	0	0	0	0	0	0	0	0
591779	TLAVFLLLTMGQLTW	NS2a	36	50	HLA-DRB1*11:01	0%	Dengue virus 1	ELISPOT	IFNg release	Positive	0	0	0	0	0	0	0	0	0	0	0	0	0	0	0	0	0	0	0	0	0	0	0	0	0	0	0	0	0
739424	LAVFLLLVMGQLTWN	NS2a	1164	1178	HLA-DRB1*11:04	0%	Dengue virus 1	ELISPOT	IFNg release	Positive	0	0	0	0	0	0	0	0	0	0	0	0	0	0	0	0	0	0	0	0	0	0	0	0	0	0	0	0	0
739578	LSLTFIKTTLSLHHA	NS2a	1277	1291	HLA-DRB1*04:07	0%	Dengue virus 1	ELISPOT	IFNg release	Positive	0	0	0	0	0	0	0	0	0	0	0	0	0	0	0	0	0	0	0	0	0	0	0	0	0	0	0	0	0
866118	AMALSIVSLFPLCLS	NS2a	1296	1310	HLA-DPB1*04:01	0%	Dengue virus 1	ELISPOT	IFNg release	Positive	0	0	0	0	0	0	0	0	0	0	0	0	0	0	0	0	0	0	0	0	0	0	0	0	0	0	0	0	0
866767	FQSYQLWATLLSLTF	NS2a	1267	1281	HLA-DPB1*04:02	0%	Dengue virus 1	ELISPOT	IFNg release	Positive	0	0	0	0	0	0	0	0	0	0	0	0	0	0	0	0	0	0	0	0	0	0	0	0	0	0	0	0	0
866873	GILAVFFLLIMGQLT	NS2a	1162	1176	HLA-DPB1*04:02	0%	Dengue virus 1	ELISPOT	IFNg release	Positive	0	0	0	0	0	0	0	0	0	0	0	0	0	0	0	0	0	0	0	0	0	0	0	0	0	0	0	0	0
867997	LTSREILLLTVGLSL	NS2a	1223	1237	HLA-DPB1*04:02	0%	Dengue virus 1	ELISPOT	IFNg release	Positive	0	0	0	0	0	0	0	0	0	0	0	0	0	0	0	0	0	0	0	0	0	0	0	0	0	0	0	0	0
868553	QPHQLWATLLSLTFV	NS2a	1268	1282	HLA-DPB1*04:02	0%	Dengue virus 1	ELISPOT	IFNg release	Positive	0	0	0	0	0	0	0	0	0	0	0	0	0	0	0	0	0	0	0	0	0	0	0	0	0	0	0	0	0
869103	TDFQPHQLWTTLLSL	NS2a	1265	1279	HLA-DPB1*04:02	0%	Dengue virus 1	ELISPOT	IFNg release	Positive	0	0	0	0	0	0	0	0	0	0	0	0	0	0	0	0	0	0	0	0	0	0	0	0	0	0	0	0	0
866200	ATLFLWYFWQKKKQR	NS2b	1461	1475	HLA-DPB1*01:01	0%	Dengue virus 1	ELISPOT	IFNg release	Positive	0	0	0	0	0	0	0	0	0	0	0	0	0	0	0	0	0	0	0	0	0	0	0	0	0	0	0	0	0
24915	HTGKEIVDLMCHATE	NS3	251	265	HLA-DPw2	0%	Dengue virus 1	ICS	IFNg release	Positive	0	0	0	0	0	0	0	0	0	0	0	0	0	0	0	0	0	0	0	0	0	0	0	0	0	0	0	0	0
539839	REAIRRKLRTLILAP	NS3	1685	1699	HLA-DRB1*14:04	0%	Dengue virus 1	ELISPOT	IFNg release	Positive	0	0	0	0	0	0	0	0	0	0	0	0	0	0	0	0	0	0	0	0	0	0	0	0	0	0	0	0	0
539993	TNCLRKNGKRVIQLS	NS3	1848	1862	HLA-DRB1*13:01	0%	Dengue virus 1	ELISPOT	IFNg release	Positive	0	0	0	0	0	0	0	0	0	0	0	0	0	0	0	0	0	0	0	0	0	0	0	0	0	0	0	0	0
540071	VREAIKKKLRTLILA	NS3	1684	1698	HLA-DRB1*12:02	0%	Dengue virus 1	ELISPOT	IFNg release	Positive	0	0	0	0	0	0	0	0	0	0	0	0	0	0	0	0	0	0	0	0	0	0	0	0	0	0	0	0	0
540075	VREAIKRKLRTLVLA	NS3	1684	1698	HLA-DRB1*12:02	0%	Dengue virus 1	ELISPOT	IFNg release	Positive	0	0	0	0	0	0	0	0	0	0	0	0	0	0	0	0	0	0	0	0	0	0	0	0	0	0	0	0	0
54391	RKLTIMDLHPGSGKT	NS3	1662	1676	HLA class II	0%	Dengue virus 1	ICS	IFNg release	Positive	0	0	0	0	0	0	0	0	0	0	0	0	0	0	0	0	0	0	0	0	0	0	0	0	0	0	0	0	0
738417	AAAIFMTATPPGSAE	NS3	1785	1799	HLA-DRB1*04:07	0%	Dengue virus 1	ELISPOT	IFNg release	Positive	0	0	0	0	0	0	0	0	0	0	0	0	0	0	0	0	0	0	0	0	0	0	0	0	0	0	0	0	0
738418	AAAIFMTATPPGSME	NS3	1785	1799	HLA-DRB1*04:07	0%	Dengue virus 1	ELISPOT	IFNg release	Positive	0	0	0	0	0	0	0	0	0	0	0	0	0	0	0	0	0	0	0	0	0	0	0	0	0	0	0	0	0
195596	KTSIGLLCVTASSAL	NS4a	2174	2188	HLA-DRB1*07:01	0%	Dengue virus 1	ELISPOT	IFNg release	Positive	0	0	0	0	0	0	0	0	0	0	0	0	0	0	0	0	0	0	0	0	0	0	0	0	0	0	0	0	0
195680	LMLLALTAVLTGGVT	NS4a	2149	2163	HLA-DRB1*01:01	0%	Dengue virus 1	ELISPOT	IFNg release	Positive	0	0	0	0	0	0	0	0	0	0	0	0	0	0	0	0	0	0	0	0	0	0	0	0	0	0	0	0	0
196046	TIETLMLLALIAALT	NS4a	2145	2159	HLA-DRB1*01:01	0%	Dengue virus 1	ELISPOT	IFNg release	Positive	0	0	0	0	0	0	0	0	0	0	0	0	0	0	0	0	0	0	0	0	0	0	0	0	0	0	0	0	0
739085	GVTLFFLSGRGLGKT	NS4a	2161	2175	HLA-DRB1*01:02	0%	Dengue virus 1	ELISPOT	IFNg release	Positive	0	0	0	0	0	0	0	0	0	0	0	0	0	0	0	0	0	0	0	0	0	0	0	0	0	0	0	0	0
195308	FMLVAHYAIIGPGLQ	NS4b	2357	2371	HLA-DRB1*07:01	0%	Dengue virus 1	ELISPOT	IFNg release	Positive	0	0	0	0	0	0	0	0	0	0	0	0	0	0	0	0	0	0	0	0	0	0	0	0	0	0	0	0	0
868336	PLTLTAAVLMLAAHY	NS4b	2349	2363	HLA-DQA1*01:02/DQB1*06:02	0%	Dengue virus 1	ELISPOT	IFNg release	Positive	0	0	0	0	0	0	0	0	0	0	0	0	0	0	0	0	0	0	0	0	0	0	0	0	0	0	0	0	0
195781	NEDHWFSRGNSLSGV	NS5	2985	2999	HLA-DRB1*04:01	0%	Dengue virus 1	ELISPOT	IFNg release	Positive	0	0	0	0	0	0	0	0	0	0	0	0	0	0	0	0	0	0	0	0	0	0	0	0	0	0	0	0	0
539509	KSEFNIYKRSGIMEV	NS5	2515	2529	HLA-DRB1*15:02	0%	Dengue virus 1	ELISPOT	IFNg release	Positive	0	0	0	0	0	0	0	0	0	0	0	0	0	0	0	0	0	0	0	0	0	0	0	0	0	0	0	0	0
539537	LAKAIFKLTYQNKVV	NS5	3056	3070	HLA-DRB1*14:04	0%	Dengue virus 1	ELISPOT	IFNg release	Positive	0	0	0	0	0	0	0	0	0	0	0	0	0	0	0	0	0	0	0	0	0	0	0	0	0	0	0	0	0
195368	GIIFILLMLVTPSMA	PreM	266	280	HLA-DRB1*01:01	100%	Dengue virus 1	ELISPOT	IFNg release	Positive	1	1	1	1	1	1	1	1	1	1	1	1	1	1	1	1	1	1	1	1	1	1	1	1	1	1	1	1	1
195562	KGIIFILLMLVTPSM	PreM	265	279	HLA-DRB1*08:02	100%	Dengue virus 1	ELISPOT	IFNg release	Positive	1	1	1	1	1	1	1	1	1	1	1	1	1	1	1	1	1	1	1	1	1	1	1	1	1	1	1	1	1
195477	IELLKTEVTNPAVLR	E	38	52	HLA-DRB1*04:01	100%	Dengue virus 1	ELISPOT	IFNg release	Positive	1	1	1	1	1	1	1	1	1	1	1	1	1	1	1	1	1	1	1	1	1	1	1	1	1	1	1	1	1
540052	VLGSQEGAMHTALTGA	E	247	262	HLA class II	100%	Dengue virus 1	ICS	IFNg release	Positive	1	1	1	1	1	1	1	1	1	1	1	1	1	1	1	1	1	1	1	1	1	1	1	1	1	1	1	1	1
540104	VVVLGSQEGAMHTALTG	E	245	261	HLA class II	100%	Dengue virus 1	ICS	IFNg release	Positive	1	1	1	1	1	1	1	1	1	1	1	1	1	1	1	1	1	1	1	1	1	1	1	1	1	1	1	1	1
95154	SSIGKMFEATARG	E	396	408	HLA-DQ8	100%	Dengue virus 1	ELISPOT	IFNg release	Positive	1	1	1	1	1	1	1	1	1	1	1	1	1	1	1	1	1	1	1	1	1	1	1	1	1	1	1	1	1
866284	DDQRAWNIWEVEDYG	NS1	181	195	HLA-DQA1*01:01/DQB1*05:01	100%	Dengue virus 1	ELISPOT	IFNg release	Positive	1	1	1	1	1	1	1	1	1	1	1	1	1	1	1	1	1	1	1	1	1	1	1	1	1	1	1	1	1
867041	HADMGYWIESEKNET	NS1	231	245	HLA-DQA1*01:01/DQB1*05:01	100%	Dengue virus 1	ELISPOT	IFNg release	Positive	1	1	1	1	1	1	1	1	1	1	1	1	1	1	1	1	1	1	1	1	1	1	1	1	1	1	1	1	1
868574	QRAWNIWEVEDYGFG	NS1	183	197	HLA-DQA1*01:01/DQB1*05:01	100%	Dengue virus 1	ELISPOT	IFNg release	Positive	1	1	1	1	1	1	1	1	1	1	1	1	1	1	1	1	1	1	1	1	1	1	1	1	1	1	1	1	1
195983	SREVLLLTIGLSLVA	NS2a	1225	1239	HLA-DRB1*15:01	100%	Dengue virus 1	ELISPOT	IFNg release	Positive	1	1	1	1	1	1	1	1	1	1	1	1	1	1	1	1	1	1	1	1	1	1	1	1	1	1	1	1	1
539888	RWSRKMLMTGTLAVF	NS2a	1153	1167	HLA-DRB1*04:03	100%	Dengue virus 1	ELISPOT	IFNg release	Positive	1	1	1	1	1	1	1	1	1	1	1	1	1	1	1	1	1	1	1	1	1	1	1	1	1	1	1	1	1
195338	FTMRLLSPVRVPNYN	NS3	1741	1755	HLA-DRB1*01:01	100%	Dengue virus 1	ELISPOT	IFNg release	Positive	1	1	1	1	1	1	1	1	1	1	1	1	1	1	1	1	1	1	1	1	1	1	1	1	1	1	1	1	1
196130	VILKDGPERVILAGP	NS3	1908	1922	HLA-DRB1*03:01	100%	Dengue virus 1	ELISPOT	IFNg release	Positive	1	1	1	1	1	1	1	1	1	1	1	1	1	1	1	1	1	1	1	1	1	1	1	1	1	1	1	1	1
539579	LKGMPIRYQTTAVKS	NS3	1711	1725	HLA-DRB1*04:03	100%	Dengue virus 1	ELISPOT	IFNg release	Positive	1	1	1	1	1	1	1	1	1	1	1	1	1	1	1	1	1	1	1	1	1	1	1	1	1	1	1	1	1
867396	KGMPIRYQTTAVKSE	NS3	1712	1726	HLA-DRB3*02:02	100%	Dengue virus 1	ELISPOT	IFNg release	Positive	1	1	1	1	1	1	1	1	1	1	1	1	1	1	1	1	1	1	1	1	1	1	1	1	1	1	1	1	1
868343	PNYNMIIMDEAHFTD	NS3	1752	1766	HLA-DQA1*05:01/DQB1*02:01	100%	Dengue virus 1	ELISPOT	IFNg release	Positive	1	1	1	1	1	1	1	1	1	1	1	1	1	1	1	1	1	1	1	1	1	1	1	1	1	1	1	1	1
195478	IETLMLLALIAVLTG	NS4a	2146	2160	HLA-DRB1*15:01	100%	Dengue virus 1	ELISPOT	IFNg release	Positive	1	1	1	1	1	1	1	1	1	1	1	1	1	1	1	1	1	1	1	1	1	1	1	1	1	1	1	1	1
195679	LMLLALIAVLTGGVT	NS4a	2149	2163	HLA-DRB1*08:02	100%	Dengue virus 1	ELISPOT	IFNg release	Positive	1	1	1	1	1	1	1	1	1	1	1	1	1	1	1	1	1	1	1	1	1	1	1	1	1	1	1	1	1
868132	MLLALIAVLTGGVTL	NS4a	2150	2164	HLA-DPB1*04:02	100%	Dengue virus 1	ELISPOT	IFNg release	Positive	1	1	1	1	1	1	1	1	1	1	1	1	1	1	1	1	1	1	1	1	1	1	1	1	1	1	1	1	1
195114	AGAGLAFSLMKSLGG	NS4b	2476	2490	HLA-DRB1*04:01	100%	Dengue virus 1	ELISPOT	IFNg release	Positive	1	1	1	1	1	1	1	1	1	1	1	1	1	1	1	1	1	1	1	1	1	1	1	1	1	1	1	1	1
195193	AVLMLVAHYAIIGPG	NS4b	2355	2369	HLA-DRB1*01:01	100%	Dengue virus 1	ELISPOT	IFNg release	Positive	1	1	1	1	1	1	1	1	1	1	1	1	1	1	1	1	1	1	1	1	1	1	1	1	1	1	1	1	1
195476	IDLDPVVYDAKFEKQ	NS4b	2398	2412	HLA-DRB1*03:01	100%	Dengue virus 1	ELISPOT	IFNg release	Positive	1	1	1	1	1	1	1	1	1	1	1	1	1	1	1	1	1	1	1	1	1	1	1	1	1	1	1	1	1
195620	LCTSQILLMRTTWAL	NS4b	2421	2435	HLA-DRB1*04:01	100%	Dengue virus 1	ELISPOT	IFNg release	Positive	1	1	1	1	1	1	1	1	1	1	1	1	1	1	1	1	1	1	1	1	1	1	1	1	1	1	1	1	1
195637	LGQIMLLILCTSQIL	NS4b	2413	2427	HLA-DRB1*08:02	100%	Dengue virus 1	ELISPOT	IFNg release	Positive	1	1	1	1	1	1	1	1	1	1	1	1	1	1	1	1	1	1	1	1	1	1	1	1	1	1	1	1	1
195682	LMLVAHYAIIGPGLQ	NS4b	2357	2371	HLA-DRB1*07:01	100%	Dengue virus 1	ELISPOT	IFNg release	Positive	1	1	1	1	1	1	1	1	1	1	1	1	1	1	1	1	1	1	1	1	1	1	1	1	1	1	1	1	1
195760	MLLILCTSQILLMRT	NS4b	2417	2431	HLA-DRB1*07:01	100%	Dengue virus 1	ELISPOT	IFNg release	Positive	1	1	1	1	1	1	1	1	1	1	1	1	1	1	1	1	1	1	1	1	1	1	1	1	1	1	1	1	1
195848	QIMLLILCTSQILLM	NS4b	2415	2429	HLA-DRB1*07:01	100%	Dengue virus 1	ELISPOT	IFNg release	Positive	1	1	1	1	1	1	1	1	1	1	1	1	1	1	1	1	1	1	1	1	1	1	1	1	1	1	1	1	1
196143	VLMLVAHYAIIGPGL	NS4b	2356	2370	HLA-DRB1*01:01	100%	Dengue virus 1	ELISPOT	IFNg release	Positive	1	1	1	1	1	1	1	1	1	1	1	1	1	1	1	1	1	1	1	1	1	1	1	1	1	1	1	1	1
1066544	ACLGKSYAQMWQLMY	NS5	3244	3258	HLA-DRB1*04:01	100%	Dengue virus 1	ELISPOT	IFNg release	Positive-Low	1	1	1	1	1	1	1	1	1	1	1	1	1	1	1	1	1	1	1	1	1	1	1	1	1	1	1	1	1
195168	AQMWQLMYFHRRDLR	NS5	3251	3265	HLA-DRB1*13:01	100%	Dengue virus 1	ELISPOT	IFNg release	Positive	1	1	1	1	1	1	1	1	1	1	1	1	1	1	1	1	1	1	1	1	1	1	1	1	1	1	1	1	1
195208	CVYNMMGKREKKLGE	NS5	2942	2956	HLA-DRB1*11:01	100%	Dengue virus 1	ELISPOT	IFNg release	Positive	1	1	1	1	1	1	1	1	1	1	1	1	1	1	1	1	1	1	1	1	1	1	1	1	1	1	1	1	1
195212	DDRFATALTALNDMG	NS5	3163	3177	HLA-DRB1*04:01	100%	Dengue virus 1	ELISPOT	IFNg release	Positive	1	1	1	1	1	1	1	1	1	1	1	1	1	1	1	1	1	1	1	1	1	1	1	1	1	1	1	1	1
195360	GGMLVRNPLSRNSTH	NS5	2694	2708	HLA-DRB1*08:02	100%	Dengue virus 1	ELISPOT	IFNg release	Positive	1	1	1	1	1	1	1	1	1	1	1	1	1	1	1	1	1	1	1	1	1	1	1	1	1	1	1	1	1
195956	SIFKLTYQNKVVRVQ	NS5	3059	3073	HLA-DRB1*09:01	100%	Dengue virus 1	ELISPOT	IFNg release	Positive	1	1	1	1	1	1	1	1	1	1	1	1	1	1	1	1	1	1	1	1	1	1	1	1	1	1	1	1	1
196031	TCVYNMMGKREKKLG	NS5	2941	2955	HLA-DRB1*11:01	100%	Dengue virus 1	ELISPOT	IFNg release	Positive	1	1	1	1	1	1	1	1	1	1	1	1	1	1	1	1	1	1	1	1	1	1	1	1	1	1	1	1	1
196039	TGNIVSAVNMTSRML	NS5	2717	2731	HLA-DRB1*04:01	100%	Dengue virus 1	ELISPOT	IFNg release	Positive	1	1	1	1	1	1	1	1	1	1	1	1	1	1	1	1	1	1	1	1	1	1	1	1	1	1	1	1	1
539107	AVNMTSRMLLNRFTM	NS5	2723	2737	HLA-DRB1*15:02	100%	Dengue virus 1	ELISPOT	IFNg release	Positive	1	1	1	1	1	1	1	1	1	1	1	1	1	1	1	1	1	1	1	1	1	1	1	1	1	1	1	1	1
540138	WQLMYFHRRDLRLAA	NS5	3254	3268	HLA-DRB1*14:04	100%	Dengue virus 1	ELISPOT	IFNg release	Positive	1	1	1	1	1	1	1	1	1	1	1	1	1	1	1	1	1	1	1	1	1	1	1	1	1	1	1	1	1
867505	KSYAQMWQLMYFHRR	NS5	3248	3262	HLA-DPB1*01:01	100%	Dengue virus 1	ELISPOT	IFNg release	Positive	1	1	1	1	1	1	1	1	1	1	1	1	1	1	1	1	1	1	1	1	1	1	1	1	1	1	1	1	1
195113	AFLRFLAIPPTAGIL	C	30	44	HLA-DRB1*01:01	81% - <100%	Dengue virus 1	ELISPOT	IFNg release	Positive	1	0	1	1	1	1	1	1	1	1	1	1	1	1	1	1	1	1	1	1	1	1	1	1	1	1	1	1	1
195117	AGILARWSSFKKNGA	C	51	65	HLA-DRB1*15:01	81% - <100%	Dengue virus 1	ELISPOT	IFNg release	Positive	1	1	1	1	1	0	1	1	1	1	1	1	1	1	0	0	1	1	1	1	1	1	1	1	1	1	1	1	1
195299	FIAFLRFLAIPPTAG	C	28	42	HLA-DRB1*01:01	81% - <100%	Dengue virus 1	ELISPOT	IFNg release	Positive	1	0	1	1	1	1	1	1	1	1	1	1	1	1	1	0	1	1	1	1	1	1	1	1	1	1	1	1	1
195314	FNMLKRARNRVSTGS	C	1	15	HLA-DRB1*08:02	81% - <100%	Dengue virus 1	ELISPOT	IFNg release	Positive	1	0	1	1	1	0	1	1	1	1	1	1	1	1	0	1	1	1	1	1	1	1	1	1	1	1	1	1	0
195468	IAFLRFLAIPPTAGI	C	29	43	HLA-DRB1*01:01	81% - <100%	Dengue virus 1	ELISPOT	IFNg release	Positive	1	0	1	1	1	1	1	1	1	1	1	1	1	1	1	1	1	1	1	1	1	1	1	1	1	1	1	1	1
195586	KRSVTMLLMLLPTAL	C	87	101	HLA-DRB1*01:01	81% - <100%	Dengue virus 1	ELISPOT	IFNg release	Positive	1	1	1	1	1	0	1	1	1	1	1	1	1	1	0	0	1	1	1	1	1	1	1	1	1	1	1	1	1
195681	LMLLPTALAFHLTTR	C	94	108	HLA-DRB1*04:01	81% - <100%	Dengue virus 1	ELISPOT	IFNg release	Positive	1	1	1	1	1	0	1	1	1	1	1	1	1	1	0	0	1	1	1	1	1	1	1	1	1	1	1	1	1
195761	MLNIMNRRKRSVTML	C	69	83	HLA-DRB1*08:02	81% - <100%	Dengue virus 1	ELISPOT	IFNg release	Positive	1	1	1	1	1	0	1	1	1	1	1	1	1	1	0	1	1	1	1	1	1	1	1	1	1	1	1	1	1

195904	RPSFNMLKRARNRVS	C	10	24	HLA-DRB1*11:01	81% - <100%	Dengue virus 1	ELISPOT	IFNg release	Positive	1	0	1	1	1	1	1	1	1	1	1	1	1	1	1	1	1	1	1	1	1	1	1	1	1	1	1	1	0
195944	SFNMLKRARNRVSTG	C	12	26	HLA-DRB1*11:01	81% - <100%	Dengue virus 1	ELISPOT	IFNg release	Positive	1	0	1	1	1	0	1	1	1	1	1	1	1	1	0	1	1	1	1	1	1	1	1	1	1	1	1	1	0
196043	TGRPSFNMLKRARNR	C	8	22	HLA-DRB1*11:01	81% - <100%	Dengue virus 1	ELISPOT	IFNg release	Positive	1	0	1	1	1	0	1	1	1	1	1	1	1	1	0	1	1	1	1	1	1	1	1	1	1	1	1	1	0
196151	VMAFIAFLRFLAIPP	C	25	39	HLA-DRB1*11:01	81% - <100%	Dengue virus 1	ELISPOT	IFNg release	Positive	1	0	1	1	1	1	1	1	1	1	1	1	1	1	1	0	1	1	1	1	1	1	1	1	1	1	1	1	1
196196	VTMLLMLLPTALAFH	C	90	104	HLA-DRB1*04:01	81% - <100%	Dengue virus 1	ELISPOT	IFNg release	Positive	1	1	1	1	1	0	1	1	1	1	1	1	1	1	0	0	1	1	1	1	1	1	1	1	1	1	1	1	1
539043	AFIAFLRFLAIPPTA	C	27	41	HLA-DRB1*12:02	81% - <100%	Dengue virus 1	ELISPOT	IFNg release	Positive	1	0	1	1	1	1	1	1	1	1	1	1	1	1	1	0	1	1	1	1	1	1	1	1	1	1	1	1	1
539259	FKKNGAIKVLRGFKK	C	50	64	HLA-DRB1*08:03	81% - <100%	Dengue virus 1	ELISPOT	IFNg release	Positive	1	1	1	1	1	1	1	1	1	1	1	1	1	1	1	0	1	1	1	1	1	1	0	1	1	1	1	1	1
539495	KNGAIKVLRGFKKEI	C	52	66	HLA-DRB1*12:02	81% - <100%	Dengue virus 1	ELISPOT	IFNg release	Positive	1	1	1	1	1	1	1	1	1	1	1	1	1	1	1	0	1	1	1	1	1	1	0	1	1	1	1	1	1
539613	LRFLAIPPTAGILAR	C	32	46	HLA-DRB1*04:03	81% - <100%	Dengue virus 1	ELISPOT	IFNg release	Positive	1	0	1	1	1	1	1	1	1	1	1	1	1	1	1	0	1	1	1	1	1	1	1	1	1	1	1	1	1
539711	NGAIKVLRGFKKEIS	C	53	67	HLA-DRB1*13:01	81% - <100%	Dengue virus 1	ELISPOT	IFNg release	Positive	1	1	1	1	1	1	1	1	1	1	1	1	1	1	1	0	1	1	1	1	1	1	0	1	1	1	1	1	1
539785	PSFNMLKRARNRVST	C	11	25	HLA-DRB1*10:01	81% - <100%	Dengue virus 1	ELISPOT	IFNg release	Positive	1	0	1	1	1	1	1	1	1	1	1	1	1	1	1	1	1	1	1	1	1	1	1	1	1	1	1	1	0
738927	FLRFLAIPPTAGILA	C	41	55	HLA-DRB1*14:02	81% - <100%	Dengue virus 1	ELISPOT	IFNg release	Positive	1	0	1	1	1	1	1	1	1	1	1	1	1	1	1	0	1	1	1	1	1	1	1	1	1	1	1	1	1
195887	RGKSLLFKTSAGVNM	PreM	111	125	HLA-DRB1*07:01	81% - <100%	Dengue virus 1	ELISPOT	IFNg release	Positive	1	1	1	1	1	1	1	1	1	1	1	1	1	1	1	0	1	1	0	1	1	1	1	1	1	1	1	1	1
539516	KSLLFKTSAGVNMCT	PreM	123	137	HLA-DRB1*09:01	81% - <100%	Dengue virus 1	ELISPOT	IFNg release	Positive	1	1	1	1	1	1	1	1	1	1	1	1	1	1	1	0	1	1	0	1	1	1	1	1	1	1	1	1	1
867912	LRHPGFTVIALFLAH	PreM	242	256	HLA-DPB1*02:01	81% - <100%	Dengue virus 1	ELISPOT	IFNg release	Positive	1	1	1	1	1	1	1	1	1	1	1	1	1	1	1	0	1	1	1	1	1	1	1	1	1	1	1	1	1
195145	AKFKCVTKLEGKIVQ	E	117	131	HLA-DRB1*11:01	81% - <100%	Dengue virus 1	ELISPOT	IFNg release	Positive	1	1	1	1	1	1	1	1	1	1	1	1	1	1	1	1	1	1	0	1	1	1	1	1	1	1	1	1	1
195216	DIELLKTEVTNPAVL	E	37	51	HLA-DRB1*04:01	81% - <100%	Dengue virus 1	ELISPOT	IFNg release	Positive	1	1	1	1	1	1	1	1	1	1	1	1	1	1	1	0	1	1	1	1	1	1	1	1	1	1	1	1	1
195260	ENLKYSVIVTVHTGD	E	128	142	HLA-DRB1*07:01	81% - <100%	Dengue virus 1	ELISPOT	IFNg release	Positive	1	1	1	1	1	1	1	1	1	1	1	1	1	1	0	1	1	1	1	1	1	1	1	1	1	1	1	1	1
195362	GGVFTSVGKLVHQVF	E	426	440	HLA-DRB1*11:01	81% - <100%	Dengue virus 1	ELISPOT	IFNg release	Positive	1	1	0	1	1	0	1	1	1	1	1	1	1	1	0	0	1	1	0	1	1	1	1	1	1	1	1	1	1
195547	IVQYENLKYSVIVTV	E	124	138	HLA-DRB1*15:02	81% - <100%	Dengue virus 1	ELISPOT	IFNg release	Positive	1	1	1	1	1	1	1	1	1	1	1	1	1	1	1	1	1	1	0	1	1	1	1	1	1	1	1	1	1
195623	LDIELLKTEVTNPAV	E	36	50	HLA-DRB1*04:01	81% - <100%	Dengue virus 1	ELISPOT	IFNg release	Positive	1	1	1	1	1	1	1	1	1	1	1	1	1	1	1	0	1	1	1	1	1	1	1	1	1	1	1	1	1
195646	LITANPIVTDKEKPV	E	346	360	HLA-DRB1*03:01	81% - <100%	Dengue virus 1	ELISPOT	IFNg release	Positive	1	0	1	1	1	1	0	1	1	0	1	1	1	1	1	1	0	1	1	1	1	1	1	1	1	1	1	1	0
195814	NRQDLLVTFKTAHAK	E	227	241	HLA-DRB1*04:01	81% - <100%	Dengue virus 1	ELISPOT	IFNg release	Positive	1	1	0	1	1	1	1	1	1	1	1	1	1	1	0	1	1	1	1	1	1	1	1	1	1	1	1	1	1
195874	QYENLKYSVIVTVHT	E	126	140	HLA-DRB1*07:01	81% - <100%	Dengue virus 1	ELISPOT	IFNg release	Positive	1	1	1	1	1	1	1	1	1	1	1	1	1	1	0	1	1	1	1	1	1	1	1	1	1	1	1	1	1
196053	TLDIELLKTEVTNPA	E	35	49	HLA-DRB1*04:01	81% - <100%	Dengue virus 1	ELISPOT	IFNg release	Positive	1	1	1	1	1	1	1	1	1	1	1	1	1	1	1	0	1	1	1	1	1	1	1	1	1	1	1	1	1
539169	DLLVTFKTAHAKKQE	E	230	244	HLA-DRB1*14:04	81% - <100%	Dengue virus 1	ELISPOT	IFNg release	Positive	1	0	0	1	1	1	1	1	1	1	1	1	1	1	0	1	1	1	1	1	1	1	1	1	1	1	1	1	1
539378	HAKKQEVVVLGSQEGAMH	E	239	256	HLA class II	81% - <100%	Dengue virus 1	ICS	IFNg release	Positive	1	0	0	1	1	1	1	1	1	1	1	1	1	1	1	1	1	1	1	1	1	1	1	1	1	1	1	1	1
539593	LLVTFKTAHAKKQEV	E	236	250	HLA-DRB1*14:02	81% - <100%	Dengue virus 1	ELISPOT	IFNg release	Positive	1	0	0	1	1	1	1	1	1	1	1	1	1	1	1	1	1	1	1	1	1	1	1	1	1	1	1	1	1
539800	QDLLVTFKTAHAKKQ	E	229	243	HLA-DRB1*09:01	81% - <100%	Dengue virus 1	ELISPOT	IFNg release	Positive	1	0	0	1	1	1	1	1	1	1	1	1	1	1	0	1	1	1	1	1	1	1	1	1	1	1	1	1	1
539869	RQDLLVTFKTAHAKK	E	228	242	HLA-DRB1*08:03	81% - <100%	Dengue virus 1	ELISPOT	IFNg release	Positive	1	1	0	1	1	1	1	1	1	1	1	1	1	1	0	1	1	1	1	1	1	1	1	1	1	1	1	1	1
741745	MRCVGIGNRDFVEGLSGATW	E	281	300	HLA class II	81% - <100%	Dengue virus 1	ELISPOT	IFNg release	Positive	1	1	1	1	1	0	1	1	1	1	1	1	1	1	0	1	1	1	1	1	1	1	1	1	1	1	1	1	1
868013	LVHKQWFLDLPLPWT	E	487	501	HLA-DQA1*01:01/DQB1*05:01	81% - <100%	Dengue virus 1	ELISPOT	IFNg release	Positive	1	1	1	1	1	1	1	1	1	1	1	1	1	1	1	0	1	1	1	1	1	1	1	1	1	1	1	1	1
869691	WLVHKQWFLDLPLPW	E	486	500	HLA-DQA1*01:01/DQB1*05:01	81% - <100%	Dengue virus 1	ELISPOT	IFNg release	Positive	1	1	1	1	1	1	1	1	1	1	1	1	1	1	1	0	1	1	1	1	1	1	1	1	1	1	1	1	1
132583	QYKFQADSPKRLSAA	NS1	31	45	HLA-DRB1*03:01	81% - <100%	Dengue virus 1	ELISPOT	IFNg release	Positive	1	1	1	1	1	1	1	1	1	1	1	1	1	1	1	0	1	1	1	1	1	1	1	1	1	1	1	1	1
195579	KNETWKLARASFIEV	NS1	206	220	HLA-DRB1*07:01	81% - <100%	Dengue virus 1	ELISPOT	IFNg release	Positive	1	1	0	1	1	1	1	1	0	1	1	1	1	1	1	0	1	1	0	1	1	1	1	1	1	1	1	1	1
195685	LNHILLENDMKFTVV	NS1	75	89	HLA-DRB1*03:01	81% - <100%	Dengue virus 1	ELISPOT	IFNg release	Positive	1	1	1	1	1	1	1	1	1	1	1	1	1	1	0	1	1	1	1	1	1	1	1	1	1	1	1	1	1
195784	NETWKLARASFIEVK	NS1	207	221	HLA-DRB1*07:01	81% - <100%	Dengue virus 1	ELISPOT	IFNg release	Positive	1	1	0	1	1	1	1	1	0	1	1	1	1	1	1	0	1	1	0	1	1	1	1	1	1	1	1	1	1
539401	IESEKNETWKLARASFI	NS1	202	218	HLA class II	81% - <100%	Dengue virus 1	ICS	IFNg release	Positive	1	1	0	1	1	1	1	1	0	1	1	1	1	1	1	0	1	1	1	1	1	1	1	1	1	1	1	1	1
869135	TEQYKFQADSPKRLS	NS1	65	79	HLA-DRB5*01:01	81% - <100%	Dengue virus 1	ELISPOT	IFNg release	Positive	1	1	1	1	1	1	1	1	1	1	1	1	1	1	1	0	1	1	1	1	1	1	1	1	1	1	1	1	1
195286	FAVGLLFRRLTSREV	NS2a	1214	1228	HLA-DRB1*08:03	81% - <100%	Dengue virus 1	ELISPOT	IFNg release	Positive	1	1	1	1	1	1	1	1	1	1	1	1	1	1	0	1	0	1	1	1	1	1	1	1	1	1	1	1	1
195391	GLLFRRLTSREVLLL	NS2a	1217	1231	HLA-DRB1*08:03	81% - <100%	Dengue virus 1	ELISPOT	IFNg release	Positive	1	1	1	1	1	1	1	1	1	1	1	1	1	1	1	1	0	1	1	1	1	1	1	1	1	1	1	1	1
195665	LLLTIGLSLVASVEL	NS2a	1229	1243	HLA-DRB1*07:01	81% - <100%	Dengue virus 1	ELISPOT	IFNg release	Positive	1	1	1	1	1	1	1	1	1	1	1	1	1	1	1	1	1	1	0	0	0	0	1	1	1	1	1	1	1
195674	LLTIGLSLVASVELP	NS2a	1230	1244	HLA-DRB1*07:01	81% - <100%	Dengue virus 1	ELISPOT	IFNg release	Positive	1	1	1	1	1	1	1	1	1	1	1	1	1	1	1	1	1	1	0	0	0	0	1	1	1	1	1	1	1
195708	LSIVSLFPLCLSTTS	NS2a	1299	1313	HLA-DRB1*01:01	81% - <100%	Dengue virus 1	ELISPOT	IFNg release	Positive	0	1	1	1	1	1	1	1	1	1	1	0	1	1	1	0	1	1	1	1	1	1	1	1	1	1	1	1	1
539094	ATFKMRPMFAVGLLF	NS2a	1206	1220	HLA-DRB1*01:02	81% - <100%	Dengue virus 1	ELISPOT	IFNg release	Positive	1	1	1	1	1	1	1	1	1	1	1	1	1	1	0	1	0	1	1	1	1	1	1	1	1	1	1	1	1
539205	EEVMRSRWSRKMLMT	NS2a	1147	1161	HLA-DRB1*13:01	81% - <100%	Dengue virus 1	ELISPOT	IFNg release	Positive	1	1	1	1	1	1	1	1	1	1	1	1	1	1	1	1	1	1	1	1	0	0	1	1	1	1	1	1	1
539398	IEEVMRSRWSRKMLM	NS2a	1146	1160	HLA-DRB1*13:01	81% - <100%	Dengue virus 1	ELISPOT	IFNg release	Positive	1	1	1	1	1	1	1	1	1	1	1	1	1	1	1	1	1	1	1	1	0	0	1	1	1	1	1	1	1
540038	VGLLFRRLTSREVLL	NS2a	1216	1230	HLA-DRB1*08:03	81% - <100%	Dengue virus 1	ELISPOT	IFNg release	Positive	1	1	1	1	1	1	1	1	1	1	1	1	1	1	1	1	0	1	1	1	1	1	1	1	1	1	1	1	1
540155	YLALMATFKMRPMFA	NS2a	1201	1215	HLA-DRB1*13:01	81% - <100%	Dengue virus 1	ELISPOT	IFNg release	Positive	1	1	1	1	1	1	1	1	1	1	1	1	1	1	0	1	0	1	1	1	1	1	1	1	1	1	1	1	1
738553	AVGLLFRRLTSREVL	NS2a	1215	1229	HLA-DRB1*01:02	81% - <100%	Dengue virus 1	ELISPOT	IFNg release	Positive	1	1	1	1	1	1	1	1	1	1	1	1	1	1	0	1	0	1	1	1	1	1	1	1	1	1	1	1	1
868098	MGMGTTYLALMATFK	NS2a	456	470	HLA-DRB5*01:01	81% - <100%	Dengue virus 1	ELISPOT	IFNg release	Positive	1	1	0	1	1	1	1	1	1	1	1	1	1	1	0	1	1	1	1	1	1	1	1	1	1	1	1	1	1
868100	MGQLTWNDLIRLCIM	NS2a	433	447	HLA-DPB1*04:02	81% - <100%	Dengue virus 1	ELISPOT	IFNg release	Positive	1	1	1	1	1	0	1	1	1	1	1	1	1	1	0	1	1	1	1	1	1	1	1	1	1	1	1	1	1
195191	AVGIVSILLSSLLKN	NS2b	1355	1369	HLA-DRB1*08:02	81% - <100%	Dengue virus 1	ELISPOT	IFNg release	Positive	1	1	0	1	1	1	1	1	1	1	1	1	1	1	1	0	1	1	1	1	1	1	1	1	1	1	1	1	1
195228	DTLTILLKATLLAVS	NS2b	1438	1452	HLA-DRB1*08:02	81% - <100%	Dengue virus 1	ELISPOT	IFNg release	Positive	1	1	0	1	1	1	1	1	1	1	1	1	1	1	1	1	1	1	1	1	0	0	1	1	1	1	1	1	1
195508	ILVEVQDDGTMKIKD	NS2b	68	82	HLA-DRB1*03:01	81% - <100%	Dengue virus 1	ELISPOT	IFNg release	Positive	1	1	0	1	1	1	1	1	1	1	1	1	1	1	1	1	1	1	1	1	1	1	1	1	1	1	1	1	1
195510	IMAVGIVSILLSSLL	NS2b	1353	1367	HLA-DRB1*07:01	81% - <100%	Dengue virus 1	ELISPOT	IFNg release	Positive	1	1	0	1	1	1	1	1	1	1	1	1	1	1	1	1	1	1	1	1	1	1	1	1	1	1	1	1	1
195715	LSSLLKNDVPLAGPL	NS2b	1363	1377	HLA-DRB1*03:01	81% - <100%	Dengue virus 1	ELISPOT	IFNg release	Positive	1	1	0	1	1	1	1	1	1	1	1	1	1	1	1	0	1	1	1	1	1	1	1	1	1	1	1	1	1
195724	LTILLKATLLAVSGV	NS2b	1440	1454	HLA-DRB1*08:02	81% - <100%	Dengue virus 1	ELISPOT	IFNg release	Positive	1	1	1	1	1	0	1	1	1	1	0	1	1	1	1	0	1	1	1	1	0	0	1	1	1	1	1	1	1
195878	RDDTLTILLKATLLA	NS2b	1436	1450	HLA-DRB1*12:02	81% - <100%	Dengue virus 1	ELISPOT	IFNg release	Positive	1	1	0	1	1	1	1	1	1	1	1	1	1	1	1	1	1	1	1	1	1	1	1	1	1	1	1	1	1
195993	SSLLKNDVPLAGPLI	NS2b	1364	1378	HLA-DRB1*03:01	81% - <100%	Dengue virus 1	ELISPOT	IFNg release	Positive	1	1	0	1	1	1	1	1	1	1	1	1	1	1	1	0	1	1	1	1	1	1	1	1	1	1	1	1	1
196111	VEVQDDGTMKIKDEE	NS2b	1421	1435	HLA-DRB1*03:01	81% - <100%	Dengue virus 1	ELISPOT	IFNg release	Positive	1	1	0	1	1	1	1	1	1	1	1	1	1	1	1	1	1	1	1	1	1	1	1	1	1	1	1	1	1
866201	ATLFVWYFWQKKKQR	NS2b	1461	1475	HLA-DPB1*01:01	81% - <100%	Dengue virus 1	ELISPOT	IFNg release	Positive	1	1	1	0	1	1	1	1	1	1	1	1	1	1	1	0	1	1	1	1	1	1	1	1	1	1	1	1	1
867359	IVSILLSSLLKNDVP	NS2b	1358	1372	HLA-DPB1*04:02	81% - <100%	Dengue virus 1	ELISPOT	IFNg release	Positive	1	1	0	1	1	1	1	1	1	1	1	1	1	1	1	0	1	1	1	1	1	1	1	1	1	1	1	1	1
867544	LAGPLIAGGMLIACY	NS2b	1373	1387	HLA-DQA1*01:02/DQB1*06:02	81% - <100%	Dengue virus 1	ELISPOT	IFNg release	Positive	1	1	1	1	1	1	1	1	1	1	1	1	1	1	1	0	1	1	1	1	1	1	1	1	1	1	1	1	1
167826	TSGTYVSAIAQAKASQE	NS3	157	173	HLA class II	81% - <100%	Dengue virus 1	ELISPOT	IFNg release	Positive	1	0	1	1	1	1	1	1	1	1	1	1	1	1	0	1	1	1	1	1	1	1	1	1	1	1	1	1	1
195143	AIVREAIKRKLRTLI	NS3	1682	1696	HLA-DRB1*11:01	81% - <100%	Dengue virus 1	ELISPOT	IFNg release	Positive	1	1	1	1	1	0	1	1	1	1	0	1	1	1	0	0	1	1	1	1	1	1	1	1	1	0	1	1	1
195163	ANCLRKNGKRVIQLS	NS3	1848	1862	HLA-DRB1*13:01	81% - <100%	Dengue virus 1	ELISPOT	IFNg release	Positive	1	1	1	1	1	1	1	1	1	1	1	1	1	1	1	0	1	1	1	1	1	1	1	1	1	1	1	1	1
195165	ANFRADRVIDPRRCL	NS3	1891	1905	HLA-DRB1*03:01	81% - <100%	Dengue virus 1	ELISPOT	IFNg release	Positive	1	1	0	1	1	1	1	1	1	1	1	1	1	1	0	1	1	1	1	1	1	1	1	1	1	1	1	1	1
195201	CHATFTMRLLSPVRV	NS3	1737	1751	HLA-DRB1*01:01	81% - <100%	Dengue virus 1	ELISPOT	IFNg release	Positive	1	1	0	1	1	1	1	1	1	1	1	1	1	1	1	1	1	1	1	1	1	1	1	1	1	1	1	1	1
195242	EGKIVGLYGNGVVTT	NS3	1618	1632	HLA-DRB1*15:01	81% - <100%	Dengue virus 1	ELISPOT	IFNg release	Positive	1	1	1	1	1	1	1	1	1	1	0	1	1	1	1	1	1	1	1	1	1	0	1	1	1	0	1	1	1
195296	FHTMWHVTRGAVLMY	NS3	1521	1535	HLA-DRB1*07:01	81% - <100%	Dengue virus 1	ELISPOT	IFNg release	Positive	1	1	1	1	1	1	1	1	1	1	1	1	1	1	1	0	1	1	1	1	0	1	1	1	1	1	1	1	1
195377	GIYRILQRGLLGRSQ	NS3	1496	1510	HLA-DRB1*11:01	81% - <100%	Dengue virus 1	ELISPOT	IFNg release	Positive	0	1	1	1	1	0	1	1	1	1	1	1	1	1	0	1	1	1	1	1	1	1	1	1	1	1	1	1	1
195379	GKIVGLYGNGVVTTS	NS3	1619	1633	HLA-DRB1*15:01	81% - <100%	Dengue virus 1	ELISPOT	IFNg release	Positive	1	1	1	1	1	1	1	1	1	1	0	1	1	1	1	1	1	1	1	1	1	0	1	1	1	0	1	1	1
195549	IYRILQRGLLGRSQV	NS3	1497	1511	HLA-DRB1*15:01	81% - <100%	Dengue virus 1	ELISPOT	IFNg release	Positive	0	1	1	1	1	0	1	1	1	1	1	1	1	1	0	1	1	1	1	1	1	1	1	1	1	1	1	1	1
195585	KRKLRTLILAPTRVV	NS3	1689	1703	HLA-DRB1*10:01	81% - <100%	Dengue virus 1	ELISPOT	IFNg release	Positive	1	1	1	1	1	0	1	1	1	1	1	1	1	1	0	0	1	1	1	1	1	1	1	1	1	1	1	1	1
195705	LRTLILAPTRVVASE	NS3	1692	1706	HLA-DRB1*08:02	81% - <100%	Dengue virus 1	ELISPOT	IFNg release	Positive	1	1	1	1	1	0	1	1	1	1	1	1	1	1	0	0	1	1	1	1	1	1	1	1	1	1	1	1	1
195777	NCLRKNGKRVIQLSR	NS3	1849	1863	HLA-DRB1*11:01	81% - <100%	Dengue virus 1	ELISPOT	IFNg release	Positive	1	1	1	1	1	1	1	1	1	1	1	1	1	1	1	0	1	1	1	1	1	1	1	1	1	1	1	1	1
195778	NDIANCLRKNGKRVI	NS3	1845	1859	HLA-DRB1*11:01	81% - <100%	Dengue virus 1	ELISPOT	IFNg release	Positive	1	1	1	1	1	1	1	1	1	1	1	1	1	1	1	0	1	1	1	1	1	1	1	1	1	1	1	1	1
195896	RKLRTLILAPTRVVA	NS3	1690	1704	HLA-DRB1*01:01	81% - <100%	Dengue virus 1	ELISPOT	IFNg release	Positive	1	1	1	1	1	0	1	1	1	1	1	1	1	1	0	0	1	1	1	1	1	1	1	1	1	1	1	1	1
195914	RTLILAPTRVVASEM	NS3	1693	1707	HLA-DRB1*10:01	81% - <100%	Dengue virus 1	ELISPOT	IFNg release	Positive	1	1	1	1	1	1	1	1	1	1	1	1	1	1	1	0	1	1	1	1	1	1	1	1	1	1	1	1	1
196138	VLDDGIYRILQRGLL	NS3	1492	1506	HLA-DRB1*11:01	81% - <100%	Dengue virus 1	ELISPOT	IFNg release	Positive	1	1	1	1	1	0	1	1	1	1	1	1	1	1	0	1	1	1	1	1	1	1	1	1	1	1	1	1	1
196197	VTRGAVLMYQGKRLE	NS3	1527	1541	HLA-DRB1*15:01	81% - <100%	Dengue virus 1	ELISPOT	IFNg release	Positive	1	1	1	1	1	1	1	1	1	1	1	1	1	1	1	0	1	1	1	1	1	1	1	1	1	1	1	1	1
30397	KEGERKKLRPRWLDA	NS3	585	599	HLA class II	81% - <100%	Dengue virus 1	ICS	IFNg release	Positive	1	1	1	1	1	1	1	1	1	1	1	1	1	1	1	1	1	1	1	1	1	1	1	1	0	1	1	1	1
45669	NREGKIVGLYGNGVV	NS3	141	155	HLA-DRB1*15:01	81% - <100%	Dengue virus 1	ICS	IFNg release	Positive	1	1	1	1	1	1	1	1	1	1	0	1	1	1	1	1	1	1	1	1	1	0	1	1	1	0	1	1	1
49768	PTRVVASEMAEALKG	NS3	224	238	HLA-DRB1*15:01	81% - <100%	Dengue virus 1	ICS	TNF release	Positive	1	1	1	1	1	1	1	1	1	1	1	1	1	1	1	0	1	1	1	1	1	1	1	1	1	1	1	1	1
539022	AAAIFMTATPPGSVE	NS3	1785	1799	HLA-DRB1*04:03	81% - <100%	Dengue virus 1	ELISPOT	IFNg release	Positive	1	1	1	1	1	1	1	1	0	1	1	1	1	1	1	0	1	1	1	1	1	1	1	1	1	1	1	1	1
539027	AAIFMTATPPGSVEA	NS3	1786	1800	HLA-DRB1*10:01	81% - <100%	Dengue virus 1	ELISPOT	IFNg release	Positive	1	1	1	1	1	1	1	1	1	1	1	1	1	1	1	0	1	1	1	1	1	1	1	1	1	1	1	1	1
539155	DGIYRILQRGLLGRS	NS3	1495	1509	HLA-DRB1*12:02	81% - <100%	Dengue virus 1	ELISPOT	IFNg release	Positive	0	1	1	1	1	0	1	1	1	1	1	1	1	1	0	1	1	1	1	1	1	1	1	1	1	1	1	1	1
539171	DLMCHATFTMRLLSP	NS3	1734	1748	HLA-DRB1*13:01	81% - <100%	Dengue virus 1	ELISPOT	IFNg release	Positive	1	1	0	1	1	1	1	1	1	1	1	1	1	1	1	1	1	1	1	1	1	1	1	1	1	1	1	1	1
539288	GEAAAIFMTATPPGS	NS3	1783	1797	HLA-DRB1*04:03	81% - <100%	Dengue virus 1	ELISPOT	IFNg release	Positive	1	1	1	1	1	1	1	1	0	1	1	1	1	1	1	0	1	1	1	1	1	1	1	1	1	1	1	1	1
539485	KLRTLILAPTRVVAS	NS3	1691	1705	HLA-DRB1*04:03	81% - <100%	Dengue virus 1	ELISPOT	IFNg release	Positive	1	1	1	1	1	0	1	1	1	1	1	1	1	1	0	0	1	1	1	1	1	1	1	1	1	1	1	1	1
539543	LDDGIYRILQRGLLG	NS3	1493	1507	HLA-DRB1*12:02	81% - <100%	Dengue virus 1	ELISPOT	IFNg release	Positive	1	1	1	1	1	0	1	1	1	1	1	1	1	1	0	1	1	1	1	1	1	1	1	1	1	1	1	1	1
539715	NGKRVIQLSRKTFDT	NS3	1854	1868	HLA-DRB1*12:02	81% - <100%	Dengue virus 1	ELISPOT	IFNg release	Positive	1	1	1	1	1	1	1	1	1	1	1	1	1	1	1	1	1	1	1	0	1	1	1	1	1	1	1	1	1
539902	SGTYVSAIAQAKASQ	NS3	1633	1647	HLA-DRB1*10:01	81% - <100%	Dengue virus 1	ELISPOT	IFNg release	Positive	1	0	1	1	1	1	1	1	1	1	1	1	1	1	0	1	1	1	1	1	1	1	1	1	1	1	1	1	1
539986	TLILAPTRVVASEMA	NS3	1694	1708	HLA-DRB1*13:01	81% - <100%	Dengue virus 1	ELISPOT	IFNg release	Positive	1	1	1	1	1	1	1	1	1	1	1	1	1	1	1	0	1	1	1	1	1	1	1	1	1	1	1	1	1
540040	VGLYGNGVVTTSGTY	NS3	1622	1636	HLA-DRB1*15:06	81% - <100%	Dengue virus 1	ELISPOT	IFNg release	Positive	1	1	1	1	1	1	1	1	1	1	1	1	1	1	1	1	1	1	1	1	1	0	1	1	1	1	1	1	1
540074	VREAIKRKLRTLILA	NS3	1684	1698	HLA-DRB1*12:02	81% - <100%	Dengue virus 1	ELISPOT	IFNg release	Positive	1	1	1	1	1	0	1	1	1	1	0	1	1	1	0	0	1	1	1	1	1	1	1	1	1	0	1	1	1
739269	IVGLYGNGVVTTSGT	NS3	132	146	HLA-DRB1*01:02	81% - <100%	Dengue virus 1	ELISPOT	IFNg release	Positive	1	1	1	1	1	1	1	1	1	1	0	1	1	1	1	1	1	1	1	1	1	0	1	1	1	0	1	1	1
739540	LPAIVREAIKRKLRT	NS3	1680	1694	HLA-DRB1*14:02	81% - <100%	Dengue virus 1	ELISPOT	IFNg release	Positive	1	1	1	1	1	0	1	1	1	1	0	1	1	1	0	0	1	1	1	1	1	1	1	1	1	0	1	1	1
866181	ASQEGPLPEIEDEVF	NS3	1645	1659	HLA-DQA1*05:01/DQB1*02:01	81% - <100%	Dengue virus 1	ELISPOT	IFNg release	Positive	1	1	1	1	1	1	0	1	1	1	1	1	1	1	0	1	1	1	1	1	1	1	1	1	1	1	1	1	1
866469	EAIKRKLRTLILAPT	NS3	1686	1700	HLA-DPB1*01:01	81% - <100%	Dengue virus 1	ELISPOT	IFNg release	Positive	1	1	1	1	1	0	1	1	1	1	0	1	1	1	0	0	1	1	1	1	1	1	1	1	1	0	1	1	1
868113	MIIMDEAHFTDPASI	NS3	1756	1770	HLA-DQA1*01:01/DQB1*05:01	81% - <100%	Dengue virus 1	ELISPOT	IFNg release	Positive	1	1	1	0	1	0	1	1	1	1	1	1	1	1	0	1	1	1	1	1	1	1	1	1	1	1	1	1	1
869341	TTSGTYVSAIAQAKA	NS3	1631	1645	HLA-DQA1*01:02/DQB1*06:02	81% - <100%	Dengue virus 1	ELISPOT	IFNg release	Positive	1	0	1	1	1	1	1	1	1	1	1	1	1	1	0	1	1	1	1	1	1	1	1	1	1	1	1	1	1
869688	WHVTRGAVLMYQGKR	NS3	1525	1539	HLA-DQA1*01:02/DQB1*06:02	81% - <100%	Dengue virus 1	ELISPOT	IFNg release	Positive	1	1	1	1	1	1	1	1	1	1	1	1	1	1	1	0	1	1	1	1	1	1	1	1	1	1	1	1	1
95169	YRILQRGLLGRSQ	NS3	23	35	HLA-DR1	81% - <100%	Dengue virus 1	ELISPOT	IFNg release	Positive	0	1	1	1	1	0	1	1	1	1	1	1	1	1	0	1	1	1	1	1	1	1	1	1	1	1	1	1	1
196214	WIAASIILEFFLMVL	NS4a	2198	2212	HLA-DRB1*15:01	81% - <100%	Dengue virus 1	ELISPOT	IFNg release	Positive	1	1	0	1	1	1	1	1	1	1	1	1	1	1	1	0	1	1	1	1	1	1	1	1	1	1	1	1	1
196255	YVVIGLLFMILTVAA	NS4a	2230	2244	HLA-DRB1*11:01	81% - <100%	Dengue virus 1	ELISPOT	IFNg release	Positive	1	1	1	1	1	1	1	1	1	1	1	1	1	1	0	1	1	1	1	0	1	1	1	1	1	1	1	1	1
539744	NQLAYVVIGLLFMIL	NS4a	2226	2240	HLA-DRB1*15:02	81% - <100%	Dengue virus 1	ELISPOT	IFNg release	Positive	1	1	1	1	1	1	1	1	1	1	1	1	1	1	0	1	1	1	1	0	1	1	1	1	1	1	1	1	1
738677	DLILEIGKLPQHLTL	NS4a	2099	2113	HLA-DRB1*11:04	81% - <100%	Dengue virus 1	ELISPOT	IFNg release	Positive	1	1	1	1	1	0	1	1	1	1	1	1	1	1	0	1	1	1	1	1	1	1	1	1	1	1	1	1	1
739034	GLGKTSIGLLCVMAS	NS4a	2171	2185	HLA-DRB1*11:04	81% - <100%	Dengue virus 1	ELISPOT	IFNg release	Positive	1	1	1	1	1	1	1	1	1	1	1	1	0	1	0	0	1	1	1	1	1	1	1	1	1	1	1	1	1
867109	HWIAASIILEFFLMV	NS4a	2197	2211	HLA-DPB1*04:01	81% - <100%	Dengue virus 1	ELISPOT	IFNg release	Positive	1	1	0	1	1	1	1	1	1	1	1	1	1	1	1	0	1	1	1	1	1	1	1	1	1	1	1	1	1
867112	IAASIILEFFLMVLL	NS4a	2199	2213	HLA-DQA1*01:01/DQB1*05:01	81% - <100%	Dengue virus 1	ELISPOT	IFNg release	Positive	1	1	0	1	1	1	1	1	1	1	1	1	1	1	1	0	1	1	1	1	1	1	1	1	1	1	1	1	1
867760	LLCVMASSVLLWMAS	NS4a	2179	2193	HLA-DQA1*01:02/DQB1*06:02	81% - <100%	Dengue virus 1	ELISPOT	IFNg release	Positive	1	1	1	1	1	0	1	1	1	1	1	1	0	1	0	1	1	1	0	1	1	1	1	1	1	1	1	1	1
539322	GLAFSLMKSLGGGRR	NS4b	2479	2493	HLA-DRB1*10:01	81% - <100%	Dengue virus 1	ELISPOT	IFNg release	Positive	1	1	1	0	1	1	1	1	1	1	1	1	1	1	1	1	1	1	0	1	1	1	1	1	1	1	1	1	1
539725	NISLTAIANQAAILM	NS4b	2307	2321	HLA-DRB1*04:03	81% - <100%	Dengue virus 1	ELISPOT	IFNg release	Positive	1	1	1	0	1	1	1	1	1	1	1	1	1	1	1	1	1	1	1	1	1	1	1	1	1	1	1	1	1
738560	AWTLYAVATTIITPM	NS4b	2282	2296	HLA-DRB1*04:07	81% - <100%	Dengue virus 1	ELISPOT	IFNg release	Positive	1	1	1	1	1	1	1	1	1	1	1	1	1	1	1	1	1	1	0	1	1	1	1	1	1	1	1	1	1
866002	AIANQAAILMGLDKG	NS4b	2312	2326	HLA-DQA1*01:02/DQB1*06:02	81% - <100%	Dengue virus 1	ELISPOT	IFNg release	Positive	1	1	1	0	1	1	1	1	1	1	1	1	1	1	1	1	1	1	1	1	1	1	1	1	1	1	1	1	1
869401	VDGIVAIDLDPVVYD	NS4b	2392	2406	HLA-DQA1*01:01/DQB1*05:01	81% - <100%	Dengue virus 1	ELISPOT	IFNg release	Positive	1	1	0	1	1	0	1	1	1	1	1	1	1	1	0	1	1	1	1	1	1	1	1	1	1	1	1	1	1
195150	AKWLWGFLSRNKKPR	NS5	2870	2884	HLA-DRB1*15:01	81% - <100%	Dengue virus 1	ELISPOT	IFNg release	Positive	1	1	0	1	1	0	1	1	1	1	1	1	1	1	0	0	1	1	1	1	1	1	1	1	1	1	1	1	1
195223	DMGKVRKDIPQWEPS	NS5	3175	3189	HLA-DRB1*03:01	81% - <100%	Dengue virus 1	ELISPOT	IFNg release	Positive	1	1	0	1	1	1	1	0	1	1	1	1	1	1	1	1	1	1	1	1	1	1	1	1	1	1	1	1	1
195236	EEFTRKVRSNAAIGA	NS5	2889	2903	HLA-DRB1*08:02	81% - <100%	Dengue virus 1	ELISPOT	IFNg release	Positive	1	1	1	1	1	1	1	1	1	1	1	1	1	1	1	1	1	1	1	1	1	1	1	0	1	1	1	1	1
195354	GEKWKRQLNQLSKSE	NS5	2503	2517	HLA-DRB1*04:01	81% - <100%	Dengue virus 1	ELISPOT	IFNg release	Positive	1	1	1	1	1	1	1	1	1	1	1	1	1	1	0	1	1	1	1	1	0	1	1	1	1	1	1	1	1
195421	GTGNIVSAVNMTSRM	NS5	2716	2730	HLA-DRB1*08:02	81% - <100%	Dengue virus 1	ELISPOT	IFNg release	Positive	1	1	0	1	1	1	1	1	1	1	1	1	1	1	1	1	1	1	1	1	1	1	1	1	1	1	1	1	1
195438	GYILRDISKIPGGNM	NS5	3008	3022	HLA-DRB1*08:02	81% - <100%	Dengue virus 1	ELISPOT	IFNg release	Positive	1	1	1	1	1	1	1	1	1	1	1	1	0	1	1	0	1	1	1	1	1	1	1	1	1	1	1	1	1

195464	HWFSRENSLSGVEGE	NS5	2988	3002	HLA-DRB1*04:01	81% - <100%	Dengue virus 1	ELISPOT	IFNg release	Positive	1	1	1	1	1	1	1	1	1	1	1	1	1	1	1	0	1	1	1	1	1	1	1	1	1	1	1	1	1
195655	LLATSIFKLTYQNKV	NS5	3055	3069	HLA-DRB1*07:01	81% - <100%	Dengue virus 1	ELISPOT	IFNg release	Positive	1	1	1	1	1	0	1	1	1	1	1	1	1	1	0	1	1	1	1	1	1	1	1	1	1	1	1	1	1
195755	MGKVRKDIPQWEPSK	NS5	3176	3190	HLA-DRB1*03:01	81% - <100%	Dengue virus 1	ELISPOT	IFNg release	Positive	1	1	0	1	1	1	1	0	1	1	1	1	1	1	1	1	1	1	1	1	1	1	1	1	1	1	1	1	1
195852	QLIMKDGREIVVPCR	NS5	3207	3221	HLA-DRB1*03:01	81% - <100%	Dengue virus 1	ELISPOT	IFNg release	Positive	1	1	1	1	1	1	1	1	1	1	1	1	1	1	0	1	1	1	1	1	1	1	1	1	1	1	1	1	1
196211	WATNIQVAINQVRRL	NS5	3351	3365	HLA-DRB1*03:01	81% - <100%	Dengue virus 1	ELISPOT	IFNg release	Positive	1	1	0	1	1	1	1	1	1	1	1	1	1	1	1	1	1	1	1	1	1	1	1	1	1	1	1	1	1
196220	WLWGFLSRNKKPRIC	NS5	2872	2886	HLA-DRB1*11:01	81% - <100%	Dengue virus 1	ELISPOT	IFNg release	Positive	1	1	1	1	1	0	1	1	1	1	1	1	1	1	0	0	1	1	1	1	1	1	1	1	1	1	1	1	1
196242	YQNKVVRVQRPAKSG	NS5	3065	3079	HLA-DRB1*08:02	81% - <100%	Dengue virus 1	ELISPOT	IFNg release	Positive	1	1	1	1	1	0	1	1	1	1	1	1	1	0	0	1	1	1	1	1	1	1	1	1	1	1	1	1	1
539096	ATSIFKLTYQNKVVR	NS5	3057	3071	HLA-DRB1*14:04	81% - <100%	Dengue virus 1	ELISPOT	IFNg release	Positive	1	1	1	1	1	0	1	1	1	1	1	1	1	1	0	1	1	1	1	1	1	1	1	1	1	1	1	1	1
539700	MVNGVVRLLTKPWDV	NS5	103	117	HLA-DRB1*12:02	81% - <100%	Dengue virus 1	ELISPOT	IFNg release	Positive	1	1	1	1	1	0	1	1	1	1	1	1	1	1	0	1	1	1	1	1	1	1	1	1	1	1	1	1	1
539702	MVTQIAMTDTTPFGQQR	NS5	2829	2845	HLA class II	81% - <100%	Dengue virus 1	ICS	IFNg release	Positive	1	1	1	1	1	1	1	1	1	1	1	1	1	1	1	0	1	1	1	1	1	1	1	1	1	1	1	1	1
539905	SHHFHQLIMKDGREI	NS5	3202	3216	HLA-DRB1*13:01	81% - <100%	Dengue virus 1	ELISPOT	IFNg release	Positive	1	1	1	1	1	1	1	1	1	1	1	1	1	1	0	1	1	1	1	1	1	1	1	1	1	1	1	1	1
540001	TSIFKLTYQNKVVRV	NS5	3058	3072	HLA-DRB1*09:01	81% - <100%	Dengue virus 1	ELISPOT	IFNg release	Positive	1	1	1	1	1	0	1	1	1	1	1	1	1	1	0	1	1	1	1	1	1	1	1	1	1	1	1	1	1
540020	TYGLNTFTNMEVQLI	NS5	3098	3112	HLA-DRB1*04:03	81% - <100%	Dengue virus 1	ELISPOT	IFNg release	Positive	1	1	1	1	1	0	1	1	1	1	1	1	1	1	0	1	1	1	1	1	1	1	1	1	1	1	1	1	1
540083	VRVQRPAKSGTVMDV	NS5	96	110	HLA-DRB1*13:01	81% - <100%	Dengue virus 1	ELISPOT	IFNg release	Positive	1	1	1	1	1	0	1	1	1	1	1	1	1	0	0	1	1	1	1	1	1	1	1	1	1	1	1	1	1
868296	PEHALLATSIFKLTY	NS5	3051	3065	HLA-DPB1*02:01	81% - <100%	Dengue virus 1	ELISPOT	IFNg release	Positive	1	1	1	1	1	0	1	1	1	1	1	1	1	1	0	1	1	1	1	1	1	1	1	1	1	1	1	1	1
868641	RFLEFEALGFMNEDH	NS5	2974	2988	HLA-DPB1*04:01	81% - <100%	Dengue virus 1	ELISPOT	IFNg release	Positive	1	1	1	1	1	1	0	1	1	0	1	1	1	1	1	0	0	1	1	1	1	1	1	1	1	1	1	1	1
195540	ISSMLNIMNRRKRSV	C	76	90	HLA-DRB1*14:02	51%-80%	Dengue virus 1	ELISPOT	IFNg release	Positive	1	1	1	1	1	0	0	1	0	0	1	1	1	1	0	1	0	1	1	1	1	1	1	1	1	1	1	1	1
195885	RGFKKEISSMLNIMN	C	60	74	HLA-DRB1*04:01	51%-80%	Dengue virus 1	ELISPOT	IFNg release	Positive	1	1	1	1	1	0	0	1	0	0	1	1	1	1	0	0	0	1	1	1	1	1	0	1	1	1	1	1	1
539258	FKKEISSMLNIMNRR	C	62	76	HLA-DRB1*10:01	51%-80%	Dengue virus 1	ELISPOT	IFNg release	Positive	1	1	1	1	1	0	0	1	0	0	1	1	1	1	0	0	0	1	1	1	1	1	1	1	1	1	1	1	1
539459	KEISSMLNIMNRRKR	C	64	78	HLA-DRB1*14:04	51%-80%	Dengue virus 1	ELISPOT	IFNg release	Positive	1	1	1	1	1	0	0	1	0	0	1	1	1	1	0	1	0	1	1	1	1	1	1	1	1	1	1	1	1
539475	KKEISSMLNIMNRRK	C	63	77	HLA-DRB1*14:04	51%-80%	Dengue virus 1	ELISPOT	IFNg release	Positive	1	1	1	1	1	0	0	1	0	0	1	1	1	1	0	0	0	1	1	1	1	1	1	1	1	1	1	1	1
539617	LRGFKKEISSMLNIM	C	59	73	HLA-DRB1*13:01	51%-80%	Dengue virus 1	ELISPOT	IFNg release	Positive	1	1	1	1	1	0	0	1	0	0	1	1	1	1	0	0	0	1	1	1	1	1	0	1	1	1	1	1	1
540059	VLRGFKKEISSMLNI	C	68	82	HLA-DRB1*04:07	51%-80%	Dengue virus 1	ELISPOT	IFNg release	Positive	1	1	1	1	1	0	0	1	0	0	1	1	1	1	0	0	0	1	1	1	1	1	0	1	1	1	1	1	1
195831	PGFTVIALFLAHAIG	PreM	245	259	HLA-DRB1*15:01	51%-80%	Dengue virus 1	ELISPOT	IFNg release	Positive	1	0	0	1	1	1	0	1	1	0	1	1	1	1	1	0	0	1	1	1	1	1	1	1	1	1	1	1	0
196085	TVIALFLAHAIGTSI	PreM	248	262	HLA-DRB1*07:01	51%-80%	Dengue virus 1	ELISPOT	IFNg release	Positive	1	0	0	1	1	1	0	1	1	0	1	1	1	1	1	0	0	1	1	1	1	1	1	1	1	1	1	1	0
867083	HPGFTVIALFLAHAI	PreM	244	258	HLA-DPB1*02:01	51%-80%	Dengue virus 1	ELISPOT	IFNg release	Positive	1	0	0	1	1	1	0	1	1	0	1	1	1	1	1	0	0	1	1	1	1	1	1	1	1	1	1	1	0
539369	GVSWTMKIGIGILLT	E	445	459	HLA-DRB1*15:02	51%-80%	Dengue virus 1	ELISPOT	IFNg release	Positive	1	1	1	0	0	0	1	0	1	1	1	1	1	1	0	0	1	1	1	1	1	1	1	1	1	1	0	0	1
539670	MKIGIGILLTWLGLN	E	450	464	HLA-DRB1*15:06	51%-80%	Dengue virus 1	ELISPOT	IFNg release	Positive	1	1	1	0	0	0	1	0	1	1	1	1	1	1	0	0	1	1	1	1	1	1	1	1	1	1	0	0	1
867101	HTALTGATEIQTSGT	E	541	555	HLA-DQA1*01:02/DQB1*06:02	51%-80%	Dengue virus 1	ELISPOT	IFNg release	Positive	1	0	1	1	1	0	0	1	1	0	1	1	1	1	0	1	0	1	1	1	1	1	1	1	1	1	1	1	0
195162	AMVLSIVSLFPLCLS	NS2a	1296	1310	HLA-DRB1*15:01	51%-80%	Dengue virus 1	ELISPOT	IFNg release	Positive	0	1	1	1	1	1	1	1	1	1	1	0	1	1	1	0	1	1	0	0	0	0	1	1	1	1	1	1	1
195595	KTMAMVLSIVSLFPL	NS2a	1293	1307	HLA-DRB1*03:01	51%-80%	Dengue virus 1	ELISPOT	IFNg release	Positive	0	0	0	1	1	1	0	1	1	0	1	0	1	1	1	0	0	1	0	0	0	0	1	1	1	1	1	1	0
195745	MAMVLSIVSLFPLCL	NS2a	1295	1309	HLA-DRB1*07:01	51%-80%	Dengue virus 1	ELISPOT	IFNg release	Positive	0	0	0	1	1	1	0	1	1	0	1	0	1	1	1	0	0	1	0	0	0	0	1	1	1	1	1	1	0
196216	WKTMAMVLSIVSLFP	NS2a	1292	1306	HLA-DRB1*08:02	51%-80%	Dengue virus 1	ELISPOT	IFNg release	Positive	0	0	0	1	1	1	0	1	1	0	1	0	1	1	1	0	0	1	0	0	0	0	1	1	1	1	1	1	0
1392180	GKEIVDLMCHATFTM	NS3	1730	1744		51%-80%	Dengue virus 1	biological activity	activation	Positive	0	0	0	1	1	1	0	1	1	0	1	1	1	1	1	1	0	1	0	0	0	0	1	1	1	1	1	1	0
869696	WNTGEEVQVIAVEPG	NS3	1564	1578	HLA-DQA1*03:01/DQB1*03:02	51%-80%	Dengue virus 1	ELISPOT	IFNg release	Positive	1	1	1	1	1	0	1	1	1	1	0	0	0	0	1	0	1	0	1	1	1	1	1	1	1	0	1	1	1
539383	HLTLRAQNALDNLVM	NS4a	2110	2124	HLA-DRB3*02:02	51%-80%	Dengue virus 1	ELISPOT	IFNg release	Positive	1	1	1	1	1	0	1	1	1	1	0	0	0	0	0	0	1	0	1	1	1	1	1	1	1	0	1	1	1
195186	ATNIQVAINQVRRLI	NS5	3352	3366	HLA-DRB1*03:01	51%-80%	Dengue virus 1	ELISPOT	IFNg release	Positive	1	1	0	1	1	1	1	1	1	1	0	0	0	0	1	0	1	0	1	1	1	1	1	1	1	0	1	1	1
195385	GLHKLGYILRDISKI	NS5	3003	3017	HLA-DRB1*08:02	51%-80%	Dengue virus 1	ELISPOT	IFNg release	Positive	1	1	1	1	1	1	0	1	1	0	1	1	1	1	1	1	0	1	1	0	0	0	1	1	1	1	1	1	1
195783	NENYLDYMTSMKRFK	NS5	3368	3382	HLA-DRB1*11:01	51%-80%	Dengue virus 1	ELISPOT	IFNg release	Positive	1	1	1	1	1	0	1	1	1	1	1	1	1	1	0	1	1	1	0	0	0	0	1	1	1	1	1	1	1
195824	NYLDYMTSMKRFKNE	NS5	3370	3384	HLA-DRB1*14:02	51%-80%	Dengue virus 1	ELISPOT	IFNg release	Positive	1	1	1	1	1	0	1	1	1	1	1	1	1	1	0	1	1	1	0	0	0	0	1	1	1	1	1	1	1
539350	GTAKLRWFVERNLVK	NS5	2551	2565	HLA-DRB1*09:01	51%-80%	Dengue virus 1	ELISPOT	IFNg release	Positive	1	0	1	1	1	1	0	1	1	0	1	1	1	1	1	1	0	1	1	1	1	0	1	1	1	1	1	1	0
539691	MTSRMLLNRFTMAHR	NS5	17	31	HLA-DRB1*14:06	51%-80%	Dengue virus 1	ELISPOT	IFNg release	Positive	1	1	1	0	0	1	1	0	1	1	0	0	0	0	1	0	1	0	1	1	1	1	0	0	1	0	0	0	1
738814	ENYLDYMTSMKRFKN	NS5	3369	3383	HLA-DRB1*14:02	51%-80%	Dengue virus 1	ELISPOT	IFNg release	Positive	1	1	1	1	1	0	1	1	1	1	1	1	1	1	0	1	1	1	0	0	0	0	1	1	1	1	1	1	1
740786	YLDYMTSMKRFKNES	NS5	3371	3385	HLA-DRB1*14:02	51%-80%	Dengue virus 1	ELISPOT	IFNg release	Positive	1	1	1	1	1	0	1	1	1	1	1	1	1	1	0	1	1	1	0	0	0	0	1	1	1	1	1	1	1
866481	EAVEDERFWDLVHRE	NS5	2916	2930	HLA-DQA1*01:01/DQB1*05:01	51%-80%	Dengue virus 1	ELISPOT	IFNg release	Positive	1	0	1	1	1	0	0	1	1	0	1	1	1	1	0	0	0	1	1	1	1	1	1	1	1	1	1	1	0
869091	TAKLRWFVERNLVKP	NS5	2552	2566	HLA-DQA1*01:01/DQB1*05:01	51%-80%	Dengue virus 1	ELISPOT	IFNg release	Positive	1	0	1	1	1	1	0	1	1	0	1	1	1	1	1	1	0	1	1	1	1	0	1	1	1	1	1	1	0
1067569	LARWGSFKKNGAIKV	C	66	80	HLA-DRB1*07:01	>0% - 50%	Dengue virus 1	ELISPOT	IFNg release	Positive-Low	0	0	0	0	0	1	0	0	0	0	0	0	0	0	1	0	0	0	0	0	0	0	0	0	0	0	0	0	0
195315	FNMLKRARNRVSTVS	C	13	27	HLA-DRB1*08:02	>0% - 50%	Dengue virus 1	ELISPOT	IFNg release	Positive	0	0	0	0	0	1	0	0	0	0	0	0	0	0	1	0	0	0	0	0	0	0	0	0	0	0	0	0	0
195498	IKVLRGFKKEISNML	C	78	92	HLA-DRB1*04:01	>0% - 50%	Dengue virus 1	ELISPOT	IFNg release	Positive	0	0	0	0	0	1	0	0	1	0	0	0	0	0	1	0	0	0	0	0	0	0	0	0	0	0	0	0	0
195535	ISNMLNIMNRRKRSV	C	88	102	HLA-DRB1*08:02	>0% - 50%	Dengue virus 1	ELISPOT	IFNg release	Positive	0	0	0	0	0	0	0	0	1	0	0	0	0	0	0	0	0	0	0	0	0	0	0	0	0	0	0	0	0
195557	KEISNMLNIMNRRKR	C	86	100	HLA-DRB1*13:01	>0% - 50%	Dengue virus 1	ELISPOT	IFNg release	Positive	0	0	0	0	0	0	0	0	1	0	0	0	0	0	0	0	0	0	0	0	0	0	0	0	0	0	0	0	0
195805	NMLNIMNRRKRSVTM	C	90	104	HLA-DRB1*11:01	>0% - 50%	Dengue virus 1	ELISPOT	IFNg release	Positive	0	0	0	0	0	0	0	0	1	0	0	0	0	0	0	0	0	0	0	0	0	0	0	0	0	0	0	0	0
195883	RGFKKEISNMLNIMN	C	82	96	HLA-DRB1*04:01	>0% - 50%	Dengue virus 1	ELISPOT	IFNg release	Positive	0	0	0	0	0	1	0	0	1	0	0	0	0	0	1	0	0	0	0	0	0	0	0	0	0	0	0	0	0
196146	VLRGFKKEISNMLNI	C	80	94	HLA-DRB1*04:01	>0% - 50%	Dengue virus 1	ELISPOT	IFNg release	Positive	0	0	0	0	0	1	0	0	1	0	0	0	0	0	1	0	0	0	0	0	0	0	0	0	0	0	0	0	0
196195	VTMLFMLLPTALAFH	C	102	116	HLA-DRB1*04:01	>0% - 50%	Dengue virus 1	ELISPOT	IFNg release	Positive	0	0	0	0	0	0	0	0	0	0	0	0	0	0	0	1	0	0	0	0	0	0	0	0	0	0	0	0	0
39096	LRGFKKEISNML	C	81	92	HLA-DPw4	>0% - 50%	Dengue virus 1	ICS	TNF release	Positive	0	0	0	0	0	1	0	0	1	0	0	0	0	0	1	0	0	0	0	0	0	0	0	0	0	0	0	0	0
539257	FKKEISNMLNIMNRR	C	84	98	HLA-DRB1*04:03	>0% - 50%	Dengue virus 1	ELISPOT	IFNg release	Positive	0	0	0	0	0	1	0	0	1	0	0	0	0	0	1	0	0	0	0	0	0	0	0	0	0	0	0	0	0
539278	GAIKVLRGFKKEISN	C	76	90	HLA-DRB1*12:02	>0% - 50%	Dengue virus 1	ELISPOT	IFNg release	Positive	0	0	0	0	0	1	0	0	1	0	0	0	0	0	1	0	0	0	0	0	0	0	0	0	0	0	0	0	0
539616	LRGFKKEISNMLNIM	C	81	95	HLA-DRB1*10:01	>0% - 50%	Dengue virus 1	ELISPOT	IFNg release	Positive	0	0	0	0	0	1	0	0	1	0	0	0	0	0	1	0	0	0	0	0	0	0	0	0	0	0	0	0	0
539780	PMKLVMAFIAFLRFL	C	21	35	HLA-DRB1*13:01	>0% - 50%	Dengue virus 1	ELISPOT	IFNg release	Positive	0	0	1	0	0	0	1	0	0	1	0	0	0	0	1	0	1	0	1	0	0	0	0	0	0	0	0	0	1
539866	RPSFNMLKRVRNRVS	C	10	24	HLA-DRB1*08:03	>0% - 50%	Dengue virus 1	ELISPOT	IFNg release	Positive	0	1	0	0	0	0	0	0	0	0	0	0	0	0	0	0	0	0	0	0	0	0	0	0	0	0	0	0	1
540117	WGSFKKNGAIKVLRG	C	47	61	HLA-DRB1*09:01	>0% - 50%	Dengue virus 1	ELISPOT	IFNg release	Positive	0	0	0	0	0	1	0	0	0	0	0	0	0	0	1	0	0	0	0	0	0	0	0	0	0	0	0	0	0
740331	SNMLNIMNRRKRSVT	C	89	103	HLA-DRB1*14:02	>0% - 50%	Dengue virus 1	ELISPOT	IFNg release	Positive	0	0	0	0	0	0	0	0	1	0	0	0	0	0	0	0	0	0	0	0	0	0	0	0	0	0	0	0	0
867523	KVLRGFKKEISNMLN	C	79	93	HLA-DRB3*02:02	>0% - 50%	Dengue virus 1	ELISPOT	IFNg release	Positive	0	0	0	0	0	1	0	0	1	0	0	0	0	0	1	0	0	0	0	0	0	0	0	0	0	0	0	0	0
95107	KLVMAFIAFLRFL	C	45	57	HLA-DPw4	>0% - 50%	Dengue virus 1	ELISPOT	IFNg release	Positive	0	0	1	0	0	1	1	0	0	1	0	0	0	0	1	0	1	0	1	0	0	0	0	0	0	0	0	0	1
195589	KSLLFKTSVGVNMCT	PreM	135	149	HLA-DRB1*07:01	>0% - 50%	Dengue virus 1	ELISPOT	IFNg release	Positive	0	0	0	0	0	0	0	0	0	0	0	0	0	0	0	0	0	0	1	0	0	0	0	0	0	0	0	0	0
869062	SVGVNMCTLIAMDLG	PreM	142	156	HLA-DQA1*01:02/DQB1*06:02	>0% - 50%	Dengue virus 1	ELISPOT	IFNg release	Positive	0	0	0	0	0	0	0	0	0	0	0	0	0	0	0	0	0	0	1	0	0	0	0	0	0	0	0	0	0
195361	GGVFTSVGKLVHQIF	E	426	440	HLA-DRB1*11:01	>0% - 50%	Dengue virus 1	ELISPOT	IFNg release	Positive	0	0	0	0	0	1	0	0	0	0	0	0	0	0	1	0	0	0	0	0	0	0	0	0	0	0	0	0	0
867378	KCRLKMDKLTLKGMS	E	564	578	HLA-DRB3*02:02	>0% - 50%	Dengue virus 1	ELISPOT	IFNg release	Positive	0	0	0	0	0	1	0	0	0	0	0	0	0	0	0	0	0	0	1	0	0	0	0	0	0	0	0	0	0
195785	NETWKLTRASFIEVK	NS1	982	996	HLA-DRB1*07:01	>0% - 50%	Dengue virus 1	ELISPOT	IFNg release	Positive	0	0	1	0	0	0	0	0	0	0	0	0	0	0	0	0	0	0	0	0	0	0	0	0	0	0	0	0	0
738503	ANGILAQGKKMIRPQ	NS1	868	882	HLA-DRB1*14:06	>0% - 50%	Dengue virus 1	ELISPOT	IFNg release	Positive	0	0	0	0	0	0	0	0	0	0	0	0	0	0	0	0	0	0	1	0	0	0	0	0	0	0	0	0	0
740562	TVVVGDANGILAQGK	NS1	862	876	HLA-DRB1*01:02	>0% - 50%	Dengue virus 1	ELISPOT	IFNg release	Positive	0	0	0	0	0	0	0	0	0	0	0	0	0	0	0	0	0	0	1	0	0	0	0	0	0	0	0	0	0
866693	FGIFTTNIWLKLRDS	NS1	196	210	HLA-DPB1*04:01	>0% - 50%	Dengue virus 1	ELISPOT	IFNg release	Positive	0	1	1	0	0	1	1	0	0	1	0	0	0	0	1	0	1	0	1	1	1	1	0	0	0	0	0	0	1
867405	KIIGADIQNTTFIID	NS1	897	911	HLA-DPB1*04:02	>0% - 50%	Dengue virus 1	ELISPOT	IFNg release	Positive	0	0	0	0	0	0	0	0	0	0	0	0	0	0	0	0	0	0	1	0	0	0	0	0	0	0	0	0	0
869721	YGFGIFTTNIWLKLR	NS1	194	208	HLA-DPB1*02:01	>0% - 50%	Dengue virus 1	ELISPOT	IFNg release	Positive	0	1	1	0	0	1	1	0	0	1	0	0	0	0	1	0	1	0	1	1	1	1	0	0	0	0	0	0	1
167816	SLVASVELPNSLEELGDGLAMGIMI	NS2a	109	133	HLA class II	>0% - 50%	Dengue virus 1	ELISPOT	IFNg release	Positive	0	0	0	0	0	1	0	0	0	0	0	0	0	0	0	0	0	0	0	0	0	0	0	0	0	0	0	0	0
539086	AMGIMILKLLTDFQS	NS2a	1255	1269	HLA-DRB1*04:03	>0% - 50%	Dengue virus 1	ELISPOT	IFNg release	Positive	0	0	0	0	0	1	0	0	0	0	0	0	0	0	0	0	0	0	0	0	0	0	0	0	0	0	0	0	0
539272	FSLHYAWKTMAMVLS	NS2a	1286	1300	HLA-DRB1*09:01	>0% - 50%	Dengue virus 1	ELISPOT	IFNg release	Positive	0	0	0	0	0	1	0	0	0	0	0	0	0	0	0	0	0	0	0	0	0	0	0	0	0	0	0	0	0
539517	KTTFSLHYAWKTMAM	NS2a	1283	1297	HLA-DRB1*09:01	>0% - 50%	Dengue virus 1	ELISPOT	IFNg release	Positive	0	0	0	0	0	1	0	0	0	0	0	0	0	0	0	0	0	0	0	0	0	0	0	0	0	0	0	0	0
539913	SLHYAWKTMAMVLSI	NS2a	1287	1301	HLA-DRB1*09:01	>0% - 50%	Dengue virus 1	ELISPOT	IFNg release	Positive	0	0	0	0	0	1	0	0	0	0	0	0	0	0	0	0	0	0	0	0	0	0	0	0	0	0	0	0	0
539970	TFSLHYAWKTMAMVL	NS2a	1285	1299	HLA-DRB1*14:06	>0% - 50%	Dengue virus 1	ELISPOT	IFNg release	Positive	0	0	0	0	0	1	0	0	0	0	0	0	0	0	0	0	0	0	0	0	0	0	0	0	0	0	0	0	0
738679	DLIRLCIMVGANASD	NS2a	1179	1193	HLA-DRB1*11:04	>0% - 50%	Dengue virus 1	ELISPOT	IFNg release	Positive	0	0	1	0	0	0	0	0	0	0	0	0	0	0	1	0	0	0	0	0	0	0	0	0	0	0	0	0	0
739508	LLCISIMIEEVMRSR	NS2a	1139	1153	HLA-DRB1*11:04	>0% - 50%	Dengue virus 1	ELISPOT	IFNg release	Positive	0	0	0	0	0	1	0	0	0	0	0	0	0	0	1	0	0	0	0	0	0	0	0	0	0	0	0	0	0
867232	ILKLLTDFQSHQLWA	NS2a	1260	1274	HLA-DPB1*01:01	>0% - 50%	Dengue virus 1	ELISPOT	IFNg release	Positive	0	0	0	0	0	1	0	0	0	0	0	0	0	0	0	0	0	0	0	0	0	0	0	0	0	0	0	0	0
195622	LDDGIYRIMQRGLLG	NS3	1493	1507	HLA-DRB1*11:01	>0% - 50%	Dengue virus 1	ELISPOT	IFNg release	Positive	0	0	0	0	0	1	0	0	0	0	0	0	0	0	1	0	0	0	0	0	0	0	0	0	0	0	0	0	0
539156	DGIYRIMQRGLLGRS	NS3	1495	1509	HLA-DRB1*12:02	>0% - 50%	Dengue virus 1	ELISPOT	IFNg release	Positive	0	0	0	0	0	1	0	0	0	0	0	0	0	0	1	0	0	0	0	0	0	0	0	0	0	0	0	0	0
539309	GIYRIMQRGLLGRSQ	NS3	1496	1510	HLA-DRB1*14:04	>0% - 50%	Dengue virus 1	ELISPOT	IFNg release	Positive	0	0	0	0	0	1	0	0	0	0	0	0	0	0	1	0	0	0	0	0	0	0	0	0	0	0	0	0	0
866721	FKTPEGEVGAIALDF	NS3	1591	1605	HLA-DQA1*03:01/DQB1*03:02	>0% - 50%	Dengue virus 1	ELISPOT	IFNg release	Positive	1	1	1	0	0	0	1	0	1	1	0	0	0	0	1	0	1	0	1	1	1	1	0	0	1	0	0	0	1
867025	GVFQENVFHTMWHVT	NS3	1514	1528	HLA-DPB1*04:01	>0% - 50%	Dengue virus 1	ELISPOT	IFNg release	Positive	0	0	0	0	0	1	0	0	0	0	0	0	0	0	1	0	0	0	0	0	0	0	0	0	0	0	0	0	0
195485	IGLLCVMASSALLWM	NS4a	2177	2191	HLA-DRB1*07:01	>0% - 50%	Dengue virus 1	ELISPOT	IFNg release	Positive	0	0	0	0	0	1	0	0	0	0	0	0	0	0	0	0	0	0	1	0	0	0	0	0	0	0	0	0	0
867567	LCVMASSALLWMANV	NS4a	2180	2194	HLA-DQA1*01:02/DQB1*06:02	>0% - 50%	Dengue virus 1	ELISPOT	IFNg release	Positive	0	0	0	0	0	0	0	0	0	0	0	0	0	0	1	0	0	0	0	0	0	0	0	0	0	0	0	0	0
195441	HATMLDVDLRPASAW	NS4b	2269	2283	HLA-DRB1*03:01	>0% - 50%	Dengue virus 1	ELISPOT	IFNg release	Positive	0	0	0	0	0	0	0	0	0	0	0	0	0	0	0	0	0	0	1	0	0	0	0	0	0	0	0	0	0
195455	HHHATMLDVDLRPAS	NS4b	2267	2281	HLA-DRB1*03:01	>0% - 50%	Dengue virus 1	ELISPOT	IFNg release	Positive	0	0	0	0	0	0	0	0	0	0	0	0	0	0	0	0	0	0	1	0	0	0	0	0	0	0	0	0	0
195561	KFWNTTIAVSMANIF	NS4b	2456	2470	HLA-DRB1*07:01	>0% - 50%	Dengue virus 1	ELISPOT	IFNg release	Positive	0	1	1	0	0	1	1	0	0	1	0	0	0	0	0	0	1	0	1	1	1	1	0	0	0	0	0	0	1
738686	DLRPASAWTLYAVAT	NS4b	2276	2290	HLA-DRB1*01:02	>0% - 50%	Dengue virus 1	ELISPOT	IFNg release	Positive	0	0	0	0	0	0	0	0	0	0	0	0	0	0	0	0	0	0	1	0	0	0	0	0	0	0	0	0	0
740732	WTLYAVATTVITPMM	NS4b	2283	2297	HLA-DRB1*04:07	>0% - 50%	Dengue virus 1	ELISPOT	IFNg release	Positive	0	0	0	0	0	0	0	0	0	0	0	0	0	0	0	0	0	0	1	0	0	0	0	0	0	0	0	0	0
195147	AKNGTVMDVISRRDQ	NS5	3076	3090	HLA-DRB1*03:01	>0% - 50%	Dengue virus 1	ELISPOT	IFNg release	Positive	0	0	0	0	0	1	0	0	0	0	0	0	0	1	1	0	0	0	0	0	0	0	0	0	0	0	0	0	0
195582	KPIDDRFATALTALN	NS5	3160	3174	HLA-DRB1*04:01	>0% - 50%	Dengue virus 1	ELISPOT	IFNg release	Positive	0	0	0	0	0	1	0	0	0	0	0	0	0	0	1	0	0	0	1	0	0	0	0	0	0	0	0	0	0
195786	NGTVMDVISRRDQRG	NS5	3078	3092	HLA-DRB1*03:01	>0% - 50%	Dengue virus 1	ELISPOT	IFNg release	Positive	0	0	0	0	0	1	0	0	0	0	0	0	0	1	1	0	0	0	0	0	0	0	0	0	0	0	0	0	0
195903	RPAKNGTVMDVISRR	NS5	3074	3088	HLA-DRB1*03:01	>0% - 50%	Dengue virus 1	ELISPOT	IFNg release	Positive	0	0	0	0	0	1	0	0	0	0	0	0	0	1	1	0	0	0	0	0	0	0	0	0	0	0	0	0	0
195923	RWLWGFLSRNKKPRI	NS5	2871	2885	HLA-DRB1*15:01	>0% - 50%	Dengue virus 1	ELISPOT	IFNg release	Positive	0	0	1	0	0	0	0	0	0	0	0	0	0	0	0	0	0	0	0	0	0	0	0	0	0	0	0	0	0
196191	VTARWLWGFLSRNKK	NS5	2868	2882	HLA-DRB1*15:01	>0% - 50%	Dengue virus 1	ELISPOT	IFNg release	Positive	0	0	1	0	0	0	0	0	0	0	0	0	0	0	0	0	0	0	0	0	0	0	0	0	0	0	0	0	0
196241	YQNKVVRVQRPAKNG	NS5	3065	3079	HLA-DRB1*08:02	>0% - 50%	Dengue virus 1	ELISPOT	IFNg release	Positive	0	0	0	0	0	1	0	0	0	0	0	0	0	1	1	0	0	0	0	0	0	0	0	0	0	0	0	0	0
539396	IDDRFATALTALNDM	NS5	3162	3176	HLA-DPB1*01:01	>0% - 50%	Dengue virus 1	ELISPOT	IFNg release	Positive	0	0	0	0	0	1	0	0	0	0	0	0	0	0	1	0	0	0	1	0	0	0	0	0	0	0	0	0	0
539435	ISGDDCVVKPIDDRFAT	NS5	3152	3168	HLA class II	>0% - 50%	Dengue virus 1	ICS	IFNg release	Positive	0	0	0	0	0	1	0	0	0	0	0	0	0	0	1	0	0	0	1	0	0	0	0	0	0	0	0	0	0
539862	RMLLNRFTMAHRKPT	NS5	20	34	HLA-DRB1*14:02	>0% - 50%	Dengue virus 1	ELISPOT	IFNg release	Positive	0	1	1	0	0	1	1	0	1	1	0	0	0	0	1	0	1	0	1	1	1	1	0	0	1	0	0	0	1
540018	TYGLNTFTNMEAQLI	NS5	3098	3112	HLA-DRB1*15:06	>0% - 50%	Dengue virus 1	ELISPOT	IFNg release	Positive	0	0	0	0	0	1	0	0	0	0	0	0	0	0	1	0	0	0	0	0	0	0	0	0	0	0	0	0	0
540100	VVKPIDDRFATALTALN	NS5	3157	3173	HLA class II	>0% - 50%	Dengue virus 1	ICS	IFNg release	Positive	0	0	0	0	0	1	0	0	0	0	0	0	0	0	1	0	0	0	1	0	0	0	0	0	0	0	0	0	0
740024	QNKVVRVQRPAKNGT	NS5	3066	3080	HLA-DRB1*14:06	>0% - 50%	Dengue virus 1	ELISPOT	IFNg release	Positive	0	0	0	0	0	1	0	0	0	0	0	0	0	1	1	0	0	0	0	0	0	0	0	0	0	0	0	0	0
740668	VQLIRQMESEGIFLP	NS5	3109	3123	HLA-DRB1*11:04	>0% - 50%	Dengue virus 1	ELISPOT	IFNg release	Positive	0	0	0	0	0	0	0	0	0	0	0	0	0	0	0	0	0	0	1	0	0	0	0	0	0	0	0	0	0
868240	NKVVRVQRPAKNGTV	NS5	3067	3081	HLA-DRB4*01:01	>0% - 50%	Dengue virus 1	ELISPOT	IFNg release	Positive	0	0	0	0	0	1	0	0	0	0	0	0	0	1	1	0	0	0	0	0	0	0	0	0	0	0	0	0	0

CD4 DENV-2 Epitopes

											NCBI IDs of DENV-2 circulating strains from India, (100% identity=1,green ; else 0, red)																																												
Epitope_ID	AA_Seq	Protein_Name	Start	End	Allele	step	Serotype	Method/Technique	Assay Group	Qualitative Measure	AFZ40227.1	AYJ72763.1	AYJ72772.1	AYJ72773.1	AYJ72774.1	AYJ72775.1	AYJ72776.1	AYJ72777.1	AYJ72778.1	AYJ72779.1	AYJ72780.1	AYJ72764.1	AYJ72765.1	AYJ72766.1	AYJ72767.1	AYJ72768.1	AYJ72769.1	AYJ72770.1	AYJ72771.1	ACQ44493.1	AFZ40121.1	ANC57597.1	QCZ24979.1	QCZ24978.1	QCZ24977.1	QCZ24976.1	QCZ24974.1	QCZ24973.1	QCZ24971.1	QCZ24970.1	QCZ24969.1	QCZ24966.1	QCZ24965.1	QBA57539.1	QBA57535.1	QBA57537.1	AFZ40226.1	AII99332.1	AYJ72757.1	AYJ72758.1	AYJ72759.1	AYJ72760.1	AYJ72761.1	AYJ72762.1	ARO52692.1	ARO52693.1	AFZ40225.1	AYE66920.1	AYE66919.1
150404	LRLFMALVAFLRFLT	C	44	58	HLA-DRB1*11:01	0%	Dengue virus 2	ELISPOT	IFNg release	Positive	0	0	0	0	0	0	0	0	0	0	0	0	0	0	0	0	0	0	0	0	0	0	0	0	0	0	0	0	0	0	0	0	0	0	0	0	0	0	0	0	0	0	0	0	0	0	0	0	0
195121	AGMIIMLIPTVVAFH	C	102	116	HLA-DRB1*08:02	0%	Dengue virus 2	ELISPOT	IFNg release	Positive	0	0	0	0	0	0	0	0	0	0	0	0	0	0	0	0	0	0	0	0	0	0	0	0	0	0	0	0	0	0	0	0	0	0	0	0	0	0	0	0	0	0	0	0	0	0	0	0	0
195369	GIIIMIIPTVMAFHL	C	103	117	HLA-DRB1*08:02	0%	Dengue virus 2	ELISPOT	IFNg release	Positive	0	0	0	0	0	0	0	0	0	0	0	0	0	0	0	0	0	0	0	0	0	0	0	0	0	0	0	0	0	0	0	0	0	0	0	0	0	0	0	0	0	0	0	0	0	0	0	0	0
195413	GPLRLFMALVAFLRF	C	42	56	HLA-DRB1*04:01	0%	Dengue virus 2	ELISPOT	IFNg release	Positive	0	0	0	0	0	0	0	0	0	0	0	0	0	0	0	0	0	0	0	0	0	0	0	0	0	0	0	0	0	0	0	0	0	0	0	0	0	0	0	0	0	0	0	0	0	0	0	0	0
195432	GVIIMMIPTVMAFHL	C	103	117	HLA-DRB1*08:02	0%	Dengue virus 2	ELISPOT	IFNg release	Positive	0	0	0	0	0	0	0	0	0	0	0	0	0	0	0	0	0	0	0	0	0	0	0	0	0	0	0	0	0	0	0	0	0	0	0	0	0	0	0	0	0	0	0	0	0	0	0	0	0
195490	IGRMLNILSRRRRTA	C	88	102	HLA-DRB1*11:01	0%	Dengue virus 2	ELISPOT	IFNg release	Positive	0	0	0	0	0	0	0	0	0	0	0	0	0	0	0	0	0	0	0	0	0	0	0	0	0	0	0	0	0	0	0	0	0	0	0	0	0	0	0	0	0	0	0	0	0	0	0	0	0
195654	LKLYMALVAFLRFLT	C	44	58	HLA-DRB1*15:01	0%	Dengue virus 2	ELISPOT	IFNg release	Positive	0	0	0	0	0	0	0	0	0	0	0	0	0	0	0	0	0	0	0	0	0	0	0	0	0	0	0	0	0	0	0	0	0	0	0	0	0	0	0	0	0	0	0	0	0	0	0	0	0
195910	RTAGVIIMMIPTVVA	C	100	114	HLA-DRB1*08:02	0%	Dengue virus 2	ELISPOT	IFNg release	Positive	0	0	0	0	0	0	0	0	0	0	0	0	0	0	0	0	0	0	0	0	0	0	0	0	0	0	0	0	0	0	0	0	0	0	0	0	0	0	0	0	0	0	0	0	0	0	0	0	0
195911	RTASVIVMLIPTVMA	C	100	114	HLA-DRB1*08:02	0%	Dengue virus 2	ELISPOT	IFNg release	Positive	0	0	0	0	0	0	0	0	0	0	0	0	0	0	0	0	0	0	0	0	0	0	0	0	0	0	0	0	0	0	0	0	0	0	0	0	0	0	0	0	0	0	0	0	0	0	0	0	0
196024	TAGVIVMLIPTVMAF	C	101	115	HLA-DRB1*08:02	0%	Dengue virus 2	ELISPOT	IFNg release	Positive	0	0	0	0	0	0	0	0	0	0	0	0	0	0	0	0	0	0	0	0	0	0	0	0	0	0	0	0	0	0	0	0	0	0	0	0	0	0	0	0	0	0	0	0	0	0	0	0	0
196084	TVGVIIMLIPTAMAF	C	101	115	HLA-DRB1*08:02	0%	Dengue virus 2	ELISPOT	IFNg release	Positive	0	0	0	0	0	0	0	0	0	0	0	0	0	0	0	0	0	0	0	0	0	0	0	0	0	0	0	0	0	0	0	0	0	0	0	0	0	0	0	0	0	0	0	0	0	0	0	0	0
53867	RGFRKEIGRMLNILNRRRS	C	17	35	HLA class II	0%	Dengue virus 2	ELISPOT	IFNg release	Positive	0	0	0	0	0	0	0	0	0	0	0	0	0	0	0	0	0	0	0	0	0	0	0	0	0	0	0	0	0	0	0	0	0	0	0	0	0	0	0	0	0	0	0	0	0	0	0	0	0
539046	AFLRFLTIPPTVGIL	C	52	66	HLA-DRB1*10:01	0%	Dengue virus 2	ELISPOT	IFNg release	Positive	0	0	0	0	0	0	0	0	0	0	0	0	0	0	0	0	0	0	0	0	0	0	0	0	0	0	0	0	0	0	0	0	0	0	0	0	0	0	0	0	0	0	0	0	0	0	0	0	0
539051	AFVAFLRFLTIPPTA	C	9	23	HLA-DRB1*15:02	0%	Dengue virus 2	ELISPOT	IFNg release	Positive	0	0	0	0	0	0	0	0	0	0	0	0	0	0	0	0	0	0	0	0	0	0	0	0	0	0	0	0	0	0	0	0	0	0	0	0	0	0	0	0	0	0	0	0	0	0	0	0	0
539213	EIGRMLNILNKRRRT	C	87	101	HLA-DRB1*13:01	0%	Dengue virus 2	ELISPOT	IFNg release	Positive	0	0	0	0	0	0	0	0	0	0	0	0	0	0	0	0	0	0	0	0	0	0	0	0	0	0	0	0	0	0	0	0	0	0	0	0	0	0	0	0	0	0	0	0	0	0	0	0	0
539343	GRMLNIMNRRRRTAG	C	89	103	HLA-DRB1*13:01	0%	Dengue virus 2	ELISPOT	IFNg release	Positive	0	0	0	0	0	0	0	0	0	0	0	0	0	0	0	0	0	0	0	0	0	0	0	0	0	0	0	0	0	0	0	0	0	0	0	0	0	0	0	0	0	0	0	0	0	0	0	0	0
539549	LFTALVAFLRFLTIP	C	46	60	HLA-DRB1*15:06	0%	Dengue virus 2	ELISPOT	IFNg release	Positive	0	0	0	0	0	0	0	0	0	0	0	0	0	0	0	0	0	0	0	0	0	0	0	0	0	0	0	0	0	0	0	0	0	0	0	0	0	0	0	0	0	0	0	0	0	0	0	0	0
62853	TAGILKRWGTIKKSKANVL	C	62	81	HLA class II	0%	Dengue virus 2	ELISPOT	IFNg release	Positive	0	0	0	0	0	0	0	0	0	0	0	0	0	0	0	0	0	0	0	0	0	0	0	0	0	0	0	0	0	0	0	0	0	0	0	0	0	0	0	0	0	0	0	0	0	0	0	0	0
739176	IGRMLNILNKRRRTA	C	88	102	HLA-DRB1*14:02	0%	Dengue virus 2	ELISPOT	IFNg release	Positive	0	0	0	0	0	0	0	0	0	0	0	0	0	0	0	0	0	0	0	0	0	0	0	0	0	0	0	0	0	0	0	0	0	0	0	0	0	0	0	0	0	0	0	0	0	0	0	0	0
739177	IGRMLNILNRRRRSA	C	88	102	HLA-DRB1*14:02	0%	Dengue virus 2	ELISPOT	IFNg release	Positive	0	0	0	0	0	0	0	0	0	0	0	0	0	0	0	0	0	0	0	0	0	0	0	0	0	0	0	0	0	0	0	0	0	0	0	0	0	0	0	0	0	0	0	0	0	0	0	0	0
739179	IGRMLNILNRRRRSV	C	88	102	HLA-DRB1*14:02	0%	Dengue virus 2	ELISPOT	IFNg release	Positive	0	0	0	0	0	0	0	0	0	0	0	0	0	0	0	0	0	0	0	0	0	0	0	0	0	0	0	0	0	0	0	0	0	0	0	0	0	0	0	0	0	0	0	0	0	0	0	0	0
739180	IGRMLNILNRRRRTT	C	88	102	HLA-DRB1*14:02	0%	Dengue virus 2	ELISPOT	IFNg release	Positive	0	0	0	0	0	0	0	0	0	0	0	0	0	0	0	0	0	0	0	0	0	0	0	0	0	0	0	0	0	0	0	0	0	0	0	0	0	0	0	0	0	0	0	0	0	0	0	0	0
739182	IGRMLNIMNRRRRTA	C	88	102	HLA-DRB1*14:02	0%	Dengue virus 2	ELISPOT	IFNg release	Positive	0	0	0	0	0	0	0	0	0	0	0	0	0	0	0	0	0	0	0	0	0	0	0	0	0	0	0	0	0	0	0	0	0	0	0	0	0	0	0	0	0	0	0	0	0	0	0	0	0
739183	IGRMMNILNRRRRTA	C	88	102	HLA-DRB1*14:02	0%	Dengue virus 2	ELISPOT	IFNg release	Positive	0	0	0	0	0	0	0	0	0	0	0	0	0	0	0	0	0	0	0	0	0	0	0	0	0	0	0	0	0	0	0	0	0	0	0	0	0	0	0	0	0	0	0	0	0	0	0	0	0
740399	STPFNMLKRERNRVS	C	10	24	HLA-DRB1*14:02	0%	Dengue virus 2	ELISPOT	IFNg release	Positive	0	0	0	0	0	0	0	0	0	0	0	0	0	0	0	0	0	0	0	0	0	0	0	0	0	0	0	0	0	0	0	0	0	0	0	0	0	0	0	0	0	0	0	0	0	0	0	0	0
740579	VAFLRFLTISPTAGI	C	51	65	HLA-DRB1*04:07	0%	Dengue virus 2	ELISPOT	IFNg release	Positive	0	0	0	0	0	0	0	0	0	0	0	0	0	0	0	0	0	0	0	0	0	0	0	0	0	0	0	0	0	0	0	0	0	0	0	0	0	0	0	0	0	0	0	0	0	0	0	0	0
866992	GPLKLYMALVAFLRF	C	42	56	HLA-DRB5*01:01	0%	Dengue virus 2	ELISPOT	IFNg release	Positive	0	0	0	0	0	0	0	0	0	0	0	0	0	0	0	0	0	0	0	0	0	0	0	0	0	0	0	0	0	0	0	0	0	0	0	0	0	0	0	0	0	0	0	0	0	0	0	0	0
867734	LKGFRKEIGRMLNIL	C	81	95	HLA-DRB3*02:02	0%	Dengue virus 2	ELISPOT	IFNg release	Positive	0	0	0	0	0	0	0	0	0	0	0	0	0	0	0	0	0	0	0	0	0	0	0	0	0	0	0	0	0	0	0	0	0	0	0	0	0	0	0	0	0	0	0	0	0	0	0	0	0
867910	LRGFRKEIGRMLNIM	C	81	95	HLA-DRB3*02:02	0%	Dengue virus 2	ELISPOT	IFNg release	Positive	0	0	0	0	0	0	0	0	0	0	0	0	0	0	0	0	0	0	0	0	0	0	0	0	0	0	0	0	0	0	0	0	0	0	0	0	0	0	0	0	0	0	0	0	0	0	0	0	0
867911	LRGFRREIGRMLNIL	C	81	95	HLA-DRB3*02:02	0%	Dengue virus 2	ELISPOT	IFNg release	Positive	0	0	0	0	0	0	0	0	0	0	0	0	0	0	0	0	0	0	0	0	0	0	0	0	0	0	0	0	0	0	0	0	0	0	0	0	0	0	0	0	0	0	0	0	0	0	0	0	0
868674	RLFMALVAFLRFLTI	C	45	59	HLA-DPB1*04:02	0%	Dengue virus 2	ELISPOT	IFNg release	Positive	0	0	0	0	0	0	0	0	0	0	0	0	0	0	0	0	0	0	0	0	0	0	0	0	0	0	0	0	0	0	0	0	0	0	0	0	0	0	0	0	0	0	0	0	0	0	0	0	0
150280	HPGFTLMAAILAYTI	PreM	244	258	HLA-DRB1*01:01	0%	Dengue virus 2	ELISPOT	IFNg release	Positive	0	0	0	0	0	0	0	0	0	0	0	0	0	0	0	0	0	0	0	0	0	0	0	0	0	0	0	0	0	0	0	0	0	0	0	0	0	0	0	0	0	0	0	0	0	0	0	0	0
180708	QRIETWILRHPGFTM	PreM	121	135	HLA class II	0%	Dengue virus 2	ELISPOT	IFNg release	Positive-Low	0	0	0	0	0	0	0	0	0	0	0	0	0	0	0	0	0	0	0	0	0	0	0	0	0	0	0	0	0	0	0	0	0	0	0	0	0	0	0	0	0	0	0	0	0	0	0	0	0
195318	FQKILIFILLTAVAP	PreM	263	277	HLA-DRB1*07:01	0%	Dengue virus 2	ELISPOT	IFNg release	Positive	0	0	0	0	0	0	0	0	0	0	0	0	0	0	0	0	0	0	0	0	0	0	0	0	0	0	0	0	0	0	0	0	0	0	0	0	0	0	0	0	0	0	0	0	0	0	0	0	0
195320	FQRTLIFILLTAVAP	PreM	263	277	HLA-DRB1*07:01	0%	Dengue virus 2	ELISPOT	IFNg release	Positive	0	0	0	0	0	0	0	0	0	0	0	0	0	0	0	0	0	0	0	0	0	0	0	0	0	0	0	0	0	0	0	0	0	0	0	0	0	0	0	0	0	0	0	0	0	0	0	0	0
195448	HFQRALIFILLTAVA	PreM	262	276	HLA-DRB1*07:01	0%	Dengue virus 2	ELISPOT	IFNg release	Positive	0	0	0	0	0	0	0	0	0	0	0	0	0	0	0	0	0	0	0	0	0	0	0	0	0	0	0	0	0	0	0	0	0	0	0	0	0	0	0	0	0	0	0	0	0	0	0	0	0
195504	ILRHPGFTLMAAILA	PreM	241	255	HLA-DRB1*15:01	0%	Dengue virus 2	ELISPOT	IFNg release	Positive	0	0	0	0	0	0	0	0	0	0	0	0	0	0	0	0	0	0	0	0	0	0	0	0	0	0	0	0	0	0	0	0	0	0	0	0	0	0	0	0	0	0	0	0	0	0	0	0	0
196045	THFQRALIFILLTAV	PreM	261	275	HLA-DRB1*07:01	0%	Dengue virus 2	ELISPOT	IFNg release	Positive	0	0	0	0	0	0	0	0	0	0	0	0	0	0	0	0	0	0	0	0	0	0	0	0	0	0	0	0	0	0	0	0	0	0	0	0	0	0	0	0	0	0	0	0	0	0	0	0	0
196232	YFQRVLIFILLTAVT	PreM	262	276	HLA-DRB1*07:01	0%	Dengue virus 2	ELISPOT	IFNg release	Positive	0	0	0	0	0	0	0	0	0	0	0	0	0	0	0	0	0	0	0	0	0	0	0	0	0	0	0	0	0	0	0	0	0	0	0	0	0	0	0	0	0	0	0	0	0	0	0	0	0
739425	LAYTIGTTHFQRALI	PreM	254	268	HLA-DRB1*04:07	0%	Dengue virus 2	ELISPOT	IFNg release	Positive	0	0	0	0	0	0	0	0	0	0	0	0	0	0	0	0	0	0	0	0	0	0	0	0	0	0	0	0	0	0	0	0	0	0	0	0	0	0	0	0	0	0	0	0	0	0	0	0	0
866485	EDGVNMCTLMAIDLG	PreM	142	156	HLA-DQA1*01:02/DQB1*06:02	0%	Dengue virus 2	ELISPOT	IFNg release	Positive	0	0	0	0	0	0	0	0	0	0	0	0	0	0	0	0	0	0	0	0	0	0	0	0	0	0	0	0	0	0	0	0	0	0	0	0	0	0	0	0	0	0	0	0	0	0	0	0	0
868650	RHPGFTLMAAILAYT	PreM	243	257	HLA-DQA1*01:02/DQB1*06:02	0%	Dengue virus 2	ELISPOT	IFNg release	Positive	0	0	0	0	0	0	0	0	0	0	0	0	0	0	0	0	0	0	0	0	0	0	0	0	0	0	0	0	0	0	0	0	0	0	0	0	0	0	0	0	0	0	0	0	0	0	0	0	0
195793	NLEYTIMITPHSGEE	E	134	148	HLA-DRB1*04:01	0%	Dengue virus 2	ELISPOT	IFNg release	Positive	0	0	0	0	0	0	0	0	0	0	0	0	0	0	0	0	0	0	0	0	0	0	0	0	0	0	0	0	0	0	0	0	0	0	0	0	0	0	0	0	0	0	0	0	0	0	0	0	0
195952	SGVSWTMKILIGVII	E	449	463	HLA-DRB1*15:01	0%	Dengue virus 2	ELISPOT	IFNg release	Positive	0	0	0	0	0	0	0	0	0	0	0	0	0	0	0	0	0	0	0	0	0	0	0	0	0	0	0	0	0	0	0	0	0	0	0	0	0	0	0	0	0	0	0	0	0	0	0	0	0
196022	SWTMKILIGVIITWI	E	452	466	HLA-DRB1*08:02	0%	Dengue virus 2	ELISPOT	IFNg release	Positive	0	0	0	0	0	0	0	0	0	0	0	0	0	0	0	0	0	0	0	0	0	0	0	0	0	0	0	0	0	0	0	0	0	0	0	0	0	0	0	0	0	0	0	0	0	0	0	0	0
539427	IQKKTLVTFKNPHAK	E	512	526	HLA-DRB1*04:03	0%	Dengue virus 2	ELISPOT	IFNg release	Positive	0	0	0	0	0	0	0	0	0	0	0	0	0	0	0	0	0	0	0	0	0	0	0	0	0	0	0	0	0	0	0	0	0	0	0	0	0	0	0	0	0	0	0	0	0	0	0	0	0
539462	KEMLVTFKNPHAKRQ	E	514	528	HLA-DRB1*04:03	0%	Dengue virus 2	ELISPOT	IFNg release	Positive	0	0	0	0	0	0	0	0	0	0	0	0	0	0	0	0	0	0	0	0	0	0	0	0	0	0	0	0	0	0	0	0	0	0	0	0	0	0	0	0	0	0	0	0	0	0	0	0	0
539479	KKTLVTFKNPHAKKQ	E	514	528	HLA-DRB1*14:04	0%	Dengue virus 2	ELISPOT	IFNg release	Positive	0	0	0	0	0	0	0	0	0	0	0	0	0	0	0	0	0	0	0	0	0	0	0	0	0	0	0	0	0	0	0	0	0	0	0	0	0	0	0	0	0	0	0	0	0	0	0	0	0
539629	LSVTLVLVGFVTLYL	E	475	489	HLA-DRB1*15:06	0%	Dengue virus 2	ELISPOT	IFNg release	Positive	0	0	0	0	0	0	0	0	0	0	0	0	0	0	0	0	0	0	0	0	0	0	0	0	0	0	0	0	0	0	0	0	0	0	0	0	0	0	0	0	0	0	0	0	0	0	0	0	0
738866	EYTIVITPHSGEEHA	E	136	150	HLA-DRB1*04:07	0%	Dengue virus 2	ELISPOT	IFNg release	Positive	0	0	0	0	0	0	0	0	0	0	0	0	0	0	0	0	0	0	0	0	0	0	0	0	0	0	0	0	0	0	0	0	0	0	0	0	0	0	0	0	0	0	0	0	0	0	0	0	0
867375	KAWLVHRQWFLDLPF	E	204	218	HLA-DQA1*01:01/DQB1*05:01	0%	Dengue virus 2	ELISPOT	IFNg release	Positive	0	0	0	0	0	0	0	0	0	0	0	0	0	0	0	0	0	0	0	0	0	0	0	0	0	0	0	0	0	0	0	0	0	0	0	0	0	0	0	0	0	0	0	0	0	0	0	0	0
868347	PPFGDSYVIIGVEPG	E	371	385	HLA-DQA1*03:01/DQB1*03:02	0%	Dengue virus 2	ELISPOT	IFNg release	Positive	0	0	0	0	0	0	0	0	0	0	0	0	0	0	0	0	0	0	0	0	0	0	0	0	0	0	0	0	0	0	0	0	0	0	0	0	0	0	0	0	0	0	0	0	0	0	0	0	0
195954	SHNQTFLIDGPETAE	NS1	903	917	HLA-DRB1*04:01	0%	Dengue virus 2	ELISPOT	IFNg release	Positive	0	0	0	0	0	0	0	0	0	0	0	0	0	0	0	0	0	0	0	0	0	0	0	0	0	0	0	0	0	0	0	0	0	0	0	0	0	0	0	0	0	0	0	0	0	0	0	0	0
539708	NDTWKMEKASFIEVK	NS1	982	996	HLA-DRB1*09:01	0%	Dengue virus 2	ELISPOT	IFNg release	Positive	0	0	0	0	0	0	0	0	0	0	0	0	0	0	0	0	0	0	0	0	0	0	0	0	0	0	0	0	0	0	0	0	0	0	0	0	0	0	0	0	0	0	0	0	0	0	0	0	0
739191	IKGIMHAGKRSLRPQ	NS1	868	882	HLA-DRB1*14:02	0%	Dengue virus 2	ELISPOT	IFNg release	Positive	0	0	0	0	0	0	0	0	0	0	0	0	0	0	0	0	0	0	0	0	0	0	0	0	0	0	0	0	0	0	0	0	0	0	0	0	0	0	0	0	0	0	0	0	0	0	0	0	0
866699	FGVFTTNIWLKLREK	NS1	935	949	HLA-DPB1*04:01	0%	Dengue virus 2	ELISPOT	IFNg release	Positive	0	0	0	0	0	0	0	0	0	0	0	0	0	0	0	0	0	0	0	0	0	0	0	0	0	0	0	0	0	0	0	0	0	0	0	0	0	0	0	0	0	0	0	0	0	0	0	0	0
866829	GFGFFTTNIWLKLRE	NS1	934	948	HLA-DPB1*01:01	0%	Dengue virus 2	ELISPOT	IFNg release	Positive	0	0	0	0	0	0	0	0	0	0	0	0	0	0	0	0	0	0	0	0	0	0	0	0	0	0	0	0	0	0	0	0	0	0	0	0	0	0	0	0	0	0	0	0	0	0	0	0	0
866831	GFGVFTTNIWLKLRE	NS1	934	948	HLA-DPB1*04:01	0%	Dengue virus 2	ELISPOT	IFNg release	Positive	0	0	0	0	0	0	0	0	0	0	0	0	0	0	0	0	0	0	0	0	0	0	0	0	0	0	0	0	0	0	0	0	0	0	0	0	0	0	0	0	0	0	0	0	0	0	0	0	0
869722	YGFGVFTTNIWLKLR	NS1	933	947	HLA-DPB1*04:01	0%	Dengue virus 2	ELISPOT	IFNg release	Positive	0	0	0	0	0	0	0	0	0	0	0	0	0	0	0	0	0	0	0	0	0	0	0	0	0	0	0	0	0	0	0	0	0	0	0	0	0	0	0	0	0	0	0	0	0	0	0	0	0
150245	GMGVTYLALLAAYKV	NS2a	1196	1210	HLA-DRB1*01:01	0%	Dengue virus 2	ELISPOT	IFNg release	Positive	0	0	0	0	0	0	0	0	0	0	0	0	0	0	0	0	0	0	0	0	0	0	0	0	0	0	0	0	0	0	0	0	0	0	0	0	0	0	0	0	0	0	0	0	0	0	0	0	0
167819	SSQQKTDWIPLALTIKGLNP	NS2a	184	203	HLA-DP	0%	Dengue virus 2	ELISPOT	IFNg release	Positive	0	0	0	0	0	0	0	0	0	0	0	0	0	0	0	0	0	0	0	0	0	0	0	0	0	0	0	0	0	0	0	0	0	0	0	0	0	0	0	0	0	0	0	0	0	0	0	0	0
195269	ERYQLAVTITAILCV	NS2a	1268	1282	HLA-DRB1*07:01	0%	Dengue virus 2	ELISPOT	IFNg release	Positive	0	0	0	0	0	0	0	0	0	0	0	0	0	0	0	0	0	0	0	0	0	0	0	0	0	0	0	0	0	0	0	0	0	0	0	0	0	0	0	0	0	0	0	0	0	0	0	0	0
195523	IPLVLIIKGLNPTAI	NS2a	1319	1333	HLA-DRB1*08:02	0%	Dengue virus 2	ELISPOT	IFNg release	Positive	0	0	0	0	0	0	0	0	0	0	0	0	0	0	0	0	0	0	0	0	0	0	0	0	0	0	0	0	0	0	0	0	0	0	0	0	0	0	0	0	0	0	0	0	0	0	0	0	0
195712	LSLTFIRSTTPLIMA	NS2a	1275	1289	HLA-DRB1*07:01	0%	Dengue virus 2	ELISPOT	IFNg release	Positive	0	0	0	0	0	0	0	0	0	0	0	0	0	0	0	0	0	0	0	0	0	0	0	0	0	0	0	0	0	0	0	0	0	0	0	0	0	0	0	0	0	0	0	0	0	0	0	0	0
195763	MMVLKIVRKMERYQL	NS2a	1258	1272	HLA-DRB1*03:01	0%	Dengue virus 2	ELISPOT	IFNg release	Positive	0	0	0	0	0	0	0	0	0	0	0	0	0	0	0	0	0	0	0	0	0	0	0	0	0	0	0	0	0	0	0	0	0	0	0	0	0	0	0	0	0	0	0	0	0	0	0	0	0
196217	WKVSCTILAAVSVSP	NS2a	1292	1306	HLA-DRB1*04:01	0%	Dengue virus 2	ELISPOT	IFNg release	Positive	0	0	0	0	0	0	0	0	0	0	0	0	0	0	0	0	0	0	0	0	0	0	0	0	0	0	0	0	0	0	0	0	0	0	0	0	0	0	0	0	0	0	0	0	0	0	0	0	0
539077	ALGIMVLKMVRSMEK	NS2a	1255	1269	HLA-DRB1*12:02	0%	Dengue virus 2	ELISPOT	IFNg release	Positive	0	0	0	0	0	0	0	0	0	0	0	0	0	0	0	0	0	0	0	0	0	0	0	0	0	0	0	0	0	0	0	0	0	0	0	0	0	0	0	0	0	0	0	0	0	0	0	0	0
539082	ALTIKGLNPTAILLT	NS2a	1322	1336	HLA-DRB1*09:01	0%	Dengue virus 2	ELISPOT	IFNg release	Positive	0	0	0	0	0	0	0	0	0	0	0	0	0	0	0	0	0	0	0	0	0	0	0	0	0	0	0	0	0	0	0	0	0	0	0	0	0	0	0	0	0	0	0	0	0	0	0	0	0
539110	AWALGMMVLKIVRNM	NS2a	1253	1267	HLA-DRB1*13:01	0%	Dengue virus 2	ELISPOT	IFNg release	Positive	0	0	0	0	0	0	0	0	0	0	0	0	0	0	0	0	0	0	0	0	0	0	0	0	0	0	0	0	0	0	0	0	0	0	0	0	0	0	0	0	0	0	0	0	0	0	0	0	0
539392	IALGIMVLKMVRSME	NS2a	1254	1268	HLA-DRB1*13:01	0%	Dengue virus 2	ELISPOT	IFNg release	Positive	0	0	0	0	0	0	0	0	0	0	0	0	0	0	0	0	0	0	0	0	0	0	0	0	0	0	0	0	0	0	0	0	0	0	0	0	0	0	0	0	0	0	0	0	0	0	0	0	0
539556	LGIMILKLVRKMEKY	NS2a	1256	1270	HLA-DRB1*12:02	0%	Dengue virus 2	ELISPOT	IFNg release	Positive	0	0	0	0	0	0	0	0	0	0	0	0	0	0	0	0	0	0	0	0	0	0	0	0	0	0	0	0	0	0	0	0	0	0	0	0	0	0	0	0	0	0	0	0	0	0	0	0	0
539557	LGIMVLKIVRSMEKY	NS2a	1256	1270	HLA-DRB1*12:02	0%	Dengue virus 2	ELISPOT	IFNg release	Positive	0	0	0	0	0	0	0	0	0	0	0	0	0	0	0	0	0	0	0	0	0	0	0	0	0	0	0	0	0	0	0	0	0	0	0	0	0	0	0	0	0	0	0	0	0	0	0	0	0
539706	NAWKVSCTILAAVSV	NS2a	1290	1304	HLA-DRB1*09:01	0%	Dengue virus 2	ELISPOT	IFNg release	Positive	0	0	0	0	0	0	0	0	0	0	0	0	0	0	0	0	0	0	0	0	0	0	0	0	0	0	0	0	0	0	0	0	0	0	0	0	0	0	0	0	0	0	0	0	0	0	0	0	0
539893	SCTILAAVSVSPLLL	NS2a	1295	1309	HLA-DRB1*10:01	0%	Dengue virus 2	ELISPOT	IFNg release	Positive	0	0	0	0	0	0	0	0	0	0	0	0	0	0	0	0	0	0	0	0	0	0	0	0	0	0	0	0	0	0	0	0	0	0	0	0	0	0	0	0	0	0	0	0	0	0	0	0	0
195913	RTGLLVISGVFPVSI	NS2b	1445	1459	HLA-DRB1*08:02	0%	Dengue virus 2	ELISPOT	IFNg release	Positive	0	0	0	0	0	0	0	0	0	0	0	0	0	0	0	0	0	0	0	0	0	0	0	0	0	0	0	0	0	0	0	0	0	0	0	0	0	0	0	0	0	0	0	0	0	0	0	0	0
539574	LIRTGLLVISGVFPV	NS2b	1443	1457	HLA-DRB1*13:01	0%	Dengue virus 2	ELISPOT	IFNg release	Positive	0	0	0	0	0	0	0	0	0	0	0	0	0	0	0	0	0	0	0	0	0	0	0	0	0	0	0	0	0	0	0	0	0	0	0	0	0	0	0	0	0	0	0	0	0	0	0	0	0
24916	HTGREIVDLMCHATE	NS3	251	265	HLA-DPw2	0%	Dengue virus 2	ICS	IFNg release	Positive	0	0	0	0	0	0	0	0	0	0	0	0	0	0	0	0	0	0	0	0	0	0	0	0	0	0	0	0	0	0	0	0	0	0	0	0	0	0	0	0	0	0	0	0	0	0	0	0	0
539283	GAYRIKQRGILGYSQ	NS3	1496	1510	HLA-DRB1*08:03	0%	Dengue virus 2	ELISPOT	IFNg release	Positive	0	0	0	0	0	0	0	0	0	0	0	0	0	0	0	0	0	0	0	0	0	0	0	0	0	0	0	0	0	0	0	0	0	0	0	0	0	0	0	0	0	0	0	0	0	0	0	0	0
54390	RKLTIMDLHPGAGKT	NS3	618	632	HLA class II	0%	Dengue virus 2	ICS	IFNg release	Positive	0	0	0	0	0	0	0	0	0	0	0	0	0	0	0	0	0	0	0	0	0	0	0	0	0	0	0	0	0	0	0	0	0	0	0	0	0	0	0	0	0	0	0	0	0	0	0	0	0
868183	MWHVTRGAVLMHRGK	NS3	1524	1538	HLA-DRB3*02:02	0%	Dengue virus 2	ELISPOT	IFNg release	Positive	0	0	0	0	0	0	0	0	0	0	0	0	0	0	0	0	0	0	0	0	0	0	0	0	0	0	0	0	0	0	0	0	0	0	0	0	0	0	0	0	0	0	0	0	0	0	0	0	0
150192	ETLLLLTLLAAVTGG	NS4a	2146	2160	HLA-DRB1*01:01	0%	Dengue virus 2	ELISPOT	IFNg release	Positive	0	0	0	0	0	0	0	0	0	0	0	0	0	0	0	0	0	0	0	0	0	0	0	0	0	0	0	0	0	0	0	0	0	0	0	0	0	0	0	0	0	0	0	0	0	0	0	0	0
195810	NQLTYVVIAILTLVA	NS4a	2225	2239	HLA-DRB1*07:01	0%	Dengue virus 2	ELISPOT	IFNg release	Positive	0	0	0	0	0	0	0	0	0	0	0	0	0	0	0	0	0	0	0	0	0	0	0	0	0	0	0	0	0	0	0	0	0	0	0	0	0	0	0	0	0	0	0	0	0	0	0	0	0
195118	AGLLFSIMRNTTSAR	NS4b	2476	2490	HLA-DRB1*04:01	0%	Dengue virus 2	ELISPOT	IFNg release	Positive	0	0	0	0	0	0	0	0	0	0	0	0	0	0	0	0	0	0	0	0	0	0	0	0	0	0	0	0	0	0	0	0	0	0	0	0	0	0	0	0	0	0	0	0	0	0	0	0	0
195270	ESESNILDIDLRPAS	NS4b	2265	2279	HLA-DRB1*03:01	0%	Dengue virus 2	ELISPOT	IFNg release	Positive	0	0	0	0	0	0	0	0	0	0	0	0	0	0	0	0	0	0	0	0	0	0	0	0	0	0	0	0	0	0	0	0	0	0	0	0	0	0	0	0	0	0	0	0	0	0	0	0	0
195387	GLLFFIMKNTTNTRR	NS4b	2477	2491	HLA-DRB1*04:01	0%	Dengue virus 2	ELISPOT	IFNg release	Positive	0	0	0	0	0	0	0	0	0	0	0	0	0	0	0	0	0	0	0	0	0	0	0	0	0	0	0	0	0	0	0	0	0	0	0	0	0	0	0	0	0	0	0	0	0	0	0	0	0
195393	GLLFSIMKNTANTRR	NS4b	2463	2477	HLA-DRB1*04:01	0%	Dengue virus 2	ELISPOT	IFNg release	Positive	0	0	0	0	0	0	0	0	0	0	0	0	0	0	0	0	0	0	0	0	0	0	0	0	0	0	0	0	0	0	0	0	0	0	0	0	0	0	0	0	0	0	0	0	0	0	0	0	0
195394	GLLFSIMKNTTNARR	NS4b	2477	2491	HLA-DRB1*04:01	0%	Dengue virus 2	ELISPOT	IFNg release	Positive	0	0	0	0	0	0	0	0	0	0	0	0	0	0	0	0	0	0	0	0	0	0	0	0	0	0	0	0	0	0	0	0	0	0	0	0	0	0	0	0	0	0	0	0	0	0	0	0	0
195395	GLLFSIMKNTTNSRR	NS4b	2477	2491	HLA-DRB1*04:01	0%	Dengue virus 2	ELISPOT	IFNg release	Positive	0	0	0	0	0	0	0	0	0	0	0	0	0	0	0	0	0	0	0	0	0	0	0	0	0	0	0	0	0	0	0	0	0	0	0	0	0	0	0	0	0	0	0	0	0	0	0	0	0
195396	GLLFSIMKNTTSARR	NS4b	2477	2491	HLA-DRB1*04:01	0%	Dengue virus 2	ELISPOT	IFNg release	Positive	0	0	0	0	0	0	0	0	0	0	0	0	0	0	0	0	0	0	0	0	0	0	0	0	0	0	0	0	0	0	0	0	0	0	0	0	0	0	0	0	0	0	0	0	0	0	0	0	0
195398	GLLFSVMKNTTNTRR	NS4b	2477	2491	HLA-DRB1*04:01	0%	Dengue virus 2	ELISPOT	IFNg release	Positive	0	0	0	0	0	0	0	0	0	0	0	0	0	0	0	0	0	0	0	0	0	0	0	0	0	0	0	0	0	0	0	0	0	0	0	0	0	0	0	0	0	0	0	0	0	0	0	0	0
195659	LLFSIMRNTTTARRG	NS4b	2478	2492	HLA-DRB1*04:01	0%	Dengue virus 2	ELISPOT	IFNg release	Positive	0	0	0	0	0	0	0	0	0	0	0	0	0	0	0	0	0	0	0	0	0	0	0	0	0	0	0	0	0	0	0	0	0	0	0	0	0	0	0	0	0	0	0	0	0	0	0	0	0
539755	PASAWTLYAVATTFV	NS4b	2277	2291	HLA-DRB1*09:01	0%	Dengue virus 2	ELISPOT	IFNg release	Positive	0	0	0	0	0	0	0	0	0	0	0	0	0	0	0	0	0	0	0	0	0	0	0	0	0	0	0	0	0	0	0	0	0	0	0	0	0	0	0	0	0	0	0	0	0	0	0	0	0
867709	LILCVTQVLMMRTTW	NS4b	2417	2431	HLA-DPB1*04:02	0%	Dengue virus 2	ELISPOT	IFNg release	Positive	0	0	0	0	0	0	0	0	0	0	0	0	0	0	0	0	0	0	0	0	0	0	0	0	0	0	0	0	0	0	0	0	0	0	0	0	0	0	0	0	0	0	0	0	0	0	0	0	0
869090	TAIANQATVLMGLGR	NS4b	2309	2323	HLA-DQA1*01:02/DQB1*06:02	0%	Dengue virus 2	ELISPOT	IFNg release	Positive	0	0	0	0	0	0	0	0	0	0	0	0	0	0	0	0	0	0	0	0	0	0	0	0	0	0	0	0	0	0	0	0	0	0	0	0	0	0	0	0	0	0	0	0	0	0	0	0	0
167817	SRLNALGKSEFQI	NS5	15	27	HLA class II	0%	Dengue virus 2	ELISPOT	IFNg release	Positive	0	0	0	0	0	0	0	0	0	0	0	0	0	0	0	0	0	0	0	0	0	0	0	0	0	0	0	0	0	0	0	0	0	0	0	0	0	0	0	0	0	0	0	0	0	0	0	0	0
195111	ACLGKSYAQMWTLMY	NS5	3243	3257	HLA-DRB1*04:01	0%	Dengue virus 2	ELISPOT	IFNg release	Positive	0	0	0	0	0	0	0	0	0	0	0	0	0	0	0	0	0	0	0	0	0	0	0	0	0	0	0	0	0	0	0	0	0	0	0	0	0	0	0	0	0	0	0	0	0	0	0	0	0
195241	EGEGVFKSIQHLTVT	NS5	3101	3115	HLA-DRB1*07:01	0%	Dengue virus 2	ELISPOT	IFNg release	Positive	0	0	0	0	0	0	0	0	0	0	0	0	0	0	0	0	0	0	0	0	0	0	0	0	0	0	0	0	0	0	0	0	0	0	0	0	0	0	0	0	0	0	0	0	0	0	0	0	0
195250	ELIMKDGRKLVVPCR	NS5	3206	3220	HLA-DRB1*03:01	0%	Dengue virus 2	ELISPOT	IFNg release	Positive	0	0	0	0	0	0	0	0	0	0	0	0	0	0	0	0	0	0	0	0	0	0	0	0	0	0	0	0	0	0	0	0	0	0	0	0	0	0	0	0	0	0	0	0	0	0	0	0	0
195384	GLFKSIQHLTSQEEI	NS5	3118	3132	HLA-DRB1*11:01	0%	Dengue virus 2	ELISPOT	IFNg release	Positive	0	0	0	0	0	0	0	0	0	0	0	0	0	0	0	0	0	0	0	0	0	0	0	0	0	0	0	0	0	0	0	0	0	0	0	0	0	0	0	0	0	0	0	0	0	0	0	0	0
195445	HFHELIMKDGRILVV	NS5	3203	3217	HLA-DRB1*03:01	0%	Dengue virus 2	ELISPOT	IFNg release	Positive	0	0	0	0	0	0	0	0	0	0	0	0	0	0	0	0	0	0	0	0	0	0	0	0	0	0	0	0	0	0	0	0	0	0	0	0	0	0	0	0	0	0	0	0	0	0	0	0	0
195446	HFHELVMKDGRVLVV	NS5	3203	3217	HLA-DRB1*13:01	0%	Dengue virus 2	ELISPOT	IFNg release	Positive	0	0	0	0	0	0	0	0	0	0	0	0	0	0	0	0	0	0	0	0	0	0	0	0	0	0	0	0	0	0	0	0	0	0	0	0	0	0	0	0	0	0	0	0	0	0	0	0	0
195460	HRLGYILRDVSRKEG	NS5	3004	3018	HLA-DRB1*08:02	0%	Dengue virus 2	ELISPOT	IFNg release	Positive	0	0	0	0	0	0	0	0	0	0	0	0	0	0	0	0	0	0	0	0	0	0	0	0	0	0	0	0	0	0	0	0	0	0	0	0	0	0	0	0	0	0	0	0	0	0	0	0	0
195513	IMKDGRKLVVPCRNQ	NS5	3207	3221	HLA-DRB1*03:01	0%	Dengue virus 2	ELISPOT	IFNg release	Positive	0	0	0	0	0	0	0	0	0	0	0	0	0	0	0	0	0	0	0	0	0	0	0	0	0	0	0	0	0	0	0	0	0	0	0	0	0	0	0	0	0	0	0	0	0	0	0	0	0
196040	TGNIVSSVNMISRML	NS5	2716	2730	HLA-DRB1*04:01	0%	Dengue virus 2	ELISPOT	IFNg release	Positive	0	0	0	0	0	0	0	0	0	0	0	0	0	0	0	0	0	0	0	0	0	0	0	0	0	0	0	0	0	0	0	0	0	0	0	0	0	0	0	0	0	0	0	0	0	0	0	0	0
196225	WTLMYFHRRDLRLAA	NS5	3253	3267	HLA-DRB1*03:01	0%	Dengue virus 2	ELISPOT	IFNg release	Positive	0	0	0	0	0	0	0	0	0	0	0	0	0	0	0	0	0	0	0	0	0	0	0	0	0	0	0	0	0	0	0	0	0	0	0	0	0	0	0	0	0	0	0	0	0	0	0	0	0
32939	KPWDIIPMV	NS5	2821	2829	HLA class II	0%	Dengue virus 2	ELISA	IL-2 release	Positive	0	0	0	0	0	0	0	0	0	0	0	0	0	0	0	0	0	0	0	0	0	0	0	0	0	0	0	0	0	0	0	0	0	0	0	0	0	0	0	0	0	0	0	0	0	0	0	0	0
32941	KPWDVLPMV	NS5	2821	2829	HLA class II	0%	Dengue virus 2	ELISA	IL-2 release	Positive	0	0	0	0	0	0	0	0	0	0	0	0	0	0	0	0	0	0	0	0	0	0	0	0	0	0	0	0	0	0	0	0	0	0	0	0	0	0	0	0	0	0	0	0	0	0	0	0	0
32942	KPWDVLPTV	NS5	2821	2829	HLA class II	0%	Dengue virus 2	ELISA	IL-2 release	Positive	0	0	0	0	0	0	0	0	0	0	0	0	0	0	0	0	0	0	0	0	0	0	0	0	0	0	0	0	0	0	0	0	0	0	0	0	0	0	0	0	0	0	0	0	0	0	0	0	0
539161	DHWFSRGNSLSGVEG	NS5	2986	3000	HLA-DRB1*09:01	0%	Dengue virus 2	ELISPOT	IFNg release	Positive	0	0	0	0	0	0	0	0	0	0	0	0	0	0	0	0	0	0	0	0	0	0	0	0	0	0	0	0	0	0	0	0	0	0	0	0	0	0	0	0	0	0	0	0	0	0	0	0	0
539348	GSAKLRWFVERNMVT	NS5	2549	2563	HLA-DRB1*09:01	0%	Dengue virus 2	ELISPOT	IFNg release	Positive	0	0	0	0	0	0	0	0	0	0	0	0	0	0	0	0	0	0	0	0	0	0	0	0	0	0	0	0	0	0	0	0	0	0	0	0	0	0	0	0	0	0	0	0	0	0	0	0	0
539560	LGKNEFQIYKKSGIQ	NS5	2511	2525	HLA-DRB1*15:02	0%	Dengue virus 2	ELISPOT	IFNg release	Positive	0	0	0	0	0	0	0	0	0	0	0	0	0	0	0	0	0	0	0	0	0	0	0	0	0	0	0	0	0	0	0	0	0	0	0	0	0	0	0	0	0	0	0	0	0	0	0	0	0
869083	SYAQMWTLMYFHRRD	NS5	3248	3262	HLA-DPB1*04:01	0%	Dengue virus 2	ELISPOT	IFNg release	Positive	0	0	0	0	0	0	0	0	0	0	0	0	0	0	0	0	0	0	0	0	0	0	0	0	0	0	0	0	0	0	0	0	0	0	0	0	0	0	0	0	0	0	0	0	0	0	0	0	0
869148	TFVESWEDIPYLGKR	NS5	3319	3333	HLA-DQA1*01:01/DQB1*05:01	0%	Dengue virus 2	ELISPOT	IFNg release	Positive	0	0	0	0	0	0	0	0	0	0	0	0	0	0	0	0	0	0	0	0	0	0	0	0	0	0	0	0	0	0	0	0	0	0	0	0	0	0	0	0	0	0	0	0	0	0	0	0	0
869292	TPVESWEEVPYLGKR	NS5	3319	3333	HLA-DQA1*01:01/DQB1*05:01	0%	Dengue virus 2	ELISPOT	IFNg release	Positive	0	0	0	0	0	0	0	0	0	0	0	0	0	0	0	0	0	0	0	0	0	0	0	0	0	0	0	0	0	0	0	0	0	0	0	0	0	0	0	0	0	0	0	0	0	0	0	0	0
195326	FRKEIGRMLNILNRR	C	84	98	HLA-DRB1*04:01	100%	Dengue virus 2	ELISPOT	IFNg release	Positive	1	1	1	1	1	1	1	1	1	1	1	1	1	1	1	1	1	1	1	1	1	1	1	1	1	1	1	1	1	1	1	1	1	1	1	1	1	1	1	1	1	1	1	1	1	1	1	1	1
195556	KEIGRMLNILNRRRR	C	86	100	HLA-DRB1*08:02	100%	Dengue virus 2	ELISPOT	IFNg release	Positive	1	1	1	1	1	1	1	1	1	1	1	1	1	1	1	1	1	1	1	1	1	1	1	1	1	1	1	1	1	1	1	1	1	1	1	1	1	1	1	1	1	1	1	1	1	1	1	1	1
195886	RGFRKEIGRMLNILN	C	82	96	HLA-DRB1*04:01	100%	Dengue virus 2	ELISPOT	IFNg release	Positive	1	1	1	1	1	1	1	1	1	1	1	1	1	1	1	1	1	1	1	1	1	1	1	1	1	1	1	1	1	1	1	1	1	1	1	1	1	1	1	1	1	1	1	1	1	1	1	1	1
39098	LRGFRKEIGRML	C	81	92	HLA-DPw4	100%	Dengue virus 2	ICS	IFNg release	Positive	1	1	1	1	1	1	1	1	1	1	1	1	1	1	1	1	1	1	1	1	1	1	1	1	1	1	1	1	1	1	1	1	1	1	1	1	1	1	1	1	1	1	1	1	1	1	1	1	1
539214	EIGRMLNILNRRRRT	C	87	101	HLA-DRB1*12:02	100%	Dengue virus 2	ELISPOT	IFNg release	Positive	1	1	1	1	1	1	1	1	1	1	1	1	1	1	1	1	1	1	1	1	1	1	1	1	1	1	1	1	1	1	1	1	1	1	1	1	1	1	1	1	1	1	1	1	1	1	1	1	1
539618	LRGFRKEIGRMLNIL	C	81	95	HLA-DRB1*09:01	100%	Dengue virus 2	ELISPOT	IFNg release	Positive	1	1	1	1	1	1	1	1	1	1	1	1	1	1	1	1	1	1	1	1	1	1	1	1	1	1	1	1	1	1	1	1	1	1	1	1	1	1	1	1	1	1	1	1	1	1	1	1	1
540060	VLRGFRKEIGRMLNI	C	80	94	HLA-DRB1*09:01	100%	Dengue virus 2	ELISPOT	IFNg release	Positive	1	1	1	1	1	1	1	1	1	1	1	1	1	1	1	1	1	1	1	1	1	1	1	1	1	1	1	1	1	1	1	1	1	1	1	1	1	1	1	1	1	1	1	1	1	1	1	1	1
180590	LDFELIKTEAKQPAT	E	41	55	HLA class II	100%	Dengue virus 2	ELISPOT	IFNg release	Positive	1	1	1	1	1	1	1	1	1	1	1	1	1	1	1	1	1	1	1	1	1	1	1	1	1	1	1	1	1	1	1	1	1	1	1	1	1	1	1	1	1	1	1	1	1	1	1	1	1
195351	GATEIQMSSGNLLFT	E	266	280	HLA-DRB1*03:01	100%	Dengue virus 2	ELISPOT	IFNg release	Positive	1	1	1	1	1	1	1	1	1	1	1	1	1	1	1	1	1	1	1	1	1	1	1	1	1	1	1	1	1	1	1	1	1	1	1	1	1	1	1	1	1	1	1	1	1	1	1	1	1
195366	GHLKCRLRMDKLQLK	E	281	295	HLA-DRB1*03:01	100%	Dengue virus 2	ELISPOT	IFNg release	Positive	1	1	1	1	1	1	1	1	1	1	1	1	1	1	1	1	1	1	1	1	1	1	1	1	1	1	1	1	1	1	1	1	1	1	1	1	1	1	1	1	1	1	1	1	1	1	1	1	1
195407	GMNSRSTSLSVSLVL	E	467	481	HLA-DRB1*07:01	100%	Dengue virus 2	ELISPOT	IFNg release	Positive	1	1	1	1	1	1	1	1	1	1	1	1	1	1	1	1	1	1	1	1	1	1	1	1	1	1	1	1	1	1	1	1	1	1	1	1	1	1	1	1	1	1	1	1	1	1	1	1	1
539648	LVTFKNPHAKKQDVV	E	237	251	HLA-DRB1*15:02	100%	Dengue virus 2	ELISPOT	IFNg release	Positive	1	1	1	1	1	1	1	1	1	1	1	1	1	1	1	1	1	1	1	1	1	1	1	1	1	1	1	1	1	1	1	1	1	1	1	1	1	1	1	1	1	1	1	1	1	1	1	1	1
539689	MRGAKRMAILGDTAW	E	406	420	HLA-DRB1*15:06	100%	Dengue virus 2	ELISPOT	IFNg release	Positive	1	1	1	1	1	1	1	1	1	1	1	1	1	1	1	1	1	1	1	1	1	1	1	1	1	1	1	1	1	1	1	1	1	1	1	1	1	1	1	1	1	1	1	1	1	1	1	1	1
741744	MRCIGISNRDFVEGVSGGSW	E	281	300	HLA class II	100%	Dengue virus 2	ELISPOT	IFNg release	Positive	1	1	1	1	1	1	1	1	1	1	1	1	1	1	1	1	1	1	1	1	1	1	1	1	1	1	1	1	1	1	1	1	1	1	1	1	1	1	1	1	1	1	1	1	1	1	1	1	1
866193	ATEIQMSSGNLLFTG	E	257	271	HLA-DRB3*02:02	100%	Dengue virus 2	ELISPOT	IFNg release	Positive	1	1	1	1	1	1	1	1	1	1	1	1	1	1	1	1	1	1	1	1	1	1	1	1	1	1	1	1	1	1	1	1	1	1	1	1	1	1	1	1	1	1	1	1	1	1	1	1	1
150273	GVTYLALLAAFKVRP	NS2a	1198	1212	HLA-DRB1*01:01	100%	Dengue virus 2	ELISPOT	IFNg release	Positive	1	1	1	1	1	1	1	1	1	1	1	1	1	1	1	1	1	1	1	1	1	1	1	1	1	1	1	1	1	1	1	1	1	1	1	1	1	1	1	1	1	1	1	1	1	1	1	1	1
540094	VTYLALLAAFKVRPT	NS2a	1199	1213	HLA-DRB1*12:02	100%	Dengue virus 2	ELISPOT	IFNg release	Positive	1	1	1	1	1	1	1	1	1	1	1	1	1	1	1	1	1	1	1	1	1	1	1	1	1	1	1	1	1	1	1	1	1	1	1	1	1	1	1	1	1	1	1	1	1	1	1	1	1
150433	MAVGMVSILASSLLK	NS2b	1354	1368	HLA-DRB1*01:01	100%	Dengue virus 2	ELISPOT	IFNg release	Positive	1	1	1	1	1	1	1	1	1	1	1	1	1	1	1	1	1	1	1	1	1	1	1	1	1	1	1	1	1	1	1	1	1	1	1	1	1	1	1	1	1	1	1	1	1	1	1	1	1
195512	IMAVGMVSILASSLL	NS2b	8	22	HLA-DRB1*07:01	100%	Dengue virus 2	ELISPOT	IFNg release	Positive	1	1	1	1	1	1	1	1	1	1	1	1	1	1	1	1	1	1	1	1	1	1	1	1	1	1	1	1	1	1	1	1	1	1	1	1	1	1	1	1	1	1	1	1	1	1	1	1	1
195992	SSLLKNDIPMTGPLV	NS2b	19	33	HLA-DRB1*03:01	100%	Dengue virus 2	ELISPOT	IFNg release	Positive	1	1	1	1	1	1	1	1	1	1	1	1	1	1	1	1	1	1	1	1	1	1	1	1	1	1	1	1	1	1	1	1	1	1	1	1	1	1	1	1	1	1	1	1	1	1	1	1	1
866986	GMVSILASSLLKNDI	NS2b	1357	1371	HLA-DPB1*01:01	100%	Dengue virus 2	ELISPOT	IFNg release	Positive	1	1	1	1	1	1	1	1	1	1	1	1	1	1	1	1	1	1	1	1	1	1	1	1	1	1	1	1	1	1	1	1	1	1	1	1	1	1	1	1	1	1	1	1	1	1	1	1	1
1067121	GKVVGLYGNGVVTRS	NS3	1619	1633	HLA-DRB1*15:01	100%	Dengue virus 2	ELISPOT	IFNg release	Positive	1	1	1	1	1	1	1	1	1	1	1	1	1	1	1	1	1	1	1	1	1	1	1	1	1	1	1	1	1	1	1	1	1	1	1	1	1	1	1	1	1	1	1	1	1	1	1	1	1
12372	EHTGREIVDLMCHAT	NS3	250	264	HLA class II	100%	Dengue virus 2	ELISPOT	IFNg release	Positive	1	1	1	1	1	1	1	1	1	1	1	1	1	1	1	1	1	1	1	1	1	1	1	1	1	1	1	1	1	1	1	1	1	1	1	1	1	1	1	1	1	1	1	1	1	1	1	1	1
12606	EIVDLMCHATFTMRL	NS3	255	269	HLA class II	100%	Dengue virus 2	ELISPOT	IFNg release	Positive	1	1	1	1	1	1	1	1	1	1	1	1	1	1	1	1	1	1	1	1	1	1	1	1	1	1	1	1	1	1	1	1	1	1	1	1	1	1	1	1	1	1	1	1	1	1	1	1	1
1392191	GREIVDLMCHATFTM	NS3	1728	1742		100%	Dengue virus 2	biological activity	activation	Positive	1	1	1	1	1	1	1	1	1	1	1	1	1	1	1	1	1	1	1	1	1	1	1	1	1	1	1	1	1	1	1	1	1	1	1	1	1	1	1	1	1	1	1	1	1	1	1	1	1
139758	GKTKRYLPAIVREAI	NS3	1673	1687	HLA-DRB1*09:01	100%	Dengue virus 2	ELISPOT	IFNg release	Positive	1	1	1	1	1	1	1	1	1	1	1	1	1	1	1	1	1	1	1	1	1	1	1	1	1	1	1	1	1	1	1	1	1	1	1	1	1	1	1	1	1	1	1	1	1	1	1	1	1
180698	PNYNLIIMDEAHFTD	NS3	1751	1765	HLA class II	100%	Dengue virus 2	ELISPOT	IFNg release	Positive	1	1	1	1	1	1	1	1	1	1	1	1	1	1	1	1	1	1	1	1	1	1	1	1	1	1	1	1	1	1	1	1	1	1	1	1	1	1	1	1	1	1	1	1	1	1	1	1	1
195295	FHTMWHVTRGAVLMH	NS3	1521	1535	HLA-DRB1*07:01	100%	Dengue virus 2	ELISPOT	IFNg release	Positive	1	1	1	1	1	1	1	1	1	1	1	1	1	1	1	1	1	1	1	1	1	1	1	1	1	1	1	1	1	1	1	1	1	1	1	1	1	1	1	1	1	1	1	1	1	1	1	1	1
195420	GTFHTMWHVTRGAVL	NS3	1519	1533	HLA-DRB1*07:01	100%	Dengue virus 2	ELISPOT	IFNg release	Positive	1	1	1	1	1	1	1	1	1	1	1	1	1	1	1	1	1	1	1	1	1	1	1	1	1	1	1	1	1	1	1	1	1	1	1	1	1	1	1	1	1	1	1	1	1	1	1	1	1
195888	RGLRTLILAPTRVVA	NS3	1689	1703	HLA-DRB1*08:02	100%	Dengue virus 2	ELISPOT	IFNg release	Positive	1	1	1	1	1	1	1	1	1	1	1	1	1	1	1	1	1	1	1	1	1	1	1	1	1	1	1	1	1	1	1	1	1	1	1	1	1	1	1	1	1	1	1	1	1	1	1	1	1
21115	GLRTLILAPTRVVAA	NS3	1690	1704	HLA-DRB1*04:03	100%	Dengue virus 2	ELISPOT	IFNg release	Positive	1	1	1	1	1	1	1	1	1	1	1	1	1	1	1	1	1	1	1	1	1	1	1	1	1	1	1	1	1	1	1	1	1	1	1	1	1	1	1	1	1	1	1	1	1	1	1	1	1
26965	ILAPTRVVAAEMEEA	NS3	1695	1709	HLA-DQA1*03:01/DQB1*03:02	100%	Dengue virus 2	ELISPOT	IFNg release	Positive	1	1	1	1	1	1	1	1	1	1	1	1	1	1	1	1	1	1	1	1	1	1	1	1	1	1	1	1	1	1	1	1	1	1	1	1	1	1	1	1	1	1	1	1	1	1	1	1	1
34231	KVVGLYGNGVVTRSG	NS3	145	159	HLA class II	100%	Dengue virus 2	ELISPOT	IFNg release	Positive	1	1	1	1	1	1	1	1	1	1	1	1	1	1	1	1	1	1	1	1	1	1	1	1	1	1	1	1	1	1	1	1	1	1	1	1	1	1	1	1	1	1	1	1	1	1	1	1	1
36560	LIIMDEAHFTDPASI	NS3	1755	1769	HLA-DQA1*01:01/DQB1*05:01	100%	Dengue virus 2	ELISPOT	IFNg release	Positive	1	1	1	1	1	1	1	1	1	1	1	1	1	1	1	1	1	1	1	1	1	1	1	1	1	1	1	1	1	1	1	1	1	1	1	1	1	1	1	1	1	1	1	1	1	1	1	1	1
39225	LRTLILAPTRVVAAE	NS3	1691	1705	HLA-DRB1*12:02	100%	Dengue virus 2	ELISPOT	IFNg release	Positive	1	1	1	1	1	1	1	1	1	1	1	1	1	1	1	1	1	1	1	1	1	1	1	1	1	1	1	1	1	1	1	1	1	1	1	1	1	1	1	1	1	1	1	1	1	1	1	1	1
49766	PTRVVAAEMEEALRG	NS3	223	237	HLA-DRB1*15:01	100%	Dengue virus 2	ICS	IFNg release	Positive	1	1	1	1	1	1	1	1	1	1	1	1	1	1	1	1	1	1	1	1	1	1	1	1	1	1	1	1	1	1	1	1	1	1	1	1	1	1	1	1	1	1	1	1	1	1	1	1	1
539025	AAGIFMTATPPGSRD	NS3	1784	1798	HLA-DRB1*04:03	100%	Dengue virus 2	ELISPOT	IFNg release	Positive	1	1	1	1	1	1	1	1	1	1	1	1	1	1	1	1	1	1	1	1	1	1	1	1	1	1	1	1	1	1	1	1	1	1	1	1	1	1	1	1	1	1	1	1	1	1	1	1	1
539041	AEMEEALRGLPIRYQ	NS3	1704	1718	HLA-DRB1*13:01	100%	Dengue virus 2	ELISPOT	IFNg release	Positive	1	1	1	1	1	1	1	1	1	1	1	1	1	1	1	1	1	1	1	1	1	1	1	1	1	1	1	1	1	1	1	1	1	1	1	1	1	1	1	1	1	1	1	1	1	1	1	1	1
539289	GEAAGIFMTATPPGS	NS3	1782	1796	HLA-DRB1*04:03	100%	Dengue virus 2	ELISPOT	IFNg release	Positive	1	1	1	1	1	1	1	1	1	1	1	1	1	1	1	1	1	1	1	1	1	1	1	1	1	1	1	1	1	1	1	1	1	1	1	1	1	1	1	1	1	1	1	1	1	1	1	1	1
539448	IVREAIKRGLRTLIL	NS3	1682	1696	HLA-DRB1*12:02	100%	Dengue virus 2	ELISPOT	IFNg release	Positive	1	1	1	1	1	1	1	1	1	1	1	1	1	1	1	1	1	1	1	1	1	1	1	1	1	1	1	1	1	1	1	1	1	1	1	1	1	1	1	1	1	1	1	1	1	1	1	1	1
539573	LILAPTRVVAAEMEE	NS3	1694	1708	HLA-DRB1*13:01	100%	Dengue virus 2	ELISPOT	IFNg release	Positive	1	1	1	1	1	1	1	1	1	1	1	1	1	1	1	1	1	1	1	1	1	1	1	1	1	1	1	1	1	1	1	1	1	1	1	1	1	1	1	1	1	1	1	1	1	1	1	1	1
540056	VLMHKGKRIEPSWAD	NS3	1518	1532	HLA-DRB1*13:01	100%	Dengue virus 2	ELISPOT	IFNg release	Positive	1	1	1	1	1	1	1	1	1	1	1	1	1	1	1	1	1	1	1	1	1	1	1	1	1	1	1	1	1	1	1	1	1	1	1	1	1	1	1	1	1	1	1	1	1	1	1	1	1
63672	TFHTMWHVTRGAVLM	NS3	45	59	HLA class II	100%	Dengue virus 2	ELISPOT	IFNg release	Positive	1	1	1	1	1	1	1	1	1	1	1	1	1	1	1	1	1	1	1	1	1	1	1	1	1	1	1	1	1	1	1	1	1	1	1	1	1	1	1	1	1	1	1	1	1	1	1	1	1
64666	TKRYLPAIVREAIKR	NS3	200	214	HLA class II	100%	Dengue virus 2	ELISPOT	IFNg release	Positive	1	1	1	1	1	1	1	1	1	1	1	1	1	1	1	1	1	1	1	1	1	1	1	1	1	1	1	1	1	1	1	1	1	1	1	1	1	1	1	1	1	1	1	1	1	1	1	1	1
740081	REAIKRGLRTLILAP	NS3	1670	1684	HLA-DRB1*14:06	100%	Dengue virus 2	ELISPOT	IFNg release	Positive	1	1	1	1	1	1	1	1	1	1	1	1	1	1	1	1	1	1	1	1	1	1	1	1	1	1	1	1	1	1	1	1	1	1	1	1	1	1	1	1	1	1	1	1	1	1	1	1	1
740193	RTLILAPTRVVAAEM	NS3	1678	1692	HLA-DRB1*14:06	100%	Dengue virus 2	ELISPOT	IFNg release	Positive	1	1	1	1	1	1	1	1	1	1	1	1	1	1	1	1	1	1	1	1	1	1	1	1	1	1	1	1	1	1	1	1	1	1	1	1	1	1	1	1	1	1	1	1	1	1	1	1	1
866477	EALRGLPIRYQTPAI	NS3	1708	1722	HLA-DRB4*01:01	100%	Dengue virus 2	ELISPOT	IFNg release	Positive	1	1	1	1	1	1	1	1	1	1	1	1	1	1	1	1	1	1	1	1	1	1	1	1	1	1	1	1	1	1	1	1	1	1	1	1	1	1	1	1	1	1	1	1	1	1	1	1	1
868734	RYLPAIVREAIKRGL	NS3	1677	1691	HLA-DRB4*01:01	100%	Dengue virus 2	ELISPOT	IFNg release	Positive	1	1	1	1	1	1	1	1	1	1	1	1	1	1	1	1	1	1	1	1	1	1	1	1	1	1	1	1	1	1	1	1	1	1	1	1	1	1	1	1	1	1	1	1	1	1	1	1	1
869224	TLILAPTRVVAAEME	NS3	1693	1707	HLA-DQA1*01:02/DQB1*06:02	100%	Dengue virus 2	ELISPOT	IFNg release	Positive	1	1	1	1	1	1	1	1	1	1	1	1	1	1	1	1	1	1	1	1	1	1	1	1	1	1	1	1	1	1	1	1	1	1	1	1	1	1	1	1	1	1	1	1	1	1	1	1	1
150362	LETLLLLTLLATVTG	NS4a	2145	2159	HLA-DRB1*01:01	100%	Dengue virus 2	ELISPOT	IFNg release	Positive	1	1	1	1	1	1	1	1	1	1	1	1	1	1	1	1	1	1	1	1	1	1	1	1	1	1	1	1	1	1	1	1	1	1	1	1	1	1	1	1	1	1	1	1	1	1	1	1	1
195107	AASIILEFFLIVLLI	NS4a	2185	2199	HLA-DRB1*15:01	100%	Dengue virus 2	ELISPOT	IFNg release	Positive	1	1	1	1	1	1	1	1	1	1	1	1	1	1	1	1	1	1	1	1	1	1	1	1	1	1	1	1	1	1	1	1	1	1	1	1	1	1	1	1	1	1	1	1	1	1	1	1	1
195767	MTLGMCCIITASILL	NS4a	2160	2174	HLA-DRB1*07:01	100%	Dengue virus 2	ELISPOT	IFNg release	Positive	1	1	1	1	1	1	1	1	1	1	1	1	1	1	1	1	1	1	1	1	1	1	1	1	1	1	1	1	1	1	1	1	1	1	1	1	1	1	1	1	1	1	1	1	1	1	1	1	1
196054	TLETLLLLTLLATVT	NS4a	2144	2158	HLA-DRB1*15:01	100%	Dengue virus 2	ELISPOT	IFNg release	Positive	1	1	1	1	1	1	1	1	1	1	1	1	1	1	1	1	1	1	1	1	1	1	1	1	1	1	1	1	1	1	1	1	1	1	1	1	1	1	1	1	1	1	1	1	1	1	1	1	1
738567	AYNHALSELPETLET	NS4a	2119	2133	HLA-DRB1*04:07	100%	Dengue virus 2	ELISPOT	IFNg release	Positive	1	1	1	1	1	1	1	1	1	1	1	1	1	1	1	1	1	1	1	1	1	1	1	1	1	1	1	1	1	1	1	1	1	1	1	1	1	1	1	1	1	1	1	1	1	1	1	1	1
195882	RFWNTTIAVSMANIF	NS4b	2454	2468	HLA-DRB1*07:01	100%	Dengue virus 2	ELISPOT	IFNg release	Positive	1	1	1	1	1	1	1	1	1	1	1	1	1	1	1	1	1	1	1	1	1	1	1	1	1	1	1	1	1	1	1	1	1	1	1	1	1	1	1	1	1	1	1	1	1	1	1	1	1
195974	SNILDIDLRPASAWT	NS4b	2155	2169	HLA-DRB1*03:01	100%	Dengue virus 2	ELISPOT	IFNg release	Positive	1	1	1	1	1	1	1	1	1	1	1	1	1	1	1	1	1	1	1	1	1	1	1	1	1	1	1	1	1	1	1	1	1	1	1	1	1	1	1	1	1	1	1	1	1	1	1	1	1
539724	NIFRGSYLAGAGLLF	NS4b	2452	2466	HLA-DRB1*09:01	100%	Dengue virus 2	ELISPOT	IFNg release	Positive	1	1	1	1	1	1	1	1	1	1	1	1	1	1	1	1	1	1	1	1	1	1	1	1	1	1	1	1	1	1	1	1	1	1	1	1	1	1	1	1	1	1	1	1	1	1	1	1	1
739437	LDIDLRPASAWTLYA	NS4b	2257	2271	HLA-DRB1*01:02	100%	Dengue virus 2	ELISPOT	IFNg release	Positive	1	1	1	1	1	1	1	1	1	1	1	1	1	1	1	1	1	1	1	1	1	1	1	1	1	1	1	1	1	1	1	1	1	1	1	1	1	1	1	1	1	1	1	1	1	1	1	1	1
866802	FWNTTIAVSMANIFR	NS4b	2455	2469	HLA-DQA1*01:02/DQB1*06:02	100%	Dengue virus 2	ELISPOT	IFNg release	Positive	1	1	1	1	1	1	1	1	1	1	1	1	1	1	1	1	1	1	1	1	1	1	1	1	1	1	1	1	1	1	1	1	1	1	1	1	1	1	1	1	1	1	1	1	1	1	1	1	1
869089	TAIANQATVLMGLGK	NS4b	2309	2323	HLA-DQA1*01:02/DQB1*06:02	100%	Dengue virus 2	ELISPOT	IFNg release	Positive	1	1	1	1	1	1	1	1	1	1	1	1	1	1	1	1	1	1	1	1	1	1	1	1	1	1	1	1	1	1	1	1	1	1	1	1	1	1	1	1	1	1	1	1	1	1	1	1	1
190862	MVTQMAMTDTTPFGQQR	NS5	2828	2844	HLA class II	100%	Dengue virus 2	ICS	IFNg release	Positive	1	1	1	1	1	1	1	1	1	1	1	1	1	1	1	1	1	1	1	1	1	1	1	1	1	1	1	1	1	1	1	1	1	1	1	1	1	1	1	1	1	1	1	1	1	1	1	1	1
195200	CETCVYNMMGKREKK	NS5	2924	2938	HLA-DRB1*11:01	100%	Dengue virus 2	ELISPOT	IFNg release	Positive	1	1	1	1	1	1	1	1	1	1	1	1	1	1	1	1	1	1	1	1	1	1	1	1	1	1	1	1	1	1	1	1	1	1	1	1	1	1	1	1	1	1	1	1	1	1	1	1	1
195213	DELIGRARISQGAGW	NS5	3223	3237	HLA-DRB1*08:02	100%	Dengue virus 2	ELISPOT	IFNg release	Positive	1	1	1	1	1	1	1	1	1	1	1	1	1	1	1	1	1	1	1	1	1	1	1	1	1	1	1	1	1	1	1	1	1	1	1	1	1	1	1	1	1	1	1	1	1	1	1	1	1
195233	EDHWFSRENSLSGVE	NS5	2985	2999	HLA-DRB1*04:01	100%	Dengue virus 2	ELISPOT	IFNg release	Positive	1	1	1	1	1	1	1	1	1	1	1	1	1	1	1	1	1	1	1	1	1	1	1	1	1	1	1	1	1	1	1	1	1	1	1	1	1	1	1	1	1	1	1	1	1	1	1	1	1

195409	GNIVSSVNMISRMLI	NS5	2717	2731	HLA-DRB1*07:01	100%	Dengue virus 2	ELISPOT	IFNg release	Positive
195948	SGNIVSSVNMISRML	NS5	2716	2730	HLA-DRB1*04:01	100%	Dengue virus 2	ELISPOT	IFNg release	Positive
196187	VSSVNMISRMLINRF	NS5	2720	2734	HLA-DRB1*15:01	100%	Dengue virus 2	ELISPOT	IFNg release	Positive
196210	WAKNIQTAINQVRSL	NS5	3336	3350	HLA-DRB1*03:01	100%	Dengue virus 2	ELISPOT	IFNg release	Positive
539436	ISGDDCVVKPLDDRFA	NS5	3151	3166	HLA class II	100%	Dengue virus 2	ICS	IFNg release	Positive
539871	RRDLRLAANAICSAV	NS5	3260	3274	HLA-DRB3*02:02	100%	Dengue virus 2	ELISPOT	IFNg release	Positive
866172	ASGNIVSSVNMISRM	NS5	2715	2729	HLA-DRB3*02:02	100%	Dengue virus 2	ELISPOT	IFNg release	Positive
867600	LEFEALGFLNEDHWF	NS5	2975	2989	HLA-DPB1*04:01	100%	Dengue virus 2	ELISPOT	IFNg release	Positive
868637	RDLRLAANAICSAVP	NS5	3261	3275	HLA-DQA1*05:01/DQB1*02:01	100%	Dengue virus 2	ELISPOT	IFNg release	Positive
868640	RFLEFEALGFLNEDH	NS5	2973	2987	HLA-DPB1*01:01	100%	Dengue virus 2	ELISPOT	IFNg release	Positive
869426	VESWEEIPYLGKRED	NS5	3321	3335	HLA-DQA1*01:01/DQB1*05:01	100%	Dengue virus 2	ELISPOT	IFNg release	Positive
1066603	ARNTPFNMLKRERNR	C	8	22	HLA-DRB1*11:01	81% - <100%	Dengue virus 2	ELISPOT	IFNg release	Positive-Low
150105	AFLRFLTIPPTAGIL	C	52	66	HLA-DRB1*01:01	81% - <100%	Dengue virus 2	ELISPOT	IFNg release	Positive
150369	LKLFMALVAFLRFLT	C	44	58	HLA-DRB1*01:01	81% - <100%	Dengue virus 2	ELISPOT	IFNg release	Positive
180597	LFMALVAFLRFLTIP	C	46	60	HLA class II	81% - <100%	Dengue virus 2	ELISPOT	IFNg release	Positive
180768	TIKKSKAINVLRGFR	C	71	85	HLA class II	81% - <100%	Dengue virus 2	ELISPOT	IFNg release	Positive
195307	FMALVAFLRFLTIPP	C	47	61	HLA-DRB1*11:01	81% - <100%	Dengue virus 2	ELISPOT	IFNg release	Positive
195573	KLFMALVAFLRFLTI	C	45	59	HLA-DRB1*15:01	81% - <100%	Dengue virus 2	ELISPOT	IFNg release	Positive
195730	LVAFLRFLTIPPTAG	C	50	64	HLA-DRB1*04:01	81% - <100%	Dengue virus 2	ELISPOT	IFNg release	Positive
195744	MALVAFLRFLTIPPT	C	48	62	HLA-DRB1*04:01	81% - <100%	Dengue virus 2	ELISPOT	IFNg release	Positive
195889	RGPLKLFMALVAFLR	C	41	55	HLA-DRB1*04:01	81% - <100%	Dengue virus 2	ELISPOT	IFNg release	Positive
196064	TPFNMLKRERNRVST	C	11	25	HLA-DRB1*08:02	81% - <100%	Dengue virus 2	ELISPOT	IFNg release	Positive
196093	VAFLRFLTIPPTAGI	C	51	65	HLA-DRB1*04:01	81% - <100%	Dengue virus 2	ELISPOT	IFNg release	Positive
196213	WGTIKKSKAINVLRG	C	69	83	HLA-DRB1*07:01	81% - <100%	Dengue virus 2	ELISPOT	IFNg release	Positive
26836	IKKSKAINVLRGFRKEIGRM	C	72	91	HLA class II	81% - <100%	Dengue virus 2	ELISPOT	IFNg release	Positive
539083	ALVAFLRFLTIPPTA	C	49	63	HLA-DRB1*12:02	81% - <100%	Dengue virus 2	ELISPOT	IFNg release	Positive
539264	FLRFLTIPPTAGILK	C	53	67	HLA-DRB1*14:02	81% - <100%	Dengue virus 2	ELISPOT	IFNg release	Positive
539410	IKKSKAINVLRGFRK	C	72	86	HLA-DRB1*14:04	81% - <100%	Dengue virus 2	ELISPOT	IFNg release	Positive
539514	KSKAINVLRGFRKEI	C	74	88	HLA-DRB1*12:02	81% - <100%	Dengue virus 2	ELISPOT	IFNg release	Positive
539612	LQGRGPLKLFMALVA	C	38	52	HLA-DRB1*15:02	81% - <100%	Dengue virus 2	ELISPOT	IFNg release	Positive
539887	RWGTIKKSKAINVLR	C	68	82	HLA-DRB1*08:03	81% - <100%	Dengue virus 2	ELISPOT	IFNg release	Positive
540069	VQQLTKRFSLGMLQG	C	26	40	HLA-DRB1*13:01	81% - <100%	Dengue virus 2	ELISPOT	IFNg release	Positive
739879	PFNMLKRERNRVSTV	C	12	26	HLA-DRB1*14:02	81% - <100%	Dengue virus 2	ELISPOT	IFNg release	Positive
857784	LKRWGTIKKSKAINV	C	66	80	HLA-DRB1*07:01	81% - <100%	Dengue virus 2	ELISPOT	IFNg release	Positive-Low
150490	PGFTIMAAILAYTIG	PreM	245	259	HLA-DRB1*01:01	81% - <100%	Dengue virus 2	ELISPOT	IFNg release	Positive
195322	FQRVLIFILLTAVAP	PreM	263	277	HLA-DRB1*07:01	81% - <100%	Dengue virus 2	ELISPOT	IFNg release	Positive
195503	ILRHPGFTIMAAILA	PreM	127	141	HLA-DRB1*07:01	81% - <100%	Dengue virus 2	ELISPOT	IFNg release	Positive
195702	LRHPGFTIMAAILAY	PreM	242	256	HLA-DRB1*11:01	81% - <100%	Dengue virus 2	ELISPOT	IFNg release	Positive
195863	QRVLIFILLTAVAPS	PreM	264	278	HLA-DRB1*08:02	81% - <100%	Dengue virus 2	ELISPOT	IFNg release	Positive
195892	RIETWILRHPGFTIM	PreM	122	136	HLA-DRB1*15:01	81% - <100%	Dengue virus 2	ELISPOT	IFNg release	Positive
539943	SVALVPHVGMGLETR	PreM	206	220	HLA-DRB1*15:02	81% - <100%	Dengue virus 2	ELISPOT	IFNg release	Positive
540130	WILRHPGFTIMAAIL	PreM	126	140	HLA-DRB1*15:06	81% - <100%	Dengue virus 2	ELISPOT	IFNg release	Positive
144940	RHVLGRLITVNPIVT	E	345	359	HLA-DRB1*08:02	81% - <100%	Dengue virus 2	ELISPOT	IFNg release	Positive
180564	KKQDVVVLGSQEGAM	E	526	540	HLA class II	81% - <100%	Dengue virus 2	ELISPOT	IFNg release	Positive
180568	KNPHAKKQDVVVLGS	E	521	535	HLA class II	81% - <100%	Dengue virus 2	ELISPOT	IFNg release	Positive
180820	WLVHRQWFLDLPLPW	E	196	210	HLA class II	81% - <100%	Dengue virus 2	ELISPOT	IFNg release	Positive
195638	LGRLITVNPIVTEKD	E	628	642	HLA-DRB1*08:02	81% - <100%	Dengue virus 2	ELISPOT	IFNg release	Positive
195716	LSVSLVLVGVVTLYL	E	755	769	HLA-DRB1*15:01	81% - <100%	Dengue virus 2	ELISPOT	IFNg release	Positive
196077	TSLSVSLVLVGVVTL	E	753	767	HLA-DRB1*07:01	81% - <100%	Dengue virus 2	ELISPOT	IFNg release	Positive
196140	VLGRLITVNPIVTEK	E	627	641	HLA-DRB1*08:02	81% - <100%	Dengue virus 2	ELISPOT	IFNg release	Positive
196215	WIQKETLVTFKNPHA	E	231	245	HLA-DRB1*08:02	81% - <100%	Dengue virus 2	ELISPOT	IFNg release	Positive
539252	FETTMRGAKRMAILG	E	402	416	HLA-DRB1*15:02	81% - <100%	Dengue virus 2	ELISPOT	IFNg release	Positive
539377	HAKKQDVVVLGSQEGAMH	E	244	261	HLA class II	81% - <100%	Dengue virus 2	ICS	IFNg release	Positive
539464	KETLVTFKNPHAKKQ	E	234	248	HLA-DRB1*10:01	81% - <100%	Dengue virus 2	ELISPOT	IFNg release	Positive
539806	QKETLVTFKNPHAKK	E	513	527	HLA-DRB1*04:03	81% - <100%	Dengue virus 2	ELISPOT	IFNg release	Positive
65140	TLVTFKNPHAKKQDV	E	236	250	HLA class II	81% - <100%	Dengue virus 2	ELISPOT	IFNg release	Positive
739010	GGVFTSIGKALHQVF	E	706	720	HLA-DRB1*14:02	81% - <100%	Dengue virus 2	ELISPOT	IFNg release	Positive
740315	SLSVSLVLVGVVTLY	E	754	768	HLA-DRB1*01:02	81% - <100%	Dengue virus 2	ELISPOT	IFNg release	Positive
868713	RTGLDFNEMVLLQME	E	178	192	HLA-DQA1*01:02/DQB1*06:02	81% - <100%	Dengue virus 2	ELISPOT	IFNg release	Positive
869123	TEKDSPVNIEAEPPF	E	349	363	HLA-DQA1*03:01/DQB1*03:02	81% - <100%	Dengue virus 2	ELISPOT	IFNg release	Positive
869153	TGLDFNEMVLLQMEN	E	179	193	HLA-DQA1*03:01/DQB1*03:02	81% - <100%	Dengue virus 2	ELISPOT	IFNg release	Positive
167759	IMTGDIKGIMQA	NS1	88	99	HLA-DQ6	81% - <100%	Dengue virus 2	ELISPOT	IFNg release	Positive
180396	DGCWYGMEIRPLKEKEENLVNS	NS1	1102	1123	HLA class II	81% - <100%	Dengue virus 2	ICS	IFNg release	Positive
180603	LKYSWKTWGKAKMLS	NS1	111	125	HLA class II	81% - <100%	Dengue virus 2	ELISPOT	IFNg release	Positive
180612	LNDTWKIEKASFIEV	NS1	981	995	HLA class II	81% - <100%	Dengue virus 2	ELISPOT	IFNg release	Positive
195458	HNQTFLIDGPETAEC	NS1	904	918	HLA-DRB1*04:01	81% - <100%	Dengue virus 2	ELISPOT	IFNg release	Positive
195576	KLTIMTGDIKGIMQA	NS1	85	99	HLA-DRB1*03:01	81% - <100%	Dengue virus 2	ELISPOT	IFNg release	Positive
195845	QDVFCDSKLMSAAIK	NS1	175	189	HLA-DRB1*03:01	81% - <100%	Dengue virus 2	ELISPOT	IFNg release	Positive
539828	QYKFQPESPSKLASA	NS1	806	820	HLA-DRB3*02:02	81% - <100%	Dengue virus 2	ELISPOT	IFNg release	Positive
738586	CGIRSVTRLENLMWK	NS1	55	69	HLA-DRB1*11:04	81% - <100%	Dengue virus 2	ELISPOT	IFNg release	Positive
738766	EGICGIRSVTRLENL	NS1	52	66	HLA-DRB1*11:04	81% - <100%	Dengue virus 2	ELISPOT	IFNg release	Positive
739192	IKGIMQAGKRSLRPQ	NS1	93	107	HLA-DRB1*14:06	81% - <100%	Dengue virus 2	ELISPOT	IFNg release	Positive
739296	KGIMQAGKRSLRPQP	NS1	94	108	HLA-DRB1*14:06	81% - <100%	Dengue virus 2	ELISPOT	IFNg release	Positive
866460	DYGFGVFTTNIWLKL	NS1	932	946	HLA-DPB1*02:01	81% - <100%	Dengue virus 2	ELISPOT	IFNg release	Positive
866830	GFGVFTTNIWLKLKE	NS1	934	948	HLA-DPB1*04:01	81% - <100%	Dengue virus 2	ELISPOT	IFNg release	Positive
867040	HADMGYWIESALNDT	NS1	970	984	HLA-DQA1*01:01/DQB1*05:01	81% - <100%	Dengue virus 2	ELISPOT	IFNg release	Positive
150131	ALTIKGLNPTAIFLT	NS2a	1308	1322	HLA-DRB1*09:01	81% - <100%	Dengue virus 2	ELISPOT	IFNg release	Positive
195153	ALLAAFKVRPTFAAG	NS2a	1203	1217	HLA-DRB1*08:02	81% - <100%	Dengue virus 2	ELISPOT	IFNg release	Positive
195650	LKIVRNMEKYQLAVT	NS2a	1261	1275	HLA-DRB1*13:01	81% - <100%	Dengue virus 2	ELISPOT	IFNg release	Positive
195752	MEKYQLAVTIMAILC	NS2a	1154	1168	HLA-DRB1*04:07	81% - <100%	Dengue virus 2	ELISPOT	IFNg release	Positive
195798	NMEKYQLAVTIMAIL	NS2a	1266	1280	HLA-DRB1*04:01	81% - <100%	Dengue virus 2	ELISPOT	IFNg release	Positive
196174	VRNMEKYQLAVTIMA	NS2a	1264	1278	HLA-DRB1*13:01	81% - <100%	Dengue virus 2	ELISPOT	IFNg release	Positive
539474	KIVRNMEKYQLAVTI	NS2a	1262	1276	HLA-DRB1*13:01	81% - <100%	Dengue virus 2	ELISPOT	IFNg release	Positive
539540	LALLAAFKVRPTFAA	NS2a	1202	1216	HLA-DRB1*14:04	81% - <100%	Dengue virus 2	ELISPOT	IFNg release	Positive
540154	YLALLAAFKVRPTFA	NS2a	1201	1215	HLA-DRB1*14:04	81% - <100%	Dengue virus 2	ELISPOT	IFNg release	Positive
866978	GMALFLEEMLRTRVG	NS2a	1141	1155	HLA-DQA1*01:01/DQB1*05:01	81% - <100%	Dengue virus 2	ELISPOT	IFNg release	Positive
867216	ILCVPNAVILQNAWK	NS2a	1279	1293	HLA-DQA1*01:02/DQB1*06:02	81% - <100%	Dengue virus 2	ELISPOT	IFNg release	Positive
867571	LCVPNAVILQNAWKV	NS2a	1280	1294	HLA-DRB4*01:01	81% - <100%	Dengue virus 2	ELISPOT	IFNg release	Positive
868918	SLGVLGMALFLEEML	NS2a	1136	1150	HLA-DQA1*05:01/DQB1*02:01	81% - <100%	Dengue virus 2	ELISPOT	IFNg release	Positive
167732	DQAEISGSSPILSITISEDG	NS2b	63	82	HLA-DP	81% - <100%	Dengue virus 2	ELISPOT	IFNg release	Positive
195239	EEQTLTILIRTGLLV	NS2b	91	105	HLA-DRB1*15:01	81% - <100%	Dengue virus 2	ELISPOT	IFNg release	Positive
195723	LTILIRTGLLVISGL	NS2b	1440	1454	HLA-DRB1*15:06	81% - <100%	Dengue virus 2	ELISPOT	IFNg release	Positive
195869	QTLTILIRTGLLVIS	NS2b	1438	1452	HLA-DRB1*15:01	81% - <100%	Dengue virus 2	ELISPOT	IFNg release	Positive
139762	GLPIRYQTPAIRAEH	NS3	1712	1726	HLA-DRB1*04:03	81% - <100%	Dengue virus 2	ELISPOT	IFNg release	Positive
180807	VTDFKGKTVWFVPSI	NS3	1826	1840	HLA class II	81% - <100%	Dengue virus 2	ELISPOT	IFNg release	Positive-Low
195154	ALRGLPIRYQTPAIR	NS3	1709	1723	HLA-DRB1*08:02	81% - <100%	Dengue virus 2	ELISPOT	IFNg release	Positive
195564	KGKVVGLYGNGVVTR	NS3	1618	1632	HLA-DRB1*15:01	81% - <100%	Dengue virus 2	ELISPOT	IFNg release	Positive
195765	MRLLSPVRVPNYNLI	NS3	1742	1756	HLA-DRB1*13:01	81% - <100%	Dengue virus 2	ELISPOT	IFNg release	Positive
31726	KKVIQLSRKTFDSEY	NS3	1855	1869	HLA-DRB1*12:02	81% - <100%	Dengue virus 2	ELISPOT	IFNg release	Positive
39103	LRGLPIRYQTPAIRA	NS3	1696	1710	HLA-DRB1*14:02	81% - <100%	Dengue virus 2	ELISPOT	IFNg release	Positive
4152	ARGYISTRVEMGEAA	NS3	1757	1771	HLA-DRB1*04:07	81% - <100%	Dengue virus 2	ELISPOT	IFNg release	Positive
539203	EERVILAGPMPVTHS	NS3	1899	1913	HLA-DRB1*09:01	81% - <100%	Dengue virus 2	ELISPOT	IFNg release	Positive
539467	KGKTVWFVPSIKAGN	NS3	1830	1844	HLA-DRB1*08:03	81% - <100%	Dengue virus 2	ELISPOT	IFNg release	Positive
539713	NGKKVIQLSRKTFDS	NS3	1853	1867	HLA-DRB1*12:02	81% - <100%	Dengue virus 2	ELISPOT	IFNg release	Positive
539850	RGLPIRYQTPAIRAE	NS3	1711	1725	HLA-DRB1*13:01	81% - <100%	Dengue virus 2	ELISPOT	IFNg release	Positive
539857	RKNGKKVIQLSRKTF	NS3	1851	1865	HLA-DRB1*08:03	81% - <100%	Dengue virus 2	ELISPOT	IFNg release	Positive
868724	RTNDWDFVVTTDISE	NS3	1873	1887	HLA-DQA1*03:01/DQB1*03:02	81% - <100%	Dengue virus 2	ELISPOT	IFNg release	Positive
8911	DKKGKVVGLYGNGVV	NS3	141	155	HLA-DRB1*15:01	81% - <100%	Dengue virus 2	ICS	IFNg release	Positive
195355	GGIFLFLMSGKGIGK	NS4a	2145	2159	HLA-DRB1*11:01	81% - <100%	Dengue virus 2	ELISPOT	IFNg release	Positive
195414	GRAYNHALSELPETL	NS4a	2131	2145	HLA-DRB1*04:01	81% - <100%	Dengue virus 2	ELISPOT	IFNg release	Positive
195635	LGMCCIITASILLWY	NS4a	2176	2190	HLA-DRB1*07:01	81% - <100%	Dengue virus 2	ELISPOT	IFNg release	Positive
196089	TVTGGIFLFLMSGKG	NS4a	2142	2156	HLA-DRB1*11:01	81% - <100%	Dengue virus 2	ELISPOT	IFNg release	Positive
539609	LPTFMTQKARNALDN	NS4a	2092	2106	HLA-DRB1*10:01	81% - <100%	Dengue virus 2	ELISPOT	IFNg release	Positive
539801	QDNQLTYVVIAILTV	NS4a	2223	2237	HLA-DRB1*15:02	81% - <100%	Dengue virus 2	ELISPOT	IFNg release	Positive
539804	QIQPHWIAASIILEF	NS4a	2192	2206	HLA-DRB1*13:01	81% - <100%	Dengue virus 2	ELISPOT	IFNg release	Positive
540148	YAQIQPHWIAASIIL	NS4a	2176	2190	HLA-DRB1*13:01	81% - <100%	Dengue virus 2	ELISPOT	IFNg release	Positive
150611	TAALLLLVAHYAIIG	NS4b	2351	2365	HLA-DRB1*01:01	81% - <100%	Dengue virus 2	ELISPOT	IFNg release	Positive
150636	TLTAALLLLVAHYAI	NS4b	2335	2349	HLA-DRB1*01:01	81% - <100%	Dengue virus 2	ELISPOT	IFNg release	Positive
195115	AGAGLLFSIMKNTTN	NS4b	2474	2488	HLA-DRB1*11:01	81% - <100%	Dengue virus 2	ELISPOT	IFNg release	Positive
195347	GAGLLFSIMKNTTNT	NS4b	2475	2489	HLA-DRB1*11:01	81% - <100%	Dengue virus 2	ELISPOT	IFNg release	Positive
195621	LCVTQVLMMRTTWAL	NS4b	2405	2419	HLA-DRB1*07:01	81% - <100%	Dengue virus 2	ELISPOT	IFNg release	Positive
195629	LFSIMKNTTNTRRGT	NS4b	2479	2493	HLA-DRB1*03:01	81% - <100%	Dengue virus 2	ELISPOT	IFNg release	Positive
195658	LLFSIMKNTTNTRRG	NS4b	2478	2492	HLA-DRB1*04:01	81% - <100%	Dengue virus 2	ELISPOT	IFNg release	Positive
195827	PESNILDIDLRPASA	NS4b	2266	2280	HLA-DRB1*03:01	81% - <100%	Dengue virus 2	ELISPOT	IFNg release	Positive
195980	SQVNPITLTAALLLL	NS4b	2343	2357	HLA-DQA1*01:02/DQB1*06:02	81% - <100%	Dengue virus 2	ELISPOT	IFNg release	Positive
539327	GLLFSIMKNTTNTRR	NS4b	2477	2491	HLA-DRB1*14:02	81% - <100%	Dengue virus 2	ELISPOT	IFNg release	Positive
867860	LLVLCVTQVLMMRTT	NS4b	2416	2430	HLA-DPB1*04:02	81% - <100%	Dengue virus 2	ELISPOT	IFNg release	Positive
1066545	ACLGKSYAQMWSLMY	NS5	3243	3257	HLA-DRB1*04:01	81% - <100%	Dengue virus 2	ELISPOT	IFNg release	Positive-Low
150597	SRAIWYMWLGARFLE	NS5	2962	2976	HLA-DRB1*01:01	81% - <100%	Dengue virus 2	ELISPOT	IFNg release	Positive
180579	KSYAQMWSLMYFHRR	NS5	3247	3261	HLA-DRB5*01:01	81% - <100%	Dengue virus 2	ELISPOT	IFNg release	Positive
180582	KTWAYHGSYETKQTG	NS5	2792	2806	HLA class II	81% - <100%	Dengue virus 2	ICS	IFNg release	Positive
195251	ELIMKDGRVLVVPCR	NS5	3206	3220	HLA-DRB1*03:01	81% - <100%	Dengue virus 2	ELISPOT	IFNg release	Positive
195380	GKSYAQMWSLMYFHR	NS5	3232	3246	HLA-DRB1*04:01	81% - <100%	Dengue virus 2	ELISPOT	IFNg release	Positive
195428	GTVMDIISRRDQRGS	NS5	3078	3092	HLA-DRB1*03:01	81% - <100%	Dengue virus 2	ELISPOT	IFNg release	Positive
195801	NMISRMLINRFTMRH	NS5	6	20	HLA-DRB1*08:02	81% - <100%	Dengue virus 2	ELISPOT	IFNg release	Positive
196161	VNMISRMLINRFTMR	NS5	2723	2737	HLA-DRB1*10:01	81% - <100%	Dengue virus 2	ELISPOT	IFNg release	Positive
196223	WSLMYFHRRDLRLAA	NS5	3253	3267	HLA-DRB1*03:01	81% - <100%	Dengue virus 2	ELISPOT	IFNg release	Positive
196243	YQNKVVRVQRPTPRG	NS5	3064	3078	HLA-DRB1*08:02	81% - <100%	Dengue virus 2	ELISPOT	IFNg release	Positive
539144	CVVKPLDDRFASALTAL	NS5	3156	3172	HLA class II	81% - <100%	Dengue virus 2	ICS	IFNg release	Positive
539150	DDRFASALTALNDMG	NS5	3162	3176	HLA-DRB1*04:03	81% - <100%	Dengue virus 2	ELISPOT	IFNg release	Positive
539195	EAIFKLTYQNKVVRV	NS5	3057	3071	HLA-DRB1*09:01	81% - <100%	Dengue virus 2	ELISPOT	IFNg release	Positive
539438	ISRMLINRFTMRHKK	NS5	8	22	HLA-DRB1*14:02	81% - <100%	Dengue virus 2	ELISPOT	IFNg release	Positive
539599	LMYFHRRDLRLAANA	NS5	3255	3269	HLA-DRB1*13:01	81% - <100%	Dengue virus 2	ELISPOT	IFNg release	Positive
539669	MISRMLINRFTMRHK	NS5	7	21	HLA-DRB1*14:06	81% - <100%	Dengue virus 2	ELISPOT	IFNg release	Positive
540064	VNGVVRLLTKPWDVI	NS5	2812	2826	HLA-DRB1*12:02	81% - <100%	Dengue virus 2	ELISPOT	IFNg release	Positive
738754	EEFTRKVRSNAALGA	NS5	2874	2888	HLA-DRB1*11:04	81% - <100%	Dengue virus 2	ELISPOT	IFNg release	Positive
739166	IFKLTYQNKVVRVQR	NS5	3045	3059	HLA-DRB1*14:06	81% - <100%	Dengue virus 2	ELISPOT	IFNg release	Positive
739791	NLVRLQSGVDVFFTP	NS5	2599	2613	HLA-DRB1*01:02	81% - <100%	Dengue virus 2	ELISPOT	IFNg release	Positive
740675	VRSNAALGAIFTDEN	NS5	2880	2894	HLA-DRB1*11:04	81% - <100%	Dengue virus 2	ELISPOT	IFNg release	Positive
866015	AIWYMWLGARFLEFE	NS5	2964	2978	HLA-DPB1*01:01	81% - <100%	Dengue virus 2	ELISPOT	IFNg release	Positive
866566	EHKKLAEAIFKLTYQ	NS5	3051	3065	HLA-DPB1*01:01	81% - <100%	Dengue virus 2	ELISPOT	IFNg release	Positive
867419	KKLAEAIFKLTYQNK	NS5	3053	3067	HLA-DPB1*02:01	81% - <100%	Dengue virus 2	ELISPOT	IFNg release	Positive
867433	KLAEAIFKLTYQNKV	NS5	3054	3068	HLA-DPB1*02:01	81% - <100%	Dengue virus 2	ELISPOT	IFNg release	Positive
867514	KTPVESWEEIPYLGK	NS5	3318	3332	HLA-DQA1*01:01/DQB1*05:01	81% - <100%	Dengue virus 2	ELISPOT	IFNg release	Positive
868668	RKVRSNAALGAIFTD	NS5	2892	2906	HLA-DRB3*02:02	81% - <100%	Dengue virus 2	ELISPOT	IFNg release	Positive
869082	SYAQMWSLMYFHRRD	NS5	3248	3262	HLA-DPB1*04:01	81% - <100%	Dengue virus 2	ELISPOT	IFNg release	Positive
869307	TRKVRSNAALGAIFT	NS5	2891	2905	HLA-DRB3*02:02	81% - <100%	Dengue virus 2	ELISPOT	IFNg release	Positive
195417	GRMLNILNRRRRTAG	C	89	103	HLA-DRB1*11:01	51%-80%	Dengue virus 2	ELISPOT	IFNg release	Positive
195489	IGRMLNILNRRRRTA	C	88	102	HLA-DRB1*08:02	51%-80%	Dengue virus 2	ELISPOT	IFNg release	Positive
195899	RMLNILNRRRRTAGV	C	71	85	HLA-DRB1*08:02	51%-80%	Dengue virus 2	ELISPOT	IFNg release	Positive
196133	VKLTIMTGDIKGIMQ	NS1	84	98	HLA-DRB1*03:01	51%-80%	Dengue virus 2	ELISPOT	IFNg release	Positive
195207	CTILAVVSVSPLLLT	NS2a	1144	1158	HLA-DRB1*07:01	51%-80%	Dengue virus 2	ELISPOT	IFNg release	Positive
195615	LAVVSVSPLLLTSSQ	NS2a	1186	1200	HLA-DRB1*07:01	51%-80%	Dengue virus 2	ELISPOT	IFNg release	Positive
196218	WKVSCTILAVVSVSP	NS2a	1292	1306	HLA-DRB1*04:01	51%-80%	Dengue virus 2	ELISPOT	IFNg release	Positive
539561	LGMMILKIVRNMEKY	NS2a	1256	1270	HLA-DRB1*13:01	51%-80%	Dengue virus 2	ELISPOT	IFNg release	Positive
740700	VVSVSPLLLTSSQQK	NS2a	1188	1202	HLA-DRB1*01:02	51%-80%	Dengue virus 2	ELISPOT	IFNg release	Positive
30396	KEGERKKLKPRWLDA	NS3	584	598	HLA class II	51%-80%	Dengue virus 2	ICS	IFNg release	Positive
539200	EDDIFRKKRLTIMDL	NS3	1654	1668	HLA-DRB1*13:01	51%-80%	Dengue virus 2	ELISPOT	IFNg release	Positive
167780	LTLNLITEMGRLPTFMTQKA	NS4a	2	21	HLA class II	51%-80%	Dengue virus 2	ELISPOT	IFNg release	Positive
150247	GMIIMLIPTVMAFHL	C	103	117	HLA-DRB1*01:01	>0% - 50%	Dengue virus 2	ELISPOT	IFNg release	Positive
195120	AGMIIMLIPTVMAFH	C	102	116	HLA-DRB1*08:02	>0% - 50%	Dengue virus 2	ELISPOT	IFNg release	Positive
195496	IIMLIPTVMAFHLTT	C	105	119	HLA-DRB1*01:02	>0% - 50%	Dengue virus 2	ELISPOT	IFNg release	Positive
739181	IGRMLNILNRRRRTV	C	42	56	HLA-DRB1*14:02	>0% - 50%	Dengue virus 2	ELISPOT	IFNg release	Positive
195862	QRALIFILLTAVAPS	PreM	264	278	HLA-DRB1*15:01	>0% - 50%	Dengue virus 2	ELISPOT	IFNg release	Positive
196141	VLIFILLTAIAPSMT	PreM	266	280	HLA-DRB1*08:02	>0% - 50%	Dengue virus 2	ELISPOT	IFNg release	Positive
539881	RVLIFILLTAIAPSM	PreM	265	279	HLA-DRB1*15:06	>0% - 50%	Dengue virus 2	ELISPOT	IFNg release	Positive
539236	ENLEYTIVITPHSGE	E	133	147	HLA-DRB1*04:07	>0% - 50%	Dengue virus 2	ELISPOT	IFNg release	Positive
539733	NLEYTIVITPHSGEE	E	134	148	HLA-DRB1*04:07	>0% - 50%	Dengue virus 2	ELISPOT	IFNg release	Positive
866243	AWMVHRQWFLDLPLP	E	205	219	HLA-DQA1*01:01/DQB1*05:01	>0% - 50%	Dengue virus 2	ELISPOT	IFNg release	Positive
869152	TGLDFNEMVLLQMED	E	189	203	HLA-DQA1*03:01/DQB1*03:02	>0% - 50%	Dengue virus 2	ELISPOT	IFNg release	Positive
195583	KQDVFCDSKLMSAAI	NS1	949	963	HLA-DRB1*03:01	>0% - 50%	Dengue virus 2	ELISPOT	IFNg release	Positive
195880	REKQDVFCDSKLMSA	NS1	947	961	HLA-DRB1*03:01	>0% - 50%	Dengue virus 2	ELISPOT	IFNg release	Positive
195406	GMMVLKIVRNMEKYQ	NS2a	1257	1271	HLA-DRB1*08:02	>0% - 50%	Dengue virus 2	ELISPOT	IFNg release	Positive

195567	KHAILLVAVSFVTLI	NS2a	1157	1171	HLA-DRB1*07:01	>0% - 50%	Dengue virus 2	ELISPOT	IFNg release	Positive	0	0	0	0	0	0	0	0	0	0	0	0	0	0	0	0	0	0	0	0	0	0	0	0	0	0	0	0	0	0	0	0	0	0	0	0	0	1	0	0	0	0	0	0	0	0	0	0	0
195610	LALGMMVLKIVRNME	NS2a	1254	1268	HLA-DRB1*13:01	>0% - 50%	Dengue virus 2	ELISPOT	IFNg release	Positive	0	0	0	0	0	0	0	0	0	0	0	0	0	0	0	0	0	0	0	0	0	0	0	0	0	0	0	0	0	0	0	0	0	0	0	0	1	0	0	0	0	0	0	0	0	0	0	0	0
195688	LNPTAIFLTTLSRTS	NS2a	1328	1342	HLA-DRB1*07:01	>0% - 50%	Dengue virus 2	ELISPOT	IFNg release	Positive	0	0	0	0	0	0	0	0	0	0	0	0	0	0	0	0	0	0	0	0	0	0	0	0	0	0	0	0	0	0	0	0	0	0	0	0	0	1	0	0	0	0	0	0	0	0	0	0	0
195753	MEKYQLAVTIMAISC	NS2a	1267	1281	HLA-DRB1*10:01	>0% - 50%	Dengue virus 2	ELISPOT	IFNg release	Positive	0	0	0	0	0	0	0	0	0	0	0	0	0	0	0	0	0	0	0	0	0	0	0	0	0	0	0	0	0	0	0	0	0	1	0	0	0	0	0	0	0	0	0	0	0	0	0	0	0
195799	NMEKYQLAVTIMAIS	NS2a	1266	1280	HLA-DRB1*04:01	>0% - 50%	Dengue virus 2	ELISPOT	IFNg release	Positive	0	0	0	0	0	0	0	0	0	0	0	0	0	0	0	0	0	0	0	0	0	0	0	0	0	0	0	0	0	0	0	0	0	1	0	0	0	0	0	0	0	0	0	0	0	0	0	0	0
196237	YQLAVTIMAISCVPN	NS2a	1270	1284	HLA-DRB1*08:02	>0% - 50%	Dengue virus 2	ELISPOT	IFNg release	Positive	0	0	0	0	0	0	0	0	0	0	0	0	0	0	0	0	0	0	0	0	0	0	0	0	0	0	0	0	0	0	0	0	0	1	0	0	0	0	0	0	0	0	0	0	0	0	0	0	0
539108	AVSFVTLITGNMSFR	NS2a	1164	1178	HLA-DRB1*10:01	>0% - 50%	Dengue virus 2	ELISPOT	IFNg release	Positive	0	0	0	0	0	0	0	0	0	0	0	0	0	0	0	0	0	0	0	0	0	0	0	0	0	0	0	0	0	0	0	0	0	0	0	0	0	1	0	0	0	0	0	0	0	0	0	0	0
539231	EMLRTRVGTKHAILL	NS2a	1148	1162	HLA-DRB1*08:03	>0% - 50%	Dengue virus 2	ELISPOT	IFNg release	Positive	0	0	0	0	0	0	0	0	0	0	0	0	0	0	1	0	0	0	0	0	0	0	0	0	0	0	0	0	0	0	0	0	0	0	0	0	0	1	0	0	0	0	0	0	0	0	0	0	0
539562	LGMMVLKIVRNMEKY	NS2a	1256	1270	HLA-DRB1*12:02	>0% - 50%	Dengue virus 2	ELISPOT	IFNg release	Positive	0	0	0	0	0	0	0	0	0	0	0	0	0	0	0	0	0	0	0	0	0	0	0	0	0	0	0	0	0	0	0	0	0	0	0	0	1	0	0	0	0	0	0	0	0	0	0	0	0
539686	MMVLKIVRNMEKYQL	NS2a	1244	1258	HLA-DRB1*14:06	>0% - 50%	Dengue virus 2	ELISPOT	IFNg release	Positive	0	0	0	0	0	0	0	0	0	0	0	0	0	0	0	0	0	0	0	0	0	0	0	0	0	0	0	0	0	0	0	0	0	0	0	0	1	0	0	0	0	0	0	0	0	0	0	0	0
539699	MVLKMVRNMEKYQLA	NS2a	1259	1273	HLA-DRB1*13:01	>0% - 50%	Dengue virus 2	ELISPOT	IFNg release	Positive	0	0	0	0	0	0	0	0	0	0	0	0	0	0	0	0	0	0	0	0	0	0	0	0	0	0	0	0	0	0	0	0	0	0	0	0	0	1	0	0	0	0	0	0	0	0	0	0	0
539789	PTAIFLTTLSRTSKK	NS2a	1316	1330	HLA-DRB1*04:03	>0% - 50%	Dengue virus 2	ELISPOT	IFNg release	Positive	0	0	0	0	0	0	0	0	0	0	0	0	0	0	0	0	0	0	0	0	0	0	0	0	0	0	0	0	0	0	0	0	0	0	0	0	0	1	0	0	0	0	0	0	0	0	0	0	0
539959	TAIFLTTLSRTSKKR	NS2a	1317	1331	HLA-DRB1*14:02	>0% - 50%	Dengue virus 2	ELISPOT	IFNg release	Positive	0	0	0	0	0	0	0	0	0	0	0	0	0	0	0	0	0	0	0	0	0	0	0	0	0	0	0	0	0	0	0	0	0	0	0	0	0	1	0	0	0	0	0	0	0	0	0	0	0
540026	VAVSFVTLITGNMSF	NS2a	1163	1177	HLA-DRB1*10:01	>0% - 50%	Dengue virus 2	ELISPOT	IFNg release	Positive	0	0	0	0	0	0	0	0	0	0	0	0	0	0	0	0	0	0	0	0	0	0	0	0	0	0	0	0	0	0	0	0	0	0	0	0	0	1	0	0	0	0	0	0	0	0	0	0	0
540086	VSFVTLITGNMSFRD	NS2a	1165	1179	HLA-DRB1*10:01	>0% - 50%	Dengue virus 2	ELISPOT	IFNg release	Positive	0	0	0	0	0	0	0	0	0	0	0	0	0	0	0	0	0	0	0	0	0	0	0	0	0	0	0	0	0	0	0	0	0	0	0	0	0	1	0	0	0	0	0	0	0	0	0	0	0
866586	EKYQLAVTIMAISCV	NS2a	1268	1282	HLA-DQA1*01:02/DQB1*06:02	>0% - 50%	Dengue virus 2	ELISPOT	IFNg release	Positive	0	0	0	0	0	0	0	0	0	0	0	0	0	0	0	0	0	0	0	0	0	0	0	0	0	0	0	0	0	0	0	0	0	1	0	0	0	0	0	0	0	0	0	0	0	0	0	0	0
869200	TKHAILLVAVSFVTL	NS2a	1156	1170	HLA-DPB1*04:01	>0% - 50%	Dengue virus 2	ELISPOT	IFNg release	Positive	0	0	0	0	0	0	0	0	0	0	0	0	0	0	0	0	0	0	0	0	0	0	0	0	0	0	0	0	0	0	0	0	0	0	0	0	0	1	0	0	0	0	0	0	0	0	0	0	0
739148	IDKKGKVVGLYGNGV	NS3	1615	1629	HLA-DRB1*01:02	>0% - 50%	Dengue virus 2	ELISPOT	IFNg release	Positive	0	0	0	0	0	0	0	0	0	0	0	0	0	0	0	0	0	0	0	0	0	0	0	0	0	0	0	0	0	0	0	0	0	0	0	0	0	0	0	0	1	0	0	0	0	0	0	0	0
195356	GGIFLFLMSGRGIGK	NS4a	2159	2173	HLA-DRB1*11:01	>0% - 50%	Dengue virus 2	ELISPOT	IFNg release	Positive	0	0	0	0	0	0	0	0	0	0	0	0	0	0	0	0	0	0	0	0	0	0	0	0	0	0	0	0	0	0	0	0	0	0	0	0	0	1	0	0	0	0	0	0	0	0	0	0	0
195397	GLLFSIMKNTTSTRR	NS4b	2477	2491	HLA-DRB1*04:01	>0% - 50%	Dengue virus 2	ELISPOT	IFNg release	Positive	0	0	0	1	0	0	0	0	0	0	0	0	0	0	0	0	0	0	0	0	0	0	0	0	0	0	0	0	0	0	0	0	0	0	0	0	0	0	0	0	0	0	0	0	0	0	0	0	0
195718	LTAALFLLVAHYAII	NS4b	2350	2364	HLA-DRB1*07:01	>0% - 50%	Dengue virus 2	ELISPOT	IFNg release	Positive	0	0	0	0	0	0	0	0	0	0	0	0	0	0	0	0	0	0	0	0	0	0	0	0	0	0	0	0	0	0	0	0	0	0	0	0	0	1	0	0	0	0	0	0	0	0	0	0	0
867335	ITLTAALLLLIAHYA	NS4b	2348	2362	HLA-DPB1*04:02	>0% - 50%	Dengue virus 2	ELISPOT	IFNg release	Positive	0	0	0	0	0	0	0	1	0	0	1	0	0	0	0	0	0	0	0	0	0	1	0	0	0	0	0	0	0	0	0	0	0	0	0	0	0	0	0	0	0	0	0	0	0	0	0	0	0
195640	LGYILRDVSKKEGGA	NS5	2992	3006	HLA-DRB1*03:01	>0% - 50%	Dengue virus 2	ELISPOT	IFNg release	Positive	1	0	0	1	1	1	1	1	1	1	1	0	0	0	0	0	0	0	0	0	1	0	0	0	0	1	0	1	1	0	1	1	0	0	0	1	0	0	1	0	1	0	0	0	1	0	0	1	1
195642	LHKLGYILRDVSKKE	NS5	3003	3017	HLA-DRB1*08:02	>0% - 50%	Dengue virus 2	ELISPOT	IFNg release	Positive	1	0	0	1	1	1	1	1	1	1	1	0	0	0	0	0	0	0	0	0	1	0	0	0	0	1	0	1	1	0	1	1	0	0	0	1	0	0	1	0	1	0	0	0	1	0	0	1	1
195750	MEGEGIFKSIQHLTA	NS5	3114	3128	HLA-DRB1*11:01	>0% - 50%	Dengue virus 2	ELISPOT	IFNg release	Positive	0	0	0	0	0	0	0	0	0	0	0	0	0	0	0	0	0	0	0	0	0	0	0	0	0	0	0	0	0	0	0	0	0	0	0	0	0	1	0	0	0	0	0	0	0	0	0	0	0
195759	MLINRFTMKHKKATY	NS5	2729	2743	HLA-DRB1*11:01	>0% - 50%	Dengue virus 2	ELISPOT	IFNg release	Positive	0	1	0	0	0	0	0	0	0	0	0	1	1	0	0	0	0	0	0	0	0	0	0	0	0	0	0	0	0	0	0	0	0	0	0	0	0	0	0	0	0	0	0	0	0	0	0	0	0
195800	NMISRMLINRFTMKH	NS5	2724	2738	HLA-DRB1*08:02	>0% - 50%	Dengue virus 2	ELISPOT	IFNg release	Positive	0	1	0	0	0	0	0	0	0	0	0	1	1	0	0	0	0	0	0	0	0	0	0	0	0	0	0	0	0	0	0	0	0	0	1	0	0	0	0	0	0	0	0	0	0	0	0	0	0
32943	KPWDVVPMV	NS5	2821	2829	HLA class II	>0% - 50%	Dengue virus 2	ELISA	IL-2 release	Positive	0	0	0	0	0	0	0	0	0	0	0	0	0	0	0	0	0	0	0	0	0	0	0	0	0	0	0	0	0	0	0	0	0	0	0	0	0	1	0	0	0	0	0	0	0	0	0	0	0
539603	LNTFTNMEAQLIRQM	NS5	3100	3114	HLA-DRB1*10:01	>0% - 50%	Dengue virus 2	ELISPOT	IFNg release	Positive	0	0	0	0	0	0	0	0	0	0	0	0	0	0	0	0	0	0	0	0	0	0	0	0	0	0	0	0	0	0	0	0	0	0	0	0	0	1	0	1	0	0	0	0	0	0	0	0	0
539668	MISRMLINRFTMKHK	NS5	2725	2739	HLA-DRB1*14:04	>0% - 50%	Dengue virus 2	ELISPOT	IFNg release	Positive	0	1	0	0	0	0	0	0	0	0	0	1	1	0	0	0	0	0	0	0	0	0	0	0	0	0	0	0	0	0	0	0	0	0	1	0	0	0	0	0	0	0	0	0	0	0	0	0	0
539932	SRMLINRFTMKHKKA	NS5	2727	2741	HLA-DRB1*08:03	>0% - 50%	Dengue virus 2	ELISPOT	IFNg release	Positive	0	1	0	0	0	0	0	0	0	0	0	1	1	0	0	0	0	0	0	0	0	0	0	0	0	0	0	0	0	0	0	0	0	0	0	0	0	0	0	0	0	0	0	0	0	0	0	0	0
540065	VNGVVRLLTKPWDVV	NS5	2812	2826	HLA-DRB1*12:02	>0% - 50%	Dengue virus 2	ELISPOT	IFNg release	Positive	0	0	0	0	0	0	0	0	0	0	0	0	0	0	0	0	0	0	0	0	0	0	0	0	0	0	0	0	0	0	0	0	0	0	0	0	0	1	0	0	0	0	0	0	0	0	0	0	0
540066	VNMISRMLINRFTMK	NS5	2723	2737	HLA-DRB1*12:02	>0% - 50%	Dengue virus 2	ELISPOT	IFNg release	Positive	0	1	0	0	0	0	0	0	0	0	0	1	1	0	0	0	0	0	0	0	0	0	0	0	0	0	0	0	0	0	0	0	0	0	1	0	0	0	0	0	0	0	0	0	0	0	0	0	0
866648	ETCVYNMMGKREKKL	NS5	2939	2953	HLA-DRB5*01:01	>0% - 50%	Dengue virus 2	ELISPOT	IFNg release	Positive	1	0	0	1	1	1	1	1	1	1	1	0	0	0	0	0	0	0	0	0	1	0	0	0	0	1	0	1	1	0	1	1	0	0	0	1	0	1	1	0	1	0	0	0	1	0	0	1	1

CD4 DENV-3 Epitopes

											NCBI IDs of DENV-3 circulating strains from India, (100% identity=1,green ; else 0, red)																			
Epitope_ID	AA_Seq	Protein_Name	Start	End	Allele	step	Serotype	Method/Technique	Assay Group	Qualitative Measure	ADM63678.1	AFZ40126.1	AFZ40127.1	ANC57607.1	ANC57612.1	QCZ25021.1	QCZ25020.1	QCZ25019.1	QCZ25016.1	QCZ25017.1	QBA57533.1	QEP41653.1	ACV04798.1	AXX75610.1	AXX75612.1	AXX75611.1	AML29863.1	AXX75613.1	QEP41661	QEP41655
195205	CLMMMLPATLAFHLT	C	104	118	HLA-DRB1*15:01	0%	Dengue virus 3	ELISPOT	IFNg release	Positive	0	0	0	0	0	0	0	0	0	0	0	0	0	0	0	0	0	0	0	0
195289	FCLMMMLPATLAFHL	C	103	117	HLA-DRB1*01:01	0%	Dengue virus 3	ELISPOT	IFNg release	Positive	0	0	0	0	0	0	0	0	0	0	0	0	0	0	0	0	0	0	0	0
195517	INMLKRVRNRVSTGP	C	13	27	HLA-DRB1*08:02	0%	Dengue virus 3	ELISPOT	IFNg release	Positive	0	0	0	0	0	0	0	0	0	0	0	0	0	0	0	0	0	0	0	0
195519	INMLKRVRNRVSTGT	C	13	27	HLA-DRB1*08:02	0%	Dengue virus 3	ELISPOT	IFNg release	Positive	0	0	0	0	0	0	0	0	0	0	0	0	0	0	0	0	0	0	0	0
195598	KTSLCLVMILPAALA	C	100	114	HLA-DRB1*01:01	0%	Dengue virus 3	ELISPOT	IFNg release	Positive	0	0	0	0	0	0	0	0	0	0	0	0	0	0	0	0	0	0	0	0
195618	LCLMMIMPAALAFHL	C	103	117	HLA-DRB1*01:01	0%	Dengue virus 3	ELISPOT	IFNg release	Positive	0	0	0	0	0	0	0	0	0	0	0	0	0	0	0	0	0	0	0	0
195803	NMLKRVRNRVSTGPQ	C	14	28	HLA-DRB1*11:01	0%	Dengue virus 3	ELISPOT	IFNg release	Positive	0	0	0	0	0	0	0	0	0	0	0	0	0	0	0	0	0	0	0	0
195966	SLCLMMIMPAALAFH	C	102	116	HLA-DRB1*08:02	0%	Dengue virus 3	ELISPOT	IFNg release	Positive	0	0	0	0	0	0	0	0	0	0	0	0	0	0	0	0	0	0	0	0
195967	SLCLMMMLPATLAFH	C	102	116	HLA-DRB1*08:02	0%	Dengue virus 3	ELISPOT	IFNg release	Positive	0	0	0	0	0	0	0	0	0	0	0	0	0	0	0	0	0	0	0	0
539447	IVNRRKKTSLCLMMM	C	94	108	HLA-DRB1*13:01	0%	Dengue virus 3	ELISPOT	IFNg release	Positive	0	0	0	0	0	0	0	0	0	0	0	0	0	0	0	0	0	0	0	0
539906	SIINRRKRTSLCLMM	C	93	107	HLA-DRB1*13:01	0%	Dengue virus 3	ELISPOT	IFNg release	Positive	0	0	0	0	0	0	0	0	0	0	0	0	0	0	0	0	0	0	0	0
540118	WGSFKKSGAVKVLRG	C	69	83	HLA-DRB1*09:01	0%	Dengue virus 3	ELISPOT	IFNg release	Positive	0	0	0	0	0	0	0	0	0	0	0	0	0	0	0	0	0	0	0	0
540119	WGTFKKLGAIKVLKG	C	69	83	HLA-DRB1*09:01	0%	Dengue virus 3	ELISPOT	IFNg release	Positive	0	0	0	0	0	0	0	0	0	0	0	0	0	0	0	0	0	0	0	0
540120	WGTFKKLGAIKVLRG	C	69	83	HLA-DRB1*09:01	0%	Dengue virus 3	ELISPOT	IFNg release	Positive	0	0	0	0	0	0	0	0	0	0	0	0	0	0	0	0	0	0	0	0
540122	WGTFKKSGAIKVLKS	C	69	83	HLA-DRB1*09:01	0%	Dengue virus 3	ELISPOT	IFNg release	Positive	0	0	0	0	0	0	0	0	0	0	0	0	0	0	0	0	0	0	0	0
540123	WGTFKKSGAIKVLRG	C	69	83	HLA-DRB1*09:01	0%	Dengue virus 3	ELISPOT	IFNg release	Positive	0	0	0	0	0	0	0	0	0	0	0	0	0	0	0	0	0	0	0	0
540124	WGTFKKSGAIRVLRG	C	69	83	HLA-DRB1*09:01	0%	Dengue virus 3	ELISPOT	IFNg release	Positive	0	0	0	0	0	0	0	0	0	0	0	0	0	0	0	0	0	0	0	0
591709	LRGFKKEISNMLSII	C	81	95	HLA-DRB1*13:02	0%	Dengue virus 3	ELISPOT	IFNg release	Positive	0	0	0	0	0	0	0	0	0	0	0	0	0	0	0	0	0	0	0	0
591746	RGFKKEISNMLSIIN	C	82	96	HLA-DRB1*04:01	0%	Dengue virus 3	ELISPOT	IFNg release	Positive	0	0	0	0	0	0	0	0	0	0	0	0	0	0	0	0	0	0	0	0
867744	LKSFKKEISNMLSII	C	81	95	HLA-DRB3*02:02	0%	Dengue virus 3	ELISPOT	IFNg release	Positive	0	0	0	0	0	0	0	0	0	0	0	0	0	0	0	0	0	0	0	0
867909	LRGFKREISNMLSII	C	64	78	HLA-DRB3*02:02	0%	Dengue virus 3	ELISPOT	IFNg release	Positive	0	0	0	0	0	0	0	0	0	0	0	0	0	0	0	0	0	0	0	0
868733	RWGTFKKSGAIKVLR	C	48	62	HLA-DRB3*02:02	0%	Dengue virus 3	ELISPOT	IFNg release	Positive	0	0	0	0	0	0	0	0	0	0	0	0	0	0	0	0	0	0	0	0
195968	SLIQKVVIFILLMLV	PreM	261	275	HLA-DRB1*15:06	0%	Dengue virus 3	ELISPOT	IFNg release	Positive	0	0	0	0	0	0	0	0	0	0	0	0	0	0	0	0	0	0	0	0
196067	TQKVVIFVLLMLVTP	PreM	263	277	HLA-DRB1*15:06	0%	Dengue virus 3	ELISPOT	IFNg release	Positive	0	0	0	0	0	0	0	0	0	0	0	0	0	0	0	0	0	0	0	0
539529	KVVIFILLILVTPSM	PreM	265	279	HLA-DRB1*15:06	0%	Dengue virus 3	ELISPOT	IFNg release	Positive	0	0	0	0	0	0	0	0	0	0	0	0	0	0	0	0	0	0	0	0
539808	QKVVIFTLLMLVTPS	PreM	264	278	HLA-DRB1*15:06	0%	Dengue virus 3	ELISPOT	IFNg release	Positive	0	0	0	0	0	0	0	0	0	0	0	0	0	0	0	0	0	0	0	0
196170	VQHENLKYTVIITVH	E	130	144	HLA-DRB1*07:01	0%	Dengue virus 3	ELISPOT	IFNg release	Positive	0	0	0	0	0	0	0	0	0	0	0	0	0	0	0	0	0	0	0	0
539476	KKELLVTFKNAHAKK	E	511	525	HLA-DRB1*14:04	0%	Dengue virus 3	ELISPOT	IFNg release	Positive	0	0	0	0	0	0	0	0	0	0	0	0	0	0	0	0	0	0	0	0
539856	RKELLTTFKNAHAKK	E	511	525	HLA-DRB1*04:03	0%	Dengue virus 3	ELISPOT	IFNg release	Positive	0	0	0	0	0	0	0	0	0	0	0	0	0	0	0	0	0	0	0	0
591605	AFVLKKEVSETQHGT	E	303	317	HLA-DRB1*04:01	0%	Dengue virus 3	ELISPOT	IFNg release	Positive	0	0	0	0	0	0	0	0	0	0	0	0	0	0	0	0	0	0	0	0
591719	NEMILLTMKNRAWMV	E	474	488	HLA-DRB1*11:01	0%	Dengue virus 3	ELISPOT	IFNg release	Positive	0	0	0	0	0	0	0	0	0	0	0	0	0	0	0	0	0	0	0	0
867374	KAWIVHRQWFFDLPL	E	482	496	HLA-DQA1*01:01/DQB1*05:01	0%	Dengue virus 3	ELISPOT	IFNg release	Positive	0	0	0	0	0	0	0	0	0	0	0	0	0	0	0	0	0	0	0	0
869693	WMVHRQWLFDLPLPW	E	87	101	HLA-DQA1*01:01/DQB1*05:01	0%	Dengue virus 3	ELISPOT	IFNg release	Positive	0	0	0	0	0	0	0	0	0	0	0	0	0	0	0	0	0	0	0	0
539070	AKIVTAETRNSSFII	NS1	121	135	HLA-DRB1*13:01	0%	Dengue virus 3	ELISPOT	IFNg release	Positive	0	0	0	0	0	0	0	0	0	0	0	0	0	0	0	0	0	0	0	0
539457	KAKIVIAETQNSSFI	NS1	893	907	HLA-DRB1*13:01	0%	Dengue virus 3	ELISPOT	IFNg release	Positive	0	0	0	0	0	0	0	0	0	0	0	0	0	0	0	0	0	0	0	0
866698	FGVFTTNIWLKLRDV	NS1	933	947	HLA-DPB1*04:01	0%	Dengue virus 3	ELISPOT	IFNg release	Positive	0	0	0	0	0	0	0	0	0	0	0	0	0	0	0	0	0	0	0	0
195128	AGVLFMFVLLLSGQI	NS2a	1159	1173	HLA-DRB1*07:01	0%	Dengue virus 3	ELISPOT	IFNg release	Positive	0	0	0	0	0	0	0	0	0	0	0	0	0	0	0	0	0	0	0	0
195133	AHTFIMIGSNASDRM	NS2a	1179	1193	HLA-DRB1*04:01	0%	Dengue virus 3	ELISPOT	IFNg release	Positive	0	0	0	0	0	0	0	0	0	0	0	0	0	0	0	0	0	0	0	0
195166	ANTFIMIGSNASDRM	NS2a	1179	1193	HLA-DRB1*04:01	0%	Dengue virus 3	ELISPOT	IFNg release	Positive	0	0	0	0	0	0	0	0	0	0	0	0	0	0	0	0	0	0	0	0
195433	GVLFTFVLLLSGQIT	NS2a	1160	1174	HLA-DRB1*15:06	0%	Dengue virus 3	ELISPOT	IFNg release	Positive	0	0	0	0	0	0	0	0	0	0	0	0	0	0	0	0	0	0	0	0
195456	HMIAGILFMFVLLLS	NS2a	1156	1170	HLA-DRB1*11:01	0%	Dengue virus 3	ELISPOT	IFNg release	Positive	0	0	0	0	0	0	0	0	0	0	0	0	0	0	0	0	0	0	0	0
195630	LFTFVLLLSGQITWR	NS2a	1162	1176	HLA-DRB1*07:01	0%	Dengue virus 3	ELISPOT	IFNg release	Positive	0	0	0	0	0	0	0	0	0	0	0	0	0	0	0	0	0	0	0	0
195734	LVSLTCSNTILTLTV	NS2a	1274	1288	HLA-DRB1*07:01	0%	Dengue virus 3	ELISPOT	IFNg release	Positive	0	0	0	0	0	0	0	0	0	0	0	0	0	0	0	0	0	0	0	0
196123	VGVLFTFVLLLSGQI	NS2a	1159	1173	HLA-DRB1*07:01	0%	Dengue virus 3	ELISPOT	IFNg release	Positive	0	0	0	0	0	0	0	0	0	0	0	0	0	0	0	0	0	0	0	0
540048	VLFTFVLLLSGQITW	NS2a	1161	1175	HLA-DRB1*09:01	0%	Dengue virus 3	ELISPOT	IFNg release	Positive	0	0	0	0	0	0	0	0	0	0	0	0	0	0	0	0	0	0	0	0
865959	AGALFTFVLLLSGQI	NS2a	1159	1173	HLA-DPB1*04:02	0%	Dengue virus 3	ELISPOT	IFNg release	Positive	0	0	0	0	0	0	0	0	0	0	0	0	0	0	0	0	0	0	0	0
867702	LIATFKIQPFLTLGF	NS2a	1202	1216	HLA-DPB1*01:01	0%	Dengue virus 3	ELISPOT	IFNg release	Positive	0	0	0	0	0	0	0	0	0	0	0	0	0	0	0	0	0	0	0	0
869592	VQPFLALGFFLRKLT	NS2a	1208	1222	HLA-DPB1*01:01	0%	Dengue virus 3	ELISPOT	IFNg release	Positive	0	0	0	0	0	0	0	0	0	0	0	0	0	0	0	0	0	0	0	0
739173	IGLVSILASSLLRND	NS2b	1354	1368	HLA-DRB1*11:04	0%	Dengue virus 3	ELISPOT	IFNg release	Positive	0	0	0	0	0	0	0	0	0	0	0	0	0	0	0	0	0	0	0	0
195297	FHTMWHVTRGAVLTY	NS3	1519	1533	HLA-DRB1*07:01	0%	Dengue virus 3	ELISPOT	IFNg release	Positive	0	0	0	0	0	0	0	0	0	0	0	0	0	0	0	0	0	0	0	0
21420	GNEWITDFVGKTVWF	NS3	31	45	HLA-DRB1*15:01	0%	Dengue virus 3	ICS	IFNg release	Positive	0	0	0	0	0	0	0	0	0	0	0	0	0	0	0	0	0	0	0	0
49767	PTRVVAAEMEEAMKG	NS3	1697	1711	HLA-DRB1*15:01	0%	Dengue virus 3	ICS	IFNg release	Positive	0	0	0	0	0	0	0	0	0	0	0	0	0	0	0	0	0	0	0	0
539777	PKNFQTMPGIFQTTT	NS3	1579	1593	HLA-DRB1*04:03	0%	Dengue virus 3	ELISPOT	IFNg release	Positive	0	0	0	0	0	0	0	0	0	0	0	0	0	0	0	0	0	0	0	0
540037	VGKTVWFVPSIKAGN	NS3	1829	1843	HLA-DRB1*08:03	0%	Dengue virus 3	ELISPOT	IFNg release	Positive	0	0	0	0	0	0	0	0	0	0	0	0	0	0	0	0	0	0	0	0
72654	WITDFVGKTVW	NS3	1824	1834	HLA-DR15	0%	Dengue virus 3	51 chromium	cytotoxicity	Positive	0	0	0	0	0	0	0	0	0	0	0	0	0	0	0	0	0	0	0	0
867106	HTMWHVTRGAVLTYN	NS3	1520	1534	HLA-DQA1*05:01/DQB1*02:01	0%	Dengue virus 3	ELISPOT	IFNg release	Positive	0	0	0	0	0	0	0	0	0	0	0	0	0	0	0	0	0	0	0	0
869689	WHVTRGAVLTYNGKR	NS3	1523	1537	HLA-DQA1*05:01/DQB1*02:01	0%	Dengue virus 3	ELISPOT	IFNg release	Positive	0	0	0	0	0	0	0	0	0	0	0	0	0	0	0	0	0	0	0	0
195199	AYVVIGILTLAAIVA	NS4a	2227	2241	HLA-DRB1*01:01	0%	Dengue virus 3	ELISPOT	IFNg release	Positive	0	0	0	0	0	0	0	0	0	0	0	0	0	0	0	0	0	0	0	0
195742	MADVPLQWIASAIVL	NS4a	2189	2203	HLA-DRB1*07:01	0%	Dengue virus 3	ELISPOT	IFNg release	Positive	0	0	0	0	0	0	0	0	0	0	0	0	0	0	0	0	0	0	0	0
539900	SGMLWMADVPLQWIA	NS4a	2184	2198	HLA-DRB1*04:03	0%	Dengue virus 3	ELISPOT	IFNg release	Positive	0	0	0	0	0	0	0	0	0	0	0	0	0	0	0	0	0	0	0	0
739020	GIGKTSIGLICVIVS	NS4a	2169	2183	HLA-DRB1*11:04	0%	Dengue virus 3	ELISPOT	IFNg release	Positive	0	0	0	0	0	0	0	0	0	0	0	0	0	0	0	0	0	0	0	0
867986	LTLTAAVLLLATHYA	NS4b	2347	2361	HLA-DQA1*01:02/DQB1*06:02	0%	Dengue virus 3	ELISPOT	IFNg release	Positive	0	0	0	0	0	0	0	0	0	0	0	0	0	0	0	0	0	0	0	0
195408	GNIVASVNMVSRLLL	NS5	3	17	HLA-DRB1*03:01	0%	Dengue virus 3	ELISPOT	IFNg release	Positive	0	0	0	0	0	0	0	0	0	0	0	0	0	0	0	0	0	0	0	0
195780	NEDHWFSRDNSYSGV	NS5	2982	2996	HLA-DRB1*04:01	0%	Dengue virus 3	ELISPOT	IFNg release	Positive	0	0	0	0	0	0	0	0	0	0	0	0	0	0	0	0	0	0	0	0
196038	TGNIVASVNMVSRLL	NS5	2	16	HLA-DRB1*04:01	0%	Dengue virus 3	ELISPOT	IFNg release	Positive	0	0	0	0	0	0	0	0	0	0	0	0	0	0	0	0	0	0	0	0
196221	WLWRTLGRNKKPRLC	NS5	157	171	HLA-DRB1*14:02	0%	Dengue virus 3	ELISPOT	IFNg release	Positive	0	0	0	0	0	0	0	0	0	0	0	0	0	0	0	0	0	0	0	0
32944	KPWDVVPTV	NS5	2819	2827	HLA class II	0%	Dengue virus 3	ELISA	IL-2 release	Positive	0	0	0	0	0	0	0	0	0	0	0	0	0	0	0	0	0	0	0	0
539444	IVASVNMVSRLLLNR	NS5	2717	2731	HLA-DRB1*12:02	0%	Dengue virus 3	ELISPOT	IFNg release	Positive	0	0	0	0	0	0	0	0	0	0	0	0	0	0	0	0	0	0	0	0
539726	NIVASVNMVSRLLLN	NS5	2716	2730	HLA-DRB1*15:06	0%	Dengue virus 3	ELISPOT	IFNg release	Positive	0	0	0	0	0	0	0	0	0	0	0	0	0	0	0	0	0	0	0	0
539739	NMEVQLVRQMEGEGV	NS5	3103	3117	HLA-DRB1*13:01	0%	Dengue virus 3	ELISPOT	IFNg release	Positive	0	0	0	0	0	0	0	0	0	0	0	0	0	0	0	0	0	0	0	0
540025	VASVNMVSRLLLNRF	NS5	2718	2732	HLA-DRB1*14:04	0%	Dengue virus 3	ELISPOT	IFNg release	Positive	0	0	0	0	0	0	0	0	0	0	0	0	0	0	0	0	0	0	0	0
591602	ACLGKAYAQMWTLMY	NS5	3242	3256	HLA-DRB1*04:01	0%	Dengue virus 3	ELISPOT	IFNg release	Positive	0	0	0	0	0	0	0	0	0	0	0	0	0	0	0	0	0	0	0	0
738537	ASVNMVSRLLLNRFT	NS5	7	21	HLA-DRB1*14:06	0%	Dengue virus 3	ELISPOT	IFNg release	Positive	0	0	0	0	0	0	0	0	0	0	0	0	0	0	0	0	0	0	0	0
740771	YGLNTFTNMEVQLVR	NS5	3096	3110	HLA-DRB1*04:07	0%	Dengue virus 3	ELISPOT	IFNg release	Positive	0	0	0	0	0	0	0	0	0	0	0	0	0	0	0	0	0	0	0	0
195830	PGFTILALFLAHYIG	PreM	245	259	HLA-DRB1*15:06	100%	Dengue virus 3	ELISPOT	IFNg release	Positive	1	1	1	1	1	1	1	1	1	1	1	1	1	1	1	1	1	1	1	1
867082	HPGFTILALFLAHYI	PreM	244	258	HLA-DPB1*02:01	100%	Dengue virus 3	ELISPOT	IFNg release	Positive	1	1	1	1	1	1	1	1	1	1	1	1	1	1	1	1	1	1	1	1
119134	EGKVVQYENLKYTVI	E	126	140	HLA-DR2	100%	Dengue virus 3	ELISPOT	IFNg release	Positive	1	1	1	1	1	1	1	1	1	1	1	1	1	1	1	1	1	1	1	1
119289	NGRLITANPVVTKKE	E	626	640	HLA-DRB1*08:02	100%	Dengue virus 3	ELISPOT	IFNg release	Positive	1	1	1	1	1	1	1	1	1	1	1	1	1	1	1	1	1	1	1	1
13376	EMILLTMKNKAWMVH	E	473	487	HLA-DRB1*13:01	100%	Dengue virus 3	ELISPOT	IFNg release	Positive	1	1	1	1	1	1	1	1	1	1	1	1	1	1	1	1	1	1	1	1
195215	DFNEMILLTMKNKAW	E	470	484	HLA-DRB1*14:04	100%	Dengue virus 3	ELISPOT	IFNg release	Positive	1	1	1	1	1	1	1	1	1	1	1	1	1	1	1	1	1	1	1	1
195341	FVLKKEVSETQHGTI	E	584	598	HLA-DRB1*04:01	100%	Dengue virus 3	ELISPOT	IFNg release	Positive	1	1	1	1	1	1	1	1	1	1	1	1	1	1	1	1	1	1	1	1
195365	GHLKCRLKMDKLELK	E	559	573	HLA-DRB1*03:01	100%	Dengue virus 3	ELISPOT	IFNg release	Positive	1	1	1	1	1	1	1	1	1	1	1	1	1	1	1	1	1	1	1	1
196033	TFVLKKEVSETQHGT	E	583	597	HLA-DRB1*04:01	100%	Dengue virus 3	ELISPOT	IFNg release	Positive	1	1	1	1	1	1	1	1	1	1	1	1	1	1	1	1	1	1	1	1
196172	VQYENLKYTVIITVH	E	410	424	HLA-DRB1*07:01	100%	Dengue virus 3	ELISPOT	IFNg release	Positive	1	1	1	1	1	1	1	1	1	1	1	1	1	1	1	1	1	1	1	1
539400	IEGKVVQYENLKYTV	E	405	419	HLA-DRB1*15:02	100%	Dengue virus 3	ELISPOT	IFNg release	Positive	1	1	1	1	1	1	1	1	1	1	1	1	1	1	1	1	1	1	1	1
539710	NEMILLTMKNKAWMV	E	472	486	HLA-DRB1*04:03	100%	Dengue virus 3	ELISPOT	IFNg release	Positive	1	1	1	1	1	1	1	1	1	1	1	1	1	1	1	1	1	1	1	1
866574	EIQNSGGTSIFAGHL	E	547	561	HLA-DQA1*01:02/DQB1*06:02	100%	Dengue virus 3	ELISPOT	IFNg release	Positive	1	1	1	1	1	1	1	1	1	1	1	1	1	1	1	1	1	1	1	1
867377	KCRLKMDKLELKGMS	E	562	576	HLA-DRB3*02:02	100%	Dengue virus 3	ELISPOT	IFNg release	Positive	1	1	1	1	1	1	1	1	1	1	1	1	1	1	1	1	1	1	1	1
195164	ANELNYILWENNIKL	NS1	845	859	HLA-DRB1*03:01	100%	Dengue virus 3	ELISPOT	IFNg release	Positive	1	1	1	1	1	1	1	1	1	1	1	1	1	1	1	1	1	1	1	1
195692	LNYILWENNIKLTVV	NS1	848	862	HLA-DRB1*03:01	100%	Dengue virus 3	ELISPOT	IFNg release	Positive	1	1	1	1	1	1	1	1	1	1	1	1	1	1	1	1	1	1	1	1
195782	NELNYILWENNIKLT	NS1	846	860	HLA-DRB1*03:01	100%	Dengue virus 3	ELISPOT	IFNg release	Positive	1	1	1	1	1	1	1	1	1	1	1	1	1	1	1	1	1	1	1	1
866700	FGVFTTNIWLKLREV	NS1	933	947	HLA-DPB1*01:01	100%	Dengue virus 3	ELISPOT	IFNg release	Positive	1	1	1	1	1	1	1	1	1	1	1	1	1	1	1	1	1	1	1	1
867042	HADMGYWIESQKNGS	NS1	968	982	HLA-DQA1*01:01/DQB1*05:01	100%	Dengue virus 3	ELISPOT	IFNg release	Positive	1	1	1	1	1	1	1	1	1	1	1	1	1	1	1	1	1	1	1	1
195134	AHTLIMIGSNASDRM	NS2a	1179	1193	HLA-DRB1*04:01	100%	Dengue virus 3	ELISPOT	IFNg release	Positive	1	1	1	1	1	1	1	1	1	1	1	1	1	1	1	1	1	1	1	1

195197	AWRTATLILAGVSLL	NS2a	1289	1303	HLA-DRB1*01:01	100%	Dengue virus 3	ELISPOT	IFNg release	Positive	1	1	1	1	1	1	1	1	1	1	1	1	1	1	1	1	1	1	1	1
195222	DMAHTLIMIGSNASD	NS2a	1177	1191	HLA-DRB1*04:01	100%	Dengue virus 3	ELISPOT	IFNg release	Positive	1	1	1	1	1	1	1	1	1	1	1	1	1	1	1	1	1	1	1	1
195337	FTLTVAWRTATLILA	NS2a	1284	1298	HLA-DRB1*07:01	100%	Dengue virus 3	ELISPOT	IFNg release	Positive	1	1	1	1	1	1	1	1	1	1	1	1	1	1	1	1	1	1	1	1
195342	FVLLLSGQITWRDMA	NS2a	1165	1179	HLA-DRB1*01:01	100%	Dengue virus 3	ELISPOT	IFNg release	Positive	1	1	1	1	1	1	1	1	1	1	1	1	1	1	1	1	1	1	1	1
195462	HTLIMIGSNASDRMG	NS2a	1180	1194	HLA-DRB1*04:01	100%	Dengue virus 3	ELISPOT	IFNg release	Positive	1	1	1	1	1	1	1	1	1	1	1	1	1	1	1	1	1	1	1	1
195481	IFTLTVAWRTATLIL	NS2a	1283	1297	HLA-DRB1*07:01	100%	Dengue virus 3	ELISPOT	IFNg release	Positive	1	1	1	1	1	1	1	1	1	1	1	1	1	1	1	1	1	1	1	1
195609	LALGFFLRKLTSREN	NS2a	1212	1226	HLA-DRB1*11:01	100%	Dengue virus 3	ELISPOT	IFNg release	Positive	1	1	1	1	1	1	1	1	1	1	1	1	1	1	1	1	1	1	1	1
195644	LIMIGSNASDRMGMG	NS2a	1182	1196	HLA-DRB1*04:01	100%	Dengue virus 3	ELISPOT	IFNg release	Positive	1	1	1	1	1	1	1	1	1	1	1	1	1	1	1	1	1	1	1	1
195729	LTVAWRTATLILAGV	NS2a	1286	1300	HLA-DPB1*04:02	100%	Dengue virus 3	ELISPOT	IFNg release	Positive	1	1	1	1	1	1	1	1	1	1	1	1	1	1	1	1	1	1	1	1
195743	MAHTLIMIGSNASDR	NS2a	1178	1192	HLA-DRB1*04:01	100%	Dengue virus 3	ELISPOT	IFNg release	Positive	1	1	1	1	1	1	1	1	1	1	1	1	1	1	1	1	1	1	1	1
195757	MGVTYLALIATFKIQ	NS2a	1195	1209	HLA-DRB1*07:01	100%	Dengue virus 3	ELISPOT	IFNg release	Positive	1	1	1	1	1	1	1	1	1	1	1	1	1	1	1	1	1	1	1	1
195819	NTIFTLTVAWRTATL	NS2a	1281	1295	HLA-DRB1*07:01	100%	Dengue virus 3	ELISPOT	IFNg release	Positive	1	1	1	1	1	1	1	1	1	1	1	1	1	1	1	1	1	1	1	1
539075	ALGFFLRKLTSRENL	NS2a	1213	1227	HLA-DRB1*12:02	100%	Dengue virus 3	ELISPOT	IFNg release	Positive	1	1	1	1	1	1	1	1	1	1	1	1	1	1	1	1	1	1	1	1
539370	GVTYLALIATFKIQP	NS2a	1196	1210	HLA-DRB1*10:01	100%	Dengue virus 3	ELISPOT	IFNg release	Positive	1	1	1	1	1	1	1	1	1	1	1	1	1	1	1	1	1	1	1	1
539429	IQPFLALGFFLRKLT	NS2a	1208	1222	HLA-DRB1*15:06	100%	Dengue virus 3	ELISPOT	IFNg release	Positive	1	1	1	1	1	1	1	1	1	1	1	1	1	1	1	1	1	1	1	1
539568	LIATFKIQPFLALGF	NS2a	1202	1216	HLA-DRB1*15:06	100%	Dengue virus 3	ELISPOT	IFNg release	Positive	1	1	1	1	1	1	1	1	1	1	1	1	1	1	1	1	1	1	1	1
540093	VTYLALIATFKIQPF	NS2a	1197	1211	HLA-DRB1*04:03	100%	Dengue virus 3	ELISPOT	IFNg release	Positive	1	1	1	1	1	1	1	1	1	1	1	1	1	1	1	1	1	1	1	1
867551	LALIATFKIQPFLAL	NS2a	1200	1214	HLA-DPB1*04:01	100%	Dengue virus 3	ELISPOT	IFNg release	Positive	1	1	1	1	1	1	1	1	1	1	1	1	1	1	1	1	1	1	1	1
868303	PFLALGFFLRKLTSR	NS2a	1210	1224	HLA-DPB1*01:01	100%	Dengue virus 3	ELISPOT	IFNg release	Positive	1	1	1	1	1	1	1	1	1	1	1	1	1	1	1	1	1	1	1	1
868551	QPFLALGFFLRKLTS	NS2a	1209	1223	HLA-DPB1*02:01	100%	Dengue virus 3	ELISPOT	IFNg release	Positive	1	1	1	1	1	1	1	1	1	1	1	1	1	1	1	1	1	1	1	1
869256	TLTVAWRTATLILAG	NS2a	1285	1299	HLA-DPB1*04:02	100%	Dengue virus 3	ELISPOT	IFNg release	Positive	1	1	1	1	1	1	1	1	1	1	1	1	1	1	1	1	1	1	1	1
195403	GLVSILASSLLRNDV	NS2b	1355	1369	HLA-DRB1*01:01	100%	Dengue virus 3	ELISPOT	IFNg release	Positive	1	1	1	1	1	1	1	1	1	1	1	1	1	1	1	1	1	1	1	1
196152	VMAVGLVSILASSLL	NS2b	1351	1365	HLA-DRB1*07:01	100%	Dengue virus 3	ELISPOT	IFNg release	Positive	1	1	1	1	1	1	1	1	1	1	1	1	1	1	1	1	1	1	1	1
539100	AVGLVSILASSLLRN	NS2b	1353	1367	HLA-DRB1*10:01	100%	Dengue virus 3	ELISPOT	IFNg release	Positive	1	1	1	1	1	1	1	1	1	1	1	1	1	1	1	1	1	1	1	1
540087	VSGIFPYSIPATLLV	NS2b	1449	1463	HLA-DRB1*09:01	100%	Dengue virus 3	ELISPOT	IFNg release	Positive	1	1	1	1	1	1	1	1	1	1	1	1	1	1	1	1	1	1	1	1
1067120	GKVVGLYGNGVVTKN	NS3	1617	1631	HLA-DRB1*15:01	100%	Dengue virus 3	ELISPOT	IFNg release	Positive	1	1	1	1	1	1	1	1	1	1	1	1	1	1	1	1	1	1	1	1
150617	TFTMRLLSPVRVPNY	NS3	1738	1752	HLA-DRB1*01:01	100%	Dengue virus 3	ELISPOT	IFNg release	Positive	1	1	1	1	1	1	1	1	1	1	1	1	1	1	1	1	1	1	1	1
190307	REIVDLMCHATF	NS3	255	266	HLA-DPA1*01:03/DPB1*02:01	100%	Dengue virus 3	ELISA	IL-2 release	Positive	1	1	1	1	1	1	1	1	1	1	1	1	1	1	1	1	1	1	1	1
190710	ANCLRKNGKKVIQLS	NS3	1846	1860	HLA-DRB1*13:01	100%	Dengue virus 3	ELISPOT	IFNg release	Positive	1	1	1	1	1	1	1	1	1	1	1	1	1	1	1	1	1	1	1	1
190774	GKTVWFVPSIKAGND	NS3	357	371	HLA-DR2	100%	Dengue virus 3	ELISPOT	IFNg release	Positive	1	1	1	1	1	1	1	1	1	1	1	1	1	1	1	1	1	1	1	1
195243	EGKVVGLYGNGVVTK	NS3	5	19	HLA-DRB1*15:01	100%	Dengue virus 3	ELISPOT	IFNg release	Positive	1	1	1	1	1	1	1	1	1	1	1	1	1	1	1	1	1	1	1	1
195430	GVFHTMWHVTRGAVL	NS3	1517	1531	HLA-DRB1*07:01	100%	Dengue virus 3	ELISPOT	IFNg release	Positive	1	1	1	1	1	1	1	1	1	1	1	1	1	1	1	1	1	1	1	1
28422	IRYQTTATK	NS3	241	249	HLA-DR15	100%	Dengue virus 3	51 chromium	cytotoxicity	Positive	1	1	1	1	1	1	1	1	1	1	1	1	1	1	1	1	1	1	1	1
45670	NREGKVVGLYGNGVV	NS3	141	155	HLA-DRB1*15:01	100%	Dengue virus 3	multimer/tetramer	qualitative binding	Positive	1	1	1	1	1	1	1	1	1	1	1	1	1	1	1	1	1	1	1	1
539052	AGKTVWFVPSIKAGN	NS3	1829	1843	HLA-DRB1*08:03	100%	Dengue virus 3	ELISPOT	IFNg release	Positive	1	1	1	1	1	1	1	1	1	1	1	1	1	1	1	1	1	1	1	1
539578	LKGLPIRYQTTATKS	NS3	1709	1723	HLA-DRB1*13:01	100%	Dengue virus 3	ELISPOT	IFNg release	Positive	1	1	1	1	1	1	1	1	1	1	1	1	1	1	1	1	1	1	1	1
539607	LPIRYQTTATKSEHT	NS3	1712	1726	HLA-DRB1*13:01	100%	Dengue virus 3	ELISPOT	IFNg release	Positive	1	1	1	1	1	1	1	1	1	1	1	1	1	1	1	1	1	1	1	1
539844	REGKVVGLYGNGVVT	NS3	1615	1629	HLA-DRB1*01:02	100%	Dengue virus 3	ELISPOT	IFNg release	Positive	1	1	1	1	1	1	1	1	1	1	1	1	1	1	1	1	1	1	1	1
539991	TMRLLSPVRVPNYNL	NS3	1740	1754	HLA-DRB1*13:01	100%	Dengue virus 3	ELISPOT	IFNg release	Positive	1	1	1	1	1	1	1	1	1	1	1	1	1	1	1	1	1	1	1	1
68015	VDLMCHATFT	NS3	258	267	HLA-DPw2	100%	Dengue virus 3	51 chromium	cytotoxicity	Positive	1	1	1	1	1	1	1	1	1	1	1	1	1	1	1	1	1	1	1	1
866771	FQTTTGEIGAIALDF	NS3	1589	1603	HLA-DQA1*03:01/DQB1*03:02	100%	Dengue virus 3	ELISPOT	IFNg release	Positive	1	1	1	1	1	1	1	1	1	1	1	1	1	1	1	1	1	1	1	1
869278	TMWHVTRGAVLTHNG	NS3	1521	1535	HLA-DQA1*05:01/DQB1*02:01	100%	Dengue virus 3	ELISPOT	IFNg release	Positive	1	1	1	1	1	1	1	1	1	1	1	1	1	1	1	1	1	1	1	1
167755	IALDLVTEIGRVPSHLAHRT	NS4a	2	21	HLA class II	100%	Dengue virus 3	ELISPOT	IFNg release	Positive	1	1	1	1	1	1	1	1	1	1	1	1	1	1	1	1	1	1	1	1
195346	GAGLAFSIMKSVGTG	NS4b	2474	2488	HLA-DRB1*04:01	100%	Dengue virus 3	ELISPOT	IFNg release	Positive	1	1	1	1	1	1	1	1	1	1	1	1	1	1	1	1	1	1	1	1
195607	LAFSIMKSVGTGKRG	NS4b	2477	2491	HLA-DRB1*04:01	100%	Dengue virus 3	ELISPOT	IFNg release	Positive	1	1	1	1	1	1	1	1	1	1	1	1	1	1	1	1	1	1	1	1
195617	LCAVQLLLMRTSWAL	NS4b	2418	2432	HLA-DRB1*15:01	100%	Dengue virus 3	ELISPOT	IFNg release	Positive	1	1	1	1	1	1	1	1	1	1	1	1	1	1	1	1	1	1	1	1
195624	LDPVIYDSKFEKQLG	NS4b	2397	2411	HLA-DRB1*03:01	100%	Dengue virus 3	ELISPOT	IFNg release	Positive	1	1	1	1	1	1	1	1	1	1	1	1	1	1	1	1	1	1	1	1
195664	LLLMRTSWALCEALT	NS4b	2423	2437	HLA-DRB1*04:01	100%	Dengue virus 3	ELISPOT	IFNg release	Positive	1	1	1	1	1	1	1	1	1	1	1	1	1	1	1	1	1	1	1	1
195871	QVMLLVLCAVQLLLM	NS4b	2412	2426	HLA-DRB1*01:01	100%	Dengue virus 3	ELISPOT	IFNg release	Positive	1	1	1	1	1	1	1	1	1	1	1	1	1	1	1	1	1	1	1	1
196137	VLCAVQLLLMRTSWA	NS4b	2417	2431	HLA-DRB1*12:02	100%	Dengue virus 3	ELISPOT	IFNg release	Positive	1	1	1	1	1	1	1	1	1	1	1	1	1	1	1	1	1	1	1	1
196155	VMLLVLCAVQLLLMR	NS4b	2413	2427	HLA-DRB1*03:01	100%	Dengue virus 3	ELISPOT	IFNg release	Positive	1	1	1	1	1	1	1	1	1	1	1	1	1	1	1	1	1	1	1	1
738453	AGLAFSIMKSVGTGK	NS4b	2475	2489	HLA-DRB1*04:07	100%	Dengue virus 3	ELISPOT	IFNg release	Positive	1	1	1	1	1	1	1	1	1	1	1	1	1	1	1	1	1	1	1	1
868878	SLAAIANQAVVLMGL	NS4b	2306	2320	HLA-DQA1*01:02/DQB1*06:02	100%	Dengue virus 3	ELISPOT	IFNg release	Positive	1	1	1	1	1	1	1	1	1	1	1	1	1	1	1	1	1	1	1	1
167769	KKLNQLSRKEFDL	NS5	15	27	HLA-DP	100%	Dengue virus 3	ELISPOT	IFNg release	Positive	1	1	1	1	1	1	1	1	1	1	1	1	1	1	1	1	1	1	1	1
195136	AIFKLTYQNKVVKVQ	NS5	3056	3070	HLA-DRB1*09:01	100%	Dengue virus 3	ELISPOT	IFNg release	Positive	1	1	1	1	1	1	1	1	1	1	1	1	1	1	1	1	1	1	1	1
195204	CLGKAYAQMWSLMYF	NS5	3243	3257	HLA-DRB1*04:01	100%	Dengue virus 3	ELISPOT	IFNg release	Positive	1	1	1	1	1	1	1	1	1	1	1	1	1	1	1	1	1	1	1	1
195273	ETKGVERLKRMAISG	NS5	3138	3152	HLA-DRB1*11:01	100%	Dengue virus 3	ELISPOT	IFNg release	Positive	1	1	1	1	1	1	1	1	1	1	1	1	1	1	1	1	1	1	1	1
195437	GYILRDISKIPGGAM	NS5	43	57	HLA-DRB1*08:02	100%	Dengue virus 3	ELISPOT	IFNg release	Positive	1	1	1	1	1	1	1	1	1	1	1	1	1	1	1	1	1	1	1	1
195568	KHGGMLVRNPLSRNS	NS5	2689	2703	HLA-DRB1*08:02	100%	Dengue virus 3	ELISPOT	IFNg release	Positive	1	1	1	1	1	1	1	1	1	1	1	1	1	1	1	1	1	1	1	1
195641	LHKLGYILRDISKIP	NS5	39	53	HLA-DRB1*03:01	100%	Dengue virus 3	ELISPOT	IFNg release	Positive	1	1	1	1	1	1	1	1	1	1	1	1	1	1	1	1	1	1	1	1
196222	WNIVKLMSGKDVFYL	NS5	2611	2625	HLA-DRB1*15:01	100%	Dengue virus 3	ELISPOT	IFNg release	Positive	1	1	1	1	1	1	1	1	1	1	1	1	1	1	1	1	1	1	1	1
196224	WSLMYFHRRDLRLAS	NS5	3252	3266	HLA-DRB1*03:01	100%	Dengue virus 3	ELISPOT	IFNg release	Positive	1	1	1	1	1	1	1	1	1	1	1	1	1	1	1	1	1	1	1	1
539600	LMYFHRRDLRLASNA	NS5	3254	3268	HLA-DRB1*13:01	100%	Dengue virus 3	ELISPOT	IFNg release	Positive	1	1	1	1	1	1	1	1	1	1	1	1	1	1	1	1	1	1	1	1
539604	LNTFTNMEAQLVRQM	NS5	3098	3112	HLA-DRB1*10:01	100%	Dengue virus 3	ELISPOT	IFNg release	Positive	1	1	1	1	1	1	1	1	1	1	1	1	1	1	1	1	1	1	1	1
539704	NAIFKLTYQNKVVKV	NS5	3055	3069	HLA-DRB1*09:01	100%	Dengue virus 3	ELISPOT	IFNg release	Positive	1	1	1	1	1	1	1	1	1	1	1	1	1	1	1	1	1	1	1	1
539872	RRDLRLASNAICSAV	NS5	3259	3273	HLA-DRB1*04:03	100%	Dengue virus 3	ELISPOT	IFNg release	Positive	1	1	1	1	1	1	1	1	1	1	1	1	1	1	1	1	1	1	1	1
539916	SLMYFHRRDLRLASN	NS5	3253	3267	HLA-DRB1*13:01	100%	Dengue virus 3	ELISPOT	IFNg release	Positive	1	1	1	1	1	1	1	1	1	1	1	1	1	1	1	1	1	1	1	1
540019	TYGLNTFTNMEAQLV	NS5	3095	3109	HLA-DRB1*04:03	100%	Dengue virus 3	ELISPOT	IFNg release	Positive	1	1	1	1	1	1	1	1	1	1	1	1	1	1	1	1	1	1	1	1
540152	YKTWAYHGSYEVKAT	NS5	2789	2803	HLA-DRB1*09:01	100%	Dengue virus 3	ELISPOT	IFNg release	Positive	1	1	1	1	1	1	1	1	1	1	1	1	1	1	1	1	1	1	1	1
738829	ERELHKLGKCGSCVY	NS5	2927	2941	HLA-DRB1*11:04	100%	Dengue virus 3	ELISPOT	IFNg release	Positive	1	1	1	1	1	1	1	1	1	1	1	1	1	1	1	1	1	1	1	1
867376	KAYAQMWSLMYFHRR	NS5	3246	3260	HLA-DRB5*01:01	100%	Dengue virus 3	ELISPOT	IFNg release	Positive	1	1	1	1	1	1	1	1	1	1	1	1	1	1	1	1	1	1	1	1
869489	VKPIDDRFANALLAL	NS5	3157	3171	HLA-DPB1*01:01	100%	Dengue virus 3	ELISPOT	IFNg release	Positive	1	1	1	1	1	1	1	1	1	1	1	1	1	1	1	1	1	1	1	1
1068391	TGKPSINMLKRVRNR	C	8	22	HLA-DRB1*11:01	81% - <100%	Dengue virus 3	ELISPOT	IFNg release	Positive-Low	1	1	1	1	1	1	1	1	1	1	1	1	1	1	1	1	1	1	0	1
195303	FKKEISNMLSIINKR	C	37	51	HLA-DRB1*04:01	81% - <100%	Dengue virus 3	ELISPOT	IFNg release	Positive	1	0	0	1	1	1	1	1	1	1	1	1	1	1	0	1	1	1	1	1
195306	FLRFLAIPPTAGVLA	C	6	20	HLA-DRB1*01:01	81% - <100%	Dengue virus 3	ELISPOT	IFNg release	Positive	1	1	1	1	1	1	1	1	1	1	1	1	1	1	0	1	1	1	1	1
195469	IAFLRFLAIPPTAGV	C	4	18	HLA-DRB1*04:01	81% - <100%	Dengue virus 3	ELISPOT	IFNg release	Positive	1	1	1	1	1	1	1	1	1	1	1	1	1	1	0	1	1	1	1	1
195518	INMLKRVRNRVSTGS	C	13	27	HLA-DRB1*08:02	81% - <100%	Dengue virus 3	ELISPOT	IFNg release	Positive	1	1	1	1	1	1	1	1	1	1	1	1	1	1	1	1	1	1	0	1
195536	ISNMLSIINKRKKTS	C	41	55	HLA-DRB1*11:01	81% - <100%	Dengue virus 3	ELISPOT	IFNg release	Positive	1	1	1	1	1	1	1	1	1	1	1	1	1	1	0	1	1	1	1	1
195559	KEISNMLSIINKRKK	C	39	53	HLA-DRB1*04:01	81% - <100%	Dengue virus 3	ELISPOT	IFNg release	Positive	1	0	0	1	1	1	1	1	1	1	1	1	1	1	0	1	1	1	1	1
195611	LARWGTFKKSGAIKV	C	19	33	HLA-DRB1*07:01	81% - <100%	Dengue virus 3	ELISPOT	IFNg release	Positive	1	1	1	1	0	1	1	1	1	1	1	1	1	1	0	1	1	1	1	1
195649	LKGFKKEISNMLSII	C	34	48	HLA-DRB1*07:01	81% - <100%	Dengue virus 3	ELISPOT	IFNg release	Positive	1	0	0	1	1	1	1	1	1	1	1	1	1	1	0	1	1	1	1	1
195961	SINMLKRVRNRVSTG	C	12	26	HLA-DRB1*08:02	81% - <100%	Dengue virus 3	ELISPOT	IFNg release	Positive	1	1	1	1	1	1	1	1	1	1	1	1	1	1	1	1	1	1	0	1
36858	LKGFKKEISNML	C	81	92	HLA-DPw4	81% - <100%	Dengue virus 3	ICS	IFNg release	Positive	1	0	0	1	1	1	1	1	1	1	1	1	1	1	0	1	1	1	1	1
539502	KPSINMLKRVRNRVS	C	10	24	HLA-DRB1*08:03	81% - <100%	Dengue virus 3	ELISPOT	IFNg release	Positive	1	1	1	1	1	1	1	1	1	1	1	1	1	1	1	1	1	1	0	1
539787	PSINMLKRVRNRVST	C	11	25	HLA-DRB1*14:04	81% - <100%	Dengue virus 3	ELISPOT	IFNg release	Positive	1	1	1	1	1	1	1	1	1	1	1	1	1	1	1	1	1	1	0	1
539926	SNMLSIINKRKKTSL	C	42	56	HLA-DRB1*12:02	81% - <100%	Dengue virus 3	ELISPOT	IFNg release	Positive	1	1	1	1	1	1	1	1	1	1	1	1	1	1	0	1	1	1	1	1
540121	WGTFKKSGAIKVLKG	C	22	36	HLA-DRB1*09:01	81% - <100%	Dengue virus 3	ELISPOT	IFNg release	Positive	1	1	1	1	0	1	1	1	1	1	1	1	1	1	1	1	1	1	1	1
738443	AFLRFLAIPPTAGVL	C	52	66	HLA-DRB1*04:07	81% - <100%	Dengue virus 3	ELISPOT	IFNg release	Positive	1	1	1	1	1	1	1	1	1	1	1	1	1	1	0	1	1	1	1	1
740207	RWGTFKKSGAIKVLK	C	68	82	HLA-DRB1*14:02	81% - <100%	Dengue virus 3	ELISPOT	IFNg release	Positive	1	1	1	1	0	1	1	1	1	1	1	1	1	1	1	1	1	1	1	1
195602	KVVIFILLMLVTPSM	PreM	265	279	HLA-DRB1*01:01	81% - <100%	Dengue virus 3	ELISPOT	IFNg release	Positive	1	1	1	1	1	1	1	1	1	1	1	1	1	1	0	1	1	1	1	1
195726	LTQKVVIFILLMLVT	PreM	262	276	HLA-DRB1*11:01	81% - <100%	Dengue virus 3	ELISPOT	IFNg release	Positive	1	1	1	1	1	1	1	1	1	1	1	1	1	1	0	1	1	1	1	1
196203	VVIFILLMLVTPSMT	PreM	266	280	HLA-DRB1*07:01	81% - <100%	Dengue virus 3	ELISPOT	IFNg release	Positive	1	1	1	1	1	1	1	1	1	1	1	1	1	1	0	1	1	1	1	1
540004	TSLTQKVVIFILLML	PreM	260	274	HLA-DPB1*04:02	81% - <100%	Dengue virus 3	ELISPOT	IFNg release	Positive	1	1	1	1	1	1	1	1	1	1	1	1	1	1	0	1	1	1	1	1
869302	TQKVVIFILLMLVTP	PreM	263	277	HLA-DPB1*04:02	81% - <100%	Dengue virus 3	ELISPOT	IFNg release	Positive	1	1	1	1	1	1	1	1	1	1	1	1	1	1	0	1	1	1	1	1
119327	RKELLVTFKNAHAKK	E	231	245	HLA-DR2	81% - <100%	Dengue virus 3	ELISPOT	IFNg release	Positive	1	1	1	1	1	1	1	1	1	1	1	1	1	1	0	1	1	1	1	1
195252	ELLVTFKNAHAKKQE	E	513	527	HLA-DRB1*04:01	81% - <100%	Dengue virus 3	ELISPOT	IFNg release	Positive	1	1	1	1	1	1	1	1	1	1	1	1	1	1	0	1	1	1	1	1
195416	GRLITANPVVTKKEE	E	627	641	HLA-DRB1*08:02	81% - <100%	Dengue virus 3	ELISPOT	IFNg release	Positive	1	1	1	1	1	1	1	1	0	1	1	1	1	1	1	1	1	1	1	1
195813	NRKELLVTFKNAHAK	E	510	524	HLA-DRB1*04:01	81% - <100%	Dengue virus 3	ELISPOT	IFNg release	Positive	1	1	1	1	1	1	1	1	1	1	1	1	1	1	0	1	1	1	1	1
539460	KELLVTFKNAHAKKQ	E	512	526	HLA-DRB1*08:03	81% - <100%	Dengue virus 3	ELISPOT	IFNg release	Positive	1	1	1	1	1	1	1	1	1	1	1	1	1	1	0	1	1	1	1	1

539559	LGKMVHQIFGSAYTA	E	710	724	HLA-DRB1*04:03	81% - <100%	Dengue virus 3	ELISPOT	IFNg release	Positive	1	1	1	1	0	1	1	1	1	1	1	1	1	1	1	0	1	1	1	1
739011	GGVLNSLGKMVHQIF	E	704	718	HLA-DRB1*11:04	81% - <100%	Dengue virus 3	ELISPOT	IFNg release	Positive	1	1	1	1	0	1	1	1	1	1	1	1	1	1	1	0	1	1	1	1
739041	GMSYAMCTNTFVLKK	E	574	588	HLA-DRB1*04:07	81% - <100%	Dengue virus 3	ELISPOT	IFNg release	Positive	1	1	1	1	0	1	1	1	1	1	1	1	1	1	1	1	1	1	1	1
739344	KMVHQIFGSAYTALF	E	31	45	HLA-DRB3*02:02	81% - <100%	Dengue virus 3	ELISPOT	IFNg release	Positive	1	1	1	1	0	1	1	1	1	1	1	1	1	1	1	0	1	1	1	1
740409	SVGGVLNSLGKMVHQ	E	702	716	HLA-DRB1*11:04	81% - <100%	Dengue virus 3	ELISPOT	IFNg release	Positive	1	1	1	1	0	1	1	1	1	1	1	1	1	1	1	0	1	1	1	1
741746	MRCVGVGNRDFVEGLSGATW	E	281	300	HLA class II	81% - <100%	Dengue virus 3	ELISPOT	IFNg release	Positive	1	1	0	1	1	1	1	1	1	1	1	1	1	1	1	1	1	1	1	1
868175	MVHRQWFFDLPLPWT	E	485	499	HLA-DQA1*01:01/DQB1*05:01	81% - <100%	Dengue virus 3	ELISPOT	IFNg release	Positive	1	1	1	1	1	1	1	1	1	1	1	1	1	0	1	1	1	1	1	1
190705	AKIVTAETQNSSFII	NS1	894	908	HLA-DRB1*04:01	81% - <100%	Dengue virus 3	ELISPOT	IFNg release	Positive	0	1	1	1	1	1	1	1	1	1	1	1	1	0	1	1	1	1	1	1
190996	TEQYKFQADSPKRLA	NS1	802	816	HLA-DRB5*01:01	81% - <100%	Dengue virus 3	ELISPOT	IFNg release	Positive	1	1	1	1	0	1	1	1	1	1	1	1	1	1	1	1	1	1	1	1
195419	GSWKLEKASLIEVKT	NS1	981	995	HLA-DRB1*01:01	81% - <100%	Dengue virus 3	ELISPOT	IFNg release	Positive	1	1	1	1	0	1	1	1	1	1	1	1	1	1	1	1	1	0	1	1
195577	KLTVVVGDTIGVLEQ	NS1	858	872	HLA-DRB1*03:01	81% - <100%	Dengue virus 3	ELISPOT	IFNg release	Positive	1	1	1	1	1	1	1	1	0	1	1	1	1	0	1	1	1	1	1	1
196235	YKFQADSPKRLATAI	NS1	805	819	HLA-DRB1*03:01	81% - <100%	Dengue virus 3	ELISPOT	IFNg release	Positive	1	1	1	1	0	1	1	1	1	1	1	1	1	1	1	1	1	1	1	1
539717	NGSWKLEKASLIEVK	NS1	980	994	HLA-DRB1*09:01	81% - <100%	Dengue virus 3	ELISPOT	IFNg release	Positive	1	1	1	1	0	1	1	1	1	1	1	1	1	1	1	1	1	0	1	1
539825	QYKFQADSPKRLATA	NS1	804	818	HLA-DRB3*02:02	81% - <100%	Dengue virus 3	ELISPOT	IFNg release	Positive	1	1	1	1	0	1	1	1	1	1	1	1	1	1	1	1	1	1	1	1
167810	RENLLLGVGLAMATTLQLPE	NS2a	1224	1243	HLA-DP	81% - <100%	Dengue virus 3	ELISPOT	IFNg release	Positive	1	1	0	1	1	1	1	1	1	1	1	1	1	1	0	1	1	1	1	1
195632	LGFFLRKLTSRENLL	NS2a	1214	1228	HLA-DRB1*11:01	81% - <100%	Dengue virus 3	ELISPOT	IFNg release	Positive	1	1	0	1	1	1	1	1	1	1	1	1	1	1	1	1	1	1	1	1
195645	LISLTCSNTIFTLTV	NS2a	1274	1288	HLA-DRB1*07:01	81% - <100%	Dengue virus 3	ELISPOT	IFNg release	Positive	1	1	1	1	1	1	1	1	1	1	1	1	1	0	1	1	1	1	1	1
195836	PLFIFSLKDTLKRRS	NS2a	1330	1344	HLA-DRB1*11:01	81% - <100%	Dengue virus 3	ELISPOT	IFNg release	Positive	1	1	1	1	1	1	1	1	1	1	1	1	1	1	1	1	1	1	0	0
195912	RTATLILAGVSLLPV	NS2a	1291	1305	HLA-DRB1*03:01	81% - <100%	Dengue virus 3	ELISPOT	IFNg release	Positive	1	1	1	0	1	1	1	1	1	1	1	1	1	1	1	1	1	0	1	1
867442	KLITQFETYQLWTAL	NS2a	1260	1274	HLA-DPB1*02:01	81% - <100%	Dengue virus 3	ELISPOT	IFNg release	Positive	1	1	1	1	1	1	1	1	0	1	1	1	1	1	1	1	1	1	1	1
868046	MALKLITQFETYQLW	NS2a	1257	1271	HLA-DPB1*01:01	81% - <100%	Dengue virus 3	ELISPOT	IFNg release	Positive	1	0	1	1	1	1	1	1	1	1	1	1	1	1	1	1	1	1	0	1
868329	PLPLFIFSLKDTLKR	NS2a	1328	1342	HLA-DPB1*01:01	81% - <100%	Dengue virus 3	ELISPOT	IFNg release	Positive	1	1	1	1	1	1	1	1	1	1	1	1	1	1	1	1	1	1	0	0
869298	TQFETYQLWTALISL	NS2a	1263	1277	HLA-DPB1*04:02	81% - <100%	Dengue virus 3	ELISPOT	IFNg release	Positive	1	1	1	1	1	1	1	1	0	1	1	1	1	0	1	1	1	1	1	1
195180	ASSLLRNDVPMAGPL	NS2b	1361	1375	HLA-DRB1*03:01	81% - <100%	Dengue virus 3	ELISPOT	IFNg release	Positive	1	1	1	1	0	1	1	1	1	1	1	1	1	1	1	1	1	1	1	1
195789	NILTVLLKTALLIVS	NS2b	1436	1450	HLA-DRB1*01:01	81% - <100%	Dengue virus 3	ELISPOT	IFNg release	Positive	1	1	0	1	1	1	1	1	0	1	1	1	1	1	1	1	1	1	0	1
195994	SSLLRNDVPMAGPLV	NS2b	1362	1376	HLA-DRB1*03:01	81% - <100%	Dengue virus 3	ELISPOT	IFNg release	Positive	1	1	1	1	0	1	1	1	1	1	1	1	1	1	1	1	1	1	1	1
150544	RRLRTLILAPTRVVA	NS3	1688	1702	HLA-DRB1*01:01	81% - <100%	Dengue virus 3	ELISPOT	IFNg release	Positive	1	1	0	1	1	1	1	1	1	1	1	1	1	1	1	1	1	1	1	1
190732	EAAAIFMTATPPGTA	NS3	1782	1796	HLA-DRB1*04:03	81% - <100%	Dengue virus 3	ELISPOT	IFNg release	Positive	0	1	1	1	1	1	1	1	1	1	1	1	0	1	1	1	1	1	1	1
195898	RLRTLILAPTRVVAA	NS3	1689	1703	HLA-DRB1*04:03	81% - <100%	Dengue virus 3	ELISPOT	IFNg release	Positive	1	1	0	1	1	1	1	1	1	1	1	1	1	1	1	1	1	1	1	1
30395	KEGEKKKLRPRWLDA	NS3	585	599	HLA class II	81% - <100%	Dengue virus 3	ICS	IFNg release	Positive	1	0	1	1	1	1	1	1	1	0	1	1	1	1	1	1	1	1	1	1
39	AAAIFMTATPPGTAD	NS3	1783	1797	HLA-DRB1*04:03	81% - <100%	Dengue virus 3	ELISPOT	IFNg release	Positive	0	1	1	1	1	1	1	1	1	1	1	1	0	1	1	1	1	1	1	1
539028	AAIFMTATPPGTADA	NS3	1784	1798	HLA-DRB1*10:01	81% - <100%	Dengue virus 3	ELISPOT	IFNg release	Positive	0	1	1	1	1	1	1	1	1	1	1	1	0	1	1	1	1	1	1	1
539838	REAIKRRLRTLILAP	NS3	1683	1697	HLA-DRB1*14:04	81% - <100%	Dengue virus 3	ELISPOT	IFNg release	Positive	1	0	0	1	1	1	1	1	1	1	1	1	1	1	1	1	1	1	1	1
540039	VGLYGNGVVTKNGGY	NS3	9	23	HLA-DRB1*15:06	81% - <100%	Dengue virus 3	ELISPOT	IFNg release	Positive	1	1	0	1	1	1	1	1	1	1	1	1	1	1	1	1	1	1	1	1
55024	RNLTIMDLHPGSGKT	NS3	187	201	HLA class II	81% - <100%	Dengue virus 3	ICS	IFNg release	Positive	1	1	0	1	1	1	1	1	1	1	1	1	1	1	1	1	1	1	1	1
66127	TRVVAAEMEEA	NS3	225	235	HLA-DR15	81% - <100%	Dengue virus 3	51 chromium	cytotoxicity	Positive	1	1	1	0	1	1	1	1	1	1	1	1	1	1	1	1	1	1	1	1
867875	LNDWDFVVTTDISEM	NS3	1873	1887	HLA-DQA1*03:01/DQB1*03:02	81% - <100%	Dengue virus 3	ELISPOT	IFNg release	Positive	1	0	1	1	1	1	1	1	1	1	1	1	1	1	1	1	1	1	1	1
195522	IPLQWIASAIVLEFF	NS4a	2192	2206	HLA-DRB1*01:01	81% - <100%	Dengue virus 3	ELISPOT	IFNg release	Positive	1	1	1	1	1	1	1	1	1	1	1	1	1	0	1	1	1	1	1	1
195828	PETMETLLLLGLMIL	NS4a	2141	2155	HLA-DRB1*15:06	81% - <100%	Dengue virus 3	ELISPOT	IFNg release	Positive	1	1	1	1	1	1	1	0	1	1	1	1	1	1	1	1	1	1	1	1
196056	TLLLLGLMILLTGGA	NS4a	2146	2160	HLA-DRB1*15:06	81% - <100%	Dengue virus 3	ELISPOT	IFNg release	Positive	1	1	1	1	1	1	1	0	1	0	1	1	1	1	1	1	1	1	1	1
739464	LFLISGKGIGKTSIG	NS4a	2162	2176	HLA-DRB1*01:02	81% - <100%	Dengue virus 3	ELISPOT	IFNg release	Positive	1	1	1	0	0	1	1	1	1	1	1	1	1	1	1	1	1	1	1	1
167753	HVNAEPETPNMDVIGERIKR	NS5	263	282	HLA class II	81% - <100%	Dengue virus 3	ELISPOT	IFNg release	Positive	1	1	1	1	1	0	1	1	1	1	1	1	1	1	1	1	1	1	1	1
195382	GKVRKDIPQWQPSKG	NS5	3175	3189	HLA-DRB1*03:01	81% - <100%	Dengue virus 3	ELISPOT	IFNg release	Positive	1	1	1	1	1	1	1	1	1	1	1	1	1	0	0	1	1	1	1	1
195668	LLNRFTMTHRRPTIE	NS5	16	30	HLA-DRB1*11:01	81% - <100%	Dengue virus 3	ELISPOT	IFNg release	Positive	1	1	1	0	0	1	1	1	1	1	1	1	1	1	1	1	1	1	1	1
195779	NDMGKVRKDIPQWQP	NS5	3172	3186	HLA-DRB1*03:01	81% - <100%	Dengue virus 3	ELISPOT	IFNg release	Positive	1	1	1	1	1	1	1	1	1	1	1	1	1	1	0	1	1	1	1	1
195812	NRFTMTHRRPTIEKD	NS5	18	32	HLA-DRB1*11:01	81% - <100%	Dengue virus 3	ELISPOT	IFNg release	Positive	1	1	1	0	0	1	1	1	1	1	1	1	1	1	1	1	1	1	1	1
195875	RAIWYMWLGARYLEF	NS5	2961	2975	HLA-DRB1*01:01	81% - <100%	Dengue virus 3	ELISPOT	IFNg release	Positive	0	1	1	1	1	1	1	1	1	1	1	1	0	0	1	1	1	1	1	1
195939	SCVYNMMGKREKKLG	NS5	2938	2952	HLA-DRB1*11:01	81% - <100%	Dengue virus 3	ELISPOT	IFNg release	Positive	1	0	1	1	1	1	1	1	1	1	1	1	1	1	1	1	1	1	1	1
231420	RYLEFEALGFLNEDH	NS5	2971	2985	HLA-DPB1*04:01	81% - <100%	Dengue virus 3	ELISPOT	IFNg release	Positive	1	1	1	1	0	1	1	1	1	1	1	1	1	0	1	1	1	1	1	1
539458	KCGSCVYNMMGKREK	NS5	2935	2949	HLA-DRB1*13:01	81% - <100%	Dengue virus 3	ELISPOT	IFNg release	Positive	1	0	1	1	1	1	1	1	1	1	1	1	1	1	1	1	1	1	1	1
539831	RATWAQNIPTAIQQV	NS5	3346	3360	HLA-DRB1*09:01	81% - <100%	Dengue virus 3	ELISPOT	IFNg release	Positive	1	1	1	1	1	1	1	1	1	1	1	1	1	0	1	1	1	1	1	1
738753	EEFLDYMPSMKRFRK	NS5	3367	3381	HLA-DRB1*14:02	81% - <100%	Dengue virus 3	ELISPOT	IFNg release	Positive	1	1	1	1	0	1	1	1	1	1	1	1	1	1	1	1	1	1	1	1
738759	EFLDYMPSMKRFRKE	NS5	3368	3382	HLA-DRB1*14:06	81% - <100%	Dengue virus 3	ELISPOT	IFNg release	Positive	1	1	1	1	0	1	1	1	1	1	1	1	1	1	1	1	1	1	1	1
857703	IWYMWLGARYLEFEA	NS5	2963	2977	HLA-DPB1*04:01	81% - <100%	Dengue virus 3	ELISPOT	IFNg release	Positive	0	1	1	1	1	1	1	1	1	1	1	1	0	0	1	1	1	1	1	1
867015	GSCVYNMMGKREKKL	NS5	2937	2951	HLA-DRB5*01:01	81% - <100%	Dengue virus 3	ELISPOT	IFNg release	Positive	1	0	1	1	1	1	1	1	1	1	1	1	1	1	1	1	1	1	1	1
868338	PMSTYGWNIVKLMSG	NS5	2605	2619	HLA-DQA1*01:01/DQB1*05:01	81% - <100%	Dengue virus 3	ELISPOT	IFNg release	Positive	1	1	1	1	1	1	1	1	1	1	1	1	1	1	1	1	0	1	1	1
869705	WYMWLGARYLEFEAL	NS5	2964	2978	HLA-DQA1*05:01/DQB1*02:01	81% - <100%	Dengue virus 3	ELISPOT	IFNg release	Positive	0	1	1	1	1	1	1	1	1	1	1	1	0	0	1	1	1	1	1	1
195497	IKVLKGFKKEISNML	C	31	45	HLA-DRB1*04:01	51%-80%	Dengue virus 3	ELISPOT	IFNg release	Positive	1	0	0	1	0	1	1	1	1	1	1	1	1	1	0	1	1	1	1	1
195597	KTSLCLMMILPAALA	C	53	67	HLA-DRB1*01:01	51%-80%	Dengue virus 3	ELISPOT	IFNg release	Positive	1	1	1	1	1	1	0	1	0	1	1	1	1	1	1	1	1	0	0	0
539277	GAIKVLKGFKKEISN	C	29	43	HLA-DRB1*12:02	51%-80%	Dengue virus 3	ELISPOT	IFNg release	Positive	1	0	0	1	0	1	1	1	1	1	1	1	1	1	0	1	1	1	1	1
539512	KSGAIKVLKGFKKEI	C	27	41	HLA-DRB1*12:02	51%-80%	Dengue virus 3	ELISPOT	IFNg release	Positive	1	0	0	1	0	1	1	1	1	1	1	1	1	1	0	1	1	1	1	1
539897	SGAIKVLKGFKKEIS	C	28	42	HLA-DRB1*14:04	51%-80%	Dengue virus 3	ELISPOT	IFNg release	Positive	1	0	0	1	0	1	1	1	1	1	1	1	1	1	0	1	1	1	1	1
190825	KQIANELNYILWENN	NS1	69	83	HLA-DR2	51%-80%	Dengue virus 3	ELISPOT	IFNg release	Positive	1	1	1	1	1	0	1	1	1	1	0	0	1	1	1	1	1	1	0	0
195123	AGVFFTFVLLLSGQI	NS2a	1159	1173	HLA-DRB1*07:01	51%-80%	Dengue virus 3	ELISPOT	IFNg release	Positive	1	1	1	1	0	0	1	1	1	1	0	0	1	0	1	1	1	1	0	0
195335	FTFVLLLSGQITWRD	NS2a	1163	1177	HLA-DRB1*15:02	51%-80%	Dengue virus 3	ELISPOT	IFNg release	Positive	1	1	1	1	1	0	1	1	1	1	0	0	1	1	1	1	1	1	0	0
195429	GVFFTFVLLLSGQIT	NS2a	1160	1174	HLA-DRB1*04:01	51%-80%	Dengue virus 3	ELISPOT	IFNg release	Positive	1	1	1	1	0	0	1	1	1	1	0	0	1	0	1	1	1	1	0	0
195457	HMIAGVFFTFVLLLS	NS2a	1156	1170	HLA-DRB1*11:01	51%-80%	Dengue virus 3	ELISPOT	IFNg release	Positive	1	1	1	1	0	0	1	1	1	1	0	0	1	0	1	1	1	1	0	0
867116	IAGVFFTFVLLLSGQ	NS2a	1158	1172	HLA-DPB1*01:01	51%-80%	Dengue virus 3	ELISPOT	IFNg release	Positive	1	1	1	1	0	0	1	1	1	1	0	0	1	0	1	1	1	1	0	0
167823	TMRIKDDETENILTVLLKTA	NS2b	83	102	HLA-DRB1*15:01	51%-80%	Dengue virus 3	ELISPOT	IFNg release	Positive	1	1	0	1	1	1	1	1	0	0	1	1	1	1	1	1	1	1	0	1
195144	AIVREAIKRRLRTLI	NS3	1680	1694	HLA-DRB1*08:02	51%-80%	Dengue virus 3	ELISPOT	IFNg release	Positive	1	0	0	0	1	1	1	1	1	1	1	0	1	1	1	1	1	0	1	1
38401	LPAIVREAIKRRLRT	NS3	1678	1692	HLA-DRB3*02:02	51%-80%	Dengue virus 3	ELISPOT	IFNg release	Positive	1	0	0	0	1	1	1	1	1	1	1	0	1	1	1	1	1	0	1	1
539714	NGKKVIQLSRKTFDT	NS3	1852	1866	HLA-DRB1*12:02	51%-80%	Dengue virus 3	ELISPOT	IFNg release	Positive	1	0	0	1	1	1	1	1	1	1	1	0	1	1	0	1	0	1	0	0
54455	RKYLPAIVRE	NS3	202	211	HLA-DR15	51%-80%	Dengue virus 3	51 chromium	cytotoxicity	Positive	1	1	0	0	1	1	1	1	1	1	1	0	1	1	1	1	1	0	1	1
70708	VREAIKRRLRTLILA	NS3	1682	1696	HLA-DRB1*12:02	51%-80%	Dengue virus 3	ELISPOT	IFNg release	Positive	1	0	0	0	1	1	1	1	1	1	1	0	1	1	1	1	1	0	1	1
867358	IVREAIKRRLRTLIL	NS3	1681	1695	HLA-DRB3*02:02	51%-80%	Dengue virus 3	ELISPOT	IFNg release	Positive	1	0	0	0	1	1	1	1	1	1	1	0	1	1	1	1	1	0	1	1
867534	KYLPAIVREAIKRRL	NS3	1676	1690	HLA-DRB4*01:01	51%-80%	Dengue virus 3	ELISPOT	IFNg release	Positive	1	0	0	0	1	1	1	1	1	1	1	0	1	1	1	1	1	0	1	1
195807	NPLTLTAAVLLLITH	NS4b	2345	2359	HLA-DRB1*07:01	51%-80%	Dengue virus 3	ELISPOT	IFNg release	Positive	1	1	1	1	1	0	0	0	1	0	0	0	1	1	1	0	1	1	0	0
196163	VNPLTLTAAVLLLIT	NS4b	2344	2358	HLA-DRB1*01:01	51%-80%	Dengue virus 3	ELISPOT	IFNg release	Positive	1	1	1	1	1	0	0	0	1	0	0	0	1	1	1	0	1	1	0	0
195410	GNIVSSVNMVSRLLL	NS5	2715	2729	HLA-DRB1*03:01	51%-80%	Dengue virus 3	ELISPOT	IFNg release	Positive	1	1	1	0	1	0	1	1	1	0	0	0	1	1	1	1	1	1	0	0
195806	NMVSRLLLNRFTMTH	NS5	2722	2736	HLA-DRB1*08:02	51%-80%	Dengue virus 3	ELISPOT	IFNg release	Positive	1	1	1	0	1	0	1	1	1	1	0	0	1	1	1	1	1	1	0	0
196041	TGNIVSSVNMVSRLL	NS5	2714	2728	HLA-DRB1*04:01	51%-80%	Dengue virus 3	ELISPOT	IFNg release	Positive	1	1	1	0	1	0	1	1	1	0	0	0	1	1	1	1	1	1	0	0
196162	VNMVSRLLLNRFTMT	NS5	9	23	HLA-DRB1*11:01	51%-80%	Dengue virus 3	ELISPOT	IFNg release	Positive	1	1	1	0	1	0	1	1	1	1	0	0	1	1	1	1	1	1	0	0
539449	IVSSVNMVSRLLLNR	NS5	2717	2731	HLA-DRB1*11:04	51%-80%	Dengue virus 3	ELISPOT	IFNg release	Positive	1	1	1	0	1	0	1	1	1	0	0	0	1	1	1	1	1	1	0	0
539945	SVNMVSRLLLNRFTM	NS5	2720	2734	HLA-DRB1*12:02	51%-80%	Dengue virus 3	ELISPOT	IFNg release	Positive	1	1	1	0	1	0	1	1	1	1	0	0	1	1	1	1	1	1	0	0
540090	VSSVNMVSRLLLNRF	NS5	2718	2732	HLA-DRB1*14:04	51%-80%	Dengue virus 3	ELISPOT	IFNg release	Positive	1	1	1	0	1	0	1	1	1	0	0	0	1	1	1	1	1	1	0	0
866168	ARYLEFEALGFLNED	NS5	2970	2984	HLA-DQA1*03:01/DQB1*03:02	51%-80%	Dengue virus 3	ELISPOT	IFNg release	Positive	0	1	1	1	0	1	1	1	1	1	1	1	0	0	1	1	1	1	1	1
868748	SAKLQWFVERNMVIP	NS5	2549	2563	HLA-DQA1*01:01/DQB1*05:01	51%-80%	Dengue virus 3	ELISPOT	IFNg release	Positive	0	1	1	1	1	1	0	1	1	1	1	1	0	0	1	1	1	1	1	1
591723	NMLSIINRRKKTSLC	C	90	104	HLA-DRB1*11:01	>0% - 50%	Dengue virus 3	ELISPOT	IFNg release	Positive	0	0	0	0	0	0	0	0	0	0	0	0	0	0	1	0	0	0	0	0
868645	RGLLNGQGPMKLVMA	C	15	29	HLA-DRB3*02:02	>0% - 50%	Dengue virus 3	ELISPOT	IFNg release	Positive	0	0	0	0	0	0	0	0	0	0	0	0	0	1	0	0	0	0	0	0
190823	KLTVVVGDIIGVLEQ	NS1	85	99	HLA-DR2	>0% - 50%	Dengue virus 3	ELISPOT	IFNg release	Positive	0	0	0	0	0	0	0	0	1	0	0	0	0	0	0	0	0	0	0	0
195125	AGVIFTFVLLLSGQI	NS2a	1159	1173	HLA-DRB1*07:01	>0% - 50%	Dengue virus 3	ELISPOT	IFNg release	Positive	0	0	0	0	1	0	0	0	0	0	0	0	0	0	0	0	0	0	0	0
195740	LWTALVSLTCSNTIF	NS2a	1270	1284	HLA-DRB1*07:01	>0% - 50%	Dengue virus 3	ELISPOT	IFNg release	Positive	0	0	0	0	0	0	0	0	0	0	0	0	0	1	0	0	0	0	0	0
539802	QFETYQLWTALVSLT	NS2a	1264	1278	HLA-DRB1*15:02	>0% - 50%	Dengue virus 3	ELISPOT	IFNg release	Positive	0	0	0	0	0	0	0	0	0	0	0	0	0	1	0	0	0	0	0	0
868109	MIAGVIFTFVLLLSG	NS2a	1157	1171	HLA-DPB1*04:02	>0% - 50%	Dengue virus 3	ELISPOT	IFNg release	Positive	0	0	0	0	1	0	0	0	0	0	0	0	0	0	0	0	0	0	0	0
539432	IREAIKRRLRTLILA	NS3	1682	1696	HLA-DRB1*08:03	>0% - 50%	Dengue virus 3	ELISPOT	IFNg release	Positive	0	0	0	1	0	0	0	0	0	0	0	1	0	0	0	0	0	1	0	0
196168	VPLQWIASAIVLEFF	NS4a	2192	2206	HLA-DRB1*01:01	>0% - 50%	Dengue virus 3	ELISPOT	IFNg release	Positive	0	0	0	0	0	0	0	0	0	0	0	0	0	1	0	0	0	0	0	0
869474	VIGILTLAAIIAANE	NS4a	2230	2244	HLA-DQA1*01:02/DQB1*06:02	>0% - 50%	Dengue virus 3	ELISPOT	IFNg release	Positive	1	1	1	1	0	0	0	0	1	0	0	0	1	0	1	0	1	1	0	0
195663	LLLITHYAIIGPGLQ	NS4b	2354	2368	HLA-DRB1*07:01	>0% - 50%	Dengue virus 3	ELISPOT	IFNg release	Positive	1	1	1	1	1	0	0	0	1	0	0	0	1	1	0	0	1	1	0	0
196023	TAAVLLLITHYAIIG	NS4b	2350	2364	HLA-DRB1*15:01	>0% - 50%	Dengue virus 3	ELISPOT	IFNg release	Positive	1	1	1	1	1	0	0	0	1	0	0	0	1	1	0	0	1	1	0	0

869526	VLLLVTHYAIIGPGL	NS4b	2353	2367	HLA-DPB1*02:01	>0% - 50%	Dengue virus 3	ELISPOT	IFNg release	Positive	0	0	0	0	0	1	1	1	0	1	1	1	0	0	0	1	0	0	1	1
196239	YQNKVVKVQRPTPKG	NS5	3062	3076	HLA-DRB1*08:02	>0% - 50%	Dengue virus 3	ELISPOT	IFNg release	Positive	0	0	0	0	0	0	0	0	0	0	0	0	0	1	0	0	0	0	0	0

CD4 DENV-4 Epitopes

											NCBI IDs of DENV-4 circulating strains from India, (100% identity=1,green ; else 0, red)								
Epitope_ID	AA_Seq	Protein_Name	Start	End	Allele	step	Serotype	Method/Technique	Assay Group	Qualitative Measure	AVW85694.1	AVW85695.1	AVW85696.1	AFZ40130.1	ANC57613.1	QBA57536.1	QCE20687.1	ARB18129.1	QDZ58859
195483	IGFRKEIGRMLNILN	C	81	95	HLA-DRB1*04:01	0%	Dengue virus 4	ELISPOT	IFNg release	Positive	0	0	0	0	0	0	0	0	0
195488	IGRMLNILNRRKRST	C	87	101	HLA-DRB1*14:02	0%	Dengue virus 4	ELISPOT	IFNg release	Positive	0	0	0	0	0	0	0	0	0
195580	KNKAIKILIGFRKEI	C	53	67	HLA-DRB1*15:01	0%	Dengue virus 4	ELISPOT	IFNg release	Positive	0	0	0	0	0	0	0	0	0
195768	MTLLCLIPTAMAFHL	C	102	116	HLA-DRB1*01:01	0%	Dengue virus 4	ELISPOT	IFNg release	Positive	0	0	0	0	0	0	0	0	0
195922	RWGQLKKTKAIKILT	C	67	81	HLA-DRB1*07:01	0%	Dengue virus 4	ELISPOT	IFNg release	Positive	0	0	0	0	0	0	0	0	0
196065	TPQGLVKRFSSGLFS	C	24	38	HLA-DRB1*15:01	0%	Dengue virus 4	ELISPOT	IFNg release	Positive	0	0	0	0	0	0	0	0	0
36533	LIGFRKEIGRML	C	80	91	HLA-DPw4	0%	Dengue virus 4	ICS	IFNg release	Positive	0	0	0	0	0	0	0	0	0
539571	LIGFRKEIGRMLNIL	C	80	94	HLA-DRB1*09:01	0%	Dengue virus 4	ELISPOT	IFNg release	Positive	0	0	0	0	0	0	0	0	0
539581	LKKNKAIKILIGFRK	C	51	65	HLA-DRB1*14:04	0%	Dengue virus 4	ELISPOT	IFNg release	Positive	0	0	0	0	0	0	0	0	0
540116	WGQLKKNKAIKILIG	C	68	82	HLA-DRB1*09:01	0%	Dengue virus 4	ELISPOT	IFNg release	Positive	0	0	0	0	0	0	0	0	0
739060	GRMLNILNGRKRSTM	C	88	102	HLA-DRB1*14:02	0%	Dengue virus 4	ELISPOT	IFNg release	Positive	0	0	0	0	0	0	0	0	0
739178	IGRMLNILNRRRRST	C	87	101	HLA-DRB1*14:02	0%	Dengue virus 4	ELISPOT	IFNg release	Positive	0	0	0	0	0	0	0	0	0
868732	RWGQLKKNKAIKILI	C	39	53	HLA-DRB3*02:02	0%	Dengue virus 4	ELISPOT	IFNg release	Positive	0	0	0	0	0	0	0	0	0
195916	RTVFFVLMMLVAPSY	PreM	264	278	HLA-DRB1*01:01	0%	Dengue virus 4	ELISPOT	IFNg release	Positive	0	0	0	0	0	0	0	0	0
869670	WCNLTSTWVMYGTCT	PreM	180	194	HLA-DQA1*01:02/DQB1*06:02	0%	Dengue virus 4	ELISPOT	IFNg release	Positive	0	0	0	0	0	0	0	0	0
167808	RDVNKEKVVGRVISSTPLAE	E	340	359	HLA class II	0%	Dengue virus 4	ELISPOT	IFNg release	Positive	0	0	0	0	0	0	0	0	0
195227	DSYIVIGVGNSALTL	E	654	668	HLA-DRB1*15:06	0%	Dengue virus 4	ELISPOT	IFNg release	Positive	0	0	0	0	0	0	0	0	0
195364	GGVSWMIRILIGILV	E	449	463	HLA-DRB1*15:06	0%	Dengue virus 4	ELISPOT	IFNg release	Positive	0	0	0	0	0	0	0	0	0
195434	GVSWMIRILIGFLVL	E	729	743	HLA-DRB1*08:02	0%	Dengue virus 4	ELISPOT	IFNg release	Positive	0	0	0	0	0	0	0	0	0
195435	GVSWMIRILIGLLVL	E	729	743	HLA-DRB1*15:06	0%	Dengue virus 4	ELISPOT	IFNg release	Positive	0	0	0	0	0	0	0	0	0
195625	LESTYRGAKRMAILG	E	681	695	HLA-DRB1*14:02	0%	Dengue virus 4	ELISPOT	IFNg release	Positive	0	0	0	0	0	0	0	0	0
196036	TGIQRTVFFVLMMLV	E	44	58	HLA-DRB1*07:01	0%	Dengue virus 4	ELISPOT	IFNg release	Positive	0	0	0	0	0	0	0	0	0
196189	VSWIIRILIGLLVLW	E	451	465	HLA-DRB1*15:06	0%	Dengue virus 4	ELISPOT	IFNg release	Positive	0	0	0	0	0	0	0	0	0
539230	EMILMKMKKKTWLVH	E	474	488	HLA-DRB1*12:02	0%	Dengue virus 4	ELISPOT	IFNg release	Positive	0	0	0	0	0	0	0	0	0
195791	NIMWKQITNELNYVL	NS1	839	853	HLA-DRB1*04:07	0%	Dengue virus 4	ELISPOT	IFNg release	Positive	0	0	0	0	0	0	0	0	0
539829	QYQFQPESPARLASA	NS1	805	819	HLA-DRB1*09:01	0%	Dengue virus 4	ELISPOT	IFNg release	Positive	0	0	0	0	0	0	0	0	0
167725	AMTTTLSIPHDLMELIDGIS	NS2a	50	69	HLA class II	0%	Dengue virus 4	ELISPOT	IFNg release	Positive	0	0	0	0	0	0	0	0	0
195156	ALSLTFIRSTMPLVM	NS2a	1274	1288	HLA-DRB1*07:01	0%	Dengue virus 4	ELISPOT	IFNg release	Positive	0	0	0	0	0	0	0	0	0
195157	ALSLTFIRSTMSLVM	NS2a	1274	1288	HLA-DRB1*04:01	0%	Dengue virus 4	ELISPOT	IFNg release	Positive	0	0	0	0	0	0	0	0	0
195158	ALSLTFIRSTTPLVM	NS2a	1274	1288	HLA-DRB1*07:01	0%	Dengue virus 4	ELISPOT	IFNg release	Positive	0	0	0	0	0	0	0	0	0
195190	AVFKMSPGYVLGVFL	NS2a	1204	1218	HLA-DRB1*01:01	0%	Dengue virus 4	ELISPOT	IFNg release	Positive	0	0	0	0	0	0	0	0	0
195221	DLMELISGISLGLIL	NS2a	1243	1257	HLA-DRB1*07:01	0%	Dengue virus 4	ELISPOT	IFNg release	Positive	0	0	0	0	0	0	0	0	0
195746	MAVFKMSPGYVLGVF	NS2a	1203	1217	HLA-DRB1*07:01	0%	Dengue virus 4	ELISPOT	IFNg release	Positive	0	0	0	0	0	0	0	0	0
196061	TMSLVMAWRTIMAVL	NS2a	1283	1297	HLA-DRB1*03:01	0%	Dengue virus 4	ELISPOT	IFNg release	Positive	0	0	0	0	0	0	0	0	0
196070	TRKHMILAVVITLCA	NS2a	1154	1168	HLA-DRB1*08:02	0%	Dengue virus 4	ELISPOT	IFNg release	Positive	0	0	0	0	0	0	0	0	0
196253	YVLGVFLRKLTSRET	NS2a	1212	1226	HLA-DRB1*11:01	0%	Dengue virus 4	ELISPOT	IFNg release	Positive	0	0	0	0	0	0	0	0	0
539130	CLRRRVTRKHMILAV	NS2a	1148	1162	HLA-DRB1*08:03	0%	Dengue virus 4	ELISPOT	IFNg release	Positive	0	0	0	0	0	0	0	0	0
539198	ECLRRRVTRKHMILA	NS2a	1147	1161	HLA-DRB1*13:01	0%	Dengue virus 4	ELISPOT	IFNg release	Positive	0	0	0	0	0	0	0	0	0
539360	GVFLRKLTSRETALM	NS2a	1215	1229	HLA-DRB1*10:01	0%	Dengue virus 4	ELISPOT	IFNg release	Positive	0	0	0	0	0	0	0	0	0
539406	IGGQIHLAIMAVFKM	NS2a	1194	1208	HLA-DRB1*13:01	0%	Dengue virus 4	ELISPOT	IFNg release	Positive	0	0	0	0	0	0	0	0	0
539407	IGGQTHLAIMAVFKM	NS2a	1194	1208	HLA-DRB1*13:01	0%	Dengue virus 4	ELISPOT	IFNg release	Positive	0	0	0	0	0	0	0	0	0
539433	IRSTMPLVMAWRTIM	NS2a	1280	1294	HLA-DRB1*13:01	0%	Dengue virus 4	ELISPOT	IFNg release	Positive	0	0	0	0	0	0	0	0	0
539434	IRSTMSLVMAWRTIM	NS2a	1280	1294	HLA-DRB1*13:01	0%	Dengue virus 4	ELISPOT	IFNg release	Positive	0	0	0	0	0	0	0	0	0
539565	LGVFLRKLTSRETAL	NS2a	1214	1228	HLA-DRB1*08:03	0%	Dengue virus 4	ELISPOT	IFNg release	Positive	0	0	0	0	0	0	0	0	0
539627	LSLTFIRSTMPLVMA	NS2a	1275	1289	HLA-DRB1*10:01	0%	Dengue virus 4	ELISPOT	IFNg release	Positive	0	0	0	0	0	0	0	0	0
539628	LSLTFIRSTTPLVMA	NS2a	1275	1289	HLA-DRB1*08:03	0%	Dengue virus 4	ELISPOT	IFNg release	Positive	0	0	0	0	0	0	0	0	0
539919	SLTFIRSTMPLVMAW	NS2a	1276	1290	HLA-DRB1*09:01	0%	Dengue virus 4	ELISPOT	IFNg release	Positive	0	0	0	0	0	0	0	0	0
539921	SLTFIRSTTPLIMAW	NS2a	1276	1290	HLA-DRB1*04:03	0%	Dengue virus 4	ELISPOT	IFNg release	Positive	0	0	0	0	0	0	0	0	0
539961	TALMVIGMAMTTTLS	NS2a	1226	1240	HLA-DRB1*01:02	0%	Dengue virus 4	ELISPOT	IFNg release	Positive	0	0	0	0	0	0	0	0	0
540033	VFKMSPGYVLGVFLR	NS2a	1205	1219	HLA-DRB1*09:01	0%	Dengue virus 4	ELISPOT	IFNg release	Positive	0	0	0	0	0	0	0	0	0
540053	VLGVFLRKLTSRETA	NS2a	1213	1227	HLA-DRB1*14:04	0%	Dengue virus 4	ELISPOT	IFNg release	Positive	0	0	0	0	0	0	0	0	0
738559	AVVITLCAIILGGLT	NS2a	1161	1175	HLA-DRB1*01:02	0%	Dengue virus 4	ELISPOT	IFNg release	Positive	0	0	0	0	0	0	0	0	0
867969	LTFIRSTTPLVMAWR	NS2a	1277	1291	HLA-DRB3*02:02	0%	Dengue virus 4	ELISPOT	IFNg release	Positive	0	0	0	0	0	0	0	0	0
45689	NRKGKIVGLYGNGVV	NS3	141	155	HLA-DRB1*15:01	0%	Dengue virus 4	ICS	IFNg release	Positive	0	0	0	0	0	0	0	0	0
53505	REGEKKKLRPRWLDR	NS3	585	599	HLA class II	0%	Dengue virus 4	ICS	IFNg release	Positive	0	0	0	0	0	0	0	0	0
539020	AAAIFMTATPPGATD	NS3	1783	1797	HLA-DRB1*10:01	0%	Dengue virus 4	ELISPOT	IFNg release	Positive	0	0	0	0	0	0	0	0	0
539021	AAAIFMTATPPGSID	NS3	1783	1797	HLA-DRB1*04:07	0%	Dengue virus 4	ELISPOT	IFNg release	Positive	0	0	0	0	0	0	0	0	0
539023	AAAIFMTATPPGTID	NS3	1783	1797	HLA-DRB1*04:03	0%	Dengue virus 4	ELISPOT	IFNg release	Positive	0	0	0	0	0	0	0	0	0
539026	AAIFMTATPPGATDP	NS3	1784	1798	HLA-DRB1*09:01	0%	Dengue virus 4	ELISPOT	IFNg release	Positive	0	0	0	0	0	0	0	0	0
540077	VREAIKRRLRTLVLA	NS3	1682	1696	HLA-DRB1*08:03	0%	Dengue virus 4	ELISPOT	IFNg release	Positive	0	0	0	0	0	0	0	0	0
868371	PRWLDARVYADPMAL	NS3	2067	2081	HLA-DQA1*03:01/DQB1*03:02	0%	Dengue virus 4	ELISPOT	IFNg release	Positive	0	0	0	0	0	0	0	0	0
195386	GLITIAVASGLLWVA	NS4a	2176	2190	HLA-DRB1*07:01	0%	Dengue virus 4	ELISPOT	IFNg release	Positive	0	0	0	0	0	0	0	0	0

195551	IYVILTILTIIALVA	NS4a	2227	2241	HLA-DRB1*01:01	0%	Dengue virus 4	ELISPOT	IFNg release	Positive	0	0	0	0	0	0	0	0	0
195648	LIYVILTILTIIALV	NS4a	2226	2240	HLA-DRB1*07:01	0%	Dengue virus 4	ELISPOT	IFNg release	Positive	0	0	0	0	0	0	0	0	0
195853	QLIYVILTILAIIGL	NS4a	2225	2239	HLA-DRB1*15:02	0%	Dengue virus 4	ELISPOT	IFNg release	Positive	0	0	0	0	0	0	0	0	0
196250	YVILAILTIIGLVAA	NS4a	2228	2242	HLA-DRB1*08:02	0%	Dengue virus 4	ELISPOT	IFNg release	Positive	0	0	0	0	0	0	0	0	0
196251	YVILALLTIISLIAA	NS4a	2228	2242	HLA-DRB1*15:02	0%	Dengue virus 4	ELISPOT	IFNg release	Positive	0	0	0	0	0	0	0	0	0
539666	MGLITIAVASGLLWV	NS4a	2175	2189	HLA-DRB1*09:01	0%	Dengue virus 4	ELISPOT	IFNg release	Positive	0	0	0	0	0	0	0	0	0
867726	LIYVILTILTIIGLI	NS4a	2226	2240	HLA-DPB1*04:02	0%	Dengue virus 4	ELISPOT	IFNg release	Positive	0	0	0	0	0	0	0	0	0
195719	LTASLAMLLVHYAII	NS4b	2346	2360	HLA-DRB1*07:01	0%	Dengue virus 4	ELISPOT	IFNg release	Positive	0	0	0	0	0	0	0	0	0
196055	TLIASLVMLLVHYAI	NS4b	2345	2359	HLA-DRB1*01:01	0%	Dengue virus 4	ELISPOT	IFNg release	Positive	0	0	0	0	0	0	0	0	0
196082	TTLIASLVMLLVHYA	NS4b	2344	2358	HLA-DRB1*13:01	0%	Dengue virus 4	ELISPOT	IFNg release	Positive	0	0	0	0	0	0	0	0	0
196209	WAFCEVLTLATGPVL	NS4b	2427	2441	HLA-DRB1*07:01	0%	Dengue virus 4	ELISPOT	IFNg release	Positive	0	0	0	0	0	0	0	0	0
539661	MGCYSQVNPTTLIAS	NS4b	2335	2349	HLA-DRB1*09:01	0%	Dengue virus 4	ELISPOT	IFNg release	Positive	0	0	0	0	0	0	0	0	0
738500	AMGCYSQVNPTTLIA	NS4b	2334	2348	HLA-DRB1*04:07	0%	Dengue virus 4	ELISPOT	IFNg release	Positive	0	0	0	0	0	0	0	0	0
195230	DYMPVMKRYSAPSES	NS5	3369	3383	HLA-DRB1*11:01	0%	Dengue virus 4	ELISPOT	IFNg release	Positive	0	0	0	0	0	0	0	0	0
195593	KTFMKDGRSLVVPCR	NS5	3202	3216	HLA-DRB1*03:01	0%	Dengue virus 4	ELISPOT	IFNg release	Positive	0	0	0	0	0	0	0	0	0
195764	MPVMKRYSAPSESEG	NS5	3371	3385	HLA-DRB1*08:02	0%	Dengue virus 4	ELISPOT	IFNg release	Positive	0	0	0	0	0	0	0	0	0
195835	PKPGTRMVMTTTANW	NS5	2853	2867	HLA-DRB1*04:01	0%	Dengue virus 4	ELISPOT	IFNg release	Positive	0	0	0	0	0	0	0	0	0
195919	RVWIEDNPNMIDKTP	NS5	3303	3317	HLA-DRB1*03:01	0%	Dengue virus 4	ELISPOT	IFNg release	Positive	0	0	0	0	0	0	0	0	0
196071	TRMVMTTTANWLWAL	NS5	2857	2871	HLA-DRB1*04:01	0%	Dengue virus 4	ELISPOT	IFNg release	Positive	0	0	0	0	0	0	0	0	0
196092	TYQNKVVKVLRPTPR	NS5	3059	3073	HLA-DRB1*08:02	0%	Dengue virus 4	ELISPOT	IFNg release	Positive	0	0	0	0	0	0	0	0	0
196106	VDYMPVMKRYSAPSE	NS5	3368	3382	HLA-DRB1*11:01	0%	Dengue virus 4	ELISPOT	IFNg release	Positive	0	0	0	0	0	0	0	0	0
539222	EKPDMTIIGRRLQRL	NS5	2757	2771	HLA-DRB1*12:02	0%	Dengue virus 4	ELISPOT	IFNg release	Positive	0	0	0	0	0	0	0	0	0
539354	GTRMVMTTTANWLWA	NS5	2856	2870	HLA-DRB1*04:03	0%	Dengue virus 4	ELISPOT	IFNg release	Positive	0	0	0	0	0	0	0	0	0
539493	KMLLNRFTTRHRKPT	NS5	2724	2738	HLA-DRB1*13:01	0%	Dengue virus 4	ELISPOT	IFNg release	Positive	0	0	0	0	0	0	0	0	0
539498	KPDMTIIGRRLQRLQ	NS5	2758	2772	HLA-DRB1*08:03	0%	Dengue virus 4	ELISPOT	IFNg release	Positive	0	0	0	0	0	0	0	0	0
539769	PGTRMVMTTTANWLW	NS5	2855	2869	HLA-DRB1*04:03	0%	Dengue virus 4	ELISPOT	IFNg release	Positive	0	0	0	0	0	0	0	0	0
540007	TTSKMLLNRFTTRHR	NS5	2721	2735	HLA-DRB1*13:01	0%	Dengue virus 4	ELISPOT	IFNg release	Positive	0	0	0	0	0	0	0	0	0
867473	KPLDERFGTSLLFLN	NS5	3156	3170	HLA-DPB1*01:01	0%	Dengue virus 4	ELISPOT	IFNg release	Positive	0	0	0	0	0	0	0	0	0
867575	LDERFGTSLLFLNDM	NS5	3158	3172	HLA-DPB1*04:01	0%	Dengue virus 4	ELISPOT	IFNg release	Positive	0	0	0	0	0	0	0	0	0
1067635	LKRWGQLKKNKAIKI	C	65	79	HLA-DRB1*07:01	100%	Dengue virus 4	ELISPOT	IFNg release	Positive-Low	1	1	1	1	1	1	1	1	1
1068575	VVRPPFNMLKRERNR	C	7	21	HLA-DRB1*11:01	100%	Dengue virus 4	ELISPOT	IFNg release	Positive-Low	1	1	1	1	1	1	1	1	1
17605	FRKEIGRML	C	83	91	HLA-DPw4	100%	Dengue virus 4	51 chromium	cytotoxicity	Positive	1	1	1	1	1	1	1	1	1
195316	FNMLKRERNRVSTPQ	C	12	26	HLA-DRB1*04:01	100%	Dengue virus 4	ELISPOT	IFNg release	Positive	1	1	1	1	1	1	1	1	1
195325	FRKEIGRMLNILNGR	C	19	33	HLA-DRB1*04:01	100%	Dengue virus 4	ELISPOT	IFNg release	Positive	1	1	1	1	1	1	1	1	1
195587	KRWGQLKKNKAIKIL	C	46	60	HLA-DRB1*08:03	100%	Dengue virus 4	ELISPOT	IFNg release	Positive	1	1	1	1	1	1	1	1	1
195651	LKKNKAIKILTGFRK	C	51	65	HLA-DRB1*14:04	100%	Dengue virus 4	ELISPOT	IFNg release	Positive	1	1	1	1	1	1	1	1	1
195900	RMVLAFITFLRVLSI	C	44	58	HLA-DRB1*11:01	100%	Dengue virus 4	ELISPOT	IFNg release	Positive	1	1	1	1	1	1	1	1	1
196134	VLAFITFLRVLSIPP	C	26	40	HLA-DRB1*04:01	100%	Dengue virus 4	ELISPOT	IFNg release	Positive	1	1	1	1	1	1	1	1	1
539044	AFITFLRVLSIPPTA	C	28	42	HLA-DRB1*04:03	100%	Dengue virus 4	ELISPOT	IFNg release	Positive	1	1	1	1	1	1	1	1	1
539255	FITFLRVLSIPPTAG	C	49	63	HLA-DRB1*04:07	100%	Dengue virus 4	ELISPOT	IFNg release	Positive	1	1	1	1	1	1	1	1	1
539294	GFRKEIGRMLNILNG	C	54	68	HLA-DRB3*02:02	100%	Dengue virus 4	ELISPOT	IFNg release	Positive	1	1	1	1	1	1	1	1	1
539496	KNKAIKILTGFRKEI	C	53	67	HLA-DRB1*12:02	100%	Dengue virus 4	ELISPOT	IFNg release	Positive	1	1	1	1	1	1	1	1	1
539536	LAFITFLRVLSIPPT	C	27	41	HLA-DRB1*12:02	100%	Dengue virus 4	ELISPOT	IFNg release	Positive	1	1	1	1	1	1	1	1	1
539632	LTGFRKEIGRMLNIL	C	52	66	HLA-DRB3*02:02	100%	Dengue virus 4	ELISPOT	IFNg release	Positive	1	1	1	1	1	1	1	1	1
69367	VLAFITFLR	C	46	54	HLA-DPw4	100%	Dengue virus 4	51 chromium	cytotoxicity	Positive	1	1	1	1	1	1	1	1	1
739175	IGRMLNILNGRKRST	C	87	101	HLA-DRB1*14:02	100%	Dengue virus 4	ELISPOT	IFNg release	Positive	1	1	1	1	1	1	1	1	1
740156	RPPFNMLKRERNRVS	C	9	23	HLA-DRB1*14:02	100%	Dengue virus 4	ELISPOT	IFNg release	Positive	1	1	1	1	1	1	1	1	1
868517	QLKKNKAIKILTGFR	C	42	56	HLA-DQA1*01:02/DQB1*06:02	100%	Dengue virus 4	ELISPOT	IFNg release	Positive	1	1	1	1	1	1	1	1	1
195283	FALLAGFMAYMIGQT	PreM	246	260	HLA-DRB1*01:01	100%	Dengue virus 4	ELISPOT	IFNg release	Positive	1	1	1	1	1	1	1	1	1
195505	ILRNPGFALLAGFMA	PreM	240	254	HLA-DRB1*01:01	100%	Dengue virus 4	ELISPOT	IFNg release	Positive	1	1	1	1	1	1	1	1	1
195901	RNPGFALLAGFMAYM	PreM	242	256	HLA-DRB1*15:01	100%	Dengue virus 4	ELISPOT	IFNg release	Positive	1	1	1	1	1	1	1	1	1
539395	IDCWCNLTSAWVMYG	PreM	117	131	HLA-DQA1*01:02/DQB1*06:02	100%	Dengue virus 4	ELISPOT	IFNg release	Positive	1	1	1	1	1	1	1	1	1
869119	TEGINKCTLIAMDLG	PreM	113	127	HLA-DQA1*01:02/DQB1*06:02	100%	Dengue virus 4	ELISPOT	IFNg release	Positive	1	1	1	1	1	1	1	1	1
195202	CIAVGGITLFLGFTV	E	758	772	HLA-DRB1*15:01	100%	Dengue virus 4	ELISPOT	IFNg release	Positive	1	1	1	1	1	1	1	1	1
195272	ESTYRGAKRMAILGE	E	682	696	HLA-DRB1*15:06	100%	Dengue virus 4	ELISPOT	IFNg release	Positive	1	1	1	1	1	1	1	1	1
539379	HAKRQDVTVLGSQEGAMH	E	523	540	HLA class II	100%	Dengue virus 4	ICS	IFNg release	Positive	1	1	1	1	1	1	1	1	1
539734	NLEYTVVVTVHNGDT	E	197	211	HLA-DRB1*04:07	100%	Dengue virus 4	ELISPOT	IFNg release	Positive	1	1	1	1	1	1	1	1	1
738809	ENLEYTVVVTVHNGD	E	196	210	HLA-DRB1*04:07	100%	Dengue virus 4	ELISPOT	IFNg release	Positive	1	1	1	1	1	1	1	1	1
738886	FESTYRGAKRMAILG	E	681	695	HLA-DRB1*14:02	100%	Dengue virus 4	ELISPOT	IFNg release	Positive	1	1	1	1	1	1	1	1	1
741747	MRCVGVGNRDFVEGVSGGAW	E	280	299	HLA class II	100%	Dengue virus 4	ELISPOT	IFNg release	Positive	1	1	1	1	1	1	1	1	1
195541	ITALILGAQALPVYL	NS2a	1320	1334	HLA-DRB1*01:01	100%	Dengue virus 4	ELISPOT	IFNg release	Positive	1	1	1	1	1	1	1	1	1
195699	LQKQSHWVEITALIL	NS2a	1311	1325	HLA-DRB1*07:01	100%	Dengue virus 4	ELISPOT	IFNg release	Positive	1	1	1	1	1	1	1	1	1
195837	PLVMAWRTIMAVLFV	NS2a	1285	1299	HLA-DRB1*01:01	100%	Dengue virus 4	ELISPOT	IFNg release	Positive	1	1	1	1	1	1	1	1	1
195864	QSHWVEITALILGAQ	NS2a	1314	1328	HLA-DRB1*07:01	100%	Dengue virus 4	ELISPOT	IFNg release	Positive	1	1	1	1	1	1	1	1	1
196060	TMPLVMAWRTIMAVL	NS2a	1283	1297	HLA-DRB1*03:01	100%	Dengue virus 4	ELISPOT	IFNg release	Positive	1	1	1	1	1	1	1	1	1
196227	WVEITALILGAQALP	NS2a	1317	1331	HLA-DRB1*01:01	100%	Dengue virus 4	ELISPOT	IFNg release	Positive	1	1	1	1	1	1	1	1	1

539131	CLRRRVTRKHMILVV	NS2a	1148	1162	HLA-DRB1*08:03	100%	Dengue virus 4	ELISPOT	IFNg release	Positive	1	1	1	1	1	1	1	1	1
539687	MPLVMAWRTIMAVLF	NS2a	1284	1298	HLA-DRB1*12:02	100%	Dengue virus 4	ELISPOT	IFNg release	Positive	1	1	1	1	1	1	1	1	1
539937	STMPLVMAWRTIMAV	NS2a	1282	1296	HLA-DRB1*13:01	100%	Dengue virus 4	ELISPOT	IFNg release	Positive	1	1	1	1	1	1	1	1	1
738742	ECLRRRVTRKHMILV	NS2a	1147	1161	HLA-DRB1*14:02	100%	Dengue virus 4	ELISPOT	IFNg release	Positive	1	1	1	1	1	1	1	1	1
867110	HWVEITALILGAQAL	NS2a	1316	1330	HLA-DQA1*03:01/DQB1*03:02	100%	Dengue virus 4	ELISPOT	IFNg release	Positive	1	1	1	1	1	1	1	1	1
195511	IMAVGLVSLLGSALL	NS2b	1352	1366	HLA-DRB1*01:01	100%	Dengue virus 4	ELISPOT	IFNg release	Positive	1	1	1	1	1	1	1	1	1
195662	LLLAAYVMSGSSADL	NS2b	1381	1395	HLA-DRB1*04:01	100%	Dengue virus 4	ELISPOT	IFNg release	Positive	1	1	1	1	1	1	1	1	1
196062	TNMITLLVKLALITV	NS2b	1436	1450	HLA-DRB1*08:02	100%	Dengue virus 4	ELISPOT	IFNg release	Positive	1	1	1	1	1	1	1	1	1
869635	VTMTLWYMWQVKTQR	NS2b	1460	1474	HLA-DPB1*01:01	100%	Dengue virus 4	ELISPOT	IFNg release	Positive	1	1	1	1	1	1	1	1	1
1067119	GKVIGLYGNGVVTKS	NS3	1618	1632	HLA-DRB1*15:01	100%	Dengue virus 4	ELISPOT	IFNg release	Positive	1	1	1	1	1	1	1	1	1
12605	EIVDLMCHAT	NS3	255	264	HLA-DPw2	100%	Dengue virus 4	51 chromium	cytotoxicity	Positive	1	1	1	1	1	1	1	1	1
156711	LAPTRVVAAEMEEAL	NS3	1695	1709	HLA class II	100%	Dengue virus 4	51 chromium	cytotoxicity	Positive	1	1	1	1	1	1	1	1	1
195298	FHTMWHVTRGSVICH	NS3	1520	1534	HLA-DRB1*07:01	100%	Dengue virus 4	ELISPOT	IFNg release	Positive	1	1	1	1	1	1	1	1	1
195563	KGKVIGLYGNGVVTK	NS3	5	19	HLA-DRB1*15:01	100%	Dengue virus 4	ELISPOT	IFNg release	Positive	1	1	1	1	1	1	1	1	1
195695	LPVWLSYKVASAGIS	NS3	2016	2030	HLA-DRB1*08:02	100%	Dengue virus 4	ELISPOT	IFNg release	Positive	1	1	1	1	1	1	1	1	1
195838	PNYNLIVMDEAHFTD	NS3	1750	1764	HLA-DRB1*03:01	100%	Dengue virus 4	ELISPOT	IFNg release	Positive	1	1	1	1	1	1	1	1	1
33192	KRLTIMDLHPGAGKT	NS3	186	200	HLA class II	100%	Dengue virus 4	ICS	IFNg release	Positive	1	1	1	1	1	1	1	1	1
539024	AAAIFMTATPPGTTD	NS3	1783	1797	HLA-DRB1*04:03	100%	Dengue virus 4	ELISPOT	IFNg release	Positive	1	1	1	1	1	1	1	1	1
539080	ALRGLPIRYQTPAVK	NS3	96	110	HLA-DRB1*13:01	100%	Dengue virus 4	ELISPOT	IFNg release	Positive	1	1	1	1	1	1	1	1	1
539359	GVFHTMWHVTRGSVI	NS3	1518	1532	HLA-DRB1*08:03	100%	Dengue virus 4	ELISPOT	IFNg release	Positive	1	1	1	1	1	1	1	1	1
539520	KVIGLYGNGVVTKSG	NS3	7	21	HLA-DRB1*15:06	100%	Dengue virus 4	ELISPOT	IFNg release	Positive	1	1	1	1	1	1	1	1	1
539803	QGKTVWFVPSIKAGN	NS3	1829	1843	HLA-DRB1*08:03	100%	Dengue virus 4	ELISPOT	IFNg release	Positive	1	1	1	1	1	1	1	1	1
68977	VIGLYGNGV	NS3	146	154	HLA-DRB1*15:01	100%	Dengue virus 4	51 chromium	cytotoxicity	Positive	1	1	1	1	1	1	1	1	1
863140	TRVEMGEAAAIFMTA	NS3	1776	1790	HLA-DQA1*03:01/DQB1*03:02	100%	Dengue virus 4	ELISPOT	IFNg release	Positive	1	1	1	1	1	1	1	1	1
867453	KLTDWDFVVTTDISE	NS3	1872	1886	HLA-DQA1*03:01/DQB1*03:02	100%	Dengue virus 4	ELISPOT	IFNg release	Positive	1	1	1	1	1	1	1	1	1
167758	ILTEIASLPTYLSSRAKL	NS4a	6	23	HLA-DP	100%	Dengue virus 4	ELISPOT	IFNg release	Positive	1	1	1	1	1	1	1	1	1
195176	ASLPTYLSSRAKLAL	NS4a	2103	2117	HLA-DRB1*07:01	100%	Dengue virus 4	ELISPOT	IFNg release	Positive	1	1	1	1	1	1	1	1	1
195224	DNQLIYVILTILTII	NS4a	2223	2237	HLA-DRB1*15:01	100%	Dengue virus 4	ELISPOT	IFNg release	Positive	1	1	1	1	1	1	1	1	1
195809	NQLIYVILTILTIIG	NS4a	2224	2238	HLA-DRB1*08:02	100%	Dengue virus 4	ELISPOT	IFNg release	Positive	1	1	1	1	1	1	1	1	1
195876	RAKLALDNIVMLHTT	NS4a	2112	2126	HLA-DRB1*03:01	100%	Dengue virus 4	ELISPOT	IFNg release	Positive	1	1	1	1	1	1	1	1	1
539165	DILTEIASLPTYLSS	NS4a	2097	2111	HLA-DRB1*04:07	100%	Dengue virus 4	ELISPOT	IFNg release	Positive	1	1	1	1	1	1	1	1	1
539313	GKAYQHALNELPESL	NS4a	2130	2144	HLA-DRB1*10:01	100%	Dengue virus 4	ELISPOT	IFNg release	Positive	1	1	1	1	1	1	1	1	1
539631	LTEIASLPTYLSSRA	NS4a	2099	2113	HLA-DRB1*04:03	100%	Dengue virus 4	ELISPOT	IFNg release	Positive	1	1	1	1	1	1	1	1	1
738568	AYQHALNELPESLET	NS4a	2132	2146	HLA-DRB1*04:07	100%	Dengue virus 4	ELISPOT	IFNg release	Positive	1	1	1	1	1	1	1	1	1
868951	SLPTYLSSRAKLALD	NS4a	2104	2118	HLA-DRB3*02:02	100%	Dengue virus 4	ELISPOT	IFNg release	Positive	1	1	1	1	1	1	1	1	1
195177	ASLVMLLVHYAIIGP	NS4b	2348	2362	HLA-DRB1*13:01	100%	Dengue virus 4	ELISPOT	IFNg release	Positive	1	1	1	1	1	1	1	1	1
195219	DLEPISYDPKFEKQL	NS4b	2393	2407	HLA-DRB1*03:01	100%	Dengue virus 4	ELISPOT	IFNg release	Positive	1	1	1	1	1	1	1	1	1
195276	ETTILDVDLRPASAW	NS4b	2263	2277	HLA-DRB1*03:01	100%	Dengue virus 4	ELISPOT	IFNg release	Positive	1	1	1	1	1	1	1	1	1
195592	KTETTILDVDLRPAS	NS4b	2261	2275	HLA-DRB1*03:01	100%	Dengue virus 4	ELISPOT	IFNg release	Positive	1	1	1	1	1	1	1	1	1
195616	LCAGQLLLMRTTWAF	NS4b	2415	2429	HLA-DRB1*04:01	100%	Dengue virus 4	ELISPOT	IFNg release	Positive	1	1	1	1	1	1	1	1	1
195720	LTASLVMLLVHYAII	NS4b	2346	2360	HLA-DRB1*12:02	100%	Dengue virus 4	ELISPOT	IFNg release	Positive	1	1	1	1	1	1	1	1	1
196048	TILDVDLRPASAWTL	NS4b	2265	2279	HLA-DRB1*03:01	100%	Dengue virus 4	ELISPOT	IFNg release	Positive	1	1	1	1	1	1	1	1	1
196208	WAFCEVLTLATGPIL	NS4b	2427	2441	HLA-DRB1*07:01	100%	Dengue virus 4	ELISPOT	IFNg release	Positive	1	1	1	1	1	1	1	1	1
196226	WTLYAVATTILTPML	NS4b	2277	2291	HLA-DRB1*07:01	100%	Dengue virus 4	ELISPOT	IFNg release	Positive	1	1	1	1	1	1	1	1	1
539057	AGQLLLMRTTWAFCE	NS4b	2417	2431	HLA-DRB1*13:01	100%	Dengue virus 4	ELISPOT	IFNg release	Positive	1	1	1	1	1	1	1	1	1
539185	DVDLRPASAWTLYAV	NS4b	2268	2282	HLA-DRB1*09:01	100%	Dengue virus 4	ELISPOT	IFNg release	Positive	1	1	1	1	1	1	1	1	1
539639	LVHYAIIGPGLQAKA	NS4b	2354	2368	HLA-DQA1*05:01/DQB1*02:01	100%	Dengue virus 4	ELISPOT	IFNg release	Positive	1	1	1	1	1	1	1	1	1
739202	ILDVDLRPASAWTLY	NS4b	2266	2280	HLA-DRB1*01:02	100%	Dengue virus 4	ELISPOT	IFNg release	Positive	1	1	1	1	1	1	1	1	1
1066543	ACLGKAYAQMWSLMY	NS5	3240	3254	HLA-DRB1*04:01	100%	Dengue virus 4	ELISPOT	IFNg release	Positive-Low	1	1	1	1	1	1	1	1	1
195235	EEFISKVRSNAAIGA	NS5	2884	2898	HLA-DRB1*08:02	100%	Dengue virus 4	ELISPOT	IFNg release	Positive	1	1	1	1	1	1	1	1	1
195240	EEYVDYMPVMKRYSA	NS5	3365	3379	HLA-DRB1*11:01	100%	Dengue virus 4	ELISPOT	IFNg release	Positive	1	1	1	1	1	1	1	1	1
195279	EYVDYMPVMKRYSAP	NS5	3366	3380	HLA-DRB1*11:01	100%	Dengue virus 4	ELISPOT	IFNg release	Positive	1	1	1	1	1	1	1	1	1
195737	LWCGSLIGLSSRATW	NS5	3333	3347	HLA-DRB1*11:01	100%	Dengue virus 4	ELISPOT	IFNg release	Positive	1	1	1	1	1	1	1	1	1
195815	NRVWIEDNPNMTDKT	NS5	3302	3316	HLA-DRB1*03:01	100%	Dengue virus 4	ELISPOT	IFNg release	Positive	1	1	1	1	1	1	1	1	1
195823	NWLWALLGKKKNPRL	NS5	2866	2880	HLA-DRB1*14:02	100%	Dengue virus 4	ELISPOT	IFNg release	Positive	1	1	1	1	1	1	1	1	1
195858	QMWSLMYFHRRDLRL	NS5	3248	3262	HLA-DRB1*15:01	100%	Dengue virus 4	ELISPOT	IFNg release	Positive	1	1	1	1	1	1	1	1	1
195879	REEFISKVRSNAAIG	NS5	2883	2897	HLA-DRB1*04:01	100%	Dengue virus 4	ELISPOT	IFNg release	Positive	1	1	1	1	1	1	1	1	1
196105	VDYMPVMKRYSAPFE	NS5	3368	3382	HLA-DRB1*11:01	100%	Dengue virus 4	ELISPOT	IFNg release	Positive	1	1	1	1	1	1	1	1	1
196230	YAQMWSLMYFHRRDL	NS5	3246	3260	HLA-DRB1*15:01	100%	Dengue virus 4	ELISPOT	IFNg release	Positive	1	1	1	1	1	1	1	1	1
539251	FEEYKRSGILEVDRT	NS5	2512	2526	HLA-DRB1*09:01	100%	Dengue virus 4	ELISPOT	IFNg release	Positive	1	1	1	1	1	1	1	1	1
539553	LGEFGRAKGSRAIWY	NS5	2949	2963	HLA-DRB1*09:01	100%	Dengue virus 4	ELISPOT	IFNg release	Positive	1	1	1	1	1	1	1	1	1
539656	MAISGDDCVVKPLDERF	NS5	3146	3162	HLA class II	100%	Dengue virus 4	ICS	IFNg release	Positive	1	1	1	1	1	1	1	1	1
539703	MVTQLAMTDTTPFGQQR	NS5	2824	2840	HLA class II	100%	Dengue virus 4	ICS	IFNg release	Positive	1	1	1	1	1	1	1	1	1
539770	PHHKILAKAIFKLTY	NS5	3046	3060	HLA-DRB1*12:02	100%	Dengue virus 4	ELISPOT	IFNg release	Positive	1	1	1	1	1	1	1	1	1
540041	VGTYGLNTFTNMEVQ	NS5	3091	3105	HLA-DRB1*15:06	100%	Dengue virus 4	ELISPOT	IFNg release	Positive	1	1	1	1	1	1	1	1	1
866633	ESCVYNMMGKREKKL	NS5	2935	2949	HLA-DRB5*01:01	100%	Dengue virus 4	ELISPOT	IFNg release	Positive	1	1	1	1	1	1	1	1	1

868638	RDLRLASMAICSAVP	NS5	3258	3272	HLA-DQA1*01:02/DQB1*06:02	100%	Dengue virus 4	ELISPOT	IFNg release	Positive	1	1	1	1	1	1	1	1	1
539898	SGKGPLRMVLAFITF	C	18	32	HLA-DRB1*15:06	81% - <100%	Dengue virus 4	ELISPOT	IFNg release	Positive	1	1	1	1	1	1	0	1	1
739059	GRMLNILNGRKRSTI	C	88	102	HLA-DRB1*14:06	81% - <100%	Dengue virus 4	ELISPOT	IFNg release	Positive	1	1	1	1	1	1	1	0	1
539463	KERMVTFKVPHAKRQ	E	513	527	HLA-DRB1*04:03	81% - <100%	Dengue virus 4	ELISPOT	IFNg release	Positive	1	1	1	1	0	1	1	1	1
540080	VRILIGFLVLWIGTN	E	455	469	HLA-DRB1*15:06	81% - <100%	Dengue virus 4	ELISPOT	IFNg release	Positive	1	1	1	1	1	0	1	1	1
195822	NVMWKQITNELNYVL	NS1	1	15	HLA-DRB1*04:07	81% - <100%	Dengue virus 4	ELISPOT	IFNg release	Positive	1	1	1	1	1	1	0	1	1
867043	HADMGYWIESSKNQT	NS1	969	983	HLA-DQA1*01:01/DQB1*05:01	81% - <100%	Dengue virus 4	ELISPOT	IFNg release	Positive	1	1	1	1	1	1	0	1	1
195571	KHMILVVVTTLCAII	NS2a	1156	1170	HLA-DRB1*08:02	81% - <100%	Dengue virus 4	ELISPOT	IFNg release	Positive	1	1	1	1	0	1	1	1	1
195404	GLVSLLGSALLKNDV	NS2b	9	23	HLA-DRB1*01:02	81% - <100%	Dengue virus 4	ELISPOT	IFNg release	Positive	1	1	1	1	1	1	0	1	1
195748	MAVGLVSLLGSALLK	NS2b	1353	1367	HLA-DRB1*01:01	81% - <100%	Dengue virus 4	ELISPOT	IFNg release	Positive	1	1	1	1	1	1	0	1	1
195931	SALLKNDVPLAGPMV	NS2b	1363	1377	HLA-DRB1*03:01	81% - <100%	Dengue virus 4	ELISPOT	IFNg release	Positive	1	1	1	1	1	1	0	1	1
539101	AVGLVSLLGSALLKN	NS2b	1354	1368	HLA-DRB1*10:01	81% - <100%	Dengue virus 4	ELISPOT	IFNg release	Positive	1	1	1	1	1	1	0	1	1
1392192	GREIVDLMCHATFTT	NS3	1727	1741		81% - <100%	Dengue virus 4	biological activity	activation	Positive	1	1	1	1	1	1	0	1	1
195244	EGVYRIMQRGLFGKT	NS3	1494	1508	HLA-DRB1*11:01	81% - <100%	Dengue virus 4	ELISPOT	IFNg release	Positive	1	1	1	1	1	1	0	1	1
195353	GEIGAVTLDFKPGTS	NS3	1595	1609	HLA-DRB1*03:01	81% - <100%	Dengue virus 4	ELISPOT	IFNg release	Positive	1	1	1	1	0	1	1	1	1
196032	TEGVYRIMQRGLFGK	NS3	146	160	HLA-DRB1*11:01	81% - <100%	Dengue virus 4	ELISPOT	IFNg release	Positive	1	1	1	1	1	1	0	1	1
539547	LFKTLTGEIGAVTLD	NS3	1589	1603	HLA-DRB1*10:01	81% - <100%	Dengue virus 4	ELISPOT	IFNg release	Positive	1	1	1	1	0	1	1	1	1
539767	PGLFKTLTGEIGAVT	NS3	1587	1601	HLA-DRB1*10:01	81% - <100%	Dengue virus 4	ELISPOT	IFNg release	Positive	1	1	1	1	0	1	1	1	1
739036	GLPIRYQTPAVKSEH	NS3	1711	1725	HLA-DRB1*14:06	81% - <100%	Dengue virus 4	ELISPOT	IFNg release	Positive	1	1	0	1	1	1	1	1	1
739563	LRGLPIRYQTPAVKS	NS3	1709	1723	HLA-DRB1*14:06	81% - <100%	Dengue virus 4	ELISPOT	IFNg release	Positive	1	1	0	1	1	1	1	1	1
195274	ETLMLVALLGAMTAG	NS4a	2145	2159	HLA-DRB1*08:02	81% - <100%	Dengue virus 4	ELISPOT	IFNg release	Positive	1	1	1	0	1	1	1	1	1
195575	KLSMGLIAIAVASGL	NS4a	2172	2186	HLA-DRB1*08:02	81% - <100%	Dengue virus 4	ELISPOT	IFNg release	Positive	1	1	1	1	0	1	1	1	1
196058	TLMLVALLGAMTAGI	NS4a	2146	2160	HLA-DRB1*01:01	81% - <100%	Dengue virus 4	ELISPOT	IFNg release	Positive	1	1	1	0	1	1	1	1	1
539638	LVALLGAMTAGIFLF	NS4a	2149	2163	HLA-DRB1*09:01	81% - <100%	Dengue virus 4	ELISPOT	IFNg release	Positive	1	1	1	0	1	1	1	1	1
539584	LLAMGCYSQVNPTTL	NS4b	2332	2346	HLA-DRB1*09:01	81% - <100%	Dengue virus 4	ELISPOT	IFNg release	Positive	1	1	1	1	0	1	1	1	1
867009	GRFWNTTIAVSTANI	NS4b	2449	2463	HLA-DQA1*01:02/DQB1*06:02	81% - <100%	Dengue virus 4	ELISPOT	IFNg release	Positive	1	1	1	1	1	1	0	1	1
195454	HHFHKIFMKDGRSLV	NS5	3199	3213	HLA-DRB1*03:01	81% - <100%	Dengue virus 4	ELISPOT	IFNg release	Positive	1	1	1	1	1	1	0	1	1
195499	ILAKAIFKLTYQNKV	NS5	3050	3064	HLA-DRB1*07:01	81% - <100%	Dengue virus 4	ELISPOT	IFNg release	Positive	1	1	1	1	0	1	1	1	1
539149	DCVVKPLDERFSTSLLF	NS5	3152	3168	HLA class II	81% - <100%	Dengue virus 4	ICS	IFNg release	Positive	1	1	1	1	0	1	1	1	1
539456	KAIFKLTYQNKVVKV	NS5	3053	3067	HLA-DRB1*09:01	81% - <100%	Dengue virus 4	ELISPOT	IFNg release	Positive	1	1	1	1	0	1	1	1	1
739405	KVRSNAAIGAVFQEE	NS5	2889	2903	HLA-DRB1*01:02	81% - <100%	Dengue virus 4	ELISPOT	IFNg release	Positive	1	1	1	0	1	1	1	1	1
866364	DERFSTSLLFLNDMG	NS5	3159	3173	HLA-DPB1*01:01	81% - <100%	Dengue virus 4	ELISPOT	IFNg release	Positive	1	1	1	1	0	1	1	1	1
867070	HKIFMKDGRSLVVPC	NS5	3202	3216	HLA-DRB3*02:02	81% - <100%	Dengue virus 4	ELISPOT	IFNg release	Positive	1	1	1	1	1	1	0	1	1
867315	ISKVRSNAAIGAVFQ	NS5	2887	2901	HLA-DRB3*02:02	81% - <100%	Dengue virus 4	ELISPOT	IFNg release	Positive	1	1	1	0	1	1	1	1	1
867411	KILAKAIFKLTYQNK	NS5	3049	3063	HLA-DPB1*04:02	81% - <100%	Dengue virus 4	ELISPOT	IFNg release	Positive	1	1	1	1	0	1	1	1	1
195436	GVSWMVRILIGFLVL	E	450	464	HLA-DRB1*08:02	51%-80%	Dengue virus 4	ELISPOT	IFNg release	Positive	1	1	1	1	1	0	0	1	1
195473	IAVGGITLFLGFTVQ	E	759	773	HLA-DRB1*15:01	51%-80%	Dengue virus 4	ELISPOT	IFNg release	Positive	1	1	1	0	0	1	1	0	1
540051	VLGSQEGAMHSALAGA	E	531	546	HLA class II	51%-80%	Dengue virus 4	ICS	IFNg release	Positive	1	1	1	0	0	1	1	0	1
195608	LAIMAVFKMSPGYVL	NS2a	1200	1214	HLA-DRB1*13:01	51%-80%	Dengue virus 4	ELISPOT	IFNg release	Positive	1	0	1	1	1	0	0	1	0
539664	MGGQIHLAIMAVFKM	NS2a	1194	1208	HLA-DRB1*13:01	51%-80%	Dengue virus 4	ELISPOT	IFNg release	Positive	1	0	1	1	1	0	0	1	0
539930	SRETALMVIGMAMTT	NS2a	1223	1237	HLA-DRB1*15:06	51%-80%	Dengue virus 4	ELISPOT	IFNg release	Positive	1	0	1	1	1	0	0	1	0
539962	TALMVIGMAMTTVLS	NS2a	1226	1240	HLA-DRB1*15:06	51%-80%	Dengue virus 4	ELISPOT	IFNg release	Positive	1	0	1	1	1	0	0	1	0
867874	LMVIGMAMTTVLSIP	NS2a	1228	1242	HLA-DQA1*01:02/DQB1*06:02	51%-80%	Dengue virus 4	ELISPOT	IFNg release	Positive	1	0	1	1	1	0	0	1	0
195962	SIVREALKRRLRTLI	NS3	1680	1694	HLA-DRB1*11:01	51%-80%	Dengue virus 4	ELISPOT	IFNg release	Positive	1	1	1	1	1	1	0	0	1
539859	RKSGKKVIQLSRKTF	NS3	1850	1864	HLA-DRB1*08:03	51%-80%	Dengue virus 4	ELISPOT	IFNg release	Positive	1	1	1	0	0	1	1	0	1
539899	SGKKVIQLSRKTFDT	NS3	1852	1866	HLA-DRB1*12:02	51%-80%	Dengue virus 4	ELISPOT	IFNg release	Positive	1	1	1	0	0	1	1	0	1
195214	DFGFYQVKTETTILD	NS4b	2254	2268	HLA-DRB1*04:01	51%-80%	Dengue virus 4	ELISPOT	IFNg release	Positive	1	1	1	0	1	1	1	0	1
195383	GLAFSLIKNAQTPRR	NS4b	2473	2487	HLA-DRB1*04:01	51%-80%	Dengue virus 4	ELISPOT	IFNg release	Positive	0	1	1	0	0	1	1	0	1
196052	TKTDFGFYQVKTETT	NS4b	2251	2265	HLA-DRB1*04:01	51%-80%	Dengue virus 4	ELISPOT	IFNg release	Positive	1	1	1	0	1	1	0	0	1
738447	AFSLIKNAQTPRRGT	NS4b	2475	2489	HLA-DRB1*14:06	51%-80%	Dengue virus 4	ELISPOT	IFNg release	Positive	0	1	1	0	0	1	1	0	1
738897	FGFYQVKTETTILDV	NS4b	2255	2269	HLA-DRB1*04:07	51%-80%	Dengue virus 4	ELISPOT	IFNg release	Positive	1	1	1	0	1	1	1	0	1
739417	LAFSLIKNAQTPRRG	NS4b	2474	2488	HLA-DRB1*14:02	51%-80%	Dengue virus 4	ELISPOT	IFNg release	Positive	0	1	1	0	0	1	1	0	1
195411	GNIVSSVNTTSKMLL	NS5	2713	2727	HLA-DRB1*04:01	51%-80%	Dengue virus 4	ELISPOT	IFNg release	Positive	1	1	1	0	1	1	1	0	1
539987	TLLCLIPTVMAFHLS	C	103	117	HLA-DRB1*04:03	>0% - 50%	Dengue virus 4	ELISPOT	IFNg release	Positive	1	0	0	1	1	0	0	1	0
195526	IQRTVFFILMMLVAP	PreM	262	276	HLA-DRB1*07:01	>0% - 50%	Dengue virus 4	ELISPOT	IFNg release	Positive	0	0	0	1	1	0	0	1	0
539820	QRTVFFILMMLVAPS	PreM	263	277	HLA-DRB1*13:01	>0% - 50%	Dengue virus 4	ELISPOT	IFNg release	Positive	0	0	0	1	1	0	0	1	0
867174	IGQTGIQRTVFFILM	PreM	257	271	HLA-DPB1*02:01	>0% - 50%	Dengue virus 4	ELISPOT	IFNg release	Positive	0	0	0	1	1	0	0	1	0
195358	GGLFTSLGKAVHQVF	E	705	719	HLA-DRB1*11:01	>0% - 50%	Dengue virus 4	ELISPOT	IFNg release	Positive	0	0	0	0	0	0	0	1	0
195472	IAVGGITLFLGFTVH	E	480	494	HLA-DRB1*15:01	>0% - 50%	Dengue virus 4	ELISPOT	IFNg release	Positive	0	0	0	1	1	0	0	1	0
539298	GGVSWMVRILIGLLV	E	449	463	HLA-DRB1*15:06	>0% - 50%	Dengue virus 4	ELISPOT	IFNg release	Positive	0	0	0	0	0	1	0	0	0
540036	VGGLFTSLGKAVHQV	E	704	718	HLA-DRB1*09:01	>0% - 50%	Dengue virus 4	ELISPOT	IFNg release	Positive	0	0	0	0	0	0	0	1	0
866696	FGMFTTNIWMKFREG	NS1	934	948	HLA-DPB1*04:01	>0% - 50%	Dengue virus 4	ELISPOT	IFNg release	Positive	0	0	0	1	1	0	0	1	0
195220	DLMEFIDGISLGLIL	NS2a	1244	1258	HLA-DRB1*07:01	>0% - 50%	Dengue virus 4	ELISPOT	IFNg release	Positive	0	1	1	0	0	0	0	0	0
196252	YVLGIFLRKLTSRET	NS2a	1212	1226	HLA-DRB1*11:01	>0% - 50%	Dengue virus 4	ELISPOT	IFNg release	Positive	1	0	0	1	1	0	0	1	0
539555	LGIFLRKLTSRETAL	NS2a	1214	1228	HLA-DRB1*08:03	>0% - 50%	Dengue virus 4	ELISPOT	IFNg release	Positive	1	0	0	1	1	0	0	1	0
740701	VVTLIPLCRTSCLQK	NS2a	1299	1313	HLA-DRB1*11:04	>0% - 50%	Dengue virus 4	ELISPOT	IFNg release	Positive	0	0	0	1	1	0	0	1	0

867048	HDLMEFIDGISLGLI	NS2a	1243	1257	HLA-DQA1*01:01/DQB1*05:01	>0% - 50%	Dengue virus 4	ELISPOT	IFNg release	Positive	0	1	1	0	0	0	0	0	0
740013	QLIRQMEAEGVITQD	NS5	3105	3119	HLA-DRB1*01:02	>0% - 50%	Dengue virus 4	ELISPOT	IFNg release	Positive	0	0	0	0	0	0	0	1	0

CD8 DENV-1 Epitopes

											NCBI IDs of DENV-1 circulating strains from India, (100% identity=1,green ; else 0, red)																												
Epitope_ID	AA_Seq	Protein_Name	Start	End	Allele	step	Serotype	Method/Technique	Assay Group	Qualitative Measure	UBI73847.1	ASD49619.1	AFZ40118.1	ANC57581.1	QCZ25006.1	QCZ25005.1	QCZ25004.1	QCZ25002.1	QCZ25000.1	QCZ24998.1	QCZ24995.1	QCZ24994.1	QBA57534.1	QBA57538.1	AJP08954.1	QCE20686.1	AXX75607.1	AXX75606.1	AFN54940.1	AFN54941.1	AFN54942.1	AFN54943.1	AHZ44537.1	AFZ40223.1	AFZ40224.1	QCE20685.1	AXX75608.1	AXX75609.1	ASD49618.1
184844	MLKRERNRV	C	15	23	HLA-B*08:01	0%	Dengue virus 1	ELISPOT	IFNg release	Positive	0	0	0	0	0	0	0	0	0	0	0	0	0	0	0	0	0	0	0	0	0	0	0	0	0	0	0	0	0
240082	MVLALITFL	C	24	32	HLA-A*02:01	0%	Dengue virus 1	ELISPOT	IFNg release	Positive	0	0	0	0	0	0	0	0	0	0	0	0	0	0	0	0	0	0	0	0	0	0	0	0	0	0	0	0	0
180641	MALSIVSLF	NS2a	1297	1305	HLA-B*51:01	0%	Dengue virus 1	ELISPOT	IFNg release	Positive	0	0	0	0	0	0	0	0	0	0	0	0	0	0	0	0	0	0	0	0	0	0	0	0	0	0	0	0	0
419037	RPMFAVGILF	NS2a	1211	1220	HLA-B*35:01	0%	Dengue virus 1	ELISPOT	IFNg release	Positive	0	0	0	0	0	0	0	0	0	0	0	0	0	0	0	0	0	0	0	0	0	0	0	0	0	0	0	0	0
150254	GPLVAGGLL	NS2b	1375	1383	HLA-B*07:02	0%	Dengue virus 1	ELISPOT	IFNg release	Positive	0	0	0	0	0	0	0	0	0	0	0	0	0	0	0	0	0	0	0	0	0	0	0	0	0	0	0	0	0
180682	NPEIEDDIF	NS3	1650	1658	HLA-B*35:01	0%	Dengue virus 1	ELISPOT	IFNg release	Positive	0	0	0	0	0	0	0	0	0	0	0	0	0	0	0	0	0	0	0	0	0	0	0	0	0	0	0	0	0
32147	KLRTLVLAPTRVVAS	NS3	216	230	HLA class I	0%	Dengue virus 1	ELISPOT	IFNg release	Positive	0	0	0	0	0	0	0	0	0	0	0	0	0	0	0	0	0	0	0	0	0	0	0	0	0	0	0	0	0
539750	NVRTLILAPTRVVASEM	NS3	1690	1706	HLA class I	0%	Dengue virus 1	ICS	IFNg release	Positive	0	0	0	0	0	0	0	0	0	0	0	0	0	0	0	0	0	0	0	0	0	0	0	0	0	0	0	0	0
65549	TPEGIIPSM	NS3	1975	1983	HLA-B*53:01	0%	Dengue virus 1	51 chromium	cytotoxicity	Positive	0	0	0	0	0	0	0	0	0	0	0	0	0	0	0	0	0	0	0	0	0	0	0	0	0	0	0	0	0
150674	TVMDIISRR	NS5	3080	3088	HLA-A*11:01	0%	Dengue virus 1	multimer/tetramer	qualitative binding	Positive	0	0	0	0	0	0	0	0	0	0	0	0	0	0	0	0	0	0	0	0	0	0	0	0	0	0	0	0	0
184787	MEVQLVRQM	NS5	3107	3115	HLA-B*40:01	0%	Dengue virus 1	ELISPOT	IFNg release	Positive	0	0	0	0	0	0	0	0	0	0	0	0	0	0	0	0	0	0	0	0	0	0	0	0	0	0	0	0	0
150378	LLMLVTPSM	PreM	271	279	HLA-B*08:01	100%	Dengue virus 1	ELISPOT	IFNg release	Positive	1	1	1	1	1	1	1	1	1	1	1	1	1	1	1	1	1	1	1	1	1	1	1	1	1	1	1	1	1
185893	TEVTNPAVL	E	329	337	HLA-B*40:01	100%	Dengue virus 1	ELISPOT	IFNg release	Positive	1	1	1	1	1	1	1	1	1	1	1	1	1	1	1	1	1	1	1	1	1	1	1	1	1	1	1	1	1
186551	YENLKYSVIV	E	412	421	HLA-B*40:01	100%	Dengue virus 1	ELISPOT	IFNg release	Positive	1	1	1	1	1	1	1	1	1	1	1	1	1	1	1	1	1	1	1	1	1	1	1	1	1	1	1	1	1
50596	QEGAMHTAL	E	536	544	HLA-A*02:01	100%	Dengue virus 1	ELISPOT	IFNg release	Positive	1	1	1	1	1	1	1	1	1	1	1	1	1	1	1	1	1	1	1	1	1	1	1	1	1	1	1	1	1
150258	GPWHLGKLEL	NS1	1041	1050	HLA-B*07:02	100%	Dengue virus 1	ELISPOT	IFNg release	Positive	1	1	1	1	1	1	1	1	1	1	1	1	1	1	1	1	1	1	1	1	1	1	1	1	1	1	1	1	1
183246	FRRLTSREVL	NS2a	1220	1229	HLA-B*08:01	100%	Dengue virus 1	ELISPOT	IFNg release	Positive	1	1	1	1	1	1	1	1	1	1	1	1	1	1	1	1	1	1	1	1	1	1	1	1	1	1	1	1	1
184274	LALMATFKM	NS2a	1202	1210	HLA-B*35:01	100%	Dengue virus 1	ELISPOT	IFNg release	Positive	1	1	1	1	1	1	1	1	1	1	1	1	1	1	1	1	1	1	1	1	1	1	1	1	1	1	1	1	1
184856	MLMTGTLAVF	NS2a	1158	1167	HLA-B*35:01	100%	Dengue virus 1	ELISPOT	IFNg release	Positive	1	1	1	1	1	1	1	1	1	1	1	1	1	1	1	1	1	1	1	1	1	1	1	1	1	1	1	1	1
13346	EMAEALKGMPIRYQT	NS3	231	245	HLA class I	100%	Dengue virus 1	ELISPOT	IFNg release	Positive	1	1	1	1	1	1	1	1	1	1	1	1	1	1	1	1	1	1	1	1	1	1	1	1	1	1	1	1	1
150341	KPGTSGSPI	NS3	1606	1614	HLA-B*07:02	100%	Dengue virus 1	ELISPOT	IFNg release	Positive	1	1	1	1	1	1	1	1	1	1	1	1	1	1	1	1	1	1	1	1	1	1	1	1	1	1	1	1	1
150538	RPRWLDART	NS3	2068	2076	HLA-B*07:02	100%	Dengue virus 1	ELISPOT	IFNg release	Positive	1	1	1	1	1	1	1	1	1	1	1	1	1	1	1	1	1	1	1	1	1	1	1	1	1	1	1	1	1
180472	GEARKTFVEL	NS3	2003	2012	HLA-B*40:01	100%	Dengue virus 1	ELISPOT	IFNg release	Positive	1	1	1	1	1	1	1	1	1	1	1	1	1	1	1	1	1	1	1	1	1	1	1	1	1	1	1	1	1
180804	VPNYNMIIM	NS3	1751	1759	HLA-B*51:01	100%	Dengue virus 1	ELISPOT	IFNg release	Positive	1	1	1	1	1	1	1	1	1	1	1	1	1	1	1	1	1	1	1	1	1	1	1	1	1	1	1	1	1
183229	FPQSNAVIQD	NS3	1801	1810	HLA-B*35:01	100%	Dengue virus 1	ELISPOT	IFNg release	Positive	1	1	1	1	1	1	1	1	1	1	1	1	1	1	1	1	1	1	1	1	1	1	1	1	1	1	1	1	1
183598	HPGSGKTRRY	NS3	1670	1679	HLA-B*35:01	100%	Dengue virus 1	ELISPOT	IFNg release	Positive	1	1	1	1	1	1	1	1	1	1	1	1	1	1	1	1	1	1	1	1	1	1	1	1	1	1	1	1	1
184316	LEENMDVEIW	NS3	2049	2058	HLA-B*44:03	100%	Dengue virus 1	ELISPOT	IFNg release	Positive	1	1	1	1	1	1	1	1	1	1	1	1	1	1	1	1	1	1	1	1	1	1	1	1	1	1	1	1	1
184884	MPIRYQTTAV	NS3	1714	1723	HLA-B*35:01	100%	Dengue virus 1	ELISPOT	IFNg release	Positive	1	1	1	1	1	1	1	1	1	1	1	1	1	1	1	1	1	1	1	1	1	1	1	1	1	1	1	1	1
185741	SPVRVPNYNM	NS3	1747	1756	HLA-B*35:01	100%	Dengue virus 1	ELISPOT	IFNg release	Positive	1	1	1	1	1	1	1	1	1	1	1	1	1	1	1	1	1	1	1	1	1	1	1	1	1	1	1	1	1
185827	SYKVASEGF	NS3	2023	2031	HLA-A*24:02	100%	Dengue virus 1	ELISPOT	IFNg release	Positive	1	1	1	1	1	1	1	1	1	1	1	1	1	1	1	1	1	1	1	1	1	1	1	1	1	1	1	1	1
186284	VLDDGIYRI	NS3	1492	1500	HLA-A*02:01	100%	Dengue virus 1	ELISPOT	IFNg release	Positive	1	1	1	1	1	1	1	1	1	1	1	1	1	1	1	1	1	1	1	1	1	1	1	1	1	1	1	1	1
186559	YKVASEGFQY	NS3	2024	2033	HLA-B*35:01	100%	Dengue virus 1	ELISPOT	IFNg release	Positive	1	1	1	1	1	1	1	1	1	1	1	1	1	1	1	1	1	1	1	1	1	1	1	1	1	1	1	1	1
22825	GTSGSPIVNR	NS3	133	142	HLA-B*57:01	100%	Dengue virus 1	51 chromium	cytotoxicity	Positive	1	1	1	1	1	1	1	1	1	1	1	1	1	1	1	1	1	1	1	1	1	1	1	1	1	1	1	1	1
38766	LPVWLSYKV	NS3	2018	2026	HLA-B*51:01	100%	Dengue virus 1	ELISPOT	IFNg release	Positive	1	1	1	1	1	1	1	1	1	1	1	1	1	1	1	1	1	1	1	1	1	1	1	1	1	1	1	1	1
39095	LRGEARKTFVELMRR	NS3	526	540	HLA class I	100%	Dengue virus 1	ELISPOT	IFNg release	Positive	1	1	1	1	1	1	1	1	1	1	1	1	1	1	1	1	1	1	1	1	1	1	1	1	1	1	1	1	1
419024	NMIIMDEAHF	NS3	1755	1764	HLA-B*15:01	100%	Dengue virus 1	ELISPOT	IFNg release	Positive	1	1	1	1	1	1	1	1	1	1	1	1	1	1	1	1	1	1	1	1	1	1	1	1	1	1	1	1	1
56309	RVIDPRRCL	NS3	1897	1905	HLA-B*07:02	100%	Dengue virus 1	ELISPOT	IFNg release	Positive	1	1	1	1	1	1	1	1	1	1	1	1	1	1	1	1	1	1	1	1	1	1	1	1	1	1	1	1	1
150649	TPQDNQLAYV	NS4a	2222	2231	HLA-B*07:02	100%	Dengue virus 1	ELISPOT	IFNg release	Positive	1	1	1	1	1	1	1	1	1	1	1	1	1	1	1	1	1	1	1	1	1	1	1	1	1	1	1	1	1
180780	TPQDNQLAY	NS4a	2222	2230	HLA-B*35:01	100%	Dengue virus 1	ELISPOT	IFNg release	Positive	1	1	1	1	1	1	1	1	1	1	1	1	1	1	1	1	1	1	1	1	1	1	1	1	1	1	1	1	1
184522	LPDTIETLM	NS4a	2142	2150	HLA-B*35:01	100%	Dengue virus 1	ELISPOT	IFNg release	Positive	1	1	1	1	1	1	1	1	1	1	1	1	1	1	1	1	1	1	1	1	1	1	1	1	1	1	1	1	1
184523	LPDTIETLML	NS4a	2142	2151	HLA-B*35:01	100%	Dengue virus 1	ELISPOT	IFNg release	Positive	1	1	1	1	1	1	1	1	1	1	1	1	1	1	1	1	1	1	1	1	1	1	1	1	1	1	1	1	1
419128	AYRHAMEEL	NS4a	2134	2142	HLA-A*24:02	100%	Dengue virus 1	ELISPOT	IFNg release	Positive-High	1	1	1	1	1	1	1	1	1	1	1	1	1	1	1	1	1	1	1	1	1	1	1	1	1	1	1	1	1
41986	MLLALIAVL	NS4a	2150	2158	HLA-A2	100%	Dengue virus 1	ICS	IFNg release	Positive	1	1	1	1	1	1	1	1	1	1	1	1	1	1	1	1	1	1	1	1	1	1	1	1	1	1	1	1	1
150474	NPLTLTAAV	NS4b	2348	2356	HLA-B*07:02	100%	Dengue virus 1	ELISPOT	IFNg release	Positive	1	1	1	1	1	1	1	1	1	1	1	1	1	1	1	1	1	1	1	1	1	1	1	1	1	1	1	1	1
186427	VVYDAKFEK	NS4b	2403	2411	HLA-A*11:01	100%	Dengue virus 1	ELISPOT	IFNg release	Positive	1	1	1	1	1	1	1	1	1	1	1	1	1	1	1	1	1	1	1	1	1	1	1	1	1	1	1	1	1
27180	ILLMRTTWA	NS4b	2426	2434	HLA-A2	100%	Dengue virus 1	ICS	IFNg release	Positive	1	1	1	1	1	1	1	1	1	1	1	1	1	1	1	1	1	1	1	1	1	1	1	1	1	1	1	1	1
41093	MANIFRGSY	NS4b	2466	2474	HLA-B*35:01	100%	Dengue virus 1	ELISPOT	IFNg release	Positive	1	1	1	1	1	1	1	1	1	1	1	1	1	1	1	1	1	1	1	1	1	1	1	1	1	1	1	1	1
69652	VLMLVAHYA	NS4b	2356	2364	HLA-A2	100%	Dengue virus 1	ICS	IFNg release	Positive	1	1	1	1	1	1	1	1	1	1	1	1	1	1	1	1	1	1	1	1	1	1	1	1	1	1	1	1	1
74558	YLAGAGLAF	NS4b	2474	2482	HLA-B*07:02	100%	Dengue virus 1	ELISPOT	IFNg release	Positive	1	1	1	1	1	1	1	1	1	1	1	1	1	1	1	1	1	1	1	1	1	1	1	1	1	1	1	1	1
133702	SRNSTHEMY	NS5	2703	2711	HLA-A*02:01	100%	Dengue virus 1	ELISPOT	IFNg release	Positive	1	1	1	1	1	1	1	1	1	1	1	1	1	1	1	1	1	1	1	1	1	1	1	1	1	1	1	1	1
14325	ETACLGKSY	NS5	3242	3250	HLA-A*26:01	100%	Dengue virus 1	ELISPOT	IFNg release	Positive	1	1	1	1	1	1	1	1	1	1	1	1	1	1	1	1	1	1	1	1	1	1	1	1	1	1	1	1	1
180548	KAKGSRAIW	NS5	2959	2967	HLA-B*57:01	100%	Dengue virus 1	ELISPOT	IFNg release	Positive	1	1	1	1	1	1	1	1	1	1	1	1	1	1	1	1	1	1	1	1	1	1	1	1	1	1	1	1	1
182599	AQMWQLMYF	NS5	3251	3259	HLA-B*15:01	100%	Dengue virus 1	ELISPOT	IFNg release	Positive	1	1	1	1	1	1	1	1	1	1	1	1	1	1	1	1	1	1	1	1	1	1	1	1	1	1	1	1	1
183147	FCSHHFHQL	NS5	3200	3208	HLA-B*08:01	100%	Dengue virus 1	ELISPOT	IFNg release	Positive	1	1	1	1	1	1	1	1	1	1	1	1	1	1	1	1	1	1	1	1	1	1	1	1	1	1	1	1	1
183828	IPMVTQIAM	NS5	2827	2835	HLA-B*07:02	100%	Dengue virus 1	ELISPOT	IFNg release	Positive	1	1	1	1	1	1	1	1	1	1	1	1	1	1	1	1	1	1	1	1	1	1	1	1	1	1	1	1	1
184898	MQRKHGGML	NS5	2689	2697	HLA-B*08:01	100%	Dengue virus 1	ELISPOT	IFNg release	Positive	1	1	1	1	1	1	1	1	1	1	1	1	1	1	1	1	1	1	1	1	1	1	1	1	1	1	1	1	1
184956	MTTEDMLSVW	NS5	3296	3305	HLA-B*58:01	100%	Dengue virus 1	ICS	IFNg release	Positive	1	1	1	1	1	1	1	1	1	1	1	1	1	1	1	1	1	1	1	1	1	1	1	1	1	1	1	1	1
185279	QMQRKHGGML	NS5	2688	2697	HLA-B*08:01	100%	Dengue virus 1	ELISPOT	IFNg release	Positive	1	1	1	1	1	1	1	1	1	1	1	1	1	1	1	1	1	1	1	1	1	1	1	1	1	1	1	1	1
185354	REIVVPCRNQ	NS5	3214	3223	HLA-B*40:01	100%	Dengue virus 1	ELISPOT	IFNg release	Positive	1	1	1	1	1	1	1	1	1	1	1	1	1	1	1	1	1	1	1	1	1	1	1	1	1	1	1	1	1
186150	TVMDVISRR	NS5	3080	3088	HLA-A*68:01	100%	Dengue virus 1	ELISPOT	IFNg release	Positive	1	1	1	1	1	1	1	1	1	1	1	1	1	1	1	1	1	1	1	1	1	1	1	1	1	1	1	1	1
186176	TWSIHAHHQW	NS5	3286	3295	HLA-A*23:01	100%	Dengue virus 1	ELISPOT	IFNg release	Positive	1	1	1	1	1	1	1	1	1	1	1	1	1	1	1	1	1	1	1	1	1	1	1	1	1	1	1	1	1
73076	WSIHAHHQW	NS5	3287	3295	HLA-B*57:01	100%	Dengue virus 1	ELISPOT	IFNg release	Positive	1	1	1	1	1	1	1	1	1	1	1	1	1	1	1	1	1	1	1	1	1	1	1	1	1	1	1	1	1
180637	MAFIAFLRF	C	48	56	HLA-B*35:01	81% - <100%	Dengue virus 1	ELISPOT	IFNg release	Positive	1	0	1	1	1	1	1	1	1	1	1	1	1	1	1	0	1	1	1	1	1	1	1	1	1	1	1	1	1
183175	FIAFLRFLA	C	50	58	HLA-A*68:02	81% - <100%	Dengue virus 1	ELISPOT	IFNg release	Positive	1	0	1	1	1	1	1	1	1	1	1	1	1	1	1	0	1	1	1	1	1	1	1	1	1	1	1	1	1
183220	FNMLKRARNR	C	14	23	HLA-A*33:01	81% - <100%	Dengue virus 1	ELISPOT	IFNg release	Positive	1	0	1	1	1	1	1	1	1	1	1	1	1	1	1	1	1	1	1	1	1	1	1	1	1	1	1	1	0
183800	IMNRRKRSV	C	94	102	HLA-B*08:01	81% - <100%	Dengue virus 1	ELISPOT	IFNg release	Positive	1	1	1	1	1	0	1	1	1	1	1	1	1	1	0	1	1	1	1	1	1	1	1	1	1	1	1	1	1
184458	LLMLLPTAL	C	83	91	HLA-B*08:01	81% - <100%	Dengue virus 1	ELISPOT	IFNg release	Positive	1	1	1	1	1	0	1	1	1	1	1	1	1	1	0	0	1	1	1	1	1	1	1	1	1	1	1	1	1
184880	MNRRKRSVT	C	95	103	HLA-B*08:01	81% - <100%	Dengue virus 1	ELISPOT	IFNg release	Positive	1	1	1	1	1	0	1	1	1	1	1	1	1	1	0	1	1	1	1	1	1	1	1	1	1	1	1	1	1
185102	NRRKRSVTM	C	96	104	HLA-B*08:01	81% - <100%	Dengue virus 1	ELISPOT	IFNg release	Positive	1	1	1	1	1	0	1	1	1	1	1	1	1	1	0	1	1	1	1	1	1	1	1	1	1	1	1	1	1
185492	RRKRSVTMLL	C	97	106	HLA-B*08:01	81% - <100%	Dengue virus 1	ELISPOT	IFNg release	Positive	1	1	1	1	1	0	1	1	1	1	1	1	1	1	0	0	1	1	1	1	1	1	1	1	1	1	1	1	1
183596	HPGFTVIALF	PreM	244	253	HLA-B*35:01	81% - <100%	Dengue virus 1	ELISPOT	IFNg release	Positive	1	1	1	1	1	1	1	1	1	1	1	1	1	1	1	0	1	1	1	1	1	1	1	1	1	1	1	1	1
16554	FLDLPLPWT	E	213	221	HLA-A2	81% - <100%	Dengue virus 1	ICS	IFNg release	Positive	1	1	1	1	1	1	1	1	1	1	1	1	1	1	1	0	1	1	1	1	1	1	1	1	1	1	1	1	1
183421	GLNSRSTSL	E	747	755	HLA-B*08:01	81% - <100%	Dengue virus 1	ELISPOT	IFNg release	Positive	1	1	1	1	1	1	1	1	1	1	1	1	1	1	1	0	1	1	1	1	0	0	1	1	1	1	1	1	1
183654	IAVGMVTLY	E	760	768	HLA-B*35:01	81% - <100%	Dengue virus 1	ELISPOT	IFNg release	Positive	1	1	1	1	1	0	1	1	1	1	1	1	1	1	0	0	0	1	0	1	1	1	1	1	1	1	1	1	1
184377	LGLNSRSTSL	E	746	755	HLA-B*08:01	81% - <100%	Dengue virus 1	ELISPOT	IFNg release	Positive	1	1	1	1	1	1	1	1	1	1	1	1	1	1	1	0	1	1	1	1	0	0	1	1	1	1	1	1	1
184626	LTDYGALTL	E	456	464	HLA-A*01:01	81% - <100%	Dengue virus 1	ELISPOT	IFNg release	Positive	1	1	1	1	1	1	1	1	0	1	1	1	1	1	1	0	1	1	1	1	1	1	1	1	1	1	0	1	1
185212	PTSEIQLTDY	E	449	458	HLA-A*01:01	81% - <100%	Dengue virus 1	ELISPOT	IFNg release	Positive	1	1	0	1	1	0	1	1	1	1	1	1	1	1	0	0	1	1	1	1	1	1	1	1	1	1	1	1	1
419306	PTLDIELLK	E	319	327	HLA-A*11:01	81% - <100%	Dengue virus 1	ELISPOT	IFNg release	Positive	1	1	1	1	1	1	1	1	1	1	1	1	1	1	1	0	1	1	1	1	1	1	1	1	1	1	1	1	1
588005	RDFVEGLSGATWVDVVL	E	289	305	HLA class I	81% - <100%	Dengue virus 1	ICS	IFNg release	Positive	1	1	1	1	1	1	1	1	1	1	1	1	1	1	0	1	1	1	1	1	1	1	1	1	1	1	1	1	1
590597	VEGLSGATWVDVVLEHG	E	292	308	HLA class I	81% - <100%	Dengue virus 1	ICS	IFNg release	Positive-Intermediate	1	1	1	1	1	1	1	1	1	1	1	1	1	1	0	1	1	1	1	1	1	1	1	1	1	1	1	1	1
180836	YGGPISQHNY	NS1	1022	1031	HLA-B*35:01	81% - <100%	Dengue virus 1	ELISPOT	IFNg release	Positive	1	1	1	1	1	1	1	1	1	1	1	1	1	1	1	1	1	1	0	1	1	1	1	1	1	1	1	1	1
183619	HTWTEQYKF	NS1	801	809	HLA-B57	81% - <100%	Dengue virus 1	51 chromium	cytotoxicity	Positive	1	0	1	1	1	1	1	1	1	1	1	1	1	1	1	0	1	1	1	1	1	1	1	1	1	1	1	1	1
60104	SPKRLSAAI	NS1	813	821	HLA-B*08:01	81% - <100%	Dengue virus 1	ELISPOT	IFNg release	Positive	1	1	1	1	1	1	1	1	1	1	1	1	1	1	1	0	1	1	1	1	1	1	1	1	1	1	1	1	1
184623	LTDFQSHQL	NS2a	1264	1272	HLA-A*01:01	81% - <100%	Dengue virus 1	ELISPOT	IFNg release	Positive	1	1	1	1	1	1	1	1	1	1	1	1	1	1	1	0	1	1	1	1	1	0	1	1	1	1	1	1	1
185468	RPMFAVGLLF	NS2a	1211	1220	HLA-B*35:01	81% - <100%	Dengue virus 1	ELISPOT	IFNg release	Positive	1	1	1	1	1	1	1	1	1	1	1	1	1	1	0	1	0	1	1	1	1	1	1	1	1	1	1	1	1
185651	SILLSSLLK	NS2b	1360	1368	HLA-A*11:01	81% - <100%	Dengue virus 1	ELISPOT	IFNg release	Positive	1	1	0	1	1	1	1	1	1	1	1	1	1	1	1	0	1	1	1	1	1	1	1	1	1	1	1	1	1
133501	AIVREAIKR	NS3	1682	1690	HLA-A*02:01	81% - <100%	Dengue virus 1	ELISPOT	IFNg release	Positive	1	1	1	1	1	1	1	1	1	1	0	1	1	1	1	0	1	1	1	1	1	1	1	1	1	0	1	1	1
150169	DPRRCLKPV	NS3	1900	1908	HLA-B*07:02	81% - <100%	Dengue virus 1	ELISPOT	IFNg release	Positive	1	1	1	1	1	1	1	1	1	1	1	1	1	1	0	1	1	1	1	1	1	0	1	1	1	1	1	1	1
150556	RVIDPRRCLK	NS3	1897	1906	HLA-A*03:01	81% - <100%	Dengue virus 1	ELISPOT	IFNg release	Positive	1	1	1	1	1	1	1	1	1	1	1	1	1	1	0	1	1	1	1	1	1	0	1	1	1	1	1	1	1
150643	TPEGIIPALF	NS3	1976	1985	HLA-B*35:01	81% - <100%	Dengue virus 1	ELISPOT	IFNg release	Positive	1	1	1	1	1	1	1	1	1	1	1	1	1	1	1	0	1	1	1	1	1	1	1	1	1	1	1	1	1
167826	TSGTYVSAIAQAKASQE	NS3	157	173	HLA class I	81% - <100%	Dengue virus 1	ELISPOT	IFNg release	Positive	1	0	1	1	1	1	1	1	1	1	1	1	1	1	0	1	1	1	1	1	1	1	1	1	1	1	1	1	1
180373	APTRVVASEM	NS3	1698	1707	HLA class I	81% - <100%	Dengue virus 1	ELISPOT	IFNg release	Positive	1	1	1	1	1	1	1	1	1	1	1	1	1	1	1	0	1	1	1	1	1	1	1	1	1	1	1	1	1
180408	DPASIAARGY	NS3	1766	1775	HLA-B*35:01	81% - <100%	Dengue virus 1	ELISPOT	IFNg release	Positive	1	1	1	0	1	0	1	1	1	1	1	1	1	1	0	1	1	1	1	1	1	1	1	1	1	1	1	1	1
180423	EERDIPERSW	NS3	1811	1820	HLA-B*44:02	81% - <100%	Dengue virus 1	ELISPOT	IFNg release	Positive	1	1	1	1	1	1	1	1	1	1	1	1	1	1	1	1	1	1	0	1	1	1	1	1	1	1	1	1	1
182519	AIKRKLRTL	NS3	1687	1695	HLA-B*08:01	81% - <100%	Dengue virus 1	ELISPOT	IFNg release	Positive	1	1	1	1	1	0	1	1	1	1	0	1	1	1	0	0	1	1	1	1	1	1	1	1	1	0	1	1	1
182772	DISEMGANF	NS3	1885	1893	HLA-A*26:01	81% - <100%	Dengue virus 1	ELISPOT	IFNg release	Positive	1	1	0	1	1	1	1	1	1	1	1	1	1	1	1	1	1	1	1	1	1	1	1	1	1	1	1	1	1
183223	FPGKTVWFVP	NS3	1830	1839	HLA-B*35:01	81% - <100%	Dengue virus 1	ELISPOT	IFNg release	Positive	1	1	1	1	1	1	0	1	1	0	1	1	1	1	1	1	0	1	1	1	1	1	1	1	1	1	1	1	1
183929	IYRILQRGLL	NS3	1497	1506	HLA-B*08:01	81% - <100%	Dengue virus 1	ELISPOT	IFNg release	Positive	1	1	1	1	1	0	1	1	1	1	1	1	1	1	0	1	1	1	1	1	1	1	1	1	1	1	1	1	1
185124	NTPEGIIPA	NS3	1975	1983	HLA-A*68:02	81% - <100%	Dengue virus 1	ELISPOT	IFNg release	Positive	1	1	1	1	1	1	1	1	1	1	1	1	1	1	1	0	1	1	1	1	1	1	1	1	1	1	1	1	1
185125	NTPEGIIPAL	NS3	1975	1984	HLA-A*68:02	81% - <100%	Dengue virus 1	ELISPOT	IFNg release	Positive	1	1	1	1	1	1	1	1	1	1	1	1	1	1	1	0	1	1	1	1	1	1	1	1	1	1	1	1	1
185327	QYIYMGQPL	NS3	1947	1955	HLA-A*24:02	81% - <100%	Dengue virus 1	ELISPOT	IFNg release	Positive	1	1	1	1	1	1	1	1	1	1	1	1	1	1	1	1	1	1	0	1	1	1	1	1	1	1	1	1	1
186603	YSDPLALREF	NS3	2077	2086	HLA-B*35:01	81% - <100%	Dengue virus 1	ELISPOT	IFNg release	Positive	1	1	1	1	1	1	1	1	1	1	1	1	1	1	0	1	1	1	1	1	1	1	1	1	1	1	1	1	1
35237	LDNINTPEGIIPALFEPERE	NS3	496	515	HLA class I	81% - <100%	Dengue virus 1	ELISPOT	IFNg release	Positive	1	1	1	1	1	1	1	1	1	1	1	1	1	1	1	0	1	1	1	1	1	1	1	1	1	1	1	1	1
38400	LPAIVREAI	NS3	1680	1688	HLA-B*53:01	81% - <100%	Dengue virus 1	ELISPOT	IFNg release	Positive	1	1	1	1	1	1	1	1	1	1	0	1	1	1	1	1	1	1	1	1	1	1	1	1	1	0	1	1	1
419124	AVEPGKNPK	NS3	1574	1582	HLA-A*11:01	81% - <100%	Dengue virus 1	ELISPOT	IFNg release	Positive	1	1	1	1	1	1	1	1	1	1	1	1	1	1	1	0	1	1	1	1	1	1	1	1	1	1	1	1	1
419229	IYRILQRGL	NS3	1497	1505	HLA-A*24:02	81% - <100%	Dengue virus 1	ELISPOT	IFNg release	Positive	1	1	1	1	1	0	1	1	1	1	1	1	1	1	0	1	1	1	1	1	1	1	1	1	1	1	1	1	1
50894	QGKRLEPSWASVKKDLISYG	NS3	1536	1555	HLA class I	81% - <100%	Dengue virus 1	ELISPOT	IFNg release	Positive	1	1	1	1	1	1	1	1	1	1	1	1	1	1	0	0	1	1	1	1	1	1	1	1	1	1	1	1	1
539170	DLMCHATF	NS3	1734	1741	HLA-B*08:01	81% - <100%	Dengue virus 1	ICS	IFNg release	Positive	1	1	0	1	1	1	1	1	1	1	1	1	1	1	1	1	1	1	1	1	1	1	1	1	1	1	1	1	1
539663	MGEAAAIFMTATPPGSV	NS3	1782	1798	HLA class I	81% - <100%	Dengue virus 1	ICS	IFNg release	Positive	1	1	1	1	1	1	1	1	0	1	1	1	1	1	1	0	1	1	1	1	1	1	1	1	1	1	1	1	1
539753	NYNMIIMDEAHFTDPA	NS3	1753	1768	HLA class I	81% - <100%	Dengue virus 1	ICS	IFNg release	Positive	1	1	1	1	1	0	1	1	1	1	1	1	1	1	0	1	1	1	1	1	1	1	1	1	1	1	1	1	1
585710	LAPTRVVASEM	NS3	83	93	HLA class I	81% - <100%	Dengue virus 1	ICS	IFNg release	Positive-High	1	1	1	1	1	1	1	1	1	1	1	1	1	1	1	0	1	1	1	1	1	1	1	1	1	1	1	1	1
62184	SVKKDLISYGGGWRF	NS3	71	85	HLA class I	81% - <100%	Dengue virus 1	ELISPOT	IFNg release	Positive	1	0	1	1	1	0	1	1	1	1	1	1	1	1	0	0	1	1	1	1	1	1	1	1	1	1	1	1	0
65546	TPEGIIPAL	NS3	1976	1984	HLA class I	81% - <100%	Dengue virus 1	51 chromium	cytotoxicity	Positive	1	1	1	1	1	1	1	1	1	1	1	1	1	1	1	0	1	1	1	1	1	1	1	1	1	1	1	1	1
182912	EFFLMVLLI	NS4a	2206	2214	HLA-A*24:02	81% - <100%	Dengue virus 1	ELISPOT	IFNg release	Positive	1	1	0	1	1	1	1	1	1	1	1	1	1	1	1	0	1	1	1	1	1	1	1	1	1	1	1	1	1
183162	FFLMVLLIPE	NS4a	2207	2216	HLA-A*33:01	81% - <100%	Dengue virus 1	ELISPOT	IFNg release	Positive	1	1	0	1	1	1	1	1	1	1	1	1	1	1	1	0	1	1	1	1	1	1	1	1	1	1	1	1	1

183813	IPEPDRQRTP	NS4a	2214	2223	HLA-B*35:01	81% - <100%	Dengue virus 1	ELISPOT	IFNg release	Positive	1	1	0	1	1	1	1	1	1	1	1	1	1	1	1	1	1	1	1	1	1	1	1	1	1	1	1	1	1
184739	MASSVLLWM	NS4a	2183	2191	HLA-B*35:01	81% - <100%	Dengue virus 1	ELISPOT	IFNg release	Positive	1	1	1	1	1	0	1	1	1	1	1	1	0	1	0	1	1	1	0	1	1	1	1	1	1	1	1	1	1
133716	TLYAVATTI	NS4b	2284	2292	HLA-A*02:01	81% - <100%	Dengue virus 1	ELISPOT	IFNg release	Positive	1	1	1	1	1	1	1	1	1	1	1	1	1	1	1	1	1	1	0	1	1	1	1	1	1	1	1	1	1
180382	ATGPLTTLW	NS4b	2442	2450	HLA-B*58:01	81% - <100%	Dengue virus 1	ELISPOT	IFNg release	Positive	1	1	1	1	1	1	1	1	1	1	1	1	1	1	0	1	1	1	1	1	1	1	1	1	1	1	1	1	1
183594	HPASAWTLYA	NS4b	2278	2287	HLA-B*35:01	81% - <100%	Dengue virus 1	ELISPOT	IFNg release	Positive	1	1	1	1	1	1	1	1	1	1	1	1	1	1	1	1	1	1	0	1	1	1	1	1	1	1	1	1	1
64286	TIENTTANISLTAIA	NS4b	2300	2314	HLA class I	81% - <100%	Dengue virus 1	ELISPOT	IFNg release	Positive	0	1	1	1	1	1	1	1	1	1	1	1	1	1	1	1	1	1	1	1	1	1	1	1	1	1	1	1	1
1068332	SSWEDVPYL	NS5	3323	3331	HLA-A*02:01	81% - <100%	Dengue virus 1	ELISPOT	IFNg release	Positive	1	1	0	1	1	1	0	1	1	1	1	1	1	1	1	1	0	1	0	1	1	1	1	1	1	1	1	1	1
180376	ASSMVNGVVR	NS5	2809	2818	HLA-B*57:01	81% - <100%	Dengue virus 1	ELISPOT	IFNg release	Positive	1	1	1	1	1	0	1	1	1	1	1	1	1	1	0	1	1	1	1	1	1	1	1	1	1	1	1	1	1
180411	DTTPFGQQR	NS5	2837	2845	HLA-A*68:01	81% - <100%	Dengue virus 1	ELISPOT	IFNg release	Positive	1	1	1	1	1	1	1	1	1	1	1	1	1	1	1	0	1	1	1	1	1	1	1	1	1	1	1	1	1
180571	KPRICTREEF	NS5	2882	2891	HLA-B*07:02	81% - <100%	Dengue virus 1	ELISPOT	IFNg release	Positive	1	1	1	1	1	1	1	1	1	1	1	1	1	1	1	0	1	1	1	1	1	1	1	1	1	1	1	1	1
180723	RFLEFEALGF	NS5	2974	2983	HLA-A*23:01	81% - <100%	Dengue virus 1	ELISPOT	IFNg release	Positive	1	1	1	1	1	1	1	1	1	1	1	1	1	1	1	0	1	1	1	1	1	1	1	1	1	1	1	1	1
182554	ALLATSIFK	NS5	3054	3062	HLA-A*11:01	81% - <100%	Dengue virus 1	ELISPOT	IFNg release	Positive	1	1	1	1	1	0	1	1	1	1	1	1	1	1	0	1	1	1	1	1	1	1	1	1	1	1	1	1	1
182836	DYMTSMKRF	NS5	3373	3381	HLA-A*24:02	81% - <100%	Dengue virus 1	ELISPOT	IFNg release	Positive	1	1	1	1	1	1	1	1	1	1	1	1	1	1	1	1	1	1	0	0	1	1	1	1	1	1	1	1	1
183275	FTNMEVQLIR	NS5	3104	3113	HLA-B*35:01	81% - <100%	Dengue virus 1	ELISPOT	IFNg release	Positive	1	1	1	1	1	0	1	1	1	1	1	1	1	1	0	1	1	1	1	1	1	1	1	1	1	1	1	1	1
184212	KVRKDIPQW	NS5	3178	3186	HLA-B*57:01	81% - <100%	Dengue virus 1	ELISPOT	IFNg release	Positive	1	1	0	1	1	1	1	0	1	1	1	1	1	1	1	1	1	1	1	1	1	1	1	1	1	1	1	1	1
539702	MVTQIAMTDTTPFGQQR	NS5	2829	2845	HLA class I	81% - <100%	Dengue virus 1	ICS	IFNg release	Positive	1	1	1	1	1	1	1	1	1	1	1	1	1	1	1	0	1	1	1	1	1	1	1	1	1	1	1	1	1
62903	TAKWLWGFLSRNKKPRICTR	NS5	2869	2888	HLA class I	81% - <100%	Dengue virus 1	ELISPOT	IFNg release	Positive	1	1	0	1	1	0	1	1	1	1	1	1	1	1	0	0	1	1	1	1	1	1	1	1	1	1	1	1	1
65567	TPFGQQRVF	NS5	2839	2847	HLA class I	81% - <100%	Dengue virus 1	ELISPOT	IFNg release	Positive	1	1	1	1	1	1	1	1	1	1	1	1	1	1	1	0	1	1	1	1	1	1	1	1	1	1	1	1	1
72563	WHYDEDNPYKTWAYHGSYEV	NS5	2784	2803	HLA class I	81% - <100%	Dengue virus 1	ELISPOT	IFNg release	Positive	1	1	0	1	1	1	1	1	1	1	1	1	1	1	1	1	1	1	1	1	1	1	1	1	1	1	1	1	1
184003	KEISSMLNIM	C	86	95	HLA-B*40:01	51%-80%	Dengue virus 1	ELISPOT	IFNg release	Positive	1	1	1	1	1	0	0	1	0	0	1	1	1	1	0	1	0	1	1	1	1	1	1	1	1	1	1	1	1
186524	WTMKIGIGIL	E	733	742	HLA-B*08:01	51%-80%	Dengue virus 1	ELISPOT	IFNg release	Positive	1	1	1	0	0	0	1	0	1	1	1	1	1	1	0	1	1	1	1	1	1	1	1	1	1	1	0	0	1
184736	MAMVLSIVS	NS2a	1295	1303	HLA-B*35:01	51%-80%	Dengue virus 1	ELISPOT	IFNg release	Positive	0	0	0	1	1	1	0	1	1	0	1	0	1	1	1	1	0	1	0	0	0	0	1	1	1	1	1	1	0
419005	LLAVSGVYPM	NS2b	1448	1457	HLA-B*15:01	51%-80%	Dengue virus 1	ELISPOT	IFNg release	Positive	1	1	1	1	1	0	1	1	1	1	0	1	1	1	0	0	1	1	1	1	0	0	1	1	1	1	1	1	1
184849	MLLNRFTMA	NS5	24	32	HLA-B*08:01	51%-80%	Dengue virus 1	ELISPOT	IFNg release	Positive	1	1	1	0	0	1	1	0	1	1	0	0	0	0	1	0	1	0	1	1	1	1	0	0	1	0	0	0	1
186217	VEDERFWDL	NS5	2918	2926	HLA-B*40:01	51%-80%	Dengue virus 1	ELISPOT	IFNg release	Positive	1	0	1	1	1	0	0	1	1	0	1	1	1	1	0	0	0	1	1	1	1	1	1	1	1	1	1	1	0
419208	GVEGEGLHK	NS5	2998	3006	HLA-A*11:01	51%-80%	Dengue virus 1	ELISPOT	IFNg release	Positive-High	1	1	1	1	1	1	0	1	1	0	1	1	1	1	1	1	0	1	1	0	0	0	1	1	1	1	1	1	1
150255	GPMKLVMAFI	C	43	52	HLA-B*07:02	>0% - 50%	Dengue virus 1	ELISPOT	IFNg release	Positive	0	1	1	0	0	0	1	0	0	1	0	0	0	0	1	0	1	0	1	0	0	0	0	0	0	0	0	0	1
184835	MKLVMAFIAF	C	44	53	HLA-B*35:01	>0% - 50%	Dengue virus 1	ELISPOT	IFNg release	Positive	0	1	1	0	0	1	1	0	0	1	0	0	0	0	1	0	1	0	1	0	0	0	0	0	0	0	0	0	1
183245	FQSHQLWATL	NS2a	1267	1276	HLA-B*15:01	>0% - 50%	Dengue virus 1	ELISPOT	IFNg release	Positive	0	0	0	0	0	1	0	0	0	0	0	0	0	0	1	0	0	0	0	0	0	0	0	0	0	0	0	0	0
183252	FSLHYAWKTM	NS2a	1286	1295	HLA-B*35:01	>0% - 50%	Dengue virus 1	ELISPOT	IFNg release	Positive	0	0	0	0	0	1	0	0	0	0	0	0	0	0	0	0	0	0	0	0	0	0	0	0	0	0	0	0	0
180670	MPVTVASAAQ	NS3	1923	1932	HLA-B*35:01	>0% - 50%	Dengue virus 1	ELISPOT	IFNg release	Positive	0	1	0	0	0	1	1	0	0	1	0	0	0	0	0	0	1	0	1	0	0	0	0	0	0	0	0	0	1
184896	MPVTVASAA	NS3	1923	1931	HLA-B*35:01	>0% - 50%	Dengue virus 1	ELISPOT	IFNg release	Positive	0	1	0	0	0	1	1	0	0	1	0	0	0	0	0	0	1	0	1	0	0	0	0	0	0	0	0	0	1
419125	AVKSEHTGR	NS3	1722	1730	HLA-A*11:01	>0% - 50%	Dengue virus 1	ELISPOT	IFNg release	Positive	1	1	1	0	0	0	1	0	0	1	0	0	0	0	0	0	1	0	1	1	1	1	0	0	0	0	0	0	1
184554	LPQHLTQRAQ	NS4a	2107	2116	HLA-B*35:01	>0% - 50%	Dengue virus 1	ELISPOT	IFNg release	Positive	0	0	0	0	0	1	0	0	0	0	0	0	0	0	1	0	0	0	0	0	0	0	0	0	0	0	0	0	0
150579	SPGKFWNTTI	NS4b	2453	2462	HLA-B*07:02	>0% - 50%	Dengue virus 1	ELISPOT	IFNg release	Positive	0	1	1	0	0	1	1	0	0	1	0	0	0	0	0	0	1	0	1	1	1	1	0	0	0	0	0	0	1
150308	IPMATYGWNL	NS5	2607	2616	HLA-B*07:02	>0% - 50%	Dengue virus 1	ELISPOT	IFNg release	Positive	0	1	1	1	0	1	1	0	0	1	0	0	1	0	0	0	1	0	1	0	0	0	0	0	0	0	0	0	1
185485	RQMESEGIFL	NS5	3113	3122	HLA-B*15:01	>0% - 50%	Dengue virus 1	ELISPOT	IFNg release	Positive	0	0	0	0	0	0	0	0	0	0	0	0	0	0	0	0	0	0	1	0	0	0	0	0	0	0	0	0	0
5223	ATYGWNLVK	NS5	2610	2618	HLA-A*03:01	>0% - 50%	Dengue virus 1	ELISPOT	IFNg release	Positive	0	1	1	1	0	1	1	0	0	1	0	0	1	0	0	0	1	0	1	0	0	0	0	0	0	0	0	0	1
539435	ISGDDCVVKPIDDRFAT	NS5	3152	3168	HLA class I	>0% - 50%	Dengue virus 1	ICS	IFNg release	Positive	0	0	0	0	0	1	0	0	0	0	0	0	0	0	1	0	0	0	1	0	0	0	0	0	0	0	0	0	0
540100	VVKPIDDRFATALTALN	NS5	3157	3173	HLA class I	>0% - 50%	Dengue virus 1	ICS	IFNg release	Positive	0	0	0	0	0	1	0	0	0	0	0	0	0	0	1	0	0	0	1	0	0	0	0	0	0	0	0	0	0
67362	TYGWNLVKL	NS5	2611	2619	HLA-A*24:02	>0% - 50%	Dengue virus 1	ELISPOT	IFNg release	Positive	0	1	1	1	0	1	1	0	0	1	0	0	1	0	0	0	1	0	1	0	0	0	0	0	0	0	0	0	1

CD8 DENV-2 Epitopes

											NCBI IDs of DENV-2 circulating strains from India, (100% identity=1,green ; else 0, red)																																									
Epitope_ID	AA_Seq	Protein_Name	Start	End	Allele	step	Serotype	Method/Technique	Assay Group	Qualitative Measure	AFZ40227.1	AYJ72763.1	AYJ72772.1	AYJ72773.1	AYJ72774.1	AYJ72775.1	AYJ72776.1	AYJ72777.1	AYJ72778.1	AYJ72779.1	AYJ72780.1	AYJ72764.1	AYJ72765.1	AYJ72766.1	AYJ72767.1	AYJ72768.1	AYJ72769.1	AYJ72770.1	AYJ72771.1	ACQ44493.1	AFZ40121.1	ANC57597.1	QCZ24979.1	QCZ24978.1	QCZ24977.1	QCZ24976.1	QCZ24974.1	QCZ24973.1	QCZ24971.1	QCZ24970.1	QCZ24969.1	QCZ24966.1	QCZ24965.1	QBA57539.1	QBA57535.1	QBA57537.1	AFZ40226.1	AII99332.1	AYJ72757.1	AYJ72758.1	AYJ72759.1	AYJ72760.1	AYJ72761.1	AYJ72762.1	ARO52692.1	ARO52693.1	AFZ40225.1	AYE66920.1	AYE66919.1
185399	RKKARSTPF	C	5	13	HLA-B*08:01	0%	Dengue virus 2	ELISPOT	IFNg release	Positive	0	0	0	0	0	0	0	0	0	0	0	0	0	0	0	0	0	0	0	0	0	0	0	0	0	0	0	0	0	0	0	0	0	0	0	0	0	0	0	0	0	0	0	0	0	0	0	0	0
180818	WILRHPGFTMMAAIL	PreM	126	140	HLA class I	0%	Dengue virus 2	ICS	IFNg release	Positive	0	0	0	0	0	0	0	0	0	0	0	0	0	0	0	0	0	0	0	0	0	0	0	0	0	0	0	0	0	0	0	0	0	0	0	0	0	0	0	0	0	0	0	0	0	0	0	0	0
183577	HFQRALIFIL	PreM	262	271	HLA-B*08:01	0%	Dengue virus 2	ELISPOT	IFNg release	Positive	0	0	0	0	0	0	0	0	0	0	0	0	0	0	0	0	0	0	0	0	0	0	0	0	0	0	0	0	0	0	0	0	0	0	0	0	0	0	0	0	0	0	0	0	0	0	0	0	0
183621	HVQRIETWIL	PreM	233	242	HLA-B*08:01	0%	Dengue virus 2	ELISPOT	IFNg release	Positive	0	0	0	0	0	0	0	0	0	0	0	0	0	0	0	0	0	0	0	0	0	0	0	0	0	0	0	0	0	0	0	0	0	0	0	0	0	0	0	0	0	0	0	0	0	0	0	0	0
186264	VGRQEKGKSL	PreM	128	137	HLA-B*08:01	0%	Dengue virus 2	ELISPOT	IFNg release	Positive	0	0	0	0	0	0	0	0	0	0	0	0	0	0	0	0	0	0	0	0	0	0	0	0	0	0	0	0	0	0	0	0	0	0	0	0	0	0	0	0	0	0	0	0	0	0	0	0	0
180563	KILIGVIITWIGMNS	E	456	470	HLA class I	0%	Dengue virus 2	ICS	IFNg release	Positive	0	0	0	0	0	0	0	0	0	0	0	0	0	0	0	0	0	0	0	0	0	0	0	0	0	0	0	0	0	0	0	0	0	0	0	0	0	0	0	0	0	0	0	0	0	0	0	0	0
183747	ILIGVIITW	E	737	745	HLA-B*58:01	0%	Dengue virus 2	ELISPOT	IFNg release	Positive	0	0	0	0	0	0	0	0	0	0	0	0	0	0	0	0	0	0	0	0	0	0	0	0	0	0	0	0	0	0	0	0	0	0	0	0	0	0	0	0	0	0	0	0	0	0	0	0	0
184300	LATLRKYCI	E	53	61	HLA-B*08:01	0%	Dengue virus 2	ELISPOT	IFNg release	Positive	0	0	0	0	0	0	0	0	0	0	0	0	0	0	0	0	0	0	0	0	0	0	0	0	0	0	0	0	0	0	0	0	0	0	0	0	0	0	0	0	0	0	0	0	0	0	0	0	0
180496	GVFTTNIWLKLKEKQ	NS1	936	950	HLA class I	0%	Dengue virus 2	ICS	IFNg release	Positive	0	0	0	0	0	0	0	0	0	0	0	0	0	0	0	0	0	0	0	0	0	0	0	0	0	0	0	0	0	0	0	0	0	0	0	0	0	0	0	0	0	0	0	0	0	0	0	0	0
150448	MMATIGIAL	NS2a	1229	1237	HLA-B*35:01	0%	Dengue virus 2	ELISPOT	IFNg release	Positive	0	0	0	0	0	0	0	0	0	0	0	0	0	0	0	0	0	0	0	0	0	0	0	0	0	0	0	0	0	0	0	0	0	0	0	0	0	0	0	0	0	0	0	0	0	0	0	0	0
150449	MMATIGIALL	NS2a	1229	1238	HLA-A*02:01	0%	Dengue virus 2	ELISPOT	IFNg release	Positive	0	0	0	0	0	0	0	0	0	0	0	0	0	0	0	0	0	0	0	0	0	0	0	0	0	0	0	0	0	0	0	0	0	0	0	0	0	0	0	0	0	0	0	0	0	0	0	0	0
182687	AYRIKQRGIL	NS3	1497	1506	HLA-B*08:01	0%	Dengue virus 2	ELISPOT	IFNg release	Positive	0	0	0	0	0	0	0	0	0	0	0	0	0	0	0	0	0	0	0	0	0	0	0	0	0	0	0	0	0	0	0	0	0	0	0	0	0	0	0	0	0	0	0	0	0	0	0	0	0
182892	EENMEVEIW	NS3	2049	2057	HLA-B*44:02	0%	Dengue virus 2	ELISPOT	IFNg release	Positive	0	0	0	0	0	0	0	0	0	0	0	0	0	0	0	0	0	0	0	0	0	0	0	0	0	0	0	0	0	0	0	0	0	0	0	0	0	0	0	0	0	0	0	0	0	0	0	0	0
184318	LEENMEVEIW	NS3	2048	2057	HLA-B*44:02	0%	Dengue virus 2	ELISPOT	IFNg release	Positive	0	0	0	0	0	0	0	0	0	0	0	0	0	0	0	0	0	0	0	0	0	0	0	0	0	0	0	0	0	0	0	0	0	0	0	0	0	0	0	0	0	0	0	0	0	0	0	0	0
186211	VDRKGKVVGL	NS3	1615	1624	HLA-B*08:01	0%	Dengue virus 2	ELISPOT	IFNg release	Positive	0	0	0	0	0	0	0	0	0	0	0	0	0	0	0	0	0	0	0	0	0	0	0	0	0	0	0	0	0	0	0	0	0	0	0	0	0	0	0	0	0	0	0	0	0	0	0	0	0
186301	VLMHRGKRI	NS3	1532	1540	HLA-B*08:01	0%	Dengue virus 2	ELISPOT	IFNg release	Positive	0	0	0	0	0	0	0	0	0	0	0	0	0	0	0	0	0	0	0	0	0	0	0	0	0	0	0	0	0	0	0	0	0	0	0	0	0	0	0	0	0	0	0	0	0	0	0	0	0
22816	GTSGSPIADK	NS3	1608	1617	HLA-A*11:01	0%	Dengue virus 2	multimer/tetramer	qualitative binding	Positive	0	0	0	0	0	0	0	0	0	0	0	0	0	0	0	0	0	0	0	0	0	0	0	0	0	0	0	0	0	0	0	0	0	0	0	0	0	0	0	0	0	0	0	0	0	0	0	0	0
22817	GTSGSPIADKK	NS3	1608	1618	HLA-A11	0%	Dengue virus 2	ELISPOT	IFNg release	Positive	0	0	0	0	0	0	0	0	0	0	0	0	0	0	0	0	0	0	0	0	0	0	0	0	0	0	0	0	0	0	0	0	0	0	0	0	0	0	0	0	0	0	0	0	0	0	0	0	0
22824	GTSGSPIVDR	NS3	1608	1617	HLA-A*11:01	0%	Dengue virus 2	51 chromium	cytotoxicity	Positive	0	0	0	0	0	0	0	0	0	0	0	0	0	0	0	0	0	0	0	0	0	0	0	0	0	0	0	0	0	0	0	0	0	0	0	0	0	0	0	0	0	0	0	0	0	0	0	0	0
46575	NYADRKWCF	NS3	2031	2039	HLA-A24	0%	Dengue virus 2	51 chromium	cytotoxicity	Positive	0	0	0	0	0	0	0	0	0	0	0	0	0	0	0	0	0	0	0	0	0	0	0	0	0	0	0	0	0	0	0	0	0	0	0	0	0	0	0	0	0	0	0	0	0	0	0	0	0
65548	TPEGIIPSL	NS3	1975	1983	HLA-B35	0%	Dengue virus 2	51 chromium	cytotoxicity	Positive	0	0	0	0	0	0	0	0	0	0	0	0	0	0	0	0	0	0	0	0	0	0	0	0	0	0	0	0	0	0	0	0	0	0	0	0	0	0	0	0	0	0	0	0	0	0	0	0	0
150373	LLILCVTQV	NS4b	2416	2424	HLA-A*02:01	0%	Dengue virus 2	ELISPOT	IFNg release	Positive	0	0	0	0	0	0	0	0	0	0	0	0	0	0	0	0	0	0	0	0	0	0	0	0	0	0	0	0	0	0	0	0	0	0	0	0	0	0	0	0	0	0	0	0	0	0	0	0	0
150445	MLLILCVTQV	NS4b	2415	2424	HLA-A*02:01	0%	Dengue virus 2	ELISPOT	IFNg release	Positive	0	0	0	0	0	0	0	0	0	0	0	0	0	0	0	0	0	0	0	0	0	0	0	0	0	0	0	0	0	0	0	0	0	0	0	0	0	0	0	0	0	0	0	0	0	0	0	0	0
150639	TLYAVATTFV	NS4b	2282	2291	HLA-A*02:01	0%	Dengue virus 2	ELISPOT	IFNg release	Positive	0	0	0	0	0	0	0	0	0	0	0	0	0	0	0	0	0	0	0	0	0	0	0	0	0	0	0	0	0	0	0	0	0	0	0	0	0	0	0	0	0	0	0	0	0	0	0	0	0
180601	LILCVTQVLM	NS4b	2417	2426	HLA-B*35:01	0%	Dengue virus 2	ELISPOT	IFNg release	Positive	0	0	0	0	0	0	0	0	0	0	0	0	0	0	0	0	0	0	0	0	0	0	0	0	0	0	0	0	0	0	0	0	0	0	0	0	0	0	0	0	0	0	0	0	0	0	0	0	0
180794	VATTFVTPM	NS4b	2286	2294	HLA-B*35:01	0%	Dengue virus 2	ELISPOT	IFNg release	Positive	0	0	0	0	0	0	0	0	0	0	0	0	0	0	0	0	0	0	0	0	0	0	0	0	0	0	0	0	0	0	0	0	0	0	0	0	0	0	0	0	0	0	0	0	0	0	0	0	0
184439	LLILCVTQVL	NS4b	2416	2425	HLA-B*08:01	0%	Dengue virus 2	ELISPOT	IFNg release	Positive	0	0	0	0	0	0	0	0	0	0	0	0	0	0	0	0	0	0	0	0	0	0	0	0	0	0	0	0	0	0	0	0	0	0	0	0	0	0	0	0	0	0	0	0	0	0	0	0	0
150698	VPYLGKREDQ	NS5	3327	3336	HLA-B*07:02	0%	Dengue virus 2	ELISPOT	IFNg release	Positive	0	0	0	0	0	0	0	0	0	0	0	0	0	0	0	0	0	0	0	0	0	0	0	0	0	0	0	0	0	0	0	0	0	0	0	0	0	0	0	0	0	0	0	0	0	0	0	0	0
163620	TITEEIAVQ	NS5	3127	3135	HLA-A*02:01	0%	Dengue virus 2	ELISPOT	IFNg release	Positive	0	0	0	0	0	0	0	0	0	0	0	0	0	0	0	0	0	0	0	0	0	0	0	0	0	0	0	0	0	0	0	0	0	0	0	0	0	0	0	0	0	0	0	0	0	0	0	0	0
184234	KWKSRLNAL	NS5	2503	2511	HLA-B*08:01	0%	Dengue virus 2	ELISPOT	IFNg release	Positive	0	0	0	0	0	0	0	0	0	0	0	0	0	0	0	0	0	0	0	0	0	0	0	0	0	0	0	0	0	0	0	0	0	0	0	0	0	0	0	0	0	0	0	0	0	0	0	0	0
32939	KPWDIIPMV	NS5	2821	2829	HLA-B*55:02	0%	Dengue virus 2	51 chromium	cytotoxicity	Positive	0	0	0	0	0	0	0	0	0	0	0	0	0	0	0	0	0	0	0	0	0	0	0	0	0	0	0	0	0	0	0	0	0	0	0	0	0	0	0	0	0	0	0	0	0	0	0	0	0
32941	KPWDVLPMV	NS5	2821	2829	HLA class I	0%	Dengue virus 2	51 chromium	cytotoxicity	Positive	0	0	0	0	0	0	0	0	0	0	0	0	0	0	0	0	0	0	0	0	0	0	0	0	0	0	0	0	0	0	0	0	0	0	0	0	0	0	0	0	0	0	0	0	0	0	0	0	0
32942	KPWDVLPTV	NS5	2821	2829	HLA class I	0%	Dengue virus 2	51 chromium	cytotoxicity	Positive	0	0	0	0	0	0	0	0	0	0	0	0	0	0	0	0	0	0	0	0	0	0	0	0	0	0	0	0	0	0	0	0	0	0	0	0	0	0	0	0	0	0	0	0	0	0	0	0	0
150252	GPLKLFMAL	C	42	50	HLA-B*08:01	100%	Dengue virus 2	ELISPOT	IFNg release	Positive	1	1	1	1	1	1	1	1	1	1	1	1	1	1	1	1	1	1	1	1	1	1	1	1	1	1	1	1	1	1	1	1	1	1	1	1	1	1	1	1	1	1	1	1	1	1	1	1	1
150634	TLMAMDLGEL	PreM	149	158	HLA-A*02:01	100%	Dengue virus 2	ELISPOT	IFNg release	Positive	1	1	1	1	1	1	1	1	1	1	1	1	1	1	1	1	1	1	1	1	1	1	1	1	1	1	1	1	1	1	1	1	1	1	1	1	1	1	1	1	1	1	1	1	1	1	1	1	1
150456	MSYSMCTGKF	E	577	586	HLA-B*35:01	100%	Dengue virus 2	ELISPOT	IFNg release	Positive	1	1	1	1	1	1	1	1	1	1	1	1	1	1	1	1	1	1	1	1	1	1	1	1	1	1	1	1	1	1	1	1	1	1	1	1	1	1	1	1	1	1	1	1	1	1	1	1	1
150531	RLRMDKLQL	E	566	574	HLA-B*08:01	100%	Dengue virus 2	ELISPOT	IFNg release	Positive	1	1	1	1	1	1	1	1	1	1	1	1	1	1	1	1	1	1	1	1	1	1	1	1	1	1	1	1	1	1	1	1	1	1	1	1	1	1	1	1	1	1	1	1	1	1	1	1	1
16553	FLDLPLPWL	E	213	221	HLA-A2	100%	Dengue virus 2	ICS	TNF release	Positive	1	1	1	1	1	1	1	1	1	1	1	1	1	1	1	1	1	1	1	1	1	1	1	1	1	1	1	1	1	1	1	1	1	1	1	1	1	1	1	1	1	1	1	1	1	1	1	1	1
180618	LPLPWLPGAD	E	496	505	HLA-B*35:01	100%	Dengue virus 2	ELISPOT	IFNg release	Positive	1	1	1	1	1	1	1	1	1	1	1	1	1	1	1	1	1	1	1	1	1	1	1	1	1	1	1	1	1	1	1	1	1	1	1	1	1	1	1	1	1	1	1	1	1	1	1	1	1
180726	RMAILGDTAWDFGSL	E	691	705	HLA-B7	100%	Dengue virus 2	ICS	IFNg release	Positive	1	1	1	1	1	1	1	1	1	1	1	1	1	1	1	1	1	1	1	1	1	1	1	1	1	1	1	1	1	1	1	1	1	1	1	1	1	1	1	1	1	1	1	1	1	1	1	1	1
180816	WGNGCGLFGKGGIVT	E	381	395	HLA class I	100%	Dengue virus 2	ICS	IFNg release	Positive	1	1	1	1	1	1	1	1	1	1	1	1	1	1	1	1	1	1	1	1	1	1	1	1	1	1	1	1	1	1	1	1	1	1	1	1	1	1	1	1	1	1	1	1	1	1	1	1	1
182846	EAKQPATLR	E	329	337	HLA-A*68:01	100%	Dengue virus 2	ELISPOT	IFNg release	Positive	1	1	1	1	1	1	1	1	1	1	1	1	1	1	1	1	1	1	1	1	1	1	1	1	1	1	1	1	1	1	1	1	1	1	1	1	1	1	1	1	1	1	1	1	1	1	1	1	1
183437	GMNSRSTSL	E	747	755	HLA-B*08:01	100%	Dengue virus 2	ELISPOT	IFNg release	Positive	1	1	1	1	1	1	1	1	1	1	1	1	1	1	1	1	1	1	1	1	1	1	1	1	1	1	1	1	1	1	1	1	1	1	1	1	1	1	1	1	1	1	1	1	1	1	1	1	1
183708	IGMNSRSTSL	E	746	755	HLA-B*08:01	100%	Dengue virus 2	ELISPOT	IFNg release	Positive	1	1	1	1	1	1	1	1	1	1	1	1	1	1	1	1	1	1	1	1	1	1	1	1	1	1	1	1	1	1	1	1	1	1	1	1	1	1	1	1	1	1	1	1	1	1	1	1	1
183841	IQMSSGNLLF	E	550	559	HLA-A*23:01	100%	Dengue virus 2	ELISPOT	IFNg release	Positive	1	1	1	1	1	1	1	1	1	1	1	1	1	1	1	1	1	1	1	1	1	1	1	1	1	1	1	1	1	1	1	1	1	1	1	1	1	1	1	1	1	1	1	1	1	1	1	1	1
184571	LQLKGMSYSM	E	572	581	HLA-B*08:01	100%	Dengue virus 2	ELISPOT	IFNg release	Positive	1	1	1	1	1	1	1	1	1	1	1	1	1	1	1	1	1	1	1	1	1	1	1	1	1	1	1	1	1	1	1	1	1	1	1	1	1	1	1	1	1	1	1	1	1	1	1	1	1
184899	MRGAKRMAI	E	686	694	HLA-B*08:01	100%	Dengue virus 2	ELISPOT	IFNg release	Positive	1	1	1	1	1	1	1	1	1	1	1	1	1	1	1	1	1	1	1	1	1	1	1	1	1	1	1	1	1	1	1	1	1	1	1	1	1	1	1	1	1	1	1	1	1	1	1	1	1
184900	MRGAKRMAIL	E	686	695	HLA-B*08:01	100%	Dengue virus 2	ELISPOT	IFNg release	Positive	1	1	1	1	1	1	1	1	1	1	1	1	1	1	1	1	1	1	1	1	1	1	1	1	1	1	1	1	1	1	1	1	1	1	1	1	1	1	1	1	1	1	1	1	1	1	1	1	1
184920	MSSGNLLFTG	E	552	561	HLA-B*58:01	100%	Dengue virus 2	ELISPOT	IFNg release	Positive	1	1	1	1	1	1	1	1	1	1	1	1	1	1	1	1	1	1	1	1	1	1	1	1	1	1	1	1	1	1	1	1	1	1	1	1	1	1	1	1	1	1	1	1	1	1	1	1	1
185758	SRSTSLSVSL	E	750	759	HLA-B*08:01	100%	Dengue virus 2	ELISPOT	IFNg release	Positive	1	1	1	1	1	1	1	1	1	1	1	1	1	1	1	1	1	1	1	1	1	1	1	1	1	1	1	1	1	1	1	1	1	1	1	1	1	1	1	1	1	1	1	1	1	1	1	1	1
185831	SYSMCTGKF	E	578	586	HLA-A*23:01	100%	Dengue virus 2	ELISPOT	IFNg release	Positive	1	1	1	1	1	1	1	1	1	1	1	1	1	1	1	1	1	1	1	1	1	1	1	1	1	1	1	1	1	1	1	1	1	1	1	1	1	1	1	1	1	1	1	1	1	1	1	1	1
27061	ILGDTAWDFG	E	96	105	HLA class I	100%	Dengue virus 2	ELISPOT	IFNg release	Positive	1	1	1	1	1	1	1	1	1	1	1	1	1	1	1	1	1	1	1	1	1	1	1	1	1	1	1	1	1	1	1	1	1	1	1	1	1	1	1	1	1	1	1	1	1	1	1	1	1
51388	QLKGMSYSM	E	573	581	HLA-B*08:01	100%	Dengue virus 2	ELISPOT	IFNg release	Positive	1	1	1	1	1	1	1	1	1	1	1	1	1	1	1	1	1	1	1	1	1	1	1	1	1	1	1	1	1	1	1	1	1	1	1	1	1	1	1	1	1	1	1	1	1	1	1	1	1
583702	GVSGGSWVDIVLEHGSCV	E	14	31	HLA class I	100%	Dengue virus 2	ICS	IFNg release	Positive-Low	1	1	1	1	1	1	1	1	1	1	1	1	1	1	1	1	1	1	1	1	1	1	1	1	1	1	1	1	1	1	1	1	1	1	1	1	1	1	1	1	1	1	1	1	1	1	1	1	1
150259	GPWHLGKLEM	NS1	1041	1050	HLA-B*35:01	100%	Dengue virus 2	ELISPOT	IFNg release	Positive	1	1	1	1	1	1	1	1	1	1	1	1	1	1	1	1	1	1	1	1	1	1	1	1	1	1	1	1	1	1	1	1	1	1	1	1	1	1	1	1	1	1	1	1	1	1	1	1	1
184512	LMWKQITPEL	NS1	841	850	HLA-B*08:01	100%	Dengue virus 2	ELISPOT	IFNg release	Positive	1	1	1	1	1	1	1	1	1	1	1	1	1	1	1	1	1	1	1	1	1	1	1	1	1	1	1	1	1	1	1	1	1	1	1	1	1	1	1	1	1	1	1	1	1	1	1	1	1
185465	RPLKEKEENL	NS1	1111	1120	HLA-B*08:01	100%	Dengue virus 2	ELISPOT	IFNg release	Positive	1	1	1	1	1	1	1	1	1	1	1	1	1	1	1	1	1	1	1	1	1	1	1	1	1	1	1	1	1	1	1	1	1	1	1	1	1	1	1	1	1	1	1	1	1	1	1	1	1
186474	WKTWGKAKML	NS1	890	899	HLA-B*08:01	100%	Dengue virus 2	ELISPOT	IFNg release	Positive	1	1	1	1	1	1	1	1	1	1	1	1	1	1	1	1	1	1	1	1	1	1	1	1	1	1	1	1	1	1	1	1	1	1	1	1	1	1	1	1	1	1	1	1	1	1	1	1	1
19225	GEDGCWYGM	NS1	1100	1108	HLA-B*40:01	100%	Dengue virus 2	ELISPOT	IFNg release	Positive	1	1	1	1	1	1	1	1	1	1	1	1	1	1	1	1	1	1	1	1	1	1	1	1	1	1	1	1	1	1	1	1	1	1	1	1	1	1	1	1	1	1	1	1	1	1	1	1	1
150415	LTDALALGM	NS2a	1250	1258	HLA-A*01:01	100%	Dengue virus 2	ELISPOT	IFNg release	Positive	1	1	1	1	1	1	1	1	1	1	1	1	1	1	1	1	1	1	1	1	1	1	1	1	1	1	1	1	1	1	1	1	1	1	1	1	1	1	1	1	1	1	1	1	1	1	1	1	1
180671	MSFRDLGRVM	NS2a	1175	1184	HLA-B*35:01	100%	Dengue virus 2	ELISPOT	IFNg release	Positive	1	1	1	1	1	1	1	1	1	1	1	1	1	1	1	1	1	1	1	1	1	1	1	1	1	1	1	1	1	1	1	1	1	1	1	1	1	1	1	1	1	1	1	1	1	1	1	1	1
150304	IMAVGMVSI	NS2b	1353	1361	HLA-A*02:01	100%	Dengue virus 2	ELISPOT	IFNg release	Positive	1	1	1	1	1	1	1	1	1	1	1	1	1	1	1	1	1	1	1	1	1	1	1	1	1	1	1	1	1	1	1	1	1	1	1	1	1	1	1	1	1	1	1	1	1	1	1	1	1
180531	IPMTGPLVAG	NS2b	1371	1380	HLA-B*35:01	100%	Dengue virus 2	ELISPOT	IFNg release	Positive	1	1	1	1	1	1	1	1	1	1	1	1	1	1	1	1	1	1	1	1	1	1	1	1	1	1	1	1	1	1	1	1	1	1	1	1	1	1	1	1	1	1	1	1	1	1	1	1	1
180646	MAVGMVSIL	NS2b	1354	1362	HLA-B*35:01	100%	Dengue virus 2	ELISPOT	IFNg release	Positive	1	1	1	1	1	1	1	1	1	1	1	1	1	1	1	1	1	1	1	1	1	1	1	1	1	1	1	1	1	1	1	1	1	1	1	1	1	1	1	1	1	1	1	1	1	1	1	1	1
183779	IMAVGMVSIL	NS2b	1353	1362	HLA-B*08:01	100%	Dengue virus 2	ELISPOT	IFNg release	Positive	1	1	1	1	1	1	1	1	1	1	1	1	1	1	1	1	1	1	1	1	1	1	1	1	1	1	1	1	1	1	1	1	1	1	1	1	1	1	1	1	1	1	1	1	1	1	1	1	1
11125	EALRGLPIR	NS3	233	241	HLA class I	100%	Dengue virus 2	ELISPOT	IFNg release	Positive	1	1	1	1	1	1	1	1	1	1	1	1	1	1	1	1	1	1	1	1	1	1	1	1	1	1	1	1	1	1	1	1	1	1	1	1	1	1	1	1	1	1	1	1	1	1	1	1	1
13358	EMEEALRGLPIRYQT	NS3	230	244	HLA class I	100%	Dengue virus 2	ELISPOT	IFNg release	Positive	1	1	1	1	1	1	1	1	1	1	1	1	1	1	1	1	1	1	1	1	1	1	1	1	1	1	1	1	1	1	1	1	1	1	1	1	1	1	1	1	1	1	1	1	1	1	1	1	1
136890	TPPGSRDPF	NS3	1792	1800	HLA-B*35:01	100%	Dengue virus 2	ELISPOT	IFNg release	Positive	1	1	1	1	1	1	1	1	1	1	1	1	1	1	1	1	1	1	1	1	1	1	1	1	1	1	1	1	1	1	1	1	1	1	1	1	1	1	1	1	1	1	1	1	1	1	1	1	1
150137	APTRVVAAEM	NS3	222	231	HLA-B7	100%	Dengue virus 2	multimer/tetramer	qualitative binding	Positive	1	1	1	1	1	1	1	1	1	1	1	1	1	1	1	1	1	1	1	1	1	1	1	1	1	1	1	1	1	1	1	1	1	1	1	1	1	1	1	1	1	1	1	1	1	1	1	1	1
150217	FPQSNAPIM	NS3	1800	1808	HLA-B*35:01	100%	Dengue virus 2	ELISPOT	IFNg release	Positive	1	1	1	1	1	1	1	1	1	1	1	1	1	1	1	1	1	1	1	1	1	1	1	1	1	1	1	1	1	1	1	1	1	1	1	1	1	1	1	1	1	1	1	1	1	1	1	1	1
150526	RIYSDPLALK	NS3	2074	2083	HLA-A*11:01	100%	Dengue virus 2	ELISPOT	IFNg release	Positive	1	1	1	1	1	1	1	1	1	1	1	1	1	1	1	1	1	1	1	1	1	1	1	1	1	1	1	1	1	1	1	1	1	1	1	1	1	1	1	1	1	1	1	1	1	1	1	1	1
150595	SQIGAGVYK	NS3	1509	1517	HLA-A*11:01	100%	Dengue virus 2	ELISPOT	IFNg release	Positive	1	1	1	1	1	1	1	1	1	1	1	1	1	1	1	1	1	1	1	1	1	1	1	1	1	1	1	1	1	1	1	1	1	1	1	1	1	1	1	1	1	1	1	1	1	1	1	1	1
150644	TPEGIIPSMF	NS3	1975	1984	HLA-B*53:01	100%	Dengue virus 2	ELISPOT	IFNg release	Positive	1	1	1	1	1	1	1	1	1	1	1	1	1	1	1	1	1	1	1	1	1	1	1	1	1	1	1	1	1	1	1	1	1	1	1	1	1	1	1	1	1	1	1	1	1	1	1	1	1
150693	VPNYNLIIM	NS3	1750	1758	HLA-B*07:02	100%	Dengue virus 2	ELISPOT	IFNg release	Positive	1	1	1	1	1	1	1	1	1	1	1	1	1	1	1	1	1	1	1	1	1	1	1	1	1	1	1	1	1	1	1	1	1	1	1	1	1	1	1	1	1	1	1	1	1	1	1	1	1
150740	YLPAIVREA	NS3	1678	1686	HLA-A*02:01	100%	Dengue virus 2	ELISPOT	IFNg release	Positive	1	1	1	1	1	1	1	1	1	1	1	1	1	1	1	1	1	1	1	1	1	1	1	1	1	1	1	1	1	1	1	1	1	1	1	1	1	1	1	1	1	1	1	1	1	1	1	1	1
180360	AAEGINYADRRWCFD	NS3	2026	2040	HLA-A24	100%	Dengue virus 2	ELISPOT	IFNg release	Positive	1	1	1	1	1	1	1	1	1	1	1	1	1	1	1	1	1	1	1	1	1	1	1	1	1	1	1	1	1	1	1	1	1	1	1	1	1	1	1	1	1	1	1	1	1	1	1	1	1
180407	DNINTPEGIIPSMFE	NS3	496	510	HLA-B35	100%	Dengue virus 2	ELISPOT	IFNg release	Positive	1	1	1	1	1	1	1	1	1	1	1	1	1	1	1	1	1	1	1	1	1	1	1	1	1	1	1	1	1	1	1	1	1	1	1	1	1	1	1	1	1	1	1	1	1	1	1	1	1
180428	EGEWKEGEEVQVLAL	NS3	1561	1575	HLA class I	100%	Dengue virus 2	ICS	IFNg release	Positive	1	1	1	1	1	1	1	1	1	1	1	1	1	1	1	1	1	1	1	1	1	1	1	1	1	1	1	1	1	1	1	1	1	1	1	1	1	1	1	1	1	1	1	1	1	1	1	1	1
180440	EREIPERSWNSGHEW	NS3	336	350	HLA-B*57:01	100%	Dengue virus 2	ELISPOT	IFNg release	Positive	1	1	1	1	1	1	1	1	1	1	1	1	1	1	1	1	1	1	1	1	1	1	1	1	1	1	1	1	1	1	1	1	1	1	1	1	1	1	1	1	1	1	1	1	1	1	1	1	1
180460	FPQSNAPIMD	NS3	1800	1809	HLA-B*35:01	100%	Dengue virus 2	ELISPOT	IFNg release	Positive	1	1	1	1	1	1	1	1	1	1	1	1	1	1	1	1	1	1	1	1	1	1	1	1	1	1	1	1	1	1	1	1	1	1	1	1	1	1	1	1	1	1	1	1	1	1	1	1	1
180503	HPGAGKTKRY	NS3	1669	1678	HLA-B*35:01	100%	Dengue virus 2	ELISPOT	IFNg release	Positive	1	1	1	1	1	1	1	1	1	1	1	1	1	1	1	1	1	1	1	1	1	1	1	1	1	1	1	1	1	1	1	1	1	1	1	1	1	1	1	1	1	1	1	1	1	1	1	1	1
180507	HTGREIVDLMCHATF	NS3	1726	1740	HLA class I	100%	Dengue virus 2	ELISPOT	IFNg release	Positive	1	1	1	1	1	1	1	1	1	1	1	1	1	1	1	1	1	1	1	1	1	1	1	1	1	1	1	1	1	1	1	1	1	1	1	1	1	1	1	1	1	1	1	1	1	1	1	1	1
180575	KRYLPAIVREAIKRG	NS3	201	215	HLA-A*31:01	100%	Dengue virus 2	ELISPOT	IFNg release	Positive	1	1	1	1	1	1	1	1	1	1	1	1	1	1	1	1	1	1	1	1	1	1	1	1	1	1	1	1	1	1	1	1	1	1	1	1	1	1	1	1	1	1	1	1	1	1	1	1	1
180589	LDARIYSDPLALKEF	NS3	596	610	HLA class I	100%	Dengue virus 2	ELISPOT	IFNg release	Positive	1	1	1	1	1	1	1	1	1	1	1	1	1	1	1	1	1	1	1	1	1	1	1	1	1	1	1	1	1	1	1	1	1	1	1	1	1	1	1	1	1	1	1	1	1	1	1	1	1
180605	LMCHATFTM	NS3	1734	1742	HLA-B*15:01	100%	Dengue virus 2	ELISPOT	IFNg release	Positive	1	1	1	1	1	1	1	1	1	1	1	1	1	1	1	1	1	1	1	1	1	1	1	1	1	1	1	1	1	1	1	1	1	1	1	1	1	1	1	1	1	1	1	1	1	1	1	1	1
180617	LPIRYQTPAI	NS3	1713	1722	HLA-B*35:01	100%	Dengue virus 2	ELISPOT	IFNg release	Positive	1	1	1	1	1	1	1	1	1	1	1	1	1	1	1	1	1	1	1	1	1	1	1	1	1	1	1	1	1	1	1	1	1	1	1	1	1	1	1	1	1	1	1	1	1	1	1	1	1
180647	MEEALRGLPIRYQTP	NS3	1706	1720	HLA class I	100%	Dengue virus 2	ELISPOT	IFNg release	Positive	1	1	1	1	1	1	1	1	1	1	1	1	1	1	1	1	1	1	1	1	1	1	1	1	1	1	1	1	1	1	1	1	1	1	1	1	1	1	1	1	1	1	1	1	1	1	1	1	1
180734	RSWNSGHEW	NS3	342	350	HLA-B*58:01	100%	Dengue virus 2	ICS	IFNg release	Positive	1	1	1	1	1	1	1	1	1	1	1	1	1	1	1	1	1	1	1	1	1	1	1	1	1	1	1	1	1	1	1	1	1	1	1	1	1	1	1	1	1	1	1	1	1	1	1	1	1
182898	EEREIPERSW	NS3	1810	1819	HLA-B*44:03	100%	Dengue virus 2	ELISPOT	IFNg release	Positive	1	1	1	1	1	1	1	1	1	1	1	1	1	1	1	1	1	1	1	1	1	1	1	1	1	1	1	1	1	1	1	1	1	1	1	1	1	1	1	1	1	1	1	1	1	1	1	1	1
183724	IKRGLRTLIL	NS3	1687	1696	HLA-B*08:01	100%	Dengue virus 2	ELISPOT	IFNg release	Positive	1	1	1	1	1	1	1	1	1	1	1	1	1	1	1	1	1	1	1	1	1	1	1	1	1	1	1	1	1	1	1	1	1	1	1	1	1	1	1	1	1	1	1	1	1	1	1	1	1
185200	PRRCMKPVIL	NS3	1900	1909	HLA-B*08:01	100%	Dengue virus 2	ELISPOT	IFNg release	Positive	1	1	1	1	1	1	1	1	1	1	1	1	1	1	1	1	1	1	1	1	1	1	1	1	1	1	1	1	1	1	1	1	1	1	1	1	1	1	1	1	1	1	1	1	1	1	1	1	1
26965	ILAPTRVVAAEMEEA	NS3	220	234	HLA class I	100%	Dengue virus 2	ELISPOT	IFNg release	Positive	1	1	1	1	1	1	1	1	1	1	1	1	1	1	1	1	1	1	1	1	1	1	1	1	1	1	1	1	1	1	1	1	1	1	1	1	1	1	1	1	1	1	1	1	1	1	1	1	1
27770	INYADRRWCF	NS3	555	564	HLA-A24	100%	Dengue virus 2	ELISPOT	IFNg release	Positive	1	1	1	1	1	1	1	1	1	1	1	1	1	1	1	1	1	1	1	1	1	1	1	1	1	1	1	1	1	1	1	1	1	1	1	1	1	1	1	1	1	1	1	1	1	1	1	1	1
2859	ALRGLPIRY	NS3	234	242	HLA-A*03:01	100%	Dengue virus 2	51 chromium	cytotoxicity	Positive	1	1	1	1	1	1	1	1	1	1	1	1	1	1	1	1	1	1	1	1	1	1	1	1	1	1	1	1	1	1	1	1	1	1	1	1	1	1	1	1	1	1	1	1	1	1	1	1	1
35238	LDNINTPEGIIPSMF	NS3	495	509	HLA class I	100%	Dengue virus 2	ELISPOT	IFNg release	Positive	1	1	1	1	1	1	1	1	1	1	1	1	1	1	1	1	1	1	1	1	1	1	1	1	1	1	1	1	1	1	1	1	1	1	1	1	1	1	1	1	1	1	1	1	1	1	1	1	1
46576	NYADRRWCF	NS3	556	564	HLA-A24	100%	Dengue virus 2	ELISPOT	IFNg release	Positive	1	1	1	1	1	1	1	1	1	1	1	1	1	1	1	1	1	1	1	1	1	1	1	1	1	1	1	1	1	1	1	1	1	1	1	1	1	1	1	1	1	1	1	1	1	1	1	1	1
539620	LRTLILAPTRVVAAEM	NS3	1691	1706	HLA class I	100%	Dengue virus 2	ICS	IFNg release	Positive	1	1	1	1	1	1	1	1	1	1	1	1	1	1	1	1	1	1	1	1	1	1	1	1	1	1	1	1	1	1	1	1	1	1	1	1	1	1	1	1	1	1	1	1	1	1	1	1	1
539751	NYNLIIMDEAHFTDPA	NS3	1752	1767	HLA class I	100%	Dengue virus 2	ICS	IFNg release	Positive	1	1	1	1	1	1	1	1	1	1	1	1	1	1	1	1	1	1	1	1	1	1	1	1	1	1	1	1	1	1	1	1	1	1	1	1	1	1	1	1	1	1	1	1	1	1	1	1	1
539752	NYNLIIMDEAHFTDPASI	NS3	1752	1769	HLA class I	100%	Dengue virus 2	ICS	IFNg release	Positive	1	1	1	1	1	1	1	1	1	1	1	1	1	1	1	1	1	1	1	1	1	1	1	1	1	1	1	1	1	1	1	1	1	1	1	1	1	1	1	1	1	1	1	1	1	1	1	1	1
539985	TLILAPTRVVAAEMEEA	NS3	1693	1709	HLA class I	100%	Dengue virus 2	ICS	IFNg release	Positive	1	1	1	1	1	1	1	1	1	1	1	1	1	1	1	1	1	1	1	1	1	1	1	1	1	1	1	1	1	1	1	1	1	1	1	1	1	1	1	1	1	1	1	1	1	1	1	1	1
5542	AVSLDFSPGTSGSPI	NS3	125	139	HLA class I	100%	Dengue virus 2	ELISPOT	IFNg release	Positive	1	1	1	1	1	1	1	1	1	1	1	1	1	1	1	1	1	1	1	1	1	1	1	1	1	1	1	1	1	1	1	1	1	1	1	1	1	1	1	1	1	1	1	1	1	1	1	1	1
56310	RVIDPRRCMK	NS3	1896	1905	HLA-A*31:01	100%	Dengue virus 2	ELISPOT	IFNg release	Positive	1	1	1	1	1	1	1	1	1	1	1	1	1	1	1	1	1	1	1	1	1	1	1	1	1	1	1	1	1	1	1	1	1	1	1	1	1	1	1	1	1	1	1	1	1	1	1	1	1
57554	SEMGANFKA	NS3	1872	1880	HLA-B*44:03	100%	Dengue virus 2	ELISPOT	IFNg release	Positive	1	1	1	1	1	1	1	1	1	1	1	1	1	1	1	1	1	1	1	1	1	1	1	1	1	1	1	1	1	1	1	1	1	1	1	1	1	1	1	1	1	1	1	1	1	1	1	1	1
65550	TPEGIIPSMFEPERE	NS3	500	514	HLA class I	100%	Dengue virus 2	ELISPOT	IFNg release	Positive	1	1	1	1	1	1	1	1	1	1	1	1	1	1	1	1	1	1	1	1	1	1	1	1	1	1	1	1	1	1	1	1	1	1	1	1	1	1	1	1	1	1	1	1	1	1	1	1	1
67210	TVWFVPSIK	NS3	358	366	HLA class I	100%	Dengue virus 2	ELISPOT	IFNg release	Positive	1	1	1	1	1	1	1	1	1	1	1	1	1	1	1	1	1	1	1	1	1	1	1	1	1	1	1	1	1	1	1	1	1	1	1	1	1	1	1	1	1	1	1	1	1	1	1	1	1
150289	IILEFFLIV	NS4a	2202	2210	HLA-A*02:01	100%	Dengue virus 2	ELISPOT	IFNg release	Positive	1	1	1	1	1	1	1	1	1	1	1	1	1	1	1	1	1	1	1	1	1	1	1	1	1	1	1	1	1	1	1	1	1	1	1	1	1	1	1	1	1	1	1	1	1	1	1	1	1
180620	LPTFMTQKAR	NS4a	2106	2115	HLA-B*35:01	100%	Dengue virus 2	ELISPOT	IFNg release	Positive	1	1	1	1	1	1	1	1	1	1	1	1	1	1	1	1	1	1	1	1	1	1	1	1	1	1	1	1	1	1	1	1	1	1	1	1	1	1	1	1	1	1	1	1	1	1	1	1	1
180781	TPQDNQLTY	NS4a	2221	2229	HLA-B*35:01	100%	Dengue virus 2	ELISPOT	IFNg release	Positive	1	1	1	1	1	1	1	1	1	1	1	1	1	1	1	1	1	1	1	1	1	1	1	1	1	1	1	1	1	1	1	1	1	1	1	1	1	1	1	1	1	1	1	1	1	1	1	1	1
2877	ALSELPETL	NS4a	2137	2145	HLA-A*02:01	100%	Dengue virus 2	ELISPOT	IFNg release	Positive	1	1	1	1	1	1	1	1	1	1	1	1	1	1	1	1	1	1	1	1	1	1	1	1	1	1	1	1	1	1	1	1	1	1	1	1	1	1	1	1	1	1	1	1	1	1	1	1	1
37557	LLLTLLATV	NS4a	56	64	HLA-A2	100%	Dengue virus 2	ICS	IFNg release	Positive	1	1	1	1	1	1	1	1	1	1	1	1	1	1	1	1	1	1	1	1	1	1	1	1	1	1	1	1	1	1	1	1	1	1	1	1	1	1	1	1	1	1	1	1	1	1	1	1	1
150147	ATVLMGLGK	NS4b	2315	2323	HLA-A*11:01	100%	Dengue virus 2	multimer/tetramer	qualitative binding	Positive	1	1	1	1	1	1	1	1	1	1	1	1	1	1	1	1	1	1	1	1	1	1	1	1	1	1	1	1	1	1	1	1	1	1	1	1	1	1	1	1	1	1	1	1	1	1	1	1	1
150387	LMGLGKGWPL	NS4b	2318	2327	HLA-A*02:01	100%	Dengue virus 2	ELISPOT	IFNg release	Positive	1	1	1	1	1	1	1	1	1	1	1	1	1	1	1	1	1	1	1	1	1	1	1	1	1	1	1	1	1	1	1	1	1	1	1	1	1	1	1	1	1	1	1	1	1	1	1	1	1
150534	RPASAWTLY	NS4b	2276	2284	HLA-B*07:02	100%	Dengue virus 2	ELISPOT	IFNg release	Positive	1	1	1	1	1	1	1	1	1	1	1	1	1	1	1	1	1	1	1	1	1	1	1	1	1	1	1	1	1	1	1	1	1	1	1	1	1	1	1	1	1	1	1	1	1	1	1	1	1
150679	VIDLDPIPY	NS4b	2395	2403	HLA-A*01:01	100%	Dengue virus 2	ELISPOT	IFNg release	Positive	1	1	1	1	1	1	1	1	1	1	1	1	1	1	1	1	1	1	1	1	1	1	1	1	1	1	1	1	1	1	1	1	1	1	1	1	1	1	1	1	1	1	1	1	1	1	1	1	1
180512	IANQATVLM	NS4b	2311	2319	HLA-B*35:01	100%	Dengue virus 2	ELISPOT	IFNg release	Positive	1	1	1	1	1	1	1	1	1	1	1	1	1	1	1	1	1	1	1	1	1	1	1	1	1	1	1	1	1	1	1	1	1	1	1	1	1	1	1	1	1	1	1	1	1	1	1	1	1
180514	IAVSMANIF	NS4b	2460	2468	HLA-B*35:01	100%	Dengue virus 2	ELISPOT	IFNg release	Positive	1	1	1	1	1	1	1	1	1	1	1	1	1	1	1	1	1	1	1	1	1	1	1	1	1	1	1	1	1	1	1	1	1	1	1	1	1	1	1	1	1	1	1	1	1	1	1	1	1
180789	TVIDLDPIPYDPKFE	NS4b	2394	2408	HLA class I	100%	Dengue virus 2	ELISPOT	IFNg release	Positive	1	1	1	1	1	1	1	1	1	1	1	1	1	1	1	1	1	1	1	1	1	1	1	1	1	1	1	1	1	1	1	1	1	1	1	1	1	1	1	1	1	1	1	1	1	1	1	1	1
184814	MGLGKGWPL	NS4b	2319	2327	HLA-B*08:01	100%	Dengue virus 2	ELISPOT	IFNg release	Positive	1	1	1	1	1	1	1	1	1	1	1	1	1	1	1	1	1	1	1	1	1	1	1	1	1	1	1	1	1	1	1	1	1	1	1	1	1	1	1	1	1	1	1	1	1	1	1	1	1
150309	IPMSTYGWNL	NS5	2605	2614	HLA-B*07:02	100%	Dengue virus 2	ELISPOT	IFNg release	Positive	1	1	1	1	1	1	1	1	1	1	1	1	1	1	1	1	1	1	1	1	1	1	1	1	1	1	1	1	1	1	1	1	1	1	1	1	1	1	1	1	1	1	1	1	1	1	1	1	1
150655	TPRMCTREEF	NS5	2881	2890	HLA-B*35:01	100%	Dengue virus 2	ELISPOT	IFNg release	Positive	1	1	1	1	1	1	1	1	1	1	1	1	1	1	1	1	1	1	1	1	1	1	1	1	1	1	1	1	1	1	1	1	1	1	1	1	1	1	1	1	1	1	1	1	1	1	1	1	1
150750	YTDYMPSMK	NS5	3370	3378	HLA-A*01:01	100%	Dengue virus 2	ELISPOT	IFNg release	Positive	1	1	1	1	1	1	1	1	1	1	1	1	1	1	1	1	1	1	1	1	1	1	1	1	1	1	1	1	1	1	1	1	1	1	1	1	1	1	1	1	1	1	1	1	1	1	1	1	1
163027	NIQTAINQV	NS5	3353	3361	HLA-A*02:01	100%	Dengue virus 2	ELISPOT	IFNg release	Positive-Low	1	1	1	1	1	1	1	1	1	1	1	1	1	1	1	1	1	1	1	1	1	1	1	1	1	1	1	1	1	1	1	1	1	1	1	1	1	1	1	1	1	1	1	1	1	1	1	1	1
180443	ETACLGKSYA	NS5	3241	3250	HLA-A*26:01	100%	Dengue virus 2	ELISPOT	IFNg release	Positive	1	1	1	1	1	1	1	1	1	1	1	1	1	1	1	1	1	1	1	1	1	1	1	1	1	1	1	1	1	1	1	1	1	1	1	1	1	1	1	1	1	1	1	1	1	1	1	1	1
180643	MAMTDTTPFGQQRVF	NS5	2832	2846	HLA class I	100%	Dengue virus 2	ELISPOT	IFNg release	Positive	1	1	1	1	1	1	1	1	1	1	1	1	1	1	1	1	1	1	1	1	1	1	1	1	1	1	1	1	1	1	1	1	1	1	1	1	1	1	1	1	1	1	1	1	1	1	1	1	1
180667	MPSMKRFRRE	NS5	3374	3383	HLA-B*53:01	100%	Dengue virus 2	ELISPOT	IFNg release	Positive	1	1	1	1	1	1	1	1	1	1	1	1	1	1	1	1	1	1	1	1	1	1	1	1	1	1	1	1	1	1	1	1	1	1	1	1	1	1	1	1	1	1	1	1	1	1	1	1	1
180675	MSTYGWNLVRLQSGV	NS5	2607	2621	HLA class I	100%	Dengue virus 2	ELISPOT	IFNg release	Positive	1	1	1	1	1	1	1	1	1	1	1	1	1	1	1	1	1	1	1	1	1	1	1	1	1	1	1	1	1	1	1	1	1	1	1	1	1	1	1	1	1	1	1	1	1	1	1	1	1
180814	WAYHGSYET	NS5	2794	2802	HLA-B*35:01	100%	Dengue virus 2	ELISPOT	IFNg release	Positive	1	1	1	1	1	1	1	1	1	1	1	1	1	1	1	1	1	1	1	1	1	1	1	1	1	1	1	1	1	1	1	1	1	1	1	1	1	1	1	1	1	1	1	1	1	1	1	1	1
180817	WHYDQDHPY	NS5	2783	2791	HLA-B*35:01	100%	Dengue virus 2	ELISPOT	IFNg release	Positive	1	1	1	1	1	1	1	1	1	1	1	1	1	1	1	1	1	1	1	1	1	1	1	1	1	1	1	1	1	1	1	1	1	1	1	1	1	1	1	1	1	1	1	1	1	1	1	1	1
184131	KREDQWCGSL	NS5	3332	3341	HLA-B*08:01	100%	Dengue virus 2	ELISPOT	IFNg release	Positive	1	1	1	1	1	1	1	1	1	1	1	1	1	1	1	1	1	1	1	1	1	1	1	1	1	1	1	1	1	1	1	1	1	1	1	1	1	1	1	1	1	1	1	1	1	1	1	1	1
185491	RREEEEAGVL	NS5	3381	3390	HLA-B*08:01	100%	Dengue virus 2	ELISPOT	IFNg release	Positive	1	1	1	1	1	1	1	1	1	1	1	1	1	1	1	1	1	1	1	1	1	1	1	1	1	1	1	1	1	1	1	1	1	1	1	1	1	1	1	1	1	1	1	1	1	1	1	1	1
185931	TKQTGSASSM	NS5	2802	2811	HLA-B*08:01	100%	Dengue virus 2	ELISPOT	IFNg release	Positive	1	1	1	1	1	1	1	1	1	1	1	1	1	1	1	1	1	1	1	1	1	1	1	1	1	1	1	1	1	1	1	1	1	1	1	1	1	1	1	1	1	1	1	1	1	1	1	1	1
190862	MVTQMAMTDTTPFGQQR	NS5	2828	2844	HLA class I	100%	Dengue virus 2	ICS	IFNg release	Positive	1	1	1	1	1	1	1	1	1	1	1	1	1	1	1	1	1	1	1	1	1	1	1	1	1	1	1	1	1	1	1	1	1	1	1	1	1	1	1	1	1	1	1	1	1	1	1	1	1
53476	REDQWCGSL	NS5	3333	3341	HLA-B*40:01	100%	Dengue virus 2	ELISPOT	IFNg release	Positive	1	1	1	1	1	1	1	1	1	1	1	1	1	1	1	1	1	1	1	1	1	1	1	1	1	1	1	1	1	1	1	1	1	1	1	1	1	1	1	1	1	1	1	1	1	1	1	1	1
539436	ISGDDCVVKPLDDRFA	NS5	3151	3166	HLA class I	100%	Dengue virus 2	ICS	IFNg release	Positive	1	1	1	1	1	1	1	1	1	1	1	1	1	1	1	1	1	1	1	1	1	1	1	1	1	1	1	1	1	1	1	1	1	1	1	1	1	1	1	1	1	1	1	1	1	1	1	1	1
69671	VLNPYMPSV	NS5	2673	2681	HLA-A*02:01	100%	Dengue virus 2	ELISPOT	IFNg release	Positive	1	1	1	1	1	1	1	1	1	1	1	1	1	1	1	1	1	1	1	1	1	1	1	1	1	1	1	1	1	1	1	1	1	1	1	1	1	1	1	1	1	1	1	1	1	1	1	1	1
110018	VAFLRFLTI	C	51	59	HLA-B*08:01	81% - <100%	Dengue virus 2	ELISPOT	IFNg release	Positive	1	1	1	1	1	1	1	1	1	1	1	1	1	1	1	1	1	1	1	1	0	1	1	1	1	1	1	1	1	1	1	1	1	1	1	1	1	1	1	1	1	1	1	1	1	1	1	1	1
184378	LGMLQGRGPL	C	35	44	HLA-B*08:01	81% - <100%	Dengue virus 2	ELISPOT	IFNg release	Positive	1	1	1	1	1	1	1	1	1	1	1	1	1	1	1	1	1	1	1	1	0	1	1	1	1	1	1	1	1	1	1	1	1	1	1	1	1	1	1	1	1	1	1	1	1	1	1	1	1
184643	LTIPPTAGIL	C	21	30	HLA-B*08:01	81% - <100%	Dengue virus 2	ELISPOT	IFNg release	Positive	1	1	1	1	1	1	1	1	1	1	1	1	1	1	0	1	1	1	1	1	0	0	1	1	1	1	1	1	1	1	1	1	1	1	1	1	1	1	1	1	1	1	1	1	1	1	1	1	1
186354	VQQLTKRFSL	C	26	35	HLA-B*08:01	81% - <100%	Dengue virus 2	ELISPOT	IFNg release	Positive	1	1	1	1	1	1	1	1	1	1	1	1	1	1	1	1	1	1	1	1	0	1	1	1	1	1	1	1	1	1	1	1	1	1	1	1	1	1	1	1	1	1	1	1	1	1	1	1	1
150224	FTIMAAILAY	PreM	247	256	HLA-B*35:01	81% - <100%	Dengue virus 2	ELISPOT	IFNg release	Positive	1	1	1	1	1	1	1	1	1	1	1	1	1	1	1	1	1	1	1	1	1	1	1	1	1	1	1	1	1	1	1	1	1	1	1	1	0	1	1	1	1	1	1	1	0	1	1	1	1
150299	ILLTAVAPSM	PreM	270	279	HLA-B*08:01	81% - <100%	Dengue virus 2	ELISPOT	IFNg release	Positive	1	1	1	1	1	1	1	1	1	1	1	1	1	1	1	1	1	1	1	1	0	1	1	1	1	1	1	1	1	1	1	1	1	1	1	1	1	0	1	1	1	1	1	1	1	1	1	1	1
150571	SLLFKTEDGV	PreM	136	145	HLA-A*02:01	81% - <100%	Dengue virus 2	ELISPOT	IFNg release	Positive	1	1	1	1	1	1	1	1	1	1	1	1	1	1	1	1	1	1	1	1	1	0	1	1	1	1	1	1	1	1	1	1	1	1	1	1	1	0	1	1	1	1	1	1	1	1	1	1	1
150115	AIYGAAFSGV	E	722	731	HLA-A*02:01	81% - <100%	Dengue virus 2	ELISPOT	IFNg release	Positive	1	1	1	1	1	1	1	1	1	1	1	1	1	1	1	1	1	1	1	1	1	1	1	1	1	1	1	1	1	1	1	1	1	0	1	1	1	1	1	1	1	1	1	1	1	1	1	1	1
150319	ITEAELTGY	E	450	458	HLA-A*01:01	81% - <100%	Dengue virus 2	ELISPOT	IFNg release	Positive	1	1	1	0	1	1	1	1	1	1	1	1	1	1	1	1	1	1	1	1	0	1	1	1	1	1	1	1	1	1	1	1	1	1	1	1	1	1	1	1	1	1	1	1	0	1	1	1	1
150399	LQMENKAWLV	E	231	240	HLA-B*08:01	81% - <100%	Dengue virus 2	ELISPOT	IFNg release	Positive	1	1	1	1	1	1	1	1	1	1	1	1	1	1	1	1	1	1	1	1	0	1	1	1	1	1	1	1	1	1	1	1	1	1	1	1	1	0	1	1	1	1	1	1	1	1	1	1	1
150529	RLITVNPIV	E	630	638	HLA-A*02:01	81% - <100%	Dengue virus 2	ELISPOT	IFNg release	Positive	1	1	1	1	1	1	1	1	1	1	1	1	1	1	1	1	1	1	1	1	0	1	1	1	1	1	1	1	1	1	1	1	1	1	1	1	1	1	1	1	1	1	1	1	1	1	1	1	1
150578	SPCKIPFEIM	E	611	620	HLA-B*35:01	81% - <100%	Dengue virus 2	ELISPOT	IFNg release	Positive	1	1	1	1	1	1	1	1	1	1	1	1	1	1	1	1	1	1	1	1	0	1	1	1	1	1	1	1	1	1	1	1	1	1	1	1	1	1	1	1	1	1	1	1	1	1	1	1	1
150725	WLVHRQWFL	E	486	494	HLA-B*08:01	81% - <100%	Dengue virus 2	ELISPOT	IFNg release	Positive	1	1	1	1	1	1	1	1	1	1	1	1	1	1	1	1	1	1	1	1	0	1	1	1	1	1	1	1	1	1	1	1	1	1	1	1	1	1	1	1	1	1	1	1	1	1	1	1	1
180453	FGAIYGAAF	E	720	728	HLA-B*35:01	81% - <100%	Dengue virus 2	ELISPOT	IFNg release	Positive	1	1	1	1	1	1	1	1	1	1	1	1	1	1	1	1	1	1	1	1	1	1	1	1	1	1	1	1	1	1	1	1	1	0	1	1	1	1	1	1	1	1	1	1	1	1	1	1	1
180528	IPFEIMDLEK	E	615	624	HLA-B*35:01	81% - <100%	Dengue virus 2	ELISPOT	IFNg release	Positive	1	1	1	1	1	1	1	1	1	1	1	1	1	1	1	1	1	1	1	1	0	1	1	1	1	1	1	1	1	1	1	1	1	1	1	1	1	1	1	1	1	1	1	1	1	1	1	1	1
183310	GAAFSGVSW	E	725	733	HLA-B*58:01	81% - <100%	Dengue virus 2	ELISPOT	IFNg release	Positive	1	1	1	1	1	1	1	1	1	1	1	1	1	1	1	1	1	1	1	1	1	1	1	1	1	1	1	1	1	1	1	1	1	0	1	1	1	1	1	1	1	1	1	1	1	1	1	1	1
183748	ILIGVVITW	E	737	745	HLA-B*58:01	81% - <100%	Dengue virus 2	ELISPOT	IFNg release	Positive	1	1	1	1	1	1	1	1	1	1	1	1	1	1	0	1	1	1	1	1	1	1	1	1	1	1	1	1	1	1	1	1	1	1	1	1	1	0	1	1	1	1	1	1	1	1	1	1	1
183781	IMDLEKRHVL	E	619	628	HLA-B*08:01	81% - <100%	Dengue virus 2	ELISPOT	IFNg release	Positive	1	1	1	0	1	1	1	1	1	1	1	1	1	1	1	1	0	1	1	1	0	1	1	1	1	1	1	1	1	1	1	1	1	1	1	1	1	1	1	1	1	1	1	1	1	1	1	1	1
183840	IQKETLVTF	E	512	520	HLA-B*15:01	81% - <100%	Dengue virus 2	ELISPOT	IFNg release	Positive	1	1	1	1	1	1	1	1	1	1	1	1	1	1	1	1	1	1	1	1	1	1	0	1	1	1	1	1	1	1	1	1	1	1	1	1	1	1	1	1	1	1	1	1	1	1	1	1	1
184337	LEKRHVLGRL	E	622	631	HLA-B*08:01	81% - <100%	Dengue virus 2	ELISPOT	IFNg release	Positive	1	1	1	0	1	1	1	1	1	1	1	1	1	1	1	1	0	1	1	1	0	1	1	1	1	1	1	1	1	1	1	1	1	1	1	1	1	1	1	1	1	1	1	1	1	1	1	1	1
184755	MDLEKRHVL	E	620	628	HLA-B*08:01	81% - <100%	Dengue virus 2	ELISPOT	IFNg release	Positive	1	1	1	0	1	1	1	1	1	1	1	1	1	1	1	1	0	1	1	1	0	1	1	1	1	1	1	1	1	1	1	1	1	1	1	1	1	1	1	1	1	1	1	1	1	1	1	1	1
41407	MENKAWLVHRQWFLD	E	201	215	HLA-B*37:01	81% - <100%	Dengue virus 2	ICS	IFNg release	Positive	1	1	1	1	1	1	1	1	1	1	1	1	1	1	1	1	1	1	1	1	0	1	1	1	1	1	1	1	1	1	1	1	1	1	1	1	1	0	1	1	1	1	1	1	1	1	1	1	1
180450	FAGPVSQHNY	NS1	1022	1031	HLA-B*35:01	81% - <100%	Dengue virus 2	ELISPOT	IFNg release	Positive	1	1	1	1	1	1	1	1	1	1	1	1	1	1	1	1	1	1	1	1	0	1	1	1	1	0	1	1	1	1	1	1	1	1	1	1	1	0	1	1	1	1	1	1	1	1	1	1	1
180689	NRAVHADMGYWIESA	NS1	966	980	HLA class I	81% - <100%	Dengue virus 2	ICS	IFNg release	Positive	1	1	0	1	1	1	1	1	0	1	1	1	1	1	1	1	1	1	1	1	1	1	0	1	1	1	1	1	1	1	1	1	1	1	1	1	1	1	1	1	1	1	1	0	1	1	1	1	1
182938	EKEENLVNSL	NS1	1115	1124	HLA-B*08:01	81% - <100%	Dengue virus 2	ELISPOT	IFNg release	Positive	1	1	1	1	1	1	1	1	1	1	1	1	1	1	1	1	1	1	1	1	1	1	1	1	1	1	1	1	1	1	1	1	1	1	1	1	1	0	1	1	1	1	1	1	1	1	1	1	1
185342	RAVHADMGY	NS1	192	200	HLA-B*58:01	81% - <100%	Dengue virus 2	ELISPOT	IFNg release	Positive	1	1	0	1	1	1	1	1	0	1	1	1	1	1	1	1	1	1	1	1	1	1	1	1	1	1	1	1	1	1	1	1	1	1	1	1	1	1	1	1	1	1	1	1	1	1	1	1	1
150206	FLEEMLRTRV	NS2a	1131	1140	HLA-A*02:01	81% - <100%	Dengue virus 2	ELISPOT	IFNg release	Positive	1	1	1	1	1	1	1	0	1	1	1	1	1	1	1	1	1	1	1	1	1	0	1	1	1	1	1	1	1	1	1	1	1	1	1	1	1	1	1	1	1	1	1	1	1	1	1	1	1
150391	LMMTTIGIVL	NS2a	1228	1237	HLA-B*08:01	81% - <100%	Dengue virus 2	ELISPOT	IFNg release	Positive	1	1	1	1	1	1	1	1	1	1	1	1	1	1	1	1	1	1	1	1	1	1	1	1	1	1	1	1	1	1	1	1	1	1	1	1	1	1	1	1	0	1	1	1	1	1	1	1	1
150457	MTDDIGMGV	NS2a	1191	1199	HLA-A*01:01	81% - <100%	Dengue virus 2	ELISPOT	IFNg release	Positive	1	1	1	1	1	1	1	1	1	1	1	1	1	1	1	1	1	1	1	0	1	1	1	1	1	1	1	1	1	1	1	1	1	1	1	1	1	1	1	1	1	1	1	1	1	1	1	1	1
150540	RPTFAAGLLL	NS2a	1211	1220	HLA-B*07:02	81% - <100%	Dengue virus 2	ELISPOT	IFNg release	Positive	1	0	1	1	1	1	1	1	1	1	1	0	0	1	1	1	1	1	1	1	1	1	1	1	1	1	1	1	1	1	1	1	1	1	0	1	1	1	1	1	1	1	1	1	1	1	1	1	1
150741	YQLAVTIMA	NS2a	1157	1165	HLA-A*02:01	81% - <100%	Dengue virus 2	ELISPOT	IFNg release	Positive	1	1	1	1	1	1	1	1	1	1	1	1	1	1	1	1	1	1	1	1	1	1	1	1	1	1	1	0	1	1	1	1	1	1	1	1	1	1	1	1	1	1	1	0	1	1	1	1	1
183741	ILCVPNAVIL	NS2a	1279	1288	HLA-B*08:01	81% - <100%	Dengue virus 2	ELISPOT	IFNg release	Positive	1	1	1	0	1	1	1	1	1	1	1	1	1	1	1	1	1	1	1	0	0	1	1	1	1	1	1	1	1	1	1	1	1	0	1	1	1	1	1	1	1	1	1	1	0	1	1	1	1
183825	IPLALTIKGL	NS2a	1319	1328	HLA-B*08:01	81% - <100%	Dengue virus 2	ELISPOT	IFNg release	Positive	1	1	0	1	1	1	1	1	1	1	1	1	1	1	1	1	0	0	0	1	1	1	1	1	1	1	1	1	1	1	1	1	1	1	1	1	1	1	1	1	1	1	1	1	1	1	1	1	1
184864	MLRTRVGTK	NS2a	1149	1157	HLA-B*08:01	81% - <100%	Dengue virus 2	ELISPOT	IFNg release	Positive	1	1	1	1	1	1	1	0	1	1	1	1	1	1	1	1	1	1	1	1	1	0	1	1	1	1	1	1	1	1	1	1	1	1	1	1	1	1	1	1	1	1	1	1	1	1	1	1	1

150101	AAAWYLWEV	NS2b	1463	1471	HLA-A*02:01	81% - <100%	Dengue virus 2	ELISPOT	IFNg release	Positive	1	1	1	1	1	1	0	0	0	0	0	1	1	1	1	1	1	1	1	1	1	1	1	1	1	1	1	1	1	1	1	1	1	1	1	1	1	1	1	1	1	1	1	1	1	1	1	1	1	1
150243	GLLTVCYVL	NS2b	1381	1389	HLA-A*02:01	81% - <100%	Dengue virus 2	ELISPOT	IFNg release	Positive	1	1	1	1	1	1	1	1	1	1	1	1	1	1	1	1	1	1	1	1	0	1	1	1	1	1	1	1	1	1	1	1	1	1	1	1	1	1	1	1	1	1	1	1	1	1	1	1	1	1
150381	LLVISGLFPV	NS2b	1448	1457	HLA-A*02:01	81% - <100%	Dengue virus 2	ELISPOT	IFNg release	Positive	1	1	1	1	1	1	1	1	1	1	1	1	1	1	1	1	1	1	1	1	0	1	1	1	1	1	1	1	1	1	1	1	1	1	1	1	1	1	1	1	1	1	1	1	1	1	1	1	1	1
150425	LVISGLFPV	NS2b	1449	1457	HLA-A*02:01	81% - <100%	Dengue virus 2	ELISPOT	IFNg release	Positive	1	1	1	1	1	1	1	1	1	1	1	1	1	1	1	1	1	1	1	1	0	1	1	1	1	1	1	1	1	1	1	1	1	1	1	1	1	1	1	1	1	1	1	1	1	1	1	1	1	1
150584	SPILSITISE	NS2b	1416	1425	HLA-B*35:01	81% - <100%	Dengue virus 2	ELISPOT	IFNg release	Positive	1	1	1	1	1	1	1	1	1	1	1	1	1	1	1	1	1	1	1	1	1	1	1	1	1	1	1	0	0	1	0	0	1	1	1	1	1	1	0	1	1	1	1	1	1	1	1	1	1	1
150610	TAAAWYLWEV	NS2b	117	126	HLA-A*02:01	81% - <100%	Dengue virus 2	ELISPOT	IFNg release	Positive	1	1	1	1	1	1	0	0	0	0	0	1	1	1	1	1	1	1	1	1	1	1	1	1	1	1	1	1	1	1	1	1	1	1	1	1	1	1	1	1	1	1	1	1	1	1	1	1	1	1
180529	IPITAAAWY	NS2b	1459	1467	HLA-B*35:01	81% - <100%	Dengue virus 2	ELISPOT	IFNg release	Positive	1	1	1	1	1	1	0	0	0	0	0	1	1	1	1	1	1	1	1	1	1	0	1	1	1	1	1	1	1	1	1	1	1	1	1	1	1	1	1	1	1	1	1	1	1	1	1	1	1	1
180538	ISGLFPVSIPITAAA	NS2b	106	120	HLA class I	81% - <100%	Dengue virus 2	ICS	IFNg release	Positive	1	1	1	1	1	1	0	0	0	0	0	1	1	1	1	1	1	1	1	1	0	0	1	1	1	1	1	1	1	1	1	1	1	1	1	1	1	1	1	1	1	1	1	1	1	1	1	1	1	1
183819	IPITAAAWYL	NS2b	1459	1468	HLA-B*08:01	81% - <100%	Dengue virus 2	ELISPOT	IFNg release	Positive	1	1	1	1	1	1	0	0	0	0	0	1	1	1	1	1	1	1	1	1	1	0	1	1	1	1	1	1	1	1	1	1	1	1	1	1	1	1	1	1	1	1	1	1	1	1	1	1	1	1
11063	EAHFTDPASIAARGY	NS3	285	299	HLA class I	81% - <100%	Dengue virus 2	ELISPOT	IFNg release	Positive	1	1	1	1	1	1	1	1	1	1	1	1	1	1	1	1	1	1	1	1	1	1	1	1	1	1	1	1	1	1	1	1	1	1	1	1	1	1	0	1	1	1	1	1	1	1	1	1	1	1
150450	MPVTHSSAA	NS3	1922	1930	HLA-B*08:01	81% - <100%	Dengue virus 2	ELISPOT	IFNg release	Positive	1	1	1	1	1	1	1	1	1	1	1	1	1	1	1	1	1	1	1	1	1	1	1	1	1	1	1	1	1	1	1	1	1	1	1	1	1	1	0	1	1	1	1	1	1	1	1	1	1	1
150451	MPVTHSSAAQ	NS3	1922	1931	HLA-B*35:01	81% - <100%	Dengue virus 2	ELISPOT	IFNg release	Positive	1	1	1	1	1	1	1	1	1	1	1	1	1	1	1	1	1	1	1	1	1	1	1	1	1	1	1	1	1	1	1	1	1	1	1	1	1	1	0	1	1	1	1	1	1	1	1	1	1	1
180611	LMRRGDLPVWLAYRV	NS3	2011	2025	HLA-B*55:02	81% - <100%	Dengue virus 2	ICS	IFNg release	Positive	1	1	1	1	1	1	1	1	1	1	1	1	1	1	1	1	1	1	1	1	1	1	1	1	1	1	1	1	1	1	1	1	1	1	1	1	1	0	1	1	1	1	1	1	1	1	1	1	1	1
180625	LPVWLAYRV	NS3	2017	2025	HLA-B*51:01	81% - <100%	Dengue virus 2	ELISPOT	IFNg release	Positive	1	1	1	1	1	1	1	1	1	1	1	1	1	1	1	1	1	1	1	1	1	1	1	1	1	1	1	1	1	1	1	1	1	1	1	1	1	0	1	1	1	1	1	1	1	1	1	1	1	1
180626	LPVWLAYRVA	NS3	2017	2026	HLA-B*51:01	81% - <100%	Dengue virus 2	ELISPOT	IFNg release	Positive	1	1	1	1	1	1	1	1	1	1	1	1	1	1	1	1	1	1	1	1	1	1	1	1	1	1	1	1	1	1	1	1	1	1	1	1	1	0	1	1	1	1	1	1	1	1	1	1	1	1
180756	STRVEMGEAAGIFMT	NS3	301	315	HLA-B*40:06	81% - <100%	Dengue virus 2	ELISPOT	IFNg release	Positive-Low	1	1	1	1	1	1	1	1	1	1	1	1	1	1	1	1	1	1	1	1	1	1	1	1	1	1	1	1	1	1	1	1	0	1	1	1	1	1	1	1	1	1	1	1	1	1	1	1	1	1
185388	RIKQKGILGY	NS3	1499	1508	HLA-B*15:01	81% - <100%	Dengue virus 2	ELISPOT	IFNg release	Positive	1	1	1	1	1	1	1	1	1	1	1	1	0	1	1	1	1	1	1	1	0	1	1	1	1	1	1	1	1	1	1	1	1	1	1	1	1	1	1	1	0	1	1	1	1	1	1	1	0
186242	VEMGEAAGIF	NS3	1779	1788	HLA-B*44:03	81% - <100%	Dengue virus 2	ELISPOT	IFNg release	Positive	1	1	1	1	1	1	1	1	1	1	1	1	1	1	1	1	1	1	1	1	1	1	1	1	1	1	1	1	1	1	1	1	0	1	1	1	1	1	1	1	1	1	1	1	1	1	1	1	1	1
22823	GTSGSPIVDK	NS3	133	142	HLA-A11	81% - <100%	Dengue virus 2	51 chromium	cytotoxicity	Positive	1	1	1	1	1	1	1	1	1	1	1	1	1	1	1	1	1	1	1	1	1	1	1	1	1	1	1	1	1	1	1	1	1	1	1	1	1	1	1	1	0	1	1	1	1	1	1	1	1
54176	RIKQKGIL	NS3	1499	1506	HLA-B8	81% - <100%	Dengue virus 2	ELISPOT	IFNg release	Positive	1	1	1	1	1	1	1	1	1	1	1	1	0	1	1	1	1	1	1	1	0	1	1	1	1	1	1	1	1	1	1	1	1	1	1	1	1	1	1	1	0	1	1	1	1	1	1	1	0
8910	DKKGKVVGL	NS3	1616	1624	HLA-B8	81% - <100%	Dengue virus 2	ELISPOT	IFNg release	Positive	1	1	0	1	1	1	1	1	1	1	1	1	1	1	1	1	0	0	0	1	1	1	1	1	1	1	1	1	1	1	1	1	1	1	1	1	1	1	1	1	1	1	1	1	1	1	1	1	1
150461	MTQKARNAL	NS4a	2110	2118	HLA-B*07:02	81% - <100%	Dengue virus 2	ELISPOT	IFNg release	Positive	1	1	1	1	1	1	1	1	1	1	1	1	1	1	1	1	1	1	1	1	1	1	1	1	1	1	1	1	1	1	1	1	1	1	1	1	1	0	1	1	1	1	1	1	1	1	1	1	1	1
150612	TAEAGGRAY	NS4a	2126	2134	HLA-B*35:01	81% - <100%	Dengue virus 2	ELISPOT	IFNg release	Positive	1	1	1	1	1	1	1	1	1	1	1	1	1	1	1	1	1	1	1	1	1	1	1	1	1	1	1	1	1	1	1	1	1	1	1	1	1	0	1	1	1	1	1	1	1	1	1	1	1	1
150752	YVVIAILTV	NS4a	2229	2237	HLA-A*02:01	81% - <100%	Dengue virus 2	ELISPOT	IFNg release	Positive	1	1	1	1	1	1	1	1	1	1	1	1	1	1	1	1	1	1	1	1	0	1	1	1	1	1	1	1	1	1	1	1	1	1	1	1	1	0	1	1	1	1	1	0	1	1	1	1	1	1
150753	YVVIAILTVV	NS4a	2229	2238	HLA-A*02:01	81% - <100%	Dengue virus 2	ELISPOT	IFNg release	Positive	1	1	1	1	1	1	1	1	1	1	1	1	1	1	1	1	1	1	1	1	0	1	1	1	1	1	1	1	1	1	1	1	1	1	1	1	1	0	1	1	1	1	1	0	1	1	1	1	1	1
185447	RNALDNLAVL	NS4a	2115	2124	HLA-B*08:01	81% - <100%	Dengue virus 2	ELISPOT	IFNg release	Positive	1	1	1	1	1	1	1	1	1	1	1	1	1	1	1	1	1	1	1	1	1	1	1	1	1	1	1	1	1	1	1	1	1	1	1	1	1	0	1	1	1	1	1	1	1	1	1	1	1	1
51562	QLTYVVIAIL	NS4a	2226	2235	HLA-B*08:01	81% - <100%	Dengue virus 2	ELISPOT	IFNg release	Positive	1	1	1	1	1	1	1	1	1	1	1	1	1	1	1	1	1	1	1	1	1	1	1	1	1	1	1	1	1	1	1	1	1	1	1	1	1	0	1	1	1	1	1	0	1	1	1	1	1	1
150377	LLLVAHYAI	NS4b	2355	2363	HLA-A*02:01	81% - <100%	Dengue virus 2	ELISPOT	IFNg release	Positive	1	1	1	1	1	1	1	0	1	1	0	1	1	1	1	1	1	1	1	1	1	0	1	1	1	1	1	1	1	1	1	1	1	1	1	1	1	0	1	1	1	1	1	1	1	1	1	1	1	1
150389	LMMRTTWAL	NS4b	2425	2433	HLA-B*08:01	81% - <100%	Dengue virus 2	ELISPOT	IFNg release	Positive	1	1	1	1	1	1	1	1	1	1	1	1	1	1	1	1	1	1	1	1	1	1	1	1	1	1	1	1	1	1	1	1	1	1	1	1	1	0	1	1	0	1	1	1	1	1	1	1	1	1
150686	VLMMRTTWA	NS4b	2424	2432	HLA-B*08:01	81% - <100%	Dengue virus 2	ELISPOT	IFNg release	Positive	1	1	1	1	1	1	1	1	1	1	1	1	1	1	1	1	1	1	1	1	1	1	1	1	1	1	1	1	1	1	1	1	1	1	1	1	1	0	1	1	0	1	1	1	1	1	1	1	1	1
180380	ATGPISTLW	NS4b	2440	2448	HLA-B*58:01	81% - <100%	Dengue virus 2	ELISPOT	IFNg release	Positive	1	1	1	1	1	1	1	1	1	1	1	1	1	1	1	1	1	1	1	1	1	1	1	1	1	1	1	1	1	1	1	1	1	1	1	1	1	0	1	1	1	1	1	1	1	1	1	1	1	1
180387	CEALTLATGPISTLW	NS4b	191	205	HLA class I	81% - <100%	Dengue virus 2	ELISPOT	IFNg release	Positive	1	1	1	1	1	1	1	1	1	1	1	1	1	1	1	1	1	1	1	1	1	1	1	1	1	1	1	1	1	1	1	1	0	1	1	1	1	0	1	1	1	1	1	1	1	1	1	1	1	1
180802	VPLLAIGCY	NS4b	2334	2342	HLA-B*35:01	81% - <100%	Dengue virus 2	ELISPOT	IFNg release	Positive	1	1	1	1	1	1	1	1	1	1	1	1	1	1	1	1	1	1	1	1	1	1	1	1	1	1	1	1	1	1	1	1	1	1	1	1	1	0	1	1	1	1	1	1	1	1	1	1	1	1
182747	CYSQVNPITL	NS4b	2341	2350	HLA-A*24:02	81% - <100%	Dengue virus 2	ELISPOT	IFNg release	Positive	1	1	1	1	1	1	1	1	1	1	1	1	1	1	1	1	1	1	1	1	1	1	1	1	1	1	1	1	1	1	1	1	1	1	1	1	1	0	1	1	1	1	1	1	1	1	1	1	1	1
183670	IENSSVNVSL	NS4b	2299	2308	HLA-B*08:01	81% - <100%	Dengue virus 2	ELISPOT	IFNg release	Positive	1	1	1	1	1	1	1	1	1	1	1	1	1	1	1	1	1	1	1	1	1	1	1	1	1	1	1	1	1	1	1	1	1	1	1	1	1	0	1	1	1	1	1	1	1	1	1	1	1	1
186002	TPMLRHSIE	NS4b	2292	2300	HLA-B*08:01	81% - <100%	Dengue virus 2	ELISPOT	IFNg release	Positive	1	1	1	1	1	1	1	1	1	1	1	1	1	1	1	1	1	1	1	1	0	1	1	1	1	1	1	1	1	1	1	1	1	1	1	1	1	1	1	1	1	1	1	1	1	1	1	1	1	1
240933	TPMLRHSI	NS4b	2292	2299	HLA-B*07:02	81% - <100%	Dengue virus 2	ELISPOT	IFNg release	Positive	1	1	1	1	1	1	1	1	1	1	1	1	1	1	1	1	1	1	1	1	0	1	1	1	1	1	1	1	1	1	1	1	1	1	1	1	1	1	1	1	1	1	1	1	1	1	1	1	1	1
10587	DVFFTPPEK	NS5	2622	2630	HLA class I	81% - <100%	Dengue virus 2	ELISPOT	IFNg release	Positive	1	1	1	0	1	1	1	1	1	1	1	1	1	1	1	1	1	1	1	1	1	1	1	1	1	1	1	1	1	1	1	1	1	1	1	1	1	0	1	1	1	1	1	1	0	1	1	1	1	1
150227	FTMRHKKATY	NS5	2734	2743	HLA-B*35:01	81% - <100%	Dengue virus 2	ELISPOT	IFNg release	Positive	1	0	1	1	1	1	1	1	1	1	1	0	0	1	1	1	1	1	1	1	1	1	1	1	1	1	1	1	1	1	1	1	1	1	0	1	1	1	1	1	1	1	1	1	1	1	1	1	1	1
150541	RPTPRGTVM	NS5	3073	3081	HLA-B*07:02	81% - <100%	Dengue virus 2	ELISPOT	IFNg release	Positive	1	1	1	1	1	1	1	1	1	1	1	1	1	1	1	1	1	1	1	1	0	1	1	1	1	1	1	1	1	1	1	1	1	1	1	1	1	1	1	1	1	1	1	1	1	1	1	1	1	1
174066	CGGLKNVREVKGLTKGG	NS5	2582	2598	HLA-A2	81% - <100%	Dengue virus 2	ICS	IFNg release	Positive	1	1	1	1	1	1	1	1	1	1	1	1	1	1	1	1	1	1	1	1	1	1	1	1	1	1	1	1	1	1	1	1	1	1	1	1	1	1	0	1	1	1	1	1	1	1	1	1	1	1
180437	EPKEGTKKLM	NS5	2856	2865	HLA-B*35:01	81% - <100%	Dengue virus 2	ELISPOT	IFNg release	Positive	1	1	1	1	1	1	1	1	1	1	1	1	1	1	1	1	1	0	1	1	1	1	1	1	1	1	1	1	1	1	1	1	1	1	1	1	1	1	1	1	1	1	1	1	1	1	1	1	1	1
180483	GKKKTPRMCTREEFT	NS5	2877	2891	HLA class I	81% - <100%	Dengue virus 2	ELISPOT	IFNg release	Positive	1	1	1	0	1	1	1	1	1	1	1	1	1	1	1	1	1	1	1	1	1	1	0	1	1	1	1	1	1	1	1	1	1	1	1	1	1	1	1	1	1	1	1	1	1	1	1	1	1	1
180488	GPGHEEPIPM	NS5	2598	2607	HLA-B*35:01	81% - <100%	Dengue virus 2	ELISPOT	IFNg release	Positive	1	1	1	1	1	1	1	1	1	1	1	1	1	1	1	1	1	1	1	1	1	1	1	1	1	1	1	1	1	1	1	1	1	1	1	1	1	1	0	1	1	1	1	1	1	1	1	1	1	1
180500	HKKLAEAIFKLTYQN	NS5	3052	3066	HLA class I	81% - <100%	Dengue virus 2	ELISPOT	IFNg release	Positive	1	1	1	1	1	1	1	1	1	1	1	1	1	1	1	1	1	1	1	1	0	1	1	1	1	1	1	1	1	1	1	1	1	1	1	1	1	1	1	1	1	1	1	1	0	1	1	1	1	1
180579	KSYAQMWSLMYFHRR	NS5	3247	3261	HLA class I	81% - <100%	Dengue virus 2	ELISPOT	IFNg release	Positive	1	1	1	1	1	1	1	1	1	1	1	1	1	1	1	1	1	1	1	1	0	0	1	1	1	1	1	1	1	1	1	1	1	1	1	1	1	1	1	1	1	1	1	1	1	1	1	1	1	1
180582	KTWAYHGSYETKQTG	NS5	2792	2806	HLA class I	81% - <100%	Dengue virus 2	ICS	IFNg release	Positive	1	1	0	1	1	1	1	1	1	1	1	1	1	1	1	1	1	1	1	1	1	1	1	1	1	1	1	1	1	1	1	1	1	1	1	1	1	1	1	1	1	1	1	1	1	1	1	1	1	1
180584	KVRKDIQQW	NS5	3177	3185	HLA-B*57:01	81% - <100%	Dengue virus 2	ELISPOT	IFNg release	Positive	1	1	1	1	1	1	1	1	1	1	1	1	1	1	1	1	1	1	1	0	0	1	1	1	1	1	1	1	1	1	1	1	1	1	1	1	1	1	1	1	0	1	1	1	1	1	1	1	1
180606	LMKITAEWLW	NS5	2864	2873	HLA-B*53:01	81% - <100%	Dengue virus 2	ELISPOT	IFNg release	Positive	1	0	1	1	1	1	1	1	0	0	0	1	1	1	1	1	1	1	1	1	0	1	1	1	1	1	1	1	1	1	1	1	1	1	1	1	1	1	1	1	0	1	1	1	1	1	1	1	0
180758	SWHYDQDHPYKTWAY	NS5	291	305	HLA class I	81% - <100%	Dengue virus 2	ELISPOT	IFNg release	Positive	1	1	0	1	1	1	1	1	1	1	1	1	1	1	1	1	1	1	1	1	1	1	1	1	1	1	1	1	1	1	1	1	1	1	1	1	1	1	1	1	1	1	1	1	1	1	1	1	1	1
180774	TLRVLNLVENWLNNN	NS5	2652	2666	HLA class I	81% - <100%	Dengue virus 2	ELISPOT	IFNg release	Positive	1	1	1	1	1	1	1	1	1	1	1	1	1	1	1	1	1	1	1	1	1	1	0	1	1	1	1	1	1	1	1	1	1	1	0	1	1	1	1	1	1	1	1	1	1	1	0	1	1
180828	YAQMWSLMYF	NS5	3249	3258	HLA-B*35:01	81% - <100%	Dengue virus 2	ELISPOT	IFNg release	Positive	1	1	1	1	1	1	1	1	1	1	1	1	1	1	1	1	1	1	1	1	0	0	1	1	1	1	1	1	1	1	1	1	1	1	1	1	1	1	1	1	1	1	1	1	1	1	1	1	1	1
184373	LGKKKTPRM	NS5	2876	2884	HLA-B*08:01	81% - <100%	Dengue virus 2	ELISPOT	IFNg release	Positive	1	1	1	0	1	1	1	1	1	1	1	1	1	1	1	1	1	1	1	1	1	1	1	1	1	1	1	1	1	1	1	1	1	1	1	1	1	1	1	1	1	1	1	1	1	1	1	1	1	1
184840	MLINRFTMR	NS5	2729	2737	HLA-B*08:01	81% - <100%	Dengue virus 2	ELISPOT	IFNg release	Positive	1	0	1	1	1	1	1	1	1	1	1	0	0	1	1	1	1	1	1	1	1	1	1	1	1	1	1	1	1	1	1	1	1	1	0	1	1	1	1	1	1	1	1	1	1	1	1	1	1	1
184957	MTTEDMLTVW	NS5	3295	3304	HLA-B*58:01	81% - <100%	Dengue virus 2	ICS	IFNg release	Positive	1	1	1	1	1	1	1	1	0	1	1	1	1	1	1	1	1	1	1	1	1	1	1	1	1	1	1	1	1	1	1	1	1	1	1	1	1	1	1	1	1	1	1	1	1	1	1	1	1	1
31747	KLAEAIFKL	NS5	3054	3062	HLA-A*02:01	81% - <100%	Dengue virus 2	ELISPOT	IFNg release	Positive-Low	1	1	1	1	1	1	1	1	1	1	1	1	1	1	1	1	1	1	1	1	0	1	1	1	1	1	1	1	1	1	1	1	1	1	1	1	1	1	1	1	1	1	1	1	0	1	1	1	1	1
539144	CVVKPLDDRFASALTAL	NS5	3156	3172	HLA class I	81% - <100%	Dengue virus 2	ICS	IFNg release	Positive	1	1	1	1	1	1	1	0	0	0	0	1	1	1	1	1	1	1	1	1	1	1	1	1	1	1	1	1	1	1	1	1	1	1	1	1	1	0	1	1	1	0	1	1	1	1	1	1	1	1
73428	YAQMWSLMY	NS5	3249	3257	HLA-B*15:01	81% - <100%	Dengue virus 2	ELISPOT	IFNg release	Positive	1	1	1	1	1	1	1	1	1	1	1	1	1	1	1	1	1	1	1	1	0	0	1	1	1	1	1	1	1	1	1	1	1	1	1	1	1	1	1	1	1	1	1	1	1	1	1	1	1	1
183766	ILNRRRRTA	C	94	102	HLA-B*08:01	51%-80%	Dengue virus 2	ELISPOT	IFNg release	Positive	0	1	1	0	0	0	0	0	0	0	0	1	1	1	1	1	1	1	1	1	0	1	1	1	1	0	1	0	0	1	0	0	1	1	1	0	1	1	0	1	1	1	1	1	0	1	1	0	1
184518	LNRRRRTAG	C	95	103	HLA-B*08:01	51%-80%	Dengue virus 2	ELISPOT	IFNg release	Positive	0	1	1	0	0	0	0	0	0	0	0	1	1	1	1	1	1	1	1	1	0	1	1	1	1	0	1	0	0	1	0	0	1	1	1	0	1	1	0	1	1	1	1	1	0	1	1	0	1
185104	NRRRRTAGV	C	96	104	HLA-B*08:01	51%-80%	Dengue virus 2	ELISPOT	IFNg release	Positive	0	1	1	0	0	0	0	0	0	0	0	1	1	1	1	1	1	1	1	1	0	1	1	1	1	0	1	0	0	1	0	0	1	1	1	0	1	0	0	1	0	1	1	1	0	1	0	0	0
124391	KTFVDLMRR	NS3	2006	2014	HLA-A*11:01	51%-80%	Dengue virus 2	ELISPOT	IFNg release	Positive	1	0	0	1	1	1	1	1	1	1	1	0	0	0	0	0	0	0	0	1	1	1	0	0	0	1	0	1	1	0	1	1	0	0	0	1	0	1	1	0	1	0	0	0	1	0	1	1	1
150167	DLMRRGDLPV	NS3	2010	2019	HLA-A*02:01	51%-80%	Dengue virus 2	ELISPOT	IFNg release	Positive	1	0	0	1	1	1	1	1	1	1	1	0	0	0	0	0	0	0	0	1	1	1	0	0	0	1	0	1	1	0	1	1	0	0	0	1	0	1	1	0	1	0	0	0	1	0	1	1	1
180471	GEARKTFVDL	NS3	2002	2011	HLA-B*40:01	51%-80%	Dengue virus 2	ELISPOT	IFNg release	Positive	1	0	0	1	1	1	1	1	1	1	1	0	0	0	0	0	0	0	0	1	1	1	0	0	0	1	0	1	1	0	1	1	0	0	0	1	0	1	1	0	1	0	0	0	1	0	1	1	1
180724	RGEARKTFVDLMRRG	NS3	2001	2015	HLA class I	51%-80%	Dengue virus 2	ELISPOT	IFNg release	Positive	1	0	0	1	1	1	1	1	1	1	1	0	0	0	0	0	0	0	0	1	1	1	0	0	0	1	0	1	1	0	1	1	0	0	0	1	0	1	1	0	1	0	0	0	1	0	1	1	1
182848	EARKTFVDL	NS3	2003	2011	HLA-B*08:01	51%-80%	Dengue virus 2	ELISPOT	IFNg release	Positive	1	0	0	1	1	1	1	1	1	1	1	0	0	0	0	0	0	0	0	1	1	1	0	0	0	1	0	1	1	0	1	1	0	0	0	1	0	1	1	0	1	0	0	0	1	0	1	1	1
183347	GERKKLKPRW	NS3	2061	2070	HLA-B*44:03	51%-80%	Dengue virus 2	ELISPOT	IFNg release	Positive	1	1	0	1	1	1	1	1	1	1	1	1	1	0	0	0	0	0	0	1	0	1	0	0	0	1	0	1	1	0	1	1	0	0	1	1	1	1	1	0	1	0	0	0	1	0	1	1	1
180668	MPSVIEKMET	NS5	2678	2687	HLA-B*53:01	51%-80%	Dengue virus 2	ELISPOT	IFNg release	Positive	0	1	1	1	0	0	0	0	0	0	0	1	1	1	1	1	1	1	1	1	1	1	1	1	1	0	1	0	0	1	0	0	1	1	1	0	1	1	0	1	0	1	1	1	0	1	0	0	0
180588	LAYTIGTTHF	PreM	254	263	HLA-B*35:01	>0% - 50%	Dengue virus 2	ELISPOT	IFNg release	Positive	0	0	0	0	0	0	0	0	0	0	0	0	0	0	0	0	0	0	0	0	0	0	0	0	0	0	0	1	1	0	1	1	0	0	0	0	1	1	1	0	0	0	0	0	0	0	1	0	0
180433	EPGQLKLNWF	E	663	672	HLA-B*35:01	>0% - 50%	Dengue virus 2	ELISPOT	IFNg release	Positive	0	0	0	0	0	0	0	0	0	0	0	0	0	0	0	0	0	0	0	0	0	0	0	0	0	0	0	0	0	0	0	0	0	0	0	0	0	1	0	0	0	0	0	0	0	0	0	0	0
184467	LLQMEDKAWL	E	478	487	HLA-B*08:01	>0% - 50%	Dengue virus 2	ELISPOT	IFNg release	Positive	0	0	0	0	0	0	0	0	0	0	0	0	0	0	0	0	0	0	0	0	0	0	0	0	0	0	0	0	0	0	0	0	0	0	0	0	0	1	0	0	0	0	0	0	0	0	0	0	0
150300	ILLVAVSFV	NS2a	1146	1154	HLA-A*02:01	>0% - 50%	Dengue virus 2	ELISPOT	IFNg release	Positive	0	0	0	0	0	0	0	0	0	0	0	0	0	0	0	0	0	0	0	0	0	0	0	0	0	0	0	0	0	0	0	0	0	0	0	0	0	1	0	0	0	0	0	0	0	0	0	0	0
150640	TMTDDIGMGV	NS2a	1176	1185	HLA-A*02:01	>0% - 50%	Dengue virus 2	ELISPOT	IFNg release	Positive	0	0	0	0	0	0	0	0	0	0	0	0	0	0	0	0	0	0	0	0	0	0	0	0	0	0	0	0	0	0	0	0	0	0	0	0	0	1	0	0	0	0	0	0	0	0	0	0	0
180717	RDLGRVMVMVGATMT	NS2a	51	65	HLA class I	>0% - 50%	Dengue virus 2	ICS	IFNg release	Positive	0	0	0	0	0	0	0	0	0	0	0	0	0	0	0	0	0	0	0	0	0	0	0	0	0	0	0	0	0	0	0	0	0	0	0	0	0	1	0	0	0	0	0	0	0	0	0	0	0
180735	RVGTKHAILLVAVSF	NS2a	1153	1167	HLA class I	>0% - 50%	Dengue virus 2	ICS	IFNg release	Positive	0	0	0	0	0	0	0	0	0	0	0	0	0	0	1	0	0	0	0	0	0	0	0	0	0	0	0	0	0	0	0	0	0	0	0	0	0	1	0	0	0	0	0	0	0	0	0	0	0
186041	TRVGTKHAIL	NS2a	1152	1161	HLA-B*08:01	>0% - 50%	Dengue virus 2	ELISPOT	IFNg release	Positive	0	0	0	0	0	0	0	0	0	0	0	0	0	0	1	0	0	0	0	0	0	0	0	0	0	0	0	0	0	0	0	0	0	0	0	0	0	1	0	0	0	0	0	0	0	0	0	0	0
10643	DVKKDLISY	NS3	71	79	HLA-B62	>0% - 50%	Dengue virus 2	51 chromium	cytotoxicity	Positive	0	0	0	0	0	0	0	0	0	0	0	0	0	0	0	0	0	0	0	0	0	0	0	0	0	0	0	0	0	0	0	0	0	0	0	0	0	1	0	0	0	0	0	0	0	0	0	0	0
150346	KTFDSEYVK	NS3	1863	1871	HLA-A*11:01	>0% - 50%	Dengue virus 2	multimer/tetramer	qualitative binding	Positive	0	0	0	0	0	0	0	0	0	0	0	0	0	0	0	0	0	0	0	0	0	0	0	0	0	0	0	0	0	0	0	0	0	0	0	0	0	1	0	0	0	0	0	0	0	0	0	0	0
17802	FSPGTSGSPIIDKKG	NS3	130	144	HLA class I	>0% - 50%	Dengue virus 2	ELISPOT	IFNg release	Positive	0	0	0	0	0	0	0	0	0	0	0	0	0	0	0	0	0	0	0	0	0	0	0	0	0	0	0	0	0	0	0	0	0	0	0	0	0	0	0	0	1	0	0	0	0	0	0	0	0
180623	LPVWLAYKV	NS3	2017	2025	HLA-B*51:01	>0% - 50%	Dengue virus 2	ELISPOT	IFNg release	Positive	0	0	0	0	0	0	0	0	0	0	0	0	0	0	0	0	0	0	0	0	0	0	0	0	0	0	0	0	0	0	0	0	0	0	0	0	0	1	0	0	0	0	0	0	0	0	0	0	0
180624	LPVWLAYKVA	NS3	2017	2026	HLA-B*35:01	>0% - 50%	Dengue virus 2	ELISPOT	IFNg release	Positive	0	0	0	0	0	0	0	0	0	0	0	0	0	0	0	0	0	0	0	0	0	0	0	0	0	0	0	0	0	0	0	0	0	0	0	0	0	1	0	0	0	0	0	0	0	0	0	0	0
180750	SPGTSGSPIIDKKGK	NS3	131	145	HLA-A*11:01	>0% - 50%	Dengue virus 2	ELISPOT	IFNg release	Positive	0	0	0	0	0	0	0	0	0	0	0	0	0	0	0	0	0	0	0	0	0	0	0	0	0	0	0	0	0	0	0	0	0	0	0	0	0	0	0	0	1	0	0	0	0	0	0	0	0
22818	GTSGSPIIDK	NS3	133	142	HLA-A*11:01	>0% - 50%	Dengue virus 2	ELISPOT	IFNg release	Positive	0	0	0	0	0	0	0	0	0	0	0	0	0	0	0	0	0	0	0	0	0	0	0	0	0	0	0	0	0	0	0	0	0	0	0	0	0	0	0	0	1	0	0	0	0	0	0	0	0
22819	GTSGSPIIDKK	NS3	133	143	HLA-A11	>0% - 50%	Dengue virus 2	51 chromium	cytotoxicity	Positive	0	0	0	0	0	0	0	0	0	0	0	0	0	0	0	0	0	0	0	0	0	0	0	0	0	0	0	0	0	0	0	0	0	0	0	0	0	0	0	0	1	0	0	0	0	0	0	0	0
25929	IEPSWADVKKDLISY	NS3	65	79	HLA class I	>0% - 50%	Dengue virus 2	ELISPOT	IFNg release	Positive	0	0	0	0	0	0	0	0	0	0	0	0	0	0	0	0	0	0	0	0	0	0	0	0	0	0	0	0	0	0	0	0	0	0	0	0	0	1	0	0	0	0	0	0	0	0	0	0	0
790	ADVKKDLISYGGGWK	NS3	70	84	HLA class I	>0% - 50%	Dengue virus 2	ELISPOT	IFNg release	Positive	0	0	0	0	0	0	0	0	0	0	0	0	0	0	0	0	0	0	0	0	0	0	0	0	0	0	0	0	0	0	0	0	0	0	0	0	0	1	0	0	0	0	0	0	0	0	0	0	0
16757	FLLVAHYAI	NS4b	112	120	HLA-A2	>0% - 50%	Dengue virus 2	ICS	TNF release	Positive	0	0	0	0	0	0	0	0	0	0	0	0	0	0	0	0	0	0	0	0	0	1	0	0	0	0	0	0	0	0	0	0	0	0	0	0	0	1	0	0	0	0	0	0	0	0	0	0	0
35568	LEKTKKDL	NS4b	2249	2256	HLA-B8	>0% - 50%	Dengue virus 2	ELISPOT	IFNg release	Positive	0	0	0	0	0	0	0	0	0	0	0	0	0	0	0	0	0	0	0	0	0	0	1	0	0	0	0	0	0	0	0	0	0	0	0	0	0	1	0	0	1	0	0	0	0	0	0	0	0
150554	RTTWSIHAK	NS5	3283	3291	HLA-A*11:01	>0% - 50%	Dengue virus 2	ELISPOT	IFNg release	Positive	1	0	0	1	1	1	1	1	1	1	1	0	0	0	0	0	0	0	0	0	0	0	0	0	0	1	0	1	1	0	1	1	0	0	0	1	0	0	1	0	0	0	0	0	0	0	0	1	1
161186	RLLTKPWDVVPMVTQ	NS5	326	340	HLA-B*55:02	>0% - 50%	Dengue virus 2	ICS	IFNg release	Positive	0	0	0	0	0	0	0	0	0	0	0	0	0	0	0	0	0	0	0	0	0	0	0	0	0	0	0	0	0	0	0	0	0	0	0	0	0	1	0	0	0	0	0	0	0	0	0	0	0
180681	NEEYTDYMPSMKRFR	NS5	3367	3381	HLA class I	>0% - 50%	Dengue virus 2	ELISPOT	IFNg release	Positive	1	0	0	1	1	1	1	1	1	1	1	0	0	0	0	0	0	0	0	0	1	0	0	0	0	1	0	1	1	0	1	1	0	0	0	1	0	1	1	0	1	0	0	0	1	0	0	1	1
182855	EAVEDGRFWE	NS5	2915	2924	HLA-B*58:01	>0% - 50%	Dengue virus 2	ELISPOT	IFNg release	Positive	0	0	0	0	0	0	0	0	0	0	0	0	0	0	0	0	0	0	0	0	0	0	0	0	0	0	0	0	0	0	0	0	0	0	0	0	0	1	0	0	0	0	0	0	0	0	0	0	0
186219	VEDGRFWEL	NS5	2917	2925	HLA-B*40:01	>0% - 50%	Dengue virus 2	ELISPOT	IFNg release	Positive	0	0	0	0	0	0	0	0	0	0	0	0	0	0	0	0	0	0	0	0	0	0	0	0	0	0	0	0	0	0	0	0	0	0	0	0	0	1	0	0	0	0	0	0	0	0	0	0	0
32943	KPWDVVPMV	NS5	2821	2829	HLA-B*51:01	>0% - 50%	Dengue virus 2	51 chromium	cytotoxicity	Positive	0	0	0	0	0	0	0	0	0	0	0	0	0	0	0	0	0	0	0	0	0	0	0	0	0	0	0	0	0	0	0	0	0	0	0	0	0	1	0	0	0	0	0	0	0	0	0	0	0
74305	YILRDVSKK	NS5	517	525	HLA class I	>0% - 50%	Dengue virus 2	ELISPOT	IFNg release	Positive	1	0	0	1	1	1	1	1	1	1	1	0	0	0	0	0	0	0	0	0	1	0	0	0	0	1	0	1	1	0	1	1	0	0	0	1	0	0	1	0	1	0	0	0	1	0	0	1	1

CD8 DENV-3 Epitopes

											NCBI IDs of DENV-3 circulating strains from India, (100% identity=1,green ; else 0, red)																			
Epitope_ID	AA_Seq	Protein_Name	Start	End	Allele	step	Serotype	Method/Technique	Assay Group	Qualitative Measure	ADM63678.1	AFZ40126.1	AFZ40127.1	ANC57607.1	ANC57612.1	QCZ25021.1	QCZ25020.1	QCZ25019.1	QCZ25016.1	QCZ25017.1	QBA57533.1	QEP41653.1	ACV04798.1	AXX75610.1	AXX75612.1	AXX75611.1	AML29863.1	AXX75613.1	QEP41661	QEP41655
180390	CLMMMLPATL	C	104	113	HLA-A*02:01	0%	Dengue virus 3	ELISPOT	IFNg release	Positive	0	0	0	0	0	0	0	0	0	0	0	0	0	0	0	0	0	0	0	0
180609	LMMMLPATL	C	105	113	HLA-A*02:01	0%	Dengue virus 3	ELISPOT	IFNg release	Positive	0	0	0	0	0	0	0	0	0	0	0	0	0	0	0	0	0	0	0	0
183793	IMKIGIGVLL	E	452	461	HLA-B*08:01	0%	Dengue virus 3	ELISPOT	IFNg release	Positive	0	0	0	0	0	0	0	0	0	0	0	0	0	0	0	0	0	0	0	0
184412	LKGMSYAMCL	E	156	165	HLA-B*08:01	0%	Dengue virus 3	ELISPOT	IFNg release	Positive	0	0	0	0	0	0	0	0	0	0	0	0	0	0	0	0	0	0	0	0
186470	WIMKIGIGVL	E	451	460	HLA-B*08:01	0%	Dengue virus 3	ELISPOT	IFNg release	Positive	0	0	0	0	0	0	0	0	0	0	0	0	0	0	0	0	0	0	0	0
185464	RPINEKEENM	NS1	1109	1118	HLA-B*35:01	0%	Dengue virus 3	ELISPOT	IFNg release	Positive	0	0	0	0	0	0	0	0	0	0	0	0	0	0	0	0	0	0	0	0
183395	GKKHMIAGVL	NS2a	1153	1162	HLA-B*08:01	0%	Dengue virus 3	ELISPOT	IFNg release	Positive	0	0	0	0	0	0	0	0	0	0	0	0	0	0	0	0	0	0	0	0
3124	AMKGLPIRY	NS3	1708	1716	HLA-B62	0%	Dengue virus 3	51 chromium	cytotoxicity	Positive	0	0	0	0	0	0	0	0	0	0	0	0	0	0	0	0	0	0	0	0
590435	TRVVAAEMEEAMKGLPI	NS3	1698	1714	HLA class I	0%	Dengue virus 3	ICS	IFNg release	Positive-Low	0	0	0	0	0	0	0	0	0	0	0	0	0	0	0	0	0	0	0	0
180572	KPRLCTREEF	NS5	2879	2888	HLA-B*07:02	0%	Dengue virus 3	ELISPOT	IFNg release	Positive	0	0	0	0	0	0	0	0	0	0	0	0	0	0	0	0	0	0	0	0
180801	VMGITAEWLW	NS5	2862	2871	HLA-B*53:01	0%	Dengue virus 3	ELISPOT	IFNg release	Positive	0	0	0	0	0	0	0	0	0	0	0	0	0	0	0	0	0	0	0	0
186127	TTWEDVPYL	NS5	3321	3329	HLA-A*02:01	0%	Dengue virus 3	ELISPOT	IFNg release	Positive	0	0	0	0	0	0	0	0	0	0	0	0	0	0	0	0	0	0	0	0
32944	KPWDVVPTV	NS5	2819	2827	HLA class I	0%	Dengue virus 3	51 chromium	cytotoxicity	Positive	0	0	0	0	0	0	0	0	0	0	0	0	0	0	0	0	0	0	0	0
180464	FTILALFLAH	PreM	247	256	HLA-B*35:01	100%	Dengue virus 3	ELISPOT	IFNg release	Positive	1	1	1	1	1	1	1	1	1	1	1	1	1	1	1	1	1	1	1	1
180504	HPGFTILALF	PreM	244	253	HLA-B*35:01	100%	Dengue virus 3	ELISPOT	IFNg release	Positive	1	1	1	1	1	1	1	1	1	1	1	1	1	1	1	1	1	1	1	1
185685	SLLFKTASG	PreM	136	144	HLA-B*08:01	100%	Dengue virus 3	ELISPOT	IFNg release	Positive	1	1	1	1	1	1	1	1	1	1	1	1	1	1	1	1	1	1	1	1
180613	LPEEQDQNY	E	82	90	HLA-B*35:01	100%	Dengue virus 3	ELISPOT	IFNg release	Positive	1	1	1	1	1	1	1	1	1	1	1	1	1	1	1	1	1	1	1	1
180657	MLVTPSMTM	E	273	281	HLA-B*35:01	100%	Dengue virus 3	ELISPOT	IFNg release	Positive	1	1	1	1	1	1	1	1	1	1	1	1	1	1	1	1	1	1	1	1
183843	IQNSGGTSIF	E	131	140	HLA-B*15:01	100%	Dengue virus 3	ELISPOT	IFNg release	Positive	1	1	1	1	1	1	1	1	1	1	1	1	1	1	1	1	1	1	1	1
184299	LATLRKLCI	E	53	61	HLA-B*08:01	100%	Dengue virus 3	ELISPOT	IFNg release	Positive	1	1	1	1	1	1	1	1	1	1	1	1	1	1	1	1	1	1	1	1
184536	LPEYGTLGLE	E	453	462	HLA-B*35:01	100%	Dengue virus 3	ELISPOT	IFNg release	Positive	1	1	1	1	1	1	1	1	1	1	1	1	1	1	1	1	1	1	1	1
185419	RLKMDKLEL	E	148	156	HLA-B*08:01	100%	Dengue virus 3	ELISPOT	IFNg release	Positive	1	1	1	1	1	1	1	1	1	1	1	1	1	1	1	1	1	1	1	1
150537	RPGYHTQTA	NS1	1030	1038	HLA-B*07:02	100%	Dengue virus 3	ELISPOT	IFNg release	Positive	1	1	1	1	1	1	1	1	1	1	1	1	1	1	1	1	1	1	1	1
150546	RSCTLPPLRY	NS1	1087	1096	HLA-A*01:01	100%	Dengue virus 3	ELISPOT	IFNg release	Positive	1	1	1	1	1	1	1	1	1	1	1	1	1	1	1	1	1	1	1	1
180738	RYMGEDGCWY	NS1	1095	1104	HLA-A*24:02	100%	Dengue virus 3	ELISPOT	IFNg release	Positive	1	1	1	1	1	1	1	1	1	1	1	1	1	1	1	1	1	1	1	1
182939	EKEENMVKSL	NS1	1113	1122	HLA-B*08:01	100%	Dengue virus 3	ELISPOT	IFNg release	Positive	1	1	1	1	1	1	1	1	1	1	1	1	1	1	1	1	1	1	1	1
184585	LRTTTVSGKL	NS1	64	73	HLA-B*08:01	100%	Dengue virus 3	ELISPOT	IFNg release	Positive	1	1	1	1	1	1	1	1	1	1	1	1	1	1	1	1	1	1	1	1
185573	RYMGEDGCW	NS1	1095	1103	HLA-A*24:02	100%	Dengue virus 3	ELISPOT	IFNg release	Positive	1	1	1	1	1	1	1	1	1	1	1	1	1	1	1	1	1	1	1	1
185734	SPKRLATAI	NS1	811	819	HLA-B*08:01	100%	Dengue virus 3	ELISPOT	IFNg release	Positive	1	1	1	1	1	1	1	1	1	1	1	1	1	1	1	1	1	1	1	1
186175	TWRDMAHTLI	NS2a	1174	1183	HLA-A*24:02	100%	Dengue virus 3	ELISPOT	IFNg release	Positive	1	1	1	1	1	1	1	1	1	1	1	1	1	1	1	1	1	1	1	1
182917	EFKDFAAGR	NS3	2083	2091	HLA-A*33:01	100%	Dengue virus 3	ELISPOT	IFNg release	Positive	1	1	1	1	1	1	1	1	1	1	1	1	1	1	1	1	1	1	1	1
186234	VEIWTKEGEK	NS3	2053	2062	HLA-B*40:01	100%	Dengue virus 3	ELISPOT	IFNg release	Positive	1	1	1	1	1	1	1	1	1	1	1	1	1	1	1	1	1	1	1	1
19434	GESRKTFVE	NS3	2001	2009	HLA-B7	100%	Dengue virus 3	51 chromium	cytotoxicity	Positive	1	1	1	1	1	1	1	1	1	1	1	1	1	1	1	1	1	1	1	1
19435	GESRKTFVEL	NS3	211	220	HLA-B*40:01	100%	Dengue virus 3	ICS	IFNg release	Positive	1	1	1	1	1	1	1	1	1	1	1	1	1	1	1	1	1	1	1	1
22820	GTSGSPIINR	NS3	133	142	HLA-A*11:01	100%	Dengue virus 3	51 chromium	cytotoxicity	Positive	1	1	1	1	1	1	1	1	1	1	1	1	1	1	1	1	1	1	1	1
22821	GTSGSPIINRE	NS3	133	143	HLA-A11	100%	Dengue virus 3	ICS	IFNg release	Positive	1	1	1	1	1	1	1	1	1	1	1	1	1	1	1	1	1	1	1	1
34605	KYTDRKWCF	NS3	240	248	HLA-A24	100%	Dengue virus 3	ELISPOT	IFNg release	Positive	1	1	1	1	1	1	1	1	1	1	1	1	1	1	1	1	1	1	1	1
62183	SVKKDLISY	NS3	71	79	HLA-B62	100%	Dengue virus 3	51 chromium	cytotoxicity	Positive	1	1	1	1	1	1	1	1	1	1	1	1	1	1	1	1	1	1	1	1
183165	FFMMVLLIPE	NS4a	2205	2214	HLA-A*33:01	100%	Dengue virus 3	ELISPOT	IFNg release	Positive	1	1	1	1	1	1	1	1	1	1	1	1	1	1	1	1	1	1	1	1
183796	IMKSVGTGK	NS4b	2481	2489	HLA-A*03:01	100%	Dengue virus 3	ELISPOT	IFNg release	Positive	1	1	1	1	1	1	1	1	1	1	1	1	1	1	1	1	1	1	1	1
37534	LLLMRTSWA	NS4b	2423	2431	HLA-A2	100%	Dengue virus 3	biological activity	proliferation	Positive	1	1	1	1	1	1	1	1	1	1	1	1	1	1	1	1	1	1	1	1
1067501	KPIDDRFAN	NS5	3158	3166	HLA-B*07:02	100%	Dengue virus 3	ELISPOT	IFNg release	Positive	1	1	1	1	1	1	1	1	1	1	1	1	1	1	1	1	1	1	1	1
180650	MEITAEWLW	NS5	2863	2871	HLA-B*58:01	100%	Dengue virus 3	ELISPOT	IFNg release	Positive	1	1	1	1	1	1	1	1	1	1	1	1	1	1	1	1	1	1	1	1
180728	RPRLCTREEF	NS5	2879	2888	HLA-B*07:02	100%	Dengue virus 3	ELISPOT	IFNg release	Positive	1	1	1	1	1	1	1	1	1	1	1	1	1	1	1	1	1	1	1	1
182553	ALLALNDMGK	NS5	3167	3176	HLA-A*03:01	100%	Dengue virus 3	ELISPOT	IFNg release	Positive	1	1	1	1	1	1	1	1	1	1	1	1	1	1	1	1	1	1	1	1
185710	SMINGVVKL	NS5	318	326	HLA-A*02:01	100%	Dengue virus 3	ELISPOT	IFNg release	Positive	1	1	1	1	1	1	1	1	1	1	1	1	1	1	1	1	1	1	1	1
180577	KSGAIKVLK	C	74	82	HLA-A*11:01	81% - <100%	Dengue virus 3	ELISPOT	IFNg release	Positive	1	1	1	1	0	1	1	1	1	1	1	1	1	1	1	1	1	1	1	1
184260	LAIPPTAGVL	C	57	66	HLA-B*08:01	81% - <100%	Dengue virus 3	ELISPOT	IFNg release	Positive	1	1	1	1	1	1	1	1	1	1	1	1	1	1	0	1	1	1	1	1
184845	MLKRVRNRV	C	15	23	HLA-B*08:01	81% - <100%	Dengue virus 3	ELISPOT	IFNg release	Positive	1	1	1	1	1	1	1	1	1	1	1	1	1	1	1	1	1	1	0	1
184658	LTQKVVIFIL	PreM	262	271	HLA-B*08:01	81% - <100%	Dengue virus 3	ELISPOT	IFNg release	Positive	1	1	1	1	1	1	1	1	1	1	1	1	1	1	0	1	1	1	1	1
13119	ELKGMSYAM	E	155	163	HLA-B*08:01	81% - <100%	Dengue virus 3	ELISPOT	IFNg release	Positive	1	1	1	1	0	1	1	1	1	1	1	1	1	1	1	1	1	1	1	1
15796	FFDLPLPWT	E	211	219	HLA-A2	81% - <100%	Dengue virus 3	ICS	IFNg release	Positive	1	1	1	1	1	1	1	1	1	1	1	1	1	0	1	1	1	1	1	1
184568	LPWTSGATT	E	496	504	HLA-B*35:01	81% - <100%	Dengue virus 3	ELISPOT	IFNg release	Positive	1	1	1	1	1	1	1	1	1	1	1	1	1	0	0	1	1	1	1	1
184924	MSYAMCTNTF	E	575	584	HLA-B*35:01	81% - <100%	Dengue virus 3	ELISPOT	IFNg release	Positive	1	1	1	1	0	1	1	1	1	1	1	1	1	1	1	1	1	1	1	1
184965	MVHQIFGSAY	E	713	722	HLA-B*15:01	81% - <100%	Dengue virus 3	ELISPOT	IFNg release	Positive	1	1	1	1	0	1	1	1	1	1	1	1	1	1	1	0	1	1	1	1
186029	TPTWNRKEL	E	226	234	HLA-B*07:02	81% - <100%	Dengue virus 3	ELISPOT	IFNg release	Positive-Low	1	1	1	0	1	1	1	1	1	1	1	1	1	1	0	1	1	1	1	1
589817	SGATWVDVVLEHGGCV	E	268	283	HLA class I	81% - <100%	Dengue virus 3	ICS	IFNg release	Positive-High	1	1	1	1	1	1	1	1	1	1	1	1	1	1	0	1	0	1	1	1
184254	LAILFEEVM	NS2a	1140	1148	HLA-B*35:01	81% - <100%	Dengue virus 3	ELISPOT	IFNg release	Positive	1	1	1	0	1	1	1	1	1	1	1	1	1	0	1	1	1	0	1	1
185978	TMGVLCLAIL	NS2a	1134	1143	HLA-B*08:01	81% - <100%	Dengue virus 3	ELISPOT	IFNg release	Positive	0	1	1	0	1	1	1	1	1	1	1	1	0	1	1	1	1	1	1	1
180622	LPVWLAHKVA	NS3	226	235	HLA-B*35:01	81% - <100%	Dengue virus 3	ELISPOT	IFNg release	Positive	1	1	1	1	1	1	1	1	1	1	1	1	1	1	1	1	0	1	1	1
184065	KLNDWDFVV	NS3	399	407	HLA-A*02:01	81% - <100%	Dengue virus 3	ELISPOT	IFNg release	Positive-High	1	0	1	1	1	1	1	1	1	1	1	1	1	1	1	1	1	1	1	1
37494	LLLGLMILL	NS4a	2148	2156	HLA-A2	81% - <100%	Dengue virus 3	ICS	IFNg release	Positive	1	1	1	1	1	1	1	0	1	1	1	1	1	1	1	1	1	1	1	1
180381	ATGPITTLW	NS4b	2439	2447	HLA-B*58:01	81% - <100%	Dengue virus 3	ELISPOT	IFNg release	Positive	1	1	0	1	1	1	1	1	1	1	1	1	1	1	1	1	1	1	1	1
180788	TTKRDLGMSK	NS4b	2250	2259	HLA-A*11:01	81% - <100%	Dengue virus 3	ELISPOT	IFNg release	Positive	1	0	1	1	1	1	1	1	1	1	1	1	1	1	1	1	1	1	1	1
180737	RYLEFEALGF	NS5	2971	2980	HLA-A*24:02	81% - <100%	Dengue virus 3	ELISPOT	IFNg release	Positive	1	1	1	1	0	1	1	1	1	1	1	1	1	0	1	1	1	1	1	1
183587	HMIAGVFFTF	NS2a	368	377	HLA-A*24:02	51%-80%	Dengue virus 3	ELISPOT	IFNg release	Positive	1	1	1	1	0	0	1	1	1	1	0	0	1	0	1	1	1	1	0	0
184819	MIAGVFFTF	NS2a	369	377	HLA-B*35:01	51%-80%	Dengue virus 3	ELISPOT	IFNg release	Positive	1	1	1	1	0	0	1	1	1	1	0	0	1	0	1	1	1	1	0	0
180678	MVSRLLLNR	NS5	2723	2731	HLA-A*11:01	51%-80%	Dengue virus 3	ELISPOT	IFNg release	Positive	1	1	1	0	1	0	1	1	1	1	0	0	1	1	1	1	1	1	0	0
184264	LAKRFSRGL	C	29	37	HLA-B*08:01	>0% - 50%	Dengue virus 3	ELISPOT	IFNg release	Positive	0	0	0	0	0	0	0	0	0	0	0	0	0	1	0	0	0	0	0	0
184265	LAKRFSRGLL	C	29	38	HLA-B*08:01	>0% - 50%	Dengue virus 3	ELISPOT	IFNg release	Positive	0	0	0	0	0	0	0	0	0	0	0	0	0	1	0	0	0	0	0	0
185260	QLAKRFSRG	C	28	36	HLA-B*08:01	>0% - 50%	Dengue virus 3	ELISPOT	IFNg release	Positive	0	0	0	0	0	0	0	0	0	0	0	0	0	1	0	0	0	0	0	0
183723	IKLTVVVGDI	NS1	857	866	HLA-B*08:01	>0% - 50%	Dengue virus 3	ELISPOT	IFNg release	Positive	0	0	0	0	0	0	0	0	1	0	0	0	0	0	0	0	0	0	0	0
69612	VLLLVTHYA	NS4b	2353	2361	HLA-A2	>0% - 50%	Dengue virus 3	ICS	IFNg release	Positive	0	0	0	0	0	1	1	1	0	1	1	1	0	0	0	1	0	0	1	1
186215	VEDEDFWKL	NS5	2915	2923	HLA-B*40:01	>0% - 50%	Dengue virus 3	ELISPOT	IFNg release	Positive	0	0	0	0	0	0	0	0	0	0	0	1	0	0	0	0	0	0	1	1

CD8 DENV-4 Epitopes

											NCBI IDs of DENV-4 circulating strains from India, (100% identity=1,green ; else 0, red)								
Epitope_ID	AA_Seq	Protein_Name	Start	End	Allele	step	Serotype	Method/Technique	Assay Group	Qualitative Measure	AVW85694.1	AVW85695.1	AVW85696.1	AFZ40130.1	ANC57613.1	QBA57536.1	QCE20687.1	ARB18129.1	QDZ58859
180810	VTYECPLLV	PreM	162	170	HLA-A*02:01	0%	Dengue virus 4	ELISPOT	IFNg release	Positive	0	0	0	0	0	0	0	0	0
182707	CNLTSTWVMY	PreM	181	190	HLA-A*30:02	0%	Dengue virus 4	ELISPOT	IFNg release	Positive	0	0	0	0	0	0	0	0	0
186303	VLMMLVAPSY	PreM	17	26	HLA-B*15:01	0%	Dengue virus 4	ELISPOT	IFNg release	Positive	0	0	0	0	0	0	0	0	0
185147	NYKERMVTF	E	511	519	HLA-A*24:02	0%	Dengue virus 4	ELISPOT	IFNg release	Positive	0	0	0	0	0	0	0	0	0
185817	SWMIRILIGF	E	161	170	HLA-A*24:02	0%	Dengue virus 4	ELISPOT	IFNg release	Positive	0	0	0	0	0	0	0	0	0
240130	PPASDLKYSW	NS1	880	889	HLA-B*53:01	0%	Dengue virus 4	ELISPOT	IFNg release	Positive	0	0	0	0	0	0	0	0	0
167725	AMTTTLSIPHDLMELIDGIS	NS2a	50	69	HLA class I	0%	Dengue virus 4	ELISPOT	IFNg release	Positive	0	0	0	0	0	0	0	0	0
239411	ALWYVWQVK	NS2b	1463	1471	HLA-A*03:01	0%	Dengue virus 4	ELISPOT	IFNg release	Positive	0	0	0	0	0	0	0	0	0
418991	IPVTMALWY	NS2b	1458	1466	HLA-B*35:01	0%	Dengue virus 4	ELISPOT	IFNg release	Positive	0	0	0	0	0	0	0	0	0
180640	MALKDFKEF	NS3	2079	2087	HLA-B*35:01	0%	Dengue virus 4	ELISPOT	IFNg release	Positive	0	0	0	0	0	0	0	0	0
182563	ALSEGVYRI	NS3	1491	1499	HLA-A*02:01	0%	Dengue virus 4	ELISPOT	IFNg release	Positive	0	0	0	0	0	0	0	0	0
186008	TPPGATDPF	NS3	1791	1799	HLA-B*35:01	0%	Dengue virus 4	ELISPOT	IFNg release	Positive	0	0	0	0	0	0	0	0	0
186602	YSDPLALRE	NS3	2077	2085	HLA-A*01:01	0%	Dengue virus 4	ELISPOT	IFNg release	Positive	0	0	0	0	0	0	0	0	0
239768	GTSGSPIINK	NS3	1607	1616	HLA-A*03:01	0%	Dengue virus 4	ELISPOT	IFNg release	Positive	0	0	0	0	0	0	0	0	0
180383	ATGPVLTLW	NS4b	2436	2444	HLA-B*58:01	0%	Dengue virus 4	ELISPOT	IFNg release	Positive	0	0	0	0	0	0	0	0	0
180587	LATGPVLTLW	NS4b	2435	2444	HLA-B*53:01	0%	Dengue virus 4	ELISPOT	IFNg release	Positive	0	0	0	0	0	0	0	0	0
184375	LGKSYAQMW	NS5	3246	3254	HLA-B*57:01	0%	Dengue virus 4	ELISPOT	IFNg release	Positive	0	0	0	0	0	0	0	0	0
419017	MPAMKRYSAP	NS5	3371	3380	HLA-B*35:01	0%	Dengue virus 4	ELISPOT	IFNg release	Positive	0	0	0	0	0	0	0	0	0
419048	SPRLCTREEF	NS5	2877	2886	HLA-B*07:02	0%	Dengue virus 4	ELISPOT	IFNg release	Positive	0	0	0	0	0	0	0	0	0
182971	EMCEDTVTY	PreM	156	164	HLA-B*35:01	100%	Dengue virus 4	ELISPOT	IFNg release	Positive	1	1	1	1	1	1	1	1	1
583701	GVSGGAWVDLVLEHGGCV	E	14	31	HLA class I	100%	Dengue virus 4	ICS	IFNg release	Positive-Low	1	1	1	1	1	1	1	1	1
183667	IEKASLIEV	NS1	986	994	HLA-B*40:01	100%	Dengue virus 4	ELISPOT	IFNg release	Positive	1	1	1	1	1	1	1	1	1
185050	NFLEVEDYGF	NS1	925	934	HLA-A*24:02	100%	Dengue virus 4	ELISPOT	IFNg release	Positive	1	1	1	1	1	1	1	1	1
182562	ALPVYLMTL	NS2a	1329	1337	HLA-A*02:01	100%	Dengue virus 4	ELISPOT	IFNg release	Positive	1	1	1	1	1	1	1	1	1
184566	LPVYLMTLMK	NS2a	1330	1339	HLA-B*35:01	100%	Dengue virus 4	ELISPOT	IFNg release	Positive	1	1	1	1	1	1	1	1	1
182901	EETNMITLL	NS2b	1434	1442	HLA-B*40:01	100%	Dengue virus 4	ELISPOT	IFNg release	Positive	1	1	1	1	1	1	1	1	1
186584	YPLAIPVTM	NS2b	1454	1462	HLA-B*53:01	100%	Dengue virus 4	ELISPOT	IFNg release	Positive	1	1	1	1	1	1	1	1	1
156711	LAPTRVVAAEMEEAL	NS3	1695	1709	HLA-B7	100%	Dengue virus 4	51 chromium	cytotoxicity	Positive	1	1	1	1	1	1	1	1	1
180473	GEFRLRGEQR	NS3	1995	2004	HLA-B*40:01	100%	Dengue virus 4	ELISPOT	IFNg release	Positive	1	1	1	1	1	1	1	1	1
180648	MEGVFHTMW	NS3	1516	1524	HLA-B*44:03	100%	Dengue virus 4	ELISPOT	IFNg release	Positive	1	1	1	1	1	1	1	1	1
180778	TPEGIIPTLF	NS3	1974	1983	HLA-B*07:02	100%	Dengue virus 4	ELISPOT	IFNg release	Positive	1	1	1	1	1	1	1	1	1
180843	YPKTKLTDWD	NS3	1868	1877	HLA-B*35:01	100%	Dengue virus 4	ELISPOT	IFNg release	Positive	1	1	1	1	1	1	1	1	1
182919	EFRLRGEQR	NS3	1996	2004	HLA-A*33:01	100%	Dengue virus 4	ELISPOT	IFNg release	Positive	1	1	1	1	1	1	1	1	1
183407	GLFGKTQVGV	NS3	1503	1512	HLA-A*02:01	100%	Dengue virus 4	ELISPOT	IFNg release	Positive	1	1	1	1	1	1	1	1	1
183427	GLYGNGVVTK	NS3	1622	1631	HLA-A*03:01	100%	Dengue virus 4	ELISPOT	IFNg release	Positive	1	1	1	1	1	1	1	1	1
184079	KLTDWDFVV	NS3	398	406	HLA-A*02:01	100%	Dengue virus 4	ELISPOT	IFNg release	Positive	1	1	1	1	1	1	1	1	1
186347	VPNYNLIVM	NS3	1749	1757	HLA-B*35:01	100%	Dengue virus 4	ELISPOT	IFNg release	Positive	1	1	1	1	1	1	1	1	1
195838	PNYNLIVMDEAHFTD	NS3	1750	1764	HLA class I	100%	Dengue virus 4	ICS	IFNg release	Positive	1	1	1	1	1	1	1	1	1
22822	GTSGSPIINRK	NS3	133	143	HLA-A11	100%	Dengue virus 4	51 chromium	cytotoxicity	Positive	1	1	1	1	1	1	1	1	1
34918	LAPTRVVAAEME	NS3	221	232	HLA-B7	100%	Dengue virus 4	51 chromium	cytotoxicity	Positive	1	1	1	1	1	1	1	1	1
539058	AHFTDPSSVAARGYIST	NS3	1760	1776	HLA class I	100%	Dengue virus 4	ICS	IFNg release	Positive	1	1	1	1	1	1	1	1	1
539576	LIVMDEAHFTDPSSVAA	NS3	1754	1770	HLA class I	100%	Dengue virus 4	ICS	IFNg release	Positive	1	1	1	1	1	1	1	1	1
540029	VEMGEAAAIFMTATPPG	NS3	1778	1794	HLA class I	100%	Dengue virus 4	ICS	IFNg release	Positive	1	1	1	1	1	1	1	1	1
581940	APTRVVAAEMEEALRGL	NS3	1558	1574	HLA class I	100%	Dengue virus 4	ICS	IFNg release	Positive-Low	1	1	1	1	1	1	1	1	1
65552	TPEGIIPTL	NS3	500	508	HLA-B*35:01	100%	Dengue virus 4	51 chromium	cytotoxicity	Positive	1	1	1	1	1	1	1	1	1
182926	EIASLPTYL	NS4a	2101	2109	HLA-A*68:02	100%	Dengue virus 4	ELISPOT	IFNg release	Positive	1	1	1	1	1	1	1	1	1
184531	LPESLETLML	NS4a	2140	2149	HLA-B*35:01	100%	Dengue virus 4	ELISPOT	IFNg release	Positive	1	1	1	1	1	1	1	1	1
185881	TEIASLPTYL	NS4a	2100	2109	HLA-B*40:01	100%	Dengue virus 4	ELISPOT	IFNg release	Positive	1	1	1	1	1	1	1	1	1
186620	YVILTILTI	NS4a	2228	2236	HLA-A*24:02	100%	Dengue virus 4	ELISPOT	IFNg release	Positive	1	1	1	1	1	1	1	1	1
150647	TPMLRHTIEN	NS4b	2288	2297	HLA-B*07:02	100%	Dengue virus 4	ELISPOT	IFNg release	Positive	1	1	1	1	1	1	1	1	1
180379	ATGPILTLW	NS4b	2436	2444	HLA-B*58:01	100%	Dengue virus 4	ELISPOT	IFNg release	Positive	1	1	1	1	1	1	1	1	1
180764	TETTILDVDL	NS4b	2262	2271	HLA-B*40:01	100%	Dengue virus 4	ELISPOT	IFNg release	Positive	1	1	1	1	1	1	1	1	1
180803	VPLLAMGCY	NS4b	2330	2338	HLA-B*35:01	100%	Dengue virus 4	ELISPOT	IFNg release	Positive	1	1	1	1	1	1	1	1	1
183672	IENTSANLSL	NS4b	2295	2304	HLA-B*40:01	100%	Dengue virus 4	ELISPOT	IFNg release	Positive	1	1	1	1	1	1	1	1	1
184453	LLLMRTTWAF	NS4b	2420	2429	HLA-A*24:02	100%	Dengue virus 4	ELISPOT	IFNg release	Positive	1	1	1	1	1	1	1	1	1
184713	LYAVATTIL	NS4b	2279	2287	HLA-A*24:02	100%	Dengue virus 4	ELISPOT	IFNg release	Positive	1	1	1	1	1	1	1	1	1
186330	VMLLVHYAI	NS4b	2351	2359	HLA-A*24:02	100%	Dengue virus 4	ELISPOT	IFNg release	Positive	1	1	1	1	1	1	1	1	1

37535	LLLMRTTWA	NS4b	76	84	HLA-A2	100%	Dengue virus 4	ICS	TNF release	Positive	1	1	1	1	1	1	1	1	1
40472	LVMLLVHYA	NS4b	6	14	HLA-A2	100%	Dengue virus 4	ICS	IFNg release	Positive	1	1	1	1	1	1	1	1	1
1067215	HSWEDIPYL	NS5	3319	3327	HLA-A*02:01	100%	Dengue virus 4	ELISPOT	IFNg release	Positive	1	1	1	1	1	1	1	1	1
180684	NPRLCTREEF	NS5	2877	2886	HLA-B*07:02	100%	Dengue virus 4	ELISPOT	IFNg release	Positive	1	1	1	1	1	1	1	1	1
180718	REDLWCGSL	NS5	3330	3338	HLA-B*40:01	100%	Dengue virus 4	ELISPOT	IFNg release	Positive	1	1	1	1	1	1	1	1	1
183353	GESSSNPTI	NS5	2635	2643	HLA-B*44:02	100%	Dengue virus 4	ELISPOT	IFNg release	Positive	1	1	1	1	1	1	1	1	1
183570	HEMYWVSGV	NS5	2703	2711	HLA-B*44:02	100%	Dengue virus 4	ELISPOT	IFNg release	Positive	1	1	1	1	1	1	1	1	1
184051	KLGEFGRAK	NS5	2948	2956	HLA-A*03:01	100%	Dengue virus 4	ELISPOT	IFNg release	Positive	1	1	1	1	1	1	1	1	1
184130	KREDLWCGSL	NS5	3329	3338	HLA-B*08:01	100%	Dengue virus 4	ELISPOT	IFNg release	Positive	1	1	1	1	1	1	1	1	1
184954	MTTEDMLKVW	NS5	3292	3301	HLA class I	100%	Dengue virus 4	ICS	IFNg release	Positive	1	1	1	1	1	1	1	1	1
186453	WEPSKGWKNW	NS5	3182	3191	HLA-B*44:02	100%	Dengue virus 4	ELISPOT	IFNg release	Positive	1	1	1	1	1	1	1	1	1
186550	YDQENPYRTW	NS5	2781	2790	HLA-B*44:02	100%	Dengue virus 4	ELISPOT	IFNg release	Positive	1	1	1	1	1	1	1	1	1
186640	YYMATLKNV	NS5	2576	2584	HLA-A*24:02	100%	Dengue virus 4	ELISPOT	IFNg release	Positive	1	1	1	1	1	1	1	1	1
186641	YYMATLKNVT	NS5	2576	2585	HLA-A*24:02	100%	Dengue virus 4	ELISPOT	IFNg release	Positive	1	1	1	1	1	1	1	1	1
539656	MAISGDDCVVKPLDERF	NS5	3146	3162	HLA class I	100%	Dengue virus 4	ICS	IFNg release	Positive	1	1	1	1	1	1	1	1	1
539703	MVTQLAMTDTTPFGQQR	NS5	2824	2840	HLA class I	100%	Dengue virus 4	ICS	IFNg release	Positive	1	1	1	1	1	1	1	1	1
180475	GEQRKTFVEL	NS3	2001	2010	HLA-B*40:01	81% - <100%	Dengue virus 4	ELISPOT	IFNg release	Positive	1	1	1	1	1	1	0	1	1
183231	FPQSNSPIED	NS3	1799	1808	HLA-B*35:01	81% - <100%	Dengue virus 4	ELISPOT	IFNg release	Positive	1	1	1	1	1	1	0	1	1
183338	GEIGAVTLDF	NS3	1595	1604	HLA-B*44:03	81% - <100%	Dengue virus 4	ELISPOT	IFNg release	Positive	1	1	1	1	0	1	1	1	1
62616	SYKDREWCF	NS3	389	397	HLA-A24	81% - <100%	Dengue virus 4	ELISPOT	IFNg release	Positive	1	1	1	1	1	1	0	1	1
180596	LETLMLVALL	NS4a	2144	2153	HLA-B*40:01	81% - <100%	Dengue virus 4	ELISPOT	IFNg release	Positive	1	1	1	0	1	1	1	1	1
182508	AIAVASGLLW	NS4a	2179	2188	HLA-B*44:02	81% - <100%	Dengue virus 4	ELISPOT	IFNg release	Positive	1	1	1	1	0	1	1	1	1
183650	IAVASGLLW	NS4a	2180	2188	HLA-B*57:01	81% - <100%	Dengue virus 4	ELISPOT	IFNg release	Positive	1	1	1	1	0	1	1	1	1
42083	MLVALLGAM	NS4a	56	64	HLA-A2	81% - <100%	Dengue virus 4	ICS	IFNg release	Positive	1	1	1	0	1	1	1	1	1
136826	CYSQVNPTTL	NS4b	2337	2346	HLA-A*24:02	81% - <100%	Dengue virus 4	ELISPOT	IFNg release	Positive	1	1	1	1	0	1	1	1	1
150548	RSNAAIGAVF	NS5	2891	2900	HLA-B*15:01	81% - <100%	Dengue virus 4	ELISPOT	IFNg release	Positive	1	1	1	0	1	1	1	1	1
185232	QEEHKETWHY	NS5	2772	2781	HLA-B*44:02	81% - <100%	Dengue virus 4	ELISPOT	IFNg release	Positive	1	1	1	1	0	1	1	1	1
539149	DCVVKPLDERFSTSLLF	NS5	3152	3168	HLA class I	81% - <100%	Dengue virus 4	ICS	IFNg release	Positive	1	1	1	1	0	1	1	1	1
184591	LSIPHDLMEF	NS2a	1239	1248	HLA-A*01:01	51%-80%	Dengue virus 4	ELISPOT	IFNg release	Positive	0	1	1	1	1	0	0	1	0
180850	YTPEGIIPTL	NS3	1973	1982	HLA-A*02:06	51%-80%	Dengue virus 4	ELISPOT	IFNg release	Positive	0	1	0	1	1	1	1	1	1
180541	ITLLCLIPTV	C	102	111	HLA-A*02:01	>0% - 50%	Dengue virus 4	ELISPOT	IFNg release	Positive	1	0	0	1	1	0	0	0	0
183818	IPHDLMELI	NS2a	1241	1249	HLA-B*53:01	>0% - 50%	Dengue virus 4	ELISPOT	IFNg release	Positive	1	0	0	0	0	1	1	0	1
184390	LIDGISLGL	NS2a	1248	1256	HLA-A*01:01	>0% - 50%	Dengue virus 4	ELISPOT	IFNg release	Positive	1	0	0	0	0	1	1	0	1

Supplementary Table S4. Globally reported dengue specific CD4 and CD8 T cell epitopes that are present in each of the six different dengue vaccine candidates*. Detailed information of the full-length sequences of the vaccine constructs for each of the six vaccines and all four serotypes within each vaccine is provided. Next, portion provides the identity of each of the globally reported CD4 epitopes and CD8 epitopes in each of the six vaccine candidates. Green color shade and number 1 indicates 100% identity in the given vaccine construct. Pink color shade and number zero indicates not identical in the given vaccine construct.*

Vaccine Information

Vaccine	Virus Type	Strain name	NCBI ID	Country	Complete Sequence	AA_Seq
TV003	DENV-1	Nauru Island Western Pacific 45AZ5	NP_059433.1	N/A	Yes	MNNQRKKTGRPSFNMLKRARNRVSTVSQLAKRFSKGLLSGQGPMKLVMAFIAFLRFLAIPPTAGILARWGSFKKNGAIKVLRGFKKEISNMLNIMNRRKRSVTMLLMLLPTALAFHLTTRGGEPHMIVSKQERGKSLLFKTSAGVNMCTLIAMDLGELCEDTMTYKCPRITETEPDDVDCWCNATETWVTYGTCSQTGEHRRDKRSVALAPHVGLGLETRTETWMSSEGAWKQIQKVETWALRHPGFTVIALFLAHAIGTSITQKGIIFILLMLVTPSMAMRCVGIGNRDFVEGLSGATWVDVVLEHGSCVTTMAKDKPTLDIELLKTEVTNPAVLRKLCIEAKISNTTTDSRCPTQGEATLVEEQDTNFVCRRTFVDRGWGNGCGLFGKGSLITCAKFKCVTKLEGKIVQYENLKYSVIVTVHTGDQHQVGNETTEHGTTATITPQAPTSEIQLTDYGALTLDCSPRTGLDFNEMVLLTMKKKSWLVHKQWFLDLPLPWTSGASTSQETWNRQDLLVTFKTAHAKKQEVVVLGSQEGAMHTALTGATEIQTSGTTTIFAGHLKCRLKMDKLILKGMSYVMCTGSFKLEKEVAETQHGTVLVQVKYEGTDAPCKIPFSSQDEKGVTQNGRLITANPIVTDKEKPVNIEAEPPFGESYIVVGAGEKALKLSWFKKGSSIGKMFEATARGARRMAILGDTAWDFGSIGGVFTSVGKLIHQIFGTAYGVLFSGVSWTMKIGIGILLTWLGLNSRSTSLSMTCIAVGMVTLYLGVMVQADSGCVINWKGRELKCGSGIFVTNEVHTWTEQYKFQADSPKRLSAAIGKAWEEGVCGIRSATRLENIMWKQISNELNHILLENDMKFTVVVGDVSGILAQGKKMIRPQPMEHKYSWKSWGKAKIIGADVQNTTFIIDGPNTPECPDNQRAWNIWEVEDYGFGIFTTNIWLKLRDSYTQVCDHRLMSAAIKDSKAVHADMGYWIESEKNETWKLARASFIEVKTCIWPKSHTLWSNGVLESEMIIPKIYGGPISQHNYRPGYFTQTAGPWHLGKLELDFDLCEGTTVVVDEHCGNRGPSLRTTTVTGKTIHEWCCRSCTLPPLRFKGEDGCWYGMEIRPVKEKEENLVKSMVSAGSGEVDSFSLGLLCISIMIEEVMRSRWSRKMLMTGTLAVFLLLTMGQLTWNDLIRLCIMVGANASDKMGMGTTYLALMATFRMRPMFAVGLLFRRLTSREVLLLTVGLSLVASVELPNSLEELGDGLAMGIMMLKLLTDFQSHQLWATLLSLTFVKTTFSLHYAWKTMAMILSIVSLFPLCLSTTSQKTTWLPVLLGSLGCKPLTMFLITENKIWGRKSWPLNEGIMAVGIVSILLSSLLKNDVPLAGPLIAGGMLIACYVISGSSADLSLEKAAEVSWEEEAEHSGASHNILVEVQDDGTMKIKDEERDDTLTILLKATLLAISGVYPMSIPATLFVWYFWQKKKQRSGVLWDTPSPPEVERAVLDDGIYRILQRGLLGRSQVGVGVFQEGVFHTMWHVTRGAVLMYQGKRLEPSWASVKKDLISYGGGWRFQGSWNAGEEVQVIAVEPGKNPKNVQTAPGTFKTPEGEVGAIALDFKPGTSGSPIVNREGKIVGLYGNGVVTTSGTYVSAIAQAKASQEGPLPEIEDEVFRKRNLTIMDLHPGSGKTRRYLPAIVREAIKRKLRTLVLAPTRVVASEMAEALKGMPIRYQTTAVKSEHTGKEIVDLMCHATFTMRLLSPVRVPNYNMIIMDEAHFTDPASIAARGYISTRVGMGEAAAIFMTATPPGSVEAFPQSNAVIQDEERDIPERSWNSGYDWITDFPGKTVWFVPSIKSGNDIANCLRKNGKRVVQLSRKTFDTEYQKTKNNDWDYVVTTDISEMGANFRADRVIDPRRCLKPVILKDGPERVILAGPMPVTVASAAQRRGRIGRNQNKEGDQYIYMGQPLNNDEDHAHWTEAKMLLDNINTPEGIIPALFEPEREKSAAIDGEYRLRGEARKTFVELMRRGDLPVWLSYKVASEGFQYSDRRWCFDGERNNQVLEENMDVEIWTKEGERKKLRPRWLDARTYSDPLALREFKEFAAGRRSVSGDLILEIGKLPQHLTQRAQNALDNLVMLHNSEQGGKAYRHAMEELPDTIETLMLLALIAVLTGGVTLFFLSGRGLGKTSIGLLCVIASSALLWMASVEPHWIAASIILEFFLMVLLIPEPDRQRTPQDNQLAYVVIGLLFMILTVAANEMGLLETTKKDLGIGHAAAENHHHAAMLDVDLHPASAWTLYAVATTIITPMMRHTIENTTANISLTAIANQAAILMGLDKGWPISKMDIGVPLLALGCYSQVNPLTLTAAVLMLVAHYAIIGPGLQAKATREAQKRTAAGIMKNPTVDGIVAIDLDPVVYDAKFEKQLGQIMLLILCTSQILLMRTTWALCESITLATGPLTTLWEGSPGKFWNTTIAVSMANIFRGSYLAGAGLAFSLMKSLGGGRRGTGAQGETLGEKWKRQLNQLSKSEFNTYKRSGIIEVDRSEAKEGLKRGETTKHAVSRGTAKLRWFVERNLVKPEGKVIDLGCGRGGWSYYCAGLKKVTEVKGYTKGGPGHEEPIPMATYGWNLVKLYSGKDVFFTPPEKCDTLLCDIGESSPNPTIEEGRTLRVLKMVEPWLRGNQFCIKILNPYMPSVVETLEQMQRKHGGMLVRNPLSRNSTHEMYWVSCGTGNIVSAVNMTSRMLLNRFTMAHRKPTYERDVDLGAGTRHVAVEPEVANLDIIGQRIENIKNEHKSTWHYDEDNPYKTWAYHGSYEVKPSGSASSMVNGVVRLLTKPWDVIPMVTQIAMTDTTPFGQQRVFKEKVDTRTPKAKRGTAQIMEVTARWLWGFLSRNKKPRICTREEFTRKVRSNAAIGAVFVDENQWNSAKEAVEDERFWDLVHRERELHKQGKCATCVYNMMGKREKKLGEFGKAKGSRAIWYMWLGARFLEFEALGFMNEDHWFSRENSLSGVEGEGLHKLGYILRDISKIPGGNMYADDTAGWDTRITEDDLQNEAKITDIMEPEHALLATSIFKLTYQNKVVRVQRPAKNGTVMDVISRRDQRGSGQVGTYGLNTFTNMEAQLIRQMESEGIFSPSELETPNLAERVLDWLKKHGTERLKRMAISGDDCVVKPIDDRFATALTALNDMGKVRKDIPQWEPSKGWNDWQQVPFCSHHFHQLIMKDGREIVVPCRNQDELVGRARVSQGAGWSLRETACLGKSYAQMWQLMYFHRRDLRLAANAICSAVPVDWVPTSRTTWSIHAHHQWMTTEDMLSVWNRVWIEENPWMEDKTHVSSWEDVPYLGKREDQWCGSLIGLTARATWATNIQVAINQVRRLIGNENYLDFMTSMKRFKNESDPEGALW
	DENV-2	ngc-2007	QBK46951.1	Thailand	Yes	FHLTTRNGEPHMIVSRQEKGKSLLFKTEDGVNMCTLMAMDLGELCEDTITYKCPFLKQNEPEDIDCWCNSTSTWVTYGTCTTTGEHRREKRSVALVPHVGMGLETRTETWMSSEGAWKHAQRIETWILRHPGFTIMAAILAYTIGTTHFQRALIFILLTAVAPSMTMRCIGISNRDFVEGVSGGSWVDIVLEHGSCVTTMAKNKPTLDFELIKTEAKQPATLRKYCIEAKLTNTTTDSRCPTQGEPSLNEEQDKRFVCKHSMVDRGWGNGCGLFGKGGIVTCAMFTCKKNMKGKVVQPENLEYTIVITPHSGEEHAVGNDTGKHGKEIKITPQSSITEAELTGYGTVTMECSPRTGLDFNEMVLLQMENKAWLVHRQWFLDLPLPWLPGADTQGSNWIQKETLVTFKNPHAKKQDVVVLGSQEGAMHTALTGATEIQMSSGNLLFTGHLKCRLRMDKLQLKGMSYSMCTGKFKVVKEIAETQHGTIVIRVQYEGDGSPCKIPFEIMDLEKRHVLGRLITVNPIVTEKDSPVNIEAEPPFGDSYIIIGVEPGQLKLNWFKKGSSIGQMIETTMRGAKRMAILGDTAWDFGSLGGVFTSIGKALHQVFGAIYGAAFSGVSWIMKILIGVIITWIGMNSRSTSLSVSLVLVGVVTLYLGVMVQA
	DENV-3	Sleman/78	AAT69740.1	Indonesia	Yes	MNNQRKKTGKPSINMLKRVRNRVSTGSQLAKRFSRGLLNGQGPMKLVMAFIAFLRFLAIPPTAGVLARWGTFKKSGAIKVLRGFKKEISNMLSIINRRKKTSLCLMMMLPATLAFHLTSRDGEPRMIVGKNERGKSLLFKTASGINMCTLIAMDLGEMCDDTVTYKCPLITEVEPEDIDCWCNLTSTWVTYGTCNQAGEHRRDKRSVALAPHVGMGLDTRTQTWMSAEGAWRQVEKVETWAFRHPGFTILALFLAHYIGTSLTQKVVIFILLMLVTPSMTMRCVGVGNRDFVEGLSGATWVDVVLEHGGCVTTMAKNKPTLDIELQKTEATQLATLRKLCIEGKITNVTTDSRCPTQGEAILPEEQDQNHVCKHTYVDRGWGNGCGLFGKGSLVTCAKFQCLESIEGKVVQHENLKYTVIITVHTGDQHQVGNETQGVTAEITPQASTVEAILPEYGTLGLECSPRTGLDFNEMILLTMKNKAWMVHRQWFFDLPLPWTSGATTETPTWNKKELLVTFKNAHAKKQEVVVLGSQEGAMHTALTGATEIQTSGGTSIFAGHLKCRLKMDKLELKGMSYAMCLNAFVLKKEVSETQHGTILIKVEYKGEDAPCKIPFSTEDGQGKAHNGRLITANPVVTKKEEPVNIEAEPPFGESNIVIGIGDKALKINWYKKGSSIGKMFEATARGARRMAILGDTAWDFGSVGGVLNSLGKMVHQIFGSAYTALFSGVSWIMKIGIGVLLTWIGLNSKNTSMSFSCIVIGIITLYLGAVVQADMGCVINWKGKELKCGSGIFVTNEVHTWTEQYKFQADSPKRLATAIAGAWENGVCGIRSTTRMENLLWKQIANELNYILWENNIKLTVVVGDIIGVLEQGKRTLTPQPMELKYSWKTWGKAKIVTAETQNSSFIIDGPNTPECPSASRAWNVWEVEDYGFGVFTTNIWLKLREMYTQLCDHRLMSAAVKDERAVHADMGYWIESQKNGSWKLEKASLIEVKTCTWPKSHTLWSNGVLESDMIIPKSLAGPISQHNYRPGYHTQTAGPWHLGKLELDFNYCEGTTVVITENCGTRGPSLRTTTVSGKLIHEWCCRSCTLPPLRYMGEDGCWYGMEIRPINEKEENMVKSLVSAGSGKVDNFTMGVLCLAILFEEVMRGKFGKKHMIAGVLFTFVLLLSGQITWRDMAHTLIMIGSNASDRMGMGVTYLALIATFKIQPFLALGFFLRKLTSRENLLLGVGLAMATTLQLPEDIEQMANGIALGLMALKLITQFETYQLWTALVSLMCSNTIFTLTVAWRTATLILAGISLLPVCQSSSMRKTDWLPMAVAAMGVPPLPLFIFSLKDTLKRRSWPLNEGVMAVGLVSILASSLLRNDVPMAGPLVAGGLLIACYVITGTSADLTVEKAADVTWEEEAEQTGVSHNLMITVDDDGTMRIKDDETENILTVLLKTALLIVSGIFPYSIPATLLVWHTWQKQTQRSGVLWDVPSPPETQKAELEEGVYRIKQQGIFGKTQVGVGVQKEGVFHTMWHVTRGAVLTHNGKRLEPNWASVKKDLISYGGGWKLSAQWQKGEEVQVIAVEPGKNPKNFQTMPGIFQTTTGEIGAIALDFKPGTSGSPIINREGKVLGLYGNGVVTKNGGYVSGIAQTNAEPDGPTPELEEEMFKKRNLTIMDLHPGSGKTRKYLPAIVREAIKRRLRTLILAPTRVVAAEMEEALKGLPIRYQTTATKSEHTGREIVDLMCHATFTMRLLSPVRVPNYNLIIMDEAHFTDPASIAARGYISTRVGMGEAAAIFMTATPPGTADAFPQSNAPIQDEERDIPERSWNSGNEWITDFAGKTVWFVPSIKAGNDIANCLRKNGKKVIQLSRKTFDTEYQKTKLNDWDFVVTTDISEMGANFKADRVIDPRRCLKPVILTDGPERVILAGPMPVTVASAAQRRGRVGRNPQKENDQYIFMGQPLNNDEDHAHWTEAKMLLDNINTPEGIIPALFEPEREKSAAIDGEYRLKGESRKTFVELMRRGDLPVWLAHKVASEGIKYTDRKWCFDGERNNQILEENMDVEIWTKEGEKKKLRPRWLDARTYSDPLALKEFKDFAAGRKSIALDLVTEIGRVPSHLAHRTRNALDNLVMLHTSEHGGRAYRHAVEELPETMETLLLLGLMILLTGGAMLFLISGKGIGKTSIGLICVAASSGMLWMADVPLQWIASAIVLEFFMMVLLIPEPEKQRTPQDNQLAYVVIGILTLAAIVAANEMGLLETTKRDLGMSKEPGVVSPTSYLDVDLHPASAWTLYAVATTVITPMLRHTIENSTANVSLAAIANQAVVLMGLDKGWPISKMDLGVPLLALGCYSQVNPLTLTAAVLLLVTHYAIIGPGLQAKATREAQKRTAAGIMKNPTVDGIMTIDLDPVIYDSKFEKQLGQVMLLVLCAVQLLLMRTSWAFCEALTLATGPITTLWEGSPGKFWNTTIAVSMANIFRGSYLAGAGLAFSIMKSVGTGKRGTGSQGETLGEKWKKKLNQLPRKEFDLYKKSGITEVDRTEAKEGLKRGEITHHAVSRGSAKLQWFVERNMVIPEGRVIDLGCGRGGWSYYCAGLKKVTEVRGYTKGGPGHEEPVPMSTYGWNIVKLMSGKDVFYLPPEKCDTLLCDIGESSPSPTVEESRTIRVLKMVEPWLKNNQFCIKVLNPYMPTVIEHLERLQRKHGGMLVRNPLSRNSTHEMYWISNGTGNIVSSVNMVSRLLLNRFTMTHRRPTIEKDVDLGAGTRHVNAEPETPNMDVIGERIRRIKEEHSSTWHYDDENPYKTWAYHGSYEVKATGSASSMINGVVKLLTKPWDVVPMVTQMAMTDTTPFGQQRVFKEKVDTRTPRPMPGTRKVMEITAEWLWRTLGRNKRPRLCTREEFTKKVRTNAAMGAVFTEENQWDSARAAVEDEEFWKLVDRERELHKLGKCGSCVYNMMGKREKKLGEFGKAKGSRAIWYMWLGARYLEFEALGFLNEDHWFSRENSYSGVEGEGLHKLGYILRDISKIPGGAMYADDTAGWDTRITEDDLHNEEKITQQMDPEHRQLANAIFKLTYQNKVVKVQRPTPKGTVMDIISRKDQRGSGQVGTYGLNTFTNMEAQLIRQMEGEGVLSKADLENPHLLEKKVTQWLETKGVERLKRMAISGDDCVVKPIDDRFANALLALNDMGKVRKDIPQWQPSKGWHDWQQVPFCSHHFHELIMKDGRKLVVPCRPQDELIGRARISQGAGWSLRETACLGKAYAQMWTLMYFHRRDLRLASNAICSAVPVHWVPTSRTTWSIHAHHQWMTTEDMLTVWNRVWIEDNPWMEDKTPVKTWEDVPYLGKREDQWCGSLIGLTSRATWAQNILTAIQQVRSLIGNEEFLDYMPSMKRFRKEEESEGAIW
	DENV-4	814669	AAK01233.1	N/A	Yes	MNQRKKVVRPPFNMLKRERNRVSTPQGLVKRFSTGLFSGKGPLRMVLAFITFLRVLSIPPTAGILKRWGQLKKNKAIKILIGFRKEIGRMLNILNGRKRSTITLLCLIPTVMAFSLSTRDGEPLMIVAKHERGRPLLFKTTEGINKCTLIAMDLGEMCEDTVTYKCPLLVNTEPEDIDCWCNLTSTWVMYGTCTQSGERRREKRSVALTPHSGMGLETRAETWMSSEGAWKHAQRVESWILRNPGFALLAGFMAYMIGQTGIQRTVFFVLMMLVAPSYGMRCVGVGNRDFVEGVSGGAWVDLVLEHGGCVTTMAQGKPTLDFELTKTTAKEVALLRTYCIEASISNITTATRCPTQGEPYLKEEQDQQYICRRDVVDRGWGNGCGLFGKGGVVTCAKFSCSGKITGNLVQIENLEYTVVVTVHNGDTHAVGNDTSNHGVTAMITPRSPSVEVKLPDYGELTLDCEPRSGIDFNEMILMKMKKKTWLVHKQWFLDLPLPWTAGADTSEVHWNYKERMVTFKVPHAKRQDVTVLGSQEGAMHSALAGATEVDSGDGNHMFAGHLKCKVRMEKLRIKGMSYTMCSGKFSIDKEMAETQHGTTVVKVKYEGAGAPCKVPIEIRDVNKEKVVGRIISSTPLAENTNSVTNIELEPPFGDSYIVIGVGNSALTLHWFRKGSSIGKMFESTYRGAKRMAILGETAWDFGSVGGLFTSLGKAVHQVFGSVYTTMFGGVSWMIRILIGFLVLWIGTNSRNTSMAMTCIAVGGITLFLGFTVQADMGCVVSWSGKELKCGSGIFVVDNVHTWTEQYKFQPESPARLASAILNAHKDGVCGIRSTTRLENVMWKQITNELNYVLWEGGHDLTVVAGDVKGVLTKGKRALTPPVSDLKYSWKTWGKAKIFTPEARNSTFLIDGPDTSECPNERRAWNSLEVEDYGFGMFTTNIWMKFREGSSEVCDHRLMSAAIKDQKAVHADMGYWIESSKNQTWQIEKASLIEVKTCLWPKTHTLWSNGVLESQMLIPKSYAGPFSQHNYRQGYATQTVGPWHLGKLEIDFGECPGTTVTIQEDCDHRGPSLRTTTASGKLVTQWCCRSCTMPPLRFLGEDGCWYGMEIRPLSEKEENMVKSQVTAGQGTSETFSMGLLCLTLFVEECLRRRVTRKHMILVVVITLCAIILGGLTWMDLLRALIMLGDTMSGRIGGQIHLAIMAVFKMSPGYVLGVFLRKLTSRETALMVIGMAMTTVLSIPHDLMELIDGISLGLILLKIVTQFDNTQVGTLALSLTFIRSTMPLVMAWRTIMAVLFVVTLIPLCRTSCLQKQSHWVEITALILGAQALPVYLMTLMKGASRRSWPLNEGIMAVGLVSLLGSALLKNDVPLAGPMVAGGLLLAAYVMSGSSADLSLEKAANVQWDEMADITGSSPIIEVKQDEDGSFSIRDVEETNMITLLVKLALITVSGLYPLAIPVTMTLWYMWQVKTQRSGALWDVPSPAATKKAALSEGVYRIMQRGLFGKTQVGVGIHMEGVFHTMWHVTRGSVICHETGRLEPSWADVRNDMISYGGGWRLGDKWDKEEDVQVLAIEPGKNPKHVQTKPGLFKTLTGEIGAVTLDFKPGTSGSPIINRKGKVIGLYGNGVVTKSGDYVSAITQAERIGEPDYEVDEDIFRKKRLTIMDLHPGAGKTKRILPSIVREALKRRLRTLILAPTRVVAAEMEEALRGLPIRYQTPAVKSEHTGREIVDLMCHATFTTRLLSSTRVPNYNLIVMDEAHFTDPSSVAARGYISTRVEMGEAAAIFMTATPPGATDPFPQSNSPIEDIEREIPERSWNTGFDWITDYQGKTVWFVPSIKAGNDIANCLRKSGKKVIQLSRKTFDTEYPKTKLTDWDFVVTTDISEMGANFRAGRVIDPRRCLKPVILTDGPERVILAGPIPVTPASAAQRRGRIGRNPAQEDDQYVFSGDPLKNDEDHAHWTEAKMLLDNIYTPEGIIPTLFGPEREKTQAIDGEFRLRGEQRKTFVELMRRGDLPVWLSYKVASAGISYKDREWCFTGERNNQILEENMEVEIWTREGEKKKLRPRWLDARVYADPMALKDFKEFASGRKSITLDILTEIASLPTYLSSRAKLALDNIVMLHTTERGGRAYQHALNELPESLETLMLVALLGAMTAGIFLFFMQGKGIGKLSMGLITIAVASGLLWVAEIQPQWIAASIILEFFLMVLLIPEPEKQRTPQDNQLIYVILTILTIIGLIAANEMGLIEKTKTDFGFYQVKTETTILDVDLRPASAWTLYAVATTILTPMLRHTIENTSANLSLAAIANQAAVLMGLGKGWPLHRMDLGVPLLAMGCYSQVNPTTLTASLVMLLVHYAIIGPGLQAKATREAQKRTAAGIMKNPTVDGITVIDLEPISYDPKFEKQLGQVMLLVLCAGQLLLMRTTWAFCEVLTLATGPILTLWEGNPGRFWNTTIAVSTANIFRGSYLAGAGLAFSLIKNAQTPRRGTGTTGETLGEKWKRQLNSLDRREFEEYKRSGILEVDRTEAKSALKDGSKIKHAVSRGSSKIRWIVERGMVKPKGKVVDLGCGRGGWSYYMATLKNVTEVKGYTKGGPGHEEPIPMATYGWNLVKLHSGVDVFYKPTEQVDTLLCDIGESSSNPTIEEGRTLRVLKMVEPWLSSKPEFCIKVLNPYMPTVIEELEKLQRKHGGNLVRCPLSRNSTHEMYWVSGASGNIVSSVNTTSKMLLNRFTTRHRKPTYEKDVDLGAGTRSVSTETEKPDMTIIGRRLQRLQEEHKETWHYDQENPYRTWAYHGSYEAPSTGSASSMVNGVVKLLTKPWDVIPMVTQLAMTDTTPFGQQRVFKEKVDTRTPQPKPGTRMVMTTTANWLWALLGKKKNPRLCTREEFISKVRSNAAIGAVFQEEQGWTSASEAVNDSRFWELVDKERALHQEGKCESCVYNMMGKREKKLGEFGRAKGSRAIWYMWLGARFLEFEALGFLNEDHWFGRENSWSGVEGEGLHRLGYILEEIDKKDGDLMYADDTAGWDTRITEDDLQNEELITEQMAPHHKILAKAIFKLTYQNKVVKVLRPTPRGAVMDIISRKDQRGSGQVGTYGLNTFTNMEVQLIRQMEAEGVITQDDMQNPKGLKERVEKWLKECGVDRLKRMAISGDDCVVKPLDERFGTSLLFLNDMGKVRKDIPQWEPSKGWKNWQEVPFCSHHFHKIFMKDGRSLVVPCRNQDELIGRARISQGAGWSLRETACLGKAYAQMWSLMYFHRRDLRLASMAICSAVPTEWFPTSRTTWSIHAHHQWMTTEDMLKVWNRVWIEDNPNMTDKTPVHSWEDIPYLGKREDLWCGSLIGLSSRATWAKNIHTAITQVRNLIGKEEYVDYMPVMKRYSAPSESEGVL
TDEN	DENV-1	Nauru Island Western Pacific 45AZ5	NP_059433.1	N/A	Yes	MNNQRKKTGRPSFNMLKRARNRVSTVSQLAKRFSKGLLSGQGPMKLVMAFIAFLRFLAIPPTAGILARWGSFKKNGAIKVLRGFKKEISNMLNIMNRRKRSVTMLLMLLPTALAFHLTTRGGEPHMIVSKQERGKSLLFKTSAGVNMCTLIAMDLGELCEDTMTYKCPRITETEPDDVDCWCNATETWVTYGTCSQTGEHRRDKRSVALAPHVGLGLETRTETWMSSEGAWKQIQKVETWALRHPGFTVIALFLAHAIGTSITQKGIIFILLMLVTPSMAMRCVGIGNRDFVEGLSGATWVDVVLEHGSCVTTMAKDKPTLDIELLKTEVTNPAVLRKLCIEAKISNTTTDSRCPTQGEATLVEEQDTNFVCRRTFVDRGWGNGCGLFGKGSLITCAKFKCVTKLEGKIVQYENLKYSVIVTVHTGDQHQVGNETTEHGTTATITPQAPTSEIQLTDYGALTLDCSPRTGLDFNEMVLLTMKKKSWLVHKQWFLDLPLPWTSGASTSQETWNRQDLLVTFKTAHAKKQEVVVLGSQEGAMHTALTGATEIQTSGTTTIFAGHLKCRLKMDKLILKGMSYVMCTGSFKLEKEVAETQHGTVLVQVKYEGTDAPCKIPFSSQDEKGVTQNGRLITANPIVTDKEKPVNIEAEPPFGESYIVVGAGEKALKLSWFKKGSSIGKMFEATARGARRMAILGDTAWDFGSIGGVFTSVGKLIHQIFGTAYGVLFSGVSWTMKIGIGILLTWLGLNSRSTSLSMTCIAVGMVTLYLGVMVQADSGCVINWKGRELKCGSGIFVTNEVHTWTEQYKFQADSPKRLSAAIGKAWEEGVCGIRSATRLENIMWKQISNELNHILLENDMKFTVVVGDVSGILAQGKKMIRPQPMEHKYSWKSWGKAKIIGADVQNTTFIIDGPNTPECPDNQRAWNIWEVEDYGFGIFTTNIWLKLRDSYTQVCDHRLMSAAIKDSKAVHADMGYWIESEKNETWKLARASFIEVKTCIWPKSHTLWSNGVLESEMIIPKIYGGPISQHNYRPGYFTQTAGPWHLGKLELDFDLCEGTTVVVDEHCGNRGPSLRTTTVTGKTIHEWCCRSCTLPPLRFKGEDGCWYGMEIRPVKEKEENLVKSMVSAGSGEVDSFSLGLLCISIMIEEVMRSRWSRKMLMTGTLAVFLLLTMGQLTWNDLIRLCIMVGANASDKMGMGTTYLALMATFRMRPMFAVGLLFRRLTSREVLLLTVGLSLVASVELPNSLEELGDGLAMGIMMLKLLTDFQSHQLWATLLSLTFVKTTFSLHYAWKTMAMILSIVSLFPLCLSTTSQKTTWLPVLLGSLGCKPLTMFLITENKIWGRKSWPLNEGIMAVGIVSILLSSLLKNDVPLAGPLIAGGMLIACYVISGSSADLSLEKAAEVSWEEEAEHSGASHNILVEVQDDGTMKIKDEERDDTLTILLKATLLAISGVYPMSIPATLFVWYFWQKKKQRSGVLWDTPSPPEVERAVLDDGIYRILQRGLLGRSQVGVGVFQEGVFHTMWHVTRGAVLMYQGKRLEPSWASVKKDLISYGGGWRFQGSWNAGEEVQVIAVEPGKNPKNVQTAPGTFKTPEGEVGAIALDFKPGTSGSPIVNREGKIVGLYGNGVVTTSGTYVSAIAQAKASQEGPLPEIEDEVFRKRNLTIMDLHPGSGKTRRYLPAIVREAIKRKLRTLVLAPTRVVASEMAEALKGMPIRYQTTAVKSEHTGKEIVDLMCHATFTMRLLSPVRVPNYNMIIMDEAHFTDPASIAARGYISTRVGMGEAAAIFMTATPPGSVEAFPQSNAVIQDEERDIPERSWNSGYDWITDFPGKTVWFVPSIKSGNDIANCLRKNGKRVVQLSRKTFDTEYQKTKNNDWDYVVTTDISEMGANFRADRVIDPRRCLKPVILKDGPERVILAGPMPVTVASAAQRRGRIGRNQNKEGDQYIYMGQPLNNDEDHAHWTEAKMLLDNINTPEGIIPALFEPEREKSAAIDGEYRLRGEARKTFVELMRRGDLPVWLSYKVASEGFQYSDRRWCFDGERNNQVLEENMDVEIWTKEGERKKLRPRWLDARTYSDPLALREFKEFAAGRRSVSGDLILEIGKLPQHLTQRAQNALDNLVMLHNSEQGGKAYRHAMEELPDTIETLMLLALIAVLTGGVTLFFLSGRGLGKTSIGLLCVIASSALLWMASVEPHWIAASIILEFFLMVLLIPEPDRQRTPQDNQLAYVVIGLLFMILTVAANEMGLLETTKKDLGIGHAAAENHHHAAMLDVDLHPASAWTLYAVATTIITPMMRHTIENTTANISLTAIANQAAILMGLDKGWPISKMDIGVPLLALGCYSQVNPLTLTAAVLMLVAHYAIIGPGLQAKATREAQKRTAAGIMKNPTVDGIVAIDLDPVVYDAKFEKQLGQIMLLILCTSQILLMRTTWALCESITLATGPLTTLWEGSPGKFWNTTIAVSMANIFRGSYLAGAGLAFSLMKSLGGGRRGTGAQGETLGEKWKRQLNQLSKSEFNTYKRSGIIEVDRSEAKEGLKRGETTKHAVSRGTAKLRWFVERNLVKPEGKVIDLGCGRGGWSYYCAGLKKVTEVKGYTKGGPGHEEPIPMATYGWNLVKLYSGKDVFFTPPEKCDTLLCDIGESSPNPTIEEGRTLRVLKMVEPWLRGNQFCIKILNPYMPSVVETLEQMQRKHGGMLVRNPLSRNSTHEMYWVSCGTGNIVSAVNMTSRMLLNRFTMAHRKPTYERDVDLGAGTRHVAVEPEVANLDIIGQRIENIKNEHKSTWHYDEDNPYKTWAYHGSYEVKPSGSASSMVNGVVRLLTKPWDVIPMVTQIAMTDTTPFGQQRVFKEKVDTRTPKAKRGTAQIMEVTARWLWGFLSRNKKPRICTREEFTRKVRSNAAIGAVFVDENQWNSAKEAVEDERFWDLVHRERELHKQGKCATCVYNMMGKREKKLGEFGKAKGSRAIWYMWLGARFLEFEALGFMNEDHWFSRENSLSGVEGEGLHKLGYILRDISKIPGGNMYADDTAGWDTRITEDDLQNEAKITDIMEPEHALLATSIFKLTYQNKVVRVQRPAKNGTVMDVISRRDQRGSGQVGTYGLNTFTNMEAQLIRQMESEGIFSPSELETPNLAERVLDWLKKHGTERLKRMAISGDDCVVKPIDDRFATALTALNDMGKVRKDIPQWEPSKGWNDWQQVPFCSHHFHQLIMKDGREIVVPCRNQDELVGRARVSQGAGWSLRETACLGKSYAQMWQLMYFHRRDLRLAANAICSAVPVDWVPTSRTTWSIHAHHQWMTTEDMLSVWNRVWIEENPWMEDKTHVSSWEDVPYLGKREDQWCGSLIGLTARATWATNIQVAINQVRRLIGNENYLDFMTSMKRFKNESDPEGALW
	DENV-2	S16803	ADA00411.1	Thailand	Yes	MNNQRKKARNTPFNMLKRERNRVSTVQQLTKRFSLGMLQGRGPLKLFMALVAFLRFLTIPPTAGILKRWGTIKKSKAINVLRGFRKEIGRMLNILNRRRRSAGMIIMLIPTVMAFHLTTRNGEPHMIVSRQEKGKSLLFKTEDGVNMCTLMAMDLGELCEDTITYNCPLLRQNEPEDIDCWCNSTSTWVTYGTCTTTGEHRREKRSVALVPHVGMGLETRTETWMSSEGAWKHAQRIETWILRHPGFTIMAAILAYTIGTTHFQRALIFILLTAVAPSMTMRCIGISNRDFVEGVSGGSWVDIVLEHGSCVTTMAKNKPTLDFELIKTEAKQPATLRKYCIEAKLTNTTTESRCPTQGEPSLNEEQDKRFVCKHSMVDRGWGNGCGLFGKGGIVTCAMFTCKKNMEGKVVQPENLEYTIVVTPHSGEEHAVGNDTGKHGKEIKVTPQSSITEAELTGYGTVTMECSPRTGLDFNEMVLLQMENKAWLVHRQWFLDLPLPWLPGADTQGSNWIQKETLVTFKNPHAKKQDVVVLGSQEGAMHTALTGATEIQMSSGNLLFTGHLKCRLRMDKLQLKGMSYSMCTGKFKVVKEIAETQHGTIVIRVQYEGDGSPCKIPFEIMDLEKRHVLGRLITVNPIVTEKDSPVNIEAEPPFGDSYIIIGVDPGQLKLNWFKKGSSIGQMFETTMRGAKRMAILGDTAWDFGSLGGVFTSIGKALHQVFGAIYGAAFSGVSWTMKILIGVIITWIGMNSRSTSLSVSLVLVGIVTLYLGVMVQADSGCVVSWKNKELKCGSGIFITDNVHTWTEQYKFQPESPSKLASAIQKAQEEGICGIRSVTRLENLMWKQITPELNHILAENEVKLTIMTGDIKGIMQAGKRSLRPQPTELKYSWKTWGKAKMLSTESHNQTFLIDGPETAECPNTNRAWNSLEVEDYGFGVFTTNIWLKLKEKQDAFCDSKLMSAAIKDNRAVHADMGYWIESALNDTWKIEKASFIEVKNCHWPKSHTLWSNGVLESEMIIPKNLAGPVSQHNYRPGYHTQIAGPWHLGKLEMDFDFCDGTTVVVTEDCGNRGPSLRTTTASGKLITEWCCRSCTLPPLRYRGEDGCWYGMEIRPLKEKEENLVNSLVTAGHGQVDNFSLGVLGMALFLEEMLRTRVGTKHAILLVAVSFVTLITGNMSFRDLGRVVVMVGATMTDDIGMGVTYLALLAAFKVRPTFAAGLLLRKLTSKELMMTTIGIVLLSQSTIPETILELTDALALGMMVLKMVRNMEKYQLAVTIMAILCVPNAVILQNAWKVSCTILAVVSVSPLLLTSSQQKTDWIPLALTIKGLNPTAIFLTTLSRTSKKRSWPLNEAIMAVGMVSILASSLLKNDIPMTGPLVAGGLLTVCYVLTGRSADLELERAADVKWEDQAEISGSSPILSITISEDGSMSIKNEEEEQTLTILIRTGLLVISGLFPVSIPITAAAWYLWEVKKQRAGVLWDVPSPPPMGKAELEDGAYRIKQKGILGYSQIGAGVYKEGTFHTMWHVTRGAVLMHKGKRIEPSWADVKKDLISYGGGWKLEGEWKEGEEVQVLALEPGKNPRAVQTKPGLFRTNAGTIGAVSLDFSPGTSGSPIIDKKGKVVGLYGNGVVTRSGAYVSAIAQTEKSIEDNPEIEDDIFRKRRLTIMDLHPGAGKTKRYLPAIVREAIKRGLRTLILAPTRVVAAEMEEALRGLPIRYQTPAIRAEHTGREIVDLMCHATFTMRLLSPVRVPNYNLIIMDEAHFTDPASIAARGYISTRVEMGEAAGIFMTATPPGSRDPFPQSNAPIIDEEREIPERSWNSGHEWVTDFKGKTVWFVPSIKAGNDIAACLRKNGKKVIQLSRKTFDSEYVKTRTNDWDFVVTTDISEMGANFKAERVIDPRRCMKPVILTDGEERVILAGPMPVTHSSAAQRRGRIGRNPKNENDQYIYMGEPLENDEDCAHWKEAKMLLDNINTPEGIIPSMFEPEREKVDAIDGEYRLRGEARKTFVDLMRRGDLPVWLAYKVAAEGINYADRRWCFDGIKNNQILEENVEVEIWTKEGERKKLKPRWLDARIYSDPLALKEFKEFAAGRKSLTLNLITEMGRLPTFMTQKARDALDNLAVLHTAEAGGRAYNHALSELPETLETLLLLTLLATVTGGIFLFLMSGRGIGKMTLGMCCIITASILLWYAQIQPHWIAASIILEFFLIVLLIPEPEKQRTPQDNQLTYVVIAILTVVAATMANEMGFLEKTKKDLGLGSIATQQPESNILDIDLRPASAWTLYAVATTFVTPMLRHSIENSSVNVSLTAIANQATVLMGLGKGWPLSKMDIGVPLLAIGCYSQVNPITLTAALLLLVAHYAIIGPGLQAKATREAQKRAAAGIMKNPTVDGITVIDLDPIPYDPKFEKQLGQVMLLVLCVTQVLMMRTTWALCEALTLATGPISTLWEGNPGRFWNTTIAVSMANIFRGSYLAGAGLLFSIMKNTTNTRRGTGNIGETLGEKWKSRLNALGKSEFQIYKKSGIQEVDRTLAKEGIKRGETDHHAVSRGSAKLRWFVERNMVTPEGKVVDLGCGRGGWSYYCGGLKNVREVKGLTKGGPGHEEPIPMSTYGWNLVRLQSGVDVFFIPPEKCDTLLCDIGESSPNPTVEAGRTLRVLNLVENWLNNNTQFCIKVLNPYMPSVIEKMETLQRKYGGALVRNPLSRNSTHEMYWVSNASGNIVSSVNMISRMLINRFTMRHKKATYEPDVDLGSGTRNIGIESEIPNLDIIGKRIEKIKQEHETSWHYDQDHPYKTWAYHGSYETKQTGSASSMVNGVVRLLTKPWDVVPMVTQMAMTDTTPFGQQRVFKEKVDTRTQEPKEGTKKLMKITAEWLWKELGKKKTPRMCTREEFTRKVRSNAALGAIFTDENKWKSAREAVEDSRFWELVDKERNLHLEGKCETCVYNMMGKREKKLGEFGKAKGSRAIWYMWLGARFLEFEALGFLNEDHWFSRENSLSGVEGEGLHKLGYILRDVSKKEGGAMYADDTAGWDTRITLEDLKNEEMVTNHMEGEHKKLAEAIFKLTYQNKVVRVQRPTPRGTVMDIISRRDQRGSGQVGTYGLNTFTNMEAQLIRQMEGEGVFKNIQHLTVTEEIAVQNWLARVGRERLSRMAISGDDCVVKPLDDRFASALTALNDMGKIRKDIQQWEPSRGWNDWTQVPFCSHHFHELIMKDGRVLVVPCRNQDELIGRARISQGAGWSLRETACLGKSYAQMWSLMYFHRRDLRLAANAICSAVPSHWVPTSRTTWSIHAKHEWMTTEDMLTVWNRVWIQENPWMEDKTPVESWEEIPYLGKREDQWCGSLIGLTSRATWAKNIQAAINQVRSLIGNEEYTDYMPSMKRFRREEEEAGVLW
	DENV-3	CH53489	AAB69126.2	Thailand	Yes	MNNQRKKTGKPSINMLKRVRNRVSTGSQLAKRFSRGLLNGQGPMKLVMAFIAFLRFLAIPPTAGVLARWGTFKKSGAIKVLKGFKKEISNMLSIINKRKKTSLCLMMMLPATLAFHLTSRDGEPRMIVGKNERGKSLLFKTASGINMCTLIAMDLGEMCDDTVTYKCPHIAEVEPEDIDCWCNLTSTWVTYGTCNQAGEHRRDKRSVALAPHVGMGLDTRTQTWMSAEGAWRQVEKVETWALRHPGFTILALFLAHYIGTSLTQKVVIFILLILVTPSMAMRCVGVGNRDFVEGLSGATWVDVVLEHGGCVTTMAKNKPTLDIELQKTEATQLATLRKLCIEGKITNITTDSRCPTQGEAILPEEQDQNYVCKHTYVDRGWGNGCGLFGKGSLVTCAKFQCLESIEGKVVQHENLKYTVIITVHTGDQHQVGNETQGVTAEITPQASTVEAILPEYGTLGLECSPRTGLDFNEMILLTMKNKAWMVHRQWFFDLPLPWTSGATTETPTWNRKELLVTFKNAHAKKQEVVVLGSQEGAMHTALTGATEIQNSGGTSIFAGHLKCRLKMDKLELKGMSYAMCLNTFVLKKEVSETQHGTILIKVEYKGEDAPCKIPFSTEDGQGKAHNGRLITANPVVTKKEEPVNIEAEPPFGESNIVIGIGDKALKINWYKKGSSIGKMFEATARGARRMAILGDTAWDFGSVGGVLNSLGKMVHQIFGSAYTALFSGVSWIMKIGIGVLLTWIGLNSKNTSMSFSCIAIGIITLYLGAVVQADMGCVINWKGKELKCGSGIFVTNEVHTWTEQYKFQADSPKRLATAIAGAWENGVCGIRSTTRMENLLWKQIANELNYILWENNIKLTVVVGDIIGVLEQGKRTLTPQPMELKYSWKTWGKAKIVTAETQNSSFIIDGPHTPECPRASRAWNVWEVEDYGFGVFTTNIWLKLREEYTQLCDHRLMSAAVKDERAVHADMGYWIESQKNGSWKLEKASLIEVKTCTWPKSHTLWSNGVLESDMIIPKSLAGPISQHNHRPGYHTQTAGPWHLGKLELDFNYCEGTTVVITENCGTRGPSLRTTTVSGKLIHEWCCRSCTLPPLRYMGEDGCWYGMEIRPINEKEENMVKSLASAGSGKVDNFTMGFLCLAILFEEVMRGKFGKKHMIAGVFLTFLLLLSGQITWRDMAHTLIMIGSNASDRMGMGVTYLALIATFKIQPFLALGFFLRKLTSRENFLLGVGLAMATTLQLPEDIEQMANGIALGLMALKLITQFETYQLWTALVSLTCSNTIFTLTVAWRTATLILAGVSLLPVCQSSSMRKTDWLPMAVAAMGVPPLPLFIFSLKDTLKRRSWPLNEGVMAVGLVSILASSLLRNDVPMAGPLVAGGLLIACYVITGTSADLTVEKAADVTWEEEAEQTGVSHNLMITVDDDGTMRIKDDETENILTVLLKTALLIVSGIFPYSIPATLLVWHTWQKQTQRSGVLWDVPSPPETQKAELEEGVYRIKQQGIFGKTQVGVGVQKEGVFHTMWHVTRGAVLTHNGKRLEPNWASVKKDLISYGGGWRLSAQWQKGEEVQVIAVEPGKNPKNFQTMPGIFQTTTGEIGAIALDFKPGTSGSPIINREGKVVGLYGNGVVTKNGGYVSGIAQTNAEPDGPTPELEEEMFKKRNLTIMDLHPGSGKTRKYLPAIVREAIKRRLRTLILAPTRVVAAEMEEALKGLPIRYQTTATKSEHTGREIVDLMCHATFTMRLLSPVRVPNYNLIIMDEAHFTDPASIAARGYISTRVGMGEAAAIFMTATPPGTADAFPQSNAPIQDEERDIPERSWNSGNEWITDFVGKTVWFVPSIKAGNDIANCLRKNGKKVIQLSRKTFDTEYQKTKLNDWDFVVTTDISEMGANFKADRVIDPRRCLKPVILTDGPERVILAGPMPVTAASAAQRRGRVGRNPQKENDQYIFRGQPLNNDEDHAHWTEAKMLLDNINTPEGIIPALFEPEREKSAAIDGEYRLKGESRKTFVELMRRGDLPVWLAHKVASEGIKYTDRKWCFDGERNNQILEENMDVEIWTKEGEKKKLRPRWLDARTYSDPLALKEFKDFAAGRKSIALDLVTEIGRVPSHLAHRTRNALDNLVMLHTSEHGGRAYRHAVEELPETMETLLLLGLMILLTGGAMLFLISGKGIGKTSIGLICVIASSGMLWMADVPLQWIASAIVLEFFMMVLLIPEPEKQRTPQDNQLAYVVIGILTLAAIVAANEMGLLETTKRDLGMSKEPGVVSPTSYLDVDLHPASAWTLYAVATTVITPMLRHTIENSTANVSLAAIANQAVVLMGLDKGWPISKMDLGVPLLALGCYSQVNPLTLTAAVLLLATHYAIIGPGLQAKATREAQKRTAAGITKNPTVDGIMTIDLDPVIYDSKFEKQLGQVMLLVLCAVQLLLMRTSWALCEALTLATGPITTLWEGSPGKFWNTTIAVSMANIFRGSYLAGAGLAFSIMKSVGTGKRGTGSQGETLGEKWKKKLNQLSRKEFDLYKKSGITEVDRTEAKEGLKRGEITRHAVSRGSAKLQWFVERNMVIPEGRVIDLGCGRGGWSYYCAGLKKVTEVRGYTKGGPGHEEPVPMSTYGWNIVKLMSGKDVFYLPPEKCDTLLCDIGESSPSPTVEESRTIRVLKMVEPWLKNNQFCIKVLNPYMPTVIEHLERLQRKHGGMLVRNPLSRNSTHEMYWISNGTGNIVSSVNMVSRLLLNRFTMTHRRPTIEKDVDLGAGTRHVNAEPETPNMDVIGERIKRIKEEHNSTWHYDDENPYKTWAYHGSYEVKATGSASSMINGVVKLLTKPWDVVPMVIQMAMTDTTPFGQQRVFKEKVDTRTPRPMPGTRKVMEITAEWLWRTLGRNKRPRLCTREEFTKKVRTNAAMGAVFTEENQWDSARAAVEDEEFWKLVDRERELHKLGKCGSCVYNMMGKREKKLGEFGKAKGSRAIWYMWLGARYLEFEALGFFNEDHWFSRENSYSGVEGEGLHKLGYILRDISKIPGGAMYADDTAGWDTRITEDDLHNEEKITQQMDPEHRQLANAIFKLTYQNKVVKVQRPTPKGTVMDIISRKDQRGSGQVGTYGLNTFTNMEAQLVRQMEGEGVLSKADLENPHLLEKKITQWLETKGVERLKRMAISGDDCVVKPIDDRFANALLALNDMGKVRKDIPQWQPSKGWHDWQQVPFCSHHFHELIMKDGRKLVVPCRPQDELIGRARISRGAGWSLKETACLGKAYAQMWSLMYFHRRDLRLASNAICSAVPVHWVPTSRTTWSIHAHHQWMTTEDMLTVWNRVWIEDNPWMEDKTPVTTWEDVPYLGKREDQWCGSLIGLTSRATWAQNILTAIQQVRSLIGNEEFLDYMPSMKRFKKEEESEGAIW

	DENV-4	341750	ADA00410.1	Colombia	Yes	MNQRKKVVRPPFNMLKRERNRVSTPQGLVKRFSTGLFSGKGPLRMVLAFITFLRVLSIPPTAGILKRWGQLKKNKAIKILIGFRKEIGRMLNILNGRKRSTITLLCLIPTVMAFHLSTRDGEPLMIVAKHERGRPLLFKTTEGINKCTLIAMDLGEMCEDTVTYKCPLLVNTEPEDIDCWCNLTSTWVMYGTCTQSGERRREKRSVALTPHSGMGLETRAETWMSSEGAWKHAQRVESWILRNPGFALLAGFMAYMIGQTGIQRTVFFVLMMLVAPSYGMRCVGVGNRDFVEGVSGGAWVDLVLEHGGCVTTMAQGKPTLDFELTKTTAKEVALLRTYCIEASISNITTATRCPTQGEPYLKEEQDQQYICRRDVVDRGWGNGCGLFGKGGVVTCAKFSCSGKITGNLVQIENLEYTVVVTVHNGDTHAVGNDTSNHGVTAMITPRSPSVEVKLPDYGELTLDCEPRSGIDFNEMILMKMKKKTWLVHKQWFLDLPLPWTAGADTSEVHWNYKERMVTFKVPHAKRQDVTVLGSQEGAMHSALAGATEVDSGDGNHMFAGHLKCKVRMEKLRIKGMSYTMCSGKFSIDKEMAETQHGTTVVKVKYEGAGAPCKVPIEIRDVNKEKVVGRIISSTPLAENTNSVTNIELEPPFGDSYIVIGVGNSALTLHWFRKGSSIGKMFESTYRGAKRMAILGETAWDFGSVGGLFTSLGKAVHQVFGSVYTTMFGGVSWMIRILIGFLVLWIGTNSRNTSMAMTCIAVGGITLFLGFTVQADMGCVVSWSGKELKCGSGIFVVDNVHTWTEQYKFQPESPARLASAILNAHKDGVCGIRSTTRLENVMWKQITNELNYVLWEGGHDLTVVAGDVKGVLTKGKRALTPPVSDLKYSWKTWGKAKIFTPEARNSTFLIDGPDTSECPNERRAWNSLEVEDYGFGMFTTNIWMKFREGSSEVCDHRLMSAAIKDQKAVHADMGYWIESSKNQTWQIEKASLIEVKTCLWPKTHTLWSNGVLESQMLIPKSYAGPFSQHNYRQGYATQTVGPWHLGKLEIDFGECPGTTVTIQEDCDHRGPSLRTTTASGKLVTQWCCRSCTMPPLRFLGEDGCWYGMEIRPLSEKEENMVKSQVTAGQGTSETFSMGLLCLTLFVEECLRRRVTRKHMILVVVITLCAIILGGLTWMDLLRALIMLGDTMSGRIGGQIHLAIMAVFKMSPGYVLGVFLRKLTSRETALMVIGMAMTTVLSIPHDLMELIDGISLGLILLKIVTQFDNTQVGTLALSLTFIRSTMPLVMAWRTIMAVLFVVTLIPLCRTSCLQKQSHWVEITALILGAQALPVYLMTLMKGASRRSWPLNEGIMAVGLVSLLGSALLKNDVPLAGPMVAGGLLLAAYVMSGSSADLSLEKAANVQWDEMADITGSSPIIEVKQDEDGSFSIRDVEETNMITLLVKLALITVSGLYPLAIPVTMTLWYMWQVKTQRSGALWDVPSPAATQKAALSEGVYRIMQRGLFGKTQVGVGIHMEGVFHTMWHVTRGSVICHETGRLEPSWADVRNDMISYGGGWRLGDKWDKEEDVQVLAIEPGKNPKHVQTKPGLFKTLTGEIGAVTLDFKPGTSGSPIINRKGKVIGLYGNGVVTKSGDYVSAITQAERIGEPDYEVDEDIFRKKRLTIMDLHPGAGKTKRILPSIVREALKRRLRTLILAPTRVVAAEMEEALRGLPIRYQTPAVKSEHTGREIVDLMCHATFTTRLLSSTRVPNYNLIVMDEAHFTDPSSVAARGYISTRVEMGEAAAIFMTATPPGATDPFPQSNSPIEDIEREIPERSWNTGFDWITDYQGKTVWFVPSIKAGNDIANCLRKSGKKVIQLSRKTFDTEYPKTKLTDWDFVVTTDISEMGANFRAGRVIDPRRCLKPVILTDGPERVILAGPIPVTPASAAQRRGRIGRNPAQEDDQYVFSGDPLKNDEDHAHWTEAKMLLDNIYTPEGIIPTLFGPEREKTQAIDGEFRLRGEQRKTFVELMRRGDLPVWLSYKVASAGISYKDREWCFTGERNNQILEENMEVEIWTREGEKKKLRPRWLDARVYADPMALKDFKEFASGRKSITLDILTEIASLPTYLSSRAKLALDNIVMLHTTERGGRAYQHALNELPESLETLMLVALLGAMTAGIFLFFMQGKGIGKLSMGLITIAVASGLLWVAEIQPQWIAASIILEFFLMVLLIPEPEKQRTPQDNQLIYVILTILTIIGLIAANEMGLIEKTKTDFGFYQVKTETTILDVDLRPASAWTLYAVATTILTPMLRHTIENTSANLSLAAIANQAAVLMGLGKGWPLHRMDLGVPLLAMGCYSQVNPTTLTASLVMLSVHYAIIGPGLQAKATREAQKRTAAGIMKNPTVDGITVIDLEPISYDPKFEKQLGQVMLLVLCAGQLLLMRTTWAFCEVLTLATGPILTLWEGNPGRFWNTTIAVSTANIFRGSYLAGAGLAFSLIKNAQTPRRGTGTTGETLGEKWKRQLNSLDRKEFEEYKRSGILEVDRTEAKSALKDGSKIKHAVSRGSSKIRWIVERGMVKPKGKVVDLGCGRGGWSYYMATLKNVTEVKGYTKGGPGHEEPIPMATYGWNLVKLHSGVDVFYKPTEQVDTLLCDIGESSSNPTIEEGRTLRVLKMVEPWLSSKPEFCIKVLNPYMPTVIEELEKLQRKHGGNLVRCPLSRNSTHEMYWVSGASGNIVSSVNTTSKMLLNRFTTRHRKPTYEKDVDLGAGTRSVSTETEKPDMTIIGRRLQRLQEEHKETWHYDQENPYRTWAYHGSYEAPSTGSASSMVNGVVKLLTKPWDVIPMVTQLAMTDTTPFGQQRVFKEKVDTRTPQPKPGTRMVMTTTANWLWALLGKKKNPRLCTREEFISKVRSNAAIGAVFQEEQGWTSASEAVNDSRFWELVDKERALHQEGKCESCVYNMMGKREKKLGEFGRAKGSRAIWYMWLGARFLEFEALGFLNEDHWFGRENSWSGVEGEGLHRLGYILEEIDKKDGDLMYADDTAGWDTRITEDDLQNEELITEQMAPHHKILAKAIFKLTYQNKVVKVLRPTPRGAVMDIISRKDQRGSGQVGTYGLNTFTNMEVQLIRQMEAEGVITQDDMQNPKGLKERVEKWLKECGVDRLKRMAISGDDCVVKPLDERFGTSLLFLNDMGKVRKDIPQWEPSKGWKNWQEVPFCSHHFHKIFMKDGRSLVVPCRNQDELIGRARISQGAGWSLRETACLGKAYAQMWSLMYFHRRDLRLASMAICSAVPTEWFPTSRTTWSIHAHHQWMTTEDMLKVWNRVWIEDNPNMTDKTPVHSWEDIPYLGKREDLWCGSLIGLSSRATWAKNIHTAITQVRNLIGKEEYVDYMPVMKRYSAPSESEGVL
	DENV-1	Nauru Island Western Pacific 45AZ5	NP_059433.1	N/A	Yes	MNNQRKKTGRPSFNMLKRARNRVSTVSQLAKRFSKGLLSGQGPMKLVMAFIAFLRFLAIPPTAGILARWGSFKKNGAIKVLRGFKKEISNMLNIMNRRKRSVTMLLMLLPTALAFHLTTRGGEPHMIVSKQERGKSLLFKTSAGVNMCTLIAMDLGELCEDTMTYKCPRITETEPDDVDCWCNATETWVTYGTCSQTGEHRRDKRSVALAPHVGLGLETRTETWMSSEGAWKQIQKVETWALRHPGFTVIALFLAHAIGTSITQKGIIFILLMLVTPSMAMRCVGIGNRDFVEGLSGATWVDVVLEHGSCVTTMAKDKPTLDIELLKTEVTNPAVLRKLCIEAKISNTTTDSRCPTQGEATLVEEQDTNFVCRRTFVDRGWGNGCGLFGKGSLITCAKFKCVTKLEGKIVQYENLKYSVIVTVHTGDQHQVGNETTEHGTTATITPQAPTSEIQLTDYGALTLDCSPRTGLDFNEMVLLTMKKKSWLVHKQWFLDLPLPWTSGASTSQETWNRQDLLVTFKTAHAKKQEVVVLGSQEGAMHTALTGATEIQTSGTTTIFAGHLKCRLKMDKLILKGMSYVMCTGSFKLEKEVAETQHGTVLVQVKYEGTDAPCKIPFSSQDEKGVTQNGRLITANPIVTDKEKPVNIEAEPPFGESYIVVGAGEKALKLSWFKKGSSIGKMFEATARGARRMAILGDTAWDFGSIGGVFTSVGKLIHQIFGTAYGVLFSGVSWTMKIGIGILLTWLGLNSRSTSLSMTCIAVGMVTLYLGVMVQADSGCVINWKGRELKCGSGIFVTNEVHTWTEQYKFQADSPKRLSAAIGKAWEEGVCGIRSATRLENIMWKQISNELNHILLENDMKFTVVVGDVSGILAQGKKMIRPQPMEHKYSWKSWGKAKIIGADVQNTTFIIDGPNTPECPDNQRAWNIWEVEDYGFGIFTTNIWLKLRDSYTQVCDHRLMSAAIKDSKAVHADMGYWIESEKNETWKLARASFIEVKTCIWPKSHTLWSNGVLESEMIIPKIYGGPISQHNYRPGYFTQTAGPWHLGKLELDFDLCEGTTVVVDEHCGNRGPSLRTTTVTGKTIHEWCCRSCTLPPLRFKGEDGCWYGMEIRPVKEKEENLVKSMVSAGSGEVDSFSLGLLCISIMIEEVMRSRWSRKMLMTGTLAVFLLLTMGQLTWNDLIRLCIMVGANASDKMGMGTTYLALMATFRMRPMFAVGLLFRRLTSREVLLLTVGLSLVASVELPNSLEELGDGLAMGIMMLKLLTDFQSHQLWATLLSLTFVKTTFSLHYAWKTMAMILSIVSLFPLCLSTTSQKTTWLPVLLGSLGCKPLTMFLITENKIWGRKSWPLNEGIMAVGIVSILLSSLLKNDVPLAGPLIAGGMLIACYVISGSSADLSLEKAAEVSWEEEAEHSGASHNILVEVQDDGTMKIKDEERDDTLTILLKATLLAISGVYPMSIPATLFVWYFWQKKKQRSGVLWDTPSPPEVERAVLDDGIYRILQRGLLGRSQVGVGVFQEGVFHTMWHVTRGAVLMYQGKRLEPSWASVKKDLISYGGGWRFQGSWNAGEEVQVIAVEPGKNPKNVQTAPGTFKTPEGEVGAIALDFKPGTSGSPIVNREGKIVGLYGNGVVTTSGTYVSAIAQAKASQEGPLPEIEDEVFRKRNLTIMDLHPGSGKTRRYLPAIVREAIKRKLRTLVLAPTRVVASEMAEALKGMPIRYQTTAVKSEHTGKEIVDLMCHATFTMRLLSPVRVPNYNMIIMDEAHFTDPASIAARGYISTRVGMGEAAAIFMTATPPGSVEAFPQSNAVIQDEERDIPERSWNSGYDWITDFPGKTVWFVPSIKSGNDIANCLRKNGKRVVQLSRKTFDTEYQKTKNNDWDYVVTTDISEMGANFRADRVIDPRRCLKPVILKDGPERVILAGPMPVTVASAAQRRGRIGRNQNKEGDQYIYMGQPLNNDEDHAHWTEAKMLLDNINTPEGIIPALFEPEREKSAAIDGEYRLRGEARKTFVELMRRGDLPVWLSYKVASEGFQYSDRRWCFDGERNNQVLEENMDVEIWTKEGERKKLRPRWLDARTYSDPLALREFKEFAAGRRSVSGDLILEIGKLPQHLTQRAQNALDNLVMLHNSEQGGKAYRHAMEELPDTIETLMLLALIAVLTGGVTLFFLSGRGLGKTSIGLLCVIASSALLWMASVEPHWIAASIILEFFLMVLLIPEPDRQRTPQDNQLAYVVIGLLFMILTVAANEMGLLETTKKDLGIGHAAAENHHHAAMLDVDLHPASAWTLYAVATTIITPMMRHTIENTTANISLTAIANQAAILMGLDKGWPISKMDIGVPLLALGCYSQVNPLTLTAAVLMLVAHYAIIGPGLQAKATREAQKRTAAGIMKNPTVDGIVAIDLDPVVYDAKFEKQLGQIMLLILCTSQILLMRTTWALCESITLATGPLTTLWEGSPGKFWNTTIAVSMANIFRGSYLAGAGLAFSLMKSLGGGRRGTGAQGETLGEKWKRQLNQLSKSEFNTYKRSGIIEVDRSEAKEGLKRGETTKHAVSRGTAKLRWFVERNLVKPEGKVIDLGCGRGGWSYYCAGLKKVTEVKGYTKGGPGHEEPIPMATYGWNLVKLYSGKDVFFTPPEKCDTLLCDIGESSPNPTIEEGRTLRVLKMVEPWLRGNQFCIKILNPYMPSVVETLEQMQRKHGGMLVRNPLSRNSTHEMYWVSCGTGNIVSAVNMTSRMLLNRFTMAHRKPTYERDVDLGAGTRHVAVEPEVANLDIIGQRIENIKNEHKSTWHYDEDNPYKTWAYHGSYEVKPSGSASSMVNGVVRLLTKPWDVIPMVTQIAMTDTTPFGQQRVFKEKVDTRTPKAKRGTAQIMEVTARWLWGFLSRNKKPRICTREEFTRKVRSNAAIGAVFVDENQWNSAKEAVEDERFWDLVHRERELHKQGKCATCVYNMMGKREKKLGEFGKAKGSRAIWYMWLGARFLEFEALGFMNEDHWFSRENSLSGVEGEGLHKLGYILRDISKIPGGNMYADDTAGWDTRITEDDLQNEAKITDIMEPEHALLATSIFKLTYQNKVVRVQRPAKNGTVMDVISRRDQRGSGQVGTYGLNTFTNMEAQLIRQMESEGIFSPSELETPNLAERVLDWLKKHGTERLKRMAISGDDCVVKPIDDRFATALTALNDMGKVRKDIPQWEPSKGWNDWQQVPFCSHHFHQLIMKDGREIVVPCRNQDELVGRARVSQGAGWSLRETACLGKSYAQMWQLMYFHRRDLRLAANAICSAVPVDWVPTSRTTWSIHAHHQWMTTEDMLSVWNRVWIEENPWMEDKTHVSSWEDVPYLGKREDQWCGSLIGLTARATWATNIQVAINQVRRLIGNENYLDFMTSMKRFKNESDPEGALW
	DENV-2	ngc-2007	QBK46951.1	Thailand	Yes	MNNQRKKARNTPFNMLKRERNRVSTVQQLTKRFSLGMLQGRGPLKLFMALVAFLRFLTIPPTAGILKRWGTIKKSKAINVLRGFRKEIGRMLNILNRRRRTAGMIIMLIPTVMAFHLTTRNGEPHMIVSRQEKGKSLLFKTEDGVNMCTLMAMDLGELCEDTITYKCPFLKQNEPEDIDCWCNSTSTWVTYGTCTTTGEHRREKRSVALVPHVGMGLETRTETWMSSEGAWKHAQRIETWILRHPGFTIMAAILAYTIGTTHFQRALIFILLTAVAPSMTMRCIGISNRDFVEGVSGGSWVDIVLEHGSCVTTMAKNKPTLDFELIKTEAKQPATLRKYCIEAKLTNTTTDSRCPTQGEPSLNEEQDKRFVCKHSMVDRGWGNGCGLFGKGGIVTCAMFTCKKNMKGKVVQPENLEYTIVITPHSGEEHAVGNDTGKHGKEIKITPQSSITEAELTGYGTVTMECSPRTGLDFNEMVLLQMENKAWLVHRQWFLDLPLPWLPGADTQGSNWIQKETLVTFKNPHAKKQDVVVLGSQEGAMHTALTGATEIQMSSGNLLFTGHLKCRLRMDKLQLKGMSYSMCTGKFKVVKEIAETQHGTIVIRVQYEGDGSPCKIPFEIMDLEKRHVLGRLITVNPIVTEKDSPVNIEAEPPFGDSYIIIGVEPGQLKLNWFKKGSSIGQMIETTMRGAKRMAILGDTAWDFGSLGGVFTSIGKALHQVFGAIYGAAFSGVSWIMKILIGVIITWIGMNSRSTSLSVSLVLVGVVTLYLGVMVQADSGCVVSWKNKELKCGSGIFITDNVHTWTEQYKFQPESPSKLASAIQKAHEEGICGIRSVTRLENLMWKQITPELNHILSENEVKLTIMTGDIKGIMQAGKRSLQPQPTELKYSWKTWGKAKMLSTESHNQTFLIDGPETAECPNTNRAWNSLEVEDYGFGVFTTNIWLKLREKQDVFCDSKLMSAAIKDNRAVHADMGYWIESALNDTWKIEKASFIEVKSCHWPKSHTLWSNGVLESEMIIPKNFAGPVSQHNYRPGYHTQTAGPWHLGKLEMDFDFCEGTTVVVTEDCGNRGPSLRTTTASGKLITEWCCRSCTLPPLRYRGEDGCWYGMEIRPLKEKEENLVNSLVTAGHGQIDNFSLGVLGMALFLEEMLRTRVGTKHAILLVAVSFVTLITGNMSFRDLGRVMVMVGATMTDDIGMGVTYLALLAAFKVRPTFAAGLLLRKLTSKELMMTTIGIVLLSQSTIPETILELTDALALGMMVLKMVRKMEKYQLAVTIMAILCVPNAVILQNAWKVSCTILAVVSVSPLFLTSSQQKADWIPLALTIKGLNPTAIFLTTLSRTNKKRSWPLNEAIMAVGMVSILASSLLKNDIPMTGPLVAGGLLTVCYVLTGRSADLELERAADVKWEDQAEISGSSPILSITISEDGSMSIKNEEEEQTLTILIRTGLLVISGLFPVSIPITAAAWYLWEVKKQRAGVLWDVPSPPPVGKAELEDGAYRIKQKGILGYSQIGAGVYKEGTFHTMWHVTRGAVLMHKGKRIEPSWADVKKDLISYGGGWKLEGEWKEGEEVQVLALEPGKNPRAVQTKPGLFKTNAGTIGAVSLDFSPGTSGSPIIDKKGKVVGLYGNGVVTRSGAYVSAIAQTEKSIEDNPEIEDDIFRKRKLTIMDLHPGAGKTKRYLPAIVREAIKRGLRTLILAPTRVVAAEMEEALRGLPIRYQTPAIRAEHTGREIVDLMCHATFTMRLLSPVRVPNYNLIIMDEAHFTDPASIAARGYISTRVEMGEAAGIFMTATPPGSRDPFPQSNAPIMDEEREIPERSWSSGHEWVTDFKGKTVWFVPSIKAGNDIAACLRKNGKKVIQLSRKTFDSEYVKTRTNDWDFVVTTDISEMGANFKAERVIDPRRCMKPVILTDGEERVILAGPMPVTHSSAAQRRGRIGRNPKNENDQYIYMGEPLENDEDCAHWKEAKMLLDNINTPEGIIPSMFEPEREKVDAIDGEYRLRGEARKTFVDLMRRGDLPVWLAYRVAAEGINYADRRWCFDGIKNNQILEENVEVEIWTKEGERKKLKPRWLDARIYSDPLALKEFKEFAAGRKSLTLNLITEMGRLPTFMTQKARDALDNLAVLHTAEAGGRAYNHALSELPETLETLLLLTLLATVTGGIFLFLMSGRGIGKMTLGMCCIITASILLWYAQIQPHWIAASIILEFFLIVLLIPEPEKQRTPQDNQLTYVVIAILTVVAATMANEMGFLEKTKKDLGLGSITTQQPESNILDIDLRPASAWTLYAVATTFVTPMLRHSIENSSVNVSLTAIANQATVLMGLGKGWPLSKMDIGVPLLAIGCYSQVNPITLTAALFLLVAHYAIIGPGLQAKATREAQKRAAAGIMKNPTVDGITVIDLDPIPYDPKFEKQLGQVMLLVLCVTQVLMMRTTWALCEALTLATGPISTLWEGNPGRFWNTTIAVSMANIFRGSYLAGAGLLFSIMKNTTNTRRGTGNIGETLGEKWKSRLNALGKSEFQIYKKSGIQEVDRTLAKEGIKRGETDHHAVSRGSAKLRWFVERNMVTPEGKVVDLGCGRGGWSYYCGGLKNVREVKGLTKGGPGHEEPIPMSTYGWNLVRLQSGVDVFFTPPEKCDTLLCDIGESSPNPTVEAGRTLRVLNLVENWLNNNTQFCIKVLNPYMPSVIEKMEALQRKYGGALVRNPLSRNSTHEMYWVSNASGNIVSSVNMISRMLINRFTMRHKKATYEPDVDLGSGTRNIGIESEIPNLDIIGKRIEKIKQEHETSWHYDQDHPYKTWAYHGSYETKQTGSASSMVNGVVRLLTKPWDVVPMVTQMAMTDTTPFGQQRVFKEKVDTRTQEPKEGTKKLMKITAEWLWKELGKKKTPRMCTREEFTRKVRSNAALGAIFTDENKWKSAREAVEDSRFWELVDKERNLHLEGKCETCVYNMMGKREKKLGEFGKAKGSRAIWYMWLGARFLEFEALGFLNEDHWFSRENSLSGVEGEGLHKLGYILRDVSKKEGGAMYADDTAGWDTRITLEDLKNEEMVTNHMEGEHKKLAEAIFKLTYQNKVVRVQRPTPRGTVMDIISRRDQRGSGQVGTYGLNTFTNMEAQLIRQMEGEGVFKSIQHLTVTEEIAVQNWLARVGRERLSRMAISGDDCVVKPLDDRFASALTALNDMGKVRKDIQQWEPSRGWNDWTQVPFCSHHFHELIMKDGRVLVVPCRNQDELIGRARISQGAGWSLRETACLGKSYAQMWSLMYFHRRDLRLAANAICSAVPSHWVPTSRTTWSIHAKHEWMTTEDMLTVWNRVWIQENPWMEDKTPVESWEEIPYLGKREDQWCGSLIGLTSRATWAKNIQTAINQVRSLIGNEEYTDYMPSMKRFRREEEEAGVLW
	DENV-3	Sleman/78	AAT69740.1	Indonesia	Yes	MNNQRKKTGKPSINMLKRVRNRVSTGSQLAKRFSRGLLNGQGPMKLVMAFIAFLRFLAIPPTAGVLARWGTFKKSGAIKVLRGFKKEISNMLSIINRRKKTSLCLMMMLPATLAFHLTSRDGEPRMIVGKNERGKSLLFKTASGINMCTLIAMDLGEMCDDTVTYKCPLITEVEPEDIDCWCNLTSTWVTYGTCNQAGEHRRDKRSVALAPHVGMGLDTRTQTWMSAEGAWRQVEKVETWAFRHPGFTILALFLAHYIGTSLTQKVVIFILLMLVTPSMTMRCVGVGNRDFVEGLSGATWVDVVLEHGGCVTTMAKNKPTLDIELQKTEATQLATLRKLCIEGKITNVTTDSRCPTQGEAILPEEQDQNHVCKHTYVDRGWGNGCGLFGKGSLVTCAKFQCLESIEGKVVQHENLKYTVIITVHTGDQHQVGNETQGVTAEITPQASTVEAILPEYGTLGLECSPRTGLDFNEMILLTMKNKAWMVHRQWFFDLPLPWTSGATTETPTWNKKELLVTFKNAHAKKQEVVVLGSQEGAMHTALTGATEIQTSGGTSIFAGHLKCRLKMDKLELKGMSYAMCLNAFVLKKEVSETQHGTILIKVEYKGEDAPCKIPFSTEDGQGKAHNGRLITANPVVTKKEEPVNIEAEPPFGESNIVIGIGDKALKINWYKKGSSIGKMFEATARGARRMAILGDTAWDFGSVGGVLNSLGKMVHQIFGSAYTALFSGVSWIMKIGIGVLLTWIGLNSKNTSMSFSCIVIGIITLYLGAVVQADMGCVINWKGKELKCGSGIFVTNEVHTWTEQYKFQADSPKRLATAIAGAWENGVCGIRSTTRMENLLWKQIANELNYILWENNIKLTVVVGDIIGVLEQGKRTLTPQPMELKYSWKTWGKAKIVTAETQNSSFIIDGPNTPECPSASRAWNVWEVEDYGFGVFTTNIWLKLREMYTQLCDHRLMSAAVKDERAVHADMGYWIESQKNGSWKLEKASLIEVKTCTWPKSHTLWSNGVLESDMIIPKSLAGPISQHNYRPGYHTQTAGPWHLGKLELDFNYCEGTTVVITENCGTRGPSLRTTTVSGKLIHEWCCRSCTLPPLRYMGEDGCWYGMEIRPINEKEENMVKSLVSAGSGKVDNFTMGVLCLAILFEEVMRGKFGKKHMIAGVLFTFVLLLSGQITWRDMAHTLIMIGSNASDRMGMGVTYLALIATFKIQPFLALGFFLRKLTSRENLLLGVGLAMATTLQLPEDIEQMANGIALGLMALKLITQFETYQLWTALVSLMCSNTIFTLTVAWRTATLILAGISLLPVCQSSSMRKTDWLPMAVAAMGVPPLPLFIFSLKDTLKRRSWPLNEGVMAVGLVSILASSLLRNDVPMAGPLVAGGLLIACYVITGTSADLTVEKAADVTWEEEAEQTGVSHNLMITVDDDGTMRIKDDETENILTVLLKTALLIVSGIFPYSIPATLLVWHTWQKQTQRSGVLWDVPSPPETQKAELEEGVYRIKQQGIFGKTQVGVGVQKEGVFHTMWHVTRGAVLTHNGKRLEPNWASVKKDLISYGGGWKLSAQWQKGEEVQVIAVEPGKNPKNFQTMPGIFQTTTGEIGAIALDFKPGTSGSPIINREGKVLGLYGNGVVTKNGGYVSGIAQTNAEPDGPTPELEEEMFKKRNLTIMDLHPGSGKTRKYLPAIVREAIKRRLRTLILAPTRVVAAEMEEALKGLPIRYQTTATKSEHTGREIVDLMCHATFTMRLLSPVRVPNYNLIIMDEAHFTDPASIAARGYISTRVGMGEAAAIFMTATPPGTADAFPQSNAPIQDEERDIPERSWNSGNEWITDFAGKTVWFVPSIKAGNDIANCLRKNGKKVIQLSRKTFDTEYQKTKLNDWDFVVTTDISEMGANFKADRVIDPRRCLKPVILTDGPERVILAGPMPVTVASAAQRRGRVGRNPQKENDQYIFMGQPLNNDEDHAHWTEAKMLLDNINTPEGIIPALFEPEREKSAAIDGEYRLKGESRKTFVELMRRGDLPVWLAHKVASEGIKYTDRKWCFDGERNNQILEENMDVEIWTKEGEKKKLRPRWLDARTYSDPLALKEFKDFAAGRKSIALDLVTEIGRVPSHLAHRTRNALDNLVMLHTSEHGGRAYRHAVEELPETMETLLLLGLMILLTGGAMLFLISGKGIGKTSIGLICVAASSGMLWMADVPLQWIASAIVLEFFMMVLLIPEPEKQRTPQDNQLAYVVIGILTLAAIVAANEMGLLETTKRDLGMSKEPGVVSPTSYLDVDLHPASAWTLYAVATTVITPMLRHTIENSTANVSLAAIANQAVVLMGLDKGWPISKMDLGVPLLALGCYSQVNPLTLTAAVLLLVTHYAIIGPGLQAKATREAQKRTAAGIMKNPTVDGIMTIDLDPVIYDSKFEKQLGQVMLLVLCAVQLLLMRTSWAFCEALTLATGPITTLWEGSPGKFWNTTIAVSMANIFRGSYLAGAGLAFSIMKSVGTGKRGTGSQGETLGEKWKKKLNQLPRKEFDLYKKSGITEVDRTEAKEGLKRGEITHHAVSRGSAKLQWFVERNMVIPEGRVIDLGCGRGGWSYYCAGLKKVTEVRGYTKGGPGHEEPVPMSTYGWNIVKLMSGKDVFYLPPEKCDTLLCDIGESSPSPTVEESRTIRVLKMVEPWLKNNQFCIKVLNPYMPTVIEHLERLQRKHGGMLVRNPLSRNSTHEMYWISNGTGNIVSSVNMVSRLLLNRFTMTHRRPTIEKDVDLGAGTRHVNAEPETPNMDVIGERIRRIKEEHSSTWHYDDENPYKTWAYHGSYEVKATGSASSMINGVVKLLTKPWDVVPMVTQMAMTDTTPFGQQRVFKEKVDTRTPRPMPGTRKVMEITAEWLWRTLGRNKRPRLCTREEFTKKVRTNAAMGAVFTEENQWDSARAAVEDEEFWKLVDRERELHKLGKCGSCVYNMMGKREKKLGEFGKAKGSRAIWYMWLGARYLEFEALGFLNEDHWFSRENSYSGVEGEGLHKLGYILRDISKIPGGAMYADDTAGWDTRITEDDLHNEEKITQQMDPEHRQLANAIFKLTYQNKVVKVQRPTPKGTVMDIISRKDQRGSGQVGTYGLNTFTNMEAQLIRQMEGEGVLSKADLENPHLLEKKVTQWLETKGVERLKRMAISGDDCVVKPIDDRFANALLALNDMGKVRKDIPQWQPSKGWHDWQQVPFCSHHFHELIMKDGRKLVVPCRPQDELIGRARISQGAGWSLRETACLGKAYAQMWTLMYFHRRDLRLASNAICSAVPVHWVPTSRTTWSIHAHHQWMTTEDMLTVWNRVWIEDNPWMEDKTPVKTWEDVPYLGKREDQWCGSLIGLTSRATWAQNILTAIQQVRSLIGNEEFLDYMPSMKRFRKEEESEGAIW
DPIV	DENV-4	814669	AAK01233.1	N/A	Yes	MNQRKKVVRPPFNMLKRERNRVSTPQGLVKRFSTGLFSGKGPLRMVLAFITFLRVLSIPPTAGILKRWGQLKKNKAIKILIGFRKEIGRMLNILNGRKRSTITLLCLIPTVMAFSLSTRDGEPLMIVAKHERGRPLLFKTTEGINKCTLIAMDLGEMCEDTVTYKCPLLVNTEPEDIDCWCNLTSTWVMYGTCTQSGERRREKRSVALTPHSGMGLETRAETWMSSEGAWKHAQRVESWILRNPGFALLAGFMAYMIGQTGIQRTVFFVLMMLVAPSYGMRCVGVGNRDFVEGVSGGAWVDLVLEHGGCVTTMAQGKPTLDFELTKTTAKEVALLRTYCIEASISNITTATRCPTQGEPYLKEEQDQQYICRRDVVDRGWGNGCGLFGKGGVVTCAKFSCSGKITGNLVQIENLEYTVVVTVHNGDTHAVGNDTSNHGVTAMITPRSPSVEVKLPDYGELTLDCEPRSGIDFNEMILMKMKKKTWLVHKQWFLDLPLPWTAGADTSEVHWNYKERMVTFKVPHAKRQDVTVLGSQEGAMHSALAGATEVDSGDGNHMFAGHLKCKVRMEKLRIKGMSYTMCSGKFSIDKEMAETQHGTTVVKVKYEGAGAPCKVPIEIRDVNKEKVVGRIISSTPLAENTNSVTNIELEPPFGDSYIVIGVGNSALTLHWFRKGSSIGKMFESTYRGAKRMAILGETAWDFGSVGGLFTSLGKAVHQVFGSVYTTMFGGVSWMIRILIGFLVLWIGTNSRNTSMAMTCIAVGGITLFLGFTVQADMGCVVSWSGKELKCGSGIFVVDNVHTWTEQYKFQPESPARLASAILNAHKDGVCGIRSTTRLENVMWKQITNELNYVLWEGGHDLTVVAGDVKGVLTKGKRALTPPVSDLKYSWKTWGKAKIFTPEARNSTFLIDGPDTSECPNERRAWNSLEVEDYGFGMFTTNIWMKFREGSSEVCDHRLMSAAIKDQKAVHADMGYWIESSKNQTWQIEKASLIEVKTCLWPKTHTLWSNGVLESQMLIPKSYAGPFSQHNYRQGYATQTVGPWHLGKLEIDFGECPGTTVTIQEDCDHRGPSLRTTTASGKLVTQWCCRSCTMPPLRFLGEDGCWYGMEIRPLSEKEENMVKSQVTAGQGTSETFSMGLLCLTLFVEECLRRRVTRKHMILVVVITLCAIILGGLTWMDLLRALIMLGDTMSGRIGGQIHLAIMAVFKMSPGYVLGVFLRKLTSRETALMVIGMAMTTVLSIPHDLMELIDGISLGLILLKIVTQFDNTQVGTLALSLTFIRSTMPLVMAWRTIMAVLFVVTLIPLCRTSCLQKQSHWVEITALILGAQALPVYLMTLMKGASRRSWPLNEGIMAVGLVSLLGSALLKNDVPLAGPMVAGGLLLAAYVMSGSSADLSLEKAANVQWDEMADITGSSPIIEVKQDEDGSFSIRDVEETNMITLLVKLALITVSGLYPLAIPVTMTLWYMWQVKTQRSGALWDVPSPAATKKAALSEGVYRIMQRGLFGKTQVGVGIHMEGVFHTMWHVTRGSVICHETGRLEPSWADVRNDMISYGGGWRLGDKWDKEEDVQVLAIEPGKNPKHVQTKPGLFKTLTGEIGAVTLDFKPGTSGSPIINRKGKVIGLYGNGVVTKSGDYVSAITQAERIGEPDYEVDEDIFRKKRLTIMDLHPGAGKTKRILPSIVREALKRRLRTLILAPTRVVAAEMEEALRGLPIRYQTPAVKSEHTGREIVDLMCHATFTTRLLSSTRVPNYNLIVMDEAHFTDPSSVAARGYISTRVEMGEAAAIFMTATPPGATDPFPQSNSPIEDIEREIPERSWNTGFDWITDYQGKTVWFVPSIKAGNDIANCLRKSGKKVIQLSRKTFDTEYPKTKLTDWDFVVTTDISEMGANFRAGRVIDPRRCLKPVILTDGPERVILAGPIPVTPASAAQRRGRIGRNPAQEDDQYVFSGDPLKNDEDHAHWTEAKMLLDNIYTPEGIIPTLFGPEREKTQAIDGEFRLRGEQRKTFVELMRRGDLPVWLSYKVASAGISYKDREWCFTGERNNQILEENMEVEIWTREGEKKKLRPRWLDARVYADPMALKDFKEFASGRKSITLDILTEIASLPTYLSSRAKLALDNIVMLHTTERGGRAYQHALNELPESLETLMLVALLGAMTAGIFLFFMQGKGIGKLSMGLITIAVASGLLWVAEIQPQWIAASIILEFFLMVLLIPEPEKQRTPQDNQLIYVILTILTIIGLIAANEMGLIEKTKTDFGFYQVKTETTILDVDLRPASAWTLYAVATTILTPMLRHTIENTSANLSLAAIANQAAVLMGLGKGWPLHRMDLGVPLLAMGCYSQVNPTTLTASLVMLLVHYAIIGPGLQAKATREAQKRTAAGIMKNPTVDGITVIDLEPISYDPKFEKQLGQVMLLVLCAGQLLLMRTTWAFCEVLTLATGPILTLWEGNPGRFWNTTIAVSTANIFRGSYLAGAGLAFSLIKNAQTPRRGTGTTGETLGEKWKRQLNSLDRREFEEYKRSGILEVDRTEAKSALKDGSKIKHAVSRGSSKIRWIVERGMVKPKGKVVDLGCGRGGWSYYMATLKNVTEVKGYTKGGPGHEEPIPMATYGWNLVKLHSGVDVFYKPTEQVDTLLCDIGESSSNPTIEEGRTLRVLKMVEPWLSSKPEFCIKVLNPYMPTVIEELEKLQRKHGGNLVRCPLSRNSTHEMYWVSGASGNIVSSVNTTSKMLLNRFTTRHRKPTYEKDVDLGAGTRSVSTETEKPDMTIIGRRLQRLQEEHKETWHYDQENPYRTWAYHGSYEAPSTGSASSMVNGVVKLLTKPWDVIPMVTQLAMTDTTPFGQQRVFKEKVDTRTPQPKPGTRMVMTTTANWLWALLGKKKNPRLCTREEFISKVRSNAAIGAVFQEEQGWTSASEAVNDSRFWELVDKERALHQEGKCESCVYNMMGKREKKLGEFGRAKGSRAIWYMWLGARFLEFEALGFLNEDHWFGRENSWSGVEGEGLHRLGYILEEIDKKDGDLMYADDTAGWDTRITEDDLQNEELITEQMAPHHKILAKAIFKLTYQNKVVKVLRPTPRGAVMDIISRKDQRGSGQVGTYGLNTFTNMEVQLIRQMEAEGVITQDDMQNPKGLKERVEKWLKECGVDRLKRMAISGDDCVVKPLDERFGTSLLFLNDMGKVRKDIPQWEPSKGWKNWQEVPFCSHHFHKIFMKDGRSLVVPCRNQDELIGRARISQGAGWSLRETACLGKAYAQMWSLMYFHRRDLRLASMAICSAVPTEWFPTSRTTWSIHAHHQWMTTEDMLKVWNRVWIEDNPNMTDKTPVHSWEDIPYLGKREDLWCGSLIGLSSRATWAKNIHTAITQVRNLIGKEEYVDYMPVMKRYSAPSESEGVL
	DENV-1	PUO 359	AAN32784.1	Thailand	No	MRCVGIGNRDFVEGLSGATWVDVVLEHGSCVTTMAKDKPTLDIELLKTEVTNPAVLRKLCIEAKISNTTTDSRCPTQGEATLVEEQDANFVCRRTFVDRGWGNGCGLFGKGSLITCAKFKCVTKLEGKIVQYENLKYSVIVTVHTGDQHQVGNESTEHGTTATITPQAPTSEIQLTDYGALTLDCSPRTGLDFNEMVLLTMKEKSWLVHKQWFLDLPLPWTSGATTSQETWNRQDLLVTFKTAHAKKQEVVVLGSQEGAMHTALTGATEIQTSGTTTIFAGHLKCRLKMDKLTLKGMSYVMCTGSFKLEKEVAETQHGTVLVQVKYEGTDAPCKIPFSTQDEKGVTQNGRVITANPIVTDKEKPVNIEAEPPFGESYIVVGAGEKALKLSWFKKGSTIGKMFEATARGARRMAILGDTAWDFGSIGGVFTSVGKLVHQIFGTAYGVLFSGVSWTMKIGIGVLLTWLGLNSRSTSLSMTCIAVGLVTLYLGVMVQAELPNSLEELGDGLAMGIMILKLLTDFQSHQLWAALLSLTFIKTTFSLHYAWKTMAMVLSIVSLFPLCLSTTSQKTTWLPVLLGSLGCKPLTMFLIAENKIWGRKSWPLNEGIMAVGIVSILLSSLLKNDVPLAGPLIAGGMLIACYVISGSSADLSLEKAAEVSWE
	DENV-2	New Guinea C/PUO-218 hybrid	VIPR_ALG4_2723943_937_2421.1	N/A	Yes	MRCIGISNRDFVEGVSGGSWVDIVLEHGSCVTTMAKNKPTLDFELIKTEAKQPATLRKYCIEAKLTNTTTESRCPTQGEPSLNEEQDKRFVCKHSMVDRGWGNGCGLFGKGGIVTCAMFTCKKNMEGKVVQPENLEYTIVVTPHSGEEHAVGNDTGKHGKEIKVTPQSSITEAELTGYGTVTMECSPRTGLDFNEMVLLQMENKAWLVHRQWFLDLPLPWLPGADTQGSNWIQKETLVTFKNPHAKKQDVVVLGSQEGAMHTALTGATEIQMSSGNLLFTGHLKCRLRMDKLQLKGMSYSMCTGKFKVVKEIAETQHGTIVIRVQYEGDGSPCKIPFEIMDLEKRHVLGRLITVNPIVTEKDSPVNIEAEPPFGDSYIIIGVEPGQLKLNWFKKGSSIGQMFETTMRGAKRMAILGDTAWDFGSLGGVFTSIGKALHQVFGAIYGAAFSGVSWTMKILIGVIITWIGMNSRSTSLSVSLVLVGVVTLYLGVMVQAFHLTTRNGEPHMIVSRQEKGKSLLFKTEDGVNMCTLMAMDLGELCEDTITYKCPLLRQNEPEDIDCWCNSTSTWVTYGTCTTTGEHRREKRSVALVPHVGMGLETRTETWMSSEGAWKHAQRIEIWILRHPGFTIMAAILAYTIGTTHFQRALIFILLTAVAPSMT
Dengvaxia	DENV-3	PaH881/88	VIPR_ALG4_13310785_595_2073.1	Thailand	No	MRCVGVGNRDFVEGLSGATWVDVVLEHGGCVTTMAKNKPTLDIELQKTEATQLATLRKLCIEGKITNITTDSRCPTQGEAILPEEQDQNYVCKHTYVDRGWGNGCGLFGKGSLVTCAKFQCLESIEGKVVQHENLKYTVIITVHTGNQHQVGNDTQGVTAEITPQASTVEAILPEYGTLGLECSPRTGLDFNEMILLTMKNKAWMVHRQWFFDLPLPWTSGATTETPTWNRKELLVTFKNAHAKKQEVVVLGSQEGAMHTALTGATEIQNSGGTSIFAGHLKCRLKMDKLELKGMSYAMCLNTFVLKKEVSETQHGTILIKVEYKGEDAPCKIPFSTEDGQGKAHNGRLITANPVVTKKEEPVNIEAEPPFGESNIVIGIGDKALKINWYKKGSSIGKMFEATARGARRMAILGDTAWDFGSVGGVLNSLGKMVHQIFGSAYTALFSGVSWIMKIGIGVLLTWIGLNSKNTSMSFSCVAIGIITLYLGAVVQA
	DENV-4	1228	AER00190.1	Indonesia	No	MRCVGVGNRDFVEGVSGGAWVDLVLEHGGCVTTMAQGKPTLDFELTKTTAKEVALLRTYCIEASISNITTATRCPTQGEPYLKEEQDQQYICRRDVVDRGWGNGCGLFGKGGVVTCAKFLCSGKITGNLVQIENLEYTVVVTVHNGDTHAVGNDTSNHGVTATITPRSPSVEVKLPDYGELTLDCEPRSGIDFNEMILMKMKKKTWLVHKQWFLDLPLPWTAGADTSEVHWNYKERMVTFKVPHAKRQDVTVLGSQEGAMHSALAGATEVDSGDGNHMFAGHLKCKVRMEKLRIKGMSYTMCSGKFSIDKEMAETQHGTTVVKVKYEGAGAPCKVPIEIRDVNKEKVVGRIISSTPFAENTNSVTNIELEPPFGDSYIVIGVGDSALTLHWFRKGSSIGKMFESTYRGAKRMAILGETAWDFGSVGGMFTSLGKAVHQVFGSVYTTMFGGVSWMVRILIGFLVLWIGTNSRNTSMAMTCIAVGGITLFLGFTVGAFHLTSRDGEPRMIVGKNERGKSLLFKTASGINMCTLIAMDLGEMCDDTVTYKCPLIAEVEPEDIDCWCNLTSTWVTYGTCNQAGEHRRDKRSVALAPHVGMGLDTRTQTWMSAEGAWRQVEKVETWALRHPGFTILALFLAHYIGTSLTQKVVIFILLMLVTPSMA
	DENV-1	16007	VIPR_ALG4_7329982_935_2419.1	N/A	Yes	MRCVGIGNRDFVEGLSGATWVDVVLEHGSCVTTMAKNKPTLDIELLKTEVTNPAVLRKLCIEAKISNTTTDSRCPTQGEATLVEEQDANFVCRRTFVDRGWGNGCGLFGKGSLITCAKFKCVTKLEGKIVQYENLKYSVIVTVHTGDQHQVGNETTEHGTTATITPQAPTSEIQLTDYGTLTLDCSPRTGLDFNEMVLLTMKERSWLVHKQWFLDLPLPWTSGASTSQETWNRQDLLVTFKTAHAKKQEVVVLGSQEGAMHTALTGATEIQTSGTTTIFAGHLKCRLKMDKLTLKGMSYVMCTGSFKLEKEVAETQHGTVLVQVKYEGTDAPCKIPFSTQDEKGATQNGRLITANPIVTDKEKPVNIEAEPPFGESYIVVGAGEKALKLSWFKKGSSIGKMFEATARGARRMAILGDTAWDFGSIGGVFTSMGKLVHQVFGTAYGVLFSGVSWTMKIGIGILLTWLGLNSRNTSLSMMCIAVGMVTLYLGVMVQAFHLTTRGGEPHMIVSKQERGKSLLFKTSAGVNMCTLIAMDLGELCEDTMTYKCPRITEAEPDDVDCWCNATDTWVTYGTCSQTGEHRRDKRSVALAPHVGLGLETRAETWMSSEGAWKQIQKVETWALRHPGFTVIALFLAHAIGTSITQKGIIFILLMLVTPSMA
	DENV-3	16562	VIPR_ALG4_1031961902_935_2413.1	N/A	Yes	MRCVGVGNRDFVEGLSGATWVDVVLEHGGCVTTMAKNKPTLDIELQKTEATQLATLRKLCIEGKITNITTDSRCPTQGEAILPEEQDQNYVCKHTYVDRGWGNGCGLFGKGSLVTCAKFQCLESIEGKVVQHENLKYTVIITVHTGDQHQVGNETQGVTAEITPQASTAEVILPEYGTLGLECSPRTGLDFNEMISLTMKNKAWMVHRQWFFDLPLPWTSGATAETPTWNRKELLVTFKNAHAKKQEVVVLGSQEGAMHTALTGATEIQTSGGTSIFAGHLKCRLKMDKLELKGMSYAMCLSSFVLKKEVSETQHGTILIKVEYKGEDAPCKIPFSTEDGQGKAHNGRLITANPVVTKKEEPVNIEAEPPFGESNIVIGIGDKALKINWYKKGSSIGKMFEATARGARRMAILGDTAWDFGSVGGVLNSLGKMVHQIFGSAYTALFGGVSWMMKIGIGVLLTWIGLNSKNTSMSFSCIAIGIITLYLGAVVQAFHLTSRDGEPRMIVGKNERGKSLLFKTASGINMCTLIAMDLGEMCDDTVTYKCPHITEVEPEDIDCWCNLTSTWVTYGTCNQAGEHRRDKRSVALAPHVGMGLDTRTQTWMSAEGAWRQVEKVETWALRHPGFTILALFLAHYIGTSLTQKVVIFILLMLVTPSMT
	DENV-4	1036	VIPR_ALG4_QTE18638_1_939_2423.1	Indonesia	Yes	MRCVGVGNRDFVEGVSGGAWVDLVLEHGGCVTTMAQGKPTLDFELTKTTAKEVALLRTYCIEASISNITTATRCPTQGEPYLKEEQDQQYICRRDVVDRGWGNGCGLFGKGGVVTCAKFSCSGKITGNLVQIENLEYTVVVTVHNGDTHAVGNDTSNHGVTATITPRSPSVEVKLPDYGELTLDCEPRSGIDFNEMILMKMKKKTWLVHKQWFLDLPLPWTAGADTSEVHWNYKERMVTFKVPHAKRQDVTVLGSQEGAMHSALAGATEVDSGDGNHMFAGHLKCKVRMEKLRIKGMSYTMCSGKFSIDKEMAETQHGTTVVKVKYEGAGAPCKVPIEIRDVNKEKVVGRIISSTPLAENTNSATNIELEPPFGDSYIVIGVGNSALTLHWFRKGSSIGKMFESTYRGAKRMAILGETAWDFGSVGGLFTSLGKAVHQVFGSVYTTMFGGVSWMIRILIGFLVLWIGTNSRNTSMAMTCIAVGGITLFLGFTVQAFHLSTRDGEPLMIVAKHERGRPLLFKTTEGINKCTLIAMDLGEMCEDTVTYKCPLLVNTEPEDIDCWCNLTSTWVMYGTCTQSGERRREKRSVALTPHSGMGLETRAETWMSSEGAWKHAQRVESWILRNPGFALLAGFMAYMIGQTGIQRTVFFVLMMLVAPSYG

DENVax	DENV-2	16681	ANG57776.1	Thailand	Yes	MNNQRKKAKNTPFNMLKRERNRVSTVQQLTKRFSLGMLQGRGPLKLFMALVAFLRFLTIPPTAGILKRWGTIKKSKAINVLRGFRKEIGRMLNILNRRRRSAGMIIMLIPTVMAFHLTTRNGEPHMIVSRQEKGKSLLFKTEDGVNMCTLMAMDLGELCEDTITYKCPLLRQNEPEDIDCWCNSTSTWVTYGTCTTMGEHRREKRSVALVPHVGMGLETRTETWMSSEGAWKHVQRIETWILRHPGFTMMAAILAYTIGTTHFQRALIFILLTAVTPSMTMRCIGMSNRDFVEGVSGGSWVDIVLEHGSCVTTMAKNKPTLDFELIKTEAKQPATLRKYCIEAKLTNTTTESRCPTQGEPSLNEEQDKRFVCKHSMVDRGWGNGCGLFGKGGIVTCAMFRCKKNMEGKVVQPENLEYTIVITPHSGEEHAVGNDTGKHGKEIKITPQSSITEAELTGYGTVTMECSPRTGLDFNEMVLLQMENKAWLVHRQWFLDLPLPWLPGADTQGSNWIQKETLVTFKNPHAKKQDVVVLGSQEGAMHTALTGATEIQMSSGNLLFTGHLKCRLRMDKLQLKGMSYSMCTGKFKVVKEIAETQHGTIVIRVQYEGDGSPCKIPFEIMDLEKRHVLGRLITVNPIVTEKDSPVNIEAEPPFGDSYIIIGVEPGQLKLNWFKKGSSIGQMFETTMRGAKRMAILGDTAWDFGSLGGVFTSIGKALHQVFGAIYGAAFSGVSWTMKILIGVIITWIGMNSRSTSLSVTLVLVGIVTLYLGVMVQADSGCVVSWKNKELKCGSGIFITDNVHTWTEQYKFQPESPSKLASAIQKAHEEGICGIRSVTRLENLMWKQITPELNHILSENEVKLTIMTGDIKGIMQAGKRSLRPQPTELKYSWKTWGKAKMLSTESHNQTFLIDGPETAECPNTNRAWNSLEVEDYGFGVFTTNIWLKLKEKQDVFCDSKLMSAAIKDNRAVHADMGYWIESALNDTWKIEKASFIEVKNCHWPKSHTLWSNGVLESEMIIPKNLAGPVSQHNYRPGYHTQITGPWHLGKLEMDFDFCDGTTVVVTEDCGNRGPSLRTTTASGKLITEWCCRSCTLPPLRYRGEDGCWYGMEIRPLKEKEENLVNSLVTAGHGQVDNFSLGVLGMALFLEEMLRTRVGTKHAILLVAVSFVTLITGNMSFRDLGRVMVMVGATMTDDIGMGVTYLALLAAFKVRPTFAAGLLLRKLTSKELMMTTIGIVLLSQSTIPETILELTDALALGMMVLKMVRNMEKYQLAVTIMAILCVPNAVILQNAWKVSCTILAVVSVSPLLLTSSQQKTDWIPLALTIKGLNPTAIFLTTLSRTSKKRSWPLNEAIMAVGMVSILASSLLKNDIPMTGPLVAGGLLTVCYVLTGRSADLELERAADVKWEDQAEISGSSPILSITISEDGSMSIKNEEEEQTLTILIRTGLLVISGLFPVSIPITAAAWYLWEVKKQRAGVLWDVPSPPPMGKAELEDGAYRIKQKGILGYSQIGAGVYKEGTFHTMWHVTRGAVLMHKGKRIEPSWADVKKDLISYGGGWKLEGEWKEGEEVQVLALEPGKNPRAVQTKPGLFKTNAGTIGAVSLDFSPGTSGSPIIDKKGKVVGLYGNGVVTRSGAYVSAIAQTEKSIEDNPEIEDDIFRKRRLTIMDLHPGAGKTKRYLPAIVREAIKRGLRTLILAPTRVVAAEMEEALRGLPIRYQTPAIRAEHTGREIVDLMCHATFTMRLLSPVRVPNYNLIIMDEAHFTDPASIAARGYISTRVEMGEAAGIFMTATPPGSRDPFPQSNAPIIDEEREIPERSWNSGHEWVTDFKGKTVWFVPSIKAGNDIAACLRKNGKKVIQLSRKTFDSEYVKTRTNDWDFVVTTDISEMGANFKAERVIDPRRCMKPVILTDGEERVILAGPMPVTHSSAAQRRGRIGRNPKNENDQYIYMGEPLENDEDCAHWKEAKMLLDNINTPEGIIPSMFEPEREKVDAIDGEYRLRGEARKTFVDLMRRGDLPVWLAYRVAAEGINYADRRWCFDGVKNNQILEENVEVEIWTKEGERKKLKPRWLDARIYSDPLALKEFKEFAAGRKSLTLNLITEMGRLPTFMTQKARDALDNLAVLHTAEAGGRAYNHALSELPETLETLLLLTLLATVTGGIFLFLMSGRGIGKMTLGMCCIITASILLWYAQIQPHWIAASIILEFFLIVLLIPEPEKQRTPQDNQLTYVVIAILTVVAATMANEMGFLEKTKKDLGLGSIATQQPESNILDIDLRPASAWTLYAVATTFVTPMLRHSIENSSVNVSLTAIANQATVLMGLGKGWPLSKMDIGVPLLAIGCYSQVNPITLTAALFLLVAHYAIIGPGLQAKATREAQKRAAAGIMKNPTVDGITVIDLDPIPYDPKFEKQLGQVMLLVLCVTQVLMMRTTWALCEALTLATGPISTLWEGNPGRFWNTTIAVSMANIFRGSYLAGAGLLFSIMKNTTNTRRGTGNIGETLGEKWKSRLNALGKSEFQIYKKSGIQEVDRTLAKEGIKRGETDHHAVSRGSAKLRWFVERNMVTPEGKVVDLGCGRGGWSYYCGGLKNVREVKGLTKGGPGHEEPIPMSTYGWNLVRLQSGVDVFFIPPEKCDTLLCDIGESSPNPTVEAGRTLRVLNLVENWLNNNTQFCIKVLNPYMPSVIEKMEALQRKYGGALVRNPLSRNSTHEMYWVSNASGNIVSSVNMISRMLINRFTMRYKKATYEPDVDLGSGTRNIGIESEIPNLDIIGKRIEKIKQEHETSWHYDQDHPYKTWAYHGSYETKQTGSASSMVNGVVRLLTKPWDVVPMVTQMAMTDTTPFGQQRVFKEKVDTRTQEPKEGTKKLMKITAEWLWKELGKKKTPRMCTREEFTRKVRSNAALGAIFTDENKWKSAREAVEDSRFWELVDKERNLHLEGKCETCVYNMMGKREKKLGEFGKAKGSRAIWYMWLGARFLEFEALGFLNEDHWFSRENSLSGVEGEGLHKLGYILRDVSKKEGGAMYADDTAGWDTRITLEDLKNEEMVTNHMEGEHKKLAEAIFKLTYQNKVVRVQRPTPRGTVMDIISRRDQRGSGQVGTYGLNTFTNMEAQLIRQMEGEGVFKSIQHLTITEEIAVQNWLARVGRERLSRMAISGDDCVVKPLDDRFASALTALNDMGKIRKDIQQWEPSRGWNDWTQVPFCSHHFHELIMKDGRVLVVPCRNQDELIGRARISQGAGWSLRETACLGKSYAQMWSLMYFHRRDLRLAANAICSAVPSHWVPTSRTTWSIHAKHEWMTTEDMLTVWNRVWIQENPWMEDKTPVESWEEIPYLGKREDQWCGSLIGLTSRATWAKNIQAAINQVRSLIGNEEYTDYMPSMKRFRREEEEAGVLW
TVDV	DENV-1	Nauru Island Western Pacific 45AZ5	NP_722460.2	N/A	Yes	MRCVGIGNRDFVEGLSGATWVDVVLEHGSCVTTMAKDKPTLDIELLKTEVTNPAVLRKLCIEAKISNTTTDSRCPTQGEATLVEEQDTNFVCRRTFVDRGWGNGCGLFGKGSLITCAKFKCVTKLEGKIVQYENLKYSVIVTVHTGDQHQVGNETTEHGTTATITPQAPTSEIQLTDYGALTLDCSPRTGLDFNEMVLLTMKKKSWLVHKQWFLDLPLPWTSGASTSQETWNRQDLLVTFKTAHAKKQEVVVLGSQEGAMHTALTGATEIQTSGTTTIFAGHLKCRLKMDKLILKGMSYVMCTGSFKLEKEVAETQHGTVLVQVKYEGTDAPCKIPFSSQDEKGVTQNGRLITANPIVTDKEKPVNIEAEPPFGESYIVVGAGEKALKLSWFKKGSSIGKMFEATARGARRMAILGDTAWDFGSIGGVFTSVGKLIHQIFGTAYGVLFSGVSWTMKIGIGILLTWLGLNSRSTSLSMTCIAVGMVTLYLGVMVQAFHLTTRGGEPHMIVSKQERGKSLLFKTSAGVNMCTLIAMDLGELCEDTMTYKCPRITETEPDDVDCWCNATETWVTYGTCSQTGEHRRDKRSVALAPHVGLGLETRTETWMSSEGAWKQIQKVETWALRHPGFTVIALFLAHAIGTSITQKGIIFILLMLVTPSMA
	DENV-3	H87	VIPR_ALG4_323525_499_1977.1	Brazil	No	MRCVGVGNRDFVEGLSGATWVDVVLEHGGCVTTMAKNKPTLDIELQKTEATQLATLRKLCIEGKITNITTDSRCPTQGEAILPEEQDQNYVCKHTYVDRGWGNGCGLFGKGSLVTCAKFQCLESIEGKVVQHENLKYTVIITVHTGDQHQVGNETQGVTAEITSQASTAEAILPEYGTLGLECSPRTGLDFNEMILLTMKNKAWMVHRQWFFDLPLPWTSGATTETPTWNRKELLVTFKNAHAKKQEVVVLGSQEGAMHTALTGATEIQTSGGTSIFAGHLKCRLKMDKLKLKGMSYAMCLNTFVLKKEVSETQHGTILIKVEYKGEDAPCKIPFSTEDGQGKAHNGRLITANPVVTKKEEPVNIEAEPPFGESNIVIGIGDKALKINWYRKGSSIGKMFEATARGARRMAILGDTAWDFGSVGGVLNSLGKMVHQIFGSAYTALFSGVSWIMKIGIGVLLTWIGLNSKNTSMSFSCIAIGIITLYLGVVVQAFHLTSRDGEPRMIVGKNERGKSLLFKTASGINMCTLIAMDLGEMCDDTVTYKCPHITEVEPEDIDCWCNLTSTWVTYGTCNQAGEHRRDKRSVALAPHVGMGLDTRTQTWMSAEGAWRQVEKVETWALRHPGFTILALFLAHYIGTSLTQKVVIFILLMLVTPSMT
	DENV-4	H241	VIPR_ALG4_61652905_931_2415.1	Philippines	Yes	MRCVGVGNRDFVEGVSGGAWVDLVLEHGGCVTTMAQGKPTLDFELIKTTAKEVALLRTYCIEASISNITTATRCPTQGEPYLKEEQDQQYICRRDVVDRGWGNGCGLFGKGGVVTCAKFSCSGKITGNLVQIENLEYTVVVTVHNGDTHAVGNDIPNHGVTATITPRSPSVEVKLPDYGELTLDCEPRSGIDFNEMILMKMKKKTWLVHKQWFLDLPLPWAAGADTSEVHWNYKERMVTFKVPHAKRQDVTVLGSQEGAMHSALTGATEVDSGDGNHMFAGHLKCKVRMEKLRIKGMSYTMCSGKFSIDKEMAETQHGTTVVKVKYEGAGAPCKVPIEIRDVNKEKVVGRIISSTPFAEYTNSVTNIELEPPFGDSYIVIGVGDSALTLHWFRKGSSIGKMLESTYRGAKRMAILGETAWDFGSVGGLLTSLGKAVHQVFGSVYTTMFGGVSWMVRILIGFLVLWIGTNSRNTSMAMTCIAVGGITLFLGFTVHAFHLSTRDGEPLMIVARHERGRPLLFKTTEGINKCTLIAMDLGEMCEDTVTYECPLLVNTEPEDIDCWCNLTSAWVMYGTCTQSGERRREKRSVALTPHSGMGLETRAETWMSSEGAWKHAQRVESWILRNPGFALLAGFMAYMIGQTGIQRTVFFVLMMLVAPSYG
	DENV-2	ngc-2007	QBK46951.1	Thailand	Yes	FHLTTRNGEPHMIVSRQEKGKSLLFKTEDGVNMCTLMAMDLGELCEDTITYKCPFLKQNEPEDIDCWCNSTSTWVTYGTCTTTGEHRREKRSVALVPHVGMGLETRTETWMSSEGAWKHAQRIETWILRHPGFTIMAAILAYTIGTTHFQRALIFILLTAVAPSMTMRCIGISNRDFVEGVSGGSWVDIVLEHGSCVTTMAKNKPTLDFELIKTEAKQPATLRKYCIEAKLTNTTTDSRCPTQGEPSLNEEQDKRFVCKHSMVDRGWGNGCGLFGKGGIVTCAMFTCKKNMKGKVVQPENLEYTIVITPHSGEEHAVGNDTGKHGKEIKITPQSSITEAELTGYGTVTMECSPRTGLDFNEMVLLQMENKAWLVHRQWFLDLPLPWLPGADTQGSNWIQKETLVTFKNPHAKKQDVVVLGSQEGAMHTALTGATEIQMSSGNLLFTGHLKCRLRMDKLQLKGMSYSMCTGKFKVVKEIAETQHGTIVIRVQYEGDGSPCKIPFEIMDLEKRHVLGRLITVNPIVTEKDSPVNIEAEPPFGDSYIIIGVEPGQLKLNWFKKG

CD4 DENV-1 Epitopes

											DENV-1 constructs of vaccines (100% identity=1,green ; else 0, red)					
Epitope_ID	AA_Seq	Protein_Name	Start	End	Allele	Step	Serotype	Method/Technique	Assay Group	Qualitative Measure	TV003	TDEN	DPIV	Dengvaxia	DENVax	TVDV
195246	EISNMLNIMNRRKRT	C	87	101	HLA-DRB1*13:01	0%	Dengue virus 1	ELISPOT	IFNg release	Positive	0	0	0	0	0	0
195560	KEVSNMLNIMNRRKR	C	86	100	HLA-DRB1*13:01	0%	Dengue virus 1	ELISPOT	IFNg release	Positive	0	0	0	0	0	0
195884	RGFKKEISNMLSIMN	C	82	96	HLA-DRB1*04:01	0%	Dengue virus 1	ELISPOT	IFNg release	Positive	0	0	0	0	0	0
196188	VSVLFMLLPTALAFH	C	102	116	HLA-DRB1*01:01	0%	Dengue virus 1	ELISPOT	IFNg release	Positive	0	0	0	0	0	0
539048	AFMAFLRFLAIPPTA	C	27	41	HLA-DRB1*15:02	0%	Dengue virus 1	ELISPOT	IFNg release	Positive	0	0	0	0	0	0
539681	MLNIRNRRKRSVTML	C	91	105	HLA-DRB1*13:01	0%	Dengue virus 1	ELISPOT	IFNg release	Positive	0	0	0	0	0	0
539816	QPSFNMLKRARNRVS	C	10	24	HLA-DRB1*08:03	0%	Dengue virus 1	ELISPOT	IFNg release	Positive	0	0	0	0	0	0
195359	GGLFTSVGKLIHQVF	E	706	720	HLA-DRB1*11:01	0%	Dengue virus 1	ELISPOT	IFNg release	Positive	0	0	0	0	0	0
195484	IGGLFTSVGKLIHQV	E	705	719	HLA-DRB1*07:01	0%	Dengue virus 1	ELISPOT	IFNg release	Positive	0	0	0	0	0	0
539840	REDLLVTFKTAHAKK	E	513	527	HLA-DRB1*04:03	0%	Dengue virus 1	ELISPOT	IFNg release	Positive	0	0	0	0	0	0
539854	RKDLLVTFKTAHAKK	E	513	527	HLA-DRB1*04:03	0%	Dengue virus 1	ELISPOT	IFNg release	Positive	0	0	0	0	0	0
867045	HAKKQEVAVLGSQEG	E	244	258	HLA-DQA1*03:01/DQB1*03:02	0%	Dengue virus 1	ELISPOT	IFNg release	Positive	0	0	0	0	0	0
869085	SYIVIGAGKKALKLS	E	376	390	HLA-DRB5*01:01	0%	Dengue virus 1	ELISPOT	IFNg release	Positive	0	0	0	0	0	0
869690	WLAHKQWFLDLPLPW	E	206	220	HLA-DQA1*01:01/DQB1*05:01	0%	Dengue virus 1	ELISPOT	IFNg release	Positive	0	0	0	0	0	0
195684	LNHILLENDIKFTVV	NS1	850	864	HLA-DRB1*03:01	0%	Dengue virus 1	ELISPOT	IFNg release	Positive	0	0	0	0	0	0
195690	LNYILLENDMKFTVV	NS1	850	864	HLA-DRB1*03:01	0%	Dengue virus 1	ELISPOT	IFNg release	Positive	0	0	0	0	0	0
591686	KFTVVVGDVSGILAQ	NS1	860	874	HLA-DRB1*03:01	0%	Dengue virus 1	ELISPOT	IFNg release	Positive	1	1	1	0	0	0
868492	QISNELNYILLENDM	NS1	845	859	HLA-DPB1*01:01	0%	Dengue virus 1	ELISPOT	IFNg release	Positive	0	0	0	0	0	0
868630	QYKFQADSPKKLSAA	NS1	806	820	HLA-DRB3*02:02	0%	Dengue virus 1	ELISPOT	IFNg release	Positive	0	0	0	0	0	0
195196	AWKTMAMALSIVSLF	NS2a	1291	1305	HLA-DRB1*08:02	0%	Dengue virus 1	ELISPOT	IFNg release	Positive	0	0	0	0	0	0
195285	FAVGLLFRRLTSREI	NS2a	1214	1228	HLA-DRB1*11:01	0%	Dengue virus 1	ELISPOT	IFNg release	Positive	0	0	0	0	0	0
195323	FQSHQLWATLLSLTF	NS2a	1267	1281	HLA-DPB1*04:02	0%	Dengue virus 1	ELISPOT	IFNg release	Positive	1	1	1	0	0	0
195389	GLLFRKLTSREVLLL	NS2a	1217	1231	HLA-DRB1*11:01	0%	Dengue virus 1	ELISPOT	IFNg release	Positive	0	0	0	0	0	0
195390	GLLFRRLTSREILLL	NS2a	1217	1231	HLA-DRB1*07:01	0%	Dengue virus 1	ELISPOT	IFNg release	Positive	0	0	0	0	0	0
195392	GLLFRRLTSREVPLL	NS2a	1217	1231	HLA-DRB1*10:01	0%	Dengue virus 1	ELISPOT	IFNg release	Positive	0	0	0	0	0	0
195422	GTLAVFLLLIMGQLT	NS2a	1162	1176	HLA-DRB1*01:01	0%	Dengue virus 1	ELISPOT	IFNg release	Positive	0	0	0	0	0	0
195423	GTLVVFFLLIMGQLT	NS2a	1162	1176	HLA-DRB1*15:01	0%	Dengue virus 1	ELISPOT	IFNg release	Positive	0	0	0	0	0	0
195613	LAVFLLLIMGQLTWN	NS2a	1164	1178	HLA-DRB1*15:06	0%	Dengue virus 1	ELISPOT	IFNg release	Positive	0	0	0	0	0	0
195614	LAVGLLFRRLTSREV	NS2a	1214	1228	HLA-DRB1*11:01	0%	Dengue virus 1	ELISPOT	IFNg release	Positive	0	0	0	0	0	0
195670	LLSLTFIKTTFSLHY	NS2a	1276	1290	HLA-DRB1*07:01	0%	Dengue virus 1	ELISPOT	IFNg release	Positive	0	0	0	1	0	0
195713	LSLTFIRTSLSLDYA	NS2a	1277	1291	HLA-DRB1*03:01	0%	Dengue virus 1	ELISPOT	IFNg release	Positive	0	0	0	0	0	0
195756	MGLLFRRLTSREVLL	NS2a	1216	1230	HLA-DRB1*11:01	0%	Dengue virus 1	ELISPOT	IFNg release	Positive	0	0	0	0	0	0
195881	REVLLLTVGLSLVAC	NS2a	1226	1240	HLA-DRB1*07:01	0%	Dengue virus 1	ELISPOT	IFNg release	Positive	0	0	0	0	0	0
195959	SILIEEVMRSRWSRK	NS2a	1143	1157	HLA-DRB1*11:04	0%	Dengue virus 1	ELISPOT	IFNg release	Positive	0	0	0	0	0	0
539624	LSLTFIKTTLSLHYA	NS2a	1277	1291	HLA-DRB1*14:04	0%	Dengue virus 1	ELISPOT	IFNg release	Positive	0	0	0	0	0	0
539625	LSLTFIKTTYSLHYA	NS2a	1277	1291	HLA-DRB1*14:04	0%	Dengue virus 1	ELISPOT	IFNg release	Positive	0	0	0	0	0	0
539626	LSLTFIRSTIPLVMA	NS2a	1275	1289	HLA-DRB1*14:04	0%	Dengue virus 1	ELISPOT	IFNg release	Positive	0	0	0	0	0	0
539651	LVVFLLLIMGQLTWS	NS2a	1164	1178	HLA-DRB1*15:06	0%	Dengue virus 1	ELISPOT	IFNg release	Positive	0	0	0	0	0	0
539657	MALSIVSLFPLCLST	NS2a	1297	1311	HLA-DRB1*15:06	0%	Dengue virus 1	ELISPOT	IFNg release	Positive	0	0	0	0	0	0
539679	MLMTGTLAVFLLLIM	NS2a	1158	1172	HLA-DRB1*15:06	0%	Dengue virus 1	ELISPOT	IFNg release	Positive	0	0	0	0	0	0
539680	MLMTGTLVVFFLLIM	NS2a	1158	1172	HLA-DRB1*15:06	0%	Dengue virus 1	ELISPOT	IFNg release	Positive	0	0	0	0	0	0
539912	SLDYAWKTMAMALSI	NS2a	1287	1301	HLA-DRB1*09:01	0%	Dengue virus 1	ELISPOT	IFNg release	Positive	0	0	0	0	0	0
539918	SLTFIKTTLSLHHAW	NS2a	1278	1292	HLA-DRB1*14:04	0%	Dengue virus 1	ELISPOT	IFNg release	Positive	0	0	0	0	0	0
539974	TGTLVVFLLLTLGQL	NS2a	1161	1175	HLA-DRB1*15:06	0%	Dengue virus 1	ELISPOT	IFNg release	Positive	0	0	0	0	0	0
540006	TTLLSLTFIRTSLSL	NS2a	1274	1288	HLA-DRB1*12:02	0%	Dengue virus 1	ELISPOT	IFNg release	Positive	0	0	0	0	0	0
591779	TLAVFLLLTMGQLTW	NS2a	36	50	HLA-DRB1*11:01	0%	Dengue virus 1	ELISPOT	IFNg release	Positive	1	1	1	0	0	0
739424	LAVFLLLVMGQLTWN	NS2a	1164	1178	HLA-DRB1*11:04	0%	Dengue virus 1	ELISPOT	IFNg release	Positive	0	0	0	0	0	0
739578	LSLTFIKTTLSLHHA	NS2a	1277	1291	HLA-DRB1*04:07	0%	Dengue virus 1	ELISPOT	IFNg release	Positive	0	0	0	0	0	0
866118	AMALSIVSLFPLCLS	NS2a	1296	1310	HLA-DPB1*04:01	0%	Dengue virus 1	ELISPOT	IFNg release	Positive	0	0	0	0	0	0
866767	FQSYQLWATLLSLTF	NS2a	1267	1281	HLA-DPB1*04:02	0%	Dengue virus 1	ELISPOT	IFNg release	Positive	0	0	0	0	0	0
866873	GILAVFFLLIMGQLT	NS2a	1162	1176	HLA-DPB1*04:02	0%	Dengue virus 1	ELISPOT	IFNg release	Positive	0	0	0	0	0	0
867997	LTSREILLLTVGLSL	NS2a	1223	1237	HLA-DPB1*04:02	0%	Dengue virus 1	ELISPOT	IFNg release	Positive	0	0	0	0	0	0
868553	QPHQLWATLLSLTFV	NS2a	1268	1282	HLA-DPB1*04:02	0%	Dengue virus 1	ELISPOT	IFNg release	Positive	0	0	0	0	0	0
869103	TDFQPHQLWTTLLSL	NS2a	1265	1279	HLA-DPB1*04:02	0%	Dengue virus 1	ELISPOT	IFNg release	Positive	0	0	0	0	0	0
866200	ATLFLWYFWQKKKQR	NS2b	1461	1475	HLA-DPB1*01:01	0%	Dengue virus 1	ELISPOT	IFNg release	Positive	0	0	0	0	0	0
24915	HTGKEIVDLMCHATE	NS3	251	265	HLA-DPw2	0%	Dengue virus 1	ICS	IFNg release	Positive	0	0	0	0	0	0
539839	REAIRRKLRTLILAP	NS3	1685	1699	HLA-DRB1*14:04	0%	Dengue virus 1	ELISPOT	IFNg release	Positive	0	0	0	0	0	0
539993	TNCLRKNGKRVIQLS	NS3	1848	1862	HLA-DRB1*13:01	0%	Dengue virus 1	ELISPOT	IFNg release	Positive	0	0	0	0	0	0
540071	VREAIKKKLRTLILA	NS3	1684	1698	HLA-DRB1*12:02	0%	Dengue virus 1	ELISPOT	IFNg release	Positive	0	0	0	0	0	0
540075	VREAIKRKLRTLVLA	NS3	1684	1698	HLA-DRB1*12:02	0%	Dengue virus 1	ELISPOT	IFNg release	Positive	1	1	1	0	0	0
54391	RKLTIMDLHPGSGKT	NS3	1662	1676	HLA class II	0%	Dengue virus 1	ICS	IFNg release	Positive	0	0	0	0	0	0
738417	AAAIFMTATPPGSAE	NS3	1785	1799	HLA-DRB1*04:07	0%	Dengue virus 1	ELISPOT	IFNg release	Positive	0	0	0	0	0	0
738418	AAAIFMTATPPGSME	NS3	1785	1799	HLA-DRB1*04:07	0%	Dengue virus 1	ELISPOT	IFNg release	Positive	0	0	0	0	0	0
195596	KTSIGLLCVTASSAL	NS4a	2174	2188	HLA-DRB1*07:01	0%	Dengue virus 1	ELISPOT	IFNg release	Positive	0	0	0	0	0	0
195680	LMLLALTAVLTGGVT	NS4a	2149	2163	HLA-DRB1*01:01	0%	Dengue virus 1	ELISPOT	IFNg release	Positive	0	0	0	0	0	0
196046	TIETLMLLALIAALT	NS4a	2145	2159	HLA-DRB1*01:01	0%	Dengue virus 1	ELISPOT	IFNg release	Positive	0	0	0	0	0	0
739085	GVTLFFLSGRGLGKT	NS4a	2161	2175	HLA-DRB1*01:02	0%	Dengue virus 1	ELISPOT	IFNg release	Positive	1	1	1	0	0	0
195308	FMLVAHYAIIGPGLQ	NS4b	2357	2371	HLA-DRB1*07:01	0%	Dengue virus 1	ELISPOT	IFNg release	Positive	0	0	0	0	0	0
868336	PLTLTAAVLMLAAHY	NS4b	2349	2363	HLA-DQA1*01:02/DQB1*06:02	0%	Dengue virus 1	ELISPOT	IFNg release	Positive	0	0	0	0	0	0
195781	NEDHWFSRGNSLSGV	NS5	2985	2999	HLA-DRB1*04:01	0%	Dengue virus 1	ELISPOT	IFNg release	Positive	0	0	0	0	0	0
539509	KSEFNIYKRSGIMEV	NS5	2515	2529	HLA-DRB1*15:02	0%	Dengue virus 1	ELISPOT	IFNg release	Positive	0	0	0	0	0	0
539537	LAKAIFKLTYQNKVV	NS5	3056	3070	HLA-DRB1*14:04	0%	Dengue virus 1	ELISPOT	IFNg release	Positive	0	0	0	0	0	0
195368	GIIFILLMLVTPSMA	PreM	266	280	HLA-DRB1*01:01	100%	Dengue virus 1	ELISPOT	IFNg release	Positive	1	1	1	0	1	1
195562	KGIIFILLMLVTPSM	PreM	265	279	HLA-DRB1*08:02	100%	Dengue virus 1	ELISPOT	IFNg release	Positive	1	1	1	0	1	1
195477	IELLKTEVTNPAVLR	E	38	52	HLA-DRB1*04:01	100%	Dengue virus 1	ELISPOT	IFNg release	Positive	1	1	1	1	1	1
540052	VLGSQEGAMHTALTGA	E	247	262	HLA class II	100%	Dengue virus 1	ICS	IFNg release	Positive	1	1	1	1	1	1
540104	VVVLGSQEGAMHTALTG	E	245	261	HLA class II	100%	Dengue virus 1	ICS	IFNg release	Positive	1	1	1	1	1	1
95154	SSIGKMFEATARG	E	396	408	HLA-DQ8	100%	Dengue virus 1	ELISPOT	IFNg release	Positive	1	1	1	0	1	1
866284	DDQRAWNIWEVEDYG	NS1	181	195	HLA-DQA1*01:01/DQB1*05:01	100%	Dengue virus 1	ELISPOT	IFNg release	Positive	0	0	0	0	0	0
867041	HADMGYWIESEKNET	NS1	231	245	HLA-DQA1*01:01/DQB1*05:01	100%	Dengue virus 1	ELISPOT	IFNg release	Positive	1	1	1	0	0	0
868574	QRAWNIWEVEDYGFG	NS1	183	197	HLA-DQA1*01:01/DQB1*05:01	100%	Dengue virus 1	ELISPOT	IFNg release	Positive	1	1	1	0	0	0
195983	SREVLLLTIGLSLVA	NS2a	1225	1239	HLA-DRB1*15:01	100%	Dengue virus 1	ELISPOT	IFNg release	Positive	0	0	0	0	0	0
539888	RWSRKMLMTGTLAVF	NS2a	1153	1167	HLA-DRB1*04:03	100%	Dengue virus 1	ELISPOT	IFNg release	Positive	1	1	1	0	0	0
195338	FTMRLLSPVRVPNYN	NS3	1741	1755	HLA-DRB1*01:01	100%	Dengue virus 1	ELISPOT	IFNg release	Positive	1	1	1	0	0	0
196130	VILKDGPERVILAGP	NS3	1908	1922	HLA-DRB1*03:01	100%	Dengue virus 1	ELISPOT	IFNg release	Positive	1	1	1	0	0	0
539579	LKGMPIRYQTTAVKS	NS3	1711	1725	HLA-DRB1*04:03	100%	Dengue virus 1	ELISPOT	IFNg release	Positive	1	1	1	0	0	0
867396	KGMPIRYQTTAVKSE	NS3	1712	1726	HLA-DRB3*02:02	100%	Dengue virus 1	ELISPOT	IFNg release	Positive	1	1	1	0	0	0
868343	PNYNMIIMDEAHFTD	NS3	1752	1766	HLA-DQA1*05:01/DQB1*02:01	100%	Dengue virus 1	ELISPOT	IFNg release	Positive	1	1	1	0	0	0
195478	IETLMLLALIAVLTG	NS4a	2146	2160	HLA-DRB1*15:01	100%	Dengue virus 1	ELISPOT	IFNg release	Positive	1	1	1	0	0	0
195679	LMLLALIAVLTGGVT	NS4a	2149	2163	HLA-DRB1*08:02	100%	Dengue virus 1	ELISPOT	IFNg release	Positive	1	1	1	0	0	0
868132	MLLALIAVLTGGVTL	NS4a	2150	2164	HLA-DPB1*04:02	100%	Dengue virus 1	ELISPOT	IFNg release	Positive	1	1	1	0	0	0
195114	AGAGLAFSLMKSLGG	NS4b	2476	2490	HLA-DRB1*04:01	100%	Dengue virus 1	ELISPOT	IFNg release	Positive	1	1	1	0	0	0
195193	AVLMLVAHYAIIGPG	NS4b	2355	2369	HLA-DRB1*01:01	100%	Dengue virus 1	ELISPOT	IFNg release	Positive	1	1	1	0	0	0
195476	IDLDPVVYDAKFEKQ	NS4b	2398	2412	HLA-DRB1*03:01	100%	Dengue virus 1	ELISPOT	IFNg release	Positive	1	1	1	0	0	0
195620	LCTSQILLMRTTWAL	NS4b	2421	2435	HLA-DRB1*04:01	100%	Dengue virus 1	ELISPOT	IFNg release	Positive	1	1	1	0	0	0
195637	LGQIMLLILCTSQIL	NS4b	2413	2427	HLA-DRB1*08:02	100%	Dengue virus 1	ELISPOT	IFNg release	Positive	1	1	1	0	0	0
195682	LMLVAHYAIIGPGLQ	NS4b	2357	2371	HLA-DRB1*07:01	100%	Dengue virus 1	ELISPOT	IFNg release	Positive	1	1	1	0	0	0
195760	MLLILCTSQILLMRT	NS4b	2417	2431	HLA-DRB1*07:01	100%	Dengue virus 1	ELISPOT	IFNg release	Positive	1	1	1	0	0	0
195848	QIMLLILCTSQILLM	NS4b	2415	2429	HLA-DRB1*07:01	100%	Dengue virus 1	ELISPOT	IFNg release	Positive	1	1	1	0	0	0
196143	VLMLVAHYAIIGPGL	NS4b	2356	2370	HLA-DRB1*01:01	100%	Dengue virus 1	ELISPOT	IFNg release	Positive	1	1	1	0	0	0
1066544	ACLGKSYAQMWQLMY	NS5	3244	3258	HLA-DRB1*04:01	100%	Dengue virus 1	ELISPOT	IFNg release	Positive-Low	1	1	1	0	0	0
195168	AQMWQLMYFHRRDLR	NS5	3251	3265	HLA-DRB1*13:01	100%	Dengue virus 1	ELISPOT	IFNg release	Positive	1	1	1	0	0	0
195208	CVYNMMGKREKKLGE	NS5	2942	2956	HLA-DRB1*11:01	100%	Dengue virus 1	ELISPOT	IFNg release	Positive	1	1	1	0	0	0
195212	DDRFATALTALNDMG	NS5	3163	3177	HLA-DRB1*04:01	100%	Dengue virus 1	ELISPOT	IFNg release	Positive	1	1	1	0	0	0
195360	GGMLVRNPLSRNSTH	NS5	2694	2708	HLA-DRB1*08:02	100%	Dengue virus 1	ELISPOT	IFNg release	Positive	1	1	1	0	0	0
195956	SIFKLTYQNKVVRVQ	NS5	3059	3073	HLA-DRB1*09:01	100%	Dengue virus 1	ELISPOT	IFNg release	Positive	1	1	1	0	0	0
196031	TCVYNMMGKREKKLG	NS5	2941	2955	HLA-DRB1*11:01	100%	Dengue virus 1	ELISPOT	IFNg release	Positive	1	1	1	0	0	0
196039	TGNIVSAVNMTSRML	NS5	2717	2731	HLA-DRB1*04:01	100%	Dengue virus 1	ELISPOT	IFNg release	Positive	1	1	1	0	0	0
539107	AVNMTSRMLLNRFTM	NS5	2723	2737	HLA-DRB1*15:02	100%	Dengue virus 1	ELISPOT	IFNg release	Positive	1	1	1	0	0	0
540138	WQLMYFHRRDLRLAA	NS5	3254	3268	HLA-DRB1*14:04	100%	Dengue virus 1	ELISPOT	IFNg release	Positive	1	1	1	0	0	0
867505	KSYAQMWQLMYFHRR	NS5	3248	3262	HLA-DPB1*01:01	100%	Dengue virus 1	ELISPOT	IFNg release	Positive	1	1	1	0	0	0
195113	AFLRFLAIPPTAGIL	C	30	44	HLA-DRB1*01:01	81% - <100%	Dengue virus 1	ELISPOT	IFNg release	Positive	1	1	1	0	0	0
195117	AGILARWSSFKKNGA	C	51	65	HLA-DRB1*15:01	81% - <100%	Dengue virus 1	ELISPOT	IFNg release	Positive	0	0	0	0	0	0
195299	FIAFLRFLAIPPTAG	C	28	42	HLA-DRB1*01:01	81% - <100%	Dengue virus 1	ELISPOT	IFNg release	Positive	1	1	1	0	0	0
195314	FNMLKRARNRVSTGS	C	1	15	HLA-DRB1*08:02	81% - <100%	Dengue virus 1	ELISPOT	IFNg release	Positive	0	0	0	0	0	0
195468	IAFLRFLAIPPTAGI	C	29	43	HLA-DRB1*01:01	81% - <100%	Dengue virus 1	ELISPOT	IFNg release	Positive	1	1	1	0	0	0
195586	KRSVTMLLMLLPTAL	C	87	101	HLA-DRB1*01:01	81% - <100%	Dengue virus 1	ELISPOT	IFNg release	Positive	1	1	1	0	0	0
195681	LMLLPTALAFHLTTR	C	94	108	HLA-DRB1*04:01	81% - <100%	Dengue virus 1	ELISPOT	IFNg release	Positive	1	1	1	0	0	0
195761	MLNIMNRRKRSVTML	C	69	83	HLA-DRB1*08:02	81% - <100%	Dengue virus 1	ELISPOT	IFNg release	Positive	1	1	1	0	0	0
195904	RPSFNMLKRARNRVS	C	10	24	HLA-DRB1*11:01	81% - <100%	Dengue virus 1	ELISPOT	IFNg release	Positive	1	1	1	0	0	0
195944	SFNMLKRARNRVSTG	C	12	26	HLA-DRB1*11:01	81% - <100%	Dengue virus 1	ELISPOT	IFNg release	Positive	0	0	0	0	0	0
196043	TGRPSFNMLKRARNR	C	8	22	HLA-DRB1*11:01	81% - <100%	Dengue virus 1	ELISPOT	IFNg release	Positive	1	1	1	0	0	0
196151	VMAFIAFLRFLAIPP	C	25	39	HLA-DRB1*11:01	81% - <100%	Dengue virus 1	ELISPOT	IFNg release	Positive	1	1	1	0	0	0
196196	VTMLLMLLPTALAFH	C	90	104	HLA-DRB1*04:01	81% - <100%	Dengue virus 1	ELISPOT	IFNg release	Positive	1	1	1	0	0	0
539043	AFIAFLRFLAIPPTA	C	27	41	HLA-DRB1*12:02	81% - <100%	Dengue virus 1	ELISPOT	IFNg release	Positive	1	1	1	0	0	0
539259	FKKNGAIKVLRGFKK	C	50	64	HLA-DRB1*08:03	81% - <100%	Dengue virus 1	ELISPOT	IFNg release	Positive	1	1	1	0	0	0

539495	KNGAIKVLRGFKKEI	C	52	66	HLA-DRB1*12:02	81% - <100%	Dengue virus 1	ELISPOT	IFNg release	Positive	1	1	1	0	0	0
539613	LRFLAIPPTAGILAR	C	32	46	HLA-DRB1*04:03	81% - <100%	Dengue virus 1	ELISPOT	IFNg release	Positive	1	1	1	0	0	0
539711	NGAIKVLRGFKKEIS	C	53	67	HLA-DRB1*13:01	81% - <100%	Dengue virus 1	ELISPOT	IFNg release	Positive	1	1	1	0	0	0
539785	PSFNMLKRARNRVST	C	11	25	HLA-DRB1*10:01	81% - <100%	Dengue virus 1	ELISPOT	IFNg release	Positive	1	1	1	0	0	0
738927	FLRFLAIPPTAGILA	C	41	55	HLA-DRB1*14:02	81% - <100%	Dengue virus 1	ELISPOT	IFNg release	Positive	1	1	1	0	0	0
195887	RGKSLLFKTSAGVNM	PreM	111	125	HLA-DRB1*07:01	81% - <100%	Dengue virus 1	ELISPOT	IFNg release	Positive	1	1	1	0	1	1
539516	KSLLFKTSAGVNMCT	PreM	123	137	HLA-DRB1*09:01	81% - <100%	Dengue virus 1	ELISPOT	IFNg release	Positive	1	1	1	0	1	1
867912	LRHPGFTVIALFLAH	PreM	242	256	HLA-DPB1*02:01	81% - <100%	Dengue virus 1	ELISPOT	IFNg release	Positive	1	1	1	0	1	1
195145	AKFKCVTKLEGKIVQ	E	117	131	HLA-DRB1*11:01	81% - <100%	Dengue virus 1	ELISPOT	IFNg release	Positive	1	1	1	1	1	1
195216	DIELLKTEVTNPAVL	E	37	51	HLA-DRB1*04:01	81% - <100%	Dengue virus 1	ELISPOT	IFNg release	Positive	1	1	1	1	1	1
195260	ENLKYSVIVTVHTGD	E	128	142	HLA-DRB1*07:01	81% - <100%	Dengue virus 1	ELISPOT	IFNg release	Positive	1	1	1	1	1	1
195362	GGVFTSVGKLVHQVF	E	426	440	HLA-DRB1*11:01	81% - <100%	Dengue virus 1	ELISPOT	IFNg release	Positive	0	0	0	0	0	0
195547	IVQYENLKYSVIVTV	E	124	138	HLA-DRB1*15:02	81% - <100%	Dengue virus 1	ELISPOT	IFNg release	Positive	1	1	1	1	1	1
195623	LDIELLKTEVTNPAV	E	36	50	HLA-DRB1*04:01	81% - <100%	Dengue virus 1	ELISPOT	IFNg release	Positive	1	1	1	1	1	1
195646	LITANPIVTDKEKPV	E	346	360	HLA-DRB1*03:01	81% - <100%	Dengue virus 1	ELISPOT	IFNg release	Positive	1	1	1	0	1	1
195814	NRQDLLVTFKTAHAK	E	227	241	HLA-DRB1*04:01	81% - <100%	Dengue virus 1	ELISPOT	IFNg release	Positive	1	1	1	1	1	1
195874	QYENLKYSVIVTVHT	E	126	140	HLA-DRB1*07:01	81% - <100%	Dengue virus 1	ELISPOT	IFNg release	Positive	1	1	1	1	1	1
196053	TLDIELLKTEVTNPA	E	35	49	HLA-DRB1*04:01	81% - <100%	Dengue virus 1	ELISPOT	IFNg release	Positive	1	1	1	1	1	1
539169	DLLVTFKTAHAKKQE	E	230	244	HLA-DRB1*14:04	81% - <100%	Dengue virus 1	ELISPOT	IFNg release	Positive	1	1	1	1	1	1
539378	HAKKQEVVVLGSQEGAMH	E	239	256	HLA class II	81% - <100%	Dengue virus 1	ICS	IFNg release	Positive	1	1	1	1	1	1
539593	LLVTFKTAHAKKQEV	E	236	250	HLA-DRB1*14:02	81% - <100%	Dengue virus 1	ELISPOT	IFNg release	Positive	1	1	1	1	1	1
539800	QDLLVTFKTAHAKKQ	E	229	243	HLA-DRB1*09:01	81% - <100%	Dengue virus 1	ELISPOT	IFNg release	Positive	1	1	1	1	1	1
539869	RQDLLVTFKTAHAKK	E	228	242	HLA-DRB1*08:03	81% - <100%	Dengue virus 1	ELISPOT	IFNg release	Positive	1	1	1	1	1	1
741745	MRCVGIGNRDFVEGLSGATW	E	281	300	HLA class II	81% - <100%	Dengue virus 1	ELISPOT	IFNg release	Positive	1	1	1	1	1	1
868013	LVHKQWFLDLPLPWT	E	487	501	HLA-DQA1*01:01/DQB1*05:01	81% - <100%	Dengue virus 1	ELISPOT	IFNg release	Positive	1	1	1	1	1	1
869691	WLVHKQWFLDLPLPW	E	486	500	HLA-DQA1*01:01/DQB1*05:01	81% - <100%	Dengue virus 1	ELISPOT	IFNg release	Positive	1	1	1	1	1	1
132583	QYKFQADSPKRLSAA	NS1	31	45	HLA-DRB1*03:01	81% - <100%	Dengue virus 1	ELISPOT	IFNg release	Positive	1	1	1	0	0	0
195579	KNETWKLARASFIEV	NS1	206	220	HLA-DRB1*07:01	81% - <100%	Dengue virus 1	ELISPOT	IFNg release	Positive	1	1	1	0	0	0
195685	LNHILLENDMKFTVV	NS1	75	89	HLA-DRB1*03:01	81% - <100%	Dengue virus 1	ELISPOT	IFNg release	Positive	1	1	1	0	0	0
195784	NETWKLARASFIEVK	NS1	207	221	HLA-DRB1*07:01	81% - <100%	Dengue virus 1	ELISPOT	IFNg release	Positive	1	1	1	0	0	0
539401	IESEKNETWKLARASFI	NS1	202	218	HLA class II	81% - <100%	Dengue virus 1	ICS	IFNg release	Positive	1	1	1	0	0	0
869135	TEQYKFQADSPKRLS	NS1	65	79	HLA-DRB5*01:01	81% - <100%	Dengue virus 1	ELISPOT	IFNg release	Positive	1	1	1	0	0	0
195286	FAVGLLFRRLTSREV	NS2a	1214	1228	HLA-DRB1*08:03	81% - <100%	Dengue virus 1	ELISPOT	IFNg release	Positive	1	1	1	0	0	0
195391	GLLFRRLTSREVLLL	NS2a	1217	1231	HLA-DRB1*08:03	81% - <100%	Dengue virus 1	ELISPOT	IFNg release	Positive	1	1	1	0	0	0
195665	LLLTIGLSLVASVEL	NS2a	1229	1243	HLA-DRB1*07:01	81% - <100%	Dengue virus 1	ELISPOT	IFNg release	Positive	0	0	0	0	0	0
195674	LLTIGLSLVASVELP	NS2a	1230	1244	HLA-DRB1*07:01	81% - <100%	Dengue virus 1	ELISPOT	IFNg release	Positive	0	0	0	0	0	0
195708	LSIVSLFPLCLSTTS	NS2a	1299	1313	HLA-DRB1*01:01	81% - <100%	Dengue virus 1	ELISPOT	IFNg release	Positive	1	1	1	1	0	0
539094	ATFKMRPMFAVGLLF	NS2a	1206	1220	HLA-DRB1*01:02	81% - <100%	Dengue virus 1	ELISPOT	IFNg release	Positive	0	0	0	0	0	0
539205	EEVMRSRWSRKMLMT	NS2a	1147	1161	HLA-DRB1*13:01	81% - <100%	Dengue virus 1	ELISPOT	IFNg release	Positive	1	1	1	0	0	0
539398	IEEVMRSRWSRKMLM	NS2a	1146	1160	HLA-DRB1*13:01	81% - <100%	Dengue virus 1	ELISPOT	IFNg release	Positive	1	1	1	0	0	0
540038	VGLLFRRLTSREVLL	NS2a	1216	1230	HLA-DRB1*08:03	81% - <100%	Dengue virus 1	ELISPOT	IFNg release	Positive	1	1	1	0	0	0
540155	YLALMATFKMRPMFA	NS2a	1201	1215	HLA-DRB1*13:01	81% - <100%	Dengue virus 1	ELISPOT	IFNg release	Positive	0	0	0	0	0	0
738553	AVGLLFRRLTSREVL	NS2a	1215	1229	HLA-DRB1*01:02	81% - <100%	Dengue virus 1	ELISPOT	IFNg release	Positive	1	1	1	0	0	0
868098	MGMGTTYLALMATFK	NS2a	456	470	HLA-DRB5*01:01	81% - <100%	Dengue virus 1	ELISPOT	IFNg release	Positive	0	0	0	0	0	0
868100	MGQLTWNDLIRLCIM	NS2a	433	447	HLA-DPB1*04:02	81% - <100%	Dengue virus 1	ELISPOT	IFNg release	Positive	1	1	1	0	0	0
195191	AVGIVSILLSSLLKN	NS2b	1355	1369	HLA-DRB1*08:02	81% - <100%	Dengue virus 1	ELISPOT	IFNg release	Positive	1	1	1	1	0	0
195228	DTLTILLKATLLAVS	NS2b	1438	1452	HLA-DRB1*08:02	81% - <100%	Dengue virus 1	ELISPOT	IFNg release	Positive	0	0	0	0	0	0
195508	ILVEVQDDGTMKIKD	NS2b	68	82	HLA-DRB1*03:01	81% - <100%	Dengue virus 1	ELISPOT	IFNg release	Positive	1	1	1	0	0	0
195510	IMAVGIVSILLSSLL	NS2b	1353	1367	HLA-DRB1*07:01	81% - <100%	Dengue virus 1	ELISPOT	IFNg release	Positive	1	1	1	1	0	0
195715	LSSLLKNDVPLAGPL	NS2b	1363	1377	HLA-DRB1*03:01	81% - <100%	Dengue virus 1	ELISPOT	IFNg release	Positive	1	1	1	1	0	0
195724	LTILLKATLLAVSGV	NS2b	1440	1454	HLA-DRB1*08:02	81% - <100%	Dengue virus 1	ELISPOT	IFNg release	Positive	0	0	0	0	0	0
195878	RDDTLTILLKATLLA	NS2b	1436	1450	HLA-DRB1*12:02	81% - <100%	Dengue virus 1	ELISPOT	IFNg release	Positive	1	1	1	0	0	0
195993	SSLLKNDVPLAGPLI	NS2b	1364	1378	HLA-DRB1*03:01	81% - <100%	Dengue virus 1	ELISPOT	IFNg release	Positive	1	1	1	1	0	0
196111	VEVQDDGTMKIKDEE	NS2b	1421	1435	HLA-DRB1*03:01	81% - <100%	Dengue virus 1	ELISPOT	IFNg release	Positive	1	1	1	0	0	0
866201	ATLFVWYFWQKKKQR	NS2b	1461	1475	HLA-DPB1*01:01	81% - <100%	Dengue virus 1	ELISPOT	IFNg release	Positive	1	1	1	0	0	0
867359	IVSILLSSLLKNDVP	NS2b	1358	1372	HLA-DPB1*04:02	81% - <100%	Dengue virus 1	ELISPOT	IFNg release	Positive	1	1	1	1	0	0
867544	LAGPLIAGGMLIACY	NS2b	1373	1387	HLA-DQA1*01:02/DQB1*06:02	81% - <100%	Dengue virus 1	ELISPOT	IFNg release	Positive	1	1	1	1	0	0
167826	TSGTYVSAIAQAKASQE	NS3	157	173	HLA class II	81% - <100%	Dengue virus 1	ELISPOT	IFNg release	Positive	1	1	1	0	0	0
195143	AIVREAIKRKLRTLI	NS3	1682	1696	HLA-DRB1*11:01	81% - <100%	Dengue virus 1	ELISPOT	IFNg release	Positive	0	0	0	0	0	0
195163	ANCLRKNGKRVIQLS	NS3	1848	1862	HLA-DRB1*13:01	81% - <100%	Dengue virus 1	ELISPOT	IFNg release	Positive	0	0	0	0	0	0
195165	ANFRADRVIDPRRCL	NS3	1891	1905	HLA-DRB1*03:01	81% - <100%	Dengue virus 1	ELISPOT	IFNg release	Positive	1	1	1	0	0	0
195201	CHATFTMRLLSPVRV	NS3	1737	1751	HLA-DRB1*01:01	81% - <100%	Dengue virus 1	ELISPOT	IFNg release	Positive	1	1	1	0	0	0
195242	EGKIVGLYGNGVVTT	NS3	1618	1632	HLA-DRB1*15:01	81% - <100%	Dengue virus 1	ELISPOT	IFNg release	Positive	1	1	1	0	0	0
195296	FHTMWHVTRGAVLMY	NS3	1521	1535	HLA-DRB1*07:01	81% - <100%	Dengue virus 1	ELISPOT	IFNg release	Positive	1	1	1	0	0	0
195377	GIYRILQRGLLGRSQ	NS3	1496	1510	HLA-DRB1*11:01	81% - <100%	Dengue virus 1	ELISPOT	IFNg release	Positive	1	1	1	0	0	0
195379	GKIVGLYGNGVVTTS	NS3	1619	1633	HLA-DRB1*15:01	81% - <100%	Dengue virus 1	ELISPOT	IFNg release	Positive	1	1	1	0	0	0
195549	IYRILQRGLLGRSQV	NS3	1497	1511	HLA-DRB1*15:01	81% - <100%	Dengue virus 1	ELISPOT	IFNg release	Positive	1	1	1	0	0	0
195585	KRKLRTLILAPTRVV	NS3	1689	1703	HLA-DRB1*10:01	81% - <100%	Dengue virus 1	ELISPOT	IFNg release	Positive	0	0	0	0	0	0
195705	LRTLILAPTRVVASE	NS3	1692	1706	HLA-DRB1*08:02	81% - <100%	Dengue virus 1	ELISPOT	IFNg release	Positive	0	0	0	0	0	0
195777	NCLRKNGKRVIQLSR	NS3	1849	1863	HLA-DRB1*11:01	81% - <100%	Dengue virus 1	ELISPOT	IFNg release	Positive	0	0	0	0	0	0
195778	NDIANCLRKNGKRVI	NS3	1845	1859	HLA-DRB1*11:01	81% - <100%	Dengue virus 1	ELISPOT	IFNg release	Positive	0	0	0	0	0	0
195896	RKLRTLILAPTRVVA	NS3	1690	1704	HLA-DRB1*01:01	81% - <100%	Dengue virus 1	ELISPOT	IFNg release	Positive	0	0	0	0	0	0
195914	RTLILAPTRVVASEM	NS3	1693	1707	HLA-DRB1*10:01	81% - <100%	Dengue virus 1	ELISPOT	IFNg release	Positive	0	0	0	0	0	0
196138	VLDDGIYRILQRGLL	NS3	1492	1506	HLA-DRB1*11:01	81% - <100%	Dengue virus 1	ELISPOT	IFNg release	Positive	1	1	1	0	0	0
196197	VTRGAVLMYQGKRLE	NS3	1527	1541	HLA-DRB1*15:01	81% - <100%	Dengue virus 1	ELISPOT	IFNg release	Positive	1	1	1	0	0	0
30397	KEGERKKLRPRWLDA	NS3	585	599	HLA class II	81% - <100%	Dengue virus 1	ICS	IFNg release	Positive	1	1	1	0	0	0
45669	NREGKIVGLYGNGVV	NS3	141	155	HLA-DRB1*15:01	81% - <100%	Dengue virus 1	ICS	IFNg release	Positive	1	1	1	0	0	0
49768	PTRVVASEMAEALKG	NS3	224	238	HLA-DRB1*15:01	81% - <100%	Dengue virus 1	ICS	TNF release	Positive	1	1	1	0	0	0
539022	AAAIFMTATPPGSVE	NS3	1785	1799	HLA-DRB1*04:03	81% - <100%	Dengue virus 1	ELISPOT	IFNg release	Positive	1	1	1	0	0	0
539027	AAIFMTATPPGSVEA	NS3	1786	1800	HLA-DRB1*10:01	81% - <100%	Dengue virus 1	ELISPOT	IFNg release	Positive	1	1	1	0	0	0
539155	DGIYRILQRGLLGRS	NS3	1495	1509	HLA-DRB1*12:02	81% - <100%	Dengue virus 1	ELISPOT	IFNg release	Positive	1	1	1	0	0	0
539171	DLMCHATFTMRLLSP	NS3	1734	1748	HLA-DRB1*13:01	81% - <100%	Dengue virus 1	ELISPOT	IFNg release	Positive	1	1	1	0	0	0
539288	GEAAAIFMTATPPGS	NS3	1783	1797	HLA-DRB1*04:03	81% - <100%	Dengue virus 1	ELISPOT	IFNg release	Positive	1	1	1	0	0	0
539485	KLRTLILAPTRVVAS	NS3	1691	1705	HLA-DRB1*04:03	81% - <100%	Dengue virus 1	ELISPOT	IFNg release	Positive	0	0	0	0	0	0
539543	LDDGIYRILQRGLLG	NS3	1493	1507	HLA-DRB1*12:02	81% - <100%	Dengue virus 1	ELISPOT	IFNg release	Positive	1	1	1	0	0	0
539715	NGKRVIQLSRKTFDT	NS3	1854	1868	HLA-DRB1*12:02	81% - <100%	Dengue virus 1	ELISPOT	IFNg release	Positive	0	0	0	0	0	0
539902	SGTYVSAIAQAKASQ	NS3	1633	1647	HLA-DRB1*10:01	81% - <100%	Dengue virus 1	ELISPOT	IFNg release	Positive	1	1	1	0	0	0
539986	TLILAPTRVVASEMA	NS3	1694	1708	HLA-DRB1*13:01	81% - <100%	Dengue virus 1	ELISPOT	IFNg release	Positive	0	0	0	0	0	0
540040	VGLYGNGVVTTSGTY	NS3	1622	1636	HLA-DRB1*15:06	81% - <100%	Dengue virus 1	ELISPOT	IFNg release	Positive	1	1	1	0	0	0
540074	VREAIKRKLRTLILA	NS3	1684	1698	HLA-DRB1*12:02	81% - <100%	Dengue virus 1	ELISPOT	IFNg release	Positive	0	0	0	0	0	0
739269	IVGLYGNGVVTTSGT	NS3	132	146	HLA-DRB1*01:02	81% - <100%	Dengue virus 1	ELISPOT	IFNg release	Positive	1	1	1	0	0	0
739540	LPAIVREAIKRKLRT	NS3	1680	1694	HLA-DRB1*14:02	81% - <100%	Dengue virus 1	ELISPOT	IFNg release	Positive	1	1	1	0	0	0
866181	ASQEGPLPEIEDEVF	NS3	1645	1659	HLA-DQA1*05:01/DQB1*02:01	81% - <100%	Dengue virus 1	ELISPOT	IFNg release	Positive	1	1	1	0	0	0
866469	EAIKRKLRTLILAPT	NS3	1686	1700	HLA-DPB1*01:01	81% - <100%	Dengue virus 1	ELISPOT	IFNg release	Positive	0	0	0	0	0	0
868113	MIIMDEAHFTDPASI	NS3	1756	1770	HLA-DQA1*01:01/DQB1*05:01	81% - <100%	Dengue virus 1	ELISPOT	IFNg release	Positive	1	1	1	0	0	0
869341	TTSGTYVSAIAQAKA	NS3	1631	1645	HLA-DQA1*01:02/DQB1*06:02	81% - <100%	Dengue virus 1	ELISPOT	IFNg release	Positive	1	1	1	0	0	0
869688	WHVTRGAVLMYQGKR	NS3	1525	1539	HLA-DQA1*01:02/DQB1*06:02	81% - <100%	Dengue virus 1	ELISPOT	IFNg release	Positive	1	1	1	0	0	0
95169	YRILQRGLLGRSQ	NS3	23	35	HLA-DR1	81% - <100%	Dengue virus 1	ELISPOT	IFNg release	Positive	1	1	1	0	0	0
196214	WIAASIILEFFLMVL	NS4a	2198	2212	HLA-DRB1*15:01	81% - <100%	Dengue virus 1	ELISPOT	IFNg release	Positive	1	1	1	0	0	0
196255	YVVIGLLFMILTVAA	NS4a	2230	2244	HLA-DRB1*11:01	81% - <100%	Dengue virus 1	ELISPOT	IFNg release	Positive	1	1	1	0	0	0
539744	NQLAYVVIGLLFMIL	NS4a	2226	2240	HLA-DRB1*15:02	81% - <100%	Dengue virus 1	ELISPOT	IFNg release	Positive	1	1	1	0	0	0
738677	DLILEIGKLPQHLTL	NS4a	2099	2113	HLA-DRB1*11:04	81% - <100%	Dengue virus 1	ELISPOT	IFNg release	Positive	0	0	0	0	0	0
739034	GLGKTSIGLLCVMAS	NS4a	2171	2185	HLA-DRB1*11:04	81% - <100%	Dengue virus 1	ELISPOT	IFNg release	Positive	0	0	0	0	0	0
867109	HWIAASIILEFFLMV	NS4a	2197	2211	HLA-DPB1*04:01	81% - <100%	Dengue virus 1	ELISPOT	IFNg release	Positive	1	1	1	0	0	0
867112	IAASIILEFFLMVLL	NS4a	2199	2213	HLA-DQA1*01:01/DQB1*05:01	81% - <100%	Dengue virus 1	ELISPOT	IFNg release	Positive	1	1	1	0	0	0
867760	LLCVMASSVLLWMAS	NS4a	2179	2193	HLA-DQA1*01:02/DQB1*06:02	81% - <100%	Dengue virus 1	ELISPOT	IFNg release	Positive	0	0	0	0	0	0
539322	GLAFSLMKSLGGGRR	NS4b	2479	2493	HLA-DRB1*10:01	81% - <100%	Dengue virus 1	ELISPOT	IFNg release	Positive	1	1	1	0	0	0
539725	NISLTAIANQAAILM	NS4b	2307	2321	HLA-DRB1*04:03	81% - <100%	Dengue virus 1	ELISPOT	IFNg release	Positive	1	1	1	0	0	0
738560	AWTLYAVATTIITPM	NS4b	2282	2296	HLA-DRB1*04:07	81% - <100%	Dengue virus 1	ELISPOT	IFNg release	Positive	1	1	1	0	0	0
866002	AIANQAAILMGLDKG	NS4b	2312	2326	HLA-DQA1*01:02/DQB1*06:02	81% - <100%	Dengue virus 1	ELISPOT	IFNg release	Positive	1	1	1	0	0	0
869401	VDGIVAIDLDPVVYD	NS4b	2392	2406	HLA-DQA1*01:01/DQB1*05:01	81% - <100%	Dengue virus 1	ELISPOT	IFNg release	Positive	1	1	1	0	0	0
195150	AKWLWGFLSRNKKPR	NS5	2870	2884	HLA-DRB1*15:01	81% - <100%	Dengue virus 1	ELISPOT	IFNg release	Positive	0	0	0	0	0	0
195223	DMGKVRKDIPQWEPS	NS5	3175	3189	HLA-DRB1*03:01	81% - <100%	Dengue virus 1	ELISPOT	IFNg release	Positive	1	1	1	0	0	0
195236	EEFTRKVRSNAAIGA	NS5	2889	2903	HLA-DRB1*08:02	81% - <100%	Dengue virus 1	ELISPOT	IFNg release	Positive	1	1	1	0	0	0
195354	GEKWKRQLNQLSKSE	NS5	2503	2517	HLA-DRB1*04:01	81% - <100%	Dengue virus 1	ELISPOT	IFNg release	Positive	1	1	1	0	0	0
195421	GTGNIVSAVNMTSRM	NS5	2716	2730	HLA-DRB1*08:02	81% - <100%	Dengue virus 1	ELISPOT	IFNg release	Positive	1	1	1	0	0	0
195438	GYILRDISKIPGGNM	NS5	3008	3022	HLA-DRB1*08:02	81% - <100%	Dengue virus 1	ELISPOT	IFNg release	Positive	1	1	1	0	0	0
195464	HWFSRENSLSGVEGE	NS5	2988	3002	HLA-DRB1*04:01	81% - <100%	Dengue virus 1	ELISPOT	IFNg release	Positive	1	1	1	0	0	0
195655	LLATSIFKLTYQNKV	NS5	3055	3069	HLA-DRB1*07:01	81% - <100%	Dengue virus 1	ELISPOT	IFNg release	Positive	1	1	1	0	0	0
195755	MGKVRKDIPQWEPSK	NS5	3176	3190	HLA-DRB1*03:01	81% - <100%	Dengue virus 1	ELISPOT	IFNg release	Positive	1	1	1	0	0	0
195852	QLIMKDGREIVVPCR	NS5	3207	3221	HLA-DRB1*03:01	81% - <100%	Dengue virus 1	ELISPOT	IFNg release	Positive	1	1	1	0	0	0
196211	WATNIQVAINQVRRL	NS5	3351	3365	HLA-DRB1*03:01	81% - <100%	Dengue virus 1	ELISPOT	IFNg release	Positive	1	1	1	0	0	0
196220	WLWGFLSRNKKPRIC	NS5	2872	2886	HLA-DRB1*11:01	81% - <100%	Dengue virus 1	ELISPOT	IFNg release	Positive	1	1	1	0	0	0
196242	YQNKVVRVQRPAKSG	NS5	3065	3079	HLA-DRB1*08:02	81% - <100%	Dengue virus 1	ELISPOT	IFNg release	Positive	0	0	0	0	0	0
539096	ATSIFKLTYQNKVVR	NS5	3057	3071	HLA-DRB1*14:04	81% - <100%	Dengue virus 1	ELISPOT	IFNg release	Positive	1	1	1	0	0	0
539700	MVNGVVRLLTKPWDV	NS5	103	117	HLA-DRB1*12:02	81% - <100%	Dengue virus 1	ELISPOT	IFNg release	Positive	1	1	1	0	0	0
539702	MVTQIAMTDTTPFGQQR	NS5	2829	2845	HLA class II	81% - <100%	Dengue virus 1	ICS	IFNg release	Positive	1	1	1	0	0	0
539905	SHHFHQLIMKDGREI	NS5	3202	3216	HLA-DRB1*13:01	81% - <100%	Dengue virus 1	ELISPOT	IFNg release	Positive	1	1	1	0	0	0
540001	TSIFKLTYQNKVVRV	NS5	3058	3072	HLA-DRB1*09:01	81% - <100%	Dengue virus 1	ELISPOT	IFNg release	Positive	1	1	1	0	0	0
540020	TYGLNTFTNMEVQLI	NS5	3098	3112	HLA-DRB1*04:03	81% - <100%	Dengue virus 1	ELISPOT	IFNg release	Positive	0	0	0	0	0	0
540083	VRVQRPAKSGTVMDV	NS5	96	110	HLA-DRB1*13:01	81% - <100%	Dengue virus 1	ELISPOT	IFNg release	Positive	0	0	0	0	0	0

868296	PEHALLATSIFKLTY	NS5	3051	3065	HLA-DPB1*02:01	81% - <100%	Dengue virus 1	ELISPOT	IFNg release	Positive	1	1	1	0	0	0
868641	RFLEFEALGFMNEDH	NS5	2974	2988	HLA-DPB1*04:01	81% - <100%	Dengue virus 1	ELISPOT	IFNg release	Positive	1	1	1	0	0	0
195540	ISSMLNIMNRRKRSV	C	76	90	HLA-DRB1*14:02	51%-80%	Dengue virus 1	ELISPOT	IFNg release	Positive	0	0	0	0	0	0
195885	RGFKKEISSMLNIMN	C	60	74	HLA-DRB1*04:01	51%-80%	Dengue virus 1	ELISPOT	IFNg release	Positive	0	0	0	0	0	0
539258	FKKEISSMLNIMNRR	C	62	76	HLA-DRB1*10:01	51%-80%	Dengue virus 1	ELISPOT	IFNg release	Positive	0	0	0	0	0	0
539459	KEISSMLNIMNRRKR	C	64	78	HLA-DRB1*14:04	51%-80%	Dengue virus 1	ELISPOT	IFNg release	Positive	0	0	0	0	0	0
539475	KKEISSMLNIMNRRK	C	63	77	HLA-DRB1*14:04	51%-80%	Dengue virus 1	ELISPOT	IFNg release	Positive	0	0	0	0	0	0
539617	LRGFKKEISSMLNIM	C	59	73	HLA-DRB1*13:01	51%-80%	Dengue virus 1	ELISPOT	IFNg release	Positive	0	0	0	0	0	0
540059	VLRGFKKEISSMLNI	C	68	82	HLA-DRB1*04:07	51%-80%	Dengue virus 1	ELISPOT	IFNg release	Positive	0	0	0	0	0	0
195831	PGFTVIALFLAHAIG	PreM	245	259	HLA-DRB1*15:01	51%-80%	Dengue virus 1	ELISPOT	IFNg release	Positive	1	1	1	0	1	1
196085	TVIALFLAHAIGTSI	PreM	248	262	HLA-DRB1*07:01	51%-80%	Dengue virus 1	ELISPOT	IFNg release	Positive	1	1	1	0	1	1
867083	HPGFTVIALFLAHAI	PreM	244	258	HLA-DPB1*02:01	51%-80%	Dengue virus 1	ELISPOT	IFNg release	Positive	1	1	1	0	1	1
539369	GVSWTMKIGIGILLT	E	445	459	HLA-DRB1*15:02	51%-80%	Dengue virus 1	ELISPOT	IFNg release	Positive	1	1	1	0	1	1
539670	MKIGIGILLTWLGLN	E	450	464	HLA-DRB1*15:06	51%-80%	Dengue virus 1	ELISPOT	IFNg release	Positive	1	1	1	0	1	1
867101	HTALTGATEIQTSGT	E	541	555	HLA-DQA1*01:02/DQB1*06:02	51%-80%	Dengue virus 1	ELISPOT	IFNg release	Positive	1	1	1	1	1	1
195162	AMVLSIVSLFPLCLS	NS2a	1296	1310	HLA-DRB1*15:01	51%-80%	Dengue virus 1	ELISPOT	IFNg release	Positive	0	0	0	1	0	0
195595	KTMAMVLSIVSLFPL	NS2a	1293	1307	HLA-DRB1*03:01	51%-80%	Dengue virus 1	ELISPOT	IFNg release	Positive	0	0	0	1	0	0
195745	MAMVLSIVSLFPLCL	NS2a	1295	1309	HLA-DRB1*07:01	51%-80%	Dengue virus 1	ELISPOT	IFNg release	Positive	0	0	0	1	0	0
196216	WKTMAMVLSIVSLFP	NS2a	1292	1306	HLA-DRB1*08:02	51%-80%	Dengue virus 1	ELISPOT	IFNg release	Positive	0	0	0	1	0	0
1392180	GKEIVDLMCHATFTM	NS3	1730	1744		51%-80%	Dengue virus 1	biological activity	activation	Positive	1	1	1	0	0	0
869696	WNTGEEVQVIAVEPG	NS3	1564	1578	HLA-DQA1*03:01/DQB1*03:02	51%-80%	Dengue virus 1	ELISPOT	IFNg release	Positive	0	0	0	0	0	0
539383	HLTLRAQNALDNLVM	NS4a	2110	2124	HLA-DRB3*02:02	51%-80%	Dengue virus 1	ELISPOT	IFNg release	Positive	0	0	0	0	0	0
195186	ATNIQVAINQVRRLI	NS5	3352	3366	HLA-DRB1*03:01	51%-80%	Dengue virus 1	ELISPOT	IFNg release	Positive	1	1	1	0	0	0
195385	GLHKLGYILRDISKI	NS5	3003	3017	HLA-DRB1*08:02	51%-80%	Dengue virus 1	ELISPOT	IFNg release	Positive	1	1	1	0	0	0
195783	NENYLDYMTSMKRFK	NS5	3368	3382	HLA-DRB1*11:01	51%-80%	Dengue virus 1	ELISPOT	IFNg release	Positive	0	0	0	0	0	0
195824	NYLDYMTSMKRFKNE	NS5	3370	3384	HLA-DRB1*14:02	51%-80%	Dengue virus 1	ELISPOT	IFNg release	Positive	0	0	0	0	0	0
539350	GTAKLRWFVERNLVK	NS5	2551	2565	HLA-DRB1*09:01	51%-80%	Dengue virus 1	ELISPOT	IFNg release	Positive	1	1	1	0	0	0
539691	MTSRMLLNRFTMAHR	NS5	17	31	HLA-DRB1*14:06	51%-80%	Dengue virus 1	ELISPOT	IFNg release	Positive	1	1	1	0	0	0
738814	ENYLDYMTSMKRFKN	NS5	3369	3383	HLA-DRB1*14:02	51%-80%	Dengue virus 1	ELISPOT	IFNg release	Positive	0	0	0	0	0	0
740786	YLDYMTSMKRFKNES	NS5	3371	3385	HLA-DRB1*14:02	51%-80%	Dengue virus 1	ELISPOT	IFNg release	Positive	0	0	0	0	0	0
866481	EAVEDERFWDLVHRE	NS5	2916	2930	HLA-DQA1*01:01/DQB1*05:01	51%-80%	Dengue virus 1	ELISPOT	IFNg release	Positive	1	1	1	0	0	0
869091	TAKLRWFVERNLVKP	NS5	2552	2566	HLA-DQA1*01:01/DQB1*05:01	51%-80%	Dengue virus 1	ELISPOT	IFNg release	Positive	1	1	1	0	0	0
1067569	LARWGSFKKNGAIKV	C	66	80	HLA-DRB1*07:01	>0% - 50%	Dengue virus 1	ELISPOT	IFNg release	Positive-Low	1	1	1	0	0	0
195315	FNMLKRARNRVSTVS	C	13	27	HLA-DRB1*08:02	>0% - 50%	Dengue virus 1	ELISPOT	IFNg release	Positive	1	1	1	0	0	0
195498	IKVLRGFKKEISNML	C	78	92	HLA-DRB1*04:01	>0% - 50%	Dengue virus 1	ELISPOT	IFNg release	Positive	1	1	1	0	0	0
195535	ISNMLNIMNRRKRSV	C	88	102	HLA-DRB1*08:02	>0% - 50%	Dengue virus 1	ELISPOT	IFNg release	Positive	1	1	1	0	0	0
195557	KEISNMLNIMNRRKR	C	86	100	HLA-DRB1*13:01	>0% - 50%	Dengue virus 1	ELISPOT	IFNg release	Positive	1	1	1	0	0	0
195805	NMLNIMNRRKRSVTM	C	90	104	HLA-DRB1*11:01	>0% - 50%	Dengue virus 1	ELISPOT	IFNg release	Positive	1	1	1	0	0	0
195883	RGFKKEISNMLNIMN	C	82	96	HLA-DRB1*04:01	>0% - 50%	Dengue virus 1	ELISPOT	IFNg release	Positive	1	1	1	0	0	0
196146	VLRGFKKEISNMLNI	C	80	94	HLA-DRB1*04:01	>0% - 50%	Dengue virus 1	ELISPOT	IFNg release	Positive	1	1	1	0	0	0
196195	VTMLFMLLPTALAFH	C	102	116	HLA-DRB1*04:01	>0% - 50%	Dengue virus 1	ELISPOT	IFNg release	Positive	0	0	0	0	0	0
39096	LRGFKKEISNML	C	81	92	HLA-DPw4	>0% - 50%	Dengue virus 1	ICS	TNF release	Positive	1	1	1	0	0	0
539257	FKKEISNMLNIMNRR	C	84	98	HLA-DRB1*04:03	>0% - 50%	Dengue virus 1	ELISPOT	IFNg release	Positive	1	1	1	0	0	0
539278	GAIKVLRGFKKEISN	C	76	90	HLA-DRB1*12:02	>0% - 50%	Dengue virus 1	ELISPOT	IFNg release	Positive	1	1	1	0	0	0
539616	LRGFKKEISNMLNIM	C	81	95	HLA-DRB1*10:01	>0% - 50%	Dengue virus 1	ELISPOT	IFNg release	Positive	1	1	1	0	0	0
539780	PMKLVMAFIAFLRFL	C	21	35	HLA-DRB1*13:01	>0% - 50%	Dengue virus 1	ELISPOT	IFNg release	Positive	1	1	1	0	0	0
539866	RPSFNMLKRVRNRVS	C	10	24	HLA-DRB1*08:03	>0% - 50%	Dengue virus 1	ELISPOT	IFNg release	Positive	0	0	0	0	0	0
540117	WGSFKKNGAIKVLRG	C	47	61	HLA-DRB1*09:01	>0% - 50%	Dengue virus 1	ELISPOT	IFNg release	Positive	1	1	1	0	0	0
740331	SNMLNIMNRRKRSVT	C	89	103	HLA-DRB1*14:02	>0% - 50%	Dengue virus 1	ELISPOT	IFNg release	Positive	1	1	1	0	0	0
867523	KVLRGFKKEISNMLN	C	79	93	HLA-DRB3*02:02	>0% - 50%	Dengue virus 1	ELISPOT	IFNg release	Positive	1	1	1	0	0	0
95107	KLVMAFIAFLRFL	C	45	57	HLA-DPw4	>0% - 50%	Dengue virus 1	ELISPOT	IFNg release	Positive	1	1	1	0	0	0
195589	KSLLFKTSVGVNMCT	PreM	135	149	HLA-DRB1*07:01	>0% - 50%	Dengue virus 1	ELISPOT	IFNg release	Positive	0	0	0	0	0	0
869062	SVGVNMCTLIAMDLG	PreM	142	156	HLA-DQA1*01:02/DQB1*06:02	>0% - 50%	Dengue virus 1	ELISPOT	IFNg release	Positive	0	0	0	0	0	0
195361	GGVFTSVGKLVHQIF	E	426	440	HLA-DRB1*11:01	>0% - 50%	Dengue virus 1	ELISPOT	IFNg release	Positive	0	0	0	1	0	0
867378	KCRLKMDKLTLKGMS	E	564	578	HLA-DRB3*02:02	>0% - 50%	Dengue virus 1	ELISPOT	IFNg release	Positive	0	0	0	1	1	0
195785	NETWKLTRASFIEVK	NS1	982	996	HLA-DRB1*07:01	>0% - 50%	Dengue virus 1	ELISPOT	IFNg release	Positive	0	0	0	0	0	0
738503	ANGILAQGKKMIRPQ	NS1	868	882	HLA-DRB1*14:06	>0% - 50%	Dengue virus 1	ELISPOT	IFNg release	Positive	0	0	0	0	0	0
740562	TVVVGDANGILAQGK	NS1	862	876	HLA-DRB1*01:02	>0% - 50%	Dengue virus 1	ELISPOT	IFNg release	Positive	0	0	0	0	0	0
866693	FGIFTTNIWLKLRDS	NS1	196	210	HLA-DPB1*04:01	>0% - 50%	Dengue virus 1	ELISPOT	IFNg release	Positive	1	1	1	0	0	0
867405	KIIGADIQNTTFIID	NS1	897	911	HLA-DPB1*04:02	>0% - 50%	Dengue virus 1	ELISPOT	IFNg release	Positive	0	0	0	0	0	0
869721	YGFGIFTTNIWLKLR	NS1	194	208	HLA-DPB1*02:01	>0% - 50%	Dengue virus 1	ELISPOT	IFNg release	Positive	1	1	1	0	0	0
167816	SLVASVELPNSLEELGDGLAMGIMI	NS2a	109	133	HLA class II	>0% - 50%	Dengue virus 1	ELISPOT	IFNg release	Positive	0	0	0	0	0	0
539086	AMGIMILKLLTDFQS	NS2a	1255	1269	HLA-DRB1*04:03	>0% - 50%	Dengue virus 1	ELISPOT	IFNg release	Positive	0	0	0	1	0	0
539272	FSLHYAWKTMAMVLS	NS2a	1286	1300	HLA-DRB1*09:01	>0% - 50%	Dengue virus 1	ELISPOT	IFNg release	Positive	0	0	0	1	0	0
539517	KTTFSLHYAWKTMAM	NS2a	1283	1297	HLA-DRB1*09:01	>0% - 50%	Dengue virus 1	ELISPOT	IFNg release	Positive	1	1	1	1	0	0
539913	SLHYAWKTMAMVLSI	NS2a	1287	1301	HLA-DRB1*09:01	>0% - 50%	Dengue virus 1	ELISPOT	IFNg release	Positive	0	0	0	1	0	0
539970	TFSLHYAWKTMAMVL	NS2a	1285	1299	HLA-DRB1*14:06	>0% - 50%	Dengue virus 1	ELISPOT	IFNg release	Positive	0	0	0	1	0	0
738679	DLIRLCIMVGANASD	NS2a	1179	1193	HLA-DRB1*11:04	>0% - 50%	Dengue virus 1	ELISPOT	IFNg release	Positive	1	1	1	0	0	0
739508	LLCISIMIEEVMRSR	NS2a	1139	1153	HLA-DRB1*11:04	>0% - 50%	Dengue virus 1	ELISPOT	IFNg release	Positive	1	1	1	0	0	0
867232	ILKLLTDFQSHQLWA	NS2a	1260	1274	HLA-DPB1*01:01	>0% - 50%	Dengue virus 1	ELISPOT	IFNg release	Positive	0	0	0	1	0	0
195622	LDDGIYRIMQRGLLG	NS3	1493	1507	HLA-DRB1*11:01	>0% - 50%	Dengue virus 1	ELISPOT	IFNg release	Positive	0	0	0	0	0	0
539156	DGIYRIMQRGLLGRS	NS3	1495	1509	HLA-DRB1*12:02	>0% - 50%	Dengue virus 1	ELISPOT	IFNg release	Positive	0	0	0	0	0	0
539309	GIYRIMQRGLLGRSQ	NS3	1496	1510	HLA-DRB1*14:04	>0% - 50%	Dengue virus 1	ELISPOT	IFNg release	Positive	0	0	0	0	0	0
866721	FKTPEGEVGAIALDF	NS3	1591	1605	HLA-DQA1*03:01/DQB1*03:02	>0% - 50%	Dengue virus 1	ELISPOT	IFNg release	Positive	1	1	1	0	0	0
867025	GVFQENVFHTMWHVT	NS3	1514	1528	HLA-DPB1*04:01	>0% - 50%	Dengue virus 1	ELISPOT	IFNg release	Positive	0	0	0	0	0	0
195485	IGLLCVMASSALLWM	NS4a	2177	2191	HLA-DRB1*07:01	>0% - 50%	Dengue virus 1	ELISPOT	IFNg release	Positive	0	0	0	0	0	0
867567	LCVMASSALLWMANV	NS4a	2180	2194	HLA-DQA1*01:02/DQB1*06:02	>0% - 50%	Dengue virus 1	ELISPOT	IFNg release	Positive	0	0	0	0	0	0
195441	HATMLDVDLRPASAW	NS4b	2269	2283	HLA-DRB1*03:01	>0% - 50%	Dengue virus 1	ELISPOT	IFNg release	Positive	0	0	0	0	0	0
195455	HHHATMLDVDLRPAS	NS4b	2267	2281	HLA-DRB1*03:01	>0% - 50%	Dengue virus 1	ELISPOT	IFNg release	Positive	0	0	0	0	0	0
195561	KFWNTTIAVSMANIF	NS4b	2456	2470	HLA-DRB1*07:01	>0% - 50%	Dengue virus 1	ELISPOT	IFNg release	Positive	1	1	1	0	0	0
738686	DLRPASAWTLYAVAT	NS4b	2276	2290	HLA-DRB1*01:02	>0% - 50%	Dengue virus 1	ELISPOT	IFNg release	Positive	0	0	0	0	0	0
740732	WTLYAVATTVITPMM	NS4b	2283	2297	HLA-DRB1*04:07	>0% - 50%	Dengue virus 1	ELISPOT	IFNg release	Positive	0	0	0	0	0	0
195147	AKNGTVMDVISRRDQ	NS5	3076	3090	HLA-DRB1*03:01	>0% - 50%	Dengue virus 1	ELISPOT	IFNg release	Positive	1	1	1	0	0	0
195582	KPIDDRFATALTALN	NS5	3160	3174	HLA-DRB1*04:01	>0% - 50%	Dengue virus 1	ELISPOT	IFNg release	Positive	1	1	1	0	0	0
195786	NGTVMDVISRRDQRG	NS5	3078	3092	HLA-DRB1*03:01	>0% - 50%	Dengue virus 1	ELISPOT	IFNg release	Positive	1	1	1	0	0	0
195903	RPAKNGTVMDVISRR	NS5	3074	3088	HLA-DRB1*03:01	>0% - 50%	Dengue virus 1	ELISPOT	IFNg release	Positive	1	1	1	0	0	0
195923	RWLWGFLSRNKKPRI	NS5	2871	2885	HLA-DRB1*15:01	>0% - 50%	Dengue virus 1	ELISPOT	IFNg release	Positive	1	1	1	0	0	0
196191	VTARWLWGFLSRNKK	NS5	2868	2882	HLA-DRB1*15:01	>0% - 50%	Dengue virus 1	ELISPOT	IFNg release	Positive	1	1	1	0	0	0
196241	YQNKVVRVQRPAKNG	NS5	3065	3079	HLA-DRB1*08:02	>0% - 50%	Dengue virus 1	ELISPOT	IFNg release	Positive	1	1	1	0	0	0
539396	IDDRFATALTALNDM	NS5	3162	3176	HLA-DPB1*01:01	>0% - 50%	Dengue virus 1	ELISPOT	IFNg release	Positive	1	1	1	0	0	0
539435	ISGDDCVVKPIDDRFAT	NS5	3152	3168	HLA class II	>0% - 50%	Dengue virus 1	ICS	IFNg release	Positive	1	1	1	0	0	0
539862	RMLLNRFTMAHRKPT	NS5	20	34	HLA-DRB1*14:02	>0% - 50%	Dengue virus 1	ELISPOT	IFNg release	Positive	1	1	1	0	0	0
540018	TYGLNTFTNMEAQLI	NS5	3098	3112	HLA-DRB1*15:06	>0% - 50%	Dengue virus 1	ELISPOT	IFNg release	Positive	1	1	1	0	0	0
540100	VVKPIDDRFATALTALN	NS5	3157	3173	HLA class II	>0% - 50%	Dengue virus 1	ICS	IFNg release	Positive	1	1	1	0	0	0
740024	QNKVVRVQRPAKNGT	NS5	3066	3080	HLA-DRB1*14:06	>0% - 50%	Dengue virus 1	ELISPOT	IFNg release	Positive	1	1	1	0	0	0
740668	VQLIRQMESEGIFLP	NS5	3109	3123	HLA-DRB1*11:04	>0% - 50%	Dengue virus 1	ELISPOT	IFNg release	Positive	0	0	0	0	0	0
868240	NKVVRVQRPAKNGTV	NS5	3067	3081	HLA-DRB4*01:01	>0% - 50%	Dengue virus 1	ELISPOT	IFNg release	Positive	1	1	1	0	0	0

CD4 DENV-2 Epitopes

Epitope_ID	AA_Seq	Protein_Name	Start	End	Allele	Step	Serotype	Method/Technique	Assay Group	Qualitative Measure	DENV-2 constructs of vaccines (100% identity=1,green ; else 0, red)					
											TV003	TDEN	DPIV	Dengvaxia	DENVax	TVDV
150404	LRLFMALVAFLRFLT	C	44	58	HLA-DRB1*11:01	0%	Dengue virus 2	ELISPOT	IFNg release	Positive	0	0	0	0	0	0
195121	AGMIIMLIPTVVAFH	C	102	116	HLA-DRB1*08:02	0%	Dengue virus 2	ELISPOT	IFNg release	Positive	0	0	0	0	0	0
195369	GIIIMIIPTVMAFHL	C	103	117	HLA-DRB1*08:02	0%	Dengue virus 2	ELISPOT	IFNg release	Positive	0	0	0	0	0	0
195413	GPLRLFMALVAFLRF	C	42	56	HLA-DRB1*04:01	0%	Dengue virus 2	ELISPOT	IFNg release	Positive	0	0	0	0	0	0
195432	GVIIMMIPTVMAFHL	C	103	117	HLA-DRB1*08:02	0%	Dengue virus 2	ELISPOT	IFNg release	Positive	0	0	0	0	0	0
195490	IGRMLNILSRRRRTA	C	88	102	HLA-DRB1*11:01	0%	Dengue virus 2	ELISPOT	IFNg release	Positive	0	0	0	0	0	0
195654	LKLYMALVAFLRFLT	C	44	58	HLA-DRB1*15:01	0%	Dengue virus 2	ELISPOT	IFNg release	Positive	0	0	0	0	0	0
195910	RTAGVIIMMIPTVVA	C	100	114	HLA-DRB1*08:02	0%	Dengue virus 2	ELISPOT	IFNg release	Positive	0	0	0	0	0	0
195911	RTASVIVMLIPTVMA	C	100	114	HLA-DRB1*08:02	0%	Dengue virus 2	ELISPOT	IFNg release	Positive	0	0	0	0	0	0
196024	TAGVIVMLIPTVMAF	C	101	115	HLA-DRB1*08:02	0%	Dengue virus 2	ELISPOT	IFNg release	Positive	0	0	0	0	0	0
196084	TVGVIIMLIPTAMAF	C	101	115	HLA-DRB1*08:02	0%	Dengue virus 2	ELISPOT	IFNg release	Positive	0	0	0	0	0	0
53867	RGFRKEIGRMLNILNRRRS	C	17	35	HLA class II	0%	Dengue virus 2	ELISPOT	IFNg release	Positive	0	0	0	0	0	0
539046	AFLRFLTIPPTVGIL	C	52	66	HLA-DRB1*10:01	0%	Dengue virus 2	ELISPOT	IFNg release	Positive	0	0	0	0	0	0
539051	AFVAFLRFLTIPPTA	C	9	23	HLA-DRB1*15:02	0%	Dengue virus 2	ELISPOT	IFNg release	Positive	0	0	0	0	0	0
539213	EIGRMLNILNKRRRT	C	87	101	HLA-DRB1*13:01	0%	Dengue virus 2	ELISPOT	IFNg release	Positive	0	0	0	0	0	0
539343	GRMLNIMNRRRRTAG	C	89	103	HLA-DRB1*13:01	0%	Dengue virus 2	ELISPOT	IFNg release	Positive	0	0	0	0	0	0
539549	LFTALVAFLRFLTIP	C	46	60	HLA-DRB1*15:06	0%	Dengue virus 2	ELISPOT	IFNg release	Positive	0	0	0	0	0	0
62853	TAGILKRWGTIKKSKANVL	C	62	81	HLA class II	0%	Dengue virus 2	ELISPOT	IFNg release	Positive	0	0	0	0	0	0
739176	IGRMLNILNKRRRTA	C	88	102	HLA-DRB1*14:02	0%	Dengue virus 2	ELISPOT	IFNg release	Positive	0	0	0	0	0	0
739177	IGRMLNILNRRRRSA	C	88	102	HLA-DRB1*14:02	0%	Dengue virus 2	ELISPOT	IFNg release	Positive	0	1	0	0	1	0
739179	IGRMLNILNRRRRSV	C	88	102	HLA-DRB1*14:02	0%	Dengue virus 2	ELISPOT	IFNg release	Positive	0	0	0	0	0	0
739180	IGRMLNILNRRRRTT	C	88	102	HLA-DRB1*14:02	0%	Dengue virus 2	ELISPOT	IFNg release	Positive	0	0	0	0	0	0
739182	IGRMLNIMNRRRRTA	C	88	102	HLA-DRB1*14:02	0%	Dengue virus 2	ELISPOT	IFNg release	Positive	0	0	0	0	0	0
739183	IGRMMNILNRRRRTA	C	88	102	HLA-DRB1*14:02	0%	Dengue virus 2	ELISPOT	IFNg release	Positive	0	0	0	0	0	0
740399	STPFNMLKRERNRVS	C	10	24	HLA-DRB1*14:02	0%	Dengue virus 2	ELISPOT	IFNg release	Positive	0	0	0	0	0	0
740579	VAFLRFLTISPTAGI	C	51	65	HLA-DRB1*04:07	0%	Dengue virus 2	ELISPOT	IFNg release	Positive	0	0	0	0	0	0
866992	GPLKLYMALVAFLRF	C	42	56	HLA-DRB5*01:01	0%	Dengue virus 2	ELISPOT	IFNg release	Positive	0	0	0	0	0	0
867734	LKGFRKEIGRMLNIL	C	81	95	HLA-DRB3*02:02	0%	Dengue virus 2	ELISPOT	IFNg release	Positive	0	0	0	0	0	0
867910	LRGFRKEIGRMLNIM	C	81	95	HLA-DRB3*02:02	0%	Dengue virus 2	ELISPOT	IFNg release	Positive	0	0	0	0	0	0
867911	LRGFRREIGRMLNIL	C	81	95	HLA-DRB3*02:02	0%	Dengue virus 2	ELISPOT	IFNg release	Positive	0	0	0	0	0	0
868674	RLFMALVAFLRFLTI	C	45	59	HLA-DPB1*04:02	0%	Dengue virus 2	ELISPOT	IFNg release	Positive	0	0	0	0	0	0
150280	HPGFTLMAAILAYTI	PreM	244	258	HLA-DRB1*01:01	0%	Dengue virus 2	ELISPOT	IFNg release	Positive	0	0	0	0	0	0
180708	QRIETWILRHPGFTM	PreM	121	135	HLA class II	0%	Dengue virus 2	ELISPOT	IFNg release	Positive-Low	0	0	0	0	1	0
195318	FQKILIFILLTAVAP	PreM	263	277	HLA-DRB1*07:01	0%	Dengue virus 2	ELISPOT	IFNg release	Positive	0	0	0	0	0	0
195320	FQRTLIFILLTAVAP	PreM	263	277	HLA-DRB1*07:01	0%	Dengue virus 2	ELISPOT	IFNg release	Positive	0	0	0	0	0	0
195448	HFQRALIFILLTAVA	PreM	262	276	HLA-DRB1*07:01	0%	Dengue virus 2	ELISPOT	IFNg release	Positive	1	1	1	1	0	1
195504	ILRHPGFTLMAAILA	PreM	241	255	HLA-DRB1*15:01	0%	Dengue virus 2	ELISPOT	IFNg release	Positive	0	0	0	0	0	0
196045	THFQRALIFILLTAV	PreM	261	275	HLA-DRB1*07:01	0%	Dengue virus 2	ELISPOT	IFNg release	Positive	1	1	1	1	1	1
196232	YFQRVLIFILLTAVT	PreM	262	276	HLA-DRB1*07:01	0%	Dengue virus 2	ELISPOT	IFNg release	Positive	0	0	0	0	0	0
739425	LAYTIGTTHFQRALI	PreM	254	268	HLA-DRB1*04:07	0%	Dengue virus 2	ELISPOT	IFNg release	Positive	1	1	1	1	1	1
866485	EDGVNMCTLMAIDLG	PreM	142	156	HLA-DQA1*01:02/DQB1*06:02	0%	Dengue virus 2	ELISPOT	IFNg release	Positive	0	0	0	0	0	0
868650	RHPGFTLMAAILAYT	PreM	243	257	HLA-DQA1*01:02/DQB1*06:02	0%	Dengue virus 2	ELISPOT	IFNg release	Positive	0	0	0	0	0	0
195793	NLEYTIMITPHSGEE	E	134	148	HLA-DRB1*04:01	0%	Dengue virus 2	ELISPOT	IFNg release	Positive	0	0	0	0	0	0
195952	SGVSWTMKILIGVII	E	449	463	HLA-DRB1*15:01	0%	Dengue virus 2	ELISPOT	IFNg release	Positive	0	1	0	1	1	0
196022	SWTMKILIGVIITWI	E	452	466	HLA-DRB1*08:02	0%	Dengue virus 2	ELISPOT	IFNg release	Positive	0	1	0	1	1	0
539427	IQKKTLVTFKNPHAK	E	512	526	HLA-DRB1*04:03	0%	Dengue virus 2	ELISPOT	IFNg release	Positive	0	0	0	0	0	0
539462	KEMLVTFKNPHAKRQ	E	514	528	HLA-DRB1*04:03	0%	Dengue virus 2	ELISPOT	IFNg release	Positive	0	0	0	0	0	0
539479	KKTLVTFKNPHAKKQ	E	514	528	HLA-DRB1*14:04	0%	Dengue virus 2	ELISPOT	IFNg release	Positive	0	0	0	0	0	0
539629	LSVTLVLVGFVTLYL	E	475	489	HLA-DRB1*15:06	0%	Dengue virus 2	ELISPOT	IFNg release	Positive	0	0	0	0	0	0
738866	EYTIVITPHSGEEHA	E	136	150	HLA-DRB1*04:07	0%	Dengue virus 2	ELISPOT	IFNg release	Positive	1	0	1	0	1	1
867375	KAWLVHRQWFLDLPF	E	204	218	HLA-DQA1*01:01/DQB1*05:01	0%	Dengue virus 2	ELISPOT	IFNg release	Positive	0	0	0	0	0	0
868347	PPFGDSYVIIGVEPG	E	371	385	HLA-DQA1*03:01/DQB1*03:02	0%	Dengue virus 2	ELISPOT	IFNg release	Positive	0	0	0	0	0	0
195954	SHNQTFLIDGPETAE	NS1	903	917	HLA-DRB1*04:01	0%	Dengue virus 2	ELISPOT	IFNg release	Positive	0	1	1	0	1	0
539708	NDTWKMEKASFIEVK	NS1	982	996	HLA-DRB1*09:01	0%	Dengue virus 2	ELISPOT	IFNg release	Positive	0	0	0	0	0	0
739191	IKGIMHAGKRSLRPQ	NS1	868	882	HLA-DRB1*14:02	0%	Dengue virus 2	ELISPOT	IFNg release	Positive	0	0	0	0	0	0
866699	FGVFTTNIWLKLREK	NS1	935	949	HLA-DPB1*04:01	0%	Dengue virus 2	ELISPOT	IFNg release	Positive	0	0	1	0	0	0
866829	GFGFFTTNIWLKLRE	NS1	934	948	HLA-DPB1*01:01	0%	Dengue virus 2	ELISPOT	IFNg release	Positive	0	0	0	0	0	0
866831	GFGVFTTNIWLKLRE	NS1	934	948	HLA-DPB1*04:01	0%	Dengue virus 2	ELISPOT	IFNg release	Positive	0	0	1	0	0	0
869722	YGFGVFTTNIWLKLR	NS1	933	947	HLA-DPB1*04:01	0%	Dengue virus 2	ELISPOT	IFNg release	Positive	0	0	1	0	0	0
150245	GMGVTYLALLAAYKV	NS2a	1196	1210	HLA-DRB1*01:01	0%	Dengue virus 2	ELISPOT	IFNg release	Positive	0	0	0	0	0	0
167819	SSQQKTDWIPLALTIKGLNP	NS2a	184	203	HLA-DP	0%	Dengue virus 2	ELISPOT	IFNg release	Positive	0	1	0	0	1	0
195269	ERYQLAVTITAILCV	NS2a	1268	1282	HLA-DRB1*07:01	0%	Dengue virus 2	ELISPOT	IFNg release	Positive	0	0	0	0	0	0
195523	IPLVLIIKGLNPTAI	NS2a	1319	1333	HLA-DRB1*08:02	0%	Dengue virus 2	ELISPOT	IFNg release	Positive	0	0	0	0	0	0
195712	LSLTFIRSTTPLIMA	NS2a	1275	1289	HLA-DRB1*07:01	0%	Dengue virus 2	ELISPOT	IFNg release	Positive	0	0	0	0	0	0
195763	MMVLKIVRKMERYQL	NS2a	1258	1272	HLA-DRB1*03:01	0%	Dengue virus 2	ELISPOT	IFNg release	Positive	0	0	0	0	0	0
196217	WKVSCTILAAVSVSP	NS2a	1292	1306	HLA-DRB1*04:01	0%	Dengue virus 2	ELISPOT	IFNg release	Positive	0	0	0	0	0	0
539077	ALGIMVLKMVRSMEK	NS2a	1255	1269	HLA-DRB1*12:02	0%	Dengue virus 2	ELISPOT	IFNg release	Positive	0	0	0	0	0	0
539082	ALTIKGLNPTAILLT	NS2a	1322	1336	HLA-DRB1*09:01	0%	Dengue virus 2	ELISPOT	IFNg release	Positive	0	0	0	0	0	0
539110	AWALGMMVLKIVRNM	NS2a	1253	1267	HLA-DRB1*13:01	0%	Dengue virus 2	ELISPOT	IFNg release	Positive	0	0	0	0	0	0
539392	IALGIMVLKMVRSME	NS2a	1254	1268	HLA-DRB1*13:01	0%	Dengue virus 2	ELISPOT	IFNg release	Positive	0	0	0	0	0	0
539556	LGIMILKLVRKMEKY	NS2a	1256	1270	HLA-DRB1*12:02	0%	Dengue virus 2	ELISPOT	IFNg release	Positive	0	0	0	0	0	0
539557	LGIMVLKIVRSMEKY	NS2a	1256	1270	HLA-DRB1*12:02	0%	Dengue virus 2	ELISPOT	IFNg release	Positive	0	0	0	0	0	0
539706	NAWKVSCTILAAVSV	NS2a	1290	1304	HLA-DRB1*09:01	0%	Dengue virus 2	ELISPOT	IFNg release	Positive	0	0	0	0	0	0
539893	SCTILAAVSVSPLLL	NS2a	1295	1309	HLA-DRB1*10:01	0%	Dengue virus 2	ELISPOT	IFNg release	Positive	0	0	0	0	0	0
195913	RTGLLVISGVFPVSI	NS2b	1445	1459	HLA-DRB1*08:02	0%	Dengue virus 2	ELISPOT	IFNg release	Positive	0	0	0	0	0	0
539574	LIRTGLLVISGVFPV	NS2b	1443	1457	HLA-DRB1*13:01	0%	Dengue virus 2	ELISPOT	IFNg release	Positive	0	0	0	0	0	0
24916	HTGREIVDLMCHATE	NS3	251	265	HLA-DPw2	0%	Dengue virus 2	ICS	IFNg release	Positive	0	0	0	0	0	0
539283	GAYRIKQRGILGYSQ	NS3	1496	1510	HLA-DRB1*08:03	0%	Dengue virus 2	ELISPOT	IFNg release	Positive	0	0	0	0	0	0
54390	RKLTIMDLHPGAGKT	NS3	618	632	HLA class II	0%	Dengue virus 2	ICS	IFNg release	Positive	0	0	1	0	0	0
868183	MWHVTRGAVLMHRGK	NS3	1524	1538	HLA-DRB3*02:02	0%	Dengue virus 2	ELISPOT	IFNg release	Positive	0	0	0	0	0	0
150192	ETLLLLTLLAAVTGG	NS4a	2146	2160	HLA-DRB1*01:01	0%	Dengue virus 2	ELISPOT	IFNg release	Positive	0	0	0	0	0	0
195810	NQLTYVVIAILTLVA	NS4a	2225	2239	HLA-DRB1*07:01	0%	Dengue virus 2	ELISPOT	IFNg release	Positive	0	0	0	0	0	0
195118	AGLLFSIMRNTTSAR	NS4b	2476	2490	HLA-DRB1*04:01	0%	Dengue virus 2	ELISPOT	IFNg release	Positive	0	0	0	0	0	0
195270	ESESNILDIDLRPAS	NS4b	2265	2279	HLA-DRB1*03:01	0%	Dengue virus 2	ELISPOT	IFNg release	Positive	0	0	0	0	0	0
195387	GLLFFIMKNTTNTRR	NS4b	2477	2491	HLA-DRB1*04:01	0%	Dengue virus 2	ELISPOT	IFNg release	Positive	0	0	0	0	0	0
195393	GLLFSIMKNTANTRR	NS4b	2463	2477	HLA-DRB1*04:01	0%	Dengue virus 2	ELISPOT	IFNg release	Positive	0	0	0	0	0	0
195394	GLLFSIMKNTTNARR	NS4b	2477	2491	HLA-DRB1*04:01	0%	Dengue virus 2	ELISPOT	IFNg release	Positive	0	0	0	0	0	0
195395	GLLFSIMKNTTNSRR	NS4b	2477	2491	HLA-DRB1*04:01	0%	Dengue virus 2	ELISPOT	IFNg release	Positive	0	0	0	0	0	0
195396	GLLFSIMKNTTSARR	NS4b	2477	2491	HLA-DRB1*04:01	0%	Dengue virus 2	ELISPOT	IFNg release	Positive	0	0	0	0	0	0
195398	GLLFSVMKNTTNTRR	NS4b	2477	2491	HLA-DRB1*04:01	0%	Dengue virus 2	ELISPOT	IFNg release	Positive	0	0	0	0	0	0
195659	LLFSIMRNTTTARRG	NS4b	2478	2492	HLA-DRB1*04:01	0%	Dengue virus 2	ELISPOT	IFNg release	Positive	0	0	0	0	0	0
539755	PASAWTLYAVATTFV	NS4b	2277	2291	HLA-DRB1*09:01	0%	Dengue virus 2	ELISPOT	IFNg release	Positive	0	1	1	0	1	0
867709	LILCVTQVLMMRTTW	NS4b	2417	2431	HLA-DPB1*04:02	0%	Dengue virus 2	ELISPOT	IFNg release	Positive	0	0	0	0	0	0
869090	TAIANQATVLMGLGR	NS4b	2309	2323	HLA-DQA1*01:02/DQB1*06:02	0%	Dengue virus 2	ELISPOT	IFNg release	Positive	0	0	0	0	0	0
167817	SRLNALGKSEFQI	NS5	15	27	HLA class II	0%	Dengue virus 2	ELISPOT	IFNg release	Positive	0	1	1	0	1	0
195111	ACLGKSYAQMWTLMY	NS5	3243	3257	HLA-DRB1*04:01	0%	Dengue virus 2	ELISPOT	IFNg release	Positive	0	0	0	0	0	0
195241	EGEGVFKSIQHLTVT	NS5	3101	3115	HLA-DRB1*07:01	0%	Dengue virus 2	ELISPOT	IFNg release	Positive	0	0	1	0	0	0
195250	ELIMKDGRKLVVPCR	NS5	3206	3220	HLA-DRB1*03:01	0%	Dengue virus 2	ELISPOT	IFNg release	Positive	0	0	0	0	0	0
195384	GLFKSIQHLTSQEEI	NS5	3118	3132	HLA-DRB1*11:01	0%	Dengue virus 2	ELISPOT	IFNg release	Positive	0	0	0	0	0	0
195445	HFHELIMKDGRILVV	NS5	3203	3217	HLA-DRB1*03:01	0%	Dengue virus 2	ELISPOT	IFNg release	Positive	0	0	0	0	0	0
195446	HFHELVMKDGRVLVV	NS5	3203	3217	HLA-DRB1*13:01	0%	Dengue virus 2	ELISPOT	IFNg release	Positive	0	0	0	0	0	0
195460	HRLGYILRDVSRKEG	NS5	3004	3018	HLA-DRB1*08:02	0%	Dengue virus 2	ELISPOT	IFNg release	Positive	0	0	0	0	0	0
195513	IMKDGRKLVVPCRNQ	NS5	3207	3221	HLA-DRB1*03:01	0%	Dengue virus 2	ELISPOT	IFNg release	Positive	0	0	0	0	0	0
196040	TGNIVSSVNMISRML	NS5	2716	2730	HLA-DRB1*04:01	0%	Dengue virus 2	ELISPOT	IFNg release	Positive	0	0	0	0	0	0
196225	WTLMYFHRRDLRLAA	NS5	3253	3267	HLA-DRB1*03:01	0%	Dengue virus 2	ELISPOT	IFNg release	Positive	0	0	0	0	0	0
32939	KPWDIIPMV	NS5	2821	2829	HLA class II	0%	Dengue virus 2	ELISA	IL-2 release	Positive	0	0	0	0	0	0
32941	KPWDVLPMV	NS5	2821	2829	HLA class II	0%	Dengue virus 2	ELISA	IL-2 release	Positive	0	0	0	0	0	0
32942	KPWDVLPTV	NS5	2821	2829	HLA class II	0%	Dengue virus 2	ELISA	IL-2 release	Positive	0	0	0	0	0	0
539161	DHWFSRGNSLSGVEG	NS5	2986	3000	HLA-DRB1*09:01	0%	Dengue virus 2	ELISPOT	IFNg release	Positive	0	0	0	0	0	0
539348	GSAKLRWFVERNMVT	NS5	2549	2563	HLA-DRB1*09:01	0%	Dengue virus 2	ELISPOT	IFNg release	Positive	0	1	1	0	1	0
539560	LGKNEFQIYKKSGIQ	NS5	2511	2525	HLA-DRB1*15:02	0%	Dengue virus 2	ELISPOT	IFNg release	Positive	0	0	0	0	0	0
869083	SYAQMWTLMYFHRRD	NS5	3248	3262	HLA-DPB1*04:01	0%	Dengue virus 2	ELISPOT	IFNg release	Positive	0	0	0	0	0	0
869148	TFVESWEDIPYLGKR	NS5	3319	3333	HLA-DQA1*01:01/DQB1*05:01	0%	Dengue virus 2	ELISPOT	IFNg release	Positive	0	0	0	0	0	0
869292	TPVESWEEVPYLGKR	NS5	3319	3333	HLA-DQA1*01:01/DQB1*05:01	0%	Dengue virus 2	ELISPOT	IFNg release	Positive	0	0	0	0	0	0
195326	FRKEIGRMLNILNRR	C	84	98	HLA-DRB1*04:01	100%	Dengue virus 2	ELISPOT	IFNg release	Positive	0	1	1	0	1	0
195556	KEIGRMLNILNRRRR	C	86	100	HLA-DRB1*08:02	100%	Dengue virus 2	ELISPOT	IFNg release	Positive	0	1	1	0	1	0
195886	RGFRKEIGRMLNILN	C	82	96	HLA-DRB1*04:01	100%	Dengue virus 2	ELISPOT	IFNg release	Positive	0	1	1	0	1	0
39098	LRGFRKEIGRML	C	81	92	HLA-DPw4	100%	Dengue virus 2	ICS	IFNg release	Positive	0	1	1	0	1	0
539214	EIGRMLNILNRRRRT	C	87	101	HLA-DRB1*12:02	100%	Dengue virus 2	ELISPOT	IFNg release	Positive	0	0	1	0	0	0
539618	LRGFRKEIGRMLNIL	C	81	95	HLA-DRB1*09:01	100%	Dengue virus 2	ELISPOT	IFNg release	Positive	0	1	1	0	1	0
540060	VLRGFRKEIGRMLNI	C	80	94	HLA-DRB1*09:01	100%	Dengue virus 2	ELISPOT	IFNg release	Positive	0	1	1	0	1	0
180590	LDFELIKTEAKQPAT	E	41	55	HLA class II	100%	Dengue virus 2	ELISPOT	IFNg release	Positive	1	1	1	1	1	1
195351	GATEIQMSSGNLLFT	E	266	280	HLA-DRB1*03:01	100%	Dengue virus 2	ELISPOT	IFNg release	Positive	1	1	1	1	1	1
195366	GHLKCRLRMDKLQLK	E	281	295	HLA-DRB1*03:01	100%	Dengue virus 2	ELISPOT	IFNg release	Positive	1	1	1	1	1	1
195407	GMNSRSTSLSVSLVL	E	467	481	HLA-DRB1*07:01	100%	Dengue virus 2	ELISPOT	IFNg release	Positive	1	1	1	1	0	0
539648	LVTFKNPHAKKQDVV	E	237	251	HLA-DRB1*15:02	100%	Dengue virus 2	ELISPOT	IFNg release	Positive	1	1	1	1	1	1

539689	MRGAKRMAILGDTAW	E	406	420	HLA-DRB1*15:06	100%	Dengue virus 2	ELISPOT	IFNg release	Positive	1	1	1	1	1	0
741744	MRCIGISNRDFVEGVSGGSW	E	281	300	HLA class II	100%	Dengue virus 2	ELISPOT	IFNg release	Positive	1	1	1	1	0	1
866193	ATEIQMSSGNLLFTG	E	257	271	HLA-DRB3*02:02	100%	Dengue virus 2	ELISPOT	IFNg release	Positive	1	1	1	1	1	1
150273	GVTYLALLAAFKVRP	NS2a	1198	1212	HLA-DRB1*01:01	100%	Dengue virus 2	ELISPOT	IFNg release	Positive	0	1	1	0	1	0
540094	VTYLALLAAFKVRPT	NS2a	1199	1213	HLA-DRB1*12:02	100%	Dengue virus 2	ELISPOT	IFNg release	Positive	0	1	1	0	1	0
150433	MAVGMVSILASSLLK	NS2b	1354	1368	HLA-DRB1*01:01	100%	Dengue virus 2	ELISPOT	IFNg release	Positive	0	1	1	0	1	0
195512	IMAVGMVSILASSLL	NS2b	8	22	HLA-DRB1*07:01	100%	Dengue virus 2	ELISPOT	IFNg release	Positive	0	1	1	0	1	0
195992	SSLLKNDIPMTGPLV	NS2b	19	33	HLA-DRB1*03:01	100%	Dengue virus 2	ELISPOT	IFNg release	Positive	0	1	1	0	1	0
866986	GMVSILASSLLKNDI	NS2b	1357	1371	HLA-DPB1*01:01	100%	Dengue virus 2	ELISPOT	IFNg release	Positive	0	1	1	0	1	0
1067121	GKVVGLYGNGVVTRS	NS3	1619	1633	HLA-DRB1*15:01	100%	Dengue virus 2	ELISPOT	IFNg release	Positive	0	1	1	0	1	0
12372	EHTGREIVDLMCHAT	NS3	250	264	HLA class II	100%	Dengue virus 2	ELISPOT	IFNg release	Positive	0	1	1	0	1	0
12606	EIVDLMCHATFTMRL	NS3	255	269	HLA class II	100%	Dengue virus 2	ELISPOT	IFNg release	Positive	0	1	1	0	1	0
1392191	GREIVDLMCHATFTM	NS3	1728	1742		100%	Dengue virus 2	biological activity	activation	Positive	0	1	1	0	1	0
139758	GKTKRYLPAIVREAI	NS3	1673	1687	HLA-DRB1*09:01	100%	Dengue virus 2	ELISPOT	IFNg release	Positive	0	1	1	0	1	0
180698	PNYNLIIMDEAHFTD	NS3	1751	1765	HLA class II	100%	Dengue virus 2	ELISPOT	IFNg release	Positive	0	1	1	0	1	0
195295	FHTMWHVTRGAVLMH	NS3	1521	1535	HLA-DRB1*07:01	100%	Dengue virus 2	ELISPOT	IFNg release	Positive	0	1	1	0	1	0
195420	GTFHTMWHVTRGAVL	NS3	1519	1533	HLA-DRB1*07:01	100%	Dengue virus 2	ELISPOT	IFNg release	Positive	0	1	1	0	1	0
195888	RGLRTLILAPTRVVA	NS3	1689	1703	HLA-DRB1*08:02	100%	Dengue virus 2	ELISPOT	IFNg release	Positive	0	1	1	0	1	0
21115	GLRTLILAPTRVVAA	NS3	1690	1704	HLA-DRB1*04:03	100%	Dengue virus 2	ELISPOT	IFNg release	Positive	0	1	1	0	1	0
26965	ILAPTRVVAAEMEEA	NS3	1695	1709	HLA-DQA1*03:01/DQB1*03:02	100%	Dengue virus 2	ELISPOT	IFNg release	Positive	0	1	1	0	1	0
34231	KVVGLYGNGVVTRSG	NS3	145	159	HLA class II	100%	Dengue virus 2	ELISPOT	IFNg release	Positive	0	1	1	0	1	0
36560	LIIMDEAHFTDPASI	NS3	1755	1769	HLA-DQA1*01:01/DQB1*05:01	100%	Dengue virus 2	ELISPOT	IFNg release	Positive	0	1	1	0	1	0
39225	LRTLILAPTRVVAAE	NS3	1691	1705	HLA-DRB1*12:02	100%	Dengue virus 2	ELISPOT	IFNg release	Positive	0	1	1	0	1	0
49766	PTRVVAAEMEEALRG	NS3	223	237	HLA-DRB1*15:01	100%	Dengue virus 2	ICS	IFNg release	Positive	0	1	1	0	1	0
539025	AAGIFMTATPPGSRD	NS3	1784	1798	HLA-DRB1*04:03	100%	Dengue virus 2	ELISPOT	IFNg release	Positive	0	1	1	0	1	0
539041	AEMEEALRGLPIRYQ	NS3	1704	1718	HLA-DRB1*13:01	100%	Dengue virus 2	ELISPOT	IFNg release	Positive	0	1	1	0	1	0
539289	GEAAGIFMTATPPGS	NS3	1782	1796	HLA-DRB1*04:03	100%	Dengue virus 2	ELISPOT	IFNg release	Positive	0	1	1	0	1	0
539448	IVREAIKRGLRTLIL	NS3	1682	1696	HLA-DRB1*12:02	100%	Dengue virus 2	ELISPOT	IFNg release	Positive	0	1	1	0	1	0
539573	LILAPTRVVAAEMEE	NS3	1694	1708	HLA-DRB1*13:01	100%	Dengue virus 2	ELISPOT	IFNg release	Positive	0	1	1	0	1	0
540056	VLMHKGKRIEPSWAD	NS3	1518	1532	HLA-DRB1*13:01	100%	Dengue virus 2	ELISPOT	IFNg release	Positive	0	1	1	0	1	0
63672	TFHTMWHVTRGAVLM	NS3	45	59	HLA class II	100%	Dengue virus 2	ELISPOT	IFNg release	Positive	0	1	1	0	1	0
64666	TKRYLPAIVREAIKR	NS3	200	214	HLA class II	100%	Dengue virus 2	ELISPOT	IFNg release	Positive	0	1	1	0	1	0
740081	REAIKRGLRTLILAP	NS3	1670	1684	HLA-DRB1*14:06	100%	Dengue virus 2	ELISPOT	IFNg release	Positive	0	1	1	0	1	0
740193	RTLILAPTRVVAAEM	NS3	1678	1692	HLA-DRB1*14:06	100%	Dengue virus 2	ELISPOT	IFNg release	Positive	0	1	1	0	1	0
866477	EALRGLPIRYQTPAI	NS3	1708	1722	HLA-DRB4*01:01	100%	Dengue virus 2	ELISPOT	IFNg release	Positive	0	1	1	0	1	0
868734	RYLPAIVREAIKRGL	NS3	1677	1691	HLA-DRB4*01:01	100%	Dengue virus 2	ELISPOT	IFNg release	Positive	0	1	1	0	1	0
869224	TLILAPTRVVAAEME	NS3	1693	1707	HLA-DQA1*01:02/DQB1*06:02	100%	Dengue virus 2	ELISPOT	IFNg release	Positive	0	1	1	0	1	0
150362	LETLLLLTLLATVTG	NS4a	2145	2159	HLA-DRB1*01:01	100%	Dengue virus 2	ELISPOT	IFNg release	Positive	0	1	1	0	1	0
195107	AASIILEFFLIVLLI	NS4a	2185	2199	HLA-DRB1*15:01	100%	Dengue virus 2	ELISPOT	IFNg release	Positive	0	1	1	0	1	0
195767	MTLGMCCIITASILL	NS4a	2160	2174	HLA-DRB1*07:01	100%	Dengue virus 2	ELISPOT	IFNg release	Positive	0	1	1	0	1	0
196054	TLETLLLLTLLATVT	NS4a	2144	2158	HLA-DRB1*15:01	100%	Dengue virus 2	ELISPOT	IFNg release	Positive	0	1	1	0	1	0
738567	AYNHALSELPETLET	NS4a	2119	2133	HLA-DRB1*04:07	100%	Dengue virus 2	ELISPOT	IFNg release	Positive	0	1	1	0	1	0
195882	RFWNTTIAVSMANIF	NS4b	2454	2468	HLA-DRB1*07:01	100%	Dengue virus 2	ELISPOT	IFNg release	Positive	0	1	1	0	1	0
195974	SNILDIDLRPASAWT	NS4b	2155	2169	HLA-DRB1*03:01	100%	Dengue virus 2	ELISPOT	IFNg release	Positive	0	1	1	0	1	0
539724	NIFRGSYLAGAGLLF	NS4b	2452	2466	HLA-DRB1*09:01	100%	Dengue virus 2	ELISPOT	IFNg release	Positive	0	1	1	0	1	0
739437	LDIDLRPASAWTLYA	NS4b	2257	2271	HLA-DRB1*01:02	100%	Dengue virus 2	ELISPOT	IFNg release	Positive	0	1	1	0	1	0
866802	FWNTTIAVSMANIFR	NS4b	2455	2469	HLA-DQA1*01:02/DQB1*06:02	100%	Dengue virus 2	ELISPOT	IFNg release	Positive	0	1	1	0	1	0
869089	TAIANQATVLMGLGK	NS4b	2309	2323	HLA-DQA1*01:02/DQB1*06:02	100%	Dengue virus 2	ELISPOT	IFNg release	Positive	0	1	1	0	1	0
190862	MVTQMAMTDTTPFGQQR	NS5	2828	2844	HLA class II	100%	Dengue virus 2	ICS	IFNg release	Positive	0	1	1	0	1	0
195200	CETCVYNMMGKREKK	NS5	2924	2938	HLA-DRB1*11:01	100%	Dengue virus 2	ELISPOT	IFNg release	Positive	0	1	1	0	1	0
195213	DELIGRARISQGAGW	NS5	3223	3237	HLA-DRB1*08:02	100%	Dengue virus 2	ELISPOT	IFNg release	Positive	0	1	1	0	1	0
195233	EDHWFSRENSLSGVE	NS5	2985	2999	HLA-DRB1*04:01	100%	Dengue virus 2	ELISPOT	IFNg release	Positive	0	1	1	0	1	0
195409	GNIVSSVNMISRMLI	NS5	2717	2731	HLA-DRB1*07:01	100%	Dengue virus 2	ELISPOT	IFNg release	Positive	0	1	1	0	1	0
195948	SGNIVSSVNMISRML	NS5	2716	2730	HLA-DRB1*04:01	100%	Dengue virus 2	ELISPOT	IFNg release	Positive	0	1	1	0	1	0
196187	VSSVNMISRMLINRF	NS5	2720	2734	HLA-DRB1*15:01	100%	Dengue virus 2	ELISPOT	IFNg release	Positive	0	1	1	0	1	0
196210	WAKNIQTAINQVRSL	NS5	3336	3350	HLA-DRB1*03:01	100%	Dengue virus 2	ELISPOT	IFNg release	Positive	0	0	1	0	0	0
539436	ISGDDCVVKPLDDRFA	NS5	3151	3166	HLA class II	100%	Dengue virus 2	ICS	IFNg release	Positive	0	1	1	0	1	0
539871	RRDLRLAANAICSAV	NS5	3260	3274	HLA-DRB3*02:02	100%	Dengue virus 2	ELISPOT	IFNg release	Positive	0	1	1	0	1	0
866172	ASGNIVSSVNMISRM	NS5	2715	2729	HLA-DRB3*02:02	100%	Dengue virus 2	ELISPOT	IFNg release	Positive	0	1	1	0	1	0
867600	LEFEALGFLNEDHWF	NS5	2975	2989	HLA-DPB1*04:01	100%	Dengue virus 2	ELISPOT	IFNg release	Positive	0	1	1	0	1	0
868637	RDLRLAANAICSAVP	NS5	3261	3275	HLA-DQA1*05:01/DQB1*02:01	100%	Dengue virus 2	ELISPOT	IFNg release	Positive	0	1	1	0	1	0
868640	RFLEFEALGFLNEDH	NS5	2973	2987	HLA-DPB1*01:01	100%	Dengue virus 2	ELISPOT	IFNg release	Positive	0	1	1	0	1	0
869426	VESWEEIPYLGKRED	NS5	3321	3335	HLA-DQA1*01:01/DQB1*05:01	100%	Dengue virus 2	ELISPOT	IFNg release	Positive	0	1	1	0	1	0
1066603	ARNTPFNMLKRERNR	C	8	22	HLA-DRB1*11:01	81% - <100%	Dengue virus 2	ELISPOT	IFNg release	Positive-Low	0	1	1	0	0	0
150105	AFLRFLTIPPTAGIL	C	52	66	HLA-DRB1*01:01	81% - <100%	Dengue virus 2	ELISPOT	IFNg release	Positive	0	1	1	0	1	0
150369	LKLFMALVAFLRFLT	C	44	58	HLA-DRB1*01:01	81% - <100%	Dengue virus 2	ELISPOT	IFNg release	Positive	0	1	1	0	1	0
180597	LFMALVAFLRFLTIP	C	46	60	HLA class II	81% - <100%	Dengue virus 2	ELISPOT	IFNg release	Positive	0	1	1	0	1	0
180768	TIKKSKAINVLRGFR	C	71	85	HLA class II	81% - <100%	Dengue virus 2	ELISPOT	IFNg release	Positive	0	1	1	0	1	0
195307	FMALVAFLRFLTIPP	C	47	61	HLA-DRB1*11:01	81% - <100%	Dengue virus 2	ELISPOT	IFNg release	Positive	0	1	1	0	1	0
195573	KLFMALVAFLRFLTI	C	45	59	HLA-DRB1*15:01	81% - <100%	Dengue virus 2	ELISPOT	IFNg release	Positive	0	1	1	0	1	0
195730	LVAFLRFLTIPPTAG	C	50	64	HLA-DRB1*04:01	81% - <100%	Dengue virus 2	ELISPOT	IFNg release	Positive	0	1	1	0	1	0
195744	MALVAFLRFLTIPPT	C	48	62	HLA-DRB1*04:01	81% - <100%	Dengue virus 2	ELISPOT	IFNg release	Positive	0	1	1	0	1	0
195889	RGPLKLFMALVAFLR	C	41	55	HLA-DRB1*04:01	81% - <100%	Dengue virus 2	ELISPOT	IFNg release	Positive	0	1	1	0	1	0
196064	TPFNMLKRERNRVST	C	11	25	HLA-DRB1*08:02	81% - <100%	Dengue virus 2	ELISPOT	IFNg release	Positive	0	1	1	0	1	0
196093	VAFLRFLTIPPTAGI	C	51	65	HLA-DRB1*04:01	81% - <100%	Dengue virus 2	ELISPOT	IFNg release	Positive	0	1	1	0	1	0
196213	WGTIKKSKAINVLRG	C	69	83	HLA-DRB1*07:01	81% - <100%	Dengue virus 2	ELISPOT	IFNg release	Positive	0	1	1	0	1	0
26836	IKKSKAINVLRGFRKEIGRM	C	72	91	HLA class II	81% - <100%	Dengue virus 2	ELISPOT	IFNg release	Positive	0	1	1	0	1	0
539083	ALVAFLRFLTIPPTA	C	49	63	HLA-DRB1*12:02	81% - <100%	Dengue virus 2	ELISPOT	IFNg release	Positive	0	1	1	0	1	0
539264	FLRFLTIPPTAGILK	C	53	67	HLA-DRB1*14:02	81% - <100%	Dengue virus 2	ELISPOT	IFNg release	Positive	0	1	1	0	1	0
539410	IKKSKAINVLRGFRK	C	72	86	HLA-DRB1*14:04	81% - <100%	Dengue virus 2	ELISPOT	IFNg release	Positive	0	1	1	0	1	0
539514	KSKAINVLRGFRKEI	C	74	88	HLA-DRB1*12:02	81% - <100%	Dengue virus 2	ELISPOT	IFNg release	Positive	0	1	1	0	1	0
539612	LQGRGPLKLFMALVA	C	38	52	HLA-DRB1*15:02	81% - <100%	Dengue virus 2	ELISPOT	IFNg release	Positive	0	1	1	0	1	0
539887	RWGTIKKSKAINVLR	C	68	82	HLA-DRB1*08:03	81% - <100%	Dengue virus 2	ELISPOT	IFNg release	Positive	0	1	1	0	1	0
540069	VQQLTKRFSLGMLQG	C	26	40	HLA-DRB1*13:01	81% - <100%	Dengue virus 2	ELISPOT	IFNg release	Positive	0	1	1	0	1	0
739879	PFNMLKRERNRVSTV	C	12	26	HLA-DRB1*14:02	81% - <100%	Dengue virus 2	ELISPOT	IFNg release	Positive	0	1	1	0	1	0
857784	LKRWGTIKKSKAINV	C	66	80	HLA-DRB1*07:01	81% - <100%	Dengue virus 2	ELISPOT	IFNg release	Positive-Low	0	1	1	0	1	0
150490	PGFTIMAAILAYTIG	PreM	245	259	HLA-DRB1*01:01	81% - <100%	Dengue virus 2	ELISPOT	IFNg release	Positive	1	1	1	1	0	1
195322	FQRVLIFILLTAVAP	PreM	263	277	HLA-DRB1*07:01	81% - <100%	Dengue virus 2	ELISPOT	IFNg release	Positive	0	0	0	0	0	0
195503	ILRHPGFTIMAAILA	PreM	127	141	HLA-DRB1*07:01	81% - <100%	Dengue virus 2	ELISPOT	IFNg release	Positive	1	1	1	1	0	1
195702	LRHPGFTIMAAILAY	PreM	242	256	HLA-DRB1*11:01	81% - <100%	Dengue virus 2	ELISPOT	IFNg release	Positive	1	1	1	1	0	1
195863	QRVLIFILLTAVAPS	PreM	264	278	HLA-DRB1*08:02	81% - <100%	Dengue virus 2	ELISPOT	IFNg release	Positive	0	0	0	0	0	0
195892	RIETWILRHPGFTIM	PreM	122	136	HLA-DRB1*15:01	81% - <100%	Dengue virus 2	ELISPOT	IFNg release	Positive	1	1	1	0	0	1
539943	SVALVPHVGMGLETR	PreM	206	220	HLA-DRB1*15:02	81% - <100%	Dengue virus 2	ELISPOT	IFNg release	Positive	1	1	1	1	1	1
540130	WILRHPGFTIMAAIL	PreM	126	140	HLA-DRB1*15:06	81% - <100%	Dengue virus 2	ELISPOT	IFNg release	Positive	1	1	1	1	0	1
144940	RHVLGRLITVNPIVT	E	345	359	HLA-DRB1*08:02	81% - <100%	Dengue virus 2	ELISPOT	IFNg release	Positive	1	1	1	1	1	1
180564	KKQDVVVLGSQEGAM	E	526	540	HLA class II	81% - <100%	Dengue virus 2	ELISPOT	IFNg release	Positive	1	1	1	1	1	1
180568	KNPHAKKQDVVVLGS	E	521	535	HLA class II	81% - <100%	Dengue virus 2	ELISPOT	IFNg release	Positive	1	1	1	1	1	1
180820	WLVHRQWFLDLPLPW	E	196	210	HLA class II	81% - <100%	Dengue virus 2	ELISPOT	IFNg release	Positive	1	1	1	1	1	1
195638	LGRLITVNPIVTEKD	E	628	642	HLA-DRB1*08:02	81% - <100%	Dengue virus 2	ELISPOT	IFNg release	Positive	1	1	1	1	1	1
195716	LSVSLVLVGVVTLYL	E	755	769	HLA-DRB1*15:01	81% - <100%	Dengue virus 2	ELISPOT	IFNg release	Positive	1	0	1	1	0	0
196077	TSLSVSLVLVGVVTL	E	753	767	HLA-DRB1*07:01	81% - <100%	Dengue virus 2	ELISPOT	IFNg release	Positive	1	0	1	1	0	0
196140	VLGRLITVNPIVTEK	E	627	641	HLA-DRB1*08:02	81% - <100%	Dengue virus 2	ELISPOT	IFNg release	Positive	1	1	1	1	1	1
196215	WIQKETLVTFKNPHA	E	231	245	HLA-DRB1*08:02	81% - <100%	Dengue virus 2	ELISPOT	IFNg release	Positive	1	1	1	1	1	1
539252	FETTMRGAKRMAILG	E	402	416	HLA-DRB1*15:02	81% - <100%	Dengue virus 2	ELISPOT	IFNg release	Positive	0	1	0	1	1	0
539377	HAKKQDVVVLGSQEGAMH	E	244	261	HLA class II	81% - <100%	Dengue virus 2	ICS	IFNg release	Positive	1	1	1	1	1	1
539464	KETLVTFKNPHAKKQ	E	234	248	HLA-DRB1*10:01	81% - <100%	Dengue virus 2	ELISPOT	IFNg release	Positive	1	1	1	1	1	1
539806	QKETLVTFKNPHAKK	E	513	527	HLA-DRB1*04:03	81% - <100%	Dengue virus 2	ELISPOT	IFNg release	Positive	1	1	1	1	1	1
65140	TLVTFKNPHAKKQDV	E	236	250	HLA class II	81% - <100%	Dengue virus 2	ELISPOT	IFNg release	Positive	1	1	1	1	1	1
739010	GGVFTSIGKALHQVF	E	706	720	HLA-DRB1*14:02	81% - <100%	Dengue virus 2	ELISPOT	IFNg release	Positive	1	1	1	1	1	0
740315	SLSVSLVLVGVVTLY	E	754	768	HLA-DRB1*01:02	81% - <100%	Dengue virus 2	ELISPOT	IFNg release	Positive	1	0	1	1	0	0
868713	RTGLDFNEMVLLQME	E	178	192	HLA-DQA1*01:02/DQB1*06:02	81% - <100%	Dengue virus 2	ELISPOT	IFNg release	Positive	1	1	1	1	1	1
869123	TEKDSPVNIEAEPPF	E	349	363	HLA-DQA1*03:01/DQB1*03:02	81% - <100%	Dengue virus 2	ELISPOT	IFNg release	Positive	1	1	1	1	1	1
869153	TGLDFNEMVLLQMEN	E	179	193	HLA-DQA1*03:01/DQB1*03:02	81% - <100%	Dengue virus 2	ELISPOT	IFNg release	Positive	1	1	1	1	1	1
167759	IMTGDIKGIMQA	NS1	88	99	HLA-DQ6	81% - <100%	Dengue virus 2	ELISPOT	IFNg release	Positive	0	1	1	0	1	0
180396	DGCWYGMEIRPLKEKEENLVNS	NS1	1102	1123	HLA class II	81% - <100%	Dengue virus 2	ICS	IFNg release	Positive	0	1	1	0	1	0
180603	LKYSWKTWGKAKMLS	NS1	111	125	HLA class II	81% - <100%	Dengue virus 2	ELISPOT	IFNg release	Positive	0	1	1	0	1	0
180612	LNDTWKIEKASFIEV	NS1	981	995	HLA class II	81% - <100%	Dengue virus 2	ELISPOT	IFNg release	Positive	0	1	1	0	1	0
195458	HNQTFLIDGPETAEC	NS1	904	918	HLA-DRB1*04:01	81% - <100%	Dengue virus 2	ELISPOT	IFNg release	Positive	0	1	1	0	1	0
195576	KLTIMTGDIKGIMQA	NS1	85	99	HLA-DRB1*03:01	81% - <100%	Dengue virus 2	ELISPOT	IFNg release	Positive	0	1	1	0	1	0
195845	QDVFCDSKLMSAAIK	NS1	175	189	HLA-DRB1*03:01	81% - <100%	Dengue virus 2	ELISPOT	IFNg release	Positive	0	0	1	0	1	0
539828	QYKFQPESPSKLASA	NS1	806	820	HLA-DRB3*02:02	81% - <100%	Dengue virus 2	ELISPOT	IFNg release	Positive	0	1	1	0	1	0
738586	CGIRSVTRLENLMWK	NS1	55	69	HLA-DRB1*11:04	81% - <100%	Dengue virus 2	ELISPOT	IFNg release	Positive	0	1	1	0	1	0
738766	EGICGIRSVTRLENL	NS1	52	66	HLA-DRB1*11:04	81% - <100%	Dengue virus 2	ELISPOT	IFNg release	Positive	0	1	1	0	1	0
739192	IKGIMQAGKRSLRPQ	NS1	93	107	HLA-DRB1*14:06	81% - <100%	Dengue virus 2	ELISPOT	IFNg release	Positive	0	1	0	0	1	0
739296	KGIMQAGKRSLRPQP	NS1	94	108	HLA-DRB1*14:06	81% - <100%	Dengue virus 2	ELISPOT	IFNg release	Positive	0	1	0	0	1	0
866460	DYGFGVFTTNIWLKL	NS1	932	946	HLA-DPB1*02:01	81% - <100%	Dengue virus 2	ELISPOT	IFNg release	Positive	0	1	1	0	1	0
866830	GFGVFTTNIWLKLKE	NS1	934	948	HLA-DPB1*04:01	81% - <100%	Dengue virus 2	ELISPOT	IFNg release	Positive	0	1	0	0	1	0
867040	HADMGYWIESALNDT	NS1	970	984	HLA-DQA1*01:01/DQB1*05:01	81% - <100%	Dengue virus 2	ELISPOT	IFNg release	Positive	0	1	1	0	1	0
150131	ALTIKGLNPTAIFLT	NS2a	1308	1322	HLA-DRB1*09:01	81% - <100%	Dengue virus 2	ELISPOT	IFNg release	Positive	0	1	1	0	1	0
195153	ALLAAFKVRPTFAAG	NS2a	1203	1217	HLA-DRB1*08:02	81% - <100%	Dengue virus 2	ELISPOT	IFNg release	Positive	0	1	1	0	1	0
195650	LKIVRNMEKYQLAVT	NS2a	1261	1275	HLA-DRB1*13:01	81% - <100%	Dengue virus 2	ELISPOT	IFNg release	Positive	0	0	0	0	0	0

195752	MEKYQLAVTIMAILC	NS2a	1154	1168	HLA-DRB1*04:07	81% - <100%	Dengue virus 2	ELISPOT	IFNg release	Positive	0	1	1	0	1	0
195798	NMEKYQLAVTIMAIL	NS2a	1266	1280	HLA-DRB1*04:01	81% - <100%	Dengue virus 2	ELISPOT	IFNg release	Positive	0	1	0	0	1	0
196174	VRNMEKYQLAVTIMA	NS2a	1264	1278	HLA-DRB1*13:01	81% - <100%	Dengue virus 2	ELISPOT	IFNg release	Positive	0	1	0	0	1	0
539474	KIVRNMEKYQLAVTI	NS2a	1262	1276	HLA-DRB1*13:01	81% - <100%	Dengue virus 2	ELISPOT	IFNg release	Positive	0	0	0	0	0	0
539540	LALLAAFKVRPTFAA	NS2a	1202	1216	HLA-DRB1*14:04	81% - <100%	Dengue virus 2	ELISPOT	IFNg release	Positive	0	1	1	0	1	0
540154	YLALLAAFKVRPTFA	NS2a	1201	1215	HLA-DRB1*14:04	81% - <100%	Dengue virus 2	ELISPOT	IFNg release	Positive	0	1	1	0	1	0
866978	GMALFLEEMLRTRVG	NS2a	1141	1155	HLA-DQA1*01:01/DQB1*05:01	81% - <100%	Dengue virus 2	ELISPOT	IFNg release	Positive	0	1	1	0	1	0
867216	ILCVPNAVILQNAWK	NS2a	1279	1293	HLA-DQA1*01:02/DQB1*06:02	81% - <100%	Dengue virus 2	ELISPOT	IFNg release	Positive	0	1	1	0	1	0
867571	LCVPNAVILQNAWKV	NS2a	1280	1294	HLA-DRB4*01:01	81% - <100%	Dengue virus 2	ELISPOT	IFNg release	Positive	0	1	1	0	1	0
868918	SLGVLGMALFLEEML	NS2a	1136	1150	HLA-DQA1*05:01/DQB1*02:01	81% - <100%	Dengue virus 2	ELISPOT	IFNg release	Positive	0	1	1	0	1	0
167732	DQAEISGSSPILSITISEDG	NS2b	63	82	HLA-DP	81% - <100%	Dengue virus 2	ELISPOT	IFNg release	Positive	0	1	1	0	1	0
195239	EEQTLTILIRTGLLV	NS2b	91	105	HLA-DRB1*15:01	81% - <100%	Dengue virus 2	ELISPOT	IFNg release	Positive	0	1	1	0	1	0
195723	LTILIRTGLLVISGL	NS2b	1440	1454	HLA-DRB1*15:06	81% - <100%	Dengue virus 2	ELISPOT	IFNg release	Positive	0	1	1	0	1	0
195869	QTLTILIRTGLLVIS	NS2b	1438	1452	HLA-DRB1*15:01	81% - <100%	Dengue virus 2	ELISPOT	IFNg release	Positive	0	1	1	0	1	0
139762	GLPIRYQTPAIRAEH	NS3	1712	1726	HLA-DRB1*04:03	81% - <100%	Dengue virus 2	ELISPOT	IFNg release	Positive	0	1	1	0	1	0
180807	VTDFKGKTVWFVPSI	NS3	1826	1840	HLA class II	81% - <100%	Dengue virus 2	ELISPOT	IFNg release	Positive-Low	0	1	1	0	1	0
195154	ALRGLPIRYQTPAIR	NS3	1709	1723	HLA-DRB1*08:02	81% - <100%	Dengue virus 2	ELISPOT	IFNg release	Positive	0	1	1	0	1	0
195564	KGKVVGLYGNGVVTR	NS3	1618	1632	HLA-DRB1*15:01	81% - <100%	Dengue virus 2	ELISPOT	IFNg release	Positive	0	1	1	0	1	0
195765	MRLLSPVRVPNYNLI	NS3	1742	1756	HLA-DRB1*13:01	81% - <100%	Dengue virus 2	ELISPOT	IFNg release	Positive	0	1	1	0	1	0
31726	KKVIQLSRKTFDSEY	NS3	1855	1869	HLA-DRB1*12:02	81% - <100%	Dengue virus 2	ELISPOT	IFNg release	Positive	0	1	1	0	1	0
39103	LRGLPIRYQTPAIRA	NS3	1696	1710	HLA-DRB1*14:02	81% - <100%	Dengue virus 2	ELISPOT	IFNg release	Positive	0	1	1	0	1	0
4152	ARGYISTRVEMGEAA	NS3	1757	1771	HLA-DRB1*04:07	81% - <100%	Dengue virus 2	ELISPOT	IFNg release	Positive	0	1	1	0	1	0
539203	EERVILAGPMPVTHS	NS3	1899	1913	HLA-DRB1*09:01	81% - <100%	Dengue virus 2	ELISPOT	IFNg release	Positive	0	1	1	0	1	0
539467	KGKTVWFVPSIKAGN	NS3	1830	1844	HLA-DRB1*08:03	81% - <100%	Dengue virus 2	ELISPOT	IFNg release	Positive	0	1	1	0	1	0
539713	NGKKVIQLSRKTFDS	NS3	1853	1867	HLA-DRB1*12:02	81% - <100%	Dengue virus 2	ELISPOT	IFNg release	Positive	0	1	1	0	1	0
539850	RGLPIRYQTPAIRAE	NS3	1711	1725	HLA-DRB1*13:01	81% - <100%	Dengue virus 2	ELISPOT	IFNg release	Positive	0	1	1	0	1	0
539857	RKNGKKVIQLSRKTF	NS3	1851	1865	HLA-DRB1*08:03	81% - <100%	Dengue virus 2	ELISPOT	IFNg release	Positive	0	1	1	0	1	0
868724	RTNDWDFVVTTDISE	NS3	1873	1887	HLA-DQA1*03:01/DQB1*03:02	81% - <100%	Dengue virus 2	ELISPOT	IFNg release	Positive	0	1	1	0	1	0
8911	DKKGKVVGLYGNGVV	NS3	141	155	HLA-DRB1*15:01	81% - <100%	Dengue virus 2	ICS	IFNg release	Positive	0	1	1	0	1	0
195355	GGIFLFLMSGKGIGK	NS4a	2145	2159	HLA-DRB1*11:01	81% - <100%	Dengue virus 2	ELISPOT	IFNg release	Positive	0	0	0	0	0	0
195414	GRAYNHALSELPETL	NS4a	2131	2145	HLA-DRB1*04:01	81% - <100%	Dengue virus 2	ELISPOT	IFNg release	Positive	0	1	1	0	1	0
195635	LGMCCIITASILLWY	NS4a	2176	2190	HLA-DRB1*07:01	81% - <100%	Dengue virus 2	ELISPOT	IFNg release	Positive	0	1	1	0	1	0
196089	TVTGGIFLFLMSGKG	NS4a	2142	2156	HLA-DRB1*11:01	81% - <100%	Dengue virus 2	ELISPOT	IFNg release	Positive	0	0	0	0	0	0
539609	LPTFMTQKARNALDN	NS4a	2092	2106	HLA-DRB1*10:01	81% - <100%	Dengue virus 2	ELISPOT	IFNg release	Positive	0	0	0	0	0	0
539801	QDNQLTYVVIAILTV	NS4a	2223	2237	HLA-DRB1*15:02	81% - <100%	Dengue virus 2	ELISPOT	IFNg release	Positive	0	1	1	0	1	0
539804	QIQPHWIAASIILEF	NS4a	2192	2206	HLA-DRB1*13:01	81% - <100%	Dengue virus 2	ELISPOT	IFNg release	Positive	0	1	1	0	1	0
540148	YAQIQPHWIAASIIL	NS4a	2176	2190	HLA-DRB1*13:01	81% - <100%	Dengue virus 2	ELISPOT	IFNg release	Positive	0	1	1	0	1	0
150611	TAALLLLVAHYAIIG	NS4b	2351	2365	HLA-DRB1*01:01	81% - <100%	Dengue virus 2	ELISPOT	IFNg release	Positive	0	1	0	0	0	0
150636	TLTAALLLLVAHYAI	NS4b	2335	2349	HLA-DRB1*01:01	81% - <100%	Dengue virus 2	ELISPOT	IFNg release	Positive	0	1	0	0	0	0
195115	AGAGLLFSIMKNTTN	NS4b	2474	2488	HLA-DRB1*11:01	81% - <100%	Dengue virus 2	ELISPOT	IFNg release	Positive	0	1	1	0	1	0
195347	GAGLLFSIMKNTTNT	NS4b	2475	2489	HLA-DRB1*11:01	81% - <100%	Dengue virus 2	ELISPOT	IFNg release	Positive	0	1	1	0	1	0
195621	LCVTQVLMMRTTWAL	NS4b	2405	2419	HLA-DRB1*07:01	81% - <100%	Dengue virus 2	ELISPOT	IFNg release	Positive	0	1	1	0	1	0
195629	LFSIMKNTTNTRRGT	NS4b	2479	2493	HLA-DRB1*03:01	81% - <100%	Dengue virus 2	ELISPOT	IFNg release	Positive	0	1	1	0	1	0
195658	LLFSIMKNTTNTRRG	NS4b	2478	2492	HLA-DRB1*04:01	81% - <100%	Dengue virus 2	ELISPOT	IFNg release	Positive	0	1	1	0	1	0
195827	PESNILDIDLRPASA	NS4b	2266	2280	HLA-DRB1*03:01	81% - <100%	Dengue virus 2	ELISPOT	IFNg release	Positive	0	1	1	0	1	0
195980	SQVNPITLTAALLLL	NS4b	2343	2357	HLA-DQA1*01:02/DQB1*06:02	81% - <100%	Dengue virus 2	ELISPOT	IFNg release	Positive	0	1	0	0	0	0
539327	GLLFSIMKNTTNTRR	NS4b	2477	2491	HLA-DRB1*14:02	81% - <100%	Dengue virus 2	ELISPOT	IFNg release	Positive	0	1	1	0	1	0
867860	LLVLCVTQVLMMRTT	NS4b	2416	2430	HLA-DPB1*04:02	81% - <100%	Dengue virus 2	ELISPOT	IFNg release	Positive	0	1	1	0	1	0
1066545	ACLGKSYAQMWSLMY	NS5	3243	3257	HLA-DRB1*04:01	81% - <100%	Dengue virus 2	ELISPOT	IFNg release	Positive-Low	0	1	1	0	1	0
150597	SRAIWYMWLGARFLE	NS5	2962	2976	HLA-DRB1*01:01	81% - <100%	Dengue virus 2	ELISPOT	IFNg release	Positive	0	1	1	0	1	0
180579	KSYAQMWSLMYFHRR	NS5	3247	3261	HLA-DRB5*01:01	81% - <100%	Dengue virus 2	ELISPOT	IFNg release	Positive	0	1	1	0	1	0
180582	KTWAYHGSYETKQTG	NS5	2792	2806	HLA class II	81% - <100%	Dengue virus 2	ICS	IFNg release	Positive	0	1	1	0	1	0
195251	ELIMKDGRVLVVPCR	NS5	3206	3220	HLA-DRB1*03:01	81% - <100%	Dengue virus 2	ELISPOT	IFNg release	Positive	0	1	1	0	1	0
195380	GKSYAQMWSLMYFHR	NS5	3232	3246	HLA-DRB1*04:01	81% - <100%	Dengue virus 2	ELISPOT	IFNg release	Positive	0	1	1	0	1	0
195428	GTVMDIISRRDQRGS	NS5	3078	3092	HLA-DRB1*03:01	81% - <100%	Dengue virus 2	ELISPOT	IFNg release	Positive	0	1	1	0	1	0
195801	NMISRMLINRFTMRH	NS5	6	20	HLA-DRB1*08:02	81% - <100%	Dengue virus 2	ELISPOT	IFNg release	Positive	0	1	1	0	0	0
196161	VNMISRMLINRFTMR	NS5	2723	2737	HLA-DRB1*10:01	81% - <100%	Dengue virus 2	ELISPOT	IFNg release	Positive	0	1	1	0	1	0
196223	WSLMYFHRRDLRLAA	NS5	3253	3267	HLA-DRB1*03:01	81% - <100%	Dengue virus 2	ELISPOT	IFNg release	Positive	0	1	1	0	1	0
196243	YQNKVVRVQRPTPRG	NS5	3064	3078	HLA-DRB1*08:02	81% - <100%	Dengue virus 2	ELISPOT	IFNg release	Positive	0	1	1	0	1	0
539144	CVVKPLDDRFASALTAL	NS5	3156	3172	HLA class II	81% - <100%	Dengue virus 2	ICS	IFNg release	Positive	0	1	1	0	1	0
539150	DDRFASALTALNDMG	NS5	3162	3176	HLA-DRB1*04:03	81% - <100%	Dengue virus 2	ELISPOT	IFNg release	Positive	0	1	1	0	1	0
539195	EAIFKLTYQNKVVRV	NS5	3057	3071	HLA-DRB1*09:01	81% - <100%	Dengue virus 2	ELISPOT	IFNg release	Positive	0	1	1	0	1	0
539438	ISRMLINRFTMRHKK	NS5	8	22	HLA-DRB1*14:02	81% - <100%	Dengue virus 2	ELISPOT	IFNg release	Positive	0	1	1	0	0	0
539599	LMYFHRRDLRLAANA	NS5	3255	3269	HLA-DRB1*13:01	81% - <100%	Dengue virus 2	ELISPOT	IFNg release	Positive	0	1	1	0	1	0
539669	MISRMLINRFTMRHK	NS5	7	21	HLA-DRB1*14:06	81% - <100%	Dengue virus 2	ELISPOT	IFNg release	Positive	0	1	1	0	0	0
540064	VNGVVRLLTKPWDVI	NS5	2812	2826	HLA-DRB1*12:02	81% - <100%	Dengue virus 2	ELISPOT	IFNg release	Positive	0	0	0	0	0	0
738754	EEFTRKVRSNAALGA	NS5	2874	2888	HLA-DRB1*11:04	81% - <100%	Dengue virus 2	ELISPOT	IFNg release	Positive	0	1	1	0	1	0
739166	IFKLTYQNKVVRVQR	NS5	3045	3059	HLA-DRB1*14:06	81% - <100%	Dengue virus 2	ELISPOT	IFNg release	Positive	0	1	1	0	1	0
739791	NLVRLQSGVDVFFTP	NS5	2599	2613	HLA-DRB1*01:02	81% - <100%	Dengue virus 2	ELISPOT	IFNg release	Positive	0	0	1	0	0	0
740675	VRSNAALGAIFTDEN	NS5	2880	2894	HLA-DRB1*11:04	81% - <100%	Dengue virus 2	ELISPOT	IFNg release	Positive	0	1	1	0	1	0
866015	AIWYMWLGARFLEFE	NS5	2964	2978	HLA-DPB1*01:01	81% - <100%	Dengue virus 2	ELISPOT	IFNg release	Positive	0	1	1	0	1	0
866566	EHKKLAEAIFKLTYQ	NS5	3051	3065	HLA-DPB1*01:01	81% - <100%	Dengue virus 2	ELISPOT	IFNg release	Positive	0	1	1	0	1	0
867419	KKLAEAIFKLTYQNK	NS5	3053	3067	HLA-DPB1*02:01	81% - <100%	Dengue virus 2	ELISPOT	IFNg release	Positive	0	1	1	0	1	0
867433	KLAEAIFKLTYQNKV	NS5	3054	3068	HLA-DPB1*02:01	81% - <100%	Dengue virus 2	ELISPOT	IFNg release	Positive	0	1	1	0	1	0
867514	KTPVESWEEIPYLGK	NS5	3318	3332	HLA-DQA1*01:01/DQB1*05:01	81% - <100%	Dengue virus 2	ELISPOT	IFNg release	Positive	0	1	1	0	1	0
868668	RKVRSNAALGAIFTD	NS5	2892	2906	HLA-DRB3*02:02	81% - <100%	Dengue virus 2	ELISPOT	IFNg release	Positive	0	1	1	0	1	0
869082	SYAQMWSLMYFHRRD	NS5	3248	3262	HLA-DPB1*04:01	81% - <100%	Dengue virus 2	ELISPOT	IFNg release	Positive	0	1	1	0	1	0
869307	TRKVRSNAALGAIFT	NS5	2891	2905	HLA-DRB3*02:02	81% - <100%	Dengue virus 2	ELISPOT	IFNg release	Positive	0	1	1	0	1	0
195417	GRMLNILNRRRRTAG	C	89	103	HLA-DRB1*11:01	51%-80%	Dengue virus 2	ELISPOT	IFNg release	Positive	0	0	1	0	0	0
195489	IGRMLNILNRRRRTA	C	88	102	HLA-DRB1*08:02	51%-80%	Dengue virus 2	ELISPOT	IFNg release	Positive	0	0	1	0	0	0
195899	RMLNILNRRRRTAGV	C	71	85	HLA-DRB1*08:02	51%-80%	Dengue virus 2	ELISPOT	IFNg release	Positive	0	0	0	0	0	0
196133	VKLTIMTGDIKGIMQ	NS1	84	98	HLA-DRB1*03:01	51%-80%	Dengue virus 2	ELISPOT	IFNg release	Positive	0	1	1	0	1	0
195207	CTILAVVSVSPLLLT	NS2a	1144	1158	HLA-DRB1*07:01	51%-80%	Dengue virus 2	ELISPOT	IFNg release	Positive	0	1	0	0	1	0
195615	LAVVSVSPLLLTSSQ	NS2a	1186	1200	HLA-DRB1*07:01	51%-80%	Dengue virus 2	ELISPOT	IFNg release	Positive	0	1	0	0	1	0
196218	WKVSCTILAVVSVSP	NS2a	1292	1306	HLA-DRB1*04:01	51%-80%	Dengue virus 2	ELISPOT	IFNg release	Positive	0	1	1	0	1	0
539561	LGMMILKIVRNMEKY	NS2a	1256	1270	HLA-DRB1*13:01	51%-80%	Dengue virus 2	ELISPOT	IFNg release	Positive	0	0	0	0	0	0
740700	VVSVSPLLLTSSQQK	NS2a	1188	1202	HLA-DRB1*01:02	51%-80%	Dengue virus 2	ELISPOT	IFNg release	Positive	0	1	0	0	1	0
30396	KEGERKKLKPRWLDA	NS3	584	598	HLA class II	51%-80%	Dengue virus 2	ICS	IFNg release	Positive	0	1	1	0	1	0
539200	EDDIFRKKRLTIMDL	NS3	1654	1668	HLA-DRB1*13:01	51%-80%	Dengue virus 2	ELISPOT	IFNg release	Positive	0	0	0	0	0	0
167780	LTLNLITEMGRLPTFMTQKA	NS4a	2	21	HLA class II	51%-80%	Dengue virus 2	ELISPOT	IFNg release	Positive	0	1	1	0	1	0
150247	GMIIMLIPTVMAFHL	C	103	117	HLA-DRB1*01:01	>0% - 50%	Dengue virus 2	ELISPOT	IFNg release	Positive	0	1	1	0	1	0
195120	AGMIIMLIPTVMAFH	C	102	116	HLA-DRB1*08:02	>0% - 50%	Dengue virus 2	ELISPOT	IFNg release	Positive	0	1	1	0	1	0
195496	IIMLIPTVMAFHLTT	C	105	119	HLA-DRB1*01:02	>0% - 50%	Dengue virus 2	ELISPOT	IFNg release	Positive	0	1	1	0	1	0
739181	IGRMLNILNRRRRTV	C	42	56	HLA-DRB1*14:02	>0% - 50%	Dengue virus 2	ELISPOT	IFNg release	Positive	0	0	0	0	0	0
195862	QRALIFILLTAVAPS	PreM	264	278	HLA-DRB1*15:01	>0% - 50%	Dengue virus 2	ELISPOT	IFNg release	Positive	1	1	1	1	0	1
196141	VLIFILLTAIAPSMT	PreM	266	280	HLA-DRB1*08:02	>0% - 50%	Dengue virus 2	ELISPOT	IFNg release	Positive	0	0	0	0	0	0
539881	RVLIFILLTAIAPSM	PreM	265	279	HLA-DRB1*15:06	>0% - 50%	Dengue virus 2	ELISPOT	IFNg release	Positive	0	0	0	0	0	0
539236	ENLEYTIVITPHSGE	E	133	147	HLA-DRB1*04:07	>0% - 50%	Dengue virus 2	ELISPOT	IFNg release	Positive	1	0	1	0	1	1
539733	NLEYTIVITPHSGEE	E	134	148	HLA-DRB1*04:07	>0% - 50%	Dengue virus 2	ELISPOT	IFNg release	Positive	1	0	1	0	1	1
866243	AWMVHRQWFLDLPLP	E	205	219	HLA-DQA1*01:01/DQB1*05:01	>0% - 50%	Dengue virus 2	ELISPOT	IFNg release	Positive	0	0	0	0	0	0
869152	TGLDFNEMVLLQMED	E	189	203	HLA-DQA1*03:01/DQB1*03:02	>0% - 50%	Dengue virus 2	ELISPOT	IFNg release	Positive	0	0	0	0	0	0
195583	KQDVFCDSKLMSAAI	NS1	949	963	HLA-DRB1*03:01	>0% - 50%	Dengue virus 2	ELISPOT	IFNg release	Positive	0	0	1	0	1	0
195880	REKQDVFCDSKLMSA	NS1	947	961	HLA-DRB1*03:01	>0% - 50%	Dengue virus 2	ELISPOT	IFNg release	Positive	0	0	1	0	0	0
195406	GMMVLKIVRNMEKYQ	NS2a	1257	1271	HLA-DRB1*08:02	>0% - 50%	Dengue virus 2	ELISPOT	IFNg release	Positive	0	0	0	0	0	0
195567	KHAILLVAVSFVTLI	NS2a	1157	1171	HLA-DRB1*07:01	>0% - 50%	Dengue virus 2	ELISPOT	IFNg release	Positive	0	1	1	0	1	0
195610	LALGMMVLKIVRNME	NS2a	1254	1268	HLA-DRB1*13:01	>0% - 50%	Dengue virus 2	ELISPOT	IFNg release	Positive	0	0	0	0	0	0
195688	LNPTAIFLTTLSRTS	NS2a	1328	1342	HLA-DRB1*07:01	>0% - 50%	Dengue virus 2	ELISPOT	IFNg release	Positive	0	1	0	0	1	0
195753	MEKYQLAVTIMAISC	NS2a	1267	1281	HLA-DRB1*10:01	>0% - 50%	Dengue virus 2	ELISPOT	IFNg release	Positive	0	0	0	0	0	0
195799	NMEKYQLAVTIMAIS	NS2a	1266	1280	HLA-DRB1*04:01	>0% - 50%	Dengue virus 2	ELISPOT	IFNg release	Positive	0	0	0	0	0	0
196237	YQLAVTIMAISCVPN	NS2a	1270	1284	HLA-DRB1*08:02	>0% - 50%	Dengue virus 2	ELISPOT	IFNg release	Positive	0	0	0	0	0	0
539108	AVSFVTLITGNMSFR	NS2a	1164	1178	HLA-DRB1*10:01	>0% - 50%	Dengue virus 2	ELISPOT	IFNg release	Positive	0	1	1	0	1	0
539231	EMLRTRVGTKHAILL	NS2a	1148	1162	HLA-DRB1*08:03	>0% - 50%	Dengue virus 2	ELISPOT	IFNg release	Positive	0	1	1	0	1	0
539562	LGMMVLKIVRNMEKY	NS2a	1256	1270	HLA-DRB1*12:02	>0% - 50%	Dengue virus 2	ELISPOT	IFNg release	Positive	0	0	0	0	0	0
539686	MMVLKIVRNMEKYQL	NS2a	1244	1258	HLA-DRB1*14:06	>0% - 50%	Dengue virus 2	ELISPOT	IFNg release	Positive	0	0	0	0	0	0
539699	MVLKMVRNMEKYQLA	NS2a	1259	1273	HLA-DRB1*13:01	>0% - 50%	Dengue virus 2	ELISPOT	IFNg release	Positive	0	1	0	0	1	0
539789	PTAIFLTTLSRTSKK	NS2a	1316	1330	HLA-DRB1*04:03	>0% - 50%	Dengue virus 2	ELISPOT	IFNg release	Positive	0	1	0	0	1	0
539959	TAIFLTTLSRTSKKR	NS2a	1317	1331	HLA-DRB1*14:02	>0% - 50%	Dengue virus 2	ELISPOT	IFNg release	Positive	0	1	0	0	1	0
540026	VAVSFVTLITGNMSF	NS2a	1163	1177	HLA-DRB1*10:01	>0% - 50%	Dengue virus 2	ELISPOT	IFNg release	Positive	0	1	1	0	1	0
540086	VSFVTLITGNMSFRD	NS2a	1165	1179	HLA-DRB1*10:01	>0% - 50%	Dengue virus 2	ELISPOT	IFNg release	Positive	0	1	1	0	1	0
866586	EKYQLAVTIMAISCV	NS2a	1268	1282	HLA-DQA1*01:02/DQB1*06:02	>0% - 50%	Dengue virus 2	ELISPOT	IFNg release	Positive	0	0	0	0	0	0
869200	TKHAILLVAVSFVTL	NS2a	1156	1170	HLA-DPB1*04:01	>0% - 50%	Dengue virus 2	ELISPOT	IFNg release	Positive	0	1	1	0	1	0
739148	IDKKGKVVGLYGNGV	NS3	1615	1629	HLA-DRB1*01:02	>0% - 50%	Dengue virus 2	ELISPOT	IFNg release	Positive	0	1	1	0	1	0
195356	GGIFLFLMSGRGIGK	NS4a	2159	2173	HLA-DRB1*11:01	>0% - 50%	Dengue virus 2	ELISPOT	IFNg release	Positive	0	1	1	0	1	0
195397	GLLFSIMKNTTSTRR	NS4b	2477	2491	HLA-DRB1*04:01	>0% - 50%	Dengue virus 2	ELISPOT	IFNg release	Positive	0	0	0	0	0	0
195718	LTAALFLLVAHYAII	NS4b	2350	2364	HLA-DRB1*07:01	>0% - 50%	Dengue virus 2	ELISPOT	IFNg release	Positive	0	0	1	0	1	0
867335	ITLTAALLLLIAHYA	NS4b	2348	2362	HLA-DPB1*04:02	>0% - 50%	Dengue virus 2	ELISPOT	IFNg release	Positive	0	0	0	0	0	0
195640	LGYILRDVSKKEGGA	NS5	2992	3006	HLA-DRB1*03:01	>0% - 50%	Dengue virus 2	ELISPOT	IFNg release	Positive	0	1	1	0	1	0
195642	LHKLGYILRDVSKKE	NS5	3003	3017	HLA-DRB1*08:02	>0% - 50%	Dengue virus 2	ELISPOT	IFNg release	Positive	0	1	1	0	1	0
195750	MEGEGIFKSIQHLTA	NS5	3114	3128	HLA-DRB1*11:01	>0% - 50%	Dengue virus 2	ELISPOT	IFNg release	Positive	0	0	0	0	0	0
195759	MLINRFTMKHKKATY	NS5	2729	2743	HLA-DRB1*11:01	>0% - 50%	Dengue virus 2	ELISPOT	IFNg release	Positive	0	0	0	0	0	0
195800	NMISRMLINRFTMKH	NS5	2724	2738	HLA-DRB1*08:02	>0% - 50%	Dengue virus 2	ELISPOT	IFNg release	Positive	0	0	0	0	0	0

32943	KPWDVVPMV	NS5	2821	2829	HLA class II	>0% - 50%	Dengue virus 2	ELISA	IL-2 release	Positive	0	1	1	0	1	0
539603	LNTFTNMEAQLIRQM	NS5	3100	3114	HLA-DRB1*10:01	>0% - 50%	Dengue virus 2	ELISPOT	IFNg release	Positive	0	1	1	0	1	0
539668	MISRMLINRFTMKHK	NS5	2725	2739	HLA-DRB1*14:04	>0% - 50%	Dengue virus 2	ELISPOT	IFNg release	Positive	0	0	0	0	0	0
539932	SRMLINRFTMKHKKA	NS5	2727	2741	HLA-DRB1*08:03	>0% - 50%	Dengue virus 2	ELISPOT	IFNg release	Positive	0	0	0	0	0	0
540065	VNGVVRLLTKPWDVV	NS5	2812	2826	HLA-DRB1*12:02	>0% - 50%	Dengue virus 2	ELISPOT	IFNg release	Positive	0	1	1	0	1	0
540066	VNMISRMLINRFTMK	NS5	2723	2737	HLA-DRB1*12:02	>0% - 50%	Dengue virus 2	ELISPOT	IFNg release	Positive	0	0	0	0	0	0
866648	ETCVYNMMGKREKKL	NS5	2939	2953	HLA-DRB5*01:01	>0% - 50%	Dengue virus 2	ELISPOT	IFNg release	Positive	0	1	1	0	1	0

CD4 DENV-3 Epitopes

											DENV-3 constructs of vaccines (100% identity=1,green ; else 0, red)					
Epitope_ID	AA_Seq	Protein_Name	Start	End	Allele	Step	Serotype	Method/Technique	Assay Group	Qualitative Measure	TV003	TDEN	DPIV	Dengvaxia	DENVax	TVDV
195205	CLMMMLPATLAFHLT	C	104	118	HLA-DRB1*15:01	0%	Dengue virus 3	ELISPOT	IFNg release	Positive	1	1	1	0	0	0
195289	FCLMMMLPATLAFHL	C	103	117	HLA-DRB1*01:01	0%	Dengue virus 3	ELISPOT	IFNg release	Positive	0	0	0	0	0	0
195517	INMLKRVRNRVSTGP	C	13	27	HLA-DRB1*08:02	0%	Dengue virus 3	ELISPOT	IFNg release	Positive	0	0	0	0	0	0
195519	INMLKRVRNRVSTGT	C	13	27	HLA-DRB1*08:02	0%	Dengue virus 3	ELISPOT	IFNg release	Positive	0	0	0	0	0	0
195598	KTSLCLVMILPAALA	C	100	114	HLA-DRB1*01:01	0%	Dengue virus 3	ELISPOT	IFNg release	Positive	0	0	0	0	0	0
195618	LCLMMIMPAALAFHL	C	103	117	HLA-DRB1*01:01	0%	Dengue virus 3	ELISPOT	IFNg release	Positive	0	0	0	0	0	0
195803	NMLKRVRNRVSTGPQ	C	14	28	HLA-DRB1*11:01	0%	Dengue virus 3	ELISPOT	IFNg release	Positive	0	0	0	0	0	0
195966	SLCLMMIMPAALAFH	C	102	116	HLA-DRB1*08:02	0%	Dengue virus 3	ELISPOT	IFNg release	Positive	0	0	0	0	0	0
195967	SLCLMMMLPATLAFH	C	102	116	HLA-DRB1*08:02	0%	Dengue virus 3	ELISPOT	IFNg release	Positive	1	1	1	0	0	0
539447	IVNRRKKTSLCLMMM	C	94	108	HLA-DRB1*13:01	0%	Dengue virus 3	ELISPOT	IFNg release	Positive	0	0	0	0	0	0
539906	SIINRRKRTSLCLMM	C	93	107	HLA-DRB1*13:01	0%	Dengue virus 3	ELISPOT	IFNg release	Positive	0	0	0	0	0	0
540118	WGSFKKSGAVKVLRG	C	69	83	HLA-DRB1*09:01	0%	Dengue virus 3	ELISPOT	IFNg release	Positive	0	0	0	0	0	0
540119	WGTFKKLGAIKVLKG	C	69	83	HLA-DRB1*09:01	0%	Dengue virus 3	ELISPOT	IFNg release	Positive	0	0	0	0	0	0
540120	WGTFKKLGAIKVLRG	C	69	83	HLA-DRB1*09:01	0%	Dengue virus 3	ELISPOT	IFNg release	Positive	0	0	0	0	0	0
540122	WGTFKKSGAIKVLKS	C	69	83	HLA-DRB1*09:01	0%	Dengue virus 3	ELISPOT	IFNg release	Positive	0	0	0	0	0	0
540123	WGTFKKSGAIKVLRG	C	69	83	HLA-DRB1*09:01	0%	Dengue virus 3	ELISPOT	IFNg release	Positive	1	0	1	0	0	0
540124	WGTFKKSGAIRVLRG	C	69	83	HLA-DRB1*09:01	0%	Dengue virus 3	ELISPOT	IFNg release	Positive	0	0	0	0	0	0
591709	LRGFKKEISNMLSII	C	81	95	HLA-DRB1*13:02	0%	Dengue virus 3	ELISPOT	IFNg release	Positive	1	0	1	0	0	0
591746	RGFKKEISNMLSIIN	C	82	96	HLA-DRB1*04:01	0%	Dengue virus 3	ELISPOT	IFNg release	Positive	1	0	1	0	0	0
867744	LKSFKKEISNMLSII	C	81	95	HLA-DRB3*02:02	0%	Dengue virus 3	ELISPOT	IFNg release	Positive	0	0	0	0	0	0
867909	LRGFKREISNMLSII	C	64	78	HLA-DRB3*02:02	0%	Dengue virus 3	ELISPOT	IFNg release	Positive	0	0	0	0	0	0
868733	RWGTFKKSGAIKVLR	C	48	62	HLA-DRB3*02:02	0%	Dengue virus 3	ELISPOT	IFNg release	Positive	1	0	1	0	0	0
195968	SLIQKVVIFILLMLV	PreM	261	275	HLA-DRB1*15:06	0%	Dengue virus 3	ELISPOT	IFNg release	Positive	0	0	0	0	0	0
196067	TQKVVIFVLLMLVTP	PreM	263	277	HLA-DRB1*15:06	0%	Dengue virus 3	ELISPOT	IFNg release	Positive	0	0	0	0	0	0
539529	KVVIFILLILVTPSM	PreM	265	279	HLA-DRB1*15:06	0%	Dengue virus 3	ELISPOT	IFNg release	Positive	0	1	0	0	0	0
539808	QKVVIFTLLMLVTPS	PreM	264	278	HLA-DRB1*15:06	0%	Dengue virus 3	ELISPOT	IFNg release	Positive	0	0	0	0	0	0
196170	VQHENLKYTVIITVH	E	130	144	HLA-DRB1*07:01	0%	Dengue virus 3	ELISPOT	IFNg release	Positive	1	1	1	1	1	1
539476	KKELLVTFKNAHAKK	E	511	525	HLA-DRB1*14:04	0%	Dengue virus 3	ELISPOT	IFNg release	Positive	1	0	1	0	0	0
539856	RKELLTTFKNAHAKK	E	511	525	HLA-DRB1*04:03	0%	Dengue virus 3	ELISPOT	IFNg release	Positive	0	0	0	0	0	0
591605	AFVLKKEVSETQHGT	E	303	317	HLA-DRB1*04:01	0%	Dengue virus 3	ELISPOT	IFNg release	Positive	1	0	1	0	0	0
591719	NEMILLTMKNRAWMV	E	474	488	HLA-DRB1*11:01	0%	Dengue virus 3	ELISPOT	IFNg release	Positive	0	0	0	0	0	0
867374	KAWIVHRQWFFDLPL	E	482	496	HLA-DQA1*01:01/DQB1*05:01	0%	Dengue virus 3	ELISPOT	IFNg release	Positive	0	0	0	0	0	0
869693	WMVHRQWLFDLPLPW	E	87	101	HLA-DQA1*01:01/DQB1*05:01	0%	Dengue virus 3	ELISPOT	IFNg release	Positive	0	0	0	0	0	0
539070	AKIVTAETRNSSFII	NS1	121	135	HLA-DRB1*13:01	0%	Dengue virus 3	ELISPOT	IFNg release	Positive	0	0	0	0	0	0
539457	KAKIVIAETQNSSFI	NS1	893	907	HLA-DRB1*13:01	0%	Dengue virus 3	ELISPOT	IFNg release	Positive	0	0	0	0	0	0
866698	FGVFTTNIWLKLRDV	NS1	933	947	HLA-DPB1*04:01	0%	Dengue virus 3	ELISPOT	IFNg release	Positive	0	0	0	0	0	0
195128	AGVLFMFVLLLSGQI	NS2a	1159	1173	HLA-DRB1*07:01	0%	Dengue virus 3	ELISPOT	IFNg release	Positive	0	0	0	0	0	0
195133	AHTFIMIGSNASDRM	NS2a	1179	1193	HLA-DRB1*04:01	0%	Dengue virus 3	ELISPOT	IFNg release	Positive	0	0	0	0	0	0
195166	ANTFIMIGSNASDRM	NS2a	1179	1193	HLA-DRB1*04:01	0%	Dengue virus 3	ELISPOT	IFNg release	Positive	0	0	0	0	0	0
195433	GVLFTFVLLLSGQIT	NS2a	1160	1174	HLA-DRB1*15:06	0%	Dengue virus 3	ELISPOT	IFNg release	Positive	1	0	1	0	0	0
195456	HMIAGILFMFVLLLS	NS2a	1156	1170	HLA-DRB1*11:01	0%	Dengue virus 3	ELISPOT	IFNg release	Positive	0	0	0	0	0	0
195630	LFTFVLLLSGQITWR	NS2a	1162	1176	HLA-DRB1*07:01	0%	Dengue virus 3	ELISPOT	IFNg release	Positive	1	0	1	0	0	0
195734	LVSLTCSNTILTLTV	NS2a	1274	1288	HLA-DRB1*07:01	0%	Dengue virus 3	ELISPOT	IFNg release	Positive	0	0	0	0	0	0
196123	VGVLFTFVLLLSGQI	NS2a	1159	1173	HLA-DRB1*07:01	0%	Dengue virus 3	ELISPOT	IFNg release	Positive	0	0	0	0	0	0
540048	VLFTFVLLLSGQITW	NS2a	1161	1175	HLA-DRB1*09:01	0%	Dengue virus 3	ELISPOT	IFNg release	Positive	1	0	1	0	0	0
865959	AGALFTFVLLLSGQI	NS2a	1159	1173	HLA-DPB1*04:02	0%	Dengue virus 3	ELISPOT	IFNg release	Positive	0	0	0	0	0	0
867702	LIATFKIQPFLTLGF	NS2a	1202	1216	HLA-DPB1*01:01	0%	Dengue virus 3	ELISPOT	IFNg release	Positive	0	0	0	0	0	0
869592	VQPFLALGFFLRKLT	NS2a	1208	1222	HLA-DPB1*01:01	0%	Dengue virus 3	ELISPOT	IFNg release	Positive	0	0	0	0	0	0
739173	IGLVSILASSLLRND	NS2b	1354	1368	HLA-DRB1*11:04	0%	Dengue virus 3	ELISPOT	IFNg release	Positive	0	0	0	0	0	0
195297	FHTMWHVTRGAVLTY	NS3	1519	1533	HLA-DRB1*07:01	0%	Dengue virus 3	ELISPOT	IFNg release	Positive	0	0	0	0	0	0
21420	GNEWITDFVGKTVWF	NS3	31	45	HLA-DRB1*15:01	0%	Dengue virus 3	ICS	IFNg release	Positive	0	1	0	0	0	0
49767	PTRVVAAEMEEAMKG	NS3	1697	1711	HLA-DRB1*15:01	0%	Dengue virus 3	ICS	IFNg release	Positive	0	0	0	0	0	0
539777	PKNFQTMPGIFQTTT	NS3	1579	1593	HLA-DRB1*04:03	0%	Dengue virus 3	ELISPOT	IFNg release	Positive	1	1	1	0	0	0
540037	VGKTVWFVPSIKAGN	NS3	1829	1843	HLA-DRB1*08:03	0%	Dengue virus 3	ELISPOT	IFNg release	Positive	0	1	0	0	0	0
72654	WITDFVGKTVW	NS3	1824	1834	HLA-DR15	0%	Dengue virus 3	51 chromium	cytotoxicity	Positive	0	1	0	0	0	0
867106	HTMWHVTRGAVLTYN	NS3	1520	1534	HLA-DQA1*05:01/DQB1*02:01	0%	Dengue virus 3	ELISPOT	IFNg release	Positive	0	0	0	0	0	0
869689	WHVTRGAVLTYNGKR	NS3	1523	1537	HLA-DQA1*05:01/DQB1*02:01	0%	Dengue virus 3	ELISPOT	IFNg release	Positive	0	0	0	0	0	0
195199	AYVVIGILTLAAIVA	NS4a	2227	2241	HLA-DRB1*01:01	0%	Dengue virus 3	ELISPOT	IFNg release	Positive	1	1	1	0	0	0
195742	MADVPLQWIASAIVL	NS4a	2189	2203	HLA-DRB1*07:01	0%	Dengue virus 3	ELISPOT	IFNg release	Positive	1	1	1	0	0	0
539900	SGMLWMADVPLQWIA	NS4a	2184	2198	HLA-DRB1*04:03	0%	Dengue virus 3	ELISPOT	IFNg release	Positive	1	1	1	0	0	0
739020	GIGKTSIGLICVIVS	NS4a	2169	2183	HLA-DRB1*11:04	0%	Dengue virus 3	ELISPOT	IFNg release	Positive	0	0	0	0	0	0
867986	LTLTAAVLLLATHYA	NS4b	2347	2361	HLA-DQA1*01:02/DQB1*06:02	0%	Dengue virus 3	ELISPOT	IFNg release	Positive	0	1	0	0	0	0
195408	GNIVASVNMVSRLLL	NS5	3	17	HLA-DRB1*03:01	0%	Dengue virus 3	ELISPOT	IFNg release	Positive	0	0	0	0	0	0
195780	NEDHWFSRDNSYSGV	NS5	2982	2996	HLA-DRB1*04:01	0%	Dengue virus 3	ELISPOT	IFNg release	Positive	0	0	0	0	0	0
196038	TGNIVASVNMVSRLL	NS5	2	16	HLA-DRB1*04:01	0%	Dengue virus 3	ELISPOT	IFNg release	Positive	0	0	0	0	0	0
196221	WLWRTLGRNKKPRLC	NS5	157	171	HLA-DRB1*14:02	0%	Dengue virus 3	ELISPOT	IFNg release	Positive	0	0	0	0	0	0
32944	KPWDVVPTV	NS5	2819	2827	HLA class II	0%	Dengue virus 3	ELISA	IL-2 release	Positive	0	0	0	0	0	0
539444	IVASVNMVSRLLLNR	NS5	2717	2731	HLA-DRB1*12:02	0%	Dengue virus 3	ELISPOT	IFNg release	Positive	0	0	0	0	0	0
539726	NIVASVNMVSRLLLN	NS5	2716	2730	HLA-DRB1*15:06	0%	Dengue virus 3	ELISPOT	IFNg release	Positive	0	0	0	0	0	0
539739	NMEVQLVRQMEGEGV	NS5	3103	3117	HLA-DRB1*13:01	0%	Dengue virus 3	ELISPOT	IFNg release	Positive	0	0	0	0	0	0
540025	VASVNMVSRLLLNRF	NS5	2718	2732	HLA-DRB1*14:04	0%	Dengue virus 3	ELISPOT	IFNg release	Positive	0	0	0	0	0	0
591602	ACLGKAYAQMWTLMY	NS5	3242	3256	HLA-DRB1*04:01	0%	Dengue virus 3	ELISPOT	IFNg release	Positive	1	0	1	0	0	0
738537	ASVNMVSRLLLNRFT	NS5	7	21	HLA-DRB1*14:06	0%	Dengue virus 3	ELISPOT	IFNg release	Positive	0	0	0	0	0	0
740771	YGLNTFTNMEVQLVR	NS5	3096	3110	HLA-DRB1*04:07	0%	Dengue virus 3	ELISPOT	IFNg release	Positive	0	0	0	0	0	0
195830	PGFTILALFLAHYIG	PreM	245	259	HLA-DRB1*15:06	100%	Dengue virus 3	ELISPOT	IFNg release	Positive	1	1	1	1	1	1
867082	HPGFTILALFLAHYI	PreM	244	258	HLA-DPB1*02:01	100%	Dengue virus 3	ELISPOT	IFNg release	Positive	1	1	1	1	1	1
119134	EGKVVQYENLKYTVI	E	126	140	HLA-DR2	100%	Dengue virus 3	ELISPOT	IFNg release	Positive	0	0	0	0	0	0
119289	NGRLITANPVVTKKE	E	626	640	HLA-DRB1*08:02	100%	Dengue virus 3	ELISPOT	IFNg release	Positive	1	1	1	1	1	1
13376	EMILLTMKNKAWMVH	E	473	487	HLA-DRB1*13:01	100%	Dengue virus 3	ELISPOT	IFNg release	Positive	1	1	1	1	0	1
195215	DFNEMILLTMKNKAW	E	470	484	HLA-DRB1*14:04	100%	Dengue virus 3	ELISPOT	IFNg release	Positive	1	1	1	1	0	1
195341	FVLKKEVSETQHGTI	E	584	598	HLA-DRB1*04:01	100%	Dengue virus 3	ELISPOT	IFNg release	Positive	1	1	1	1	1	1
195365	GHLKCRLKMDKLELK	E	559	573	HLA-DRB1*03:01	100%	Dengue virus 3	ELISPOT	IFNg release	Positive	1	1	1	1	1	0
196033	TFVLKKEVSETQHGT	E	583	597	HLA-DRB1*04:01	100%	Dengue virus 3	ELISPOT	IFNg release	Positive	0	1	0	1	0	1
196172	VQYENLKYTVIITVH	E	410	424	HLA-DRB1*07:01	100%	Dengue virus 3	ELISPOT	IFNg release	Positive	0	0	0	0	0	0
539400	IEGKVVQYENLKYTV	E	405	419	HLA-DRB1*15:02	100%	Dengue virus 3	ELISPOT	IFNg release	Positive	0	0	0	0	0	0
539710	NEMILLTMKNKAWMV	E	472	486	HLA-DRB1*04:03	100%	Dengue virus 3	ELISPOT	IFNg release	Positive	1	1	1	1	0	1
866574	EIQNSGGTSIFAGHL	E	547	561	HLA-DQA1*01:02/DQB1*06:02	100%	Dengue virus 3	ELISPOT	IFNg release	Positive	0	1	0	1	0	0
867377	KCRLKMDKLELKGMS	E	562	576	HLA-DRB3*02:02	100%	Dengue virus 3	ELISPOT	IFNg release	Positive	1	1	1	1	1	0
195164	ANELNYILWENNIKL	NS1	845	859	HLA-DRB1*03:01	100%	Dengue virus 3	ELISPOT	IFNg release	Positive	1	1	1	0	0	0
195692	LNYILWENNIKLTVV	NS1	848	862	HLA-DRB1*03:01	100%	Dengue virus 3	ELISPOT	IFNg release	Positive	1	1	1	0	0	0
195782	NELNYILWENNIKLT	NS1	846	860	HLA-DRB1*03:01	100%	Dengue virus 3	ELISPOT	IFNg release	Positive	1	1	1	0	0	0
866700	FGVFTTNIWLKLREV	NS1	933	947	HLA-DPB1*01:01	100%	Dengue virus 3	ELISPOT	IFNg release	Positive	0	0	0	0	0	0
867042	HADMGYWIESQKNGS	NS1	968	982	HLA-DQA1*01:01/DQB1*05:01	100%	Dengue virus 3	ELISPOT	IFNg release	Positive	1	1	1	0	0	0
195134	AHTLIMIGSNASDRM	NS2a	1179	1193	HLA-DRB1*04:01	100%	Dengue virus 3	ELISPOT	IFNg release	Positive	1	1	1	0	0	0
195197	AWRTATLILAGVSLL	NS2a	1289	1303	HLA-DRB1*01:01	100%	Dengue virus 3	ELISPOT	IFNg release	Positive	0	1	0	0	0	0
195222	DMAHTLIMIGSNASD	NS2a	1177	1191	HLA-DRB1*04:01	100%	Dengue virus 3	ELISPOT	IFNg release	Positive	1	1	1	0	0	0
195337	FTLTVAWRTATLILA	NS2a	1284	1298	HLA-DRB1*07:01	100%	Dengue virus 3	ELISPOT	IFNg release	Positive	1	1	1	0	0	0
195342	FVLLLSGQITWRDMA	NS2a	1165	1179	HLA-DRB1*01:01	100%	Dengue virus 3	ELISPOT	IFNg release	Positive	1	0	1	0	0	0
195462	HTLIMIGSNASDRMG	NS2a	1180	1194	HLA-DRB1*04:01	100%	Dengue virus 3	ELISPOT	IFNg release	Positive	1	1	1	0	0	0
195481	IFTLTVAWRTATLIL	NS2a	1283	1297	HLA-DRB1*07:01	100%	Dengue virus 3	ELISPOT	IFNg release	Positive	1	1	1	0	0	0
195609	LALGFFLRKLTSREN	NS2a	1212	1226	HLA-DRB1*11:01	100%	Dengue virus 3	ELISPOT	IFNg release	Positive	1	1	1	0	0	0
195644	LIMIGSNASDRMGMG	NS2a	1182	1196	HLA-DRB1*04:01	100%	Dengue virus 3	ELISPOT	IFNg release	Positive	1	1	1	0	0	0
195729	LTVAWRTATLILAGV	NS2a	1286	1300	HLA-DPB1*04:02	100%	Dengue virus 3	ELISPOT	IFNg release	Positive	0	1	0	0	0	0
195743	MAHTLIMIGSNASDR	NS2a	1178	1192	HLA-DRB1*04:01	100%	Dengue virus 3	ELISPOT	IFNg release	Positive	1	1	1	0	0	0
195757	MGVTYLALIATFKIQ	NS2a	1195	1209	HLA-DRB1*07:01	100%	Dengue virus 3	ELISPOT	IFNg release	Positive	1	1	1	0	0	0
195819	NTIFTLTVAWRTATL	NS2a	1281	1295	HLA-DRB1*07:01	100%	Dengue virus 3	ELISPOT	IFNg release	Positive	1	1	1	0	0	0
539075	ALGFFLRKLTSRENL	NS2a	1213	1227	HLA-DRB1*12:02	100%	Dengue virus 3	ELISPOT	IFNg release	Positive	1	0	1	0	0	0
539370	GVTYLALIATFKIQP	NS2a	1196	1210	HLA-DRB1*10:01	100%	Dengue virus 3	ELISPOT	IFNg release	Positive	1	1	1	0	0	0
539429	IQPFLALGFFLRKLT	NS2a	1208	1222	HLA-DRB1*15:06	100%	Dengue virus 3	ELISPOT	IFNg release	Positive	1	1	1	0	0	0
539568	LIATFKIQPFLALGF	NS2a	1202	1216	HLA-DRB1*15:06	100%	Dengue virus 3	ELISPOT	IFNg release	Positive	1	1	1	0	0	0
540093	VTYLALIATFKIQPF	NS2a	1197	1211	HLA-DRB1*04:03	100%	Dengue virus 3	ELISPOT	IFNg release	Positive	1	1	1	0	0	0
867551	LALIATFKIQPFLAL	NS2a	1200	1214	HLA-DPB1*04:01	100%	Dengue virus 3	ELISPOT	IFNg release	Positive	1	1	1	0	0	0
868303	PFLALGFFLRKLTSR	NS2a	1210	1224	HLA-DPB1*01:01	100%	Dengue virus 3	ELISPOT	IFNg release	Positive	1	1	1	0	0	0
868551	QPFLALGFFLRKLTS	NS2a	1209	1223	HLA-DPB1*02:01	100%	Dengue virus 3	ELISPOT	IFNg release	Positive	1	1	1	0	0	0
869256	TLTVAWRTATLILAG	NS2a	1285	1299	HLA-DPB1*04:02	100%	Dengue virus 3	ELISPOT	IFNg release	Positive	1	1	1	0	0	0
195403	GLVSILASSLLRNDV	NS2b	1355	1369	HLA-DRB1*01:01	100%	Dengue virus 3	ELISPOT	IFNg release	Positive	1	1	1	0	0	0
196152	VMAVGLVSILASSLL	NS2b	1351	1365	HLA-DRB1*07:01	100%	Dengue virus 3	ELISPOT	IFNg release	Positive	1	1	1	0	0	0
539100	AVGLVSILASSLLRN	NS2b	1353	1367	HLA-DRB1*10:01	100%	Dengue virus 3	ELISPOT	IFNg release	Positive	1	1	1	0	0	0
540087	VSGIFPYSIPATLLV	NS2b	1449	1463	HLA-DRB1*09:01	100%	Dengue virus 3	ELISPOT	IFNg release	Positive	1	1	1	0	0	0
1067120	GKVVGLYGNGVVTKN	NS3	1617	1631	HLA-DRB1*15:01	100%	Dengue virus 3	ELISPOT	IFNg release	Positive	0	1	0	0	0	0
150617	TFTMRLLSPVRVPNY	NS3	1738	1752	HLA-DRB1*01:01	100%	Dengue virus 3	ELISPOT	IFNg release	Positive	1	1	1	0	0	0
190307	REIVDLMCHATF	NS3	255	266	HLA-DPA1*01:03/DPB1*02:01	100%	Dengue virus 3	ELISA	IL-2 release	Positive	1	1	1	0	0	0
190710	ANCLRKNGKKVIQLS	NS3	1846	1860	HLA-DRB1*13:01	100%	Dengue virus 3	ELISPOT	IFNg release	Positive	1	1	1	0	0	0
190774	GKTVWFVPSIKAGND	NS3	357	371	HLA-DR2	100%	Dengue virus 3	ELISPOT	IFNg release	Positive	1	1	1	0	0	0
195243	EGKVVGLYGNGVVTK	NS3	5	19	HLA-DRB1*15:01	100%	Dengue virus 3	ELISPOT	IFNg release	Positive	0	1	0	0	0	0
195430	GVFHTMWHVTRGAVL	NS3	1517	1531	HLA-DRB1*07:01	100%	Dengue virus 3	ELISPOT	IFNg release	Positive	1	1	1	0	0	0

28422	IRYQTTATK	NS3	241	249	HLA-DR15	100%	Dengue virus 3	51 chromium	cytotoxicity	Positive	1	1	1	0	0	0
45670	NREGKVVGLYGNGVV	NS3	141	155	HLA-DRB1*15:01	100%	Dengue virus 3	multimer/tetramer	qualitative binding	Positive	0	1	0	0	0	0
539052	AGKTVWFVPSIKAGN	NS3	1829	1843	HLA-DRB1*08:03	100%	Dengue virus 3	ELISPOT	IFNg release	Positive	1	0	1	0	0	0
539578	LKGLPIRYQTTATKS	NS3	1709	1723	HLA-DRB1*13:01	100%	Dengue virus 3	ELISPOT	IFNg release	Positive	1	1	1	0	0	0
539607	LPIRYQTTATKSEHT	NS3	1712	1726	HLA-DRB1*13:01	100%	Dengue virus 3	ELISPOT	IFNg release	Positive	1	1	1	0	0	0
539844	REGKVVGLYGNGVVT	NS3	1615	1629	HLA-DRB1*01:02	100%	Dengue virus 3	ELISPOT	IFNg release	Positive	0	1	0	0	0	0
539991	TMRLLSPVRVPNYNL	NS3	1740	1754	HLA-DRB1*13:01	100%	Dengue virus 3	ELISPOT	IFNg release	Positive	1	1	1	0	0	0
68015	VDLMCHATFT	NS3	258	267	HLA-DPw2	100%	Dengue virus 3	51 chromium	cytotoxicity	Positive	1	1	1	0	0	0
866771	FQTTTGEIGAIALDF	NS3	1589	1603	HLA-DQA1*03:01/DQB1*03:02	100%	Dengue virus 3	ELISPOT	IFNg release	Positive	1	1	1	0	0	0
869278	TMWHVTRGAVLTHNG	NS3	1521	1535	HLA-DQA1*05:01/DQB1*02:01	100%	Dengue virus 3	ELISPOT	IFNg release	Positive	1	1	1	0	0	0
167755	IALDLVTEIGRVPSHLAHRT	NS4a	2	21	HLA class II	100%	Dengue virus 3	ELISPOT	IFNg release	Positive	1	1	1	0	0	0
195346	GAGLAFSIMKSVGTG	NS4b	2474	2488	HLA-DRB1*04:01	100%	Dengue virus 3	ELISPOT	IFNg release	Positive	1	1	1	0	0	0
195607	LAFSIMKSVGTGKRG	NS4b	2477	2491	HLA-DRB1*04:01	100%	Dengue virus 3	ELISPOT	IFNg release	Positive	1	1	1	0	0	0
195617	LCAVQLLLMRTSWAL	NS4b	2418	2432	HLA-DRB1*15:01	100%	Dengue virus 3	ELISPOT	IFNg release	Positive	0	1	0	0	0	0
195624	LDPVIYDSKFEKQLG	NS4b	2397	2411	HLA-DRB1*03:01	100%	Dengue virus 3	ELISPOT	IFNg release	Positive	1	1	1	0	0	0
195664	LLLMRTSWALCEALT	NS4b	2423	2437	HLA-DRB1*04:01	100%	Dengue virus 3	ELISPOT	IFNg release	Positive	0	1	0	0	0	0
195871	QVMLLVLCAVQLLLM	NS4b	2412	2426	HLA-DRB1*01:01	100%	Dengue virus 3	ELISPOT	IFNg release	Positive	1	1	1	0	0	0
196137	VLCAVQLLLMRTSWA	NS4b	2417	2431	HLA-DRB1*12:02	100%	Dengue virus 3	ELISPOT	IFNg release	Positive	1	1	1	0	0	0
196155	VMLLVLCAVQLLLMR	NS4b	2413	2427	HLA-DRB1*03:01	100%	Dengue virus 3	ELISPOT	IFNg release	Positive	1	1	1	0	0	0
738453	AGLAFSIMKSVGTGK	NS4b	2475	2489	HLA-DRB1*04:07	100%	Dengue virus 3	ELISPOT	IFNg release	Positive	1	1	1	0	0	0
868878	SLAAIANQAVVLMGL	NS4b	2306	2320	HLA-DQA1*01:02/DQB1*06:02	100%	Dengue virus 3	ELISPOT	IFNg release	Positive	1	1	1	0	0	0
167769	KKLNQLSRKEFDL	NS5	15	27	HLA-DP	100%	Dengue virus 3	ELISPOT	IFNg release	Positive	0	1	0	0	0	0
195136	AIFKLTYQNKVVKVQ	NS5	3056	3070	HLA-DRB1*09:01	100%	Dengue virus 3	ELISPOT	IFNg release	Positive	1	1	1	0	0	0
195204	CLGKAYAQMWSLMYF	NS5	3243	3257	HLA-DRB1*04:01	100%	Dengue virus 3	ELISPOT	IFNg release	Positive	0	1	0	0	0	0
195273	ETKGVERLKRMAISG	NS5	3138	3152	HLA-DRB1*11:01	100%	Dengue virus 3	ELISPOT	IFNg release	Positive	1	1	1	0	0	0
195437	GYILRDISKIPGGAM	NS5	43	57	HLA-DRB1*08:02	100%	Dengue virus 3	ELISPOT	IFNg release	Positive	1	1	1	0	0	0
195568	KHGGMLVRNPLSRNS	NS5	2689	2703	HLA-DRB1*08:02	100%	Dengue virus 3	ELISPOT	IFNg release	Positive	1	1	1	0	0	0
195641	LHKLGYILRDISKIP	NS5	39	53	HLA-DRB1*03:01	100%	Dengue virus 3	ELISPOT	IFNg release	Positive	1	1	1	0	0	0
196222	WNIVKLMSGKDVFYL	NS5	2611	2625	HLA-DRB1*15:01	100%	Dengue virus 3	ELISPOT	IFNg release	Positive	1	1	1	0	0	0
196224	WSLMYFHRRDLRLAS	NS5	3252	3266	HLA-DRB1*03:01	100%	Dengue virus 3	ELISPOT	IFNg release	Positive	0	1	0	0	0	0
539600	LMYFHRRDLRLASNA	NS5	3254	3268	HLA-DRB1*13:01	100%	Dengue virus 3	ELISPOT	IFNg release	Positive	1	1	1	0	0	0
539604	LNTFTNMEAQLVRQM	NS5	3098	3112	HLA-DRB1*10:01	100%	Dengue virus 3	ELISPOT	IFNg release	Positive	0	1	0	0	0	0
539704	NAIFKLTYQNKVVKV	NS5	3055	3069	HLA-DRB1*09:01	100%	Dengue virus 3	ELISPOT	IFNg release	Positive	1	1	1	0	0	0
539872	RRDLRLASNAICSAV	NS5	3259	3273	HLA-DRB1*04:03	100%	Dengue virus 3	ELISPOT	IFNg release	Positive	1	1	1	0	0	0
539916	SLMYFHRRDLRLASN	NS5	3253	3267	HLA-DRB1*13:01	100%	Dengue virus 3	ELISPOT	IFNg release	Positive	0	1	0	0	0	0
540019	TYGLNTFTNMEAQLV	NS5	3095	3109	HLA-DRB1*04:03	100%	Dengue virus 3	ELISPOT	IFNg release	Positive	0	1	0	0	0	0
540152	YKTWAYHGSYEVKAT	NS5	2789	2803	HLA-DRB1*09:01	100%	Dengue virus 3	ELISPOT	IFNg release	Positive	1	1	1	0	0	0
738829	ERELHKLGKCGSCVY	NS5	2927	2941	HLA-DRB1*11:04	100%	Dengue virus 3	ELISPOT	IFNg release	Positive	1	1	1	0	0	0
867376	KAYAQMWSLMYFHRR	NS5	3246	3260	HLA-DRB5*01:01	100%	Dengue virus 3	ELISPOT	IFNg release	Positive	0	1	0	0	0	0
869489	VKPIDDRFANALLAL	NS5	3157	3171	HLA-DPB1*01:01	100%	Dengue virus 3	ELISPOT	IFNg release	Positive	1	1	1	0	0	0
1068391	TGKPSINMLKRVRNR	C	8	22	HLA-DRB1*11:01	81% - <100%	Dengue virus 3	ELISPOT	IFNg release	Positive-Low	1	1	1	0	0	0
195303	FKKEISNMLSIINKR	C	37	51	HLA-DRB1*04:01	81% - <100%	Dengue virus 3	ELISPOT	IFNg release	Positive	0	1	0	0	0	0
195306	FLRFLAIPPTAGVLA	C	6	20	HLA-DRB1*01:01	81% - <100%	Dengue virus 3	ELISPOT	IFNg release	Positive	1	1	1	0	0	0
195469	IAFLRFLAIPPTAGV	C	4	18	HLA-DRB1*04:01	81% - <100%	Dengue virus 3	ELISPOT	IFNg release	Positive	1	1	1	0	0	0
195518	INMLKRVRNRVSTGS	C	13	27	HLA-DRB1*08:02	81% - <100%	Dengue virus 3	ELISPOT	IFNg release	Positive	1	1	1	0	0	0
195536	ISNMLSIINKRKKTS	C	41	55	HLA-DRB1*11:01	81% - <100%	Dengue virus 3	ELISPOT	IFNg release	Positive	0	1	0	0	0	0
195559	KEISNMLSIINKRKK	C	39	53	HLA-DRB1*04:01	81% - <100%	Dengue virus 3	ELISPOT	IFNg release	Positive	0	1	0	0	0	0
195611	LARWGTFKKSGAIKV	C	19	33	HLA-DRB1*07:01	81% - <100%	Dengue virus 3	ELISPOT	IFNg release	Positive	1	1	1	0	0	0
195649	LKGFKKEISNMLSII	C	34	48	HLA-DRB1*07:01	81% - <100%	Dengue virus 3	ELISPOT	IFNg release	Positive	0	1	0	0	0	0
195961	SINMLKRVRNRVSTG	C	12	26	HLA-DRB1*08:02	81% - <100%	Dengue virus 3	ELISPOT	IFNg release	Positive	1	1	1	0	0	0
36858	LKGFKKEISNML	C	81	92	HLA-DPw4	81% - <100%	Dengue virus 3	ICS	IFNg release	Positive	0	1	0	0	0	0
539502	KPSINMLKRVRNRVS	C	10	24	HLA-DRB1*08:03	81% - <100%	Dengue virus 3	ELISPOT	IFNg release	Positive	1	1	1	0	0	0
539787	PSINMLKRVRNRVST	C	11	25	HLA-DRB1*14:04	81% - <100%	Dengue virus 3	ELISPOT	IFNg release	Positive	1	1	1	0	0	0
539926	SNMLSIINKRKKTSL	C	42	56	HLA-DRB1*12:02	81% - <100%	Dengue virus 3	ELISPOT	IFNg release	Positive	0	1	0	0	0	0
540121	WGTFKKSGAIKVLKG	C	22	36	HLA-DRB1*09:01	81% - <100%	Dengue virus 3	ELISPOT	IFNg release	Positive	0	1	0	0	0	0
738443	AFLRFLAIPPTAGVL	C	52	66	HLA-DRB1*04:07	81% - <100%	Dengue virus 3	ELISPOT	IFNg release	Positive	1	1	1	0	0	0
740207	RWGTFKKSGAIKVLK	C	68	82	HLA-DRB1*14:02	81% - <100%	Dengue virus 3	ELISPOT	IFNg release	Positive	0	1	0	0	0	0
195602	KVVIFILLMLVTPSM	PreM	265	279	HLA-DRB1*01:01	81% - <100%	Dengue virus 3	ELISPOT	IFNg release	Positive	1	0	1	1	1	1
195726	LTQKVVIFILLMLVT	PreM	262	276	HLA-DRB1*11:01	81% - <100%	Dengue virus 3	ELISPOT	IFNg release	Positive	1	0	1	1	1	1
196203	VVIFILLMLVTPSMT	PreM	266	280	HLA-DRB1*07:01	81% - <100%	Dengue virus 3	ELISPOT	IFNg release	Positive	1	0	1	0	1	1
540004	TSLTQKVVIFILLML	PreM	260	274	HLA-DPB1*04:02	81% - <100%	Dengue virus 3	ELISPOT	IFNg release	Positive	1	0	1	1	1	1
869302	TQKVVIFILLMLVTP	PreM	263	277	HLA-DPB1*04:02	81% - <100%	Dengue virus 3	ELISPOT	IFNg release	Positive	1	0	1	1	1	1
119327	RKELLVTFKNAHAKK	E	231	245	HLA-DR2	81% - <100%	Dengue virus 3	ELISPOT	IFNg release	Positive	0	1	0	1	1	1
195252	ELLVTFKNAHAKKQE	E	513	527	HLA-DRB1*04:01	81% - <100%	Dengue virus 3	ELISPOT	IFNg release	Positive	1	1	1	1	1	1
195416	GRLITANPVVTKKEE	E	627	641	HLA-DRB1*08:02	81% - <100%	Dengue virus 3	ELISPOT	IFNg release	Positive	1	1	1	1	1	1
195813	NRKELLVTFKNAHAK	E	510	524	HLA-DRB1*04:01	81% - <100%	Dengue virus 3	ELISPOT	IFNg release	Positive	0	1	0	1	1	1
539460	KELLVTFKNAHAKKQ	E	512	526	HLA-DRB1*08:03	81% - <100%	Dengue virus 3	ELISPOT	IFNg release	Positive	1	1	1	1	1	1
539559	LGKMVHQIFGSAYTA	E	710	724	HLA-DRB1*04:03	81% - <100%	Dengue virus 3	ELISPOT	IFNg release	Positive	1	1	1	1	1	1
739011	GGVLNSLGKMVHQIF	E	704	718	HLA-DRB1*11:04	81% - <100%	Dengue virus 3	ELISPOT	IFNg release	Positive	1	1	1	1	1	1
739041	GMSYAMCTNTFVLKK	E	574	588	HLA-DRB1*04:07	81% - <100%	Dengue virus 3	ELISPOT	IFNg release	Positive	0	0	0	0	0	0
739344	KMVHQIFGSAYTALF	E	31	45	HLA-DRB3*02:02	81% - <100%	Dengue virus 3	ELISPOT	IFNg release	Positive	1	1	1	1	1	1
740409	SVGGVLNSLGKMVHQ	E	702	716	HLA-DRB1*11:04	81% - <100%	Dengue virus 3	ELISPOT	IFNg release	Positive	1	1	1	1	1	1
741746	MRCVGVGNRDFVEGLSGATW	E	281	300	HLA class II	81% - <100%	Dengue virus 3	ELISPOT	IFNg release	Positive	1	1	1	1	1	1
868175	MVHRQWFFDLPLPWT	E	485	499	HLA-DQA1*01:01/DQB1*05:01	81% - <100%	Dengue virus 3	ELISPOT	IFNg release	Positive	1	1	1	1	1	1
190705	AKIVTAETQNSSFII	NS1	894	908	HLA-DRB1*04:01	81% - <100%	Dengue virus 3	ELISPOT	IFNg release	Positive	1	1	1	0	0	0
190996	TEQYKFQADSPKRLA	NS1	802	816	HLA-DRB5*01:01	81% - <100%	Dengue virus 3	ELISPOT	IFNg release	Positive	1	1	1	0	0	0
195419	GSWKLEKASLIEVKT	NS1	981	995	HLA-DRB1*01:01	81% - <100%	Dengue virus 3	ELISPOT	IFNg release	Positive	1	1	1	0	0	0
195577	KLTVVVGDTIGVLEQ	NS1	858	872	HLA-DRB1*03:01	81% - <100%	Dengue virus 3	ELISPOT	IFNg release	Positive	0	0	0	0	0	0
196235	YKFQADSPKRLATAI	NS1	805	819	HLA-DRB1*03:01	81% - <100%	Dengue virus 3	ELISPOT	IFNg release	Positive	1	1	1	0	0	0
539717	NGSWKLEKASLIEVK	NS1	980	994	HLA-DRB1*09:01	81% - <100%	Dengue virus 3	ELISPOT	IFNg release	Positive	1	1	1	0	0	0
539825	QYKFQADSPKRLATA	NS1	804	818	HLA-DRB3*02:02	81% - <100%	Dengue virus 3	ELISPOT	IFNg release	Positive	1	1	1	0	0	0
167810	RENLLLGVGLAMATTLQLPE	NS2a	1224	1243	HLA-DP	81% - <100%	Dengue virus 3	ELISPOT	IFNg release	Positive	1	0	1	0	0	0
195632	LGFFLRKLTSRENLL	NS2a	1214	1228	HLA-DRB1*11:01	81% - <100%	Dengue virus 3	ELISPOT	IFNg release	Positive	1	0	1	0	0	0
195645	LISLTCSNTIFTLTV	NS2a	1274	1288	HLA-DRB1*07:01	81% - <100%	Dengue virus 3	ELISPOT	IFNg release	Positive	0	0	0	0	0	0
195836	PLFIFSLKDTLKRRS	NS2a	1330	1344	HLA-DRB1*11:01	81% - <100%	Dengue virus 3	ELISPOT	IFNg release	Positive	1	1	1	0	0	0
195912	RTATLILAGVSLLPV	NS2a	1291	1305	HLA-DRB1*03:01	81% - <100%	Dengue virus 3	ELISPOT	IFNg release	Positive	0	1	0	0	0	0
867442	KLITQFETYQLWTAL	NS2a	1260	1274	HLA-DPB1*02:01	81% - <100%	Dengue virus 3	ELISPOT	IFNg release	Positive	1	1	1	0	0	0
868046	MALKLITQFETYQLW	NS2a	1257	1271	HLA-DPB1*01:01	81% - <100%	Dengue virus 3	ELISPOT	IFNg release	Positive	1	1	1	0	0	0
868329	PLPLFIFSLKDTLKR	NS2a	1328	1342	HLA-DPB1*01:01	81% - <100%	Dengue virus 3	ELISPOT	IFNg release	Positive	1	1	1	0	0	0
869298	TQFETYQLWTALISL	NS2a	1263	1277	HLA-DPB1*04:02	81% - <100%	Dengue virus 3	ELISPOT	IFNg release	Positive	0	0	0	0	0	0
195180	ASSLLRNDVPMAGPL	NS2b	1361	1375	HLA-DRB1*03:01	81% - <100%	Dengue virus 3	ELISPOT	IFNg release	Positive	1	1	1	0	0	0
195789	NILTVLLKTALLIVS	NS2b	1436	1450	HLA-DRB1*01:01	81% - <100%	Dengue virus 3	ELISPOT	IFNg release	Positive	1	1	1	0	0	0
195994	SSLLRNDVPMAGPLV	NS2b	1362	1376	HLA-DRB1*03:01	81% - <100%	Dengue virus 3	ELISPOT	IFNg release	Positive	1	1	1	0	0	0
150544	RRLRTLILAPTRVVA	NS3	1688	1702	HLA-DRB1*01:01	81% - <100%	Dengue virus 3	ELISPOT	IFNg release	Positive	1	1	1	0	0	0
190732	EAAAIFMTATPPGTA	NS3	1782	1796	HLA-DRB1*04:03	81% - <100%	Dengue virus 3	ELISPOT	IFNg release	Positive	1	1	1	0	0	0
195898	RLRTLILAPTRVVAA	NS3	1689	1703	HLA-DRB1*04:03	81% - <100%	Dengue virus 3	ELISPOT	IFNg release	Positive	1	1	1	0	0	0
30395	KEGEKKKLRPRWLDA	NS3	585	599	HLA class II	81% - <100%	Dengue virus 3	ICS	IFNg release	Positive	1	1	1	0	0	0
39	AAAIFMTATPPGTAD	NS3	1783	1797	HLA-DRB1*04:03	81% - <100%	Dengue virus 3	ELISPOT	IFNg release	Positive	1	1	1	0	0	0
539028	AAIFMTATPPGTADA	NS3	1784	1798	HLA-DRB1*10:01	81% - <100%	Dengue virus 3	ELISPOT	IFNg release	Positive	1	1	1	0	0	0
539838	REAIKRRLRTLILAP	NS3	1683	1697	HLA-DRB1*14:04	81% - <100%	Dengue virus 3	ELISPOT	IFNg release	Positive	1	1	1	0	0	0
540039	VGLYGNGVVTKNGGY	NS3	9	23	HLA-DRB1*15:06	81% - <100%	Dengue virus 3	ELISPOT	IFNg release	Positive	0	1	0	0	0	0
55024	RNLTIMDLHPGSGKT	NS3	187	201	HLA class II	81% - <100%	Dengue virus 3	ICS	IFNg release	Positive	1	1	1	0	0	0
66127	TRVVAAEMEEA	NS3	225	235	HLA-DR15	81% - <100%	Dengue virus 3	51 chromium	cytotoxicity	Positive	1	1	1	0	0	0
867875	LNDWDFVVTTDISEM	NS3	1873	1887	HLA-DQA1*03:01/DQB1*03:02	81% - <100%	Dengue virus 3	ELISPOT	IFNg release	Positive	1	1	1	0	0	0
195522	IPLQWIASAIVLEFF	NS4a	2192	2206	HLA-DRB1*01:01	81% - <100%	Dengue virus 3	ELISPOT	IFNg release	Positive	0	0	0	0	0	0
195828	PETMETLLLLGLMIL	NS4a	2141	2155	HLA-DRB1*15:06	81% - <100%	Dengue virus 3	ELISPOT	IFNg release	Positive	1	1	1	0	0	0
196056	TLLLLGLMILLTGGA	NS4a	2146	2160	HLA-DRB1*15:06	81% - <100%	Dengue virus 3	ELISPOT	IFNg release	Positive	1	1	1	0	0	0
739464	LFLISGKGIGKTSIG	NS4a	2162	2176	HLA-DRB1*01:02	81% - <100%	Dengue virus 3	ELISPOT	IFNg release	Positive	1	1	1	0	0	0
167753	HVNAEPETPNMDVIGERIKR	NS5	263	282	HLA class II	81% - <100%	Dengue virus 3	ELISPOT	IFNg release	Positive	0	1	0	0	0	0
195382	GKVRKDIPQWQPSKG	NS5	3175	3189	HLA-DRB1*03:01	81% - <100%	Dengue virus 3	ELISPOT	IFNg release	Positive	1	1	1	0	0	0
195668	LLNRFTMTHRRPTIE	NS5	16	30	HLA-DRB1*11:01	81% - <100%	Dengue virus 3	ELISPOT	IFNg release	Positive	1	1	1	0	0	0
195779	NDMGKVRKDIPQWQP	NS5	3172	3186	HLA-DRB1*03:01	81% - <100%	Dengue virus 3	ELISPOT	IFNg release	Positive	1	1	1	0	0	0
195812	NRFTMTHRRPTIEKD	NS5	18	32	HLA-DRB1*11:01	81% - <100%	Dengue virus 3	ELISPOT	IFNg release	Positive	1	1	1	0	0	0
195875	RAIWYMWLGARYLEF	NS5	2961	2975	HLA-DRB1*01:01	81% - <100%	Dengue virus 3	ELISPOT	IFNg release	Positive	1	1	1	0	0	0
195939	SCVYNMMGKREKKLG	NS5	2938	2952	HLA-DRB1*11:01	81% - <100%	Dengue virus 3	ELISPOT	IFNg release	Positive	1	1	1	0	0	0
231420	RYLEFEALGFLNEDH	NS5	2971	2985	HLA-DPB1*04:01	81% - <100%	Dengue virus 3	ELISPOT	IFNg release	Positive	1	0	1	0	0	0
539458	KCGSCVYNMMGKREK	NS5	2935	2949	HLA-DRB1*13:01	81% - <100%	Dengue virus 3	ELISPOT	IFNg release	Positive	1	1	1	0	0	0
539831	RATWAQNIPTAIQQV	NS5	3346	3360	HLA-DRB1*09:01	81% - <100%	Dengue virus 3	ELISPOT	IFNg release	Positive	0	0	0	0	0	0
738753	EEFLDYMPSMKRFRK	NS5	3367	3381	HLA-DRB1*14:02	81% - <100%	Dengue virus 3	ELISPOT	IFNg release	Positive	1	0	1	0	0	0
738759	EFLDYMPSMKRFRKE	NS5	3368	3382	HLA-DRB1*14:06	81% - <100%	Dengue virus 3	ELISPOT	IFNg release	Positive	1	0	1	0	0	0
857703	IWYMWLGARYLEFEA	NS5	2963	2977	HLA-DPB1*04:01	81% - <100%	Dengue virus 3	ELISPOT	IFNg release	Positive	1	1	1	0	0	0
867015	GSCVYNMMGKREKKL	NS5	2937	2951	HLA-DRB5*01:01	81% - <100%	Dengue virus 3	ELISPOT	IFNg release	Positive	1	1	1	0	0	0
868338	PMSTYGWNIVKLMSG	NS5	2605	2619	HLA-DQA1*01:01/DQB1*05:01	81% - <100%	Dengue virus 3	ELISPOT	IFNg release	Positive	1	1	1	0	0	0
869705	WYMWLGARYLEFEAL	NS5	2964	2978	HLA-DQA1*05:01/DQB1*02:01	81% - <100%	Dengue virus 3	ELISPOT	IFNg release	Positive	1	1	1	0	0	0
195497	IKVLKGFKKEISNML	C	31	45	HLA-DRB1*04:01	51%-80%	Dengue virus 3	ELISPOT	IFNg release	Positive	0	1	0	0	0	0
195597	KTSLCLMMILPAALA	C	53	67	HLA-DRB1*01:01	51%-80%	Dengue virus 3	ELISPOT	IFNg release	Positive	0	0	0	0	0	0
539277	GAIKVLKGFKKEISN	C	29	43	HLA-DRB1*12:02	51%-80%	Dengue virus 3	ELISPOT	IFNg release	Positive	0	1	0	0	0	0
539512	KSGAIKVLKGFKKEI	C	27	41	HLA-DRB1*12:02	51%-80%	Dengue virus 3	ELISPOT	IFNg release	Positive	0	1	0	0	0	0
539897	SGAIKVLKGFKKEIS	C	28	42	HLA-DRB1*14:04	51%-80%	Dengue virus 3	ELISPOT	IFNg release	Positive	0	1	0	0	0	0
190825	KQIANELNYILWENN	NS1	69	83	HLA-DR2	51%-80%	Dengue virus 3	ELISPOT	IFNg release	Positive	1	1	1	0	0	0
195123	AGVFFTFVLLLSGQI	NS2a	1159	1173	HLA-DRB1*07:01	51%-80%	Dengue virus 3	ELISPOT	IFNg release	Positive	0	0	0	0	0	0

195335	FTFVLLLSGQITWRD	NS2a	1163	1177	HLA-DRB1*15:02	51%-80%	Dengue virus 3	ELISPOT	IFNg release	Positive	1	0	1	0	0	0
195429	GVFFTFVLLLSGQIT	NS2a	1160	1174	HLA-DRB1*04:01	51%-80%	Dengue virus 3	ELISPOT	IFNg release	Positive	0	0	0	0	0	0
195457	HMIAGVFFTFVLLLS	NS2a	1156	1170	HLA-DRB1*11:01	51%-80%	Dengue virus 3	ELISPOT	IFNg release	Positive	0	0	0	0	0	0
867116	IAGVFFTFVLLLSGQ	NS2a	1158	1172	HLA-DPB1*01:01	51%-80%	Dengue virus 3	ELISPOT	IFNg release	Positive	0	0	0	0	0	0
167823	TMRIKDDETENILTVLLKTA	NS2b	83	102	HLA-DRB1*15:01	51%-80%	Dengue virus 3	ELISPOT	IFNg release	Positive	1	1	1	0	0	0
195144	AIVREAIKRRLRTLI	NS3	1680	1694	HLA-DRB1*08:02	51%-80%	Dengue virus 3	ELISPOT	IFNg release	Positive	1	1	1	0	0	0
38401	LPAIVREAIKRRLRT	NS3	1678	1692	HLA-DRB3*02:02	51%-80%	Dengue virus 3	ELISPOT	IFNg release	Positive	1	1	1	0	0	0
539714	NGKKVIQLSRKTFDT	NS3	1852	1866	HLA-DRB1*12:02	51%-80%	Dengue virus 3	ELISPOT	IFNg release	Positive	1	1	1	0	0	0
54455	RKYLPAIVRE	NS3	202	211	HLA-DR15	51%-80%	Dengue virus 3	51 chromium	cytotoxicity	Positive	1	1	1	0	0	0
70708	VREAIKRRLRTLILA	NS3	1682	1696	HLA-DRB1*12:02	51%-80%	Dengue virus 3	ELISPOT	IFNg release	Positive	1	1	1	0	0	0
867358	IVREAIKRRLRTLIL	NS3	1681	1695	HLA-DRB3*02:02	51%-80%	Dengue virus 3	ELISPOT	IFNg release	Positive	1	1	1	0	0	0
867534	KYLPAIVREAIKRRL	NS3	1676	1690	HLA-DRB4*01:01	51%-80%	Dengue virus 3	ELISPOT	IFNg release	Positive	1	1	1	0	0	0
195807	NPLTLTAAVLLLITH	NS4b	2345	2359	HLA-DRB1*07:01	51%-80%	Dengue virus 3	ELISPOT	IFNg release	Positive	0	0	0	0	0	0
196163	VNPLTLTAAVLLLIT	NS4b	2344	2358	HLA-DRB1*01:01	51%-80%	Dengue virus 3	ELISPOT	IFNg release	Positive	0	0	0	0	0	0
195410	GNIVSSVNMVSRLLL	NS5	2715	2729	HLA-DRB1*03:01	51%-80%	Dengue virus 3	ELISPOT	IFNg release	Positive	1	1	1	0	0	0
195806	NMVSRLLLNRFTMTH	NS5	2722	2736	HLA-DRB1*08:02	51%-80%	Dengue virus 3	ELISPOT	IFNg release	Positive	1	1	1	0	0	0
196041	TGNIVSSVNMVSRLL	NS5	2714	2728	HLA-DRB1*04:01	51%-80%	Dengue virus 3	ELISPOT	IFNg release	Positive	1	1	1	0	0	0
196162	VNMVSRLLLNRFTMT	NS5	9	23	HLA-DRB1*11:01	51%-80%	Dengue virus 3	ELISPOT	IFNg release	Positive	1	1	1	0	0	0
539449	IVSSVNMVSRLLLNR	NS5	2717	2731	HLA-DRB1*11:04	51%-80%	Dengue virus 3	ELISPOT	IFNg release	Positive	1	1	1	0	0	0
539945	SVNMVSRLLLNRFTM	NS5	2720	2734	HLA-DRB1*12:02	51%-80%	Dengue virus 3	ELISPOT	IFNg release	Positive	1	1	1	0	0	0
540090	VSSVNMVSRLLLNRF	NS5	2718	2732	HLA-DRB1*14:04	51%-80%	Dengue virus 3	ELISPOT	IFNg release	Positive	1	1	1	0	0	0
866168	ARYLEFEALGFLNED	NS5	2970	2984	HLA-DQA1*03:01/DQB1*03:02	51%-80%	Dengue virus 3	ELISPOT	IFNg release	Positive	1	0	1	0	0	0
868748	SAKLQWFVERNMVIP	NS5	2549	2563	HLA-DQA1*01:01/DQB1*05:01	51%-80%	Dengue virus 3	ELISPOT	IFNg release	Positive	1	1	1	0	0	0
591723	NMLSIINRRKKTSLC	C	90	104	HLA-DRB1*11:01	>0% - 50%	Dengue virus 3	ELISPOT	IFNg release	Positive	1	0	1	0	0	0
868645	RGLLNGQGPMKLVMA	C	15	29	HLA-DRB3*02:02	>0% - 50%	Dengue virus 3	ELISPOT	IFNg release	Positive	1	1	1	0	0	0
190823	KLTVVVGDIIGVLEQ	NS1	85	99	HLA-DR2	>0% - 50%	Dengue virus 3	ELISPOT	IFNg release	Positive	1	1	1	0	0	0
195125	AGVIFTFVLLLSGQI	NS2a	1159	1173	HLA-DRB1*07:01	>0% - 50%	Dengue virus 3	ELISPOT	IFNg release	Positive	0	0	0	0	0	0
195740	LWTALVSLTCSNTIF	NS2a	1270	1284	HLA-DRB1*07:01	>0% - 50%	Dengue virus 3	ELISPOT	IFNg release	Positive	0	1	0	0	0	0
539802	QFETYQLWTALVSLT	NS2a	1264	1278	HLA-DRB1*15:02	>0% - 50%	Dengue virus 3	ELISPOT	IFNg release	Positive	0	1	0	0	0	0
868109	MIAGVIFTFVLLLSG	NS2a	1157	1171	HLA-DPB1*04:02	>0% - 50%	Dengue virus 3	ELISPOT	IFNg release	Positive	0	0	0	0	0	0
539432	IREAIKRRLRTLILA	NS3	1682	1696	HLA-DRB1*08:03	>0% - 50%	Dengue virus 3	ELISPOT	IFNg release	Positive	0	0	0	0	0	0
196168	VPLQWIASAIVLEFF	NS4a	2192	2206	HLA-DRB1*01:01	>0% - 50%	Dengue virus 3	ELISPOT	IFNg release	Positive	1	1	1	0	0	0
869474	VIGILTLAAIIAANE	NS4a	2230	2244	HLA-DQA1*01:02/DQB1*06:02	>0% - 50%	Dengue virus 3	ELISPOT	IFNg release	Positive	0	0	0	0	0	0
195663	LLLITHYAIIGPGLQ	NS4b	2354	2368	HLA-DRB1*07:01	>0% - 50%	Dengue virus 3	ELISPOT	IFNg release	Positive	0	0	0	0	0	0
196023	TAAVLLLITHYAIIG	NS4b	2350	2364	HLA-DRB1*15:01	>0% - 50%	Dengue virus 3	ELISPOT	IFNg release	Positive	0	0	0	0	0	0
869526	VLLLVTHYAIIGPGL	NS4b	2353	2367	HLA-DPB1*02:01	>0% - 50%	Dengue virus 3	ELISPOT	IFNg release	Positive	1	0	1	0	0	0
196239	YQNKVVKVQRPTPKG	NS5	3062	3076	HLA-DRB1*08:02	>0% - 50%	Dengue virus 3	ELISPOT	IFNg release	Positive	1	1	1	0	0	0

CD4 DENV-4 Epitopes

											DENV-4 constructs of vaccines (100% identity=1,green ; else 0, red)					
**Epitope_ID**	**AA_Seq**	**Protein_Name**	**Start**	**End**	**Allele**	**Step**	**Serotype**	**Method/Technique**	**Assay Group**	**Qualitative Measure**	**TV003**	**TDEN**	**DPIV**	**Dengvaxia**	**DENVax**	**TVDV**
195483	IGFRKEIGRMLNILN	C	81	95	HLA-DRB1*04:01	0%	Dengue virus 4	ELISPOT	IFNg release	Positive	1	1	1	0	0	0
195488	IGRMLNILNRRKRST	C	87	101	HLA-DRB1*14:02	0%	Dengue virus 4	ELISPOT	IFNg release	Positive	0	0	0	0	0	0
195580	KNKAIKILIGFRKEI	C	53	67	HLA-DRB1*15:01	0%	Dengue virus 4	ELISPOT	IFNg release	Positive	1	1	1	0	0	0
195768	MTLLCLIPTAMAFHL	C	102	116	HLA-DRB1*01:01	0%	Dengue virus 4	ELISPOT	IFNg release	Positive	0	0	0	0	0	0
195922	RWGQLKKTKAIKILT	C	67	81	HLA-DRB1*07:01	0%	Dengue virus 4	ELISPOT	IFNg release	Positive	0	0	0	0	0	0
196065	TPQGLVKRFSSGLFS	C	24	38	HLA-DRB1*15:01	0%	Dengue virus 4	ELISPOT	IFNg release	Positive	0	0	0	0	0	0
36533	LIGFRKEIGRML	C	80	91	HLA-DPw4	0%	Dengue virus 4	ICS	IFNg release	Positive	1	1	1	0	0	0
539571	LIGFRKEIGRMLNIL	C	80	94	HLA-DRB1*09:01	0%	Dengue virus 4	ELISPOT	IFNg release	Positive	1	1	1	0	0	0
539581	LKKNKAIKILIGFRK	C	51	65	HLA-DRB1*14:04	0%	Dengue virus 4	ELISPOT	IFNg release	Positive	1	1	1	0	0	0
540116	WGQLKKNKAIKILIG	C	68	82	HLA-DRB1*09:01	0%	Dengue virus 4	ELISPOT	IFNg release	Positive	1	1	1	0	0	0
739060	GRMLNILNGRKRSTM	C	88	102	HLA-DRB1*14:02	0%	Dengue virus 4	ELISPOT	IFNg release	Positive	0	0	0	0	0	0
739178	IGRMLNILNRRRRST	C	87	101	HLA-DRB1*14:02	0%	Dengue virus 4	ELISPOT	IFNg release	Positive	0	0	0	0	0	0
868732	RWGQLKKNKAIKILI	C	39	53	HLA-DRB3*02:02	0%	Dengue virus 4	ELISPOT	IFNg release	Positive	1	1	1	0	0	0
195916	RTVFFVLMMLVAPSY	PreM	264	278	HLA-DRB1*01:01	0%	Dengue virus 4	ELISPOT	IFNg release	Positive	1	1	1	0	1	1
869670	WCNLTSTWVMYGTCT	PreM	180	194	HLA-DQA1*01:02/DQB1*06:02	0%	Dengue virus 4	ELISPOT	IFNg release	Positive	1	1	1	0	1	0
167808	RDVNKEKVVGRVISSTPLAE	E	340	359	HLA class II	0%	Dengue virus 4	ELISPOT	IFNg release	Positive	0	0	0	0	0	0
195227	DSYIVIGVGNSALTL	E	654	668	HLA-DRB1*15:06	0%	Dengue virus 4	ELISPOT	IFNg release	Positive	1	1	1	0	1	0
195364	GGVSWMIRILIGILV	E	449	463	HLA-DRB1*15:06	0%	Dengue virus 4	ELISPOT	IFNg release	Positive	0	0	0	0	0	0
195434	GVSWMIRILIGFLVL	E	729	743	HLA-DRB1*08:02	0%	Dengue virus 4	ELISPOT	IFNg release	Positive	1	1	1	0	1	0
195435	GVSWMIRILIGLLVL	E	729	743	HLA-DRB1*15:06	0%	Dengue virus 4	ELISPOT	IFNg release	Positive	0	0	0	0	0	0
195625	LESTYRGAKRMAILG	E	681	695	HLA-DRB1*14:02	0%	Dengue virus 4	ELISPOT	IFNg release	Positive	0	0	0	0	0	1
196036	TGIQRTVFFVLMMLV	E	44	58	HLA-DRB1*07:01	0%	Dengue virus 4	ELISPOT	IFNg release	Positive	1	1	1	0	1	1
196189	VSWIIRILIGLLVLW	E	451	465	HLA-DRB1*15:06	0%	Dengue virus 4	ELISPOT	IFNg release	Positive	0	0	0	0	0	0
539230	EMILMKMKKKTWLVH	E	474	488	HLA-DRB1*12:02	0%	Dengue virus 4	ELISPOT	IFNg release	Positive	1	1	1	1	1	1
195791	NIMWKQITNELNYVL	NS1	839	853	HLA-DRB1*04:07	0%	Dengue virus 4	ELISPOT	IFNg release	Positive	0	0	0	0	0	0
539829	QYQFQPESPARLASA	NS1	805	819	HLA-DRB1*09:01	0%	Dengue virus 4	ELISPOT	IFNg release	Positive	0	0	0	0	0	0
167725	AMTTTLSIPHDLMELIDGIS	NS2a	50	69	HLA class II	0%	Dengue virus 4	ELISPOT	IFNg release	Positive	0	0	0	0	0	0
195156	ALSLTFIRSTMPLVM	NS2a	1274	1288	HLA-DRB1*07:01	0%	Dengue virus 4	ELISPOT	IFNg release	Positive	1	1	1	0	0	0
195157	ALSLTFIRSTMSLVM	NS2a	1274	1288	HLA-DRB1*04:01	0%	Dengue virus 4	ELISPOT	IFNg release	Positive	0	0	0	0	0	0
195158	ALSLTFIRSTTPLVM	NS2a	1274	1288	HLA-DRB1*07:01	0%	Dengue virus 4	ELISPOT	IFNg release	Positive	0	0	0	0	0	0
195190	AVFKMSPGYVLGVFL	NS2a	1204	1218	HLA-DRB1*01:01	0%	Dengue virus 4	ELISPOT	IFNg release	Positive	1	1	1	0	0	0
195221	DLMELISGISLGLIL	NS2a	1243	1257	HLA-DRB1*07:01	0%	Dengue virus 4	ELISPOT	IFNg release	Positive	0	0	0	0	0	0
195746	MAVFKMSPGYVLGVF	NS2a	1203	1217	HLA-DRB1*07:01	0%	Dengue virus 4	ELISPOT	IFNg release	Positive	1	1	1	0	0	0
196061	TMSLVMAWRTIMAVL	NS2a	1283	1297	HLA-DRB1*03:01	0%	Dengue virus 4	ELISPOT	IFNg release	Positive	0	0	0	0	0	0
196070	TRKHMILAVVITLCA	NS2a	1154	1168	HLA-DRB1*08:02	0%	Dengue virus 4	ELISPOT	IFNg release	Positive	0	0	0	0	0	0
196253	YVLGVFLRKLTSRET	NS2a	1212	1226	HLA-DRB1*11:01	0%	Dengue virus 4	ELISPOT	IFNg release	Positive	1	1	1	0	0	0
539130	CLRRRVTRKHMILAV	NS2a	1148	1162	HLA-DRB1*08:03	0%	Dengue virus 4	ELISPOT	IFNg release	Positive	0	0	0	0	0	0
539198	ECLRRRVTRKHMILA	NS2a	1147	1161	HLA-DRB1*13:01	0%	Dengue virus 4	ELISPOT	IFNg release	Positive	0	0	0	0	0	0
539360	GVFLRKLTSRETALM	NS2a	1215	1229	HLA-DRB1*10:01	0%	Dengue virus 4	ELISPOT	IFNg release	Positive	1	1	1	0	0	0
539406	IGGQIHLAIMAVFKM	NS2a	1194	1208	HLA-DRB1*13:01	0%	Dengue virus 4	ELISPOT	IFNg release	Positive	1	1	1	0	0	0
539407	IGGQTHLAIMAVFKM	NS2a	1194	1208	HLA-DRB1*13:01	0%	Dengue virus 4	ELISPOT	IFNg release	Positive	0	0	0	0	0	0
539433	IRSTMPLVMAWRTIM	NS2a	1280	1294	HLA-DRB1*13:01	0%	Dengue virus 4	ELISPOT	IFNg release	Positive	1	1	1	0	0	0
539434	IRSTMSLVMAWRTIM	NS2a	1280	1294	HLA-DRB1*13:01	0%	Dengue virus 4	ELISPOT	IFNg release	Positive	0	0	0	0	0	0
539565	LGVFLRKLTSRETAL	NS2a	1214	1228	HLA-DRB1*08:03	0%	Dengue virus 4	ELISPOT	IFNg release	Positive	1	1	1	0	0	0
539627	LSLTFIRSTMPLVMA	NS2a	1275	1289	HLA-DRB1*10:01	0%	Dengue virus 4	ELISPOT	IFNg release	Positive	1	1	1	0	0	0
539628	LSLTFIRSTTPLVMA	NS2a	1275	1289	HLA-DRB1*08:03	0%	Dengue virus 4	ELISPOT	IFNg release	Positive	0	0	0	0	0	0
539919	SLTFIRSTMPLVMAW	NS2a	1276	1290	HLA-DRB1*09:01	0%	Dengue virus 4	ELISPOT	IFNg release	Positive	1	1	1	0	0	0
539921	SLTFIRSTTPLIMAW	NS2a	1276	1290	HLA-DRB1*04:03	0%	Dengue virus 4	ELISPOT	IFNg release	Positive	0	0	0	0	0	0
539961	TALMVIGMAMTTTLS	NS2a	1226	1240	HLA-DRB1*01:02	0%	Dengue virus 4	ELISPOT	IFNg release	Positive	0	0	0	0	0	0
540033	VFKMSPGYVLGVFLR	NS2a	1205	1219	HLA-DRB1*09:01	0%	Dengue virus 4	ELISPOT	IFNg release	Positive	1	1	1	0	0	0
540053	VLGVFLRKLTSRETA	NS2a	1213	1227	HLA-DRB1*14:04	0%	Dengue virus 4	ELISPOT	IFNg release	Positive	1	1	1	0	0	0
738559	AVVITLCAIILGGLT	NS2a	1161	1175	HLA-DRB1*01:02	0%	Dengue virus 4	ELISPOT	IFNg release	Positive	0	0	0	0	0	0
867969	LTFIRSTTPLVMAWR	NS2a	1277	1291	HLA-DRB3*02:02	0%	Dengue virus 4	ELISPOT	IFNg release	Positive	0	0	0	0	0	0
45689	NRKGKIVGLYGNGVV	NS3	141	155	HLA-DRB1*15:01	0%	Dengue virus 4	ICS	IFNg release	Positive	0	0	0	0	0	0
53505	REGEKKKLRPRWLDR	NS3	585	599	HLA class II	0%	Dengue virus 4	ICS	IFNg release	Positive	0	0	0	0	0	0
539020	AAAIFMTATPPGATD	NS3	1783	1797	HLA-DRB1*10:01	0%	Dengue virus 4	ELISPOT	IFNg release	Positive	1	1	1	0	0	0
539021	AAAIFMTATPPGSID	NS3	1783	1797	HLA-DRB1*04:07	0%	Dengue virus 4	ELISPOT	IFNg release	Positive	0	0	0	0	0	0
539023	AAAIFMTATPPGTID	NS3	1783	1797	HLA-DRB1*04:03	0%	Dengue virus 4	ELISPOT	IFNg release	Positive	0	0	0	0	0	0
539026	AAIFMTATPPGATDP	NS3	1784	1798	HLA-DRB1*09:01	0%	Dengue virus 4	ELISPOT	IFNg release	Positive	1	1	1	0	0	0
540077	VREAIKRRLRTLVLA	NS3	1682	1696	HLA-DRB1*08:03	0%	Dengue virus 4	ELISPOT	IFNg release	Positive	0	0	0	0	0	0
868371	PRWLDARVYADPMAL	NS3	2067	2081	HLA-DQA1*03:01/DQB1*03:02	0%	Dengue virus 4	ELISPOT	IFNg release	Positive	1	1	1	0	0	0
195386	GLITIAVASGLLWVA	NS4a	2176	2190	HLA-DRB1*07:01	0%	Dengue virus 4	ELISPOT	IFNg release	Positive	1	1	1	0	0	0
195551	IYVILTILTIIALVA	NS4a	2227	2241	HLA-DRB1*01:01	0%	Dengue virus 4	ELISPOT	IFNg release	Positive	0	0	0	0	0	0
195648	LIYVILTILTIIALV	NS4a	2226	2240	HLA-DRB1*07:01	0%	Dengue virus 4	ELISPOT	IFNg release	Positive	0	0	0	0	0	0
195853	QLIYVILTILAIIGL	NS4a	2225	2239	HLA-DRB1*15:02	0%	Dengue virus 4	ELISPOT	IFNg release	Positive	0	0	0	0	0	0
196250	YVILAILTIIGLVAA	NS4a	2228	2242	HLA-DRB1*08:02	0%	Dengue virus 4	ELISPOT	IFNg release	Positive	0	0	0	0	0	0
196251	YVILALLTIISLIAA	NS4a	2228	2242	HLA-DRB1*15:02	0%	Dengue virus 4	ELISPOT	IFNg release	Positive	0	0	0	0	0	0
539666	MGLITIAVASGLLWV	NS4a	2175	2189	HLA-DRB1*09:01	0%	Dengue virus 4	ELISPOT	IFNg release	Positive	1	1	1	0	0	0
867726	LIYVILTILTIIGLI	NS4a	2226	2240	HLA-DPB1*04:02	0%	Dengue virus 4	ELISPOT	IFNg release	Positive	1	1	1	0	0	0
195719	LTASLAMLLVHYAII	NS4b	2346	2360	HLA-DRB1*07:01	0%	Dengue virus 4	ELISPOT	IFNg release	Positive	0	0	0	0	0	0
196055	TLIASLVMLLVHYAI	NS4b	2345	2359	HLA-DRB1*01:01	0%	Dengue virus 4	ELISPOT	IFNg release	Positive	0	0	0	0	0	0
196082	TTLIASLVMLLVHYA	NS4b	2344	2358	HLA-DRB1*13:01	0%	Dengue virus 4	ELISPOT	IFNg release	Positive	0	0	0	0	0	0
196209	WAFCEVLTLATGPVL	NS4b	2427	2441	HLA-DRB1*07:01	0%	Dengue virus 4	ELISPOT	IFNg release	Positive	0	0	0	0	0	0
539661	MGCYSQVNPTTLIAS	NS4b	2335	2349	HLA-DRB1*09:01	0%	Dengue virus 4	ELISPOT	IFNg release	Positive	0	0	0	0	0	0
738500	AMGCYSQVNPTTLIA	NS4b	2334	2348	HLA-DRB1*04:07	0%	Dengue virus 4	ELISPOT	IFNg release	Positive	0	0	0	0	0	0
195230	DYMPVMKRYSAPSES	NS5	3369	3383	HLA-DRB1*11:01	0%	Dengue virus 4	ELISPOT	IFNg release	Positive	1	1	1	0	0	0
195593	KTFMKDGRSLVVPCR	NS5	3202	3216	HLA-DRB1*03:01	0%	Dengue virus 4	ELISPOT	IFNg release	Positive	0	0	0	0	0	0
195764	MPVMKRYSAPSESEG	NS5	3371	3385	HLA-DRB1*08:02	0%	Dengue virus 4	ELISPOT	IFNg release	Positive	1	1	1	0	0	0
195835	PKPGTRMVMTTTANW	NS5	2853	2867	HLA-DRB1*04:01	0%	Dengue virus 4	ELISPOT	IFNg release	Positive	1	1	1	0	0	0
195919	RVWIEDNPNMIDKTP	NS5	3303	3317	HLA-DRB1*03:01	0%	Dengue virus 4	ELISPOT	IFNg release	Positive	0	0	0	0	0	0
196071	TRMVMTTTANWLWAL	NS5	2857	2871	HLA-DRB1*04:01	0%	Dengue virus 4	ELISPOT	IFNg release	Positive	1	1	1	0	0	0
196092	TYQNKVVKVLRPTPR	NS5	3059	3073	HLA-DRB1*08:02	0%	Dengue virus 4	ELISPOT	IFNg release	Positive	1	1	1	0	0	0
196106	VDYMPVMKRYSAPSE	NS5	3368	3382	HLA-DRB1*11:01	0%	Dengue virus 4	ELISPOT	IFNg release	Positive	1	1	1	0	0	0
539222	EKPDMTIIGRRLQRL	NS5	2757	2771	HLA-DRB1*12:02	0%	Dengue virus 4	ELISPOT	IFNg release	Positive	1	1	1	0	0	0
539354	GTRMVMTTTANWLWA	NS5	2856	2870	HLA-DRB1*04:03	0%	Dengue virus 4	ELISPOT	IFNg release	Positive	1	1	1	0	0	0
539493	KMLLNRFTTRHRKPT	NS5	2724	2738	HLA-DRB1*13:01	0%	Dengue virus 4	ELISPOT	IFNg release	Positive	1	1	1	0	0	0
539498	KPDMTIIGRRLQRLQ	NS5	2758	2772	HLA-DRB1*08:03	0%	Dengue virus 4	ELISPOT	IFNg release	Positive	1	1	1	0	0	0
539769	PGTRMVMTTTANWLW	NS5	2855	2869	HLA-DRB1*04:03	0%	Dengue virus 4	ELISPOT	IFNg release	Positive	1	1	1	0	0	0
540007	TTSKMLLNRFTTRHR	NS5	2721	2735	HLA-DRB1*13:01	0%	Dengue virus 4	ELISPOT	IFNg release	Positive	1	1	1	0	0	0
867473	KPLDERFGTSLLFLN	NS5	3156	3170	HLA-DPB1*01:01	0%	Dengue virus 4	ELISPOT	IFNg release	Positive	1	1	1	0	0	0
867575	LDERFGTSLLFLNDM	NS5	3158	3172	HLA-DPB1*04:01	0%	Dengue virus 4	ELISPOT	IFNg release	Positive	1	1	1	0	0	0
1067635	LKRWGQLKKNKAIKI	C	65	79	HLA-DRB1*07:01	100%	Dengue virus 4	ELISPOT	IFNg release	Positive-Low	1	1	1	0	0	0
1068575	VVRPPFNMLKRERNR	C	7	21	HLA-DRB1*11:01	100%	Dengue virus 4	ELISPOT	IFNg release	Positive-Low	1	1	1	0	0	0
17605	FRKEIGRML	C	83	91	HLA-DPw4	100%	Dengue virus 4	51 chromium	cytotoxicity	Positive	1	1	1	0	0	0
195316	FNMLKRERNRVSTPQ	C	12	26	HLA-DRB1*04:01	100%	Dengue virus 4	ELISPOT	IFNg release	Positive	1	1	1	0	0	0
195325	FRKEIGRMLNILNGR	C	19	33	HLA-DRB1*04:01	100%	Dengue virus 4	ELISPOT	IFNg release	Positive	1	1	1	0	0	0
195587	KRWGQLKKNKAIKIL	C	46	60	HLA-DRB1*08:03	100%	Dengue virus 4	ELISPOT	IFNg release	Positive	1	1	1	0	0	0
195651	LKKNKAIKILTGFRK	C	51	65	HLA-DRB1*14:04	100%	Dengue virus 4	ELISPOT	IFNg release	Positive	0	0	0	0	0	0
195900	RMVLAFITFLRVLSI	C	44	58	HLA-DRB1*11:01	100%	Dengue virus 4	ELISPOT	IFNg release	Positive	1	1	1	0	0	0
196134	VLAFITFLRVLSIPP	C	26	40	HLA-DRB1*04:01	100%	Dengue virus 4	ELISPOT	IFNg release	Positive	1	1	1	0	0	0
539044	AFITFLRVLSIPPTA	C	28	42	HLA-DRB1*04:03	100%	Dengue virus 4	ELISPOT	IFNg release	Positive	1	1	1	0	0	0
539255	FITFLRVLSIPPTAG	C	49	63	HLA-DRB1*04:07	100%	Dengue virus 4	ELISPOT	IFNg release	Positive	1	1	1	0	0	0
539294	GFRKEIGRMLNILNG	C	54	68	HLA-DRB3*02:02	100%	Dengue virus 4	ELISPOT	IFNg release	Positive	1	1	1	0	0	0
539496	KNKAIKILTGFRKEI	C	53	67	HLA-DRB1*12:02	100%	Dengue virus 4	ELISPOT	IFNg release	Positive	0	0	0	0	0	0
539536	LAFITFLRVLSIPPT	C	27	41	HLA-DRB1*12:02	100%	Dengue virus 4	ELISPOT	IFNg release	Positive	1	1	1	0	0	0
539632	LTGFRKEIGRMLNIL	C	52	66	HLA-DRB3*02:02	100%	Dengue virus 4	ELISPOT	IFNg release	Positive	0	0	0	0	0	0
69367	VLAFITFLR	C	46	54	HLA-DPw4	100%	Dengue virus 4	51 chromium	cytotoxicity	Positive	1	1	1	0	0	0
739175	IGRMLNILNGRKRST	C	87	101	HLA-DRB1*14:02	100%	Dengue virus 4	ELISPOT	IFNg release	Positive	1	1	1	0	0	0
740156	RPPFNMLKRERNRVS	C	9	23	HLA-DRB1*14:02	100%	Dengue virus 4	ELISPOT	IFNg release	Positive	1	1	1	0	0	0
868517	QLKKNKAIKILTGFR	C	42	56	HLA-DQA1*01:02/DQB1*06:02	100%	Dengue virus 4	ELISPOT	IFNg release	Positive	0	0	0	0	0	0
195283	FALLAGFMAYMIGQT	PreM	246	260	HLA-DRB1*01:01	100%	Dengue virus 4	ELISPOT	IFNg release	Positive	1	1	1	0	1	1
195505	ILRNPGFALLAGFMA	PreM	240	254	HLA-DRB1*01:01	100%	Dengue virus 4	ELISPOT	IFNg release	Positive	1	1	1	0	1	1
195901	RNPGFALLAGFMAYM	PreM	242	256	HLA-DRB1*15:01	100%	Dengue virus 4	ELISPOT	IFNg release	Positive	1	1	1	0	1	1
539395	IDCWCNLTSAWVMYG	PreM	117	131	HLA-DQA1*01:02/DQB1*06:02	100%	Dengue virus 4	ELISPOT	IFNg release	Positive	0	0	0	0	0	1
869119	TEGINKCTLIAMDLG	PreM	113	127	HLA-DQA1*01:02/DQB1*06:02	100%	Dengue virus 4	ELISPOT	IFNg release	Positive	1	1	1	0	1	1
195202	CIAVGGITLFLGFTV	E	758	772	HLA-DRB1*15:01	100%	Dengue virus 4	ELISPOT	IFNg release	Positive	1	1	1	1	1	1
195272	ESTYRGAKRMAILGE	E	682	696	HLA-DRB1*15:06	100%	Dengue virus 4	ELISPOT	IFNg release	Positive	1	1	1	1	1	1
539379	HAKRQDVTVLGSQEGAMH	E	523	540	HLA class II	100%	Dengue virus 4	ICS	IFNg release	Positive	1	1	1	1	1	1
539734	NLEYTVVVTVHNGDT	E	197	211	HLA-DRB1*04:07	100%	Dengue virus 4	ELISPOT	IFNg release	Positive	1	1	1	1	1	1
738809	ENLEYTVVVTVHNGD	E	196	210	HLA-DRB1*04:07	100%	Dengue virus 4	ELISPOT	IFNg release	Positive	1	1	1	1	1	1
738886	FESTYRGAKRMAILG	E	681	695	HLA-DRB1*14:02	100%	Dengue virus 4	ELISPOT	IFNg release	Positive	1	1	1	1	1	0
741747	MRCVGVGNRDFVEGVSGGAW	E	280	299	HLA class II	100%	Dengue virus 4	ELISPOT	IFNg release	Positive	1	1	1	1	1	1
195541	ITALILGAQALPVYL	NS2a	1320	1334	HLA-DRB1*01:01	100%	Dengue virus 4	ELISPOT	IFNg release	Positive	1	1	1	0	0	0
195699	LQKQSHWVEITALIL	NS2a	1311	1325	HLA-DRB1*07:01	100%	Dengue virus 4	ELISPOT	IFNg release	Positive	1	1	1	0	0	0
195837	PLVMAWRTIMAVLFV	NS2a	1285	1299	HLA-DRB1*01:01	100%	Dengue virus 4	ELISPOT	IFNg release	Positive	1	1	1	0	0	0
195864	QSHWVEITALILGAQ	NS2a	1314	1328	HLA-DRB1*07:01	100%	Dengue virus 4	ELISPOT	IFNg release	Positive	1	1	1	0	0	0

196060	TMPLVMAWRTIMAVL	NS2a	1283	1297	HLA-DRB1*03:01	100%	Dengue virus 4	ELISPOT	IFNg release	Positive	1	1	1	0	0	0
196227	WVEITALILGAQALP	NS2a	1317	1331	HLA-DRB1*01:01	100%	Dengue virus 4	ELISPOT	IFNg release	Positive	1	1	1	0	0	0
539131	CLRRRVTRKHMILVV	NS2a	1148	1162	HLA-DRB1*08:03	100%	Dengue virus 4	ELISPOT	IFNg release	Positive	1	1	1	0	0	0
539687	MPLVMAWRTIMAVLF	NS2a	1284	1298	HLA-DRB1*12:02	100%	Dengue virus 4	ELISPOT	IFNg release	Positive	1	1	1	0	0	0
539937	STMPLVMAWRTIMAV	NS2a	1282	1296	HLA-DRB1*13:01	100%	Dengue virus 4	ELISPOT	IFNg release	Positive	1	1	1	0	0	0
738742	ECLRRRVTRKHMILV	NS2a	1147	1161	HLA-DRB1*14:02	100%	Dengue virus 4	ELISPOT	IFNg release	Positive	1	1	1	0	0	0
867110	HWVEITALILGAQAL	NS2a	1316	1330	HLA-DQA1*03:01/DQB1*03:02	100%	Dengue virus 4	ELISPOT	IFNg release	Positive	1	1	1	0	0	0
195511	IMAVGLVSLLGSALL	NS2b	1352	1366	HLA-DRB1*01:01	100%	Dengue virus 4	ELISPOT	IFNg release	Positive	1	1	1	0	0	0
195662	LLLAAYVMSGSSADL	NS2b	1381	1395	HLA-DRB1*04:01	100%	Dengue virus 4	ELISPOT	IFNg release	Positive	1	1	1	0	0	0
196062	TNMITLLVKLALITV	NS2b	1436	1450	HLA-DRB1*08:02	100%	Dengue virus 4	ELISPOT	IFNg release	Positive	1	1	1	0	0	0
869635	VTMTLWYMWQVKTQR	NS2b	1460	1474	HLA-DPB1*01:01	100%	Dengue virus 4	ELISPOT	IFNg release	Positive	1	1	1	0	0	0
1067119	GKVIGLYGNGVVTKS	NS3	1618	1632	HLA-DRB1*15:01	100%	Dengue virus 4	ELISPOT	IFNg release	Positive	1	1	1	0	0	0
12605	EIVDLMCHAT	NS3	255	264	HLA-DPw2	100%	Dengue virus 4	51 chromium	cytotoxicity	Positive	1	1	1	0	0	0
156711	LAPTRVVAAEMEEAL	NS3	1695	1709	HLA class II	100%	Dengue virus 4	51 chromium	cytotoxicity	Positive	1	1	1	0	0	0
195298	FHTMWHVTRGSVICH	NS3	1520	1534	HLA-DRB1*07:01	100%	Dengue virus 4	ELISPOT	IFNg release	Positive	1	1	1	0	0	0
195563	KGKVIGLYGNGVVTK	NS3	5	19	HLA-DRB1*15:01	100%	Dengue virus 4	ELISPOT	IFNg release	Positive	1	1	1	0	0	0
195695	LPVWLSYKVASAGIS	NS3	2016	2030	HLA-DRB1*08:02	100%	Dengue virus 4	ELISPOT	IFNg release	Positive	1	1	1	0	0	0
195838	PNYNLIVMDEAHFTD	NS3	1750	1764	HLA-DRB1*03:01	100%	Dengue virus 4	ELISPOT	IFNg release	Positive	1	1	1	0	0	0
33192	KRLTIMDLHPGAGKT	NS3	186	200	HLA class II	100%	Dengue virus 4	ICS	IFNg release	Positive	1	1	1	0	0	0
539024	AAAIFMTATPPGTTD	NS3	1783	1797	HLA-DRB1*04:03	100%	Dengue virus 4	ELISPOT	IFNg release	Positive	0	0	0	0	0	0
539080	ALRGLPIRYQTPAVK	NS3	96	110	HLA-DRB1*13:01	100%	Dengue virus 4	ELISPOT	IFNg release	Positive	1	1	1	0	0	0
539359	GVFHTMWHVTRGSVI	NS3	1518	1532	HLA-DRB1*08:03	100%	Dengue virus 4	ELISPOT	IFNg release	Positive	1	1	1	0	0	0
539520	KVIGLYGNGVVTKSG	NS3	7	21	HLA-DRB1*15:06	100%	Dengue virus 4	ELISPOT	IFNg release	Positive	1	1	1	0	0	0
539803	QGKTVWFVPSIKAGN	NS3	1829	1843	HLA-DRB1*08:03	100%	Dengue virus 4	ELISPOT	IFNg release	Positive	1	1	1	0	0	0
68977	VIGLYGNGV	NS3	146	154	HLA-DRB1*15:01	100%	Dengue virus 4	51 chromium	cytotoxicity	Positive	1	1	1	0	0	0
863140	TRVEMGEAAAIFMTA	NS3	1776	1790	HLA-DQA1*03:01/DQB1*03:02	100%	Dengue virus 4	ELISPOT	IFNg release	Positive	1	1	1	0	0	0
867453	KLTDWDFVVTTDISE	NS3	1872	1886	HLA-DQA1*03:01/DQB1*03:02	100%	Dengue virus 4	ELISPOT	IFNg release	Positive	1	1	1	0	0	0
167758	ILTEIASLPTYLSSRAKL	NS4a	6	23	HLA-DP	100%	Dengue virus 4	ELISPOT	IFNg release	Positive	1	1	1	0	0	0
195176	ASLPTYLSSRAKLAL	NS4a	2103	2117	HLA-DRB1*07:01	100%	Dengue virus 4	ELISPOT	IFNg release	Positive	1	1	1	0	0	0
195224	DNQLIYVILTILTII	NS4a	2223	2237	HLA-DRB1*15:01	100%	Dengue virus 4	ELISPOT	IFNg release	Positive	1	1	1	0	0	0
195809	NQLIYVILTILTIIG	NS4a	2224	2238	HLA-DRB1*08:02	100%	Dengue virus 4	ELISPOT	IFNg release	Positive	1	1	1	0	0	0
195876	RAKLALDNIVMLHTT	NS4a	2112	2126	HLA-DRB1*03:01	100%	Dengue virus 4	ELISPOT	IFNg release	Positive	1	1	1	0	0	0
539165	DILTEIASLPTYLSS	NS4a	2097	2111	HLA-DRB1*04:07	100%	Dengue virus 4	ELISPOT	IFNg release	Positive	1	1	1	0	0	0
539313	GKAYQHALNELPESL	NS4a	2130	2144	HLA-DRB1*10:01	100%	Dengue virus 4	ELISPOT	IFNg release	Positive	0	0	0	0	0	0
539631	LTEIASLPTYLSSRA	NS4a	2099	2113	HLA-DRB1*04:03	100%	Dengue virus 4	ELISPOT	IFNg release	Positive	1	1	1	0	0	0
738568	AYQHALNELPESLET	NS4a	2132	2146	HLA-DRB1*04:07	100%	Dengue virus 4	ELISPOT	IFNg release	Positive	1	1	1	0	0	0
868951	SLPTYLSSRAKLALD	NS4a	2104	2118	HLA-DRB3*02:02	100%	Dengue virus 4	ELISPOT	IFNg release	Positive	1	1	1	0	0	0
195177	ASLVMLLVHYAIIGP	NS4b	2348	2362	HLA-DRB1*13:01	100%	Dengue virus 4	ELISPOT	IFNg release	Positive	1	0	1	0	0	0
195219	DLEPISYDPKFEKQL	NS4b	2393	2407	HLA-DRB1*03:01	100%	Dengue virus 4	ELISPOT	IFNg release	Positive	1	1	1	0	0	0
195276	ETTILDVDLRPASAW	NS4b	2263	2277	HLA-DRB1*03:01	100%	Dengue virus 4	ELISPOT	IFNg release	Positive	1	1	1	0	0	0
195592	KTETTILDVDLRPAS	NS4b	2261	2275	HLA-DRB1*03:01	100%	Dengue virus 4	ELISPOT	IFNg release	Positive	1	1	1	0	0	0
195616	LCAGQLLLMRTTWAF	NS4b	2415	2429	HLA-DRB1*04:01	100%	Dengue virus 4	ELISPOT	IFNg release	Positive	1	1	1	0	0	0
195720	LTASLVMLLVHYAII	NS4b	2346	2360	HLA-DRB1*12:02	100%	Dengue virus 4	ELISPOT	IFNg release	Positive	1	0	1	0	0	0
196048	TILDVDLRPASAWTL	NS4b	2265	2279	HLA-DRB1*03:01	100%	Dengue virus 4	ELISPOT	IFNg release	Positive	1	1	1	0	0	0
196208	WAFCEVLTLATGPIL	NS4b	2427	2441	HLA-DRB1*07:01	100%	Dengue virus 4	ELISPOT	IFNg release	Positive	1	1	1	0	0	0
196226	WTLYAVATTILTPML	NS4b	2277	2291	HLA-DRB1*07:01	100%	Dengue virus 4	ELISPOT	IFNg release	Positive	1	1	1	0	0	0
539057	AGQLLLMRTTWAFCE	NS4b	2417	2431	HLA-DRB1*13:01	100%	Dengue virus 4	ELISPOT	IFNg release	Positive	1	1	1	0	0	0
539185	DVDLRPASAWTLYAV	NS4b	2268	2282	HLA-DRB1*09:01	100%	Dengue virus 4	ELISPOT	IFNg release	Positive	1	1	1	0	0	0
539639	LVHYAIIGPGLQAKA	NS4b	2354	2368	HLA-DQA1*05:01/DQB1*02:01	100%	Dengue virus 4	ELISPOT	IFNg release	Positive	1	0	1	0	0	0
739202	ILDVDLRPASAWTLY	NS4b	2266	2280	HLA-DRB1*01:02	100%	Dengue virus 4	ELISPOT	IFNg release	Positive	1	1	1	0	0	0
1066543	ACLGKAYAQMWSLMY	NS5	3240	3254	HLA-DRB1*04:01	100%	Dengue virus 4	ELISPOT	IFNg release	Positive-Low	1	1	1	0	0	0
195235	EEFISKVRSNAAIGA	NS5	2884	2898	HLA-DRB1*08:02	100%	Dengue virus 4	ELISPOT	IFNg release	Positive	1	1	1	0	0	0
195240	EEYVDYMPVMKRYSA	NS5	3365	3379	HLA-DRB1*11:01	100%	Dengue virus 4	ELISPOT	IFNg release	Positive	1	1	1	0	0	0
195279	EYVDYMPVMKRYSAP	NS5	3366	3380	HLA-DRB1*11:01	100%	Dengue virus 4	ELISPOT	IFNg release	Positive	1	1	1	0	0	0
195737	LWCGSLIGLSSRATW	NS5	3333	3347	HLA-DRB1*11:01	100%	Dengue virus 4	ELISPOT	IFNg release	Positive	1	1	1	0	0	0
195815	NRVWIEDNPNMTDKT	NS5	3302	3316	HLA-DRB1*03:01	100%	Dengue virus 4	ELISPOT	IFNg release	Positive	1	1	1	0	0	0
195823	NWLWALLGKKKNPRL	NS5	2866	2880	HLA-DRB1*14:02	100%	Dengue virus 4	ELISPOT	IFNg release	Positive	1	1	1	0	0	0
195858	QMWSLMYFHRRDLRL	NS5	3248	3262	HLA-DRB1*15:01	100%	Dengue virus 4	ELISPOT	IFNg release	Positive	1	1	1	0	0	0
195879	REEFISKVRSNAAIG	NS5	2883	2897	HLA-DRB1*04:01	100%	Dengue virus 4	ELISPOT	IFNg release	Positive	1	1	1	0	0	0
196105	VDYMPVMKRYSAPFE	NS5	3368	3382	HLA-DRB1*11:01	100%	Dengue virus 4	ELISPOT	IFNg release	Positive	0	0	0	0	0	0
196230	YAQMWSLMYFHRRDL	NS5	3246	3260	HLA-DRB1*15:01	100%	Dengue virus 4	ELISPOT	IFNg release	Positive	1	1	1	0	0	0
539251	FEEYKRSGILEVDRT	NS5	2512	2526	HLA-DRB1*09:01	100%	Dengue virus 4	ELISPOT	IFNg release	Positive	1	1	1	0	0	0
539553	LGEFGRAKGSRAIWY	NS5	2949	2963	HLA-DRB1*09:01	100%	Dengue virus 4	ELISPOT	IFNg release	Positive	1	1	1	0	0	0
539656	MAISGDDCVVKPLDERF	NS5	3146	3162	HLA class II	100%	Dengue virus 4	ICS	IFNg release	Positive	1	1	1	0	0	0
539703	MVTQLAMTDTTPFGQQR	NS5	2824	2840	HLA class II	100%	Dengue virus 4	ICS	IFNg release	Positive	1	1	1	0	0	0
539770	PHHKILAKAIFKLTY	NS5	3046	3060	HLA-DRB1*12:02	100%	Dengue virus 4	ELISPOT	IFNg release	Positive	1	1	1	0	0	0
540041	VGTYGLNTFTNMEVQ	NS5	3091	3105	HLA-DRB1*15:06	100%	Dengue virus 4	ELISPOT	IFNg release	Positive	1	1	1	0	0	0
866633	ESCVYNMMGKREKKL	NS5	2935	2949	HLA-DRB5*01:01	100%	Dengue virus 4	ELISPOT	IFNg release	Positive	1	1	1	0	0	0
868638	RDLRLASMAICSAVP	NS5	3258	3272	HLA-DQA1*01:02/DQB1*06:02	100%	Dengue virus 4	ELISPOT	IFNg release	Positive	1	1	1	0	0	0
539898	SGKGPLRMVLAFITF	C	18	32	HLA-DRB1*15:06	81% - <100%	Dengue virus 4	ELISPOT	IFNg release	Positive	1	1	1	0	0	0
739059	GRMLNILNGRKRSTI	C	88	102	HLA-DRB1*14:06	81% - <100%	Dengue virus 4	ELISPOT	IFNg release	Positive	1	1	1	0	0	0
539463	KERMVTFKVPHAKRQ	E	513	527	HLA-DRB1*04:03	81% - <100%	Dengue virus 4	ELISPOT	IFNg release	Positive	1	1	1	1	1	1
540080	VRILIGFLVLWIGTN	E	455	469	HLA-DRB1*15:06	81% - <100%	Dengue virus 4	ELISPOT	IFNg release	Positive	0	0	0	1	0	1
195822	NVMWKQITNELNYVL	NS1	1	15	HLA-DRB1*04:07	81% - <100%	Dengue virus 4	ELISPOT	IFNg release	Positive	1	1	1	0	0	0
867043	HADMGYWIESSKNQT	NS1	969	983	HLA-DQA1*01:01/DQB1*05:01	81% - <100%	Dengue virus 4	ELISPOT	IFNg release	Positive	1	1	1	0	0	0
195571	KHMILVVVTTLCAII	NS2a	1156	1170	HLA-DRB1*08:02	81% - <100%	Dengue virus 4	ELISPOT	IFNg release	Positive	0	0	0	0	0	0
195404	GLVSLLGSALLKNDV	NS2b	9	23	HLA-DRB1*01:02	81% - <100%	Dengue virus 4	ELISPOT	IFNg release	Positive	1	1	1	0	0	0
195748	MAVGLVSLLGSALLK	NS2b	1353	1367	HLA-DRB1*01:01	81% - <100%	Dengue virus 4	ELISPOT	IFNg release	Positive	1	1	1	0	0	0
195931	SALLKNDVPLAGPMV	NS2b	1363	1377	HLA-DRB1*03:01	81% - <100%	Dengue virus 4	ELISPOT	IFNg release	Positive	1	1	1	0	0	0
539101	AVGLVSLLGSALLKN	NS2b	1354	1368	HLA-DRB1*10:01	81% - <100%	Dengue virus 4	ELISPOT	IFNg release	Positive	1	1	1	0	0	0
1392192	GREIVDLMCHATFTT	NS3	1727	1741		81% - <100%	Dengue virus 4	biological activity	activation	Positive	1	1	1	0	0	0
195244	EGVYRIMQRGLFGKT	NS3	1494	1508	HLA-DRB1*11:01	81% - <100%	Dengue virus 4	ELISPOT	IFNg release	Positive	1	1	1	0	0	0
195353	GEIGAVTLDFKPGTS	NS3	1595	1609	HLA-DRB1*03:01	81% - <100%	Dengue virus 4	ELISPOT	IFNg release	Positive	1	1	1	0	0	0
196032	TEGVYRIMQRGLFGK	NS3	146	160	HLA-DRB1*11:01	81% - <100%	Dengue virus 4	ELISPOT	IFNg release	Positive	0	0	0	0	0	0
539547	LFKTLTGEIGAVTLD	NS3	1589	1603	HLA-DRB1*10:01	81% - <100%	Dengue virus 4	ELISPOT	IFNg release	Positive	1	1	1	0	0	0
539767	PGLFKTLTGEIGAVT	NS3	1587	1601	HLA-DRB1*10:01	81% - <100%	Dengue virus 4	ELISPOT	IFNg release	Positive	1	1	1	0	0	0
739036	GLPIRYQTPAVKSEH	NS3	1711	1725	HLA-DRB1*14:06	81% - <100%	Dengue virus 4	ELISPOT	IFNg release	Positive	1	1	1	0	0	0
739563	LRGLPIRYQTPAVKS	NS3	1709	1723	HLA-DRB1*14:06	81% - <100%	Dengue virus 4	ELISPOT	IFNg release	Positive	1	1	1	0	0	0
195274	ETLMLVALLGAMTAG	NS4a	2145	2159	HLA-DRB1*08:02	81% - <100%	Dengue virus 4	ELISPOT	IFNg release	Positive	1	1	1	0	0	0
195575	KLSMGLIAIAVASGL	NS4a	2172	2186	HLA-DRB1*08:02	81% - <100%	Dengue virus 4	ELISPOT	IFNg release	Positive	0	0	0	0	0	0
196058	TLMLVALLGAMTAGI	NS4a	2146	2160	HLA-DRB1*01:01	81% - <100%	Dengue virus 4	ELISPOT	IFNg release	Positive	1	1	1	0	0	0
539638	LVALLGAMTAGIFLF	NS4a	2149	2163	HLA-DRB1*09:01	81% - <100%	Dengue virus 4	ELISPOT	IFNg release	Positive	1	1	1	0	0	0
539584	LLAMGCYSQVNPTTL	NS4b	2332	2346	HLA-DRB1*09:01	81% - <100%	Dengue virus 4	ELISPOT	IFNg release	Positive	1	1	1	0	0	0
867009	GRFWNTTIAVSTANI	NS4b	2449	2463	HLA-DQA1*01:02/DQB1*06:02	81% - <100%	Dengue virus 4	ELISPOT	IFNg release	Positive	1	1	1	0	0	0
195454	HHFHKIFMKDGRSLV	NS5	3199	3213	HLA-DRB1*03:01	81% - <100%	Dengue virus 4	ELISPOT	IFNg release	Positive	1	1	1	0	0	0
195499	ILAKAIFKLTYQNKV	NS5	3050	3064	HLA-DRB1*07:01	81% - <100%	Dengue virus 4	ELISPOT	IFNg release	Positive	1	1	1	0	0	0
539149	DCVVKPLDERFSTSLLF	NS5	3152	3168	HLA class II	81% - <100%	Dengue virus 4	ICS	IFNg release	Positive	0	0	0	0	0	0
539456	KAIFKLTYQNKVVKV	NS5	3053	3067	HLA-DRB1*09:01	81% - <100%	Dengue virus 4	ELISPOT	IFNg release	Positive	1	1	1	0	0	0
739405	KVRSNAAIGAVFQEE	NS5	2889	2903	HLA-DRB1*01:02	81% - <100%	Dengue virus 4	ELISPOT	IFNg release	Positive	1	1	1	0	0	0
866364	DERFSTSLLFLNDMG	NS5	3159	3173	HLA-DPB1*01:01	81% - <100%	Dengue virus 4	ELISPOT	IFNg release	Positive	0	0	0	0	0	0
867070	HKIFMKDGRSLVVPC	NS5	3202	3216	HLA-DRB3*02:02	81% - <100%	Dengue virus 4	ELISPOT	IFNg release	Positive	1	1	1	0	0	0
867315	ISKVRSNAAIGAVFQ	NS5	2887	2901	HLA-DRB3*02:02	81% - <100%	Dengue virus 4	ELISPOT	IFNg release	Positive	1	1	1	0	0	0
867411	KILAKAIFKLTYQNK	NS5	3049	3063	HLA-DPB1*04:02	81% - <100%	Dengue virus 4	ELISPOT	IFNg release	Positive	1	1	1	0	0	0
195436	GVSWMVRILIGFLVL	E	450	464	HLA-DRB1*08:02	51%-80%	Dengue virus 4	ELISPOT	IFNg release	Positive	0	0	0	1	0	1
195473	IAVGGITLFLGFTVQ	E	759	773	HLA-DRB1*15:01	51%-80%	Dengue virus 4	ELISPOT	IFNg release	Positive	1	1	1	0	1	0
540051	VLGSQEGAMHSALAGA	E	531	546	HLA class II	51%-80%	Dengue virus 4	ICS	IFNg release	Positive	1	1	1	1	1	0
195608	LAIMAVFKMSPGYVL	NS2a	1200	1214	HLA-DRB1*13:01	51%-80%	Dengue virus 4	ELISPOT	IFNg release	Positive	1	1	1	0	0	0
539664	MGGQIHLAIMAVFKM	NS2a	1194	1208	HLA-DRB1*13:01	51%-80%	Dengue virus 4	ELISPOT	IFNg release	Positive	0	0	0	0	0	0
539930	SRETALMVIGMAMTT	NS2a	1223	1237	HLA-DRB1*15:06	51%-80%	Dengue virus 4	ELISPOT	IFNg release	Positive	1	1	1	0	0	0
539962	TALMVIGMAMTTVLS	NS2a	1226	1240	HLA-DRB1*15:06	51%-80%	Dengue virus 4	ELISPOT	IFNg release	Positive	1	1	1	0	0	0
867874	LMVIGMAMTTVLSIP	NS2a	1228	1242	HLA-DQA1*01:02/DQB1*06:02	51%-80%	Dengue virus 4	ELISPOT	IFNg release	Positive	1	1	1	0	0	0
195962	SIVREALKRRLRTLI	NS3	1680	1694	HLA-DRB1*11:01	51%-80%	Dengue virus 4	ELISPOT	IFNg release	Positive	1	1	1	0	0	0
539859	RKSGKKVIQLSRKTF	NS3	1850	1864	HLA-DRB1*08:03	51%-80%	Dengue virus 4	ELISPOT	IFNg release	Positive	1	1	1	0	0	0
539899	SGKKVIQLSRKTFDT	NS3	1852	1866	HLA-DRB1*12:02	51%-80%	Dengue virus 4	ELISPOT	IFNg release	Positive	1	1	1	0	0	0
195214	DFGFYQVKTETTILD	NS4b	2254	2268	HLA-DRB1*04:01	51%-80%	Dengue virus 4	ELISPOT	IFNg release	Positive	1	1	1	0	0	0
195383	GLAFSLIKNAQTPRR	NS4b	2473	2487	HLA-DRB1*04:01	51%-80%	Dengue virus 4	ELISPOT	IFNg release	Positive	1	1	1	0	0	0
196052	TKTDFGFYQVKTETT	NS4b	2251	2265	HLA-DRB1*04:01	51%-80%	Dengue virus 4	ELISPOT	IFNg release	Positive	1	1	1	0	0	0
738447	AFSLIKNAQTPRRGT	NS4b	2475	2489	HLA-DRB1*14:06	51%-80%	Dengue virus 4	ELISPOT	IFNg release	Positive	1	1	1	0	0	0
738897	FGFYQVKTETTILDV	NS4b	2255	2269	HLA-DRB1*04:07	51%-80%	Dengue virus 4	ELISPOT	IFNg release	Positive	1	1	1	0	0	0
739417	LAFSLIKNAQTPRRG	NS4b	2474	2488	HLA-DRB1*14:02	51%-80%	Dengue virus 4	ELISPOT	IFNg release	Positive	1	1	1	0	0	0
195411	GNIVSSVNTTSKMLL	NS5	2713	2727	HLA-DRB1*04:01	51%-80%	Dengue virus 4	ELISPOT	IFNg release	Positive	1	1	1	0	0	0
539987	TLLCLIPTVMAFHLS	C	103	117	HLA-DRB1*04:03	>0% - 50%	Dengue virus 4	ELISPOT	IFNg release	Positive	0	1	0	0	0	0
195526	IQRTVFFILMMLVAP	PreM	262	276	HLA-DRB1*07:01	>0% - 50%	Dengue virus 4	ELISPOT	IFNg release	Positive	0	0	0	0	0	0
539820	QRTVFFILMMLVAPS	PreM	263	277	HLA-DRB1*13:01	>0% - 50%	Dengue virus 4	ELISPOT	IFNg release	Positive	0	0	0	0	0	0
867174	IGQTGIQRTVFFILM	PreM	257	271	HLA-DPB1*02:01	>0% - 50%	Dengue virus 4	ELISPOT	IFNg release	Positive	0	0	0	0	0	0
195358	GGLFTSLGKAVHQVF	E	705	719	HLA-DRB1*11:01	>0% - 50%	Dengue virus 4	ELISPOT	IFNg release	Positive	1	1	1	0	1	0
195472	IAVGGITLFLGFTVH	E	480	494	HLA-DRB1*15:01	>0% - 50%	Dengue virus 4	ELISPOT	IFNg release	Positive	0	0	0	0	0	1
539298	GGVSWMVRILIGLLV	E	449	463	HLA-DRB1*15:06	>0% - 50%	Dengue virus 4	ELISPOT	IFNg release	Positive	0	0	0	0	0	0
540036	VGGLFTSLGKAVHQV	E	704	718	HLA-DRB1*09:01	>0% - 50%	Dengue virus 4	ELISPOT	IFNg release	Positive	1	1	1	0	1	0
866696	FGMFTTNIWMKFREG	NS1	934	948	HLA-DPB1*04:01	>0% - 50%	Dengue virus 4	ELISPOT	IFNg release	Positive	1	1	1	0	0	0
195220	DLMEFIDGISLGLIL	NS2a	1244	1258	HLA-DRB1*07:01	>0% - 50%	Dengue virus 4	ELISPOT	IFNg release	Positive	0	0	0	0	0	0

196252	YVLGIFLRKLTSRET	NS2a	1212	1226	HLA-DRB1*11:01	>0% - 50%	Dengue virus 4	ELISPOT	IFNg release	Positive	0	0	0	0	0	0
539555	LGIFLRKLTSRETAL	NS2a	1214	1228	HLA-DRB1*08:03	>0% - 50%	Dengue virus 4	ELISPOT	IFNg release	Positive	0	0	0	0	0	0
740701	VVTLIPLCRTSCLQK	NS2a	1299	1313	HLA-DRB1*11:04	>0% - 50%	Dengue virus 4	ELISPOT	IFNg release	Positive	1	1	1	0	0	0
867048	HDLMEFIDGISLGLI	NS2a	1243	1257	HLA-DQA1*01:01/DQB1*05:01	>0% - 50%	Dengue virus 4	ELISPOT	IFNg release	Positive	0	0	0	0	0	0
740013	QLIRQMEAEGVITQD	NS5	3105	3119	HLA-DRB1*01:02	>0% - 50%	Dengue virus 4	ELISPOT	IFNg release	Positive	1	1	1	0	0	0

CD8 DENV-1 Epitopes

											DENV-1 constructs of vaccines (100% identity=1,green ; else 0, red)					
Epitope_ID	AA_Seq	Protein_Name	Start	End	Allele	Step	Serotype	Method/Technique	Assay Group	Qualitative Measure	TV003	TDEN	DPIV	Dengvaxia	DENVax	TVDV
184844	MLKRERNRV	C	15	23	HLA-B*08:01	0%	Dengue virus 1	ELISPOT	IFNg release	Positive	0	0	0	0	0	0
240082	MVLALITFL	C	24	32	HLA-A*02:01	0%	Dengue virus 1	ELISPOT	IFNg release	Positive	0	0	0	0	0	0
180641	MALSIVSLF	NS2a	1297	1305	HLA-B*51:01	0%	Dengue virus 1	ELISPOT	IFNg release	Positive	0	0	0	0	0	0
419037	RPMFAVGILF	NS2a	1211	1220	HLA-B*35:01	0%	Dengue virus 1	ELISPOT	IFNg release	Positive	0	0	0	0	0	0
150254	GPLVAGGLL	NS2b	1375	1383	HLA-B*07:02	0%	Dengue virus 1	ELISPOT	IFNg release	Positive	0	0	0	0	0	0
180682	NPEIEDDIF	NS3	1650	1658	HLA-B*35:01	0%	Dengue virus 1	ELISPOT	IFNg release	Positive	0	0	0	0	0	0
32147	KLRTLVLAPTRVVAS	NS3	216	230	HLA class I	0%	Dengue virus 1	ELISPOT	IFNg release	Positive	1	1	1	0	0	0
539750	NVRTLILAPTRVVASEM	NS3	1690	1706	HLA class I	0%	Dengue virus 1	ICS	IFNg release	Positive	0	0	0	0	0	0
65549	TPEGIIPSM	NS3	1975	1983	HLA-B*53:01	0%	Dengue virus 1	51 chromium	cytotoxicity	Positive	0	0	0	0	0	0
150674	TVMDIISRR	NS5	3080	3088	HLA-A*11:01	0%	Dengue virus 1	multimer/tetramer	qualitative binding	Positive	0	0	0	0	0	0
184787	MEVQLVRQM	NS5	3107	3115	HLA-B*40:01	0%	Dengue virus 1	ELISPOT	IFNg release	Positive	0	0	0	0	0	0
150378	LLMLVTPSM	PreM	271	279	HLA-B*08:01	100%	Dengue virus 1	ELISPOT	IFNg release	Positive	1	1	1	0	1	1
185893	TEVTNPAVL	E	329	337	HLA-B*40:01	100%	Dengue virus 1	ELISPOT	IFNg release	Positive	1	1	1	1	1	1
186551	YENLKYSVIV	E	412	421	HLA-B*40:01	100%	Dengue virus 1	ELISPOT	IFNg release	Positive	1	1	1	1	1	1
50596	QEGAMHTAL	E	536	544	HLA-A*02:01	100%	Dengue virus 1	ELISPOT	IFNg release	Positive	1	1	1	1	1	1
150258	GPWHLGKLEL	NS1	1041	1050	HLA-B*07:02	100%	Dengue virus 1	ELISPOT	IFNg release	Positive	1	1	1	0	0	0
183246	FRRLTSREVL	NS2a	1220	1229	HLA-B*08:01	100%	Dengue virus 1	ELISPOT	IFNg release	Positive	1	1	1	0	0	0
184274	LALMATFKM	NS2a	1202	1210	HLA-B*35:01	100%	Dengue virus 1	ELISPOT	IFNg release	Positive	0	0	0	0	0	0
184856	MLMTGTLAVF	NS2a	1158	1167	HLA-B*35:01	100%	Dengue virus 1	ELISPOT	IFNg release	Positive	1	1	1	0	0	0
13346	EMAEALKGMPIRYQT	NS3	231	245	HLA class I	100%	Dengue virus 1	ELISPOT	IFNg release	Positive	1	1	1	0	0	0
150341	KPGTSGSPI	NS3	1606	1614	HLA-B*07:02	100%	Dengue virus 1	ELISPOT	IFNg release	Positive	1	1	1	0	0	0
150538	RPRWLDART	NS3	2068	2076	HLA-B*07:02	100%	Dengue virus 1	ELISPOT	IFNg release	Positive	1	1	1	0	0	0
180472	GEARKTFVEL	NS3	2003	2012	HLA-B*40:01	100%	Dengue virus 1	ELISPOT	IFNg release	Positive	1	1	1	0	0	0
180804	VPNYNMIIM	NS3	1751	1759	HLA-B*51:01	100%	Dengue virus 1	ELISPOT	IFNg release	Positive	1	1	1	0	0	0
183229	FPQSNAVIQD	NS3	1801	1810	HLA-B*35:01	100%	Dengue virus 1	ELISPOT	IFNg release	Positive	1	1	1	0	0	0
183598	HPGSGKTRRY	NS3	1670	1679	HLA-B*35:01	100%	Dengue virus 1	ELISPOT	IFNg release	Positive	1	1	1	0	0	0
184316	LEENMDVEIW	NS3	2049	2058	HLA-B*44:03	100%	Dengue virus 1	ELISPOT	IFNg release	Positive	1	1	1	0	0	0
184884	MPIRYQTTAV	NS3	1714	1723	HLA-B*35:01	100%	Dengue virus 1	ELISPOT	IFNg release	Positive	1	1	1	0	0	0
185741	SPVRVPNYNM	NS3	1747	1756	HLA-B*35:01	100%	Dengue virus 1	ELISPOT	IFNg release	Positive	1	1	1	0	0	0
185827	SYKVASEGF	NS3	2023	2031	HLA-A*24:02	100%	Dengue virus 1	ELISPOT	IFNg release	Positive	1	1	1	0	0	0
186284	VLDDGIYRI	NS3	1492	1500	HLA-A*02:01	100%	Dengue virus 1	ELISPOT	IFNg release	Positive	1	1	1	0	0	0
186559	YKVASEGFQY	NS3	2024	2033	HLA-B*35:01	100%	Dengue virus 1	ELISPOT	IFNg release	Positive	1	1	1	0	0	0
22825	GTSGSPIVNR	NS3	133	142	HLA-B*57:01	100%	Dengue virus 1	51 chromium	cytotoxicity	Positive	1	1	1	0	0	0
38766	LPVWLSYKV	NS3	2018	2026	HLA-B*51:01	100%	Dengue virus 1	ELISPOT	IFNg release	Positive	1	1	1	0	0	0
39095	LRGEARKTFVELMRR	NS3	526	540	HLA class I	100%	Dengue virus 1	ELISPOT	IFNg release	Positive	1	1	1	0	0	0
419024	NMIIMDEAHF	NS3	1755	1764	HLA-B*15:01	100%	Dengue virus 1	ELISPOT	IFNg release	Positive	1	1	1	0	0	0
56309	RVIDPRRCL	NS3	1897	1905	HLA-B*07:02	100%	Dengue virus 1	ELISPOT	IFNg release	Positive	1	1	1	0	0	0
150649	TPQDNQLAYV	NS4a	2222	2231	HLA-B*07:02	100%	Dengue virus 1	ELISPOT	IFNg release	Positive	1	1	1	0	0	0
180780	TPQDNQLAY	NS4a	2222	2230	HLA-B*35:01	100%	Dengue virus 1	ELISPOT	IFNg release	Positive	1	1	1	0	0	0
184522	LPDTIETLM	NS4a	2142	2150	HLA-B*35:01	100%	Dengue virus 1	ELISPOT	IFNg release	Positive	1	1	1	0	0	0
184523	LPDTIETLML	NS4a	2142	2151	HLA-B*35:01	100%	Dengue virus 1	ELISPOT	IFNg release	Positive	1	1	1	0	0	0
419128	AYRHAMEEL	NS4a	2134	2142	HLA-A*24:02	100%	Dengue virus 1	ELISPOT	IFNg release	Positive-High	1	1	1	0	0	0
41986	MLLALIAVL	NS4a	2150	2158	HLA-A2	100%	Dengue virus 1	ICS	IFNg release	Positive	1	1	1	0	0	0
150474	NPLTLTAAV	NS4b	2348	2356	HLA-B*07:02	100%	Dengue virus 1	ELISPOT	IFNg release	Positive	1	1	1	0	0	0
186427	VVYDAKFEK	NS4b	2403	2411	HLA-A*11:01	100%	Dengue virus 1	ELISPOT	IFNg release	Positive	1	1	1	0	0	0
27180	ILLMRTTWA	NS4b	2426	2434	HLA-A2	100%	Dengue virus 1	ICS	IFNg release	Positive	1	1	1	0	0	0
41093	MANIFRGSY	NS4b	2466	2474	HLA-B*35:01	100%	Dengue virus 1	ELISPOT	IFNg release	Positive	1	1	1	0	0	0
69652	VLMLVAHYA	NS4b	2356	2364	HLA-A2	100%	Dengue virus 1	ICS	IFNg release	Positive	1	1	1	0	0	0
74558	YLAGAGLAF	NS4b	2474	2482	HLA-B*07:02	100%	Dengue virus 1	ELISPOT	IFNg release	Positive	1	1	1	0	0	0
133702	SRNSTHEMY	NS5	2703	2711	HLA-A*02:01	100%	Dengue virus 1	ELISPOT	IFNg release	Positive	1	1	1	0	0	0
14325	ETACLGKSY	NS5	3242	3250	HLA-A*26:01	100%	Dengue virus 1	ELISPOT	IFNg release	Positive	1	1	1	0	0	0
180548	KAKGSRAIW	NS5	2959	2967	HLA-B*57:01	100%	Dengue virus 1	ELISPOT	IFNg release	Positive	1	1	1	0	0	0
182599	AQMWQLMYF	NS5	3251	3259	HLA-B*15:01	100%	Dengue virus 1	ELISPOT	IFNg release	Positive	1	1	1	0	0	0
183147	FCSHHFHQL	NS5	3200	3208	HLA-B*08:01	100%	Dengue virus 1	ELISPOT	IFNg release	Positive	1	1	1	0	0	0
183828	IPMVTQIAM	NS5	2827	2835	HLA-B*07:02	100%	Dengue virus 1	ELISPOT	IFNg release	Positive	1	1	1	0	0	0
184898	MQRKHGGML	NS5	2689	2697	HLA-B*08:01	100%	Dengue virus 1	ELISPOT	IFNg release	Positive	1	1	1	0	0	0
184956	MTTEDMLSVW	NS5	3296	3305	HLA-B*58:01	100%	Dengue virus 1	ICS	IFNg release	Positive	1	1	1	0	0	0
185279	QMQRKHGGML	NS5	2688	2697	HLA-B*08:01	100%	Dengue virus 1	ELISPOT	IFNg release	Positive	1	1	1	0	0	0
185354	REIVVPCRNQ	NS5	3214	3223	HLA-B*40:01	100%	Dengue virus 1	ELISPOT	IFNg release	Positive	1	1	1	0	0	0
186150	TVMDVISRR	NS5	3080	3088	HLA-A*68:01	100%	Dengue virus 1	ELISPOT	IFNg release	Positive	1	1	1	0	0	0
186176	TWSIHAHHQW	NS5	3286	3295	HLA-A*23:01	100%	Dengue virus 1	ELISPOT	IFNg release	Positive	1	1	1	0	0	0
73076	WSIHAHHQW	NS5	3287	3295	HLA-B*57:01	100%	Dengue virus 1	ELISPOT	IFNg release	Positive	1	1	1	0	0	0
180637	MAFIAFLRF	C	48	56	HLA-B*35:01	81% - <100%	Dengue virus 1	ELISPOT	IFNg release	Positive	1	1	1	0	0	0
183175	FIAFLRFLA	C	50	58	HLA-A*68:02	81% - <100%	Dengue virus 1	ELISPOT	IFNg release	Positive	1	1	1	0	0	0
183220	FNMLKRARNR	C	14	23	HLA-A*33:01	81% - <100%	Dengue virus 1	ELISPOT	IFNg release	Positive	1	1	1	0	0	0
183800	IMNRRKRSV	C	94	102	HLA-B*08:01	81% - <100%	Dengue virus 1	ELISPOT	IFNg release	Positive	1	1	1	0	0	0
184458	LLMLLPTAL	C	83	91	HLA-B*08:01	81% - <100%	Dengue virus 1	ELISPOT	IFNg release	Positive	1	1	1	0	0	0
184880	MNRRKRSVT	C	95	103	HLA-B*08:01	81% - <100%	Dengue virus 1	ELISPOT	IFNg release	Positive	1	1	1	0	0	0
185102	NRRKRSVTM	C	96	104	HLA-B*08:01	81% - <100%	Dengue virus 1	ELISPOT	IFNg release	Positive	1	1	1	0	0	0
185492	RRKRSVTMLL	C	97	106	HLA-B*08:01	81% - <100%	Dengue virus 1	ELISPOT	IFNg release	Positive	1	1	1	0	0	0
183596	HPGFTVIALF	PreM	244	253	HLA-B*35:01	81% - <100%	Dengue virus 1	ELISPOT	IFNg release	Positive	1	1	1	0	1	1
16554	FLDLPLPWT	E	213	221	HLA-A2	81% - <100%	Dengue virus 1	ICS	IFNg release	Positive	1	1	1	1	1	1
183421	GLNSRSTSL	E	747	755	HLA-B*08:01	81% - <100%	Dengue virus 1	ELISPOT	IFNg release	Positive	1	1	1	1	0	1
183654	IAVGMVTLY	E	760	768	HLA-B*35:01	81% - <100%	Dengue virus 1	ELISPOT	IFNg release	Positive	1	1	1	0	1	1
184377	LGLNSRSTSL	E	746	755	HLA-B*08:01	81% - <100%	Dengue virus 1	ELISPOT	IFNg release	Positive	1	1	1	1	0	1
184626	LTDYGALTL	E	456	464	HLA-A*01:01	81% - <100%	Dengue virus 1	ELISPOT	IFNg release	Positive	1	1	1	1	0	1
185212	PTSEIQLTDY	E	449	458	HLA-A*01:01	81% - <100%	Dengue virus 1	ELISPOT	IFNg release	Positive	1	1	1	1	1	1
419306	PTLDIELLK	E	319	327	HLA-A*11:01	81% - <100%	Dengue virus 1	ELISPOT	IFNg release	Positive	1	1	1	1	1	1
588005	RDFVEGLSGATWVDVVL	E	289	305	HLA class I	81% - <100%	Dengue virus 1	ICS	IFNg release	Positive	1	1	1	1	1	1
590597	VEGLSGATWVDVVLEHG	E	292	308	HLA class I	81% - <100%	Dengue virus 1	ICS	IFNg release	Positive-Intermediate	1	1	1	1	1	1
180836	YGGPISQHNY	NS1	1022	1031	HLA-B*35:01	81% - <100%	Dengue virus 1	ELISPOT	IFNg release	Positive	1	1	1	0	0	0
183619	HTWTEQYKF	NS1	801	809	HLA-B57	81% - <100%	Dengue virus 1	51 chromium	cytotoxicity	Positive	1	1	1	0	0	0
60104	SPKRLSAAI	NS1	813	821	HLA-B*08:01	81% - <100%	Dengue virus 1	ELISPOT	IFNg release	Positive	1	1	1	0	0	0
184623	LTDFQSHQL	NS2a	1264	1272	HLA-A*01:01	81% - <100%	Dengue virus 1	ELISPOT	IFNg release	Positive	1	1	1	1	0	0
185468	RPMFAVGLLF	NS2a	1211	1220	HLA-B*35:01	81% - <100%	Dengue virus 1	ELISPOT	IFNg release	Positive	1	1	1	0	0	0
185651	SILLSSLLK	NS2b	1360	1368	HLA-A*11:01	81% - <100%	Dengue virus 1	ELISPOT	IFNg release	Positive	1	1	1	1	0	0
133501	AIVREAIKR	NS3	1682	1690	HLA-A*02:01	81% - <100%	Dengue virus 1	ELISPOT	IFNg release	Positive	1	1	1	0	0	0
150169	DPRRCLKPV	NS3	1900	1908	HLA-B*07:02	81% - <100%	Dengue virus 1	ELISPOT	IFNg release	Positive	1	1	1	0	0	0
150556	RVIDPRRCLK	NS3	1897	1906	HLA-A*03:01	81% - <100%	Dengue virus 1	ELISPOT	IFNg release	Positive	1	1	1	0	0	0
150643	TPEGIIPALF	NS3	1976	1985	HLA-B*35:01	81% - <100%	Dengue virus 1	ELISPOT	IFNg release	Positive	1	1	1	0	0	0
167826	TSGTYVSAIAQAKASQE	NS3	157	173	HLA class I	81% - <100%	Dengue virus 1	ELISPOT	IFNg release	Positive	1	1	1	0	0	0
180373	APTRVVASEM	NS3	1698	1707	HLA class I	81% - <100%	Dengue virus 1	ELISPOT	IFNg release	Positive	1	1	1	0	0	0
180408	DPASIAARGY	NS3	1766	1775	HLA-B*35:01	81% - <100%	Dengue virus 1	ELISPOT	IFNg release	Positive	1	1	1	0	0	0
180423	EERDIPERSW	NS3	1811	1820	HLA-B*44:02	81% - <100%	Dengue virus 1	ELISPOT	IFNg release	Positive	1	1	1	0	0	0
182519	AIKRKLRTL	NS3	1687	1695	HLA-B*08:01	81% - <100%	Dengue virus 1	ELISPOT	IFNg release	Positive	1	1	1	0	0	0
182772	DISEMGANF	NS3	1885	1893	HLA-A*26:01	81% - <100%	Dengue virus 1	ELISPOT	IFNg release	Positive	1	1	1	0	0	0
183223	FPGKTVWFVP	NS3	1830	1839	HLA-B*35:01	81% - <100%	Dengue virus 1	ELISPOT	IFNg release	Positive	1	1	1	0	0	0
183929	IYRILQRGLL	NS3	1497	1506	HLA-B*08:01	81% - <100%	Dengue virus 1	ELISPOT	IFNg release	Positive	1	1	1	0	0	0
185124	NTPEGIIPA	NS3	1975	1983	HLA-A*68:02	81% - <100%	Dengue virus 1	ELISPOT	IFNg release	Positive	1	1	1	0	0	0
185125	NTPEGIIPAL	NS3	1975	1984	HLA-A*68:02	81% - <100%	Dengue virus 1	ELISPOT	IFNg release	Positive	1	1	1	0	0	0
185327	QYIYMGQPL	NS3	1947	1955	HLA-A*24:02	81% - <100%	Dengue virus 1	ELISPOT	IFNg release	Positive	1	1	1	0	0	0
186603	YSDPLALREF	NS3	2077	2086	HLA-B*35:01	81% - <100%	Dengue virus 1	ELISPOT	IFNg release	Positive	1	1	1	0	0	0
35237	LDNINTPEGIIPALFEPERE	NS3	496	515	HLA class I	81% - <100%	Dengue virus 1	ELISPOT	IFNg release	Positive	1	1	1	0	0	0
38400	LPAIVREAI	NS3	1680	1688	HLA-B*53:01	81% - <100%	Dengue virus 1	ELISPOT	IFNg release	Positive	1	1	1	0	0	0
419124	AVEPGKNPK	NS3	1574	1582	HLA-A*11:01	81% - <100%	Dengue virus 1	ELISPOT	IFNg release	Positive	1	1	1	0	0	0
419229	IYRILQRGL	NS3	1497	1505	HLA-A*24:02	81% - <100%	Dengue virus 1	ELISPOT	IFNg release	Positive	1	1	1	0	0	0
50894	QGKRLEPSWASVKKDLISYG	NS3	1536	1555	HLA class I	81% - <100%	Dengue virus 1	ELISPOT	IFNg release	Positive	1	1	1	0	0	0
539170	DLMCHATF	NS3	1734	1741	HLA-B*08:01	81% - <100%	Dengue virus 1	ICS	IFNg release	Positive	1	1	1	0	0	0
539663	MGEAAAIFMTATPPGSV	NS3	1782	1798	HLA class I	81% - <100%	Dengue virus 1	ICS	IFNg release	Positive	1	1	1	0	0	0
539753	NYNMIIMDEAHFTDPA	NS3	1753	1768	HLA class I	81% - <100%	Dengue virus 1	ICS	IFNg release	Positive	1	1	1	0	0	0
585710	LAPTRVVASEM	NS3	83	93	HLA class I	81% - <100%	Dengue virus 1	ICS	IFNg release	Positive-High	1	1	1	0	0	0
62184	SVKKDLISYGGGWRF	NS3	71	85	HLA class I	81% - <100%	Dengue virus 1	ELISPOT	IFNg release	Positive	1	1	1	0	0	0
65546	TPEGIIPAL	NS3	1976	1984	HLA class I	81% - <100%	Dengue virus 1	51 chromium	cytotoxicity	Positive	1	1	1	0	0	0
182912	EFFLMVLLI	NS4a	2206	2214	HLA-A*24:02	81% - <100%	Dengue virus 1	ELISPOT	IFNg release	Positive	1	1	1	0	0	0
183162	FFLMVLLIPE	NS4a	2207	2216	HLA-A*33:01	81% - <100%	Dengue virus 1	ELISPOT	IFNg release	Positive	1	1	1	0	0	0
183813	IPEPDRQRTP	NS4a	2214	2223	HLA-B*35:01	81% - <100%	Dengue virus 1	ELISPOT	IFNg release	Positive	1	1	1	0	0	0
184739	MASSVLLWM	NS4a	2183	2191	HLA-B*35:01	81% - <100%	Dengue virus 1	ELISPOT	IFNg release	Positive	0	0	0	0	0	0
133716	TLYAVATTI	NS4b	2284	2292	HLA-A*02:01	81% - <100%	Dengue virus 1	ELISPOT	IFNg release	Positive	1	1	1	0	0	0
180382	ATGPLTTLW	NS4b	2442	2450	HLA-B*58:01	81% - <100%	Dengue virus 1	ELISPOT	IFNg release	Positive	1	1	1	0	0	0
183594	HPASAWTLYA	NS4b	2278	2287	HLA-B*35:01	81% - <100%	Dengue virus 1	ELISPOT	IFNg release	Positive	1	1	1	0	0	0

64286	TIENTTANISLTAIA	NS4b	2300	2314	HLA class I	81% - <100%	Dengue virus 1	ELISPOT	IFNg release	Positive	1	1	1	0	0	0
1068332	SSWEDVPYL	NS5	3323	3331	HLA-A*02:01	81% - <100%	Dengue virus 1	ELISPOT	IFNg release	Positive	1	1	1	0	0	0
180376	ASSMVNGVVR	NS5	2809	2818	HLA-B*57:01	81% - <100%	Dengue virus 1	ELISPOT	IFNg release	Positive	1	1	1	0	0	0
180411	DTTPFGQQR	NS5	2837	2845	HLA-A*68:01	81% - <100%	Dengue virus 1	ELISPOT	IFNg release	Positive	1	1	1	0	0	0
180571	KPRICTREEF	NS5	2882	2891	HLA-B*07:02	81% - <100%	Dengue virus 1	ELISPOT	IFNg release	Positive	1	1	1	0	0	0
180723	RFLEFEALGF	NS5	2974	2983	HLA-A*23:01	81% - <100%	Dengue virus 1	ELISPOT	IFNg release	Positive	1	1	1	0	0	0
182554	ALLATSIFK	NS5	3054	3062	HLA-A*11:01	81% - <100%	Dengue virus 1	ELISPOT	IFNg release	Positive	1	1	1	0	0	0
182836	DYMTSMKRF	NS5	3373	3381	HLA-A*24:02	81% - <100%	Dengue virus 1	ELISPOT	IFNg release	Positive	0	0	0	0	0	0
183275	FTNMEVQLIR	NS5	3104	3113	HLA-B*35:01	81% - <100%	Dengue virus 1	ELISPOT	IFNg release	Positive	0	0	0	0	0	0
184212	KVRKDIPQW	NS5	3178	3186	HLA-B*57:01	81% - <100%	Dengue virus 1	ELISPOT	IFNg release	Positive	1	1	1	0	0	0
539702	MVTQIAMTDTTPFGQQR	NS5	2829	2845	HLA class I	81% - <100%	Dengue virus 1	ICS	IFNg release	Positive	1	1	1	0	0	0
62903	TAKWLWGFLSRNKKPRICTR	NS5	2869	2888	HLA class I	81% - <100%	Dengue virus 1	ELISPOT	IFNg release	Positive	0	0	0	0	0	0
65567	TPFGQQRVF	NS5	2839	2847	HLA class I	81% - <100%	Dengue virus 1	ELISPOT	IFNg release	Positive	1	1	1	0	0	0
72563	WHYDEDNPYKTWAYHGSYEV	NS5	2784	2803	HLA class I	81% - <100%	Dengue virus 1	ELISPOT	IFNg release	Positive	1	1	1	0	0	0
184003	KEISSMLNIM	C	86	95	HLA-B*40:01	51%-80%	Dengue virus 1	ELISPOT	IFNg release	Positive	0	0	0	0	0	0
186524	WTMKIGIGIL	E	733	742	HLA-B*08:01	51%-80%	Dengue virus 1	ELISPOT	IFNg release	Positive	1	1	1	0	1	1
184736	MAMVLSIVS	NS2a	1295	1303	HLA-B*35:01	51%-80%	Dengue virus 1	ELISPOT	IFNg release	Positive	0	0	0	1	0	0
419005	LLAVSGVYPM	NS2b	1448	1457	HLA-B*15:01	51%-80%	Dengue virus 1	ELISPOT	IFNg release	Positive	0	0	0	0	0	0
184849	MLLNRFTMA	NS5	24	32	HLA-B*08:01	51%-80%	Dengue virus 1	ELISPOT	IFNg release	Positive	1	1	1	0	0	0
186217	VEDERFWDL	NS5	2918	2926	HLA-B*40:01	51%-80%	Dengue virus 1	ELISPOT	IFNg release	Positive	1	1	1	0	0	0
419208	GVEGEGLHK	NS5	2998	3006	HLA-A*11:01	51%-80%	Dengue virus 1	ELISPOT	IFNg release	Positive-High	1	1	1	0	0	0
150255	GPMKLVMAFI	C	43	52	HLA-B*07:02	>0% - 50%	Dengue virus 1	ELISPOT	IFNg release	Positive	1	1	1	0	0	0
184835	MKLVMAFIAF	C	44	53	HLA-B*35:01	>0% - 50%	Dengue virus 1	ELISPOT	IFNg release	Positive	1	1	1	0	0	0
183245	FQSHQLWATL	NS2a	1267	1276	HLA-B*15:01	>0% - 50%	Dengue virus 1	ELISPOT	IFNg release	Positive	1	1	1	0	0	0
183252	FSLHYAWKTM	NS2a	1286	1295	HLA-B*35:01	>0% - 50%	Dengue virus 1	ELISPOT	IFNg release	Positive	1	1	1	1	0	0
180670	MPVTVASAAQ	NS3	1923	1932	HLA-B*35:01	>0% - 50%	Dengue virus 1	ELISPOT	IFNg release	Positive	1	1	1	0	0	0
184896	MPVTVASAA	NS3	1923	1931	HLA-B*35:01	>0% - 50%	Dengue virus 1	ELISPOT	IFNg release	Positive	1	1	1	0	0	0
419125	AVKSEHTGR	NS3	1722	1730	HLA-A*11:01	>0% - 50%	Dengue virus 1	ELISPOT	IFNg release	Positive	0	0	0	0	0	0
184554	LPQHLTQRAQ	NS4a	2107	2116	HLA-B*35:01	>0% - 50%	Dengue virus 1	ELISPOT	IFNg release	Positive	1	1	1	0	0	0
150579	SPGKFWNTTI	NS4b	2453	2462	HLA-B*07:02	>0% - 50%	Dengue virus 1	ELISPOT	IFNg release	Positive	1	1	1	0	0	0
150308	IPMATYGWNL	NS5	2607	2616	HLA-B*07:02	>0% - 50%	Dengue virus 1	ELISPOT	IFNg release	Positive	1	1	1	0	0	0
185485	RQMESEGIFL	NS5	3113	3122	HLA-B*15:01	>0% - 50%	Dengue virus 1	ELISPOT	IFNg release	Positive	0	0	0	0	0	0
5223	ATYGWNLVK	NS5	2610	2618	HLA-A*03:01	>0% - 50%	Dengue virus 1	ELISPOT	IFNg release	Positive	1	1	1	0	0	0
539435	ISGDDCVVKPIDDRFAT	NS5	3152	3168	HLA class I	>0% - 50%	Dengue virus 1	ICS	IFNg release	Positive	1	1	1	0	0	0
540100	VVKPIDDRFATALTALN	NS5	3157	3173	HLA class I	>0% - 50%	Dengue virus 1	ICS	IFNg release	Positive	1	1	1	0	0	0
67362	TYGWNLVKL	NS5	2611	2619	HLA-A*24:02	>0% - 50%	Dengue virus 1	ELISPOT	IFNg release	Positive	1	1	1	0	0	0

CD8 DENV-2 Epitopes

Epitope_ID	AA_Seq	Protein_Name	Start	End	Allele	Step	Serotype	Method/Technique	Assay Group	Qualitative Measure	DENV-2 constructs of vaccines (100% identity=1,green ; else 0, red)					
											TV003	TDEN	DPIV	Dengvaxia	DENVax	TVDV
185399	RKKARSTPF	C	5	13	HLA-B*08:01	0%	Dengue virus 2	ELISPOT	IFNg release	Positive	0	0	0	0	0	0
180818	WILRHPGFTMMAAIL	PreM	126	140	HLA class I	0%	Dengue virus 2	ICS	IFNg release	Positive	0	0	0	0	1	0
183577	HFQRALIFIL	PreM	262	271	HLA-B*08:01	0%	Dengue virus 2	ELISPOT	IFNg release	Positive	1	1	1	1	1	1
183621	HVQRIETWIL	PreM	233	242	HLA-B*08:01	0%	Dengue virus 2	ELISPOT	IFNg release	Positive	0	0	0	0	1	0
186264	VGRQEKGKSL	PreM	128	137	HLA-B*08:01	0%	Dengue virus 2	ELISPOT	IFNg release	Positive	0	0	0	0	0	0
180563	KILIGVIITWIGMNS	E	456	470	HLA class I	0%	Dengue virus 2	ICS	IFNg release	Positive	1	1	1	1	1	0
183747	ILIGVIITW	E	737	745	HLA-B*58:01	0%	Dengue virus 2	ELISPOT	IFNg release	Positive	1	1	1	1	1	0
184300	LATLRKYCI	E	53	61	HLA-B*08:01	0%	Dengue virus 2	ELISPOT	IFNg release	Positive	0	0	0	0	0	0
180496	GVFTTNIWLKLKEKQ	NS1	936	950	HLA class I	0%	Dengue virus 2	ICS	IFNg release	Positive	0	1	0	0	1	0
150448	MMATIGIAL	NS2a	1229	1237	HLA-B*35:01	0%	Dengue virus 2	ELISPOT	IFNg release	Positive	0	0	0	0	0	0
150449	MMATIGIALL	NS2a	1229	1238	HLA-A*02:01	0%	Dengue virus 2	ELISPOT	IFNg release	Positive	0	0	0	0	0	0
182687	AYRIKQRGIL	NS3	1497	1506	HLA-B*08:01	0%	Dengue virus 2	ELISPOT	IFNg release	Positive	0	0	0	0	0	0
182892	EENMEVEIW	NS3	2049	2057	HLA-B*44:02	0%	Dengue virus 2	ELISPOT	IFNg release	Positive	0	0	0	0	0	0
184318	LEENMEVEIW	NS3	2048	2057	HLA-B*44:02	0%	Dengue virus 2	ELISPOT	IFNg release	Positive	0	0	0	0	0	0
186211	VDRKGKVVGL	NS3	1615	1624	HLA-B*08:01	0%	Dengue virus 2	ELISPOT	IFNg release	Positive	0	0	0	0	0	0
186301	VLMHRGKRI	NS3	1532	1540	HLA-B*08:01	0%	Dengue virus 2	ELISPOT	IFNg release	Positive	0	0	0	0	0	0
22816	GTSGSPIADK	NS3	1608	1617	HLA-A*11:01	0%	Dengue virus 2	multimer/tetramer	qualitative binding	Positive	0	0	0	0	0	0
22817	GTSGSPIADKK	NS3	1608	1618	HLA-A11	0%	Dengue virus 2	ELISPOT	IFNg release	Positive	0	0	0	0	0	0
22824	GTSGSPIVDR	NS3	1608	1617	HLA-A*11:01	0%	Dengue virus 2	51 chromium	cytotoxicity	Positive	0	0	0	0	0	0
46575	NYADRKWCF	NS3	2031	2039	HLA-A24	0%	Dengue virus 2	51 chromium	cytotoxicity	Positive	0	0	0	0	0	0
65548	TPEGIIPSL	NS3	1975	1983	HLA-B35	0%	Dengue virus 2	51 chromium	cytotoxicity	Positive	0	0	0	0	0	0
150373	LLILCVTQV	NS4b	2416	2424	HLA-A*02:01	0%	Dengue virus 2	ELISPOT	IFNg release	Positive	0	0	0	0	0	0
150445	MLLILCVTQV	NS4b	2415	2424	HLA-A*02:01	0%	Dengue virus 2	ELISPOT	IFNg release	Positive	0	0	0	0	0	0
150639	TLYAVATTFV	NS4b	2282	2291	HLA-A*02:01	0%	Dengue virus 2	ELISPOT	IFNg release	Positive	0	1	1	0	1	0
180601	LILCVTQVLM	NS4b	2417	2426	HLA-B*35:01	0%	Dengue virus 2	ELISPOT	IFNg release	Positive	0	0	0	0	0	0
180794	VATTFVTPM	NS4b	2286	2294	HLA-B*35:01	0%	Dengue virus 2	ELISPOT	IFNg release	Positive	0	1	1	0	1	0
184439	LLILCVTQVL	NS4b	2416	2425	HLA-B*08:01	0%	Dengue virus 2	ELISPOT	IFNg release	Positive	0	0	0	0	0	0
150698	VPYLGKREDQ	NS5	3327	3336	HLA-B*07:02	0%	Dengue virus 2	ELISPOT	IFNg release	Positive	0	0	0	0	0	0
163620	TITEEIAVQ	NS5	3127	3135	HLA-A*02:01	0%	Dengue virus 2	ELISPOT	IFNg release	Positive	0	0	0	0	1	0
184234	KWKSRLNAL	NS5	2503	2511	HLA-B*08:01	0%	Dengue virus 2	ELISPOT	IFNg release	Positive	0	1	1	0	1	0
32939	KPWDIIPMV	NS5	2821	2829	HLA-B*55:02	0%	Dengue virus 2	51 chromium	cytotoxicity	Positive	0	0	0	0	0	0
32941	KPWDVLPMV	NS5	2821	2829	HLA class I	0%	Dengue virus 2	51 chromium	cytotoxicity	Positive	0	0	0	0	0	0
32942	KPWDVLPTV	NS5	2821	2829	HLA class I	0%	Dengue virus 2	51 chromium	cytotoxicity	Positive	0	0	0	0	0	0
150252	GPLKLFMAL	C	42	50	HLA-B*08:01	100%	Dengue virus 2	ELISPOT	IFNg release	Positive	0	1	1	0	1	0
150634	TLMAMDLGEL	PreM	149	158	HLA-A*02:01	100%	Dengue virus 2	ELISPOT	IFNg release	Positive	1	1	1	1	1	1
150456	MSYSMCTGKF	E	577	586	HLA-B*35:01	100%	Dengue virus 2	ELISPOT	IFNg release	Positive	1	1	1	1	1	1
150531	RLRMDKLQL	E	566	574	HLA-B*08:01	100%	Dengue virus 2	ELISPOT	IFNg release	Positive	1	1	1	1	1	1
16553	FLDLPLPWL	E	213	221	HLA-A2	100%	Dengue virus 2	ICS	TNF release	Positive	1	1	1	1	1	1
180618	LPLPWLPGAD	E	496	505	HLA-B*35:01	100%	Dengue virus 2	ELISPOT	IFNg release	Positive	1	1	1	1	1	1
180726	RMAILGDTAWDFGSL	E	691	705	HLA-B7	100%	Dengue virus 2	ICS	IFNg release	Positive	1	1	1	1	1	0
180816	WGNGCGLFGKGGIVT	E	381	395	HLA class I	100%	Dengue virus 2	ICS	IFNg release	Positive	1	1	1	1	1	1
182846	EAKQPATLR	E	329	337	HLA-A*68:01	100%	Dengue virus 2	ELISPOT	IFNg release	Positive	1	1	1	1	1	1
183437	GMNSRSTSL	E	747	755	HLA-B*08:01	100%	Dengue virus 2	ELISPOT	IFNg release	Positive	1	1	1	1	1	0
183708	IGMNSRSTSL	E	746	755	HLA-B*08:01	100%	Dengue virus 2	ELISPOT	IFNg release	Positive	1	1	1	1	1	0
183841	IQMSSGNLLF	E	550	559	HLA-A*23:01	100%	Dengue virus 2	ELISPOT	IFNg release	Positive	1	1	1	1	1	1
184571	LQLKGMSYSM	E	572	581	HLA-B*08:01	100%	Dengue virus 2	ELISPOT	IFNg release	Positive	1	1	1	1	1	1
184899	MRGAKRMAI	E	686	694	HLA-B*08:01	100%	Dengue virus 2	ELISPOT	IFNg release	Positive	1	1	1	1	1	0
184900	MRGAKRMAIL	E	686	695	HLA-B*08:01	100%	Dengue virus 2	ELISPOT	IFNg release	Positive	1	1	1	1	1	0
184920	MSSGNLLFTG	E	552	561	HLA-B*58:01	100%	Dengue virus 2	ELISPOT	IFNg release	Positive	1	1	1	1	1	1
185758	SRSTSLSVSL	E	750	759	HLA-B*08:01	100%	Dengue virus 2	ELISPOT	IFNg release	Positive	1	1	1	1	0	0
185831	SYSMCTGKF	E	578	586	HLA-A*23:01	100%	Dengue virus 2	ELISPOT	IFNg release	Positive	1	1	1	1	1	1
27061	ILGDTAWDFG	E	96	105	HLA class I	100%	Dengue virus 2	ELISPOT	IFNg release	Positive	1	1	1	1	1	0
51388	QLKGMSYSM	E	573	581	HLA-B*08:01	100%	Dengue virus 2	ELISPOT	IFNg release	Positive	1	1	1	1	1	1
583702	GVSGGSWVDIVLEHGSCV	E	14	31	HLA class I	100%	Dengue virus 2	ICS	IFNg release	Positive-Low	1	1	1	1	1	1
150259	GPWHLGKLEM	NS1	1041	1050	HLA-B*35:01	100%	Dengue virus 2	ELISPOT	IFNg release	Positive	0	1	1	0	1	0
184512	LMWKQITPEL	NS1	841	850	HLA-B*08:01	100%	Dengue virus 2	ELISPOT	IFNg release	Positive	0	1	1	0	1	0
185465	RPLKEKEENL	NS1	1111	1120	HLA-B*08:01	100%	Dengue virus 2	ELISPOT	IFNg release	Positive	0	1	1	0	1	0
186474	WKTWGKAKML	NS1	890	899	HLA-B*08:01	100%	Dengue virus 2	ELISPOT	IFNg release	Positive	0	1	1	0	1	0
19225	GEDGCWYGM	NS1	1100	1108	HLA-B*40:01	100%	Dengue virus 2	ELISPOT	IFNg release	Positive	0	1	1	0	1	0
150415	LTDALALGM	NS2a	1250	1258	HLA-A*01:01	100%	Dengue virus 2	ELISPOT	IFNg release	Positive	0	1	1	0	1	0
180671	MSFRDLGRVM	NS2a	1175	1184	HLA-B*35:01	100%	Dengue virus 2	ELISPOT	IFNg release	Positive	0	0	1	0	1	0
150304	IMAVGMVSI	NS2b	1353	1361	HLA-A*02:01	100%	Dengue virus 2	ELISPOT	IFNg release	Positive	0	1	1	0	1	0
180531	IPMTGPLVAG	NS2b	1371	1380	HLA-B*35:01	100%	Dengue virus 2	ELISPOT	IFNg release	Positive	0	1	1	0	1	0
180646	MAVGMVSIL	NS2b	1354	1362	HLA-B*35:01	100%	Dengue virus 2	ELISPOT	IFNg release	Positive	0	1	1	0	1	0
183779	IMAVGMVSIL	NS2b	1353	1362	HLA-B*08:01	100%	Dengue virus 2	ELISPOT	IFNg release	Positive	0	1	1	0	1	0
11125	EALRGLPIR	NS3	233	241	HLA class I	100%	Dengue virus 2	ELISPOT	IFNg release	Positive	0	1	1	0	1	0
13358	EMEEALRGLPIRYQT	NS3	230	244	HLA class I	100%	Dengue virus 2	ELISPOT	IFNg release	Positive	0	1	1	0	1	0
136890	TPPGSRDPF	NS3	1792	1800	HLA-B*35:01	100%	Dengue virus 2	ELISPOT	IFNg release	Positive	0	1	1	0	1	0
150137	APTRVVAAEM	NS3	222	231	HLA-B7	100%	Dengue virus 2	multimer/tetramer	qualitative binding	Positive	0	1	1	0	1	0
150217	FPQSNAPIM	NS3	1800	1808	HLA-B*35:01	100%	Dengue virus 2	ELISPOT	IFNg release	Positive	0	0	1	0	0	0
150526	RIYSDPLALK	NS3	2074	2083	HLA-A*11:01	100%	Dengue virus 2	ELISPOT	IFNg release	Positive	0	1	1	0	1	0
150595	SQIGAGVYK	NS3	1509	1517	HLA-A*11:01	100%	Dengue virus 2	ELISPOT	IFNg release	Positive	0	1	1	0	1	0
150644	TPEGIIPSMF	NS3	1975	1984	HLA-B*53:01	100%	Dengue virus 2	ELISPOT	IFNg release	Positive	0	1	1	0	1	0
150693	VPNYNLIIM	NS3	1750	1758	HLA-B*07:02	100%	Dengue virus 2	ELISPOT	IFNg release	Positive	0	1	1	0	1	0
150740	YLPAIVREA	NS3	1678	1686	HLA-A*02:01	100%	Dengue virus 2	ELISPOT	IFNg release	Positive	0	1	1	0	1	0
180360	AAEGINYADRRWCFD	NS3	2026	2040	HLA-A24	100%	Dengue virus 2	ELISPOT	IFNg release	Positive	0	1	1	0	1	0
180407	DNINTPEGIIPSMFE	NS3	496	510	HLA-B35	100%	Dengue virus 2	ELISPOT	IFNg release	Positive	0	1	1	0	1	0
180428	EGEWKEGEEVQVLAL	NS3	1561	1575	HLA class I	100%	Dengue virus 2	ICS	IFNg release	Positive	0	1	1	0	1	0
180440	EREIPERSWNSGHEW	NS3	336	350	HLA-B*57:01	100%	Dengue virus 2	ELISPOT	IFNg release	Positive	0	1	0	0	1	0
180460	FPQSNAPIMD	NS3	1800	1809	HLA-B*35:01	100%	Dengue virus 2	ELISPOT	IFNg release	Positive	0	0	1	0	0	0
180503	HPGAGKTKRY	NS3	1669	1678	HLA-B*35:01	100%	Dengue virus 2	ELISPOT	IFNg release	Positive	0	1	1	0	1	0
180507	HTGREIVDLMCHATF	NS3	1726	1740	HLA class I	100%	Dengue virus 2	ELISPOT	IFNg release	Positive	0	1	1	0	1	0
180575	KRYLPAIVREAIKRG	NS3	201	215	HLA-A*31:01	100%	Dengue virus 2	ELISPOT	IFNg release	Positive	0	1	1	0	1	0
180589	LDARIYSDPLALKEF	NS3	596	610	HLA class I	100%	Dengue virus 2	ELISPOT	IFNg release	Positive	0	1	1	0	1	0
180605	LMCHATFTM	NS3	1734	1742	HLA-B*15:01	100%	Dengue virus 2	ELISPOT	IFNg release	Positive	0	1	1	0	1	0
180617	LPIRYQTPAI	NS3	1713	1722	HLA-B*35:01	100%	Dengue virus 2	ELISPOT	IFNg release	Positive	0	1	1	0	1	0
180647	MEEALRGLPIRYQTP	NS3	1706	1720	HLA class I	100%	Dengue virus 2	ELISPOT	IFNg release	Positive	0	1	1	0	1	0
180734	RSWNSGHEW	NS3	342	350	HLA-B*58:01	100%	Dengue virus 2	ICS	IFNg release	Positive	0	1	0	0	1	0
182898	EEREIPERSW	NS3	1810	1819	HLA-B*44:03	100%	Dengue virus 2	ELISPOT	IFNg release	Positive	0	1	1	0	1	0
183724	IKRGLRTLIL	NS3	1687	1696	HLA-B*08:01	100%	Dengue virus 2	ELISPOT	IFNg release	Positive	0	1	1	0	1	0
185200	PRRCMKPVIL	NS3	1900	1909	HLA-B*08:01	100%	Dengue virus 2	ELISPOT	IFNg release	Positive	0	1	1	0	1	0
26965	ILAPTRVVAAEMEEA	NS3	220	234	HLA class I	100%	Dengue virus 2	ELISPOT	IFNg release	Positive	0	1	1	0	1	0
27770	INYADRRWCF	NS3	555	564	HLA-A24	100%	Dengue virus 2	ELISPOT	IFNg release	Positive	0	1	1	0	1	0
2859	ALRGLPIRY	NS3	234	242	HLA-A*03:01	100%	Dengue virus 2	51 chromium	cytotoxicity	Positive	0	1	1	0	1	0
35238	LDNINTPEGIIPSMF	NS3	495	509	HLA class I	100%	Dengue virus 2	ELISPOT	IFNg release	Positive	0	1	1	0	1	0
46576	NYADRRWCF	NS3	556	564	HLA-A24	100%	Dengue virus 2	ELISPOT	IFNg release	Positive	0	1	1	0	1	0
539620	LRTLILAPTRVVAAEM	NS3	1691	1706	HLA class I	100%	Dengue virus 2	ICS	IFNg release	Positive	0	1	1	0	1	0
539751	NYNLIIMDEAHFTDPA	NS3	1752	1767	HLA class I	100%	Dengue virus 2	ICS	IFNg release	Positive	0	1	1	0	1	0
539752	NYNLIIMDEAHFTDPASI	NS3	1752	1769	HLA class I	100%	Dengue virus 2	ICS	IFNg release	Positive	0	1	1	0	1	0
539985	TLILAPTRVVAAEMEEA	NS3	1693	1709	HLA class I	100%	Dengue virus 2	ICS	IFNg release	Positive	0	1	1	0	1	0
5542	AVSLDFSPGTSGSPI	NS3	125	139	HLA class I	100%	Dengue virus 2	ELISPOT	IFNg release	Positive	0	1	1	0	1	0
56310	RVIDPRRCMK	NS3	1896	1905	HLA-A*31:01	100%	Dengue virus 2	ELISPOT	IFNg release	Positive	0	1	1	0	1	0
57554	SEMGANFKA	NS3	1872	1880	HLA-B*44:03	100%	Dengue virus 2	ELISPOT	IFNg release	Positive	0	1	1	0	1	0
65550	TPEGIIPSMFEPERE	NS3	500	514	HLA class I	100%	Dengue virus 2	ELISPOT	IFNg release	Positive	0	1	1	0	1	0
67210	TVWFVPSIK	NS3	358	366	HLA class I	100%	Dengue virus 2	ELISPOT	IFNg release	Positive	0	1	1	0	1	0
150289	IILEFFLIV	NS4a	2202	2210	HLA-A*02:01	100%	Dengue virus 2	ELISPOT	IFNg release	Positive	0	1	1	0	1	0
180620	LPTFMTQKAR	NS4a	2106	2115	HLA-B*35:01	100%	Dengue virus 2	ELISPOT	IFNg release	Positive	0	1	1	0	1	0
180781	TPQDNQLTY	NS4a	2221	2229	HLA-B*35:01	100%	Dengue virus 2	ELISPOT	IFNg release	Positive	0	1	1	0	1	0
2877	ALSELPETL	NS4a	2137	2145	HLA-A*02:01	100%	Dengue virus 2	ELISPOT	IFNg release	Positive	0	1	1	0	1	0
37557	LLLTLLATV	NS4a	56	64	HLA-A2	100%	Dengue virus 2	ICS	IFNg release	Positive	0	1	1	0	1	0
150147	ATVLMGLGK	NS4b	2315	2323	HLA-A*11:01	100%	Dengue virus 2	multimer/tetramer	qualitative binding	Positive	0	1	1	0	1	0
150387	LMGLGKGWPL	NS4b	2318	2327	HLA-A*02:01	100%	Dengue virus 2	ELISPOT	IFNg release	Positive	0	1	1	0	1	0
150534	RPASAWTLY	NS4b	2276	2284	HLA-B*07:02	100%	Dengue virus 2	ELISPOT	IFNg release	Positive	0	1	1	0	1	0
150679	VIDLDPIPY	NS4b	2395	2403	HLA-A*01:01	100%	Dengue virus 2	ELISPOT	IFNg release	Positive	0	1	1	0	1	0
180512	IANQATVLM	NS4b	2311	2319	HLA-B*35:01	100%	Dengue virus 2	ELISPOT	IFNg release	Positive	0	1	1	0	1	0
180514	IAVSMANIF	NS4b	2460	2468	HLA-B*35:01	100%	Dengue virus 2	ELISPOT	IFNg release	Positive	0	1	1	0	1	0
180789	TVIDLDPIPYDPKFE	NS4b	2394	2408	HLA class I	100%	Dengue virus 2	ELISPOT	IFNg release	Positive	0	1	1	0	1	0

184814	MGLGKGWPL	NS4b	2319	2327	HLA-B*08:01	100%	Dengue virus 2	ELISPOT	IFNg release	Positive	0	1	1	0	1	0
150309	IPMSTYGWNL	NS5	2605	2614	HLA-B*07:02	100%	Dengue virus 2	ELISPOT	IFNg release	Positive	0	1	1	0	1	0
150655	TPRMCTREEF	NS5	2881	2890	HLA-B*35:01	100%	Dengue virus 2	ELISPOT	IFNg release	Positive	0	1	1	0	1	0
150750	YTDYMPSMK	NS5	3370	3378	HLA-A*01:01	100%	Dengue virus 2	ELISPOT	IFNg release	Positive	0	1	1	0	1	0
163027	NIQTAINQV	NS5	3353	3361	HLA-A*02:01	100%	Dengue virus 2	ELISPOT	IFNg release	Positive-Low	0	0	1	0	0	0
180443	ETACLGKSYA	NS5	3241	3250	HLA-A*26:01	100%	Dengue virus 2	ELISPOT	IFNg release	Positive	0	1	1	0	1	0
180643	MAMTDTTPFGQQRVF	NS5	2832	2846	HLA class I	100%	Dengue virus 2	ELISPOT	IFNg release	Positive	0	1	1	0	1	0
180667	MPSMKRFRRE	NS5	3374	3383	HLA-B*53:01	100%	Dengue virus 2	ELISPOT	IFNg release	Positive	0	1	1	0	1	0
180675	MSTYGWNLVRLQSGV	NS5	2607	2621	HLA class I	100%	Dengue virus 2	ELISPOT	IFNg release	Positive	0	1	1	0	1	0
180814	WAYHGSYET	NS5	2794	2802	HLA-B*35:01	100%	Dengue virus 2	ELISPOT	IFNg release	Positive	0	1	1	0	1	0
180817	WHYDQDHPY	NS5	2783	2791	HLA-B*35:01	100%	Dengue virus 2	ELISPOT	IFNg release	Positive	0	1	1	0	1	0
184131	KREDQWCGSL	NS5	3332	3341	HLA-B*08:01	100%	Dengue virus 2	ELISPOT	IFNg release	Positive	0	1	1	0	1	0
185491	RREEEEAGVL	NS5	3381	3390	HLA-B*08:01	100%	Dengue virus 2	ELISPOT	IFNg release	Positive	0	1	1	0	1	0
185931	TKQTGSASSM	NS5	2802	2811	HLA-B*08:01	100%	Dengue virus 2	ELISPOT	IFNg release	Positive	0	1	1	0	1	0
190862	MVTQMAMTDTTPFGQQR	NS5	2828	2844	HLA class I	100%	Dengue virus 2	ICS	IFNg release	Positive	0	1	1	0	1	0
53476	REDQWCGSL	NS5	3333	3341	HLA-B*40:01	100%	Dengue virus 2	ELISPOT	IFNg release	Positive	0	1	1	0	1	0
539436	ISGDDCVVKPLDDRFA	NS5	3151	3166	HLA class I	100%	Dengue virus 2	ICS	IFNg release	Positive	0	1	1	0	1	0
69671	VLNPYMPSV	NS5	2673	2681	HLA-A*02:01	100%	Dengue virus 2	ELISPOT	IFNg release	Positive	0	1	1	0	1	0
110018	VAFLRFLTI	C	51	59	HLA-B*08:01	81% - <100%	Dengue virus 2	ELISPOT	IFNg release	Positive	0	1	1	0	1	0
184378	LGMLQGRGPL	C	35	44	HLA-B*08:01	81% - <100%	Dengue virus 2	ELISPOT	IFNg release	Positive	0	1	1	0	1	0
184643	LTIPPTAGIL	C	21	30	HLA-B*08:01	81% - <100%	Dengue virus 2	ELISPOT	IFNg release	Positive	0	1	1	0	1	0
186354	VQQLTKRFSL	C	26	35	HLA-B*08:01	81% - <100%	Dengue virus 2	ELISPOT	IFNg release	Positive	0	1	1	0	1	0
150224	FTIMAAILAY	PreM	247	256	HLA-B*35:01	81% - <100%	Dengue virus 2	ELISPOT	IFNg release	Positive	1	1	1	1	0	1
150299	ILLTAVAPSM	PreM	270	279	HLA-B*08:01	81% - <100%	Dengue virus 2	ELISPOT	IFNg release	Positive	1	1	1	1	0	1
150571	SLLFKTEDGV	PreM	136	145	HLA-A*02:01	81% - <100%	Dengue virus 2	ELISPOT	IFNg release	Positive	1	1	1	1	1	1
150115	AIYGAAFSGV	E	722	731	HLA-A*02:01	81% - <100%	Dengue virus 2	ELISPOT	IFNg release	Positive	1	1	1	1	1	0
150319	ITEAELTGY	E	450	458	HLA-A*01:01	81% - <100%	Dengue virus 2	ELISPOT	IFNg release	Positive	1	1	1	1	1	1
150399	LQMENKAWLV	E	231	240	HLA-B*08:01	81% - <100%	Dengue virus 2	ELISPOT	IFNg release	Positive	1	1	1	1	1	1
150529	RLITVNPIV	E	630	638	HLA-A*02:01	81% - <100%	Dengue virus 2	ELISPOT	IFNg release	Positive	1	1	1	1	1	1
150578	SPCKIPFEIM	E	611	620	HLA-B*35:01	81% - <100%	Dengue virus 2	ELISPOT	IFNg release	Positive	1	1	1	1	1	1
150725	WLVHRQWFL	E	486	494	HLA-B*08:01	81% - <100%	Dengue virus 2	ELISPOT	IFNg release	Positive	1	1	1	1	1	1
180453	FGAIYGAAF	E	720	728	HLA-B*35:01	81% - <100%	Dengue virus 2	ELISPOT	IFNg release	Positive	1	1	1	1	1	0
180528	IPFEIMDLEK	E	615	624	HLA-B*35:01	81% - <100%	Dengue virus 2	ELISPOT	IFNg release	Positive	1	1	1	1	1	1
183310	GAAFSGVSW	E	725	733	HLA-B*58:01	81% - <100%	Dengue virus 2	ELISPOT	IFNg release	Positive	1	1	1	1	1	0
183748	ILIGVVITW	E	737	745	HLA-B*58:01	81% - <100%	Dengue virus 2	ELISPOT	IFNg release	Positive	0	0	0	0	0	0
183781	IMDLEKRHVL	E	619	628	HLA-B*08:01	81% - <100%	Dengue virus 2	ELISPOT	IFNg release	Positive	1	1	1	1	1	1
183840	IQKETLVTF	E	512	520	HLA-B*15:01	81% - <100%	Dengue virus 2	ELISPOT	IFNg release	Positive	1	1	1	1	1	1
184337	LEKRHVLGRL	E	622	631	HLA-B*08:01	81% - <100%	Dengue virus 2	ELISPOT	IFNg release	Positive	1	1	1	1	1	1
184755	MDLEKRHVL	E	620	628	HLA-B*08:01	81% - <100%	Dengue virus 2	ELISPOT	IFNg release	Positive	1	1	1	1	1	1
41407	MENKAWLVHRQWFLD	E	201	215	HLA-B*37:01	81% - <100%	Dengue virus 2	ICS	IFNg release	Positive	1	1	1	1	1	1
180450	FAGPVSQHNY	NS1	1022	1031	HLA-B*35:01	81% - <100%	Dengue virus 2	ELISPOT	IFNg release	Positive	0	0	1	0	0	0
180689	NRAVHADMGYWIESA	NS1	966	980	HLA class I	81% - <100%	Dengue virus 2	ICS	IFNg release	Positive	0	1	1	0	1	0
182938	EKEENLVNSL	NS1	1115	1124	HLA-B*08:01	81% - <100%	Dengue virus 2	ELISPOT	IFNg release	Positive	0	1	1	0	1	0
185342	RAVHADMGY	NS1	192	200	HLA-B*58:01	81% - <100%	Dengue virus 2	ELISPOT	IFNg release	Positive	0	1	1	0	1	0
150206	FLEEMLRTRV	NS2a	1131	1140	HLA-A*02:01	81% - <100%	Dengue virus 2	ELISPOT	IFNg release	Positive	0	1	1	0	1	0
150391	LMMTTIGIVL	NS2a	1228	1237	HLA-B*08:01	81% - <100%	Dengue virus 2	ELISPOT	IFNg release	Positive	0	1	1	0	1	0
150457	MTDDIGMGV	NS2a	1191	1199	HLA-A*01:01	81% - <100%	Dengue virus 2	ELISPOT	IFNg release	Positive	0	1	1	0	1	0
150540	RPTFAAGLLL	NS2a	1211	1220	HLA-B*07:02	81% - <100%	Dengue virus 2	ELISPOT	IFNg release	Positive	0	1	1	0	1	0
150741	YQLAVTIMA	NS2a	1157	1165	HLA-A*02:01	81% - <100%	Dengue virus 2	ELISPOT	IFNg release	Positive	0	1	1	0	1	0
183741	ILCVPNAVIL	NS2a	1279	1288	HLA-B*08:01	81% - <100%	Dengue virus 2	ELISPOT	IFNg release	Positive	0	1	1	0	1	0
183825	IPLALTIKGL	NS2a	1319	1328	HLA-B*08:01	81% - <100%	Dengue virus 2	ELISPOT	IFNg release	Positive	0	1	1	0	1	0
184864	MLRTRVGTK	NS2a	1149	1157	HLA-B*08:01	81% - <100%	Dengue virus 2	ELISPOT	IFNg release	Positive	0	1	1	0	1	0
150101	AAAWYLWEV	NS2b	1463	1471	HLA-A*02:01	81% - <100%	Dengue virus 2	ELISPOT	IFNg release	Positive	0	1	1	0	1	0
150243	GLLTVCYVL	NS2b	1381	1389	HLA-A*02:01	81% - <100%	Dengue virus 2	ELISPOT	IFNg release	Positive	0	1	1	0	1	0
150381	LLVISGLFPV	NS2b	1448	1457	HLA-A*02:01	81% - <100%	Dengue virus 2	ELISPOT	IFNg release	Positive	0	1	1	0	1	0
150425	LVISGLFPV	NS2b	1449	1457	HLA-A*02:01	81% - <100%	Dengue virus 2	ELISPOT	IFNg release	Positive	0	1	1	0	1	0
150584	SPILSITISE	NS2b	1416	1425	HLA-B*35:01	81% - <100%	Dengue virus 2	ELISPOT	IFNg release	Positive	0	1	1	0	1	0
150610	TAAAWYLWEV	NS2b	117	126	HLA-A*02:01	81% - <100%	Dengue virus 2	ELISPOT	IFNg release	Positive	0	1	1	0	1	0
180529	IPITAAAWY	NS2b	1459	1467	HLA-B*35:01	81% - <100%	Dengue virus 2	ELISPOT	IFNg release	Positive	0	1	1	0	1	0
180538	ISGLFPVSIPITAAA	NS2b	106	120	HLA class I	81% - <100%	Dengue virus 2	ICS	IFNg release	Positive	0	1	1	0	1	0
183819	IPITAAAWYL	NS2b	1459	1468	HLA-B*08:01	81% - <100%	Dengue virus 2	ELISPOT	IFNg release	Positive	0	1	1	0	1	0
11063	EAHFTDPASIAARGY	NS3	285	299	HLA class I	81% - <100%	Dengue virus 2	ELISPOT	IFNg release	Positive	0	1	1	0	1	0
150450	MPVTHSSAA	NS3	1922	1930	HLA-B*08:01	81% - <100%	Dengue virus 2	ELISPOT	IFNg release	Positive	0	1	1	0	1	0
150451	MPVTHSSAAQ	NS3	1922	1931	HLA-B*35:01	81% - <100%	Dengue virus 2	ELISPOT	IFNg release	Positive	0	1	1	0	1	0
180611	LMRRGDLPVWLAYRV	NS3	2011	2025	HLA-B*55:02	81% - <100%	Dengue virus 2	ICS	IFNg release	Positive	0	0	1	0	1	0
180625	LPVWLAYRV	NS3	2017	2025	HLA-B*51:01	81% - <100%	Dengue virus 2	ELISPOT	IFNg release	Positive	0	0	1	0	1	0
180626	LPVWLAYRVA	NS3	2017	2026	HLA-B*51:01	81% - <100%	Dengue virus 2	ELISPOT	IFNg release	Positive	0	0	1	0	1	0
180756	STRVEMGEAAGIFMT	NS3	301	315	HLA-B*40:06	81% - <100%	Dengue virus 2	ELISPOT	IFNg release	Positive-Low	0	1	1	0	1	0
185388	RIKQKGILGY	NS3	1499	1508	HLA-B*15:01	81% - <100%	Dengue virus 2	ELISPOT	IFNg release	Positive	0	1	1	0	1	0
186242	VEMGEAAGIF	NS3	1779	1788	HLA-B*44:03	81% - <100%	Dengue virus 2	ELISPOT	IFNg release	Positive	0	1	1	0	1	0
22823	GTSGSPIVDK	NS3	133	142	HLA-A11	81% - <100%	Dengue virus 2	51 chromium	cytotoxicity	Positive	0	0	0	0	0	0
54176	RIKQKGIL	NS3	1499	1506	HLA-B8	81% - <100%	Dengue virus 2	ELISPOT	IFNg release	Positive	0	1	1	0	1	0
8910	DKKGKVVGL	NS3	1616	1624	HLA-B8	81% - <100%	Dengue virus 2	ELISPOT	IFNg release	Positive	0	1	1	0	1	0
150461	MTQKARNAL	NS4a	2110	2118	HLA-B*07:02	81% - <100%	Dengue virus 2	ELISPOT	IFNg release	Positive	0	0	0	0	0	0
150612	TAEAGGRAY	NS4a	2126	2134	HLA-B*35:01	81% - <100%	Dengue virus 2	ELISPOT	IFNg release	Positive	0	1	1	0	1	0
150752	YVVIAILTV	NS4a	2229	2237	HLA-A*02:01	81% - <100%	Dengue virus 2	ELISPOT	IFNg release	Positive	0	1	1	0	1	0
150753	YVVIAILTVV	NS4a	2229	2238	HLA-A*02:01	81% - <100%	Dengue virus 2	ELISPOT	IFNg release	Positive	0	1	1	0	1	0
185447	RNALDNLAVL	NS4a	2115	2124	HLA-B*08:01	81% - <100%	Dengue virus 2	ELISPOT	IFNg release	Positive	0	0	0	0	0	0
51562	QLTYVVIAIL	NS4a	2226	2235	HLA-B*08:01	81% - <100%	Dengue virus 2	ELISPOT	IFNg release	Positive	0	1	1	0	1	0
150377	LLLVAHYAI	NS4b	2355	2363	HLA-A*02:01	81% - <100%	Dengue virus 2	ELISPOT	IFNg release	Positive	0	1	0	0	0	0
150389	LMMRTTWAL	NS4b	2425	2433	HLA-B*08:01	81% - <100%	Dengue virus 2	ELISPOT	IFNg release	Positive	0	1	1	0	1	0
150686	VLMMRTTWA	NS4b	2424	2432	HLA-B*08:01	81% - <100%	Dengue virus 2	ELISPOT	IFNg release	Positive	0	1	1	0	1	0
180380	ATGPISTLW	NS4b	2440	2448	HLA-B*58:01	81% - <100%	Dengue virus 2	ELISPOT	IFNg release	Positive	0	1	1	0	1	0
180387	CEALTLATGPISTLW	NS4b	191	205	HLA class I	81% - <100%	Dengue virus 2	ELISPOT	IFNg release	Positive	0	1	1	0	1	0
180802	VPLLAIGCY	NS4b	2334	2342	HLA-B*35:01	81% - <100%	Dengue virus 2	ELISPOT	IFNg release	Positive	0	1	1	0	1	0
182747	CYSQVNPITL	NS4b	2341	2350	HLA-A*24:02	81% - <100%	Dengue virus 2	ELISPOT	IFNg release	Positive	0	1	1	0	1	0
183670	IENSSVNVSL	NS4b	2299	2308	HLA-B*08:01	81% - <100%	Dengue virus 2	ELISPOT	IFNg release	Positive	0	1	1	0	1	0
186002	TPMLRHSIE	NS4b	2292	2300	HLA-B*08:01	81% - <100%	Dengue virus 2	ELISPOT	IFNg release	Positive	0	1	1	0	1	0
240933	TPMLRHSI	NS4b	2292	2299	HLA-B*07:02	81% - <100%	Dengue virus 2	ELISPOT	IFNg release	Positive	0	1	1	0	1	0
10587	DVFFTPPEK	NS5	2622	2630	HLA class I	81% - <100%	Dengue virus 2	ELISPOT	IFNg release	Positive	0	0	1	0	0	0
150227	FTMRHKKATY	NS5	2734	2743	HLA-B*35:01	81% - <100%	Dengue virus 2	ELISPOT	IFNg release	Positive	0	1	1	0	0	0
150541	RPTPRGTVM	NS5	3073	3081	HLA-B*07:02	81% - <100%	Dengue virus 2	ELISPOT	IFNg release	Positive	0	1	1	0	1	0
174066	CGGLKNVREVKGLTKGG	NS5	2582	2598	HLA-A2	81% - <100%	Dengue virus 2	ICS	IFNg release	Positive	0	1	1	0	1	0
180437	EPKEGTKKLM	NS5	2856	2865	HLA-B*35:01	81% - <100%	Dengue virus 2	ELISPOT	IFNg release	Positive	0	1	1	0	1	0
180483	GKKKTPRMCTREEFT	NS5	2877	2891	HLA class I	81% - <100%	Dengue virus 2	ELISPOT	IFNg release	Positive	0	1	1	0	1	0
180488	GPGHEEPIPM	NS5	2598	2607	HLA-B*35:01	81% - <100%	Dengue virus 2	ELISPOT	IFNg release	Positive	0	1	1	0	1	0
180500	HKKLAEAIFKLTYQN	NS5	3052	3066	HLA class I	81% - <100%	Dengue virus 2	ELISPOT	IFNg release	Positive	0	1	1	0	1	0
180579	KSYAQMWSLMYFHRR	NS5	3247	3261	HLA class I	81% - <100%	Dengue virus 2	ELISPOT	IFNg release	Positive	0	1	1	0	1	0
180582	KTWAYHGSYETKQTG	NS5	2792	2806	HLA class I	81% - <100%	Dengue virus 2	ICS	IFNg release	Positive	0	1	1	0	1	0
180584	KVRKDIQQW	NS5	3177	3185	HLA-B*57:01	81% - <100%	Dengue virus 2	ELISPOT	IFNg release	Positive	0	0	1	0	0	0
180606	LMKITAEWLW	NS5	2864	2873	HLA-B*53:01	81% - <100%	Dengue virus 2	ELISPOT	IFNg release	Positive	0	1	1	0	1	0
180758	SWHYDQDHPYKTWAY	NS5	291	305	HLA class I	81% - <100%	Dengue virus 2	ELISPOT	IFNg release	Positive	0	1	1	0	1	0
180774	TLRVLNLVENWLNNN	NS5	2652	2666	HLA class I	81% - <100%	Dengue virus 2	ELISPOT	IFNg release	Positive	0	1	1	0	1	0
180828	YAQMWSLMYF	NS5	3249	3258	HLA-B*35:01	81% - <100%	Dengue virus 2	ELISPOT	IFNg release	Positive	0	1	1	0	1	0
184373	LGKKKTPRM	NS5	2876	2884	HLA-B*08:01	81% - <100%	Dengue virus 2	ELISPOT	IFNg release	Positive	0	1	1	0	1	0
184840	MLINRFTMR	NS5	2729	2737	HLA-B*08:01	81% - <100%	Dengue virus 2	ELISPOT	IFNg release	Positive	0	1	1	0	1	0
184957	MTTEDMLTVW	NS5	3295	3304	HLA-B*58:01	81% - <100%	Dengue virus 2	ICS	IFNg release	Positive	0	1	1	0	1	0
31747	KLAEAIFKL	NS5	3054	3062	HLA-A*02:01	81% - <100%	Dengue virus 2	ELISPOT	IFNg release	Positive-Low	0	1	1	0	1	0
539144	CVVKPLDDRFASALTAL	NS5	3156	3172	HLA class I	81% - <100%	Dengue virus 2	ICS	IFNg release	Positive	0	1	1	0	1	0
73428	YAQMWSLMY	NS5	3249	3257	HLA-B*15:01	81% - <100%	Dengue virus 2	ELISPOT	IFNg release	Positive	0	1	1	0	1	0
183766	ILNRRRRTA	C	94	102	HLA-B*08:01	51%-80%	Dengue virus 2	ELISPOT	IFNg release	Positive	0	0	1	0	0	0
184518	LNRRRRTAG	C	95	103	HLA-B*08:01	51%-80%	Dengue virus 2	ELISPOT	IFNg release	Positive	0	0	1	0	0	0
185104	NRRRRTAGV	C	96	104	HLA-B*08:01	51%-80%	Dengue virus 2	ELISPOT	IFNg release	Positive	0	0	0	0	0	0
124391	KTFVDLMRR	NS3	2006	2014	HLA-A*11:01	51%-80%	Dengue virus 2	ELISPOT	IFNg release	Positive	0	1	1	0	1	0
150167	DLMRRGDLPV	NS3	2010	2019	HLA-A*02:01	51%-80%	Dengue virus 2	ELISPOT	IFNg release	Positive	0	1	1	0	1	0
180471	GEARKTFVDL	NS3	2002	2011	HLA-B*40:01	51%-80%	Dengue virus 2	ELISPOT	IFNg release	Positive	0	1	1	0	1	0
180724	RGEARKTFVDLMRRG	NS3	2001	2015	HLA class I	51%-80%	Dengue virus 2	ELISPOT	IFNg release	Positive	0	1	1	0	1	0
182848	EARKTFVDL	NS3	2003	2011	HLA-B*08:01	51%-80%	Dengue virus 2	ELISPOT	IFNg release	Positive	0	1	1	0	1	0
183347	GERKKLKPRW	NS3	2061	2070	HLA-B*44:03	51%-80%	Dengue virus 2	ELISPOT	IFNg release	Positive	0	1	1	0	1	0
180668	MPSVIEKMET	NS5	2678	2687	HLA-B*53:01	51%-80%	Dengue virus 2	ELISPOT	IFNg release	Positive	0	1	0	0	0	0
180588	LAYTIGTTHF	PreM	254	263	HLA-B*35:01	>0% - 50%	Dengue virus 2	ELISPOT	IFNg release	Positive	1	1	1	1	1	1

180433	EPGQLKLNWF	E	663	672	HLA-B*35:01	>0% - 50%	Dengue virus 2	ELISPOT	IFNg release	Positive	1	0	1	1	1	1
184467	LLQMEDKAWL	E	478	487	HLA-B*08:01	>0% - 50%	Dengue virus 2	ELISPOT	IFNg release	Positive	0	0	0	0	0	0
150300	ILLVAVSFV	NS2a	1146	1154	HLA-A*02:01	>0% - 50%	Dengue virus 2	ELISPOT	IFNg release	Positive	0	1	1	0	1	0
150640	TMTDDIGMGV	NS2a	1176	1185	HLA-A*02:01	>0% - 50%	Dengue virus 2	ELISPOT	IFNg release	Positive	0	1	1	0	1	0
180717	RDLGRVMVMVGATMT	NS2a	51	65	HLA class I	>0% - 50%	Dengue virus 2	ICS	IFNg release	Positive	0	0	1	0	1	0
180735	RVGTKHAILLVAVSF	NS2a	1153	1167	HLA class I	>0% - 50%	Dengue virus 2	ICS	IFNg release	Positive	0	1	1	0	1	0
186041	TRVGTKHAIL	NS2a	1152	1161	HLA-B*08:01	>0% - 50%	Dengue virus 2	ELISPOT	IFNg release	Positive	0	1	1	0	1	0
10643	DVKKDLISY	NS3	71	79	HLA-B62	>0% - 50%	Dengue virus 2	51 chromium	cytotoxicity	Positive	0	1	1	0	1	0
150346	KTFDSEYVK	NS3	1863	1871	HLA-A*11:01	>0% - 50%	Dengue virus 2	multimer/tetramer	qualitative binding	Positive	0	1	1	0	1	0
17802	FSPGTSGSPIIDKKG	NS3	130	144	HLA class I	>0% - 50%	Dengue virus 2	ELISPOT	IFNg release	Positive	0	1	1	0	1	0
180623	LPVWLAYKV	NS3	2017	2025	HLA-B*51:01	>0% - 50%	Dengue virus 2	ELISPOT	IFNg release	Positive	0	1	0	0	0	0
180624	LPVWLAYKVA	NS3	2017	2026	HLA-B*35:01	>0% - 50%	Dengue virus 2	ELISPOT	IFNg release	Positive	0	1	0	0	0	0
180750	SPGTSGSPIIDKKGK	NS3	131	145	HLA-A*11:01	>0% - 50%	Dengue virus 2	ELISPOT	IFNg release	Positive	0	1	1	0	1	0
22818	GTSGSPIIDK	NS3	133	142	HLA-A*11:01	>0% - 50%	Dengue virus 2	ELISPOT	IFNg release	Positive	0	1	1	0	1	0
22819	GTSGSPIIDKK	NS3	133	143	HLA-A11	>0% - 50%	Dengue virus 2	51 chromium	cytotoxicity	Positive	0	1	1	0	1	0
25929	IEPSWADVKKDLISY	NS3	65	79	HLA class I	>0% - 50%	Dengue virus 2	ELISPOT	IFNg release	Positive	0	1	1	0	1	0
790	ADVKKDLISYGGGWK	NS3	70	84	HLA class I	>0% - 50%	Dengue virus 2	ELISPOT	IFNg release	Positive	0	1	1	0	1	0
16757	FLLVAHYAI	NS4b	112	120	HLA-A2	>0% - 50%	Dengue virus 2	ICS	TNF release	Positive	0	0	1	0	1	0
35568	LEKTKKDL	NS4b	2249	2256	HLA-B8	>0% - 50%	Dengue virus 2	ELISPOT	IFNg release	Positive	0	1	1	0	1	0
150554	RTTWSIHAK	NS5	3283	3291	HLA-A*11:01	>0% - 50%	Dengue virus 2	ELISPOT	IFNg release	Positive	0	1	1	0	1	0
161186	RLLTKPWDVVPMVTQ	NS5	326	340	HLA-B*55:02	>0% - 50%	Dengue virus 2	ICS	IFNg release	Positive	0	1	1	0	1	0
180681	NEEYTDYMPSMKRFR	NS5	3367	3381	HLA class I	>0% - 50%	Dengue virus 2	ELISPOT	IFNg release	Positive	0	1	1	0	1	0
182855	EAVEDGRFWE	NS5	2915	2924	HLA-B*58:01	>0% - 50%	Dengue virus 2	ELISPOT	IFNg release	Positive	0	0	0	0	0	0
186219	VEDGRFWEL	NS5	2917	2925	HLA-B*40:01	>0% - 50%	Dengue virus 2	ELISPOT	IFNg release	Positive	0	0	0	0	0	0
32943	KPWDVVPMV	NS5	2821	2829	HLA-B*51:01	>0% - 50%	Dengue virus 2	51 chromium	cytotoxicity	Positive	0	1	1	0	1	0
74305	YILRDVSKK	NS5	517	525	HLA class I	>0% - 50%	Dengue virus 2	ELISPOT	IFNg release	Positive	0	1	1	0	1	0

CD8 DENV-3 Epitopes

											DENV-3 constructs of vaccines (100% identity=1,green ; else 0, red)					
Epitope_ID	AA_Seq	Protein_Name	Start	End	Allele	Step	Serotype	Method/Technique	Assay Group	Qualitative Measure	TV003	TDEN	DPIV	Dengvaxia	DENVax	TVDV
180390	CLMMMLPATL	C	104	113	HLA-A*02:01	0%	Dengue virus 3	ELISPOT	IFNg release	Positive	1	1	1	0	0	0
180609	LMMMLPATL	C	105	113	HLA-A*02:01	0%	Dengue virus 3	ELISPOT	IFNg release	Positive	1	1	1	0	0	0
183793	IMKIGIGVLL	E	452	461	HLA-B*08:01	0%	Dengue virus 3	ELISPOT	IFNg release	Positive	1	1	1	1	0	1
184412	LKGMSYAMCL	E	156	165	HLA-B*08:01	0%	Dengue virus 3	ELISPOT	IFNg release	Positive	1	1	1	1	1	1
186470	WIMKIGIGVL	E	451	460	HLA-B*08:01	0%	Dengue virus 3	ELISPOT	IFNg release	Positive	1	1	1	1	0	1
185464	RPINEKEENM	NS1	1109	1118	HLA-B*35:01	0%	Dengue virus 3	ELISPOT	IFNg release	Positive	1	1	1	0	0	0
183395	GKKHMIAGVL	NS2a	1153	1162	HLA-B*08:01	0%	Dengue virus 3	ELISPOT	IFNg release	Positive	1	0	1	0	0	0
3124	AMKGLPIRY	NS3	1708	1716	HLA-B62	0%	Dengue virus 3	51 chromium	cytotoxicity	Positive	0	0	0	0	0	0
590435	TRVVAAEMEEAMKGLPI	NS3	1698	1714	HLA class I	0%	Dengue virus 3	ICS	IFNg release	Positive-Low	0	0	0	0	0	0
180572	KPRLCTREEF	NS5	2879	2888	HLA-B*07:02	0%	Dengue virus 3	ELISPOT	IFNg release	Positive	0	0	0	0	0	0
180801	VMGITAEWLW	NS5	2862	2871	HLA-B*53:01	0%	Dengue virus 3	ELISPOT	IFNg release	Positive	0	0	0	0	0	0
186127	TTWEDVPYL	NS5	3321	3329	HLA-A*02:01	0%	Dengue virus 3	ELISPOT	IFNg release	Positive	0	1	0	0	0	0
32944	KPWDVVPTV	NS5	2819	2827	HLA class I	0%	Dengue virus 3	51 chromium	cytotoxicity	Positive	0	0	0	0	0	0
180464	FTILALFLAH	PreM	247	256	HLA-B*35:01	100%	Dengue virus 3	ELISPOT	IFNg release	Positive	1	1	1	1	1	1
180504	HPGFTILALF	PreM	244	253	HLA-B*35:01	100%	Dengue virus 3	ELISPOT	IFNg release	Positive	1	1	1	1	1	1
185685	SLLFKTASG	PreM	136	144	HLA-B*08:01	100%	Dengue virus 3	ELISPOT	IFNg release	Positive	1	1	1	1	1	1
180613	LPEEQDQNY	E	82	90	HLA-B*35:01	100%	Dengue virus 3	ELISPOT	IFNg release	Positive	0	1	0	1	1	1
180657	MLVTPSMTM	E	273	281	HLA-B*35:01	100%	Dengue virus 3	ELISPOT	IFNg release	Positive	1	0	1	0	0	0
183843	IQNSGGTSIF	E	131	140	HLA-B*15:01	100%	Dengue virus 3	ELISPOT	IFNg release	Positive	0	1	0	1	0	0
184299	LATLRKLCI	E	53	61	HLA-B*08:01	100%	Dengue virus 3	ELISPOT	IFNg release	Positive	1	1	1	1	1	1
184536	LPEYGTLGLE	E	453	462	HLA-B*35:01	100%	Dengue virus 3	ELISPOT	IFNg release	Positive	1	1	1	1	1	1
185419	RLKMDKLEL	E	148	156	HLA-B*08:01	100%	Dengue virus 3	ELISPOT	IFNg release	Positive	1	1	1	1	1	0
150537	RPGYHTQTA	NS1	1030	1038	HLA-B*07:02	100%	Dengue virus 3	ELISPOT	IFNg release	Positive	1	1	1	0	0	0
150546	RSCTLPPLRY	NS1	1087	1096	HLA-A*01:01	100%	Dengue virus 3	ELISPOT	IFNg release	Positive	1	1	1	0	0	0
180738	RYMGEDGCWY	NS1	1095	1104	HLA-A*24:02	100%	Dengue virus 3	ELISPOT	IFNg release	Positive	1	1	1	0	0	0
182939	EKEENMVKSL	NS1	1113	1122	HLA-B*08:01	100%	Dengue virus 3	ELISPOT	IFNg release	Positive	1	1	1	0	0	0
184585	LRTTTVSGKL	NS1	64	73	HLA-B*08:01	100%	Dengue virus 3	ELISPOT	IFNg release	Positive	1	1	1	0	0	0
185573	RYMGEDGCW	NS1	1095	1103	HLA-A*24:02	100%	Dengue virus 3	ELISPOT	IFNg release	Positive	1	1	1	0	0	0
185734	SPKRLATAI	NS1	811	819	HLA-B*08:01	100%	Dengue virus 3	ELISPOT	IFNg release	Positive	1	1	1	0	0	0
186175	TWRDMAHTLI	NS2a	1174	1183	HLA-A*24:02	100%	Dengue virus 3	ELISPOT	IFNg release	Positive	1	1	1	0	0	0
182917	EFKDFAAGR	NS3	2083	2091	HLA-A*33:01	100%	Dengue virus 3	ELISPOT	IFNg release	Positive	1	1	1	0	0	0
186234	VEIWTKEGEK	NS3	2053	2062	HLA-B*40:01	100%	Dengue virus 3	ELISPOT	IFNg release	Positive	1	1	1	0	0	0
19434	GESRKTFVE	NS3	2001	2009	HLA-B7	100%	Dengue virus 3	51 chromium	cytotoxicity	Positive	1	1	1	0	0	0
19435	GESRKTFVEL	NS3	211	220	HLA-B*40:01	100%	Dengue virus 3	ICS	IFNg release	Positive	1	1	1	0	0	0
22820	GTSGSPIINR	NS3	133	142	HLA-A*11:01	100%	Dengue virus 3	51 chromium	cytotoxicity	Positive	1	1	1	0	0	0
22821	GTSGSPIINRE	NS3	133	143	HLA-A11	100%	Dengue virus 3	ICS	IFNg release	Positive	1	1	1	0	0	0
34605	KYTDRKWCF	NS3	240	248	HLA-A24	100%	Dengue virus 3	ELISPOT	IFNg release	Positive	1	1	1	0	0	0
62183	SVKKDLISY	NS3	71	79	HLA-B62	100%	Dengue virus 3	51 chromium	cytotoxicity	Positive	1	1	1	0	0	0
183165	FFMMVLLIPE	NS4a	2205	2214	HLA-A*33:01	100%	Dengue virus 3	ELISPOT	IFNg release	Positive	1	1	1	0	0	0
183796	IMKSVGTGK	NS4b	2481	2489	HLA-A*03:01	100%	Dengue virus 3	ELISPOT	IFNg release	Positive	1	1	1	0	0	0
37534	LLLMRTSWA	NS4b	2423	2431	HLA-A2	100%	Dengue virus 3	biological activity	proliferation	Positive	1	1	1	0	0	0
1067501	KPIDDRFAN	NS5	3158	3166	HLA-B*07:02	100%	Dengue virus 3	ELISPOT	IFNg release	Positive	1	1	1	0	0	0
180650	MEITAEWLW	NS5	2863	2871	HLA-B*58:01	100%	Dengue virus 3	ELISPOT	IFNg release	Positive	1	1	1	0	0	0
180728	RPRLCTREEF	NS5	2879	2888	HLA-B*07:02	100%	Dengue virus 3	ELISPOT	IFNg release	Positive	1	1	1	0	0	0
182553	ALLALNDMGK	NS5	3167	3176	HLA-A*03:01	100%	Dengue virus 3	ELISPOT	IFNg release	Positive	1	1	1	0	0	0
185710	SMINGVVKL	NS5	318	326	HLA-A*02:01	100%	Dengue virus 3	ELISPOT	IFNg release	Positive	1	1	1	0	0	0
180577	KSGAIKVLK	C	74	82	HLA-A*11:01	81% - <100%	Dengue virus 3	ELISPOT	IFNg release	Positive	0	1	0	0	0	0
184260	LAIPPTAGVL	C	57	66	HLA-B*08:01	81% - <100%	Dengue virus 3	ELISPOT	IFNg release	Positive	1	1	1	0	0	0
184845	MLKRVRNRV	C	15	23	HLA-B*08:01	81% - <100%	Dengue virus 3	ELISPOT	IFNg release	Positive	1	1	1	0	0	0
184658	LTQKVVIFIL	PreM	262	271	HLA-B*08:01	81% - <100%	Dengue virus 3	ELISPOT	IFNg release	Positive	1	1	1	1	1	1
13119	ELKGMSYAM	E	155	163	HLA-B*08:01	81% - <100%	Dengue virus 3	ELISPOT	IFNg release	Positive	1	1	1	1	1	0
15796	FFDLPLPWT	E	211	219	HLA-A2	81% - <100%	Dengue virus 3	ICS	IFNg release	Positive	1	1	1	1	1	1
184568	LPWTSGATT	E	496	504	HLA-B*35:01	81% - <100%	Dengue virus 3	ELISPOT	IFNg release	Positive	1	1	1	1	0	1
184924	MSYAMCTNTF	E	575	584	HLA-B*35:01	81% - <100%	Dengue virus 3	ELISPOT	IFNg release	Positive	0	0	0	0	0	0
184965	MVHQIFGSAY	E	713	722	HLA-B*15:01	81% - <100%	Dengue virus 3	ELISPOT	IFNg release	Positive	1	1	1	1	1	1
186029	TPTWNRKEL	E	226	234	HLA-B*07:02	81% - <100%	Dengue virus 3	ELISPOT	IFNg release	Positive-Low	0	1	0	1	1	1
589817	SGATWVDVVLEHGGCV	E	268	283	HLA class I	81% - <100%	Dengue virus 3	ICS	IFNg release	Positive-High	1	1	1	1	1	1
184254	LAILFEEVM	NS2a	1140	1148	HLA-B*35:01	81% - <100%	Dengue virus 3	ELISPOT	IFNg release	Positive	1	1	1	0	0	0
185978	TMGVLCLAIL	NS2a	1134	1143	HLA-B*08:01	81% - <100%	Dengue virus 3	ELISPOT	IFNg release	Positive	1	0	1	0	0	0
180622	LPVWLAHKVA	NS3	226	235	HLA-B*35:01	81% - <100%	Dengue virus 3	ELISPOT	IFNg release	Positive	1	1	1	0	0	0
184065	KLNDWDFVV	NS3	399	407	HLA-A*02:01	81% - <100%	Dengue virus 3	ELISPOT	IFNg release	Positive-High	1	1	1	0	0	0
37494	LLLGLMILL	NS4a	2148	2156	HLA-A2	81% - <100%	Dengue virus 3	ICS	IFNg release	Positive	1	1	1	0	0	0
180381	ATGPITTLW	NS4b	2439	2447	HLA-B*58:01	81% - <100%	Dengue virus 3	ELISPOT	IFNg release	Positive	1	1	1	0	0	0
180788	TTKRDLGMSK	NS4b	2250	2259	HLA-A*11:01	81% - <100%	Dengue virus 3	ELISPOT	IFNg release	Positive	1	1	1	0	0	0
180737	RYLEFEALGF	NS5	2971	2980	HLA-A*24:02	81% - <100%	Dengue virus 3	ELISPOT	IFNg release	Positive	1	1	1	0	0	0
183587	HMIAGVFFTF	NS2a	368	377	HLA-A*24:02	51%-80%	Dengue virus 3	ELISPOT	IFNg release	Positive	0	0	0	0	0	0
184819	MIAGVFFTF	NS2a	369	377	HLA-B*35:01	51%-80%	Dengue virus 3	ELISPOT	IFNg release	Positive	0	0	0	0	0	0
180678	MVSRLLLNR	NS5	2723	2731	HLA-A*11:01	51%-80%	Dengue virus 3	ELISPOT	IFNg release	Positive	1	1	1	0	0	0
184264	LAKRFSRGL	C	29	37	HLA-B*08:01	>0% - 50%	Dengue virus 3	ELISPOT	IFNg release	Positive	1	1	1	0	0	0
184265	LAKRFSRGLL	C	29	38	HLA-B*08:01	>0% - 50%	Dengue virus 3	ELISPOT	IFNg release	Positive	1	1	1	0	0	0
185260	QLAKRFSRG	C	28	36	HLA-B*08:01	>0% - 50%	Dengue virus 3	ELISPOT	IFNg release	Positive	1	1	1	0	0	0
183723	IKLTVVVGDI	NS1	857	866	HLA-B*08:01	>0% - 50%	Dengue virus 3	ELISPOT	IFNg release	Positive	1	1	1	0	0	0
69612	VLLLVTHYA	NS4b	2353	2361	HLA-A2	>0% - 50%	Dengue virus 3	ICS	IFNg release	Positive	1	0	1	0	0	0
186215	VEDEDFWKL	NS5	2915	2923	HLA-B*40:01	>0% - 50%	Dengue virus 3	ELISPOT	IFNg release	Positive	0	0	0	0	0	0

CD8 DENV-4 Epitopes

											DENV-4 constructs of vaccines (100% identity=1,green ; else 0, red)					
Epitope_ID	AA_Seq	Protein_Name	Start	End	Allele	Step	Serotype	Method/Technique	Assay Group	Qualitative Measure	TV003	TDEN	DPIV	Dengvaxia	DENVax	TVDV
180810	VTYECPLLV	PreM	162	170	HLA-A*02:01	0%	Dengue virus 4	ELISPOT	IFNg release	Positive	0	0	0	0	0	1
182707	CNLTSTWVMY	PreM	181	190	HLA-A*30:02	0%	Dengue virus 4	ELISPOT	IFNg release	Positive	1	1	1	0	1	0
186303	VLMMLVAPSY	PreM	17	26	HLA-B*15:01	0%	Dengue virus 4	ELISPOT	IFNg release	Positive	1	1	1	0	1	1
185147	NYKERMVTF	E	511	519	HLA-A*24:02	0%	Dengue virus 4	ELISPOT	IFNg release	Positive	1	1	1	1	1	1
185817	SWMIRILIGF	E	161	170	HLA-A*24:02	0%	Dengue virus 4	ELISPOT	IFNg release	Positive	1	1	1	0	1	0
240130	PPASDLKYSW	NS1	880	889	HLA-B*53:01	0%	Dengue virus 4	ELISPOT	IFNg release	Positive	0	0	0	0	0	0
167725	AMTTTLSIPHDLMELIDGIS	NS2a	50	69	HLA class I	0%	Dengue virus 4	ELISPOT	IFNg release	Positive	0	0	0	0	0	0
239411	ALWYVWQVK	NS2b	1463	1471	HLA-A*03:01	0%	Dengue virus 4	ELISPOT	IFNg release	Positive	0	0	0	0	0	0
418991	IPVTMALWY	NS2b	1458	1466	HLA-B*35:01	0%	Dengue virus 4	ELISPOT	IFNg release	Positive	0	0	0	0	0	0
180640	MALKDFKEF	NS3	2079	2087	HLA-B*35:01	0%	Dengue virus 4	ELISPOT	IFNg release	Positive	1	1	1	0	0	0
182563	ALSEGVYRI	NS3	1491	1499	HLA-A*02:01	0%	Dengue virus 4	ELISPOT	IFNg release	Positive	1	1	1	0	0	0
186008	TPPGATDPF	NS3	1791	1799	HLA-B*35:01	0%	Dengue virus 4	ELISPOT	IFNg release	Positive	1	1	1	0	0	0
186602	YSDPLALRE	NS3	2077	2085	HLA-A*01:01	0%	Dengue virus 4	ELISPOT	IFNg release	Positive	0	0	0	0	0	0
239768	GTSGSPIINK	NS3	1607	1616	HLA-A*03:01	0%	Dengue virus 4	ELISPOT	IFNg release	Positive	0	0	0	0	0	0
180383	ATGPVLTLW	NS4b	2436	2444	HLA-B*58:01	0%	Dengue virus 4	ELISPOT	IFNg release	Positive	0	0	0	0	0	0
180587	LATGPVLTLW	NS4b	2435	2444	HLA-B*53:01	0%	Dengue virus 4	ELISPOT	IFNg release	Positive	0	0	0	0	0	0
184375	LGKSYAQMW	NS5	3246	3254	HLA-B*57:01	0%	Dengue virus 4	ELISPOT	IFNg release	Positive	0	0	0	0	0	0
419017	MPAMKRYSAP	NS5	3371	3380	HLA-B*35:01	0%	Dengue virus 4	ELISPOT	IFNg release	Positive	0	0	0	0	0	0
419048	SPRLCTREEF	NS5	2877	2886	HLA-B*07:02	0%	Dengue virus 4	ELISPOT	IFNg release	Positive	0	0	0	0	0	0
182971	EMCEDTVTY	PreM	156	164	HLA-B*35:01	100%	Dengue virus 4	ELISPOT	IFNg release	Positive	1	1	1	0	1	1
583701	GVSGGAWVDLVLEHGGCV	E	14	31	HLA class I	100%	Dengue virus 4	ICS	IFNg release	Positive-Low	1	1	1	1	1	1
183667	IEKASLIEV	NS1	986	994	HLA-B*40:01	100%	Dengue virus 4	ELISPOT	IFNg release	Positive	1	1	1	0	0	0
185050	NFLEVEDYGF	NS1	925	934	HLA-A*24:02	100%	Dengue virus 4	ELISPOT	IFNg release	Positive	0	0	0	0	0	0
182562	ALPVYLMTL	NS2a	1329	1337	HLA-A*02:01	100%	Dengue virus 4	ELISPOT	IFNg release	Positive	1	1	1	0	0	0
184566	LPVYLMTLMK	NS2a	1330	1339	HLA-B*35:01	100%	Dengue virus 4	ELISPOT	IFNg release	Positive	1	1	1	0	0	0
182901	EETNMITLL	NS2b	1434	1442	HLA-B*40:01	100%	Dengue virus 4	ELISPOT	IFNg release	Positive	1	1	1	0	0	0
186584	YPLAIPVTM	NS2b	1454	1462	HLA-B*53:01	100%	Dengue virus 4	ELISPOT	IFNg release	Positive	1	1	1	0	0	0
156711	LAPTRVVAAEMEEAL	NS3	1695	1709	HLA-B7	100%	Dengue virus 4	51 chromium	cytotoxicity	Positive	1	1	1	0	0	0
180473	GEFRLRGEQR	NS3	1995	2004	HLA-B*40:01	100%	Dengue virus 4	ELISPOT	IFNg release	Positive	1	1	1	0	0	0
180648	MEGVFHTMW	NS3	1516	1524	HLA-B*44:03	100%	Dengue virus 4	ELISPOT	IFNg release	Positive	1	1	1	0	0	0
180778	TPEGIIPTLF	NS3	1974	1983	HLA-B*07:02	100%	Dengue virus 4	ELISPOT	IFNg release	Positive	1	1	1	0	0	0
180843	YPKTKLTDWD	NS3	1868	1877	HLA-B*35:01	100%	Dengue virus 4	ELISPOT	IFNg release	Positive	1	1	1	0	0	0
182919	EFRLRGEQR	NS3	1996	2004	HLA-A*33:01	100%	Dengue virus 4	ELISPOT	IFNg release	Positive	1	1	1	0	0	0
183407	GLFGKTQVGV	NS3	1503	1512	HLA-A*02:01	100%	Dengue virus 4	ELISPOT	IFNg release	Positive	1	1	1	0	0	0
183427	GLYGNGVVTK	NS3	1622	1631	HLA-A*03:01	100%	Dengue virus 4	ELISPOT	IFNg release	Positive	1	1	1	0	0	0
184079	KLTDWDFVV	NS3	398	406	HLA-A*02:01	100%	Dengue virus 4	ELISPOT	IFNg release	Positive	1	1	1	0	0	0
186347	VPNYNLIVM	NS3	1749	1757	HLA-B*35:01	100%	Dengue virus 4	ELISPOT	IFNg release	Positive	1	1	1	0	0	0
195838	PNYNLIVMDEAHFTD	NS3	1750	1764	HLA class I	100%	Dengue virus 4	ICS	IFNg release	Positive	1	1	1	0	0	0
22822	GTSGSPIINRK	NS3	133	143	HLA-A11	100%	Dengue virus 4	51 chromium	cytotoxicity	Positive	1	1	1	0	0	0
34918	LAPTRVVAAEME	NS3	221	232	HLA-B7	100%	Dengue virus 4	51 chromium	cytotoxicity	Positive	1	1	1	0	0	0
539058	AHFTDPSSVAARGYIST	NS3	1760	1776	HLA class I	100%	Dengue virus 4	ICS	IFNg release	Positive	1	1	1	0	0	0
539576	LIVMDEAHFTDPSSVAA	NS3	1754	1770	HLA class I	100%	Dengue virus 4	ICS	IFNg release	Positive	1	1	1	0	0	0
540029	VEMGEAAAIFMTATPPG	NS3	1778	1794	HLA class I	100%	Dengue virus 4	ICS	IFNg release	Positive	1	1	1	0	0	0
581940	APTRVVAAEMEEALRGL	NS3	1558	1574	HLA class I	100%	Dengue virus 4	ICS	IFNg release	Positive-Low	1	1	1	0	0	0
65552	TPEGIIPTL	NS3	500	508	HLA-B*35:01	100%	Dengue virus 4	51 chromium	cytotoxicity	Positive	1	1	1	0	0	0
182926	EIASLPTYL	NS4a	2101	2109	HLA-A*68:02	100%	Dengue virus 4	ELISPOT	IFNg release	Positive	1	1	1	0	0	0
184531	LPESLETLML	NS4a	2140	2149	HLA-B*35:01	100%	Dengue virus 4	ELISPOT	IFNg release	Positive	1	1	1	0	0	0
185881	TEIASLPTYL	NS4a	2100	2109	HLA-B*40:01	100%	Dengue virus 4	ELISPOT	IFNg release	Positive	1	1	1	0	0	0
186620	YVILTILTI	NS4a	2228	2236	HLA-A*24:02	100%	Dengue virus 4	ELISPOT	IFNg release	Positive	1	1	1	0	0	0
150647	TPMLRHTIEN	NS4b	2288	2297	HLA-B*07:02	100%	Dengue virus 4	ELISPOT	IFNg release	Positive	1	1	1	0	0	0
180379	ATGPILTLW	NS4b	2436	2444	HLA-B*58:01	100%	Dengue virus 4	ELISPOT	IFNg release	Positive	1	1	1	0	0	0
180764	TETTILDVDL	NS4b	2262	2271	HLA-B*40:01	100%	Dengue virus 4	ELISPOT	IFNg release	Positive	1	1	1	0	0	0
180803	VPLLAMGCY	NS4b	2330	2338	HLA-B*35:01	100%	Dengue virus 4	ELISPOT	IFNg release	Positive	1	1	1	0	0	0
183672	IENTSANLSL	NS4b	2295	2304	HLA-B*40:01	100%	Dengue virus 4	ELISPOT	IFNg release	Positive	1	1	1	0	0	0
184453	LLLMRTTWAF	NS4b	2420	2429	HLA-A*24:02	100%	Dengue virus 4	ELISPOT	IFNg release	Positive	1	1	1	0	0	0
184713	LYAVATTIL	NS4b	2279	2287	HLA-A*24:02	100%	Dengue virus 4	ELISPOT	IFNg release	Positive	1	1	1	0	0	0
186330	VMLLVHYAI	NS4b	2351	2359	HLA-A*24:02	100%	Dengue virus 4	ELISPOT	IFNg release	Positive	1	0	1	0	0	0
37535	LLLMRTTWA	NS4b	76	84	HLA-A2	100%	Dengue virus 4	ICS	TNF release	Positive	1	1	1	0	0	0
40472	LVMLLVHYA	NS4b	6	14	HLA-A2	100%	Dengue virus 4	ICS	IFNg release	Positive	1	0	1	0	0	0
1067215	HSWEDIPYL	NS5	3319	3327	HLA-A*02:01	100%	Dengue virus 4	ELISPOT	IFNg release	Positive	1	1	1	0	0	0
180684	NPRLCTREEF	NS5	2877	2886	HLA-B*07:02	100%	Dengue virus 4	ELISPOT	IFNg release	Positive	1	1	1	0	0	0
180718	REDLWCGSL	NS5	3330	3338	HLA-B*40:01	100%	Dengue virus 4	ELISPOT	IFNg release	Positive	1	1	1	0	0	0
183353	GESSSNPTI	NS5	2635	2643	HLA-B*44:02	100%	Dengue virus 4	ELISPOT	IFNg release	Positive	1	1	1	0	0	0
183570	HEMYWVSGV	NS5	2703	2711	HLA-B*44:02	100%	Dengue virus 4	ELISPOT	IFNg release	Positive	0	0	0	0	0	0
184051	KLGEFGRAK	NS5	2948	2956	HLA-A*03:01	100%	Dengue virus 4	ELISPOT	IFNg release	Positive	1	1	1	0	0	0
184130	KREDLWCGSL	NS5	3329	3338	HLA-B*08:01	100%	Dengue virus 4	ELISPOT	IFNg release	Positive	1	1	1	0	0	0
184954	MTTEDMLKVW	NS5	3292	3301	HLA class I	100%	Dengue virus 4	ICS	IFNg release	Positive	1	1	1	0	0	0
186453	WEPSKGWKNW	NS5	3182	3191	HLA-B*44:02	100%	Dengue virus 4	ELISPOT	IFNg release	Positive	1	1	1	0	0	0
186550	YDQENPYRTW	NS5	2781	2790	HLA-B*44:02	100%	Dengue virus 4	ELISPOT	IFNg release	Positive	1	1	1	0	0	0
186640	YYMATLKNV	NS5	2576	2584	HLA-A*24:02	100%	Dengue virus 4	ELISPOT	IFNg release	Positive	1	1	1	0	0	0
186641	YYMATLKNVT	NS5	2576	2585	HLA-A*24:02	100%	Dengue virus 4	ELISPOT	IFNg release	Positive	1	1	1	0	0	0
539656	MAISGDDCVVKPLDERF	NS5	3146	3162	HLA class I	100%	Dengue virus 4	ICS	IFNg release	Positive	1	1	1	0	0	0
539703	MVTQLAMTDTTPFGQQR	NS5	2824	2840	HLA class I	100%	Dengue virus 4	ICS	IFNg release	Positive	1	1	1	0	0	0
180475	GEQRKTFVEL	NS3	2001	2010	HLA-B*40:01	81% - <100%	Dengue virus 4	ELISPOT	IFNg release	Positive	1	1	1	0	0	0
183231	FPQSNSPIED	NS3	1799	1808	HLA-B*35:01	81% - <100%	Dengue virus 4	ELISPOT	IFNg release	Positive	1	1	1	0	0	0
183338	GEIGAVTLDF	NS3	1595	1604	HLA-B*44:03	81% - <100%	Dengue virus 4	ELISPOT	IFNg release	Positive	1	1	1	0	0	0
62616	SYKDREWCF	NS3	389	397	HLA-A24	81% - <100%	Dengue virus 4	ELISPOT	IFNg release	Positive	1	1	1	0	0	0
180596	LETLMLVALL	NS4a	2144	2153	HLA-B*40:01	81% - <100%	Dengue virus 4	ELISPOT	IFNg release	Positive	1	1	1	0	0	0
182508	AIAVASGLLW	NS4a	2179	2188	HLA-B*44:02	81% - <100%	Dengue virus 4	ELISPOT	IFNg release	Positive	0	0	0	0	0	0
183650	IAVASGLLW	NS4a	2180	2188	HLA-B*57:01	81% - <100%	Dengue virus 4	ELISPOT	IFNg release	Positive	1	1	1	0	0	0
42083	MLVALLGAM	NS4a	56	64	HLA-A2	81% - <100%	Dengue virus 4	ICS	IFNg release	Positive	1	1	1	0	0	0
136826	CYSQVNPTTL	NS4b	2337	2346	HLA-A*24:02	81% - <100%	Dengue virus 4	ELISPOT	IFNg release	Positive	1	1	1	0	0	0
150548	RSNAAIGAVF	NS5	2891	2900	HLA-B*15:01	81% - <100%	Dengue virus 4	ELISPOT	IFNg release	Positive	1	1	1	0	0	0
185232	QEEHKETWHY	NS5	2772	2781	HLA-B*44:02	81% - <100%	Dengue virus 4	ELISPOT	IFNg release	Positive	1	1	1	0	0	0
539149	DCVVKPLDERFSTSLLF	NS5	3152	3168	HLA class I	81% - <100%	Dengue virus 4	ICS	IFNg release	Positive	0	0	0	0	0	0
184591	LSIPHDLMEF	NS2a	1239	1248	HLA-A*01:01	51%-80%	Dengue virus 4	ELISPOT	IFNg release	Positive	0	0	0	0	0	0
180850	YTPEGIIPTL	NS3	1973	1982	HLA-A*02:06	51%-80%	Dengue virus 4	ELISPOT	IFNg release	Positive	1	1	1	0	0	0
180541	ITLLCLIPTV	C	102	111	HLA-A*02:01	>0% - 50%	Dengue virus 4	ELISPOT	IFNg release	Positive	1	1	1	0	0	0
183818	IPHDLMELI	NS2a	1241	1249	HLA-B*53:01	>0% - 50%	Dengue virus 4	ELISPOT	IFNg release	Positive	1	1	1	0	0	0
184390	LIDGISLGL	NS2a	1248	1256	HLA-A*01:01	>0% - 50%	Dengue virus 4	ELISPOT	IFNg release	Positive	1	1	1	0	0	0

Supplementary Table S5. *Number of the complete polyprotein sequences of the dengue viral isolates from Thailand, Brazil and Mexico for each of the four dengue serotypes.*

Country	DENV-1 sequences	DENV-2 sequences	DENV-3 sequences	DENV-4 sequences	Total Sequences
**Thailand**	140	13	67	83	303
**Brazil**	25	35	45	36	141
**Mexico**	81	20	2	0	103

Supplementary Table S6. Alignment of CD4 and CD8 epitopes in circulating dengue viruses from Thailand and six vaccine candidates. *Detailed information, for Thailand, on each of the vaccine carried CD4 and CD8 T cell epitopes that are not present in any of the viral isolates, present in all of the viral isolates, present in only 81% – <100% or 51% – 80% or in only >0% – 50% of viral isolates for each of the epitope within each of the six vaccine candidates and the four dengue serotypes. The first table summarizes information retrieved on circulating viral isolate sequences available from Thailand. Next, the globally reported CD4/CD8 T cell epitopes are organized into: DENV-1 specific CD4 epitopes, DENV-2 specific CD4 epitopes, DENV-3 specific CD4 epitopes, DENV-4 specific CD4 epitopes, DENV-1 specific CD8 epitopes, DENV-2 specific CD8 epitopes, DENV-3 specific CD8 epitopes, DENV-4 specific CD8 epitopes. In each of these tables, identity of the epitope to each of the corresponding viral isolate serotype and each of the vaccine constructs are indicated. Green color shade and number 1 indicates 100% identity in the given virus isolate/vaccine construct. Pink color shade and number zero indicates not identical in the given virus isolate/vaccine construct.*

NCBI ID	Strain Name	Host	Date	Continent	Country	Prot_Name	VirusType
QFS19153.1	KDC0587A_06/15/2011	Human	2011-06-14	Asia	Thailand	polyprotein	Dengue virus 1
QFS19173.1	KDC1209A_11/05/2012	Human	2012-11-06	Asia	Thailand	polyprotein	Dengue virus 1
QFS19144.1	KDC0456A_02/07/2011	Human	2011-02-08	Asia	Thailand	polyprotein	Dengue virus 1
QFS19145.1	KDC0492A_04/21/2011	Human	2011-04-21	Asia	Thailand	polyprotein	Dengue virus 1
QFS19146.1	KDC0493A_04/21/2011	Human	2011-04-21	Asia	Thailand	polyprotein	Dengue virus 1
QFS19166.1	KDC0885A_11/29/2011	Human	2011-11-29	Asia	Thailand	polyprotein	Dengue virus 1
QFS19168.1	KDC1149A_10/10/2012	Human	2012-10-10	Asia	Thailand	polyprotein	Dengue virus 1
QFS19190.1	KDH0129A_06/02/2011	Human	2011-06-01	Asia	Thailand	polyprotein	Dengue virus 1
QFS19205.1	KDH0156A_07/21/2011	Human	2011-07-20	Asia	Thailand	polyprotein	Dengue virus 1
QFS19215.1	KDH0176A_08/23/2011	Human	2011-08-22	Asia	Thailand	polyprotein	Dengue virus 1
QFS19222.1	KDH0201A_11/28/2011	Human	2011-11-28	Asia	Thailand	polyprotein	Dengue virus 1
QFS19223.1	KDH0202A_11/29/2011	Human	2011-11-28	Asia	Thailand	polyprotein	Dengue virus 1
QFS19232.1	KDH0288A_09/11/2012	Human	2012-09-10	Asia	Thailand	polyprotein	Dengue virus 1
QFS19235.1	KDH0304A_10/02/2012	Human	2012-10-01	Asia	Thailand	polyprotein	Dengue virus 1
QTX92594.1	00750/11	Human	2011-01-01	Asia	Thailand	polyprotein	Dengue virus 1
QTX92758.1	00485/14	Human	2014-01-01	Asia	Thailand	polyprotein	Dengue virus 1
QTX92757.1	01937/06	Human	2006-01-01	Asia	Thailand	polyprotein	Dengue virus 1
QTX92448.1	00690/06	Human	2006-01-01	Asia	Thailand	polyprotein	Dengue virus 1
QTX92560.1	02673/06	Human	2006-01-01	Asia	Thailand	polyprotein	Dengue virus 1
QTX92606.1	00192/13	Human	2013-01-01	Asia	Thailand	polyprotein	Dengue virus 1
QTX92609.1	01616/05	Human	2005-01-01	Asia	Thailand	polyprotein	Dengue virus 1
QTX92611.1	00444/14	Human	2014-01-01	Asia	Thailand	polyprotein	Dengue virus 1
QTX92660.1	01578/05	Human	2005-01-01	Asia	Thailand	polyprotein	Dengue virus 1
QTX92680.1	01274/04	Human	2004-01-01	Asia	Thailand	polyprotein	Dengue virus 1
QTX92723.1	01133/12	Human	2012-01-01	Asia	Thailand	polyprotein	Dengue virus 1
QTX92771.1	02553/06	Human	2006-01-01	Asia	Thailand	polyprotein	Dengue virus 1
QTX92802.1	00817/06	Human	2006-01-01	Asia	Thailand	polyprotein	Dengue virus 1
QTX92829.1	01499/06	Human	2006-01-01	Asia	Thailand	polyprotein	Dengue virus 1
QTX92844.1	01535/06	Human	2006-01-01	Asia	Thailand	polyprotein	Dengue virus 1
QTX92575.1	00559/14	Human	2014-01-01	Asia	Thailand	polyprotein	Dengue virus 1
ANC57582.1	DENV1-384	Human	2009-01-01	Asia	Thailand	polyprotein	Dengue virus 1
AHI88623.1	DENV-1/8/Thailand/01/2013	Human	2013-01-01	Asia	Thailand	polyprotein	Dengue virus 1
QTX92652.1	00878/04	Human	2004-01-01	Asia	Thailand	polyprotein	Dengue virus 1

QTX92811.1	00479/04	Human	2004-01-01	Asia	Thailand	polyprotein	Dengue virus 1
QTX92858.1	02129/06	Human	2006-01-01	Asia	Thailand	polyprotein	Dengue virus 1
QTX92431.1	01302/10	Human	2010-01-01	Asia	Thailand	polyprotein	Dengue virus 1
QTX92434.1	00647/05	Human	2005-01-01	Asia	Thailand	polyprotein	Dengue virus 1
QTX92513.1	00840/12	Human	2012-01-01	Asia	Thailand	polyprotein	Dengue virus 1
QTX92537.1	02723/06	Human	2006-01-01	Asia	Thailand	polyprotein	Dengue virus 1
QTX92551.1	02499/06	Human	2006-01-01	Asia	Thailand	polyprotein	Dengue virus 1
QFS19164.1	KDC0760A_08/10/2011	Human	2011-08-11	Asia	Thailand	polyprotein	Dengue virus 1
QFS19187.1	KDH0102A_02/15/2011	Human	2011-02-11	Asia	Thailand	polyprotein	Dengue virus 1
QFS19199.1	KDH0144A_06/30/2011	Human	2011-06-29	Asia	Thailand	polyprotein	Dengue virus 1
QFS19203.1	KDH0152A_07/13/2011	Human	2011-07-12	Asia	Thailand	polyprotein	Dengue virus 1
QFS19233.1	KDH0289A_09/12/2012	Human	2012-09-11	Asia	Thailand	polyprotein	Dengue virus 1
QFS19237.1	KDH0309A_10/09/2012	Human	2012-10-09	Asia	Thailand	polyprotein	Dengue virus 1
QFS19239.1	KDH0323A_11/26/2012	Human	2012-11-26	Asia	Thailand	polyprotein	Dengue virus 1
QTX92593.1	01381/10	Human	2010-01-01	Asia	Thailand	polyprotein	Dengue virus 1
QTX92599.1	02776/06	Human	2006-01-01	Asia	Thailand	polyprotein	Dengue virus 1
QTX92604.1	00479/05	Human	2005-01-01	Asia	Thailand	polyprotein	Dengue virus 1
QTX92657.1	00258/05	Human	2005-01-01	Asia	Thailand	polyprotein	Dengue virus 1
QTX92662.1	02848/06	Human	2006-01-01	Asia	Thailand	polyprotein	Dengue virus 1
QTX92768.1	02717/06	Human	2006-01-01	Asia	Thailand	polyprotein	Dengue virus 1
QTX92879.1	01710/06	Human	2006-01-01	Asia	Thailand	polyprotein	Dengue virus 1
QTX92881.1	00758/14	Human	2014-01-01	Asia	Thailand	polyprotein	Dengue virus 1
QTX92571.1	00572/14	Human	2014-01-01	Asia	Thailand	polyprotein	Dengue virus 1
QFS19177.1	KDH0032A_07/07/2010	Human	2010-07-07	Asia	Thailand	polyprotein	Dengue virus 1
QTX92679.1	01453/11	Human	2011-01-01	Asia	Thailand	polyprotein	Dengue virus 1
QTX92686.1	00068/06	Human	2006-01-01	Asia	Thailand	polyprotein	Dengue virus 1
QTX92693.1	00432/11	Human	2011-01-01	Asia	Thailand	polyprotein	Dengue virus 1
QTX92726.1	00186/05	Human	2005-01-01	Asia	Thailand	polyprotein	Dengue virus 1
QTX92784.1	02789/06	Human	2006-01-01	Asia	Thailand	polyprotein	Dengue virus 1
QTX92808.1	01006/12	Human	2012-01-01	Asia	Thailand	polyprotein	Dengue virus 1
QTX92812.1	02585/06	Human	2006-01-01	Asia	Thailand	polyprotein	Dengue virus 1
QTX92815.1	02547/06	Human	2006-01-01	Asia	Thailand	polyprotein	Dengue virus 1
QTX92845.1	00208/05	Human	2005-01-01	Asia	Thailand	polyprotein	Dengue virus 1
QTX92847.1	01518/10	Human	2010-01-01	Asia	Thailand	polyprotein	Dengue virus 1

QTX92851.1	01510/10	Human	2010-01-01	Asia	Thailand	polyprotein	Dengue virus 1
QTX92867.1	00738/14	Human	2014-01-01	Asia	Thailand	polyprotein	Dengue virus 1
QTX92429.1	01313/04	Human	2004-01-01	Asia	Thailand	polyprotein	Dengue virus 1
QTX92443.1	00554/14	Human	2014-01-01	Asia	Thailand	polyprotein	Dengue virus 1
QTX92444.1	01040/05	Human	2005-01-01	Asia	Thailand	polyprotein	Dengue virus 1
QTX92486.1	01690/06	Human	2006-01-01	Asia	Thailand	polyprotein	Dengue virus 1
QTX92515.1	00369/04	Human	2004-01-01	Asia	Thailand	polyprotein	Dengue virus 1
QTX92516.1	01479/07	Human	2007-01-01	Asia	Thailand	polyprotein	Dengue virus 1
QTX92531.1	01377/06	Human	2006-01-01	Asia	Thailand	polyprotein	Dengue virus 1
QTX92541.1	02835/06	Human	2006-01-01	Asia	Thailand	polyprotein	Dengue virus 1
QTX92547.1	02725/06	Human	2006-01-01	Asia	Thailand	polyprotein	Dengue virus 1
QTX92557.1	01604/04	Human	2004-01-01	Asia	Thailand	polyprotein	Dengue virus 1
CDF77359.1	KDH0030A	Unknown	2010-01-01	Asia	Thailand	polyprotein	Dengue virus 1
AXS75991.1	Thai2013	Human	2013-01-01	Asia	Thailand	polyprotein	Dengue virus 1
QFS19160.1	KDC0650A_07/06/2011	Human	2011-07-07	Asia	Thailand	polyprotein	Dengue virus 1
QFS19185.1	KDH0087A_12/08/2010	Human	2010-12-07	Asia	Thailand	polyprotein	Dengue virus 1
QFS19207.1	KDH0161A_07/27/2011	Human	2011-07-26	Asia	Thailand	polyprotein	Dengue virus 1
QFS19234.1	KDH0295A_09/19/2012	Human	2012-09-18	Asia	Thailand	polyprotein	Dengue virus 1
QTX92586.1	02340/06	Human	2006-01-01	Asia	Thailand	polyprotein	Dengue virus 1
QTX92753.1	00493/11	Human	2011-01-01	Asia	Thailand	polyprotein	Dengue virus 1
QTX92761.1	02679/06	Human	2006-01-01	Asia	Thailand	polyprotein	Dengue virus 1
QTX92765.1	00841/06	Human	2006-01-01	Asia	Thailand	polyprotein	Dengue virus 1
QTX92445.1	01175/10	Human	2010-01-01	Asia	Thailand	polyprotein	Dengue virus 1
QTX92446.1	00943/12	Human	2012-01-01	Asia	Thailand	polyprotein	Dengue virus 1
QTX92452.1	02548/06	Human	2006-01-01	Asia	Thailand	polyprotein	Dengue virus 1
QTX92457.1	02296/06	Human	2006-01-01	Asia	Thailand	polyprotein	Dengue virus 1
QTX92627.1	00090/13	Human	2013-01-01	Asia	Thailand	polyprotein	Dengue virus 1
QTX92646.1	00002/06	Human	2006-01-01	Asia	Thailand	polyprotein	Dengue virus 1
QTX92655.1	00358/12	Human	2012-01-01	Asia	Thailand	polyprotein	Dengue virus 1
QTX92688.1	00458/06	Human	2006-01-01	Asia	Thailand	polyprotein	Dengue virus 1
QTX92689.1	01277/06	Human	2006-01-01	Asia	Thailand	polyprotein	Dengue virus 1
QTX92701.1	00975/04	Human	2004-01-01	Asia	Thailand	polyprotein	Dengue virus 1
QTX92725.1	01600/10	Human	2010-01-01	Asia	Thailand	polyprotein	Dengue virus 1
QTX92781.1	01651/05	Human	2005-01-01	Asia	Thailand	polyprotein	Dengue virus 1

QTX92800.1	01591/05	Human	2005-01-01	Asia	Thailand	polyprotein	Dengue virus 1
QTX92801.1	00272/11	Human	2011-01-01	Asia	Thailand	polyprotein	Dengue virus 1
QTX92846.1	00330/11	Human	2011-01-01	Asia	Thailand	polyprotein	Dengue virus 1
QTX92418.1	02592/06	Human	2006-01-01	Asia	Thailand	polyprotein	Dengue virus 1
QTX92538.1	00777/12	Human	2012-01-01	Asia	Thailand	polyprotein	Dengue virus 1
ADU03651.1	01096/07	Human	2007-07-27	Asia	Thailand	polyprotein	Dengue virus 1
ANC57592.1	DENV1-18805	Human	2012-01-01	Asia	Thailand	polyprotein	Dengue virus 1
QFS19172.1	KDC1153A_10/10/2012	Human	2012-10-10	Asia	Thailand	polyprotein	Dengue virus 1
QFS19175.1	KDH0026A_06/15/2010	Human	2010-06-15	Asia	Thailand	polyprotein	Dengue virus 1
QTX92861.1	00785/11	Human	2011-01-01	Asia	Thailand	polyprotein	Dengue virus 1
QTX92545.1	01411/04	Human	2004-01-01	Asia	Thailand	polyprotein	Dengue virus 1
QTX92553.1	00211/05	Human	2005-01-01	Asia	Thailand	polyprotein	Dengue virus 1
QTX92559.1	00371/06	Human	2006-01-01	Asia	Thailand	polyprotein	Dengue virus 1
BDB33210.1	Th15-DV1-TM002	Human	2015-01-11	Asia	Thailand	polyprotein	Dengue virus 1
QFS19159.1	KDC0640A2_06/30/2011	Human	2011-07-01	Asia	Thailand	polyprotein	Dengue virus 1
QFS19189.1	KDH0112A_04/21/2011	Human	2011-04-20	Asia	Thailand	polyprotein	Dengue virus 1
QFS19217.1	KDH0182A_09/07/2011	Human	2011-09-05	Asia	Thailand	polyprotein	Dengue virus 1
QFS19226.1	KDH0228A_06/07/2012	Human	2012-06-06	Asia	Thailand	polyprotein	Dengue virus 1
QPQ49765.1	P3.1 Thailand 2005	Human	2005-01-11	Asia	Thailand	polyprotein	Dengue virus 1
QTX92595.1	01530/06	Human	2006-01-01	Asia	Thailand	polyprotein	Dengue virus 1
QTX92612.1	00259/04	Human	2004-01-01	Asia	Thailand	polyprotein	Dengue virus 1
QTX92670.1	00349/11	Human	2011-01-01	Asia	Thailand	polyprotein	Dengue virus 1
QTX92745.1	00674/06	Human	2006-01-01	Asia	Thailand	polyprotein	Dengue virus 1
QTX92454.1	02344/06	Human	2006-01-01	Asia	Thailand	polyprotein	Dengue virus 1
QTX92462.1	00878/12	Human	2012-01-01	Asia	Thailand	polyprotein	Dengue virus 1
QTX92576.1	00438/06	Human	2006-01-01	Asia	Thailand	polyprotein	Dengue virus 1
QTX92577.1	02584/06	Human	2006-01-01	Asia	Thailand	polyprotein	Dengue virus 1
QFS19151.1	KDC0575A_06/02/2011	Human	2011-06-03	Asia	Thailand	polyprotein	Dengue virus 1
QTX92707.1	00059/13	Human	2013-01-01	Asia	Thailand	polyprotein	Dengue virus 1
QTX92713.1	02634/06	Human	2006-01-01	Asia	Thailand	polyprotein	Dengue virus 1
QTX92716.1	00543/12	Human	2012-01-01	Asia	Thailand	polyprotein	Dengue virus 1
QTX92718.1	00743/05	Human	2005-01-01	Asia	Thailand	polyprotein	Dengue virus 1
QTX92724.1	00369/06	Human	2006-01-01	Asia	Thailand	polyprotein	Dengue virus 1
QTX92857.1	01302/11	Human	2011-01-01	Asia	Thailand	polyprotein	Dengue virus 1

QTX92428.1	00343/13	Human	2013-01-01	Asia	Thailand	polyprotein	Dengue virus 1
QTX92489.1	01152/13	Human	2013-01-01	Asia	Thailand	polyprotein	Dengue virus 1
ANC57585.1	DENV1-2878	Human	2008-01-01	Asia	Thailand	polyprotein	Dengue virus 1
ANC57583.1	DENV1-1990	Human	2008-01-01	Asia	Thailand	polyprotein	Dengue virus 1
QFS19155.1	KDC0599A_06/21/2011	Human	2011-06-21	Asia	Thailand	polyprotein	Dengue virus 1
ANC57599.1	DENV2-3229	Human	2011-01-01	Asia	Thailand	polyprotein	Dengue virus 2
QXY23100.1	DV2C-TM19-26	Human	2019-11-24	Asia	Thailand	polyprotein	Dengue virus 2
QXY23099.1	DV2A-TM19-13	Human	2019-11-19	Asia	Thailand	polyprotein	Dengue virus 2
BBG31508.1	Th16-026DV2	Human	2016-01-10	Asia	Thailand	polyprotein	Dengue virus 2
QBK46950.1	16681-2007	Unknown	2007-01-06	Asia	Thailand	polyprotein	Dengue virus 2
ANC57598.1	DENV2-1365	Human	2009-01-01	Asia	Thailand	polyprotein	Dengue virus 2
BBG31503.1	Th16-035DV2	Human	2016-01-11	Asia	Thailand	polyprotein	Dengue virus 2
BBG31505.1	Th17-061DV2	Human	2017-01-01	Asia	Thailand	polyprotein	Dengue virus 2
QFS19150.1	KDC0574A2_06/02/2011	Human	2011-06-04	Asia	Thailand	polyprotein	Dengue virus 2
QBA29681.1	CNR_25326	Human	2014-08-15	Asia	Thailand	polyprotein	Dengue virus 2
QXY23103.1	DV2C-TM19-80	Human	2019-12-30	Asia	Thailand	polyprotein	Dengue virus 2
BBG31506.1	Th17-074DV2	Human	2017-01-02	Asia	Thailand	polyprotein	Dengue virus 2
BBG31507.1	Th16-011DV2	Human	2016-01-10	Asia	Thailand	polyprotein	Dengue virus 2
BBG31513.1	Th17-059DV3	Human	2017-01-01	Asia	Thailand	polyprotein	Dengue virus 3
QTX93239.1	01434/10	Human	2010-01-01	Asia	Thailand	polyprotein	Dengue virus 3
QTX93295.1	02185/06	Human	2006-01-01	Asia	Thailand	polyprotein	Dengue virus 3
QTX93456.1	01530/05	Human	2005-01-01	Asia	Thailand	polyprotein	Dengue virus 3
QTX93571.1	01284/06	Human	2006-01-01	Asia	Thailand	polyprotein	Dengue virus 3
QTX93578.1	01536/06	Human	2006-01-01	Asia	Thailand	polyprotein	Dengue virus 3
QTX93347.1	01131/12	Human	2012-01-01	Asia	Thailand	polyprotein	Dengue virus 3
QTX93386.1	02763/06	Human	2006-01-01	Asia	Thailand	polyprotein	Dengue virus 3
QTX93396.1	01400/05	Human	2005-01-01	Asia	Thailand	polyprotein	Dengue virus 3
QTX93427.1	00608/12	Human	2012-01-01	Asia	Thailand	polyprotein	Dengue virus 3
QTX93468.1	00999/12	Human	2012-01-01	Asia	Thailand	polyprotein	Dengue virus 3
QTX93479.1	02490/06	Human	2006-01-01	Asia	Thailand	polyprotein	Dengue virus 3
QTX93537.1	00288/06	Human	2006-01-01	Asia	Thailand	polyprotein	Dengue virus 3
QTX93597.1	01569/05	Human	2005-01-01	Asia	Thailand	polyprotein	Dengue virus 3
QBA29682.1	CNR_15418	Human	2012-07-23	Asia	Thailand	polyprotein	Dengue virus 3
QTX93435.1	01385/10	Human	2010-01-01	Asia	Thailand	polyprotein	Dengue virus 3

QTX93561.1	00923/11	Human	2011-01-01	Asia	Thailand	polyprotein	Dengue virus 3
QTX93564.1	01253/06	Human	2006-01-01	Asia	Thailand	polyprotein	Dengue virus 3
BBG31510.1	Th16-016DV3	Human	2016-01-10	Asia	Thailand	polyprotein	Dengue virus 3
QTX93258.1	01045/06	Human	2006-01-01	Asia	Thailand	polyprotein	Dengue virus 3
QTX93260.1	00695/13	Human	2013-01-01	Asia	Thailand	polyprotein	Dengue virus 3
QTX93237.1	00266/12	Human	2012-01-01	Asia	Thailand	polyprotein	Dengue virus 3
QTX93241.1	00710/06	Human	2006-01-01	Asia	Thailand	polyprotein	Dengue virus 3
QTX93288.1	01398/08	Human	2008-01-01	Asia	Thailand	polyprotein	Dengue virus 3
QTX93317.1	00432/14	Human	2014-01-01	Asia	Thailand	polyprotein	Dengue virus 3
QTX93321.1	00739/14	Human	2014-01-01	Asia	Thailand	polyprotein	Dengue virus 3
QTX93389.1	01278/11	Human	2011-01-01	Asia	Thailand	polyprotein	Dengue virus 3
QTX93391.1	01508/10	Human	2010-01-01	Asia	Thailand	polyprotein	Dengue virus 3
QTX93470.1	00654/14	Human	2014-01-01	Asia	Thailand	polyprotein	Dengue virus 3
QTX93527.1	00372/06	Human	2006-01-01	Asia	Thailand	polyprotein	Dengue virus 3
QTX93565.1	02784/06	Human	2006-01-01	Asia	Thailand	polyprotein	Dengue virus 3
QTX93594.1	02292/06	Human	2006-01-01	Asia	Thailand	polyprotein	Dengue virus 3
QTX93331.1	01401/13	Human	2013-01-01	Asia	Thailand	polyprotein	Dengue virus 3
QTX93412.1	00065/13	Human	2013-01-01	Asia	Thailand	polyprotein	Dengue virus 3
QTX93547.1	01651/06	Human	2006-01-01	Asia	Thailand	polyprotein	Dengue virus 3
ANC57606.1	DENV3-1631	Human	2011-01-01	Asia	Thailand	polyprotein	Dengue virus 3
QTX93249.1	02380/06	Human	2006-01-01	Asia	Thailand	polyprotein	Dengue virus 3
QTX93252.1	00852/05	Human	2005-01-01	Asia	Thailand	polyprotein	Dengue virus 3
QTX93291.1	01445/11	Human	2011-01-01	Asia	Thailand	polyprotein	Dengue virus 3
QTX93442.1	02678/06	Human	2006-01-01	Asia	Thailand	polyprotein	Dengue virus 3
QTX93493.1	00557/06	Human	2006-01-01	Asia	Thailand	polyprotein	Dengue virus 3
QTX93499.1	00507/14	Human	2014-01-01	Asia	Thailand	polyprotein	Dengue virus 3
QTX93408.1	00872/05	Human	2005-01-01	Asia	Thailand	polyprotein	Dengue virus 3
QTX93410.1	00817/12	Human	2012-01-01	Asia	Thailand	polyprotein	Dengue virus 3
QTX93416.1	01029/10	Human	2010-01-01	Asia	Thailand	polyprotein	Dengue virus 3
QTX93429.1	00759/14	Human	2014-01-01	Asia	Thailand	polyprotein	Dengue virus 3
QTX93518.1	01035/11	Human	2011-01-01	Asia	Thailand	polyprotein	Dengue virus 3
QTX93539.1	02814/06	Human	2006-01-01	Asia	Thailand	polyprotein	Dengue virus 3
QTX93589.1	01251/06	Human	2006-01-01	Asia	Thailand	polyprotein	Dengue virus 3
QTX93602.1	01257/06	Human	2006-01-01	Asia	Thailand	polyprotein	Dengue virus 3

AMA21734.1	Pythium	Parasitic Oomycote	2014-08-28	Asia	Thailand	polyprotein	Dengue virus 3
QTX93362.1	00638/12	Human	2012-01-01	Asia	Thailand	polyprotein	Dengue virus 3
QTX93251.1	00798/12	Human	2012-01-01	Asia	Thailand	polyprotein	Dengue virus 3
QTX93374.1	01467/10	Human	2010-01-01	Asia	Thailand	polyprotein	Dengue virus 3
QTX93406.1	00912/13	Human	2013-01-01	Asia	Thailand	polyprotein	Dengue virus 3
QTX93451.1	02704/06	Human	2006-01-01	Asia	Thailand	polyprotein	Dengue virus 3
QTX93458.1	01423/06	Human	2006-01-01	Asia	Thailand	polyprotein	Dengue virus 3
QTX93484.1	01601/10	Human	2010-01-01	Asia	Thailand	polyprotein	Dengue virus 3
QTX93495.1	00831/06	Human	2006-01-01	Asia	Thailand	polyprotein	Dengue virus 3
QTX93504.1	00651/06	Human	2006-01-01	Asia	Thailand	polyprotein	Dengue virus 3
QTX93507.1	00749/12	Human	2012-01-01	Asia	Thailand	polyprotein	Dengue virus 3
QTX93524.1	01041/06	Human	2006-01-01	Asia	Thailand	polyprotein	Dengue virus 3
QTX93529.1	00323/11	Human	2011-01-01	Asia	Thailand	polyprotein	Dengue virus 3
QTX93595.1	01379/06	Human	2006-01-01	Asia	Thailand	polyprotein	Dengue virus 3
QTX93603.1	00112/06	Human	2006-01-01	Asia	Thailand	polyprotein	Dengue virus 3
QTX93540.1	00509/11	Human	2011-01-01	Asia	Thailand	polyprotein	Dengue virus 3
QBK46952.1	h87-2007	Unknown	2007-01-08	Asia	Thailand	polyprotein	Dengue virus 3
ASS36950.1	CTI2-13	Unknown	2013-01-06	Asia	Thailand	polyprotein	Dengue virus 4
BBG31516.1	Th16-010DV4	Human	2016-01-10	Asia	Thailand	polyprotein	Dengue virus 4
BBG31520.1	Th17-091DV4	Human	2017-01-06	Asia	Thailand	polyprotein	Dengue virus 4
QTX92101.1	01107/06	Human	2006-01-01	Asia	Thailand	polyprotein	Dengue virus 4
QTX92124.1	00789/13	Human	2013-01-01	Asia	Thailand	polyprotein	Dengue virus 4
QTX92133.1	00097/04	Human	2004-01-01	Asia	Thailand	polyprotein	Dengue virus 4
QTX92164.1	00471/08	Human	2008-01-01	Asia	Thailand	polyprotein	Dengue virus 4
QTX92178.1	00567/04	Human	2004-01-01	Asia	Thailand	polyprotein	Dengue virus 4
QTX92188.1	01862/06	Human	2006-01-01	Asia	Thailand	polyprotein	Dengue virus 4
QTX92199.1	00789/14	Human	2014-01-01	Asia	Thailand	polyprotein	Dengue virus 4
QTX92226.1	01622/04	Human	2004-01-01	Asia	Thailand	polyprotein	Dengue virus 4
QTX92377.1	01268/08	Human	2008-01-01	Asia	Thailand	polyprotein	Dengue virus 4
QTX92269.1	00674/04	Human	2004-01-01	Asia	Thailand	polyprotein	Dengue virus 4
QTX92280.1	00225/06	Human	2006-01-01	Asia	Thailand	polyprotein	Dengue virus 4
QTX92286.1	01295/06	Human	2006-01-01	Asia	Thailand	polyprotein	Dengue virus 4
QTX92304.1	00746/04	Human	2004-01-01	Asia	Thailand	polyprotein	Dengue virus 4
QTX92312.1	00109/06	Human	2006-01-01	Asia	Thailand	polyprotein	Dengue virus 4

QTX92394.1	01769/04	Human	2004-01-01	Asia	Thailand	polyprotein	Dengue virus 4
AIG60035.1	VIROAF8	Human	2006-05-26	Asia	Thailand	polyprotein	Dengue virus 4
QTX92358.1	02456/06	Human	2006-01-01	Asia	Thailand	polyprotein	Dengue virus 4
QTX92370.1	00842/06	Human	2006-01-01	Asia	Thailand	polyprotein	Dengue virus 4
QTE18637.1	PDK48	Human	2021-01-01	Asia	Thailand	polyprotein	Dengue virus 4
QTX92135.1	01923/07	Human	2007-01-01	Asia	Thailand	polyprotein	Dengue virus 4
QTX92136.1	00369/11	Human	2011-01-01	Asia	Thailand	polyprotein	Dengue virus 4
QTX92142.1	00685/14	Human	2014-01-01	Asia	Thailand	polyprotein	Dengue virus 4
QTX92146.1	01130/07	Human	2007-01-01	Asia	Thailand	polyprotein	Dengue virus 4
QTX92150.1	00694/07	Human	2007-01-01	Asia	Thailand	polyprotein	Dengue virus 4
QTX92207.1	01380/08	Human	2008-01-01	Asia	Thailand	polyprotein	Dengue virus 4
QTX92209.1	00311/06	Human	2006-01-01	Asia	Thailand	polyprotein	Dengue virus 4
QTX92210.1	01711/06	Human	2006-01-01	Asia	Thailand	polyprotein	Dengue virus 4
QTX92096.1	00650/06	Human	2006-01-01	Asia	Thailand	polyprotein	Dengue virus 4
QTX92109.1	01258/04	Human	2004-01-01	Asia	Thailand	polyprotein	Dengue virus 4
QTX92117.1	00564/04	Human	2004-01-01	Asia	Thailand	polyprotein	Dengue virus 4
QTX92130.1	00171/06	Human	2006-01-01	Asia	Thailand	polyprotein	Dengue virus 4
QTX92180.1	00307/07	Human	2007-01-01	Asia	Thailand	polyprotein	Dengue virus 4
QTX92241.1	01993/07	Human	2007-01-01	Asia	Thailand	polyprotein	Dengue virus 4
QTX92247.1	00777/14	Human	2014-01-01	Asia	Thailand	polyprotein	Dengue virus 4
QTX92265.1	01494/05	Human	2005-01-01	Asia	Thailand	polyprotein	Dengue virus 4
QTX92267.1	00270/06	Human	2006-01-01	Asia	Thailand	polyprotein	Dengue virus 4
QTX92283.1	02383/06	Human	2006-01-01	Asia	Thailand	polyprotein	Dengue virus 4
QTX92292.1	00965/04	Human	2004-01-01	Asia	Thailand	polyprotein	Dengue virus 4
QTX92328.1	00717/04	Human	2004-01-01	Asia	Thailand	polyprotein	Dengue virus 4
QTX92383.1	01928/06	Human	2006-01-01	Asia	Thailand	polyprotein	Dengue virus 4
QTX92369.1	01278/06	Human	2006-01-01	Asia	Thailand	polyprotein	Dengue virus 4
QTX92371.1	02392/07	Human	2007-01-01	Asia	Thailand	polyprotein	Dengue virus 4
QTX92093.1	00575/14	Human	2014-01-01	Asia	Thailand	polyprotein	Dengue virus 4
QTX92104.1	01559/09	Human	2009-01-01	Asia	Thailand	polyprotein	Dengue virus 4
QTX92106.1	02606/06	Human	2006-01-01	Asia	Thailand	polyprotein	Dengue virus 4
QTX92152.1	00637/12	Human	2012-01-01	Asia	Thailand	polyprotein	Dengue virus 4
QTX92157.1	00618/05	Human	2005-01-01	Asia	Thailand	polyprotein	Dengue virus 4
QTX92162.1	01174/06	Human	2006-01-01	Asia	Thailand	polyprotein	Dengue virus 4

QTX92173.1	00524/06	Human	2006-01-01	Asia	Thailand	polyprotein	Dengue virus 4
QTX92184.1	01012/13	Human	2013-01-01	Asia	Thailand	polyprotein	Dengue virus 4
QTX92187.1	01382/06	Human	2006-01-01	Asia	Thailand	polyprotein	Dengue virus 4
QTX92192.1	01270/10	Human	2010-01-01	Asia	Thailand	polyprotein	Dengue virus 4
QTX92201.1	01677/10	Human	2010-01-01	Asia	Thailand	polyprotein	Dengue virus 4
QTX92309.1	00775/05	Human	2005-01-01	Asia	Thailand	polyprotein	Dengue virus 4
QTX92393.1	00365/14	Human	2014-01-01	Asia	Thailand	polyprotein	Dengue virus 4
QTX92342.1	00728/14	Human	2014-01-01	Asia	Thailand	polyprotein	Dengue virus 4
QTX92347.1	00714/05	Human	2005-01-01	Asia	Thailand	polyprotein	Dengue virus 4
BBG31515.1	Th16-006DV4	Human	2016-01-09	Asia	Thailand	polyprotein	Dengue virus 4
BBG31521.1	Th17-098DV4	Human	2017-01-06	Asia	Thailand	polyprotein	Dengue virus 4
QTX92111.1	02674/06	Human	2006-01-01	Asia	Thailand	polyprotein	Dengue virus 4
QTX92113.1	01261/06	Human	2006-01-01	Asia	Thailand	polyprotein	Dengue virus 4
QTX92138.1	01270/06	Human	2006-01-01	Asia	Thailand	polyprotein	Dengue virus 4
QTX92154.1	02198/06	Human	2006-01-01	Asia	Thailand	polyprotein	Dengue virus 4
QTX92167.1	00801/13	Human	2013-01-01	Asia	Thailand	polyprotein	Dengue virus 4
QTX92183.1	00854/05	Human	2005-01-01	Asia	Thailand	polyprotein	Dengue virus 4
QTX92189.1	00274/08	Human	2008-01-01	Asia	Thailand	polyprotein	Dengue virus 4
QTX92200.1	01009/05	Human	2005-01-01	Asia	Thailand	polyprotein	Dengue virus 4
QTX92203.1	01579/06	Human	2006-01-01	Asia	Thailand	polyprotein	Dengue virus 4
QTX92204.1	01391/07	Human	2007-01-01	Asia	Thailand	polyprotein	Dengue virus 4
QTX92129.1	02187/06	Human	2006-01-01	Asia	Thailand	polyprotein	Dengue virus 4
QTX92220.1	00433/07	Human	2007-01-01	Asia	Thailand	polyprotein	Dengue virus 4
QTX92225.1	00752/14	Human	2014-01-01	Asia	Thailand	polyprotein	Dengue virus 4
QTX92231.1	00514/14	Human	2014-01-01	Asia	Thailand	polyprotein	Dengue virus 4
QTX92256.1	00615/14	Human	2014-01-01	Asia	Thailand	polyprotein	Dengue virus 4
QTX92293.1	02498/06	Human	2006-01-01	Asia	Thailand	polyprotein	Dengue virus 4
QTX92319.1	02194/06	Human	2006-01-01	Asia	Thailand	polyprotein	Dengue virus 4
QTX92335.1	01104/12	Human	2012-01-01	Asia	Thailand	polyprotein	Dengue virus 4
QTX92337.1	00367/06	Human	2006-01-01	Asia	Thailand	polyprotein	Dengue virus 4
QTX92380.1	00781/09	Human	2009-01-01	Asia	Thailand	polyprotein	Dengue virus 4
QTX92357.1	01719/04	Human	2004-01-01	Asia	Thailand	polyprotein	Dengue virus 4

CD4 DENV-1 Epitopes

Epitope_ID	AA_Seq	Protein_Name	Start	End	Allele	step	Serotype	Method/Technique	Assay Group	Qualitative Measure	QFS19153.1	QFS19173.1	QFS19144.1	[illegible]
195246	EISNMLNIMNRRKRT	C	87	101	HLA-DRB1*13:01	0%	Dengue virus 1	ELISPOT	IFNg release	Positive	0	0	0	[illegible]
195314	FNMLKRARNRVSTGS	C	1	15	HLA-DRB1*08:02	0%	Dengue virus 1	ELISPOT	IFNg release	Positive	0	0	0	[illegible]
195560	KEVSNMLNIMNRRKR	C	86	100	HLA-DRB1*13:01	0%	Dengue virus 1	ELISPOT	IFNg release	Positive	0	0	0	[illegible]
195586	KRSVTMLLMLLPTAL	C	87	101	HLA-DRB1*01:01	0%	Dengue virus 1	ELISPOT	IFNg release	Positive	0	0	0	[illegible]
195681	LMLLPTALAFHLTTR	C	94	108	HLA-DRB1*04:01	0%	Dengue virus 1	ELISPOT	IFNg release	Positive	0	0	0	[illegible]
195884	RGFKKEISNMLSIMN	C	82	96	HLA-DRB1*04:01	0%	Dengue virus 1	ELISPOT	IFNg release	Positive	0	0	0	[illegible]
195944	SFNMLKRARNRVSTG	C	12	26	HLA-DRB1*11:01	0%	Dengue virus 1	ELISPOT	IFNg release	Positive	0	0	0	[illegible]
196043	TGRPSFNMLKRARNR	C	8	22	HLA-DRB1*11:01	0%	Dengue virus 1	ELISPOT	IFNg release	Positive	0	0	0	[illegible]
196188	VSVLFMLLPTALAFH	C	102	116	HLA-DRB1*01:01	0%	Dengue virus 1	ELISPOT	IFNg release	Positive	0	0	0	[illegible]
196195	VTMLFMLLPTALAFH	C	102	116	HLA-DRB1*04:01	0%	Dengue virus 1	ELISPOT	IFNg release	Positive	0	0	0	[illegible]
196196	VTMLLMLLPTALAFH	C	90	104	HLA-DRB1*04:01	0%	Dengue virus 1	ELISPOT	IFNg release	Positive	0	0	0	[illegible]
539048	AFMAFLRFLAIPPTA	C	27	41	HLA-DRB1*15:02	0%	Dengue virus 1	ELISPOT	IFNg release	Positive	0	0	0	[illegible]
539681	MLNIRNRRKRSVTML	C	91	105	HLA-DRB1*13:01	0%	Dengue virus 1	ELISPOT	IFNg release	Positive	0	0	0	[illegible]
539816	QPSFNMLKRARNRVS	C	10	24	HLA-DRB1*08:03	0%	Dengue virus 1	ELISPOT	IFNg release	Positive	0	0	0	[illegible]
195359	GGLFTSVGKLIHQVF	E	706	720	HLA-DRB1*11:01	0%	Dengue virus 1	ELISPOT	IFNg release	Positive	0	0	0	[illegible]
195362	GGVFTSVGKLVHQVF	E	426	440	HLA-DRB1*11:01	0%	Dengue virus 1	ELISPOT	IFNg release	Positive	0	0	0	[illegible]
195484	IGGLFTSVGKLIHQV	E	705	719	HLA-DRB1*07:01	0%	Dengue virus 1	ELISPOT	IFNg release	Positive	0	0	0	[illegible]
539369	GVSWTMKIGIGILLT	E	445	459	HLA-DRB1*15:02	0%	Dengue virus 1	ELISPOT	IFNg release	Positive	0	0	0	[illegible]
539670	MKIGIGILLTWLGLN	E	450	464	HLA-DRB1*15:06	0%	Dengue virus 1	ELISPOT	IFNg release	Positive	0	0	0	[illegible]
539840	REDLLVTFKTAHAKK	E	513	527	HLA-DRB1*04:03	0%	Dengue virus 1	ELISPOT	IFNg release	Positive	0	0	0	[illegible]
539854	RKDLLVTFKTAHAKK	E	513	527	HLA-DRB1*04:03	0%	Dengue virus 1	ELISPOT	IFNg release	Positive	0	0	0	[illegible]
741745	MRCVGIGNRDFVEGLSGATW	E	281	300	HLA class II	0%	Dengue virus 1	ELISPOT	IFNg release	Positive	0	0	0	[illegible]
867045	HAKKQEVAVLGSQEG	E	244	258	HLA-DQA1*03:01/DQB1*03:02	0%	Dengue virus 1	ELISPOT	IFNg release	Positive	0	0	0	[illegible]
869085	SYIVIGAGKKALKLS	E	376	390	HLA-DRB5*01:01	0%	Dengue virus 1	ELISPOT	IFNg release	Positive	0	0	0	[illegible]
869690	WLAHKQWFLDLPLPW	E	206	220	HLA-DQA1*01:01/DQB1*05:01	0%	Dengue virus 1	ELISPOT	IFNg release	Positive	0	0	0	[illegible]
195684	LNHILLENDIKFTVV	NS1	850	864	HLA-DRB1*03:01	0%	Dengue virus 1	ELISPOT	IFNg release	Positive	0	0	0	[illegible]
195690	LNYILLENDMKFTVV	NS1	850	864	HLA-DRB1*03:01	0%	Dengue virus 1	ELISPOT	IFNg release	Positive	0	0	0	[illegible]
195785	NETWKLTRASFIEVK	NS1	982	996	HLA-DRB1*07:01	0%	Dengue virus 1	ELISPOT	IFNg release	Positive	0	0	0	[illegible]
591686	KFTVVVGDVSGILAQ	NS1	860	874	HLA-DRB1*03:01	0%	Dengue virus 1	ELISPOT	IFNg release	Positive	0	0	0	[illegible]
738503	ANGILAQGKKMIRPQ	NS1	868	882	HLA-DRB1*14:06	0%	Dengue virus 1	ELISPOT	IFNg release	Positive	0	0	0	[illegible]
740562	TVVVGDANGILAQGK	NS1	862	876	HLA-DRB1*01:02	0%	Dengue virus 1	ELISPOT	IFNg release	Positive	0	0	0	[illegible]
867405	KIIGADIQNTTFIID	NS1	897	911	HLA-DPB1*04:02	0%	Dengue virus 1	ELISPOT	IFNg release	Positive	0	0	0	[illegible]
868492	QISNELNYILLENDM	NS1	845	859	HLA-DPB1*01:01	0%	Dengue virus 1	ELISPOT	IFNg release	Positive	0	0	0	[illegible]
868630	QYKFQADSPKKLSAA	NS1	806	820	HLA-DRB3*02:02	0%	Dengue virus 1	ELISPOT	IFNg release	Positive	0	0	0	[illegible]
195196	AWKTMAMALSIVSLF	NS2a	1291	1305	HLA-DRB1*08:02	0%	Dengue virus 1	ELISPOT	IFNg release	Positive	0	0	0	[illegible]
195285	FAVGLLFRRLTSREI	NS2a	1214	1228	HLA-DRB1*11:01	0%	Dengue virus 1	ELISPOT	IFNg release	Positive	0	0	0	[illegible]
195389	GLLFRKLTSREVLLL	NS2a	1217	1231	HLA-DRB1*11:01	0%	Dengue virus 1	ELISPOT	IFNg release	Positive	0	0	0	[illegible]
195390	GLLFRRLTSREILLL	NS2a	1217	1231	HLA-DRB1*07:01	0%	Dengue virus 1	ELISPOT	IFNg release	Positive	0	0	0	[illegible]
195392	GLLFRRLTSREVPLL	NS2a	1217	1231	HLA-DRB1*10:01	0%	Dengue virus 1	ELISPOT	IFNg release	Positive	0	0	0	[illegible]
195713	LSLTFIRTSLSLDYA	NS2a	1277	1291	HLA-DRB1*03:01	0%	Dengue virus 1	ELISPOT	IFNg release	Positive	0	0	0	[illegible]
195756	MGLLFRRLTSREVLL	NS2a	1216	1230	HLA-DRB1*11:01	0%	Dengue virus 1	ELISPOT	IFNg release	Positive	0	0	0	[illegible]
195881	REVLLLTVGLSLVAC	NS2a	1226	1240	HLA-DRB1*07:01	0%	Dengue virus 1	ELISPOT	IFNg release	Positive	0	0	0	[illegible]
539625	LSLTFIKTTYSLHYA	NS2a	1277	1291	HLA-DRB1*14:04	0%	Dengue virus 1	ELISPOT	IFNg release	Positive	0	0	0	[illegible]
539626	LSLTFIRSTIPLVMA	NS2a	1275	1289	HLA-DRB1*14:04	0%	Dengue virus 1	ELISPOT	IFNg release	Positive	0	0	0	[illegible]
539651	LVVFLLLIMGQLTWS	NS2a	1164	1178	HLA-DRB1*15:06	0%	Dengue virus 1	ELISPOT	IFNg release	Positive	0	0	0	[illegible]
539657	MALSIVSLFPLCLST	NS2a	1297	1311	HLA-DRB1*15:06	0%	Dengue virus 1	ELISPOT	IFNg release	Positive	0	0	0	[illegible]
539912	SLDYAWKTMAMALSI	NS2a	1287	1301	HLA-DRB1*09:01	0%	Dengue virus 1	ELISPOT	IFNg release	Positive	0	0	0	[illegible]
539918	SLTFIKTTLSLHHAW	NS2a	1278	1292	HLA-DRB1*14:04	0%	Dengue virus 1	ELISPOT	IFNg release	Positive	0	0	0	[illegible]
539974	TGTLVVFLLLTLGQL	NS2a	1161	1175	HLA-DRB1*15:06	0%	Dengue virus 1	ELISPOT	IFNg release	Positive	0	0	0	[illegible]
540006	TTLLSLTFIRTSLSL	NS2a	1274	1288	HLA-DRB1*12:02	0%	Dengue virus 1	ELISPOT	IFNg release	Positive	0	0	0	[illegible]
591779	TLAVFLLLTMGQLTW	NS2a	36	50	HLA-DRB1*11:01	0%	Dengue virus 1	ELISPOT	IFNg release	Positive	0	0	0	[illegible]
739424	LAVFLLLVMGQLTWN	NS2a	1164	1178	HLA-DRB1*11:04	0%	Dengue virus 1	ELISPOT	IFNg release	Positive	0	0	0	[illegible]
739578	LSLTFIKTTLSLHHA	NS2a	1277	1291	HLA-DRB1*04:07	0%	Dengue virus 1	ELISPOT	IFNg release	Positive	0	0	0	[illegible]
866118	AMALSIVSLFPLCLS	NS2a	1296	1310	HLA-DPB1*04:01	0%	Dengue virus 1	ELISPOT	IFNg release	Positive	0	0	0	[illegible]
867997	LTSREILLLTVGLSL	NS2a	1223	1237	HLA-DPB1*04:02	0%	Dengue virus 1	ELISPOT	IFNg release	Positive	0	0	0	[illegible]
868553	QPHQLWATLLSLTFV	NS2a	1268	1282	HLA-DPB1*04:02	0%	Dengue virus 1	ELISPOT	IFNg release	Positive	0	0	0	[illegible]
869103	TDFQPHQLWTTLLSL	NS2a	1265	1279	HLA-DPB1*04:02	0%	Dengue virus 1	ELISPOT	IFNg release	Positive	0	0	0	[illegible]
866200	ATLFLWYFWQKKKQR	NS2b	1461	1475	HLA-DPB1*01:01	0%	Dengue virus 1	ELISPOT	IFNg release	Positive	0	0	0	[illegible]
195377	GIYRILQRGLLGRSQ	NS3	1496	1510	HLA-DRB1*11:01	0%	Dengue virus 1	ELISPOT	IFNg release	Positive	0	0	0	[illegible]
195549	IYRILQRGLLGRSQV	NS3	1497	1511	HLA-DRB1*15:01	0%	Dengue virus 1	ELISPOT	IFNg release	Positive	0	0	0	[illegible]
196138	VLDDGIYRILQRGLL	NS3	1492	1506	HLA-DRB1*11:01	0%	Dengue virus 1	ELISPOT	IFNg release	Positive	0	0	0	[illegible]
24915	HTGKEIVDLMCHATE	NS3	251	265	HLA-DPw2	0%	Dengue virus 1	ICS	IFNg release	Positive	0	0	0	[illegible]
539155	DGIYRILQRGLLGRS	NS3	1495	1509	HLA-DRB1*12:02	0%	Dengue virus 1	ELISPOT	IFNg release	Positive	0	0	0	[illegible]
539543	LDDGIYRILQRGLLG	NS3	1493	1507	HLA-DRB1*12:02	0%	Dengue virus 1	ELISPOT	IFNg release	Positive	0	0	0	[illegible]
539839	REAIRRKLRTLILAP	NS3	1685	1699	HLA-DRB1*14:04	0%	Dengue virus 1	ELISPOT	IFNg release	Positive	0	0	0	[illegible]
539993	TNCLRKNGKRVIQLS	NS3	1848	1862	HLA-DRB1*13:01	0%	Dengue virus 1	ELISPOT	IFNg release	Positive	0	0	0	[illegible]
540071	VREAIKKKLRTLILA	NS3	1684	1698	HLA-DRB1*12:02	0%	Dengue virus 1	ELISPOT	IFNg release	Positive	0	0	0	[illegible]
540075	VREAIKRKLRTLVLA	NS3	1684	1698	HLA-DRB1*12:02	0%	Dengue virus 1	ELISPOT	IFNg release	Positive	0	0	0	[illegible]
54391	RKLTIMDLHPGSGKT	NS3	1662	1676	HLA class II	0%	Dengue virus 1	ICS	IFNg release	Positive	0	0	0	[illegible]
738417	AAAIFMTATPPGSAE	NS3	1785	1799	HLA-DRB1*04:07	0%	Dengue virus 1	ELISPOT	IFNg release	Positive	0	0	0	[illegible]
868113	MIIMDEAHFTDPASI	NS3	1756	1770	HLA-DQA1*01:01/DQB1*05:01	0%	Dengue virus 1	ELISPOT	IFNg release	Positive	0	0	0	[illegible]
95169	YRILQRGLLGRSQ	NS3	23	35	HLA-DR1	0%	Dengue virus 1	ELISPOT	IFNg release	Positive	0	0	0	[illegible]
195596	KTSIGLLCVTASSAL	NS4a	2174	2188	HLA-DRB1*07:01	0%	Dengue virus 1	ELISPOT	IFNg release	Positive	0	0	0	[illegible]
195680	LMLLALTAVLTGGVT	NS4a	2149	2163	HLA-DRB1*01:01	0%	Dengue virus 1	ELISPOT	IFNg release	Positive	0	0	0	[illegible]
196046	TIETLMLLALIAALT	NS4a	2145	2159	HLA-DRB1*01:01	0%	Dengue virus 1	ELISPOT	IFNg release	Positive	0	0	0	[illegible]
539383	HLTLRAQNALDNLVM	NS4a	2110	2124	HLA-DRB3*02:02	0%	Dengue virus 1	ELISPOT	IFNg release	Positive	0	0	0	[illegible]
738677	DLILEIGKLPQHLTL	NS4a	2099	2113	HLA-DRB1*11:04	0%	Dengue virus 1	ELISPOT	IFNg release	Positive	0	0	0	[illegible]
867567	LCVMASSALLWMANV	NS4a	2180	2194	HLA-DQA1*01:02/DQB1*06:02	0%	Dengue virus 1	ELISPOT	IFNg release	Positive	0	0	0	[illegible]
195308	FMLVAHYAIIGPGLQ	NS4b	2357	2371	HLA-DRB1*07:01	0%	Dengue virus 1	ELISPOT	IFNg release	Positive	0	0	0	[illegible]
195441	HATMLDVDLRPASAW	NS4b	2269	2283	HLA-DRB1*03:01	0%	Dengue virus 1	ELISPOT	IFNg release	Positive	0	0	0	[illegible]
195455	HHHATMLDVDLRPAS	NS4b	2267	2281	HLA-DRB1*03:01	0%	Dengue virus 1	ELISPOT	IFNg release	Positive	0	0	0	[illegible]
738686	DLRPASAWTLYAVAT	NS4b	2276	2290	HLA-DRB1*01:02	0%	Dengue virus 1	ELISPOT	IFNg release	Positive	0	0	0	[illegible]
740732	WTLYAVATTVITPMM	NS4b	2283	2297	HLA-DRB1*04:07	0%	Dengue virus 1	ELISPOT	IFNg release	Positive	0	0	0	[illegible]
868336	PLTLTAAVLMLAAHY	NS4b	2349	2363	HLA-DQA1*01:02/DQB1*06:02	0%	Dengue virus 1	ELISPOT	IFNg release	Positive	0	0	0	[illegible]
195655	LLATSIFKLTYQNKV	NS5	3055	3069	HLA-DRB1*07:01	0%	Dengue virus 1	ELISPOT	IFNg release	Positive	0	0	0	[illegible]
195781	NEDHWFSRGNSLSGV	NS5	2985	2999	HLA-DRB1*04:01	0%	Dengue virus 1	ELISPOT	IFNg release	Positive	0	0	0	[illegible]
196242	YQNKVVRVQRPAKSG	NS5	3065	3079	HLA-DRB1*08:02	0%	Dengue virus 1	ELISPOT	IFNg release	Positive	0	0	0	[illegible]
539096	ATSIFKLTYQNKVVR	NS5	3057	3071	HLA-DRB1*14:04	0%	Dengue virus 1	ELISPOT	IFNg release	Positive	0	0	0	[illegible]
539700	MVNGVVRLLTKPWDV	NS5	103	117	HLA-DRB1*12:02	0%	Dengue virus 1	ELISPOT	IFNg release	Positive	0	0	0	[illegible]
540001	TSIFKLTYQNKVVRV	NS5	3058	3072	HLA-DRB1*09:01	0%	Dengue virus 1	ELISPOT	IFNg release	Positive	0	0	0	[illegible]
540020	TYGLNTFTNMEVQLI	NS5	3098	3112	HLA-DRB1*04:03	0%	Dengue virus 1	ELISPOT	IFNg release	Positive	0	0	0	[illegible]
540083	VRVQRPAKSGTVMDV	NS5	96	110	HLA-DRB1*13:01	0%	Dengue virus 1	ELISPOT	IFNg release	Positive	0	0	0	[illegible]
740668	VQLIRQMESEGIFLP	NS5	3109	3123	HLA-DRB1*11:04	0%	Dengue virus 1	ELISPOT	IFNg release	Positive	0	0	0	[illegible]
868296	PEHALLATSIFKLTY	NS5	3051	3065	HLA-DPB1*02:01	0%	Dengue virus 1	ELISPOT	IFNg release	Positive	0	0	0	[illegible]
195145	AKFKCVTKLEGKIVQ	E	117	131	HLA-DRB1*11:01	100%	Dengue virus 1	ELISPOT	IFNg release	Positive	1	1	1	[illegible]
195260	ENLKYSVIVTVHTGD	E	128	142	HLA-DRB1*07:01	100%	Dengue virus 1	ELISPOT	IFNg release	Positive	1	1	1	[illegible]
195361	GGVFTSVGKLVHQIF	E	426	440	HLA-DRB1*11:01	100%	Dengue virus 1	ELISPOT	IFNg release	Positive	1	1	1	[illegible]
195547	IVQYENLKYSVIVTV	E	124	138	HLA-DRB1*15:02	100%	Dengue virus 1	ELISPOT	IFNg release	Positive	1	1	1	[illegible]
195814	NRQDLLVTFKTAHAK	E	227	241	HLA-DRB1*04:01	100%	Dengue virus 1	ELISPOT	IFNg release	Positive	1	1	1	[illegible]
195874	QYENLKYSVIVTVHT	E	126	140	HLA-DRB1*07:01	100%	Dengue virus 1	ELISPOT	IFNg release	Positive	1	1	1	[illegible]
196053	TLDIELLKTEVTNPA	E	35	49	HLA-DRB1*04:01	100%	Dengue virus 1	ELISPOT	IFNg release	Positive	1	1	1	[illegible]
539169	DLLVTFKTAHAKKQE	E	230	244	HLA-DRB1*14:04	100%	Dengue virus 1	ELISPOT	IFNg release	Positive	1	1	1	[illegible]
539378	HAKKQEVVVLGSQEGAMH	E	239	256	HLA class II	100%	Dengue virus 1	ICS	IFNg release	Positive	1	1	1	[illegible]
539593	LLVTFKTAHAKKQEV	E	236	250	HLA-DRB1*14:02	100%	Dengue virus 1	ELISPOT	IFNg release	Positive	1	1	1	[illegible]
539800	QDLLVTFKTAHAKKQ	E	229	243	HLA-DRB1*09:01	100%	Dengue virus 1	ELISPOT	IFNg release	Positive	1	1	1	[illegible]
539869	RQDLLVTFKTAHAKK	E	228	242	HLA-DRB1*08:03	100%	Dengue virus 1	ELISPOT	IFNg release	Positive	1	1	1	[illegible]
540052	VLGSQEGAMHTALTGA	E	247	262	HLA class II	100%	Dengue virus 1	ICS	IFNg release	Positive	1	1	1	[illegible]
540104	VVVLGSQEGAMHTALTG	E	245	261	HLA class II	100%	Dengue virus 1	ICS	IFNg release	Positive	1	1	1	[illegible]
868013	LVHKQWFLDLPLPWT	E	487	501	HLA-DQA1*01:01/DQB1*05:01	100%	Dengue virus 1	ELISPOT	IFNg release	Positive	1	1	1	[illegible]
869691	WLVHKQWFLDLPLPW	E	486	500	HLA-DQA1*01:01/DQB1*05:01	100%	Dengue virus 1	ELISPOT	IFNg release	Positive	1	1	1	[illegible]
95154	SSIGKMFEATARG	E	396	408	HLA-DQ8	100%	Dengue virus 1	ELISPOT	IFNg release	Positive	1	1	1	[illegible]
132583	QYKFQADSPKRLSAA	NS1	31	45	HLA-DRB1*03:01	100%	Dengue virus 1	ELISPOT	IFNg release	Positive	1	1	1	[illegible]
195579	KNETWKLARASFIEV	NS1	206	220	HLA-DRB1*07:01	100%	Dengue virus 1	ELISPOT	IFNg release	Positive	1	1	1	[illegible]
195784	NETWKLARASFIEVK	NS1	207	221	HLA-DRB1*07:01	100%	Dengue virus 1	ELISPOT	IFNg release	Positive	1	1	1	[illegible]
866284	DDQRAWNIWEVEDYG	NS1	181	195	HLA-DQA1*01:01/DQB1*05:01	100%	Dengue virus 1	ELISPOT	IFNg release	Positive	1	1	1	[illegible]
867041	HADMGYWIESEKNET	NS1	231	245	HLA-DQA1*01:01/DQB1*05:01	100%	Dengue virus 1	ELISPOT	IFNg release	Positive	1	1	1	[illegible]
868574	QRAWNIWEVEDYGFG	NS1	183	197	HLA-DQA1*01:01/DQB1*05:01	100%	Dengue virus 1	ELISPOT	IFNg release	Positive	1	1	1	[illegible]
869135	TEQYKFQADSPKRLS	NS1	65	79	HLA-DRB5*01:01	100%	Dengue virus 1	ELISPOT	IFNg release	Positive	1	1	1	[illegible]
195391	GLLFRRLTSREVLLL	NS2a	1217	1231	HLA-DRB1*08:03	100%	Dengue virus 1	ELISPOT	IFNg release	Positive	1	1	1	[illegible]
195508	ILVEVQDDGTMKIKD	NS2b	68	82	HLA-DRB1*03:01	100%	Dengue virus 1	ELISPOT	IFNg release	Positive	1	1	1	[illegible]
195715	LSSLLKNDVPLAGPL	NS2b	1363	1377	HLA-DRB1*03:01	100%	Dengue virus 1	ELISPOT	IFNg release	Positive	1	1	1	[illegible]
196111	VEVQDDGTMKIKDEE	NS2b	1421	1435	HLA-DRB1*03:01	100%	Dengue virus 1	ELISPOT	IFNg release	Positive	1	1	1	[illegible]
867544	LAGPLIAGGMLIACY	NS2b	1373	1387	HLA-DQA1*01:02/DQB1*06:02	100%	Dengue virus 1	ELISPOT	IFNg release	Positive	1	1	1	[illegible]
1392180	GKEIVDLMCHATFTM	NS3	1730	1744		100%	Dengue virus 1	biological activity	activation	Positive	1	1	1	[illegible]
195165	ANFRADRVIDPRRCL	NS3	1891	1905	HLA-DRB1*03:01	100%	Dengue virus 1	ELISPOT	IFNg release	Positive	1	1	1	[illegible]
195201	CHATFTMRLLSPVRV	NS3	1737	1751	HLA-DRB1*01:01	100%	Dengue virus 1	ELISPOT	IFNg release	Positive	1	1	1	[illegible]
195242	EGKIVGLYGNGVVTT	NS3	1618	1632	HLA-DRB1*15:01	100%	Dengue virus 1	ELISPOT	IFNg release	Positive	1	1	1	[illegible]
195379	GKIVGLYGNGVVTTS	NS3	1619	1633	HLA-DRB1*15:01	100%	Dengue virus 1	ELISPOT	IFNg release	Positive	1	1	1	[illegible]
195622	LDDGIYRIMQRGLLG	NS3	1493	1507	HLA-DRB1*11:01	100%	Dengue virus 1	ELISPOT	IFNg release	Positive	1	1	1	[illegible]
195914	RTLILAPTRVVASEM	NS3	1693	1707	HLA-DRB1*10:01	100%	Dengue virus 1	ELISPOT	IFNg release	Positive	1	1	1	[illegible]
30397	KEGERKKLRPRWLDA	NS3	585	599	HLA class II	100%	Dengue virus 1	ICS	IFNg release	Positive	1	1	1	[illegible]
45669	NREGKIVGLYGNGVV	NS3	141	155	HLA-DRB1*15:01	100%	Dengue virus 1	ICS	IFNg release	Positive	1	1	1	[illegible]
49768	PTRVVASEMAEALKG	NS3	224	238	HLA-DRB1*15:01	100%	Dengue virus 1	ICS	TNF release	Positive	1	1	1	[illegible]
539171	DLMCHATFTMRLLSP	NS3	1734	1748	HLA-DRB1*13:01	100%	Dengue virus 1	ELISPOT	IFNg release	Positive	1	1	1	[illegible]
539288	GEAAAIFMTATPPGS	NS3	1783	1797	HLA-DRB1*04:03	100%	Dengue virus 1	ELISPOT	IFNg release	Positive	1	1	1	[illegible]
539902	SGTYVSAIAQAKASQ	NS3	1633	1647	HLA-DRB1*10:01	100%	Dengue virus 1	ELISPOT	IFNg release	Positive	1	1	1	[illegible]
539986	TLILAPTRVVASEMA	NS3	1694	1708	HLA-DRB1*13:01	100%	Dengue virus 1	ELISPOT	IFNg release	Positive	1	1	1	[illegible]
540040	VGLYGNGVVTTSGTY	NS3	1622	1636	HLA-DRB1*15:06	100%	Dengue virus 1	ELISPOT	IFNg release	Positive	1	1	1	[illegible]
739269	IVGLYGNGVVTTSGT	NS3	132	146	HLA-DRB1*01:02	100%	Dengue virus 1	ELISPOT	IFNg release	Positive	1	1	1	[illegible]
867025	GVFQENVFHTMWHVT	NS3	1514	1528	HLA-DPB1*04:01	100%	Dengue virus 1	ELISPOT	IFNg release	Positive	1	1	1	[illegible]
869341	TTSGTYVSAIAQAKA	NS3	1631	1645	HLA-DQA1*01:02/DQB1*06:02	100%	Dengue virus 1	ELISPOT	IFNg release	Positive	1	1	1	[illegible]
195478	IETLMLLALIAVLTG	NS4a	2146	2160	HLA-DRB1*15:01	100%	Dengue virus 1	ELISPOT	IFNg release	Positive	1	1	1	[illegible]
196214	WIAASIILEFFLMVL	NS4a	2198	2212	HLA-DRB1*15:01	100%	Dengue virus 1	ELISPOT	IFNg release	Positive	1	1	1	[illegible]
867109	HWIAASIILEFFLMV	NS4a	2197	2211	HLA-DPB1*04:01	100%	Dengue virus 1	ELISPOT	IFNg release	Positive	1	1	1	[illegible]
867112	IAASIILEFFLMVLL	NS4a	2199	2213	HLA-DQA1*01:01/DQB1*05:01	100%	Dengue virus 1	ELISPOT	IFNg release	Positive	1	1	1	[illegible]
195114	AGAGLAFSLMKSLGG	NS4b	2476	2490	HLA-DRB1*04:01	100%	Dengue virus 1	ELISPOT	IFNg release	Positive	1	1	1	[illegible]
195476	IDLDPVVYDAKFEKQ	NS4b	2398	2412	HLA-DRB1*03:01	100%	Dengue virus 1	ELISPOT	IFNg release	Positive	1	1	1	[illegible]
195620	LCTSQILLMRTTWAL	NS4b	2421	2435	HLA-DRB1*04:01	100%	Dengue virus 1	ELISPOT	IFNg release	Positive	1	1	1	[illegible]
195637	LGQIMLLILCTSQIL	NS4b	2413	2427	HLA-DRB1*08:02	100%	Dengue virus 1	ELISPOT	IFNg release	Positive	1	1	1	[illegible]
195760	MLLILCTSQILLMRT	NS4b	2417	2431	HLA-DRB1*07:01	100%	Dengue virus 1	ELISPOT	IFNg release	Positive	1	1	1	[illegible]
195848	QIMLLILCTSQILLM	NS4b	2415	2429	HLA-DRB1*07:01	100%	Dengue virus 1	ELISPOT	IFNg release	Positive	1	1	1	[illegible]
539322	GLAFSLMKSLGGGRR	NS4b	2479	2493	HLA-DRB1*10:01	100%	Dengue virus 1	ELISPOT	IFNg release	Positive	1	1	1	[illegible]
539725	NISLTAIANQAAILM	NS4b	2307	2321	HLA-DRB1*04:03	100%	Dengue virus 1	ELISPOT	IFNg release	Positive	1	1	1	[illegible]
738560	AWTLYAVATTIITPM	NS4b	2282	2296	HLA-DRB1*04:07	100%	Dengue virus 1	ELISPOT	IFNg release	Positive	1	1	1	[illegible]
866002	AIANQAAILMGLDKG	NS4b	2312	2326	HLA-DQA1*01:02/DQB1*06:02	100%	Dengue virus 1	ELISPOT	IFNg release	Positive	1	1	1	[illegible]
1066544	ACLGKSYAQMWQLMY	NS5	3244	3258	HLA-DRB1*04:01	100%	Dengue virus 1	ELISPOT	IFNg release	Positive-Low	1	1	1	[illegible]
195168	AQMWQLMYFHRRDLR	NS5	3251	3265	HLA-DRB1*13:01	100%	Dengue virus 1	ELISPOT	IFNg release	Positive	1	1	1	[illegible]
195208	CVYNMMGKREKKLGE	NS5	2942	2956	HLA-DRB1*11:01	100%	Dengue virus 1	ELISPOT	IFNg release	Positive	1	1	1	[illegible]
195360	GGMLVRNPLSRNSTH	NS5	2694	2708	HLA-DRB1*08:02	100%	Dengue virus 1	ELISPOT	IFNg release	Positive	1	1	1	[illegible]
195852	QLIMKDGREIVVPCR	NS5	3207	3221	HLA-DRB1*03:01	100%	Dengue virus 1	ELISPOT	IFNg release	Positive	1	1	1	[illegible]
196031	TCVYNMMGKREKKLG	NS5	2941	2955	HLA-DRB1*11:01	100%	Dengue virus 1	ELISPOT	IFNg release	Positive	1	1	1	[illegible]
196241	YQNKVVRVQRPAKNG	NS5	3065	3079	HLA-DRB1*08:02	100%	Dengue virus 1	ELISPOT	IFNg release	Positive	1	1	1	[illegible]
539350	GTAKLRWFVERNLVK	NS5	2551	2565	HLA-DRB1*09:01	100%	Dengue virus 1	ELISPOT	IFNg release	Positive	1	1	1	[illegible]
539905	SHHFHQLIMKDGREI	NS5	3202	3216	HLA-DRB1*13:01	100%	Dengue virus 1	ELISPOT	IFNg release	Positive	1	1	1	[illegible]
540138	WQLMYFHRRDLRLAA	NS5	3254	3268	HLA-DRB1*14:04	100%	Dengue virus 1	ELISPOT	IFNg release	Positive	1	1	1	[illegible]
740024	QNKVVRVQRPAKNGT	NS5	3066	3080	HLA-DRB1*14:06	100%	Dengue virus 1	ELISPOT	IFNg release	Positive	1	1	1	[illegible]
867505	KSYAQMWQLMYFHRR	NS5	3248	3262	HLA-DPB1*01:01	100%	Dengue virus 1	ELISPOT	IFNg release	Positive	1	1	1	[illegible]
868240	NKVVRVQRPAKNGTV	NS5	3067	3081	HLA-DRB4*01:01	100%	Dengue virus 1	ELISPOT	IFNg release	Positive	1	1	1	[illegible]
868641	RFLEFEALGFMNEDH	NS5	2974	2988	HLA-DPB1*04:01	100%	Dengue virus 1	ELISPOT	IFNg release	Positive	1	1	1	[illegible]
869091	TAKLRWFVERNLVKP	NS5	2552	2566	HLA-DQA1*01:01/DQB1*05:01	100%	Dengue virus 1	ELISPOT	IFNg release	Positive	1	1	1	[illegible]
1067569	LARWGSFKKNGAIKV	C	66	80	HLA-DRB1*07:01	81% - <100%	Dengue virus 1	ELISPOT	IFNg release	Positive-Low	1	1	1	[illegible]
195113	AFLRFLAIPPTAGIL	C	30	44	HLA-DRB1*01:01	81% - <100%	Dengue virus 1	ELISPOT	IFNg release	Positive	1	1	1	[illegible]
195299	FIAFLRFLAIPPTAG	C	28	42	HLA-DRB1*01:01	81% - <100%	Dengue virus 1	ELISPOT	IFNg release	Positive	1	1	1	[illegible]
195315	FNMLKRARNRVSTVS	C	13	27	HLA-DRB1*08:02	81% - <100%	Dengue virus 1	ELISPOT	IFNg release	Positive	1	1	1	[illegible]
195468	IAFLRFLAIPPTAGI	C	29	43	HLA-DRB1*01:01	81% - <100%	Dengue virus 1	ELISPOT	IFNg release	Positive	1	1	1	[illegible]
195498	IKVLRGFKKEISNML	C	78	92	HLA-DRB1*04:01	81% - <100%	Dengue virus 1	ELISPOT	IFNg release	Positive	1	1	1	[illegible]
195883	RGFKKEISNMLNIMN	C	82	96	HLA-DRB1*04:01	81% - <100%	Dengue virus 1	ELISPOT	IFNg release	Positive	1	1	1	[illegible]
195904	RPSFNMLKRARNRVS	C	10	24	HLA-DRB1*11:01	81% - <100%	Dengue virus 1	ELISPOT	IFNg release	Positive	1	1	1	[illegible]
196146	VLRGFKKEISNMLNI	C	80	94	HLA-DRB1*04:01	81% - <100%	Dengue virus 1	ELISPOT	IFNg release	Positive	1	1	1	[illegible]
196151	VMAFIAFLRFLAIPP	C	25	39	HLA-DRB1*11:01	81% - <100%	Dengue virus 1	ELISPOT	IFNg release	Positive	1	1	1	[illegible]
39096	LRGFKKEISNML	C	81	92	HLA-DPw4	81% - <100%	Dengue virus 1	ICS	TNF release	Positive	1	1	1	[illegible]
539043	AFIAFLRFLAIPPTA	C	27	41	HLA-DRB1*12:02	81% - <100%	Dengue virus 1	ELISPOT	IFNg release	Positive	1	1	1	[illegible]
539257	FKKEISNMLNIMNRR	C	84	98	HLA-DRB1*04:03	81% - <100%	Dengue virus 1	ELISPOT	IFNg release	Positive	1	1	1	[illegible]
539259	FKKNGAIKVLRGFKK	C	50	64	HLA-DRB1*08:03	81% - <100%	Dengue virus 1	ELISPOT	IFNg release	Positive	1	1	1	[illegible]
539278	GAIKVLRGFKKEISN	C	76	90	HLA-DRB1*12:02	81% - <100%	Dengue virus 1	ELISPOT	IFNg release	Positive	1	1	1	[illegible]
539495	KNGAIKVLRGFKKEI	C	52	66	HLA-DRB1*12:02	81% - <100%	Dengue virus 1	ELISPOT	IFNg release	Positive	1	1	1	[illegible]
539613	LRFLAIPPTAGILAR	C	32	46	HLA-DRB1*04:03	81% - <100%	Dengue virus 1	ELISPOT	IFNg release	Positive	1	1	1	[illegible]
539616	LRGFKKEISNMLNIM	C	81	95	HLA-DRB1*10:01	81% - <100%	Dengue virus 1	ELISPOT	IFNg release	Positive	1	1	1	[illegible]
539711	NGAIKVLRGFKKEIS	C	53	67	HLA-DRB1*13:01	81% - <100%	Dengue virus 1	ELISPOT	IFNg release	Positive	1	1	1	[illegible]
539780	PMKLVMAFIAFLRFL	C	21	35	HLA-DRB1*13:01	81% - <100%	Dengue virus 1	ELISPOT	IFNg release	Positive	1	1	1	[illegible]
539785	PSFNMLKRARNRVST	C	11	25	HLA-DRB1*10:01	81% - <100%	Dengue virus 1	ELISPOT	IFNg release	Positive	1	1	1	[illegible]
540117	WGSFKKNGAIKVLRG	C	47	61	HLA-DRB1*09:01	81% - <100%	Dengue virus 1	ELISPOT	IFNg release	Positive	1	1	1	[illegible]
738927	FLRFLAIPPTAGILA	C	41	55	HLA-DRB1*14:02	81% - <100%	Dengue virus 1	ELISPOT	IFNg release	Positive	1	1	1	[illegible]
867523	KVLRGFKKEISNMLN	C	79	93	HLA-DRB3*02:02	81% - <100%	Dengue virus 1	ELISPOT	IFNg release	Positive	1	1	1	[illegible]
95107	KLVMAFIAFLRFL	C	45	57	HLA-DPw4	81% - <100%	Dengue virus 1	ELISPOT	IFNg release	Positive	1	1	1	[illegible]
195368	GIIFILLMLVTPSMA	PreM	266	280	HLA-DRB1*01:01	81% - <100%	Dengue virus 1	ELISPOT	IFNg release	Positive	1	1	1	[illegible]
195562	KGIIFILLMLVTPSM	PreM	265	279	HLA-DRB1*08:02	81% - <100%	Dengue virus 1	ELISPOT	IFNg release	Positive	1	1	1	[illegible]
195831	PGFTVIALFLAHAIG	PreM	245	259	HLA-DRB1*15:01	81% - <100%	Dengue virus 1	ELISPOT	IFNg release	Positive	1	1	1	[illegible]
195887	RGKSLLFKTSAGVNM	PreM	111	125	HLA-DRB1*07:01	81% - <100%	Dengue virus 1	ELISPOT	IFNg release	Positive	1	1	1	[illegible]
196085	TVIALFLAHAIGTSI	PreM	248	262	HLA-DRB1*07:01	81% - <100%	Dengue virus 1	ELISPOT	IFNg release	Positive	1	1	1	[illegible]
539516	KSLLFKTSAGVNMCT	PreM	123	137	HLA-DRB1*09:01	81% - <100%	Dengue virus 1	ELISPOT	IFNg release	Positive	1	1	1	[illegible]
867083	HPGFTVIALFLAHAI	PreM	244	258	HLA-DPB1*02:01	81% - <100%	Dengue virus 1	ELISPOT	IFNg release	Positive	1	1	1	[illegible]
867912	LRHPGFTVIALFLAH	PreM	242	256	HLA-DPB1*02:01	81% - <100%	Dengue virus 1	ELISPOT	IFNg release	Positive	1	1	1	[illegible]
195216	DIELLKTEVTNPAVL	E	37	51	HLA-DRB1*04:01	81% - <100%	Dengue virus 1	ELISPOT	IFNg release	Positive	1	1	1	[illegible]
195477	IELLKTEVTNPAVLR	E	38	52	HLA-DRB1*04:01	81% - <100%	Dengue virus 1	ELISPOT	IFNg release	Positive	1	1	1	[illegible]
195623	LDIELLKTEVTNPAV	E	36	50	HLA-DRB1*04:01	81% - <100%	Dengue virus 1	ELISPOT	IFNg release	Positive	1	1	1	[illegible]
195646	LITANPIVTDKEKPV	E	346	360	HLA-DRB1*03:01	81% - <100%	Dengue virus 1	ELISPOT	IFNg release	Positive	1	1	1	[illegible]
867101	HTALTGATEIQTSGT	E	541	555	HLA-DQA1*01:02/DQB1*06:02	81% - <100%	Dengue virus 1	ELISPOT	IFNg release	Positive	1	1	1	[illegible]
867378	KCRLKMDKLTLKGMS	E	564	578	HLA-DRB3*02:02	81% - <100%	Dengue virus 1	ELISPOT	IFNg release	Positive	1	1	1	[illegible]
195685	LNHILLENDMKFTVV	NS1	75	89	HLA-DRB1*03:01	81% - <100%	Dengue virus 1	ELISPOT	IFNg release	Positive	1	1	1	[illegible]
539401	IESEKNETWKLARASFI	NS1	202	218	HLA class II	81% - <100%	Dengue virus 1	ICS	IFNg release	Positive	1	1	1	[illegible]
866693	FGIFTTNIWLKLRDS	NS1	196	210	HLA-DPB1*04:01	81% - <100%	Dengue virus 1	ELISPOT	IFNg release	Positive	1	1	1	[illegible]
869721	YGFGIFTTNIWLKLR	NS1	194	208	HLA-DPB1*02:01	81% - <100%	Dengue virus 1	ELISPOT	IFNg release	Positive	1	1	1	[illegible]
167816	SLVASVELPNSLEELGDGLAMGIMI	NS2a	109	133	HLA class II	81% - <100%	Dengue virus 1	ELISPOT	IFNg release	Positive	1	1	1	[illegible]
195162	AMVLSIVSLFPLCLS	NS2a	1296	1310	HLA-DRB1*15:01	81% - <100%	Dengue virus 1	ELISPOT	IFNg release	Positive	1	1	1	[illegible]
195286	FAVGLLFRRLTSREV	NS2a	1214	1228	HLA-DRB1*08:03	81% - <100%	Dengue virus 1	ELISPOT	IFNg release	Positive	1	1	1	[illegible]
195595	KTMAMVLSIVSLFPL	NS2a	1293	1307	HLA-DRB1*03:01	81% - <100%	Dengue virus 1	ELISPOT	IFNg release	Positive	1	1	1	[illegible]
195665	LLLTIGLSLVASVEL	NS2a	1229	1243	HLA-DRB1*07:01	81% - <100%	Dengue virus 1	ELISPOT	IFNg release	Positive	1	1	1	[illegible]
195674	LLTIGLSLVASVELP	NS2a	1230	1244	HLA-DRB1*07:01	81% - <100%	Dengue virus 1	ELISPOT	IFNg release	Positive	1	1	1	[illegible]
195708	LSIVSLFPLCLSTTS	NS2a	1299	1313	HLA-DRB1*01:01	81% - <100%	Dengue virus 1	ELISPOT	IFNg release	Positive	1	1	1	[illegible]
195745	MAMVLSIVSLFPLCL	NS2a	1295	1309	HLA-DRB1*07:01	81% - <100%	Dengue virus 1	ELISPOT	IFNg release	Positive	1	1	1	[illegible]
195983	SREVLLLTIGLSLVA	NS2a	1225	1239	HLA-DRB1*15:01	81% - <100%	Dengue virus 1	ELISPOT	IFNg release	Positive	1	1	1	[illegible]
196216	WKTMAMVLSIVSLFP	NS2a	1292	1306	HLA-DRB1*08:02	81% - <100%	Dengue virus 1	ELISPOT	IFNg release	Positive	1	1	1	[illegible]
539086	AMGIMILKLLTDFQS	NS2a	1255	1269	HLA-DRB1*04:03	81% - <100%	Dengue virus 1	ELISPOT	IFNg release	Positive	1	1	1	[illegible]
539094	ATFKMRPMFAVGLLF	NS2a	1206	1220	HLA-DRB1*01:02	81% - <100%	Dengue virus 1	ELISPOT	IFNg release	Positive	1	1	1	[illegible]
539205	EEVMRSRWSRKMLMT	NS2a	1147	1161	HLA-DRB1*13:01	81% - <100%	Dengue virus 1	ELISPOT	IFNg release	Positive	1	1	1	[illegible]
539272	FSLHYAWKTMAMVLS	NS2a	1286	1300	HLA-DRB1*09:01	81% - <100%	Dengue virus 1	ELISPOT	IFNg release	Positive	1	1	1	[illegible]
539398	IEEVMRSRWSRKMLM	NS2a	1146	1160	HLA-DRB1*13:01	81% - <100%	Dengue virus 1	ELISPOT	IFNg release	Positive	1	1	1	[illegible]
539517	KTTFSLHYAWKTMAM	NS2a	1283	1297	HLA-DRB1*09:01	81% - <100%	Dengue virus 1	ELISPOT	IFNg release	Positive	1	1	1	[illegible]
539888	RWSRKMLMTGTLAVF	NS2a	1153	1167	HLA-DRB1*04:03	81% - <100%	Dengue virus 1	ELISPOT	IFNg release	Positive	1	1	1	[illegible]
539913	SLHYAWKTMAMVLSI	NS2a	1287	1301	HLA-DRB1*09:01	81% - <100%	Dengue virus 1	ELISPOT	IFNg release	Positive	1	1	1	[illegible]
539970	TFSLHYAWKTMAMVL	NS2a	1285	1299	HLA-DRB1*14:06	81% - <100%	Dengue virus 1	ELISPOT	IFNg release	Positive	1	1	1	[illegible]
540038	VGLLFRRLTSREVLL	NS2a	1216	1230	HLA-DRB1*08:03	81% - <100%	Dengue virus 1	ELISPOT	IFNg release	Positive	1	1	1	[illegible]
540155	YLALMATFKMRPMFA	NS2a	1201	1215	HLA-DRB1*13:01	81% - <100%	Dengue virus 1	ELISPOT	IFNg release	Positive	1	1	1	[illegible]
738553	AVGLLFRRLTSREVL	NS2a	1215	1229	HLA-DRB1*01:02	81% - <100%	Dengue virus 1	ELISPOT	IFNg release	Positive	1	1	1	[illegible]
738679	DLIRLCIMVGANASD	NS2a	1179	1193	HLA-DRB1*11:04	81% - <100%	Dengue virus 1	ELISPOT	IFNg release	Positive	1	1	1	[illegible]
739508	LLCISIMIEEVMRSR	NS2a	1139	1153	HLA-DRB1*11:04	81% - <100%	Dengue virus 1	ELISPOT	IFNg release	Positive	1	1	1	[illegible]
867232	ILKLLTDFQSHQLWA	NS2a	1260	1274	HLA-DPB1*01:01	81% - <100%	Dengue virus 1	ELISPOT	IFNg release	Positive	1	1	1	[illegible]
868098	MGMGTTYLALMATFK	NS2a	456	470	HLA-DRB5*01:01	81% - <100%	Dengue virus 1	ELISPOT	IFNg release	Positive	1	1	1	[illegible]
868100	MGQLTWNDLIRLCIM	NS2a	433	447	HLA-DPB1*04:02	81% - <100%	Dengue virus 1	ELISPOT	IFNg release	Positive	1	1	1	[illegible]
195191	AVGIVSILLSSLLKN	NS2b	1355	1369	HLA-DRB1*08:02	81% - <100%	Dengue virus 1	ELISPOT	IFNg release	Positive	1	0	1	[illegible]
195228	DTLTILLKATLLAVS	NS2b	1438	1452	HLA-DRB1*08:02	81% - <100%	Dengue virus 1	ELISPOT	IFNg release	Positive	1	1	1	[illegible]
195510	IMAVGIVSILLSSLL	NS2b	1353	1367	HLA-DRB1*07:01	81% - <100%	Dengue virus 1	ELISPOT	IFNg release	Positive	1	0	1	[illegible]
195724	LTILLKATLLAVSGV	NS2b	1440	1454	HLA-DRB1*08:02	81% - <100%	Dengue virus 1	ELISPOT	IFNg release	Positive	1	1	1	[illegible]
195878	RDDTLTILLKATLLA	NS2b	1436	1450	HLA-DRB1*12:02	81% - <100%	Dengue virus 1	ELISPOT	IFNg release	Positive	1	1	1	[illegible]
195993	SSLLKNDVPLAGPLI	NS2b	1364	1378	HLA-DRB1*03:01	81% - <100%	Dengue virus 1	ELISPOT	IFNg release	Positive	1	1	1	[illegible]
866201	ATLFVWYFWQKKKQR	NS2b	1461	1475	HLA-DPB1*01:01	81% - <100%	Dengue virus 1	ELISPOT	IFNg release	Positive	1	1	1	[illegible]
867359	IVSILLSSLLKNDVP	NS2b	1358	1372	HLA-DPB1*04:02	81% - <100%	Dengue virus 1	ELISPOT	IFNg release	Positive	1	0	1	[illegible]
167826	TSGTYVSAIAQAKASQE	NS3	157	173	HLA class II	81% - <100%	Dengue virus 1	ELISPOT	IFNg release	Positive	1	1	1	[illegible]
195163	ANCLRKNGKRVIQLS	NS3	1848	1862	HLA-DRB1*13:01	81% - <100%	Dengue virus 1	ELISPOT	IFNg release	Positive	1	1	1	[illegible]
195296	FHTMWHVTRGAVLMY	NS3	1521	1535	HLA-DRB1*07:01	81% - <100%	Dengue virus 1	ELISPOT	IFNg release	Positive	1	1	1	[illegible]
195338	FTMRLLSPVRVPNYN	NS3	1741	1755	HLA-DRB1*01:01	81% - <100%	Dengue virus 1	ELISPOT	IFNg release	Positive	1	1	1	[illegible]
195777	NCLRKNGKRVIQLSR	NS3	1849	1863	HLA-DRB1*11:01	81% - <100%	Dengue virus 1	ELISPOT	IFNg release	Positive	1	1	1	[illegible]
195778	NDIANCLRKNGKRVI	NS3	1845	1859	HLA-DRB1*11:01	81% - <100%	Dengue virus 1	ELISPOT	IFNg release	Positive	1	1	1	[illegible]
196130	VILKDGPERVILAGP	NS3	1908	1922	HLA-DRB1*03:01	81% - <100%	Dengue virus 1	ELISPOT	IFNg release	Positive	0	1	1	[illegible]
196197	VTRGAVLMYQGKRLE	NS3	1527	1541	HLA-DRB1*15:01	81% - <100%	Dengue virus 1	ELISPOT	IFNg release	Positive	1	1	1	[illegible]
539022	AAAIFMTATPPGSVE	NS3	1785	1799	HLA-DRB1*04:03	81% - <100%	Dengue virus 1	ELISPOT	IFNg release	Positive	1	1	1	[illegible]
539027	AAIFMTATPPGSVEA	NS3	1786	1800	HLA-DRB1*10:01	81% - <100%	Dengue virus 1	ELISPOT	IFNg release	Positive	1	1	1	[illegible]
539156	DGIYRIMQRGLLGRS	NS3	1495	1509	HLA-DRB1*12:02	81% - <100%	Dengue virus 1	ELISPOT	IFNg release	Positive	1	1	1	[illegible]
539309	GIYRIMQRGLLGRSQ	NS3	1496	1510	HLA-DRB1*14:04	81% - <100%	Dengue virus 1	ELISPOT	IFNg release	Positive	1	1	1	[illegible]
539579	LKGMPIRYQTTAVKS	NS3	1711	1725	HLA-DRB1*04:03	81% - <100%	Dengue virus 1	ELISPOT	IFNg release	Positive	1	1	1	[illegible]
539715	NGKRVIQLSRKTFDT	NS3	1854	1868	HLA-DRB1*12:02	81% - <100%	Dengue virus 1	ELISPOT	IFNg release	Positive	1	1	1	[illegible]
866181	ASQEGPLPEIEDEVF	NS3	1645	1659	HLA-DQA1*05:01/DQB1*02:01	81% - <100%	Dengue virus 1	ELISPOT	IFNg release	Positive	1	1	1	[illegible]
866721	FKTPEGEVGAIALDF	NS3	1591	1605	HLA-DQA1*03:01/DQB1*03:02	81% - <100%	Dengue virus 1	ELISPOT	IFNg release	Positive	1	1	1	[illegible]
867396	KGMPIRYQTTAVKSE	NS3	1712	1726	HLA-DRB3*02:02	81% - <100%	Dengue virus 1	ELISPOT	IFNg release	Positive	1	1	1	[illegible]
868343	PNYNMIIMDEAHFTD	NS3	1752	1766	HLA-DQA1*05:01/DQB1*02:01	81% - <100%	Dengue virus 1	ELISPOT	IFNg release	Positive	1	1	1	[illegible]
869688	WHVTRGAVLMYQGKR	NS3	1525	1539	HLA-DQA1*01:02/DQB1*06:02	81% - <100%	Dengue virus 1	ELISPOT	IFNg release	Positive	1	1	1	[illegible]
869696	WNTGEEVQVIAVEPG	NS3	1564	1578	HLA-DQA1*03:01/DQB1*03:02	81% - <100%	Dengue virus 1	ELISPOT	IFNg release	Positive	1	1	1	[illegible]
195679	LMLLALIAVLTGGVT	NS4a	2149	2163	HLA-DRB1*08:02	81% - <100%	Dengue virus 1	ELISPOT	IFNg release	Positive	1	1	1	[illegible]
196255	YVVIGLLFMILTVAA	NS4a	2230	2244	HLA-DRB1*11:01	81% - <100%	Dengue virus 1	ELISPOT	IFNg release	Positive	1	1	1	[illegible]
539744	NQLAYVVIGLLFMIL	NS4a	2226	2240	HLA-DRB1*15:02	81% - <100%	Dengue virus 1	ELISPOT	IFNg release	Positive	1	1	1	[illegible]
739034	GLGKTSIGLLCVMAS	NS4a	2171	2185	HLA-DRB1*11:04	81% - <100%	Dengue virus 1	ELISPOT	IFNg release	Positive	1	1	1	[illegible]
868132	MLLALIAVLTGGVTL	NS4a	2150	2164	HLA-DPB1*04:02	81% - <100%	Dengue virus 1	ELISPOT	IFNg release	Positive	1	1	1	[illegible]
195193	AVLMLVAHYAIIGPG	NS4b	2355	2369	HLA-DRB1*01:01	81% - <100%	Dengue virus 1	ELISPOT	IFNg release	Positive	1	1	1	[illegible]
195561	KFWNTTIAVSMANIF	NS4b	2456	2470	HLA-DRB1*07:01	81% - <100%	Dengue virus 1	ELISPOT	IFNg release	Positive	1	1	1	[illegible]
195682	LMLVAHYAIIGPGLQ	NS4b	2357	2371	HLA-DRB1*07:01	81% - <100%	Dengue virus 1	ELISPOT	IFNg release	Positive	1	1	1	[illegible]
196143	VLMLVAHYAIIGPGL	NS4b	2356	2370	HLA-DRB1*01:01	81% - <100%	Dengue virus 1	ELISPOT	IFNg release	Positive	1	1	1	[illegible]
195147	AKNGTVMDVISRRDQ	NS5	3076	3090	HLA-DRB1*03:01	81% - <100%	Dengue virus 1	ELISPOT	IFNg release	Positive	1	1	1	[illegible]
195186	ATNIQVAINQVRRLI	NS5	3352	3366	HLA-DRB1*03:01	81% - <100%	Dengue virus 1	ELISPOT	IFNg release	Positive	1	1	1	[illegible]
195212	DDRFATALTALNDMG	NS5	3163	3177	HLA-DRB1*04:01	81% - <100%	Dengue virus 1	ELISPOT	IFNg release	Positive	1	1	1	[illegible]
195223	DMGKVRKDIPQWEPS	NS5	3175	3189	HLA-DRB1*03:01	81% - <100%	Dengue virus 1	ELISPOT	IFNg release	Positive	1	1	1	[illegible]
195236	EEFTRKVRSNAAIGA	NS5	2889	2903	HLA-DRB1*08:02	81% - <100%	Dengue virus 1	ELISPOT	IFNg release	Positive	1	1	1	[illegible]
195354	GEKWKRQLNQLSKSE	NS5	2503	2517	HLA-DRB1*04:01	81% - <100%	Dengue virus 1	ELISPOT	IFNg release	Positive	1	1	1	[illegible]
195385	GLHKLGYILRDISKI	NS5	3003	3017	HLA-DRB1*08:02	81% - <100%	Dengue virus 1	ELISPOT	IFNg release	Positive	1	1	1	[illegible]
195421	GTGNIVSAVNMTSRM	NS5	2716	2730	HLA-DRB1*08:02	81% - <100%	Dengue virus 1	ELISPOT	IFNg release	Positive	0	1	0	[illegible]
195438	GYILRDISKIPGGNM	NS5	3008	3022	HLA-DRB1*08:02	81% - <100%	Dengue virus 1	ELISPOT	IFNg release	Positive	1	1	1	[illegible]
195464	HWFSRENSLSGVEGE	NS5	2988	3002	HLA-DRB1*04:01	81% - <100%	Dengue virus 1	ELISPOT	IFNg release	Positive	1	1	1	[illegible]
195582	KPIDDRFATALTALN	NS5	3160	3174	HLA-DRB1*04:01	81% - <100%	Dengue virus 1	ELISPOT	IFNg release	Positive	1	1	1	[illegible]
195755	MGKVRKDIPQWEPSK	NS5	3176	3190	HLA-DRB1*03:01	81% - <100%	Dengue virus 1	ELISPOT	IFNg release	Positive	1	1	1	[illegible]
195786	NGTVMDVISRRDQRG	NS5	3078	3092	HLA-DRB1*03:01	81% - <100%	Dengue virus 1	ELISPOT	IFNg release	Positive	1	1	1	[illegible]
195903	RPAKNGTVMDVISRR	NS5	3074	3088	HLA-DRB1*03:01	81% - <100%	Dengue virus 1	ELISPOT	IFNg release	Positive	1	1	1	[illegible]
196039	TGNIVSAVNMTSRML	NS5	2717	2731	HLA-DRB1*04:01	81% - <100%	Dengue virus 1	ELISPOT	IFNg release	Positive	0	1	0	[illegible]
196211	WATNIQVAINQVRRL	NS5	3351	3365	HLA-DRB1*03:01	81% - <100%	Dengue virus 1	ELISPOT	IFNg release	Positive	1	1	1	[illegible]
539107	AVNMTSRMLLNRFTM	NS5	2723	2737	HLA-DRB1*15:02	81% - <100%	Dengue virus 1	ELISPOT	IFNg release	Positive	0	1	0	[illegible]
539396	IDDRFATALTALNDM	NS5	3162	3176	HLA-DPB1*01:01	81% - <100%	Dengue virus 1	ELISPOT	IFNg release	Positive	1	1	1	[illegible]
539435	ISGDDCVVKPIDDRFAT	NS5	3152	3168	HLA class II	81% - <100%	Dengue virus 1	ICS	IFNg release	Positive	1	1	1	[illegible]
539691	MTSRMLLNRFTMAHR	NS5	17	31	HLA-DRB1*14:06	81% - <100%	Dengue virus 1	ELISPOT	IFNg release	Positive	1	0	1	[illegible]
539702	MVTQIAMTDTTPFGQQR	NS5	2829	2845	HLA class II	81% - <100%	Dengue virus 1	ICS	IFNg release	Positive	1	1	1	[illegible]
539862	RMLLNRFTMAHRKPT	NS5	20	34	HLA-DRB1*14:02	81% - <100%	Dengue virus 1	ELISPOT	IFNg release	Positive	1	0	1	[illegible]
540018	TYGLNTFTNMEAQLI	NS5	3098	3112	HLA-DRB1*15:06	81% - <100%	Dengue virus 1	ELISPOT	IFNg release	Positive	1	1	1	[illegible]
540100	VVKPIDDRFATALTALN	NS5	3157	3173	HLA class II	81% - <100%	Dengue virus 1	ICS	IFNg release	Positive	1	1	1	[illegible]
195535	ISNMLNIMNRRKRSV	C	88	102	HLA-DRB1*08:02	51%-80%	Dengue virus 1	ELISPOT	IFNg release	Positive	1	1	1	[illegible]
195557	KEISNMLNIMNRRKR	C	86	100	HLA-DRB1*13:01	51%-80%	Dengue virus 1	ELISPOT	IFNg release	Positive	1	1	1	[illegible]
195761	MLNIMNRRKRSVTML	C	69	83	HLA-DRB1*08:02	51%-80%	Dengue virus 1	ELISPOT	IFNg release	Positive	1	1	1	[illegible]
195805	NMLNIMNRRKRSVTM	C	90	104	HLA-DRB1*11:01	51%-80%	Dengue virus 1	ELISPOT	IFNg release	Positive	1	1	1	[illegible]
740331	SNMLNIMNRRKRSVT	C	89	103	HLA-DRB1*14:02	51%-80%	Dengue virus 1	ELISPOT	IFNg release	Positive	1	1	1	[illegible]
195323	FQSHQLWATLLSLTF	NS2a	1267	1281	HLA-DPB1*04:02	51%-80%	Dengue virus 1	ELISPOT	IFNg release	Positive	1	0	1	[illegible]
195670	LLSLTFIKTTFSLHY	NS2a	1276	1290	HLA-DRB1*07:01	51%-80%	Dengue virus 1	ELISPOT	IFNg release	Positive	1	0	1	[illegible]
195143	AIVREAIKRKLRTLI	NS3	1682	1696	HLA-DRB1*11:01	51%-80%	Dengue virus 1	ELISPOT	IFNg release	Positive	1	1	1	[illegible]
195585	KRKLRTLILAPTRVV	NS3	1689	1703	HLA-DRB1*10:01	51%-80%	Dengue virus 1	ELISPOT	IFNg release	Positive	1	1	1	[illegible]
195705	LRTLILAPTRVVASE	NS3	1692	1706	HLA-DRB1*08:02	51%-80%	Dengue virus 1	ELISPOT	IFNg release	Positive	1	1	1	[illegible]
195896	RKLRTLILAPTRVVA	NS3	1690	1704	HLA-DRB1*01:01	51%-80%	Dengue virus 1	ELISPOT	IFNg release	Positive	1	1	1	[illegible]
539485	KLRTLILAPTRVVAS	NS3	1691	1705	HLA-DRB1*04:03	51%-80%	Dengue virus 1	ELISPOT	IFNg release	Positive	1	1	1	[illegible]
540074	VREAIKRKLRTLILA	NS3	1684	1698	HLA-DRB1*12:02	51%-80%	Dengue virus 1	ELISPOT	IFNg release	Positive	1	1	1	[illegible]
739540	LPAIVREAIKRKLRT	NS3	1680	1694	HLA-DRB1*14:02	51%-80%	Dengue virus 1	ELISPOT	IFNg release	Positive	1	1	1	[illegible]
866469	EAIKRKLRTLILAPT	NS3	1686	1700	HLA-DPB1*01:01	51%-80%	Dengue virus 1	ELISPOT	IFNg release	Positive	1	1	1	[illegible]
739085	GVTLFFLSGRGLGKT	NS4a	2161	2175	HLA-DRB1*01:02	51%-80%	Dengue virus 1	ELISPOT	IFNg release	Positive	1	1	1	[illegible]
867760	LLCVMASSVLLWMAS	NS4a	2179	2193	HLA-DQA1*01:02/DQB1*06:02	51%-80%	Dengue virus 1	ELISPOT	IFNg release	Positive	1	1	1	[illegible]
869401	VDGIVAIDLDPVVYD	NS4b	2392	2406	HLA-DQA1*01:01/DQB1*05:01	51%-80%	Dengue virus 1	ELISPOT	IFNg release	Positive	1	1	1	[illegible]
195783	NENYLDYMTSMKRFK	NS5	3368	3382	HLA-DRB1*11:01	51%-80%	Dengue virus 1	ELISPOT	IFNg release	Positive	1	1	1	[illegible]
195824	NYLDYMTSMKRFKNE	NS5	3370	3384	HLA-DRB1*14:02	51%-80%	Dengue virus 1	ELISPOT	IFNg release	Positive	1	1	1	[illegible]
195923	RWLWGFLSRNKKPRI	NS5	2871	2885	HLA-DRB1*15:01	51%-80%	Dengue virus 1	ELISPOT	IFNg release	Positive	1	1	1	[illegible]
195956	SIFKLTYQNKVVRVQ	NS5	3059	3073	HLA-DRB1*09:01	51%-80%	Dengue virus 1	ELISPOT	IFNg release	Positive	0	0	0	[illegible]
196191	VTARWLWGFLSRNKK	NS5	2868	2882	HLA-DRB1*15:01	51%-80%	Dengue virus 1	ELISPOT	IFNg release	Positive	1	1	1	[illegible]
196220	WLWGFLSRNKKPRIC	NS5	2872	2886	HLA-DRB1*11:01	51%-80%	Dengue virus 1	ELISPOT	IFNg release	Positive	1	1	1	[illegible]
738814	ENYLDYMTSMKRFKN	NS5	3369	3383	HLA-DRB1*14:02	51%-80%	Dengue virus 1	ELISPOT	IFNg release	Positive	1	1	1	[illegible]
740786	YLDYMTSMKRFKNES	NS5	3371	3385	HLA-DRB1*14:02	51%-80%	Dengue virus 1	ELISPOT	IFNg release	Positive	1	1	1	[illegible]
866481	EAVEDERFWDLVHRE	NS5	2916	2930	HLA-DQA1*01:01/DQB1*05:01	51%-80%	Dengue virus 1	ELISPOT	IFNg release	Positive	1	1	1	[illegible]
195117	AGILARWSSFKKNGA	C	51	65	HLA-DRB1*15:01	>0% - 50%	Dengue virus 1	ELISPOT	IFNg release	Positive	0	0	0	[illegible]
195540	ISSMLNIMNRRKRSV	C	76	90	HLA-DRB1*14:02	>0% - 50%	Dengue virus 1	ELISPOT	IFNg release	Positive	0	0	0	[illegible]
195885	RGFKKEISSMLNIMN	C	60	74	HLA-DRB1*04:01	>0% - 50%	Dengue virus 1	ELISPOT	IFNg release	Positive	0	0	0	[illegible]
539258	FKKEISSMLNIMNRR	C	62	76	HLA-DRB1*10:01	>0% - 50%	Dengue virus 1	ELISPOT	IFNg release	Positive	0	0	0	[illegible]
539459	KEISSMLNIMNRRKR	C	64	78	HLA-DRB1*14:04	>0% - 50%	Dengue virus 1	ELISPOT	IFNg release	Positive	0	0	0	[illegible]
539475	KKEISSMLNIMNRRK	C	63	77	HLA-DRB1*14:04	>0% - 50%	Dengue virus 1	ELISPOT	IFNg release	Positive	0	0	0	[illegible]
539617	LRGFKKEISSMLNIM	C	59	73	HLA-DRB1*13:01	>0% - 50%	Dengue virus 1	ELISPOT	IFNg release	Positive	0	0	0	[illegible]
539866	RPSFNMLKRVRNRVS	C	10	24	HLA-DRB1*08:03	>0% - 50%	Dengue virus 1	ELISPOT	IFNg release	Positive	0	0	0	[illegible]
540059	VLRGFKKEISSMLNI	C	68	82	HLA-DRB1*04:07	>0% - 50%	Dengue virus 1	ELISPOT	IFNg release	Positive	0	0	0	[illegible]
195589	KSLLFKTSVGVNMCT	PreM	135	149	HLA-DRB1*07:01	>0% - 50%	Dengue virus 1	ELISPOT	IFNg release	Positive	0	0	0	[illegible]
869062	SVGVNMCTLIAMDLG	PreM	142	156	HLA-DQA1*01:02/DQB1*06:02	>0% - 50%	Dengue virus 1	ELISPOT	IFNg release	Positive	0	0	0	[illegible]
195422	GTLAVFLLLIMGQLT	NS2a	1162	1176	HLA-DRB1*01:01	>0% - 50%	Dengue virus 1	ELISPOT	IFNg release	Positive	0	0	0	[illegible]
195423	GTLVVFLLLIMGQLT	NS2a	1162	1176	HLA-DRB1*15:01	>0% - 50%	Dengue virus 1	ELISPOT	IFNg release	Positive	0	0	0	[illegible]
195613	LAVFLLLIMGQLTWN	NS2a	1164	1178	HLA-DRB1*15:06	>0% - 50%	Dengue virus 1	ELISPOT	IFNg release	Positive	0	0	0	[illegible]
195614	LAVGLLFRRLTSREV	NS2a	1214	1228	HLA-DRB1*11:01	>0% - 50%	Dengue virus 1	ELISPOT	IFNg release	Positive	0	0	0	[illegible]

195959	SILIEEVMRSRWSRK	NS2a	1143	1157	HLA-DRB1*11:04	>0% - 50%	Dengue virus 1	ELISPOT	IFNg release	Positive	0	0	0	0	0	0	0	0	0	0	0	0	0	0	0	0	0	0	0	0	0	0	0	0	0	0	0	0	0	0	0	0	0	0	0	0	0	0	0	0	0	0	0	0	0	0	0	0	0	0	0	0	0	0	0	0	0	0	0	0	0	0	0	0	0	0	0	0	0	0	0	0	0	0	0	0	0	0	0	0	0	0	0	0	0	0	0	0	0
539624	LSLTFIKTTLSLHYA	NS2a	1277	1291	HLA-DRB1*14:04	>0% - 50%	Dengue virus 1	ELISPOT	IFNg release	Positive	0	0	0	0	0	0	0	0	0	0	0	0	0	0	0	0	0	0	0	0	0	0	0	0	0	0	0	0	0	0	0	0	0	0	0	0	0	0	0	0	0	0	0	0	0	0	0	0	0	0	0	0	0	0	0	0	0	0	0	0	0	0	0	0	0	0	0	0	0	0	0	0	0	0	0	0	0	0	0	0	0	0	0	0	0	0	0	0	0
539679	MLMTGTLAVFLLLIM	NS2a	1158	1172	HLA-DRB1*15:06	>0% - 50%	Dengue virus 1	ELISPOT	IFNg release	Positive	0	0	0	0	0	0	0	0	0	0	0	0	0	0	0	0	0	0	0	1	0	0	1	0	0	1	0	0	0	0	0	0	0	0	0	0	0	0	0	1	0	0	0	0	0	0	1	0	0	0	0	0	1	0	0	0	0	0	0	0	0	0	0	1	0	0	1	0	0	0	0	0	1	0	0	0	0	0	0	0	0	0	0	0	0	0	0	0	0	0	0	0	1	0	0	0	0	0	0
539680	MLMTGTLVVFFLLIM	NS2a	1158	1172	HLA-DRB1*15:06	>0% - 50%	Dengue virus 1	ELISPOT	IFNg release	Positive	0	0	0	0	0	0	0	0	0	0	0	0	0	0	0	0	0	0	0	0	0	0	0	0	0	0	0	0	0	0	0	0	0	0	0	0	0	0	0	0	0	0	0	0	0	0	0	0	0	0	0	0	0	0	0	0	0	1	0	0	0	0	1	0	0	0	0	0	0	0	0	0	0	0	0	0	0	0	0	0	0	0	0	0	1	0	0	0	0	0	0	0	0	0	0	0	0	0	0	0	0	0	0
866767	FQSYQLWATLLSLTF	NS2a	1267	1281	HLA-DPB1*04:02	>0% - 50%	Dengue virus 1	ELISPOT	IFNg release	Positive	0	0	0	0	0	0	0	0	0	0	0	0	0	0	0	0	0	0	0	0	0	0	1	0	0	1	0	0	0	0	0	0	0	0	0	1	0	0	0	0	0	0	0	0	0	0	0	0	0	0	0	0	1	0	0	0	0	0	0	0	0	0	0	1	0	0	1	0	0	0	0	0	1	0	0	0	0	0	0	0	0	0	0	0	1	0	0	0	0	0	0	0	1	0	0	0	0	0	0
866873	GILAVFFLLIMGQLT	NS2a	1162	1176	HLA-DPB1*04:02	>0% - 50%	Dengue virus 1	ELISPOT	IFNg release	Positive	0	0	0	0	0	0	0	0	0	0	0	0	0	0	0	0	0	0	0	0	0	0	0	0	0	0	0	0	0	0	0	0	0	0	0	0	0	0	0	0	0	0	0	0	0	0	0	0	0	0	0	0	0	1	0	0	0	0	0	0	0	0	0	0	0	0	0	0	0	0	0	0	0	0	0	0	0	0	0	0	0	0	0	0	0	0	0	0	0	0	0	0	0	0	0	0	0	0	0
738418	AAAIFMTATPPGSME	NS3	1785	1799	HLA-DRB1*04:07	>0% - 50%	Dengue virus 1	ELISPOT	IFNg release	Positive	0	0	0	0	0	1	0	0	0	0	1	1	0	0	0	0	0	0	0	0	0	0	0	0	0	0	0	0	0	0	0	0	0	1	0	0	1	0	0	0	0	0	0	0	0	0	0	0	0	0	0	0	0	0	0	0	0	0	0	0	0	0	0	0	0	1	0	0	0	0	0	0	0	1	0	0	0	1	1	0	0	0	0	0	0	0	0	1	0	0	0	1	0	0	1	0	0	0	1
195485	IGLLCVMASSALLWM	NS4a	2177	2191	HLA-DRB1*07:01	>0% - 50%	Dengue virus 1	ELISPOT	IFNg release	Positive	0	0	0	0	0	1	1	0	0	0	1	1	0	0	1	0	0	0	0	0	0	1	0	0	1	0	0	0	0	0	0	0	0	0	0	0	0	0	0	0	0	0	1	1	0	1	0	0	0	0	0	0	0	0	0	1	0	1	0	1	0	0	0	0	0	0	0	1	0	0	0	0	0	0	0	0	0	0	0	0	0	0	1	0	0	0	0	0	0	0	1	0	0	1	0	0	0	0	0
195150	AKWLWGFLSRNKKPR	NS5	2870	2884	HLA-DRB1*15:01	>0% - 50%	Dengue virus 1	ELISPOT	IFNg release	Positive	0	0	0	0	0	0	0	0	0	0	0	0	0	0	0	0	0	0	0	1	0	0	0	0	0	0	0	0	0	0	0	1	0	0	0	0	0	1	0	0	0	0	0	0	0	0	1	0	0	0	0	0	0	0	0	0	0	0	0	0	0	0	0	0	0	0	0	0	0	0	0	0	0	0	0	0	0	0	0	0	0	0	0	0	0	0	0	0	0	0	0	0	0	0	0	0	0	0	0
539509	KSEFNIYKRSGIMEV	NS5	2515	2529	HLA-DRB1*15:02	>0% - 50%	Dengue virus 1	ELISPOT	IFNg release	Positive	0	0	0	0	0	0	0	0	0	0	0	0	0	0	0	0	0	0	0	0	0	0	0	0	0	0	0	0	0	0	0	0	0	0	1	0	0	0	0	0	0	0	0	0	0	0	0	0	0	0	0	0	0	0	0	0	0	0	0	0	0	0	0	0	0	0	0	0	0	0	0	0	0	0	0	0	0	0	0	0	0	0	0	0	0	1	0	0	0	0	0	0	0	0	0	0	0	0	0
539537	LAKAIFKLTYQNKVV	NS5	3056	3070	HLA-DRB1*14:04	>0% - 50%	Dengue virus 1	ELISPOT	IFNg release	Positive	1	1	1	1	1	0	0	1	0	1	0	0	1	1	0	1	1	1	0	0	0	0	0	0	0	0	0	1	0	1	0	0	0	0	0	1	0	0	0	0	1	1	0	0	1	0	0	1	1	1	0	0	0	1	1	0	1	0	0	0	0	0	1	0	0	0	0	0	1	0	1	0	0	0	1	1	0	0	0	1	1	1	0	1	1	0	1	0	1	1	0	0	0	0	0	0	0	0	0

QTX92725.1	QTX92781.1	QTX92800.1	QTX92801.1	QTX92846.1	QTX92418.1	QTX92538.1	ADU03651.1	ANC57592.1	QFS19172.1	QFS19175.1	QTX92861.1	QTX92545.1	QTX92553.1	QTX92559.1	BDB33210.1	QFS19159.1	QFS19189.1	QFS19217.1	QFS19226.1	QPQ49765.1	QTX92595.1	QTX92612.1	QTX92670.1	QTX92745.1	QTX92454.1	QTX92462.1	QTX92576.1	QTX92577.1	QFS19151.1	QTX92707.1	QTX92713.1	QTX92716.1	QTX92718.1	QTX92724.1	QTX92857.1	QTX92428.1	QTX92489.1	ANC57585.1	ANC57583.1	QFS19155.1	DENV-1 constructs of vaccines: TV003	TDEN	DPIV	Dengvaxia	DENVax	TVDV
0	0	0	0	0	0	0	0	0	0	0	0	0	0	0	0	0	0	0	0	0	0	0	0	0	0	0	0	0	0	0	0	0	0	0	0	0	0	0	0	0	0	0	0	0	0	0
0	0	0	0	0	0	0	0	0	0	0	0	0	0	0	0	0	0	0	0	0	0	0	0	0	0	0	0	0	0	0	0	0	0	0	0	0	0	0	0	0	0	0	0	0	0	0
0	0	0	0	0	0	0	0	0	0	0	0	0	0	0	0	0	0	0	0	0	0	0	0	0	0	0	0	0	0	0	0	0	0	0	0	0	0	0	0	0	0	0	0	0	0	0
0	0	0	0	0	0	0	0	0	0	0	0	0	0	0	0	0	0	0	0	0	0	0	0	0	0	0	0	0	0	0	0	0	0	0	0	0	0	0	0	0	1	1	1	0	0	0
0	0	0	0	0	0	0	0	0	0	0	0	0	0	0	0	0	0	0	0	0	0	0	0	0	0	0	0	0	0	0	0	0	0	0	0	0	0	0	0	0	1	1	1	0	0	0
0	0	0	0	0	0	0	0	0	0	0	0	0	0	0	0	0	0	0	0	0	0	0	0	0	0	0	0	0	0	0	0	0	0	0	0	0	0	0	0	0	0	0	0	0	0	0
0	0	0	0	0	0	0	0	0	0	0	0	0	0	0	0	0	0	0	0	0	0	0	0	0	0	0	0	0	0	0	0	0	0	0	0	0	0	0	0	0	0	0	0	0	0	0
0	0	0	0	0	0	0	0	0	0	0	0	0	0	0	0	0	0	0	0	0	0	0	0	0	0	0	0	0	0	0	0	0	0	0	0	0	0	0	0	0	1	1	1	0	0	0
0	0	0	0	0	0	0	0	0	0	0	0	0	0	0	0	0	0	0	0	0	0	0	0	0	0	0	0	0	0	0	0	0	0	0	0	0	0	0	0	0	0	0	0	0	0	0
0	0	0	0	0	0	0	0	0	0	0	0	0	0	0	0	0	0	0	0	0	0	0	0	0	0	0	0	0	0	0	0	0	0	0	0	0	0	0	0	0	0	0	0	0	0	0
0	0	0	0	0	0	0	0	0	0	0	0	0	0	0	0	0	0	0	0	0	0	0	0	0	0	0	0	0	0	0	0	0	0	0	0	0	0	0	0	0	1	1	1	0	0	0
0	0	0	0	0	0	0	0	0	0	0	0	0	0	0	0	0	0	0	0	0	0	0	0	0	0	0	0	0	0	0	0	0	0	0	0	0	0	0	0	0	0	0	0	0	0	0
0	0	0	0	0	0	0	0	0	0	0	0	0	0	0	0	0	0	0	0	0	0	0	0	0	0	0	0	0	0	0	0	0	0	0	0	0	0	0	0	0	0	0	0	0	0	0
0	0	0	0	0	0	0	0	0	0	0	0	0	0	0	0	0	0	0	0	0	0	0	0	0	0	0	0	0	0	0	0	0	0	0	0	0	0	0	0	0	0	0	0	0	0	0
0	0	0	0	0	0	0	0	0	0	0	0	0	0	0	0	0	0	0	0	0	0	0	0	0	0	0	0	0	0	0	0	0	0	0	0	0	0	0	0	0	0	0	0	0	0	0
0	0	0	0	0	0	0	0	0	0	0	0	0	0	0	0	0	0	0	0	0	0	0	0	0	0	0	0	0	0	0	0	0	0	0	0	0	0	0	0	0	0	0	0	0	0	0
0	0	0	0	0	0	0	0	0	0	0	0	0	0	0	0	0	0	0	0	0	0	0	0	0	0	0	0	0	0	0	0	0	0	0	0	0	0	0	0	0	0	0	0	0	0	0
0	0	0	0	0	0	0	0	0	0	0	0	0	0	0	0	0	0	0	0	0	0	0	0	0	0	0	0	0	0	0	0	0	0	0	0	0	0	0	0	0	1	1	1	0	1	1
0	0	0	0	0	0	0	0	0	0	0	0	0	0	0	0	0	0	0	0	0	0	0	0	0	0	0	0	0	0	0	0	0	0	0	0	0	0	0	0	0	1	1	1	0	1	1
0	0	0	0	0	0	0	0	0	0	0	0	0	0	0	0	0	0	0	0	0	0	0	0	0	0	0	0	0	0	0	0	0	0	0	0	0	0	0	0	0	0	0	0	0	0	0
0	0	0	0	0	0	0	0	0	0	0	0	0	0	0	0	0	0	0	0	0	0	0	0	0	0	0	0	0	0	0	0	0	0	0	0	0	0	0	0	0	0	0	0	0	0	0
0	0	0	0	0	0	0	0	0	0	0	0	0	0	0	0	0	0	0	0	0	0	0	0	0	0	0	0	0	0	0	0	0	0	0	0	0	0	0	0	0	1	1	1	1	1	1
0	0	0	0	0	0	0	0	0	0	0	0	0	0	0	0	0	0	0	0	0	0	0	0	0	0	0	0	0	0	0	0	0	0	0	0	0	0	0	0	0	0	0	0	0	0	0
0	0	0	0	0	0	0	0	0	0	0	0	0	0	0	0	0	0	0	0	0	0	0	0	0	0	0	0	0	0	0	0	0	0	0	0	0	0	0	0	0	0	0	0	0	0	0
[illegible]	[illegible]	[illegible]	[illegible]	[illegible]	[illegible]	[illegible]	[illegible]	[illegible]	[illegible]	[illegible]	[illegible]	[illegible]	[illegible]	[illegible]	[illegible]	[illegible]	[illegible]	[illegible]	[illegible]	[illegible]	[illegible]	[illegible]	[illegible]	[illegible]	[illegible]	[illegible]	[illegible]	[illegible]	[illegible]	[illegible]	[illegible]	[illegible]	[illegible]	[illegible]	[illegible]	[illegible]	[illegible]	[illegible]	[illegible]	[illegible]	[illegible]	[illegible]	[illegible]	[illegible]	[illegible]	[illegible]

0	0	0	0	0	0	0	0	0	0	0	0	0	0	0	0	0	0	0	0	0	0	0	0	0	0	0	0	0	0	0	0	0	1	0	0	0	0	0	0	0	0	0	0	0	0	0
0	0	0	0	0	0	0	0	0	0	0	0	0	0	0	0	0	0	0	0	0	0	0	0	0	0	0	0	0	0	0	0	1	0	0	0	0	0	0	0	0	0	0	0	0	0	0
0	1	0	1	0	1	0	0	0	0	0	0	0	0	0	0	0	0	0	0	0	0	1	0	0	1	0	0	0	0	0	0	0	0	0	0	1	0	0	0	0	0	0	0	0	0	0
0	0	0	0	0	0	0	0	0	0	0	0	0	0	0	0	0	0	0	0	0	0	0	0	0	0	0	0	0	0	0	0	0	0	0	0	0	0	0	0	0	0	0	0	0	0	0
0	1	0	1	0	1	0	0	0	0	0	0	0	0	0	1	0	0	0	0	0	0	1	0	0	1	0	0	0	0	0	0	0	0	0	0	0	0	0	0	0	0	0	0	0	0	0
0	0	0	0	0	0	0	0	0	0	0	0	0	0	0	0	0	0	0	0	0	0	0	0	0	0	0	0	0	0	0	0	0	0	0	0	0	0	0	0	0	0	0	0	0	0	0
0	0	0	0	0	0	0	0	0	0	0	0	0	0	0	0	0	0	0	0	0	0	0	0	0	0	0	0	0	0	0	0	0	0	1	0	0	0	0	0	0	0	0	0	0	0	0
1	0	0	0	1	0	1	0	0	1	0	1	0	0	0	0	1	0	0	0	0	0	0	0	0	0	1	0	0	0	1	0	0	0	0	0	0	0	0	0	0	0	0	0	0	0	0
0	1	0	0	0	0	0	0	0	0	0	0	0	0	0	0	0	0	0	0	0	0	0	0	0	0	0	0	0	0	0	0	0	0	0	0	1	0	0	0	0	0	0	0	0	0	0
0	0	0	0	0	0	0	0	0	0	0	0	0	0	1	0	0	0	0	0	0	0	0	0	0	0	0	0	0	0	0	0	0	0	0	0	0	0	0	0	0	0	0	0	0	0	0
0	0	0	0	0	0	0	1	0	0	0	0	0	0	0	1	0	1	1	1	1	0	0	1	0	0	0	1	0	0	0	0	1	0	0	1	0	1	0	1	1	0	0	0	0	0	0

CD4 DENV-2 Epitopes

											NCBI IDs of DENV-2 circulating strains from Thailand, (100% identity=1,green ; else 0, red)													DENV-2 constructs of vaccines					
Epitope_ID	AA_Seq	Protein_Name	Start	End	Allele	step	Serotype	Method/Technique	Assay Group	Qualitative Measure	ANC57599.1	QXY23100.1	QXY23099.1	BBG31508.1	QBK46950.1	ANC57598.1	BBG31503.1	BBG31505.1	QFS19150.1	QBA29681.1	QXY23103.1	BBG31506.1	BBG31507.1	TV003	TDEN	DPIV	Dengvaxia	DENVax	TVDV
150404	LRLFMALVAFLRFLT	C	44	58	HLA-DRB1*11:01	0%	Dengue virus 2	ELISPOT	IFNg release	Positive	0	0	0	0	0	0	0	0	0	0	0	0	0	0	0	0	0	0	0
195121	AGMIIMLIPTVVAFH	C	102	116	HLA-DRB1*08:02	0%	Dengue virus 2	ELISPOT	IFNg release	Positive	0	0	0	0	0	0	0	0	0	0	0	0	0	0	0	0	0	0	0
195369	GIIIMIIPTVMAFHL	C	103	117	HLA-DRB1*08:02	0%	Dengue virus 2	ELISPOT	IFNg release	Positive	0	0	0	0	0	0	0	0	0	0	0	0	0	0	0	0	0	0	0
195413	GPLRLFMALVAFLRF	C	42	56	HLA-DRB1*04:01	0%	Dengue virus 2	ELISPOT	IFNg release	Positive	0	0	0	0	0	0	0	0	0	0	0	0	0	0	0	0	0	0	0
195432	GVIIMMIPTVMAFHL	C	103	117	HLA-DRB1*08:02	0%	Dengue virus 2	ELISPOT	IFNg release	Positive	0	0	0	0	0	0	0	0	0	0	0	0	0	0	0	0	0	0	0
195490	IGRMLNILSRRRRTA	C	88	102	HLA-DRB1*11:01	0%	Dengue virus 2	ELISPOT	IFNg release	Positive	0	0	0	0	0	0	0	0	0	0	0	0	0	0	0	0	0	0	0
195654	LKLYMALVAFLRFLT	C	44	58	HLA-DRB1*15:01	0%	Dengue virus 2	ELISPOT	IFNg release	Positive	0	0	0	0	0	0	0	0	0	0	0	0	0	0	0	0	0	0	0
195899	RMLNILNRRRRTAGV	C	71	85	HLA-DRB1*08:02	0%	Dengue virus 2	ELISPOT	IFNg release	Positive	0	0	0	0	0	0	0	0	0	0	0	0	0	0	0	0	0	0	0
195910	RTAGVIIMMIPTVVA	C	100	114	HLA-DRB1*08:02	0%	Dengue virus 2	ELISPOT	IFNg release	Positive	0	0	0	0	0	0	0	0	0	0	0	0	0	0	0	0	0	0	0
195911	RTASVIVMLIPTVMA	C	100	114	HLA-DRB1*08:02	0%	Dengue virus 2	ELISPOT	IFNg release	Positive	0	0	0	0	0	0	0	0	0	0	0	0	0	0	0	0	0	0	0
196024	TAGVIVMLIPTVMAF	C	101	115	HLA-DRB1*08:02	0%	Dengue virus 2	ELISPOT	IFNg release	Positive	0	0	0	0	0	0	0	0	0	0	0	0	0	0	0	0	0	0	0
196084	TVGVIIMLIPTAMAF	C	101	115	HLA-DRB1*08:02	0%	Dengue virus 2	ELISPOT	IFNg release	Positive	0	0	0	0	0	0	0	0	0	0	0	0	0	0	0	0	0	0	0
53867	RGFRKEIGRMLNILNRRRS	C	17	35	HLA class II	0%	Dengue virus 2	ELISPOT	IFNg release	Positive	0	0	0	0	0	0	0	0	0	0	0	0	0	0	0	0	0	0	0
539046	AFLRFLTIPPTVGIL	C	52	66	HLA-DRB1*10:01	0%	Dengue virus 2	ELISPOT	IFNg release	Positive	0	0	0	0	0	0	0	0	0	0	0	0	0	0	0	0	0	0	0
539051	AFVAFLRFLTIPPTA	C	9	23	HLA-DRB1*15:02	0%	Dengue virus 2	ELISPOT	IFNg release	Positive	0	0	0	0	0	0	0	0	0	0	0	0	0	0	0	0	0	0	0
539213	EIGRMLNILNKRRRT	C	87	101	HLA-DRB1*13:01	0%	Dengue virus 2	ELISPOT	IFNg release	Positive	0	0	0	0	0	0	0	0	0	0	0	0	0	0	0	0	0	0	0
539343	GRMLNIMNRRRRTAG	C	89	103	HLA-DRB1*13:01	0%	Dengue virus 2	ELISPOT	IFNg release	Positive	0	0	0	0	0	0	0	0	0	0	0	0	0	0	0	0	0	0	0
539549	LFTALVAFLRFLTIP	C	46	60	HLA-DRB1*15:06	0%	Dengue virus 2	ELISPOT	IFNg release	Positive	0	0	0	0	0	0	0	0	0	0	0	0	0	0	0	0	0	0	0
62853	TAGILKRWGTIKKSKANVL	C	62	81	HLA class II	0%	Dengue virus 2	ELISPOT	IFNg release	Positive	0	0	0	0	0	0	0	0	0	0	0	0	0	0	0	0	0	0	0
739176	IGRMLNILNKRRRTA	C	88	102	HLA-DRB1*14:02	0%	Dengue virus 2	ELISPOT	IFNg release	Positive	0	0	0	0	0	0	0	0	0	0	0	0	0	0	0	0	0	0	0
739179	IGRMLNILNRRRRSV	C	88	102	HLA-DRB1*14:02	0%	Dengue virus 2	ELISPOT	IFNg release	Positive	0	0	0	0	0	0	0	0	0	0	0	0	0	0	0	0	0	0	0
739180	IGRMLNILNRRRRTT	C	88	102	HLA-DRB1*14:02	0%	Dengue virus 2	ELISPOT	IFNg release	Positive	0	0	0	0	0	0	0	0	0	0	0	0	0	0	0	0	0	0	0
739181	IGRMLNILNRRRRTV	C	42	56	HLA-DRB1*14:02	0%	Dengue virus 2	ELISPOT	IFNg release	Positive	0	0	0	0	0	0	0	0	0	0	0	0	0	0	0	0	0	0	0
739182	IGRMLNIMNRRRRTA	C	88	102	HLA-DRB1*14:02	0%	Dengue virus 2	ELISPOT	IFNg release	Positive	0	0	0	0	0	0	0	0	0	0	0	0	0	0	0	0	0	0	0
739183	IGRMMNILNRRRRTA	C	88	102	HLA-DRB1*14:02	0%	Dengue virus 2	ELISPOT	IFNg release	Positive	0	0	0	0	0	0	0	0	0	0	0	0	0	0	0	0	0	0	0
740579	VAFLRFLTISPTAGI	C	51	65	HLA-DRB1*04:07	0%	Dengue virus 2	ELISPOT	IFNg release	Positive	0	0	0	0	0	0	0	0	0	0	0	0	0	0	0	0	0	0	0
866992	GPLKLYMALVAFLRF	C	42	56	HLA-DRB5*01:01	0%	Dengue virus 2	ELISPOT	IFNg release	Positive	0	0	0	0	0	0	0	0	0	0	0	0	0	0	0	0	0	0	0
867734	LKGFRKEIGRMLNIL	C	81	95	HLA-DRB3*02:02	0%	Dengue virus 2	ELISPOT	IFNg release	Positive	0	0	0	0	0	0	0	0	0	0	0	0	0	0	0	0	0	0	0
867910	LRGFRKEIGRMLNIM	C	81	95	HLA-DRB3*02:02	0%	Dengue virus 2	ELISPOT	IFNg release	Positive	0	0	0	0	0	0	0	0	0	0	0	0	0	0	0	0	0	0	0
867911	LRGFRREIGRMLNIL	C	81	95	HLA-DRB3*02:02	0%	Dengue virus 2	ELISPOT	IFNg release	Positive	0	0	0	0	0	0	0	0	0	0	0	0	0	0	0	0	0	0	0
868674	RLFMALVAFLRFLTI	C	45	59	HLA-DPB1*04:02	0%	Dengue virus 2	ELISPOT	IFNg release	Positive	0	0	0	0	0	0	0	0	0	0	0	0	0	0	0	0	0	0	0
150280	HPGFTLMAAILAYTI	PreM	244	258	HLA-DRB1*01:01	0%	Dengue virus 2	ELISPOT	IFNg release	Positive	0	0	0	0	0	0	0	0	0	0	0	0	0	0	0	0	0	0	0
195318	FQKILIFILLTAVAP	PreM	263	277	HLA-DRB1*07:01	0%	Dengue virus 2	ELISPOT	IFNg release	Positive	0	0	0	0	0	0	0	0	0	0	0	0	0	0	0	0	0	0	0
195320	FQRTLIFILLTAVAP	PreM	263	277	HLA-DRB1*07:01	0%	Dengue virus 2	ELISPOT	IFNg release	Positive	0	0	0	0	0	0	0	0	0	0	0	0	0	0	0	0	0	0	0
195448	HFQRALIFILLTAVA	PreM	262	276	HLA-DRB1*07:01	0%	Dengue virus 2	ELISPOT	IFNg release	Positive	0	0	0	0	0	0	0	0	0	0	0	0	0	1	1	1	1	0	1
195504	ILRHPGFTLMAAILA	PreM	241	255	HLA-DRB1*15:01	0%	Dengue virus 2	ELISPOT	IFNg release	Positive	0	0	0	0	0	0	0	0	0	0	0	0	0	0	0	0	0	0	0
195862	QRALIFILLTAVAPS	PreM	264	278	HLA-DRB1*15:01	0%	Dengue virus 2	ELISPOT	IFNg release	Positive	0	0	0	0	0	0	0	0	0	0	0	0	0	1	1	1	1	0	1
196141	VLIFILLTAIAPSMT	PreM	266	280	HLA-DRB1*08:02	0%	Dengue virus 2	ELISPOT	IFNg release	Positive	0	0	0	0	0	0	0	0	0	0	0	0	0	0	0	0	0	0	0
539881	RVLIFILLTAIAPSM	PreM	265	279	HLA-DRB1*15:06	0%	Dengue virus 2	ELISPOT	IFNg release	Positive	0	0	0	0	0	0	0	0	0	0	0	0	0	0	0	0	0	0	0
866485	EDGVNMCTLMAIDLG	PreM	142	156	HLA-DQA1*01:02/DQB1*06:02	0%	Dengue virus 2	ELISPOT	IFNg release	Positive	0	0	0	0	0	0	0	0	0	0	0	0	0	0	0	0	0	0	0
868650	RHPGFTLMAAILAYT	PreM	243	257	HLA-DQA1*01:02/DQB1*06:02	0%	Dengue virus 2	ELISPOT	IFNg release	Positive	0	0	0	0	0	0	0	0	0	0	0	0	0	0	0	0	0	0	0
195793	NLEYTIMITPHSGEE	E	134	148	HLA-DRB1*04:01	0%	Dengue virus 2	ELISPOT	IFNg release	Positive	0	0	0	0	0	0	0	0	0	0	0	0	0	0	0	0	0	0	0
539427	IQKKTLVTFKNPHAK	E	512	526	HLA-DRB1*04:03	0%	Dengue virus 2	ELISPOT	IFNg release	Positive	0	0	0	0	0	0	0	0	0	0	0	0	0	0	0	0	0	0	0
539462	KEMLVTFKNPHAKRQ	E	514	528	HLA-DRB1*04:03	0%	Dengue virus 2	ELISPOT	IFNg release	Positive	0	0	0	0	0	0	0	0	0	0	0	0	0	0	0	0	0	0	0
539479	KKTLVTFKNPHAKKQ	E	514	528	HLA-DRB1*14:04	0%	Dengue virus 2	ELISPOT	IFNg release	Positive	0	0	0	0	0	0	0	0	0	0	0	0	0	0	0	0	0	0	0
539629	LSVTLVLVGFVTLYL	E	475	489	HLA-DRB1*15:06	0%	Dengue virus 2	ELISPOT	IFNg release	Positive	0	0	0	0	0	0	0	0	0	0	0	0	0	0	0	0	0	0	0
866243	AWMVHRQWFLDLPLP	E	205	219	HLA-DQA1*01:01/DQB1*05:01	0%	Dengue virus 2	ELISPOT	IFNg release	Positive	0	0	0	0	0	0	0	0	0	0	0	0	0	0	0	0	0	0	0
867375	KAWLVHRQWFLDLPF	E	204	218	HLA-DQA1*01:01/DQB1*05:01	0%	Dengue virus 2	ELISPOT	IFNg release	Positive	0	0	0	0	0	0	0	0	0	0	0	0	0	0	0	0	0	0	0
868347	PPFGDSYVIIGVEPG	E	371	385	HLA-DQA1*03:01/DQB1*03:02	0%	Dengue virus 2	ELISPOT	IFNg release	Positive	0	0	0	0	0	0	0	0	0	0	0	0	0	0	0	0	0	0	0
869152	TGLDFNEMVLLQMED	E	189	203	HLA-DQA1*03:01/DQB1*03:02	0%	Dengue virus 2	ELISPOT	IFNg release	Positive	0	0	0	0	0	0	0	0	0	0	0	0	0	0	0	0	0	0	0
195583	KQDVFCDSKLMSAAI	NS1	949	963	HLA-DRB1*03:01	0%	Dengue virus 2	ELISPOT	IFNg release	Positive	0	0	0	0	0	0	0	0	0	0	0	0	0	0	0	1	0	1	0
195880	REKQDVFCDSKLMSA	NS1	947	961	HLA-DRB1*03:01	0%	Dengue virus 2	ELISPOT	IFNg release	Positive	0	0	0	0	0	0	0	0	0	0	0	0	0	0	0	1	0	0	0
539708	NDTWKMEKASFIEVK	NS1	982	996	HLA-DRB1*09:01	0%	Dengue virus 2	ELISPOT	IFNg release	Positive	0	0	0	0	0	0	0	0	0	0	0	0	0	0	0	0	0	0	0
739191	IKGIMHAGKRSLRPQ	NS1	868	882	HLA-DRB1*14:02	0%	Dengue virus 2	ELISPOT	IFNg release	Positive	0	0	0	0	0	0	0	0	0	0	0	0	0	0	0	0	0	0	0
866699	FGVFTTNIWLKLREK	NS1	935	949	HLA-DPB1*04:01	0%	Dengue virus 2	ELISPOT	IFNg release	Positive	0	0	0	0	0	0	0	0	0	0	0	0	0	0	0	1	0	0	0
866829	GFGFFTTNIWLKLRE	NS1	934	948	HLA-DPB1*01:01	0%	Dengue virus 2	ELISPOT	IFNg release	Positive	0	0	0	0	0	0	0	0	0	0	0	0	0	0	0	0	0	0	0
866831	GFGVFTTNIWLKLRE	NS1	934	948	HLA-DPB1*04:01	0%	Dengue virus 2	ELISPOT	IFNg release	Positive	0	0	0	0	0	0	0	0	0	0	0	0	0	0	0	1	0	0	0
869722	YGFGVFTTNIWLKLR	NS1	933	947	HLA-DPB1*04:01	0%	Dengue virus 2	ELISPOT	IFNg release	Positive	0	0	0	0	0	0	0	0	0	0	0	0	0	0	0	1	0	0	0
150245	GMGVTYLALLAAYKV	NS2a	1196	1210	HLA-DRB1*01:01	0%	Dengue virus 2	ELISPOT	IFNg release	Positive	0	0	0	0	0	0	0	0	0	0	0	0	0	0	0	0	0	0	0
195269	ERYQLAVTITAILCV	NS2a	1268	1282	HLA-DRB1*07:01	0%	Dengue virus 2	ELISPOT	IFNg release	Positive	0	0	0	0	0	0	0	0	0	0	0	0	0	0	0	0	0	0	0
195523	IPLVLIIKGLNPTAI	NS2a	1319	1333	HLA-DRB1*08:02	0%	Dengue virus 2	ELISPOT	IFNg release	Positive	0	0	0	0	0	0	0	0	0	0	0	0	0	0	0	0	0	0	0
195712	LSLTFIRSTTPLIMA	NS2a	1275	1289	HLA-DRB1*07:01	0%	Dengue virus 2	ELISPOT	IFNg release	Positive	0	0	0	0	0	0	0	0	0	0	0	0	0	0	0	0	0	0	0
195753	MEKYQLAVTIMAISC	NS2a	1267	1281	HLA-DRB1*10:01	0%	Dengue virus 2	ELISPOT	IFNg release	Positive	0	0	0	0	0	0	0	0	0	0	0	0	0	0	0	0	0	0	0
195763	MMVLKIVRKMERYQL	NS2a	1258	1272	HLA-DRB1*03:01	0%	Dengue virus 2	ELISPOT	IFNg release	Positive	0	0	0	0	0	0	0	0	0	0	0	0	0	0	0	0	0	0	0
195799	NMEKYQLAVTIMAIS	NS2a	1266	1280	HLA-DRB1*04:01	0%	Dengue virus 2	ELISPOT	IFNg release	Positive	0	0	0	0	0	0	0	0	0	0	0	0	0	0	0	0	0	0	0
196217	WKVSCTILAAVSVSP	NS2a	1292	1306	HLA-DRB1*04:01	0%	Dengue virus 2	ELISPOT	IFNg release	Positive	0	0	0	0	0	0	0	0	0	0	0	0	0	0	0	0	0	0	0
196237	YQLAVTIMAISCVPN	NS2a	1270	1284	HLA-DRB1*08:02	0%	Dengue virus 2	ELISPOT	IFNg release	Positive	0	0	0	0	0	0	0	0	0	0	0	0	0	0	0	0	0	0	0
539077	ALGIMVLKMVRSMEK	NS2a	1255	1269	HLA-DRB1*12:02	0%	Dengue virus 2	ELISPOT	IFNg release	Positive	0	0	0	0	0	0	0	0	0	0	0	0	0	0	0	0	0	0	0
539082	ALTIKGLNPTAILLT	NS2a	1322	1336	HLA-DRB1*09:01	0%	Dengue virus 2	ELISPOT	IFNg release	Positive	0	0	0	0	0	0	0	0	0	0	0	0	0	0	0	0	0	0	0
539110	AWALGMMVLKIVRNM	NS2a	1253	1267	HLA-DRB1*13:01	0%	Dengue virus 2	ELISPOT	IFNg release	Positive	0	0	0	0	0	0	0	0	0	0	0	0	0	0	0	0	0	0	0
539392	IALGIMVLKMVRSME	NS2a	1254	1268	HLA-DRB1*13:01	0%	Dengue virus 2	ELISPOT	IFNg release	Positive	0	0	0	0	0	0	0	0	0	0	0	0	0	0	0	0	0	0	0
539556	LGIMILKLVRKMEKY	NS2a	1256	1270	HLA-DRB1*12:02	0%	Dengue virus 2	ELISPOT	IFNg release	Positive	0	0	0	0	0	0	0	0	0	0	0	0	0	0	0	0	0	0	0
539557	LGIMVLKIVRSMEKY	NS2a	1256	1270	HLA-DRB1*12:02	0%	Dengue virus 2	ELISPOT	IFNg release	Positive	0	0	0	0	0	0	0	0	0	0	0	0	0	0	0	0	0	0	0
539561	LGMMILKIVRNMEKY	NS2a	1256	1270	HLA-DRB1*13:01	0%	Dengue virus 2	ELISPOT	IFNg release	Positive	0	0	0	0	0	0	0	0	0	0	0	0	0	0	0	0	0	0	0
539706	NAWKVSCTILAAVSV	NS2a	1290	1304	HLA-DRB1*09:01	0%	Dengue virus 2	ELISPOT	IFNg release	Positive	0	0	0	0	0	0	0	0	0	0	0	0	0	0	0	0	0	0	0
539893	SCTILAAVSVSPLLL	NS2a	1295	1309	HLA-DRB1*10:01	0%	Dengue virus 2	ELISPOT	IFNg release	Positive	0	0	0	0	0	0	0	0	0	0	0	0	0	0	0	0	0	0	0
866586	EKYQLAVTIMAISCV	NS2a	1268	1282	HLA-DQA1*01:02/DQB1*06:02	0%	Dengue virus 2	ELISPOT	IFNg release	Positive	0	0	0	0	0	0	0	0	0	0	0	0	0	0	0	0	0	0	0
195913	RTGLLVISGVFPVSI	NS2b	1445	1459	HLA-DRB1*08:02	0%	Dengue virus 2	ELISPOT	IFNg release	Positive	0	0	0	0	0	0	0	0	0	0	0	0	0	0	0	0	0	0	0
539574	LIRTGLLVISGVFPV	NS2b	1443	1457	HLA-DRB1*13:01	0%	Dengue virus 2	ELISPOT	IFNg release	Positive	0	0	0	0	0	0	0	0	0	0	0	0	0	0	0	0	0	0	0
24916	HTGREIVDLMCHATE	NS3	251	265	HLA-DPw2	0%	Dengue virus 2	ICS	IFNg release	Positive	0	0	0	0	0	0	0	0	0	0	0	0	0	0	0	0	0	0	0
539283	GAYRIKQRGILGYSQ	NS3	1496	1510	HLA-DRB1*08:03	0%	Dengue virus 2	ELISPOT	IFNg release	Positive	0	0	0	0	0	0	0	0	0	0	0	0	0	0	0	0	0	0	0
54390	RKLTIMDLHPGAGKT	NS3	618	632	HLA class II	0%	Dengue virus 2	ICS	IFNg release	Positive	0	0	0	0	0	0	0	0	0	0	0	0	0	0	0	1	0	0	0
868183	MWHVTRGAVLMHRGK	NS3	1524	1538	HLA-DRB3*02:02	0%	Dengue virus 2	ELISPOT	IFNg release	Positive	0	0	0	0	0	0	0	0	0	0	0	0	0	0	0	0	0	0	0
150192	ETLLLLTLLAAVTGG	NS4a	2146	2160	HLA-DRB1*01:01	0%	Dengue virus 2	ELISPOT	IFNg release	Positive	0	0	0	0	0	0	0	0	0	0	0	0	0	0	0	0	0	0	0
195810	NQLTYVVIAILTLVA	NS4a	2225	2239	HLA-DRB1*07:01	0%	Dengue virus 2	ELISPOT	IFNg release	Positive	0	0	0	0	0	0	0	0	0	0	0	0	0	0	0	0	0	0	0
195118	AGLLFSIMRNTTSAR	NS4b	2476	2490	HLA-DRB1*04:01	0%	Dengue virus 2	ELISPOT	IFNg release	Positive	0	0	0	0	0	0	0	0	0	0	0	0	0	0	0	0	0	0	0
195270	ESESNILDIDLRPAS	NS4b	2265	2279	HLA-DRB1*03:01	0%	Dengue virus 2	ELISPOT	IFNg release	Positive	0	0	0	0	0	0	0	0	0	0	0	0	0	0	0	0	0	0	0
195387	GLLFFIMKNTTNTRR	NS4b	2477	2491	HLA-DRB1*04:01	0%	Dengue virus 2	ELISPOT	IFNg release	Positive	0	0	0	0	0	0	0	0	0	0	0	0	0	0	0	0	0	0	0
195394	GLLFSIMKNTTNARR	NS4b	2477	2491	HLA-DRB1*04:01	0%	Dengue virus 2	ELISPOT	IFNg release	Positive	0	0	0	0	0	0	0	0	0	0	0	0	0	0	0	0	0	0	0
195395	GLLFSIMKNTTNSRR	NS4b	2477	2491	HLA-DRB1*04:01	0%	Dengue virus 2	ELISPOT	IFNg release	Positive	0	0	0	0	0	0	0	0	0	0	0	0	0	0	0	0	0	0	0
195396	GLLFSIMKNTTSARR	NS4b	2477	2491	HLA-DRB1*04:01	0%	Dengue virus 2	ELISPOT	IFNg release	Positive	0	0	0	0	0	0	0	0	0	0	0	0	0	0	0	0	0	0	0
195397	GLLFSIMKNTTSTRR	NS4b	2477	2491	HLA-DRB1*04:01	0%	Dengue virus 2	ELISPOT	IFNg release	Positive	0	0	0	0	0	0	0	0	0	0	0	0	0	0	0	0	0	0	0
195398	GLLFSVMKNTTNTRR	NS4b	2477	2491	HLA-DRB1*04:01	0%	Dengue virus 2	ELISPOT	IFNg release	Positive	0	0	0	0	0	0	0	0	0	0	0	0	0	0	0	0	0	0	0
195659	LLFSIMRNTTTARRG	NS4b	2478	2492	HLA-DRB1*04:01	0%	Dengue virus 2	ELISPOT	IFNg release	Positive	0	0	0	0	0	0	0	0	0	0	0	0	0	0	0	0	0	0	0

867335	ITLTAALLLLIAHYA	NS4b	2348	2362	HLA-DPB1*04:02	0%	Dengue virus 2	ELISPOT	IFNg release	Positive	0	0	0	0	0	0	0	0	0	0	0	0	0	0	0	0	0	0	0
869090	TAIANQATVLMGLGR	NS4b	2309	2323	HLA-DQA1*01:02/DQB1*06:02	0%	Dengue virus 2	ELISPOT	IFNg release	Positive	0	0	0	0	0	0	0	0	0	0	0	0	0	0	0	0	0	0	0
195111	ACLGKSYAQMWTLMY	NS5	3243	3257	HLA-DRB1*04:01	0%	Dengue virus 2	ELISPOT	IFNg release	Positive	0	0	0	0	0	0	0	0	0	0	0	0	0	0	0	0	0	0	0
195241	EGEGVFKSIQHLTVT	NS5	3101	3115	HLA-DRB1*07:01	0%	Dengue virus 2	ELISPOT	IFNg release	Positive	0	0	0	0	0	0	0	0	0	0	0	0	0	0	0	1	0	0	0
195250	ELIMKDGRKLVVPCR	NS5	3206	3220	HLA-DRB1*03:01	0%	Dengue virus 2	ELISPOT	IFNg release	Positive	0	0	0	0	0	0	0	0	0	0	0	0	0	0	0	0	0	0	0
195384	GLFKSIQHLTSQEEI	NS5	3118	3132	HLA-DRB1*11:01	0%	Dengue virus 2	ELISPOT	IFNg release	Positive	0	0	0	0	0	0	0	0	0	0	0	0	0	0	0	0	0	0	0
195445	HFHELIMKDGRILVV	NS5	3203	3217	HLA-DRB1*03:01	0%	Dengue virus 2	ELISPOT	IFNg release	Positive	0	0	0	0	0	0	0	0	0	0	0	0	0	0	0	0	0	0	0
195446	HFHELVMKDGRVLVV	NS5	3203	3217	HLA-DRB1*13:01	0%	Dengue virus 2	ELISPOT	IFNg release	Positive	0	0	0	0	0	0	0	0	0	0	0	0	0	0	0	0	0	0	0
195460	HRLGYILRDVSRKEG	NS5	3004	3018	HLA-DRB1*08:02	0%	Dengue virus 2	ELISPOT	IFNg release	Positive	0	0	0	0	0	0	0	0	0	0	0	0	0	0	0	0	0	0	0
195513	IMKDGRKLVVPCRNQ	NS5	3207	3221	HLA-DRB1*03:01	0%	Dengue virus 2	ELISPOT	IFNg release	Positive	0	0	0	0	0	0	0	0	0	0	0	0	0	0	0	0	0	0	0
195750	MEGEGIFKSIQHLTA	NS5	3114	3128	HLA-DRB1*11:01	0%	Dengue virus 2	ELISPOT	IFNg release	Positive	0	0	0	0	0	0	0	0	0	0	0	0	0	0	0	0	0	0	0
195759	MLINRFTMKHKKATY	NS5	2729	2743	HLA-DRB1*11:01	0%	Dengue virus 2	ELISPOT	IFNg release	Positive	0	0	0	0	0	0	0	0	0	0	0	0	0	0	0	0	0	0	0
195800	NMISRMLINRFTMKH	NS5	2724	2738	HLA-DRB1*08:02	0%	Dengue virus 2	ELISPOT	IFNg release	Positive	0	0	0	0	0	0	0	0	0	0	0	0	0	0	0	0	0	0	0
196040	TGNIVSSVNMISRML	NS5	2716	2730	HLA-DRB1*04:01	0%	Dengue virus 2	ELISPOT	IFNg release	Positive	0	0	0	0	0	0	0	0	0	0	0	0	0	0	0	0	0	0	0
196225	WTLMYFHRRDLRLAA	NS5	3253	3267	HLA-DRB1*03:01	0%	Dengue virus 2	ELISPOT	IFNg release	Positive	0	0	0	0	0	0	0	0	0	0	0	0	0	0	0	0	0	0	0
32939	KPWDIIPMV	NS5	2821	2829	HLA class II	0%	Dengue virus 2	ELISA	IL-2 release	Positive	0	0	0	0	0	0	0	0	0	0	0	0	0	0	0	0	0	0	0
32941	KPWDVLPMV	NS5	2821	2829	HLA class II	0%	Dengue virus 2	ELISA	IL-2 release	Positive	0	0	0	0	0	0	0	0	0	0	0	0	0	0	0	0	0	0	0
539161	DHWFSRGNSLSGVEG	NS5	2986	3000	HLA-DRB1*09:01	0%	Dengue virus 2	ELISPOT	IFNg release	Positive	0	0	0	0	0	0	0	0	0	0	0	0	0	0	0	0	0	0	0
539560	LGKNEFQIYKKSGIQ	NS5	2511	2525	HLA-DRB1*15:02	0%	Dengue virus 2	ELISPOT	IFNg release	Positive	0	0	0	0	0	0	0	0	0	0	0	0	0	0	0	0	0	0	0
539668	MISRMLINRFTMKHK	NS5	2725	2739	HLA-DRB1*14:04	0%	Dengue virus 2	ELISPOT	IFNg release	Positive	0	0	0	0	0	0	0	0	0	0	0	0	0	0	0	0	0	0	0
539932	SRMLINRFTMKHKKA	NS5	2727	2741	HLA-DRB1*08:03	0%	Dengue virus 2	ELISPOT	IFNg release	Positive	0	0	0	0	0	0	0	0	0	0	0	0	0	0	0	0	0	0	0
540066	VNMISRMLINRFTMK	NS5	2723	2737	HLA-DRB1*12:02	0%	Dengue virus 2	ELISPOT	IFNg release	Positive	0	0	0	0	0	0	0	0	0	0	0	0	0	0	0	0	0	0	0
869083	SYAQMWTLMYFHRRD	NS5	3248	3262	HLA-DPB1*04:01	0%	Dengue virus 2	ELISPOT	IFNg release	Positive	0	0	0	0	0	0	0	0	0	0	0	0	0	0	0	0	0	0	0
869148	TFVESWEDIPYLGKR	NS5	3319	3333	HLA-DQA1*01:01/DQB1*05:01	0%	Dengue virus 2	ELISPOT	IFNg release	Positive	0	0	0	0	0	0	0	0	0	0	0	0	0	0	0	0	0	0	0
869292	TPVESWEEVPYLGKR	NS5	3319	3333	HLA-DQA1*01:01/DQB1*05:01	0%	Dengue virus 2	ELISPOT	IFNg release	Positive	0	0	0	0	0	0	0	0	0	0	0	0	0	0	0	0	0	0	0
12606	EIVDLMCHATFTMRL	NS3	255	269	HLA class II	100%	Dengue virus 2	ELISPOT	IFNg release	Positive	1	1	1	1	1	1	1	1	1	1	1	1	1	0	1	1	0	1	0
866802	FWNTTIAVSMANIFR	NS4b	2455	2469	HLA-DQA1*01:02/DQB1*06:02	100%	Dengue virus 2	ELISPOT	IFNg release	Positive	1	1	1	1	1	1	1	1	1	1	1	1	1	0	1	1	0	1	0
150597	SRAIWYMWLGARFLE	NS5	2962	2976	HLA-DRB1*01:01	100%	Dengue virus 2	ELISPOT	IFNg release	Positive	1	1	1	1	1	1	1	1	1	1	1	1	1	0	1	1	0	1	0
195233	EDHWFSRENSLSGVE	NS5	2985	2999	HLA-DRB1*04:01	100%	Dengue virus 2	ELISPOT	IFNg release	Positive	1	1	1	1	1	1	1	1	1	1	1	1	1	0	1	1	0	1	0
539599	LMYFHRRDLRLAANA	NS5	3255	3269	HLA-DRB1*13:01	100%	Dengue virus 2	ELISPOT	IFNg release	Positive	1	1	1	1	1	1	1	1	1	1	1	1	1	0	1	1	0	1	0
539603	LNTFTNMEAQLIRQM	NS5	3100	3114	HLA-DRB1*10:01	100%	Dengue virus 2	ELISPOT	IFNg release	Positive	1	1	1	1	1	1	1	1	1	1	1	1	1	0	1	1	0	1	0
539871	RRDLRLAANAICSAV	NS5	3260	3274	HLA-DRB3*02:02	100%	Dengue virus 2	ELISPOT	IFNg release	Positive	1	1	1	1	1	1	1	1	1	1	1	1	1	0	1	1	0	1	0
866015	AIWYMWLGARFLEFE	NS5	2964	2978	HLA-DPB1*01:01	100%	Dengue virus 2	ELISPOT	IFNg release	Positive	1	1	1	1	1	1	1	1	1	1	1	1	1	0	1	1	0	1	0
868637	RDLRLAANAICSAVP	NS5	3261	3275	HLA-DQA1*05:01/DQB1*02:01	100%	Dengue virus 2	ELISPOT	IFNg release	Positive	1	1	1	1	1	1	1	1	1	1	1	1	1	0	1	1	0	1	0
150369	LKLFMALVAFLRFLT	C	44	58	HLA-DRB1*01:01	81% - <100%	Dengue virus 2	ELISPOT	IFNg release	Positive	1	1	1	1	1	1	1	1	0	1	0	1	1	0	1	1	0	1	0
180768	TIKKSKAINVLRGFR	C	71	85	HLA class II	81% - <100%	Dengue virus 2	ELISPOT	IFNg release	Positive	0	1	1	1	1	1	1	1	0	1	1	1	1	0	1	1	0	1	0
195326	FRKEIGRMLNILNRR	C	84	98	HLA-DRB1*04:01	81% - <100%	Dengue virus 2	ELISPOT	IFNg release	Positive	0	1	1	1	1	1	1	1	0	1	1	1	1	0	1	1	0	1	0
195556	KEIGRMLNILNRRRR	C	86	100	HLA-DRB1*08:02	81% - <100%	Dengue virus 2	ELISPOT	IFNg release	Positive	0	1	1	1	1	1	1	1	0	1	1	1	1	0	1	1	0	1	0
195573	KLFMALVAFLRFLTI	C	45	59	HLA-DRB1*15:01	81% - <100%	Dengue virus 2	ELISPOT	IFNg release	Positive	1	1	1	1	1	1	1	1	0	1	0	1	1	0	1	1	0	1	0
195886	RGFRKEIGRMLNILN	C	82	96	HLA-DRB1*04:01	81% - <100%	Dengue virus 2	ELISPOT	IFNg release	Positive	0	1	1	1	1	1	1	1	0	1	1	1	1	0	1	1	0	1	0
195889	RGPLKLFMALVAFLR	C	41	55	HLA-DRB1*04:01	81% - <100%	Dengue virus 2	ELISPOT	IFNg release	Positive	1	1	1	1	1	1	1	1	0	1	0	1	1	0	1	1	0	1	0
196064	TPFNMLKRERNRVST	C	11	25	HLA-DRB1*08:02	81% - <100%	Dengue virus 2	ELISPOT	IFNg release	Positive	1	1	1	1	1	1	1	1	0	1	1	1	1	0	1	1	0	1	0
196213	WGTIKKSKAINVLRG	C	69	83	HLA-DRB1*07:01	81% - <100%	Dengue virus 2	ELISPOT	IFNg release	Positive	0	1	1	1	1	1	1	1	0	1	1	1	1	0	1	1	0	1	0
26836	IKKSKAINVLRGFRKEIGRM	C	72	91	HLA class II	81% - <100%	Dengue virus 2	ELISPOT	IFNg release	Positive	0	1	1	1	1	1	1	1	0	1	1	1	1	0	1	1	0	1	0
39098	LRGFRKEIGRML	C	81	92	HLA-DPw4	81% - <100%	Dengue virus 2	ICS	IFNg release	Positive	0	1	1	1	1	1	1	1	0	1	1	1	1	0	1	1	0	1	0
539264	FLRFLTIPPTAGILK	C	53	67	HLA-DRB1*14:02	81% - <100%	Dengue virus 2	ELISPOT	IFNg release	Positive	0	1	1	1	1	1	1	1	0	1	1	1	1	0	1	1	0	1	0
539410	IKKSKAINVLRGFRK	C	72	86	HLA-DRB1*14:04	81% - <100%	Dengue virus 2	ELISPOT	IFNg release	Positive	0	1	1	1	1	1	1	1	0	1	1	1	1	0	1	1	0	1	0
539514	KSKAINVLRGFRKEI	C	74	88	HLA-DRB1*12:02	81% - <100%	Dengue virus 2	ELISPOT	IFNg release	Positive	0	1	1	1	1	1	1	1	0	1	1	1	1	0	1	1	0	1	0
539612	LQGRGPLKLFMALVA	C	38	52	HLA-DRB1*15:02	81% - <100%	Dengue virus 2	ELISPOT	IFNg release	Positive	1	1	1	1	1	1	1	1	0	1	0	1	1	0	1	1	0	1	0
539618	LRGFRKEIGRMLNIL	C	81	95	HLA-DRB1*09:01	81% - <100%	Dengue virus 2	ELISPOT	IFNg release	Positive	0	1	1	1	1	1	1	1	0	1	1	1	1	0	1	1	0	1	0
539887	RWGTIKKSKAINVLR	C	68	82	HLA-DRB1*08:03	81% - <100%	Dengue virus 2	ELISPOT	IFNg release	Positive	0	1	1	1	1	1	1	1	0	1	1	1	1	0	1	1	0	1	0
540060	VLRGFRKEIGRMLNI	C	80	94	HLA-DRB1*09:01	81% - <100%	Dengue virus 2	ELISPOT	IFNg release	Positive	0	1	1	1	1	1	1	1	0	1	1	1	1	0	1	1	0	1	0
540069	VQQLTKRFSLGMLQG	C	26	40	HLA-DRB1*13:01	81% - <100%	Dengue virus 2	ELISPOT	IFNg release	Positive	1	1	1	1	1	1	1	1	0	1	1	1	1	0	1	1	0	1	0
739879	PFNMLKRERNRVSTV	C	12	26	HLA-DRB1*14:02	81% - <100%	Dengue virus 2	ELISPOT	IFNg release	Positive	1	1	1	1	1	1	1	1	0	1	1	1	1	0	1	1	0	1	0
857784	LKRWGTIKKSKAINV	C	66	80	HLA-DRB1*07:01	81% - <100%	Dengue virus 2	ELISPOT	IFNg release	Positive-Low	0	1	1	1	1	1	1	1	0	1	1	1	1	0	1	1	0	1	0
539943	SVALVPHVGMGLETR	PreM	206	220	HLA-DRB1*15:02	81% - <100%	Dengue virus 2	ELISPOT	IFNg release	Positive	1	1	1	1	1	1	1	1	0	1	1	1	1	1	1	1	1	1	1
180564	KKQDVVVLGSQEGAM	E	526	540	HLA class II	81% - <100%	Dengue virus 2	ELISPOT	IFNg release	Positive	1	1	1	1	1	1	1	1	0	1	1	1	1	1	1	1	1	1	1
180568	KNPHAKKQDVVVLGS	E	521	535	HLA class II	81% - <100%	Dengue virus 2	ELISPOT	IFNg release	Positive	1	1	1	1	1	1	1	1	0	1	1	1	1	1	1	1	1	1	1
180820	WLVHRQWFLDLPLPW	E	196	210	HLA class II	81% - <100%	Dengue virus 2	ELISPOT	IFNg release	Positive	1	1	1	1	1	1	1	1	0	1	1	1	1	1	1	1	1	1	1
195351	GATEIQMSSGNLLFT	E	266	280	HLA-DRB1*03:01	81% - <100%	Dengue virus 2	ELISPOT	IFNg release	Positive	1	1	1	1	1	1	1	1	0	1	1	1	1	1	1	1	1	1	1
195366	GHLKCRLRMDKLQLK	E	281	295	HLA-DRB1*03:01	81% - <100%	Dengue virus 2	ELISPOT	IFNg release	Positive	1	1	1	1	1	1	1	1	0	1	1	1	1	1	1	1	1	1	1
195407	GMNSRSTSLSVSLVL	E	467	481	HLA-DRB1*07:01	81% - <100%	Dengue virus 2	ELISPOT	IFNg release	Positive	1	1	1	1	0	1	1	1	0	1	1	1	1	1	1	1	1	0	0
195638	LGRLITVNPIVTEKD	E	628	642	HLA-DRB1*08:02	81% - <100%	Dengue virus 2	ELISPOT	IFNg release	Positive	1	1	1	1	1	1	1	1	0	1	1	1	1	1	1	1	1	1	1
196140	VLGRLITVNPIVTEK	E	627	641	HLA-DRB1*08:02	81% - <100%	Dengue virus 2	ELISPOT	IFNg release	Positive	1	1	1	1	1	1	1	1	0	1	1	1	0	1	1	1	1	1	1
196215	WIQKETLVTFKNPHA	E	231	245	HLA-DRB1*08:02	81% - <100%	Dengue virus 2	ELISPOT	IFNg release	Positive	1	1	1	1	1	1	1	1	0	1	1	1	1	1	1	1	1	1	1
539252	FETTMRGAKRMAILG	E	402	416	HLA-DRB1*15:02	81% - <100%	Dengue virus 2	ELISPOT	IFNg release	Positive	1	1	1	1	1	1	1	1	0	1	1	1	1	0	1	0	1	1	0
539377	HAKKQDVVVLGSQEGAMH	E	244	261	HLA class II	81% - <100%	Dengue virus 2	ICS	IFNg release	Positive	1	1	1	1	1	1	1	1	0	1	1	1	1	1	1	1	1	1	1
539464	KETLVTFKNPHAKKQ	E	234	248	HLA-DRB1*10:01	81% - <100%	Dengue virus 2	ELISPOT	IFNg release	Positive	1	1	1	1	1	1	1	1	0	1	1	1	1	1	1	1	1	1	1
539648	LVTFKNPHAKKQDVV	E	237	251	HLA-DRB1*15:02	81% - <100%	Dengue virus 2	ELISPOT	IFNg release	Positive	1	1	1	1	1	1	1	1	0	1	1	1	1	1	1	1	1	1	1
539689	MRGAKRMAILGDTAW	E	406	420	HLA-DRB1*15:06	81% - <100%	Dengue virus 2	ELISPOT	IFNg release	Positive	1	1	1	1	1	1	1	1	0	1	1	1	1	1	1	1	1	1	0
539806	QKETLVTFKNPHAKK	E	513	527	HLA-DRB1*04:03	81% - <100%	Dengue virus 2	ELISPOT	IFNg release	Positive	1	1	1	1	1	1	1	1	0	1	1	1	1	1	1	1	1	1	1
65140	TLVTFKNPHAKKQDV	E	236	250	HLA class II	81% - <100%	Dengue virus 2	ELISPOT	IFNg release	Positive	1	1	1	1	1	1	1	1	0	1	1	1	1	1	1	1	1	1	1
739010	GGVFTSIGKALHQVF	E	706	720	HLA-DRB1*14:02	81% - <100%	Dengue virus 2	ELISPOT	IFNg release	Positive	1	1	1	1	1	1	1	1	0	1	1	1	1	1	1	1	1	1	0
741744	MRCIGISNRDFVEGVSGGSW	E	281	300	HLA class II	81% - <100%	Dengue virus 2	ELISPOT	IFNg release	Positive	1	1	1	1	0	1	1	1	0	1	1	1	1	1	1	1	1	0	1
866193	ATEIQMSSGNLLFTG	E	257	271	HLA-DRB3*02:02	81% - <100%	Dengue virus 2	ELISPOT	IFNg release	Positive	1	1	1	1	1	1	1	1	0	1	1	1	1	1	1	1	1	1	1
868713	RTGLDFNEMVLLQME	E	178	192	HLA-DQA1*01:02/DQB1*06:02	81% - <100%	Dengue virus 2	ELISPOT	IFNg release	Positive	1	1	1	1	1	1	1	1	0	1	1	1	1	1	1	1	1	1	1
869123	TEKDSPVNIEAEPPF	E	349	363	HLA-DQA1*03:01/DQB1*03:02	81% - <100%	Dengue virus 2	ELISPOT	IFNg release	Positive	1	1	1	1	1	0	1	1	0	1	1	1	1	1	1	1	1	1	1
869153	TGLDFNEMVLLQMEN	E	179	193	HLA-DQA1*03:01/DQB1*03:02	81% - <100%	Dengue virus 2	ELISPOT	IFNg release	Positive	1	1	1	1	1	1	1	1	0	1	1	1	1	1	1	1	1	1	1
167759	IMTGDIKGIMQA	NS1	88	99	HLA-DQ6	81% - <100%	Dengue virus 2	ELISPOT	IFNg release	Positive	1	1	1	1	1	1	1	1	0	1	1	1	1	0	1	1	0	1	0
180396	DGCWYGMEIRPLKEKEENLVNS	NS1	1102	1123	HLA class II	81% - <100%	Dengue virus 2	ICS	IFNg release	Positive	1	1	0	1	1	1	1	1	0	1	1	1	1	0	1	1	0	1	0
180612	LNDTWKIEKASFIEV	NS1	981	995	HLA class II	81% - <100%	Dengue virus 2	ELISPOT	IFNg release	Positive	1	1	1	1	1	1	1	1	0	1	1	1	1	0	1	1	0	1	0
195576	KLTIMTGDIKGIMQA	NS1	85	99	HLA-DRB1*03:01	81% - <100%	Dengue virus 2	ELISPOT	IFNg release	Positive	1	1	1	1	1	1	1	1	0	1	1	1	1	0	1	1	0	1	0
196133	VKLTIMTGDIKGIMQ	NS1	84	98	HLA-DRB1*03:01	81% - <100%	Dengue virus 2	ELISPOT	IFNg release	Positive	1	1	1	1	1	1	1	1	0	1	1	1	1	0	1	1	0	1	0
539828	QYKFQPESPSKLASA	NS1	806	820	HLA-DRB3*02:02	81% - <100%	Dengue virus 2	ELISPOT	IFNg release	Positive	1	1	1	1	1	1	1	1	0	1	1	1	1	0	1	1	0	1	0
738586	CGIRSVTRLENLMWK	NS1	55	69	HLA-DRB1*11:04	81% - <100%	Dengue virus 2	ELISPOT	IFNg release	Positive	1	1	1	1	1	1	1	1	0	1	1	1	1	0	1	1	0	1	0
738766	EGICGIRSVTRLENL	NS1	52	66	HLA-DRB1*11:04	81% - <100%	Dengue virus 2	ELISPOT	IFNg release	Positive	1	1	1	1	1	1	1	1	0	1	1	1	1	0	1	1	0	1	0
739192	IKGIMQAGKRSLRPQ	NS1	93	107	HLA-DRB1*14:06	81% - <100%	Dengue virus 2	ELISPOT	IFNg release	Positive	1	1	1	1	1	1	1	1	0	1	1	1	1	0	1	0	0	1	0
739296	KGIMQAGKRSLRPQP	NS1	94	108	HLA-DRB1*14:06	81% - <100%	Dengue virus 2	ELISPOT	IFNg release	Positive	1	1	1	1	1	1	1	1	0	1	1	1	1	0	1	0	0	1	0
866460	DYGFGVFTTNIWLKL	NS1	932	946	HLA-DPB1*02:01	81% - <100%	Dengue virus 2	ELISPOT	IFNg release	Positive	1	1	1	1	1	1	1	1	0	1	1	1	1	0	1	1	0	1	0
866830	GFGVFTTNIWLKLKE	NS1	934	948	HLA-DPB1*04:01	81% - <100%	Dengue virus 2	ELISPOT	IFNg release	Positive	1	1	1	1	1	1	1	1	0	1	1	1	1	0	1	0	0	1	0
867040	HADMGYWIESALNDT	NS1	970	984	HLA-DQA1*01:01/DQB1*05:01	81% - <100%	Dengue virus 2	ELISPOT	IFNg release	Positive	1	1	1	1	1	1	1	1	0	1	1	1	1	0	1	1	0	1	0
150131	ALTIKGLNPTAIFLT	NS2a	1308	1322	HLA-DRB1*09:01	81% - <100%	Dengue virus 2	ELISPOT	IFNg release	Positive	1	1	1	1	1	1	1	1	0	1	1	1	1	0	1	1	0	1	0
150273	GVTYLALLAAFKVRP	NS2a	1198	1212	HLA-DRB1*01:01	81% - <100%	Dengue virus 2	ELISPOT	IFNg release	Positive	1	1	1	1	1	1	1	1	0	1	1	1	1	0	1	1	0	1	0
167819	SSQQKTDWIPLALTIKGLNP	NS2a	184	203	HLA-DP	81% - <100%	Dengue virus 2	ELISPOT	IFNg release	Positive	1	1	1	1	1	0	1	1	0	1	1	1	1	0	1	0	0	1	0
195153	ALLAAFKVRPTFAAG	NS2a	1203	1217	HLA-DRB1*08:02	81% - <100%	Dengue virus 2	ELISPOT	IFNg release	Positive	1	1	1	1	1	1	1	1	0	1	1	1	1	0	1	1	0	1	0
195406	GMMVLKIVRNMEKYQ	NS2a	1257	1271	HLA-DRB1*08:02	81% - <100%	Dengue virus 2	ELISPOT	IFNg release	Positive	1	1	1	1	0	1	1	1	0	1	1	1	1	0	0	0	0	0	0
195567	KHAILLVAVSFVTLI	NS2a	1157	1171	HLA-DRB1*07:01	81% - <100%	Dengue virus 2	ELISPOT	IFNg release	Positive	1	1	1	1	1	1	1	1	0	1	1	1	1	0	1	1	0	1	0
195610	LALGMMVLKIVRNME	NS2a	1254	1268	HLA-DRB1*13:01	81% - <100%	Dengue virus 2	ELISPOT	IFNg release	Positive	1	1	1	1	0	1	1	1	0	1	1	1	1	0	0	0	0	0	0
195615	LAVVSVSPLLLTSSQ	NS2a	1186	1200	HLA-DRB1*07:01	81% - <100%	Dengue virus 2	ELISPOT	IFNg release	Positive	1	1	1	1	1	1	1	1	0	1	1	1	1	0	1	0	0	1	0

539231	EMLRTRVGTKHAILL	NS2a	1148	1162	HLA-DRB1*08:03	81% - <100%	Dengue virus 2	ELISPOT	IFNg release	Positive	1	1	1	1	1	1	1	1	0	1	1	1	1	0	1	1	0	1	0
539540	LALLAAFKVRPTFAA	NS2a	1202	1216	HLA-DRB1*14:04	81% - <100%	Dengue virus 2	ELISPOT	IFNg release	Positive	1	1	1	1	1	1	1	1	0	1	1	1	1	0	1	1	0	1	0
539562	LGMMVLKIVRNMEKY	NS2a	1256	1270	HLA-DRB1*12:02	81% - <100%	Dengue virus 2	ELISPOT	IFNg release	Positive	1	1	1	1	0	1	1	1	0	1	1	1	1	0	0	0	0	0	0
539686	MMVLKIVRNMEKYQL	NS2a	1244	1258	HLA-DRB1*14:06	81% - <100%	Dengue virus 2	ELISPOT	IFNg release	Positive	1	1	1	1	0	1	1	1	0	1	1	1	1	0	0	0	0	0	0
540026	VAVSFVTLITGNMSF	NS2a	1163	1177	HLA-DRB1*10:01	81% - <100%	Dengue virus 2	ELISPOT	IFNg release	Positive	1	1	1	1	1	1	1	1	0	1	1	1	1	0	1	1	0	1	0
540094	VTYLALLAAFKVRPT	NS2a	1199	1213	HLA-DRB1*12:02	81% - <100%	Dengue virus 2	ELISPOT	IFNg release	Positive	1	1	1	1	1	1	1	1	0	1	1	1	1	0	1	1	0	1	0
540154	YLALLAAFKVRPTFA	NS2a	1201	1215	HLA-DRB1*14:04	81% - <100%	Dengue virus 2	ELISPOT	IFNg release	Positive	1	1	1	1	1	1	1	1	0	1	1	1	1	0	1	1	0	1	0
740700	VVSVSPLLLTSSQQK	NS2a	1188	1202	HLA-DRB1*01:02	81% - <100%	Dengue virus 2	ELISPOT	IFNg release	Positive	1	1	1	1	1	1	1	1	0	1	1	1	1	0	1	0	0	1	0
866978	GMALFLEEMLRTRVG	NS2a	1141	1155	HLA-DQA1*01:01/DQB1*05:01	81% - <100%	Dengue virus 2	ELISPOT	IFNg release	Positive	1	1	1	1	1	1	1	1	0	1	1	1	1	0	1	1	0	1	0
868918	SLGVLGMALFLEEML	NS2a	1136	1150	HLA-DQA1*05:01/DQB1*02:01	81% - <100%	Dengue virus 2	ELISPOT	IFNg release	Positive	1	1	1	1	1	1	1	1	0	1	1	1	1	0	1	1	0	1	0
869200	TKHAILLVAVSFVTL	NS2a	1156	1170	HLA-DPB1*04:01	81% - <100%	Dengue virus 2	ELISPOT	IFNg release	Positive	1	1	1	1	1	1	1	1	0	1	1	1	1	0	1	1	0	1	0
195239	EEQTLTILIRTGLLV	NS2b	91	105	HLA-DRB1*15:01	81% - <100%	Dengue virus 2	ELISPOT	IFNg release	Positive	1	1	1	1	1	1	1	1	0	1	1	1	1	0	1	1	0	1	0
195723	LTILIRTGLLVISGL	NS2b	1440	1454	HLA-DRB1*15:06	81% - <100%	Dengue virus 2	ELISPOT	IFNg release	Positive	1	1	1	1	1	1	1	1	0	1	1	1	1	0	1	1	0	1	0
195869	QTLTILIRTGLLVIS	NS2b	1438	1452	HLA-DRB1*15:01	81% - <100%	Dengue virus 2	ELISPOT	IFNg release	Positive	1	1	1	1	1	1	1	1	0	1	1	1	1	0	1	1	0	1	0
1067121	GKVVGLYGNGVVTRS	NS3	1619	1633	HLA-DRB1*15:01	81% - <100%	Dengue virus 2	ELISPOT	IFNg release	Positive	1	1	1	1	1	1	1	1	0	1	1	1	1	0	1	1	0	1	0
12372	EHTGREIVDLMCHAT	NS3	250	264	HLA class II	81% - <100%	Dengue virus 2	ELISPOT	IFNg release	Positive	1	1	1	1	1	1	1	1	0	1	1	1	1	0	1	1	0	1	0
1392191	GREIVDLMCHATFTM	NS3	1728	1742		81% - <100%	Dengue virus 2	biological activity	activation	Positive	1	1	1	1	1	1	1	1	0	1	1	1	1	0	1	1	0	1	0
139758	GKTKRYLPAIVREAI	NS3	1673	1687	HLA-DRB1*09:01	81% - <100%	Dengue virus 2	ELISPOT	IFNg release	Positive	1	1	0	1	1	1	1	1	0	1	1	1	1	0	1	1	0	1	0
139762	GLPIRYQTPAIRAEH	NS3	1712	1726	HLA-DRB1*04:03	81% - <100%	Dengue virus 2	ELISPOT	IFNg release	Positive	1	1	1	1	1	1	1	1	0	1	1	1	1	0	1	1	0	1	0
180698	PNYNLIIMDEAHFTD	NS3	1751	1765	HLA class II	81% - <100%	Dengue virus 2	ELISPOT	IFNg release	Positive	1	1	1	1	1	1	1	1	0	1	1	1	1	0	1	1	0	1	0
180807	VTDFKGKTVWFVPSI	NS3	1826	1840	HLA class II	81% - <100%	Dengue virus 2	ELISPOT	IFNg release	Positive-Low	1	1	1	1	1	1	1	1	0	1	1	1	1	0	1	1	0	1	0
195154	ALRGLPIRYQTPAIR	NS3	1709	1723	HLA-DRB1*08:02	81% - <100%	Dengue virus 2	ELISPOT	IFNg release	Positive	1	1	1	1	1	1	1	1	0	1	1	1	1	0	1	1	0	1	0
195295	FHTMWHVTRGAVLMH	NS3	1521	1535	HLA-DRB1*07:01	81% - <100%	Dengue virus 2	ELISPOT	IFNg release	Positive	1	1	1	1	1	1	1	1	0	1	1	1	1	0	1	1	0	1	0
195420	GTFHTMWHVTRGAVL	NS3	1519	1533	HLA-DRB1*07:01	81% - <100%	Dengue virus 2	ELISPOT	IFNg release	Positive	1	1	1	1	1	1	1	1	0	1	1	1	1	0	1	1	0	1	0
195564	KGKVVGLYGNGVVTR	NS3	1618	1632	HLA-DRB1*15:01	81% - <100%	Dengue virus 2	ELISPOT	IFNg release	Positive	1	1	1	1	1	1	1	1	0	1	1	1	1	0	1	1	0	1	0
195888	RGLRTLILAPTRVVA	NS3	1689	1703	HLA-DRB1*08:02	81% - <100%	Dengue virus 2	ELISPOT	IFNg release	Positive	1	1	1	1	1	1	1	1	0	1	1	1	1	0	1	1	0	1	0
21115	GLRTLILAPTRVVAA	NS3	1690	1704	HLA-DRB1*04:03	81% - <100%	Dengue virus 2	ELISPOT	IFNg release	Positive	1	1	1	1	1	1	1	1	0	1	1	1	1	0	1	1	0	1	0
26965	ILAPTRVVAAEMEEA	NS3	1695	1709	HLA-DQA1*03:01/DQB1*03:02	81% - <100%	Dengue virus 2	ELISPOT	IFNg release	Positive	1	1	1	1	1	1	1	1	0	1	1	1	1	0	1	1	0	1	0
30396	KEGERKKLKPRWLDA	NS3	584	598	HLA class II	81% - <100%	Dengue virus 2	ICS	IFNg release	Positive	1	1	1	1	1	1	1	1	0	1	1	1	1	0	1	1	0	1	0
31726	KKVIQLSRKTFDSEY	NS3	1855	1869	HLA-DRB1*12:02	81% - <100%	Dengue virus 2	ELISPOT	IFNg release	Positive	1	1	1	1	1	1	1	1	0	1	1	1	1	0	1	1	0	1	0
34231	KVVGLYGNGVVTRSG	NS3	145	159	HLA class II	81% - <100%	Dengue virus 2	ELISPOT	IFNg release	Positive	1	1	1	1	1	1	1	1	0	1	1	1	1	0	1	1	0	1	0
36560	LIIMDEAHFTDPASI	NS3	1755	1769	HLA-DQA1*01:01/DQB1*05:01	81% - <100%	Dengue virus 2	ELISPOT	IFNg release	Positive	1	1	1	1	1	1	1	1	0	1	1	1	1	0	1	1	0	1	0
39103	LRGLPIRYQTPAIRA	NS3	1696	1710	HLA-DRB1*14:02	81% - <100%	Dengue virus 2	ELISPOT	IFNg release	Positive	1	1	1	1	1	1	1	1	0	1	1	1	1	0	1	1	0	1	0
39225	LRTLILAPTRVVAAE	NS3	1691	1705	HLA-DRB1*12:02	81% - <100%	Dengue virus 2	ELISPOT	IFNg release	Positive	1	1	1	1	1	1	1	1	0	1	1	1	1	0	1	1	0	1	0
4152	ARGYISTRVEMGEAA	NS3	1757	1771	HLA-DRB1*04:07	81% - <100%	Dengue virus 2	ELISPOT	IFNg release	Positive	1	1	1	1	1	1	1	1	0	1	1	1	1	0	1	1	0	1	0
49766	PTRVVAAEMEEALRG	NS3	223	237	HLA-DRB1*15:01	81% - <100%	Dengue virus 2	ICS	IFNg release	Positive	1	1	1	1	1	1	1	1	0	1	1	1	1	0	1	1	0	1	0
539025	AAGIFMTATPPGSRD	NS3	1784	1798	HLA-DRB1*04:03	81% - <100%	Dengue virus 2	ELISPOT	IFNg release	Positive	0	1	1	1	1	1	1	1	0	1	1	1	1	0	1	1	0	1	0
539041	AEMEEALRGLPIRYQ	NS3	1704	1718	HLA-DRB1*13:01	81% - <100%	Dengue virus 2	ELISPOT	IFNg release	Positive	1	1	1	1	1	1	1	1	0	1	1	1	1	0	1	1	0	1	0
539203	EERVILAGPMPVTHS	NS3	1899	1913	HLA-DRB1*09:01	81% - <100%	Dengue virus 2	ELISPOT	IFNg release	Positive	1	1	1	1	1	1	1	1	0	1	1	1	1	0	1	1	0	1	0
539289	GEAAGIFMTATPPGS	NS3	1782	1796	HLA-DRB1*04:03	81% - <100%	Dengue virus 2	ELISPOT	IFNg release	Positive	0	1	1	1	1	1	1	1	0	1	1	1	1	0	1	1	0	1	0
539448	IVREAIKRGLRTLIL	NS3	1682	1696	HLA-DRB1*12:02	81% - <100%	Dengue virus 2	ELISPOT	IFNg release	Positive	1	1	1	1	1	1	1	1	0	1	1	1	1	0	1	1	0	1	0
539467	KGKTVWFVPSIKAGN	NS3	1830	1844	HLA-DRB1*08:03	81% - <100%	Dengue virus 2	ELISPOT	IFNg release	Positive	1	1	1	1	1	1	1	1	0	1	1	1	1	0	1	1	0	1	0
539573	LILAPTRVVAAEMEE	NS3	1694	1708	HLA-DRB1*13:01	81% - <100%	Dengue virus 2	ELISPOT	IFNg release	Positive	1	1	1	1	1	1	1	1	0	1	1	1	1	0	1	1	0	1	0
539713	NGKKVIQLSRKTFDS	NS3	1853	1867	HLA-DRB1*12:02	81% - <100%	Dengue virus 2	ELISPOT	IFNg release	Positive	1	1	1	1	1	1	1	1	0	1	1	1	1	0	1	1	0	1	0
539850	RGLPIRYQTPAIRAE	NS3	1711	1725	HLA-DRB1*13:01	81% - <100%	Dengue virus 2	ELISPOT	IFNg release	Positive	1	1	1	1	1	1	1	1	0	1	1	1	1	0	1	1	0	1	0
539857	RKNGKKVIQLSRKTF	NS3	1851	1865	HLA-DRB1*08:03	81% - <100%	Dengue virus 2	ELISPOT	IFNg release	Positive	1	1	1	1	1	1	1	1	0	1	1	1	1	0	1	1	0	1	0
540056	VLMHKGKRIEPSWAD	NS3	1518	1532	HLA-DRB1*13:01	81% - <100%	Dengue virus 2	ELISPOT	IFNg release	Positive	1	1	1	1	1	1	1	1	0	1	1	1	1	0	1	1	0	1	0
63672	TFHTMWHVTRGAVLM	NS3	45	59	HLA class II	81% - <100%	Dengue virus 2	ELISPOT	IFNg release	Positive	1	1	1	1	1	1	1	1	0	1	1	1	1	0	1	1	0	1	0
64666	TKRYLPAIVREAIKR	NS3	200	214	HLA class II	81% - <100%	Dengue virus 2	ELISPOT	IFNg release	Positive	1	1	0	1	1	1	1	1	0	1	1	1	1	0	1	1	0	1	0
740081	REAIKRGLRTLILAP	NS3	1670	1684	HLA-DRB1*14:06	81% - <100%	Dengue virus 2	ELISPOT	IFNg release	Positive	1	1	1	1	1	1	1	1	0	1	1	1	1	0	1	1	0	1	0
740193	RTLILAPTRVVAAEM	NS3	1678	1692	HLA-DRB1*14:06	81% - <100%	Dengue virus 2	ELISPOT	IFNg release	Positive	1	1	1	1	1	1	1	1	0	1	1	1	1	0	1	1	0	1	0
866477	EALRGLPIRYQTPAI	NS3	1708	1722	HLA-DRB4*01:01	81% - <100%	Dengue virus 2	ELISPOT	IFNg release	Positive	1	1	1	1	1	1	1	1	0	1	1	1	1	0	1	1	0	1	0
868724	RTNDWDFVVTTDISE	NS3	1873	1887	HLA-DQA1*03:01/DQB1*03:02	81% - <100%	Dengue virus 2	ELISPOT	IFNg release	Positive	1	1	1	1	1	1	1	1	0	1	1	1	1	0	1	1	0	1	0
868734	RYLPAIVREAIKRGL	NS3	1677	1691	HLA-DRB4*01:01	81% - <100%	Dengue virus 2	ELISPOT	IFNg release	Positive	1	1	0	1	1	1	1	1	0	1	1	1	1	0	1	1	0	1	0
869224	TLILAPTRVVAAEME	NS3	1693	1707	HLA-DQA1*01:02/DQB1*06:02	81% - <100%	Dengue virus 2	ELISPOT	IFNg release	Positive	1	1	1	1	1	1	1	1	0	1	1	1	1	0	1	1	0	1	0
8911	DKKGKVVGLYGNGVV	NS3	141	155	HLA-DRB1*15:01	81% - <100%	Dengue virus 2	ICS	IFNg release	Positive	1	1	1	1	1	1	1	1	0	1	1	1	1	0	1	1	0	1	0
150362	LETLLLLTLLATVTG	NS4a	2145	2159	HLA-DRB1*01:01	81% - <100%	Dengue virus 2	ELISPOT	IFNg release	Positive	1	1	1	1	1	1	1	1	0	1	1	1	1	0	1	1	0	1	0
195107	AASIILEFFLIVLLI	NS4a	2185	2199	HLA-DRB1*15:01	81% - <100%	Dengue virus 2	ELISPOT	IFNg release	Positive	1	1	1	1	1	1	1	1	0	1	1	1	1	0	1	1	0	1	0
195414	GRAYNHALSELPETL	NS4a	2131	2145	HLA-DRB1*04:01	81% - <100%	Dengue virus 2	ELISPOT	IFNg release	Positive	1	1	1	1	1	1	1	1	0	1	1	1	1	0	1	1	0	1	0
196054	TLETLLLLTLLATVT	NS4a	2144	2158	HLA-DRB1*15:01	81% - <100%	Dengue virus 2	ELISPOT	IFNg release	Positive	1	1	1	1	1	1	1	1	0	1	1	1	1	0	1	1	0	1	0
539801	QDNQLTYVVIAILTV	NS4a	2223	2237	HLA-DRB1*15:02	81% - <100%	Dengue virus 2	ELISPOT	IFNg release	Positive	1	1	1	1	1	1	1	1	0	1	1	1	1	0	1	1	0	1	0
539804	QIQPHWIAASIILEF	NS4a	2192	2206	HLA-DRB1*13:01	81% - <100%	Dengue virus 2	ELISPOT	IFNg release	Positive	1	1	1	1	1	1	1	1	0	1	1	1	1	0	1	1	0	1	0
540148	YAQIQPHWIAASIIL	NS4a	2176	2190	HLA-DRB1*13:01	81% - <100%	Dengue virus 2	ELISPOT	IFNg release	Positive	1	1	1	1	1	1	1	1	0	1	1	1	1	0	1	1	0	1	0
738567	AYNHALSELPETLET	NS4a	2119	2133	HLA-DRB1*04:07	81% - <100%	Dengue virus 2	ELISPOT	IFNg release	Positive	1	1	1	1	1	1	1	1	0	1	1	1	1	0	1	1	0	1	0
195621	LCVTQVLMMRTTWAL	NS4b	2405	2419	HLA-DRB1*07:01	81% - <100%	Dengue virus 2	ELISPOT	IFNg release	Positive	1	1	1	1	1	1	1	1	0	1	1	1	1	0	1	1	0	1	0
195827	PESNILDIDLRPASA	NS4b	2266	2280	HLA-DRB1*03:01	81% - <100%	Dengue virus 2	ELISPOT	IFNg release	Positive	1	1	1	1	1	1	1	1	0	1	1	1	1	0	1	1	0	1	0
195882	RFWNTTIAVSMANIF	NS4b	2454	2468	HLA-DRB1*07:01	81% - <100%	Dengue virus 2	ELISPOT	IFNg release	Positive	1	1	1	1	1	1	1	1	0	1	1	1	1	0	1	1	0	1	0
195974	SNILDIDLRPASAWT	NS4b	2155	2169	HLA-DRB1*03:01	81% - <100%	Dengue virus 2	ELISPOT	IFNg release	Positive	1	1	1	1	1	1	1	1	0	1	1	1	1	0	1	1	0	1	0
539724	NIFRGSYLAGAGLLF	NS4b	2452	2466	HLA-DRB1*09:01	81% - <100%	Dengue virus 2	ELISPOT	IFNg release	Positive	1	1	1	1	1	1	1	1	0	1	1	1	1	0	1	1	0	1	0
739437	LDIDLRPASAWTLYA	NS4b	2257	2271	HLA-DRB1*01:02	81% - <100%	Dengue virus 2	ELISPOT	IFNg release	Positive	1	1	1	1	1	1	1	1	0	1	1	1	1	0	1	1	0	1	0
869089	TAIANQATVLMGLGK	NS4b	2309	2323	HLA-DQA1*01:02/DQB1*06:02	81% - <100%	Dengue virus 2	ELISPOT	IFNg release	Positive	1	1	1	1	1	1	1	1	0	1	1	1	1	0	1	1	0	1	0
1066545	ACLGKSYAQMWSLMY	NS5	3243	3257	HLA-DRB1*04:01	81% - <100%	Dengue virus 2	ELISPOT	IFNg release	Positive-Low	1	1	1	1	1	1	1	1	0	1	1	1	1	0	1	1	0	1	0
180579	KSYAQMWSLMYFHRR	NS5	3247	3261	HLA-DRB5*01:01	81% - <100%	Dengue virus 2	ELISPOT	IFNg release	Positive	1	1	1	1	1	1	1	1	0	1	1	1	1	0	1	1	0	1	0
195200	CETCVYNMMGKREKK	NS5	2924	2938	HLA-DRB1*11:01	81% - <100%	Dengue virus 2	ELISPOT	IFNg release	Positive	1	1	1	1	1	1	1	1	0	1	1	1	1	0	1	1	0	1	0
195213	DELIGRARISQGAGW	NS5	3223	3237	HLA-DRB1*08:02	81% - <100%	Dengue virus 2	ELISPOT	IFNg release	Positive	1	1	1	1	1	1	1	1	0	1	1	1	1	0	1	1	0	1	0
195251	ELIMKDGRVLVVPCR	NS5	3206	3220	HLA-DRB1*03:01	81% - <100%	Dengue virus 2	ELISPOT	IFNg release	Positive	1	1	1	1	1	1	1	1	0	1	1	1	1	0	1	1	0	1	0
195380	GKSYAQMWSLMYFHR	NS5	3232	3246	HLA-DRB1*04:01	81% - <100%	Dengue virus 2	ELISPOT	IFNg release	Positive	1	1	1	1	1	1	1	1	0	1	1	1	1	0	1	1	0	1	0
195409	GNIVSSVNMISRMLI	NS5	2717	2731	HLA-DRB1*07:01	81% - <100%	Dengue virus 2	ELISPOT	IFNg release	Positive	1	1	1	1	1	1	1	1	0	1	1	1	1	0	1	1	0	1	0
195428	GTVMDIISRRDQRGS	NS5	3078	3092	HLA-DRB1*03:01	81% - <100%	Dengue virus 2	ELISPOT	IFNg release	Positive	1	1	1	1	1	1	1	1	0	1	1	1	1	0	1	1	0	1	0
195640	LGYILRDVSKKEGGA	NS5	2992	3006	HLA-DRB1*03:01	81% - <100%	Dengue virus 2	ELISPOT	IFNg release	Positive	1	1	1	1	1	0	1	1	0	1	1	1	1	0	1	1	0	1	0
195801	NMISRMLINRFTMRH	NS5	6	20	HLA-DRB1*08:02	81% - <100%	Dengue virus 2	ELISPOT	IFNg release	Positive	1	1	1	1	0	1	1	1	0	1	1	1	1	0	1	1	0	0	0
195948	SGNIVSSVNMISRML	NS5	2716	2730	HLA-DRB1*04:01	81% - <100%	Dengue virus 2	ELISPOT	IFNg release	Positive	1	1	1	1	1	1	1	1	0	1	1	1	1	0	1	1	0	1	0
196161	VNMISRMLINRFTMR	NS5	2723	2737	HLA-DRB1*10:01	81% - <100%	Dengue virus 2	ELISPOT	IFNg release	Positive	1	1	1	1	1	1	1	1	0	1	1	1	1	0	1	1	0	1	0
196187	VSSVNMISRMLINRF	NS5	2720	2734	HLA-DRB1*15:01	81% - <100%	Dengue virus 2	ELISPOT	IFNg release	Positive	1	1	1	1	1	1	1	1	0	1	1	1	1	0	1	1	0	1	0
196223	WSLMYFHRRDLRLAA	NS5	3253	3267	HLA-DRB1*03:01	81% - <100%	Dengue virus 2	ELISPOT	IFNg release	Positive	1	1	1	1	1	1	1	1	0	1	1	1	1	0	1	1	0	1	0
196243	YQNKVVRVQRPTPRG	NS5	3064	3078	HLA-DRB1*08:02	81% - <100%	Dengue virus 2	ELISPOT	IFNg release	Positive	1	1	1	1	1	1	1	1	0	1	1	1	1	0	1	1	0	1	0
539436	ISGDDCVVKPLDDRFA	NS5	3151	3166	HLA class II	81% - <100%	Dengue virus 2	ICS	IFNg release	Positive	1	1	1	1	1	1	1	1	0	1	1	1	1	0	1	1	0	1	0
539438	ISRMLINRFTMRHKK	NS5	8	22	HLA-DRB1*14:02	81% - <100%	Dengue virus 2	ELISPOT	IFNg release	Positive	1	1	1	1	0	1	1	1	0	1	1	1	1	0	1	1	0	0	0
539669	MISRMLINRFTMRHK	NS5	7	21	HLA-DRB1*14:06	81% - <100%	Dengue virus 2	ELISPOT	IFNg release	Positive	1	1	1	1	0	1	1	1	0	1	1	1	1	0	1	1	0	0	0
738754	EEFTRKVRSNAALGA	NS5	2874	2888	HLA-DRB1*11:04	81% - <100%	Dengue virus 2	ELISPOT	IFNg release	Positive	1	1	1	1	1	1	1	1	0	1	1	1	1	0	1	1	0	1	0
740675	VRSNAALGAIFTDEN	NS5	2880	2894	HLA-DRB1*11:04	81% - <100%	Dengue virus 2	ELISPOT	IFNg release	Positive	1	1	1	1	1	1	1	1	0	1	1	1	1	0	1	1	0	1	0
866172	ASGNIVSSVNMISRM	NS5	2715	2729	HLA-DRB3*02:02	81% - <100%	Dengue virus 2	ELISPOT	IFNg release	Positive	1	1	1	1	1	1	1	1	0	1	1	1	1	0	1	1	0	1	0
866648	ETCVYNMMGKREKKL	NS5	2939	2953	HLA-DRB5*01:01	81% - <100%	Dengue virus 2	ELISPOT	IFNg release	Positive	1	1	1	1	1	1	1	1	0	1	1	1	1	0	1	1	0	1	0
867514	KTPVESWEEIPYLGK	NS5	3318	3332	HLA-DQA1*01:01/DQB1*05:01	81% - <100%	Dengue virus 2	ELISPOT	IFNg release	Positive	1	1	1	1	1	1	1	1	0	1	1	1	1	0	1	1	0	1	0
867600	LEFEALGFLNEDHWF	NS5	2975	2989	HLA-DPB1*04:01	81% - <100%	Dengue virus 2	ELISPOT	IFNg release	Positive	1	1	1	1	1	1	1	1	0	1	1	1	1	0	1	1	0	1	0
868640	RFLEFEALGFLNEDH	NS5	2973	2987	HLA-DPB1*01:01	81% - <100%	Dengue virus 2	ELISPOT	IFNg release	Positive	1	1	1	1	1	1	1	1	0	1	1	1	1	0	1	1	0	1	0
868668	RKVRSNAALGAIFTD	NS5	2892	2906	HLA-DRB3*02:02	81% - <100%	Dengue virus 2	ELISPOT	IFNg release	Positive	1	1	1	1	1	1	1	1	0	1	1	1	1	0	1	1	0	1	0
869082	SYAQMWSLMYFHRRD	NS5	3248	3262	HLA-DPB1*04:01	81% - <100%	Dengue virus 2	ELISPOT	IFNg release	Positive	1	1	1	1	1	1	1	1	0	1	1	1	1	0	1	1	0	1	0
869307	TRKVRSNAALGAIFT	NS5	2891	2905	HLA-DRB3*02:02	81% - <100%	Dengue virus 2	ELISPOT	IFNg release	Positive	1	1	1	1	1	1	1	1	0	1	1	1	1	0	1	1	0	1	0

869426	VESWEEIPYLGKRED	NS5	3321	3335	HLA-DQA1*01:01/DQB1*05:01	81% - <100%	Dengue virus 2	ELISPOT	IFNg release	Positive	1	1	1	1	1	1	1	1	0	1	1	1	1	0	1	1	0	1	0
150105	AFLRFLTIPPTAGIL	C	52	66	HLA-DRB1*01:01	51%-80%	Dengue virus 2	ELISPOT	IFNg release	Positive	0	1	1	1	1	1	1	1	0	1	0	1	1	0	1	1	0	1	0
150247	GMIIMLIPTVMAFHL	C	103	117	HLA-DRB1*01:01	51%-80%	Dengue virus 2	ELISPOT	IFNg release	Positive	1	0	1	0	1	0	1	1	0	1	0	1	0	0	1	1	0	1	0
180597	LFMALVAFLRFLTIP	C	46	60	HLA class II	51%-80%	Dengue virus 2	ELISPOT	IFNg release	Positive	0	1	1	1	1	1	1	1	0	1	0	1	1	0	1	1	0	1	0
195120	AGMIIMLIPTVMAFH	C	102	116	HLA-DRB1*08:02	51%-80%	Dengue virus 2	ELISPOT	IFNg release	Positive	1	0	1	0	1	0	1	1	0	1	0	1	0	0	1	1	0	1	0
195307	FMALVAFLRFLTIPP	C	47	61	HLA-DRB1*11:01	51%-80%	Dengue virus 2	ELISPOT	IFNg release	Positive	0	1	1	1	1	1	1	1	0	1	0	1	1	0	1	1	0	1	0
195496	IIMLIPTVMAFHLTT	C	105	119	HLA-DRB1*01:02	51%-80%	Dengue virus 2	ELISPOT	IFNg release	Positive	1	0	1	0	1	0	1	1	0	1	0	1	0	0	1	1	0	1	0
195730	LVAFLRFLTIPPTAG	C	50	64	HLA-DRB1*04:01	51%-80%	Dengue virus 2	ELISPOT	IFNg release	Positive	0	1	1	1	1	1	1	1	0	1	0	1	1	0	1	1	0	1	0
195744	MALVAFLRFLTIPPT	C	48	62	HLA-DRB1*04:01	51%-80%	Dengue virus 2	ELISPOT	IFNg release	Positive	0	1	1	1	1	1	1	1	0	1	0	1	1	0	1	1	0	1	0
196093	VAFLRFLTIPPTAGI	C	51	65	HLA-DRB1*04:01	51%-80%	Dengue virus 2	ELISPOT	IFNg release	Positive	0	1	1	1	1	1	1	1	0	1	0	1	1	0	1	1	0	1	0
539083	ALVAFLRFLTIPPTA	C	49	63	HLA-DRB1*12:02	51%-80%	Dengue virus 2	ELISPOT	IFNg release	Positive	0	1	1	1	1	1	1	1	0	1	0	1	1	0	1	1	0	1	0
150490	PGFTIMAAILAYTIG	PreM	245	259	HLA-DRB1*01:01	51%-80%	Dengue virus 2	ELISPOT	IFNg release	Positive	1	1	1	1	0	1	1	1	0	0	1	1	1	1	1	1	1	0	1
195322	FQRVLIFILLTAVAP	PreM	263	277	HLA-DRB1*07:01	51%-80%	Dengue virus 2	ELISPOT	IFNg release	Positive	1	1	1	1	0	0	1	1	0	1	1	1	1	0	0	0	0	0	0
195702	LRHPGFTIMAAILAY	PreM	242	256	HLA-DRB1*11:01	51%-80%	Dengue virus 2	ELISPOT	IFNg release	Positive	1	1	1	1	0	1	1	1	0	0	1	1	1	1	1	1	1	0	1
195863	QRVLIFILLTAVAPS	PreM	264	278	HLA-DRB1*08:02	51%-80%	Dengue virus 2	ELISPOT	IFNg release	Positive	1	1	1	1	0	0	1	1	0	1	1	1	1	0	0	0	0	0	0
195892	RIETWILRHPGFTIM	PreM	122	136	HLA-DRB1*15:01	51%-80%	Dengue virus 2	ELISPOT	IFNg release	Positive	1	0	1	0	0	1	1	1	0	1	0	1	0	1	1	1	0	0	1
180590	LDFELIKTEAKQPAT	E	41	55	HLA class II	51%-80%	Dengue virus 2	ELISPOT	IFNg release	Positive	1	0	1	0	1	0	1	1	0	1	0	1	0	1	1	1	1	1	1
195952	SGVSWTMKILIGVII	E	449	463	HLA-DRB1*15:01	51%-80%	Dengue virus 2	ELISPOT	IFNg release	Positive	1	0	1	0	1	0	1	1	0	1	0	1	0	0	1	0	1	1	0
196022	SWTMKILIGVIITWI	E	452	466	HLA-DRB1*08:02	51%-80%	Dengue virus 2	ELISPOT	IFNg release	Positive	1	0	1	0	1	0	1	1	0	1	0	1	0	0	1	0	1	1	0
180603	LKYSWKTWGKAKMLS	NS1	111	125	HLA class II	51%-80%	Dengue virus 2	ELISPOT	IFNg release	Positive	1	0	1	0	1	0	1	1	0	1	0	1	0	0	1	1	0	1	0
195207	CTILAVVSVSPLLLT	NS2a	1144	1158	HLA-DRB1*07:01	51%-80%	Dengue virus 2	ELISPOT	IFNg release	Positive	1	0	1	0	1	0	1	1	0	1	0	1	0	0	1	0	0	1	0
195650	LKIVRNMEKYQLAVT	NS2a	1261	1275	HLA-DRB1*13:01	51%-80%	Dengue virus 2	ELISPOT	IFNg release	Positive	1	1	0	1	0	1	1	1	0	1	1	1	1	0	0	0	0	0	0
195752	MEKYQLAVTIMAILC	NS2a	1154	1168	HLA-DRB1*04:07	51%-80%	Dengue virus 2	ELISPOT	IFNg release	Positive	1	1	0	1	1	1	0	1	0	1	1	1	1	0	1	1	0	1	0
195798	NMEKYQLAVTIMAIL	NS2a	1266	1280	HLA-DRB1*04:01	51%-80%	Dengue virus 2	ELISPOT	IFNg release	Positive	1	1	0	1	1	1	0	1	0	1	1	1	1	0	1	0	0	1	0
196174	VRNMEKYQLAVTIMA	NS2a	1264	1278	HLA-DRB1*13:01	51%-80%	Dengue virus 2	ELISPOT	IFNg release	Positive	1	1	0	1	1	1	0	1	0	1	1	1	1	0	1	0	0	1	0
196218	WKVSCTILAVVSVSP	NS2a	1292	1306	HLA-DRB1*04:01	51%-80%	Dengue virus 2	ELISPOT	IFNg release	Positive	1	0	1	0	1	0	1	1	0	1	0	1	0	0	1	1	0	1	0
539474	KIVRNMEKYQLAVTI	NS2a	1262	1276	HLA-DRB1*13:01	51%-80%	Dengue virus 2	ELISPOT	IFNg release	Positive	1	1	0	1	0	1	1	1	0	1	1	1	1	0	0	0	0	0	0
867216	ILCVPNAVILQNAWK	NS2a	1279	1293	HLA-DQA1*01:02/DQB1*06:02	51%-80%	Dengue virus 2	ELISPOT	IFNg release	Positive	0	1	0	1	1	0	1	0	0	1	1	0	1	0	1	1	0	1	0
867571	LCVPNAVILQNAWKV	NS2a	1280	1294	HLA-DRB4*01:01	51%-80%	Dengue virus 2	ELISPOT	IFNg release	Positive	0	1	0	1	1	0	1	0	0	1	1	0	1	0	1	1	0	1	0
150433	MAVGMVSILASSLLK	NS2b	1354	1368	HLA-DRB1*01:01	51%-80%	Dengue virus 2	ELISPOT	IFNg release	Positive	1	0	1	0	1	1	1	1	0	1	0	1	0	0	1	1	0	1	0
167732	DQAEISGSSPILSITISEDG	NS2b	63	82	HLA-DP	51%-80%	Dengue virus 2	ELISPOT	IFNg release	Positive	1	0	1	0	1	0	1	1	0	1	0	1	0	0	1	1	0	1	0
195512	IMAVGMVSILASSLL	NS2b	8	22	HLA-DRB1*07:01	51%-80%	Dengue virus 2	ELISPOT	IFNg release	Positive	1	0	1	0	1	1	1	1	0	1	0	1	0	0	1	1	0	1	0
195992	SSLLKNDIPMTGPLV	NS2b	19	33	HLA-DRB1*03:01	51%-80%	Dengue virus 2	ELISPOT	IFNg release	Positive	1	0	1	0	1	1	1	1	0	1	0	1	0	0	1	1	0	1	0
866986	GMVSILASSLLKNDI	NS2b	1357	1371	HLA-DPB1*01:01	51%-80%	Dengue virus 2	ELISPOT	IFNg release	Positive	1	0	1	0	1	1	1	1	0	1	0	1	0	0	1	1	0	1	0
195765	MRLLSPVRVPNYNLI	NS3	1742	1756	HLA-DRB1*13:01	51%-80%	Dengue virus 2	ELISPOT	IFNg release	Positive	1	0	1	0	1	0	1	1	0	1	0	1	0	0	1	1	0	1	0
739148	IDKKGKVVGLYGNGV	NS3	1615	1629	HLA-DRB1*01:02	51%-80%	Dengue virus 2	ELISPOT	IFNg release	Positive	1	0	1	0	1	0	1	1	0	1	0	1	0	0	1	1	0	1	0
195635	LGMCCIITASILLWY	NS4a	2176	2190	HLA-DRB1*07:01	51%-80%	Dengue virus 2	ELISPOT	IFNg release	Positive	1	0	1	0	1	1	1	1	0	1	0	1	0	0	1	1	0	1	0
195767	MTLGMCCIITASILL	NS4a	2160	2174	HLA-DRB1*07:01	51%-80%	Dengue virus 2	ELISPOT	IFNg release	Positive	1	0	1	0	1	1	1	1	0	1	0	1	0	0	1	1	0	1	0
150611	TAALLLLVAHYAIIG	NS4b	2351	2365	HLA-DRB1*01:01	51%-80%	Dengue virus 2	ELISPOT	IFNg release	Positive	0	1	1	1	0	1	1	1	0	0	1	1	1	0	1	0	0	0	0
150636	TLTAALLLLVAHYAI	NS4b	2335	2349	HLA-DRB1*01:01	51%-80%	Dengue virus 2	ELISPOT	IFNg release	Positive	0	1	1	1	0	1	1	1	0	0	1	1	1	0	1	0	0	0	0
195115	AGAGLLFSIMKNTTN	NS4b	2474	2488	HLA-DRB1*11:01	51%-80%	Dengue virus 2	ELISPOT	IFNg release	Positive	1	0	1	0	1	0	1	1	0	1	0	1	0	0	1	1	0	1	0
195347	GAGLLFSIMKNTTNT	NS4b	2475	2489	HLA-DRB1*11:01	51%-80%	Dengue virus 2	ELISPOT	IFNg release	Positive	1	0	1	0	1	0	1	1	0	1	0	1	0	0	1	1	0	1	0
195629	LFSIMKNTTNTRRGT	NS4b	2479	2493	HLA-DRB1*03:01	51%-80%	Dengue virus 2	ELISPOT	IFNg release	Positive	1	0	1	0	1	0	1	1	0	1	0	1	0	0	1	1	0	1	0
195658	LLFSIMKNTTNTRRG	NS4b	2478	2492	HLA-DRB1*04:01	51%-80%	Dengue virus 2	ELISPOT	IFNg release	Positive	1	0	1	0	1	0	1	1	0	1	0	1	0	0	1	1	0	1	0
195980	SQVNPITLTAALLLL	NS4b	2343	2357	HLA-DQA1*01:02/DQB1*06:02	51%-80%	Dengue virus 2	ELISPOT	IFNg release	Positive	0	1	1	1	0	1	1	1	0	0	1	1	1	0	1	0	0	0	0
539327	GLLFSIMKNTTNTRR	NS4b	2477	2491	HLA-DRB1*14:02	51%-80%	Dengue virus 2	ELISPOT	IFNg release	Positive	1	0	1	0	1	0	1	1	0	1	0	1	0	0	1	1	0	1	0
539755	PASAWTLYAVATTFV	NS4b	2277	2291	HLA-DRB1*09:01	51%-80%	Dengue virus 2	ELISPOT	IFNg release	Positive	1	0	1	0	1	0	1	1	0	1	0	1	0	0	1	1	0	1	0
180582	KTWAYHGSYETKQTG	NS5	2792	2806	HLA class II	51%-80%	Dengue virus 2	ICS	IFNg release	Positive	0	1	0	1	1	1	1	0	0	0	1	0	1	0	1	1	0	1	0
195642	LHKLGYILRDVSKKE	NS5	3003	3017	HLA-DRB1*08:02	51%-80%	Dengue virus 2	ELISPOT	IFNg release	Positive	1	1	1	1	1	0	1	1	0	0	1	1	1	0	1	1	0	1	0
539195	EAIFKLTYQNKVVRV	NS5	3057	3071	HLA-DRB1*09:01	51%-80%	Dengue virus 2	ELISPOT	IFNg release	Positive	1	0	1	0	1	1	1	1	0	1	0	1	0	0	1	1	0	1	0
539348	GSAKLRWFVERNMVT	NS5	2549	2563	HLA-DRB1*09:01	51%-80%	Dengue virus 2	ELISPOT	IFNg release	Positive	1	0	1	0	1	0	1	1	0	1	0	1	0	0	1	1	0	1	0
739166	IFKLTYQNKVVRVQR	NS5	3045	3059	HLA-DRB1*14:06	51%-80%	Dengue virus 2	ELISPOT	IFNg release	Positive	1	0	1	0	1	1	1	1	1	1	0	1	0	0	1	1	0	1	0
866566	EHKKLAEAIFKLTYQ	NS5	3051	3065	HLA-DPB1*01:01	51%-80%	Dengue virus 2	ELISPOT	IFNg release	Positive	1	0	1	0	1	1	1	1	0	1	0	1	0	0	1	1	0	1	0
867419	KKLAEAIFKLTYQNK	NS5	3053	3067	HLA-DPB1*02:01	51%-80%	Dengue virus 2	ELISPOT	IFNg release	Positive	1	0	1	0	1	1	1	1	0	1	0	1	0	0	1	1	0	1	0
867433	KLAEAIFKLTYQNKV	NS5	3054	3068	HLA-DPB1*02:01	51%-80%	Dengue virus 2	ELISPOT	IFNg release	Positive	1	0	1	0	1	1	1	1	0	1	0	1	0	0	1	1	0	1	0
1066603	ARNTPFNMLKRERNR	C	8	22	HLA-DRB1*11:01	>0% - 50%	Dengue virus 2	ELISPOT	IFNg release	Positive-Low	0	1	0	1	0	1	0	0	0	0	1	0	1	0	1	1	0	0	0
195417	GRMLNILNRRRRTAG	C	89	103	HLA-DRB1*11:01	>0% - 50%	Dengue virus 2	ELISPOT	IFNg release	Positive	0	1	0	1	0	1	0	0	0	0	1	0	1	0	0	1	0	0	0
195489	IGRMLNILNRRRRTA	C	88	102	HLA-DRB1*08:02	>0% - 50%	Dengue virus 2	ELISPOT	IFNg release	Positive	0	1	0	1	0	1	0	0	0	0	1	0	1	0	0	1	0	0	0
539214	EIGRMLNILNRRRRT	C	87	101	HLA-DRB1*12:02	>0% - 50%	Dengue virus 2	ELISPOT	IFNg release	Positive	0	1	0	1	0	1	0	0	0	0	1	0	1	0	0	1	0	0	0
739177	IGRMLNILNRRRRSA	C	88	102	HLA-DRB1*14:02	>0% - 50%	Dengue virus 2	ELISPOT	IFNg release	Positive	0	0	1	0	1	0	1	1	0	1	0	1	0	0	1	0	0	1	0
740399	STPFNMLKRERNRVS	C	10	24	HLA-DRB1*14:02	>0% - 50%	Dengue virus 2	ELISPOT	IFNg release	Positive	1	0	0	0	0	0	0	0	0	0	0	0	0	0	0	0	0	0	0
180708	QRIETWILRHPGFTM	PreM	121	135	HLA class II	>0% - 50%	Dengue virus 2	ELISPOT	IFNg release	Positive-Low	0	0	0	0	1	0	0	0	0	0	0	0	0	0	0	0	0	1	0
195503	ILRHPGFTIMAAILA	PreM	127	141	HLA-DRB1*07:01	>0% - 50%	Dengue virus 2	ELISPOT	IFNg release	Positive	1	0	1	0	0	1	1	1	0	0	0	1	0	1	1	1	1	0	1
196045	THFQRALIFILLTAV	PreM	261	275	HLA-DRB1*07:01	>0% - 50%	Dengue virus 2	ELISPOT	IFNg release	Positive	0	0	0	0	1	0	0	0	0	0	0	0	0	1	1	1	1	1	1
196232	YFQRVLIFILLTAVT	PreM	262	276	HLA-DRB1*07:01	>0% - 50%	Dengue virus 2	ELISPOT	IFNg release	Positive	0	0	0	0	0	1	0	0	0	0	0	0	0	0	0	0	0	0	0
540130	WILRHPGFTIMAAIL	PreM	126	140	HLA-DRB1*15:06	>0% - 50%	Dengue virus 2	ELISPOT	IFNg release	Positive	1	0	1	0	0	1	1	1	0	0	0	1	0	1	1	1	1	0	1
739425	LAYTIGTTHFQRALI	PreM	254	268	HLA-DRB1*04:07	>0% - 50%	Dengue virus 2	ELISPOT	IFNg release	Positive	0	0	0	0	1	0	0	0	0	0	0	0	0	1	1	1	1	1	1
144940	RHVLGRLITVNPIVT	E	345	359	HLA-DRB1*08:02	>0% - 50%	Dengue virus 2	ELISPOT	IFNg release	Positive	0	1	0	1	1	1	0	0	0	0	1	0	0	1	1	1	1	1	1
195716	LSVSLVLVGVVTLYL	E	755	769	HLA-DRB1*15:01	>0% - 50%	Dengue virus 2	ELISPOT	IFNg release	Positive	0	1	0	1	0	1	0	0	0	0	1	0	1	1	0	1	1	0	0
196077	TSLSVSLVLVGVVTL	E	753	767	HLA-DRB1*07:01	>0% - 50%	Dengue virus 2	ELISPOT	IFNg release	Positive	0	1	0	1	0	1	0	0	0	0	1	0	1	1	0	1	1	0	0
539236	ENLEYTIVITPHSGE	E	133	147	HLA-DRB1*04:07	>0% - 50%	Dengue virus 2	ELISPOT	IFNg release	Positive	0	1	0	1	1	1	0	0	0	0	1	0	1	1	0	1	0	1	1
539733	NLEYTIVITPHSGEE	E	134	148	HLA-DRB1*04:07	>0% - 50%	Dengue virus 2	ELISPOT	IFNg release	Positive	0	1	0	1	1	1	0	0	0	0	1	0	1	1	0	1	0	1	1
738866	EYTIVITPHSGEEHA	E	136	150	HLA-DRB1*04:07	>0% - 50%	Dengue virus 2	ELISPOT	IFNg release	Positive	0	0	0	0	1	0	0	0	0	0	0	0	0	1	0	1	0	1	1
740315	SLSVSLVLVGVVTLY	E	754	768	HLA-DRB1*01:02	>0% - 50%	Dengue virus 2	ELISPOT	IFNg release	Positive	0	1	0	1	0	1	0	0	0	0	1	0	1	1	0	1	1	0	0
195458	HNQTFLIDGPETAEC	NS1	904	918	HLA-DRB1*04:01	>0% - 50%	Dengue virus 2	ELISPOT	IFNg release	Positive	0	0	0	0	1	0	1	0	0	0	0	0	0	0	1	1	0	1	0
195845	QDVFCDSKLMSAAIK	NS1	175	189	HLA-DRB1*03:01	>0% - 50%	Dengue virus 2	ELISPOT	IFNg release	Positive	0	0	0	1	1	1	0	0	0	0	0	0	0	0	0	1	0	1	0
195954	SHNQTFLIDGPETAE	NS1	903	917	HLA-DRB1*04:01	>0% - 50%	Dengue virus 2	ELISPOT	IFNg release	Positive	0	0	0	0	1	0	1	0	0	0	0	0	0	0	1	1	0	1	0
195688	LNPTAIFLTTLSRTS	NS2a	1328	1342	HLA-DRB1*07:01	>0% - 50%	Dengue virus 2	ELISPOT	IFNg release	Positive	0	1	0	1	1	1	0	0	0	0	1	0	1	0	1	0	0	1	0
539108	AVSFVTLITGNMSFR	NS2a	1164	1178	HLA-DRB1*10:01	>0% - 50%	Dengue virus 2	ELISPOT	IFNg release	Positive	0	1	0	1	1	1	0	0	0	0	1	0	1	0	1	1	0	1	0
539699	MVLKMVRNMEKYQLA	NS2a	1259	1273	HLA-DRB1*13:01	>0% - 50%	Dengue virus 2	ELISPOT	IFNg release	Positive	0	0	0	0	1	0	0	0	0	0	0	0	0	0	1	0	0	1	0
539789	PTAIFLTTLSRTSKK	NS2a	1316	1330	HLA-DRB1*04:03	>0% - 50%	Dengue virus 2	ELISPOT	IFNg release	Positive	0	1	0	1	1	1	0	0	0	0	1	0	1	0	1	0	0	1	0
539959	TAIFLTTLSRTSKKR	NS2a	1317	1331	HLA-DRB1*14:02	>0% - 50%	Dengue virus 2	ELISPOT	IFNg release	Positive	0	1	0	1	1	1	0	0	0	0	1	0	1	0	1	0	0	1	0
540086	VSFVTLITGNMSFRD	NS2a	1165	1179	HLA-DRB1*10:01	>0% - 50%	Dengue virus 2	ELISPOT	IFNg release	Positive	0	1	0	1	1	1	0	0	0	0	1	0	1	0	1	1	0	1	0
539200	EDDIFRKKRLTIMDL	NS3	1654	1668	HLA-DRB1*13:01	>0% - 50%	Dengue virus 2	ELISPOT	IFNg release	Positive	0	1	0	1	0	1	0	0	0	0	1	0	1	0	0	0	0	0	0
167780	LTLNLITEMGRLPTFMTQKA	NS4a	2	21	HLA class II	>0% - 50%	Dengue virus 2	ELISPOT	IFNg release	Positive	0	1	0	1	1	1	0	0	0	0	1	0	1	0	1	1	0	1	0
195355	GGIFLFLMSGKGIGK	NS4a	2145	2159	HLA-DRB1*11:01	>0% - 50%	Dengue virus 2	ELISPOT	IFNg release	Positive	0	1	0	1	0	1	0	0	0	0	1	0	1	0	0	0	0	0	0
195356	GGIFLFLMSGRGIGK	NS4a	2159	2173	HLA-DRB1*11:01	>0% - 50%	Dengue virus 2	ELISPOT	IFNg release	Positive	1	0	1	0	1	0	0	1	0	1	0	1	0	0	1	1	0	1	0
196089	TVTGGIFLFLMSGKG	NS4a	2142	2156	HLA-DRB1*11:01	>0% - 50%	Dengue virus 2	ELISPOT	IFNg release	Positive	0	1	0	1	0	1	0	0	0	0	1	0	1	0	0	0	0	0	0
539609	LPTFMTQKARNALDN	NS4a	2092	2106	HLA-DRB1*10:01	>0% - 50%	Dengue virus 2	ELISPOT	IFNg release	Positive	0	1	0	1	0	0	0	0	0	0	1	0	1	0	0	0	0	0	0
195393	GLLFSIMKNTANTRR	NS4b	2463	2477	HLA-DRB1*04:01	>0% - 50%	Dengue virus 2	ELISPOT	IFNg release	Positive	0	1	0	1	0	1	0	0	0	0	1	0	1	0	0	0	0	0	0
195718	LTAALFLLVAHYAII	NS4b	2350	2364	HLA-DRB1*07:01	>0% - 50%	Dengue virus 2	ELISPOT	IFNg release	Positive	1	0	0	0	1	0	0	0	0	0	0	0	0	0	0	1	0	1	0
867709	LILCVTQVLMMRTTW	NS4b	2417	2431	HLA-DPB1*04:02	>0% - 50%	Dengue virus 2	ELISPOT	IFNg release	Positive	1	0	1	0	0	0	1	1	0	1	0	1	0	0	0	0	0	0	0
867860	LLVLCVTQVLMMRTT	NS4b	2416	2430	HLA-DPB1*04:02	>0% - 50%	Dengue virus 2	ELISPOT	IFNg release	Positive	0	1	0	1	1	1	0	0	0	0	1	0	1	0	1	1	0	1	0
167817	SRLNALGKSEFQI	NS5	15	27	HLA class II	>0% - 50%	Dengue virus 2	ELISPOT	IFNg release	Positive	1	0	1	0	1	0	1	1	0	0	0	1	0	0	1	1	0	1	0
190862	MVTQMAMTDTTPFGQQR	NS5	2828	2844	HLA class II	>0% - 50%	Dengue virus 2	ICS	IFNg release	Positive	0	1	0	1	1	1	0	0	0	0	1	0	1	0	1	1	0	1	0
196210	WAKNIQTAINQVRSL	NS5	3336	3350	HLA-DRB1*03:01	>0% - 50%	Dengue virus 2	ELISPOT	IFNg release	Positive	0	1	0	1	0	1	0	0	0	0	1	0	1	0	0	1	0	0	0
32942	KPWDVLPTV	NS5	2821	2829	HLA class II	>0% - 50%	Dengue virus 2	ELISA	IL-2 release	Positive	1	0	1	0	0	0	1	1	0	1	0	1	0	0	0	0	0	0	0
32943	KPWDVVPMV	NS5	2821	2829	HLA class II	>0% - 50%	Dengue virus 2	ELISA	IL-2 release	Positive	0	0	0	0	1	0	0	0	0	0	0	0	0	0	1	1	0	1	0
539144	CVVKPLDDRFASALTAL	NS5	3156	3172	HLA class II	>0% - 50%	Dengue virus 2	ICS	IFNg release	Positive	0	0	0	0	1	1	0	0	0	0	0	0	0	0	1	1	0	1	0

539150	DDRFASALTALNDMG	NS5	3162	3176	HLA-DRB1*04:03	>0% - 50%	Dengue virus 2	ELISPOT	IFNg release	Positive	0	0	0	0	1	1	0	0	0	0	0	0	0	0	1	1	0	1	0
540064	VNGVVRLLTKPWDVI	NS5	2812	2826	HLA-DRB1*12:02	>0% - 50%	Dengue virus 2	ELISPOT	IFNg release	Positive	0	1	0	1	0	1	0	0	0	0	1	0	1	0	0	0	0	0	0
540065	VNGVVRLLTKPWDVV	NS5	2812	2826	HLA-DRB1*12:02	>0% - 50%	Dengue virus 2	ELISPOT	IFNg release	Positive	0	0	0	0	1	0	0	0	0	0	0	0	0	0	1	1	0	1	0
739791	NLVRLQSGVDVFFTP	NS5	2599	2613	HLA-DRB1*01:02	>0% - 50%	Dengue virus 2	ELISPOT	IFNg release	Positive	0	1	0	1	0	1	0	0	0	0	1	0	1	0	0	1	0	0	0

CD4 DENV-3 Epitopes

Epitope_ID	AA_Seq	Protein_Name	Start	End	Allele	step	Serotype	Method/Technique	Assay Group	Qualitative Measure
195517	INMLKRVRNRVSTGP	C	13	27	HLA-DRB1*08:02	0%	Dengue virus 3	ELISPOT	IFNg release	Positive
195519	INMLKRVRNRVSTGT	C	13	27	HLA-DRB1*08:02	0%	Dengue virus 3	ELISPOT	IFNg release	Positive
195598	KTSLCLVMILPAALA	C	100	114	HLA-DRB1*01:01	0%	Dengue virus 3	ELISPOT	IFNg release	Positive
195618	LCLMMIMPAALAFHL	C	103	117	HLA-DRB1*01:01	0%	Dengue virus 3	ELISPOT	IFNg release	Positive
195803	NMLKRVRNRVSTGPQ	C	14	28	HLA-DRB1*11:01	0%	Dengue virus 3	ELISPOT	IFNg release	Positive
195966	SLCLMMIMPAALAFH	C	102	116	HLA-DRB1*08:02	0%	Dengue virus 3	ELISPOT	IFNg release	Positive
539447	IVNRRKKTSLCLMMM	C	94	108	HLA-DRB1*13:01	0%	Dengue virus 3	ELISPOT	IFNg release	Positive
539906	SIINRRKRTSLCLMM	C	93	107	HLA-DRB1*13:01	0%	Dengue virus 3	ELISPOT	IFNg release	Positive
540118	WGSFKKSGAVKVLRG	C	69	83	HLA-DRB1*09:01	0%	Dengue virus 3	ELISPOT	IFNg release	Positive
540119	WGTFKKLGAIKVLKG	C	69	83	HLA-DRB1*09:01	0%	Dengue virus 3	ELISPOT	IFNg release	Positive
540120	WGTFKKLGAIKVLRG	C	69	83	HLA-DRB1*09:01	0%	Dengue virus 3	ELISPOT	IFNg release	Positive
540122	WGTFKKSGAIKVLKS	C	69	83	HLA-DRB1*09:01	0%	Dengue virus 3	ELISPOT	IFNg release	Positive
540123	WGTFKKSGAIKVLRG	C	69	83	HLA-DRB1*09:01	0%	Dengue virus 3	ELISPOT	IFNg release	Positive
540124	WGTFKKSGAIRVLRG	C	69	83	HLA-DRB1*09:01	0%	Dengue virus 3	ELISPOT	IFNg release	Positive
591709	LRGFKKEISNMLSII	C	81	95	HLA-DRB1*13:02	0%	Dengue virus 3	ELISPOT	IFNg release	Positive
591723	NMLSIINRRKKTSLC	C	90	104	HLA-DRB1*11:01	0%	Dengue virus 3	ELISPOT	IFNg release	Positive
591746	RGFKKEISNMLSIIN	C	82	96	HLA-DRB1*04:01	0%	Dengue virus 3	ELISPOT	IFNg release	Positive
867744	LKSFKKEISNMLSII	C	81	95	HLA-DRB3*02:02	0%	Dengue virus 3	ELISPOT	IFNg release	Positive
867909	LRGFKREISNMLSII	C	64	78	HLA-DRB3*02:02	0%	Dengue virus 3	ELISPOT	IFNg release	Positive
868733	RWGTFKKSGAIKVLR	C	48	62	HLA-DRB3*02:02	0%	Dengue virus 3	ELISPOT	IFNg release	Positive
195968	SLIQKVVIFILLMLV	PreM	261	275	HLA-DRB1*15:06	0%	Dengue virus 3	ELISPOT	IFNg release	Positive
539529	KVVIFILLILVTPSM	PreM	265	279	HLA-DRB1*15:06	0%	Dengue virus 3	ELISPOT	IFNg release	Positive
539808	QKVVIFTLLMLVTPS	PreM	264	278	HLA-DRB1*15:06	0%	Dengue virus 3	ELISPOT	IFNg release	Positive
539856	RKELLTTFKNAHAKK	E	511	525	HLA-DRB1*04:03	0%	Dengue virus 3	ELISPOT	IFNg release	Positive
591719	NEMILLTMKNRAWMV	E	474	488	HLA-DRB1*11:01	0%	Dengue virus 3	ELISPOT	IFNg release	Positive
867374	KAWIVHRQWFFDLPL	E	482	496	HLA-DQA1*01:01/DQB1*05:01	0%	Dengue virus 3	ELISPOT	IFNg release	Positive
869693	WMVHRQWLFDLPLPW	E	87	101	HLA-DQA1*01:01/DQB1*05:01	0%	Dengue virus 3	ELISPOT	IFNg release	Positive
539070	AKIVTAETRNSSFII	NS1	121	135	HLA-DRB1*13:01	0%	Dengue virus 3	ELISPOT	IFNg release	Positive
539457	KAKIVIAETQNSSFI	NS1	893	907	HLA-DRB1*13:01	0%	Dengue virus 3	ELISPOT	IFNg release	Positive
866698	FGVFTTNIWLKLRDV	NS1	933	947	HLA-DPB1*04:01	0%	Dengue virus 3	ELISPOT	IFNg release	Positive
195125	AGVIFTFVLLLSGQI	NS2a	1159	1173	HLA-DRB1*07:01	0%	Dengue virus 3	ELISPOT	IFNg release	Positive
195128	AGVLFMFVLLLSGQI	NS2a	1159	1173	HLA-DRB1*07:01	0%	Dengue virus 3	ELISPOT	IFNg release	Positive
195133	AHTFIMIGSNASDRM	NS2a	1179	1193	HLA-DRB1*04:01	0%	Dengue virus 3	ELISPOT	IFNg release	Positive
195166	ANTFIMIGSNASDRM	NS2a	1179	1193	HLA-DRB1*04:01	0%	Dengue virus 3	ELISPOT	IFNg release	Positive
[illegible]	[illegible]	[illegible]	[illegible]	[illegible]	[illegible]	[illegible]	[illegible]	[illegible]	[illegible]	[illegible]

NCBI IDs of DENV-3 circulating strains from Thailand, (100% identity=1,green ; else 0, red) / DENV-3 constructs of vaccines

Epitope_ID	BBG31513.1	QTX93239.1	QTX93295.1	QTX93456.1	QTX93571.1	QTX93578.1	QTX93347.1	QTX93386.1	QTX93396.1	QTX93427.1	QTX93468.1	QTX93479.1	QTX93537.1	QTX93597.1	QBA29682.1	QTX93435.1	QTX93561.1	QTX93564.1	BBG31510.1	QTX93258.1	QTX93260.1	QTX93237.1	QTX93241.1	QTX93288.1	QTX93317.1	QTX93321.1	QTX93389.1	QTX93391.1	QTX93470.1	QTX93527.1	QTX93565.1	QTX93594.1	QTX93331.1	QTX93412.1	QTX93547.1	ANC57606.1	QTX93249.1	QTX93252.1	QTX93291.1	QTX93442.1	QTX93493.1	QTX93499.1	QTX93408.1	QTX93410.1	QTX93416.1	QTX93429.1	QTX93518.1	QTX93539.1	QTX93589.1	QTX93602.1	AMA21734.1	QTX93362.1	QTX93251.1	QTX93374.1	QTX93406.1	QTX93451.1	QTX93458.1	QTX93484.1	QTX93495.1	QTX93504.1	QTX93507.1	QTX93524.1	QTX93529.1	QTX93595.1	QTX93603.1	QTX93540.1	QBK46952.1	TV003	TDEN	DPIV	Dengvaxia	DENVax	TVDV
[illegible]	[illegible]	[illegible]	[illegible]	[illegible]	[illegible]	[illegible]	[illegible]	[illegible]	[illegible]	[illegible]	[illegible]	[illegible]	[illegible]	[illegible]	[illegible]	[illegible]	[illegible]	[illegible]	[illegible]	[illegible]	[illegible]	[illegible]	[illegible]	[illegible]	[illegible]	[illegible]	[illegible]	[illegible]	[illegible]	[illegible]	[illegible]	[illegible]	[illegible]	[illegible]	[illegible]	[illegible]	[illegible]	[illegible]	[illegible]	[illegible]	[illegible]	[illegible]	[illegible]	[illegible]	[illegible]	[illegible]	[illegible]	[illegible]	[illegible]	[illegible]	[illegible]	[illegible]	[illegible]	[illegible]	[illegible]	[illegible]	[illegible]	[illegible]	[illegible]	[illegible]	[illegible]	[illegible]	[illegible]	[illegible]	[illegible]	[illegible]	[illegible]	[illegible]	[illegible]	[illegible]	[illegible]	[illegible]

195123	AGVFFTFVLLLSGQI	NS2a	1159	1173	HLA-DRB1*07:01	>0% - 50%	Dengue virus 3	ELISPOT	IFNg release	Positive	0	1	0	0	0	0	0	0	0	1	1	0	0	0	0	1	0	0	0	0	1	0	0	0	1	1	0	0	1	0	0	0	1	0	0	0	0	0	0	1	0	0	0	1	0	1	1	0	0	0	0	0	0	0	0	0	0	0	0	0	0	0	0	0	0	0	0	0	0	0	0	0	0
195429	GVFFTFVLLLSGQIT	NS2a	1160	1174	HLA-DRB1*04:01	>0% - 50%	Dengue virus 3	ELISPOT	IFNg release	Positive	0	1	0	0	0	0	0	0	0	1	1	0	0	0	0	1	0	0	0	0	1	0	0	0	1	1	0	0	1	0	0	0	1	0	0	0	0	0	0	1	0	0	0	1	0	1	1	0	0	0	0	0	0	0	0	0	0	0	0	0	0	0	0	0	0	0	0	0	0	0	0	0	0
195457	HMIAGVFFTFVLLLS	NS2a	1156	1170	HLA-DRB1*11:01	>0% - 50%	Dengue virus 3	ELISPOT	IFNg release	Positive	0	1	0	0	0	0	0	0	0	1	1	0	0	0	0	1	0	0	0	0	1	0	0	0	1	1	0	0	1	0	0	0	1	0	0	0	0	0	0	1	0	0	0	1	0	1	1	0	0	0	0	0	0	0	0	0	0	0	0	0	0	0	0	0	0	0	0	0	0	0	0	0	0
195645	LISLTCSNTIFTLTV	NS2a	1274	1288	HLA-DRB1*07:01	>0% - 50%	Dengue virus 3	ELISPOT	IFNg release	Positive	1	1	0	0	0	0	0	0	0	1	1	0	0	0	0	1	0	0	1	0	1	0	0	0	1	1	0	0	1	0	0	0	0	0	0	0	0	0	0	1	0	1	0	1	0	1	1	0	0	0	0	0	0	0	0	0	0	0	0	0	0	0	0	0	0	0	0	0	0	0	0	0	0
195836	PLFIFSLKDTLKRRS	NS2a	1330	1344	HLA-DRB1*11:01	>0% - 50%	Dengue virus 3	ELISPOT	IFNg release	Positive	1	1	1	1	1	0	0	0	0	1	1	1	0	0	1	1	0	1	1	0	1	0	0	1	1	1	0	1	1	0	1	0	1	0	0	0	0	0	1	1	0	0	0	1	1	1	1	0	0	0	1	0	0	1	1	1	0	0	1	0	0	0	1	0	0	0	1	1	1	1	0	0	0
195912	RTATLILAGVSLLPV	NS2a	1291	1305	HLA-DRB1*03:01	>0% - 50%	Dengue virus 3	ELISPOT	IFNg release	Positive	1	1	1	1	1	0	0	0	0	1	1	1	0	0	1	1	0	1	1	0	1	0	0	1	1	1	0	1	1	0	1	0	1	0	0	0	0	0	1	1	0	1	0	1	1	1	1	0	0	0	1	0	0	1	1	1	0	0	1	0	0	0	1	0	0	0	0	0	1	0	0	0	0
196123	VGVLFTFVLLLSGQI	NS2a	1159	1173	HLA-DRB1*07:01	>0% - 50%	Dengue virus 3	ELISPOT	IFNg release	Positive	0	0	0	0	0	0	1	1	0	0	0	0	1	0	0	0	1	0	0	0	0	1	0	0	0	0	1	0	0	0	0	0	0	1	1	1	0	0	0	0	0	0	0	0	0	0	0	0	0	0	0	1	1	0	0	0	1	1	0	0	1	0	0	0	1	1	0	0	0	0	0	0	0
867116	IAGVFFTFVLLLSGQ	NS2a	1158	1172	HLA-DPB1*01:01	>0% - 50%	Dengue virus 3	ELISPOT	IFNg release	Positive	0	1	0	0	0	0	0	0	0	1	1	0	0	0	0	1	0	0	0	0	1	0	0	0	1	1	0	0	1	0	0	0	1	0	0	0	0	0	0	1	0	0	0	1	0	1	1	0	0	0	0	0	0	0	0	0	0	0	0	0	0	0	0	0	0	0	0	0	0	0	0	0	0
868046	MALKLITQFETYQLW	NS2a	1257	1271	HLA-DPB1*01:01	>0% - 50%	Dengue virus 3	ELISPOT	IFNg release	Positive	0	1	1	1	1	0	0	0	0	1	0	1	0	0	1	1	0	1	0	0	1	0	0	1	0	0	0	1	1	0	1	0	1	0	0	0	0	0	1	1	0	1	0	1	1	0	0	0	0	0	1	0	0	1	1	1	0	0	1	0	0	0	1	0	0	0	1	1	1	1	0	0	0
868329	PLPLFIFSLKDTLKR	NS2a	1328	1342	HLA-DPB1*01:01	>0% - 50%	Dengue virus 3	ELISPOT	IFNg release	Positive	1	1	1	1	1	0	0	0	0	1	1	1	0	0	1	1	0	1	1	0	1	0	0	1	1	1	0	1	1	0	1	0	1	0	0	0	0	0	1	1	0	0	0	1	1	1	1	0	0	0	1	0	0	1	1	1	0	0	1	0	0	0	1	0	0	0	1	1	1	1	0	0	0
869298	TQFETYQLWTALISL	NS2a	1263	1277	HLA-DPB1*04:02	>0% - 50%	Dengue virus 3	ELISPOT	IFNg release	Positive	0	1	0	0	0	0	0	0	0	1	0	0	0	0	0	1	0	0	0	0	1	0	0	0	0	0	0	0	1	0	0	0	1	0	0	0	0	0	0	1	0	1	0	1	0	0	0	0	0	0	0	0	0	0	0	0	0	0	0	0	0	0	0	0	0	0	0	0	0	0	0	0	0
30395	KEGEKKKLRPRWLDA	NS3	585	599	HLA class II	>0% - 50%	Dengue virus 3	ICS	IFNg release	Positive	1	1	0	0	0	0	0	0	0	1	1	0	0	0	0	1	0	0	1	0	1	0	0	0	1	1	0	0	1	0	0	0	1	0	1	1	0	0	0	1	0	1	0	1	0	1	1	0	0	0	0	0	0	0	0	0	0	0	0	0	0	0	0	0	0	1	1	1	1	1	0	0	0
39	AAAIFMTATPPGTAD	NS3	1783	1797	HLA-DRB1*04:03	>0% - 50%	Dengue virus 3	ELISPOT	IFNg release	Positive	1	1	0	0	0	0	0	0	0	1	1	0	0	0	0	1	0	0	1	0	1	0	0	0	1	1	0	0	1	0	0	0	1	0	0	0	0	0	0	1	0	1	0	1	0	1	1	0	0	0	0	0	0	0	0	0	0	0	0	0	0	0	0	0	0	0	1	1	1	1	0	0	0
539028	AAIFMTATPPGTADA	NS3	1784	1798	HLA-DRB1*10:01	>0% - 50%	Dengue virus 3	ELISPOT	IFNg release	Positive	1	1	0	0	0	0	0	0	0	1	1	0	0	0	0	1	0	0	1	0	1	0	0	0	1	1	0	0	1	0	0	0	1	0	0	0	0	0	0	1	0	1	0	1	0	1	1	0	0	0	0	0	0	0	0	0	0	0	0	0	0	0	0	0	0	0	1	1	1	1	0	0	0
539052	AGKTVWFVPSIKAGN	NS3	1829	1843	HLA-DRB1*08:03	>0% - 50%	Dengue virus 3	ELISPOT	IFNg release	Positive	1	1	0	0	0	0	0	0	0	1	1	0	0	0	0	1	0	0	1	0	1	0	0	0	1	1	0	0	1	0	0	0	1	0	0	0	0	0	0	1	0	1	0	1	0	1	1	0	0	0	0	0	0	0	0	0	0	0	0	0	0	0	0	0	0	0	0	1	0	1	0	0	0
195522	IPLQWIASAIVLEFF	NS4a	2192	2206	HLA-DRB1*01:01	>0% - 50%	Dengue virus 3	ELISPOT	IFNg release	Positive	1	1	0	0	0	0	0	0	0	1	1	0	0	0	0	0	0	0	1	0	1	0	0	0	1	1	0	0	1	0	0	0	1	0	0	0	0	0	0	1	0	1	0	1	0	1	1	0	0	1	0	0	0	0	0	0	0	0	0	1	1	1	0	0	0	0	0	0	0	0	0	0	0
869474	VIGILTLAAIIAANE	NS4a	2230	2244	HLA-DQA1*01:02/DQB1*06:02	>0% - 50%	Dengue virus 3	ELISPOT	IFNg release	Positive	1	1	0	0	0	0	0	0	0	1	1	0	0	0	0	1	0	0	1	0	1	0	0	0	1	1	0	0	1	0	0	0	1	0	0	0	0	0	0	1	0	1	0	1	0	1	1	0	0	0	0	0	0	0	0	0	0	0	0	1	0	0	0	0	0	0	0	0	0	0	0	0	0
195663	LLLITHYAIIGPGLQ	NS4b	2354	2368	HLA-DRB1*07:01	>0% - 50%	Dengue virus 3	ELISPOT	IFNg release	Positive	1	1	0	0	0	0	0	0	0	1	1	0	0	0	0	1	0	0	1	0	1	0	0	0	1	1	0	0	1	0	0	0	1	0	0	0	0	0	0	1	0	1	0	1	0	1	1	0	0	0	0	0	0	0	0	0	0	0	0	0	0	0	0	0	0	0	0	0	0	0	0	0	0
195807	NPLTLTAAVLLLITH	NS4b	2345	2359	HLA-DRB1*07:01	>0% - 50%	Dengue virus 3	ELISPOT	IFNg release	Positive	1	1	0	0	0	0	0	0	0	1	1	0	0	0	0	1	0	0	1	0	1	0	0	0	1	1	0	0	1	0	0	0	1	0	0	0	0	0	0	1	0	1	0	1	0	1	1	0	0	0	0	0	0	0	0	0	0	0	0	0	0	0	0	0	0	0	0	0	0	0	0	0	0
196023	TAAVLLLITHYAIIG	NS4b	2350	2364	HLA-DRB1*15:01	>0% - 50%	Dengue virus 3	ELISPOT	IFNg release	Positive	1	1	0	0	0	0	0	0	0	1	1	0	0	0	0	1	0	0	1	0	1	0	0	0	1	1	0	0	1	0	0	0	1	0	0	0	0	0	0	1	0	1	0	1	0	1	1	0	0	0	0	0	0	0	0	0	0	0	0	0	0	0	0	0	0	0	0	0	0	0	0	0	0
196163	VNPLTLTAAVLLLIT	NS4b	2344	2358	HLA-DRB1*01:01	>0% - 50%	Dengue virus 3	ELISPOT	IFNg release	Positive	1	1	0	0	0	0	0	0	0	1	1	0	0	0	0	1	0	0	1	0	1	0	0	0	1	1	0	0	1	0	0	0	1	0	0	0	0	0	0	1	0	1	0	1	0	1	1	0	0	0	0	0	0	0	0	0	0	0	0	0	0	0	0	0	0	0	0	0	0	0	0	0	0
195408	GNIVASVNMVSRLLL	NS5	3	17	HLA-DRB1*03:01	>0% - 50%	Dengue virus 3	ELISPOT	IFNg release	Positive	0	0	0	0	0	0	0	0	0	0	0	0	0	0	0	0	0	0	0	0	0	0	0	0	0	0	0	0	0	0	0	0	0	0	0	0	0	0	0	0	0	0	0	0	0	0	0	0	0	0	0	0	0	0	0	0	0	0	0	0	0	0	0	0	0	1	0	0	0	0	0	0	0
196038	TGNIVASVNMVSRLL	NS5	2	16	HLA-DRB1*04:01	>0% - 50%	Dengue virus 3	ELISPOT	IFNg release	Positive	0	0	0	0	0	0	0	0	0	0	0	0	0	0	0	0	0	0	0	0	0	0	0	0	0	0	0	0	0	0	0	0	0	0	0	0	0	0	0	0	0	0	0	0	0	0	0	0	0	0	0	0	0	0	0	0	0	0	0	0	0	0	0	0	0	1	0	0	0	0	0	0	0
196221	WLWRTLGRNKKPRLC	NS5	157	171	HLA-DRB1*14:02	>0% - 50%	Dengue virus 3	ELISPOT	IFNg release	Positive	0	0	0	0	0	1	1	1	1	0	0	0	1	1	0	0	1	0	0	0	0	1	1	0	0	0	1	0	0	1	0	1	0	1	1	1	1	1	0	0	1	0	1	0	0	0	0	1	0	1	0	1	1	0	0	0	1	1	0	1	1	1	0	1	1	1	0	0	0	0	0	0	0
32944	KPWDVVPTV	NS5	2819	2827	HLA class II	>0% - 50%	Dengue virus 3	ELISA	IL-2 release	Positive	0	0	0	0	0	0	0	0	1	0	0	0	0	0	0	0	0	0	0	0	0	0	0	0	0	0	0	0	0	0	0	0	0	0	0	0	0	0	0	0	0	0	0	0	0	0	0	0	0	0	0	0	0	0	0	0	0	0	0	0	0	0	0	0	0	0	0	0	0	0	0	0	0
539444	IVASVNMVSRLLLNR	NS5	2717	2731	HLA-DRB1*12:02	>0% - 50%	Dengue virus 3	ELISPOT	IFNg release	Positive	0	0	0	0	0	0	0	0	0	0	0	0	0	0	0	0	0	0	0	0	0	0	0	0	0	0	0	0	0	0	0	0	0	0	0	0	0	0	0	0	0	0	0	0	0	0	0	0	0	0	0	0	0	0	0	0	0	0	0	0	0	0	0	0	0	1	0	0	0	0	0	0	0
539726	NIVASVNMVSRLLLN	NS5	2716	2730	HLA-DRB1*15:06	>0% - 50%	Dengue virus 3	ELISPOT	IFNg release	Positive	0	0	0	0	0	0	0	0	0	0	0	0	0	0	0	0	0	0	0	0	0	0	0	0	0	0	0	0	0	0	0	0	0	0	0	0	0	0	0	0	0	0	0	0	0	0	0	0	0	0	0	0	0	0	0	0	0	0	0	0	0	0	0	0	0	1	0	0	0	0	0	0	0
539831	RATWAQNIPTAIQQV	NS5	3346	3360	HLA-DRB1*09:01	>0% - 50%	Dengue virus 3	ELISPOT	IFNg release	Positive	0	1	0	0	0	0	0	0	0	1	0	0	0	0	0	1	0	0	0	0	1	0	0	0	0	0	0	0	1	0	0	0	1	0	0	0	0	0	0	1	0	0	0	1	0	0	0	0	0	0	0	0	0	0	0	0	0	0	0	0	0	0	0	0	0	0	0	0	0	0	0	0	0
540025	VASVNMVSRLLLNRF	NS5	2718	2732	HLA-DRB1*14:04	>0% - 50%	Dengue virus 3	ELISPOT	IFNg release	Positive	0	0	0	0	0	0	0	0	0	0	0	0	0	0	0	0	0	0	0	0	0	0	0	0	0	0	0	0	0	0	0	0	0	0	0	0	0	0	0	0	0	0	0	0	0	0	0	0	0	0	0	0	0	0	0	0	0	0	0	0	0	0	0	0	0	1	0	0	0	0	0	0	0
591602	ACLGKAYAQMWTLMY	NS5	3242	3256	HLA-DRB1*04:01	>0% - 50%	Dengue virus 3	ELISPOT	IFNg release	Positive	0	0	0	0	0	0	0	0	0	0	0	0	0	0	0	0	0	0	0	0	0	0	0	0	0	0	0	0	0	0	0	0	0	0	0	0	0	0	0	0	0	0	0	0	0	0	0	0	0	0	0	0	0	0	0	0	0	0	0	0	0	0	0	0	0	0	1	1	0	1	0	0	0
738537	ASVNMVSRLLLNRFT	NS5	7	21	HLA-DRB1*14:06	>0% - 50%	Dengue virus 3	ELISPOT	IFNg release	Positive	0	0	0	0	0	0	0	0	0	0	0	0	0	0	0	0	0	0	0	0	0	0	0	0	0	0	0	0	0	0	0	0	0	0	0	0	0	0	0	0	0	0	0	0	0	0	0	0	0	0	0	0	0	0	0	0	0	0	0	0	0	0	0	0	0	1	0	0	0	0	0	0	0

CD4 DENV-4 Epitopes

Epitope_ID	AA_Seq	Protein_Name	Start	End	Allele	step	Serotype	Method/Technique	Assay Group	Qualitative Measure
195488	IGRMLNILNRRKRST	C	87	101	HLA-DRB1*14:02	0%	Denguevirus 4	ELISPOT	IFNg release	Positive
195768	MTLLCLIPTAMAFHL	C	102	116	HLA-DRB1*01:01	0%	Denguevirus 4	ELISPOT	IFNg release	Positive
195922	RWGQLKKTKAIKILT	C	67	81	HLA-DRB1*07:01	0%	Denguevirus 4	ELISPOT	IFNg release	Positive
196065	TPQGLVKRFSSGLFS	C	24	38	HLA-DRB1*15:01	0%	Denguevirus 4	ELISPOT	IFNg release	Positive
739060	GRMLNILNGRKRSTM	C	88	102	HLA-DRB1*14:02	0%	Denguevirus 4	ELISPOT	IFNg release	Positive
739178	IGRMLNILNRRRRST	C	87	101	HLA-DRB1*14:02	0%	Denguevirus 4	ELISPOT	IFNg release	Positive
167808	RDVNKEKVVGRVISSTPLAE	E	340	359	HLA class II	0%	Denguevirus 4	ELISPOT	IFNg release	Positive
195364	GGVSWMIRILIGILV	E	449	463	HLA-DRB1*15:06	0%	Denguevirus 4	ELISPOT	IFNg release	Positive
195435	GVSWMIRILIGLLVL	E	729	743	HLA-DRB1*15:06	0%	Denguevirus 4	ELISPOT	IFNg release	Positive
195436	GVSWMVRILIGFLVL	E	450	464	HLA-DRB1*08:02	0%	Denguevirus 4	ELISPOT	IFNg release	Positive
195625	LESTYRGAKRMAILG	E	681	695	HLA-DRB1*14:02	0%	Denguevirus 4	ELISPOT	IFNg release	Positive
196189	VSWIIRILIGLLVLW	E	451	465	HLA-DRB1*15:06	0%	Denguevirus 4	ELISPOT	IFNg release	Positive
540080	VRILIGFLVLWIGTN	E	455	469	HLA-DRB1*15:06	0%	Denguevirus 4	ELISPOT	IFNg release	Positive
539829	QYQFQPESPARLASA	NS1	805	819	HLA-DRB1*09:01	0%	Denguevirus 4	ELISPOT	IFNg release	Positive
167725	AMTTTLSIPHDLMELIDGIS	NS2a	50	69	HLA class II	0%	Denguevirus 4	ELISPOT	IFNg release	Positive
195157	ALSLTFIRSTMSLVM	NS2a	1274	1288	HLA-DRB1*04:01	0%	Denguevirus 4	ELISPOT	IFNg release	Positive
195158	ALSLTFIRSTTPLVM	NS2a	1274	1288	HLA-DRB1*07:01	0%	Denguevirus 4	ELISPOT	IFNg release	Positive
195221	DLMELISGISLGLIL	NS2a	1243	1257	HLA-DRB1*07:01	0%	Denguevirus 4	ELISPOT	IFNg release	Positive
196061	TMSLVMAWRTIMAVL	NS2a	1283	1297	HLA-DRB1*03:01	0%	Denguevirus 4	ELISPOT	IFNg release	Positive
196070	TRKHMILAVVITLCA	NS2a	1154	1168	HLA-DRB1*08:02	0%	Denguevirus 4	ELISPOT	IFNg release	Positive
539130	CLRRRVTRKHMILAV	NS2a	1148	1162	HLA-DRB1*08:03	0%	Denguevirus 4	ELISPOT	IFNg release	Positive
539198	ECLRRRVTRKHMILA	NS2a	1147	1161	HLA-DRB1*13:01	0%	Denguevirus 4	ELISPOT	IFNg release	Positive
539407	IGGQTHLAIMAVFKM	NS2a	1194	1208	HLA-DRB1*13:01	0%	Denguevirus 4	ELISPOT	IFNg release	Positive
539434	IRSTMSLVMAWRTIM	NS2a	1280	1294	HLA-DRB1*13:01	0%	Denguevirus 4	ELISPOT	IFNg release	Positive
539628	LSLTFIRSTTPLVMA	NS2a	1275	1289	HLA-DRB1*08:03	0%	Denguevirus 4	ELISPOT	IFNg release	Positive
539921	SLTFIRSTTPLIMAW	NS2a	1276	1290	HLA-DRB1*04:03	0%	Denguevirus 4	ELISPOT	IFNg release	Positive
539961	TALMVIGMAMTTTLS	NS2a	1226	1240	HLA-DRB1*01:02	0%	Denguevirus 4	ELISPOT	IFNg release	Positive
738559	AVVITLCAILLGGLT	NS2a	1161	1175	HLA-DRB1*01:02	0%	Denguevirus 4	ELISPOT	IFNg release	Positive
867969	LTFIRSTTPLVMAWR	NS2a	1277	1291	HLA-DRB3*02:02	0%	Denguevirus 4	ELISPOT	IFNg release	Positive
45689	NRKGKIVGLYGNGVV	NS3	141	155	HLA-DRB1*15:01	0%	Denguevirus 4	ICS	IFNg release	Positive
53505	REGEKKKLRPRWLDR	NS3	585	599	HLA class II	0%	Denguevirus 4	ICS	IFNg release	Positive
539021	AAAIFMTATPPGSID	NS3	1783	1797	HLA-DRB1*04:07	0%	Denguevirus 4	ELISPOT	IFNg release	Positive
540077	VREAIKRRLRTLVLA	NS3	1682	1696	HLA-DRB1*08:03	0%	Denguevirus 4	ELISPOT	IFNg release	Positive
195853	QLIYVILTILAIIGL	NS4a	2225	2239	HLA-DRB1*15:02	0%	Denguevirus 4	ELISPOT	IFNg release	Positive
196250	YVILAILTIIGLVAA	NS4a	2228	2242	HLA-DRB1*08:02	0%	Denguevirus 4	ELISPOT	IFNg release	Positive
196251	YVILALLTIISLIAA	NS4a	2228	2242	HLA-DRB1*15:02	0%	Denguevirus 4	ELISPOT	IFNg release	Positive
195719	LTASLAMLLVHYAII	NS4b	2346	2360	HLA-DRB1*07:01	0%	Denguevirus 4	ELISPOT	IFNg release	Positive
196055	TLIASLVMLLVHYAI	NS4b	2345	2359	HLA-DRB1*01:01	0%	Denguevirus 4	ELISPOT	IFNg release	Positive
196082	TTLIASLVMLLVHYA	NS4b	2344	2358	HLA-DRB1*13:01	0%	Denguevirus 4	ELISPOT	IFNg release	Positive
539661	MGCYSQVNPTTLIAS	NS4b	2335	2349	HLA-DRB1*09:01	0%	Denguevirus 4	ELISPOT	IFNg release	Positive
738500	AMGCYSQVNPTTLIA	NS4b	2334	2348	HLA-DRB1*04:07	0%	Denguevirus 4	ELISPOT	IFNg release	Positive
195593	KTFMKDGRSLVVPCR	NS5	3202	3216	HLA-DRB1*03:01	0%	Denguevirus 4	ELISPOT	IFNg release	Positive
196105	VDYMPVMKRYSAPFE	NS5	3368	3382	HLA-DRB1*11:01	0%	Denguevirus 4	ELISPOT	IFNg release	Positive
17605	FRKEIGRML	C	83	91	HLA-DPw4	100%	Denguevirus 4	51 chromium	cytotoxicity	Positive
195316	FNMLKRERNRVSTPQ	C	12	26	HLA-DRB1*04:01	100%	Denguevirus 4	ELISPOT	IFNg release	Positive
195325	FRKEIGRMLNILNGR	C	19	33	HLA-DRB1*04:01	100%	Denguevirus 4	ELISPOT	IFNg release	Positive
195900	RMVLAFITFLRVLSI	C	44	58	HLA-DRB1*11:01	100%	Denguevirus 4	ELISPOT	IFNg release	Positive
196134	VLAFITFLRVLSIPP	C	26	40	HLA-DRB1*04:01	100%	Denguevirus 4	ELISPOT	IFNg release	Positive
539044	AFITFLRVLSIPPTA	C	28	42	HLA-DRB1*04:03	100%	Denguevirus 4	ELISPOT	IFNg release	Positive
539255	FITFLRVLSIPPTAG	C	49	63	HLA-DRB1*04:07	100%	Denguevirus 4	ELISPOT	IFNg release	Positive
539294	GFRKEIGRMLNILNG	C	54	68	HLA-DRB3*02:02	100%	Denguevirus 4	ELISPOT	IFNg release	Positive
539536	LAFITFLRVLSIPPT	C	27	41	HLA-DRB1*12:02	100%	Denguevirus 4	ELISPOT	IFNg release	Positive
69367	VLAFITFLR	C	46	54	HLA-DPw4	100%	Denguevirus 4	51 chromium	cytotoxicity	Positive
740156	RPPFNMLKRERNRVS	C	9	23	HLA-DRB1*14:02	100%	Denguevirus 4	ELISPOT	IFNg release	Positive
195272	ESTYRGAKRMAILGE	E	682	696	HLA-DRB1*15:06	100%	Denguevirus 4	ELISPOT	IFNg release	Positive
539379	HAKRQDVTVLGSQEGAMH	E	523	540	HLA class II	100%	Denguevirus 4	ICS	IFNg release	Positive
539463	KERMVTFKVPHAKRQ	E	513	527	HLA-DRB1*04:03	100%	Denguevirus 4	ELISPOT	IFNg release	Positive
738886	FESTYRGAKRMAILG	E	681	695	HLA-DRB1*14:02	100%	Denguevirus 4	ELISPOT	IFNg release	Positive
741747	MRCVGVGNRDFVEGVSGGAW	E	280	299	HLA class II	100%	Denguevirus 4	ELISPOT	IFNg release	Positive
866696	FGMFTTNIWMKFREG	NS1	934	948	HLA-DPB1*04:01	100%	Denguevirus 4	ELISPOT	IFNg release	Positive
1067119	GKVIGLYGNGVVTKS	NS3	1618	1632	HLA-DRB1*15:01	100%	Denguevirus 4	ELISPOT	IFNg release	Positive
195298	FHTMWHVTRGSVICH	NS3	1520	1534	HLA-DRB1*07:01	100%	Denguevirus 4	ELISPOT	IFNg release	Positive
195563	KGKVIGLYGNGVVTK	NS3	5	19	HLA-DRB1*15:01	100%	Denguevirus 4	ELISPOT	IFNg release	Positive
195838	PNYNLIVMDEAHFTD	NS3	1750	1764	HLA-DRB1*03:01	100%	Denguevirus 4	ELISPOT	IFNg release	Positive
195962	SIVREALKRRLRTLI	NS3	1680	1694	HLA-DRB1*11:01	100%	Denguevirus 4	ELISPOT	IFNg release	Positive
33192	KRLTIMDLHPGAGKT	NS3	186	200	HLA class II	100%	Denguevirus 4	ICS	IFNg release	Positive
539080	ALRGLPIRYQTPAVK	NS3	96	110	HLA-DRB1*13:01	100%	Denguevirus 4	ELISPOT	IFNg release	Positive
539359	GVFHTMWHVTRGSVI	NS3	1518	1532	HLA-DRB1*08:03	100%	Denguevirus 4	ELISPOT	IFNg release	Positive
539520	KVIGLYGNGVVTKSG	NS3	7	21	HLA-DRB1*15:06	100%	Denguevirus 4	ELISPOT	IFNg release	Positive
68977	VIGLYGNGV	NS3	146	154	HLA-DRB1*15:01	100%	Denguevirus 4	51 chromium	cytotoxicity	Positive
739036	GLPIRYQTPAVKSEH	NS3	1711	1725	HLA-DRB1*14:06	100%	Denguevirus 4	ELISPOT	IFNg release	Positive
739563	LRGLPIRYQTPAVKS	NS3	1709	1723	HLA-DRB1*14:06	100%	Denguevirus 4	ELISPOT	IFNg release	Positive
195274	ETLMLVALLGAMTAG	NS4a	2145	2159	HLA-DRB1*08:02	100%	Denguevirus 4	ELISPOT	IFNg release	Positive
195876	RAKLALDNIVMLHTT	NS4a	2112	2126	HLA-DRB1*03:01	100%	Denguevirus 4	ELISPOT	IFNg release	Positive
196058	TLMLVALLGAMTAGI	NS4a	2146	2160	HLA-DRB1*01:01	100%	Denguevirus 4	ELISPOT	IFNg release	Positive
539638	LVALLGAMTAGIFLF	NS4a	2149	2163	HLA-DRB1*09:01	100%	Denguevirus 4	ELISPOT	IFNg release	Positive
738568	AYQHALNELPESLET	NS4a	2132	2146	HLA-DRB1*04:07	100%	Denguevirus 4	ELISPOT	IFNg release	Positive
195219	DLEPISYDPKFEKQL	NS4b	2393	2407	HLA-DRB1*03:01	100%	Denguevirus 4	ELISPOT	IFNg release	Positive
195276	ETTILDVDLRPASAW	NS4b	2263	2277	HLA-DRB1*03:01	100%	Denguevirus 4	ELISPOT	IFNg release	Positive
196048	TILDVDLRPASAWTL	NS4b	2265	2279	HLA-DRB1*03:01	100%	Denguevirus 4	ELISPOT	IFNg release	Positive
539185	DVDLRPASAWTLYAV	NS4b	2268	2282	HLA-DRB1*09:01	100%	Denguevirus 4	ELISPOT	IFNg release	Positive
739202	ILDVDLRPASAWTLY	NS4b	2266	2280	HLA-DRB1*01:02	100%	Denguevirus 4	ELISPOT	IFNg release	Positive
867009	GRFWNTTIAVSTANI	NS4b	2449	2463	HLA-DQA1*01:02/DQB1*06:02	100%	Denguevirus 4	ELISPOT	IFNg release	Positive
1066543	ACLGKAYAQMWSLMY	NS5	3240	3254	HLA-DRB1*04:01	100%	Denguevirus 4	ELISPOT	IFNg release	Positive-Low
195411	GNIVSSVNTTSKMLL	NS5	2713	2727	HLA-DRB1*04:01	100%	Denguevirus 4	ELISPOT	IFNg release	Positive
195737	LWCGSLIGLSSRATW	NS5	3333	3347	HLA-DRB1*11:01	100%	Denguevirus 4	ELISPOT	IFNg release	Positive
195858	QMWSLMYFHRRDLRL	NS5	3248	3262	HLA-DRB1*15:01	100%	Denguevirus 4	ELISPOT	IFNg release	Positive
196230	YAQMWSLMYFHRRDL	NS5	3246	3260	HLA-DRB1*15:01	100%	Denguevirus 4	ELISPOT	IFNg release	Positive
539251	FEEYKRSGILEVDRT	NS5	2512	2526	HLA-DRB1*09:01	100%	Denguevirus 4	ELISPOT	IFNg release	Positive
539553	LGEFGRAKGSRAIWY	NS5	2949	2963	HLA-DRB1*09:01	100%	Denguevirus 4	ELISPOT	IFNg release	Positive
539703	MVTQLAMTDTTPFGQQR	NS5	2824	2840	HLA class II	100%	Denguevirus 4	ICS	IFNg release	Positive
739405	KVRSNAAIGAVFQEE	NS5	2889	2903	HLA-DRB1*01:02	100%	Denguevirus 4	ELISPOT	IFNg release	Positive
868638	RDLRLASMAICSAVP	NS5	3258	3272	HLA-DQA1*01:02/DQB1*06:02	100%	Denguevirus 4	ELISPOT	IFNg release	Positive
1067635	LKRWGQLKKNKAIKI	C	65	79	HLA-DRB1*07:01	81% - <100%	Denguevirus 4	ELISPOT	IFNg release	Positive-Low
195587	KRWGQLKKNKAIKIL	C	46	60	HLA-DRB1*08:03	81% - <100%	Denguevirus 4	ELISPOT	IFNg release	Positive
195651	LKKNKAIKILTGFRK	C	51	65	HLA-DRB1*14:04	81% - <100%	Denguevirus 4	ELISPOT	IFNg release	Positive
539496	KNKAIKILTGFRKEI	C	53	67	HLA-DRB1*12:02	81% - <100%	Denguevirus 4	ELISPOT	IFNg release	Positive
539632	LTGFRKEIGRMLNIL	C	52	66	HLA-DRB3*02:02	81% - <100%	Denguevirus 4	ELISPOT	IFNg release	Positive
539898	SGKGPLRMVLAFITF	C	18	32	HLA-DRB1*15:06	81% - <100%	Denguevirus 4	ELISPOT	IFNg release	Positive
539987	TLLCLIPTVMAFHLS	C	103	117	HLA-DRB1*04:03	81% - <100%	Denguevirus 4	ELISPOT	IFNg release	Positive
739059	GRMLNILNGRKRSTI	C	88	102	HLA-DRB1*14:06	81% - <100%	Denguevirus 4	ELISPOT	IFNg release	Positive
739175	IGRMLNILNGRKRST	C	87	101	HLA-DRB1*14:02	81% - <100%	Denguevirus 4	ELISPOT	IFNg release	Positive
868517	QLKKNKAIKILTGFR	C	42	56	HLA-DQA1*01:02/DQB1*06:02	81% - <100%	Denguevirus 4	ELISPOT	IFNg release	Positive
195283	FALLAGFMAYMIGQT	PreM	246	260	HLA-DRB1*01:01	81% - <100%	Denguevirus 4	ELISPOT	IFNg release	Positive
195505	ILRNPGFALLAGFMA	PreM	240	254	HLA-DRB1*01:01	81% - <100%	Denguevirus 4	ELISPOT	IFNg release	Positive
195526	IQRTVFFILMMLVAP	PreM	262	276	HLA-DRB1*07:01	81% - <100%	Denguevirus 4	ELISPOT	IFNg release	Positive
195901	RNPGFALLAGFMAYM	PreM	242	256	HLA-DRB1*15:01	81% - <100%	Denguevirus 4	ELISPOT	IFNg release	Positive
539395	IDCWCNLTSAWVMYG	PreM	117	131	HLA-DQA1*01:02/DQB1*06:02	81% - <100%	Denguevirus 4	ELISPOT	IFNg release	Positive
539820	QRTVFFILMMLVAPS	PreM	263	277	HLA-DRB1*13:01	81% - <100%	Denguevirus 4	ELISPOT	IFNg release	Positive
867174	IGQTGIQRTVFFILM	PreM	257	271	HLA-DPB1*02:01	81% - <100%	Denguevirus 4	ELISPOT	IFNg release	Positive
869119	TEGINKCTLIAMDLG	PreM	113	127	HLA-DQA1*01:02/DQB1*06:02	81% - <100%	Denguevirus 4	ELISPOT	IFNg release	Positive
195202	CIAVGGITLFLGFTV	E	758	772	HLA-DRB1*15:01	81% - <100%	Denguevirus 4	ELISPOT	IFNg release	Positive
195472	IAVGGITLFLGFTVH	E	480	494	HLA-DRB1*15:01	81% - <100%	Denguevirus 4	ELISPOT	IFNg release	Positive
539298	GGVSWMVRILIGLLV	E	449	463	HLA-DRB1*15:06	81% - <100%	Denguevirus 4	ELISPOT	IFNg release	Positive
539734	NLEYTVVVTVHNGDT	E	197	211	HLA-DRB1*04:07	81% - <100%	Denguevirus 4	ELISPOT	IFNg release	Positive
540051	VLGSQEGAMHSALAGA	E	531	546	HLA class II	81% - <100%	Denguevirus 4	ICS	IFNg release	Positive
738809	ENLEYTVVVTVHNGD	E	196	210	HLA-DRB1*04:07	81% - <100%	Denguevirus 4	ELISPOT	IFNg release	Positive
195822	NVMWKQITNELNYVL	NS1	1	15	HLA-DRB1*04:07	81% - <100%	Denguevirus 4	ELISPOT	IFNg release	Positive
867043	HADMGYWIESSKNQT	NS1	969	983	HLA-DQA1*01:01/DQB1*05:01	81% - <100%	Denguevirus 4	ELISPOT	IFNg release	Positive
195220	DLMEFIDGISLGLIL	NS2a	1244	1258	HLA-DRB1*07:01	81% - <100%	Denguevirus 4	ELISPOT	IFNg release	Positive
195541	ITALILGAQALPVYL	NS2a	1320	1334	HLA-DRB1*01:01	81% - <100%	Denguevirus 4	ELISPOT	IFNg release	Positive
195571	KHMILVVVTTLCAII	NS2a	1156	1170	HLA-DRB1*08:02	81% - <100%	Denguevirus 4	ELISPOT	IFNg release	Positive
195608	LAIMAVFKMSPGYVL	NS2a	1200	1214	HLA-DRB1*13:01	81% - <100%	Denguevirus 4	ELISPOT	IFNg release	Positive
195699	LQKQSHWVEITALIL	NS2a	1311	1325	HLA-DRB1*07:01	81% - <100%	Denguevirus 4	ELISPOT	IFNg release	Positive
195837	PLVMAWRTIMAVLFV	NS2a	1285	1299	HLA-DRB1*01:01	81% - <100%	Denguevirus 4	ELISPOT	IFNg release	Positive
195864	QSHWVEITALILGAQ	NS2a	1314	1328	HLA-DRB1*07:01	81% - <100%	Denguevirus 4	ELISPOT	IFNg release	Positive
196060	TMPLVMAWRTIMAVL	NS2a	1283	1297	HLA-DRB1*03:01	81% - <100%	Denguevirus 4	ELISPOT	IFNg release	Positive
196227	WVEITALILGAQALP	NS2a	1317	1331	HLA-DRB1*01:01	81% - <100%	Denguevirus 4	ELISPOT	IFNg release	Positive
196252	YVLGIFLRKLTSRET	NS2a	1212	1226	HLA-DRB1*11:01	81% - <100%	Denguevirus 4	ELISPOT	IFNg release	Positive
539131	CLRRRVTRKHMILVV	NS2a	1148	1162	HLA-DRB1*08:03	81% - <100%	Denguevirus 4	ELISPOT	IFNg release	Positive
539555	LGIFLRKLTSRETAL	NS2a	1214	1228	HLA-DRB1*08:03	81% - <100%	Denguevirus 4	ELISPOT	IFNg release	Positive
539664	MGGQIHLAIMAVFKM	NS2a	1194	1208	HLA-DRB1*13:01	81% - <100%	Denguevirus 4	ELISPOT	IFNg release	Positive
539687	MPLVMAWRTIMAVLF	NS2a	1284	1298	HLA-DRB1*12:02	81% - <100%	Denguevirus 4	ELISPOT	IFNg release	Positive
539930	SRETALMVIGMAMTT	NS2a	1223	1237	HLA-DRB1*15:06	81% - <100%	Denguevirus 4	ELISPOT	IFNg release	Positive
539937	STMPLVMAWRTIMAV	NS2a	1282	1296	HLA-DRB1*13:01	81% - <100%	Denguevirus 4	ELISPOT	IFNg release	Positive
539962	TALMVIGMAMTTVLS	NS2a	1226	1240	HLA-DRB1*15:06	81% - <100%	Denguevirus 4	ELISPOT	IFNg release	Positive
738742	ECLRRRVTRKHMILV	NS2a	1147	1161	HLA-DRB1*14:02	81% - <100%	Denguevirus 4	ELISPOT	IFNg release	Positive
740701	VVTLIPLCRTSCLQK	NS2a	1299	1313	HLA-DRB1*11:04	81% - <100%	Denguevirus 4	ELISPOT	IFNg release	Positive
867048	HDLMEFIDGISLGLI	NS2a	1243	1257	HLA-DQA1*01:01/DQB1*05:01	81% - <100%	Denguevirus 4	ELISPOT	IFNg release	Positive
867110	HWVEITALILGAQAL	NS2a	1316	1330	HLA-DQA1*03:01/DQB1*03:02	81% - <100%	Denguevirus 4	ELISPOT	IFNg release	Positive
867874	LMVIGMAMTTVLSIP	NS2a	1228	1242	HLA-DQA1*01:02/DQB1*06:02	81% - <100%	Denguevirus 4	ELISPOT	IFNg release	Positive
195404	GLVSLLGSALLKNDV	NS2b	9	23	HLA-DRB1*01:02	81% - <100%	Denguevirus 4	ELISPOT	IFNg release	Positive
195511	IMAVGLVSLLGSALL	NS2b	1352	1366	HLA-DRB1*01:01	81% - <100%	Denguevirus 4	ELISPOT	IFNg release	Positive
195662	LLLAAYVMSGSSADL	NS2b	1381	1395	HLA-DRB1*04:01	81% - <100%	Denguevirus 4	ELISPOT	IFNg release	Positive
195748	MAVGLVSLLGSALLK	NS2b	1353	1367	HLA-DRB1*01:01	81% - <100%	Denguevirus 4	ELISPOT	IFNg release	Positive
195931	SALLKNDVPLAGPMV	NS2b	1363	1377	HLA-DRB1*03:01	81% - <100%	Denguevirus 4	ELISPOT	IFNg release	Positive
196062	TNMITLLVKLALITV	NS2b	1436	1450	HLA-DRB1*08:02	81% - <100%	Denguevirus 4	ELISPOT	IFNg release	Positive
539101	AVGLVSLLGSALLKN	NS2b	1354	1368	HLA-DRB1*10:01	81% - <100%	Denguevirus 4	ELISPOT	IFNg release	Positive
869635	VTMTLWYMWQVKTQR	NS2b	1460	1474	HLA-DPB1*01:01	81% - <100%	Denguevirus 4	ELISPOT	IFNg release	Positive
12605	EIVDLMCHAT	NS3	255	264	HLA-DPw2	81% - <100%	Denguevirus 4	51 chromium	cytotoxicity	Positive
1392192	GREIVDLMCHATFTT	NS3	1727	1741		81% - <100%	Denguevirus 4	biological activity	activation	Positive
156711	LAPTRVVAAEMEEAL	NS3	1695	1709	HLA class II	81% - <100%	Denguevirus 4	51 chromium	cytotoxicity	Positive
195353	GEIGAVTLDFKPGTS	NS3	1595	1609	HLA-DRB1*03:01	81% - <100%	Denguevirus 4	ELISPOT	IFNg release	Positive
195695	LPVWLSYKVASAGIS	NS3	2016	2030	HLA-DRB1*08:02	81% - <100%	Denguevirus 4	ELISPOT	IFNg release	Positive
539024	AAAIFMTATPPGTTD	NS3	1783	1797	HLA-DRB1*04:03	81% - <100%	Denguevirus 4	ELISPOT	IFNg release	Positive
539547	LFKTLTGEIGAVTLD	NS3	1589	1603	HLA-DRB1*10:01	81% - <100%	Denguevirus 4	ELISPOT	IFNg release	Positive
539767	PGLFKTLTGEIGAVT	NS3	1587	1601	HLA-DRB1*10:01	81% - <100%	Denguevirus 4	ELISPOT	IFNg release	Positive
539803	QGKTVWFVPSIKAGN	NS3	1829	1843	HLA-DRB1*08:03	81% - <100%	Denguevirus 4	ELISPOT	IFNg release	Positive
539859	RKSGKKVIQLSRKTF	NS3	1850	1864	HLA-DRB1*08:03	81% - <100%	Denguevirus 4	ELISPOT	IFNg release	Positive
539899	SGKKVIQLSRKTFDT	NS3	1852	1866	HLA-DRB1*12:02	81% - <100%	Denguevirus 4	ELISPOT	IFNg release	Positive
863140	TRVEMGEAAAIFMTA	NS3	1776	1790	HLA-DQA1*03:01/DQB1*03:02	81% - <100%	Denguevirus 4	ELISPOT	IFNg release	Positive
867453	KLTDWDFVVTTDISE	NS3	1872	1886	HLA-DQA1*03:01/DQB1*03:02	81% - <100%	Denguevirus 4	ELISPOT	IFNg release	Positive
167758	ILTEIASLPTYLSSRAKL	NS4a	6	23	HLA-DP	81% - <100%	Denguevirus 4	ELISPOT	IFNg release	Positive
195176	ASLPTYLSSRAKLAL	NS4a	2103	2117	HLA-DRB1*07:01	81% - <100%	Denguevirus 4	ELISPOT	IFNg release	Positive
195224	DNQLIYVILTILTII	NS4a	2223	2237	HLA-DRB1*15:01	81% - <100%	Denguevirus 4	ELISPOT	IFNg release	Positive
195551	IYVILTILTIIALVA	NS4a	2227	2241	HLA-DRB1*01:01	81% - <100%	Denguevirus 4	ELISPOT	IFNg release	Positive
195575	KLSMGLIAIAVASGL	NS4a	2172	2186	HLA-DRB1*08:02	81% - <100%	Denguevirus 4	ELISPOT	IFNg release	Positive
195648	LIYVILTILTIIALV	NS4a	2226	2240	HLA-DRB1*07:01	81% - <100%	Denguevirus 4	ELISPOT	IFNg release	Positive
539165	DILTEIASLPTYLSS	NS4a	2097	2111	HLA-DRB1*04:07	81% - <100%	Denguevirus 4	ELISPOT	IFNg release	Positive
539313	GKAYQHALNELPESL	NS4a	2130	2144	HLA-DRB1*10:01	81% - <100%	Denguevirus 4	ELISPOT	IFNg release	Positive
539631	LTEIASLPTYLSSRA	NS4a	2099	2113	HLA-DRB1*04:03	81% - <100%	Denguevirus 4	ELISPOT	IFNg release	Positive
868951	SLPTYLSSRAKLALD	NS4a	2104	2118	HLA-DRB3*02:02	81% - <100%	Denguevirus 4	ELISPOT	IFNg release	Positive
195177	ASLVMLLVHYAIIGP	NS4b	2348	2362	HLA-DRB1*13:01	81% - <100%	Denguevirus 4	ELISPOT	IFNg release	Positive
195383	GLAFSLIKNAQTPRR	NS4b	2473	2487	HLA-DRB1*04:01	81% - <100%	Denguevirus 4	ELISPOT	IFNg release	Positive
195616	LCAGQLLLMRTTWAF	NS4b	2415	2429	HLA-DRB1*04:01	81% - <100%	Denguevirus 4	ELISPOT	IFNg release	Positive
195720	LTASLVMLLVHYAII	NS4b	2346	2360	HLA-DRB1*12:02	81% - <100%	Denguevirus 4	ELISPOT	IFNg release	Positive
196208	WAFCEVLTLATGPIL	NS4b	2427	2441	HLA-DRB1*07:01	81% - <100%	Denguevirus 4	ELISPOT	IFNg release	Positive
196226	WTLYAVATTILTPML	NS4b	2277	2291	HLA-DRB1*07:01	81% - <100%	Denguevirus 4	ELISPOT	IFNg release	Positive
539057	AGQLLLMRTTWAFCE	NS4b	2417	2431	HLA-DRB1*13:01	81% - <100%	Denguevirus 4	ELISPOT	IFNg release	Positive
539584	LLAMGCYSQVNPTTL	NS4b	2332	2346	HLA-DRB1*09:01	81% - <100%	Denguevirus 4	ELISPOT	IFNg release	Positive
539639	LVHYAIIGPGLQAKA	NS4b	2354	2368	HLA-DQA1*05:01/DQB1*02:01	81% - <100%	Denguevirus 4	ELISPOT	IFNg release	Positive
738447	AFSLIKNAQTPRRGT	NS4b	2475	2489	HLA-DRB1*14:06	81% - <100%	Denguevirus 4	ELISPOT	IFNg release	Positive
739417	LAFSLIKNAQTPRRG	NS4b	2474	2488	HLA-DRB1*14:02	81% - <100%	Denguevirus 4	ELISPOT	IFNg release	Positive
195235	EEFISKVRSNAAIGA	NS5	2884	2898	HLA-DRB1*08:02	81% - <100%	Denguevirus 4	ELISPOT	IFNg release	Positive
195454	HHFHKIFMKDGRSLV	NS5	3199	3213	HLA-DRB1*03:01	81% - <100%	Denguevirus 4	ELISPOT	IFNg release	Positive
195499	ILAKAIFKLTYQNKV	NS5	3050	3064	HLA-DRB1*07:01	81% - <100%	Denguevirus 4	ELISPOT	IFNg release	Positive
195879	REEFISKVRSNAAIG	NS5	2883	2897	HLA-DRB1*04:01	81% - <100%	Denguevirus 4	ELISPOT	IFNg release	Positive
195919	RVWIEDNPNMIDKTP	NS5	3303	3317	HLA-DRB1*03:01	81% - <100%	Denguevirus 4	ELISPOT	IFNg release	Positive
539149	DCVVKPLDERFSTSLLF	NS5	3152	3168	HLA class II	81% - <100%	Denguevirus 4	ICS	IFNg release	Positive
539222	EKPDMTIIGRRLQRL	NS5	2757	2771	HLA-DRB1*12:02	81% - <100%	Denguevirus 4	ELISPOT	IFNg release	Positive
539456	KAIFKLTYQNKVVKV	NS5	3053	3067	HLA-DRB1*09:01	81% - <100%	Denguevirus 4	ELISPOT	IFNg release	Positive
539493	KMLLNRFTTRHRKPT	NS5	2724	2738	HLA-DRB1*13:01	81% - <100%	Denguevirus 4	ELISPOT	IFNg release	Positive
539498	KPDMTIIGRRLQRLQ	NS5	2758	2772	HLA-DRB1*08:03	81% - <100%	Denguevirus 4	ELISPOT	IFNg release	Positive
539656	MAISGDDCVVKPLDERF	NS5	3146	3162	HLA class II	81% - <100%	Denguevirus 4	ICS	IFNg release	Positive
539770	PHHKILAKAIFKLTY	NS5	3046	3060	HLA-DRB1*12:02	81% - <100%	Denguevirus 4	ELISPOT	IFNg release	Positive
540007	TTSKMLLNRFTTRHR	NS5	2721	2735	HLA-DRB1*13:01	81% - <100%	Denguevirus 4	ELISPOT	IFNg release	Positive
540041	VGTYGLNTFTNMEVQ	NS5	3091	3105	HLA-DRB1*15:06	81% - <100%	Denguevirus 4	ELISPOT	IFNg release	Positive
866364	DERFSTSLLFLNDMG	NS5	3159	3173	HLA-DPB1*01:01	81% - <100%	Denguevirus 4	ELISPOT	IFNg release	Positive
866633	ESCVYNMMGKREKKL	NS5	2935	2949	HLA-DRB5*01:01	81% - <100%	Denguevirus 4	ELISPOT	IFNg release	Positive
867070	HKIFMKDGRSLVVPC	NS5	3202	3216	HLA-DRB3*02:02	81% - <100%	Denguevirus 4	ELISPOT	IFNg release	Positive
867315	ISKVRSNAAIGAVFQ	NS5	2887	2901	HLA-DRB3*02:02	81% - <100%	Denguevirus 4	ELISPOT	IFNg release	Positive
867411	KILAKAIFKLTYQNK	NS5	3049	3063	HLA-DPB1*04:02	81% - <100%	Denguevirus 4	ELISPOT	IFNg release	Positive
195244	EGVYRIMQRGLFGKT	NS3	1494	1508	HLA-DRB1*11:01	51%-80%	Denguevirus 4	ELISPOT	IFNg release	Positive
196032	TEGVYRIMQRGLFGK	NS3	146	160	HLA-DRB1*11:01	51%-80%	Denguevirus 4	ELISPOT	IFNg release	Positive
1068575	VVRPPFNMLKRERNR	C	7	21	HLA-DRB1*11:01	>0% - 50%	Denguevirus 4	ELISPOT	IFNg release	Positive-Low
195483	IGFRKEIGRMLNILN	C	81	95	HLA-DRB1*04:01	>0% - 50%	Denguevirus 4	ELISPOT	IFNg release	Positive
195580	KNKAIKILIGFRKEI	C	53	67	HLA-DRB1*15:01	>0% - 50%	Denguevirus 4	ELISPOT	IFNg release	Positive
36533	LIGFRKEIGRML	C	80	91	HLA-DPw4	>0% - 50%	Denguevirus 4	ICS	IFNg release	Positive
539571	LIGFRKEIGRMLNIL	C	80	94	HLA-DRB1*09:01	>0% - 50%	Denguevirus 4	ELISPOT	IFNg release	Positive
539581	LKKNKAIKILIGFRK	C	51	65	HLA-DRB1*14:04	>0% - 50%	Denguevirus 4	ELISPOT	IFNg release	Positive
540116	WGQLKKNKAIKILIG	C	68	82	HLA-DRB1*09:01	>0% - 50%	Denguevirus 4	ELISPOT	IFNg release	Positive
868732	RWGQLKKNKAIKILI	C	39	53	HLA-DRB3*02:02	>0% - 50%	Denguevirus 4	ELISPOT	IFNg release	Positive
195916	RTVFFVLMMLVAPSY	PreM	264	278	HLA-DRB1*01:01	>0% - 50%	Denguevirus 4	ELISPOT	IFNg release	Positive
869670	WCNLTSTWVMYGTCT	PreM	180	194	HLA-DQA1*01:02/DQB1*06:02	>0% - 50%	Denguevirus 4	ELISPOT	IFNg release	Positive
195227	DSYIVIGVGNSALTL	E	654	668	HLA-DRB1*15:06	>0% - 50%	Denguevirus 4	ELISPOT	IFNg release	Positive
195358	GGLFTSLGKAVHQVF	E	705	719	HLA-DRB1*11:01	>0% - 50%	Denguevirus 4	ELISPOT	IFNg release	Positive
195434	GVSWMIRILIGFLVL	E	729	743	HLA-DRB1*08:02	>0% - 50%	Denguevirus 4	ELISPOT	IFNg release	Positive
195473	IAVGGITLFLGFTVQ	E	759	773	HLA-DRB1*15:01	>0% - 50%	Denguevirus 4	ELISPOT	IFNg release	Positive
196036	TGIQRTVFFVLMMLV	E	44	58	HLA-DRB1*07:01	>0% - 50%	Denguevirus 4	ELISPOT	IFNg release	Positive
539230	EMILMKMKKKTWLVH	E	474	488	HLA-DRB1*12:02	>0% - 50%	Denguevirus 4	ELISPOT	IFNg release	Positive
540036	VGGLFTSLGKAVHQV	E	704	718	HLA-DRB1*09:01	>0% - 50%	Denguevirus 4	ELISPOT	IFNg release	Positive
195791	NIMWKQITNELNYVL	NS1	839	853	HLA-DRB1*04:07	>0% - 50%	Denguevirus 4	ELISPOT	IFNg release	Positive
195156	ALSLTFIRSTMPLVM	NS2a	1274	1288	HLA-DRB1*07:01	>0% - 50%	Denguevirus 4	ELISPOT	IFNg release	Positive
195190	AVFKMSPGYVLGVFL	NS2a	1204	1218	HLA-DRB1*01:01	>0% - 50%	Denguevirus 4	ELISPOT	IFNg release	Positive
195746	MAVFKMSPGYVLGVF	NS2a	1203	1217	HLA-DRB1*07:01	>0% - 50%	Denguevirus 4	ELISPOT	IFNg release	Positive
196253	YVLGVFLRKLTSRET	NS2a	1212	1226	HLA-DRB1*11:01	>0% - 50%	Denguevirus 4	ELISPOT	IFNg release	Positive
539360	GVFLRKLTSRETALM	NS2a	1215	1229	HLA-DRB1*10:01	>0% - 50%	Denguevirus 4	ELISPOT	IFNg release	Positive
539406	IGGQIHLAIMAVFKM	NS2a	1194	1208	HLA-DRB1*13:01	>0% - 50%	Denguevirus 4	ELISPOT	IFNg release	Positive
539433	IRSTMPLVMAWRTIM	NS2a	1280	1294	HLA-DRB1*13:01	>0% - 50%	Denguevirus 4	ELISPOT	IFNg release	Positive
539565	LGVFLRKLTSRETAL	NS2a	1214	1228	HLA-DRB1*08:03	>0% - 50%	Denguevirus 4	ELISPOT	IFNg release	Positive
539627	LSLTFIRSTMPLVMA	NS2a	1275	1289	HLA-DRB1*10:01	>0% - 50%	Denguevirus 4	ELISPOT	IFNg release	Positive
539919	SLTFIRSTMPLVMAW	NS2a	1276	1290	HLA-DRB1*09:01	>0% - 50%	Denguevirus 4	ELISPOT	IFNg release	Positive
540033	VFKMSPGYVLGVFLR	NS2a	1205	1219	HLA-DRB1*09:01	>0% - 50%	Denguevirus 4	ELISPOT	IFNg release	Positive
540053	VLGVFLRKLTSRETA	NS2a	1213	1227	HLA-DRB1*14:04	>0% - 50%	Denguevirus 4	ELISPOT	IFNg release	Positive
539020	AAAIFMTATPPGATD	NS3	1783	1797	HLA-DRB1*10:01	>0% - 50%	Denguevirus 4	ELISPOT	IFNg release	Positive
539023	AAAIFMTATPPGTID	NS3	1783	1797	HLA-DRB1*04:03	>0% - 50%	Denguevirus 4	ELISPOT	IFNg release	Positive
539026	AAIFMTATPPGATDP	NS3	1784	1798	HLA-DRB1*09:01	>0% - 50%	Denguevirus 4	ELISPOT	IFNg release	Positive
868371	PRWLDARVYADPMAL	NS3	2067	2081	HLA-DQA1*03:01/DQB1*03:02	>0% - 50%	Denguevirus 4	ELISPOT	IFNg release	Positive
195386	GLITIAVASGLLWVA	NS4a	2176	2190	HLA-DRB1*07:01	>0% - 50%	Denguevirus 4	ELISPOT	IFNg release	Positive
195809	NQLIYVILTILTIIG	NS4a	2224	2238	HLA-DRB1*08:02	>0% - 50%	Denguevirus 4	ELISPOT	IFNg release	Positive
539666	MGLITIAVASGLLWV	NS4a	2175	2189	HLA-DRB1*09:01	>0% - 50%	Denguevirus 4	ELISPOT	IFNg release	Positive
867726	LIYVILTILTIIGLI	NS4a	2226	2240	HLA-DPB1*04:02	>0% - 50%	Denguevirus 4	ELISPOT	IFNg release	Positive
195214	DFGFYQVKTETTILD	NS4b	2254	2268	HLA-DRB1*04:01	>0% - 50%	Denguevirus 4	ELISPOT	IFNg release	Positive
195592	KTETTILDVDLRPAS	NS4b	2261	2275	HLA-DRB1*03:01	>0% - 50%	Denguevirus 4	ELISPOT	IFNg release	Positive
196052	TKTDFGFYQVKTETT	NS4b	2251	2265	HLA-DRB1*04:01	>0% - 50%	Denguevirus 4	ELISPOT	IFNg release	Positive
196209	WAFCEVLTLATGPVL	NS4b	2427	2441	HLA-DRB1*07:01	>0% - 50%	Denguevirus 4	ELISPOT	IFNg release	Positive
738897	FGFYQVKTETTILDV	NS4b	2255	2269	HLA-DRB1*04:07	>0% - 50%	Denguevirus 4	ELISPOT	IFNg release	Positive
195230	DYMPVMKRYSAPSES	NS5	3369	3383	HLA-DRB1*11:01	>0% - 50%	Denguevirus 4	ELISPOT	IFNg release	Positive
195240	EEYVDYMPVMKRYSA	NS5	3365	3379	HLA-DRB1*11:01	>0% - 50%	Denguevirus 4	ELISPOT	IFNg release	Positive
195279	EYVDYMPVMKRYSAP	NS5	3366	3380	HLA-DRB1*11:01	>0% - 50%	Denguevirus 4	ELISPOT	IFNg release	Positive
195764	MPVMKRYSAPSESEG	NS5	3371	3385	HLA-DRB1*08:02	>0% - 50%	Denguevirus 4	ELISPOT	IFNg release	Positive
195815	NRVWIEDNPNMTDKT	NS5	3302	3316	HLA-DRB1*03:01	>0% - 50%	Denguevirus 4	ELISPOT	IFNg release	Positive
195823	NWLWALLGKKKNPRL	NS5	2866	2880	HLA-DRB1*14:02	>0% - 50%	Denguevirus 4	ELISPOT	IFNg release	Positive
195835	PKPGTRMVMTTTANW	NS5	2853	2867	HLA-DRB1*04:01	>0% - 50%	Denguevirus 4	ELISPOT	IFNg release	Positive
196071	TRMVMTTTANWLWAL	NS5	2857	2871	HLA-DRB1*04:01	>0% - 50%	Denguevirus 4	ELISPOT	IFNg release	Positive
196092	TYQNKVVKVLRPTPR	NS5	3059	3073	HLA-DRB1*08:02	>0% - 50%	Denguevirus 4	ELISPOT	IFNg release	Positive
196106	VDYMPVMKRYSAPSE	NS5	3368	3382	HLA-DRB1*11:01	>0% - 50%	Denguevirus 4	ELISPOT	IFNg release	Positive
539354	GTRMVMTTTANWLWA	NS5	2856	2870	HLA-DRB1*04:03	>0% - 50%	Denguevirus 4	ELISPOT	IFNg release	Positive
539769	PGTRMVMTTTANWLW	NS5	2855	2869	HLA-DRB1*04:03	>0% - 50%	Denguevirus 4	ELISPOT	IFNg release	Positive
740013	QLIRQMEAEGVITQD	NS5	3105	3119	HLA-DRB1*01:02	>0% - 50%	Denguevirus 4	ELISPOT	IFNg release	Positive
867473	KPLDERFGTSLLFLN	NS5	3156	3170	HLA-DPB1*01:01	>0% - 50%	Denguevirus 4	ELISPOT	IFNg release	Positive
867575	LDERFGTSLLFLNDM	NS5	3158	3172	HLA-DPB1*04:01	>0% - 50%	Denguevirus 4	ELISPOT	IFNg release	Positive

CD8 DENV-1 Epitopes

Epitope_ID	AA_Seq	Protein_Name	Start	End	Allele	step	Serotype	Method/Technique	Assay Group	Qualitative Measure	NCBI IDs of DENV-1 circulating strains from Thailand, (100% identity=1, green ; else 0, red) QFS19153.1	QFS19173.1	[illegible]
184458	LLMLLPTAL	C	83	91	HLA-B*08:01	0%	Dengue virus 1	ELISPOT	IFNg release	Positive	0	0	[illegible]
184844	MLKRERNRV	C	15	23	HLA-B*08:01	0%	Dengue virus 1	ELISPOT	IFNg release	Positive	0	0	[illegible]
240082	MVLALITFL	C	24	32	HLA-A*02:01	0%	Dengue virus 1	ELISPOT	IFNg release	Positive	0	0	[illegible]
183654	IAVGMVTLY	E	760	768	HLA-B*35:01	0%	Dengue virus 1	ELISPOT	IFNg release	Positive	0	0	[illegible]
185212	PTSEIQLTDY	E	449	458	HLA-A*01:01	0%	Dengue virus 1	ELISPOT	IFNg release	Positive	0	0	[illegible]
186524	WTMKIGIGIL	E	733	742	HLA-B*08:01	0%	Dengue virus 1	ELISPOT	IFNg release	Positive	0	0	[illegible]
180641	MALSIVSLF	NS2a	1297	1305	HLA-B*51:01	0%	Dengue virus 1	ELISPOT	IFNg release	Positive	0	0	[illegible]
419037	RPMFAVGILF	NS2a	1211	1220	HLA-B*35:01	0%	Dengue virus 1	ELISPOT	IFNg release	Positive	0	0	[illegible]
150254	GPLVAGGLL	NS2b	1375	1383	HLA-B*07:02	0%	Dengue virus 1	ELISPOT	IFNg release	Positive	0	0	[illegible]
419005	LLAVSGVYPM	NS2b	1448	1457	HLA-B*15:01	0%	Dengue virus 1	ELISPOT	IFNg release	Positive	0	0	[illegible]
180408	DPASIAARGY	NS3	1766	1775	HLA-B*35:01	0%	Dengue virus 1	ELISPOT	IFNg release	Positive	0	0	[illegible]
180682	NPEIEDDIF	NS3	1650	1658	HLA-B*35:01	0%	Dengue virus 1	ELISPOT	IFNg release	Positive	0	0	[illegible]
183929	IYRILQRGLL	NS3	1497	1506	HLA-B*08:01	0%	Dengue virus 1	ELISPOT	IFNg release	Positive	0	0	[illegible]
32147	KLRTLVLAPTRVVAS	NS3	216	230	HLA class I	0%	Dengue virus 1	ELISPOT	IFNg release	Positive	0	0	[illegible]
419125	AVKSEHTGR	NS3	1722	1730	HLA-A*11:01	0%	Dengue virus 1	ELISPOT	IFNg release	Positive	0	0	[illegible]
419229	IYRILQRGL	NS3	1497	1505	HLA-A*24:02	0%	Dengue virus 1	ELISPOT	IFNg release	Positive	0	0	[illegible]
539750	NVRTLILAPTRVVASEM	NS3	1690	1706	HLA class I	0%	Dengue virus 1	ICS	IFNg release	Positive	0	0	[illegible]
539753	NYNMIIMDEAHFTDPA	NS3	1753	1768	HLA class I	0%	Dengue virus 1	ICS	IFNg release	Positive	0	0	[illegible]
62184	SVKKDLISYGGGWRF	NS3	71	85	HLA class I	0%	Dengue virus 1	ELISPOT	IFNg release	Positive	0	0	[illegible]
65549	TPEGIIPSM	NS3	1975	1983	HLA-B*53:01	0%	Dengue virus 1	51 chromium	cytotoxicity	Positive	0	0	[illegible]
150674	TVMDIISRR	NS5	3080	3088	HLA-A*11:01	0%	Dengue virus 1	multimer/tetramer	qualitative binding	Positive	0	0	[illegible]
180376	ASSMVNGVVR	NS5	2809	2818	HLA-B*57:01	0%	Dengue virus 1	ELISPOT	IFNg release	Positive	0	0	[illegible]
182554	ALLATSIFK	NS5	3054	3062	HLA-A*11:01	0%	Dengue virus 1	ELISPOT	IFNg release	Positive	0	0	[illegible]
183275	FTNMEVQLIR	NS5	3104	3113	HLA-B*35:01	0%	Dengue virus 1	ELISPOT	IFNg release	Positive	0	0	[illegible]
184787	MEVQLVRQM	NS5	3107	3115	HLA-B*40:01	0%	Dengue virus 1	ELISPOT	IFNg release	Positive	0	0	[illegible]
150255	GPMKLVMAFI	C	43	52	HLA-B*07:02	100%	Dengue virus 1	ELISPOT	IFNg release	Positive	1	1	[illegible]
184835	MKLVMAFIAF	C	44	53	HLA-B*35:01	100%	Dengue virus 1	ELISPOT	IFNg release	Positive	1	1	[illegible]
150378	LLMLVTPSM	PreM	271	279	HLA-B*08:01	100%	Dengue virus 1	ELISPOT	IFNg release	Positive	1	1	[illegible]
16554	FLDLPLPWT	E	213	221	HLA-A2	100%	Dengue virus 1	ICS	IFNg release	Positive	1	1	[illegible]
183421	GLNSRSTSL	E	747	755	HLA-B*08:01	100%	Dengue virus 1	ELISPOT	IFNg release	Positive	1	1	[illegible]
184377	LGLNSRSTSL	E	746	755	HLA-B*08:01	100%	Dengue virus 1	ELISPOT	IFNg release	Positive	1	1	[illegible]
184626	LTDYGALTL	E	456	464	HLA-A*01:01	100%	Dengue virus 1	ELISPOT	IFNg release	Positive	1	1	[illegible]
186551	YENLKYSVIV	E	412	421	HLA-B*40:01	100%	Dengue virus 1	ELISPOT	IFNg release	Positive	1	1	[illegible]
419306	PTLDIELLK	E	319	327	HLA-A*11:01	100%	Dengue virus 1	ELISPOT	IFNg release	Positive	1	1	[illegible]
50596	QEGAMHTAL	E	536	544	HLA-A*02:01	100%	Dengue virus 1	ELISPOT	IFNg release	Positive	1	1	[illegible]
588005	RDFVEGLSGATWVDVVL	E	289	305	HLA class I	100%	Dengue virus 1	ICS	IFNg release	Positive	1	1	[illegible]
590597	VEGLSGATWVDVVLEHG	E	292	308	HLA class I	100%	Dengue virus 1	ICS	IFNg release	Positive-Intermediate	1	1	[illegible]
150258	GPWHLGKLEL	NS1	1041	1050	HLA-B*07:02	100%	Dengue virus 1	ELISPOT	IFNg release	Positive	1	1	[illegible]
183619	HTWTEQYKF	NS1	801	809	HLA-B57	100%	Dengue virus 1	51 chromium	cytotoxicity	Positive	1	1	[illegible]
60104	SPKRLSAAI	NS1	813	821	HLA-B*08:01	100%	Dengue virus 1	ELISPOT	IFNg release	Positive	1	1	[illegible]
183246	FRRLTSREVL	NS2a	1220	1229	HLA-B*08:01	100%	Dengue virus 1	ELISPOT	IFNg release	Positive	1	1	[illegible]
185651	SILLSSLLK	NS2b	1360	1368	HLA-A*11:01	100%	Dengue virus 1	ELISPOT	IFNg release	Positive	1	1	[illegible]
133501	AIVREAIKR	NS3	1682	1690	HLA-A*02:01	100%	Dengue virus 1	ELISPOT	IFNg release	Positive	1	1	[illegible]
150169	DPRRCLKPV	NS3	1900	1908	HLA-B*07:02	100%	Dengue virus 1	ELISPOT	IFNg release	Positive	1	1	[illegible]
150341	KPGTSGSPI	NS3	1606	1614	HLA-B*07:02	100%	Dengue virus 1	ELISPOT	IFNg release	Positive	1	1	[illegible]
150538	RPRWLDART	NS3	2068	2076	HLA-B*07:02	100%	Dengue virus 1	ELISPOT	IFNg release	Positive	1	1	[illegible]
150556	RVIDPRRCLK	NS3	1897	1906	HLA-A*03:01	100%	Dengue virus 1	ELISPOT	IFNg release	Positive	1	1	[illegible]
150643	TPEGIIPALF	NS3	1976	1985	HLA-B*35:01	100%	Dengue virus 1	ELISPOT	IFNg release	Positive	1	1	[illegible]
180373	APTRVVASEM	NS3	1698	1707	HLA class I	100%	Dengue virus 1	ELISPOT	IFNg release	Positive	1	1	[illegible]
180423	EERDIPERSW	NS3	1811	1820	HLA-B*44:02	100%	Dengue virus 1	ELISPOT	IFNg release	Positive	1	1	[illegible]
180472	GEARKTFVEL	NS3	2003	2012	HLA-B*40:01	100%	Dengue virus 1	ELISPOT	IFNg release	Positive	1	1	[illegible]
180670	MPVTVASAAQ	NS3	1923	1932	HLA-B*35:01	100%	Dengue virus 1	ELISPOT	IFNg release	Positive	1	1	[illegible]
182772	DISEMGANF	NS3	1885	1893	HLA-A*26:01	100%	Dengue virus 1	ELISPOT	IFNg release	Positive	1	1	[illegible]
183598	HPGSGKTRRY	NS3	1670	1679	HLA-B*35:01	100%	Dengue virus 1	ELISPOT	IFNg release	Positive	1	1	[illegible]
184316	LEENMDVEIW	NS3	2049	2058	HLA-B*44:03	100%	Dengue virus 1	ELISPOT	IFNg release	Positive	1	1	[illegible]
184896	MPVTVASAA	NS3	1923	1931	HLA-B*35:01	100%	Dengue virus 1	ELISPOT	IFNg release	Positive	1	1	[illegible]
185124	NTPEGIIPA	NS3	1975	1983	HLA-A*68:02	100%	Dengue virus 1	ELISPOT	IFNg release	Positive	1	1	[illegible]
185125	NTPEGIIPAL	NS3	1975	1984	HLA-A*68:02	100%	Dengue virus 1	ELISPOT	IFNg release	Positive	1	1	[illegible]
185327	QYIYMGQPL	NS3	1947	1955	HLA-A*24:02	100%	Dengue virus 1	ELISPOT	IFNg release	Positive	1	1	[illegible]
186284	VLDDGIYRI	NS3	1492	1500	HLA-A*02:01	100%	Dengue virus 1	ELISPOT	IFNg release	Positive	1	1	[illegible]
186603	YSDPLALREF	NS3	2077	2086	HLA-B*35:01	100%	Dengue virus 1	ELISPOT	IFNg release	Positive	1	1	[illegible]
22825	GTSGSPIVNR	NS3	133	142	HLA-B*57:01	100%	Dengue virus 1	51 chromium	cytotoxicity	Positive	1	1	[illegible]
38400	LPAIVREAI	NS3	1680	1688	HLA-B*53:01	100%	Dengue virus 1	ELISPOT	IFNg release	Positive	1	1	[illegible]
38766	LPVWLSYKV	NS3	2018	2026	HLA-B*51:01	100%	Dengue virus 1	ELISPOT	IFNg release	Positive	1	1	[illegible]
39095	LRGEARKTFVELMRR	NS3	526	540	HLA class I	100%	Dengue virus 1	ELISPOT	IFNg release	Positive	1	1	[illegible]
419024	NMIIMDEAHF	NS3	1755	1764	HLA-B*15:01	100%	Dengue virus 1	ELISPOT	IFNg release	Positive	1	1	[illegible]
50894	QGKRLEPSWASVKKDLISYG	NS3	1536	1555	HLA class I	100%	Dengue virus 1	ELISPOT	IFNg release	Positive	1	1	[illegible]
539170	DLMCHATF	NS3	1734	1741	HLA-B*08:01	100%	Dengue virus 1	ICS	IFNg release	Positive	1	1	[illegible]
56309	RVIDPRRCL	NS3	1897	1905	HLA-B*07:02	100%	Dengue virus 1	ELISPOT	IFNg release	Positive	1	1	[illegible]
585710	LAPTRVVASEM	NS3	83	93	HLA class I	100%	Dengue virus 1	ICS	IFNg release	Positive-High	1	1	[illegible]
65546	TPEGIIPAL	NS3	1976	1984	HLA class I	100%	Dengue virus 1	51 chromium	cytotoxicity	Positive	1	1	[illegible]
150649	TPQDNQLAYV	NS4a	2222	2231	HLA-B*07:02	100%	Dengue virus 1	ELISPOT	IFNg release	Positive	1	1	[illegible]
180780	TPQDNQLAY	NS4a	2222	2230	HLA-B*35:01	100%	Dengue virus 1	ELISPOT	IFNg release	Positive	1	1	[illegible]
182912	EFFLMVLLI	NS4a	2206	2214	HLA-A*24:02	100%	Dengue virus 1	ELISPOT	IFNg release	Positive	1	1	[illegible]
183162	FFLMVLLIPE	NS4a	2207	2216	HLA-A*33:01	100%	Dengue virus 1	ELISPOT	IFNg release	Positive	1	1	[illegible]
183813	IPEPDRQRTP	NS4a	2214	2223	HLA-B*35:01	100%	Dengue virus 1	ELISPOT	IFNg release	Positive	1	1	[illegible]
184522	LPDTIETLM	NS4a	2142	2150	HLA-B*35:01	100%	Dengue virus 1	ELISPOT	IFNg release	Positive	1	1	[illegible]
184523	LPDTIETLML	NS4a	2142	2151	HLA-B*35:01	100%	Dengue virus 1	ELISPOT	IFNg release	Positive	1	1	[illegible]
41986	MLLALIAVL	NS4a	2150	2158	HLA-A2	100%	Dengue virus 1	ICS	IFNg release	Positive	1	1	[illegible]
133716	TLYAVATTI	NS4b	2284	2292	HLA-A*02:01	100%	Dengue virus 1	ELISPOT	IFNg release	Positive	1	1	[illegible]
150474	NPLTLTAAV	NS4b	2348	2356	HLA-B*07:02	100%	Dengue virus 1	ELISPOT	IFNg release	Positive	1	1	[illegible]
150579	SPGKFWNTTI	NS4b	2453	2462	HLA-B*07:02	100%	Dengue virus 1	ELISPOT	IFNg release	Positive	1	1	[illegible]
180382	ATGPLTTLW	NS4b	2442	2450	HLA-B*58:01	100%	Dengue virus 1	ELISPOT	IFNg release	Positive	1	1	[illegible]
183594	HPASAWTLYA	NS4b	2278	2287	HLA-B*35:01	100%	Dengue virus 1	ELISPOT	IFNg release	Positive	1	1	[illegible]
186427	VVYDAKFEK	NS4b	2403	2411	HLA-A*11:01	100%	Dengue virus 1	ELISPOT	IFNg release	Positive	1	1	[illegible]
27180	ILLMRTTWA	NS4b	2426	2434	HLA-A2	100%	Dengue virus 1	ICS	IFNg release	Positive	1	1	[illegible]
64286	TIENTTANISLTAIA	NS4b	2300	2314	HLA class I	100%	Dengue virus 1	ELISPOT	IFNg release	Positive	1	1	[illegible]
74558	YLAGAGLAF	NS4b	2474	2482	HLA-B*07:02	100%	Dengue virus 1	ELISPOT	IFNg release	Positive	1	1	[illegible]
133702	SRNSTHEMY	NS5	2703	2711	HLA-A*02:01	100%	Dengue virus 1	ELISPOT	IFNg release	Positive	1	1	[illegible]
14325	ETACLGKSY	NS5	3242	3250	HLA-A*26:01	100%	Dengue virus 1	ELISPOT	IFNg release	Positive	1	1	[illegible]
150308	IPMATYGWNL	NS5	2607	2616	HLA-B*07:02	100%	Dengue virus 1	ELISPOT	IFNg release	Positive	1	1	[illegible]
180411	DTTPFGQQR	NS5	2837	2845	HLA-A*68:01	100%	Dengue virus 1	ELISPOT	IFNg release	Positive	1	1	[illegible]
180548	KAKGSRAIW	NS5	2959	2967	HLA-B*57:01	100%	Dengue virus 1	ELISPOT	IFNg release	Positive	1	1	[illegible]
180571	KPRICTREEF	NS5	2882	2891	HLA-B*07:02	100%	Dengue virus 1	ELISPOT	IFNg release	Positive	1	1	[illegible]
180723	RFLEFEALGF	NS5	2974	2983	HLA-A*23:01	100%	Dengue virus 1	ELISPOT	IFNg release	Positive	1	1	[illegible]
182599	AQMWQLMYF	NS5	3251	3259	HLA-B*15:01	100%	Dengue virus 1	ELISPOT	IFNg release	Positive	1	1	[illegible]
182836	DYMTSMKRF	NS5	3373	3381	HLA-A*24:02	100%	Dengue virus 1	ELISPOT	IFNg release	Positive	1	1	[illegible]
183147	FCSHHFHQL	NS5	3200	3208	HLA-B*08:01	100%	Dengue virus 1	ELISPOT	IFNg release	Positive	1	1	[illegible]
184898	MQRKHGGML	NS5	2689	2697	HLA-B*08:01	100%	Dengue virus 1	ELISPOT	IFNg release	Positive	1	1	[illegible]
184956	MTTEDMLSVW	NS5	3296	3305	HLA-B*58:01	100%	Dengue virus 1	ICS	IFNg release	Positive	1	1	[illegible]
185279	QMQRKHGGML	NS5	2688	2697	HLA-B*08:01	100%	Dengue virus 1	ELISPOT	IFNg release	Positive	1	1	[illegible]
185354	REIVVPCRNQ	NS5	3214	3223	HLA-B*40:01	100%	Dengue virus 1	ELISPOT	IFNg release	Positive	1	1	[illegible]
186176	TWSIHAHHQW	NS5	3286	3295	HLA-A*23:01	100%	Dengue virus 1	ELISPOT	IFNg release	Positive	1	1	[illegible]
65567	TPFGQQRVF	NS5	2839	2847	HLA class I	100%	Dengue virus 1	ELISPOT	IFNg release	Positive	1	1	[illegible]
73076	WSIHAHHQW	NS5	3287	3295	HLA-B*57:01	100%	Dengue virus 1	ELISPOT	IFNg release	Positive	1	1	[illegible]
180637	MAFIAFLRF	C	48	56	HLA-B*35:01	81% - <100%	Dengue virus 1	ELISPOT	IFNg release	Positive	1	1	[illegible]
183175	FIAFLRFLA	C	50	58	HLA-A*68:02	81% - <100%	Dengue virus 1	ELISPOT	IFNg release	Positive	1	1	[illegible]
183220	FNMLKRARNR	C	14	23	HLA-A*33:01	81% - <100%	Dengue virus 1	ELISPOT	IFNg release	Positive	1	1	[illegible]
183596	HPGFTVIALF	PreM	244	253	HLA-B*35:01	81% - <100%	Dengue virus 1	ELISPOT	IFNg release	Positive	1	1	[illegible]
185893	TEVTNPAVL	E	329	337	HLA-B*40:01	81% - <100%	Dengue virus 1	ELISPOT	IFNg release	Positive	1	1	[illegible]
180836	YGGPISQHNY	NS1	1022	1031	HLA-B*35:01	81% - <100%	Dengue virus 1	ELISPOT	IFNg release	Positive	1	1	[illegible]
183245	FQSHQLWATL	NS2a	1267	1276	HLA-B*15:01	81% - <100%	Dengue virus 1	ELISPOT	IFNg release	Positive	1	0	[illegible]
183252	FSLHYAWKTM	NS2a	1286	1295	HLA-B*35:01	81% - <100%	Dengue virus 1	ELISPOT	IFNg release	Positive	1	1	[illegible]
184274	LALMATFKM	NS2a	1202	1210	HLA-B*35:01	81% - <100%	Dengue virus 1	ELISPOT	IFNg release	Positive	1	1	[illegible]
184623	LTDFQSHQL	NS2a	1264	1272	HLA-A*01:01	81% - <100%	Dengue virus 1	ELISPOT	IFNg release	Positive	1	1	[illegible]
184736	MAMVLSIVS	NS2a	1295	1303	HLA-B*35:01	81% - <100%	Dengue virus 1	ELISPOT	IFNg release	Positive	1	1	[illegible]
184856	MLMTGTLAVF	NS2a	1158	1167	HLA-B*35:01	81% - <100%	Dengue virus 1	ELISPOT	IFNg release	Positive	1	1	[illegible]
185468	RPMFAVGLLF	NS2a	1211	1220	HLA-B*35:01	81% - <100%	Dengue virus 1	ELISPOT	IFNg release	Positive	1	1	[illegible]
13346	EMAEALKGMPIRYQT	NS3	231	245	HLA class I	81% - <100%	Dengue virus 1	ELISPOT	IFNg release	Positive	1	1	[illegible]
167826	TSGTYVSAIAQAKASQE	NS3	157	173	HLA class I	81% - <100%	Dengue virus 1	ELISPOT	IFNg release	Positive	1	1	[illegible]
180804	VPNYNMIIM	NS3	1751	1759	HLA-B*51:01	81% - <100%	Dengue virus 1	ELISPOT	IFNg release	Positive	1	1	[illegible]
183223	FPGKTVWFVP	NS3	1830	1839	HLA-B*35:01	81% - <100%	Dengue virus 1	ELISPOT	IFNg release	Positive	1	0	[illegible]
183229	FPQSNAVIQD	NS3	1801	1810	HLA-B*35:01	81% - <100%	Dengue virus 1	ELISPOT	IFNg release	Positive	1	0	[illegible]
184884	MPIRYQTTAV	NS3	1714	1723	HLA-B*35:01	81% - <100%	Dengue virus 1	ELISPOT	IFNg release	Positive	1	1	[illegible]
185741	SPVRVPNYNM	NS3	1747	1756	HLA-B*35:01	81% - <100%	Dengue virus 1	ELISPOT	IFNg release	Positive	1	1	[illegible]
185827	SYKVASEGF	NS3	2023	2031	HLA-A*24:02	81% - <100%	Dengue virus 1	ELISPOT	IFNg release	Positive	1	1	[illegible]
186559	YKVASEGFQY	NS3	2024	2033	HLA-B*35:01	81% - <100%	Dengue virus 1	ELISPOT	IFNg release	Positive	1	1	[illegible]
35237	LDNINTPEGIIPALFEPERE	NS3	496	515	HLA class I	81% - <100%	Dengue virus 1	ELISPOT	IFNg release	Positive	1	1	[illegible]
419124	AVEPGKNPK	NS3	1574	1582	HLA-A*11:01	81% - <100%	Dengue virus 1	ELISPOT	IFNg release	Positive	1	1	[illegible]
539663	MGEAAAIFMTATPPGSV	NS3	1782	1798	HLA class I	81% - <100%	Dengue virus 1	ICS	IFNg release	Positive	1	1	[illegible]
184554	LPQHLTQRAQ	NS4a	2107	2116	HLA-B*35:01	81% - <100%	Dengue virus 1	ELISPOT	IFNg release	Positive	1	1	[illegible]
419128	AYRHAMEEL	NS4a	2134	2142	HLA-A*24:02	81% - <100%	Dengue virus 1	ELISPOT	IFNg release	Positive-High	1	1	[illegible]
41093	MANIFRGSY	NS4b	2466	2474	HLA-B*35:01	81% - <100%	Dengue virus 1	ELISPOT	IFNg release	Positive	1	1	[illegible]
69652	VLMLVAHYA	NS4b	2356	2364	HLA-A2	81% - <100%	Dengue virus 1	ICS	IFNg release	Positive	1	1	[illegible]
1068332	SSWEDVPYL	NS5	3323	3331	HLA-A*02:01	81% - <100%	Dengue virus 1	ELISPOT	IFNg release	Positive	1	1	[illegible]
183828	IPMVTQIAM	NS5	2827	2835	HLA-B*07:02	81% - <100%	Dengue virus 1	ELISPOT	IFNg release	Positive	1	1	[illegible]
184212	KVRKDIPQW	NS5	3178	3186	HLA-B*57:01	81% - <100%	Dengue virus 1	ELISPOT	IFNg release	Positive	1	1	[illegible]
184849	MLLNRFTMA	NS5	24	32	HLA-B*08:01	81% - <100%	Dengue virus 1	ELISPOT	IFNg release	Positive	1	0	[illegible]
186150	TVMDVISRR	NS5	3080	3088	HLA-A*68:01	81% - <100%	Dengue virus 1	ELISPOT	IFNg release	Positive	1	1	[illegible]
419208	GVEGEGLHK	NS5	2998	3006	HLA-A*11:01	81% - <100%	Dengue virus 1	ELISPOT	IFNg release	Positive-High	1	1	[illegible]
5223	ATYGWNLVK	NS5	2610	2618	HLA-A*03:01	81% - <100%	Dengue virus 1	ELISPOT	IFNg release	Positive	1	1	[illegible]
539435	ISGDDCVVKPIDDRFAT	NS5	3152	3168	HLA class I	81% - <100%	Dengue virus 1	ICS	IFNg release	Positive	1	1	[illegible]
539702	MVTQIAMTDTTPFGQQR	NS5	2829	2845	HLA class I	81% - <100%	Dengue virus 1	ICS	IFNg release	Positive	1	1	[illegible]
540100	VVKPIDDRFATALTALN	NS5	3157	3173	HLA class I	81% - <100%	Dengue virus 1	ICS	IFNg release	Positive	1	1	[illegible]
67362	TYGWNLVKL	NS5	2611	2619	HLA-A*24:02	81% - <100%	Dengue virus 1	ELISPOT	IFNg release	Positive	1	1	[illegible]
72563	WHYDEDNPYKTWAYHGSYEV	NS5	2784	2803	HLA class I	81% - <100%	Dengue virus 1	ELISPOT	IFNg release	Positive	1	1	[illegible]
183800	IMNRRKRSV	C	94	102	HLA-B*08:01	51%-80%	Dengue virus 1	ELISPOT	IFNg release	Positive	1	1	[illegible]
184880	MNRRKRSVT	C	95	103	HLA-B*08:01	51%-80%	Dengue virus 1	ELISPOT	IFNg release	Positive	1	1	[illegible]
185102	NRRKRSVTM	C	96	104	HLA-B*08:01	51%-80%	Dengue virus 1	ELISPOT	IFNg release	Positive	1	1	[illegible]
185492	RRKRSVTMLL	C	97	106	HLA-B*08:01	51%-80%	Dengue virus 1	ELISPOT	IFNg release	Positive	1	1	[illegible]
182519	AIKRKLRTL	NS3	1687	1695	HLA-B*08:01	51%-80%	Dengue virus 1	ELISPOT	IFNg release	Positive	1	1	[illegible]
184739	MASSVLLWM	NS4a	2183	2191	HLA-B*35:01	51%-80%	Dengue virus 1	ELISPOT	IFNg release	Positive	1	1	[illegible]
186217	VEDERFWDL	NS5	2918	2926	HLA-B*40:01	51%-80%	Dengue virus 1	ELISPOT	IFNg release	Positive	1	1	[illegible]
184003	KEISSMLNIM	C	86	95	HLA-B*40:01	>0% - 50%	Dengue virus 1	ELISPOT	IFNg release	Positive	0	0	[illegible]
185485	RQMESEGIFL	NS5	3113	3122	HLA-B*15:01	>0% - 50%	Dengue virus 1	ELISPOT	IFNg release	Positive	1	0	[illegible]
62903	TAKWLWGFLSRNKKPRICTR	NS5	2869	2888	HLA class I	>0% - 50%	Dengue virus 1	ELISPOT	IFNg release	Positive	0	0	[illegible]

QTX92800.1	QTX92801.1	QTX92846.1	QTX92418.1	QTX92538.1	ADU03651.1	ANC57592.1	QFS19172.1	QFS19175.1	QTX92861.1	QTX92545.1	QTX92553.1	QTX92559.1	BDB33210.1	QFS19159.1	QFS19189.1	QFS19217.1	QFS19226.1	QPQ49765.1	QTX92595.1	QTX92612.1	QTX92670.1	QTX92745.1	QTX92454.1	QTX92462.1	QTX92576.1	QTX92577.1	QFS19151.1	QTX92707.1	QTX92713.1	QTX92716.1	QTX92718.1	QTX92724.1	QTX92857.1	QTX92428.1	QTX92489.1	ANC57585.1	ANC57583.1	QFS19155.1	DENV-1 constructs of vaccines					
																																							TV003	TDEN	DPIV	Dengvaxia	DENVax	TVDV
0	0	0	0	0	0	0	0	0	0	0	0	0	0	0	0	0	0	0	0	0	0	0	0	0	0	0	0	0	0	0	0	0	0	0	0	0	0	0	1	1	1	0	0	0
0	0	0	0	0	0	0	0	0	0	0	0	0	0	0	0	0	0	0	0	0	0	0	0	0	0	0	0	0	0	0	0	0	0	0	0	0	0	0	0	0	0	0	0	0
0	0	0	0	0	0	0	0	0	0	0	0	0	0	0	0	0	0	0	0	0	0	0	0	0	0	0	0	0	0	0	0	0	0	0	0	0	0	0	0	0	0	0	0	0
0	0	0	0	0	0	0	0	0	0	0	0	0	0	0	0	0	0	0	0	0	0	0	0	0	0	0	0	0	0	0	0	0	0	0	0	0	0	0	1	1	1	0	1	1
0	0	0	0	0	0	0	0	0	0	0	0	0	0	0	0	0	0	0	0	0	0	0	0	0	0	0	0	0	0	0	0	0	0	0	0	0	0	0	1	1	1	1	1	1
0	0	0	0	0	0	0	0	0	0	0	0	0	0	0	0	0	0	0	0	0	0	0	0	0	0	0	0	0	0	0	0	0	0	0	0	0	0	0	1	1	1	0	1	1
0	0	0	0	0	0	0	0	0	0	0	0	0	0	0	0	0	0	0	0	0	0	0	0	0	0	0	0	0	0	0	0	0	0	0	0	0	0	0	0	0	0	0	0	0
0	0	0	0	0	0	0	0	0	0	0	0	0	0	0	0	0	0	0	0	0	0	0	0	0	0	0	0	0	0	0	0	0	0	0	0	0	0	0	0	0	0	0	0	0
0	0	0	0	0	0	0	0	0	0	0	0	0	0	0	0	0	0	0	0	0	0	0	0	0	0	0	0	0	0	0	0	0	0	0	0	0	0	0	0	0	0	0	0	0
0	0	0	0	0	0	0	0	0	0	0	0	0	0	0	0	0	0	0	0	0	0	0	0	0	0	0	0	0	0	0	0	0	0	0	0	0	0	0	0	0	0	0	0	0
0	0	0	0	0	0	0	0	0	0	0	0	0	0	0	0	0	0	0	0	0	0	0	0	0	0	0	0	0	0	0	0	0	0	0	0	0	0	0	1	1	1	0	0	0
0	0	0	0	0	0	0	0	0	0	0	0	0	0	0	0	0	0	0	0	0	0	0	0	0	0	0	0	0	0	0	0	0	0	0	0	0	0	0	0	0	0	0	0	0
0	0	0	0	0	0	0	0	0	0	0	0	0	0	0	0	0	0	0	0	0	0	0	0	0	0	0	0	0	0	0	0	0	0	0	0	0	0	0	1	1	1	0	0	0
0	0	0	0	0	0	0	0	0	0	0	0	0	0	0	0	0	0	0	0	0	0	0	0	0	0	0	0	0	0	0	0	0	0	0	0	0	0	0	1	1	1	0	0	0
0	0	0	0	0	0	0	0	0	0	0	0	0	0	0	0	0	0	0	0	0	0	0	0	0	0	0	0	0	0	0	0	0	0	0	0	0	0	0	0	0	0	0	0	0
0	0	0	0	0	0	0	0	0	0	0	0	0	0	0	0	0	0	0	0	0	0	0	0	0	0	0	0	0	0	0	0	0	0	0	0	0	0	0	1	1	1	0	0	0
0	0	0	0	0	0	0	0	0	0	0	0	0	0	0	0	0	0	0	0	0	0	0	0	0	0	0	0	0	0	0	0	0	0	0	0	0	0	0	0	0	0	0	0	0
0	0	0	0	0	0	0	0	0	0	0	0	0	0	0	0	0	0	0	0	0	0	0	0	0	0	0	0	0	0	0	0	0	0	0	0	0	0	0	1	1	1	0	0	0
0	0	0	0	0	0	0	0	0	0	0	0	0	0	0	0	0	0	0	0	0	0	0	0	0	0	0	0	0	0	0	0	0	0	0	0	0	0	0	1	1	1	0	0	0
0	0	0	0	0	0	0	0	0	0	0	0	0	0	0	0	0	0	0	0	0	0	0	0	0	0	0	0	0	0	0	0	0	0	0	0	0	0	0	0	0	0	0	0	0
0	0	0	0	0	0	0	0	0	0	0	0	0	0	0	0	0	0	0	0	0	0	0	0	0	0	0	0	0	0	0	0	0	0	0	0	0	0	0	0	0	0	0	0	0
0	0	0	0	0	0	0	0	0	0	0	0	0	0	0	0	0	0	0	0	0	0	0	0	0	0	0	0	0	0	0	0	0	0	0	0	0	0	0	1	1	1	0	0	0
[illegible]	[illegible]	[illegible]	[illegible]	[illegible]	[illegible]	[illegible]	[illegible]	[illegible]	[illegible]	[illegible]	[illegible]	[illegible]	[illegible]	[illegible]	[illegible]	[illegible]	[illegible]	[illegible]	[illegible]	[illegible]	[illegible]	[illegible]	[illegible]	[illegible]	[illegible]	[illegible]	[illegible]	[illegible]	[illegible]	[illegible]	[illegible]	[illegible]	[illegible]	[illegible]	[illegible]	[illegible]	[illegible]	[illegible]	[illegible]	[illegible]	[illegible]	[illegible]	[illegible]	[illegible]

CD8 DENV-2 Epitopes

											NCBI IDs of DENV-2 circulating strains from Thailand, (100% identity=1,green ; else 0, red)													DENV-2 constructs of vaccines					
Epitope_ID	AA_Seq	Protein_Name	Start	End	Allele	step	Serotype	Method/Technique	Assay Group	Qualitative Measure	ANC57599.1	QXY23100.1	QXY23099.1	BBG31508.1	QBK46950.1	ANC57598.1	BBG31503.1	BBG31505.1	QFS19150.1	QBA29681.1	QXY23103.1	BBG31506.1	BBG31507.1	TV003	TDEN	DPIV	Dengvaxia	DENVax	TVDV
185104	NRRRRTAGV	C	96	104	HLA-B*08:01	0%	Dengue virus 2	ELISPOT	IFNg release	Positive	0	0	0	0	0	0	0	0	0	0	0	0	0	0	0	0	0	0	0
185399	RKKARSTPF	C	5	13	HLA-B*08:01	0%	Dengue virus 2	ELISPOT	IFNg release	Positive	0	0	0	0	0	0	0	0	0	0	0	0	0	0	0	0	0	0	0
186264	VGRQEKGKSL	PreM	128	137	HLA-B*08:01	0%	Dengue virus 2	ELISPOT	IFNg release	Positive	0	0	0	0	0	0	0	0	0	0	0	0	0	0	0	0	0	0	0
184300	LATLRKYCI	E	53	61	HLA-B*08:01	0%	Dengue virus 2	ELISPOT	IFNg release	Positive	0	0	0	0	0	0	0	0	0	0	0	0	0	0	0	0	0	0	0
184467	LLQMEDKAWL	E	478	487	HLA-B*08:01	0%	Dengue virus 2	ELISPOT	IFNg release	Positive	0	0	0	0	0	0	0	0	0	0	0	0	0	0	0	0	0	0	0
150448	MMATIGIAL	NS2a	1229	1237	HLA-B*35:01	0%	Dengue virus 2	ELISPOT	IFNg release	Positive	0	0	0	0	0	0	0	0	0	0	0	0	0	0	0	0	0	0	0
150449	MMATIGIALL	NS2a	1229	1238	HLA-A*02:01	0%	Dengue virus 2	ELISPOT	IFNg release	Positive	0	0	0	0	0	0	0	0	0	0	0	0	0	0	0	0	0	0	0
182687	AYRIKQRGIL	NS3	1497	1506	HLA-B*08:01	0%	Dengue virus 2	ELISPOT	IFNg release	Positive	0	0	0	0	0	0	0	0	0	0	0	0	0	0	0	0	0	0	0
182892	EENMEVEIW	NS3	2049	2057	HLA-B*44:02	0%	Dengue virus 2	ELISPOT	IFNg release	Positive	0	0	0	0	0	0	0	0	0	0	0	0	0	0	0	0	0	0	0
184318	LEENMEVEIW	NS3	2048	2057	HLA-B*44:02	0%	Dengue virus 2	ELISPOT	IFNg release	Positive	0	0	0	0	0	0	0	0	0	0	0	0	0	0	0	0	0	0	0
186211	VDRKGKVVGL	NS3	1615	1624	HLA-B*08:01	0%	Dengue virus 2	ELISPOT	IFNg release	Positive	0	0	0	0	0	0	0	0	0	0	0	0	0	0	0	0	0	0	0
186301	VLMHRGKRI	NS3	1532	1540	HLA-B*08:01	0%	Dengue virus 2	ELISPOT	IFNg release	Positive	0	0	0	0	0	0	0	0	0	0	0	0	0	0	0	0	0	0	0
22816	GTSGSPIADK	NS3	1608	1617	HLA-A*11:01	0%	Dengue virus 2	multimer/tetramer	qualitative binding	Positive	0	0	0	0	0	0	0	0	0	0	0	0	0	0	0	0	0	0	0
22817	GTSGSPIADKK	NS3	1608	1618	HLA-A11	0%	Dengue virus 2	ELISPOT	IFNg release	Positive	0	0	0	0	0	0	0	0	0	0	0	0	0	0	0	0	0	0	0
22824	GTSGSPIVDR	NS3	1608	1617	HLA-A*11:01	0%	Dengue virus 2	51 chromium	cytotoxicity	Positive	0	0	0	0	0	0	0	0	0	0	0	0	0	0	0	0	0	0	0
46575	NYADRKWCF	NS3	2031	2039	HLA-A24	0%	Dengue virus 2	51 chromium	cytotoxicity	Positive	0	0	0	0	0	0	0	0	0	0	0	0	0	0	0	0	0	0	0
65548	TPEGIIPSL	NS3	1975	1983	HLA-B35	0%	Dengue virus 2	51 chromium	cytotoxicity	Positive	0	0	0	0	0	0	0	0	0	0	0	0	0	0	0	0	0	0	0
32939	KPWDIIPMV	NS5	2821	2829	HLA-B*55:02	0%	Dengue virus 2	51 chromium	cytotoxicity	Positive	0	0	0	0	0	0	0	0	0	0	0	0	0	0	0	0	0	0	0
32941	KPWDVLPMV	NS5	2821	2829	HLA class I	0%	Dengue virus 2	51 chromium	cytotoxicity	Positive	0	0	0	0	0	0	0	0	0	0	0	0	0	0	0	0	0	0	0
27061	ILGDTAWDFG	E	96	105	HLA class I	100%	Dengue virus 2	ELISPOT	IFNg release	Positive	1	1	1	1	1	1	1	1	1	1	1	1	1	1	1	1	1	1	0
19225	GEDGCWYGM	NS1	1100	1108	HLA-B*40:01	100%	Dengue virus 2	ELISPOT	IFNg release	Positive	1	1	1	1	1	1	1	1	1	1	1	1	1	0	1	1	0	1	0
180605	LMCHATFTM	NS3	1734	1742	HLA-B*15:01	100%	Dengue virus 2	ELISPOT	IFNg release	Positive	1	1	1	1	1	1	1	1	1	1	1	1	1	0	1	1	0	1	0
67210	TVWFVPSIK	NS3	358	366	HLA class I	100%	Dengue virus 2	ELISPOT	IFNg release	Positive	1	1	1	1	1	1	1	1	1	1	1	1	1	0	1	1	0	1	0
180514	IAVSMANIF	NS4b	2460	2468	HLA-B*35:01	100%	Dengue virus 2	ELISPOT	IFNg release	Positive	1	1	1	1	1	1	1	1	1	1	1	1	1	0	1	1	0	1	0
180443	ETACLGKSYA	NS5	3241	3250	HLA-A*26:01	100%	Dengue virus 2	ELISPOT	IFNg release	Positive	1	1	1	1	1	1	1	1	1	1	1	1	1	0	1	1	0	1	0
180488	GPGHEEPIPM	NS5	2598	2607	HLA-B*35:01	100%	Dengue virus 2	ELISPOT	IFNg release	Positive	1	1	1	1	1	1	1	1	1	1	1	1	1	0	1	1	0	1	0
184131	KREDQWCGSL	NS5	3332	3341	HLA-B*08:01	100%	Dengue virus 2	ELISPOT	IFNg release	Positive	1	1	1	1	1	1	1	1	1	1	1	1	1	0	1	1	0	1	0
53476	REDQWCGSL	NS5	3333	3341	HLA-B*40:01	100%	Dengue virus 2	ELISPOT	IFNg release	Positive	1	1	1	1	1	1	1	1	1	1	1	1	1	0	1	1	0	1	0
110018	VAFLRFLTI	C	51	59	HLA-B*08:01	81% - <100%	Dengue virus 2	ELISPOT	IFNg release	Positive	1	1	1	1	1	1	1	1	0	1	0	1	1	0	1	1	0	1	0
150252	GPLKLFMAL	C	42	50	HLA-B*08:01	81% - <100%	Dengue virus 2	ELISPOT	IFNg release	Positive	1	1	1	1	1	1	1	1	0	1	1	1	1	0	1	1	0	1	0
184378	LGMLQGRGPL	C	35	44	HLA-B*08:01	81% - <100%	Dengue virus 2	ELISPOT	IFNg release	Positive	1	1	1	1	1	1	1	1	0	1	1	1	1	0	1	1	0	1	0
184643	LTIPPTAGIL	C	21	30	HLA-B*08:01	81% - <100%	Dengue virus 2	ELISPOT	IFNg release	Positive	0	1	1	1	1	1	1	1	0	1	1	1	1	0	1	1	0	1	0
186354	VQQLTKRFSL	C	26	35	HLA-B*08:01	81% - <100%	Dengue virus 2	ELISPOT	IFNg release	Positive	1	1	1	1	1	1	1	1	0	1	1	1	1	0	1	1	0	1	0
150634	TLMAMDLGEL	PreM	149	158	HLA-A*02:01	81% - <100%	Dengue virus 2	ELISPOT	IFNg release	Positive	1	1	1	1	1	1	1	1	0	1	1	1	1	1	1	1	1	1	1
150115	AIYGAAFSGV	E	722	731	HLA-A*02:01	81% - <100%	Dengue virus 2	ELISPOT	IFNg release	Positive	1	1	1	1	1	1	1	1	0	1	1	1	1	1	1	1	1	1	0
150319	ITEAELTGY	E	450	458	HLA-A*01:01	81% - <100%	Dengue virus 2	ELISPOT	IFNg release	Positive	1	1	1	1	1	1	1	1	0	1	1	1	1	1	1	1	1	1	1
150399	LQMENKAWLV	E	231	240	HLA-B*08:01	81% - <100%	Dengue virus 2	ELISPOT	IFNg release	Positive	1	1	1	1	1	1	1	1	0	1	1	1	1	1	1	1	1	1	1
150456	MSYSMCTGKF	E	577	586	HLA-B*35:01	81% - <100%	Dengue virus 2	ELISPOT	IFNg release	Positive	1	1	1	1	1	1	1	1	0	1	1	1	1	1	1	1	1	1	1
150529	RLITVNPIV	E	630	638	HLA-A*02:01	81% - <100%	Dengue virus 2	ELISPOT	IFNg release	Positive	1	1	1	1	1	1	1	1	0	1	1	1	1	1	1	1	1	1	1
150531	RLRMDKLQL	E	566	574	HLA-B*08:01	81% - <100%	Dengue virus 2	ELISPOT	IFNg release	Positive	1	1	1	1	1	1	1	1	0	1	1	1	1	1	1	1	1	1	1
150578	SPCKIPFEIM	E	611	620	HLA-B*35:01	81% - <100%	Dengue virus 2	ELISPOT	IFNg release	Positive	1	1	1	1	1	0	1	1	0	1	1	1	1	1	1	1	1	1	1
150725	WLVHRQWFL	E	486	494	HLA-B*08:01	81% - <100%	Dengue virus 2	ELISPOT	IFNg release	Positive	1	1	1	1	1	1	1	1	0	1	1	1	1	1	1	1	1	1	1
16553	FLDLPLPWL	E	213	221	HLA-A2	81% - <100%	Dengue virus 2	ICS	TNF release	Positive	1	1	1	1	1	1	1	1	0	1	1	1	1	1	1	1	1	1	1
180453	FGAIYGAAF	E	720	728	HLA-B*35:01	81% - <100%	Dengue virus 2	ELISPOT	IFNg release	Positive	1	1	1	1	1	1	1	1	0	1	1	1	1	1	1	1	1	1	0
180528	IPFEIMDLEK	E	615	624	HLA-B*35:01	81% - <100%	Dengue virus 2	ELISPOT	IFNg release	Positive	1	1	1	1	1	0	1	1	0	1	1	1	1	1	1	1	1	1	1
180618	LPLPWLPGAD	E	496	505	HLA-B*35:01	81% - <100%	Dengue virus 2	ELISPOT	IFNg release	Positive	1	1	1	1	1	1	1	1	0	1	1	1	1	1	1	1	1	1	1
180726	RMAILGDTAWDFGSL	E	691	705	HLA-B7	81% - <100%	Dengue virus 2	ICS	IFNg release	Positive	1	1	1	1	1	1	1	1	0	1	1	1	1	1	1	1	1	1	0
180816	WGNGCGLFGKGGIVT	E	381	395	HLA class I	81% - <100%	Dengue virus 2	ICS	IFNg release	Positive	1	1	1	1	1	1	1	1	0	1	1	1	1	1	1	1	1	1	1
183310	GAAFSGVSW	E	725	733	HLA-B*58:01	81% - <100%	Dengue virus 2	ELISPOT	IFNg release	Positive	1	1	1	1	1	1	1	1	0	1	1	1	1	1	1	1	1	1	0
183437	GMNSRSTSL	E	747	755	HLA-B*08:01	81% - <100%	Dengue virus 2	ELISPOT	IFNg release	Positive	1	1	1	1	1	1	1	1	0	1	1	1	1	1	1	1	1	1	0
183708	IGMNSRSTSL	E	746	755	HLA-B*08:01	81% - <100%	Dengue virus 2	ELISPOT	IFNg release	Positive	1	1	1	1	1	1	1	1	0	1	1	1	1	1	1	1	1	1	0
183840	IQKETLVTF	E	512	520	HLA-B*15:01	81% - <100%	Dengue virus 2	ELISPOT	IFNg release	Positive	1	1	1	1	1	1	1	1	0	1	1	1	1	1	1	1	1	1	1
183841	IQMSSGNLLF	E	550	559	HLA-A*23:01	81% - <100%	Dengue virus 2	ELISPOT	IFNg release	Positive	1	1	1	1	1	1	1	1	0	1	1	1	1	1	1	1	1	1	1
184571	LQLKGMSYSM	E	572	581	HLA-B*08:01	81% - <100%	Dengue virus 2	ELISPOT	IFNg release	Positive	1	1	1	1	1	1	1	1	0	1	1	1	1	1	1	1	1	1	1
184899	MRGAKRMAI	E	686	694	HLA-B*08:01	81% - <100%	Dengue virus 2	ELISPOT	IFNg release	Positive	1	1	1	1	1	1	1	1	0	1	1	1	1	1	1	1	1	1	0
184900	MRGAKRMAIL	E	686	695	HLA-B*08:01	81% - <100%	Dengue virus 2	ELISPOT	IFNg release	Positive	1	1	1	1	1	1	1	1	0	1	1	1	1	1	1	1	1	1	0
184920	MSSGNLLFTG	E	552	561	HLA-B*58:01	81% - <100%	Dengue virus 2	ELISPOT	IFNg release	Positive	1	1	1	1	1	1	1	1	0	1	1	1	1	1	1	1	1	1	1
185758	SRSTSLSVSL	E	750	759	HLA-B*08:01	81% - <100%	Dengue virus 2	ELISPOT	IFNg release	Positive	1	1	1	1	0	1	1	1	0	1	1	1	1	1	1	1	1	0	0
185831	SYSMCTGKF	E	578	586	HLA-A*23:01	81% - <100%	Dengue virus 2	ELISPOT	IFNg release	Positive	1	1	1	1	1	1	1	1	0	1	1	1	1	1	1	1	1	1	1
41407	MENKAWLVHRQWFLD	E	201	215	HLA-B*37:01	81% - <100%	Dengue virus 2	ICS	IFNg release	Positive	1	1	1	1	1	1	1	1	0	1	1	1	1	1	1	1	1	1	1
51388	QLKGMSYSM	E	573	581	HLA-B*08:01	81% - <100%	Dengue virus 2	ELISPOT	IFNg release	Positive	1	1	1	1	1	1	1	1	0	1	1	1	1	1	1	1	1	1	1
583702	GVSGGSWVDIVLEHGSCV	E	14	31	HLA class I	81% - <100%	Dengue virus 2	ICS	IFNg release	Positive-Low	1	1	1	1	1	1	1	1	0	1	1	1	1	1	1	1	1	1	1
180689	NRAVHADMGYWIESA	NS1	966	980	HLA class I	81% - <100%	Dengue virus 2	ICS	IFNg release	Positive	1	1	1	1	1	1	1	1	0	1	1	1	1	0	1	1	0	1	0
182938	EKEENLVNSL	NS1	1115	1124	HLA-B*08:01	81% - <100%	Dengue virus 2	ELISPOT	IFNg release	Positive	1	1	0	1	1	1	1	1	0	1	1	1	1	0	1	1	0	1	0
184512	LMWKQITPEL	NS1	841	850	HLA-B*08:01	81% - <100%	Dengue virus 2	ELISPOT	IFNg release	Positive	1	1	1	1	1	1	1	1	0	1	1	1	1	0	1	1	0	1	0
185342	RAVHADMGY	NS1	192	200	HLA-B*58:01	81% - <100%	Dengue virus 2	ELISPOT	IFNg release	Positive	1	1	1	1	1	1	1	1	0	1	1	1	1	0	1	1	0	1	0
185465	RPLKEKEENL	NS1	1111	1120	HLA-B*08:01	81% - <100%	Dengue virus 2	ELISPOT	IFNg release	Positive	1	1	0	1	1	1	1	1	0	1	1	1	1	0	1	1	0	1	0
150206	FLEEMLRTRV	NS2a	1131	1140	HLA-A*02:01	81% - <100%	Dengue virus 2	ELISPOT	IFNg release	Positive	1	1	1	1	1	1	1	1	0	1	1	1	1	0	1	1	0	1	0
150300	ILLVAVSFV	NS2a	1146	1154	HLA-A*02:01	81% - <100%	Dengue virus 2	ELISPOT	IFNg release	Positive	1	1	1	1	1	1	1	1	0	1	1	1	1	0	1	1	0	1	0
150391	LMMTTIGIVL	NS2a	1228	1237	HLA-B*08:01	81% - <100%	Dengue virus 2	ELISPOT	IFNg release	Positive	1	1	1	1	1	1	1	1	0	1	1	1	1	0	1	1	0	1	0
150415	LTDALALGM	NS2a	1250	1258	HLA-A*01:01	81% - <100%	Dengue virus 2	ELISPOT	IFNg release	Positive	1	1	1	1	1	1	1	1	0	1	1	1	1	0	1	1	0	1	0
150540	RPTFAAGLLL	NS2a	1211	1220	HLA-B*07:02	81% - <100%	Dengue virus 2	ELISPOT	IFNg release	Positive	1	1	1	1	1	1	1	1	0	1	1	1	1	0	1	1	0	1	0
180735	RVGTKHAILLVAVSF	NS2a	1153	1167	HLA class I	81% - <100%	Dengue virus 2	ICS	IFNg release	Positive	1	1	1	1	1	1	1	1	0	1	1	1	1	0	1	1	0	1	0
183825	IPLALTIKGL	NS2a	1319	1328	HLA-B*08:01	81% - <100%	Dengue virus 2	ELISPOT	IFNg release	Positive	1	1	1	1	1	1	1	1	0	1	1	1	1	0	1	1	0	1	0
184864	MLRTRVGTK	NS2a	1149	1157	HLA-B*08:01	81% - <100%	Dengue virus 2	ELISPOT	IFNg release	Positive	1	1	1	1	1	1	1	1	0	1	1	1	1	0	1	1	0	1	0
186041	TRVGTKHAIL	NS2a	1152	1161	HLA-B*08:01	81% - <100%	Dengue virus 2	ELISPOT	IFNg release	Positive	1	1	1	1	1	1	1	1	0	1	1	1	1	0	1	1	0	1	0
150101	AAAWYLWEV	NS2b	1463	1471	HLA-A*02:01	81% - <100%	Dengue virus 2	ELISPOT	IFNg release	Positive	0	1	1	1	1	1	1	1	0	1	1	1	1	0	1	1	0	1	0
150243	GLLTVCYVL	NS2b	1381	1389	HLA-A*02:01	81% - <100%	Dengue virus 2	ELISPOT	IFNg release	Positive	1	1	1	1	1	1	1	1	0	1	1	1	1	0	1	1	0	1	0
150304	IMAVGMVSI	NS2b	1353	1361	HLA-A*02:01	81% - <100%	Dengue virus 2	ELISPOT	IFNg release	Positive	1	1	1	1	1	1	1	1	0	1	1	1	1	0	1	1	0	1	0
150584	SPILSITISE	NS2b	1416	1425	HLA-B*35:01	81% - <100%	Dengue virus 2	ELISPOT	IFNg release	Positive	1	1	1	1	1	1	1	1	0	1	1	1	1	0	1	1	0	1	0
150610	TAAAWYLWEV	NS2b	117	126	HLA-A*02:01	81% - <100%	Dengue virus 2	ELISPOT	IFNg release	Positive	0	1	1	1	1	1	1	1	0	1	1	1	1	0	1	1	0	1	0
180529	IPITAAAWY	NS2b	1459	1467	HLA-B*35:01	81% - <100%	Dengue virus 2	ELISPOT	IFNg release	Positive	0	1	1	1	1	1	1	1	0	1	1	1	1	0	1	1	0	1	0
180531	IPMTGPLVAG	NS2b	1371	1380	HLA-B*35:01	81% - <100%	Dengue virus 2	ELISPOT	IFNg release	Positive	1	1	1	1	1	1	1	1	0	1	1	1	1	0	1	1	0	1	0
180646	MAVGMVSIL	NS2b	1354	1362	HLA-B*35:01	81% - <100%	Dengue virus 2	ELISPOT	IFNg release	Positive	1	1	1	1	1	1	1	1	0	1	1	1	1	0	1	1	0	1	0
183779	IMAVGMVSIL	NS2b	1353	1362	HLA-B*08:01	81% - <100%	Dengue virus 2	ELISPOT	IFNg release	Positive	1	1	1	1	1	1	1	1	0	1	1	1	1	0	1	1	0	1	0
183819	IPITAAAWYL	NS2b	1459	1468	HLA-B*08:01	81% - <100%	Dengue virus 2	ELISPOT	IFNg release	Positive	0	1	1	1	1	1	1	1	0	1	1	1	1	0	1	1	0	1	0
10643	DVKKDLISY	NS3	71	79	HLA-B62	81% - <100%	Dengue virus 2	51 chromium	cytotoxicity	Positive	1	1	0	1	1	1	1	1	0	1	1	1	1	0	1	1	0	1	0
11063	EAHFTDPASIAARGY	NS3	285	299	HLA class I	81% - <100%	Dengue virus 2	ELISPOT	IFNg release	Positive	1	1	1	1	1	1	1	1	0	1	1	1	1	0	1	1	0	1	0
11125	EALRGLPIR	NS3	233	241	HLA class I	81% - <100%	Dengue virus 2	ELISPOT	IFNg release	Positive	1	1	1	1	1	1	1	1	0	1	1	1	1	0	1	1	0	1	0

124391	KTFVDLMRR	NS3	2006	2014	HLA-A*11:01	81% - <100%	Dengue virus 2	ELISPOT	IFNg release	Positive	1	1	1	1	1	1	1	1	0	1	1	1	1	0	1	1	0	1	0
13358	EMEEALRGLPIRYQT	NS3	230	244	HLA class I	81% - <100%	Dengue virus 2	ELISPOT	IFNg release	Positive	1	1	1	1	1	1	1	1	0	1	1	1	1	0	1	1	0	1	0
136890	TPPGSRDPF	NS3	1792	1800	HLA-B*35:01	81% - <100%	Dengue virus 2	ELISPOT	IFNg release	Positive	1	1	1	1	1	1	1	1	0	1	1	1	1	0	1	1	0	1	0
150137	APTRVVAAEM	NS3	222	231	HLA-B7	81% - <100%	Dengue virus 2	multimer/tetramer	qualitative binding	Positive	1	1	1	1	1	1	1	1	0	1	1	1	1	0	1	1	0	1	0
150167	DLMRRGDLPV	NS3	2010	2019	HLA-A*02:01	81% - <100%	Dengue virus 2	ELISPOT	IFNg release	Positive	1	1	1	1	1	1	1	1	0	1	1	1	1	0	1	1	0	1	0
150450	MPVTHSSAA	NS3	1922	1930	HLA-B*08:01	81% - <100%	Dengue virus 2	ELISPOT	IFNg release	Positive	1	1	1	1	1	1	1	1	0	1	1	1	1	0	1	1	0	1	0
150451	MPVTHSSAAQ	NS3	1922	1931	HLA-B*35:01	81% - <100%	Dengue virus 2	ELISPOT	IFNg release	Positive	1	1	1	1	1	1	1	1	0	1	1	1	1	0	1	1	0	1	0
150526	RIYSDPLALK	NS3	2074	2083	HLA-A*11:01	81% - <100%	Dengue virus 2	ELISPOT	IFNg release	Positive	1	1	1	1	1	1	1	1	0	1	1	1	1	0	1	1	0	1	0
150595	SQIGAGVYK	NS3	1509	1517	HLA-A*11:01	81% - <100%	Dengue virus 2	ELISPOT	IFNg release	Positive	1	1	1	1	1	1	1	1	0	1	1	1	1	0	1	1	0	1	0
150644	TPEGIIPSMF	NS3	1975	1984	HLA-B*53:01	81% - <100%	Dengue virus 2	ELISPOT	IFNg release	Positive	1	1	1	1	1	1	1	1	0	1	1	1	1	0	1	1	0	1	0
150693	VPNYNLIIM	NS3	1750	1758	HLA-B*07:02	81% - <100%	Dengue virus 2	ELISPOT	IFNg release	Positive	1	1	1	1	1	1	1	1	0	1	1	1	1	0	1	1	0	1	0
150740	YLPAIVREA	NS3	1678	1686	HLA-A*02:01	81% - <100%	Dengue virus 2	ELISPOT	IFNg release	Positive	1	1	0	1	1	1	1	1	1	1	1	1	1	0	1	1	0	1	0
180360	AAEGINYADRRWCFD	NS3	2026	2040	HLA-A24	81% - <100%	Dengue virus 2	ELISPOT	IFNg release	Positive	1	1	1	1	1	1	1	1	0	1	1	1	1	0	1	1	0	1	0
180407	DNINTPEGIIPSMFE	NS3	496	510	HLA-B35	81% - <100%	Dengue virus 2	ELISPOT	IFNg release	Positive	1	1	1	1	1	1	1	1	0	1	1	1	1	0	1	1	0	1	0
180428	EGEWKEGEEVQVLAL	NS3	1561	1575	HLA class I	81% - <100%	Dengue virus 2	ICS	IFNg release	Positive	1	1	1	1	1	1	1	1	0	1	1	1	1	0	1	1	0	1	0
180440	EREIPERSWNSGHEW	NS3	336	350	HLA-B*57:01	81% - <100%	Dengue virus 2	ELISPOT	IFNg release	Positive	1	1	1	1	1	1	1	1	0	1	1	1	1	0	1	0	0	1	0
180471	GEARKTFVDL	NS3	2002	2011	HLA-B*40:01	81% - <100%	Dengue virus 2	ELISPOT	IFNg release	Positive	1	1	1	1	1	1	1	1	0	1	1	1	1	0	1	1	0	1	0
180503	HPGAGKTKRY	NS3	1669	1678	HLA-B*35:01	81% - <100%	Dengue virus 2	ELISPOT	IFNg release	Positive	1	1	1	1	1	1	1	1	0	1	1	1	1	0	1	1	0	1	0
180507	HTGREIVDLMCHATF	NS3	1726	1740	HLA class I	81% - <100%	Dengue virus 2	ELISPOT	IFNg release	Positive	1	1	1	1	1	1	1	1	0	1	1	1	1	0	1	1	0	1	0
180575	KRYLPAIVREAIKRG	NS3	201	215	HLA-A*31:01	81% - <100%	Dengue virus 2	ELISPOT	IFNg release	Positive	1	1	0	1	1	1	1	1	0	1	1	1	1	0	1	1	0	1	0
180589	LDARIYSDPLALKEF	NS3	596	610	HLA class I	81% - <100%	Dengue virus 2	ELISPOT	IFNg release	Positive	1	1	1	1	1	1	1	1	0	1	1	1	1	0	1	1	0	1	0
180617	LPIRYQTPAI	NS3	1713	1722	HLA-B*35:01	81% - <100%	Dengue virus 2	ELISPOT	IFNg release	Positive	1	1	1	1	1	1	1	1	0	1	1	1	1	0	1	1	0	1	0
180647	MEEALRGLPIRYQTP	NS3	1706	1720	HLA class I	81% - <100%	Dengue virus 2	ELISPOT	IFNg release	Positive	1	1	1	1	1	1	1	1	0	1	1	1	1	0	1	1	0	1	0
180724	RGEARKTFVDLMRRG	NS3	2001	2015	HLA class I	81% - <100%	Dengue virus 2	ELISPOT	IFNg release	Positive	1	1	1	1	1	1	1	1	0	1	1	1	1	0	1	1	0	1	0
180734	RSWNSGHEW	NS3	342	350	HLA-B*58:01	81% - <100%	Dengue virus 2	ICS	IFNg release	Positive	1	1	1	1	1	1	1	1	0	1	1	1	1	0	1	0	0	1	0
180756	STRVEMGEAAGIFMT	NS3	301	315	HLA-B*40:06	81% - <100%	Dengue virus 2	ELISPOT	IFNg release	Positive-Low	1	1	1	1	1	1	1	1	0	1	1	1	1	0	1	1	0	1	0
182848	EARKTFVDL	NS3	2003	2011	HLA-B*08:01	81% - <100%	Dengue virus 2	ELISPOT	IFNg release	Positive	1	1	1	1	1	1	1	1	0	1	1	1	1	0	1	1	0	1	0
182898	EEREIPERSW	NS3	1810	1819	HLA-B*44:03	81% - <100%	Dengue virus 2	ELISPOT	IFNg release	Positive	1	1	1	1	1	1	1	1	0	1	1	1	1	0	1	1	0	1	0
183347	GERKKLKPRW	NS3	2061	2070	HLA-B*44:03	81% - <100%	Dengue virus 2	ELISPOT	IFNg release	Positive	1	1	1	1	1	1	1	1	0	1	1	1	1	0	1	1	0	1	0
183724	IKRGLRTLIL	NS3	1687	1696	HLA-B*08:01	81% - <100%	Dengue virus 2	ELISPOT	IFNg release	Positive	1	1	1	1	1	1	1	1	0	1	1	1	1	0	1	1	0	1	0
185200	PRRCMKPVIL	NS3	1900	1909	HLA-B*08:01	81% - <100%	Dengue virus 2	ELISPOT	IFNg release	Positive	1	1	1	1	1	1	1	1	0	1	1	1	1	0	1	1	0	1	0
186242	VEMGEAAGIF	NS3	1779	1788	HLA-B*44:03	81% - <100%	Dengue virus 2	ELISPOT	IFNg release	Positive	1	1	1	1	1	1	1	1	0	1	1	1	1	0	1	1	0	1	0
25929	IEPSWADVKKDLISY	NS3	65	79	HLA class I	81% - <100%	Dengue virus 2	ELISPOT	IFNg release	Positive	1	1	0	1	1	1	1	1	0	1	1	1	1	0	1	1	0	1	0
26965	ILAPTRVVAAEMEEA	NS3	220	234	HLA class I	81% - <100%	Dengue virus 2	ELISPOT	IFNg release	Positive	1	1	1	1	1	1	1	1	0	1	1	1	1	0	1	1	0	1	0
27770	INYADRRWCF	NS3	555	564	HLA-A24	81% - <100%	Dengue virus 2	ELISPOT	IFNg release	Positive	1	1	1	1	1	1	1	1	0	1	1	1	1	0	1	1	0	1	0
2859	ALRGLPIRY	NS3	234	242	HLA-A*03:01	81% - <100%	Dengue virus 2	51 chromium	cytotoxicity	Positive	1	1	1	1	1	1	1	1	0	1	1	1	1	0	1	1	0	1	0
35238	LDNINTPEGIIPSMF	NS3	495	509	HLA class I	81% - <100%	Dengue virus 2	ELISPOT	IFNg release	Positive	1	1	1	1	1	1	1	1	0	1	1	1	1	0	1	1	0	1	0
46576	NYADRRWCF	NS3	556	564	HLA-A24	81% - <100%	Dengue virus 2	ELISPOT	IFNg release	Positive	1	1	1	1	1	1	1	1	0	1	1	1	1	0	1	1	0	1	0
539620	LRTLILAPTRVVAAEM	NS3	1691	1706	HLA class I	81% - <100%	Dengue virus 2	ICS	IFNg release	Positive	1	1	1	1	1	1	1	1	0	1	1	1	1	0	1	1	0	1	0
539751	NYNLIIMDEAHFTDPA	NS3	1752	1767	HLA class I	81% - <100%	Dengue virus 2	ICS	IFNg release	Positive	1	1	1	1	1	1	1	1	0	1	1	1	1	0	1	1	0	1	0
539752	NYNLIIMDEAHFTDPASI	NS3	1752	1769	HLA class I	81% - <100%	Dengue virus 2	ICS	IFNg release	Positive	1	1	1	1	1	1	1	1	0	1	1	1	1	0	1	1	0	1	0
539985	TLILAPTRVVAAEMEEA	NS3	1693	1709	HLA class I	81% - <100%	Dengue virus 2	ICS	IFNg release	Positive	1	1	1	1	1	1	1	1	0	1	1	1	1	0	1	1	0	1	0
5542	AVSLDFSPGTSGSPI	NS3	125	139	HLA class I	81% - <100%	Dengue virus 2	ELISPOT	IFNg release	Positive	1	1	1	1	1	1	1	1	0	1	1	1	1	0	1	1	0	1	0
56310	RVIDPRRCMK	NS3	1896	1905	HLA-A*31:01	81% - <100%	Dengue virus 2	ELISPOT	IFNg release	Positive	1	1	1	1	1	1	1	1	0	1	1	1	1	0	1	1	0	1	0
57554	SEMGANFKA	NS3	1872	1880	HLA-B*44:03	81% - <100%	Dengue virus 2	ELISPOT	IFNg release	Positive	1	1	1	1	1	1	1	1	0	1	1	1	1	0	1	1	0	1	0
65550	TPEGIIPSMFEPERE	NS3	500	514	HLA class I	81% - <100%	Dengue virus 2	ELISPOT	IFNg release	Positive	1	1	1	1	1	1	1	1	0	1	1	1	1	0	1	1	0	1	0
790	ADVKKDLISYGGGWK	NS3	70	84	HLA class I	81% - <100%	Dengue virus 2	ELISPOT	IFNg release	Positive	1	1	0	1	1	1	1	1	0	1	1	1	1	0	1	1	0	1	0
8910	DKKGKVVGL	NS3	1616	1624	HLA-B8	81% - <100%	Dengue virus 2	ELISPOT	IFNg release	Positive	1	1	1	1	1	1	1	1	0	1	1	1	1	0	1	1	0	1	0
150289	IILEFFLIV	NS4a	2202	2210	HLA-A*02:01	81% - <100%	Dengue virus 2	ELISPOT	IFNg release	Positive	1	1	1	1	1	1	1	1	0	1	1	1	1	0	1	1	0	1	0
150612	TAEAGGRAY	NS4a	2126	2134	HLA-B*35:01	81% - <100%	Dengue virus 2	ELISPOT	IFNg release	Positive	1	1	1	1	1	1	1	1	0	1	1	1	1	0	1	1	0	1	0
150752	YVVIAILTV	NS4a	2229	2237	HLA-A*02:01	81% - <100%	Dengue virus 2	ELISPOT	IFNg release	Positive	1	1	1	1	1	1	1	1	0	1	1	1	1	0	1	1	0	1	0
150753	YVVIAILTVV	NS4a	2229	2238	HLA-A*02:01	81% - <100%	Dengue virus 2	ELISPOT	IFNg release	Positive	1	1	1	1	1	1	1	1	0	1	1	1	1	0	1	1	0	1	0
180781	TPQDNQLTY	NS4a	2221	2229	HLA-B*35:01	81% - <100%	Dengue virus 2	ELISPOT	IFNg release	Positive	1	1	1	1	1	1	1	1	0	1	1	1	1	0	1	1	0	1	0
2877	ALSELPETL	NS4a	2137	2145	HLA-A*02:01	81% - <100%	Dengue virus 2	ELISPOT	IFNg release	Positive	1	1	1	1	1	1	1	1	0	1	1	1	1	0	1	1	0	1	0
37557	LLLTLLATV	NS4a	56	64	HLA-A2	81% - <100%	Dengue virus 2	ICS	IFNg release	Positive	1	1	1	1	1	1	1	1	0	1	1	1	1	0	1	1	0	1	0
51562	QLTYVVIAIL	NS4a	2226	2235	HLA-B*08:01	81% - <100%	Dengue virus 2	ELISPOT	IFNg release	Positive	1	1	1	1	1	1	1	1	0	1	1	1	1	0	1	1	0	1	0
150147	ATVLMGLGK	NS4b	2315	2323	HLA-A*11:01	81% - <100%	Dengue virus 2	multimer/tetramer	qualitative binding	Positive	1	1	1	1	1	1	1	1	0	1	1	1	1	0	1	1	0	1	0
150387	LMGLGKGWPL	NS4b	2318	2327	HLA-A*02:01	81% - <100%	Dengue virus 2	ELISPOT	IFNg release	Positive	1	1	1	1	1	1	1	1	0	1	1	1	1	0	1	1	0	1	0
150389	LMMRTTWAL	NS4b	2425	2433	HLA-B*08:01	81% - <100%	Dengue virus 2	ELISPOT	IFNg release	Positive	1	1	1	1	1	1	1	1	0	1	1	1	1	0	1	1	0	1	0
150534	RPASAWTLY	NS4b	2276	2284	HLA-B*07:02	81% - <100%	Dengue virus 2	ELISPOT	IFNg release	Positive	1	1	1	1	1	1	1	1	0	1	1	1	1	0	1	1	0	1	0
150679	VIDLDPIPY	NS4b	2395	2403	HLA-A*01:01	81% - <100%	Dengue virus 2	ELISPOT	IFNg release	Positive	1	1	1	1	1	1	1	1	0	1	1	1	1	0	1	1	0	1	0
150686	VLMMRTTWA	NS4b	2424	2432	HLA-B*08:01	81% - <100%	Dengue virus 2	ELISPOT	IFNg release	Positive	1	1	1	1	1	1	1	1	0	1	1	1	1	0	1	1	0	1	0
180380	ATGPISTLW	NS4b	2440	2448	HLA-B*58:01	81% - <100%	Dengue virus 2	ELISPOT	IFNg release	Positive	1	1	1	1	1	1	1	1	0	1	1	1	1	0	1	1	0	1	0
180387	CEALTLATGPISTLW	NS4b	191	205	HLA class I	81% - <100%	Dengue virus 2	ELISPOT	IFNg release	Positive	1	1	1	1	1	1	1	1	0	1	1	1	1	0	1	1	0	1	0
180512	IANQATVLM	NS4b	2311	2319	HLA-B*35:01	81% - <100%	Dengue virus 2	ELISPOT	IFNg release	Positive	1	1	1	1	1	1	1	1	0	1	1	1	1	0	1	1	0	1	0
180789	TVIDLDPIPYDPKFE	NS4b	2394	2408	HLA class I	81% - <100%	Dengue virus 2	ELISPOT	IFNg release	Positive	1	1	1	1	1	1	1	1	0	1	1	1	1	0	1	1	0	1	0
180802	VPLLAIGCY	NS4b	2334	2342	HLA-B*35:01	81% - <100%	Dengue virus 2	ELISPOT	IFNg release	Positive	1	1	1	1	1	1	1	1	0	1	1	1	1	0	1	1	0	1	0
182747	CYSQVNPITL	NS4b	2341	2350	HLA-A*24:02	81% - <100%	Dengue virus 2	ELISPOT	IFNg release	Positive	1	1	1	1	1	1	1	1	0	1	1	1	1	0	1	1	0	1	0
183670	IENSSVNVSL	NS4b	2299	2308	HLA-B*08:01	81% - <100%	Dengue virus 2	ELISPOT	IFNg release	Positive	1	1	1	1	1	1	1	1	0	1	1	1	1	0	1	1	0	1	0
184814	MGLGKGWPL	NS4b	2319	2327	HLA-B*08:01	81% - <100%	Dengue virus 2	ELISPOT	IFNg release	Positive	1	1	1	1	1	1	1	1	0	1	1	1	1	0	1	1	0	1	0
186002	TPMLRHSIE	NS4b	2292	2300	HLA-B*08:01	81% - <100%	Dengue virus 2	ELISPOT	IFNg release	Positive	1	1	1	1	1	1	1	1	0	1	1	1	1	0	1	1	0	1	0
240933	TPMLRHSI	NS4b	2292	2299	HLA-B*07:02	81% - <100%	Dengue virus 2	ELISPOT	IFNg release	Positive	1	1	1	1	1	1	1	1	0	1	1	1	1	0	1	1	0	1	0
150227	FTMRHKKATY	NS5	2734	2743	HLA-B*35:01	81% - <100%	Dengue virus 2	ELISPOT	IFNg release	Positive	1	1	1	1	0	1	1	1	0	1	1	1	1	0	1	1	0	0	0
150309	IPMSTYGWNL	NS5	2605	2614	HLA-B*07:02	81% - <100%	Dengue virus 2	ELISPOT	IFNg release	Positive	1	1	1	1	1	1	1	1	0	1	1	1	1	0	1	1	0	1	0
150541	RPTPRGTVM	NS5	3073	3081	HLA-B*07:02	81% - <100%	Dengue virus 2	ELISPOT	IFNg release	Positive	1	1	1	1	1	1	1	1	0	1	1	1	1	0	1	1	0	1	0
150655	TPRMCTREEF	NS5	2881	2890	HLA-B*35:01	81% - <100%	Dengue virus 2	ELISPOT	IFNg release	Positive	1	1	1	1	1	1	1	1	0	1	1	1	1	0	1	1	0	1	0
150750	YTDYMPSMK	NS5	3370	3378	HLA-A*01:01	81% - <100%	Dengue virus 2	ELISPOT	IFNg release	Positive	1	1	1	1	1	1	1	1	0	1	1	1	1	0	1	1	0	1	0
180437	EPKEGTKKLM	NS5	2856	2865	HLA-B*35:01	81% - <100%	Dengue virus 2	ELISPOT	IFNg release	Positive	1	1	1	1	1	1	1	1	0	1	1	1	1	0	1	1	0	1	0
180483	GKKKTPRMCTREEFT	NS5	2877	2891	HLA class I	81% - <100%	Dengue virus 2	ELISPOT	IFNg release	Positive	1	1	1	1	1	1	1	1	0	1	1	1	1	0	1	1	0	1	0
180579	KSYAQMWSLMYFHRR	NS5	3247	3261	HLA class I	81% - <100%	Dengue virus 2	ELISPOT	IFNg release	Positive	1	1	1	1	1	1	1	1	0	1	1	1	1	0	1	1	0	1	0
180606	LMKITAEWLW	NS5	2864	2873	HLA-B*53:01	81% - <100%	Dengue virus 2	ELISPOT	IFNg release	Positive	1	1	1	1	1	1	1	1	0	1	1	1	1	0	1	1	0	1	0
180643	MAMTDTTPFGQQRVF	NS5	2832	2846	HLA class I	81% - <100%	Dengue virus 2	ELISPOT	IFNg release	Positive	1	1	1	1	1	1	1	1	0	1	1	1	1	0	1	1	0	1	0
180667	MPSMKRFRRE	NS5	3374	3383	HLA-B*53:01	81% - <100%	Dengue virus 2	ELISPOT	IFNg release	Positive	1	1	1	1	1	1	1	1	0	1	1	1	1	0	1	1	0	1	0
180675	MSTYGWNLVRLQSGV	NS5	2607	2621	HLA class I	81% - <100%	Dengue virus 2	ELISPOT	IFNg release	Positive	1	1	1	1	1	1	1	1	0	1	1	1	1	0	1	1	0	1	0
180681	NEEYTDYMPSMKRFR	NS5	3367	3381	HLA class I	81% - <100%	Dengue virus 2	ELISPOT	IFNg release	Positive	1	1	1	1	1	1	1	1	0	1	1	1	1	0	1	1	0	1	0
180774	TLRVLNLVENWLNNN	NS5	2652	2666	HLA class I	81% - <100%	Dengue virus 2	ELISPOT	IFNg release	Positive	1	1	1	1	1	1	1	1	0	1	1	1	1	0	1	1	0	1	0
180814	WAYHGSYET	NS5	2794	2802	HLA-B*35:01	81% - <100%	Dengue virus 2	ELISPOT	IFNg release	Positive	1	1	1	1	1	1	1	1	0	1	1	1	1	0	1	1	0	1	0
180817	WHYDQDHPY	NS5	2783	2791	HLA-B*35:01	81% - <100%	Dengue virus 2	ELISPOT	IFNg release	Positive	1	1	1	1	1	1	1	1	0	1	1	1	1	0	1	1	0	1	0
180828	YAQMWSLMYF	NS5	3249	3258	HLA-B*35:01	81% - <100%	Dengue virus 2	ELISPOT	IFNg release	Positive	1	1	1	1	1	1	1	1	0	1	1	1	1	0	1	1	0	1	0
184373	LGKKKTPRM	NS5	2876	2884	HLA-B*08:01	81% - <100%	Dengue virus 2	ELISPOT	IFNg release	Positive	1	1	1	1	1	1	1	1	0	1	1	1	1	0	1	1	0	1	0
184840	MLINRFTMR	NS5	2729	2737	HLA-B*08:01	81% - <100%	Dengue virus 2	ELISPOT	IFNg release	Positive	1	1	1	1	1	1	1	1	0	1	1	1	1	0	1	1	0	1	0
184957	MTTEDMLTVW	NS5	3295	3304	HLA-B*58:01	81% - <100%	Dengue virus 2	ICS	IFNg release	Positive	1	1	1	1	1	1	1	1	0	1	1	1	1	0	1	1	0	1	0
185491	RREEEEAGVL	NS5	3381	3390	HLA-B*08:01	81% - <100%	Dengue virus 2	ELISPOT	IFNg release	Positive	1	1	1	1	1	1	1	1	0	1	1	1	1	0	1	1	0	1	0

185931	TKQTGSASSM	NS5	2802	2811	HLA-B*08:01	81% - <100%	Dengue virus 2	ELISPOT	IFNg release	Positive	1	1	1	1	1	1	1	1	0	1	1	1	1	0	1	1	0	1	0
539436	ISGDDCVVKPLDDRFA	NS5	3151	3166	HLA class I	81% - <100%	Dengue virus 2	ICS	IFNg release	Positive	1	1	1	1	1	1	1	1	0	1	1	1	1	0	1	1	0	1	0
69671	VLNPYMPSV	NS5	2673	2681	HLA-A*02:01	81% - <100%	Dengue virus 2	ELISPOT	IFNg release	Positive	1	1	1	1	1	1	1	1	0	1	1	1	1	0	1	1	0	1	0
73428	YAQMWSLMY	NS5	3249	3257	HLA-B*15:01	81% - <100%	Dengue virus 2	ELISPOT	IFNg release	Positive	1	1	1	1	1	1	1	1	0	1	1	1	1	0	1	1	0	1	0
74305	YILRDVSKK	NS5	517	525	HLA class I	81% - <100%	Dengue virus 2	ELISPOT	IFNg release	Positive	1	1	1	1	1	1	1	1	0	1	1	1	1	0	1	1	0	1	0
150224	FTIMAAILAY	PreM	247	256	HLA-B*35:01	51%-80%	Dengue virus 2	ELISPOT	IFNg release	Positive	1	1	1	1	0	1	1	1	0	0	1	1	1	1	1	1	1	0	1
150299	ILLTAVAPSM	PreM	270	279	HLA-B*08:01	51%-80%	Dengue virus 2	ELISPOT	IFNg release	Positive	1	1	1	1	0	0	1	1	0	1	1	1	1	1	1	1	1	0	1
150571	SLLFKTEDGV	PreM	136	145	HLA-A*02:01	51%-80%	Dengue virus 2	ELISPOT	IFNg release	Positive	1	0	1	0	1	0	1	1	0	1	0	1	0	1	1	1	1	1	1
180588	LAYTIGTTHF	PreM	254	263	HLA-B*35:01	51%-80%	Dengue virus 2	ELISPOT	IFNg release	Positive	1	0	1	0	1	0	1	1	0	1	0	1	0	1	1	1	1	1	1
180433	EPGQLKLNWF	E	663	672	HLA-B*35:01	51%-80%	Dengue virus 2	ELISPOT	IFNg release	Positive	1	0	1	0	1	0	1	1	0	1	0	1	0	1	0	1	1	1	1
180563	KILIGVIITWIGMNS	E	456	470	HLA class I	51%-80%	Dengue virus 2	ICS	IFNg release	Positive	1	0	1	0	1	0	1	1	0	1	0	1	0	1	1	1	1	1	0
182846	EAKQPATLR	E	329	337	HLA-A*68:01	51%-80%	Dengue virus 2	ELISPOT	IFNg release	Positive	1	0	1	0	1	0	1	1	0	1	0	1	0	1	1	1	1	1	1
183747	ILIGVIITW	E	737	745	HLA-B*58:01	51%-80%	Dengue virus 2	ELISPOT	IFNg release	Positive	1	0	1	0	1	0	1	1	0	1	0	1	0	1	1	1	1	1	0
150259	GPWHLGKLEM	NS1	1041	1050	HLA-B*35:01	51%-80%	Dengue virus 2	ELISPOT	IFNg release	Positive	1	0	1	0	1	0	1	1	0	1	0	1	0	0	1	1	0	1	0
186474	WKTWGKAKML	NS1	890	899	HLA-B*08:01	51%-80%	Dengue virus 2	ELISPOT	IFNg release	Positive	1	0	1	0	1	0	1	1	0	1	0	1	0	0	1	1	0	1	0
150741	YQLAVTIMA	NS2a	1157	1165	HLA-A*02:01	51%-80%	Dengue virus 2	ELISPOT	IFNg release	Positive	1	1	0	1	1	1	0	1	0	1	1	1	1	0	1	1	0	1	0
183741	ILCVPNAVIL	NS2a	1279	1288	HLA-B*08:01	51%-80%	Dengue virus 2	ELISPOT	IFNg release	Positive	0	1	0	1	1	1	1	0	0	1	1	0	1	0	1	1	0	1	0
150346	KTFDSEYVK	NS3	1863	1871	HLA-A*11:01	51%-80%	Dengue virus 2	multimer/tetramer	qualitative binding	Positive	1	0	1	0	1	0	1	1	0	1	0	1	0	0	1	1	0	1	0
17802	FSPGTSGSPIIDKKG	NS3	130	144	HLA class I	51%-80%	Dengue virus 2	ELISPOT	IFNg release	Positive	1	0	1	0	1	0	1	1	0	1	0	1	0	0	1	1	0	1	0
180623	LPVWLAYKV	NS3	2017	2025	HLA-B*51:01	51%-80%	Dengue virus 2	ELISPOT	IFNg release	Positive	1	0	1	0	0	1	1	1	0	1	0	1	0	0	1	0	0	0	0
180624	LPVWLAYKVA	NS3	2017	2026	HLA-B*35:01	51%-80%	Dengue virus 2	ELISPOT	IFNg release	Positive	1	0	1	0	0	1	1	1	0	1	0	1	0	0	1	0	0	0	0
180750	SPGTSGSPIIDKKGK	NS3	131	145	HLA-A*11:01	51%-80%	Dengue virus 2	ELISPOT	IFNg release	Positive	1	0	1	0	1	0	1	1	0	1	0	1	0	0	1	1	0	1	0
185388	RIKQKGILGY	NS3	1499	1508	HLA-B*15:01	51%-80%	Dengue virus 2	ELISPOT	IFNg release	Positive	0	1	1	1	1	1	1	1	0	0	1	1	1	0	1	1	0	1	0
22818	GTSGSPIIDK	NS3	133	142	HLA-A*11:01	51%-80%	Dengue virus 2	ELISPOT	IFNg release	Positive	1	0	1	0	1	0	1	1	0	1	0	1	0	0	1	1	0	1	0
22819	GTSGSPIIDKK	NS3	133	143	HLA-A11	51%-80%	Dengue virus 2	51 chromium	cytotoxicity	Positive	1	0	1	0	1	0	1	1	0	1	0	1	0	0	1	1	0	1	0
54176	RIKQKGIL	NS3	1499	1506	HLA-B8	51%-80%	Dengue virus 2	ELISPOT	IFNg release	Positive	0	1	1	1	1	1	1	1	0	0	1	1	1	0	1	1	0	1	0
150377	LLLVAHYAI	NS4b	2355	2363	HLA-A*02:01	51%-80%	Dengue virus 2	ELISPOT	IFNg release	Positive	0	1	1	1	0	1	1	1	0	1	1	1	1	0	1	0	0	0	0
150639	TLYAVATTFV	NS4b	2282	2291	HLA-A*02:01	51%-80%	Dengue virus 2	ELISPOT	IFNg release	Positive	1	0	1	0	1	0	1	1	0	1	0	1	0	0	1	1	0	1	0
180794	VATTFVTPM	NS4b	2286	2294	HLA-B*35:01	51%-80%	Dengue virus 2	ELISPOT	IFNg release	Positive	1	0	1	0	1	0	1	1	0	1	0	1	0	0	1	1	0	1	0
35568	LEKTKKDL	NS4b	2249	2256	HLA-B8	51%-80%	Dengue virus 2	ELISPOT	IFNg release	Positive	1	0	1	0	1	0	1	1	0	1	0	1	0	0	1	1	0	1	0
150554	RTTWSIHAK	NS5	3283	3291	HLA-A*11:01	51%-80%	Dengue virus 2	ELISPOT	IFNg release	Positive	1	0	1	0	1	0	1	1	0	1	0	1	0	0	1	1	0	1	0
174066	CGGLKNVREVKGLTKGG	NS5	2582	2598	HLA-A2	51%-80%	Dengue virus 2	ICS	IFNg release	Positive	1	0	1	0	1	0	1	1	0	1	0	1	0	0	1	1	0	1	0
180500	HKKLAEAIFKLTYQN	NS5	3052	3066	HLA class I	51%-80%	Dengue virus 2	ELISPOT	IFNg release	Positive	1	0	1	0	1	1	1	1	0	1	0	1	0	0	1	1	0	1	0
180582	KTWAYHGSYETKQTG	NS5	2792	2806	HLA class I	51%-80%	Dengue virus 2	ICS	IFNg release	Positive	0	1	0	1	1	1	1	0	0	0	1	0	1	0	1	1	0	1	0
180758	SWHYDQDHPYKTWAY	NS5	291	305	HLA class I	51%-80%	Dengue virus 2	ELISPOT	IFNg release	Positive	0	1	0	1	1	1	1	0	0	0	1	0	1	0	1	1	0	1	0
31747	KLAEAIFKL	NS5	3054	3062	HLA-A*02:01	51%-80%	Dengue virus 2	ELISPOT	IFNg release	Positive-Low	1	0	1	0	1	1	1	1	0	1	0	1	0	0	1	1	0	1	0
183766	ILNRRRRTA	C	94	102	HLA-B*08:01	>0% - 50%	Dengue virus 2	ELISPOT	IFNg release	Positive	0	1	0	1	0	1	0	0	0	0	1	0	1	0	0	1	0	0	0
184518	LNRRRRTAG	C	95	103	HLA-B*08:01	>0% - 50%	Dengue virus 2	ELISPOT	IFNg release	Positive	0	1	0	1	0	1	0	0	0	0	1	0	1	0	0	1	0	0	0
180818	WILRHPGFTMMAAIL	PreM	126	140	HLA class I	>0% - 50%	Dengue virus 2	ICS	IFNg release	Positive	0	0	0	0	1	0	0	0	0	0	0	0	0	0	0	0	0	1	0
183577	HFQRALIFIL	PreM	262	271	HLA-B*08:01	>0% - 50%	Dengue virus 2	ELISPOT	IFNg release	Positive	0	0	0	0	1	0	0	0	0	0	0	0	0	1	1	1	1	1	1
183621	HVQRIETWIL	PreM	233	242	HLA-B*08:01	>0% - 50%	Dengue virus 2	ELISPOT	IFNg release	Positive	0	0	0	0	1	0	0	0	0	0	0	0	0	0	0	0	0	1	0
183748	ILIGVVITW	E	737	745	HLA-B*58:01	>0% - 50%	Dengue virus 2	ELISPOT	IFNg release	Positive	0	1	0	1	0	1	0	0	0	0	1	0	1	0	0	0	0	0	0
183781	IMDLEKRHVL	E	619	628	HLA-B*08:01	>0% - 50%	Dengue virus 2	ELISPOT	IFNg release	Positive	0	1	0	1	1	0	0	0	0	0	1	0	0	1	1	1	1	1	1
184337	LEKRHVLGRL	E	622	631	HLA-B*08:01	>0% - 50%	Dengue virus 2	ELISPOT	IFNg release	Positive	0	1	0	1	1	1	0	0	0	0	1	0	0	1	1	1	1	1	1
184755	MDLEKRHVL	E	620	628	HLA-B*08:01	>0% - 50%	Dengue virus 2	ELISPOT	IFNg release	Positive	0	1	0	1	1	0	0	0	0	0	1	0	0	1	1	1	1	1	1
180450	FAGPVSQHNY	NS1	1022	1031	HLA-B*35:01	>0% - 50%	Dengue virus 2	ELISPOT	IFNg release	Positive	0	1	0	1	0	1	0	0	0	0	1	0	1	0	0	1	0	0	0
180496	GVFTTNIWLKLKEKQ	NS1	936	950	HLA class I	>0% - 50%	Dengue virus 2	ICS	IFNg release	Positive	1	0	1	0	0	0	1	1	0	1	0	1	0	0	1	0	0	1	0
150457	MTDDIGMGV	NS2a	1191	1199	HLA-A*01:01	>0% - 50%	Dengue virus 2	ELISPOT	IFNg release	Positive	0	1	0	1	1	1	0	0	0	0	1	0	1	0	1	1	0	1	0
150640	TMTDDIGMGV	NS2a	1176	1185	HLA-A*02:01	>0% - 50%	Dengue virus 2	ELISPOT	IFNg release	Positive	0	1	0	1	1	1	0	0	0	0	1	0	1	0	1	1	0	1	0
180671	MSFRDLGRVM	NS2a	1175	1184	HLA-B*35:01	>0% - 50%	Dengue virus 2	ELISPOT	IFNg release	Positive	0	1	0	1	1	1	0	0	0	0	1	0	1	0	0	1	0	1	0
180717	RDLGRVMVMVGATMT	NS2a	51	65	HLA class I	>0% - 50%	Dengue virus 2	ICS	IFNg release	Positive	0	1	0	1	1	1	0	0	0	0	1	0	1	0	0	1	0	1	0
150381	LLVISGLFPV	NS2b	1448	1457	HLA-A*02:01	>0% - 50%	Dengue virus 2	ELISPOT	IFNg release	Positive	0	1	0	1	1	1	0	0	0	0	1	0	1	0	1	1	0	1	0
150425	LVISGLFPV	NS2b	1449	1457	HLA-A*02:01	>0% - 50%	Dengue virus 2	ELISPOT	IFNg release	Positive	0	1	0	1	1	1	0	0	0	0	1	0	1	0	1	1	0	1	0
180538	ISGLFPVSIPITAAA	NS2b	106	120	HLA class I	>0% - 50%	Dengue virus 2	ICS	IFNg release	Positive	0	1	0	1	1	1	0	0	0	0	1	0	1	0	1	1	0	1	0
150217	FPQSNAPIM	NS3	1800	1808	HLA-B*35:01	>0% - 50%	Dengue virus 2	ELISPOT	IFNg release	Positive	0	1	0	1	0	1	0	0	0	0	1	0	1	0	0	1	0	0	0
180460	FPQSNAPIMD	NS3	1800	1809	HLA-B*35:01	>0% - 50%	Dengue virus 2	ELISPOT	IFNg release	Positive	0	1	0	1	0	1	0	0	0	0	1	0	1	0	0	1	0	0	0
180611	LMRRGDLPVWLAYRV	NS3	2011	2025	HLA-B*55:02	>0% - 50%	Dengue virus 2	ICS	IFNg release	Positive	0	1	0	1	1	0	0	0	0	0	1	0	1	0	0	1	0	1	0
180625	LPVWLAYRV	NS3	2017	2025	HLA-B*51:01	>0% - 50%	Dengue virus 2	ELISPOT	IFNg release	Positive	0	1	0	1	1	0	0	0	0	0	1	0	1	0	0	1	0	1	0
180626	LPVWLAYRVA	NS3	2017	2026	HLA-B*51:01	>0% - 50%	Dengue virus 2	ELISPOT	IFNg release	Positive	0	1	0	1	1	0	0	0	0	0	1	0	1	0	0	1	0	1	0
22823	GTSGSPIVDK	NS3	133	142	HLA-A11	>0% - 50%	Dengue virus 2	51 chromium	cytotoxicity	Positive	0	1	0	1	0	1	0	0	0	0	1	0	1	0	0	0	0	0	0
150461	MTQKARNAL	NS4a	2110	2118	HLA-B*07:02	>0% - 50%	Dengue virus 2	ELISPOT	IFNg release	Positive	0	1	0	1	0	0	0	0	0	0	1	0	1	0	0	0	0	0	0
180620	LPTFMTQKAR	NS4a	2106	2115	HLA-B*35:01	>0% - 50%	Dengue virus 2	ELISPOT	IFNg release	Positive	0	1	0	1	1	0	0	0	0	0	1	0	1	0	1	1	0	1	0
185447	RNALDNLAVL	NS4a	2115	2124	HLA-B*08:01	>0% - 50%	Dengue virus 2	ELISPOT	IFNg release	Positive	0	1	0	1	0	0	0	0	0	0	1	0	1	0	0	0	0	0	0
150373	LLILCVTQV	NS4b	2416	2424	HLA-A*02:01	>0% - 50%	Dengue virus 2	ELISPOT	IFNg release	Positive	1	0	1	0	0	0	1	1	0	1	0	1	0	0	0	0	0	0	0
150445	MLLILCVTQV	NS4b	2415	2424	HLA-A*02:01	>0% - 50%	Dengue virus 2	ELISPOT	IFNg release	Positive	1	0	1	0	0	0	1	1	0	1	0	1	0	0	0	0	0	0	0
16757	FLLVAHYAI	NS4b	112	120	HLA-A2	>0% - 50%	Dengue virus 2	ICS	TNF release	Positive	1	0	0	0	1	0	0	0	0	0	0	0	0	0	0	1	0	1	0
180601	LILCVTQVLM	NS4b	2417	2426	HLA-B*35:01	>0% - 50%	Dengue virus 2	ELISPOT	IFNg release	Positive	1	0	1	0	0	0	1	1	0	1	0	1	0	0	0	0	0	0	0
184439	LLILCVTQVL	NS4b	2416	2425	HLA-B*08:01	>0% - 50%	Dengue virus 2	ELISPOT	IFNg release	Positive	1	0	1	0	0	0	1	1	0	1	0	1	0	0	0	0	0	0	0
10587	DVFFTPPEK	NS5	2622	2630	HLA class I	>0% - 50%	Dengue virus 2	ELISPOT	IFNg release	Positive	0	1	0	1	0	1	0	0	0	0	1	0	1	0	0	1	0	0	0
150698	VPYLGKREDQ	NS5	3327	3336	HLA-B*07:02	>0% - 50%	Dengue virus 2	ELISPOT	IFNg release	Positive	0	0	0	0	0	0	0	0	1	0	0	0	0	0	0	0	0	0	0
161186	RLLTKPWDVVPMVTQ	NS5	326	340	HLA-B*55:02	>0% - 50%	Dengue virus 2	ICS	IFNg release	Positive	0	0	0	0	1	0	0	0	0	0	0	0	0	0	1	1	0	1	0
163027	NIQTAINQV	NS5	3353	3361	HLA-A*02:01	>0% - 50%	Dengue virus 2	ELISPOT	IFNg release	Positive-Low	0	1	0	1	0	1	0	0	0	0	1	0	1	0	0	1	0	0	0
163620	TITEEIAVQ	NS5	3127	3135	HLA-A*02:01	>0% - 50%	Dengue virus 2	ELISPOT	IFNg release	Positive	0	0	0	0	1	0	0	0	0	0	0	0	0	0	0	0	0	1	0
180584	KVRKDIQQW	NS5	3177	3185	HLA-B*57:01	>0% - 50%	Dengue virus 2	ELISPOT	IFNg release	Positive	0	1	0	1	0	1	0	0	0	0	1	0	1	0	0	1	0	0	0
180668	MPSVIEKMET	NS5	2678	2687	HLA-B*53:01	>0% - 50%	Dengue virus 2	ELISPOT	IFNg release	Positive	0	0	0	0	1	0	0	0	0	0	0	0	0	0	1	0	0	0	0
182855	EAVEDGRFWE	NS5	2915	2924	HLA-B*58:01	>0% - 50%	Dengue virus 2	ELISPOT	IFNg release	Positive	1	0	1	0	0	0	0	1	0	1	0	1	0	0	0	0	0	0	0
184234	KWKSRLNAL	NS5	2503	2511	HLA-B*08:01	>0% - 50%	Dengue virus 2	ELISPOT	IFNg release	Positive	1	0	1	0	1	0	1	1	0	0	0	1	0	0	1	1	0	1	0
186219	VEDGRFWEL	NS5	2917	2925	HLA-B*40:01	>0% - 50%	Dengue virus 2	ELISPOT	IFNg release	Positive	1	0	1	0	0	0	0	1	0	1	0	1	0	0	0	0	0	0	0
190862	MVTQMAMTDTTPFGQQR	NS5	2828	2844	HLA class I	>0% - 50%	Dengue virus 2	ICS	IFNg release	Positive	0	1	0	1	1	1	0	0	0	0	1	0	1	0	1	1	0	1	0
32942	KPWDVLPTV	NS5	2821	2829	HLA class I	>0% - 50%	Dengue virus 2	51 chromium	cytotoxicity	Positive	1	0	1	0	0	0	1	1	0	1	0	1	0	0	0	0	0	0	0
32943	KPWDVVPMV	NS5	2821	2829	HLA-B*51:01	>0% - 50%	Dengue virus 2	51 chromium	cytotoxicity	Positive	0	0	0	0	1	0	0	0	0	0	0	0	0	0	1	1	0	1	0
539144	CVVKPLDDRFASALTAL	NS5	3156	3172	HLA class I	>0% - 50%	Dengue virus 2	ICS	IFNg release	Positive	0	0	0	0	1	1	0	0	0	0	0	0	0	0	1	1	0	1	0

CD8 DENV-3 Epitopes

NCBI IDs of DENV-3 circulating strains from Thailand, (100% identity=1,green ; else 0, red)

Epitope_ID	AA_Seq	Protein_Name	Start	End	Allele	step	Serotype	Method/Technique	Assay Group	Qualitative Measure	TV003	TDEN	DPIV	Dengvaxia	DENVax	TVDV
											DENV-3 constructs of vaccines					
3124	AMKGLPIRY	NS3	1708	1716	HLA-B62	0%	Dengue virus 3	51 chromium	cytotoxicity	Positive	0	0	0	0	0	0
590435	TRVVAAEMEEAMKGLPI	NS3	1698	1714	HLA class I	0%	Dengue virus 3	ICS	IFNg release	Positive-Low	0	0	0	0	0	0
180801	VMGITAEWLW	NS5	2862	2871	HLA-B*53:01	0%	Dengue virus 3	ELISPOT	IFNg release	Positive	0	0	0	0	0	0
186215	VEDEDFWKL	NS5	2915	2923	HLA-B*40:01	0%	Dengue virus 3	ELISPOT	IFNg release	Positive	0	0	0	0	0	0
184845	MLKRVRNRV	C	15	23	HLA-B*08:01	100%	Dengue virus 3	ELISPOT	IFNg release	Positive	1	1	1	0	0	0
180464	FTILALFLAH	PreM	247	256	HLA-B*35:01	100%	Dengue virus 3	ELISPOT	IFNg release	Positive	1	1	1	1	1	1
180504	HPGFTILALF	PreM	244	253	HLA-B*35:01	100%	Dengue virus 3	ELISPOT	IFNg release	Positive	1	1	1	1	1	1
185685	SLLFKTASG	PreM	136	144	HLA-B*08:01	100%	Dengue virus 3	ELISPOT	IFNg release	Positive	1	1	1	1	1	1
13119	ELKGMSYAM	E	155	163	HLA-B*08:01	100%	Dengue virus 3	ELISPOT	IFNg release	Positive	1	1	1	1	1	0
15796	FFDLPLPWT	E	211	219	HLA-A2	100%	Dengue virus 3	ICS	IFNg release	Positive	1	1	1	1	1	1
185419	RLKMDKLEL	E	148	156	HLA-B*08:01	100%	Dengue virus 3	ELISPOT	IFNg release	Positive	1	1	1	1	1	0
589817	SGATWVDVVLEHGGCV	E	268	283	HLA class I	100%	Dengue virus 3	ICS	IFNg release	Positive-High	1	1	1	1	1	1
150537	RPGYHTQTA	NS1	1030	1038	HLA-B*07:02	100%	Dengue virus 3	ELISPOT	IFNg release	Positive	1	1	1	0	0	0
150546	RSCTLPPLRY	NS1	1087	1096	HLA-A*01:01	100%	Dengue virus 3	ELISPOT	IFNg release	Positive	1	1	1	0	0	0
180738	RYMGEDGCWY	NS1	1095	1104	HLA-A*24:02	100%	Dengue virus 3	ELISPOT	IFNg release	Positive	1	1	1	0	0	0
182939	EKEENMVKSL	NS1	1113	1122	HLA-B*08:01	100%	Dengue virus 3	ELISPOT	IFNg release	Positive	1	1	1	0	0	0
184585	LRTTTVSGKL	NS1	64	73	HLA-B*08:01	100%	Dengue virus 3	ELISPOT	IFNg release	Positive	1	1	1	0	0	0
185573	RYMGEDGCW	NS1	1095	1103	HLA-A*24:02	100%	Dengue virus 3	ELISPOT	IFNg release	Positive	1	1	1	0	0	0
185734	SPKRLATAI	NS1	811	819	HLA-B*08:01	100%	Dengue virus 3	ELISPOT	IFNg release	Positive	1	1	1	0	0	0
182917	EFKDFAAGR	NS3	2083	2091	HLA-A*33:01	100%	Dengue virus 3	ELISPOT	IFNg release	Positive	1	1	1	0	0	0
19434	GESRKTFVE	NS3	2001	2009	HLA-B7	100%	Dengue virus 3	51 chromium	cytotoxicity	Positive	1	1	1	0	0	0
19435	GESRKTFVEL	NS3	211	220	HLA-B*40:01	100%	Dengue virus 3	ICS	IFNg release	Positive	1	1	1	0	0	0
183165	FFMMVLLIPE	NS4a	2205	2214	HLA-A*33:01	100%	Dengue virus 3	ELISPOT	IFNg release	Positive	1	1	1	0	0	0
180381	ATGPITTLW	NS4b	2439	2447	HLA-B*58:01	100%	Dengue virus 3	ELISPOT	IFNg release	Positive	1	1	1	0	0	0
180788	TTKRDLGMSK	NS4b	2250	2259	HLA-A*11:01	100%	Dengue virus 3	ELISPOT	IFNg release	Positive	1	1	1	0	0	0
37534	LLLMRTSWA	NS4b	2423	2431	HLA-A2	100%	Dengue virus 3	biological activity	proliferation	Positive	1	1	1	0	0	0
1067501	KPIDDRFAN	NS5	3158	3166	HLA-B*07:02	100%	Dengue virus 3	ELISPOT	IFNg release	Positive	1	1	1	0	0	0
180650	MEITAEWLW	NS5	2863	2871	HLA-B*58:01	100%	Dengue virus 3	ELISPOT	IFNg release	Positive	1	1	1	0	0	0
185710	SMINGVVKL	NS5	318	326	HLA-A*02:01	100%	Dengue virus 3	ELISPOT	IFNg release	Positive	1	1	1	0	0	0
180577	KSGAIKVLK	C	74	82	HLA-A*11:01	81% - <100%	Dengue virus 3	ELISPOT	IFNg release	Positive	0	1	0	0	0	0
184260	LAIPPTAGVL	C	57	66	HLA-B*08:01	81% - <100%	Dengue virus 3	ELISPOT	IFNg release	Positive	1	1	1	0	0	0
184658	LTQKVVIFIL	PreM	262	271	HLA-B*08:01	81% - <100%	Dengue virus 3	ELISPOT	IFNg release	Positive	1	1	1	1	1	1
180613	LPEEQDQNY	E	82	90	HLA-B*35:01	81% - <100%	Dengue virus 3	ELISPOT	IFNg release	Positive	0	1	0	1	1	1
184299	LATLRKLCI	E	53	61	HLA-B*08:01	81% - <100%	Dengue virus 3	ELISPOT	IFNg release	Positive	1	1	1	1	1	1
184536	LPEYGTLGLE	E	453	462	HLA-B*35:01	81% - <100%	Dengue virus 3	ELISPOT	IFNg release	Positive	1	1	1	1	1	1
184568	LPWTSGATT	E	496	504	HLA-B*35:01	81% - <100%	Dengue virus 3	ELISPOT	IFNg release	Positive	1	1	1	1	0	1
184965	MVHQIFGSAY	E	713	722	HLA-B*15:01	81% - <100%	Dengue virus 3	ELISPOT	IFNg release	Positive	1	1	1	1	1	1
186029	TPTWNRKEL	E	226	234	HLA-B*07:02	81% - <100%	Dengue virus 3	ELISPOT	IFNg release	Positive-Low	0	1	0	1	1	1
184254	LAILFEEVM	NS2a	1140	1148	HLA-B*35:01	81% - <100%	Dengue virus 3	ELISPOT	IFNg release	Positive	1	1	1	0	0	0
185978	TMGVLCLAIL	NS2a	1134	1143	HLA-B*08:01	81% - <100%	Dengue virus 3	ELISPOT	IFNg release	Positive	1	0	1	0	0	0
180622	LPVWLAHKVA	NS3	226	235	HLA-B*35:01	81% - <100%	Dengue virus 3	ELISPOT	IFNg release	Positive	1	1	1	0	0	0
22820	GTSGSPIINR	NS3	133	142	HLA-A*11:01	81% - <100%	Dengue virus 3	51 chromium	cytotoxicity	Positive	1	1	1	0	0	0
22821	GTSGSPIINRE	NS3	133	143	HLA-A11	81% - <100%	Dengue virus 3	ICS	IFNg release	Positive	1	1	1	0	0	0
34605	KYTDRKWCF	NS3	240	248	HLA-A24	81% - <100%	Dengue virus 3	ELISPOT	IFNg release	Positive	1	1	1	0	0	0
62183	SVKKDLISY	NS3	71	79	HLA-B62	81% - <100%	Dengue virus 3	51 chromium	cytotoxicity	Positive	1	1	1	0	0	0
37494	LLLGLMILL	NS4a	2148	2156	HLA-A2	81% - <100%	Dengue virus 3	ICS	IFNg release	Positive	1	1	1	0	0	0
180678	MVSRLLLNR	NS5	2723	2731	HLA-A*11:01	81% - <100%	Dengue virus 3	ELISPOT	IFNg release	Positive	1	1	1	0	0	0
180737	RYLEFEALGF	NS5	2971	2980	HLA-A*24:02	81% - <100%	Dengue virus 3	ELISPOT	IFNg release	Positive	1	1	1	0	0	0
182553	ALLALNDMGK	NS5	3167	3176	HLA-A*03:01	81% - <100%	Dengue virus 3	ELISPOT	IFNg release	Positive	1	1	1	0	0	0
180390	CLMMMLPATL	C	104	113	HLA-A*02:01	51%-80%	Dengue virus 3	ELISPOT	IFNg release	Positive	1	1	1	0	0	0
180609	LMMMLPATL	C	105	113	HLA-A*02:01	51%-80%	Dengue virus 3	ELISPOT	IFNg release	Positive	1	1	1	0	0	0
184264	LAKRFSRGL	C	29	37	HLA-B*08:01	51%-80%	Dengue virus 3	ELISPOT	IFNg release	Positive	1	1	1	0	0	0
184265	LAKRFSRGLL	C	29	38	HLA-B*08:01	51%-80%	Dengue virus 3	ELISPOT	IFNg release	Positive	1	1	1	0	0	0
185260	QLAKRFSRG	C	28	36	HLA-B*08:01	51%-80%	Dengue virus 3	ELISPOT	IFNg release	Positive	1	1	1	0	0	0
180657	MLVTPSMTM	E	273	281	HLA-B*35:01	51%-80%	Dengue virus 3	ELISPOT	IFNg release	Positive	1	0	1	0	0	0
183793	IMKIGIGVLL	E	452	461	HLA-B*08:01	51%-80%	Dengue virus 3	ELISPOT	IFNg release	Positive	1	1	1	1	0	1
183843	IQNSGGTSIF	E	131	140	HLA-B*15:01	51%-80%	Dengue virus 3	ELISPOT	IFNg release	Positive	0	1	0	1	0	0
184412	LKGMSYAMCL	E	156	165	HLA-B*08:01	51%-80%	Dengue virus 3	ELISPOT	IFNg release	Positive	1	1	1	1	1	1
186470	WIMKIGIGVL	E	451	460	HLA-B*08:01	51%-80%	Dengue virus 3	ELISPOT	IFNg release	Positive	1	1	1	1	0	1
183723	IKLTVVVGDI	NS1	857	866	HLA-B*08:01	51%-80%	Dengue virus 3	ELISPOT	IFNg release	Positive	1	1	1	0	0	0
185464	RPINEKEENM	NS1	1109	1118	HLA-B*35:01	51%-80%	Dengue virus 3	ELISPOT	IFNg release	Positive	1	1	1	0	0	0
183395	GKKHMIAGVL	NS2a	1153	1162	HLA-B*08:01	51%-80%	Dengue virus 3	ELISPOT	IFNg release	Positive	1	0	1	0	0	0
186175	TWRDMAHTLI	NS2a	1174	1183	HLA-A*24:02	51%-80%	Dengue virus 3	ELISPOT	IFNg release	Positive	1	1	1	0	0	0
183796	IMKSVGTGK	NS4b	2481	2489	HLA-A*03:01	51%-80%	Dengue virus 3	ELISPOT	IFNg release	Positive	1	1	1	0	0	0
69612	VLLLVTHYA	NS4b	2353	2361	HLA-A2	51%-80%	Dengue virus 3	ICS	IFNg release	Positive	1	0	1	0	0	0
180728	RPRLCTREEF	NS5	2879	2888	HLA-B*07:02	51%-80%	Dengue virus 3	ELISPOT	IFNg release	Positive	1	1	1	0	0	0
186127	TTWEDVPYL	NS5	3321	3329	HLA-A*02:01	51%-80%	Dengue virus 3	ELISPOT	IFNg release	Positive	0	1	0	0	0	0
184924	MSYAMCTNTF	E	575	584	HLA-B*35:01	>0% - 50%	Dengue virus 3	ELISPOT	IFNg release	Positive	0	0	0	0	0	0
183587	HMIAGVFFTF	NS2a	368	377	HLA-A*24:02	>0% - 50%	Dengue virus 3	ELISPOT	IFNg release	Positive	0	0	0	0	0	0
184819	MIAGVFFTF	NS2a	369	377	HLA-B*35:01	>0% - 50%	Dengue virus 3	ELISPOT	IFNg release	Positive	0	0	0	0	0	0
184065	KLNDWDFVV	NS3	399	407	HLA-A*02:01	>0% - 50%	Dengue virus 3	ELISPOT	IFNg release	Positive-High	1	1	1	0	0	0
186234	VEIWTKEGEK	NS3	2053	2062	HLA-B*40:01	>0% - 50%	Dengue virus 3	ELISPOT	IFNg release	Positive	1	1	1	0	0	0
180572	KPRLCTREEF	NS5	2879	2888	HLA-B*07:02	>0% - 50%	Dengue virus 3	ELISPOT	IFNg release	Positive	0	0	0	0	0	0
32944	KPWDVVPTV	NS5	2819	2827	HLA class I	>0% - 50%	Dengue virus 3	51 chromium	cytotoxicity	Positive	0	0	0	0	0	0

CD8 DENV-4 Epitopes

Epitope_ID	AA_Seq	Protein_Name	Start	End	Allele	step	Serotype	Method/Technique	Assay Group	Qualitative Measure	TV003	TDEN	DPIV	Dengvaxia	DENVax	TVDV
											DENV-4 constructs of vaccines					
180810	VTYECPLLV	PreM	162	170	HLA-A*02:01	0%	Dengue virus 4	ELISPOT	IFNg release	Positive	0	0	0	0	0	1
240130	PPASDLKYSW	NS1	880	889	HLA-B*53:01	0%	Dengue virus 4	ELISPOT	IFNg release	Positive	0	0	0	0	0	0
167725	AMTTTLSIPHDLMELIDGIS	NS2a	50	69	HLA class I	0%	Dengue virus 4	ELISPOT	IFNg release	Positive	0	0	0	0	0	0
239411	ALWYVWQVK	NS2b	1463	1471	HLA-A*03:01	0%	Dengue virus 4	ELISPOT	IFNg release	Positive	0	0	0	0	0	0
418991	IPVTMALWY	NS2b	1458	1466	HLA-B*35:01	0%	Dengue virus 4	ELISPOT	IFNg release	Positive	0	0	0	0	0	0
186602	YSDPLALRE	NS3	2077	2085	HLA-A*01:01	0%	Dengue virus 4	ELISPOT	IFNg release	Positive	0	0	0	0	0	0
239768	GTSGSPIINK	NS3	1607	1616	HLA-A*03:01	0%	Dengue virus 4	ELISPOT	IFNg release	Positive	0	0	0	0	0	0
184375	LGKSYAQMW	NS5	3246	3254	HLA-B*57:01	0%	Dengue virus 4	ELISPOT	IFNg release	Positive	0	0	0	0	0	0
419017	MPAMKRYSAP	NS5	3371	3380	HLA-B*35:01	0%	Dengue virus 4	ELISPOT	IFNg release	Positive	0	0	0	0	0	0
182971	EMCEDTVTY	PreM	156	164	HLA-B*35:01	100%	Dengue virus 4	ELISPOT	IFNg release	Positive	1	1	1	0	1	1
583701	GVSGGAWVDLVLEHGGCV	E	14	31	HLA class I	100%	Dengue virus 4	ICS	IFNg release	Positive-Low	1	1	1	1	1	1
182562	ALPVYLMTL	NS2a	1329	1337	HLA-A*02:01	100%	Dengue virus 4	ELISPOT	IFNg release	Positive	1	1	1	0	0	0
184566	LPVYLMTLMK	NS2a	1330	1339	HLA-B*35:01	100%	Dengue virus 4	ELISPOT	IFNg release	Positive	1	1	1	0	0	0
180475	GEQRKTFVEL	NS3	2001	2010	HLA-B*40:01	100%	Dengue virus 4	ELISPOT	IFNg release	Positive	1	1	1	0	0	0
180640	MALKDFKEF	NS3	2079	2087	HLA-B*35:01	100%	Dengue virus 4	ELISPOT	IFNg release	Positive	1	1	1	0	0	0
180778	TPEGIIPTLF	NS3	1974	1983	HLA-B*07:02	100%	Dengue virus 4	ELISPOT	IFNg release	Positive	1	1	1	0	0	0
183231	FPQSNSPIED	NS3	1799	1808	HLA-B*35:01	100%	Dengue virus 4	ELISPOT	IFNg release	Positive	1	1	1	0	0	0
183427	GLYGNGVVTK	NS3	1622	1631	HLA-A*03:01	100%	Dengue virus 4	ELISPOT	IFNg release	Positive	1	1	1	0	0	0
186347	VPNYNLIVM	NS3	1749	1757	HLA-B*35:01	100%	Dengue virus 4	ELISPOT	IFNg release	Positive	1	1	1	0	0	0
195838	PNYNLIVMDEAHFTD	NS3	1750	1764	HLA class I	100%	Dengue virus 4	ICS	IFNg release	Positive	1	1	1	0	0	0
22822	GTSGSPIINRK	NS3	133	143	HLA-A11	100%	Dengue virus 4	51 chromium	cytotoxicity	Positive	1	1	1	0	0	0
539058	AHFTDPSSVAARGYIST	NS3	1760	1776	HLA class I	100%	Dengue virus 4	ICS	IFNg release	Positive	1	1	1	0	0	0
539576	LIVMDEAHFTDPSSVAA	NS3	1754	1770	HLA class I	100%	Dengue virus 4	ICS	IFNg release	Positive	1	1	1	0	0	0
62616	SYKDREWCF	NS3	389	397	HLA-A24	100%	Dengue virus 4	ELISPOT	IFNg release	Positive	1	1	1	0	0	0
65552	TPEGIIPTL	NS3	500	508	HLA-B*35:01	100%	Dengue virus 4	51 chromium	cytotoxicity	Positive	1	1	1	0	0	0
180596	LETLMLVALL	NS4a	2144	2153	HLA-B*40:01	100%	Dengue virus 4	ELISPOT	IFNg release	Positive	1	1	1	0	0	0
184531	LPESLETLML	NS4a	2140	2149	HLA-B*35:01	100%	Dengue virus 4	ELISPOT	IFNg release	Positive	1	1	1	0	0	0
42083	MLVALLGAM	NS4a	56	64	HLA-A2	100%	Dengue virus 4	ICS	IFNg release	Positive	1	1	1	0	0	0
150647	TPMLRHTIEN	NS4b	2288	2297	HLA-B*07:02	100%	Dengue virus 4	ELISPOT	IFNg release	Positive	1	1	1	0	0	0
1067215	HSWEDIPYL	NS5	3319	3327	HLA-A*02:01	100%	Dengue virus 4	ELISPOT	IFNg release	Positive	1	1	1	0	0	0
150548	RSNAAIGAVF	NS5	2891	2900	HLA-B*15:01	100%	Dengue virus 4	ELISPOT	IFNg release	Positive	1	1	1	0	0	0
180718	REDLWCGSL	NS5	3330	3338	HLA-B*40:01	100%	Dengue virus 4	ELISPOT	IFNg release	Positive	1	1	1	0	0	0
183353	GESSSNPTI	NS5	2635	2643	HLA-B*44:02	100%	Dengue virus 4	ELISPOT	IFNg release	Positive	1	1	1	0	0	0
184130	KREDLWCGSL	NS5	3329	3338	HLA-B*08:01	100%	Dengue virus 4	ELISPOT	IFNg release	Positive	1	1	1	0	0	0
186453	WEPSKGWKNW	NS5	3182	3191	HLA-B*44:02	100%	Dengue virus 4	ELISPOT	IFNg release	Positive	1	1	1	0	0	0
186640	YYMATLKNV	NS5	2576	2584	HLA-A*24:02	100%	Dengue virus 4	ELISPOT	IFNg release	Positive	1	1	1	0	0	0
186641	YYMATLKNVT	NS5	2576	2585	HLA-A*24:02	100%	Dengue virus 4	ELISPOT	IFNg release	Positive	1	1	1	0	0	0
539703	MVTQLAMTDTTPFGQQR	NS5	2824	2840	HLA class I	100%	Dengue virus 4	ICS	IFNg release	Positive	1	1	1	0	0	0
180541	ITLLCLIPTV	C	102	111	HLA-A*02:01	81% - <100%	Dengue virus 4	ELISPOT	IFNg release	Positive	1	1	1	0	0	0
184591	LSIPHDLMEF	NS2a	1239	1248	HLA-A*01:01	81% - <100%	Dengue virus 4	ELISPOT	IFNg release	Positive	0	0	0	0	0	0
182901	EETNMITLL	NS2b	1434	1442	HLA-B*40:01	81% - <100%	Dengue virus 4	ELISPOT	IFNg release	Positive	1	1	1	0	0	0
186584	YPLAIPVTM	NS2b	1454	1462	HLA-B*53:01	81% - <100%	Dengue virus 4	ELISPOT	IFNg release	Positive	1	1	1	0	0	0
156711	LAPTRVVAAEMEEAL	NS3	1695	1709	HLA-B7	81% - <100%	Dengue virus 4	51 chromium	cytotoxicity	Positive	1	1	1	0	0	0
180473	GEFRLRGEQR	NS3	1995	2004	HLA-B*40:01	81% - <100%	Dengue virus 4	ELISPOT	IFNg release	Positive	1	1	1	0	0	0
180843	YPKTKLTDWD	NS3	1868	1877	HLA-B*35:01	81% - <100%	Dengue virus 4	ELISPOT	IFNg release	Positive	1	1	1	0	0	0
180850	YTPEGIIPTL	NS3	1973	1982	HLA-A*02:06	81% - <100%	Dengue virus 4	ELISPOT	IFNg release	Positive	1	1	1	0	0	0
182919	EFRLRGEQR	NS3	1996	2004	HLA-A*33:01	81% - <100%	Dengue virus 4	ELISPOT	IFNg release	Positive	1	1	1	0	0	0
183338	GEIGAVTLDF	NS3	1595	1604	HLA-B*44:03	81% - <100%	Dengue virus 4	ELISPOT	IFNg release	Positive	1	1	1	0	0	0
184079	KLTDWDFVV	NS3	398	406	HLA-A*02:01	81% - <100%	Dengue virus 4	ELISPOT	IFNg release	Positive	1	1	1	0	0	0
34918	LAPTRVVAAEME	NS3	221	232	HLA-B7	81% - <100%	Dengue virus 4	51 chromium	cytotoxicity	Positive	1	1	1	0	0	0
540029	VEMGEAAAIFMTATPPG	NS3	1778	1794	HLA class I	81% - <100%	Dengue virus 4	ICS	IFNg release	Positive	1	1	1	0	0	0
581940	APTRVVAAEMEEALRGL	NS3	1558	1574	HLA class I	81% - <100%	Dengue virus 4	ICS	IFNg release	Positive-Low	1	1	1	0	0	0
182508	AIAVASGLLW	NS4a	2179	2188	HLA-B*44:02	81% - <100%	Dengue virus 4	ELISPOT	IFNg release	Positive	0	0	0	0	0	0
182926	EIASLPTYL	NS4a	2101	2109	HLA-A*68:02	81% - <100%	Dengue virus 4	ELISPOT	IFNg release	Positive	1	1	1	0	0	0
183650	IAVASGLLW	NS4a	2180	2188	HLA-B*57:01	81% - <100%	Dengue virus 4	ELISPOT	IFNg release	Positive	1	1	1	0	0	0
185881	TEIASLPTYL	NS4a	2100	2109	HLA-B*40:01	81% - <100%	Dengue virus 4	ELISPOT	IFNg release	Positive	1	1	1	0	0	0
186620	YVILTILTI	NS4a	2228	2236	HLA-A*24:02	81% - <100%	Dengue virus 4	ELISPOT	IFNg release	Positive	1	1	1	0	0	0
136826	CYSQVNPTTL	NS4b	2337	2346	HLA-A*24:02	81% - <100%	Dengue virus 4	ELISPOT	IFNg release	Positive	1	1	1	0	0	0
180379	ATGPILTLW	NS4b	2436	2444	HLA-B*58:01	81% - <100%	Dengue virus 4	ELISPOT	IFNg release	Positive	1	1	1	0	0	0
180803	VPLLAMGCY	NS4b	2330	2338	HLA-B*35:01	81% - <100%	Dengue virus 4	ELISPOT	IFNg release	Positive	1	1	1	0	0	0
183672	IENTSANLSL	NS4b	2295	2304	HLA-B*40:01	81% - <100%	Dengue virus 4	ELISPOT	IFNg release	Positive	1	1	1	0	0	0
184453	LLLMRTTWAF	NS4b	2420	2429	HLA-A*24:02	81% - <100%	Dengue virus 4	ELISPOT	IFNg release	Positive	1	1	1	0	0	0
184713	LYAVATTIL	NS4b	2279	2287	HLA-A*24:02	81% - <100%	Dengue virus 4	ELISPOT	IFNg release	Positive	1	1	1	0	0	0
186330	VMLLVHYAI	NS4b	2351	2359	HLA-A*24:02	81% - <100%	Dengue virus 4	ELISPOT	IFNg release	Positive	1	0	1	0	0	0
37535	LLLMRTTWA	NS4b	76	84	HLA-A2	81% - <100%	Dengue virus 4	ICS	TNF release	Positive	1	1	1	0	0	0
40472	LVMLLVHYA	NS4b	6	14	HLA-A2	81% - <100%	Dengue virus 4	ICS	IFNg release	Positive	1	0	1	0	0	0
180684	NPRLCTREEF	NS5	2877	2886	HLA-B*07:02	81% - <100%	Dengue virus 4	ELISPOT	IFNg release	Positive	1	1	1	0	0	0
183570	HEMYWVSGV	NS5	2703	2711	HLA-B*44:02	81% - <100%	Dengue virus 4	ELISPOT	IFNg release	Positive	0	0	0	0	0	0
184051	KLGEFGRAK	NS5	2948	2956	HLA-A*03:01	81% - <100%	Dengue virus 4	ELISPOT	IFNg release	Positive	1	1	1	0	0	0
184954	MTTEDMLKVW	NS5	3292	3301	HLA class I	81% - <100%	Dengue virus 4	ICS	IFNg release	Positive	1	1	1	0	0	0
185232	QEEHKETWHY	NS5	2772	2781	HLA-B*44:02	81% - <100%	Dengue virus 4	ELISPOT	IFNg release	Positive	1	1	1	0	0	0
539149	DCVVKPLDERFSTSLLF	NS5	3152	3168	HLA class I	81% - <100%	Dengue virus 4	ICS	IFNg release	Positive	0	0	0	0	0	0
539656	MAISGDDCVVKPLDERF	NS5	3146	3162	HLA class I	81% - <100%	Dengue virus 4	ICS	IFNg release	Positive	1	1	1	0	0	0
183667	IEKASLIEV	NS1	986	994	HLA-B*40:01	51%-80%	Dengue virus 4	ELISPOT	IFNg release	Positive	1	1	1	0	0	0
185050	NFLEVEDYGF	NS1	925	934	HLA-A*24:02	51%-80%	Dengue virus 4	ELISPOT	IFNg release	Positive	1	1	1	0	0	0
183407	GLFGKTQVGV	NS3	1503	1512	HLA-A*02:01	51%-80%	Dengue virus 4	ELISPOT	IFNg release	Positive	0	0	0	0	0	0
182707	CNLTSTWVMY	PreM	181	190	HLA-A*30:02	>0% - 50%	Dengue virus 4	ELISPOT	IFNg release	Positive	1	1	1	0	1	0
186303	VLMMLVAPSY	PreM	17	26	HLA-B*15:01	>0% - 50%	Dengue virus 4	ELISPOT	IFNg release	Positive	1	1	1	0	1	1
185147	NYKERMVTF	E	511	519	HLA-A*24:02	>0% - 50%	Dengue virus 4	ELISPOT	IFNg release	Positive	1	1	1	1	1	1
185817	SWMIRILIGF	E	161	170	HLA-A*24:02	>0% - 50%	Dengue virus 4	ELISPOT	IFNg release	Positive	1	1	1	0	1	0
183818	IPHDLMELI	NS2a	1241	1249	HLA-B*53:01	>0% - 50%	Dengue virus 4	ELISPOT	IFNg release	Positive	1	1	1	0	0	0
184390	LIDGISLGL	NS2a	1248	1256	HLA-A*01:01	>0% - 50%	Dengue virus 4	ELISPOT	IFNg release	Positive	1	1	1	0	0	0
180648	MEGVFHTMW	NS3	1516	1524	HLA-B*44:03	>0% - 50%	Dengue virus 4	ELISPOT	IFNg release	Positive	1	1	1	0	0	0
182563	ALSEGVYRI	NS3	1491	1499	HLA-A*02:01	>0% - 50%	Dengue virus 4	ELISPOT	IFNg release	Positive	1	1	1	0	0	0
186008	TPPGATDPF	NS3	1791	1799	HLA-B*35:01	>0% - 50%	Dengue virus 4	ELISPOT	IFNg release	Positive	1	1	1	0	0	0
180383	ATGPVLTLW	NS4b	2436	2444	HLA-B*58:01	>0% - 50%	Dengue virus 4	ELISPOT	IFNg release	Positive	0	0	0	0	0	0
180587	LATGPVLTLW	NS4b	2435	2444	HLA-B*53:01	>0% - 50%	Dengue virus 4	ELISPOT	IFNg release	Positive	0	0	0	0	0	0
180764	TETTILDVDL	NS4b	2262	2271	HLA-B*40:01	>0% - 50%	Dengue virus 4	ELISPOT	IFNg release	Positive	1	1	1	0	0	0
186550	YDQENPYRTW	NS5	2781	2790	HLA-B*44:02	>0% - 50%	Dengue virus 4	ELISPOT	IFNg release	Positive	1	1	1	0	0	0
419048	SPRLCTREEF	NS5	2877	2886	HLA-B*07:02	>0% - 50%	Dengue virus 4	ELISPOT	IFNg release	Positive	0	0	0	0	0	0

Supplementary Table S7. Alignment of CD4 and CD8 epitopes in circulating dengue viruses from Brazil and six vaccine candidates. *Detailed information, for Brazil, on each of the vaccine carried CD4 and CD8 T cell epitopes that are not present in any of the viral isolates, present in all of the viral isolates, present in only 81% – <100% viral isolates or 51% – 80% viral isolates or in only >0% – 50% of viral isolates for each of the epitope within each of the six vaccine candidates and for all the four dengue serotypes. The first table summarizes information retrieved on circulating viral isolate sequences available from Brazil. Next, the globally reported CD4/CD8 T cell epitopes are organized into: DENV-1 specific CD4 epitopes, DENV-2 specific CD4 epitopes, DENV-3 specific CD4 epitopes, DENV-4 specific CD4 epitopes, DENV-1 specific CD8 epitopes, DENV-2 specific CD8 epitopes, DENV-3 specific CD8 epitopes, DENV-4 specific CD8 epitopes. In each of these tables, identity of the epitope to each of the corresponding viral isolate serotype and each of the vaccine constructs are indicated. Green color shade and number 1 indicates 100% identity in the given virus isolate/vaccine construct. Pink color shade and number zero indicates not identical in the given virus isolate/vaccine construct.*

NCBI ID	Strain Name	Host	Date	Continent	Country	Prot_Name	VirusType
ACO06167.1	DENV-1/BR/BID-V2395/2006	Human	2006-01-01	South America	Brazil	polyprotein	Dengue virus 1
ACO06161.1	DENV-1/BR/BID-V2389/2004	Human	2004-01-01	South America	Brazil	polyprotein	Dengue virus 1
ACO06170.1	DENV-1/BR/BID-V2398/2007	Human	2007-01-01	South America	Brazil	polyprotein	Dengue virus 1
AGN94867.1	13501/BR-PE/10	Human	2010-01-01	South America	Brazil	polyprotein	Dengue virus 1
AGN94869.1	13861/BR-PE/10	Human	2010-01-01	South America	Brazil	polyprotein	Dengue virus 1
AKQ00018.1	DENV1 BR/SJRP/807/2013	Human	2013-01-05	South America	Brazil	polyprotein	Dengue virus 1
AKQ00012.1	DENV1 BR/SJRP/354/2011	Human	2011-05-24	South America	Brazil	polyprotein	Dengue virus 1
AKQ00011.1	DENV1 BR/SJRP/287/2011	Human	2011-05-03	South America	Brazil	polyprotein	Dengue virus 1
ACO06173.1	DENV-1/BR/BID-V2401/2008	Human	2008-01-01	South America	Brazil	polyprotein	Dengue virus 1
AGN94868.1	13671/BR-PE/10	Human	2010-01-01	South America	Brazil	polyprotein	Dengue virus 1
AGN94865.1	9808/BR-PE/10	Human	2010-01-01	South America	Brazil	polyprotein	Dengue virus 1
AGN94870.1	14985/BR-PE/10	Human	2010-01-01	South America	Brazil	polyprotein	Dengue virus 1
AKQ00039.1	BR/SJRP/2271/2014	Human	2014-02-10	South America	Brazil	polyprotein	Dengue virus 1
AKQ00038.1	BR/SJRP/1107/2013	Human	2013-02-26	South America	Brazil	polyprotein	Dengue virus 1
QOW96373.1	USP-CB-54	Human	2018-11-18	South America	Brazil	polyprotein	Dengue virus 1
AKQ00016.1	BR/SJRP/709/2013	Human	2013-10-25	South America	Brazil	polyprotein	Dengue virus 1
AKQ00015.1	DENV1 BR/SJRP/509/2012	Human	2012-03-16	South America	Brazil	polyprotein	Dengue virus 1
AKQ00013.1	BR/SJRP/395/2011	Human	2011-06-08	South America	Brazil	polyprotein	Dengue virus 1
QOW96372.1	USP-CB-53	Human	2018-11-11	South America	Brazil	polyprotein	Dengue virus 1
QOW96385.1	USP-CB-111	Human	2019-06-05	South America	Brazil	polyprotein	Dengue virus 1
ACO06164.1	DENV-1/BR/BID-V2392/2005	Human	2005-01-01	South America	Brazil	polyprotein	Dengue virus 1
ACY70762.1	DENV-1/BR/BID-V3490/2008	Human	2008-01-01	South America	Brazil	polyprotein	Dengue virus 1
AGN94866.1	12898/BR-PE/10	Human	2010-01-01	South America	Brazil	polyprotein	Dengue virus 1
AKQ00014.1	DENV1 BR/SJRP/484/2012	Human	2012-02-13	South America	Brazil	polyprotein	Dengue virus 1
AKQ00010.1	DENV1 BR/SJRP/17/2010	Human	2010-10-13	South America	Brazil	polyprotein	Dengue virus 1
ACY70783.1	DENV-2/BR/BID-V3650/2008	Human	2008-01-01	South America	Brazil	polyprotein	Dengue virus 2
ACW82875.1	DENV-2/BR/BID-V3486/2008	Human	2008-01-01	South America	Brazil	polyprotein	Dengue virus 2
ACO06162.1	DENV-2/BR/BID-V2390/2004	Human	2004-01-01	South America	Brazil	polyprotein	Dengue virus 2
ACS32031.1	DENV-2/BR/BID-V2402/2008	Human	2008-01-01	South America	Brazil	polyprotein	Dengue virus 2
ACO06171.1	DENV-2/BR/BID-V2399/2007	Human	2007-01-01	South America	Brazil	polyprotein	Dengue virus 2
ACY70780.1	DENV-2/BR/BID-V3644/2008	Human	2008-01-01	South America	Brazil	polyprotein	Dengue virus 2
ADI80655.1	DENV-2/BR/BID-V3637/2008	Human	2008-01-01	South America	Brazil	polyprotein	Dengue virus 2
AGK36290.1	ACS46_II	Human	2010-03-01	South America	Brazil	polyprotein	Dengue virus 2

AGK36292.1	ACS542	Human	2010-05-04	South America	Brazil	polyprotein	Dengue virus 2
AGK36297.1	DGV106	Human	2010-04-15	South America	Brazil	polyprotein	Dengue virus 2
AKQ00025.1	BR/SJRP/779/2013	Human	2013-02-04	South America	Brazil	polyprotein	Dengue virus 2
AKQ00022.1	BR/SJRP/567/2012	Human	2012-10-31	South America	Brazil	polyprotein	Dengue virus 2
AKQ00021.1	BR/SJRP/350/2008	Human	2008-06-17	South America	Brazil	polyprotein	Dengue virus 2
QDZ58871.1	BD-RP	Human	2019-01-02	South America	Brazil	polyprotein	Dengue virus 2
QOW96380.1	USP-CB-96	Human	2019-04-18	South America	Brazil	polyprotein	Dengue virus 2
QOW96384.1	USP-CB-110	Human	2019-05-21	South America	Brazil	polyprotein	Dengue virus 2
ACO06165.1	DENV-2/BR/BID-V2393/2005	Human	2005-01-01	South America	Brazil	polyprotein	Dengue virus 2
AGK36291.1	ACS538	Human	2010-04-12	South America	Brazil	polyprotein	Dengue virus 2
AGK36294.1	ACS721	Human	2010-05-04	South America	Brazil	polyprotein	Dengue virus 2
AGN94882.1	19190/BR-PE/10	Human	2010-01-01	South America	Brazil	polyprotein	Dengue virus 2
AKQ00027.1	BR/SJRP/869/2013	Human	2013-02-08	South America	Brazil	polyprotein	Dengue virus 2
AKQ00040.1	BR/SJRP/2298/2014	Human	2014-04-03	South America	Brazil	polyprotein	Dengue virus 2
QOW96379.1	USP-CB-95	Human	2019-04-09	South America	Brazil	polyprotein	Dengue virus 2
QOW96388.1	USP-CB-121	Human	2019-07-11	South America	Brazil	polyprotein	Dengue virus 2
ACO06168.1	DENV-2/BR/BID-V2396/2006	Human	2006-01-01	South America	Brazil	polyprotein	Dengue virus 2
AGK36293.1	DGV37	Human	2010-02-24	South America	Brazil	polyprotein	Dengue virus 2
AGN94880.1	13858/BR-PE/10	Human	2010-01-01	South America	Brazil	polyprotein	Dengue virus 2
AGN94881.1	14905/BR-PE/10	Human	2010-01-01	South America	Brazil	polyprotein	Dengue virus 2
AKQ00024.1	BR/SJRP/615/2013	Human	2013-01-10	South America	Brazil	polyprotein	Dengue virus 2
AKQ00020.1	BR/SJRP/327/2006	Human	2006-05-26	South America	Brazil	polyprotein	Dengue virus 2
QBL56211.1	BeAr849487	Unknown	2017-03-28	South America	Brazil	polyprotein	Dengue virus 2
AGK36298.1	DGV69	Human	2010-03-09	South America	Brazil	polyprotein	Dengue virus 2
AGN94883.1	9479/BR-PE/10	Human	2010-01-01	South America	Brazil	polyprotein	Dengue virus 2
AKQ00026.1	BR/SJRP/846/2013	Human	2013-02-07	South America	Brazil	polyprotein	Dengue virus 2
QTX93212.1	BR-161	Human	2004-01-01	South America	Brazil	polyprotein	Dengue virus 2
ACY70744.1	DENV-3/BR/BID-V3423/2006	Human	2006-01-01	South America	Brazil	polyprotein	Dengue virus 3
ACY70765.1	DENV-3/BR/BID-V3585/2007	Human	2007-01-01	South America	Brazil	polyprotein	Dengue virus 3
ACY70760.1	DENV-3/BR/BID-V3469/2007	Human	2007-01-01	South America	Brazil	polyprotein	Dengue virus 3
ACY70761.1	DENV-3/BR/BID-V3470/2007	Human	2007-01-01	South America	Brazil	polyprotein	Dengue virus 3
ACY70767.1	DENV-3/BR/BID-V3589/2007	Human	2007-01-01	South America	Brazil	polyprotein	Dengue virus 3
ACY70771.1	DENV-3/BR/BID-V3597/2007	Human	2007-01-01	South America	Brazil	polyprotein	Dengue virus 3
ACY70777.1	DENV-3/BR/BID-V3615/2007	Human	2007-01-01	South America	Brazil	polyprotein	Dengue virus 3

ACY70764.1	DENV-3/BR/BID-V3584/2006	Human	2006-01-01	South America	Brazil	polyprotein	Dengue virus 3
ACY70772.1	DENV-3/BR/BID-V3598/2007	Human	2007-01-01	South America	Brazil	polyprotein	Dengue virus 3
ACY70769.1	DENV-3/BR/BID-V3591/2007	Human	2007-01-01	South America	Brazil	polyprotein	Dengue virus 3
ACY70768.1	DENV-3/BR/BID-V3590/2007	Human	2007-01-01	South America	Brazil	polyprotein	Dengue virus 3
ACY70748.1	DENV-3/BR/BID-V3430/2006	Human	2006-01-01	South America	Brazil	polyprotein	Dengue virus 3
ACY70755.1	DENV-3/BR/BID-V3456/2006	Human	2006-01-01	South America	Brazil	polyprotein	Dengue virus 3
ACO06166.1	DENV-3/BR/BID-V2394/2005	Human	2005-01-01	South America	Brazil	polyprotein	Dengue virus 3
AGN94904.1	249/BR-PE/05	Human	2005-01-01	South America	Brazil	polyprotein	Dengue virus 3
AGN94907.1	314/BR-PE/06	Human	2005-01-01	South America	Brazil	polyprotein	Dengue virus 3
AGN94896.1	206/BR-PE/05	Human	2005-01-01	South America	Brazil	polyprotein	Dengue virus 3
AGN94897.1	339/BR-PE/05	Human	2005-01-01	South America	Brazil	polyprotein	Dengue virus 3
AFK83756.1	D3BR/ACN/2007	Human	2007-01-01	South America	Brazil	polyprotein	Dengue virus 3
ACY70773.1	DENV-3/BR/BID-V3601/2007	Human	2007-01-01	South America	Brazil	polyprotein	Dengue virus 3
ACO06174.1	DENV-3/BR/BID-V2403/2008	Human	2008-01-01	South America	Brazil	polyprotein	Dengue virus 3
ACY70750.1	DENV-3/BR/BID-V3435/2006	Human	2006-01-01	South America	Brazil	polyprotein	Dengue virus 3
ACY70774.1	DENV-3/BR/BID-V3605/2007	Human	2007-01-01	South America	Brazil	polyprotein	Dengue virus 3
AGN94902.1	129/BR-PE/04	Human	2004-01-01	South America	Brazil	polyprotein	Dengue virus 3
AGN94899.1	145/BR-PE/04	Human	2004-01-01	South America	Brazil	polyprotein	Dengue virus 3
AGN94908.1	411/BR-PE/06	Human	2006-01-01	South America	Brazil	polyprotein	Dengue virus 3
AGN94910.1	420/BR-PE/06	Human	2006-01-01	South America	Brazil	polyprotein	Dengue virus 3
AFK83755.1	D3BR/AL95/2009	Human	2009-01-01	South America	Brazil	polyprotein	Dengue virus 3
ACY70770.1	DENV-3/BR/BID-V3593/2007	Human	2007-01-01	South America	Brazil	polyprotein	Dengue virus 3
ACY70776.1	DENV-3/BR/BID-V3609/2007	Human	2007-01-01	South America	Brazil	polyprotein	Dengue virus 3
ACW82871.1	DENV-3/BR/BID-V3444/2006	Human	2006-01-01	South America	Brazil	polyprotein	Dengue virus 3
ACY70757.1	DENV-3/BR/BID-V3460/2006	Human	2006-01-01	South America	Brazil	polyprotein	Dengue virus 3
ACO06163.1	DENV-3/BR/BID-V2391/2004	Human	2004-01-01	South America	Brazil	polyprotein	Dengue virus 3
ACW82872.1	DENV-3/BR/BID-V3463/2006	Human	2006-01-01	South America	Brazil	polyprotein	Dengue virus 3
ACY70756.1	DENV-3/BR/BID-V3457/2006	Human	2006-01-01	South America	Brazil	polyprotein	Dengue virus 3
ACO06172.1	DENV-3/BR/BID-V2400/2007	Human	2007-01-01	South America	Brazil	polyprotein	Dengue virus 3
AGN94898.1	277/BR-PE/05	Human	2005-01-01	South America	Brazil	polyprotein	Dengue virus 3
AGN94900.1	603/BR-PE/06	Human	2006-01-01	South America	Brazil	polyprotein	Dengue virus 3
AGN94903.1	161/BR-PE/04	Human	2004-01-01	South America	Brazil	polyprotein	Dengue virus 3
ACO06169.1	DENV-3/BR/BID-V2397/2006	Human	2006-01-01	South America	Brazil	polyprotein	Dengue virus 3
ACY70749.1	DENV-3/BR/BID-V3434/2006	Human	2006-01-01	South America	Brazil	polyprotein	Dengue virus 3

AEV42062.1	DENV3/BR/D3LIMHO/2006	Human	2006-01-01	South America	Brazil	polyprotein	Dengue virus 3
AGN94905.1	263/BR-PE/05	Human	2005-01-01	South America	Brazil	polyprotein	Dengue virus 3
AGN94901.1	255/BR-PE/05	Human	2005-01-01	South America	Brazil	polyprotein	Dengue virus 3
AFK83754.1	D3BR/BR8/04	Unknown	2004-01-01	South America	Brazil	polyprotein	Dengue virus 3
AEW50183.1	H772854	Human	2010-07-21	South America	Brazil	polyprotein	Dengue virus 4
AFX65867.1	H772852	Human	2010-07-18	South America	Brazil	polyprotein	Dengue virus 4
AFX65875.1	H779652	Human	2011-01-24	South America	Brazil	polyprotein	Dengue virus 4
AFX65870.1	H775222	Human	2010-11-10	South America	Brazil	polyprotein	Dengue virus 4
AFX65878.1	H780556	Human	2011-01-29	South America	Brazil	polyprotein	Dengue virus 4
AFX65880.1	H780571	Human	2011-01-13	South America	Brazil	polyprotein	Dengue virus 4
AFX65879.1	H780563	Human	2011-01-29	South America	Brazil	polyprotein	Dengue virus 4
AKQ00033.1	BR/SJRP/580/2012	Human	2012-12-10	South America	Brazil	polyprotein	Dengue virus 4
AKQ00035.1	BR/SJRP/614/2013	Human	2013-01-10	South America	Brazil	polyprotein	Dengue virus 4
AIQ84228.1	DENV-4/MT/BR28_TVP17914/2012	Human	2012-04-19	South America	Brazil	polyprotein	Dengue virus 4
AIQ84240.1	DENV-4/MT/BR48_TVP17934/2012	Human	2012-03-21	South America	Brazil	polyprotein	Dengue virus 4
AIQ84244.1	DENV-4/MT/BR35_TVP17921/2012	Human	2012-03-30	South America	Brazil	polyprotein	Dengue virus 4
AIQ84242.1	DENV-4/MT/BR44_TVP17930/2012	Human	2012-04-05	South America	Brazil	polyprotein	Dengue virus 4
AIQ84233.1	DENV-4/MT/BR76_TVP17953/2012	Human	2012-04-19	South America	Brazil	polyprotein	Dengue virus 4
AIQ84223.1	DENV-4/MT/BR12_TVP17898/2012	Human	2012-03-28	South America	Brazil	polyprotein	Dengue virus 4
AFX65872.1	H778504	Human	2011-01-11	South America	Brazil	polyprotein	Dengue virus 4
AMP43484.1	BR005AM_2011	Human	2011-03-07	South America	Brazil	polyprotein precursor	Dengue virus 4
AKQ00031.1	BR/SJRP/514/2012	Human	2012-04-02	South America	Brazil	polyprotein	Dengue virus 4
AIQ84245.1	DENV-4/MT/BR33_TVP17919/2012	Human	2012-04-20	South America	Brazil	polyprotein	Dengue virus 4
AIQ84235.1	DENV-4/MT/BR60_TVP17946/2012	Human	2012-03-14	South America	Brazil	polyprotein	Dengue virus 4
AIQ84238.1	DENV-4/MT/BR52_TVP17938/2012	Human	2012-03-20	South America	Brazil	polyprotein	Dengue virus 4
AIQ84220.1	DENV-4/MT/BR2_TVP17888/2012	Human	2012-04-23	South America	Brazil	polyprotein	Dengue virus 4
AIQ84226.1	DENV-4/MT/BR24_TVP17910/2012	Human	2012-04-19	South America	Brazil	polyprotein	Dengue virus 4
AEX91754.1	Br246RR/10	Human	2010-09-08	South America	Brazil	polyprotein precursor	Dengue virus 4
AFX65881.1	H781363	Human	2011-03-18	South America	Brazil	polyprotein	Dengue virus 4
AKQ00036.1	BR/SJRP/733/2013	Human	2013-01-30	South America	Brazil	polyprotein	Dengue virus 4
AKQ00030.1	BR/SJRP/506/2012	Human	2012-03-16	South America	Brazil	polyprotein	Dengue virus 4
ANK35834.1	LRV13/422	Human	2013-01-01	South America	Brazil	polyprotein	Dengue virus 4
AIQ84231.1	DENV-4/MT/BR91_TVP17968/2012	Human	2012-02-03	South America	Brazil	polyprotein	Dengue virus 4
AIQ84221.1	DENV-4/MT/BR8_TVP17894/2012	Human	2012-04-23	South America	Brazil	polyprotein	Dengue virus 4

AIQ84236.1	DENV-4/MT/BR55_TVP17941/2012	Human	2012-03-14	South America	Brazil	polyprotein	Dengue virus 4
AFX65874.1	H779228	Human	2011-01-14	South America	Brazil	polyprotein	Dengue virus 4
AKQ00032.1	BR/SJRP/556/2012	Human	2012-07-11	South America	Brazil	polyprotein	Dengue virus 4
AKQ00034.1	BR/SJRP/610/2013	Human	2013-01-07	South America	Brazil	polyprotein	Dengue virus 4
AKQ00028.1	BR/SJRP/500/2012	Human	2012-03-09	South America	Brazil	polyprotein	Dengue virus 4
AIQ84222.1	DENV-4/MT/BR9_TVP17895/2012	Human	2012-04-18	South America	Brazil	polyprotein	Dengue virus 4

CD4 DENV-1 Epitopes

											NCBI IDs of DENV-1 circulating strains from Brazil, (100% identity=1,green ; else 0, red)																									DENV-1 constructs of vaccines					
Epitope_ID	AA_Seq	Protein_Name	Start	End	Allele	step	Serotype	Method/Technique	Assay Group	Qualitative Measure	ACO06167.1	ACO06161.1	ACO06170.1	AGN94867.1	AGN94869.1	AKQ00018.1	AKQ00012.1	AKQ00011.1	ACO06173.1	AGN94868.1	AGN94865.1	AGN94870.1	AKQ00039.1	AKQ00038.1	QOW96373.1	AKQ00016.1	AKQ00015.1	AKQ00013.1	QOW96372.1	QOW96385.1	ACO06164.1	ACY70762.1	AGN94866.1	AKQ00014.1	AKQ00010.1	TV003	TDEN	DPIV	Dengvaxia	DENVax	TVDV
1067569	LARWGSFKKNGAIKV	C	66	80	HLA-DRB1*07:01	0%	Dengue virus 1	ELISPOT	IFNg release	Positive-Low	0	0	0	0	0	0	0	0	0	0	0	0	0	0	0	0	0	0	0	0	0	0	0	0	0	1	1	1	0	0	0
195246	EISNMLNIMNRRKRT	C	87	101	HLA-DRB1*13:01	0%	Dengue virus 1	ELISPOT	IFNg release	Positive	0	0	0	0	0	0	0	0	0	0	0	0	0	0	0	0	0	0	0	0	0	0	0	0	0	0	0	0	0	0	0
195315	FNMLKRARNRVSTVS	C	13	27	HLA-DRB1*08:02	0%	Dengue virus 1	ELISPOT	IFNg release	Positive	0	0	0	0	0	0	0	0	0	0	0	0	0	0	0	0	0	0	0	0	0	0	0	0	0	1	1	1	0	0	0
195535	ISNMLNIMNRRKRSV	C	88	102	HLA-DRB1*08:02	0%	Dengue virus 1	ELISPOT	IFNg release	Positive	0	0	0	0	0	0	0	0	0	0	0	0	0	0	0	0	0	0	0	0	0	0	0	0	0	1	1	1	0	0	0
195557	KEISNMLNIMNRRKR	C	86	100	HLA-DRB1*13:01	0%	Dengue virus 1	ELISPOT	IFNg release	Positive	0	0	0	0	0	0	0	0	0	0	0	0	0	0	0	0	0	0	0	0	0	0	0	0	0	1	1	1	0	0	0
195560	KEVSNMLNIMNRRKR	C	86	100	HLA-DRB1*13:01	0%	Dengue virus 1	ELISPOT	IFNg release	Positive	0	0	0	0	0	0	0	0	0	0	0	0	0	0	0	0	0	0	0	0	0	0	0	0	0	0	0	0	0	0	0
195805	NMLNIMNRRKRSVTM	C	90	104	HLA-DRB1*11:01	0%	Dengue virus 1	ELISPOT	IFNg release	Positive	0	0	0	0	0	0	0	0	0	0	0	0	0	0	0	0	0	0	0	0	0	0	0	0	0	1	1	1	0	0	0
195883	RGFKKEISNMLNIMN	C	82	96	HLA-DRB1*04:01	0%	Dengue virus 1	ELISPOT	IFNg release	Positive	0	0	0	0	0	0	0	0	0	0	0	0	0	0	0	0	0	0	0	0	0	0	0	0	0	1	1	1	0	0	0
195884	RGFKKEISNMLSIMN	C	82	96	HLA-DRB1*04:01	0%	Dengue virus 1	ELISPOT	IFNg release	Positive	0	0	0	0	0	0	0	0	0	0	0	0	0	0	0	0	0	0	0	0	0	0	0	0	0	0	0	0	0	0	0
196188	VSVLFMLLPTALAFH	C	102	116	HLA-DRB1*01:01	0%	Dengue virus 1	ELISPOT	IFNg release	Positive	0	0	0	0	0	0	0	0	0	0	0	0	0	0	0	0	0	0	0	0	0	0	0	0	0	0	0	0	0	0	0
196195	VTMLFMLLPTALAFH	C	102	116	HLA-DRB1*04:01	0%	Dengue virus 1	ELISPOT	IFNg release	Positive	0	0	0	0	0	0	0	0	0	0	0	0	0	0	0	0	0	0	0	0	0	0	0	0	0	0	0	0	0	0	0
539048	AFMAFLRFLAIPPTA	C	27	41	HLA-DRB1*15:02	0%	Dengue virus 1	ELISPOT	IFNg release	Positive	0	0	0	0	0	0	0	0	0	0	0	0	0	0	0	0	0	0	0	0	0	0	0	0	0	0	0	0	0	0	0
539257	FKKEISNMLNIMNRR	C	84	98	HLA-DRB1*04:03	0%	Dengue virus 1	ELISPOT	IFNg release	Positive	0	0	0	0	0	0	0	0	0	0	0	0	0	0	0	0	0	0	0	0	0	0	0	0	0	1	1	1	0	0	0
539616	LRGFKKEISNMLNIM	C	81	95	HLA-DRB1*10:01	0%	Dengue virus 1	ELISPOT	IFNg release	Positive	0	0	0	0	0	0	0	0	0	0	0	0	0	0	0	0	0	0	0	0	0	0	0	0	0	1	1	1	0	0	0
539681	MLNIRNRRKRSVTML	C	91	105	HLA-DRB1*13:01	0%	Dengue virus 1	ELISPOT	IFNg release	Positive	0	0	0	0	0	0	0	0	0	0	0	0	0	0	0	0	0	0	0	0	0	0	0	0	0	0	0	0	0	0	0
539816	QPSFNMLKRARNRVS	C	10	24	HLA-DRB1*08:03	0%	Dengue virus 1	ELISPOT	IFNg release	Positive	0	0	0	0	0	0	0	0	0	0	0	0	0	0	0	0	0	0	0	0	0	0	0	0	0	0	0	0	0	0	0
539866	RPSFNMLKRVRNRVS	C	10	24	HLA-DRB1*08:03	0%	Dengue virus 1	ELISPOT	IFNg release	Positive	0	0	0	0	0	0	0	0	0	0	0	0	0	0	0	0	0	0	0	0	0	0	0	0	0	0	0	0	0	0	0
540117	WGSFKKNGAIKVLRG	C	47	61	HLA-DRB1*09:01	0%	Dengue virus 1	ELISPOT	IFNg release	Positive	0	0	0	0	0	0	0	0	0	0	0	0	0	0	0	0	0	0	0	0	0	0	0	0	0	1	1	1	0	0	0
740331	SNMLNIMNRRKRSVT	C	89	103	HLA-DRB1*14:02	0%	Dengue virus 1	ELISPOT	IFNg release	Positive	0	0	0	0	0	0	0	0	0	0	0	0	0	0	0	0	0	0	0	0	0	0	0	0	0	1	1	1	0	0	0
195361	GGVFTSVGKLVHQIF	E	426	440	HLA-DRB1*11:01	0%	Dengue virus 1	ELISPOT	IFNg release	Positive	0	0	0	0	0	0	0	0	0	0	0	0	0	0	0	0	0	0	0	0	0	0	0	0	0	0	0	0	1	0	0
539840	REDLLVTFKTAHAKK	E	513	527	HLA-DRB1*04:03	0%	Dengue virus 1	ELISPOT	IFNg release	Positive	0	0	0	0	0	0	0	0	0	0	0	0	0	0	0	0	0	0	0	0	0	0	0	0	0	0	0	0	0	0	0
539854	RKDLLVTFKTAHAKK	E	513	527	HLA-DRB1*04:03	0%	Dengue virus 1	ELISPOT	IFNg release	Positive	0	0	0	0	0	0	0	0	0	0	0	0	0	0	0	0	0	0	0	0	0	0	0	0	0	0	0	0	0	0	0
867045	HAKKQEVAVLGSQEG	E	244	258	HLA-DQA1*03:01/DQB1*03:02	0%	Dengue virus 1	ELISPOT	IFNg release	Positive	0	0	0	0	0	0	0	0	0	0	0	0	0	0	0	0	0	0	0	0	0	0	0	0	0	0	0	0	0	0	0
869085	SYIVIGAGKKALKLS	E	376	390	HLA-DRB5*01:01	0%	Dengue virus 1	ELISPOT	IFNg release	Positive	0	0	0	0	0	0	0	0	0	0	0	0	0	0	0	0	0	0	0	0	0	0	0	0	0	0	0	0	0	0	0
869690	WLAHKQWFLDLPLPW	E	206	220	HLA-DQA1*01:01/DQB1*05:01	0%	Dengue virus 1	ELISPOT	IFNg release	Positive	0	0	0	0	0	0	0	0	0	0	0	0	0	0	0	0	0	0	0	0	0	0	0	0	0	0	0	0	0	0	0
195684	LNHILLENDIKFTVV	NS1	850	864	HLA-DRB1*03:01	0%	Dengue virus 1	ELISPOT	IFNg release	Positive	0	0	0	0	0	0	0	0	0	0	0	0	0	0	0	0	0	0	0	0	0	0	0	0	0	0	0	0	0	0	0
195785	NETWKLTRASFIEVK	NS1	982	996	HLA-DRB1*07:01	0%	Dengue virus 1	ELISPOT	IFNg release	Positive	0	0	0	0	0	0	0	0	0	0	0	0	0	0	0	0	0	0	0	0	0	0	0	0	0	0	0	0	0	0	0
591686	KFTVVVGDVSGILAQ	NS1	860	874	HLA-DRB1*03:01	0%	Dengue virus 1	ELISPOT	IFNg release	Positive	0	0	0	0	0	0	0	0	0	0	0	0	0	0	0	0	0	0	0	0	0	0	0	0	0	1	1	1	0	0	0
868630	QYKFQADSPKKLSAA	NS1	806	820	HLA-DRB3*02:02	0%	Dengue virus 1	ELISPOT	IFNg release	Positive	0	0	0	0	0	0	0	0	0	0	0	0	0	0	0	0	0	0	0	0	0	0	0	0	0	0	0	0	0	0	0
167816	SLVASVELPNSLEELGDGLAMGIMI	NS2a	109	133	HLA class II	0%	Dengue virus 1	ELISPOT	IFNg release	Positive	0	0	0	0	0	0	0	0	0	0	0	0	0	0	0	0	0	0	0	0	0	0	0	0	0	0	0	0	0	0	0
195285	FAVGLLFRRLTSREI	NS2a	1214	1228	HLA-DRB1*11:01	0%	Dengue virus 1	ELISPOT	IFNg release	Positive	0	0	0	0	0	0	0	0	0	0	0	0	0	0	0	0	0	0	0	0	0	0	0	0	0	0	0	0	0	0	0
195323	FQSHQLWATLLSLTF	NS2a	1267	1281	HLA-DPB1*04:02	0%	Dengue virus 1	ELISPOT	IFNg release	Positive	0	0	0	0	0	0	0	0	0	0	0	0	0	0	0	0	0	0	0	0	0	0	0	0	0	1	1	1	0	0	0
195390	GLLFRRLTSREILLL	NS2a	1217	1231	HLA-DRB1*07:01	0%	Dengue virus 1	ELISPOT	IFNg release	Positive	0	0	0	0	0	0	0	0	0	0	0	0	0	0	0	0	0	0	0	0	0	0	0	0	0	0	0	0	0	0	0
195392	GLLFRRLTSREVPLL	NS2a	1217	1231	HLA-DRB1*10:01	0%	Dengue virus 1	ELISPOT	IFNg release	Positive	0	0	0	0	0	0	0	0	0	0	0	0	0	0	0	0	0	0	0	0	0	0	0	0	0	0	0	0	0	0	0
195423	GTLVVFFLLIMGQLT	NS2a	1162	1176	HLA-DRB1*15:01	0%	Dengue virus 1	ELISPOT	IFNg release	Positive	0	0	0	0	0	0	0	0	0	0	0	0	0	0	0	0	0	0	0	0	0	0	0	0	0	0	0	0	0	0	0
195595	KTMAMVLSIVSLFPL	NS2a	1293	1307	HLA-DRB1*03:01	0%	Dengue virus 1	ELISPOT	IFNg release	Positive	0	0	0	0	0	0	0	0	0	0	0	0	0	0	0	0	0	0	0	0	0	0	0	0	0	0	0	0	1	0	0
195614	LAVGLLFRRLTSREV	NS2a	1214	1228	HLA-DRB1*11:01	0%	Dengue virus 1	ELISPOT	IFNg release	Positive	0	0	0	0	0	0	0	0	0	0	0	0	0	0	0	0	0	0	0	0	0	0	0	0	0	0	0	0	0	0	0
195670	LLSLTFIKTTFSLHY	NS2a	1276	1290	HLA-DRB1*07:01	0%	Dengue virus 1	ELISPOT	IFNg release	Positive	0	0	0	0	0	0	0	0	0	0	0	0	0	0	0	0	0	0	0	0	0	0	0	0	0	0	0	0	1	0	0
195745	MAMVLSIVSLFPLCL	NS2a	1295	1309	HLA-DRB1*07:01	0%	Dengue virus 1	ELISPOT	IFNg release	Positive	0	0	0	0	0	0	0	0	0	0	0	0	0	0	0	0	0	0	0	0	0	0	0	0	0	0	0	0	1	0	0
195756	MGLLFRRLTSREVLL	NS2a	1216	1230	HLA-DRB1*11:01	0%	Dengue virus 1	ELISPOT	IFNg release	Positive	0	0	0	0	0	0	0	0	0	0	0	0	0	0	0	0	0	0	0	0	0	0	0	0	0	0	0	0	0	0	0
195881	REVLLLTVGLSLVAC	NS2a	1226	1240	HLA-DRB1*07:01	0%	Dengue virus 1	ELISPOT	IFNg release	Positive	0	0	0	0	0	0	0	0	0	0	0	0	0	0	0	0	0	0	0	0	0	0	0	0	0	0	0	0	0	0	0
196216	WKTMAMVLSIVSLFP	NS2a	1292	1306	HLA-DRB1*08:02	0%	Dengue virus 1	ELISPOT	IFNg release	Positive	0	0	0	0	0	0	0	0	0	0	0	0	0	0	0	0	0	0	0	0	0	0	0	0	0	0	0	0	1	0	0
539086	AMGIMILKLLTDFQS	NS2a	1255	1269	HLA-DRB1*04:03	0%	Dengue virus 1	ELISPOT	IFNg release	Positive	0	0	0	0	0	0	0	0	0	0	0	0	0	0	0	0	0	0	0	0	0	0	0	0	0	0	0	0	1	0	0
539272	FSLHYAWKTMAMVLS	NS2a	1286	1300	HLA-DRB1*09:01	0%	Dengue virus 1	ELISPOT	IFNg release	Positive	0	0	0	0	0	0	0	0	0	0	0	0	0	0	0	0	0	0	0	0	0	0	0	0	0	0	0	0	1	0	0
539517	KTTFSLHYAWKTMAM	NS2a	1283	1297	HLA-DRB1*09:01	0%	Dengue virus 1	ELISPOT	IFNg release	Positive	0	0	0	0	0	0	0	0	0	0	0	0	0	0	0	0	0	0	0	0	0	0	0	0	0	1	1	1	1	0	0
539624	LSLTFIKTTLSLHYA	NS2a	1277	1291	HLA-DRB1*14:04	0%	Dengue virus 1	ELISPOT	IFNg release	Positive	0	0	0	0	0	0	0	0	0	0	0	0	0	0	0	0	0	0	0	0	0	0	0	0	0	0	0	0	0	0	0
539625	LSLTFIKTTYSLHYA	NS2a	1277	1291	HLA-DRB1*14:04	0%	Dengue virus 1	ELISPOT	IFNg release	Positive	0	0	0	0	0	0	0	0	0	0	0	0	0	0	0	0	0	0	0	0	0	0	0	0	0	0	0	0	0	0	0
539626	LSLTFIRSTIPLVMA	NS2a	1275	1289	HLA-DRB1*14:04	0%	Dengue virus 1	ELISPOT	IFNg release	Positive	0	0	0	0	0	0	0	0	0	0	0	0	0	0	0	0	0	0	0	0	0	0	0	0	0	0	0	0	0	0	0
539651	LVVFLLLIMGQLTWS	NS2a	1164	1178	HLA-DRB1*15:06	0%	Dengue virus 1	ELISPOT	IFNg release	Positive	0	0	0	0	0	0	0	0	0	0	0	0	0	0	0	0	0	0	0	0	0	0	0	0	0	0	0	0	0	0	0
539680	MLMTGTLVVFFLLIM	NS2a	1158	1172	HLA-DRB1*15:06	0%	Dengue virus 1	ELISPOT	IFNg release	Positive	0	0	0	0	0	0	0	0	0	0	0	0	0	0	0	0	0	0	0	0	0	0	0	0	0	0	0	0	0	0	0
539913	SLHYAWKTMAMVLSI	NS2a	1287	1301	HLA-DRB1*09:01	0%	Dengue virus 1	ELISPOT	IFNg release	Positive	0	0	0	0	0	0	0	0	0	0	0	0	0	0	0	0	0	0	0	0	0	0	0	0	0	0	0	0	1	0	0
539918	SLTFIKTTLSLHHAW	NS2a	1278	1292	HLA-DRB1*14:04	0%	Dengue virus 1	ELISPOT	IFNg release	Positive	0	0	0	0	0	0	0	0	0	0	0	0	0	0	0	0	0	0	0	0	0	0	0	0	0	0	0	0	0	0	0
539970	TFSLHYAWKTMAMVL	NS2a	1285	1299	HLA-DRB1*14:06	0%	Dengue virus 1	ELISPOT	IFNg release	Positive	0	0	0	0	0	0	0	0	0	0	0	0	0	0	0	0	0	0	0	0	0	0	0	0	0	0	0	0	1	0	0
539974	TGTLVVFLLLTLGQL	NS2a	1161	1175	HLA-DRB1*15:06	0%	Dengue virus 1	ELISPOT	IFNg release	Positive	0	0	0	0	0	0	0	0	0	0	0	0	0	0	0	0	0	0	0	0	0	0	0	0	0	0	0	0	0	0	0
591779	TLAVFLLLTMGQLTW	NS2a	36	50	HLA-DRB1*11:01	0%	Dengue virus 1	ELISPOT	IFNg release	Positive	0	0	0	0	0	0	0	0	0	0	0	0	0	0	0	0	0	0	0	0	0	0	0	0	0	1	1	1	0	0	0
739424	LAVFLLLVMGQLTWN	NS2a	1164	1178	HLA-DRB1*11:04	0%	Dengue virus 1	ELISPOT	IFNg release	Positive	0	0	0	0	0	0	0	0	0	0	0	0	0	0	0	0	0	0	0	0	0	0	0	0	0	0	0	0	0	0	0
739508	LLCISIMIEEVMRSR	NS2a	1139	1153	HLA-DRB1*11:04	0%	Dengue virus 1	ELISPOT	IFNg release	Positive	0	0	0	0	0	0	0	0	0	0	0	0	0	0	0	0	0	0	0	0	0	0	0	0	0	1	1	1	0	0	0
739578	LSLTFIKTTLSLHHA	NS2a	1277	1291	HLA-DRB1*04:07	0%	Dengue virus 1	ELISPOT	IFNg release	Positive	0	0	0	0	0	0	0	0	0	0	0	0	0	0	0	0	0	0	0	0	0	0	0	0	0	0	0	0	0	0	0
866767	FQSYQLWATLLSLTF	NS2a	1267	1281	HLA-DPB1*04:02	0%	Dengue virus 1	ELISPOT	IFNg release	Positive	0	0	0	0	0	0	0	0	0	0	0	0	0	0	0	0	0	0	0	0	0	0	0	0	0	0	0	0	0	0	0
866873	GILAVFFLLIMGQLT	NS2a	1162	1176	HLA-DPB1*04:02	0%	Dengue virus 1	ELISPOT	IFNg release	Positive	0	0	0	0	0	0	0	0	0	0	0	0	0	0	0	0	0	0	0	0	0	0	0	0	0	0	0	0	0	0	0
867232	ILKLLTDFQSHQLWA	NS2a	1260	1274	HLA-DPB1*01:01	0%	Dengue virus 1	ELISPOT	IFNg release	Positive	0	0	0	0	0	0	0	0	0	0	0	0	0	0	0	0	0	0	0	0	0	0	0	0	0	0	0	0	1	0	0
867997	LTSREILLLTVGLSL	NS2a	1223	1237	HLA-DPB1*04:02	0%	Dengue virus 1	ELISPOT	IFNg release	Positive	0	0	0	0	0	0	0	0	0	0	0	0	0	0	0	0	0	0	0	0	0	0	0	0	0	0	0	0	0	0	0
868553	QPHQLWATLLSLTFV	NS2a	1268	1282	HLA-DPB1*04:02	0%	Dengue virus 1	ELISPOT	IFNg release	Positive	0	0	0	0	0	0	0	0	0	0	0	0	0	0	0	0	0	0	0	0	0	0	0	0	0	0	0	0	0	0	0
866200	ATLFLWYFWQKKKQR	NS2b	1461	1475	HLA-DPB1*01:01	0%	Dengue virus 1	ELISPOT	IFNg release	Positive	0	0	0	0	0	0	0	0	0	0	0	0	0	0	0	0	0	0	0	0	0	0	0	0	0	0	0	0	0	0	0
1392180	GKEIVDLMCHATFTM	NS3	1730	1744		0%	Dengue virus 1	biological activity	activation	Positive	0	0	0	0	0	0	0	0	0	0	0	0	0	0	0	0	0	0	0	0	0	0	0	0	0	1	1	1	0	0	0
195622	LDDGIYRIMQRGLLG	NS3	1493	1507	HLA-DRB1*11:01	0%	Dengue virus 1	ELISPOT	IFNg release	Positive	0	0	0	0	0	0	0	0	0	0	0	0	0	0	0	0	0	0	0	0	0	0	0	0	0	0	0	0	0	0	0
24915	HTGKEIVDLMCHATE	NS3	251	265	HLA-DPw2	0%	Dengue virus 1	ICS	IFNg release	Positive	0	0	0	0	0	0	0	0	0	0	0	0	0	0	0	0	0	0	0	0	0	0	0	0	0	0	0	0	0	0	0
539156	DGIYRIMQRGLLGRS	NS3	1495	1509	HLA-DRB1*12:02	0%	Dengue virus 1	ELISPOT	IFNg release	Positive	0	0	0	0	0	0	0	0	0	0	0	0	0	0	0	0	0	0	0	0	0	0	0	0	0	0	0	0	0	0	0
539309	GIYRIMQRGLLGRSQ	NS3	1496	1510	HLA-DRB1*14:04	0%	Dengue virus 1	ELISPOT	IFNg release	Positive	0	0	0	0	0	0	0	0	0	0	0	0	0	0	0	0	0	0	0	0	0	0	0	0	0	0	0	0	0	0	0
539839	REAIRRKLRTLILAP	NS3	1685	1699	HLA-DRB1*14:04	0%	Dengue virus 1	ELISPOT	IFNg release	Positive	0	0	0	0	0	0	0	0	0	0	0	0	0	0	0	0	0	0	0	0	0	0	0	0	0	0	0	0	0	0	0
539993	TNCLRKNGKRVIQLS	NS3	1848	1862	HLA-DRB1*13:01	0%	Dengue virus 1	ELISPOT	IFNg release	Positive	0	0	0	0	0	0	0	0	0	0	0	0	0	0	0	0	0	0	0	0	0	0	0	0	0	0	0	0	0	0	0
540071	VREAIKKKLRTLILA	NS3	1684	1698	HLA-DRB1*12:02	0%	Dengue virus 1	ELISPOT	IFNg release	Positive	0	0	0	0	0	0	0	0	0	0	0	0	0	0	0	0	0	0	0	0	0	0	0	0	0	0	0	0	0	0	0
540075	VREAIKRKLRTLVLA	NS3	1684	1698	HLA-DRB1*12:02	0%	Dengue virus 1	ELISPOT	IFNg release	Positive	0	0	0	0	0	0	0	0	0	0	0	0	0	0	0	0	0	0	0	0	0	0	0	0	0	1	1	1	0	0	0
54391	RKLTIMDLHPGSGKT	NS3	1662	1676	HLA class II	0%	Dengue virus 1	ICS	IFNg release	Positive	0	0	0	0	0	0	0	0	0	0	0	0	0	0	0	0	0	0	0	0	0	0	0	0	0	0	0	0	0	0	0
738418	AAAIFMTATPPGSME	NS3	1785	1799	HLA-DRB1*04:07	0%	Dengue virus 1	ELISPOT	IFNg release	Positive	0	0	0	0	0	0	0	0	0	0	0	0	0	0	0	0	0	0	0	0	0	0	0	0	0	0	0	0	0	0	0
867025	GVFQENVFHTMWHVT	NS3	1514	1528	HLA-DPB1*04:01	0%	Dengue virus 1	ELISPOT	IFNg release	Positive	0	0	0	0	0	0	0	0	0	0	0	0	0	0	0	0	0	0	0	0	0	0	0	0	0	0	0	0	0	0	0
195680	LMLLALTAVLTGGVT	NS4a	2149	2163	HLA-DRB1*01:01	0%	Dengue virus 1	ELISPOT	IFNg release	Positive	0	0	0	0	0	0	0	0	0	0	0	0	0	0	0	0	0	0	0	0	0	0	0	0	0	0	0	0	0	0	0
196046	TIETLMLLALIAALT	NS4a	2145	2159	HLA-DRB1*01:01	0%	Dengue virus 1	ELISPOT	IFNg release	Positive	0	0	0	0	0	0	0	0	0	0	0	0	0	0	0	0	0	0	0	0	0	0	0	0	0	0	0	0	0	0	0
739085	GVTLFFLSGRGLGKT	NS4a	2161	2175	HLA-DRB1*01:02	0%	Dengue virus 1	ELISPOT	IFNg release	Positive	0	0	0	0	0	0	0	0	0	0	0	0	0	0	0	0	0	0	0	0	0	0	0	0	0	1	1	1	0	0	0
867567	LCVMASSALLWMANV	NS4a	2180	2194	HLA-DQA1*01:02/DQB1*06:02	0%	Dengue virus 1	ELISPOT	IFNg release	Positive	0	0	0	0	0	0	0	0	0	0	0	0	0	0	0	0	0	0	0	0	0	0	0	0	0	0	0	0	0	0	0
867760	LLCVMASSVLLWMAS	NS4a	2179	2193	HLA-DQA1*01:02/DQB1*06:02	0%	Dengue virus 1	ELISPOT	IFNg release	Positive	0	0	0	0	0	0	0	0	0	0	0	0	0	0	0	0	0	0	0	0	0	0	0	0	0	0	0	0	0	0	0
195308	FMLVAHYAIIGPGLQ	NS4b	2357	2371	HLA-DRB1*07:01	0%	Dengue virus 1	ELISPOT	IFNg release	Positive	0	0	0	0	0	0	0	0	0	0	0	0	0	0	0	0	0	0	0	0	0	0	0	0	0	0	0	0	0	0	0
738560	AWTLYAVATTIITPM	NS4b	2282	2296	HLA-DRB1*04:07	0%	Dengue virus 1	ELISPOT	IFNg release	Positive	0	0	0	0	0	0	0	0	0	0	0	0	0	0	0	0	0	0	0	0	0	0	0	0	0	1	1	1	0	0	0
868336	PLTLTAAVLMLAAHY	NS4b	2349	2363	HLA-DQA1*01:02/DQB1*06:02	0%	Dengue virus 1	ELISPOT	IFNg release	Positive	0	0	0	0	0	0	0	0	0	0	0	0	0	0	0	0	0	0	0	0	0	0	0	0	0	0	0	0	0	0	0
195781	NEDHWFSRGNSLSGV	NS5	2985	2999	HLA-DRB1*04:01	0%	Dengue virus 1	ELISPOT	IFNg release	Positive	0	0	0	0	0	0	0	0	0	0	0	0	0	0	0	0	0	0	0	0	0	0	0	0	0	0	0	0	0	0	0
195923	RWLWGFLSRNKKPRI	NS5	2871	2885	HLA-DRB1*15:01	0%	Dengue virus 1	ELISPOT	IFNg release	Positive	0	0	0	0	0	0	0	0	0	0	0	0	0	0	0	0	0	0	0	0	0	0	0	0	0	1	1	1	0	0	0
196191	VTARWLWGFLSRNKK	NS5	2868	2882	HLA-DRB1*15:01	0%	Dengue virus 1	ELISPOT	IFNg release	Positive	0	0	0	0	0	0	0	0	0	0	0	0	0	0	0	0	0	0	0	0	0	0	0	0	0	1	1	1	0	0	0
539509	KSEFNIYKRSGIMEV	NS5	2515	2529	HLA-DRB1*15:02	0%	Dengue virus 1	ELISPOT	IFNg release	Positive	0	0	0	0	0	0	0	0	0	0	0	0	0	0	0	0	0	0	0	0	0	0	0	0	0	0	0	0	0	0	0
539537	LAKAIFKLTYQNKVV	NS5	3056	3070	HLA-DRB1*14:04	0%	Dengue virus 1	ELISPOT	IFNg release	Positive	0	0	0	0	0	0	0	0	0	0	0	0	0	0	0	0	0	0	0	0	0	0	0	0	0	0	0	0	0	0	0
540018	TYGLNTFTNMEAQLI	NS5	3098	3112	HLA-DRB1*15:06	0%	Dengue virus 1	ELISPOT	IFNg release	Positive	0	0	0	0	0	0	0	0	0	0	0	0	0	0	0	0	0	0	0	0	0	0	0	0	0	1	1	1	0	0	0
195113	AFLRFLAIPPTAGIL	C	30	44	HLA-DRB1*01:01	100%	Dengue virus 1	ELISPOT	IFNg release	Positive	1	1	1	1	1	1	1	1	1	1	1	1	1	1	1	1	1	1	1	1	1	1	1	1	1	1	1	1	0	0	0
195117	AGILARWSSFKKNGA	C	51	65	HLA-DRB1*15:01	100%	Dengue virus 1	ELISPOT	IFNg release	Positive	1	1	1	1	1	1	1	1	1	1	1	1	1	1	1	1	1	1	1	1	1	1	1	1	1	0	0	0	0	0	0
195299	FIAFLRFLAIPPTAG	C	28	42	HLA-DRB1*01:01	100%	Dengue virus 1	ELISPOT	IFNg release	Positive	1	1	1	1	1	1	1	1	1	1	1	1	1	1	1	1	1	1	1	1	1	1	1	1	1	1	1	1	0	0	0
195468	IAFLRFLAIPPTAGI	C	29	43	HLA-DRB1*01:01	100%	Dengue virus 1	ELISPOT	IFNg release	Positive	1	1	1	1	1	1	1	1	1	1	1	1	1	1	1	1	1	1	1	1	1	1	1	1	1	1	1	1	0	0	0
195586	KRSVTMLLMLLPTAL	C	87	101	HLA-DRB1*01:01	100%	Dengue virus 1	ELISPOT	IFNg release	Positive	1	1	1	1	1	1	1	1	1	1	1	1	1	1	1	1	1	1	1	1	1	1	1	1	1	1	1	1	0	0	0
195681	LMLLPTALAFHLTTR	C	94	108	HLA-DRB1*04:01	100%	Dengue virus 1	ELISPOT	IFNg release	Positive	1	1	1	1	1	1	1	1	1	1	1	1	1	1	1	1	1	1	1	1	1	1	1	1	1	1	1	1	0	0	0
195904	RPSFNMLKRARNRVS	C	10	24	HLA-DRB1*11:01	100%	Dengue virus 1	ELISPOT	IFNg release	Positive	1	1	1	1	1	1	1	1	1	1	1	1	1	1	1	1	1	1	1	1	1	1	1	1	1	1	1	1	0	0	0
196043	TGRPSFNMLKRARNR	C	8	22	HLA-DRB1*11:01	100%	Dengue virus 1	ELISPOT	IFNg release	Positive	1	1	1	1	1	1	1	1	1	1	1	1	1	1	1	1	1	1	1	1	1	1	1	1	1	1	1	1	0	0	0
196151	VMAFIAFLRFLAIPP	C	25	39	HLA-DRB1*11:01	100%	Dengue virus 1	ELISPOT	IFNg release	Positive	1	1	1	1	1	1	1	1	1	1	1	1	1	1	1	1	1	1	1	1	1	1	1	1	1	1	1	1	0	0	0
196196	VTMLLMLLPTALAFH	C	90	104	HLA-DRB1*04:01	100%	Dengue virus 1	ELISPOT	IFNg release	Positive	1	1	1	1	1	1	1	1	1	1	1	1	1	1	1	1	1	1	1	1	1	1	1	1	1	1	1	1	0	0	0
539043	AFIAFLRFLAIPPTA	C	27	41	HLA-DRB1*12:02	100%	Dengue virus 1	ELISPOT	IFNg release	Positive	1	1	1	1	1	1	1	1	1	1	1	1	1	1	1	1	1	1	1	1	1	1	1	1	1	1	1	1	0	0	0
539259	FKKNGAIKVLRGFKK	C	50	64	HLA-DRB1*08:03	100%	Dengue virus 1	ELISPOT	IFNg release	Positive	1	1	1	1	1	1	1	1	1	1	1	1	1	1	1	1	1	1	1	1	1	1	1	1	1	1	1	1	0	0	0
539495	KNGAIKVLRGFKKEI	C	52	66	HLA-DRB1*12:02	100%	Dengue virus 1	ELISPOT	IFNg release	Positive	1	1	1	1	1	1	1	1	1	1	1	1	1	1	1	1	1	1	1	1	1	1	1	1	1	1	1	1	0	0	0
539613	LRFLAIPPTAGILAR	C	32	46	HLA-DRB1*04:03	100%	Dengue virus 1	ELISPOT	IFNg release	Positive	1	1	1	1	1	1	1	1	1	1	1	1	1	1	1	1	1	1	1	1	1	1	1	1	1	1	1	1	0	0	0
539711	NGAIKVLRGFKKEIS	C	53	67	HLA-DRB1*13:01	100%	Dengue virus 1	ELISPOT	IFNg release	Positive	1	1	1	1	1	1	1	1	1	1	1	1	1	1	1	1	1	1	1	1	1	1	1	1	1	1	1	1	0	0	0
539780	PMKLVMAFIAFLRFL	C	21	35	HLA-DRB1*13:01	100%	Dengue virus 1	ELISPOT	IFNg release	Positive	1	1	1	1	1	1	1	1	1	1	1	1	1	1	1	1	1	1	1	1	1	1	1	1	1	1	1	1	0	0	0
539785	PSFNMLKRARNRVST	C	11	25	HLA-DRB1*10:01	100%	Dengue virus 1	ELISPOT	IFNg release	Positive	1	1	1	1	1	1	1	1	1	1	1	1	1	1	1	1	1	1	1	1	1	1	1	1	1	1	1	1	0	0	0
738927	FLRFLAIPPTAGILA	C	41	55	HLA-DRB1*14:02	100%	Dengue virus 1	ELISPOT	IFNg release	Positive	1	1	1	1	1	1	1	1	1	1	1	1	1	1	1	1	1	1	1	1	1	1	1	1	1	1	1	1	0	0	0
95107	KLVMAFIAFLRFL	C	45	57	HLA-DPw4	100%	Dengue virus 1	ELISPOT	IFNg release	Positive	1	1	1	1	1	1	1	1	1	1	1	1	1	1	1	1	1	1	1	1	1	1	1	1	1	1	1	1	0	0	0
195368	GIIFILLMLVTPSMA	PreM	266	280	HLA-DRB1*01:01	100%	Dengue virus 1	ELISPOT	IFNg release	Positive	1	1	1	1	1	1	1	1	1	1	1	1	1	1	1	1	1	1	1	1	1	1	1	1	1	1	1	1	0	1	1
195562	KGIIFILLMLVTPSM	PreM	265	279	HLA-DRB1*08:02	100%	Dengue virus 1	ELISPOT	IFNg release	Positive	1	1	1	1	1	1	1	1	1	1	1	1	1	1	1	1	1	1	1	1	1	1	1	1	1	1	1	1	0	1	1
195145	AKFKCVTKLEGKIVQ	E	117	131	HLA-DRB1*11:01	100%	Dengue virus 1	ELISPOT	IFNg release	Positive	1	1	1	1	1	1	1	1	1	1	1	1	1	1	1	1	1	1	1	1	1	1	1	1	1	1	1	1	1	1	1
195260	ENLKYSVIVTVHTGD	E	128	142	HLA-DRB1*07:01	100%	Dengue virus 1	ELISPOT	IFNg release	Positive	1	1	1	1	1	1	1	1	1	1	1	1	1	1	1	1	1	1	1	1	1	1	1	1	1	1	1	1	1	1	1
195547	IVQYENLKYSVIVTV	E	124	138	HLA-DRB1*15:02	100%	Dengue virus 1	ELISPOT	IFNg release	Positive	1	1	1	1	1	1	1	1	1	1	1	1	1	1	1	1	1	1	1	1	1	1	1	1	1	1	1	1	1	1	1
195646	LITANPIVTDKEKPV	E	346	360	HLA-DRB1*03:01	100%	Dengue virus 1	ELISPOT	IFNg release	Positive	1	1	1	1	1	1	1	1	1	1	1	1	1	1	1	1	1	1	1	1	1	1	1	1	1	1	1	1	0	1	1
195814	NRQDLLVTFKTAHAK	E	227	241	HLA-DRB1*04:01	100%	Dengue virus 1	ELISPOT	IFNg release	Positive	1	1	1	1	1	1	1	1	1	1	1	1	1	1	1	1	1	1	1	1	1	1	1	1	1	1	1	1	1	1	1
195874	QYENLKYSVIVTVHT	E	126	140	HLA-DRB1*07:01	100%	Dengue virus 1	ELISPOT	IFNg release	Positive	1	1	1	1	1	1	1	1	1	1	1	1	1	1	1	1	1	1	1	1	1	1	1	1	1	1	1	1	1	1	1
539169	DLLVTFKTAHAKKQE	E	230	244	HLA-DRB1*14:04	100%	Dengue virus 1	ELISPOT	IFNg release	Positive	1	1	1	1	1	1	1	1	1	1	1	1	1	1	1	1	1	1	1	1	1	1	1	1	1	1	1	1	1	1	1
539369	GVSWTMKIGIGILLT	E	445	459	HLA-DRB1*15:02	100%	Dengue virus 1	ELISPOT	IFNg release	Positive	1	1	1	1	1	1	1	1	1	1	1	1	1	1	1	1	1	1	1	1	1	1	1	1	1	1	1	1	0	1	1
539378	HAKKQEVVVLGSQEGAMH	E	239	256	HLA class II	100%	Dengue virus 1	ICS	IFNg release	Positive	1	1	1	1	1	1	1	1	1	1	1	1	1	1	1	1	1	1	1	1	1	1	1	1	1	1	1	1	1	1	1
539593	LLVTFKTAHAKKQEV	E	236	250	HLA-DRB1*14:02	100%	Dengue virus 1	ELISPOT	IFNg release	Positive	1	1	1	1	1	1	1	1	1	1	1	1	1	1	1	1	1	1	1	1	1	1	1	1	1	1	1	1	1	1	1
539670	MKIGIGILLTWLGLN	E	450	464	HLA-DRB1*15:06	100%	Dengue virus 1	ELISPOT	IFNg release	Positive	1	1	1	1	1	1	1	1	1	1	1	1	1	1	1	1	1	1	1	1	1	1	1	1	1	1	1	1	0	1	1
539800	QDLLVTFKTAHAKKQ	E	229	243	HLA-DRB1*09:01	100%	Dengue virus 1	ELISPOT	IFNg release	Positive	1	1	1	1	1	1	1	1	1	1	1	1	1	1	1	1	1	1	1	1	1	1	1	1	1	1	1	1	1	1	1
539869	RQDLLVTFKTAHAKK	E	228	242	HLA-DRB1*08:03	100%	Dengue virus 1	ELISPOT	IFNg release	Positive	1	1	1	1	1	1	1	1	1	1	1	1	1	1	1	1	1	1	1	1	1	1	1	1	1	1	1	1	1	1	1
540052	VLGSQEGAMHTALTGA	E	247	262	HLA class II	100%	Dengue virus 1	ICS	IFNg release	Positive	1	1	1	1	1	1	1	1	1	1	1	1	1	1	1	1	1	1	1	1	1	1	1	1	1	1	1	1	1	1	1
540104	VVVLGSQEGAMHTALTG	E	245	261	HLA class II	100%	Dengue virus 1	ICS	IFNg release	Positive	1	1	1	1	1	1	1	1	1	1	1	1	1	1	1	1	1	1	1	1	1	1	1	1	1	1	1	1	1	1	1
741745	MRCVGIGNRDFVEGLSGATW	E	281	300	HLA class II	100%	Dengue virus 1	ELISPOT	IFNg release	Positive	1	1	1	1	1	1	1	1	1	1	1	1	1	1	1	1	1	1	1	1	1	1	1	1	1	1	1	1	1	1	1
867101	HTALTGATEIQTSGT	E	541	555	HLA-DQA1*01:02/DQB1*06:02	100%	Dengue virus 1	ELISPOT	IFNg release	Positive	1	1	1	1	1	1	1	1	1	1	1	1	1	1	1	1	1	1	1	1	1	1	1	1	1	1	1	1	1	1	1
868013	LVHKQWFLDLPLPWT	E	487	501	HLA-DQA1*01:01/DQB1*05:01	100%	Dengue virus 1	ELISPOT	IFNg release	Positive	1	1	1	1	1	1	1	1	1	1	1	1	1	1	1	1	1	1	1	1	1	1	1	1	1	1	1	1	1	1	1
869691	WLVHKQWFLDLPLPW	E	486	500	HLA-DQA1*01:01/DQB1*05:01	100%	Dengue virus 1	ELISPOT	IFNg release	Positive	1	1	1	1	1	1	1	1	1	1	1	1	1	1	1	1	1	1	1	1	1	1	1	1	1	1	1	1	1	1	1
95154	SSIGKMFEATARG	E	396	408	HLA-DQ8	100%	Dengue virus 1	ELISPOT	IFNg release	Positive	1	1	1	1	1	1	1	1	1	1	1	1	1	1	1	1	1	1	1	1	1	1	1	1	1	1	1	1	0	1	1
132583	QYKFQADSPKRLSAA	NS1	31	45	HLA-DRB1*03:01	100%	Dengue virus 1	ELISPOT	IFNg release	Positive	1	1	1	1	1	1	1	1	1	1	1	1	1	1	1	1	1	1	1	1	1	1	1	1	1	1	1	1	0	0	0
195579	KNETWKLARASFIEV	NS1	206	220	HLA-DRB1*07:01	100%	Dengue virus 1	ELISPOT	IFNg release	Positive	1	1	1	1	1	1	1	1	1	1	1	1	1	1	1	1	1	1	1	1	1	1	1	1	1	1	1	1	0	0	0
195784	NETWKLARASFIEVK	NS1	207	221	HLA-DRB1*07:01	100%	Dengue virus 1	ELISPOT	IFNg release	Positive	1	1	1	1	1	1	1	1	1	1	1	1	1	1	1	1	1	1	1	1	1	1	1	1	1	1	1	1	0	0	0

869135	TEQYKFQADSPKRLS	NS1	65	79	HLA-DRB5*01:01	100%	Dengue virus 1	ELISPOT	IFNg release	Positive	1	1	1	1	1	1	1	1	1	1	1	1	1	1	1	1	1	1	1	1	1	1	1	1	1	1	1	1	0	0	0
195708	LSIVSLFPLCLSTTS	NS2a	1299	1313	HLA-DRB1*01:01	100%	Dengue virus 1	ELISPOT	IFNg release	Positive	1	1	1	1	1	1	1	1	1	1	1	1	1	1	1	1	1	1	1	1	1	1	1	1	1	1	1	1	1	0	0
539205	EEVMRSRWSRKMLMT	NS2a	1147	1161	HLA-DRB1*13:01	100%	Dengue virus 1	ELISPOT	IFNg release	Positive	1	1	1	1	1	1	1	1	1	1	1	1	1	1	1	1	1	1	1	1	1	1	1	1	1	1	1	1	0	0	0
539398	IEEVMRSRWSRKMLM	NS2a	1146	1160	HLA-DRB1*13:01	100%	Dengue virus 1	ELISPOT	IFNg release	Positive	1	1	1	1	1	1	1	1	1	1	1	1	1	1	1	1	1	1	1	1	1	1	1	1	1	1	1	1	0	0	0
868100	MGQLTWNDLIRLCIM	NS2a	433	447	HLA-DPB1*04:02	100%	Dengue virus 1	ELISPOT	IFNg release	Positive	1	1	1	1	1	1	1	1	1	1	1	1	1	1	1	1	1	1	1	1	1	1	1	1	1	1	1	1	0	0	0
195228	DTLTILLKATLLAVS	NS2b	1438	1452	HLA-DRB1*08:02	100%	Dengue virus 1	ELISPOT	IFNg release	Positive	1	1	1	1	1	1	1	1	1	1	1	1	1	1	1	1	1	1	1	1	1	1	1	1	1	0	0	0	0	0	0
195724	LTILLKATLLAVSGV	NS2b	1440	1454	HLA-DRB1*08:02	100%	Dengue virus 1	ELISPOT	IFNg release	Positive	1	1	1	1	1	1	1	1	1	1	1	1	1	1	1	1	1	1	1	1	1	1	1	1	1	0	0	0	0	0	0
195878	RDDTLTILLKATLLA	NS2b	1436	1450	HLA-DRB1*12:02	100%	Dengue virus 1	ELISPOT	IFNg release	Positive	1	1	1	1	1	1	1	1	1	1	1	1	1	1	1	1	1	1	1	1	1	1	1	1	1	1	1	1	0	0	0
866201	ATLFVWYFWQKKKQR	NS2b	1461	1475	HLA-DPB1*01:01	100%	Dengue virus 1	ELISPOT	IFNg release	Positive	1	1	1	1	1	1	1	1	1	1	1	1	1	1	1	1	1	1	1	1	1	1	1	1	1	1	1	1	0	0	0
867544	LAGPLIAGGMLIACY	NS2b	1373	1387	HLA-DQA1*01:02/DQB1*06:02	100%	Dengue virus 1	ELISPOT	IFNg release	Positive	1	1	1	1	1	1	1	1	1	1	1	1	1	1	1	1	1	1	1	1	1	1	1	1	1	1	1	1	1	0	0
195163	ANCLRKNGKRVIQLS	NS3	1848	1862	HLA-DRB1*13:01	100%	Dengue virus 1	ELISPOT	IFNg release	Positive	1	1	1	1	1	1	1	1	1	1	1	1	1	1	1	1	1	1	1	1	1	1	1	1	1	0	0	0	0	0	0
195165	ANFRADRVIDPRRCL	NS3	1891	1905	HLA-DRB1*03:01	100%	Dengue virus 1	ELISPOT	IFNg release	Positive	1	1	1	1	1	1	1	1	1	1	1	1	1	1	1	1	1	1	1	1	1	1	1	1	1	1	1	1	0	0	0
195201	CHATFTMRLLSPVRV	NS3	1737	1751	HLA-DRB1*01:01	100%	Dengue virus 1	ELISPOT	IFNg release	Positive	1	1	1	1	1	1	1	1	1	1	1	1	1	1	1	1	1	1	1	1	1	1	1	1	1	1	1	1	0	0	0
195242	EGKIVGLYGNGVVTT	NS3	1618	1632	HLA-DRB1*15:01	100%	Dengue virus 1	ELISPOT	IFNg release	Positive	1	1	1	1	1	1	1	1	1	1	1	1	1	1	1	1	1	1	1	1	1	1	1	1	1	1	1	1	0	0	0
195338	FTMRLLSPVRVPNYN	NS3	1741	1755	HLA-DRB1*01:01	100%	Dengue virus 1	ELISPOT	IFNg release	Positive	1	1	1	1	1	1	1	1	1	1	1	1	1	1	1	1	1	1	1	1	1	1	1	1	1	1	1	1	0	0	0
195377	GIYRILQRGLLGRSQ	NS3	1496	1510	HLA-DRB1*11:01	100%	Dengue virus 1	ELISPOT	IFNg release	Positive	1	1	1	1	1	1	1	1	1	1	1	1	1	1	1	1	1	1	1	1	1	1	1	1	1	1	1	1	0	0	0
195379	GKIVGLYGNGVVTTS	NS3	1619	1633	HLA-DRB1*15:01	100%	Dengue virus 1	ELISPOT	IFNg release	Positive	1	1	1	1	1	1	1	1	1	1	1	1	1	1	1	1	1	1	1	1	1	1	1	1	1	1	1	1	0	0	0
195549	IYRILQRGLLGRSQV	NS3	1497	1511	HLA-DRB1*15:01	100%	Dengue virus 1	ELISPOT	IFNg release	Positive	1	1	1	1	1	1	1	1	1	1	1	1	1	1	1	1	1	1	1	1	1	1	1	1	1	1	1	1	0	0	0
195585	KRKLRTLILAPTRVV	NS3	1689	1703	HLA-DRB1*10:01	100%	Dengue virus 1	ELISPOT	IFNg release	Positive	1	1	1	1	1	1	1	1	1	1	1	1	1	1	1	1	1	1	1	1	1	1	1	1	1	0	0	0	0	0	0
195705	LRTLILAPTRVVASE	NS3	1692	1706	HLA-DRB1*08:02	100%	Dengue virus 1	ELISPOT	IFNg release	Positive	1	1	1	1	1	1	1	1	1	1	1	1	1	1	1	1	1	1	1	1	1	1	1	1	1	0	0	0	0	0	0
195777	NCLRKNGKRVIQLSR	NS3	1849	1863	HLA-DRB1*11:01	100%	Dengue virus 1	ELISPOT	IFNg release	Positive	1	1	1	1	1	1	1	1	1	1	1	1	1	1	1	1	1	1	1	1	1	1	1	1	1	0	0	0	0	0	0
195778	NDIANCLRKNGKRVI	NS3	1845	1859	HLA-DRB1*11:01	100%	Dengue virus 1	ELISPOT	IFNg release	Positive	1	1	1	1	1	1	1	1	1	1	1	1	1	1	1	1	1	1	1	1	1	1	1	1	1	0	0	0	0	0	0
195896	RKLRTLILAPTRVVA	NS3	1690	1704	HLA-DRB1*01:01	100%	Dengue virus 1	ELISPOT	IFNg release	Positive	1	1	1	1	1	1	1	1	1	1	1	1	1	1	1	1	1	1	1	1	1	1	1	1	1	0	0	0	0	0	0
195914	RTLILAPTRVVASEM	NS3	1693	1707	HLA-DRB1*10:01	100%	Dengue virus 1	ELISPOT	IFNg release	Positive	1	1	1	1	1	1	1	1	1	1	1	1	1	1	1	1	1	1	1	1	1	1	1	1	1	0	0	0	0	0	0
196138	VLDDGIYRILQRGLL	NS3	1492	1506	HLA-DRB1*11:01	100%	Dengue virus 1	ELISPOT	IFNg release	Positive	1	1	1	1	1	1	1	1	1	1	1	1	1	1	1	1	1	1	1	1	1	1	1	1	1	1	1	1	0	0	0
30397	KEGERKKLRPRWLDA	NS3	585	599	HLA class II	100%	Dengue virus 1	ICS	IFNg release	Positive	1	1	1	1	1	1	1	1	1	1	1	1	1	1	1	1	1	1	1	1	1	1	1	1	1	1	1	1	0	0	0
45669	NREGKIVGLYGNGVV	NS3	141	155	HLA-DRB1*15:01	100%	Dengue virus 1	ICS	IFNg release	Positive	1	1	1	1	1	1	1	1	1	1	1	1	1	1	1	1	1	1	1	1	1	1	1	1	1	1	1	1	0	0	0
49768	PTRVVASEMAEALKG	NS3	224	238	HLA-DRB1*15:01	100%	Dengue virus 1	ICS	TNF release	Positive	1	1	1	1	1	1	1	1	1	1	1	1	1	1	1	1	1	1	1	1	1	1	1	1	1	1	1	1	0	0	0
539155	DGIYRILQRGLLGRS	NS3	1495	1509	HLA-DRB1*12:02	100%	Dengue virus 1	ELISPOT	IFNg release	Positive	1	1	1	1	1	1	1	1	1	1	1	1	1	1	1	1	1	1	1	1	1	1	1	1	1	1	1	1	0	0	0
539485	KLRTLILAPTRVVAS	NS3	1691	1705	HLA-DRB1*04:03	100%	Dengue virus 1	ELISPOT	IFNg release	Positive	1	1	1	1	1	1	1	1	1	1	1	1	1	1	1	1	1	1	1	1	1	1	1	1	1	0	0	0	0	0	0
539543	LDDGIYRILQRGLLG	NS3	1493	1507	HLA-DRB1*12:02	100%	Dengue virus 1	ELISPOT	IFNg release	Positive	1	1	1	1	1	1	1	1	1	1	1	1	1	1	1	1	1	1	1	1	1	1	1	1	1	1	1	1	0	0	0
539579	LKGMPIRYQTTAVKS	NS3	1711	1725	HLA-DRB1*04:03	100%	Dengue virus 1	ELISPOT	IFNg release	Positive	1	1	1	1	1	1	1	1	1	1	1	1	1	1	1	1	1	1	1	1	1	1	1	1	1	1	1	1	0	0	0
539715	NGKRVIQLSRKTFDT	NS3	1854	1868	HLA-DRB1*12:02	100%	Dengue virus 1	ELISPOT	IFNg release	Positive	1	1	1	1	1	1	1	1	1	1	1	1	1	1	1	1	1	1	1	1	1	1	1	1	1	0	0	0	0	0	0
539986	TLILAPTRVVASEMA	NS3	1694	1708	HLA-DRB1*13:01	100%	Dengue virus 1	ELISPOT	IFNg release	Positive	1	1	1	1	1	1	1	1	1	1	1	1	1	1	1	1	1	1	1	1	1	1	1	1	1	0	0	0	0	0	0
540040	VGLYGNGVVTTSGTY	NS3	1622	1636	HLA-DRB1*15:06	100%	Dengue virus 1	ELISPOT	IFNg release	Positive	1	1	1	1	1	1	1	1	1	1	1	1	1	1	1	1	1	1	1	1	1	1	1	1	1	1	1	1	0	0	0
540074	VREAIKRKLRTLILA	NS3	1684	1698	HLA-DRB1*12:02	100%	Dengue virus 1	ELISPOT	IFNg release	Positive	1	1	1	1	1	1	1	1	1	1	1	1	1	1	1	1	1	1	1	1	1	1	1	1	1	0	0	0	0	0	0
739269	IVGLYGNGVVTTSGT	NS3	132	146	HLA-DRB1*01:02	100%	Dengue virus 1	ELISPOT	IFNg release	Positive	1	1	1	1	1	1	1	1	1	1	1	1	1	1	1	1	1	1	1	1	1	1	1	1	1	1	1	1	0	0	0
866469	EAIKRKLRTLILAPT	NS3	1686	1700	HLA-DPB1*01:01	100%	Dengue virus 1	ELISPOT	IFNg release	Positive	1	1	1	1	1	1	1	1	1	1	1	1	1	1	1	1	1	1	1	1	1	1	1	1	1	0	0	0	0	0	0
867396	KGMPIRYQTTAVKSE	NS3	1712	1726	HLA-DRB3*02:02	100%	Dengue virus 1	ELISPOT	IFNg release	Positive	1	1	1	1	1	1	1	1	1	1	1	1	1	1	1	1	1	1	1	1	1	1	1	1	1	1	1	1	0	0	0
868113	MIIMDEAHFTDPASI	NS3	1756	1770	HLA-DQA1*01:01/DQB1*05:01	100%	Dengue virus 1	ELISPOT	IFNg release	Positive	1	1	1	1	1	1	1	1	1	1	1	1	1	1	1	1	1	1	1	1	1	1	1	1	1	1	1	1	0	0	0
868343	PNYNMIIMDEAHFTD	NS3	1752	1766	HLA-DQA1*05:01/DQB1*02:01	100%	Dengue virus 1	ELISPOT	IFNg release	Positive	1	1	1	1	1	1	1	1	1	1	1	1	1	1	1	1	1	1	1	1	1	1	1	1	1	1	1	1	0	0	0
869341	TTSGTYVSAIAQAKA	NS3	1631	1645	HLA-DQA1*01:02/DQB1*06:02	100%	Dengue virus 1	ELISPOT	IFNg release	Positive	1	1	1	1	1	1	1	1	1	1	1	1	1	1	1	1	1	1	1	1	1	1	1	1	1	1	1	1	0	0	0
869696	WNTGEEVQVIAVEPG	NS3	1564	1578	HLA-DQA1*03:01/DQB1*03:02	100%	Dengue virus 1	ELISPOT	IFNg release	Positive	1	1	1	1	1	1	1	1	1	1	1	1	1	1	1	1	1	1	1	1	1	1	1	1	1	0	0	0	0	0	0
95169	YRILQRGLLGRSQ	NS3	23	35	HLA-DR1	100%	Dengue virus 1	ELISPOT	IFNg release	Positive	1	1	1	1	1	1	1	1	1	1	1	1	1	1	1	1	1	1	1	1	1	1	1	1	1	1	1	1	0	0	0
195478	IETLMLLALIAVLTG	NS4a	2146	2160	HLA-DRB1*15:01	100%	Dengue virus 1	ELISPOT	IFNg release	Positive	1	1	1	1	1	1	1	1	1	1	1	1	1	1	1	1	1	1	1	1	1	1	1	1	1	1	1	1	0	0	0
196214	WIAASIILEFFLMVL	NS4a	2198	2212	HLA-DRB1*15:01	100%	Dengue virus 1	ELISPOT	IFNg release	Positive	1	1	1	1	1	1	1	1	1	1	1	1	1	1	1	1	1	1	1	1	1	1	1	1	1	1	1	1	0	0	0
196255	YVVIGLLFMILTVAA	NS4a	2230	2244	HLA-DRB1*11:01	100%	Dengue virus 1	ELISPOT	IFNg release	Positive	1	1	1	1	1	1	1	1	1	1	1	1	1	1	1	1	1	1	1	1	1	1	1	1	1	1	1	1	0	0	0
539383	HLTLRAQNALDNLVM	NS4a	2110	2124	HLA-DRB3*02:02	100%	Dengue virus 1	ELISPOT	IFNg release	Positive	1	1	1	1	1	1	1	1	1	1	1	1	1	1	1	1	1	1	1	1	1	1	1	1	1	0	0	0	0	0	0
539744	NQLAYVVIGLLFMIL	NS4a	2226	2240	HLA-DRB1*15:02	100%	Dengue virus 1	ELISPOT	IFNg release	Positive	1	1	1	1	1	1	1	1	1	1	1	1	1	1	1	1	1	1	1	1	1	1	1	1	1	1	1	1	0	0	0
738677	DLILEIGKLPQHLTL	NS4a	2099	2113	HLA-DRB1*11:04	100%	Dengue virus 1	ELISPOT	IFNg release	Positive	1	1	1	1	1	1	1	1	1	1	1	1	1	1	1	1	1	1	1	1	1	1	1	1	1	0	0	0	0	0	0
867109	HWIAASIILEFFLMV	NS4a	2197	2211	HLA-DPB1*04:01	100%	Dengue virus 1	ELISPOT	IFNg release	Positive	1	1	1	1	1	1	1	1	1	1	1	1	1	1	1	1	1	1	1	1	1	1	1	1	1	1	1	1	0	0	0
867112	IAASIILEFFLMVLL	NS4a	2199	2213	HLA-DQA1*01:01/DQB1*05:01	100%	Dengue virus 1	ELISPOT	IFNg release	Positive	1	1	1	1	1	1	1	1	1	1	1	1	1	1	1	1	1	1	1	1	1	1	1	1	1	1	1	1	0	0	0
195476	IDLDPVVYDAKFEKQ	NS4b	2398	2412	HLA-DRB1*03:01	100%	Dengue virus 1	ELISPOT	IFNg release	Positive	1	1	1	1	1	1	1	1	1	1	1	1	1	1	1	1	1	1	1	1	1	1	1	1	1	1	1	1	0	0	0
195561	KFWNTTIAVSMANIF	NS4b	2456	2470	HLA-DRB1*07:01	100%	Dengue virus 1	ELISPOT	IFNg release	Positive	1	1	1	1	1	1	1	1	1	1	1	1	1	1	1	1	1	1	1	1	1	1	1	1	1	1	1	1	0	0	0
195620	LCTSQILLMRTTWAL	NS4b	2421	2435	HLA-DRB1*04:01	100%	Dengue virus 1	ELISPOT	IFNg release	Positive	1	1	1	1	1	1	1	1	1	1	1	1	1	1	1	1	1	1	1	1	1	1	1	1	1	1	1	1	0	0	0
195637	LGQIMLLILCTSQIL	NS4b	2413	2427	HLA-DRB1*08:02	100%	Dengue virus 1	ELISPOT	IFNg release	Positive	1	1	1	1	1	1	1	1	1	1	1	1	1	1	1	1	1	1	1	1	1	1	1	1	1	1	1	1	0	0	0
195682	LMLVAHYAIIGPGLQ	NS4b	2357	2371	HLA-DRB1*07:01	100%	Dengue virus 1	ELISPOT	IFNg release	Positive	1	1	1	1	1	1	1	1	1	1	1	1	1	1	1	1	1	1	1	1	1	1	1	1	1	1	1	1	0	0	0
195760	MLLILCTSQILLMRT	NS4b	2417	2431	HLA-DRB1*07:01	100%	Dengue virus 1	ELISPOT	IFNg release	Positive	1	1	1	1	1	1	1	1	1	1	1	1	1	1	1	1	1	1	1	1	1	1	1	1	1	1	1	1	0	0	0
195848	QIMLLILCTSQILLM	NS4b	2415	2429	HLA-DRB1*07:01	100%	Dengue virus 1	ELISPOT	IFNg release	Positive	1	1	1	1	1	1	1	1	1	1	1	1	1	1	1	1	1	1	1	1	1	1	1	1	1	1	1	1	0	0	0
196143	VLMLVAHYAIIGPGL	NS4b	2356	2370	HLA-DRB1*01:01	100%	Dengue virus 1	ELISPOT	IFNg release	Positive	1	1	1	1	1	1	1	1	1	1	1	1	1	1	1	1	1	1	1	1	1	1	1	1	1	1	1	1	0	0	0
539725	NISLTAIANQAAILM	NS4b	2307	2321	HLA-DRB1*04:03	100%	Dengue virus 1	ELISPOT	IFNg release	Positive	1	1	1	1	1	1	1	1	1	1	1	1	1	1	1	1	1	1	1	1	1	1	1	1	1	1	1	1	0	0	0
740732	WTLYAVATTVITPMM	NS4b	2283	2297	HLA-DRB1*04:07	100%	Dengue virus 1	ELISPOT	IFNg release	Positive	1	1	1	1	1	1	1	1	1	1	1	1	1	1	1	1	1	1	1	1	1	1	1	1	1	0	0	0	0	0	0
866002	AIANQAAILMGLDKG	NS4b	2312	2326	HLA-DQA1*01:02/DQB1*06:02	100%	Dengue virus 1	ELISPOT	IFNg release	Positive	1	1	1	1	1	1	1	1	1	1	1	1	1	1	1	1	1	1	1	1	1	1	1	1	1	1	1	1	0	0	0
1066544	ACLGKSYAQMWQLMY	NS5	3244	3258	HLA-DRB1*04:01	100%	Dengue virus 1	ELISPOT	IFNg release	Positive-Low	1	1	1	1	1	1	1	1	1	1	1	1	1	1	1	1	1	1	1	1	1	1	1	1	1	1	1	1	0	0	0
195186	ATNIQVAINQVRRLI	NS5	3352	3366	HLA-DRB1*03:01	100%	Dengue virus 1	ELISPOT	IFNg release	Positive	1	1	1	1	1	1	1	1	1	1	1	1	1	1	1	1	1	1	1	1	1	1	1	1	1	1	1	1	0	0	0
195208	CVYNMMGKREKKLGE	NS5	2942	2956	HLA-DRB1*11:01	100%	Dengue virus 1	ELISPOT	IFNg release	Positive	1	1	1	1	1	1	1	1	1	1	1	1	1	1	1	1	1	1	1	1	1	1	1	1	1	1	1	1	0	0	0
195236	EEFTRKVRSNAAIGA	NS5	2889	2903	HLA-DRB1*08:02	100%	Dengue virus 1	ELISPOT	IFNg release	Positive	1	1	1	1	1	1	1	1	1	1	1	1	1	1	1	1	1	1	1	1	1	1	1	1	1	1	1	1	0	0	0
195354	GEKWKRQLNQLSKSE	NS5	2503	2517	HLA-DRB1*04:01	100%	Dengue virus 1	ELISPOT	IFNg release	Positive	1	1	1	1	1	1	1	1	1	1	1	1	1	1	1	1	1	1	1	1	1	1	1	1	1	1	1	1	0	0	0
195360	GGMLVRNPLSRNSTH	NS5	2694	2708	HLA-DRB1*08:02	100%	Dengue virus 1	ELISPOT	IFNg release	Positive	1	1	1	1	1	1	1	1	1	1	1	1	1	1	1	1	1	1	1	1	1	1	1	1	1	1	1	1	0	0	0
195385	GLHKLGYILRDISKI	NS5	3003	3017	HLA-DRB1*08:02	100%	Dengue virus 1	ELISPOT	IFNg release	Positive	1	1	1	1	1	1	1	1	1	1	1	1	1	1	1	1	1	1	1	1	1	1	1	1	1	1	1	1	0	0	0
195421	GTGNIVSAVNMTSRM	NS5	2716	2730	HLA-DRB1*08:02	100%	Dengue virus 1	ELISPOT	IFNg release	Positive	1	1	1	1	1	1	1	1	1	1	1	1	1	1	1	1	1	1	1	1	1	1	1	1	1	1	1	1	0	0	0
195438	GYILRDISKIPGGNM	NS5	3008	3022	HLA-DRB1*08:02	100%	Dengue virus 1	ELISPOT	IFNg release	Positive	1	1	1	1	1	1	1	1	1	1	1	1	1	1	1	1	1	1	1	1	1	1	1	1	1	1	1	1	0	0	0
195464	HWFSRENSLSGVEGE	NS5	2988	3002	HLA-DRB1*04:01	100%	Dengue virus 1	ELISPOT	IFNg release	Positive	1	1	1	1	1	1	1	1	1	1	1	1	1	1	1	1	1	1	1	1	1	1	1	1	1	1	1	1	0	0	0
195655	LLATSIFKLTYQNKV	NS5	3055	3069	HLA-DRB1*07:01	100%	Dengue virus 1	ELISPOT	IFNg release	Positive	1	1	1	1	1	1	1	1	1	1	1	1	1	1	1	1	1	1	1	1	1	1	1	1	1	1	1	1	0	0	0
195783	NENYLDYMTSMKRFK	NS5	3368	3382	HLA-DRB1*11:01	100%	Dengue virus 1	ELISPOT	IFNg release	Positive	1	1	1	1	1	1	1	1	1	1	1	1	1	1	1	1	1	1	1	1	1	1	1	1	1	0	0	0	0	0	0
195824	NYLDYMTSMKRFKNE	NS5	3370	3384	HLA-DRB1*14:02	100%	Dengue virus 1	ELISPOT	IFNg release	Positive	1	1	1	1	1	1	1	1	1	1	1	1	1	1	1	1	1	1	1	1	1	1	1	1	1	0	0	0	0	0	0
195956	SIFKLTYQNKVVRVQ	NS5	3059	3073	HLA-DRB1*09:01	100%	Dengue virus 1	ELISPOT	IFNg release	Positive	1	1	1	1	1	1	1	1	1	1	1	1	1	1	1	1	1	1	1	1	1	1	1	1	1	1	1	1	0	0	0
196031	TCVYNMMGKREKKLG	NS5	2941	2955	HLA-DRB1*11:01	100%	Dengue virus 1	ELISPOT	IFNg release	Positive	1	1	1	1	1	1	1	1	1	1	1	1	1	1	1	1	1	1	1	1	1	1	1	1	1	1	1	1	0	0	0
196039	TGNIVSAVNMTSRML	NS5	2717	2731	HLA-DRB1*04:01	100%	Dengue virus 1	ELISPOT	IFNg release	Positive	1	1	1	1	1	1	1	1	1	1	1	1	1	1	1	1	1	1	1	1	1	1	1	1	1	1	1	1	0	0	0
196211	WATNIQVAINQVRRL	NS5	3351	3365	HLA-DRB1*03:01	100%	Dengue virus 1	ELISPOT	IFNg release	Positive	1	1	1	1	1	1	1	1	1	1	1	1	1	1	1	1	1	1	1	1	1	1	1	1	1	1	1	1	0	0	0
539096	ATSIFKLTYQNKVVR	NS5	3057	3071	HLA-DRB1*14:04	100%	Dengue virus 1	ELISPOT	IFNg release	Positive	1	1	1	1	1	1	1	1	1	1	1	1	1	1	1	1	1	1	1	1	1	1	1	1	1	1	1	1	0	0	0
539107	AVNMTSRMLLNRFTM	NS5	2723	2737	HLA-DRB1*15:02	100%	Dengue virus 1	ELISPOT	IFNg release	Positive	1	1	1	1	1	1	1	1	1	1	1	1	1	1	1	1	1	1	1	1	1	1	1	1	1	1	1	1	0	0	0
539350	GTAKLRWFVERNLVK	NS5	2551	2565	HLA-DRB1*09:01	100%	Dengue virus 1	ELISPOT	IFNg release	Positive	1	1	1	1	1	1	1	1	1	1	1	1	1	1	1	1	1	1	1	1	1	1	1	1	1	1	1	1	0	0	0
539435	ISGDDCVVKPIDDRFAT	NS5	3152	3168	HLA class II	100%	Dengue virus 1	ICS	IFNg release	Positive	1	1	1	1	1	1	1	1	1	1	1	1	1	1	1	1	1	1	1	1	1	1	1	1	1	1	1	1	0	0	0
539691	MTSRMLLNRFTMAHR	NS5	17	31	HLA-DRB1*14:06	100%	Dengue virus 1	ELISPOT	IFNg release	Positive	1	1	1	1	1	1	1	1	1	1	1	1	1	1	1	1	1	1	1	1	1	1	1	1	1	1	1	1	0	0	0
539700	MVNGVVRLLTKPWDV	NS5	103	117	HLA-DRB1*12:02	100%	Dengue virus 1	ELISPOT	IFNg release	Positive	1	1	1	1	1	1	1	1	1	1	1	1	1	1	1	1	1	1	1	1	1	1	1	1	1	1	1	1	0	0	0
539702	MVTQIAMTDTTPFGQQR	NS5	2829	2845	HLA class II	100%	Dengue virus 1	ICS	IFNg release	Positive	1	1	1	1	1	1	1	1	1	1	1	1	1	1	1	1	1	1	1	1	1	1	1	1	1	1	1	1	0	0	0
539862	RMLLNRFTMAHRKPT	NS5	20	34	HLA-DRB1*14:02	100%	Dengue virus 1	ELISPOT	IFNg release	Positive	1	1	1	1	1	1	1	1	1	1	1	1	1	1	1	1	1	1	1	1	1	1	1	1	1	1	1	1	0	0	0
540001	TSIFKLTYQNKVVRV	NS5	3058	3072	HLA-DRB1*09:01	100%	Dengue virus 1	ELISPOT	IFNg release	Positive	1	1	1	1	1	1	1	1	1	1	1	1	1	1	1	1	1	1	1	1	1	1	1	1	1	1	1	1	0	0	0
540020	TYGLNTFTNMEVQLI	NS5	3098	3112	HLA-DRB1*04:03	100%	Dengue virus 1	ELISPOT	IFNg release	Positive	1	1	1	1	1	1	1	1	1	1	1	1	1	1	1	1	1	1	1	1	1	1	1	1	1	0	0	0	0	0	0
738814	ENYLDYMTSMKRFKN	NS5	3369	3383	HLA-DRB1*14:02	100%	Dengue virus 1	ELISPOT	IFNg release	Positive	1	1	1	1	1	1	1	1	1	1	1	1	1	1	1	1	1	1	1	1	1	1	1	1	1	0	0	0	0	0	0
740786	YLDYMTSMKRFKNES	NS5	3371	3385	HLA-DRB1*14:02	100%	Dengue virus 1	ELISPOT	IFNg release	Positive	1	1	1	1	1	1	1	1	1	1	1	1	1	1	1	1	1	1	1	1	1	1	1	1	1	0	0	0	0	0	0
866481	EAVEDERFWDLVHRE	NS5	2916	2930	HLA-DQA1*01:01/DQB1*05:01	100%	Dengue virus 1	ELISPOT	IFNg release	Positive	1	1	1	1	1	1	1	1	1	1	1	1	1	1	1	1	1	1	1	1	1	1	1	1	1	1	1	1	0	0	0
867505	KSYAQMWQLMYFHRR	NS5	3248	3262	HLA-DPB1*01:01	100%	Dengue virus 1	ELISPOT	IFNg release	Positive	1	1	1	1	1	1	1	1	1	1	1	1	1	1	1	1	1	1	1	1	1	1	1	1	1	1	1	1	0	0	0
868296	PEHALLATSIFKLTY	NS5	3051	3065	HLA-DPB1*02:01	100%	Dengue virus 1	ELISPOT	IFNg release	Positive	1	1	1	1	1	1	1	1	1	1	1	1	1	1	1	1	1	1	1	1	1	1	1	1	1	1	1	1	0	0	0
868641	RFLEFEALGFMNEDH	NS5	2974	2988	HLA-DPB1*04:01	100%	Dengue virus 1	ELISPOT	IFNg release	Positive	1	1	1	1	1	1	1	1	1	1	1	1	1	1	1	1	1	1	1	1	1	1	1	1	1	1	1	1	0	0	0
869091	TAKLRWFVERNLVKP	NS5	2552	2566	HLA-DQA1*01:01/DQB1*05:01	100%	Dengue virus 1	ELISPOT	IFNg release	Positive	1	1	1	1	1	1	1	1	1	1	1	1	1	1	1	1	1	1	1	1	1	1	1	1	1	1	1	1	0	0	0
195314	FNMLKRARNRVSTGS	C	1	15	HLA-DRB1*08:02	81% - <100%	Dengue virus 1	ELISPOT	IFNg release	Positive	1	1	1	1	1	1	1	1	1	1	1	1	1	1	1	0	0	1	1	1	1	1	1	1	1	0	0	0	0	0	0
195944	SFNMLKRARNRVSTG	C	12	26	HLA-DRB1*11:01	81% - <100%	Dengue virus 1	ELISPOT	IFNg release	Positive	1	1	1	1	1	1	1	1	1	1	1	1	1	1	1	0	0	1	1	1	1	1	1	1	1	0	0	0	0	0	0
540059	VLRGFKKEISSMLNI	C	68	82	HLA-DRB1*04:07	81% - <100%	Dengue virus 1	ELISPOT	IFNg release	Positive	1	1	1	1	1	1	1	1	1	1	1	1	1	1	1	1	1	1	1	1	1	1	1	0	1	0	0	0	0	0	0
195216	DIELLKTEVTNPAVL	E	37	51	HLA-DRB1*04:01	81% - <100%	Dengue virus 1	ELISPOT	IFNg release	Positive	1	1	1	1	1	0	1	1	1	1	1	1	1	0	0	1	1	1	0	1	1	1	1	1	1	1	1	1	1	1	1
195477	IELLKTEVTNPAVLR	E	38	52	HLA-DRB1*04:01	81% - <100%	Dengue virus 1	ELISPOT	IFNg release	Positive	1	1	1	1	1	0	1	1	1	1	1	1	1	0	0	1	1	1	0	1	1	1	1	1	1	1	1	1	1	1	1
195623	LDIELLKTEVTNPAV	E	36	50	HLA-DRB1*04:01	81% - <100%	Dengue virus 1	ELISPOT	IFNg release	Positive	1	1	1	1	1	0	1	1	1	1	1	1	1	0	0	1	1	1	0	1	1	1	1	1	1	1	1	1	1	1	1
196053	TLDIELLKTEVTNPA	E	35	49	HLA-DRB1*04:01	81% - <100%	Dengue virus 1	ELISPOT	IFNg release	Positive	1	1	1	1	1	1	1	1	1	1	1	1	1	1	0	1	1	1	0	1	1	1	1	1	1	1	1	1	1	1	1
867378	KCRLKMDKLTLKGMS	E	564	578	HLA-DRB3*02:02	81% - <100%	Dengue virus 1	ELISPOT	IFNg release	Positive	1	1	1	1	1	1	1	1	1	1	1	1	1	1	0	1	1	1	1	1	1	1	1	1	1	0	0	0	1	1	0
539401	IESEKNETWKLARASFI	NS1	202	218	HLA class II	81% - <100%	Dengue virus 1	ICS	IFNg release	Positive	1	1	1	1	1	1	1	1	1	1	1	1	1	1	0	1	1	1	1	1	1	1	1	1	1	1	1	1	0	0	0
867041	HADMGYWIESEKNET	NS1	231	245	HLA-DQA1*01:01/DQB1*05:01	81% - <100%	Dengue virus 1	ELISPOT	IFNg release	Positive	1	1	1	1	1	1	1	1	1	1	1	1	1	1	0	1	1	1	1	1	1	1	1	1	1	1	1	1	0	0	0
867405	KIIGADIQNTTFIID	NS1	897	911	HLA-DPB1*04:02	81% - <100%	Dengue virus 1	ELISPOT	IFNg release	Positive	1	1	1	1	0	1	1	1	1	1	1	1	1	1	0	1	1	1	0	1	1	1	1	1	1	0	0	0	0	0	0
868574	QRAWNIWEVEDYGFG	NS1	183	197	HLA-DQA1*01:01/DQB1*05:01	81% - <100%	Dengue virus 1	ELISPOT	IFNg release	Positive	1	1	1	1	1	1	1	1	1	1	1	1	1	1	1	1	1	1	0	1	1	1	1	1	1	1	1	1	0	0	0
195286	FAVGLLFRRLTSREV	NS2a	1214	1228	HLA-DRB1*08:03	81% - <100%	Dengue virus 1	ELISPOT	IFNg release	Positive	1	1	0	1	1	1	1	1	1	1	1	1	1	1	1	1	0	1	1	1	0	1	1	1	1	1	1	1	0	0	0
195391	GLLFRRLTSREVLLL	NS2a	1217	1231	HLA-DRB1*08:03	81% - <100%	Dengue virus 1	ELISPOT	IFNg release	Positive	1	1	0	1	1	1	1	1	1	1	1	1	1	1	1	1	0	1	1	1	0	1	1	1	1	1	1	1	0	0	0
195422	GTLAVFLLLIMGQLT	NS2a	1162	1176	HLA-DRB1*01:01	81% - <100%	Dengue virus 1	ELISPOT	IFNg release	Positive	0	1	1	1	1	1	1	1	1	1	1	1	0	1	1	0	1	1	1	1	1	1	1	1	1	0	0	0	0	0	0
195613	LAVFLLLIMGQLTWN	NS2a	1164	1178	HLA-DRB1*15:06	81% - <100%	Dengue virus 1	ELISPOT	IFNg release	Positive	0	1	1	1	1	1	1	1	1	1	1	1	0	1	1	0	1	1	1	1	1	1	1	1	1	0	0	0	0	0	0
539094	ATFKMRPMFAVGLLF	NS2a	1206	1220	HLA-DRB1*01:02	81% - <100%	Dengue virus 1	ELISPOT	IFNg release	Positive	1	1	1	0	1	1	1	1	1	1	0	1	1	1	1	1	1	1	1	1	1	1	0	1	1	0	0	0	0	0	0
539679	MLMTGTLAVFLLLIM	NS2a	1158	1172	HLA-DRB1*15:06	81% - <100%	Dengue virus 1	ELISPOT	IFNg release	Positive	0	1	1	1	1	1	1	1	1	1	1	1	0	1	1	0	1	1	1	1	1	1	1	1	1	0	0	0	0	0	0
539888	RWSRKMLMTGTLAVF	NS2a	1153	1167	HLA-DRB1*04:03	81% - <100%	Dengue virus 1	ELISPOT	IFNg release	Positive	0	1	1	1	1	1	1	1	1	1	1	1	1	1	1	1	1	1	1	1	1	1	1	1	1	1	1	1	0	0	0
540038	VGLLFRRLTSREVLL	NS2a	1216	1230	HLA-DRB1*08:03	81% - <100%	Dengue virus 1	ELISPOT	IFNg release	Positive	1	1	0	1	1	1	1	1	1	1	1	1	1	1	1	1	0	1	1	1	0	1	1	1	1	1	1	1	0	0	0
540155	YLALMATFKMRPMFA	NS2a	1201	1215	HLA-DRB1*13:01	81% - <100%	Dengue virus 1	ELISPOT	IFNg release	Positive	1	1	1	0	1	1	1	1	0	1	0	1	1	1	1	1	1	1	1	1	1	1	0	1	1	0	0	0	0	0	0
738553	AVGLLFRRLTSREVL	NS2a	1215	1229	HLA-DRB1*01:02	81% - <100%	Dengue virus 1	ELISPOT	IFNg release	Positive	1	1	0	1	1	1	1	1	1	1	1	1	1	1	1	1	0	1	1	1	0	1	1	1	1	1	1	1	0	0	0
738679	DLIRLCIMVGANASD	NS2a	1179	1193	HLA-DRB1*11:04	81% - <100%	Dengue virus 1	ELISPOT	IFNg release	Positive	1	1	1	1	1	0	1	1	1	1	1	1	1	0	0	1	1	1	0	1	1	1	1	1	1	1	1	1	0	0	0
868098	MGMGTTYLALMATFK	NS2a	456	470	HLA-DRB5*01:01	81% - <100%	Dengue virus 1	ELISPOT	IFNg release	Positive	1	1	1	0	1	1	1	1	0	1	0	1	1	1	1	1	1	1	1	1	1	1	0	1	1	0	0	0	0	0	0
195508	ILVEVQDDGTMKIKD	NS2b	68	82	HLA-DRB1*03:01	81% - <100%	Dengue virus 1	ELISPOT	IFNg release	Positive	1	1	1	1	1	1	1	1	1	1	1	1	1	1	0	1	1	1	1	1	1	1	1	1	1	1	1	1	0	0	0
195715	LSSLLKNDVPLAGPL	NS2b	1363	1377	HLA-DRB1*03:01	81% - <100%	Dengue virus 1	ELISPOT	IFNg release	Positive	1	1	1	1	1	1	1	1	1	1	1	1	1	1	1	1	1	1	1	1	1	1	1	1	0	1	1	1	1	0	0
195993	SSLLKNDVPLAGPLI	NS2b	1364	1378	HLA-DRB1*03:01	81% - <100%	Dengue virus 1	ELISPOT	IFNg release	Positive	1	1	1	1	1	1	1	1	1	1	1	1	1	1	1	1	1	1	1	1	1	1	1	1	0	1	1	1	1	0	0
196111	VEVQDDGTMKIKDEE	NS2b	1421	1435	HLA-DRB1*03:01	81% - <100%	Dengue virus 1	ELISPOT	IFNg release	Positive	1	1	1	1	1	1	1	1	1	1	1	1	1	1	0	1	1	1	1	1	1	1	1	1	1	1	1	1	0	0	0
867359	IVSILLSSLLKNDVP	NS2b	1358	1372	HLA-DPB1*04:02	81% - <100%	Dengue virus 1	ELISPOT	IFNg release	Positive	1	1	1	1	1	1	1	1	1	1	1	1	1	1	1	1	1	1	1	1	1	1	1	1	0	1	1	1	1	0	0
195143	AIVREAIKRKLRTLI	NS3	1682	1696	HLA-DRB1*11:01	81% - <100%	Dengue virus 1	ELISPOT	IFNg release	Positive	1	1	1	1	1	1	1	1	1	1	1	1	0	1	1	1	1	1	1	1	1	1	1	1	1	0	0	0	0	0	0
195296	FHTMWHVTRGAVLMY	NS3	1521	1535	HLA-DRB1*07:01	81% - <100%	Dengue virus 1	ELISPOT	IFNg release	Positive	1	1	1	1	1	1	1	1	1	1	1	1	1	1	1	1	0	1	1	1	1	1	1	1	1	1	1	1	0	0	0
196197	VTRGAVLMYQGKRLE	NS3	1527	1541	HLA-DRB1*15:01	81% - <100%	Dengue virus 1	ELISPOT	IFNg release	Positive	1	1	1	1	1	1	1	1	1	1	1	1	1	1	1	1	0	1	1	1	1	1	1	1	1	1	1	1	0	0	0
539022	AAAIFMTATPPGSVE	NS3	1785	1799	HLA-DRB1*04:03	81% - <100%	Dengue virus 1	ELISPOT	IFNg release	Positive	1	0	1	1	1	1	1	1	1	1	1	1	1	1	1	1	1	1	0	1	1	1	1	1	1	1	1	1	0	0	0
539027	AAIFMTATPPGSVEA	NS3	1786	1800	HLA-DRB1*10:01	81% - <100%	Dengue virus 1	ELISPOT	IFNg release	Positive	1	0	1	1	1	1	1	1	1	1	1	1	1	1	1	0	1	1	0	1	1	1	1	1	1	1	1	1	0	0	0
539171	DLMCHATFTMRLLSP	NS3	1734	1748	HLA-DRB1*13:01	81% - <100%	Dengue virus 1	ELISPOT	IFNg release	Positive	1	1	1	1	1	1	1	1	1	1	1	1	1	1	1	1	1	0	1	1	1	1	1	1	1	1	1	1	0	0	0
539288	GEAAAIFMTATPPGS	NS3	1783	1797	HLA-DRB1*04:03	81% - <100%	Dengue virus 1	ELISPOT	IFNg release	Positive	1	1	1	1	1	1	1	1	1	1	1	1	1	1	1	1	1	1	0	1	1	1	1	1	1	1	1	1	0	0	0
739540	LPAIVREAIKRKLRT	NS3	1680	1694	HLA-DRB1*14:02	81% - <100%	Dengue virus 1	ELISPOT	IFNg release	Positive	1	1	1	1	1	1	1	1	1	1	1	1	0	1	1	1	1	1	1	1	1	1	1	1	1	1	1	1	0	0	0
866721	FKTPEGEVGAIALDF	NS3	1591	1605	HLA-DQA1*03:01/DQB1*03:02	81% - <100%	Dengue virus 1	ELISPOT	IFNg release	Positive	1	1	1	1	1	1	1	1	1	0	1	1	1	1	1	1	1	0	1	1	1	1	1	1	1	1	1	1	0	0	0
869688	WHVTRGAVLMYQGKR	NS3	1525	1539	HLA-DQA1*01:02/DQB1*06:02	81% - <100%	Dengue virus 1	ELISPOT	IFNg release	Positive	1	1	1	1	1	1	1	1	1	1	1	1	1	1	1	1	0	1	1	1	1	1	1	1	1	1	1	1	0	0	0
195679	LMLLALIAVLTGGVT	NS4a	2149	2163	HLA-DRB1*08:02	81% - <100%	Dengue virus 1	ELISPOT	IFNg release	Positive	1	1	1	1	1	1	1	0	1	1	1	1	1	1	1	1	1	0	1	1	1	1	1	1	1	1	1	1	0	0	0
868132	MLLALIAVLTGGVTL	NS4a	2150	2164	HLA-DPB1*04:02	81% - <100%	Dengue virus 1	ELISPOT	IFNg release	Positive	1	1	1	1	1	1	1	0	1	1	1	1	1	1	1	1	1	0	1	1	1	1	1	1	1	1	1	1	0	0	0
195114	AGAGLAFSLMKSLGG	NS4b	2476	2490	HLA-DRB1*04:01	81% - <100%	Dengue virus 1	ELISPOT	IFNg release	Positive	1	1	1	1	1	1	1	1	1	1	1	1	0	1	1	1	1	1	1	1	1	1	1	1	1	1	1	1	0	0	0

195193	AVLMLVAHYAIIGPG	NS4b	2355	2369	HLA-DRB1*01:01	81% - <100%	Dengue virus 1	ELISPOT	IFNg release	Positive	1	1	0	1	1	1	1	1	1	1	1	1	1	1	1	1	1	1	1	1	0	1	1	1	1	1	1	1	0	0	0
539322	GLAFSLMKSLGGGRR	NS4b	2479	2493	HLA-DRB1*10:01	81% - <100%	Dengue virus 1	ELISPOT	IFNg release	Positive	1	1	1	1	1	1	1	1	1	1	1	1	0	1	1	1	1	1	1	1	1	1	1	1	1	1	1	1	0	0	0
195150	AKWLWGFLSRNKKPR	NS5	2870	2884	HLA-DRB1*15:01	81% - <100%	Dengue virus 1	ELISPOT	IFNg release	Positive	1	1	0	1	1	1	1	1	1	1	1	1	1	1	1	1	1	1	1	1	1	1	1	1	0	0	0	0	0	0	0
195168	AQMWQLMYFHRRDLR	NS5	3251	3265	HLA-DRB1*13:01	81% - <100%	Dengue virus 1	ELISPOT	IFNg release	Positive	1	1	1	1	1	1	1	1	1	1	1	1	1	1	1	0	1	1	1	1	1	1	1	1	1	1	1	1	0	0	0
195212	DDRFATALTALNDMG	NS5	3163	3177	HLA-DRB1*04:01	81% - <100%	Dengue virus 1	ELISPOT	IFNg release	Positive	1	1	1	1	1	1	1	1	1	1	1	1	1	1	0	1	1	1	0	1	1	1	1	1	1	1	1	1	0	0	0
195223	DMGKVRKDIPQWEPS	NS5	3175	3189	HLA-DRB1*03:01	81% - <100%	Dengue virus 1	ELISPOT	IFNg release	Positive	1	1	1	1	0	1	1	1	1	1	1	1	1	1	1	1	1	1	1	1	1	1	1	1	1	1	1	1	0	0	0
195582	KPIDDRFATALTALN	NS5	3160	3174	HLA-DRB1*04:01	81% - <100%	Dengue virus 1	ELISPOT	IFNg release	Positive	1	1	1	1	1	1	1	1	1	1	1	1	1	1	0	1	1	1	0	1	1	1	1	1	1	1	1	1	0	0	0
195755	MGKVRKDIPQWEPSK	NS5	3176	3190	HLA-DRB1*03:01	81% - <100%	Dengue virus 1	ELISPOT	IFNg release	Positive	1	1	1	1	0	1	1	1	1	1	1	1	1	1	1	1	1	1	1	1	1	1	1	1	1	1	1	1	0	0	0
195852	QLIMKDGREIVVPCR	NS5	3207	3221	HLA-DRB1*03:01	81% - <100%	Dengue virus 1	ELISPOT	IFNg release	Positive	1	1	0	1	1	1	1	1	1	1	1	1	1	1	1	1	1	1	1	1	0	1	1	1	1	1	1	1	0	0	0
196220	WLWGFLSRNKKPRIC	NS5	2872	2886	HLA-DRB1*11:01	81% - <100%	Dengue virus 1	ELISPOT	IFNg release	Positive	1	1	0	1	1	1	1	1	1	1	1	1	1	1	1	1	1	1	1	1	1	1	1	1	0	1	1	1	0	0	0
539396	IDDRFATALTALNDM	NS5	3162	3176	HLA-DPB1*01:01	81% - <100%	Dengue virus 1	ELISPOT	IFNg release	Positive	1	1	1	1	1	1	1	1	1	1	1	1	1	1	0	1	1	1	0	1	1	1	1	1	1	1	1	1	0	0	0
539905	SHHFHQLIMKDGREI	NS5	3202	3216	HLA-DRB1*13:01	81% - <100%	Dengue virus 1	ELISPOT	IFNg release	Positive	1	1	0	1	1	1	1	1	1	1	1	1	1	1	1	1	1	1	1	1	0	1	1	1	1	1	1	1	0	0	0
540100	VVKPIDDRFATALTALN	NS5	3157	3173	HLA class II	81% - <100%	Dengue virus 1	ICS	IFNg release	Positive	1	1	1	1	1	1	1	1	1	1	1	1	1	1	0	1	1	1	0	1	1	1	1	1	1	1	1	1	0	0	0
540138	WQLMYFHRRDLRLAA	NS5	3254	3268	HLA-DRB1*14:04	81% - <100%	Dengue virus 1	ELISPOT	IFNg release	Positive	1	1	1	1	1	1	1	1	1	1	1	1	1	1	1	0	1	1	1	1	1	1	1	1	1	1	1	1	0	0	0
195589	KSLLFKTSVGVNMCT	PreM	135	149	HLA-DRB1*07:01	51%-80%	Dengue virus 1	ELISPOT	IFNg release	Positive	1	1	1	0	1	1	0	0	0	1	1	0	1	1	1	1	1	0	1	0	1	1	0	1	1	0	0	0	0	0	0
195831	PGFTVIALFLAHAIG	PreM	245	259	HLA-DRB1*15:01	51%-80%	Dengue virus 1	ELISPOT	IFNg release	Positive	1	1	1	0	1	1	0	0	1	1	1	0	1	1	1	1	1	0	1	1	1	1	0	1	1	1	1	1	0	1	1
196085	TVIALFLAHAIGTSI	PreM	248	262	HLA-DRB1*07:01	51%-80%	Dengue virus 1	ELISPOT	IFNg release	Positive	1	1	1	0	1	1	0	0	1	1	1	0	1	1	1	1	1	0	1	1	1	1	0	1	1	1	1	1	0	1	1
867083	HPGFTVIALFLAHAI	PreM	244	258	HLA-DPB1*02:01	51%-80%	Dengue virus 1	ELISPOT	IFNg release	Positive	1	1	1	0	1	1	0	0	1	1	1	0	1	1	1	1	1	0	1	1	1	1	0	1	1	1	1	1	0	1	1
867912	LRHPGFTVIALFLAH	PreM	242	256	HLA-DPB1*02:01	51%-80%	Dengue virus 1	ELISPOT	IFNg release	Positive	1	1	1	0	1	1	0	0	1	1	1	0	1	1	1	1	1	0	1	1	1	1	0	1	1	1	1	1	0	1	1
869062	SVGVNMCTLIAMDLG	PreM	142	156	HLA-DQA1*01:02/DQB1*06:02	51%-80%	Dengue virus 1	ELISPOT	IFNg release	Positive	1	1	1	0	1	1	0	0	0	1	1	0	1	1	1	1	1	0	1	0	1	1	0	1	1	0	0	0	0	0	0
195359	GGLFTSVGKLIHQVF	E	706	720	HLA-DRB1*11:01	51%-80%	Dengue virus 1	ELISPOT	IFNg release	Positive	1	1	1	0	1	1	0	0	0	1	1	0	1	1	1	1	1	0	1	0	1	1	0	1	1	0	0	0	0	0	0
195484	IGGLFTSVGKLIHQV	E	705	719	HLA-DRB1*07:01	51%-80%	Dengue virus 1	ELISPOT	IFNg release	Positive	1	1	1	0	1	1	0	0	0	1	1	0	1	1	1	1	1	0	1	0	1	1	0	1	1	0	0	0	0	0	0
195690	LNYILLENDMKFTVV	NS1	850	864	HLA-DRB1*03:01	51%-80%	Dengue virus 1	ELISPOT	IFNg release	Positive	1	1	1	0	1	1	0	0	0	1	1	0	0	1	1	0	1	0	0	0	1	1	0	1	1	0	0	0	0	0	0
866284	DDQRAWNIWEVEDYG	NS1	181	195	HLA-DQA1*01:01/DQB1*05:01	51%-80%	Dengue virus 1	ELISPOT	IFNg release	Positive	1	1	1	0	1	1	0	0	0	1	1	0	1	1	0	1	1	0	0	0	1	1	0	1	1	0	0	0	0	0	0
866693	FGIFTTNIWLKLRDS	NS1	196	210	HLA-DPB1*04:01	51%-80%	Dengue virus 1	ELISPOT	IFNg release	Positive	1	1	1	0	1	1	0	0	1	1	1	0	1	1	0	1	1	0	0	0	1	1	0	1	1	1	1	1	0	0	0
868492	QISNELNYILLENDM	NS1	845	859	HLA-DPB1*01:01	51%-80%	Dengue virus 1	ELISPOT	IFNg release	Positive	1	1	1	0	1	1	0	0	0	1	1	0	0	1	1	0	1	0	0	0	1	1	0	1	1	0	0	0	0	0	0
869721	YGFGIFTTNIWLKLR	NS1	194	208	HLA-DPB1*02:01	51%-80%	Dengue virus 1	ELISPOT	IFNg release	Positive	1	1	1	0	1	1	0	0	1	1	1	0	1	1	0	1	1	0	0	0	1	1	0	1	1	1	1	1	0	0	0
195196	AWKTMAMALSIVSLF	NS2a	1291	1305	HLA-DRB1*08:02	51%-80%	Dengue virus 1	ELISPOT	IFNg release	Positive	1	1	1	0	1	1	0	0	0	1	1	0	1	1	1	1	1	0	1	0	1	1	0	1	1	0	0	0	0	0	0
195713	LSLTFIRTSLSLDYA	NS2a	1277	1291	HLA-DRB1*03:01	51%-80%	Dengue virus 1	ELISPOT	IFNg release	Positive	1	1	1	0	1	1	0	0	0	1	1	0	1	1	0	1	1	0	0	0	1	1	0	1	1	0	0	0	0	0	0
195959	SILIEEVMRSRWSRK	NS2a	1143	1157	HLA-DRB1*11:04	51%-80%	Dengue virus 1	ELISPOT	IFNg release	Positive	1	1	1	0	1	1	0	0	0	1	1	0	1	1	1	1	1	0	1	0	1	1	0	1	1	0	0	0	0	0	0
539657	MALSIVSLFPLCLST	NS2a	1297	1311	HLA-DRB1*15:06	51%-80%	Dengue virus 1	ELISPOT	IFNg release	Positive	1	1	1	0	1	1	0	0	0	1	1	0	1	1	1	1	1	0	1	0	1	1	0	1	1	0	0	0	0	0	0
539912	SLDYAWKTMAMALSI	NS2a	1287	1301	HLA-DRB1*09:01	51%-80%	Dengue virus 1	ELISPOT	IFNg release	Positive	1	1	1	0	1	1	0	0	0	1	1	0	1	1	1	1	1	0	0	0	1	1	0	1	1	0	0	0	0	0	0
540006	TTLLSLTFIRTSLSL	NS2a	1274	1288	HLA-DRB1*12:02	51%-80%	Dengue virus 1	ELISPOT	IFNg release	Positive	1	1	1	0	1	1	0	0	0	1	1	0	1	1	0	1	1	0	0	0	1	1	0	1	1	0	0	0	0	0	0
866118	AMALSIVSLFPLCLS	NS2a	1296	1310	HLA-DPB1*04:01	51%-80%	Dengue virus 1	ELISPOT	IFNg release	Positive	1	1	1	0	1	1	0	0	0	1	1	0	1	1	1	1	1	0	1	0	1	1	0	1	1	0	0	0	0	0	0
869103	TDFQPHQLWTTLLSL	NS2a	1265	1279	HLA-DPB1*04:02	51%-80%	Dengue virus 1	ELISPOT	IFNg release	Positive	1	1	1	0	1	1	0	0	0	1	1	0	1	1	1	1	1	0	1	0	1	1	0	1	1	0	0	0	0	0	0
195191	AVGIVSILLSSLLKN	NS2b	1355	1369	HLA-DRB1*08:02	51%-80%	Dengue virus 1	ELISPOT	IFNg release	Positive	1	1	1	0	1	1	0	0	0	1	1	0	1	1	1	1	1	0	1	0	1	1	0	1	0	1	1	1	1	0	0
195510	IMAVGIVSILLSSLL	NS2b	1353	1367	HLA-DRB1*07:01	51%-80%	Dengue virus 1	ELISPOT	IFNg release	Positive	1	1	1	0	1	1	0	0	0	1	1	0	1	1	1	1	1	0	1	0	1	1	0	1	1	1	1	1	1	0	0
195485	IGLLCVMASSALLWM	NS4a	2177	2191	HLA-DRB1*07:01	51%-80%	Dengue virus 1	ELISPOT	IFNg release	Positive	1	1	1	0	1	1	0	0	0	1	1	0	0	1	0	0	1	0	0	0	1	1	0	1	1	0	0	0	0	0	0
739034	GLGKTSIGLLCVMAS	NS4a	2171	2185	HLA-DRB1*11:04	51%-80%	Dengue virus 1	ELISPOT	IFNg release	Positive	1	1	1	0	1	1	0	0	0	1	1	0	1	1	0	1	1	0	0	0	1	1	0	1	1	0	0	0	0	0	0
195441	HATMLDVDLRPASAW	NS4b	2269	2283	HLA-DRB1*03:01	51%-80%	Dengue virus 1	ELISPOT	IFNg release	Positive	1	1	1	0	1	1	0	0	1	1	1	0	1	1	1	1	1	0	1	0	1	1	0	1	1	0	0	0	0	0	0
195455	HHHATMLDVDLRPAS	NS4b	2267	2281	HLA-DRB1*03:01	51%-80%	Dengue virus 1	ELISPOT	IFNg release	Positive	1	1	1	0	1	1	0	0	0	1	1	0	1	1	1	1	1	0	1	0	1	1	0	1	1	0	0	0	0	0	0
738686	DLRPASAWTLYAVAT	NS4b	2276	2290	HLA-DRB1*01:02	51%-80%	Dengue virus 1	ELISPOT	IFNg release	Positive	1	1	1	0	1	1	0	0	1	1	1	0	1	1	1	1	1	0	1	0	1	1	0	1	1	0	0	0	0	0	0
196242	YQNKVVRVQRPAKSG	NS5	3065	3079	HLA-DRB1*08:02	51%-80%	Dengue virus 1	ELISPOT	IFNg release	Positive	1	1	1	0	1	1	0	0	0	1	1	0	1	1	1	1	1	0	1	0	1	1	0	1	1	0	0	0	0	0	0
540083	VRVQRPAKSGTVMDV	NS5	96	110	HLA-DRB1*13:01	51%-80%	Dengue virus 1	ELISPOT	IFNg release	Positive	1	1	1	0	1	1	0	0	0	1	1	0	1	1	1	1	1	0	1	0	1	1	0	1	1	0	0	0	0	0	0
195498	IKVLRGFKKEISNML	C	78	92	HLA-DRB1*04:01	>0% - 50%	Dengue virus 1	ELISPOT	IFNg release	Positive	0	0	0	0	0	0	0	0	0	0	0	0	0	0	0	0	0	0	0	0	0	0	0	1	0	1	1	1	0	0	0
195540	ISSMLNIMNRRKRSV	C	76	90	HLA-DRB1*14:02	>0% - 50%	Dengue virus 1	ELISPOT	IFNg release	Positive	0	0	1	1	0	0	1	1	1	0	0	1	0	0	0	0	0	1	0	1	1	0	1	0	0	0	0	0	0	0	0
195761	MLNIMNRRKRSVTML	C	69	83	HLA-DRB1*08:02	>0% - 50%	Dengue virus 1	ELISPOT	IFNg release	Positive	0	0	1	1	0	0	1	1	1	0	0	1	0	0	0	0	0	1	0	1	1	0	1	0	0	1	1	1	0	0	0
195885	RGFKKEISSMLNIMN	C	60	74	HLA-DRB1*04:01	>0% - 50%	Dengue virus 1	ELISPOT	IFNg release	Positive	0	0	1	1	0	0	1	1	1	0	0	1	0	0	0	0	0	1	0	1	1	0	1	0	0	0	0	0	0	0	0
196146	VLRGFKKEISNMLNI	C	80	94	HLA-DRB1*04:01	>0% - 50%	Dengue virus 1	ELISPOT	IFNg release	Positive	0	0	0	0	0	0	0	0	0	0	0	0	0	0	0	0	0	0	0	0	0	0	0	1	0	1	1	1	0	0	0
39096	LRGFKKEISNML	C	81	92	HLA-DPw4	>0% - 50%	Dengue virus 1	ICS	TNF release	Positive	0	0	0	0	0	0	0	0	0	0	0	0	0	0	0	0	0	0	0	0	0	0	0	1	0	1	1	1	0	0	0
539258	FKKEISSMLNIMNRR	C	62	76	HLA-DRB1*10:01	>0% - 50%	Dengue virus 1	ELISPOT	IFNg release	Positive	0	0	1	1	0	0	1	1	1	0	0	1	0	0	0	0	0	1	0	1	1	0	1	0	0	0	0	0	0	0	0
539278	GAIKVLRGFKKEISN	C	76	90	HLA-DRB1*12:02	>0% - 50%	Dengue virus 1	ELISPOT	IFNg release	Positive	0	0	0	0	0	0	0	0	0	0	0	0	0	0	0	0	0	0	0	0	0	0	0	1	0	1	1	1	0	0	0
539459	KEISSMLNIMNRRKR	C	64	78	HLA-DRB1*14:04	>0% - 50%	Dengue virus 1	ELISPOT	IFNg release	Positive	0	0	1	1	0	0	1	1	1	0	0	1	0	0	0	0	0	1	0	1	1	0	1	0	0	0	0	0	0	0	0
539475	KKEISSMLNIMNRRK	C	63	77	HLA-DRB1*14:04	>0% - 50%	Dengue virus 1	ELISPOT	IFNg release	Positive	0	0	1	1	0	0	1	1	1	0	0	1	0	0	0	0	0	1	0	1	1	0	1	0	0	0	0	0	0	0	0
539617	LRGFKKEISSMLNIM	C	59	73	HLA-DRB1*13:01	>0% - 50%	Dengue virus 1	ELISPOT	IFNg release	Positive	0	0	1	1	0	0	1	1	1	0	0	1	0	0	0	0	0	1	0	1	1	0	1	0	0	0	0	0	0	0	0
867523	KVLRGFKKEISNMLN	C	79	93	HLA-DRB3*02:02	>0% - 50%	Dengue virus 1	ELISPOT	IFNg release	Positive	0	0	0	0	0	0	0	0	0	0	0	0	0	0	0	0	0	0	0	0	0	0	0	1	0	1	1	1	0	0	0
195887	RGKSLLFKTSAGVNM	PreM	111	125	HLA-DRB1*07:01	>0% - 50%	Dengue virus 1	ELISPOT	IFNg release	Positive	0	0	0	1	0	0	1	1	1	0	0	1	0	0	0	0	0	1	0	1	0	0	1	0	0	1	1	1	0	1	1
539516	KSLLFKTSAGVNMCT	PreM	123	137	HLA-DRB1*09:01	>0% - 50%	Dengue virus 1	ELISPOT	IFNg release	Positive	0	0	0	1	0	0	1	1	1	0	0	1	0	0	0	0	0	1	0	1	0	0	1	0	0	1	1	1	0	1	1
195362	GGVFTSVGKLVHQVF	E	426	440	HLA-DRB1*11:01	>0% - 50%	Dengue virus 1	ELISPOT	IFNg release	Positive	0	0	0	1	0	0	1	1	1	0	0	1	0	0	0	0	0	1	0	1	0	0	1	0	0	0	0	0	0	0	0
195685	LNHILLENDMKFTVV	NS1	75	89	HLA-DRB1*03:01	>0% - 50%	Dengue virus 1	ELISPOT	IFNg release	Positive	0	0	0	1	0	0	1	1	1	0	0	1	1	0	0	1	0	1	0	1	0	0	1	0	0	1	1	1	0	0	0
738503	ANGILAQGKKMIRPQ	NS1	868	882	HLA-DRB1*14:06	>0% - 50%	Dengue virus 1	ELISPOT	IFNg release	Positive	0	1	0	1	0	0	1	1	1	0	0	1	0	0	1	0	0	1	1	1	0	0	1	0	0	0	0	0	0	0	0
740562	TVVVGDANGILAQGK	NS1	862	876	HLA-DRB1*01:02	>0% - 50%	Dengue virus 1	ELISPOT	IFNg release	Positive	0	1	0	1	0	0	1	1	1	0	0	1	0	0	1	0	0	1	1	1	0	0	1	0	0	0	0	0	0	0	0
195162	AMVLSIVSLFPLCLS	NS2a	1296	1310	HLA-DRB1*15:01	>0% - 50%	Dengue virus 1	ELISPOT	IFNg release	Positive	0	0	0	1	0	0	1	1	0	0	0	1	0	0	0	0	0	1	0	1	0	0	1	0	0	0	0	0	1	0	0
195389	GLLFRKLTSREVLLL	NS2a	1217	1231	HLA-DRB1*11:01	>0% - 50%	Dengue virus 1	ELISPOT	IFNg release	Positive	0	0	1	0	0	0	0	0	0	0	0	0	0	0	0	0	0	0	0	0	1	0	0	0	0	0	0	0	0	0	0
195665	LLLTIGLSLVASVEL	NS2a	1229	1243	HLA-DRB1*07:01	>0% - 50%	Dengue virus 1	ELISPOT	IFNg release	Positive	0	1	1	1	0	0	1	1	0	0	0	1	0	0	0	0	0	1	0	1	1	0	1	0	0	0	0	0	0	0	0
195674	LLTIGLSLVASVELP	NS2a	1230	1244	HLA-DRB1*07:01	>0% - 50%	Dengue virus 1	ELISPOT	IFNg release	Positive	0	1	1	1	0	0	1	1	0	0	0	1	0	0	0	0	0	1	0	1	1	0	1	0	0	0	0	0	0	0	0
195983	SREVLLLTIGLSLVA	NS2a	1225	1239	HLA-DRB1*15:01	>0% - 50%	Dengue virus 1	ELISPOT	IFNg release	Positive	0	1	1	1	0	0	1	1	0	0	0	1	0	0	0	0	0	1	0	1	1	0	1	0	0	0	0	0	0	0	0
167826	TSGTYVSAIAQAKASQE	NS3	157	173	HLA class II	>0% - 50%	Dengue virus 1	ELISPOT	IFNg release	Positive	0	1	1	1	0	0	1	1	1	0	0	1	0	0	0	0	0	1	0	1	1	0	1	0	0	1	1	1	0	0	0
196130	VILKDGPERVILAGP	NS3	1908	1922	HLA-DRB1*03:01	>0% - 50%	Dengue virus 1	ELISPOT	IFNg release	Positive	0	1	1	1	0	0	1	1	1	0	0	1	0	0	0	0	0	1	0	1	1	0	1	0	0	1	1	1	0	0	0
539902	SGTYVSAIAQAKASQ	NS3	1633	1647	HLA-DRB1*10:01	>0% - 50%	Dengue virus 1	ELISPOT	IFNg release	Positive	0	1	1	1	0	0	1	1	1	0	0	1	0	0	0	0	0	1	0	1	1	0	1	0	0	1	1	1	0	0	0
738417	AAAIFMTATPPGSAE	NS3	1785	1799	HLA-DRB1*04:07	>0% - 50%	Dengue virus 1	ELISPOT	IFNg release	Positive	0	1	0	0	0	0	0	0	0	0	0	0	0	0	0	0	0	0	0	0	0	0	0	0	0	0	0	0	0	0	0
866181	ASQEGPLPEIEDEVF	NS3	1645	1659	HLA-DQA1*05:01/DQB1*02:01	>0% - 50%	Dengue virus 1	ELISPOT	IFNg release	Positive	0	1	1	1	0	0	0	0	1	0	0	1	0	0	0	0	0	0	0	1	1	0	1	0	0	1	1	1	0	0	0
195596	KTSIGLLCVTASSAL	NS4a	2174	2188	HLA-DRB1*07:01	>0% - 50%	Dengue virus 1	ELISPOT	IFNg release	Positive	0	0	0	1	0	0	1	1	1	0	0	1	0	0	1	0	0	1	1	1	0	0	1	0	0	0	0	0	0	0	0
869401	VDGIVAIDLDPVVYD	NS4b	2392	2406	HLA-DQA1*01:01/DQB1*05:01	>0% - 50%	Dengue virus 1	ELISPOT	IFNg release	Positive	0	0	0	1	0	0	1	1	1	0	0	1	0	0	0	0	0	1	0	1	0	0	1	0	0	1	1	1	0	0	0
195147	AKNGTVMDVISRRDQ	NS5	3076	3090	HLA-DRB1*03:01	>0% - 50%	Dengue virus 1	ELISPOT	IFNg release	Positive	0	0	0	1	0	0	1	1	1	0	0	1	0	0	0	0	0	1	0	1	0	0	1	0	0	1	1	1	0	0	0
195786	NGTVMDVISRRDQRG	NS5	3078	3092	HLA-DRB1*03:01	>0% - 50%	Dengue virus 1	ELISPOT	IFNg release	Positive	0	0	0	1	0	0	1	1	1	0	0	1	0	0	0	0	0	1	0	1	0	0	1	0	0	1	1	1	0	0	0
195903	RPAKNGTVMDVISRR	NS5	3074	3088	HLA-DRB1*03:01	>0% - 50%	Dengue virus 1	ELISPOT	IFNg release	Positive	0	0	0	1	0	0	1	1	1	0	0	1	0	0	0	0	0	1	0	1	0	0	1	0	0	1	1	1	0	0	0
196241	YQNKVVRVQRPAKNG	NS5	3065	3079	HLA-DRB1*08:02	>0% - 50%	Dengue virus 1	ELISPOT	IFNg release	Positive	0	0	0	1	0	0	1	1	1	0	0	1	0	0	0	0	0	1	0	1	0	0	1	0	0	1	1	1	0	0	0
740024	QNKVVRVQRPAKNGT	NS5	3066	3080	HLA-DRB1*14:06	>0% - 50%	Dengue virus 1	ELISPOT	IFNg release	Positive	0	0	0	1	0	0	1	1	1	0	0	1	0	0	0	0	0	1	0	1	0	0	1	0	0	1	1	1	0	0	0
740668	VQLIRQMESEGIFLP	NS5	3109	3123	HLA-DRB1*11:04	>0% - 50%	Dengue virus 1	ELISPOT	IFNg release	Positive	0	0	0	1	0	0	1	1	1	1	0	1	0	0	0	0	0	1	0	1	0	0	1	0	0	0	0	0	0	0	0
868240	NKVVRVQRPAKNGTV	NS5	3067	3081	HLA-DRB4*01:01	>0% - 50%	Dengue virus 1	ELISPOT	IFNg release	Positive	0	0	0	1	0	0	1	1	1	0	0	1	0	0	0	0	0	1	0	1	0	0	1	0	0	1	1	1	0	0	0

CD4 DENV-2 Epitopes

											NCBI IDs of DENV-2 circulating strains from Brazil, (100% identity=1,green ; else 0, red)																																			DENV-2 constructs of vaccines					
Epitope_ID	AA_Seq	Protein_Name	Start	End	Allele	step	Serotype	Method/Technique	Assay Group	Qualitative Measure	ACY70783.1	ACW82875.1	ACO06162.1	ACS32031.1	ACO06171.1	ACY70780.1	ADI80655.1	AGK36290.1	AGK36292.1	AGK36297.1	AKQ00025.1	AKQ00022.1	AKQ00021.1	QDZ58871.1	QOW96380.1	QOW96384.1	ACO06165.1	AGK36291.1	AGK36294.1	AGN94882.1	AKQ00027.1	AKQ00040.1	QOW96379.1	QOW96388.1	ACO06168.1	AGK36293.1	AGN94880.1	AGN94881.1	AKQ00024.1	AKQ00020.1	QBL56211.1	AGK36298.1	AGN94883.1	AKQ00026.1	QTX93212.1	TV003	TDEN	DPIV	Dengvaxia	DENVax	TVDV
1066603	ARNTPFNMLKRERNR	C	8	22	HLA-DRB1*11:01	0%	Dengue virus 2	ELISPOT	IFNg release	Positive-Low	0	0	0	0	0	0	0	0	0	0	0	0	0	0	0	0	0	0	0	0	0	0	0	0	0	0	0	0	0	0	0	0	0	0	0	0	1	1	0	0	0
150247	GMIIMLIPTVMAFHL	C	103	117	HLA-DRB1*01:01	0%	Dengue virus 2	ELISPOT	IFNg release	Positive	0	0	0	0	0	0	0	0	0	0	0	0	0	0	0	0	0	0	0	0	0	0	0	0	0	0	0	0	0	0	0	0	0	0	0	0	1	1	0	1	0
150404	LRLFMALVAFLRFLT	C	44	58	HLA-DRB1*11:01	0%	Dengue virus 2	ELISPOT	IFNg release	Positive	0	0	0	0	0	0	0	0	0	0	0	0	0	0	0	0	0	0	0	0	0	0	0	0	0	0	0	0	0	0	0	0	0	0	0	0	0	0	0	0	0
195120	AGMIIMLIPTVMAFH	C	102	116	HLA-DRB1*08:02	0%	Dengue virus 2	ELISPOT	IFNg release	Positive	0	0	0	0	0	0	0	0	0	0	0	0	0	0	0	0	0	0	0	0	0	0	0	0	0	0	0	0	0	0	0	0	0	0	0	0	1	1	0	1	0
195121	AGMIIMLIPTVVAFH	C	102	116	HLA-DRB1*08:02	0%	Dengue virus 2	ELISPOT	IFNg release	Positive	0	0	0	0	0	0	0	0	0	0	0	0	0	0	0	0	0	0	0	0	0	0	0	0	0	0	0	0	0	0	0	0	0	0	0	0	0	0	0	0	0
195369	GIIIMIIPTVMAFHL	C	103	117	HLA-DRB1*08:02	0%	Dengue virus 2	ELISPOT	IFNg release	Positive	0	0	0	0	0	0	0	0	0	0	0	0	0	0	0	0	0	0	0	0	0	0	0	0	0	0	0	0	0	0	0	0	0	0	0	0	0	0	0	0	0
195413	GPLRLFMALVAFLRF	C	42	56	HLA-DRB1*04:01	0%	Dengue virus 2	ELISPOT	IFNg release	Positive	0	0	0	0	0	0	0	0	0	0	0	0	0	0	0	0	0	0	0	0	0	0	0	0	0	0	0	0	0	0	0	0	0	0	0	0	0	0	0	0	0
195432	GVIIMMIPTVMAFHL	C	103	117	HLA-DRB1*08:02	0%	Dengue virus 2	ELISPOT	IFNg release	Positive	0	0	0	0	0	0	0	0	0	0	0	0	0	0	0	0	0	0	0	0	0	0	0	0	0	0	0	0	0	0	0	0	0	0	0	0	0	0	0	0	0
195490	IGRMLNILSRRRRTA	C	88	102	HLA-DRB1*11:01	0%	Dengue virus 2	ELISPOT	IFNg release	Positive	0	0	0	0	0	0	0	0	0	0	0	0	0	0	0	0	0	0	0	0	0	0	0	0	0	0	0	0	0	0	0	0	0	0	0	0	0	0	0	0	0
195496	IIMLIPTVMAFHLTT	C	105	119	HLA-DRB1*01:02	0%	Dengue virus 2	ELISPOT	IFNg release	Positive	0	0	0	0	0	0	0	0	0	0	0	0	0	0	0	0	0	0	0	0	0	0	0	0	0	0	0	0	0	0	0	0	0	0	0	0	1	1	0	1	0
195654	LKLYMALVAFLRFLT	C	44	58	HLA-DRB1*15:01	0%	Dengue virus 2	ELISPOT	IFNg release	Positive	0	0	0	0	0	0	0	0	0	0	0	0	0	0	0	0	0	0	0	0	0	0	0	0	0	0	0	0	0	0	0	0	0	0	0	0	0	0	0	0	0
195910	RTAGVIIMMIPTVVA	C	100	114	HLA-DRB1*08:02	0%	Dengue virus 2	ELISPOT	IFNg release	Positive	0	0	0	0	0	0	0	0	0	0	0	0	0	0	0	0	0	0	0	0	0	0	0	0	0	0	0	0	0	0	0	0	0	0	0	0	0	0	0	0	0
195911	RTASVIVMLIPTVMA	C	100	114	HLA-DRB1*08:02	0%	Dengue virus 2	ELISPOT	IFNg release	Positive	0	0	0	0	0	0	0	0	0	0	0	0	0	0	0	0	0	0	0	0	0	0	0	0	0	0	0	0	0	0	0	0	0	0	0	0	0	0	0	0	0
196084	TVGVIIMLIPTAMAF	C	101	115	HLA-DRB1*08:02	0%	Dengue virus 2	ELISPOT	IFNg release	Positive	0	0	0	0	0	0	0	0	0	0	0	0	0	0	0	0	0	0	0	0	0	0	0	0	0	0	0	0	0	0	0	0	0	0	0	0	0	0	0	0	0
53867	RGFRKEIGRMLNILNRRRS	C	17	35	HLA class II	0%	Dengue virus 2	ELISPOT	IFNg release	Positive	0	0	0	0	0	0	0	0	0	0	0	0	0	0	0	0	0	0	0	0	0	0	0	0	0	0	0	0	0	0	0	0	0	0	0	0	0	0	0	0	0
539046	AFLRFLTIPPTVGIL	C	52	66	HLA-DRB1*10:01	0%	Dengue virus 2	ELISPOT	IFNg release	Positive	0	0	0	0	0	0	0	0	0	0	0	0	0	0	0	0	0	0	0	0	0	0	0	0	0	0	0	0	0	0	0	0	0	0	0	0	0	0	0	0	0
539051	AFVAFLRFLTIPPTA	C	9	23	HLA-DRB1*15:02	0%	Dengue virus 2	ELISPOT	IFNg release	Positive	0	0	0	0	0	0	0	0	0	0	0	0	0	0	0	0	0	0	0	0	0	0	0	0	0	0	0	0	0	0	0	0	0	0	0	0	0	0	0	0	0
539213	EIGRMLNILNKRRRT	C	87	101	HLA-DRB1*13:01	0%	Dengue virus 2	ELISPOT	IFNg release	Positive	0	0	0	0	0	0	0	0	0	0	0	0	0	0	0	0	0	0	0	0	0	0	0	0	0	0	0	0	0	0	0	0	0	0	0	0	0	0	0	0	0
539343	GRMLNIMNRRRRTAG	C	89	103	HLA-DRB1*13:01	0%	Dengue virus 2	ELISPOT	IFNg release	Positive	0	0	0	0	0	0	0	0	0	0	0	0	0	0	0	0	0	0	0	0	0	0	0	0	0	0	0	0	0	0	0	0	0	0	0	0	0	0	0	0	0
539549	LFTALVAFLRFLTIP	C	46	60	HLA-DRB1*15:06	0%	Dengue virus 2	ELISPOT	IFNg release	Positive	0	0	0	0	0	0	0	0	0	0	0	0	0	0	0	0	0	0	0	0	0	0	0	0	0	0	0	0	0	0	0	0	0	0	0	0	0	0	0	0	0
62853	TAGILKRWGTIKKSKANVL	C	62	81	HLA class II	0%	Dengue virus 2	ELISPOT	IFNg release	Positive	0	0	0	0	0	0	0	0	0	0	0	0	0	0	0	0	0	0	0	0	0	0	0	0	0	0	0	0	0	0	0	0	0	0	0	0	0	0	0	0	0
739176	IGRMLNILNKRRRTA	C	88	102	HLA-DRB1*14:02	0%	Dengue virus 2	ELISPOT	IFNg release	Positive	0	0	0	0	0	0	0	0	0	0	0	0	0	0	0	0	0	0	0	0	0	0	0	0	0	0	0	0	0	0	0	0	0	0	0	0	0	0	0	0	0
739177	IGRMLNILNRRRRSA	C	88	102	HLA-DRB1*14:02	0%	Dengue virus 2	ELISPOT	IFNg release	Positive	0	0	0	0	0	0	0	0	0	0	0	0	0	0	0	0	0	0	0	0	0	0	0	0	0	0	0	0	0	0	0	0	0	0	0	0	1	0	0	1	0
739179	IGRMLNILNRRRRSV	C	88	102	HLA-DRB1*14:02	0%	Dengue virus 2	ELISPOT	IFNg release	Positive	0	0	0	0	0	0	0	0	0	0	0	0	0	0	0	0	0	0	0	0	0	0	0	0	0	0	0	0	0	0	0	0	0	0	0	0	0	0	0	0	0
739180	IGRMLNILNRRRRTT	C	88	102	HLA-DRB1*14:02	0%	Dengue virus 2	ELISPOT	IFNg release	Positive	0	0	0	0	0	0	0	0	0	0	0	0	0	0	0	0	0	0	0	0	0	0	0	0	0	0	0	0	0	0	0	0	0	0	0	0	0	0	0	0	0
739182	IGRMLNIMNRRRRTA	C	88	102	HLA-DRB1*14:02	0%	Dengue virus 2	ELISPOT	IFNg release	Positive	0	0	0	0	0	0	0	0	0	0	0	0	0	0	0	0	0	0	0	0	0	0	0	0	0	0	0	0	0	0	0	0	0	0	0	0	0	0	0	0	0
739183	IGRMMNILNRRRRTA	C	88	102	HLA-DRB1*14:02	0%	Dengue virus 2	ELISPOT	IFNg release	Positive	0	0	0	0	0	0	0	0	0	0	0	0	0	0	0	0	0	0	0	0	0	0	0	0	0	0	0	0	0	0	0	0	0	0	0	0	0	0	0	0	0
740579	VAFLRFLTISPTAGI	C	51	65	HLA-DRB1*04:07	0%	Dengue virus 2	ELISPOT	IFNg release	Positive	0	0	0	0	0	0	0	0	0	0	0	0	0	0	0	0	0	0	0	0	0	0	0	0	0	0	0	0	0	0	0	0	0	0	0	0	0	0	0	0	0
866992	GPLKLYMALVAFLRF	C	42	56	HLA-DRB5*01:01	0%	Dengue virus 2	ELISPOT	IFNg release	Positive	0	0	0	0	0	0	0	0	0	0	0	0	0	0	0	0	0	0	0	0	0	0	0	0	0	0	0	0	0	0	0	0	0	0	0	0	0	0	0	0	0
867734	LKGFRKEIGRMLNIL	C	81	95	HLA-DRB3*02:02	0%	Dengue virus 2	ELISPOT	IFNg release	Positive	0	0	0	0	0	0	0	0	0	0	0	0	0	0	0	0	0	0	0	0	0	0	0	0	0	0	0	0	0	0	0	0	0	0	0	0	0	0	0	0	0
867910	LRGFRKEIGRMLNIM	C	81	95	HLA-DRB3*02:02	0%	Dengue virus 2	ELISPOT	IFNg release	Positive	0	0	0	0	0	0	0	0	0	0	0	0	0	0	0	0	0	0	0	0	0	0	0	0	0	0	0	0	0	0	0	0	0	0	0	0	0	0	0	0	0
868674	RLFMALVAFLRFLTI	C	45	59	HLA-DPB1*04:02	0%	Dengue virus 2	ELISPOT	IFNg release	Positive	0	0	0	0	0	0	0	0	0	0	0	0	0	0	0	0	0	0	0	0	0	0	0	0	0	0	0	0	0	0	0	0	0	0	0	0	0	0	0	0	0
150280	HPGFTLMAAILAYTI	PreM	244	258	HLA-DRB1*01:01	0%	Dengue virus 2	ELISPOT	IFNg release	Positive	0	0	0	0	0	0	0	0	0	0	0	0	0	0	0	0	0	0	0	0	0	0	0	0	0	0	0	0	0	0	0	0	0	0	0	0	0	0	0	0	0
195318	FQKILIFILLTAVAP	PreM	263	277	HLA-DRB1*07:01	0%	Dengue virus 2	ELISPOT	IFNg release	Positive	0	0	0	0	0	0	0	0	0	0	0	0	0	0	0	0	0	0	0	0	0	0	0	0	0	0	0	0	0	0	0	0	0	0	0	0	0	0	0	0	0
195320	FQRTLIFILLTAVAP	PreM	263	277	HLA-DRB1*07:01	0%	Dengue virus 2	ELISPOT	IFNg release	Positive	0	0	0	0	0	0	0	0	0	0	0	0	0	0	0	0	0	0	0	0	0	0	0	0	0	0	0	0	0	0	0	0	0	0	0	0	0	0	0	0	0
195322	FQRVLIFILLTAVAP	PreM	263	277	HLA-DRB1*07:01	0%	Dengue virus 2	ELISPOT	IFNg release	Positive	0	0	0	0	0	0	0	0	0	0	0	0	0	0	0	0	0	0	0	0	0	0	0	0	0	0	0	0	0	0	0	0	0	0	0	0	0	0	0	0	0
195504	ILRHPGFTLMAAILA	PreM	241	255	HLA-DRB1*15:01	0%	Dengue virus 2	ELISPOT	IFNg release	Positive	0	0	0	0	0	0	0	0	0	0	0	0	0	0	0	0	0	0	0	0	0	0	0	0	0	0	0	0	0	0	0	0	0	0	0	0	0	0	0	0	0
195863	QRVLIFILLTAVAPS	PreM	264	278	HLA-DRB1*08:02	0%	Dengue virus 2	ELISPOT	IFNg release	Positive	0	0	0	0	0	0	0	0	0	0	0	0	0	0	0	0	0	0	0	0	0	0	0	0	0	0	0	0	0	0	0	0	0	0	0	0	0	0	0	0	0
196141	VLIFILLTAIAPSMT	PreM	266	280	HLA-DRB1*08:02	0%	Dengue virus 2	ELISPOT	IFNg release	Positive	0	0	0	0	0	0	0	0	0	0	0	0	0	0	0	0	0	0	0	0	0	0	0	0	0	0	0	0	0	0	0	0	0	0	0	0	0	0	0	0	0
196232	YFQRVLIFILLTAVT	PreM	262	276	HLA-DRB1*07:01	0%	Dengue virus 2	ELISPOT	IFNg release	Positive	0	0	0	0	0	0	0	0	0	0	0	0	0	0	0	0	0	0	0	0	0	0	0	0	0	0	0	0	0	0	0	0	0	0	0	0	0	0	0	0	0
539881	RVLIFILLTAIAPSM	PreM	265	279	HLA-DRB1*15:06	0%	Dengue virus 2	ELISPOT	IFNg release	Positive	0	0	0	0	0	0	0	0	0	0	0	0	0	0	0	0	0	0	0	0	0	0	0	0	0	0	0	0	0	0	0	0	0	0	0	0	0	0	0	0	0
868650	RHPGFTLMAAILAYT	PreM	243	257	HLA-DQA1*01:02/DQB1*06:02	0%	Dengue virus 2	ELISPOT	IFNg release	Positive	0	0	0	0	0	0	0	0	0	0	0	0	0	0	0	0	0	0	0	0	0	0	0	0	0	0	0	0	0	0	0	0	0	0	0	0	0	0	0	0	0
195793	NLEYTIMITPHSGEE	E	134	148	HLA-DRB1*04:01	0%	Dengue virus 2	ELISPOT	IFNg release	Positive	0	0	0	0	0	0	0	0	0	0	0	0	0	0	0	0	0	0	0	0	0	0	0	0	0	0	0	0	0	0	0	0	0	0	0	0	0	0	0	0	0
539427	IQKKTLVTFKNPHAK	E	512	526	HLA-DRB1*04:03	0%	Dengue virus 2	ELISPOT	IFNg release	Positive	0	0	0	0	0	0	0	0	0	0	0	0	0	0	0	0	0	0	0	0	0	0	0	0	0	0	0	0	0	0	0	0	0	0	0	0	0	0	0	0	0
539462	KEMLVTFKNPHAKRQ	E	514	528	HLA-DRB1*04:03	0%	Dengue virus 2	ELISPOT	IFNg release	Positive	0	0	0	0	0	0	0	0	0	0	0	0	0	0	0	0	0	0	0	0	0	0	0	0	0	0	0	0	0	0	0	0	0	0	0	0	0	0	0	0	0
539479	KKTLVTFKNPHAKKQ	E	514	528	HLA-DRB1*14:04	0%	Dengue virus 2	ELISPOT	IFNg release	Positive	0	0	0	0	0	0	0	0	0	0	0	0	0	0	0	0	0	0	0	0	0	0	0	0	0	0	0	0	0	0	0	0	0	0	0	0	0	0	0	0	0
539629	LSVTLVLVGFVTLYL	E	475	489	HLA-DRB1*15:06	0%	Dengue virus 2	ELISPOT	IFNg release	Positive	0	0	0	0	0	0	0	0	0	0	0	0	0	0	0	0	0	0	0	0	0	0	0	0	0	0	0	0	0	0	0	0	0	0	0	0	0	0	0	0	0
866243	AWMVHRQWFLDLPLP	E	205	219	HLA-DQA1*01:01/DQB1*05:01	0%	Dengue virus 2	ELISPOT	IFNg release	Positive	0	0	0	0	0	0	0	0	0	0	0	0	0	0	0	0	0	0	0	0	0	0	0	0	0	0	0	0	0	0	0	0	0	0	0	0	0	0	0	0	0
867375	KAWLVHRQWFLDLPF	E	204	218	HLA-DQA1*01:01/DQB1*05:01	0%	Dengue virus 2	ELISPOT	IFNg release	Positive	0	0	0	0	0	0	0	0	0	0	0	0	0	0	0	0	0	0	0	0	0	0	0	0	0	0	0	0	0	0	0	0	0	0	0	0	0	0	0	0	0
868347	PPFGDSYVIIGVEPG	E	371	385	HLA-DQA1*03:01/DQB1*03:02	0%	Dengue virus 2	ELISPOT	IFNg release	Positive	0	0	0	0	0	0	0	0	0	0	0	0	0	0	0	0	0	0	0	0	0	0	0	0	0	0	0	0	0	0	0	0	0	0	0	0	0	0	0	0	0
869153	TGLDFNEMVLLQMEN	E	179	193	HLA-DQA1*03:01/DQB1*03:02	0%	Dengue virus 2	ELISPOT	IFNg release	Positive	0	0	0	0	0	0	0	0	0	0	0	0	0	0	0	0	0	0	0	0	0	0	0	0	0	0	0	0	0	0	0	0	0	0	0	1	1	1	1	1	1
180612	LNDTWKIEKASFIEV	NS1	981	995	HLA class II	0%	Dengue virus 2	ELISPOT	IFNg release	Positive	0	0	0	0	0	0	0	0	0	0	0	0	0	0	0	0	0	0	0	0	0	0	0	0	0	0	0	0	0	0	0	0	0	0	0	0	1	1	0	1	0
739191	IKGIMHAGKRSLRPQ	NS1	868	882	HLA-DRB1*14:02	0%	Dengue virus 2	ELISPOT	IFNg release	Positive	0	0	0	0	0	0	0	0	0	0	0	0	0	0	0	0	0	0	0	0	0	0	0	0	0	0	0	0	0	0	0	0	0	0	0	0	0	0	0	0	0
866829	GFGFFTTNIWLKLRE	NS1	934	948	HLA-DPB1*01:01	0%	Dengue virus 2	ELISPOT	IFNg release	Positive	0	0	0	0	0	0	0	0	0	0	0	0	0	0	0	0	0	0	0	0	0	0	0	0	0	0	0	0	0	0	0	0	0	0	0	0	0	0	0	0	0
866830	GFGVFTTNIWLKLKE	NS1	934	948	HLA-DPB1*04:01	0%	Dengue virus 2	ELISPOT	IFNg release	Positive	0	0	0	0	0	0	0	0	0	0	0	0	0	0	0	0	0	0	0	0	0	0	0	0	0	0	0	0	0	0	0	0	0	0	0	0	1	0	0	1	0
150245	GMGVTYLALLAAYKV	NS2a	1196	1210	HLA-DRB1*01:01	0%	Dengue virus 2	ELISPOT	IFNg release	Positive	0	0	0	0	0	0	0	0	0	0	0	0	0	0	0	0	0	0	0	0	0	0	0	0	0	0	0	0	0	0	0	0	0	0	0	0	0	0	0	0	0
195207	CTILAVVSVSPLLLT	NS2a	1144	1158	HLA-DRB1*07:01	0%	Dengue virus 2	ELISPOT	IFNg release	Positive	0	0	0	0	0	0	0	0	0	0	0	0	0	0	0	0	0	0	0	0	0	0	0	0	0	0	0	0	0	0	0	0	0	0	0	0	1	0	0	1	0
195269	ERYQLAVTITAILCV	NS2a	1268	1282	HLA-DRB1*07:01	0%	Dengue virus 2	ELISPOT	IFNg release	Positive	0	0	0	0	0	0	0	0	0	0	0	0	0	0	0	0	0	0	0	0	0	0	0	0	0	0	0	0	0	0	0	0	0	0	0	0	0	0	0	0	0
195523	IPLVLIIKGLNPTAI	NS2a	1319	1333	HLA-DRB1*08:02	0%	Dengue virus 2	ELISPOT	IFNg release	Positive	0	0	0	0	0	0	0	0	0	0	0	0	0	0	0	0	0	0	0	0	0	0	0	0	0	0	0	0	0	0	0	0	0	0	0	0	0	0	0	0	0
195615	LAVVSVSPLLLTSSQ	NS2a	1186	1200	HLA-DRB1*07:01	0%	Dengue virus 2	ELISPOT	IFNg release	Positive	0	0	0	0	0	0	0	0	0	0	0	0	0	0	0	0	0	0	0	0	0	0	0	0	0	0	0	0	0	0	0	0	0	0	0	0	1	0	0	1	0
195712	LSLTFIRSTTPLIMA	NS2a	1275	1289	HLA-DRB1*07:01	0%	Dengue virus 2	ELISPOT	IFNg release	Positive	0	0	0	0	0	0	0	0	0	0	0	0	0	0	0	0	0	0	0	0	0	0	0	0	0	0	0	0	0	0	0	0	0	0	0	0	0	0	0	0	0
195752	MEKYQLAVTIMAILC	NS2a	1154	1168	HLA-DRB1*04:07	0%	Dengue virus 2	ELISPOT	IFNg release	Positive	0	0	0	0	0	0	0	0	0	0	0	0	0	0	0	0	0	0	0	0	0	0	0	0	0	0	0	0	0	0	0	0	0	0	0	0	1	1	0	1	0
195763	MMVLKIVRKMERYQL	NS2a	1258	1272	HLA-DRB1*03:01	0%	Dengue virus 2	ELISPOT	IFNg release	Positive	0	0	0	0	0	0	0	0	0	0	0	0	0	0	0	0	0	0	0	0	0	0	0	0	0	0	0	0	0	0	0	0	0	0	0	0	0	0	0	0	0
195798	NMEKYQLAVTIMAIL	NS2a	1266	1280	HLA-DRB1*04:01	0%	Dengue virus 2	ELISPOT	IFNg release	Positive	0	0	0	0	0	0	0	0	0	0	0	0	0	0	0	0	0	0	0	0	0	0	0	0	0	0	0	0	0	0	0	0	0	0	0	0	1	0	0	1	0
196218	WKVSCTILAVVSVSP	NS2a	1292	1306	HLA-DRB1*04:01	0%	Dengue virus 2	ELISPOT	IFNg release	Positive	0	0	0	0	0	0	0	0	0	0	0	0	0	0	0	0	0	0	0	0	0	0	0	0	0	0	0	0	0	0	0	0	0	0	0	0	1	1	0	1	0
539077	ALGIMVLKMVRSMEK	NS2a	1255	1269	HLA-DRB1*12:02	0%	Dengue virus 2	ELISPOT	IFNg release	Positive	0	0	0	0	0	0	0	0	0	0	0	0	0	0	0	0	0	0	0	0	0	0	0	0	0	0	0	0	0	0	0	0	0	0	0	0	0	0	0	0	0
539082	ALTIKGLNPTAILLT	NS2a	1322	1336	HLA-DRB1*09:01	0%	Dengue virus 2	ELISPOT	IFNg release	Positive	0	0	0	0	0	0	0	0	0	0	0	0	0	0	0	0	0	0	0	0	0	0	0	0	0	0	0	0	0	0	0	0	0	0	0	0	0	0	0	0	0
539110	AWALGMMVLKIVRNM	NS2a	1253	1267	HLA-DRB1*13:01	0%	Dengue virus 2	ELISPOT	IFNg release	Positive	0	0	0	0	0	0	0	0	0	0	0	0	0	0	0	0	0	0	0	0	0	0	0	0	0	0	0	0	0	0	0	0	0	0	0	0	0	0	0	0	0
539392	IALGIMVLKMVRSME	NS2a	1254	1268	HLA-DRB1*13:01	0%	Dengue virus 2	ELISPOT	IFNg release	Positive	0	0	0	0	0	0	0	0	0	0	0	0	0	0	0	0	0	0	0	0	0	0	0	0	0	0	0	0	0	0	0	0	0	0	0	0	0	0	0	0	0
539556	LGIMILKLVRKMEKY	NS2a	1256	1270	HLA-DRB1*12:02	0%	Dengue virus 2	ELISPOT	IFNg release	Positive	0	0	0	0	0	0	0	0	0	0	0	0	0	0	0	0	0	0	0	0	0	0	0	0	0	0	0	0	0	0	0	0	0	0	0	0	0	0	0	0	0
539557	LGIMVLKIVRSMEKY	NS2a	1256	1270	HLA-DRB1*12:02	0%	Dengue virus 2	ELISPOT	IFNg release	Positive	0	0	0	0	0	0	0	0	0	0	0	0	0	0	0	0	0	0	0	0	0	0	0	0	0	0	0	0	0	0	0	0	0	0	0	0	0	0	0	0	0
539561	LGMMILKIVRNMEKY	NS2a	1256	1270	HLA-DRB1*13:01	0%	Dengue virus 2	ELISPOT	IFNg release	Positive	0	0	0	0	0	0	0	0	0	0	0	0	0	0	0	0	0	0	0	0	0	0	0	0	0	0	0	0	0	0	0	0	0	0	0	0	0	0	0	0	0
539699	MVLKMVRNMEKYQLA	NS2a	1259	1273	HLA-DRB1*13:01	0%	Dengue virus 2	ELISPOT	IFNg release	Positive	0	0	0	0	0	0	0	0	0	0	0	0	0	0	0	0	0	0	0	0	0	0	0	0	0	0	0	0	0	0	0	0	0	0	0	0	1	0	0	1	0
740700	VVSVSPLLLTSSQQK	NS2a	1188	1202	HLA-DRB1*01:02	0%	Dengue virus 2	ELISPOT	IFNg release	Positive	0	0	0	0	0	0	0	0	0	0	0	0	0	0	0	0	0	0	0	0	0	0	0	0	0	0	0	0	0	0	0	0	0	0	0	0	1	0	0	1	0
867216	ILCVPNAVILQNAWK	NS2a	1279	1293	HLA-DQA1*01:02/DQB1*06:02	0%	Dengue virus 2	ELISPOT	IFNg release	Positive	0	0	0	0	0	0	0	0	0	0	0	0	0	0	0	0	0	0	0	0	0	0	0	0	0	0	0	0	0	0	0	0	0	0	0	0	1	1	0	1	0
867571	LCVPNAVILQNAWKV	NS2a	1280	1294	HLA-DRB4*01:01	0%	Dengue virus 2	ELISPOT	IFNg release	Positive	0	0	0	0	0	0	0	0	0	0	0	0	0	0	0	0	0	0	0	0	0	0	0	0	0	0	0	0	0	0	0	0	0	0	0	0	1	1	0	1	0
195723	LTILIRTGLLVISGL	NS2b	1440	1454	HLA-DRB1*15:06	0%	Dengue virus 2	ELISPOT	IFNg release	Positive	0	0	0	0	0	0	0	0	0	0	0	0	0	0	0	0	0	0	0	0	0	0	0	0	0	0	0	0	0	0	0	0	0	0	0	0	1	1	0	1	0
24916	HTGREIVDLMCHATE	NS3	251	265	HLA-DPw2	0%	Dengue virus 2	ICS	IFNg release	Positive	0	0	0	0	0	0	0	0	0	0	0	0	0	0	0	0	0	0	0	0	0	0	0	0	0	0	0	0	0	0	0	0	0	0	0	0	0	0	0	0	0
540056	VLMHKGKRIEPSWAD	NS3	1518	1532	HLA-DRB1*13:01	0%	Dengue virus 2	ELISPOT	IFNg release	Positive	0	0	0	0	0	0	0	0	0	0	0	0	0	0	0	0	0	0	0	0	0	0	0	0	0	0	0	0	0	0	0	0	0	0	0	0	1	1	0	1	0
54390	RKLTIMDLHPGAGKT	NS3	618	632	HLA class II	0%	Dengue virus 2	ICS	IFNg release	Positive	0	0	0	0	0	0	0	0	0	0	0	0	0	0	0	0	0	0	0	0	0	0	0	0	0	0	0	0	0	0	0	0	0	0	0	0	0	1	0	0	0
739148	IDKKGKVVGLYGNGV	NS3	1615	1629	HLA-DRB1*01:02	0%	Dengue virus 2	ELISPOT	IFNg release	Positive	0	0	0	0	0	0	0	0	0	0	0	0	0	0	0	0	0	0	0	0	0	0	0	0	0	0	0	0	0	0	0	0	0	0	0	0	1	1	0	1	0
868724	RTNDWDFVVTTDISE	NS3	1873	1887	HLA-DQA1*03:01/DQB1*03:02	0%	Dengue virus 2	ELISPOT	IFNg release	Positive	0	0	0	0	0	0	0	0	0	0	0	0	0	0	0	0	0	0	0	0	0	0	0	0	0	0	0	0	0	0	0	0	0	0	0	0	1	1	0	1	0
8911	DKKGKVVGLYGNGVV	NS3	141	155	HLA-DRB1*15:01	0%	Dengue virus 2	ICS	IFNg release	Positive	0	0	0	0	0	0	0	0	0	0	0	0	0	0	0	0	0	0	0	0	0	0	0	0	0	0	0	0	0	0	0	0	0	0	0	0	1	1	0	1	0
195356	GGIFLFLMSGRGIGK	NS4a	2159	2173	HLA-DRB1*11:01	0%	Dengue virus 2	ELISPOT	IFNg release	Positive	0	0	0	0	0	0	0	0	0	0	0	0	0	0	0	0	0	0	0	0	0	0	0	0	0	0	0	0	0	0	0	0	0	0	0	0	1	1	0	1	0
195414	GRAYNHALSELPETL	NS4a	2131	2145	HLA-DRB1*04:01	0%	Dengue virus 2	ELISPOT	IFNg release	Positive	0	0	0	0	0	0	0	0	0	0	0	0	0	0	0	0	0	0	0	0	0	0	0	0	0	0	0	0	0	0	0	0	0	0	0	0	1	1	0	1	0
195810	NQLTYVVIAILTLVA	NS4a	2225	2239	HLA-DRB1*07:01	0%	Dengue virus 2	ELISPOT	IFNg release	Positive	0	0	0	0	0	0	0	0	0	0	0	0	0	0	0	0	0	0	0	0	0	0	0	0	0	0	0	0	0	0	0	0	0	0	0	0	0	0	0	0	0
195118	AGLLFSIMRNTTSAR	NS4b	2476	2490	HLA-DRB1*04:01	0%	Dengue virus 2	ELISPOT	IFNg release	Positive	0	0	0	0	0	0	0	0	0	0	0	0	0	0	0	0	0	0	0	0	0	0	0	0	0	0	0	0	0	0	0	0	0	0	0	0	0	0	0	0	0
195387	GLLFFIMKNTTNTRR	NS4b	2477	2491	HLA-DRB1*04:01	0%	Dengue virus 2	ELISPOT	IFNg release	Positive	0	0	0	0	0	0	0	0	0	0	0	0	0	0	0	0	0	0	0	0	0	0	0	0	0	0	0	0	0	0	0	0	0	0	0	0	0	0	0	0	0
195393	GLLFSIMKNTANTRR	NS4b	2463	2477	HLA-DRB1*04:01	0%	Dengue virus 2	ELISPOT	IFNg release	Positive	0	0	0	0	0	0	0	0	0	0	0	0	0	0	0	0	0	0	0	0	0	0	0	0	0	0	0	0	0	0	0	0	0	0	0	0	0	0	0	0	0
195394	GLLFSIMKNTTNARR	NS4b	2477	2491	HLA-DRB1*04:01	0%	Dengue virus 2	ELISPOT	IFNg release	Positive	0	0	0	0	0	0	0	0	0	0	0	0	0	0	0	0	0	0	0	0	0	0	0	0	0	0	0	0	0	0	0	0	0	0	0	0	0	0	0	0	0
195395	GLLFSIMKNTTNSRR	NS4b	2477	2491	HLA-DRB1*04:01	0%	Dengue virus 2	ELISPOT	IFNg release	Positive	0	0	0	0	0	0	0	0	0	0	0	0	0	0	0	0	0	0	0	0	0	0	0	0	0	0	0	0	0	0	0	0	0	0	0	0	0	0	0	0	0
195396	GLLFSIMKNTTSARR	NS4b	2477	2491	HLA-DRB1*04:01	0%	Dengue virus 2	ELISPOT	IFNg release	Positive	0	0	0	0	0	0	0	0	0	0	0	0	0	0	0	0	0	0	0	0	0	0	0	0	0	0	0	0	0	0	0	0	0	0	0	0	0	0	0	0	0
195397	GLLFSIMKNTTSTRR	NS4b	2477	2491	HLA-DRB1*04:01	0%	Dengue virus 2	ELISPOT	IFNg release	Positive	0	0	0	0	0	0	0	0	0	0	0	0	0	0	0	0	0	0	0	0	0	0	0	0	0	0	0	0	0	0	0	0	0	0	0	0	0	0	0	0	0
195659	LLFSIMRNTTTARRG	NS4b	2478	2492	HLA-DRB1*04:01	0%	Dengue virus 2	ELISPOT	IFNg release	Positive	0	0	0	0	0	0	0	0	0	0	0	0	0	0	0	0	0	0	0	0	0	0	0	0	0	0	0	0	0	0	0	0	0	0	0	0	0	0	0	0	0
195718	LTAALFLLVAHYAII	NS4b	2350	2364	HLA-DRB1*07:01	0%	Dengue virus 2	ELISPOT	IFNg release	Positive	0	0	0	0	0	0	0	0	0	0	0	0	0	0	0	0	0	0	0	0	0	0	0	0	0	0	0	0	0	0	0	0	0	0	0	0	0	1	0	1	0
195827	PESNILDIDLRPASA	NS4b	2266	2280	HLA-DRB1*03:01	0%	Dengue virus 2	ELISPOT	IFNg release	Positive	0	0	0	0	0	0	0	0	0	0	0	0	0	0	0	0	0	0	0	0	0	0	0	0	0	0	0	0	0	0	0	0	0	0	0	0	1	1	0	1	0
867335	ITLTAALLLLIAHYA	NS4b	2348	2362	HLA-DPB1*04:02	0%	Dengue virus 2	ELISPOT	IFNg release	Positive	0	0	0	0	0	0	0	0	0	0	0	0	0	0	0	0	0	0	0	0	0	0	0	0	0	0	0	0	0	0	0	0	0	0	0	0	0	0	0	0	0
867860	LLVLCVTQVLMMRTT	NS4b	2416	2430	HLA-DPB1*04:02	0%	Dengue virus 2	ELISPOT	IFNg release	Positive	0	0	0	0	0	0	0	0	0	0	0	0	0	0	0	0	0	0	0	0	0	0	0	0	0	0	0	0	0	0	0	0	0	0	0	0	1	1	0	1	0
869090	TAIANQATVLMGLGR	NS4b	2309	2323	HLA-DQA1*01:02/DQB1*06:02	0%	Dengue virus 2	ELISPOT	IFNg release	Positive	0	0	0	0	0	0	0	0	0	0	0	0	0	0	0	0	0	0	0	0	0	0	0	0	0	0	0	0	0	0	0	0	0	0	0	0	0	0	0	0	0
1066545	ACLGKSYAQMWSLMY	NS5	3243	3257	HLA-DRB1*04:01	0%	Dengue virus 2	ELISPOT	IFNg release	Positive-Low	0	0	0	0	0	0	0	0	0	0	0	0	0	0	0	0	0	0	0	0	0	0	0	0	0	0	0	0	0	0	0	0	0	0	0	0	1	1	0	1	0
180579	KSYAQMWSLMYFHRR	NS5	3247	3261	HLA-DRB5*01:01	0%	Dengue virus 2	ELISPOT	IFNg release	Positive	0	0	0	0	0	0	0	0	0	0	0	0	0	0	0	0	0	0	0	0	0	0	0	0	0	0	0	0	0	0	0	0	0	0	0	0	1	1	0	1	0
195241	EGEGVFKSIQHLTVT	NS5	3101	3115	HLA-DRB1*07:01	0%	Dengue virus 2	ELISPOT	IFNg release	Positive	0	0	0	0	0	0	0	0	0	0	0	0	0	0	0	0	0	0	0	0	0	0	0	0	0	0	0	0	0	0	0	0	0	0	0	0	0	1	0	0	0
195250	ELIMKDGRKLVVPCR	NS5	3206	3220	HLA-DRB1*03:01	0%	Dengue virus 2	ELISPOT	IFNg release	Positive	0	0	0	0	0	0	0	0	0	0	0	0	0	0	0	0	0	0	0	0	0	0	0	0	0	0	0	0	0	0	0	0	0	0	0	0	0	0	0	0	0
195251	ELIMKDGRVLVVPCR	NS5	3206	3220	HLA-DRB1*03:01	0%	Dengue virus 2	ELISPOT	IFNg release	Positive	0	0	0	0	0	0	0	0	0	0	0	0	0	0	0	0	0	0	0	0	0	0	0	0	0	0	0	0	0	0	0	0	0	0	0	0	1	1	0	1	0
195380	GKSYAQMWSLMYFHR	NS5	3232	3246	HLA-DRB1*04:01	0%	Dengue virus 2	ELISPOT	IFNg release	Positive	0	0	0	0	0	0	0	0	0	0	0	0	0	0	0	0	0	0	0	0	0	0	0	0	0	0	0	0	0	0	0	0	0	0	0	0	1	1	0	1	0
195384	GLFKSIQHLTSQEEI	NS5	3118	3132	HLA-DRB1*11:01	0%	Dengue virus 2	ELISPOT	IFNg release	Positive	0	0	0	0	0	0	0	0	0	0	0	0	0	0	0	0	0	0	0	0	0	0	0	0	0	0	0	0	0	0	0	0	0	0	0	0	0	0	0	0	0
195445	HFHELIMKDGRILVV	NS5	3203	3217	HLA-DRB1*03:01	0%	Dengue virus 2	ELISPOT	IFNg release	Positive	0	0	0	0	0	0	0	0	0	0	0	0	0	0	0	0	0	0	0	0	0	0	0	0	0	0	0	0	0	0	0	0	0	0	0	0	0	0	0	0	0
195513	IMKDGRKLVVPCRNQ	NS5	3207	3221	HLA-DRB1*03:01	0%	Dengue virus 2	ELISPOT	IFNg release	Positive	0	0	0	0	0	0	0	0	0	0	0	0	0	0	0	0	0	0	0	0	0	0	0	0	0	0	0	0	0	0	0	0	0	0	0	0	0	0	0	0	0
195642	LHKLGYILRDVSKKE	NS5	3003	3017	HLA-DRB1*08:02	0%	Dengue virus 2	ELISPOT	IFNg release	Positive	0	0	0	0	0	0	0	0	0	0	0	0	0	0	0	0	0	0	0	0	0	0	0	0	0	0	0	0	0	0	0	0	0	0	0	0	1	1	0	1	0
195801	NMISRMLINRFTMRH	NS5	6	20	HLA-DRB1*08:02	0%	Dengue virus 2	ELISPOT	IFNg release	Positive	0	0	0	0	0	0	0	0	0	0	0	0	0	0	0	0	0	0	0	0	0	0	0	0	0	0	0	0	0	0	0	0	0	0	0	0	1	1	0	0	0
195948	SGNIVSSVNMISRML	NS5	2716	2730	HLA-DRB1*04:01	0%	Dengue virus 2	ELISPOT	IFNg release	Positive	0	0	0	0	0	0	0	0	0	0	0	0	0	0	0	0	0	0	0	0	0	0	0	0	0	0	0	0	0	0	0	0	0	0	0	0	1	1	0	1	0
196161	VNMISRMLINRFTMR	NS5	2723	2737	HLA-DRB1*10:01	0%	Dengue virus 2	ELISPOT	IFNg release	Positive	0	0	0	0	0	0	0	0	0	0	0	0	0	0	0	0	0	0	0	0	0	0	0	0	0	0	0	0	0	0	0	0	0	0	0	0	1	1	0	1	0
196223	WSLMYFHRRDLRLAA	NS5	3253	3267	HLA-DRB1*03:01	0%	Dengue virus 2	ELISPOT	IFNg release	Positive	0	0	0	0	0	0	0	0	0	0	0	0	0	0	0	0	0	0	0	0	0	0	0	0	0	0	0	0	0	0	0	0	0	0	0	0	1	1	0	1	0
32939	KPWDIIPMV	NS5	2821	2829	HLA class II	0%	Dengue virus 2	ELISA	IL-2 release	Positive	0	0	0	0	0	0	0	0	0	0	0	0	0	0	0	0	0	0	0	0	0	0	0	0	0	0	0	0	0	0	0	0	0	0	0	0	0	0	0	0	0
32941	KPWDVLPMV	NS5	2821	2829	HLA class II	0%	Dengue virus 2	ELISA	IL-2 release	Positive	0	0	0	0	0	0	0	0	0	0	0	0	0	0	0	0	0	0	0	0	0	0	0	0	0	0	0	0	0	0	0	0	0	0	0	0	0	0	0	0	0
32942	KPWDVLPTV	NS5	2821	2829	HLA class II	0%	Dengue virus 2	ELISA	IL-2 release	Positive	0	0	0	0	0	0	0	0	0	0	0	0	0	0	0	0	0	0	0	0	0	0	0	0	0	0	0	0	0	0	0	0	0	0	0	0	0	0	0	0	0
539144	CVVKPLDDRFASALTAL	NS5	3156	3172	HLA class II	0%	Dengue virus 2	ICS	IFNg release	Positive	0	0	0	0	0	0	0	0	0	0	0	0	0	0	0	0	0	0	0	0	0	0	0	0	0	0	0	0	0	0	0	0	0	0	0	0	1	1	0	1	0
539436	ISGDDCVVKPLDDRFA	NS5	3151	3166	HLA class II	0%	Dengue virus 2	ICS	IFNg release	Positive	0	0	0	0	0	0	0	0	0	0	0	0	0	0	0	0	0	0	0	0	0	0	0	0	0	0	0	0	0	0	0	0	0	0	0	0	1	1	0	1	0
539438	ISRMLINRFTMRHKK	NS5	8	22	HLA-DRB1*14:02	0%	Dengue virus 2	ELISPOT	IFNg release	Positive	0	0	0	0	0	0	0	0	0	0	0	0	0	0	0	0	0	0	0	0	0	0	0	0	0	0	0	0	0	0	0	0	0	0	0	0	1	1	0	0	0
539560	LGKNEFQIYKKSGIQ	NS5	2511	2525	HLA-DRB1*15:02	0%	Dengue virus 2	ELISPOT	IFNg release	Positive	0	0	0	0	0	0	0	0	0	0	0	0	0	0	0	0	0	0	0	0	0	0	0	0	0	0	0	0	0	0	0	0	0	0	0	0	0	0	0	0	0
539669	MISRMLINRFTMRHK	NS5	7	21	HLA-DRB1*14:06	0%	Dengue virus 2	ELISPOT	IFNg release	Positive	0	0	0	0	0	0	0	0	0	0	0	0	0	0	0	0	0	0	0	0	0	0	0	0	0	0	0	0	0	0	0	0	0	0	0	0	1	1	0	0	0
866172	ASGNIVSSVNMISRM	NS5	2715	2729	HLA-DRB3*02:02	0%	Dengue virus 2	ELISPOT	IFNg release	Positive	0	0	0	0	0	0	0	0	0	0	0	0	0	0	0	0	0	0	0	0	0	0	0	0	0	0	0	0	0	0	0	0	0	0	0	0	1	1	0	1	0
867514	KTPVESWEEIPYLGK	NS5	3318	3332	HLA-DQA1*01:01/DQB1*05:01	0%	Dengue virus 2	ELISPOT	IFNg release	Positive	0	0	0	0	0	0	0	0	0	0	0	0	0	0	0	0	0	0	0	0	0	0	0	0	0	0	0	0	0	0	0	0	0	0	0	0	1	1	0	1	0
869082	SYAQMWSLMYFHRRD	NS5	3248	3262	HLA-DPB1*04:01	0%	Dengue virus 2	ELISPOT	IFNg release	Positive	0	0	0	0	0	0	0	0	0	0	0	0	0	0	0	0	0	0	0	0	0	0	0	0	0	0	0	0	0	0	0	0	0	0	0	0	1	1	0	1	0
869148	TFVESWEDIPYLGKR	NS5	3319	3333	HLA-DQA1*01:01/DQB1*05:01	0%	Dengue virus 2	ELISPOT	IFNg release	Positive	0	0	0	0	0	0	0	0	0	0	0	0	0	0	0	0	0	0	0	0	0	0	0	0	0	0	0	0	0	0	0	0	0	0	0	0	0	0	0	0	0
869426	VESWEEIPYLGKRED	NS5	3321	3335	HLA-DQA1*01:01/DQB1*05:01	0%	Dengue virus 2	ELISPOT	IFNg release	Positive	0	0	0	0	0	0	0	0	0	0	0	0	0	0	0	0	0	0	0	0	0	0	0	0	0	0	0	0	0	0	0	0	0	0	0	0	1	1	0	1	0
150369	LKLFMALVAFLRFLT	C	44	58	HLA-DRB1*01:01	100%	Dengue virus 2	ELISPOT	IFNg release	Positive	1	1	1	1	1	1	1	1	1	1	1	1	1	1	1	1	1	1	1	1	1	1	1	1	1	1	1	1	1	1	1	1	1	1	1	0	1	1	0	1	0
180768	TIKKSKAINVLRGFR	C	71	85	HLA class II	100%	Dengue virus 2	ELISPOT	IFNg release	Positive	1	1	1	1	1	1	1	1	1	1	1	1	1	1	1	1	1	1	1	1	1	1	1	1	1	1	1	1	1	1	1	1	1	1	1	0	1	1	0	1	0
195889	RGPLKLFMALVAFLR	C	41	55	HLA-DRB1*04:01	100%	Dengue virus 2	ELISPOT	IFNg release	Positive	1	1	1	1	1	1	1	1	1	1	1	1	1	1	1	1	1	1	1	1	1	1	1	1	1	1	1	1	1	1	1	1	1	1	1	0	1	1	0	1	0
196064	TPFNMLKRERNRVST	C	11	25	HLA-DRB1*08:02	100%	Dengue virus 2	ELISPOT	IFNg release	Positive	1	1	1	1	1	1	1	1	1	1	1	1	1	1	1	1	1	1	1	1	1	1	1	1	1	1	1	1	1	1	1	1	1	1	1	0	1	1	0	1	0
196213	WGTIKKSKAINVLRG	C	69	83	HLA-DRB1*07:01	100%	Dengue virus 2	ELISPOT	IFNg release	Positive	1	1	1	1	1	1	1	1	1	1	1	1	1	1	1	1	1	1	1	1	1	1	1	1	1	1	1	1	1	1	1	1	1	1	1	0	1	1	0	1	0
539214	EIGRMLNILNRRRRT	C	87	101	HLA-DRB1*12:02	100%	Dengue virus 2	ELISPOT	IFNg release	Positive	1	1	1	1	1	1	1	1	1	1	1	1	1	1	1	1	1	1	1	1	1	1	1	1	1	1	1	1	1	1	1	1	1	1	1	0	0	1	0	0	0
539887	RWGTIKKSKAINVLR	C	68	82	HLA-DRB1*08:03	100%	Dengue virus 2	ELISPOT	IFNg release	Positive	1	1	1	1	1	1	1	1	1	1	1	1	1	1	1	1	1	1	1	1	1	1	1	1	1	1	1	1	1	1	1	1	1	1	1	0	1	1	0	1	0
739879	PFNMLKRERNRVSTV	C	12	26	HLA-DRB1*14:02	100%	Dengue virus 2	ELISPOT	IFNg release	Positive	1	1	1	1	1	1	1	1	1	1	1	1	1	1	1	1	1	1	1	1	1	1	1	1	1	1	1	1	1	1	1	1	1	1	1	0	1	1	0	1	0
740399	STPFNMLKRERNRVS	C	10	24	HLA-DRB1*14:02	100%	Dengue virus 2	ELISPOT	IFNg release	Positive	1	1	1	1	1	1	1	1	1	1	1	1	1	1	1	1	1	1	1	1	1	1	1	1	1	1	1	1	1	1	1	1	1	1	1	0	0	0	0	0	0
857784	LKRWGTIKKSKAINV	C	66	80	HLA-DRB1*07:01	100%	Dengue virus 2	ELISPOT	IFNg release	Positive-Low	1	1	1	1	1	1	1	1	1	1	1	1	1	1	1	1	1	1	1	1	1	1	1	1	1	1	1	1	1	1	1	1	1	1	1	0	1	1	0	1	0
195448	HFQRALIFILLTAVA	PreM	262	276	HLA-DRB1*07:01	100%	Dengue virus 2	ELISPOT	IFNg release	Positive	1	1	1	1	1	1	1	1	1	1	1	1	1	1	1	1	1	1	1	1	1	1	1	1	1	1	1	1	1	1	1	1	1	1	1	1	1	1	1	0	1
195862	QRALIFILLTAVAPS	PreM	264	278	HLA-DRB1*15:01	100%	Dengue virus 2	ELISPOT	IFNg release	Positive	1	1	1	1	1	1	1	1	1	1	1	1	1	1	1	1	1	1	1	1	1	1	1	1	1	1	1	1	1	1	1	1	1	1	1	1	1	1	1	0	1
196045	THFQRALIFILLTAV	PreM	261	275	HLA-DRB1*07:01	100%	Dengue virus 2	ELISPOT	IFNg release	Positive	1	1	1	1	1	1	1	1	1	1	1	1	1	1	1	1	1	1	1	1	1	1	1	1	1	1	1	1	1	1	1	1	1	1	1	1	1	1	1	1	1
539943	SVALVPHVGMGLETR	PreM	206	220	HLA-DRB1*15:02	100%	Dengue virus 2	ELISPOT	IFNg release	Positive	1	1	1	1	1	1	1	1	1	1	1	1	1	1	1	1	1	1	1	1	1	1	1	1	1	1	1	1	1	1	1	1	1	1	1	1	1	1	1	1	1
739425	LAYTIGTTHFQRALI	PreM	254	268	HLA-DRB1*04:07	100%	Dengue virus 2	ELISPOT	IFNg release	Positive	1	1	1	1	1	1	1	1	1	1	1	1	1	1	1	1	1	1	1	1	1	1	1	1	1	1	1	1	1	1	1	1	1	1	1	1	1	1	1	1	1
180564	KKQDVVVLGSQEGAM	E	526	540	HLA class II	100%	Dengue virus 2	ELISPOT	IFNg release	Positive	1	1	1	1	1	1	1	1	1	1	1	1	1	1	1	1	1	1	1	1	1	1	1	1	1	1	1	1	1	1	1	1	1	1	1	1	1	1	1	1	1
180820	WLVHRQWFLDLPLPW	E	196	210	HLA class II	100%	Dengue virus 2	ELISPOT	IFNg release	Positive	1	1	1	1	1	1	1	1	1	1	1	1	1	1	1	1	1	1	1	1	1	1	1	1	1	1	1	1	1	1	1	1	1	1	1	1	1	1	1	1	1
195351	GATEIQMSSGNLLFT	E	266	280	HLA-DRB1*03:01	100%	Dengue virus 2	ELISPOT	IFNg release	Positive	1	1	1	1	1	1	1	1	1	1	1	1	1	1	1	1	1	1	1	1	1	1	1	1	1	1	1	1	1	1	1	1	1	1	1	1	1	1	1	1	1
195366	GHLKCRLRMDKLQLK	E	281	295	HLA-DRB1*03:01	100%	Dengue virus 2	ELISPOT	IFNg release	Positive	1	1	1	1	1	1	1	1	1	1	1	1	1	1	1	1	1	1	1	1	1	1	1	1	1	1	1	1	1	1	1	1	1	1	1	1	1	1	1	1	1
195407	GMNSRSTSLSVSLVL	E	467	481	HLA-DRB1*07:01	100%	Dengue virus 2	ELISPOT	IFNg release	Positive	1	1	1	1	1	1	1	1	1	1	1	1	1	1	1	1	1	1	1	1	1	1	1	1	1	1	1	1	1	1	1	1	1	1	1	1	1	1	1	0	0
195716	LSVSLVLVGVVTLYL	E	755	769	HLA-DRB1*15:01	100%	Dengue virus 2	ELISPOT	IFNg release	Positive	1	1	1	1	1	1	1	1	1	1	1	1	1	1	1	1	1	1	1	1	1	1	1	1	1	1	1	1	1	1	1	1	1	1	1	1	0	1	1	0	0
196022	SWTMKILIGVIITWI	E	452	466	HLA-DRB1*08:02	100%	Dengue virus 2	ELISPOT	IFNg release	Positive	1	1	1	1	1	1	1	1	1	1	1	1	1	1	1	1	1	1	1	1	1	1	1	1	1	1	1	1	1	1	1	1	1	1	1	0	1	0	1	1	0
196077	TSLSVSLVLVGVVTL	E	753	767	HLA-DRB1*07:01	100%	Dengue virus 2	ELISPOT	IFNg release	Positive	1	1	1	1	1	1	1	1	1	1	1	1	1	1	1	1	1	1	1	1	1	1	1	1	1	1	1	1	1	1	1	1	1	1	1	1	0	1	1	0	0
539236	ENLEYTIVITPHSGE	E	133	147	HLA-DRB1*04:07	100%	Dengue virus 2	ELISPOT	IFNg release	Positive	1	1	1	1	1	1	1	1	1	1	1	1	1	1	1	1	1	1	1	1	1	1	1	1	1	1	1	1	1	1	1	1	1	1	1	1	0	1	0	1	1
539377	HAKKQDVVVLGSQEGAMH	E	244	261	HLA class II	100%	Dengue virus 2	ICS	IFNg release	Positive	1	1	1	1	1	1	1	1	1	1	1	1	1	1	1	1	1	1	1	1	1	1	1	1	1	1	1	1	1	1	1	1	1	1	1	1	1	1	1	1	1
539733	NLEYTIVITPHSGEE	E	134	148	HLA-DRB1*04:07	100%	Dengue virus 2	ELISPOT	IFNg release	Positive	1	1	1	1	1	1	1	1	1	1	1	1	1	1	1	1	1	1	1	1	1	1	1	1	1	1	1	1	1	1	1	1	1	1	1	1	0	1	0	1	1
740315	SLSVSLVLVGVVTLY	E	754	768	HLA-DRB1*01:02	100%	Dengue virus 2	ELISPOT	IFNg release	Positive	1	1	1	1	1	1	1	1	1	1	1	1	1	1	1	1	1	1	1	1	1	1	1	1	1	1	1	1	1	1	1	1	1	1	1	1	0	1	1	0	0
741744	MRCIGISNRDFVEGVSGGSW	E	281	300	HLA class II	100%	Dengue virus 2	ELISPOT	IFNg release	Positive	1	1	1	1	1	1	1	1	1	1	1	1	1	1	1	1	1	1	1	1	1	1	1	1	1	1	1	1	1	1	1	1	1	1	1	1	1	1	1	0	1
866193	ATEIQMSSGNLLFTG	E	257	271	HLA-DRB3*02:02	100%	Dengue virus 2	ELISPOT	IFNg release	Positive	1	1	1	1	1	1	1	1	1	1	1	1	1	1	1	1	1	1	1	1	1	1	1	1	1	1	1	1	1	1	1	1	1	1	1	1	1	1	1	1	1
868713	RTGLDFNEMVLLQME	E	178	192	HLA-DQA1*01:02/DQB1*06:02	100%	Dengue virus 2	ELISPOT	IFNg release	Positive	1	1	1	1	1	1	1	1	1	1	1	1	1	1	1	1	1	1	1	1	1	1	1	1	1	1	1	1	1	1	1	1	1	1	1	1	1	1	1	1	1
869152	TGLDFNEMVLLQMED	E	189	203	HLA-DQA1*03:01/DQB1*03:02	100%	Dengue virus 2	ELISPOT	IFNg release	Positive	1	1	1	1	1	1	1	1	1	1	1	1	1	1	1	1	1	1	1	1	1	1	1	1	1	1	1	1	1	1	1	1	1	1	1	0	0	0	0	0	0
167759	IMTGDIKGIMQA	NS1	88	99	HLA-DQ6	100%	Dengue virus 2	ELISPOT	IFNg release	Positive	1	1	1	1	1	1	1	1	1	1	1	1	1	1	1	1	1	1	1	1	1	1	1	1	1	1	1	1	1	1	1	1	1	1	1	0	1	1	0	1	0
180396	DGCWYGMEIRPLKEKEENLVNS	NS1	1102	1123	HLA class II	100%	Dengue virus 2	ICS	IFNg release	Positive	1	1	1	1	1	1	1	1	1	1	1	1	1	1	1	1	1	1	1	1	1	1	1	1	1	1	1	1	1	1	1	1	1	1	1	0	1	1	0	1	0
195576	KLTIMTGDIKGIMQA	NS1	85	99	HLA-DRB1*03:01	100%	Dengue virus 2	ELISPOT	IFNg release	Positive	1	1	1	1	1	1	1	1	1	1	1	1	1	1	1	1	1	1	1	1	1	1	1	1	1	1	1	1	1	1	1	1	1	1	1	0	1	1	0	1	0
195880	REKQDVFCDSKLMSA	NS1	947	961	HLA-DRB1*03:01	100%	Dengue virus 2	ELISPOT	IFNg release	Positive	1	1	1	1	1	1	1	1	1	1	1	1	1	1	1	1	1	1	1	1	1	1	1	1	1	1	1	1	1	1	1	1	1	1	1	0	0	1	0	0	0

738586	CGIRSVTRLENLMWK	NS1	55	69	HLA-DRB1*11:04	100%	Dengue virus 2	ELISPOT	IFNg release	Positive	1
738766	EGICGIRSVTRLENL	NS1	52	66	HLA-DRB1*11:04	100%	Dengue virus 2	ELISPOT	IFNg release	Positive	1
739192	IKGIMQAGKRSLRPQ	NS1	93	107	HLA-DRB1*14:06	100%	Dengue virus 2	ELISPOT	IFNg release	Positive	1
739296	KGIMQAGKRSLRPQP	NS1	94	108	HLA-DRB1*14:06	100%	Dengue virus 2	ELISPOT	IFNg release	Positive	1
867040	HADMGYWIESALNDT	NS1	970	984	HLA-DQA1*01:01/DQB1*05:01	100%	Dengue virus 2	ELISPOT	IFNg release	Positive	1
150131	ALTIKGLNPTAIFLT	NS2a	1308	1322	HLA-DRB1*09:01	100%	Dengue virus 2	ELISPOT	IFNg release	Positive	1
195688	LNPTAIFLTTLSRTS	NS2a	1328	1342	HLA-DRB1*07:01	100%	Dengue virus 2	ELISPOT	IFNg release	Positive	1
539231	EMLRTRVGTKHAILL	NS2a	1148	1162	HLA-DRB1*08:03	100%	Dengue virus 2	ELISPOT	IFNg release	Positive	1
539789	PTAIFLTTLSRTSKK	NS2a	1316	1330	HLA-DRB1*04:03	100%	Dengue virus 2	ELISPOT	IFNg release	Positive	1
539893	SCTILAAVSVSPLLL	NS2a	1295	1309	HLA-DRB1*10:01	100%	Dengue virus 2	ELISPOT	IFNg release	Positive	1
539959	TAIFLTTLSRTSKKR	NS2a	1317	1331	HLA-DRB1*14:02	100%	Dengue virus 2	ELISPOT	IFNg release	Positive	1
866978	GMALFLEEMLRTRVG	NS2a	1141	1155	HLA-DQA1*01:01/DQB1*05:01	100%	Dengue virus 2	ELISPOT	IFNg release	Positive	1
868918	SLGVLGMALFLEEML	NS2a	1136	1150	HLA-DQA1*05:01/DQB1*02:01	100%	Dengue virus 2	ELISPOT	IFNg release	Positive	1
150433	MAVGMVSILASSLLK	NS2b	1354	1368	HLA-DRB1*01:01	100%	Dengue virus 2	ELISPOT	IFNg release	Positive	1
167732	DQAEISGSSPILSITISEDG	NS2b	63	82	HLA-DP	100%	Dengue virus 2	ELISPOT	IFNg release	Positive	1
195239	EEQTLTILIRTGLLV	NS2b	91	105	HLA-DRB1*15:01	100%	Dengue virus 2	ELISPOT	IFNg release	Positive	1
195869	QTLTILIRTGLLVIS	NS2b	1438	1452	HLA-DRB1*15:01	100%	Dengue virus 2	ELISPOT	IFNg release	Positive	1
195913	RTGLLVISGVFPVSI	NS2b	1445	1459	HLA-DRB1*08:02	100%	Dengue virus 2	ELISPOT	IFNg release	Positive	1
195992	SSLLKNDIPMTGPLV	NS2b	19	33	HLA-DRB1*03:01	100%	Dengue virus 2	ELISPOT	IFNg release	Positive	1
539574	LIRTGLLVISGVFPV	NS2b	1443	1457	HLA-DRB1*13:01	100%	Dengue virus 2	ELISPOT	IFNg release	Positive	1
866986	GMVSILASSLLKNDI	NS2b	1357	1371	HLA-DPB1*01:01	100%	Dengue virus 2	ELISPOT	IFNg release	Positive	1
1067121	GKVVGLYGNGVVTRS	NS3	1619	1633	HLA-DRB1*15:01	100%	Dengue virus 2	ELISPOT	IFNg release	Positive	1
12372	EHTGREIVDLMCHAT	NS3	250	264	HLA class II	100%	Dengue virus 2	ELISPOT	IFNg release	Positive	1
12606	EIVDLMCHATFTMRL	NS3	255	269	HLA class II	100%	Dengue virus 2	ELISPOT	IFNg release	Positive	1
1392191	GREIVDLMCHATFTM	NS3	1728	1742		100%	Dengue virus 2	biological activity	activation	Positive	1
139758	GKTKRYLPAIVREAI	NS3	1673	1687	HLA-DRB1*09:01	100%	Dengue virus 2	ELISPOT	IFNg release	Positive	1
139762	GLPIRYQTPAIRAEH	NS3	1712	1726	HLA-DRB1*04:03	100%	Dengue virus 2	ELISPOT	IFNg release	Positive	1
180698	PNYNLIIMDEAHFTD	NS3	1751	1765	HLA class II	100%	Dengue virus 2	ELISPOT	IFNg release	Positive	1
180807	VTDFKGKTVWFVPSI	NS3	1826	1840	HLA class II	100%	Dengue virus 2	ELISPOT	IFNg release	Positive-Low	1
195154	ALRGLPIRYQTPAIR	NS3	1709	1723	HLA-DRB1*08:02	100%	Dengue virus 2	ELISPOT	IFNg release	Positive	1
195295	FHTMWHVTRGAVLMH	NS3	1521	1535	HLA-DRB1*07:01	100%	Dengue virus 2	ELISPOT	IFNg release	Positive	1
195420	GTFHTMWHVTRGAVL	NS3	1519	1533	HLA-DRB1*07:01	100%	Dengue virus 2	ELISPOT	IFNg release	Positive	1
195564	KGKVVGLYGNGVVTR	NS3	1618	1632	HLA-DRB1*15:01	100%	Dengue virus 2	ELISPOT	IFNg release	Positive	1
195888	RGLRTLILAPTRVVA	NS3	1689	1703	HLA-DRB1*08:02	100%	Dengue virus 2	ELISPOT	IFNg release	Positive	1
21115	GLRTLILAPTRVVAA	NS3	1690	1704	HLA-DRB1*04:03	100%	Dengue virus 2	ELISPOT	IFNg release	Positive	1
26965	ILAPTRVVAAEMEEA	NS3	1695	1709	HLA-DQA1*03:01/DQB1*03:02	100%	Dengue virus 2	ELISPOT	IFNg release	Positive	1
30396	KEGERKKLKPRWLDA	NS3	584	598	HLA class II	100%	Dengue virus 2	ICS	IFNg release	Positive	1
34231	KVVGLYGNGVVTRSG	NS3	145	159	HLA class II	100%	Dengue virus 2	ELISPOT	IFNg release	Positive	1
36560	LIIMDEAHFTDPASI	NS3	1755	1769	HLA-DQA1*01:01/DQB1*05:01	100%	Dengue virus 2	ELISPOT	IFNg release	Positive	1
39103	LRGLPIRYQTPAIRA	NS3	1696	1710	HLA-DRB1*14:02	100%	Dengue virus 2	ELISPOT	IFNg release	Positive	1
39225	LRTLILAPTRVVAAE	NS3	1691	1705	HLA-DRB1*12:02	100%	Dengue virus 2	ELISPOT	IFNg release	Positive	1
4152	ARGYISTRVEMGEAA	NS3	1757	1771	HLA-DRB1*04:07	100%	Dengue virus 2	ELISPOT	IFNg release	Positive	1
49766	PTRVVAAEMEEALRG	NS3	223	237	HLA-DRB1*15:01	100%	Dengue virus 2	ICS	IFNg release	Positive	1
539025	AAGIFMTATPPGSRD	NS3	1784	1798	HLA-DRB1*04:03	100%	Dengue virus 2	ELISPOT	IFNg release	Positive	1
539041	AEMEEALRGLPIRYQ	NS3	1704	1718	HLA-DRB1*13:01	100%	Dengue virus 2	ELISPOT	IFNg release	Positive	1
539203	EERVILAGPMPVTHS	NS3	1899	1913	HLA-DRB1*09:01	100%	Dengue virus 2	ELISPOT	IFNg release	Positive	1
539289	GEAAGIFMTATPPGS	NS3	1782	1796	HLA-DRB1*04:03	100%	Dengue virus 2	ELISPOT	IFNg release	Positive	1
539448	IVREAIKRGLRTLIL	NS3	1682	1696	HLA-DRB1*12:02	100%	Dengue virus 2	ELISPOT	IFNg release	Positive	1
539467	KGKTVWFVPSIKAGN	NS3	1830	1844	HLA-DRB1*08:03	100%	Dengue virus 2	ELISPOT	IFNg release	Positive	1
539573	LILAPTRVVAAEMEE	NS3	1694	1708	HLA-DRB1*13:01	100%	Dengue virus 2	ELISPOT	IFNg release	Positive	1
539850	RGLPIRYQTPAIRAE	NS3	1711	1725	HLA-DRB1*13:01	100%	Dengue virus 2	ELISPOT	IFNg release	Positive	1
63672	TFHTMWHVTRGAVLM	NS3	45	59	HLA class II	100%	Dengue virus 2	ELISPOT	IFNg release	Positive	1
64666	TKRYLPAIVREAIKR	NS3	200	214	HLA class II	100%	Dengue virus 2	ELISPOT	IFNg release	Positive	1
740081	REAIKRGLRTLILAP	NS3	1670	1684	HLA-DRB1*14:06	100%	Dengue virus 2	ELISPOT	IFNg release	Positive	1
740193	RTLILAPTRVVAAEM	NS3	1678	1692	HLA-DRB1*14:06	100%	Dengue virus 2	ELISPOT	IFNg release	Positive	1
866477	EALRGLPIRYQTPAI	NS3	1708	1722	HLA-DRB4*01:01	100%	Dengue virus 2	ELISPOT	IFNg release	Positive	1
868183	MWHVTRGAVLMHRGK	NS3	1524	1538	HLA-DRB3*02:02	100%	Dengue virus 2	ELISPOT	IFNg release	Positive	1
868734	RYLPAIVREAIKRGL	NS3	1677	1691	HLA-DRB4*01:01	100%	Dengue virus 2	ELISPOT	IFNg release	Positive	1
869224	TLILAPTRVVAAEME	NS3	1693	1707	HLA-DQA1*01:02/DQB1*06:02	100%	Dengue virus 2	ELISPOT	IFNg release	Positive	1
167780	LTLNLITEMGRLPTFMTQKA	NS4a	2	21	HLA class II	100%	Dengue virus 2	ELISPOT	IFNg release	Positive	1
195107	AASIILEFFLIVLLI	NS4a	2185	2199	HLA-DRB1*15:01	100%	Dengue virus 2	ELISPOT	IFNg release	Positive	1
195355	GGIFLFLMSGKGIGK	NS4a	2145	2159	HLA-DRB1*11:01	100%	Dengue virus 2	ELISPOT	IFNg release	Positive	1
195635	LGMCCIITASILLWY	NS4a	2176	2190	HLA-DRB1*07:01	100%	Dengue virus 2	ELISPOT	IFNg release	Positive	1
195767	MTLGMCCIITASILL	NS4a	2160	2174	HLA-DRB1*07:01	100%	Dengue virus 2	ELISPOT	IFNg release	Positive	1
539801	QDNQLTYVVIAILTV	NS4a	2223	2237	HLA-DRB1*15:02	100%	Dengue virus 2	ELISPOT	IFNg release	Positive	1
539804	QIQPHWIAASIILEF	NS4a	2192	2206	HLA-DRB1*13:01	100%	Dengue virus 2	ELISPOT	IFNg release	Positive	1
540148	YAQIQPHWIAASIIL	NS4a	2176	2190	HLA-DRB1*13:01	100%	Dengue virus 2	ELISPOT	IFNg release	Positive	1
150611	TAALLLLVAHYAIIG	NS4b	2351	2365	HLA-DRB1*01:01	100%	Dengue virus 2	ELISPOT	IFNg release	Positive	1
150636	TLTAALLLLVAHYAI	NS4b	2335	2349	HLA-DRB1*01:01	100%	Dengue virus 2	ELISPOT	IFNg release	Positive	1
195621	LCVTQVLMMRTTWAL	NS4b	2405	2419	HLA-DRB1*07:01	100%	Dengue virus 2	ELISPOT	IFNg release	Positive	1
195882	RFWNTTIAVSMANIF	NS4b	2454	2468	HLA-DRB1*07:01	100%	Dengue virus 2	ELISPOT	IFNg release	Positive	1
195980	SQVNPITLTAALLLL	NS4b	2343	2357	HLA-DQA1*01:02/DQB1*06:02	100%	Dengue virus 2	ELISPOT	IFNg release	Positive	1
539724	NIFRGSYLAGAGLLF	NS4b	2452	2466	HLA-DRB1*09:01	100%	Dengue virus 2	ELISPOT	IFNg release	Positive	1
539755	PASAWTLYAVATTFV	NS4b	2277	2291	HLA-DRB1*09:01	100%	Dengue virus 2	ELISPOT	IFNg release	Positive	1
739437	LDIDLRPASAWTLYA	NS4b	2257	2271	HLA-DRB1*01:02	100%	Dengue virus 2	ELISPOT	IFNg release	Positive	1
866802	FWNTTIAVSMANIFR	NS4b	2455	2469	HLA-DQA1*01:02/DQB1*06:02	100%	Dengue virus 2	ELISPOT	IFNg release	Positive	1
867709	LILCVTQVLMMRTTW	NS4b	2417	2431	HLA-DPB1*04:02	100%	Dengue virus 2	ELISPOT	IFNg release	Positive	1
869089	TAIANQATVLMGLGK	NS4b	2309	2323	HLA-DQA1*01:02/DQB1*06:02	100%	Dengue virus 2	ELISPOT	IFNg release	Positive	1
150597	SRAIWYMWLGARFLE	NS5	2962	2976	HLA-DRB1*01:01	100%	Dengue virus 2	ELISPOT	IFNg release	Positive	1
180582	KTWAYHGSYETKQTG	NS5	2792	2806	HLA class II	100%	Dengue virus 2	ICS	IFNg release	Positive	1
190862	MVTQMAMTDTTPFGQQR	NS5	2828	2844	HLA class II	100%	Dengue virus 2	ICS	IFNg release	Positive	1
195111	ACLGKSYAQMWTLMY	NS5	3243	3257	HLA-DRB1*04:01	100%	Dengue virus 2	ELISPOT	IFNg release	Positive	1
195200	CETCVYNMMGKREKK	NS5	2924	2938	HLA-DRB1*11:01	100%	Dengue virus 2	ELISPOT	IFNg release	Positive	1
195213	DELIGRARISQGAGW	NS5	3223	3237	HLA-DRB1*08:02	100%	Dengue virus 2	ELISPOT	IFNg release	Positive	1
195409	GNIVSSVNMISRMLI	NS5	2717	2731	HLA-DRB1*07:01	100%	Dengue virus 2	ELISPOT	IFNg release	Positive	1
195759	MLINRFTMKHKKATY	NS5	2729	2743	HLA-DRB1*11:01	100%	Dengue virus 2	ELISPOT	IFNg release	Positive	1
195800	NMISRMLINRFTMKH	NS5	2724	2738	HLA-DRB1*08:02	100%	Dengue virus 2	ELISPOT	IFNg release	Positive	1
196040	TGNIVSSVNMISRML	NS5	2716	2730	HLA-DRB1*04:01	100%	Dengue virus 2	ELISPOT	IFNg release	Positive	1
196187	VSSVNMISRMLINRF	NS5	2720	2734	HLA-DRB1*15:01	100%	Dengue virus 2	ELISPOT	IFNg release	Positive	1
196210	WAKNIQTAINQVRSL	NS5	3336	3350	HLA-DRB1*03:01	100%	Dengue virus 2	ELISPOT	IFNg release	Positive	1
196225	WTLMYFHRRDLRLAA	NS5	3253	3267	HLA-DRB1*03:01	100%	Dengue virus 2	ELISPOT	IFNg release	Positive	1
196243	YQNKVVRVQRPTPRG	NS5	3064	3078	HLA-DRB1*08:02	100%	Dengue virus 2	ELISPOT	IFNg release	Positive	1
539195	EAIFKLTYQNKVVRV	NS5	3057	3071	HLA-DRB1*09:01	100%	Dengue virus 2	ELISPOT	IFNg release	Positive	1
539348	GSAKLRWFVERNMVT	NS5	2549	2563	HLA-DRB1*09:01	100%	Dengue virus 2	ELISPOT	IFNg release	Positive	1
539599	LMYFHRRDLRLAANA	NS5	3255	3269	HLA-DRB1*13:01	100%	Dengue virus 2	ELISPOT	IFNg release	Positive	1
539603	LNTFTNMEAQLIRQM	NS5	3100	3114	HLA-DRB1*10:01	100%	Dengue virus 2	ELISPOT	IFNg release	Positive	1
539668	MISRMLINRFTMKHK	NS5	2725	2739	HLA-DRB1*14:04	100%	Dengue virus 2	ELISPOT	IFNg release	Positive	1
539871	RRDLRLAANAICSAV	NS5	3260	3274	HLA-DRB3*02:02	100%	Dengue virus 2	ELISPOT	IFNg release	Positive	1
539932	SRMLINRFTMKHKKA	NS5	2727	2741	HLA-DRB1*08:03	100%	Dengue virus 2	ELISPOT	IFNg release	Positive	1
540066	VNMISRMLINRFTMK	NS5	2723	2737	HLA-DRB1*12:02	100%	Dengue virus 2	ELISPOT	IFNg release	Positive	1
738754	EEFTRKVRSNAALGA	NS5	2874	2888	HLA-DRB1*11:04	100%	Dengue virus 2	ELISPOT	IFNg release	Positive	1
739166	IFKLTYQNKVVRVQR	NS5	3045	3059	HLA-DRB1*14:06	100%	Dengue virus 2	ELISPOT	IFNg release	Positive	1
739791	NLVRLQSGVDVFFTP	NS5	2599	2613	HLA-DRB1*01:02	100%	Dengue virus 2	ELISPOT	IFNg release	Positive	1
866015	AIWYMWLGARFLEFE	NS5	2964	2978	HLA-DPB1*01:01	100%	Dengue virus 2	ELISPOT	IFNg release	Positive	1
866566	EHKKLAEAIFKLTYQ	NS5	3051	3065	HLA-DPB1*01:01	100%	Dengue virus 2	ELISPOT	IFNg release	Positive	1
866648	ETCVYNMMGKREKKL	NS5	2939	2953	HLA-DRB5*01:01	100%	Dengue virus 2	ELISPOT	IFNg release	Positive	1
867419	KKLAEAIFKLTYQNK	NS5	3053	3067	HLA-DPB1*02:01	100%	Dengue virus 2	ELISPOT	IFNg release	Positive	1
867433	KLAEAIFKLTYQNKV	NS5	3054	3068	HLA-DPB1*02:01	100%	Dengue virus 2	ELISPOT	IFNg release	Positive	1
867600	LEFEALGFLNEDHWF	NS5	2975	2989	HLA-DPB1*04:01	100%	Dengue virus 2	ELISPOT	IFNg release	Positive	1
868637	RDLRLAANAICSAVP	NS5	3261	3275	HLA-DQA1*05:01/DQB1*02:01	100%	Dengue virus 2	ELISPOT	IFNg release	Positive	1
868640	RFLEFEALGFLNEDH	NS5	2973	2987	HLA-DPB1*01:01	100%	Dengue virus 2	ELISPOT	IFNg release	Positive	1
869083	SYAQMWTLMYFHRRD	NS5	3248	3262	HLA-DPB1*04:01	100%	Dengue virus 2	ELISPOT	IFNg release	Positive	1
869292	TPVESWEEVPYLGKR	NS5	3319	3333	HLA-DQA1*01:01/DQB1*05:01	100%	Dengue virus 2	ELISPOT	IFNg release	Positive	1
150105	AFLRFLTIPPTAGIL	C	52	66	HLA-DRB1*01:01	81% - <100%	Dengue virus 2	ELISPOT	IFNg release	Positive	1
180597	LFMALVAFLRFLTIP	C	46	60	HLA class II	81% - <100%	Dengue virus 2	ELISPOT	IFNg release	Positive	1
195307	FMALVAFLRFLTIPP	C	47	61	HLA-DRB1*11:01	81% - <100%	Dengue virus 2	ELISPOT	IFNg release	Positive	1
195326	FRKEIGRMLNILNRR	C	84	98	HLA-DRB1*04:01	81% - <100%	Dengue virus 2	ELISPOT	IFNg release	Positive	1
195417	GRMLNILNRRRRTAG	C	89	103	HLA-DRB1*11:01	81% - <100%	Dengue virus 2	ELISPOT	IFNg release	Positive	1
195489	IGRMLNILNRRRRTA	C	88	102	HLA-DRB1*08:02	81% - <100%	Dengue virus 2	ELISPOT	IFNg release	Positive	1
195556	KEIGRMLNILNRRRR	C	86	100	HLA-DRB1*08:02	81% - <100%	Dengue virus 2	ELISPOT	IFNg release	Positive	1
195573	KLFMALVAFLRFLTI	C	45	59	HLA-DRB1*15:01	81% - <100%	Dengue virus 2	ELISPOT	IFNg release	Positive	1
195730	LVAFLRFLTIPPTAG	C	50	64	HLA-DRB1*04:01	81% - <100%	Dengue virus 2	ELISPOT	IFNg release	Positive	1
195744	MALVAFLRFLTIPPT	C	48	62	HLA-DRB1*04:01	81% - <100%	Dengue virus 2	ELISPOT	IFNg release	Positive	1
195886	RGFRKEIGRMLNILN	C	82	96	HLA-DRB1*04:01	81% - <100%	Dengue virus 2	ELISPOT	IFNg release	Positive	1
195899	RMLNILNRRRRTAGV	C	71	85	HLA-DRB1*08:02	81% - <100%	Dengue virus 2	ELISPOT	IFNg release	Positive	1
196093	VAFLRFLTIPPTAGI	C	51	65	HLA-DRB1*04:01	81% - <100%	Dengue virus 2	ELISPOT	IFNg release	Positive	1
26836	IKKSKAINVLRGFRKEIGRM	C	72	91	HLA class II	81% - <100%	Dengue virus 2	ELISPOT	IFNg release	Positive	1
39098	LRGFRKEIGRML	C	81	92	HLA-DPw4	81% - <100%	Dengue virus 2	ICS	IFNg release	Positive	1
539083	ALVAFLRFLTIPPTA	C	49	63	HLA-DRB1*12:02	81% - <100%	Dengue virus 2	ELISPOT	IFNg release	Positive	1
539264	FLRFLTIPPTAGILK	C	53	67	HLA-DRB1*14:02	81% - <100%	Dengue virus 2	ELISPOT	IFNg release	Positive	1
539410	IKKSKAINVLRGFRK	C	72	86	HLA-DRB1*14:04	81% - <100%	Dengue virus 2	ELISPOT	IFNg release	Positive	1
539514	KSKAINVLRGFRKEI	C	74	88	HLA-DRB1*12:02	81% - <100%	Dengue virus 2	ELISPOT	IFNg release	Positive	1
539612	LQGRGPLKLFMALVA	C	38	52	HLA-DRB1*15:02	81% - <100%	Dengue virus 2	ELISPOT	IFNg release	Positive	1
539618	LRGFRKEIGRMLNIL	C	81	95	HLA-DRB1*09:01	81% - <100%	Dengue virus 2	ELISPOT	IFNg release	Positive	1
540060	VLRGFRKEIGRMLNI	C	80	94	HLA-DRB1*09:01	81% - <100%	Dengue virus 2	ELISPOT	IFNg release	Positive	1
540069	VQQLTKRFSLGMLQG	C	26	40	HLA-DRB1*13:01	81% - <100%	Dengue virus 2	ELISPOT	IFNg release	Positive	1
150490	PGFTIMAAILAYTIG	PreM	245	259	HLA-DRB1*01:01	81% - <100%	Dengue virus 2	ELISPOT	IFNg release	Positive	1
195503	ILRHPGFTIMAAILA	PreM	127	141	HLA-DRB1*07:01	81% - <100%	Dengue virus 2	ELISPOT	IFNg release	Positive	1
195702	LRHPGFTIMAAILAY	PreM	242	256	HLA-DRB1*11:01	81% - <100%	Dengue virus 2	ELISPOT	IFNg release	Positive	1
195892	RIETWILRHPGFTIM	PreM	122	136	HLA-DRB1*15:01	81% - <100%	Dengue virus 2	ELISPOT	IFNg release	Positive	1
540130	WILRHPGFTIMAAIL	PreM	126	140	HLA-DRB1*15:06	81% - <100%	Dengue virus 2	ELISPOT	IFNg release	Positive	1
866485	EDGVNMCTLMAIDLG	PreM	142	156	HLA-DQA1*01:02/DQB1*06:02	81% - <100%	Dengue virus 2	ELISPOT	IFNg release	Positive	1
144940	RHVLGRLITVNPIVT	E	345	359	HLA-DRB1*08:02	81% - <100%	Dengue virus 2	ELISPOT	IFNg release	Positive	1
180568	KNPHAKKQDVVVLGS	E	521	535	HLA class II	81% - <100%	Dengue virus 2	ELISPOT	IFNg release	Positive	1
180590	LDFELIKTEAKQPAT	E	41	55	HLA class II	81% - <100%	Dengue virus 2	ELISPOT	IFNg release	Positive	1
195638	LGRLITVNPIVTEKD	E	628	642	HLA-DRB1*08:02	81% - <100%	Dengue virus 2	ELISPOT	IFNg release	Positive	1
195952	SGVSWTMKILIGVII	E	449	463	HLA-DRB1*15:01	81% - <100%	Dengue virus 2	ELISPOT	IFNg release	Positive	1
196140	VLGRLITVNPIVTEK	E	627	641	HLA-DRB1*08:02	81% - <100%	Dengue virus 2	ELISPOT	IFNg release	Positive	1
196215	WIQKETLVTFKNPHA	E	231	245	HLA-DRB1*08:02	81% - <100%	Dengue virus 2	ELISPOT	IFNg release	Positive	1
539252	FETTMRGAKRMAILG	E	402	416	HLA-DRB1*15:02	81% - <100%	Dengue virus 2	ELISPOT	IFNg release	Positive	1
539464	KETLVTFKNPHAKKQ	E	234	248	HLA-DRB1*10:01	81% - <100%	Dengue virus 2	ELISPOT	IFNg release	Positive	1
539648	LVTFKNPHAKKQDVV	E	237	251	HLA-DRB1*15:02	81% - <100%	Dengue virus 2	ELISPOT	IFNg release	Positive	1
539689	MRGAKRMAILGDTAW	E	406	420	HLA-DRB1*15:06	81% - <100%	Dengue virus 2	ELISPOT	IFNg release	Positive	1
539806	QKETLVTFKNPHAKK	E	513	527	HLA-DRB1*04:03	81% - <100%	Dengue virus 2	ELISPOT	IFNg release	Positive	1
65140	TLVTFKNPHAKKQDV	E	236	250	HLA class II	81% - <100%	Dengue virus 2	ELISPOT	IFNg release	Positive	1
738866	EYTIVITPHSGEEHA	E	136	150	HLA-DRB1*04:07	81% - <100%	Dengue virus 2	ELISPOT	IFNg release	Positive	1
739010	GGVFTSIGKALHQVF	E	706	720	HLA-DRB1*14:02	81% - <100%	Dengue virus 2	ELISPOT	IFNg release	Positive	1
180603	LKYSWKTWGKAKMLS	NS1	111	125	HLA class II	81% - <100%	Dengue virus 2	ELISPOT	IFNg release	Positive	1
195458	HNQTFLIDGPETAEC	NS1	904	918	HLA-DRB1*04:01	81% - <100%	Dengue virus 2	ELISPOT	IFNg release	Positive	1
195583	KQDVFCDSKLMSAAI	NS1	949	963	HLA-DRB1*03:01	81% - <100%	Dengue virus 2	ELISPOT	IFNg release	Positive	1
195845	QDVFCDSKLMSAAIK	NS1	175	189	HLA-DRB1*03:01	81% - <100%	Dengue virus 2	ELISPOT	IFNg release	Positive	1
195954	SHNQTFLIDGPETAE	NS1	903	917	HLA-DRB1*04:01	81% - <100%	Dengue virus 2	ELISPOT	IFNg release	Positive	1
196133	VKLTIMTGDIKGIMQ	NS1	84	98	HLA-DRB1*03:01	81% - <100%	Dengue virus 2	ELISPOT	IFNg release	Positive	1
539708	NDTWKMEKASFIEVK	NS1	982	996	HLA-DRB1*09:01	81% - <100%	Dengue virus 2	ELISPOT	IFNg release	Positive	1
539828	QYKFQPESPSKLASA	NS1	806	820	HLA-DRB3*02:02	81% - <100%	Dengue virus 2	ELISPOT	IFNg release	Positive	1

866460	DYGFGVFTTNIWLKL	NS1	932	946	HLA-DPB1*02:01	81% - <100%	Dengue virus 2	ELISPOT	IFNg release	Positive	1	1	1	1	1	1	1	1	1	1	1	1	1	1	0	1	1	1	1	1	1	1	1	1	1	1	1	1	1	1	1	1	1	1	1	0	1	1	0	1	0
866699	FGVFTTNIWLKLREK	NS1	935	949	HLA-DPB1*04:01	81% - <100%	Dengue virus 2	ELISPOT	IFNg release	Positive	1	1	1	1	1	1	1	1	1	1	1	1	1	1	0	1	1	1	1	1	1	1	1	1	1	1	1	1	1	1	1	1	1	1	1	0	0	1	0	0	0
866831	GFGVFTTNIWLKLRE	NS1	934	948	HLA-DPB1*04:01	81% - <100%	Dengue virus 2	ELISPOT	IFNg release	Positive	1	1	1	1	1	1	1	1	1	1	1	1	1	1	0	1	1	1	1	1	1	1	1	1	1	1	1	1	1	1	1	1	1	1	1	0	0	1	0	0	0
869722	YGFGVFTTNIWLKLR	NS1	933	947	HLA-DPB1*04:01	81% - <100%	Dengue virus 2	ELISPOT	IFNg release	Positive	1	1	1	1	1	1	1	1	1	1	1	1	1	1	0	1	1	1	1	1	1	1	1	1	1	1	1	1	1	1	1	1	1	1	1	0	0	1	0	0	0
150273	GVTYLALLAAFKVRP	NS2a	1198	1212	HLA-DRB1*01:01	81% - <100%	Dengue virus 2	ELISPOT	IFNg release	Positive	1	1	1	1	1	1	1	1	1	1	1	1	1	0	1	1	1	1	1	1	1	1	1	1	1	1	1	1	1	1	1	1	1	1	1	0	1	1	0	1	0
195153	ALLAAFKVRPTFAAG	NS2a	1203	1217	HLA-DRB1*08:02	81% - <100%	Dengue virus 2	ELISPOT	IFNg release	Positive	1	1	1	1	1	1	1	1	1	1	1	1	1	0	1	1	1	1	1	1	1	1	1	1	1	1	1	1	1	1	1	1	1	1	1	0	1	1	0	1	0
195650	LKIVRNMEKYQLAVT	NS2a	1261	1275	HLA-DRB1*13:01	81% - <100%	Dengue virus 2	ELISPOT	IFNg release	Positive	1	1	0	1	1	1	1	1	1	1	1	1	1	1	1	1	0	1	1	1	1	1	1	1	0	1	1	1	1	1	1	1	1	1	1	0	0	0	0	0	0
195753	MEKYQLAVTIMAISC	NS2a	1267	1281	HLA-DRB1*10:01	81% - <100%	Dengue virus 2	ELISPOT	IFNg release	Positive	1	1	1	1	1	1	1	1	1	1	1	1	1	0	1	1	1	1	1	1	1	1	1	1	1	1	1	1	1	1	1	1	1	1	1	0	0	0	0	0	0
195799	NMEKYQLAVTIMAIS	NS2a	1266	1280	HLA-DRB1*04:01	81% - <100%	Dengue virus 2	ELISPOT	IFNg release	Positive	1	1	1	1	1	1	1	1	1	1	1	1	1	0	1	1	1	1	1	1	1	1	1	1	1	1	1	1	1	1	1	1	1	1	1	0	0	0	0	0	0
196174	VRNMEKYQLAVTIMA	NS2a	1264	1278	HLA-DRB1*13:01	81% - <100%	Dengue virus 2	ELISPOT	IFNg release	Positive	1	1	0	1	1	1	1	1	1	1	1	1	1	1	1	1	0	1	1	1	1	1	1	1	0	1	1	1	1	1	1	1	1	1	1	0	1	0	0	1	0
196217	WKVSCTILAAVSVSP	NS2a	1292	1306	HLA-DRB1*04:01	81% - <100%	Dengue virus 2	ELISPOT	IFNg release	Positive	1	1	1	1	1	1	1	1	1	1	1	1	1	1	0	1	1	1	1	1	1	1	1	1	1	1	1	1	1	1	1	1	1	1	1	0	0	0	0	0	0
196237	YQLAVTIMAISCVPN	NS2a	1270	1284	HLA-DRB1*08:02	81% - <100%	Dengue virus 2	ELISPOT	IFNg release	Positive	1	1	1	1	1	1	1	1	1	1	1	1	1	0	1	1	1	1	1	1	1	1	1	1	1	1	1	1	1	1	1	1	1	1	1	0	0	0	0	0	0
539474	KIVRNMEKYQLAVTI	NS2a	1262	1276	HLA-DRB1*13:01	81% - <100%	Dengue virus 2	ELISPOT	IFNg release	Positive	1	1	0	1	1	1	1	1	1	1	1	1	1	1	1	1	0	1	1	1	1	1	1	1	0	1	1	1	1	1	1	1	1	1	1	0	0	0	0	0	0
539540	LALLAAFKVRPTFAA	NS2a	1202	1216	HLA-DRB1*14:04	81% - <100%	Dengue virus 2	ELISPOT	IFNg release	Positive	1	1	1	1	1	1	1	1	1	1	1	1	1	0	1	1	1	1	1	1	1	1	1	1	1	1	1	1	1	1	1	1	1	1	1	0	1	1	0	1	0
539706	NAWKVSCTILAAVSV	NS2a	1290	1304	HLA-DRB1*09:01	81% - <100%	Dengue virus 2	ELISPOT	IFNg release	Positive	1	1	1	1	1	1	1	1	1	1	1	1	1	1	0	1	1	1	1	1	1	1	1	1	1	1	1	1	1	1	1	1	1	1	1	0	0	0	0	0	0
540094	VTYLALLAAFKVRPT	NS2a	1199	1213	HLA-DRB1*12:02	81% - <100%	Dengue virus 2	ELISPOT	IFNg release	Positive	1	1	1	1	1	1	1	1	1	1	1	1	1	0	1	1	1	1	1	1	1	1	1	1	1	1	1	1	1	1	1	1	1	1	1	0	1	1	0	1	0
540154	YLALLAAFKVRPTFA	NS2a	1201	1215	HLA-DRB1*14:04	81% - <100%	Dengue virus 2	ELISPOT	IFNg release	Positive	1	1	1	1	1	1	1	1	1	1	1	1	1	0	1	1	1	1	1	1	1	1	1	1	1	1	1	1	1	1	1	1	1	1	1	0	1	1	0	1	0
866586	EKYQLAVTIMAISCV	NS2a	1268	1282	HLA-DQA1*01:02/DQB1*06:02	81% - <100%	Dengue virus 2	ELISPOT	IFNg release	Positive	1	1	1	1	1	1	1	1	1	1	1	1	1	0	1	1	1	1	1	1	1	1	1	1	1	1	1	1	1	1	1	1	1	1	1	0	0	0	0	0	0
195512	IMAVGMVSILASSLL	NS2b	8	22	HLA-DRB1*07:01	81% - <100%	Dengue virus 2	ELISPOT	IFNg release	Positive	1	1	1	1	1	1	1	1	1	1	1	1	1	1	1	1	1	1	1	1	1	1	1	1	0	1	1	1	1	1	1	1	1	1	1	0	1	1	0	1	0
195765	MRLLSPVRVPNYNLI	NS3	1742	1756	HLA-DRB1*13:01	81% - <100%	Dengue virus 2	ELISPOT	IFNg release	Positive	1	1	1	1	1	1	1	1	1	1	1	1	1	1	1	1	1	1	1	1	1	1	1	1	1	1	1	1	1	1	1	0	1	1	1	0	1	1	0	1	0
31726	KKVIQLSRKTFDSEY	NS3	1855	1869	HLA-DRB1*12:02	81% - <100%	Dengue virus 2	ELISPOT	IFNg release	Positive	1	1	1	1	1	1	1	1	1	1	1	1	1	1	1	1	1	1	1	1	1	1	1	1	1	1	1	1	0	1	1	1	1	1	1	0	1	1	0	1	0
539200	EDDIFRKKRLTIMDL	NS3	1654	1668	HLA-DRB1*13:01	81% - <100%	Dengue virus 2	ELISPOT	IFNg release	Positive	1	1	1	1	1	1	1	1	1	1	1	1	1	1	0	1	1	1	1	1	1	1	1	1	1	1	1	1	1	1	1	1	1	1	1	0	0	0	0	0	0
539283	GAYRIKQRGILGYSQ	NS3	1496	1510	HLA-DRB1*08:03	81% - <100%	Dengue virus 2	ELISPOT	IFNg release	Positive	1	1	1	1	1	1	1	1	1	1	1	1	1	1	1	1	1	1	1	0	1	1	1	1	1	1	1	1	1	1	1	1	1	1	0	0	0	0	0	0	0
539713	NGKKVIQLSRKTFDS	NS3	1853	1867	HLA-DRB1*12:02	81% - <100%	Dengue virus 2	ELISPOT	IFNg release	Positive	1	1	1	1	1	1	1	1	1	1	1	1	1	1	1	1	1	1	1	1	1	1	1	1	1	1	1	1	0	1	1	1	1	1	1	0	1	1	0	1	0
539857	RKNGKKVIQLSRKTF	NS3	1851	1865	HLA-DRB1*08:03	81% - <100%	Dengue virus 2	ELISPOT	IFNg release	Positive	1	1	1	1	1	1	1	1	1	1	1	1	1	1	1	1	1	1	1	1	1	1	1	1	1	1	1	1	0	1	1	1	1	1	1	0	1	1	0	1	0
539609	LPTFMTQKARNALDN	NS4a	2092	2106	HLA-DRB1*10:01	81% - <100%	Dengue virus 2	ELISPOT	IFNg release	Positive	1	1	1	1	1	1	1	1	0	1	1	1	1	1	1	1	1	1	1	1	1	1	1	1	1	1	1	1	1	1	1	1	1	1	1	0	0	0	0	0	0
195115	AGAGLLFSIMKNTTN	NS4b	2474	2488	HLA-DRB1*11:01	81% - <100%	Dengue virus 2	ELISPOT	IFNg release	Positive	1	1	1	1	1	1	1	1	1	1	1	1	1	1	0	1	1	1	1	1	1	1	1	1	0	1	1	1	1	1	1	1	1	1	1	0	1	1	0	1	0
195270	ESESNILDIDLRPAS	NS4b	2265	2279	HLA-DRB1*03:01	81% - <100%	Dengue virus 2	ELISPOT	IFNg release	Positive	1	1	1	1	1	1	1	1	1	1	1	1	1	1	0	1	1	1	1	1	1	1	1	1	1	1	1	1	1	1	1	1	1	1	1	0	0	0	0	0	0
195347	GAGLLFSIMKNTTNT	NS4b	2475	2489	HLA-DRB1*11:01	81% - <100%	Dengue virus 2	ELISPOT	IFNg release	Positive	1	1	1	1	1	1	1	1	1	1	1	1	1	1	0	1	1	1	1	1	1	1	1	1	0	1	1	1	1	1	1	1	1	1	1	0	1	1	0	1	0
195629	LFSIMKNTTNTRRGT	NS4b	2479	2493	HLA-DRB1*03:01	81% - <100%	Dengue virus 2	ELISPOT	IFNg release	Positive	1	1	1	1	1	1	1	1	1	1	1	1	1	1	0	1	1	1	1	1	1	1	1	1	0	1	1	1	1	1	1	1	1	1	1	0	1	1	0	1	0
195658	LLFSIMKNTTNTRRG	NS4b	2478	2492	HLA-DRB1*04:01	81% - <100%	Dengue virus 2	ELISPOT	IFNg release	Positive	1	1	1	1	1	1	1	1	1	1	1	1	1	1	0	1	1	1	1	1	1	1	1	1	0	1	1	1	1	1	1	1	1	1	1	0	1	1	0	1	0
195974	SNILDIDLRPASAWT	NS4b	2155	2169	HLA-DRB1*03:01	81% - <100%	Dengue virus 2	ELISPOT	IFNg release	Positive	1	1	1	1	1	1	1	1	1	1	1	1	1	1	0	1	1	1	1	1	1	1	1	1	1	1	1	1	1	1	1	1	1	1	1	0	1	1	0	1	0
539327	GLLFSIMKNTTNTRR	NS4b	2477	2491	HLA-DRB1*14:02	81% - <100%	Dengue virus 2	ELISPOT	IFNg release	Positive	1	1	1	1	1	1	1	1	1	1	1	1	1	1	0	1	1	1	1	1	1	1	1	1	0	1	1	1	1	1	1	1	1	1	1	0	1	1	0	1	0
167817	SRLNALGKSEFQI	NS5	15	27	HLA class II	81% - <100%	Dengue virus 2	ELISPOT	IFNg release	Positive	1	1	1	1	1	1	1	1	1	1	1	1	1	1	1	1	1	1	1	1	1	1	1	1	1	1	1	1	1	1	1	1	0	1	1	0	1	1	0	1	0
195446	HFHELVMKDGRVLVV	NS5	3203	3217	HLA-DRB1*13:01	81% - <100%	Dengue virus 2	ELISPOT	IFNg release	Positive	1	1	1	1	1	1	1	1	1	1	1	1	1	1	0	1	1	1	1	1	1	1	1	1	1	1	1	1	1	1	1	1	1	1	1	0	0	0	0	0	0
195750	MEGEGIFKSIQHLTA	NS5	3114	3128	HLA-DRB1*11:01	81% - <100%	Dengue virus 2	ELISPOT	IFNg release	Positive	1	1	0	1	1	1	1	1	1	1	1	1	0	1	1	1	0	1	1	1	1	1	1	1	0	1	1	0	1	1	1	1	1	1	1	0	0	0	0	0	0
32943	KPWDVVPMV	NS5	2821	2829	HLA class II	81% - <100%	Dengue virus 2	ELISA	IL-2 release	Positive	1	1	1	1	1	1	1	1	1	1	1	1	1	1	1	1	1	1	1	0	1	1	1	1	1	1	1	1	1	1	1	1	1	1	1	0	1	1	0	1	0
539150	DDRFASALTALNDMG	NS5	3162	3176	HLA-DRB1*04:03	81% - <100%	Dengue virus 2	ELISPOT	IFNg release	Positive	1	1	0	1	1	1	1	1	1	1	1	1	1	1	1	1	0	1	1	1	1	1	1	1	0	1	1	1	1	1	1	1	1	1	1	0	1	1	0	1	0
539161	DHWFSRGNSLSGVEG	NS5	2986	3000	HLA-DRB1*09:01	81% - <100%	Dengue virus 2	ELISPOT	IFNg release	Positive	1	1	1	1	1	1	1	1	1	1	1	1	1	1	1	1	1	1	1	1	1	1	1	1	1	1	1	0	1	1	1	1	1	1	1	0	0	0	0	0	0
540065	VNGVVRLLTKPWDVV	NS5	2812	2826	HLA-DRB1*12:02	81% - <100%	Dengue virus 2	ELISPOT	IFNg release	Positive	1	1	1	1	1	1	1	1	1	1	1	1	1	1	1	1	1	1	1	0	1	1	1	1	1	1	1	1	1	1	1	1	1	1	1	0	1	1	0	1	0
869123	TEKDSPVNIEAEPPF	E	349	363	HLA-DQA1*03:01/DQB1*03:02	51%-80%	Dengue virus 2	ELISPOT	IFNg release	Positive	1	1	1	1	1	1	1	1	1	1	1	1	1	0	0	0	0	1	1	1	1	1	0	0	1	1	1	1	1	1	0	1	1	0	1	1	1	1	1	1	1
150192	ETLLLLTLLAAVTGG	NS4a	2146	2160	HLA-DRB1*01:01	51%-80%	Dengue virus 2	ELISPOT	IFNg release	Positive	1	1	0	0	1	1	1	1	1	1	1	1	0	0	0	0	0	1	1	1	1	1	0	0	0	1	1	1	1	1	0	1	1	1	1	0	0	0	0	0	0
196024	TAGVIVMLIPTVMAF	C	101	115	HLA-DRB1*08:02	>0% - 50%	Dengue virus 2	ELISPOT	IFNg release	Positive	0	0	0	0	0	0	0	0	0	0	0	0	1	0	0	0	0	0	0	0	0	0	0	0	1	0	0	0	0	0	0	0	0	0	0	0	0	0	0	0	0
739181	IGRMLNILNRRRRTV	C	42	56	HLA-DRB1*14:02	>0% - 50%	Dengue virus 2	ELISPOT	IFNg release	Positive	0	0	1	0	0	0	0	0	0	0	0	0	0	0	0	0	1	0	0	0	0	0	0	0	0	0	0	0	0	0	0	0	0	0	0	0	0	0	0	0	0
867911	LRGFRREIGRMLNIL	C	81	95	HLA-DRB3*02:02	>0% - 50%	Dengue virus 2	ELISPOT	IFNg release	Positive	0	0	0	0	0	0	0	0	0	0	0	0	1	0	0	0	0	0	0	0	0	0	0	0	0	0	0	0	0	0	0	0	0	0	0	0	0	0	0	0	0
180708	QRIETWILRHPGFTM	PreM	121	135	HLA class II	>0% - 50%	Dengue virus 2	ELISPOT	IFNg release	Positive-Low	0	0	0	0	0	0	0	0	0	0	0	0	0	0	0	0	0	0	0	0	0	0	0	0	1	0	0	0	0	0	0	0	0	0	0	0	0	0	0	1	0
167819	SSQQKTDWIPLALTIKGLNP	NS2a	184	203	HLA-DP	>0% - 50%	Dengue virus 2	ELISPOT	IFNg release	Positive	0	0	0	0	0	0	0	0	0	0	0	0	0	0	0	0	0	0	0	0	0	0	0	0	0	0	1	0	0	0	0	0	0	0	0	0	1	0	0	1	0
195406	GMMVLKIVRNMEKYQ	NS2a	1257	1271	HLA-DRB1*08:02	>0% - 50%	Dengue virus 2	ELISPOT	IFNg release	Positive	0	0	0	0	0	0	0	0	0	0	0	0	0	1	1	1	0	0	0	0	0	0	1	1	0	0	0	0	0	0	1	0	0	0	0	0	0	0	0	0	0
195567	KHAILLVAVSFVTLI	NS2a	1157	1171	HLA-DRB1*07:01	>0% - 50%	Dengue virus 2	ELISPOT	IFNg release	Positive	0	0	1	0	0	0	0	0	0	0	0	0	0	0	0	0	1	0	0	0	0	0	0	0	0	0	0	0	0	0	0	0	0	0	0	0	1	1	0	1	0
195610	LALGMMVLKIVRNME	NS2a	1254	1268	HLA-DRB1*13:01	>0% - 50%	Dengue virus 2	ELISPOT	IFNg release	Positive	0	0	0	0	0	0	0	0	0	0	0	0	0	1	1	1	0	0	0	0	0	0	1	1	0	0	0	0	0	0	1	0	0	0	0	0	0	0	0	0	0
539108	AVSFVTLITGNMSFR	NS2a	1164	1178	HLA-DRB1*10:01	>0% - 50%	Dengue virus 2	ELISPOT	IFNg release	Positive	0	0	1	0	0	0	0	0	0	0	0	0	0	0	0	0	1	0	0	0	0	0	0	0	0	0	0	0	0	0	0	0	0	0	0	0	1	1	0	1	0
539562	LGMMVLKIVRNMEKY	NS2a	1256	1270	HLA-DRB1*12:02	>0% - 50%	Dengue virus 2	ELISPOT	IFNg release	Positive	0	0	0	0	0	0	0	0	0	0	0	0	0	1	1	1	0	0	0	0	0	0	1	1	0	0	0	0	0	0	1	0	0	0	0	0	0	0	0	0	0
539686	MMVLKIVRNMEKYQL	NS2a	1244	1258	HLA-DRB1*14:06	>0% - 50%	Dengue virus 2	ELISPOT	IFNg release	Positive	0	0	0	0	0	0	0	0	0	0	0	0	0	1	1	1	0	0	0	0	0	0	1	1	0	0	0	0	0	0	1	0	0	0	0	0	0	0	0	0	0
540026	VAVSFVTLITGNMSF	NS2a	1163	1177	HLA-DRB1*10:01	>0% - 50%	Dengue virus 2	ELISPOT	IFNg release	Positive	0	0	1	0	0	0	0	0	0	0	0	0	0	0	0	0	1	0	0	0	0	0	0	0	0	0	0	0	0	0	0	0	0	0	0	0	1	1	0	1	0
540086	VSFVTLITGNMSFRD	NS2a	1165	1179	HLA-DRB1*10:01	>0% - 50%	Dengue virus 2	ELISPOT	IFNg release	Positive	0	0	1	0	0	0	0	0	0	0	0	0	0	0	0	0	1	0	0	0	0	0	0	0	0	0	0	0	0	0	0	0	0	0	0	0	1	1	0	1	0
869200	TKHAILLVAVSFVTL	NS2a	1156	1170	HLA-DPB1*04:01	>0% - 50%	Dengue virus 2	ELISPOT	IFNg release	Positive	0	0	1	0	0	0	0	0	0	0	0	0	0	0	0	0	1	0	0	0	0	0	0	0	0	0	0	0	0	0	0	0	0	0	0	0	1	1	0	1	0
150362	LETLLLLTLLATVTG	NS4a	2145	2159	HLA-DRB1*01:01	>0% - 50%	Dengue virus 2	ELISPOT	IFNg release	Positive	0	0	1	0	0	0	0	0	0	0	0	0	1	1	1	1	1	0	0	0	0	0	1	1	0	0	0	0	0	0	1	0	0	0	0	0	1	1	0	1	0
196054	TLETLLLLTLLATVT	NS4a	2144	2158	HLA-DRB1*15:01	>0% - 50%	Dengue virus 2	ELISPOT	IFNg release	Positive	0	0	1	0	0	0	0	0	0	0	0	0	1	1	1	1	1	0	0	0	0	0	1	1	0	0	0	0	0	0	1	0	0	0	0	0	1	1	0	1	0
196089	TVTGGIFLFLMSGKG	NS4a	2142	2156	HLA-DRB1*11:01	>0% - 50%	Dengue virus 2	ELISPOT	IFNg release	Positive	0	0	1	0	0	0	0	0	0	0	0	0	1	1	1	1	1	0	0	0	0	0	1	1	1	0	0	0	0	0	1	0	0	0	0	0	0	0	0	0	0
738567	AYNHALSELPETLET	NS4a	2119	2133	HLA-DRB1*04:07	>0% - 50%	Dengue virus 2	ELISPOT	IFNg release	Positive	0	0	1	0	0	0	0	0	0	0	0	0	0	0	0	0	1	0	0	0	0	0	0	0	1	0	0	0	0	0	0	0	0	0	0	0	1	1	0	1	0
195398	GLLFSVMKNTTNTRR	NS4b	2477	2491	HLA-DRB1*04:01	>0% - 50%	Dengue virus 2	ELISPOT	IFNg release	Positive	0	0	0	0	0	0	0	0	0	0	0	0	0	0	0	0	0	0	0	0	0	0	0	0	1	0	0	0	0	0	0	0	0	0	0	0	0	0	0	0	0
195233	EDHWFSRENSLSGVE	NS5	2985	2999	HLA-DRB1*04:01	>0% - 50%	Dengue virus 2	ELISPOT	IFNg release	Positive	0	0	0	0	0	0	0	0	0	0	0	0	0	0	0	0	0	0	0	0	0	0	0	0	0	0	0	1	0	0	0	0	0	0	0	0	1	1	0	1	0
195428	GTVMDIISRRDQRGS	NS5	3078	3092	HLA-DRB1*03:01	>0% - 50%	Dengue virus 2	ELISPOT	IFNg release	Positive	0	0	1	0	0	0	0	0	0	0	0	0	0	0	0	0	1	0	0	0	0	0	0	0	1	0	0	0	0	0	0	0	0	0	0	0	1	1	0	1	0
195460	HRLGYILRDVSRKEG	NS5	3004	3018	HLA-DRB1*08:02	>0% - 50%	Dengue virus 2	ELISPOT	IFNg release	Positive	0	0	0	0	0	0	0	0	0	0	0	0	0	0	0	0	0	0	0	0	0	0	0	0	1	0	0	0	0	0	0	0	0	0	0	0	0	0	0	0	0
195640	LGYILRDVSKKEGGA	NS5	2992	3006	HLA-DRB1*03:01	>0% - 50%	Dengue virus 2	ELISPOT	IFNg release	Positive	0	0	1	0	0	0	0	0	0	0	0	0	0	0	0	0	1	0	0	0	0	0	0	0	0	0	0	1	0	0	0	0	0	0	0	0	1	1	0	1	0
540064	VNGVVRLLTKPWDVI	NS5	2812	2826	HLA-DRB1*12:02	>0% - 50%	Dengue virus 2	ELISPOT	IFNg release	Positive	0	0	0	0	0	0	0	0	0	0	0	0	0	0	0	0	0	0	0	1	0	0	0	0	0	0	0	0	0	0	0	0	0	0	0	0	0	0	0	0	0
740675	VRSNAALGAIFTDEN	NS5	2880	2894	HLA-DRB1*11:04	>0% - 50%	Dengue virus 2	ELISPOT	IFNg release	Positive	0	0	1	1	0	0	0	0	0	0	0	0	1	1	1	1	1	0	0	0	0	0	1	1	1	0	0	0	0	0	1	0	0	0	0	0	1	1	0	1	0
868668	RKVRSNAALGAIFTD	NS5	2892	2906	HLA-DRB3*02:02	>0% - 50%	Dengue virus 2	ELISPOT	IFNg release	Positive	0	0	1	1	0	0	0	0	0	0	0	0	1	1	1	1	1	0	0	0	0	0	1	1	1	0	0	0	0	0	1	0	0	0	0	0	1	1	0	1	0
869307	TRKVRSNAALGAIFT	NS5	2891	2905	HLA-DRB3*02:02	>0% - 50%	Dengue virus 2	ELISPOT	IFNg release	Positive	0	0	1	1	0	0	0	0	0	0	0	0	1	1	1	1	1	0	0	0	0	0	1	1	1	0	0	0	0	0	1	0	0	0	0	0	1	1	0	1	0

CD4 DENV-3 Epitopes

Epitope_ID	AA_Seq	Protein_Name	Start	End	Allele	step	Serotype	Method/Technique	Assay Group	Qualitative Measure
195289	FCLMMMLPATLAFHL	C	103	117	HLA-DRB1*01:01	0%	Dengue virus 3	ELISPOT	IFNg release	Positive
195517	INMLKRVRNRVSTGP	C	13	27	HLA-DRB1*08:02	0%	Dengue virus 3	ELISPOT	IFNg release	Positive
195519	INMLKRVRNRVSTGT	C	13	27	HLA-DRB1*08:02	0%	Dengue virus 3	ELISPOT	IFNg release	Positive
195618	LCLMMIMPAALAFHL	C	103	117	HLA-DRB1*01:01	0%	Dengue virus 3	ELISPOT	IFNg release	Positive
195803	NMLKRVRNRVSTGPQ	C	14	28	HLA-DRB1*11:01	0%	Dengue virus 3	ELISPOT	IFNg release	Positive
195966	SLCLMMIMPAALAFH	C	102	116	HLA-DRB1*08:02	0%	Dengue virus 3	ELISPOT	IFNg release	Positive
539447	IVNRRKKTSLCLMMM	C	94	108	HLA-DRB1*13:01	0%	Dengue virus 3	ELISPOT	IFNg release	Positive
539906	SIINRRKRTSLCLMM	C	93	107	HLA-DRB1*13:01	0%	Dengue virus 3	ELISPOT	IFNg release	Positive
540118	WGSFKKSGAVKVLRG	C	69	83	HLA-DRB1*09:01	0%	Dengue virus 3	ELISPOT	IFNg release	Positive
540119	WGTFKKLGAIKVLKG	C	69	83	HLA-DRB1*09:01	0%	Dengue virus 3	ELISPOT	IFNg release	Positive
540120	WGTFKKLGAIKVLRG	C	69	83	HLA-DRB1*09:01	0%	Dengue virus 3	ELISPOT	IFNg release	Positive
540122	WGTFKKSGAIKVLKS	C	69	83	HLA-DRB1*09:01	0%	Dengue virus 3	ELISPOT	IFNg release	Positive
540123	WGTFKKSGAIKVLRG	C	69	83	HLA-DRB1*09:01	0%	Dengue virus 3	ELISPOT	IFNg release	Positive
540124	WGTFKKSGAIRVLRG	C	69	83	HLA-DRB1*09:01	0%	Dengue virus 3	ELISPOT	IFNg release	Positive
591709	LRGFKKEISNMLSII	C	81	95	HLA-DRB1*13:02	0%	Dengue virus 3	ELISPOT	IFNg release	Positive
591723	NMLSIINRRKKTSLC	C	90	104	HLA-DRB1*11:01	0%	Dengue virus 3	ELISPOT	IFNg release	Positive
591746	RGFKKEISNMLSIIN	C	82	96	HLA-DRB1*04:01	0%	Dengue virus 3	ELISPOT	IFNg release	Positive
867744	LKSFKKEISNMLSII	C	81	95	HLA-DRB3*02:02	0%	Dengue virus 3	ELISPOT	IFNg release	Positive
867909	LRGFKREISNMLSII	C	64	78	HLA-DRB3*02:02	0%	Dengue virus 3	ELISPOT	IFNg release	Positive
868733	RWGTFKKSGAIKVLR	C	48	62	HLA-DRB3*02:02	0%	Dengue virus 3	ELISPOT	IFNg release	Positive
195968	SLIQKVVIFILLMLV	PreM	261	275	HLA-DRB1*15:06	0%	Dengue virus 3	ELISPOT	IFNg release	Positive
196067	TQKVVIFVLLMLVTP	PreM	263	277	HLA-DRB1*15:06	0%	Dengue virus 3	ELISPOT	IFNg release	Positive
539529	KVVIFILLILVTPSM	PreM	265	279	HLA-DRB1*15:06	0%	Dengue virus 3	ELISPOT	IFNg release	Positive
539808	QKVVIFTLLMLVTPS	PreM	264	278	HLA-DRB1*15:06	0%	Dengue virus 3	ELISPOT	IFNg release	Positive
539476	KKELLVTFKNAHAKK	E	511	525	HLA-DRB1*14:04	0%	Dengue virus 3	ELISPOT	IFNg release	Positive
539856	RKELLTTFKNAHAKK	E	511	525	HLA-DRB1*04:03	0%	Dengue virus 3	ELISPOT	IFNg release	Positive
591605	AFVLKKEVSETQHGT	E	303	317	HLA-DRB1*04:01	0%	Dengue virus 3	ELISPOT	IFNg release	Positive
591719	NEMILLTMKNRAWMV	E	474	488	HLA-DRB1*11:01	0%	Dengue virus 3	ELISPOT	IFNg release	Positive
867374	KAWIVHRQWFFDLPL	E	482	496	HLA-DQA1*01:01/DQB1*05:01	0%	Dengue virus 3	ELISPOT	IFNg release	Positive
539070	AKIVTAETRNSSFII	NS1	121	135	HLA-DRB1*13:01	0%	Dengue virus 3	ELISPOT	IFNg release	Positive
539457	KAKIVIAETQNSSFI	NS1	893	907	HLA-DRB1*13:01	0%	Dengue virus 3	ELISPOT	IFNg release	Positive
866698	FGVFTTNIWLKLRDV	NS1	933	947	HLA-DPB1*04:01	0%	Dengue virus 3	ELISPOT	IFNg release	Positive
195125	AGVIFTFVLLLSGQI	NS2a	1159	1173	HLA-DRB1*07:01	0%	Dengue virus 3	ELISPOT	IFNg release	Positive
195128	AGVLFMFVLLLSGQI	NS2a	1159	1173	HLA-DRB1*07:01	0%	Dengue virus 3	ELISPOT	IFNg release	Positive
195133	AHTFIMIGSNASDRM	NS2a	1179	1193	HLA-DRB1*04:01	0%	Dengue virus 3	ELISPOT	IFNg release	Positive
195166	ANTFIMIGSNASDRM	NS2a	1179	1193	HLA-DRB1*04:01	0%	Dengue virus 3	ELISPOT	IFNg release	Positive
195456	HMIAGILFMFVLLLS	NS2a	1156	1170	HLA-DRB1*11:01	0%	Dengue virus 3	ELISPOT	IFNg release	Positive
195645	LISLTCSNTIFTLTV	NS2a	1274	1288	HLA-DRB1*07:01	0%	Dengue virus 3	ELISPOT	IFNg release	Positive
195734	LVSLTCSNTILTLTV	NS2a	1274	1288	HLA-DRB1*07:01	0%	Dengue virus 3	ELISPOT	IFNg release	Positive
196123	VGVLFTFVLLLSGQI	NS2a	1159	1173	HLA-DRB1*07:01	0%	Dengue virus 3	ELISPOT	IFNg release	Positive
865959	AGALFTFVLLLSGQI	NS2a	1159	1173	HLA-DPB1*04:02	0%	Dengue virus 3	ELISPOT	IFNg release	Positive
867702	LIATFKIQPFLTLGF	NS2a	1202	1216	HLA-DPB1*01:01	0%	Dengue virus 3	ELISPOT	IFNg release	Positive
868109	MIAGVIFTFVLLLSG	NS2a	1157	1171	HLA-DPB1*04:02	0%	Dengue virus 3	ELISPOT	IFNg release	Positive
869592	VQPFLALGFFLRKLT	NS2a	1208	1222	HLA-DPB1*01:01	0%	Dengue virus 3	ELISPOT	IFNg release	Positive
739173	IGLVSILASSLLRND	NS2b	1354	1368	HLA-DRB1*11:04	0%	Dengue virus 3	ELISPOT	IFNg release	Positive
49767	PTRVVAAEMEEAMKG	NS3	1697	1711	HLA-DRB1*15:01	0%	Dengue virus 3	ICS	IFNg release	Positive
739020	GIGKTSIGLICVIVS	NS4a	2169	2183	HLA-DRB1*11:04	0%	Dengue virus 3	ELISPOT	IFNg release	Positive
867986	LTLTAAVLLLATHYA	NS4b	2347	2361	HLA-DQA1*01:02/DQB1*06:02	0%	Dengue virus 3	ELISPOT	IFNg release	Positive
195780	NEDHWFSRDNSYSGV	NS5	2982	2996	HLA-DRB1*04:01	0%	Dengue virus 3	ELISPOT	IFNg release	Positive
32944	KPWDVVPTV	NS5	2819	2827	HLA class II	0%	Dengue virus 3	ELISA	IL-2 release	Positive
539604	LNTFTNMEAQLVRQM	NS5	3098	3112	HLA-DRB1*10:01	0%	Dengue virus 3	ELISPOT	IFNg release	Positive
539739	NMEVQLVRQMEGEGV	NS5	3103	3117	HLA-DRB1*13:01	0%	Dengue virus 3	ELISPOT	IFNg release	Positive
540019	TYGLNTFTNMEAQLV	NS5	3095	3109	HLA-DRB1*04:03	0%	Dengue virus 3	ELISPOT	IFNg release	Positive
740771	YGLNTFTNMEVQLVR	NS5	3096	3110	HLA-DRB1*04:07	0%	Dengue virus 3	ELISPOT	IFNg release	Positive
195303	FKKEISNMLSIINKR	C	37	51	HLA-DRB1*04:01	100%	Dengue virus 3	ELISPOT	IFNg release	Positive
195306	FLRFLAIPPTAGVLA	C	6	20	HLA-DRB1*01:01	100%	Dengue virus 3	ELISPOT	IFNg release	Positive
195469	IAFLRFLAIPPTAGV	C	4	18	HLA-DRB1*04:01	100%	Dengue virus 3	ELISPOT	IFNg release	Positive
195497	IKVLKGFKKEISNML	C	31	45	HLA-DRB1*04:01	100%	Dengue virus 3	ELISPOT	IFNg release	Positive
195518	INMLKRVRNRVSTGS	C	13	27	HLA-DRB1*08:02	100%	Dengue virus 3	ELISPOT	IFNg release	Positive
195611	LARWGTFKKSGAIKV	C	19	33	HLA-DRB1*07:01	100%	Dengue virus 3	ELISPOT	IFNg release	Positive
195649	LKGFKKEISNMLSII	C	34	48	HLA-DRB1*07:01	100%	Dengue virus 3	ELISPOT	IFNg release	Positive
195961	SINMLKRVRNRVSTG	C	12	26	HLA-DRB1*08:02	100%	Dengue virus 3	ELISPOT	IFNg release	Positive
36858	LKGFKKEISNML	C	81	92	HLA-DPw4	100%	Dengue virus 3	ICS	IFNg release	Positive
539277	GAIKVLKGFKKEISN	C	29	43	HLA-DRB1*12:02	100%	Dengue virus 3	ELISPOT	IFNg release	Positive
539512	KSGAIKVLKGFKKEI	C	27	41	HLA-DRB1*12:02	100%	Dengue virus 3	ELISPOT	IFNg release	Positive
539787	PSINMLKRVRNRVST	C	11	25	HLA-DRB1*14:04	100%	Dengue virus 3	ELISPOT	IFNg release	Positive
539897	SGAIKVLKGFKKEIS	C	28	42	HLA-DRB1*14:04	100%	Dengue virus 3	ELISPOT	IFNg release	Positive
540121	WGTFKKSGAIKVLKG	C	22	36	HLA-DRB1*09:01	100%	Dengue virus 3	ELISPOT	IFNg release	Positive
738443	AFLRFLAIPPTAGVL	C	52	66	HLA-DRB1*04:07	100%	Dengue virus 3	ELISPOT	IFNg release	Positive
740207	RWGTFKKSGAIKVLK	C	68	82	HLA-DRB1*14:02	100%	Dengue virus 3	ELISPOT	IFNg release	Positive
195602	KVVIFILLMLVTPSM	PreM	265	279	HLA-DRB1*01:01	100%	Dengue virus 3	ELISPOT	IFNg release	Positive
195726	LTQKVVIFILLMLVT	PreM	262	276	HLA-DRB1*11:01	100%	Dengue virus 3	ELISPOT	IFNg release	Positive
195830	PGFTILALFLAHYIG	PreM	245	259	HLA-DRB1*15:06	100%	Dengue virus 3	ELISPOT	IFNg release	Positive
196203	VVIFILLMLVTPSMT	PreM	266	280	HLA-DRB1*07:01	100%	Dengue virus 3	ELISPOT	IFNg release	Positive
540004	TSLTQKVVIFILLML	PreM	260	274	HLA-DPB1*04:02	100%	Dengue virus 3	ELISPOT	IFNg release	Positive
867082	HPGFTILALFLAHYI	PreM	244	258	HLA-DPB1*02:01	100%	Dengue virus 3	ELISPOT	IFNg release	Positive
869302	TQKVVIFILLMLVTP	PreM	263	277	HLA-DPB1*04:02	100%	Dengue virus 3	ELISPOT	IFNg release	Positive
13376	EMILLTMKNKAWMVH	E	473	487	HLA-DRB1*13:01	100%	Dengue virus 3	ELISPOT	IFNg release	Positive
195215	DFNEMILLTMKNKAW	E	470	484	HLA-DRB1*14:04	100%	Dengue virus 3	ELISPOT	IFNg release	Positive
539710	NEMILLTMKNKAWMV	E	472	486	HLA-DRB1*04:03	100%	Dengue virus 3	ELISPOT	IFNg release	Positive
741746	MRCVGVGNRDFVEGLSGATW	E	281	300	HLA class II	100%	Dengue virus 3	ELISPOT	IFNg release	Positive
190996	TEQYKFQADSPKRLA	NS1	802	816	HLA-DRB5*01:01	100%	Dengue virus 3	ELISPOT	IFNg release	Positive
195419	GSWKLEKASLIEVKT	NS1	981	995	HLA-DRB1*01:01	100%	Dengue virus 3	ELISPOT	IFNg release	Positive
196235	YKFQADSPKRLATAI	NS1	805	819	HLA-DRB1*03:01	100%	Dengue virus 3	ELISPOT	IFNg release	Positive
539717	NGSWKLEKASLIEVK	NS1	980	994	HLA-DRB1*09:01	100%	Dengue virus 3	ELISPOT	IFNg release	Positive
539825	QYKFQADSPKRLATA	NS1	804	818	HLA-DRB3*02:02	100%	Dengue virus 3	ELISPOT	IFNg release	Positive
866700	FGVFTTNIWLKLREV	NS1	933	947	HLA-DPB1*01:01	100%	Dengue virus 3	ELISPOT	IFNg release	Positive
867042	HADMGYWIESQKNGS	NS1	968	982	HLA-DQA1*01:01/DQB1*05:01	100%	Dengue virus 3	ELISPOT	IFNg release	Positive
195337	FTLTVAWRTATLILA	NS2a	1284	1298	HLA-DRB1*07:01	100%	Dengue virus 3	ELISPOT	IFNg release	Positive
195609	LALGFFLRKLTSREN	NS2a	1212	1226	HLA-DRB1*11:01	100%	Dengue virus 3	ELISPOT	IFNg release	Positive
195632	LGFFLRKLTSRENLL	NS2a	1214	1228	HLA-DRB1*11:01	100%	Dengue virus 3	ELISPOT	IFNg release	Positive
539075	ALGFFLRKLTSRENL	NS2a	1213	1227	HLA-DRB1*12:02	100%	Dengue virus 3	ELISPOT	IFNg release	Positive
539429	IQPFLALGFFLRKLT	NS2a	1208	1222	HLA-DRB1*15:06	100%	Dengue virus 3	ELISPOT	IFNg release	Positive
539568	LIATFKIQPFLALGF	NS2a	1202	1216	HLA-DRB1*15:06	100%	Dengue virus 3	ELISPOT	IFNg release	Positive
867551	LALIATFKIQPFLAL	NS2a	1200	1214	HLA-DPB1*04:01	100%	Dengue virus 3	ELISPOT	IFNg release	Positive
868046	MALKLITQFETYQLW	NS2a	1257	1271	HLA-DPB1*01:01	100%	Dengue virus 3	ELISPOT	IFNg release	Positive
868303	PFLALGFFLRKLTSR	NS2a	1210	1224	HLA-DPB1*01:01	100%	Dengue virus 3	ELISPOT	IFNg release	Positive
868551	QPFLALGFFLRKLTS	NS2a	1209	1223	HLA-DPB1*02:01	100%	Dengue virus 3	ELISPOT	IFNg release	Positive
869256	TLTVAWRTATLILAG	NS2a	1285	1299	HLA-DPB1*04:02	100%	Dengue virus 3	ELISPOT	IFNg release	Positive
167823	TMRIKDDETENILTVLLKTA	NS2b	83	102	HLA-DRB1*15:01	100%	Dengue virus 3	ELISPOT	IFNg release	Positive
195180	ASSLLRNDVPMAGPL	NS2b	1361	1375	HLA-DRB1*03:01	100%	Dengue virus 3	ELISPOT	IFNg release	Positive
195403	GLVSILASSLLRNDV	NS2b	1355	1369	HLA-DRB1*01:01	100%	Dengue virus 3	ELISPOT	IFNg release	Positive
195994	SSLLRNDVPMAGPLV	NS2b	1362	1376	HLA-DRB1*03:01	100%	Dengue virus 3	ELISPOT	IFNg release	Positive
196152	VMAVGLVSILASSLL	NS2b	1351	1365	HLA-DRB1*07:01	100%	Dengue virus 3	ELISPOT	IFNg release	Positive
539100	AVGLVSILASSLLRN	NS2b	1353	1367	HLA-DRB1*10:01	100%	Dengue virus 3	ELISPOT	IFNg release	Positive
1067120	GKVVGLYGNGVVTKN	NS3	1617	1631	HLA-DRB1*15:01	100%	Dengue virus 3	ELISPOT	IFNg release	Positive
150544	RRLRTLILAPTRVVA	NS3	1688	1702	HLA-DRB1*01:01	100%	Dengue virus 3	ELISPOT	IFNg release	Positive
150617	TFTMRLLSPVRVPNY	NS3	1738	1752	HLA-DRB1*01:01	100%	Dengue virus 3	ELISPOT	IFNg release	Positive
190710	ANCLRKNGKKVIQLS	NS3	1846	1860	HLA-DRB1*13:01	100%	Dengue virus 3	ELISPOT	IFNg release	Positive
195243	EGKVVGLYGNGVVTK	NS3	5	19	HLA-DRB1*15:01	100%	Dengue virus 3	ELISPOT	IFNg release	Positive
195430	GVFHTMWHVTRGAVL	NS3	1517	1531	HLA-DRB1*07:01	100%	Dengue virus 3	ELISPOT	IFNg release	Positive
195898	RLRTLILAPTRVVAA	NS3	1689	1703	HLA-DRB1*04:03	100%	Dengue virus 3	ELISPOT	IFNg release	Positive
30395	KEGEKKKLRPRWLDA	NS3	585	599	HLA class II	100%	Dengue virus 3	ICS	IFNg release	Positive
45670	NREGKVVGLYGNGVV	NS3	141	155	HLA-DRB1*15:01	100%	Dengue virus 3	multimer/tetramer	qualitative binding	Positive
539714	NGKKVIQLSRKTFDT	NS3	1852	1866	HLA-DRB1*12:02	100%	Dengue virus 3	ELISPOT	IFNg release	Positive
539838	REAIKRRLRTLILAP	NS3	1683	1697	HLA-DRB1*14:04	100%	Dengue virus 3	ELISPOT	IFNg release	Positive
539844	REGKVVGLYGNGVVT	NS3	1615	1629	HLA-DRB1*01:02	100%	Dengue virus 3	ELISPOT	IFNg release	Positive
539991	TMRLLSPVRVPNYNL	NS3	1740	1754	HLA-DRB1*13:01	100%	Dengue virus 3	ELISPOT	IFNg release	Positive
540039	VGLYGNGVVTKNGGY	NS3	9	23	HLA-DRB1*15:06	100%	Dengue virus 3	ELISPOT	IFNg release	Positive
55024	RNLTIMDLHPGSGKT	NS3	187	201	HLA class II	100%	Dengue virus 3	ICS	IFNg release	Positive
66127	TRVVAAEMEEA	NS3	225	235	HLA-DR15	100%	Dengue virus 3	51 chromium	cytotoxicity	Positive
866771	FQTTTGEIGAIALDF	NS3	1589	1603	HLA-DQA1*03:01/DQB1*03:02	100%	Dengue virus 3	ELISPOT	IFNg release	Positive
867875	LNDWDFVVTTDISEM	NS3	1873	1887	HLA-DQA1*03:01/DQB1*03:02	100%	Dengue virus 3	ELISPOT	IFNg release	Positive
195828	PETMETLLLLGLMIL	NS4a	2141	2155	HLA-DRB1*15:06	100%	Dengue virus 3	ELISPOT	IFNg release	Positive
196056	TLLLLGLMILLTGGA	NS4a	2146	2160	HLA-DRB1*15:06	100%	Dengue virus 3	ELISPOT	IFNg release	Positive
195346	GAGLAFSIMKSVGTG	NS4b	2474	2488	HLA-DRB1*04:01	100%	Dengue virus 3	ELISPOT	IFNg release	Positive
195607	LAFSIMKSVGTGKRG	NS4b	2477	2491	HLA-DRB1*04:01	100%	Dengue virus 3	ELISPOT	IFNg release	Positive
738453	AGLAFSIMKSVGTGK	NS4b	2475	2489	HLA-DRB1*04:07	100%	Dengue virus 3	ELISPOT	IFNg release	Positive
868878	SLAAIANQAVVLMGL	NS4b	2306	2320	HLA-DQA1*01:02/DQB1*06:02	100%	Dengue virus 3	ELISPOT	IFNg release	Positive
195136	AIFKLTYQNKVVKVQ	NS5	3056	3070	HLA-DRB1*09:01	100%	Dengue virus 3	ELISPOT	IFNg release	Positive
195273	ETKGVERLKRMAISG	NS5	3138	3152	HLA-DRB1*11:01	100%	Dengue virus 3	ELISPOT	IFNg release	Positive
195382	GKVRKDIPQWQPSKG	NS5	3175	3189	HLA-DRB1*03:01	100%	Dengue virus 3	ELISPOT	IFNg release	Positive
195437	GYILRDISKIPGGAM	NS5	43	57	HLA-DRB1*08:02	100%	Dengue virus 3	ELISPOT	IFNg release	Positive
195568	KHGGMLVRNPLSRNS	NS5	2689	2703	HLA-DRB1*08:02	100%	Dengue virus 3	ELISPOT	IFNg release	Positive
195641	LHKLGYILRDISKIP	NS5	39	53	HLA-DRB1*03:01	100%	Dengue virus 3	ELISPOT	IFNg release	Positive
195779	NDMGKVRKDIPQWQP	NS5	3172	3186	HLA-DRB1*03:01	100%	Dengue virus 3	ELISPOT	IFNg release	Positive
195875	RAIWYMWLGARYLEF	NS5	2961	2975	HLA-DRB1*01:01	100%	Dengue virus 3	ELISPOT	IFNg release	Positive
195939	SCVYNMMGKREKKLG	NS5	2938	2952	HLA-DRB1*11:01	100%	Dengue virus 3	ELISPOT	IFNg release	Positive
231420	RYLEFEALGFLNEDH	NS5	2971	2985	HLA-DPB1*04:01	100%	Dengue virus 3	ELISPOT	IFNg release	Positive
539458	KCGSCVYNMMGKREK	NS5	2935	2949	HLA-DRB1*13:01	100%	Dengue virus 3	ELISPOT	IFNg release	Positive
539600	LMYFHRRDLRLASNA	NS5	3254	3268	HLA-DRB1*13:01	100%	Dengue virus 3	ELISPOT	IFNg release	Positive
539704	NAIFKLTYQNKVVKV	NS5	3055	3069	HLA-DRB1*09:01	100%	Dengue virus 3	ELISPOT	IFNg release	Positive
539872	RRDLRLASNAICSAV	NS5	3259	3273	HLA-DRB1*04:03	100%	Dengue virus 3	ELISPOT	IFNg release	Positive
540152	YKTWAYHGSYEVKAT	NS5	2789	2803	HLA-DRB1*09:01	100%	Dengue virus 3	ELISPOT	IFNg release	Positive
738753	EEFLDYMPSMKRFRK	NS5	3367	3381	HLA-DRB1*14:02	100%	Dengue virus 3	ELISPOT	IFNg release	Positive
738759	EFLDYMPSMKRFRKE	NS5	3368	3382	HLA-DRB1*14:06	100%	Dengue virus 3	ELISPOT	IFNg release	Positive
738829	ERELHKLGKCGSCVY	NS5	2927	2941	HLA-DRB1*11:04	100%	Dengue virus 3	ELISPOT	IFNg release	Positive
857703	IWYMWLGARYLEFEA	NS5	2963	2977	HLA-DPB1*04:01	100%	Dengue virus 3	ELISPOT	IFNg release	Positive
866168	ARYLEFEALGFLNED	NS5	2970	2984	HLA-DQA1*03:01/DQB1*03:02	100%	Dengue virus 3	ELISPOT	IFNg release	Positive
867015	GSCVYNMMGKREKKL	NS5	2937	2951	HLA-DRB5*01:01	100%	Dengue virus 3	ELISPOT	IFNg release	Positive
868748	SAKLQWFVERNMVIP	NS5	2549	2563	HLA-DQA1*01:01/DQB1*05:01	100%	Dengue virus 3	ELISPOT	IFNg release	Positive
869705	WYMWLGARYLEFEAL	NS5	2964	2978	HLA-DQA1*05:01/DQB1*02:01	100%	Dengue virus 3	ELISPOT	IFNg release	Positive
1068391	TGKPSINMLKRVRNR	C	8	22	HLA-DRB1*11:01	81% - <100%	Dengue virus 3	ELISPOT	IFNg release	Positive-Low
195536	ISNMLSIINKRKKTS	C	41	55	HLA-DRB1*11:01	81% - <100%	Dengue virus 3	ELISPOT	IFNg release	Positive
195559	KEISNMLSIINKRKK	C	39	53	HLA-DRB1*04:01	81% - <100%	Dengue virus 3	ELISPOT	IFNg release	Positive
539502	KPSINMLKRVRNRVS	C	10	24	HLA-DRB1*08:03	81% - <100%	Dengue virus 3	ELISPOT	IFNg release	Positive
539926	SNMLSIINKRKKTSL	C	42	56	HLA-DRB1*12:02	81% - <100%	Dengue virus 3	ELISPOT	IFNg release	Positive
119134	EGKVVQYENLKYTVI	E	126	140	HLA-DR2	81% - <100%	Dengue virus 3	ELISPOT	IFNg release	Positive
119327	RKELLVTFKNAHAKK	E	231	245	HLA-DR2	81% - <100%	Dengue virus 3	ELISPOT	IFNg release	Positive
195252	ELLVTFKNAHAKKQE	E	513	527	HLA-DRB1*04:01	81% - <100%	Dengue virus 3	ELISPOT	IFNg release	Positive
195341	FVLKKEVSETQHGTI	E	584	598	HLA-DRB1*04:01	81% - <100%	Dengue virus 3	ELISPOT	IFNg release	Positive
195365	GHLKCRLKMDKLELK	E	559	573	HLA-DRB1*03:01	81% - <100%	Dengue virus 3	ELISPOT	IFNg release	Positive
195813	NRKELLVTFKNAHAK	E	510	524	HLA-DRB1*04:01	81% - <100%	Dengue virus 3	ELISPOT	IFNg release	Positive
196033	TFVLKKEVSETQHGT	E	583	597	HLA-DRB1*04:01	81% - <100%	Dengue virus 3	ELISPOT	IFNg release	Positive
196172	VQYENLKYTVIITVH	E	410	424	HLA-DRB1*07:01	81% - <100%	Dengue virus 3	ELISPOT	IFNg release	Positive
539400	IEGKVVQYENLKYTV	E	405	419	HLA-DRB1*15:02	81% - <100%	Dengue virus 3	ELISPOT	IFNg release	Positive
539460	KELLVTFKNAHAKKQ	E	512	526	HLA-DRB1*08:03	81% - <100%	Dengue virus 3	ELISPOT	IFNg release	Positive
539559	LGKMVHQIFGSAYTA	E	710	724	HLA-DRB1*04:03	81% - <100%	Dengue virus 3	ELISPOT	IFNg release	Positive
739011	GGVLNSLGKMVHQIF	E	704	718	HLA-DRB1*11:04	81% - <100%	Dengue virus 3	ELISPOT	IFNg release	Positive
739041	GMSYAMCTNTFVLKK	E	574	588	HLA-DRB1*04:07	81% - <100%	Dengue virus 3	ELISPOT	IFNg release	Positive
739344	KMVHQIFGSAYTALF	E	31	45	HLA-DRB3*02:02	81% - <100%	Dengue virus 3	ELISPOT	IFNg release	Positive
740409	SVGGVLNSLGKMVHQ	E	702	716	HLA-DRB1*11:04	81% - <100%	Dengue virus 3	ELISPOT	IFNg release	Positive
866574	EIQNSGGTSIFAGHL	E	547	561	HLA-DQA1*01:02/DQB1*06:02	81% - <100%	Dengue virus 3	ELISPOT	IFNg release	Positive
867377	KCRLKMDKLELKGMS	E	562	576	HLA-DRB3*02:02	81% - <100%	Dengue virus 3	ELISPOT	IFNg release	Positive
868175	MVHRQWFFDLPLPWT	E	485	499	HLA-DQA1*01:01/DQB1*05:01	81% - <100%	Dengue virus 3	ELISPOT	IFNg release	Positive
190705	AKIVTAETQNSSFII	NS1	894	908	HLA-DRB1*04:01	81% - <100%	Dengue virus 3	ELISPOT	IFNg release	Positive
190823	KLTVVVGDIIGVLEQ	NS1	85	99	HLA-DR2	81% - <100%	Dengue virus 3	ELISPOT	IFNg release	Positive
190825	KQIANELNYILWENN	NS1	69	83	HLA-DR2	81% - <100%	Dengue virus 3	ELISPOT	IFNg release	Positive
195164	ANELNYILWENNIKL	NS1	845	859	HLA-DRB1*03:01	81% - <100%	Dengue virus 3	ELISPOT	IFNg release	Positive
195692	LNYILWENNIKLTVV	NS1	848	862	HLA-DRB1*03:01	81% - <100%	Dengue virus 3	ELISPOT	IFNg release	Positive
195782	NELNYILWENNIKLT	NS1	846	860	HLA-DRB1*03:01	81% - <100%	Dengue virus 3	ELISPOT	IFNg release	Positive
167810	RENLLLGVGLAMATTLQLPE	NS2a	1224	1243	HLA-DP	81% - <100%	Dengue virus 3	ELISPOT	IFNg release	Positive
195123	AGVFFTFVLLLSGQI	NS2a	1159	1173	HLA-DRB1*07:01	81% - <100%	Dengue virus 3	ELISPOT	IFNg release	Positive
195197	AWRTATLILAGVSLL	NS2a	1289	1303	HLA-DRB1*01:01	81% - <100%	Dengue virus 3	ELISPOT	IFNg release	Positive
195335	FTFVLLLSGQITWRD	NS2a	1163	1177	HLA-DRB1*15:02	81% - <100%	Dengue virus 3	ELISPOT	IFNg release	Positive
195429	GVFFTFVLLLSGQIT	NS2a	1160	1174	HLA-DRB1*04:01	81% - <100%	Dengue virus 3	ELISPOT	IFNg release	Positive
195457	HMIAGVFFTFVLLLS	NS2a	1156	1170	HLA-DRB1*11:01	81% - <100%	Dengue virus 3	ELISPOT	IFNg release	Positive
195462	HTLIMIGSNASDRMG	NS2a	1180	1194	HLA-DRB1*04:01	81% - <100%	Dengue virus 3	ELISPOT	IFNg release	Positive
195644	LIMIGSNASDRMGMG	NS2a	1182	1196	HLA-DRB1*04:01	81% - <100%	Dengue virus 3	ELISPOT	IFNg release	Positive
195729	LTVAWRTATLILAGV	NS2a	1286	1300	HLA-DPB1*04:02	81% - <100%	Dengue virus 3	ELISPOT	IFNg release	Positive
195757	MGVTYLALIATFKIQ	NS2a	1195	1209	HLA-DRB1*07:01	81% - <100%	Dengue virus 3	ELISPOT	IFNg release	Positive
195836	PLFIFSLKDTLKRRS	NS2a	1330	1344	HLA-DRB1*11:01	81% - <100%	Dengue virus 3	ELISPOT	IFNg release	Positive
195912	RTATLILAGVSLLPV	NS2a	1291	1305	HLA-DRB1*03:01	81% - <100%	Dengue virus 3	ELISPOT	IFNg release	Positive
539370	GVTYLALIATFKIQP	NS2a	1196	1210	HLA-DRB1*10:01	81% - <100%	Dengue virus 3	ELISPOT	IFNg release	Positive
540093	VTYLALIATFKIQPF	NS2a	1197	1211	HLA-DRB1*04:03	81% - <100%	Dengue virus 3	ELISPOT	IFNg release	Positive
867116	IAGVFFTFVLLLSGQ	NS2a	1158	1172	HLA-DPB1*01:01	81% - <100%	Dengue virus 3	ELISPOT	IFNg release	Positive
867442	KLITQFETYQLWTAL	NS2a	1260	1274	HLA-DPB1*02:01	81% - <100%	Dengue virus 3	ELISPOT	IFNg release	Positive
868329	PLPLFIFSLKDTLKR	NS2a	1328	1342	HLA-DPB1*01:01	81% - <100%	Dengue virus 3	ELISPOT	IFNg release	Positive
869298	TQFETYQLWTALISL	NS2a	1263	1277	HLA-DPB1*04:02	81% - <100%	Dengue virus 3	ELISPOT	IFNg release	Positive
195789	NILTVLLKTALLIVS	NS2b	1436	1450	HLA-DRB1*01:01	81% - <100%	Dengue virus 3	ELISPOT	IFNg release	Positive

190307	REIVDLMCHATF	NS3	255	266	HLA-DPA1*01:03/DPB1*02:01	81% - <100%	Dengue virus 3	ELISA	IL-2 release	Positive	1	1	1	1	1	1	1	1	1	1	1	1	1	1	1	1	1	1	1	1	1	1	1	1	1	1	1	0	1	1	1	1	1	1	1	1	1	1	1	1	1	1	1	1	1	1	1	1	1	0	0	0
190732	EAAAIFMTATPPGTA	NS3	1782	1796	HLA-DRB1*04:03	81% - <100%	Dengue virus 3	ELISPOT	IFNg release	Positive	1	1	1	1	0	1	1	1	1	1	1	1	1	1	1	1	1	1	1	1	1	1	1	1	1	1	1	1	1	1	1	1	1	1	1	1	1	1	1	1	1	1	1	1	1	1	1	1	1	0	0	0
190774	GKTVWFVPSIKAGND	NS3	357	371	HLA-DR2	81% - <100%	Dengue virus 3	ELISPOT	IFNg release	Positive	1	1	1	1	1	1	1	1	1	1	1	1	1	1	1	1	1	1	1	1	0	1	1	1	1	1	1	1	1	1	1	1	1	1	1	1	1	1	1	1	1	1	1	1	1	1	1	1	1	0	0	0
195297	FHTMWHVTRGAVLTY	NS3	1519	1533	HLA-DRB1*07:01	81% - <100%	Dengue virus 3	ELISPOT	IFNg release	Positive	1	1	1	1	1	1	1	1	1	1	1	1	1	1	1	1	1	1	1	1	1	1	1	1	1	1	1	1	1	1	1	1	1	1	1	1	1	1	1	1	1	0	1	1	1	0	0	0	0	0	0
28422	IRYQTTATK	NS3	241	249	HLA-DR15	81% - <100%	Dengue virus 3	51 chromium	cytotoxicity	Positive	1	1	0	1	1	1	1	0	1	1	0	1	1	1	1	1	1	1	1	1	1	1	1	1	1	1	1	1	1	1	1	1	1	1	1	1	1	1	1	1	1	1	1	1	1	1	1	1	1	0	0	0
39	AAAIFMTATPPGTAD	NS3	1783	1797	HLA-DRB1*04:03	81% - <100%	Dengue virus 3	ELISPOT	IFNg release	Positive	1	1	1	1	0	1	1	1	1	1	1	1	1	1	1	1	1	1	1	1	1	1	1	1	1	1	1	1	1	1	1	1	1	1	1	1	1	1	1	1	1	1	1	1	1	1	1	1	1	0	0	0
539028	AAIFMTATPPGTADA	NS3	1784	1798	HLA-DRB1*10:01	81% - <100%	Dengue virus 3	ELISPOT	IFNg release	Positive	1	1	1	1	0	1	1	1	1	1	1	1	1	1	1	1	1	1	1	1	1	1	1	1	1	1	1	1	1	1	1	1	1	1	1	1	1	1	1	1	1	1	1	1	1	1	1	1	1	0	0	0
539052	AGKTVWFVPSIKAGN	NS3	1829	1843	HLA-DRB1*08:03	81% - <100%	Dengue virus 3	ELISPOT	IFNg release	Positive	1	1	1	0	1	1	1	1	1	1	1	1	1	1	1	1	1	1	1	1	1	1	1	1	1	1	1	1	1	0	1	0	1	0	0	1	1	1	1	1	0	0	1	1	1	1	0	1	0	0	0
539578	LKGLPIRYQTTATKS	NS3	1709	1723	HLA-DRB1*13:01	81% - <100%	Dengue virus 3	ELISPOT	IFNg release	Positive	1	1	0	1	1	1	1	0	1	1	0	1	1	1	1	1	1	1	1	1	1	1	1	1	1	1	1	1	1	1	1	1	1	1	1	1	1	1	1	1	1	1	1	1	1	1	1	1	1	0	0	0
539607	LPIRYQTTATKSEHT	NS3	1712	1726	HLA-DRB1*13:01	81% - <100%	Dengue virus 3	ELISPOT	IFNg release	Positive	1	1	0	1	1	1	1	0	1	1	0	1	1	1	1	1	1	1	1	1	1	1	1	1	1	1	1	1	1	1	1	1	1	1	1	1	1	1	1	1	1	1	1	1	1	1	1	1	1	0	0	0
68015	VDLMCHATFT	NS3	258	267	HLA-DPw2	81% - <100%	Dengue virus 3	51 chromium	cytotoxicity	Positive	1	1	1	1	1	1	1	1	1	1	1	1	1	1	1	1	1	1	1	1	1	1	1	1	1	1	1	0	1	1	1	1	1	1	1	1	1	1	1	1	1	1	1	1	1	1	1	1	1	0	0	0
867106	HTMWHVTRGAVLTYN	NS3	1520	1534	HLA-DQA1*05:01/DQB1*02:01	81% - <100%	Dengue virus 3	ELISPOT	IFNg release	Positive	1	1	1	1	1	1	1	1	1	1	1	1	1	1	1	1	1	1	1	1	1	1	1	1	1	1	1	1	1	1	1	1	1	1	1	1	1	1	1	1	1	0	1	1	1	0	0	0	0	0	0
869689	WHVTRGAVLTYNGKR	NS3	1523	1537	HLA-DQA1*05:01/DQB1*02:01	81% - <100%	Dengue virus 3	ELISPOT	IFNg release	Positive	1	1	1	1	1	1	1	1	1	1	1	1	1	1	1	1	1	1	1	1	1	1	1	1	1	1	1	1	1	1	1	1	1	1	1	1	1	1	1	1	1	0	1	1	1	0	0	0	0	0	0
167755	IALDLVTEIGRVPSHLAHRT	NS4a	2	21	HLA class II	81% - <100%	Dengue virus 3	ELISPOT	IFNg release	Positive	1	1	1	0	1	1	1	1	1	1	1	1	1	1	1	1	1	1	1	1	1	1	1	1	1	1	1	1	1	1	1	1	1	1	1	1	1	1	1	1	1	1	1	1	1	1	1	1	1	0	0	0
195522	IPLQWIASAIVLEFF	NS4a	2192	2206	HLA-DRB1*01:01	81% - <100%	Dengue virus 3	ELISPOT	IFNg release	Positive	1	1	0	1	1	1	1	0	1	1	1	1	1	1	1	1	1	1	1	1	1	1	1	1	1	1	1	1	1	1	1	1	1	1	1	1	1	1	1	1	1	0	1	1	1	0	0	0	0	0	0
739464	LFLISGKGIGKTSIG	NS4a	2162	2176	HLA-DRB1*01:02	81% - <100%	Dengue virus 3	ELISPOT	IFNg release	Positive	1	1	1	1	1	1	1	1	0	1	1	1	1	1	1	1	1	1	1	1	1	1	1	1	1	1	1	1	1	1	1	1	1	1	1	1	1	1	1	1	1	1	1	1	1	1	1	1	1	0	0	0
869474	VIGILTLAAIIAANE	NS4a	2230	2244	HLA-DQA1*01:02/DQB1*06:02	81% - <100%	Dengue virus 3	ELISPOT	IFNg release	Positive	1	1	1	1	1	1	1	1	1	1	1	1	1	1	1	1	1	1	1	1	1	1	1	1	1	1	1	1	1	1	1	1	1	1	1	1	1	1	1	1	1	0	1	1	1	0	0	0	0	0	0
195617	LCAVQLLLMRTSWAL	NS4b	2418	2432	HLA-DRB1*15:01	81% - <100%	Dengue virus 3	ELISPOT	IFNg release	Positive	1	1	1	1	1	1	1	1	1	1	1	1	0	1	1	1	1	1	1	0	1	1	1	1	1	1	1	1	1	1	1	1	1	1	1	1	1	1	1	1	1	1	1	1	1	0	1	0	0	0	0
195624	LDPVIYDSKFEKQLG	NS4b	2397	2411	HLA-DRB1*03:01	81% - <100%	Dengue virus 3	ELISPOT	IFNg release	Positive	1	1	1	1	1	1	1	1	1	1	1	1	1	1	1	1	1	1	1	1	1	1	1	1	1	1	1	1	1	1	1	1	1	1	1	1	1	1	1	1	1	1	0	1	1	1	1	1	0	0	0
195663	LLLITHYAIIGPGLQ	NS4b	2354	2368	HLA-DRB1*07:01	81% - <100%	Dengue virus 3	ELISPOT	IFNg release	Positive	1	1	1	1	0	1	1	1	1	1	1	1	1	1	1	1	1	1	1	1	1	1	1	1	1	1	1	1	1	1	1	1	1	1	1	1	1	1	1	1	1	0	1	1	1	0	0	0	0	0	0
195664	LLLMRTSWALCEALT	NS4b	2423	2437	HLA-DRB1*04:01	81% - <100%	Dengue virus 3	ELISPOT	IFNg release	Positive	1	1	1	1	1	1	1	1	1	1	1	1	1	1	1	1	1	1	1	1	1	1	1	1	1	1	1	1	1	1	1	1	1	1	1	1	1	1	1	1	1	0	1	1	1	0	1	0	0	0	0
195807	NPLTLTAAVLLLITH	NS4b	2345	2359	HLA-DRB1*07:01	81% - <100%	Dengue virus 3	ELISPOT	IFNg release	Positive	1	1	1	1	0	1	1	1	1	1	1	1	1	1	1	1	1	1	1	1	1	1	1	1	1	1	1	1	1	1	1	1	1	1	1	1	1	1	1	1	1	0	1	1	1	0	0	0	0	0	0
195871	QVMLLVLCAVQLLLM	NS4b	2412	2426	HLA-DRB1*01:01	81% - <100%	Dengue virus 3	ELISPOT	IFNg release	Positive	1	1	1	1	1	1	1	1	1	1	1	1	0	1	1	1	1	1	1	0	1	1	1	1	1	1	1	1	1	1	1	1	1	1	1	1	1	1	1	1	1	1	1	1	1	1	1	1	0	0	0
196023	TAAVLLLITHYAIIG	NS4b	2350	2364	HLA-DRB1*15:01	81% - <100%	Dengue virus 3	ELISPOT	IFNg release	Positive	1	1	1	1	0	1	1	1	1	1	1	1	1	1	1	1	1	1	1	1	1	1	1	1	1	1	1	1	1	1	1	1	1	1	1	1	1	1	1	1	1	0	1	1	1	0	0	0	0	0	0
196137	VLCAVQLLLMRTSWA	NS4b	2417	2431	HLA-DRB1*12:02	81% - <100%	Dengue virus 3	ELISPOT	IFNg release	Positive	1	1	1	1	1	1	1	1	1	1	1	1	0	1	1	1	1	1	1	0	1	1	1	1	1	1	1	1	1	1	1	1	1	1	1	1	1	1	1	1	1	1	1	1	1	1	1	1	0	0	0
196155	VMLLVLCAVQLLLMR	NS4b	2413	2427	HLA-DRB1*03:01	81% - <100%	Dengue virus 3	ELISPOT	IFNg release	Positive	1	1	1	1	1	1	1	1	1	1	1	1	0	1	1	1	1	1	1	0	1	1	1	1	1	1	1	1	1	1	1	1	1	1	1	1	1	1	1	1	1	1	1	1	1	1	1	1	0	0	0
196163	VNPLTLTAAVLLLIT	NS4b	2344	2358	HLA-DRB1*01:01	81% - <100%	Dengue virus 3	ELISPOT	IFNg release	Positive	1	1	1	1	0	1	1	1	1	1	1	1	1	1	1	1	1	1	1	1	1	1	1	1	1	1	1	1	1	1	1	1	1	1	1	1	1	1	1	1	1	0	1	1	1	0	0	0	0	0	0
167753	HVNAEPETPNMDVIGERIKR	NS5	263	282	HLA class II	81% - <100%	Dengue virus 3	ELISPOT	IFNg release	Positive	1	1	0	1	1	1	1	1	1	1	1	1	1	1	1	1	1	1	1	1	1	1	1	1	1	1	1	1	1	1	1	1	1	1	1	1	1	1	1	1	1	1	1	1	1	0	1	0	0	0	0
167769	KKLNQLSRKEFDL	NS5	15	27	HLA-DP	81% - <100%	Dengue virus 3	ELISPOT	IFNg release	Positive	1	1	0	1	1	1	1	0	1	1	1	1	1	1	1	1	1	1	1	1	1	1	1	1	1	1	1	1	0	1	1	1	1	1	1	1	1	1	1	1	1	1	1	1	1	0	1	0	0	0	0
195204	CLGKAYAQMWSLMYF	NS5	3243	3257	HLA-DRB1*04:01	81% - <100%	Dengue virus 3	ELISPOT	IFNg release	Positive	1	1	1	1	1	1	1	1	1	1	1	1	1	1	1	1	1	1	1	1	1	1	1	1	1	1	1	1	1	1	1	1	1	1	1	1	1	1	1	1	1	0	1	1	1	0	1	0	0	0	0
195408	GNIVASVNMVSRLLL	NS5	3	17	HLA-DRB1*03:01	81% - <100%	Dengue virus 3	ELISPOT	IFNg release	Positive	1	1	1	1	1	1	1	1	1	1	1	1	1	1	1	1	1	1	1	1	1	1	1	1	1	0	1	1	1	1	1	1	1	1	1	1	0	0	1	1	1	0	1	1	1	0	0	0	0	0	0
195668	LLNRFTMTHRRPTIE	NS5	16	30	HLA-DRB1*11:01	81% - <100%	Dengue virus 3	ELISPOT	IFNg release	Positive	1	1	1	1	1	1	1	1	1	1	1	1	1	1	1	1	1	1	0	1	1	1	1	1	1	0	1	1	1	1	1	1	1	1	1	1	0	0	1	1	1	1	1	1	1	1	1	1	0	0	0
195806	NMVSRLLLNRFTMTH	NS5	2722	2736	HLA-DRB1*08:02	81% - <100%	Dengue virus 3	ELISPOT	IFNg release	Positive	1	1	1	1	1	1	1	1	1	1	1	1	1	1	1	1	1	1	0	1	1	1	1	1	1	0	1	1	1	1	1	1	1	1	1	1	0	0	1	1	1	1	1	1	1	1	1	1	0	0	0
195812	NRFTMTHRRPTIEKD	NS5	18	32	HLA-DRB1*11:01	81% - <100%	Dengue virus 3	ELISPOT	IFNg release	Positive	1	1	1	1	1	1	1	1	1	1	1	1	1	1	1	1	1	1	0	1	1	1	1	1	1	0	1	1	1	1	1	1	1	1	1	1	0	0	1	1	1	1	1	1	1	1	1	1	0	0	0
196038	TGNIVASVNMVSRLL	NS5	2	16	HLA-DRB1*04:01	81% - <100%	Dengue virus 3	ELISPOT	IFNg release	Positive	1	1	1	1	1	1	1	1	1	1	1	1	1	1	1	1	1	1	1	1	1	1	1	1	1	0	1	1	1	1	1	1	1	1	1	1	0	0	1	1	1	0	1	1	1	0	0	0	0	0	0
196162	VNMVSRLLLNRFTMT	NS5	9	23	HLA-DRB1*11:01	81% - <100%	Dengue virus 3	ELISPOT	IFNg release	Positive	1	1	1	1	1	1	1	1	1	1	1	1	1	1	1	1	1	1	0	1	1	1	1	1	1	0	1	1	1	1	1	1	1	1	1	1	0	0	1	1	1	1	1	1	1	1	1	1	0	0	0
196221	WLWRTLGRNKKPRLC	NS5	157	171	HLA-DRB1*14:02	81% - <100%	Dengue virus 3	ELISPOT	IFNg release	Positive	1	1	1	0	1	1	1	1	1	1	1	1	1	1	1	1	1	1	0	0	1	1	1	1	1	1	1	1	1	1	1	1	1	1	1	1	1	1	1	1	1	0	1	1	1	0	0	0	0	0	0
196222	WNIVKLMSGKDVFYL	NS5	2611	2625	HLA-DRB1*15:01	81% - <100%	Dengue virus 3	ELISPOT	IFNg release	Positive	1	1	1	1	1	1	1	0	1	1	1	1	1	0	1	1	1	1	1	1	1	1	1	1	1	1	1	1	1	1	1	1	1	1	1	1	1	1	1	1	1	1	1	1	0	1	1	1	0	0	0
196224	WSLMYFHRRDLRLAS	NS5	3252	3266	HLA-DRB1*03:01	81% - <100%	Dengue virus 3	ELISPOT	IFNg release	Positive	1	1	1	1	1	1	1	1	1	1	1	1	1	1	1	1	1	1	1	1	1	1	1	1	1	1	1	1	1	1	1	1	1	1	1	1	1	1	1	1	1	0	1	1	1	0	1	0	0	0	0
539444	IVASVNMVSRLLLNR	NS5	2717	2731	HLA-DRB1*12:02	81% - <100%	Dengue virus 3	ELISPOT	IFNg release	Positive	1	1	1	1	1	1	1	1	1	1	1	1	1	1	1	1	1	1	1	1	1	1	1	1	1	0	1	1	1	1	1	1	1	1	1	1	0	0	1	1	1	0	1	1	1	0	0	0	0	0	0
539726	NIVASVNMVSRLLLN	NS5	2716	2730	HLA-DRB1*15:06	81% - <100%	Dengue virus 3	ELISPOT	IFNg release	Positive	1	1	1	1	1	1	1	1	1	1	1	1	1	1	1	1	1	1	1	1	1	1	1	1	1	0	1	1	1	1	1	1	1	1	1	1	0	0	1	1	1	0	1	1	1	0	0	0	0	0	0
539831	RATWAQNIPTAIQQV	NS5	3346	3360	HLA-DRB1*09:01	81% - <100%	Dengue virus 3	ELISPOT	IFNg release	Positive	1	1	1	1	1	1	1	1	1	1	1	1	1	1	1	1	1	1	1	1	1	1	1	1	1	1	1	1	1	1	1	1	1	1	1	1	1	1	1	1	1	0	1	1	1	0	0	0	0	0	0
539916	SLMYFHRRDLRLASN	NS5	3253	3267	HLA-DRB1*13:01	81% - <100%	Dengue virus 3	ELISPOT	IFNg release	Positive	1	1	1	1	1	1	1	1	1	1	1	1	1	1	1	1	1	1	1	1	1	1	1	1	1	1	1	1	1	1	1	1	1	1	1	1	1	1	1	1	1	0	1	1	1	0	1	0	0	0	0
539945	SVNMVSRLLLNRFTM	NS5	2720	2734	HLA-DRB1*12:02	81% - <100%	Dengue virus 3	ELISPOT	IFNg release	Positive	1	1	1	1	1	1	1	1	1	1	1	1	1	1	1	1	1	1	1	1	1	1	1	1	1	0	1	1	1	1	1	1	1	1	1	1	0	0	1	1	1	1	1	1	1	1	1	1	0	0	0
540025	VASVNMVSRLLLNRF	NS5	2718	2732	HLA-DRB1*14:04	81% - <100%	Dengue virus 3	ELISPOT	IFNg release	Positive	1	1	1	1	1	1	1	1	1	1	1	1	1	1	1	1	1	1	1	1	1	1	1	1	1	0	1	1	1	1	1	1	1	1	1	1	0	0	1	1	1	0	1	1	1	0	0	0	0	0	0
738537	ASVNMVSRLLLNRFT	NS5	7	21	HLA-DRB1*14:06	81% - <100%	Dengue virus 3	ELISPOT	IFNg release	Positive	1	1	1	1	1	1	1	1	1	1	1	1	1	1	1	1	1	1	1	1	1	1	1	1	1	0	1	1	1	1	1	1	1	1	1	1	0	0	1	1	1	0	1	1	1	0	0	0	0	0	0
867376	KAYAQMWSLMYFHRR	NS5	3246	3260	HLA-DRB5*01:01	81% - <100%	Dengue virus 3	ELISPOT	IFNg release	Positive	1	1	1	1	1	1	1	1	1	1	1	1	1	1	1	1	1	1	1	1	1	1	1	1	1	1	1	1	1	1	1	1	1	1	1	1	1	1	1	1	1	0	1	1	1	0	1	0	0	0	0
868338	PMSTYGWNIVKLMSG	NS5	2605	2619	HLA-DQA1*01:01/DQB1*05:01	81% - <100%	Dengue virus 3	ELISPOT	IFNg release	Positive	1	1	1	1	1	1	1	0	1	1	1	1	1	0	1	1	1	1	1	1	1	1	1	1	1	1	1	0	1	1	1	1	1	1	1	1	1	1	1	1	1	1	1	1	0	1	1	1	0	0	0
869489	VKPIDDRFANALLAL	NS5	3157	3171	HLA-DPB1*01:01	81% - <100%	Dengue virus 3	ELISPOT	IFNg release	Positive	1	1	1	1	1	1	1	1	1	0	1	1	1	1	1	1	1	1	1	1	1	1	1	0	1	1	1	1	1	1	1	1	1	1	1	1	1	1	1	1	1	1	1	1	0	1	1	1	0	0	0
195597	KTSLCLMMILPAALA	C	53	67	HLA-DRB1*01:01	51%-80%	Dengue virus 3	ELISPOT	IFNg release	Positive	0	1	0	0	0	0	1	0	1	1	0	0	1	1	1	0	0	0	1	1	1	0	0	1	1	1	0	1	0	1	0	0	1	0	0	1	1	1	1	1	0	0	1	1	0	0	0	0	0	0	0
119289	NGRLITANPVVTKKE	E	626	640	HLA-DRB1*08:02	51%-80%	Dengue virus 3	ELISPOT	IFNg release	Positive	1	1	1	0	1	1	1	1	1	1	1	1	1	0	1	1	1	1	1	1	0	1	1	1	1	1	1	1	1	1	1	0	0	1	1	0	1	1	1	0	0	1	1	1	0	1	1	1	1	1	1
195416	GRLITANPVVTKKEE	E	627	641	HLA-DRB1*08:02	51%-80%	Dengue virus 3	ELISPOT	IFNg release	Positive	1	1	1	0	1	1	1	1	1	1	1	1	1	0	1	1	1	1	1	1	0	1	1	1	1	1	1	1	1	1	1	0	0	1	1	0	1	1	1	0	0	1	1	1	0	1	1	1	1	1	1
195134	AHTLIMIGSNASDRM	NS2a	1179	1193	HLA-DRB1*04:01	51%-80%	Dengue virus 3	ELISPOT	IFNg release	Positive	0	1	0	0	1	0	1	0	1	0	0	0	1	1	1	1	1	1	0	1	1	0	0	1	1	1	1	1	0	1	0	0	1	0	0	1	1	1	1	1	0	0	0	1	1	1	1	1	0	0	0
195222	DMAHTLIMIGSNASD	NS2a	1177	1191	HLA-DRB1*04:01	51%-80%	Dengue virus 3	ELISPOT	IFNg release	Positive	0	1	0	0	1	0	1	0	1	0	0	0	1	1	1	1	1	1	0	1	1	0	0	1	1	1	1	1	0	1	0	0	1	0	0	1	1	1	1	1	0	0	0	1	1	1	1	1	0	0	0
195342	FVLLLSGQITWRDMA	NS2a	1165	1179	HLA-DRB1*01:01	51%-80%	Dengue virus 3	ELISPOT	IFNg release	Positive	0	1	0	0	1	0	1	0	1	0	0	0	1	1	1	1	1	1	1	1	1	0	0	1	1	0	1	1	0	1	0	0	1	0	0	1	1	1	1	1	0	1	1	1	1	1	0	1	0	0	0
195743	MAHTLIMIGSNASDR	NS2a	1178	1192	HLA-DRB1*04:01	51%-80%	Dengue virus 3	ELISPOT	IFNg release	Positive	0	1	0	0	1	0	1	0	1	0	0	0	1	1	1	1	1	1	0	1	1	0	0	1	1	1	1	1	0	1	0	0	1	0	0	1	1	1	1	1	0	0	0	1	1	1	1	1	0	0	0
195144	AIVREAIKRRLRTLI	NS3	1680	1694	HLA-DRB1*08:02	51%-80%	Dengue virus 3	ELISPOT	IFNg release	Positive	0	1	0	0	1	0	1	0	1	0	0	0	1	1	1	1	1	1	1	1	1	0	0	1	1	1	1	1	0	0	0	0	1	0	0	1	1	1	1	1	0	1	1	1	1	1	1	1	0	0	0
38401	LPAIVREAIKRRLRT	NS3	1678	1692	HLA-DRB3*02:02	51%-80%	Dengue virus 3	ELISPOT	IFNg release	Positive	0	1	0	0	1	0	1	0	1	0	0	0	1	1	1	1	1	1	1	1	1	0	0	1	1	1	1	1	0	0	0	0	1	0	0	1	1	1	1	1	0	1	1	1	1	1	1	1	0	0	0
54455	RKYLPAIVRE	NS3	202	211	HLA-DR15	51%-80%	Dengue virus 3	51 chromium	cytotoxicity	Positive	0	1	0	0	1	0	1	0	1	0	0	0	1	1	1	1	1	1	1	1	1	0	0	1	1	1	1	1	0	0	0	0	1	0	0	1	1	1	1	1	0	1	1	1	1	1	1	1	0	0	0
70708	VREAIKRRLRTLILA	NS3	1682	1696	HLA-DRB1*12:02	51%-80%	Dengue virus 3	ELISPOT	IFNg release	Positive	0	1	0	0	1	0	1	0	1	0	0	0	1	1	1	1	1	1	1	1	1	0	0	1	1	1	1	1	0	0	0	0	1	0	0	1	1	1	1	1	0	1	1	1	1	1	1	1	0	0	0
867358	IVREAIKRRLRTLIL	NS3	1681	1695	HLA-DRB3*02:02	51%-80%	Dengue virus 3	ELISPOT	IFNg release	Positive	0	1	0	0	1	0	1	0	1	0	0	0	1	1	1	1	1	1	1	1	1	0	0	1	1	1	1	1	0	0	0	0	1	0	0	1	1	1	1	1	0	1	1	1	1	1	1	1	0	0	0
867534	KYLPAIVREAIKRRL	NS3	1676	1690	HLA-DRB4*01:01	51%-80%	Dengue virus 3	ELISPOT	IFNg release	Positive	0	1	0	0	1	0	1	0	1	0	0	0	1	1	1	1	1	1	1	1	1	0	0	1	1	1	1	1	0	0	0	0	1	0	0	1	1	1	1	1	0	1	1	1	1	1	1	1	0	0	0
195205	CLMMMLPATLAFHLT	C	104	118	HLA-DRB1*15:01	>0% - 50%	Dengue virus 3	ELISPOT	IFNg release	Positive	0	0	0	0	0	0	0	0	0	0	0	0	0	0	0	0	0	0	0	0	0	0	0	0	0	0	0	0	0	0	0	0	0	0	0	0	0	0	0	0	0	1	0	0	0	1	1	1	0	0	0
195598	KTSLCLVMILPAALA	C	100	114	HLA-DRB1*01:01	>0% - 50%	Dengue virus 3	ELISPOT	IFNg release	Positive	1	0	1	1	0	1	0	1	0	0	1	1	0	0	0	0	0	0	0	0	0	1	1	0	0	0	0	0	1	0	1	1	0	1	1	0	0	0	0	0	1	0	0	0	0	0	0	0	0	0	0
195967	SLCLMMMLPATLAFH	C	102	116	HLA-DRB1*08:02	>0% - 50%	Dengue virus 3	ELISPOT	IFNg release	Positive	0	0	0	0	0	0	0	0	0	0	0	0	0	0	0	0	0	0	0	0	0	0	0	0	0	0	0	0	0	0	0	0	0	0	0	0	0	0	0	0	0	1	0	0	0	1	1	1	0	0	0
868645	RGLLNGQGPMKLVMA	C	15	29	HLA-DRB3*02:02	>0% - 50%	Dengue virus 3	ELISPOT	IFNg release	Positive	0	0	0	0	0	0	0	0	0	0	0	0	0	0	0	0	0	0	0	0	0	0	0	0	0	0	0	0	0	0	0	0	0	0	0	0	0	0	0	0	0	1	0	0	0	1	1	1	0	0	0
196170	VQHENLKYTVIITVH	E	130	144	HLA-DRB1*07:01	>0% - 50%	Dengue virus 3	ELISPOT	IFNg release	Positive	0	0	0	0	0	0	0	0	0	0	0	0	0	0	0	0	0	0	0	0	0	0	0	0	0	0	0	0	0	0	0	0	1	1	0	1	0	0	0	1	0	1	0	0	0	1	1	1	1	1	1
869693	WMVHRQWLFDLPLPW	E	87	101	HLA-DQA1*01:01/DQB1*05:01	>0% - 50%	Dengue virus 3	ELISPOT	IFNg release	Positive	0	0	0	0	0	0	0	0	0	0	0	0	0	0	0	0	0	0	0	0	0	1	0	0	0	0	0	0	0	0	0	0	0	0	0	0	0	0	0	0	0	0	0	0	0	0	0	0	0	0	0
195577	KLTVVVGDTIGVLEQ	NS1	858	872	HLA-DRB1*03:01	>0% - 50%	Dengue virus 3	ELISPOT	IFNg release	Positive	0	0	0	0	0	0	0	0	0	0	0	0	0	0	0	0	0	0	0	0	1	0	0	0	0	0	0	0	0	0	0	0	0	0	0	0	0	0	0	0	0	0	0	0	0	0	0	0	0	0	0
195433	GVLFTFVLLLSGQIT	NS2a	1160	1174	HLA-DRB1*15:06	>0% - 50%	Dengue virus 3	ELISPOT	IFNg release	Positive	0	0	0	0	0	0	0	0	0	0	0	0	0	0	0	0	0	0	0	0	0	0	0	0	0	0	0	0	0	0	0	0	0	0	0	0	1	1	0	0	0	1	0	0	0	1	0	1	0	0	0
195481	IFTLTVAWRTATLIL	NS2a	1283	1297	HLA-DRB1*07:01	>0% - 50%	Dengue virus 3	ELISPOT	IFNg release	Positive	0	0	0	0	0	0	0	0	0	0	0	0	0	0	0	0	0	0	0	0	0	0	0	0	0	0	0	0	0	0	0	0	0	0	0	0	0	0	0	0	0	1	0	0	0	1	1	1	0	0	0
195630	LFTFVLLLSGQITWR	NS2a	1162	1176	HLA-DRB1*07:01	>0% - 50%	Dengue virus 3	ELISPOT	IFNg release	Positive	0	0	0	0	0	0	0	0	0	0	0	0	0	0	0	0	0	0	0	0	0	0	0	0	0	0	0	0	0	0	0	0	0	0	0	0	1	1	0	0	0	1	0	0	0	1	0	1	0	0	0
195740	LWTALVSLTCSNTIF	NS2a	1270	1284	HLA-DRB1*07:01	>0% - 50%	Dengue virus 3	ELISPOT	IFNg release	Positive	0	0	0	0	0	0	0	0	0	0	0	0	0	0	0	0	0	0	0	0	0	0	0	0	0	0	0	0	0	0	0	0	0	0	0	0	0	0	0	0	0	1	0	0	0	0	1	0	0	0	0
195819	NTIFTLTVAWRTATL	NS2a	1281	1295	HLA-DRB1*07:01	>0% - 50%	Dengue virus 3	ELISPOT	IFNg release	Positive	0	0	0	0	0	0	0	0	0	0	0	0	0	0	0	0	0	0	0	0	0	0	0	0	0	0	0	0	0	0	0	0	0	0	0	0	0	0	0	0	0	1	0	0	0	1	1	1	0	0	0
539802	QFETYQLWTALVSLT	NS2a	1264	1278	HLA-DRB1*15:02	>0% - 50%	Dengue virus 3	ELISPOT	IFNg release	Positive	0	0	0	0	0	0	0	0	0	0	0	0	0	0	0	0	0	0	0	0	0	0	0	0	0	0	0	0	0	0	0	0	0	0	0	0	0	0	0	0	0	1	0	0	0	0	1	0	0	0	0
540048	VLFTFVLLLSGQITW	NS2a	1161	1175	HLA-DRB1*09:01	>0% - 50%	Dengue virus 3	ELISPOT	IFNg release	Positive	0	0	0	0	0	0	0	0	0	0	0	0	0	0	0	0	0	0	0	0	0	0	0	0	0	0	0	0	0	0	0	0	0	0	0	0	1	1	0	0	0	1	0	0	0	1	0	1	0	0	0
540087	VSGIFPYSIPATLLV	NS2b	1449	1463	HLA-DRB1*09:01	>0% - 50%	Dengue virus 3	ELISPOT	IFNg release	Positive	0	0	0	0	0	0	0	0	0	0	0	0	0	0	0	0	0	0	0	0	0	0	0	0	0	0	0	0	0	0	0	0	0	0	0	0	0	0	0	0	0	1	0	0	0	1	1	1	0	0	0
21420	GNEWITDFVGKTVWF	NS3	31	45	HLA-DRB1*15:01	>0% - 50%	Dengue virus 3	ICS	IFNg release	Positive	0	0	0	1	0	0	0	0	0	0	0	0	0	0	0	0	0	0	0	0	0	0	0	0	0	0	0	0	0	0	0	1	0	1	1	0	0	0	0	0	1	1	0	0	0	0	1	0	0	0	0
539432	IREAIKRRLRTLILA	NS3	1682	1696	HLA-DRB1*08:03	>0% - 50%	Dengue virus 3	ELISPOT	IFNg release	Positive	1	0	1	1	0	1	0	1	0	1	1	1	0	0	0	0	0	0	0	0	0	1	1	0	0	0	0	0	1	1	1	1	0	1	1	0	0	0	0	0	1	0	0	0	0	0	0	0	0	0	0
539777	PKNFQTMPGIFQTTT	NS3	1579	1593	HLA-DRB1*04:03	>0% - 50%	Dengue virus 3	ELISPOT	IFNg release	Positive	0	0	0	0	0	0	0	0	0	0	0	0	0	0	0	0	0	0	0	0	0	0	0	0	0	0	0	0	0	0	0	0	0	0	0	0	0	0	0	0	0	1	0	0	0	1	1	1	0	0	0
540037	VGKTVWFVPSIKAGN	NS3	1829	1843	HLA-DRB1*08:03	>0% - 50%	Dengue virus 3	ELISPOT	IFNg release	Positive	0	0	0	1	0	0	0	0	0	0	0	0	0	0	0	0	0	0	0	0	0	0	0	0	0	0	0	0	0	0	0	1	0	1	1	0	0	0	0	0	1	1	0	0	0	0	1	0	0	0	0
72654	WITDFVGKTVW	NS3	1824	1834	HLA-DR15	>0% - 50%	Dengue virus 3	51 chromium	cytotoxicity	Positive	0	0	0	1	0	0	0	0	0	0	0	0	0	0	0	0	0	0	0	0	0	0	0	0	0	0	0	0	0	0	0	1	0	1	1	0	0	0	0	0	1	1	0	0	0	0	1	0	0	0	0
869278	TMWHVTRGAVLTHNG	NS3	1521	1535	HLA-DQA1*05:01/DQB1*02:01	>0% - 50%	Dengue virus 3	ELISPOT	IFNg release	Positive	0	0	0	0	0	0	0	0	0	0	0	0	0	0	0	0	0	0	0	0	0	0	0	0	0	0	0	0	0	0	0	0	0	0	0	0	0	0	0	0	0	1	0	0	0	1	1	1	0	0	0
195199	AYVVIGILTLAAIVA	NS4a	2227	2241	HLA-DRB1*01:01	>0% - 50%	Dengue virus 3	ELISPOT	IFNg release	Positive	0	0	0	0	0	0	0	0	0	0	0	0	0	0	0	0	0	0	0	0	0	0	0	0	0	0	0	0	0	0	0	0	0	0	0	0	0	0	0	0	0	1	0	0	0	1	1	1	0	0	0
195742	MADVPLQWIASAIVL	NS4a	2189	2203	HLA-DRB1*07:01	>0% - 50%	Dengue virus 3	ELISPOT	IFNg release	Positive	0	0	0	0	0	0	0	0	0	0	0	0	0	0	0	0	0	0	0	0	0	0	0	0	0	0	0	0	0	0	0	0	0	0	0	0	0	0	0	0	0	1	0	0	0	1	1	1	0	0	0
196168	VPLQWIASAIVLEFF	NS4a	2192	2206	HLA-DRB1*01:01	>0% - 50%	Dengue virus 3	ELISPOT	IFNg release	Positive	0	0	0	0	0	0	0	0	0	0	0	0	0	0	0	0	0	0	0	0	0	0	0	0	0	0	0	0	0	0	0	0	0	0	0	0	0	0	0	0	0	1	0	0	0	1	1	1	0	0	0
539900	SGMLWMADVPLQWIA	NS4a	2184	2198	HLA-DRB1*04:03	>0% - 50%	Dengue virus 3	ELISPOT	IFNg release	Positive	0	0	0	0	0	0	0	0	0	0	0	0	0	0	0	0	0	0	0	0	0	0	0	0	0	0	0	0	0	0	0	0	0	0	0	0	0	0	0	0	0	1	0	0	0	1	1	1	0	0	0
869526	VLLLVTHYAIIGPGL	NS4b	2353	2367	HLA-DPB1*02:01	>0% - 50%	Dengue virus 3	ELISPOT	IFNg release	Positive	0	0	0	0	0	0	0	0	0	0	0	0	0	0	0	0	0	0	0	0	0	0	0	0	0	0	0	0	0	0	0	0	0	0	0	0	0	0	0	0	0	1	0	0	0	1	0	1	0	0	0
195410	GNIVSSVNMVSRLLL	NS5	2715	2729	HLA-DRB1*03:01	>0% - 50%	Dengue virus 3	ELISPOT	IFNg release	Positive	0	0	0	0	0	0	0	0	0	0	0	0	0	0	0	0	0	0	0	0	0	0	0	0	0	0	0	0	0	0	0	0	0	0	0	0	0	0	0	0	0	1	0	0	0	1	1	1	0	0	0
196041	TGNIVSSVNMVSRLL	NS5	2714	2728	HLA-DRB1*04:01	>0% - 50%	Dengue virus 3	ELISPOT	IFNg release	Positive	0	0	0	0	0	0	0	0	0	0	0	0	0	0	0	0	0	0	0	0	0	0	0	0	0	0	0	0	0	0	0	0	0	0	0	0	0	0	0	0	0	1	0	0	0	1	1	1	0	0	0
196239	YQNKVVKVQRPTPKG	NS5	3062	3076	HLA-DRB1*08:02	>0% - 50%	Dengue virus 3	ELISPOT	IFNg release	Positive	0	0	0	0	0	0	0	0	0	0	0	0	0	0	0	0	0	0	0	0	0	0	0	0	0	0	0	0	0	0	0	0	0	0	0	0	0	0	0	0	0	1	0	0	0	1	1	1	0	0	0
539449	IVSSVNMVSRLLLNR	NS5	2717	2731	HLA-DRB1*11:04	>0% - 50%	Dengue virus 3	ELISPOT	IFNg release	Positive	0	0	0	0	0	0	0	0	0	0	0	0	0	0	0	0	0	0	0	0	0	0	0	0	0	0	0	0	0	0	0	0	0	0	0	0	0	0	0	0	0	1	0	0	0	1	1	1	0	0	0
540090	VSSVNMVSRLLLNRF	NS5	2718	2732	HLA-DRB1*14:04	>0% - 50%	Dengue virus 3	ELISPOT	IFNg release	Positive	0	0	0	0	0	0	0	0	0	0	0	0	0	0	0	0	0	0	0	0	0	0	0	0	0	0	0	0	0	0	0	0	0	0	0	0	0	0	0	0	0	1	0	0	0	1	1	1	0	0	0
591602	ACLGKAYAQMWTLMY	NS5	3242	3256	HLA-DRB1*04:01	>0% - 50%	Dengue virus 3	ELISPOT	IFNg release	Positive	0	0	0	0	0	0	0	0	0	0	0	0	0	0	0	0	0	0	0	0	0	0	0	0	0	0	0	0	0	0	0	0	0	0	0	0	0	0	0	0	0	1	0	0	0	1	0	1	0	0	0

CD4 DENV-4 Epitopes

											NCBI IDs of DENV-4 circulating strains from Brazil, (100% identity=1,green ; else 0, red)																																			DENV-4 constructs of vaccines					
Epitope_ID	AA_Seq	Protein_Name	Start	End	Allele	step	Serotype	Method/Technique	Assay Group	Qualitative Measure	AEW50183.1	AFX65867.1	AFX65875.1	AFX65870.1	AFX65878.1	AFX65880.1	AFX65879.1	AKQ00033.1	AKQ00035.1	AIQ84228.1	AIQ84240.1	AIQ84244.1	AIQ84242.1	AIQ84233.1	AIQ84223.1	AFX65872.1	AMP43484.1	AKQ00031.1	AIQ84245.1	AIQ84235.1	AIQ84238.1	AIQ84220.1	AIQ84226.1	AEX91754.1	AFX65881.1	AKQ00036.1	AKQ00030.1	ANK35834.1	AIQ84231.1	AIQ84221.1	AIQ84236.1	AFX65874.1	AKQ00032.1	AKQ00034.1	AKQ00028.1	AIQ84222.1	TV003	TDEN	DPIV	Dengvaxia	DENVax	TVDV
195488	IGRMLNILNRRKRST	C	87	101	HLA-DRB1*14:02	0%	Dengue virus 4	ELISPOT	IFNg release	Positive	0	0	0	0	0	0	0	0	0	0	0	0	0	0	0	0	0	0	0	0	0	0	0	0	0	0	0	0	0	0	0	0	0	0	0	0	0	0	0	0	0	0
195768	MTLLCLIPTAMAFHL	C	102	116	HLA-DRB1*01:01	0%	Dengue virus 4	ELISPOT	IFNg release	Positive	0	0	0	0	0	0	0	0	0	0	0	0	0	0	0	0	0	0	0	0	0	0	0	0	0	0	0	0	0	0	0	0	0	0	0	0	0	0	0	0	0	0
195922	RWGQLKKTKAIKILT	C	67	81	HLA-DRB1*07:01	0%	Dengue virus 4	ELISPOT	IFNg release	Positive	0	0	0	0	0	0	0	0	0	0	0	0	0	0	0	0	0	0	0	0	0	0	0	0	0	0	0	0	0	0	0	0	0	0	0	0	0	0	0	0	0	0
196065	TPQGLVKRFSSGLFS	C	24	38	HLA-DRB1*15:01	0%	Dengue virus 4	ELISPOT	IFNg release	Positive	0	0	0	0	0	0	0	0	0	0	0	0	0	0	0	0	0	0	0	0	0	0	0	0	0	0	0	0	0	0	0	0	0	0	0	0	0	0	0	0	0	0
739060	GRMLNILNGRKRSTM	C	88	102	HLA-DRB1*14:02	0%	Dengue virus 4	ELISPOT	IFNg release	Positive	0	0	0	0	0	0	0	0	0	0	0	0	0	0	0	0	0	0	0	0	0	0	0	0	0	0	0	0	0	0	0	0	0	0	0	0	0	0	0	0	0	0
739178	IGRMLNILNRRRRST	C	87	101	HLA-DRB1*14:02	0%	Dengue virus 4	ELISPOT	IFNg release	Positive	0	0	0	0	0	0	0	0	0	0	0	0	0	0	0	0	0	0	0	0	0	0	0	0	0	0	0	0	0	0	0	0	0	0	0	0	0	0	0	0	0	0
167808	RDVNKEKVVGRVISSTPLAE	E	340	359	HLA class II	0%	Dengue virus 4	ELISPOT	IFNg release	Positive	0	0	0	0	0	0	0	0	0	0	0	0	0	0	0	0	0	0	0	0	0	0	0	0	0	0	0	0	0	0	0	0	0	0	0	0	0	0	0	0	0	0
195364	GGVSWMIRILIGILV	E	449	463	HLA-DRB1*15:06	0%	Dengue virus 4	ELISPOT	IFNg release	Positive	0	0	0	0	0	0	0	0	0	0	0	0	0	0	0	0	0	0	0	0	0	0	0	0	0	0	0	0	0	0	0	0	0	0	0	0	0	0	0	0	0	0
195435	GVSWMIRILIGLLVL	E	729	743	HLA-DRB1*15:06	0%	Dengue virus 4	ELISPOT	IFNg release	Positive	0	0	0	0	0	0	0	0	0	0	0	0	0	0	0	0	0	0	0	0	0	0	0	0	0	0	0	0	0	0	0	0	0	0	0	0	0	0	0	0	0	0
195436	GVSWMVRILIGFLVL	E	450	464	HLA-DRB1*08:02	0%	Dengue virus 4	ELISPOT	IFNg release	Positive	0	0	0	0	0	0	0	0	0	0	0	0	0	0	0	0	0	0	0	0	0	0	0	0	0	0	0	0	0	0	0	0	0	0	0	0	0	0	0	1	0	1
195625	LESTYRGAKRMAILG	E	681	695	HLA-DRB1*14:02	0%	Dengue virus 4	ELISPOT	IFNg release	Positive	0	0	0	0	0	0	0	0	0	0	0	0	0	0	0	0	0	0	0	0	0	0	0	0	0	0	0	0	0	0	0	0	0	0	0	0	0	0	0	0	0	1
196189	VSWIIRILIGLLVLW	E	451	465	HLA-DRB1*15:06	0%	Dengue virus 4	ELISPOT	IFNg release	Positive	0	0	0	0	0	0	0	0	0	0	0	0	0	0	0	0	0	0	0	0	0	0	0	0	0	0	0	0	0	0	0	0	0	0	0	0	0	0	0	0	0	0
540080	VRILIGFLVLWIGTN	E	455	469	HLA-DRB1*15:06	0%	Dengue virus 4	ELISPOT	IFNg release	Positive	0	0	0	0	0	0	0	0	0	0	0	0	0	0	0	0	0	0	0	0	0	0	0	0	0	0	0	0	0	0	0	0	0	0	0	0	0	0	0	1	0	1
195791	NIMWKQITNELNYVL	NS1	839	853	HLA-DRB1*04:07	0%	Dengue virus 4	ELISPOT	IFNg release	Positive	0	0	0	0	0	0	0	0	0	0	0	0	0	0	0	0	0	0	0	0	0	0	0	0	0	0	0	0	0	0	0	0	0	0	0	0	0	0	0	0	0	0
539829	QYQFQPESPARLASA	NS1	805	819	HLA-DRB1*09:01	0%	Dengue virus 4	ELISPOT	IFNg release	Positive	0	0	0	0	0	0	0	0	0	0	0	0	0	0	0	0	0	0	0	0	0	0	0	0	0	0	0	0	0	0	0	0	0	0	0	0	0	0	0	0	0	0
195156	ALSLTFIRSTMPLVM	NS2a	1274	1288	HLA-DRB1*07:01	0%	Dengue virus 4	ELISPOT	IFNg release	Positive	0	0	0	0	0	0	0	0	0	0	0	0	0	0	0	0	0	0	0	0	0	0	0	0	0	0	0	0	0	0	0	0	0	0	0	0	1	1	1	0	0	0
195158	ALSLTFIRSTTPLVM	NS2a	1274	1288	HLA-DRB1*07:01	0%	Dengue virus 4	ELISPOT	IFNg release	Positive	0	0	0	0	0	0	0	0	0	0	0	0	0	0	0	0	0	0	0	0	0	0	0	0	0	0	0	0	0	0	0	0	0	0	0	0	0	0	0	0	0	0
195221	DLMELISGISLGLIL	NS2a	1243	1257	HLA-DRB1*07:01	0%	Dengue virus 4	ELISPOT	IFNg release	Positive	0	0	0	0	0	0	0	0	0	0	0	0	0	0	0	0	0	0	0	0	0	0	0	0	0	0	0	0	0	0	0	0	0	0	0	0	0	0	0	0	0	0
539406	IGGQIHLAIMAVFKM	NS2a	1194	1208	HLA-DRB1*13:01	0%	Dengue virus 4	ELISPOT	IFNg release	Positive	0	0	0	0	0	0	0	0	0	0	0	0	0	0	0	0	0	0	0	0	0	0	0	0	0	0	0	0	0	0	0	0	0	0	0	0	1	1	1	0	0	0
539433	IRSTMPLVMAWRTIM	NS2a	1280	1294	HLA-DRB1*13:01	0%	Dengue virus 4	ELISPOT	IFNg release	Positive	0	0	0	0	0	0	0	0	0	0	0	0	0	0	0	0	0	0	0	0	0	0	0	0	0	0	0	0	0	0	0	0	0	0	0	0	1	1	1	0	0	0
539627	LSLTFIRSTMPLVMA	NS2a	1275	1289	HLA-DRB1*10:01	0%	Dengue virus 4	ELISPOT	IFNg release	Positive	0	0	0	0	0	0	0	0	0	0	0	0	0	0	0	0	0	0	0	0	0	0	0	0	0	0	0	0	0	0	0	0	0	0	0	0	1	1	1	0	0	0
539628	LSLTFIRSTTPLVMA	NS2a	1275	1289	HLA-DRB1*08:03	0%	Dengue virus 4	ELISPOT	IFNg release	Positive	0	0	0	0	0	0	0	0	0	0	0	0	0	0	0	0	0	0	0	0	0	0	0	0	0	0	0	0	0	0	0	0	0	0	0	0	0	0	0	0	0	0
539919	SLTFIRSTMPLVMAW	NS2a	1276	1290	HLA-DRB1*09:01	0%	Dengue virus 4	ELISPOT	IFNg release	Positive	0	0	0	0	0	0	0	0	0	0	0	0	0	0	0	0	0	0	0	0	0	0	0	0	0	0	0	0	0	0	0	0	0	0	0	0	1	1	1	0	0	0
539921	SLTFIRSTTPLIMAW	NS2a	1276	1290	HLA-DRB1*04:03	0%	Dengue virus 4	ELISPOT	IFNg release	Positive	0	0	0	0	0	0	0	0	0	0	0	0	0	0	0	0	0	0	0	0	0	0	0	0	0	0	0	0	0	0	0	0	0	0	0	0	0	0	0	0	0	0
867969	LTFIRSTTPLVMAWR	NS2a	1277	1291	HLA-DRB3*02:02	0%	Dengue virus 4	ELISPOT	IFNg release	Positive	0	0	0	0	0	0	0	0	0	0	0	0	0	0	0	0	0	0	0	0	0	0	0	0	0	0	0	0	0	0	0	0	0	0	0	0	0	0	0	0	0	0
45689	NRKGKIVGLYGNGVV	NS3	141	155	HLA-DRB1*15:01	0%	Dengue virus 4	ICS	IFNg release	Positive	0	0	0	0	0	0	0	0	0	0	0	0	0	0	0	0	0	0	0	0	0	0	0	0	0	0	0	0	0	0	0	0	0	0	0	0	0	0	0	0	0	0
53505	REGEKKKLRPRWLDR	NS3	585	599	HLA class II	0%	Dengue virus 4	ICS	IFNg release	Positive	0	0	0	0	0	0	0	0	0	0	0	0	0	0	0	0	0	0	0	0	0	0	0	0	0	0	0	0	0	0	0	0	0	0	0	0	0	0	0	0	0	0
539021	AAAIFMTATPPGSID	NS3	1783	1797	HLA-DRB1*04:07	0%	Dengue virus 4	ELISPOT	IFNg release	Positive	0	0	0	0	0	0	0	0	0	0	0	0	0	0	0	0	0	0	0	0	0	0	0	0	0	0	0	0	0	0	0	0	0	0	0	0	0	0	0	0	0	0
539023	AAAIFMTATPPGTID	NS3	1783	1797	HLA-DRB1*04:03	0%	Dengue virus 4	ELISPOT	IFNg release	Positive	0	0	0	0	0	0	0	0	0	0	0	0	0	0	0	0	0	0	0	0	0	0	0	0	0	0	0	0	0	0	0	0	0	0	0	0	0	0	0	0	0	0
540077	VREAIKRRLRTLVLA	NS3	1682	1696	HLA-DRB1*08:03	0%	Dengue virus 4	ELISPOT	IFNg release	Positive	0	0	0	0	0	0	0	0	0	0	0	0	0	0	0	0	0	0	0	0	0	0	0	0	0	0	0	0	0	0	0	0	0	0	0	0	0	0	0	0	0	0
195853	QLIYVILTILAIIGL	NS4a	2225	2239	HLA-DRB1*15:02	0%	Dengue virus 4	ELISPOT	IFNg release	Positive	0	0	0	0	0	0	0	0	0	0	0	0	0	0	0	0	0	0	0	0	0	0	0	0	0	0	0	0	0	0	0	0	0	0	0	0	0	0	0	0	0	0
196250	YVILAILTIIGLVAA	NS4a	2228	2242	HLA-DRB1*08:02	0%	Dengue virus 4	ELISPOT	IFNg release	Positive	0	0	0	0	0	0	0	0	0	0	0	0	0	0	0	0	0	0	0	0	0	0	0	0	0	0	0	0	0	0	0	0	0	0	0	0	0	0	0	0	0	0
196251	YVILALLTIISLIAA	NS4a	2228	2242	HLA-DRB1*15:02	0%	Dengue virus 4	ELISPOT	IFNg release	Positive	0	0	0	0	0	0	0	0	0	0	0	0	0	0	0	0	0	0	0	0	0	0	0	0	0	0	0	0	0	0	0	0	0	0	0	0	0	0	0	0	0	0
195719	LTASLAMLLVHYAII	NS4b	2346	2360	HLA-DRB1*07:01	0%	Dengue virus 4	ELISPOT	IFNg release	Positive	0	0	0	0	0	0	0	0	0	0	0	0	0	0	0	0	0	0	0	0	0	0	0	0	0	0	0	0	0	0	0	0	0	0	0	0	0	0	0	0	0	0
196055	TLIASLVMLLVHYAI	NS4b	2345	2359	HLA-DRB1*01:01	0%	Dengue virus 4	ELISPOT	IFNg release	Positive	0	0	0	0	0	0	0	0	0	0	0	0	0	0	0	0	0	0	0	0	0	0	0	0	0	0	0	0	0	0	0	0	0	0	0	0	0	0	0	0	0	0
196082	TTLIASLVMLLVHYA	NS4b	2344	2358	HLA-DRB1*13:01	0%	Dengue virus 4	ELISPOT	IFNg release	Positive	0	0	0	0	0	0	0	0	0	0	0	0	0	0	0	0	0	0	0	0	0	0	0	0	0	0	0	0	0	0	0	0	0	0	0	0	0	0	0	0	0	0
196209	WAFCEVLTLATGPVL	NS4b	2427	2441	HLA-DRB1*07:01	0%	Dengue virus 4	ELISPOT	IFNg release	Positive	0	0	0	0	0	0	0	0	0	0	0	0	0	0	0	0	0	0	0	0	0	0	0	0	0	0	0	0	0	0	0	0	0	0	0	0	0	0	0	0	0	0
539661	MGCYSQVNPTTLIAS	NS4b	2335	2349	HLA-DRB1*09:01	0%	Dengue virus 4	ELISPOT	IFNg release	Positive	0	0	0	0	0	0	0	0	0	0	0	0	0	0	0	0	0	0	0	0	0	0	0	0	0	0	0	0	0	0	0	0	0	0	0	0	0	0	0	0	0	0
738500	AMGCYSQVNPTTLIA	NS4b	2334	2348	HLA-DRB1*04:07	0%	Dengue virus 4	ELISPOT	IFNg release	Positive	0	0	0	0	0	0	0	0	0	0	0	0	0	0	0	0	0	0	0	0	0	0	0	0	0	0	0	0	0	0	0	0	0	0	0	0	0	0	0	0	0	0
195593	KTFMKDGRSLVVPCR	NS5	3202	3216	HLA-DRB1*03:01	0%	Dengue virus 4	ELISPOT	IFNg release	Positive	0	0	0	0	0	0	0	0	0	0	0	0	0	0	0	0	0	0	0	0	0	0	0	0	0	0	0	0	0	0	0	0	0	0	0	0	0	0	0	0	0	0
195815	NRVWIEDNPNMTDKT	NS5	3302	3316	HLA-DRB1*03:01	0%	Dengue virus 4	ELISPOT	IFNg release	Positive	0	0	0	0	0	0	0	0	0	0	0	0	0	0	0	0	0	0	0	0	0	0	0	0	0	0	0	0	0	0	0	0	0	0	0	0	1	1	1	0	0	0
196105	VDYMPVMKRYSAPFE	NS5	3368	3382	HLA-DRB1*11:01	0%	Dengue virus 4	ELISPOT	IFNg release	Positive	0	0	0	0	0	0	0	0	0	0	0	0	0	0	0	0	0	0	0	0	0	0	0	0	0	0	0	0	0	0	0	0	0	0	0	0	0	0	0	0	0	0
17605	FRKEIGRML	C	83	91	HLA-DPw4	100%	Dengue virus 4	51 chromium	cytotoxicity	Positive	1	1	1	1	1	1	1	1	1	1	1	1	1	1	1	1	1	1	1	1	1	1	1	1	1	1	1	1	1	1	1	1	1	1	1	1	1	1	1	0	0	0
195316	FNMLKRERNRVSTPQ	C	12	26	HLA-DRB1*04:01	100%	Dengue virus 4	ELISPOT	IFNg release	Positive	1	1	1	1	1	1	1	1	1	1	1	1	1	1	1	1	1	1	1	1	1	1	1	1	1	1	1	1	1	1	1	1	1	1	1	1	1	1	1	0	0	0
195325	FRKEIGRMLNILNGR	C	19	33	HLA-DRB1*04:01	100%	Dengue virus 4	ELISPOT	IFNg release	Positive	1	1	1	1	1	1	1	1	1	1	1	1	1	1	1	1	1	1	1	1	1	1	1	1	1	1	1	1	1	1	1	1	1	1	1	1	1	1	1	0	0	0
539294	GFRKEIGRMLNILNG	C	54	68	HLA-DRB3*02:02	100%	Dengue virus 4	ELISPOT	IFNg release	Positive	1	1	1	1	1	1	1	1	1	1	1	1	1	1	1	1	1	1	1	1	1	1	1	1	1	1	1	1	1	1	1	1	1	1	1	1	1	1	1	0	0	0
739059	GRMLNILNGRKRSTI	C	88	102	HLA-DRB1*14:06	100%	Dengue virus 4	ELISPOT	IFNg release	Positive	1	1	1	1	1	1	1	1	1	1	1	1	1	1	1	1	1	1	1	1	1	1	1	1	1	1	1	1	1	1	1	1	1	1	1	1	1	1	1	0	0	0
739175	IGRMLNILNGRKRST	C	87	101	HLA-DRB1*14:02	100%	Dengue virus 4	ELISPOT	IFNg release	Positive	1	1	1	1	1	1	1	1	1	1	1	1	1	1	1	1	1	1	1	1	1	1	1	1	1	1	1	1	1	1	1	1	1	1	1	1	1	1	1	0	0	0
740156	RPPFNMLKRERNRVS	C	9	23	HLA-DRB1*14:02	100%	Dengue virus 4	ELISPOT	IFNg release	Positive	1	1	1	1	1	1	1	1	1	1	1	1	1	1	1	1	1	1	1	1	1	1	1	1	1	1	1	1	1	1	1	1	1	1	1	1	1	1	1	0	0	0
195283	FALLAGFMAYMIGQT	PreM	246	260	HLA-DRB1*01:01	100%	Dengue virus 4	ELISPOT	IFNg release	Positive	1	1	1	1	1	1	1	1	1	1	1	1	1	1	1	1	1	1	1	1	1	1	1	1	1	1	1	1	1	1	1	1	1	1	1	1	1	1	1	0	1	1
195505	ILRNPGFALLAGFMA	PreM	240	254	HLA-DRB1*01:01	100%	Dengue virus 4	ELISPOT	IFNg release	Positive	1	1	1	1	1	1	1	1	1	1	1	1	1	1	1	1	1	1	1	1	1	1	1	1	1	1	1	1	1	1	1	1	1	1	1	1	1	1	1	0	1	1
195901	RNPGFALLAGFMAYM	PreM	242	256	HLA-DRB1*15:01	100%	Dengue virus 4	ELISPOT	IFNg release	Positive	1	1	1	1	1	1	1	1	1	1	1	1	1	1	1	1	1	1	1	1	1	1	1	1	1	1	1	1	1	1	1	1	1	1	1	1	1	1	1	0	1	1
869119	TEGINKCTLIAMDLG	PreM	113	127	HLA-DQA1*01:02/DQB1*06:02	100%	Dengue virus 4	ELISPOT	IFNg release	Positive	1	1	1	1	1	1	1	1	1	1	1	1	1	1	1	1	1	1	1	1	1	1	1	1	1	1	1	1	1	1	1	1	1	1	1	1	1	1	1	0	1	1
195202	CIAVGGITLFLGFTV	E	758	772	HLA-DRB1*15:01	100%	Dengue virus 4	ELISPOT	IFNg release	Positive	1	1	1	1	1	1	1	1	1	1	1	1	1	1	1	1	1	1	1	1	1	1	1	1	1	1	1	1	1	1	1	1	1	1	1	1	1	1	1	1	1	1
741747	MRCVGVGNRDFVEGVSGGAW	E	280	299	HLA class II	100%	Dengue virus 4	ELISPOT	IFNg release	Positive	1	1	1	1	1	1	1	1	1	1	1	1	1	1	1	1	1	1	1	1	1	1	1	1	1	1	1	1	1	1	1	1	1	1	1	1	1	1	1	1	1	1
195822	NVMWKQITNELNYVL	NS1	1	15	HLA-DRB1*04:07	100%	Dengue virus 4	ELISPOT	IFNg release	Positive	1	1	1	1	1	1	1	1	1	1	1	1	1	1	1	1	1	1	1	1	1	1	1	1	1	1	1	1	1	1	1	1	1	1	1	1	1	1	1	0	0	0
866696	FGMFTTNIWMKFREG	NS1	934	948	HLA-DPB1*04:01	100%	Dengue virus 4	ELISPOT	IFNg release	Positive	1	1	1	1	1	1	1	1	1	1	1	1	1	1	1	1	1	1	1	1	1	1	1	1	1	1	1	1	1	1	1	1	1	1	1	1	1	1	1	0	0	0
867043	HADMGYWIESSKNQT	NS1	969	983	HLA-DQA1*01:01/DQB1*05:01	100%	Dengue virus 4	ELISPOT	IFNg release	Positive	1	1	1	1	1	1	1	1	1	1	1	1	1	1	1	1	1	1	1	1	1	1	1	1	1	1	1	1	1	1	1	1	1	1	1	1	1	1	1	0	0	0
539930	SRETALMVIGMAMTT	NS2a	1223	1237	HLA-DRB1*15:06	100%	Dengue virus 4	ELISPOT	IFNg release	Positive	1	1	1	1	1	1	1	1	1	1	1	1	1	1	1	1	1	1	1	1	1	1	1	1	1	1	1	1	1	1	1	1	1	1	1	1	1	1	1	0	0	0
740701	VVTLIPLCRTSCLQK	NS2a	1299	1313	HLA-DRB1*11:04	100%	Dengue virus 4	ELISPOT	IFNg release	Positive	1	1	1	1	1	1	1	1	1	1	1	1	1	1	1	1	1	1	1	1	1	1	1	1	1	1	1	1	1	1	1	1	1	1	1	1	1	1	1	0	0	0
195404	GLVSLLGSALLKNDV	NS2b	9	23	HLA-DRB1*01:02	100%	Dengue virus 4	ELISPOT	IFNg release	Positive	1	1	1	1	1	1	1	1	1	1	1	1	1	1	1	1	1	1	1	1	1	1	1	1	1	1	1	1	1	1	1	1	1	1	1	1	1	1	1	0	0	0
195511	IMAVGLVSLLGSALL	NS2b	1352	1366	HLA-DRB1*01:01	100%	Dengue virus 4	ELISPOT	IFNg release	Positive	1	1	1	1	1	1	1	1	1	1	1	1	1	1	1	1	1	1	1	1	1	1	1	1	1	1	1	1	1	1	1	1	1	1	1	1	1	1	1	0	0	0
195748	MAVGLVSLLGSALLK	NS2b	1353	1367	HLA-DRB1*01:01	100%	Dengue virus 4	ELISPOT	IFNg release	Positive	1	1	1	1	1	1	1	1	1	1	1	1	1	1	1	1	1	1	1	1	1	1	1	1	1	1	1	1	1	1	1	1	1	1	1	1	1	1	1	0	0	0
195931	SALLKNDVPLAGPMV	NS2b	1363	1377	HLA-DRB1*03:01	100%	Dengue virus 4	ELISPOT	IFNg release	Positive	1	1	1	1	1	1	1	1	1	1	1	1	1	1	1	1	1	1	1	1	1	1	1	1	1	1	1	1	1	1	1	1	1	1	1	1	1	1	1	0	0	0
196062	TNMITLLVKLALITV	NS2b	1436	1450	HLA-DRB1*08:02	100%	Dengue virus 4	ELISPOT	IFNg release	Positive	1	1	1	1	1	1	1	1	1	1	1	1	1	1	1	1	1	1	1	1	1	1	1	1	1	1	1	1	1	1	1	1	1	1	1	1	1	1	1	0	0	0
539101	AVGLVSLLGSALLKN	NS2b	1354	1368	HLA-DRB1*10:01	100%	Dengue virus 4	ELISPOT	IFNg release	Positive	1	1	1	1	1	1	1	1	1	1	1	1	1	1	1	1	1	1	1	1	1	1	1	1	1	1	1	1	1	1	1	1	1	1	1	1	1	1	1	0	0	0
1067119	GKVIGLYGNGVVTKS	NS3	1618	1632	HLA-DRB1*15:01	100%	Dengue virus 4	ELISPOT	IFNg release	Positive	1	1	1	1	1	1	1	1	1	1	1	1	1	1	1	1	1	1	1	1	1	1	1	1	1	1	1	1	1	1	1	1	1	1	1	1	1	1	1	0	0	0
12605	EIVDLMCHAT	NS3	255	264	HLA-DPw2	100%	Dengue virus 4	51 chromium	cytotoxicity	Positive	1	1	1	1	1	1	1	1	1	1	1	1	1	1	1	1	1	1	1	1	1	1	1	1	1	1	1	1	1	1	1	1	1	1	1	1	1	1	1	0	0	0
1392192	GREIVDLMCHATFTT	NS3	1727	1741		100%	Dengue virus 4	biological activity	activation	Positive	1	1	1	1	1	1	1	1	1	1	1	1	1	1	1	1	1	1	1	1	1	1	1	1	1	1	1	1	1	1	1	1	1	1	1	1	1	1	1	0	0	0
156711	LAPTRVVAAEMEEAL	NS3	1695	1709	HLA class II	100%	Dengue virus 4	51 chromium	cytotoxicity	Positive	1	1	1	1	1	1	1	1	1	1	1	1	1	1	1	1	1	1	1	1	1	1	1	1	1	1	1	1	1	1	1	1	1	1	1	1	1	1	1	0	0	0
195244	EGVYRIMQRGLFGKT	NS3	1494	1508	HLA-DRB1*11:01	100%	Dengue virus 4	ELISPOT	IFNg release	Positive	1	1	1	1	1	1	1	1	1	1	1	1	1	1	1	1	1	1	1	1	1	1	1	1	1	1	1	1	1	1	1	1	1	1	1	1	1	1	1	0	0	0
195298	FHTMWHVTRGSVICH	NS3	1520	1534	HLA-DRB1*07:01	100%	Dengue virus 4	ELISPOT	IFNg release	Positive	1	1	1	1	1	1	1	1	1	1	1	1	1	1	1	1	1	1	1	1	1	1	1	1	1	1	1	1	1	1	1	1	1	1	1	1	1	1	1	0	0	0
195353	GEIGAVTLDFKPGTS	NS3	1595	1609	HLA-DRB1*03:01	100%	Dengue virus 4	ELISPOT	IFNg release	Positive	1	1	1	1	1	1	1	1	1	1	1	1	1	1	1	1	1	1	1	1	1	1	1	1	1	1	1	1	1	1	1	1	1	1	1	1	1	1	1	0	0	0
195563	KGKVIGLYGNGVVTK	NS3	5	19	HLA-DRB1*15:01	100%	Dengue virus 4	ELISPOT	IFNg release	Positive	1	1	1	1	1	1	1	1	1	1	1	1	1	1	1	1	1	1	1	1	1	1	1	1	1	1	1	1	1	1	1	1	1	1	1	1	1	1	1	0	0	0
195695	LPVWLSYKVASAGIS	NS3	2016	2030	HLA-DRB1*08:02	100%	Dengue virus 4	ELISPOT	IFNg release	Positive	1	1	1	1	1	1	1	1	1	1	1	1	1	1	1	1	1	1	1	1	1	1	1	1	1	1	1	1	1	1	1	1	1	1	1	1	1	1	1	0	0	0
195838	PNYNLIVMDEAHFTD	NS3	1750	1764	HLA-DRB1*03:01	100%	Dengue virus 4	ELISPOT	IFNg release	Positive	1	1	1	1	1	1	1	1	1	1	1	1	1	1	1	1	1	1	1	1	1	1	1	1	1	1	1	1	1	1	1	1	1	1	1	1	1	1	1	0	0	0
195962	SIVREALKRRLRTLI	NS3	1680	1694	HLA-DRB1*11:01	100%	Dengue virus 4	ELISPOT	IFNg release	Positive	1	1	1	1	1	1	1	1	1	1	1	1	1	1	1	1	1	1	1	1	1	1	1	1	1	1	1	1	1	1	1	1	1	1	1	1	1	1	1	0	0	0
33192	KRLTIMDLHPGAGKT	NS3	186	200	HLA class II	100%	Dengue virus 4	ICS	IFNg release	Positive	1	1	1	1	1	1	1	1	1	1	1	1	1	1	1	1	1	1	1	1	1	1	1	1	1	1	1	1	1	1	1	1	1	1	1	1	1	1	1	0	0	0
539080	ALRGLPIRYQTPAVK	NS3	96	110	HLA-DRB1*13:01	100%	Dengue virus 4	ELISPOT	IFNg release	Positive	1	1	1	1	1	1	1	1	1	1	1	1	1	1	1	1	1	1	1	1	1	1	1	1	1	1	1	1	1	1	1	1	1	1	1	1	1	1	1	0	0	0
539359	GVFHTMWHVTRGSVI	NS3	1518	1532	HLA-DRB1*08:03	100%	Dengue virus 4	ELISPOT	IFNg release	Positive	1	1	1	1	1	1	1	1	1	1	1	1	1	1	1	1	1	1	1	1	1	1	1	1	1	1	1	1	1	1	1	1	1	1	1	1	1	1	1	0	0	0
539520	KVIGLYGNGVVTKSG	NS3	7	21	HLA-DRB1*15:06	100%	Dengue virus 4	ELISPOT	IFNg release	Positive	1	1	1	1	1	1	1	1	1	1	1	1	1	1	1	1	1	1	1	1	1	1	1	1	1	1	1	1	1	1	1	1	1	1	1	1	1	1	1	0	0	0
539547	LFKTLTGEIGAVTLD	NS3	1589	1603	HLA-DRB1*10:01	100%	Dengue virus 4	ELISPOT	IFNg release	Positive	1	1	1	1	1	1	1	1	1	1	1	1	1	1	1	1	1	1	1	1	1	1	1	1	1	1	1	1	1	1	1	1	1	1	1	1	1	1	1	0	0	0
539767	PGLFKTLTGEIGAVT	NS3	1587	1601	HLA-DRB1*10:01	100%	Dengue virus 4	ELISPOT	IFNg release	Positive	1	1	1	1	1	1	1	1	1	1	1	1	1	1	1	1	1	1	1	1	1	1	1	1	1	1	1	1	1	1	1	1	1	1	1	1	1	1	1	0	0	0
539803	QGKTVWFVPSIKAGN	NS3	1829	1843	HLA-DRB1*08:03	100%	Dengue virus 4	ELISPOT	IFNg release	Positive	1	1	1	1	1	1	1	1	1	1	1	1	1	1	1	1	1	1	1	1	1	1	1	1	1	1	1	1	1	1	1	1	1	1	1	1	1	1	1	0	0	0
539859	RKSGKKVIQLSRKTF	NS3	1850	1864	HLA-DRB1*08:03	100%	Dengue virus 4	ELISPOT	IFNg release	Positive	1	1	1	1	1	1	1	1	1	1	1	1	1	1	1	1	1	1	1	1	1	1	1	1	1	1	1	1	1	1	1	1	1	1	1	1	1	1	1	0	0	0
539899	SGKKVIQLSRKTFDT	NS3	1852	1866	HLA-DRB1*12:02	100%	Dengue virus 4	ELISPOT	IFNg release	Positive	1	1	1	1	1	1	1	1	1	1	1	1	1	1	1	1	1	1	1	1	1	1	1	1	1	1	1	1	1	1	1	1	1	1	1	1	1	1	1	0	0	0
68977	VIGLYGNGV	NS3	146	154	HLA-DRB1*15:01	100%	Dengue virus 4	51 chromium	cytotoxicity	Positive	1	1	1	1	1	1	1	1	1	1	1	1	1	1	1	1	1	1	1	1	1	1	1	1	1	1	1	1	1	1	1	1	1	1	1	1	1	1	1	0	0	0
739036	GLPIRYQTPAVKSEH	NS3	1711	1725	HLA-DRB1*14:06	100%	Dengue virus 4	ELISPOT	IFNg release	Positive	1	1	1	1	1	1	1	1	1	1	1	1	1	1	1	1	1	1	1	1	1	1	1	1	1	1	1	1	1	1	1	1	1	1	1	1	1	1	1	0	0	0
739563	LRGLPIRYQTPAVKS	NS3	1709	1723	HLA-DRB1*14:06	100%	Dengue virus 4	ELISPOT	IFNg release	Positive	1	1	1	1	1	1	1	1	1	1	1	1	1	1	1	1	1	1	1	1	1	1	1	1	1	1	1	1	1	1	1	1	1	1	1	1	1	1	1	0	0	0
863140	TRVEMGEAAAIFMTA	NS3	1776	1790	HLA-DQA1*03:01/DQB1*03:02	100%	Dengue virus 4	ELISPOT	IFNg release	Positive	1	1	1	1	1	1	1	1	1	1	1	1	1	1	1	1	1	1	1	1	1	1	1	1	1	1	1	1	1	1	1	1	1	1	1	1	1	1	1	0	0	0
867453	KLTDWDFVVTTDISE	NS3	1872	1886	HLA-DQA1*03:01/DQB1*03:02	100%	Dengue virus 4	ELISPOT	IFNg release	Positive	1	1	1	1	1	1	1	1	1	1	1	1	1	1	1	1	1	1	1	1	1	1	1	1	1	1	1	1	1	1	1	1	1	1	1	1	1	1	1	0	0	0
167758	ILTEIASLPTYLSSRAKL	NS4a	6	23	HLA-DP	100%	Dengue virus 4	ELISPOT	IFNg release	Positive	1	1	1	1	1	1	1	1	1	1	1	1	1	1	1	1	1	1	1	1	1	1	1	1	1	1	1	1	1	1	1	1	1	1	1	1	1	1	1	0	0	0
195176	ASLPTYLSSRAKLAL	NS4a	2103	2117	HLA-DRB1*07:01	100%	Dengue virus 4	ELISPOT	IFNg release	Positive	1	1	1	1	1	1	1	1	1	1	1	1	1	1	1	1	1	1	1	1	1	1	1	1	1	1	1	1	1	1	1	1	1	1	1	1	1	1	1	0	0	0
195274	ETLMLVALLGAMTAG	NS4a	2145	2159	HLA-DRB1*08:02	100%	Dengue virus 4	ELISPOT	IFNg release	Positive	1	1	1	1	1	1	1	1	1	1	1	1	1	1	1	1	1	1	1	1	1	1	1	1	1	1	1	1	1	1	1	1	1	1	1	1	1	1	1	0	0	0
195876	RAKLALDNIVMLHTT	NS4a	2112	2126	HLA-DRB1*03:01	100%	Dengue virus 4	ELISPOT	IFNg release	Positive	1	1	1	1	1	1	1	1	1	1	1	1	1	1	1	1	1	1	1	1	1	1	1	1	1	1	1	1	1	1	1	1	1	1	1	1	1	1	1	0	0	0
196058	TLMLVALLGAMTAGI	NS4a	2146	2160	HLA-DRB1*01:01	100%	Dengue virus 4	ELISPOT	IFNg release	Positive	1	1	1	1	1	1	1	1	1	1	1	1	1	1	1	1	1	1	1	1	1	1	1	1	1	1	1	1	1	1	1	1	1	1	1	1	1	1	1	0	0	0
539165	DILTEIASLPTYLSS	NS4a	2097	2111	HLA-DRB1*04:07	100%	Dengue virus 4	ELISPOT	IFNg release	Positive	1	1	1	1	1	1	1	1	1	1	1	1	1	1	1	1	1	1	1	1	1	1	1	1	1	1	1	1	1	1	1	1	1	1	1	1	1	1	1	0	0	0
539631	LTEIASLPTYLSSRA	NS4a	2099	2113	HLA-DRB1*04:03	100%	Dengue virus 4	ELISPOT	IFNg release	Positive	1	1	1	1	1	1	1	1	1	1	1	1	1	1	1	1	1	1	1	1	1	1	1	1	1	1	1	1	1	1	1	1	1	1	1	1	1	1	1	0	0	0
539638	LVALLGAMTAGIFLF	NS4a	2149	2163	HLA-DRB1*09:01	100%	Dengue virus 4	ELISPOT	IFNg release	Positive	1	1	1	1	1	1	1	1	1	1	1	1	1	1	1	1	1	1	1	1	1	1	1	1	1	1	1	1	1	1	1	1	1	1	1	1	1	1	1	0	0	0
738568	AYQHALNELPESLET	NS4a	2132	2146	HLA-DRB1*04:07	100%	Dengue virus 4	ELISPOT	IFNg release	Positive	1	1	1	1	1	1	1	1	1	1	1	1	1	1	1	1	1	1	1	1	1	1	1	1	1	1	1	1	1	1	1	1	1	1	1	1	1	1	1	0	0	0
868951	SLPTYLSSRAKLALD	NS4a	2104	2118	HLA-DRB3*02:02	100%	Dengue virus 4	ELISPOT	IFNg release	Positive	1	1	1	1	1	1	1	1	1	1	1	1	1	1	1	1	1	1	1	1	1	1	1	1	1	1	1	1	1	1	1	1	1	1	1	1	1	1	1	0	0	0
195177	ASLVMLLVHYAIIGP	NS4b	2348	2362	HLA-DRB1*13:01	100%	Dengue virus 4	ELISPOT	IFNg release	Positive	1	1	1	1	1	1	1	1	1	1	1	1	1	1	1	1	1	1	1	1	1	1	1	1	1	1	1	1	1	1	1	1	1	1	1	1	1	0	1	0	0	0
195219	DLEPISYDPKFEKQL	NS4b	2393	2407	HLA-DRB1*03:01	100%	Dengue virus 4	ELISPOT	IFNg release	Positive	1	1	1	1	1	1	1	1	1	1	1	1	1	1	1	1	1	1	1	1	1	1	1	1	1	1	1	1	1	1	1	1	1	1	1	1	1	1	1	0	0	0
195276	ETTILDVDLRPASAW	NS4b	2263	2277	HLA-DRB1*03:01	100%	Dengue virus 4	ELISPOT	IFNg release	Positive	1	1	1	1	1	1	1	1	1	1	1	1	1	1	1	1	1	1	1	1	1	1	1	1	1	1	1	1	1	1	1	1	1	1	1	1	1	1	1	0	0	0
195592	KTETTILDVDLRPAS	NS4b	2261	2275	HLA-DRB1*03:01	100%	Dengue virus 4	ELISPOT	IFNg release	Positive	1	1	1	1	1	1	1	1	1	1	1	1	1	1	1	1	1	1	1	1	1	1	1	1	1	1	1	1	1	1	1	1	1	1	1	1	1	1	1	0	0	0
195616	LCAGQLLLMRTTWAF	NS4b	2415	2429	HLA-DRB1*04:01	100%	Dengue virus 4	ELISPOT	IFNg release	Positive	1	1	1	1	1	1	1	1	1	1	1	1	1	1	1	1	1	1	1	1	1	1	1	1	1	1	1	1	1	1	1	1	1	1	1	1	1	1	1	0	0	0
195720	LTASLVMLLVHYAII	NS4b	2346	2360	HLA-DRB1*12:02	100%	Dengue virus 4	ELISPOT	IFNg release	Positive	1	1	1	1	1	1	1	1	1	1	1	1	1	1	1	1	1	1	1	1	1	1	1	1	1	1	1	1	1	1	1	1	1	1	1	1	1	0	1	0	0	0
196048	TILDVDLRPASAWTL	NS4b	2265	2279	HLA-DRB1*03:01	100%	Dengue virus 4	ELISPOT	IFNg release	Positive	1	1	1	1	1	1	1	1	1	1	1	1	1	1	1	1	1	1	1	1	1	1	1	1	1	1	1	1	1	1	1	1	1	1	1	1	1	1	1	0	0	0
196226	WTLYAVATTILTPML	NS4b	2277	2291	HLA-DRB1*07:01	100%	Dengue virus 4	ELISPOT	IFNg release	Positive	1	1	1	1	1	1	1	1	1	1	1	1	1	1	1	1	1	1	1	1	1	1	1	1	1	1	1	1	1	1	1	1	1	1	1	1	1	1	1	0	0	0
539057	AGQLLLMRTTWAFCE	NS4b	2417	2431	HLA-DRB1*13:01	100%	Dengue virus 4	ELISPOT	IFNg release	Positive	1	1	1	1	1	1	1	1	1	1	1	1	1	1	1	1	1	1	1	1	1	1	1	1	1	1	1	1	1	1	1	1	1	1	1	1	1	1	1	0	0	0
539185	DVDLRPASAWTLYAV	NS4b	2268	2282	HLA-DRB1*09:01	100%	Dengue virus 4	ELISPOT	IFNg release	Positive	1	1	1	1	1	1	1	1	1	1	1	1	1	1	1	1	1	1	1	1	1	1	1	1	1	1	1	1	1	1	1	1	1	1	1	1	1	1	1	0	0	0
539584	LLAMGCYSQVNPTTL	NS4b	2332	2346	HLA-DRB1*09:01	100%	Dengue virus 4	ELISPOT	IFNg release	Positive	1	1	1	1	1	1	1	1	1	1	1	1	1	1	1	1	1	1	1	1	1	1	1	1	1	1	1	1	1	1	1	1	1	1	1	1	1	1	1	0	0	0
539639	LVHYAIIGPGLQAKA	NS4b	2354	2368	HLA-DQA1*05:01/DQB1*02:01	100%	Dengue virus 4	ELISPOT	IFNg release	Positive	1	1	1	1	1	1	1	1	1	1	1	1	1	1	1	1	1	1	1	1	1	1	1	1	1	1	1	1	1	1	1	1	1	1	1	1	1	0	1	0	0	0
739202	ILDVDLRPASAWTLY	NS4b	2266	2280	HLA-DRB1*01:02	100%	Dengue virus 4	ELISPOT	IFNg release	Positive	1	1	1	1	1	1	1	1	1	1	1	1	1	1	1	1	1	1	1	1	1	1	1	1	1	1	1	1	1	1	1	1	1	1	1	1	1	1	1	0	0	0
1066543	ACLGKAYAQMWSLMY	NS5	3240	3254	HLA-DRB1*04:01	100%	Dengue virus 4	ELISPOT	IFNg release	Positive-Low	1	1	1	1	1	1	1	1	1	1	1	1	1	1	1	1	1	1	1	1	1	1	1	1	1	1	1	1	1	1	1	1	1	1	1	1	1	1	1	0	0	0
195235	EEFISKVRSNAAIGA	NS5	2884	2898	HLA-DRB1*08:02	100%	Dengue virus 4	ELISPOT	IFNg release	Positive	1	1	1	1	1	1	1	1	1	1	1	1	1	1	1	1	1	1	1	1	1	1	1	1	1	1	1	1	1	1	1	1	1	1	1	1	1	1	1	0	0	0
195454	HHFHKIFMKDGRSLV	NS5	3199	3213	HLA-DRB1*03:01	100%	Dengue virus 4	ELISPOT	IFNg release	Positive	1	1	1	1	1	1	1	1	1	1	1	1	1	1	1	1	1	1	1	1	1	1	1	1	1	1	1	1	1	1	1	1	1	1	1	1	1	1	1	0	0	0
195737	LWCGSLIGLSSRATW	NS5	3333	3347	HLA-DRB1*11:01	100%	Dengue virus 4	ELISPOT	IFNg release	Positive	1	1	1	1	1	1	1	1	1	1	1	1	1	1	1	1	1	1	1	1	1	1	1	1	1	1	1	1	1	1	1	1	1	1	1	1	1	1	1	0	0	0
195858	QMWSLMYFHRRDLRL	NS5	3248	3262	HLA-DRB1*15:01	100%	Dengue virus 4	ELISPOT	IFNg release	Positive	1	1	1	1	1	1	1	1	1	1	1	1	1	1	1	1	1	1	1	1	1	1	1	1	1	1	1	1	1	1	1	1	1	1	1	1	1	1	1	0	0	0
195879	REEFISKVRSNAAIG	NS5	2883	2897	HLA-DRB1*04:01	100%	Dengue virus 4	ELISPOT	IFNg release	Positive	1	1	1	1	1	1	1	1	1	1	1	1	1	1	1	1	1	1	1	1	1	1	1	1	1	1	1	1	1	1	1	1	1	1	1	1	1	1	1	0	0	0
195919	RVWIEDNPNMIDKTP	NS5	3303	3317	HLA-DRB1*03:01	100%	Dengue virus 4	ELISPOT	IFNg release	Positive	1	1	1	1	1	1	1	1	1	1	1	1	1	1	1	1	1	1	1	1	1	1	1	1	1	1	1	1	1	1	1	1	1	1	1	1	0	0	0	0	0	0
196230	YAQMWSLMYFHRRDL	NS5	3246	3260	HLA-DRB1*15:01	100%	Dengue virus 4	ELISPOT	IFNg release	Positive	1	1	1	1	1	1	1	1	1	1	1	1	1	1	1	1	1	1	1	1	1	1	1	1	1	1	1	1	1	1	1	1	1	1	1	1	1	1	1	0	0	0
539222	EKPDMTIIGRRLQRL	NS5	2757	2771	HLA-DRB1*12:02	100%	Dengue virus 4	ELISPOT	IFNg release	Positive	1	1	1	1	1	1	1	1	1	1	1	1	1	1	1	1	1	1	1	1	1	1	1	1	1	1	1	1	1	1	1	1	1	1	1	1	1	1	1	0	0	0
539251	FEEYKRSGILEVDRT	NS5	2512	2526	HLA-DRB1*09:01	100%	Dengue virus 4	ELISPOT	IFNg release	Positive	1	1	1	1	1	1	1	1	1	1	1	1	1	1	1	1	1	1	1	1	1	1	1	1	1	1	1	1	1	1	1	1	1	1	1	1	1	1	1	0	0	0
539456	KAIFKLTYQNKVVKV	NS5	3053	3067	HLA-DRB1*09:01	100%	Dengue virus 4	ELISPOT	IFNg release	Positive	1	1	1	1	1	1	1	1	1	1	1	1	1	1	1	1	1	1	1	1	1	1	1	1	1	1	1	1	1	1	1	1	1	1	1	1	1	1	1	0	0	0
539493	KMLLNRFTTRHRKPT	NS5	2724	2738	HLA-DRB1*13:01	100%	Dengue virus 4	ELISPOT	IFNg release	Positive	1	1	1	1	1	1	1	1	1	1	1	1	1	1	1	1	1	1	1	1	1	1	1	1	1	1	1	1	1	1	1	1	1	1	1	1	1	1	1	0	0	0
539498	KPDMTIIGRRLQRLQ	NS5	2758	2772	HLA-DRB1*08:03	100%	Dengue virus 4	ELISPOT	IFNg release	Positive	1	1	1	1	1	1	1	1	1	1	1	1	1	1	1	1	1	1	1	1	1	1	1	1	1	1	1	1	1	1	1	1	1	1	1	1	1	1	1	0	0	0
539553	LGEFGRAKGSRAIWY	NS5	2949	2963	HLA-DRB1*09:01	100%	Dengue virus 4	ELISPOT	IFNg release	Positive	1	1	1	1	1	1	1	1	1	1	1	1	1	1	1	1	1	1	1	1	1	1	1	1	1	1	1	1	1	1	1	1	1	1	1	1	1	1	1	0	0	0
539656	MAISGDDCVVKPLDERF	NS5	3146	3162	HLA class II	100%	Dengue virus 4	ICS	IFNg release	Positive	1	1	1	1	1	1	1	1	1	1	1	1	1	1	1	1	1	1	1	1	1	1	1	1	1	1	1	1	1	1	1	1	1	1	1	1	1	1	1	0	0	0
539703	MVTQLAMTDTTPFGQQR	NS5	2824	2840	HLA class II	100%	Dengue virus 4	ICS	IFNg release	Positive	1	1	1	1	1	1	1	1	1	1	1	1	1	1	1	1	1	1	1	1	1	1	1	1	1	1	1	1	1	1	1	1	1	1	1	1	1	1	1	0	0	0
540007	TTSKMLLNRFTTRHR	NS5	2721	2735	HLA-DRB1*13:01	100%	Dengue virus 4	ELISPOT	IFNg release	Positive	1	1	1	1	1	1	1	1	1	1	1	1	1	1	1	1	1	1	1	1	1	1	1	1	1	1	1	1	1	1	1	1	1	1	1	1	1	1	1	0	0	0
540041	VGTYGLNTFTNMEVQ	NS5	3091	3105	HLA-DRB1*15:06	100%	Dengue virus 4	ELISPOT	IFNg release	Positive	1	1	1	1	1	1	1	1	1	1	1	1	1	1	1	1	1	1	1	1	1	1	1	1	1	1	1	1	1	1	1	1	1	1	1	1	1	1	1	0	0	0
739405	KVRSNAAIGAVFQEE	NS5	2889	2903	HLA-DRB1*01:02	100%	Dengue virus 4	ELISPOT	IFNg release	Positive	1	1	1	1	1	1	1	1	1	1	1	1	1	1	1	1	1	1	1	1	1	1	1	1	1	1	1	1	1	1	1	1	1	1	1	1	1	1	1	0	0	0
866633	ESCVYNMMGKREKKL	NS5	2935	2949	HLA-DRB5*01:01	100%	Dengue virus 4	ELISPOT	IFNg release	Positive	1	1	1	1	1	1	1	1	1	1	1	1	1	1	1	1	1	1	1	1	1	1	1	1	1	1	1	1	1	1	1	1	1	1	1	1	1	1	1	0	0	0
867070	HKIFMKDGRSLVVPC	NS5	3202	3216	HLA-DRB3*02:02	100%	Dengue virus 4	ELISPOT	IFNg release	Positive	1	1	1	1	1	1	1	1	1	1	1	1	1	1	1	1	1	1	1	1	1	1	1	1	1	1	1	1	1	1	1	1	1	1	1	1	1	1	1	0	0	0
867315	ISKVRSNAAIGAVFQ	NS5	2887	2901	HLA-DRB3*02:02	100%	Dengue virus 4	ELISPOT	IFNg release	Positive	1	1	1	1	1	1	1	1	1	1	1	1	1	1	1	1	1	1	1	1	1	1	1	1	1	1	1	1	1	1	1	1	1	1	1	1	1	1	1	0	0	0
868638	RDLRLASMAICSAVP	NS5	3258	3272	HLA-DQA1*01:02/DQB1*06:02	100%	Dengue virus 4	ELISPOT	IFNg release	Positive	1	1	1	1	1	1	1	1	1	1	1	1	1	1	1	1	1	1	1	1	1	1	1	1	1	1	1	1	1	1	1	1	1	1	1	1	1	1	1	0	0	0
1067635	LKRWGQLKKNKAIKI	C	65	79	HLA-DRB1*07:01	81% - <100%	Dengue virus 4	ELISPOT	IFNg release	Positive-Low	1	1	1	1	1	1	1	1	1	1	1	1	1	1	1	1	0	1	1	1	1	1	1	1	1	1	1	1	1	1	1	1	1	1	1	1	1	1	1	0	0	0
1068575	VVRPPFNMLKRERNR	C	7	21	HLA-DRB1*11:01	81% - <100%	Dengue virus 4	ELISPOT	IFNg release	Positive-Low	1	1	1	1	1	1	1	1	1	1	1	1	1	1	1	1	1	1	1	1	1	1	1	1	0	1	1	1	1	1	1	1	1	1	1	1	1	1	1	0	0	0
195483	IGFRKEIGRMLNILN	C	81	95	HLA-DRB1*04:01	81% - <100%	Dengue virus 4	ELISPOT	IFNg release	Positive	1	1	1	1	1	1	1	1	1	1	1	1	1	1	1	1	1	1	1	1	1	1	1	1	0	1	1	1	1	1	1	1	1	1	1	1	1	1	1	0	0	0
195580	KNKAIKILIGFRKEI	C	53	67	HLA-DRB1*15:01	81% - <100%	Dengue virus 4	ELISPOT	IFNg release	Positive	1	1	1	1	1	1	1	1	1	1	1	1	1	1	1	1	1	1	1	1	1	1	1	1	0	1	1	1	1	1	1	1	1	1	1	1	1	1	1	0	0	0
195587	KRWGQLKKNKAIKIL	C	46	60	HLA-DRB1*08:03	81% - <100%	Dengue virus 4	ELISPOT	IFNg release	Positive	1	1	1	1	1	1	1	1	1	1	1	1	1	1	1	1	0	1	1	1	1	1	1	1	1	1	1	1	1	1	1	1	1	1	1	1	1	1	1	0	0	0
36533	LIGFRKEIGRML	C	80	91	HLA-DPw4	81% - <100%	Dengue virus 4	ICS	IFNg release	Positive	1	1	1	1	1	1	1	1	1	1	1	1	1	1	1	1	1	1	1	1	1	1	1	1	0	1	1	1	1	1	1	1	1	1	1	1	1	1	1	0	0	0
539571	LIGFRKEIGRMLNIL	C	80	94	HLA-DRB1*09:01	81% - <100%	Dengue virus 4	ELISPOT	IFNg release	Positive	1	1	1	1	1	1	1	1	1	1	1	1	1	1	1	1	1	1	1	1	1	1	1	1	0	1	1	1	1	1	1	1	1	1	1	1	1	1	1	0	0	0
539581	LKKNKAIKILIGFRK	C	51	65	HLA-DRB1*14:04	81% - <100%	Dengue virus 4	ELISPOT	IFNg release	Positive	1	1	1	1	1	1	1	1	1	1	1	1	1	1	1	1	1	1	1	1	1	1	1	1	0	1	1	1	1	1	1	1	1	1	1	1	1	1	1	0	0	0
539987	TLLCLIPTVMAFHLS	C	103	117	HLA-DRB1*04:03	81% - <100%	Dengue virus 4	ELISPOT	IFNg release	Positive	1	1	1	1	1	1	1	1	1	1	1	1	1	1	1	1	1	1	1	1	1	1	1	1	1	0	1	1	1	1	1	1	1	1	1	1	0	1	0	0	0	0
540116	WGQLKKNKAIKILIG	C	68	82	HLA-DRB1*09:01	81% - <100%	Dengue virus 4	ELISPOT	IFNg release	Positive	1	1	1	1	1	1	1	1	1	1	1	1	1	1	1	1	1	1	1	1	1	1	1	1	0	1	1	1	1	1	1	1	1	1	1	1	1	1	1	0	0	0
868732	RWGQLKKNKAIKILI	C	39	53	HLA-DRB3*02:02	81% - <100%	Dengue virus 4	ELISPOT	IFNg release	Positive	1	1	1	1	1	1	1	1	1	1	1	1	1	1	1	1	1	1	1	1	1	1	1	1	0	1	1	1	1	1	1	1	1	1	1	1	1	1	1	0	0	0
195916	RTVFFVLMMLVAPSY	PreM	264	278	HLA-DRB1*01:01	81% - <100%	Dengue virus 4	ELISPOT	IFNg release	Positive	1	1	1	1	1	1	1	1	1	1	1	1	1	1	1	1	1	1	1	1	1	1	1	1	0	1	1	1	1	1	1	1	1	1	1	1	1	1	1	0	1	1
869670	WCNLTSTWVMYGTCT	PreM	180	194	HLA-DQA1*01:02/DQB1*06:02	81% - <100%	Dengue virus 4	ELISPOT	IFNg release	Positive	1	1	1	1	1	1	1	1	1	1	1	1	1	1	1	1	1	1	1	1	1	1	1	1	0	1	1	1	1	1	1	1	1	1	1	1	1	1	1	0	1	0
195227	DSYIVIGVGNSALTL	E	654	668	HLA-DRB1*15:06	81% - <100%	Dengue virus 4	ELISPOT	IFNg release	Positive	1	1	1	1	1	0	1	1	1	1	1	1	1	1	1	1	1	1	1	1	1	1	1	1	0	1	1	1	1	1	1	0	1	1	1	1	1	1	1	0	1	0
195272	ESTYRGAKRMAILGE	E	682	696	HLA-DRB1*15:06	81% - <100%	Dengue virus 4	ELISPOT	IFNg release	Positive	1	1	1	0	1	1	1	1	1	1	1	1	1	1	1	1	1	1	1	1	1	1	1	1	1	1	1	1	1	1	1	1	0	1	1	1	1	1	1	1	1	1
195358	GGLFTSLGKAVHQVF	E	705	719	HLA-DRB1*11:01	81% - <100%	Dengue virus 4	ELISPOT	IFNg release	Positive	1	1	1	1	1	1	1	1	1	1	1	1	1	1	1	1	1	1	1	1	1	1	1	1	0	1	1	1	1	1	1	1	1	1	1	1	1	1	1	0	1	0
195434	GVSWMIRILIGFLVL	E	729	743	HLA-DRB1*08:02	81% - <100%	Dengue virus 4	ELISPOT	IFNg release	Positive	1	1	1	0	1	1	1	1	1	1	1	1	1	1	0	0	1	1	1	1	1	1	1	1	0	1	1	1	1	1	1	1	1	1	1	1	1	1	1	0	1	0
195473	IAVGGITLFLGFTVQ	E	759	773	HLA-DRB1*15:01	81% - <100%	Dengue virus 4	ELISPOT	IFNg release	Positive	1	1	1	1	1	1	1	1	1	1	1	1	1	1	1	1	1	1	1	1	1	1	1	1	0	1	1	1	1	1	1	1	1	1	1	1	1	1	1	0	1	1
196036	TGIQRTVFFVLMMLV	E	44	58	HLA-DRB1*07:01	81% - <100%	Dengue virus 4	ELISPOT	IFNg release	Positive	1	1	1	1	1	1	1	1	1	1	1	1	1	1	1	1	1	1	1	1	1	1	1	1	0	1	1	1	1	1	1	1	1	1	1	1	1	1	1	1	1	1
539230	EMILMKMKKKTWLVH	E	474	488	HLA-DRB1*12:02	81% - <100%	Dengue virus 4	ELISPOT	IFNg release	Positive	1	1	1	1	1	1	1	1	1	1	1	1	1	1	1	1	1	1	1	1	1	1	1	1	0	1	1	1	1	1	1	1	1	1	1	1	1	1	1	1	1	1
539379	HAKRQDVTVLGSQEGAMH	E	523	540	HLA class II	81% - <100%	Dengue virus 4	ICS	IFNg release	Positive	1	1	0	1	0	0	0	1	1	1	1	1	1	1	1	1	1	1	1	1	1	1	1	1	1	1	1	1	1	1	1	1	0	1	1	1	1	1	1	1	1	1
539463	KERMVTFKVPHAKRQ	E	513	527	HLA-DRB1*04:03	81% - <100%	Dengue virus 4	ELISPOT	IFNg release	Positive	1	1	0	1	0	1	0	1	1	1	1	1	1	1	1	1	1	1	1	1	1	1	1	1	1	1	1	1	1	0	1	1	1	1	1	1	1	1	1	1	1	1
539734	NLEYTVVVTVHNGDT	E	197	211	HLA-DRB1*04:07	81% - <100%	Dengue virus 4	ELISPOT	IFNg release	Positive	1	1	1	1	1	0	1	1	1	1	1	1	1	1	1	1	1	0	1	1	1	1	1	1	1	1	1	1	1	1	1	0	1	1	1	1	1	1	1	0	1	0
540036	VGGLFTSLGKAVHQV	E	704	718	HLA-DRB1*09:01	81% - <100%	Dengue virus 4	ELISPOT	IFNg release	Positive	1	1	1	1	1	1	1	1	1	1	1	1	1	1	1	1	1	1	1	1	1	1	1	1	0	1	1	1	1	1	1	1	1	1	1	1	1	1	1	1	1	0
540051	VLGSQEGAMHSALAGA	E	531	546	HLA class II	81% - <100%	Dengue virus 4	ICS	IFNg release	Positive	1	1	0	1	0	0	0	1	1	1	1	1	1	1	1	1	1	1	1	1	1	1	1	1	1	1	1	1	1	1	1	1	1	1	1	1	1	1	1	1	1	1
738809	ENLEYTVVVTVHNGD	E	196	210	HLA-DRB1*04:07	81% - <100%	Dengue virus 4	ELISPOT	IFNg release	Positive	1	1	1	1	1	0	1	1	1	1	1	1	1	1	1	1	1	0	1	1	1	1	1	1	1	1	1	1	1	1	1	0	1	1	1	1	1	1	1	1	1	0
738886	FESTYRGAKRMAILG	E	681	695	HLA-DRB1*14:02	81% - <100%	Dengue virus 4	ELISPOT	IFNg release	Positive	1	1	1	0	1	1	1	1	1	1	1	1	1	1	1	1	1	1	1	1	1	1	1	1	1	1	1	1	1	1	1	1	0	1	1	1	1	1	[illegible]	[illegible]	[illegible]	[illegible]
167725	AMTTTLSIPHDLMELIDGIS	NS2a	50	69	HLA class II	81% - <100%	Dengue virus 4	ELISPOT	IFNg release	Positive	1	1	1	1	1	1	1	1	1	1	1	1	1	1	1	1	1	1	1	1	1	1	1	1	0	1	1	1	1	1	1	1	1	1	1	1	0	0	0	0	0	0
195157	ALSLTFIRSTMSLVM	NS2a	1274	1288	HLA-DRB1*04:01	81% - <100%	Dengue virus 4	ELISPOT	IFNg release	Positive	1	1	1	1	1	1	1	1	1	1	1	1	1	1	1	1	1	0	1	1	1	1	1	1	0	1	0	1	1	1	1	1	1	1	1	1	0	0	0	0	0	0
195190	AVFKMSPGYVLGVFL	NS2a	1204	1218	HLA-DRB1*01:01	81% - <100%	Dengue virus 4	ELISPOT	IFNg release	Positive	1	1	1	1	1	1	1	1	1	1	1	1	1	1	1	1	1	1	1	1	1	1	1	1	0	1	1	1	1	1	1	1	1	1	1	1	1	1	1	0	0	0
195541	ITALILGAQALPVYL	NS2a	1320	1334	HLA-DRB1*01:01	81% - <100%	Dengue virus 4	ELISPOT	IFNg release	Positive	1	1	1	1	1	1	1	1	1	1	1	1	1	1	1	1	1	1	1	1	1	1	1	1	1	0	1	1	1	1	1	1	1	1	1	1	1	1	1	0	0	0
195699	LQKQSHWVEITALIL	NS2a	1311	1325	HLA-DRB1*07:01	81% - <100%	Dengue virus 4	ELISPOT	IFNg release	Positive	1	1	1	1	1	1	1	1	1	1	1	1	1	1	1	1	1	1	1	1	1	1	1	1	1	0	1	1	1	1	1	1	1	1	1	1	1	1	1	0	0	0

195864	QSHWVEITALILGAQ	NS2a	1314	1328	HLA-DRB1*07:01	81% - <100%	Dengue virus 4	ELISPOT	IFNg release	Positive	1	1	1	1	1	1	1	1	1	1	1	1	1	1	1	1	1	1	1	1	1	1	1	1	1	0	1	1	1	1	1	1	1	1	1	1	1	1	1	0	0	0
196061	TMSLVMAWRTIMAVL	NS2a	1283	1297	HLA-DRB1*03:01	81% - <100%	Dengue virus 4	ELISPOT	IFNg release	Positive	1	1	1	1	1	1	1	1	1	1	1	1	1	1	1	1	1	0	1	1	1	1	1	1	0	1	0	1	1	1	1	1	1	1	1	1	0	0	0	0	0	0
196070	TRKHMILAVVITLCA	NS2a	1154	1168	HLA-DRB1*08:02	81% - <100%	Dengue virus 4	ELISPOT	IFNg release	Positive	1	1	1	1	1	1	1	1	1	1	1	1	1	1	1	1	1	1	1	1	1	1	1	1	0	1	1	1	1	1	1	1	1	1	1	1	0	0	0	0	0	0
196227	WVEITALILGAQALP	NS2a	1317	1331	HLA-DRB1*01:01	81% - <100%	Dengue virus 4	ELISPOT	IFNg release	Positive	1	1	1	1	1	1	1	1	1	1	1	1	1	1	1	1	1	1	1	1	1	1	1	1	1	0	1	1	1	1	1	1	1	1	1	1	1	1	1	0	0	0
196253	YVLGVFLRKLTSRET	NS2a	1212	1226	HLA-DRB1*11:01	81% - <100%	Dengue virus 4	ELISPOT	IFNg release	Positive	1	1	1	1	1	1	1	1	1	1	1	1	1	1	1	1	1	1	1	1	1	1	1	1	0	1	1	1	1	1	1	1	1	1	1	1	1	1	1	0	0	0
539130	CLRRRVTRKHMILAV	NS2a	1148	1162	HLA-DRB1*08:03	81% - <100%	Dengue virus 4	ELISPOT	IFNg release	Positive	1	1	1	1	1	1	1	1	1	1	1	1	1	1	1	1	1	1	1	1	1	1	1	1	0	1	1	1	1	1	1	1	1	1	1	1	0	0	0	0	0	0
539198	ECLRRRVTRKHMILA	NS2a	1147	1161	HLA-DRB1*13:01	81% - <100%	Dengue virus 4	ELISPOT	IFNg release	Positive	1	1	1	1	1	1	1	1	1	1	1	1	1	1	1	1	1	1	1	1	1	1	1	1	0	1	1	1	1	1	1	1	1	1	1	1	0	0	0	0	0	0
539360	GVFLRKLTSRETALM	NS2a	1215	1229	HLA-DRB1*10:01	81% - <100%	Dengue virus 4	ELISPOT	IFNg release	Positive	1	1	1	1	1	1	1	1	1	1	1	1	1	1	1	1	1	1	1	1	1	1	1	1	0	1	1	1	1	1	1	1	1	1	1	1	1	1	1	0	0	0
539434	IRSTMSLVMAWRTIM	NS2a	1280	1294	HLA-DRB1*13:01	81% - <100%	Dengue virus 4	ELISPOT	IFNg release	Positive	1	1	1	1	1	1	1	1	1	1	1	1	1	1	1	1	1	0	1	1	1	1	1	1	0	1	0	1	1	1	1	1	1	1	1	1	0	0	0	0	0	0
539565	LGVFLRKLTSRETAL	NS2a	1214	1228	HLA-DRB1*08:03	81% - <100%	Dengue virus 4	ELISPOT	IFNg release	Positive	1	1	1	1	1	1	1	1	1	1	1	1	1	1	1	1	1	1	1	1	1	1	1	1	0	1	1	1	1	1	1	1	1	1	1	1	1	1	1	0	0	0
539961	TALMVIGMAMTTTLS	NS2a	1226	1240	HLA-DRB1*01:02	81% - <100%	Dengue virus 4	ELISPOT	IFNg release	Positive	1	1	1	1	1	1	1	1	1	1	1	1	1	1	1	1	1	1	1	1	1	1	1	1	0	1	1	1	1	1	1	1	1	1	1	1	0	0	0	0	0	0
540033	VFKMSPGYVLGVFLR	NS2a	1205	1219	HLA-DRB1*09:01	81% - <100%	Dengue virus 4	ELISPOT	IFNg release	Positive	1	1	1	1	1	1	1	1	1	1	1	1	1	1	1	1	1	1	1	1	1	1	1	1	0	1	1	1	1	1	1	1	1	1	1	1	1	1	1	0	0	0
540053	VLGVFLRKLTSRETA	NS2a	1213	1227	HLA-DRB1*14:04	81% - <100%	Dengue virus 4	ELISPOT	IFNg release	Positive	1	1	1	1	1	1	1	1	1	1	1	1	1	1	1	1	1	1	1	1	1	1	1	1	0	1	1	1	1	1	1	1	1	1	1	1	1	1	1	0	0	0
738559	AVVITLCAIILGGLT	NS2a	1161	1175	HLA-DRB1*01:02	81% - <100%	Dengue virus 4	ELISPOT	IFNg release	Positive	1	1	1	1	1	1	1	1	1	1	1	1	1	0	1	1	1	1	1	1	1	1	1	1	0	1	1	1	1	1	1	1	1	1	1	1	0	0	0	0	0	0
867110	HWVEITALILGAQAL	NS2a	1316	1330	HLA-DQA1*03:01/DQB1*03:02	81% - <100%	Dengue virus 4	ELISPOT	IFNg release	Positive	1	1	1	1	1	1	1	1	1	1	1	1	1	1	1	1	1	1	1	1	1	1	1	1	1	0	1	1	1	1	1	1	1	1	1	1	1	1	1	0	0	0
195662	LLLAAYVMSGSSADL	NS2b	1381	1395	HLA-DRB1*04:01	81% - <100%	Dengue virus 4	ELISPOT	IFNg release	Positive	1	1	1	1	1	1	1	1	1	1	1	1	1	1	1	1	1	1	1	1	1	1	1	1	1	0	1	1	1	1	1	1	1	1	1	1	1	1	1	0	0	0
539020	AAAIFMTATPPGATD	NS3	1783	1797	HLA-DRB1*10:01	81% - <100%	Dengue virus 4	ELISPOT	IFNg release	Positive	1	1	1	1	1	1	1	1	1	1	1	1	1	1	1	1	1	1	1	1	1	1	1	1	0	1	1	1	1	1	1	1	1	1	1	1	1	1	1	0	0	0
539026	AAIFMTATPPGATDP	NS3	1784	1798	HLA-DRB1*09:01	81% - <100%	Dengue virus 4	ELISPOT	IFNg release	Positive	1	1	1	1	1	1	1	1	1	1	1	1	1	1	1	1	1	1	1	1	1	1	1	1	0	1	1	1	1	1	1	1	1	1	1	1	1	1	1	0	0	0
195224	DNQLIYVILTILTII	NS4a	2223	2237	HLA-DRB1*15:01	81% - <100%	Dengue virus 4	ELISPOT	IFNg release	Positive	1	1	1	0	1	1	1	1	1	1	1	1	1	1	1	0	1	1	1	1	1	1	1	1	1	1	1	1	1	1	1	1	1	1	1	1	1	1	1	0	0	0
195386	GLITIAVASGLLWVA	NS4a	2176	2190	HLA-DRB1*07:01	81% - <100%	Dengue virus 4	ELISPOT	IFNg release	Positive	1	1	1	1	1	1	1	1	1	1	1	1	1	1	1	1	1	1	1	1	1	1	1	1	0	1	1	1	1	1	1	1	1	1	1	1	1	1	1	0	0	0
195809	NQLIYVILTILTIIG	NS4a	2224	2238	HLA-DRB1*08:02	81% - <100%	Dengue virus 4	ELISPOT	IFNg release	Positive	1	1	1	0	1	1	1	1	1	1	1	1	1	1	1	0	1	1	1	1	1	1	1	1	0	1	1	1	1	1	1	1	1	1	1	1	1	1	1	0	0	0
539666	MGLITIAVASGLLWV	NS4a	2175	2189	HLA-DRB1*09:01	81% - <100%	Dengue virus 4	ELISPOT	IFNg release	Positive	1	1	1	1	1	1	1	1	1	1	1	1	1	1	1	1	1	1	1	1	1	1	1	1	0	1	1	1	1	1	1	1	1	1	1	1	1	1	1	0	0	0
867726	LIYVILTILTIIGLI	NS4a	2226	2240	HLA-DPB1*04:02	81% - <100%	Dengue virus 4	ELISPOT	IFNg release	Positive	1	1	1	0	1	1	1	1	1	1	1	1	1	1	1	0	1	1	1	1	1	1	1	1	0	1	1	1	1	1	1	1	1	1	1	1	1	1	1	0	0	0
195214	DFGFYQVKTETTILD	NS4b	2254	2268	HLA-DRB1*04:01	81% - <100%	Dengue virus 4	ELISPOT	IFNg release	Positive	1	1	1	1	1	1	1	1	1	1	1	1	1	1	1	1	1	1	1	1	1	1	1	1	0	1	1	1	1	1	1	1	1	1	1	1	1	1	1	0	0	0
195383	GLAFSLIKNAQTPRR	NS4b	2473	2487	HLA-DRB1*04:01	81% - <100%	Dengue virus 4	ELISPOT	IFNg release	Positive	1	1	1	1	1	1	1	1	1	1	1	1	1	1	1	1	1	1	1	1	1	1	1	1	1	1	1	1	1	1	1	1	1	1	1	0	1	1	1	0	0	0
196052	TKTDFGFYQVKTETT	NS4b	2251	2265	HLA-DRB1*04:01	81% - <100%	Dengue virus 4	ELISPOT	IFNg release	Positive	1	1	1	1	1	1	1	1	1	1	1	1	1	1	1	1	1	1	1	1	1	1	1	1	0	1	1	1	1	1	1	1	1	1	1	1	1	1	1	0	0	0
196208	WAFCEVLTLATGPIL	NS4b	2427	2441	HLA-DRB1*07:01	81% - <100%	Dengue virus 4	ELISPOT	IFNg release	Positive	1	1	1	1	1	1	1	1	0	1	1	1	0	1	1	1	1	1	1	1	1	1	1	1	1	1	1	1	1	1	1	1	1	1	1	1	1	1	1	0	0	0
738447	AFSLIKNAQTPRRGT	NS4b	2475	2489	HLA-DRB1*14:06	81% - <100%	Dengue virus 4	ELISPOT	IFNg release	Positive	1	1	1	1	1	1	1	1	1	1	1	1	1	1	1	1	1	1	1	1	1	1	1	1	1	1	1	1	1	1	1	1	1	1	1	0	1	1	1	0	0	0
738897	FGFYQVKTETTILDV	NS4b	2255	2269	HLA-DRB1*04:07	81% - <100%	Dengue virus 4	ELISPOT	IFNg release	Positive	1	1	1	1	1	1	1	1	1	1	1	1	1	1	1	1	1	1	1	1	1	1	1	1	0	1	1	1	1	1	1	1	1	1	1	1	1	1	1	0	0	0
739417	LAFSLIKNAQTPRRG	NS4b	2474	2488	HLA-DRB1*14:02	81% - <100%	Dengue virus 4	ELISPOT	IFNg release	Positive	1	1	1	1	1	1	1	1	1	1	1	1	1	1	1	1	1	1	1	1	1	1	1	1	1	1	1	1	1	1	1	1	1	1	1	0	1	1	1	0	0	0
867009	GRFWNTTIAVSTANI	NS4b	2449	2463	HLA-DQA1*01:02/DQB1*06:02	81% - <100%	Dengue virus 4	ELISPOT	IFNg release	Positive	1	1	1	1	1	1	1	1	1	1	1	1	1	1	1	1	1	1	1	1	1	1	1	1	1	0	1	1	1	1	1	1	1	1	1	1	1	1	1	0	0	0
195411	GNIVSSVNTTSKMLL	NS5	2713	2727	HLA-DRB1*04:01	81% - <100%	Dengue virus 4	ELISPOT	IFNg release	Positive	1	1	1	1	1	1	1	1	1	1	1	1	1	1	1	1	1	1	1	1	1	1	1	1	1	0	1	1	1	1	1	1	1	1	1	1	1	1	1	0	0	0
195499	ILAKAIFKLTYQNKV	NS5	3050	3064	HLA-DRB1*07:01	81% - <100%	Dengue virus 4	ELISPOT	IFNg release	Positive	1	1	1	1	1	1	1	1	1	1	0	1	1	1	1	1	1	1	1	1	1	1	1	1	1	1	1	1	1	1	1	1	1	1	1	1	1	1	1	0	0	0
195823	NWLWALLGKKKNPRL	NS5	2866	2880	HLA-DRB1*14:02	81% - <100%	Dengue virus 4	ELISPOT	IFNg release	Positive	1	1	1	1	1	1	1	1	1	1	1	1	1	1	1	1	1	1	1	1	1	1	1	1	0	1	1	1	1	1	1	1	1	1	1	1	1	1	1	0	0	0
195835	PKPGTRMVMTTTANW	NS5	2853	2867	HLA-DRB1*04:01	81% - <100%	Dengue virus 4	ELISPOT	IFNg release	Positive	1	1	1	1	1	1	1	1	1	1	1	1	1	1	1	1	1	1	1	1	1	1	1	1	0	1	1	1	1	1	1	1	1	1	1	1	1	1	1	0	0	0
196071	TRMVMTTTANWLWAL	NS5	2857	2871	HLA-DRB1*04:01	81% - <100%	Dengue virus 4	ELISPOT	IFNg release	Positive	1	1	1	1	1	1	1	1	1	1	1	1	1	1	1	1	1	1	1	1	1	1	1	1	0	1	1	1	1	1	1	1	1	1	1	1	1	1	1	0	0	0
196092	TYQNKVVKVLRPTPR	NS5	3059	3073	HLA-DRB1*08:02	81% - <100%	Dengue virus 4	ELISPOT	IFNg release	Positive	1	1	1	1	1	1	1	1	1	1	1	1	1	1	1	1	1	1	1	1	1	1	1	1	0	1	1	1	1	1	1	1	1	1	1	1	1	1	1	0	0	0
539354	GTRMVMTTTANWLWA	NS5	2856	2870	HLA-DRB1*04:03	81% - <100%	Dengue virus 4	ELISPOT	IFNg release	Positive	1	1	1	1	1	1	1	1	1	1	1	1	1	1	1	1	1	1	1	1	1	1	1	1	0	1	1	1	1	1	1	1	1	1	1	1	1	1	1	0	0	0
539769	PGTRMVMTTTANWLW	NS5	2855	2869	HLA-DRB1*04:03	81% - <100%	Dengue virus 4	ELISPOT	IFNg release	Positive	1	1	1	1	1	1	1	1	1	1	1	1	1	1	1	1	1	1	1	1	1	1	1	1	0	1	1	1	1	1	1	1	1	1	1	1	1	1	1	0	0	0
539770	PHHKILAKAIFKLTY	NS5	3046	3060	HLA-DRB1*12:02	81% - <100%	Dengue virus 4	ELISPOT	IFNg release	Positive	1	1	1	1	1	1	1	1	1	1	0	1	1	1	1	1	1	1	1	1	1	1	1	1	1	1	1	1	1	1	1	1	1	1	1	1	1	1	1	0	0	0
740013	QLIRQMEAEGVITQD	NS5	3105	3119	HLA-DRB1*01:02	81% - <100%	Dengue virus 4	ELISPOT	IFNg release	Positive	1	1	1	1	1	1	1	1	1	1	1	1	1	1	1	1	1	1	1	1	1	1	1	1	0	1	1	1	1	1	1	1	1	1	1	1	1	1	1	0	0	0
867411	KILAKAIFKLTYQNK	NS5	3049	3063	HLA-DPB1*04:02	81% - <100%	Dengue virus 4	ELISPOT	IFNg release	Positive	1	1	1	1	1	1	1	1	1	1	0	1	1	1	1	1	1	1	1	1	1	1	1	1	1	1	1	1	1	1	1	1	1	1	1	1	1	1	1	0	0	0
867473	KPLDERFGTSLLFLN	NS5	3156	3170	HLA-DPB1*01:01	81% - <100%	Dengue virus 4	ELISPOT	IFNg release	Positive	1	1	1	1	1	1	1	1	1	1	1	1	1	1	1	1	1	1	1	1	1	1	1	1	0	1	1	1	1	1	1	1	1	1	1	1	1	1	1	0	0	0
867575	LDERFGTSLLFLNDM	NS5	3158	3172	HLA-DPB1*04:01	81% - <100%	Dengue virus 4	ELISPOT	IFNg release	Positive	1	1	1	1	1	1	1	1	1	1	1	1	1	1	1	1	1	1	1	1	1	1	1	1	0	1	0	1	1	1	1	1	1	1	1	1	1	1	1	0	0	0
195608	LAIMAVFKMSPGYVL	NS2a	1200	1214	HLA-DRB1*13:01	51%-80%	Dengue virus 4	ELISPOT	IFNg release	Positive	1	1	1	1	1	1	1	0	1	0	1	1	1	0	1	1	1	1	1	1	1	0	1	1	1	0	1	0	0	1	0	1	1	0	1	1	1	1	1	0	0	0
195746	MAVFKMSPGYVLGVF	NS2a	1203	1217	HLA-DRB1*07:01	51%-80%	Dengue virus 4	ELISPOT	IFNg release	Positive	1	1	1	1	1	1	1	0	1	0	1	1	1	0	1	1	1	1	1	1	1	0	1	1	0	0	1	0	0	1	0	1	1	0	1	1	1	1	1	0	0	0
539407	IGGQTHLAIMAVFKM	NS2a	1194	1208	HLA-DRB1*13:01	51%-80%	Dengue virus 4	ELISPOT	IFNg release	Positive	1	1	1	1	1	1	1	0	1	0	1	1	1	0	1	1	1	1	1	1	1	0	1	1	0	0	1	0	0	1	0	1	1	0	1	1	0	0	0	0	0	0
195230	DYMPVMKRYSAPSES	NS5	3369	3383	HLA-DRB1*11:01	51%-80%	Dengue virus 4	ELISPOT	IFNg release	Positive	1	1	1	1	1	1	1	0	1	0	1	1	1	0	1	1	1	1	1	1	1	0	1	1	0	0	1	0	0	1	0	1	1	0	1	1	1	1	1	0	0	0
195240	EEYVDYMPVMKRYSA	NS5	3365	3379	HLA-DRB1*11:01	51%-80%	Dengue virus 4	ELISPOT	IFNg release	Positive	1	1	1	1	1	1	1	0	1	0	1	1	1	0	1	1	1	1	1	1	1	0	1	1	1	0	1	0	0	1	0	1	1	0	1	1	1	1	1	0	0	0
195279	EYVDYMPVMKRYSAP	NS5	3366	3380	HLA-DRB1*11:01	51%-80%	Dengue virus 4	ELISPOT	IFNg release	Positive	1	1	1	1	1	1	1	0	1	0	1	1	1	0	1	1	1	1	1	1	1	0	1	1	0	0	1	0	0	1	0	1	1	0	1	1	1	1	1	0	0	0
195764	MPVMKRYSAPSESEG	NS5	3371	3385	HLA-DRB1*08:02	51%-80%	Dengue virus 4	ELISPOT	IFNg release	Positive	1	1	1	1	1	1	1	0	1	0	1	1	1	0	1	1	1	1	1	1	1	0	1	1	0	0	1	0	0	1	0	1	1	0	1	1	1	1	1	0	0	0
196106	VDYMPVMKRYSAPSE	NS5	3368	3382	HLA-DRB1*11:01	51%-80%	Dengue virus 4	ELISPOT	IFNg release	Positive	1	1	1	1	1	1	1	0	1	0	1	1	1	0	1	1	1	1	1	1	1	0	1	1	0	0	1	0	0	1	0	1	1	0	1	1	1	1	1	0	0	0
195651	LKKNKAIKILTGFRK	C	51	65	HLA-DRB1*14:04	>0% - 50%	Dengue virus 4	ELISPOT	IFNg release	Positive	0	0	0	0	0	0	0	0	0	0	0	0	0	0	0	0	0	0	0	0	0	0	0	0	1	0	0	0	0	0	0	0	0	0	0	0	0	0	0	0	0	0
195900	RMVLAFITFLRVLSI	C	44	58	HLA-DRB1*11:01	>0% - 50%	Dengue virus 4	ELISPOT	IFNg release	Positive	0	1	1	1	1	1	1	0	0	0	0	0	0	0	0	1	0	0	0	0	0	0	0	0	1	0	0	0	0	0	0	1	0	0	0	0	1	1	1	0	0	0
196134	VLAFITFLRVLSIPP	C	26	40	HLA-DRB1*04:01	>0% - 50%	Dengue virus 4	ELISPOT	IFNg release	Positive	0	1	1	1	1	1	1	0	0	0	0	0	0	0	0	1	0	0	0	0	0	0	0	0	1	0	0	0	0	0	0	1	0	0	0	0	1	1	1	0	0	0
539044	AFITFLRVLSIPPTA	C	28	42	HLA-DRB1*04:03	>0% - 50%	Dengue virus 4	ELISPOT	IFNg release	Positive	0	1	1	1	1	1	1	0	0	0	0	0	0	0	0	1	0	0	0	0	0	0	0	0	1	0	0	0	0	0	0	1	0	0	0	0	1	1	1	0	0	0
539255	FITFLRVLSIPPTAG	C	49	63	HLA-DRB1*04:07	>0% - 50%	Dengue virus 4	ELISPOT	IFNg release	Positive	0	1	1	1	1	1	1	0	0	0	0	0	0	0	0	1	0	0	0	0	0	0	0	0	1	0	0	0	0	0	0	1	0	0	0	0	1	1	1	0	0	0
539496	KNKAIKILTGFRKEI	C	53	67	HLA-DRB1*12:02	>0% - 50%	Dengue virus 4	ELISPOT	IFNg release	Positive	0	0	0	0	0	0	0	0	0	0	0	0	0	0	0	0	0	0	0	0	0	0	0	0	1	0	0	0	0	0	0	0	0	0	0	0	0	0	0	0	0	0
539536	LAFITFLRVLSIPPT	C	27	41	HLA-DRB1*12:02	>0% - 50%	Dengue virus 4	ELISPOT	IFNg release	Positive	0	1	1	1	1	1	1	0	0	0	0	0	0	0	0	1	0	0	0	0	0	0	0	0	1	0	0	0	0	0	0	1	0	0	0	0	1	1	1	0	0	0
539632	LTGFRKEIGRMLNIL	C	52	66	HLA-DRB3*02:02	>0% - 50%	Dengue virus 4	ELISPOT	IFNg release	Positive	0	0	0	0	0	0	0	0	0	0	0	0	0	0	0	0	0	0	0	0	0	0	0	0	1	0	0	0	0	0	0	0	0	0	0	0	0	0	0	0	0	0
539898	SGKGPLRMVLAFITF	C	18	32	HLA-DRB1*15:06	>0% - 50%	Dengue virus 4	ELISPOT	IFNg release	Positive	0	1	1	1	1	1	1	0	0	0	0	0	0	0	0	1	0	0	0	0	0	0	0	0	1	0	0	0	0	0	0	1	0	0	0	0	1	1	1	0	0	0
69367	VLAFITFLR	C	46	54	HLA-DPw4	>0% - 50%	Dengue virus 4	51 chromium	cytotoxicity	Positive	0	1	1	1	1	1	1	0	0	0	0	0	0	0	0	1	0	0	0	0	0	0	0	0	1	0	0	0	0	0	0	1	0	0	0	0	1	1	1	0	0	0
868517	QLKKNKAIKILTGFR	C	42	56	HLA-DQA1*01:02/DQB1*06:02	>0% - 50%	Dengue virus 4	ELISPOT	IFNg release	Positive	0	0	0	0	0	0	0	0	0	0	0	0	0	0	0	0	0	0	0	0	0	0	0	0	1	0	0	0	0	0	0	0	0	0	0	0	0	0	0	0	0	0
195526	IQRTVFFILMMLVAP	PreM	262	276	HLA-DRB1*07:01	>0% - 50%	Dengue virus 4	ELISPOT	IFNg release	Positive	0	0	0	0	0	0	0	0	0	0	0	0	0	0	0	0	0	0	0	0	0	0	0	0	1	0	0	0	0	0	0	0	0	0	0	0	0	0	0	0	0	0
539395	IDCWCNLTSAWVMYG	PreM	117	131	HLA-DQA1*01:02/DQB1*06:02	>0% - 50%	Dengue virus 4	ELISPOT	IFNg release	Positive	0	0	0	0	0	0	0	0	0	0	0	0	0	0	0	0	0	0	0	0	0	0	0	0	1	0	0	0	0	0	0	0	0	0	0	0	0	0	0	0	0	1
539820	QRTVFFILMMLVAPS	PreM	263	277	HLA-DRB1*13:01	>0% - 50%	Dengue virus 4	ELISPOT	IFNg release	Positive	0	0	0	0	0	0	0	0	0	0	0	0	0	0	0	0	0	0	0	0	0	0	0	0	1	0	0	0	0	0	0	0	0	0	0	0	0	0	0	0	0	0
867174	IGQTGIQRTVFFILM	PreM	257	271	HLA-DPB1*02:01	>0% - 50%	Dengue virus 4	ELISPOT	IFNg release	Positive	0	0	0	0	0	0	0	0	0	0	0	0	0	0	0	0	0	0	0	0	0	0	0	0	1	0	0	0	0	0	0	0	0	0	0	0	0	0	0	0	0	0
195472	IAVGGITLFLGFTVH	E	480	494	HLA-DRB1*15:01	>0% - 50%	Dengue virus 4	ELISPOT	IFNg release	Positive	0	0	0	0	0	0	0	0	0	0	0	0	0	0	0	0	0	0	0	0	0	0	0	0	1	0	0	0	0	0	0	0	0	0	0	0	0	0	0	0	0	1
539298	GGVSWMVRILIGLLV	E	449	463	HLA-DRB1*15:06	>0% - 50%	Dengue virus 4	ELISPOT	IFNg release	Positive	0	0	0	1	0	0	0	0	0	0	0	0	0	0	0	1	0	0	0	0	0	0	0	0	1	0	0	0	0	0	0	0	0	0	0	0	0	0	0	0	0	0
195220	DLMEFIDGISLGLIL	NS2a	1244	1258	HLA-DRB1*07:01	>0% - 50%	Dengue virus 4	ELISPOT	IFNg release	Positive	0	0	0	0	0	0	0	0	0	0	0	0	0	0	0	0	0	0	0	0	0	0	0	0	1	0	0	0	0	0	0	0	0	0	0	0	0	0	0	0	0	0
195571	KHMILVVVTTLCAII	NS2a	1156	1170	HLA-DRB1*08:02	>0% - 50%	Dengue virus 4	ELISPOT	IFNg release	Positive	0	0	0	0	0	0	0	0	0	0	0	0	0	0	0	0	0	0	0	0	0	0	0	0	1	0	0	0	0	0	0	0	0	0	0	0	0	0	0	0	0	0
195837	PLVMAWRTIMAVLFV	NS2a	1285	1299	HLA-DRB1*01:01	>0% - 50%	Dengue virus 4	ELISPOT	IFNg release	Positive	0	0	0	0	0	0	0	0	0	0	0	0	0	0	0	0	0	0	0	0	0	0	0	0	1	0	0	0	0	0	0	0	0	0	0	0	1	1	1	0	0	0
196060	TMPLVMAWRTIMAVL	NS2a	1283	1297	HLA-DRB1*03:01	>0% - 50%	Dengue virus 4	ELISPOT	IFNg release	Positive	0	0	0	0	0	0	0	0	0	0	0	0	0	0	0	0	0	0	0	0	0	0	0	0	1	0	0	0	0	0	0	0	0	0	0	0	1	1	1	0	0	0
196252	YVLGIFLRKLTSRET	NS2a	1212	1226	HLA-DRB1*11:01	>0% - 50%	Dengue virus 4	ELISPOT	IFNg release	Positive	0	0	0	0	0	0	0	0	0	0	0	0	0	0	0	0	0	0	0	0	0	0	0	0	1	0	0	0	0	0	0	0	0	0	0	0	0	0	0	0	0	0
539131	CLRRRVTRKHMILVV	NS2a	1148	1162	HLA-DRB1*08:03	>0% - 50%	Dengue virus 4	ELISPOT	IFNg release	Positive	0	0	0	0	0	0	0	0	0	0	0	0	0	0	0	0	0	0	0	0	0	0	0	0	1	0	0	0	0	0	0	0	0	0	0	0	1	1	1	0	0	0
539555	LGIFLRKLTSRETAL	NS2a	1214	1228	HLA-DRB1*08:03	>0% - 50%	Dengue virus 4	ELISPOT	IFNg release	Positive	0	0	0	0	0	0	0	0	0	0	0	0	0	0	0	0	0	0	0	0	0	0	0	0	1	0	0	0	0	0	0	0	0	0	0	0	0	0	0	0	0	0
539664	MGGQIHLAIMAVFKM	NS2a	1194	1208	HLA-DRB1*13:01	>0% - 50%	Dengue virus 4	ELISPOT	IFNg release	Positive	0	0	0	0	0	0	0	0	0	0	0	0	0	0	0	0	0	0	0	0	0	0	0	0	1	0	0	0	0	0	0	0	0	0	0	0	0	0	0	0	0	0
539687	MPLVMAWRTIMAVLF	NS2a	1284	1298	HLA-DRB1*12:02	>0% - 50%	Dengue virus 4	ELISPOT	IFNg release	Positive	0	0	0	0	0	0	0	0	0	0	0	0	0	0	0	0	0	0	0	0	0	0	0	0	1	0	0	0	0	0	0	0	0	0	0	0	1	1	1	0	0	0
539937	STMPLVMAWRTIMAV	NS2a	1282	1296	HLA-DRB1*13:01	>0% - 50%	Dengue virus 4	ELISPOT	IFNg release	Positive	0	0	0	0	0	0	0	0	0	0	0	0	0	0	0	0	0	0	0	0	0	0	0	0	1	0	0	0	0	0	0	0	0	0	0	0	1	1	1	0	0	0
539962	TALMVIGMAMTTVLS	NS2a	1226	1240	HLA-DRB1*15:06	>0% - 50%	Dengue virus 4	ELISPOT	IFNg release	Positive	0	0	0	0	0	0	0	0	0	0	0	0	0	0	0	0	0	0	0	0	0	0	0	0	1	0	0	0	0	0	0	0	0	0	0	0	1	1	1	0	0	0
738742	ECLRRRVTRKHMILV	NS2a	1147	1161	HLA-DRB1*14:02	>0% - 50%	Dengue virus 4	ELISPOT	IFNg release	Positive	0	0	0	0	0	0	0	0	0	0	0	0	0	0	0	0	0	0	0	0	0	0	0	0	1	0	0	0	0	0	0	0	0	0	0	0	1	1	1	0	0	0
867048	HDLMEFIDGISLGLI	NS2a	1243	1257	HLA-DQA1*01:01/DQB1*05:01	>0% - 50%	Dengue virus 4	ELISPOT	IFNg release	Positive	0	0	0	0	0	0	0	0	0	0	0	0	0	0	0	0	0	0	0	0	0	0	0	0	1	0	0	0	0	0	0	0	0	0	0	0	0	0	0	0	0	0
867874	LMVIGMAMTTVLSIP	NS2a	1228	1242	HLA-DQA1*01:02/DQB1*06:02	>0% - 50%	Dengue virus 4	ELISPOT	IFNg release	Positive	0	0	0	0	0	0	0	0	0	0	0	0	0	0	0	0	0	0	0	0	0	0	0	0	1	0	0	0	0	0	0	0	0	0	0	0	1	1	1	0	0	0
869635	VTMTLWYMWQVKTQR	NS2b	1460	1474	HLA-DPB1*01:01	>0% - 50%	Dengue virus 4	ELISPOT	IFNg release	Positive	0	0	0	1	0	0	0	0	0	0	0	0	0	0	0	1	0	0	0	1	0	0	0	0	1	0	0	0	0	0	0	0	0	0	0	0	1	1	1	0	0	0
196032	TEGVYRIMQRGLFGK	NS3	146	160	HLA-DRB1*11:01	>0% - 50%	Dengue virus 4	ELISPOT	IFNg release	Positive	0	0	0	0	0	0	0	0	0	0	0	0	0	0	0	0	0	0	0	0	0	0	0	0	1	0	0	0	0	0	0	0	0	0	0	0	0	0	0	0	0	0
539024	AAAIFMTATPPGTTD	NS3	1783	1797	HLA-DRB1*04:03	>0% - 50%	Dengue virus 4	ELISPOT	IFNg release	Positive	0	0	0	0	0	0	0	0	0	0	0	0	0	0	0	0	0	0	0	0	0	0	0	0	1	0	0	0	0	0	0	0	0	0	0	0	0	0	0	0	0	0
868371	PRWLDARVYADPMAL	NS3	2067	2081	HLA-DQA1*03:01/DQB1*03:02	>0% - 50%	Dengue virus 4	ELISPOT	IFNg release	Positive	0	0	0	1	0	0	0	0	0	0	0	0	0	0	0	1	0	0	0	0	0	0	0	0	0	0	0	0	0	0	0	0	0	0	0	0	1	1	1	0	0	0
195551	IYVILTILTIIALVA	NS4a	2227	2241	HLA-DRB1*01:01	>0% - 50%	Dengue virus 4	ELISPOT	IFNg release	Positive	0	0	0	0	0	0	0	0	0	0	0	0	0	0	0	0	0	0	0	0	0	0	0	0	1	0	0	0	0	0	0	0	0	0	0	0	0	0	0	0	0	0
195575	KLSMGLIAIAVASGL	NS4a	2172	2186	HLA-DRB1*08:02	>0% - 50%	Dengue virus 4	ELISPOT	IFNg release	Positive	0	0	0	0	0	0	0	0	0	0	0	0	0	0	0	0	0	0	0	0	0	0	0	0	1	0	0	0	0	0	0	0	0	0	0	0	0	0	0	0	0	0
195648	LIYVILTILTIIALV	NS4a	2226	2240	HLA-DRB1*07:01	>0% - 50%	Dengue virus 4	ELISPOT	IFNg release	Positive	0	0	0	0	0	0	0	0	0	0	0	0	0	0	0	0	0	0	0	0	0	0	0	0	1	0	0	0	0	0	0	0	0	0	0	0	0	0	0	0	0	0
539313	GKAYQHALNELPESL	NS4a	2130	2144	HLA-DRB1*10:01	>0% - 50%	Dengue virus 4	ELISPOT	IFNg release	Positive	0	0	0	0	0	0	0	0	0	0	0	0	0	0	0	0	0	0	0	0	0	0	0	0	1	0	0	0	0	0	0	0	0	0	0	0	0	0	0	0	0	0
539149	DCVVKPLDERFSTSLLF	NS5	3152	3168	HLA class II	>0% - 50%	Dengue virus 4	ICS	IFNg release	Positive	0	0	0	0	0	0	0	0	0	0	0	0	0	0	0	0	0	0	0	0	0	0	0	0	1	0	0	0	0	0	0	0	0	0	0	0	0	0	0	0	0	0
866364	DERFSTSLLFLNDMG	NS5	3159	3173	HLA-DPB1*01:01	>0% - 50%	Dengue virus 4	ELISPOT	IFNg release	Positive	0	0	0	0	0	0	0	0	0	0	0	0	0	0	0	0	0	0	0	0	0	0	0	0	1	0	0	0	0	0	0	0	0	0	0	0	0	0	0	0	0	0

CD8 DENV-1 Epitopes

											NCBI IDs of DENV-1 circulating strains from Brazil, (100% identity=1,green ; else 0, red)																									DENV-1 constructs of vaccines					
Epitope_ID	AA_Seq	Protein_Name	Start	End	Allele	step	Serotype	Method/Technique	Assay Group	Qualitative Measure	ACO06167.1	ACO06161.1	ACO06170.1	AGN94867.1	AGN94869.1	AKQ00018.1	AKQ00012.1	AKQ00011.1	ACO06173.1	AGN94868.1	AGN94865.1	AGN94870.1	AKQ00039.1	AKQ00038.1	QOW96373.1	AKQ00016.1	AKQ00015.1	AKQ00013.1	QOW96372.1	QOW96385.1	ACO06164.1	ACY70762.1	AGN94866.1	AKQ00014.1	AKQ00010.1	TV003	TDEN	DPIV	Dengvaxia	DENVax	TVDV
184844	MLKRERNRV	C	15	23	HLA-B*08:01	0%	Dengue virus 1	ELISPOT	IFNg release	Positive	0	0	0	0	0	0	0	0	0	0	0	0	0	0	0	0	0	0	0	0	0	0	0	0	0	0	0	0	0	0	0
240082	MVLALITFL	C	24	32	HLA-A*02:01	0%	Dengue virus 1	ELISPOT	IFNg release	Positive	0	0	0	0	0	0	0	0	0	0	0	0	0	0	0	0	0	0	0	0	0	0	0	0	0	0	0	0	0	0	0
183245	FQSHQLWATL	NS2a	1267	1276	HLA-B*15:01	0%	Dengue virus 1	ELISPOT	IFNg release	Positive	0	0	0	0	0	0	0	0	0	0	0	0	0	0	0	0	0	0	0	0	0	0	0	0	0	1	1	1	0	0	0
183252	FSLHYAWKTM	NS2a	1286	1295	HLA-B*35:01	0%	Dengue virus 1	ELISPOT	IFNg release	Positive	0	0	0	0	0	0	0	0	0	0	0	0	0	0	0	0	0	0	0	0	0	0	0	0	0	1	1	1	1	0	0
184623	LTDFQSHQL	NS2a	1264	1272	HLA-A*01:01	0%	Dengue virus 1	ELISPOT	IFNg release	Positive	0	0	0	0	0	0	0	0	0	0	0	0	0	0	0	0	0	0	0	0	0	0	0	0	0	1	1	1	1	0	0
184736	MAMVLSIVS	NS2a	1295	1303	HLA-B*35:01	0%	Dengue virus 1	ELISPOT	IFNg release	Positive	0	0	0	0	0	0	0	0	0	0	0	0	0	0	0	0	0	0	0	0	0	0	0	0	0	0	0	0	1	0	0
419037	RPMFAVGILF	NS2a	1211	1220	HLA-B*35:01	0%	Dengue virus 1	ELISPOT	IFNg release	Positive	0	0	0	0	0	0	0	0	0	0	0	0	0	0	0	0	0	0	0	0	0	0	0	0	0	0	0	0	0	0	0
150254	GPLVAGGLL	NS2b	1375	1383	HLA-B*07:02	0%	Dengue virus 1	ELISPOT	IFNg release	Positive	0	0	0	0	0	0	0	0	0	0	0	0	0	0	0	0	0	0	0	0	0	0	0	0	0	0	0	0	0	0	0
180682	NPEIEDDIF	NS3	1650	1658	HLA-B*35:01	0%	Dengue virus 1	ELISPOT	IFNg release	Positive	0	0	0	0	0	0	0	0	0	0	0	0	0	0	0	0	0	0	0	0	0	0	0	0	0	0	0	0	0	0	0
32147	KLRTLVLAPTRVVAS	NS3	216	230	HLA class I	0%	Dengue virus 1	ELISPOT	IFNg release	Positive	0	0	0	0	0	0	0	0	0	0	0	0	0	0	0	0	0	0	0	0	0	0	0	0	0	1	1	1	0	0	0
539750	NVRTLILAPTRVVASEM	NS3	1690	1706	HLA class I	0%	Dengue virus 1	ICS	IFNg release	Positive	0	0	0	0	0	0	0	0	0	0	0	0	0	0	0	0	0	0	0	0	0	0	0	0	0	0	0	0	0	0	0
65549	TPEGIIPSM	NS3	1975	1983	HLA-B*53:01	0%	Dengue virus 1	51 chromium	cytotoxicity	Positive	0	0	0	0	0	0	0	0	0	0	0	0	0	0	0	0	0	0	0	0	0	0	0	0	0	0	0	0	0	0	0
184554	LPQHLTQRAQ	NS4a	2107	2116	HLA-B*35:01	0%	Dengue virus 1	ELISPOT	IFNg release	Positive	0	0	0	0	0	0	0	0	0	0	0	0	0	0	0	0	0	0	0	0	0	0	0	0	0	1	1	1	0	0	0
184739	MASSVLLWM	NS4a	2183	2191	HLA-B*35:01	0%	Dengue virus 1	ELISPOT	IFNg release	Positive	0	0	0	0	0	0	0	0	0	0	0	0	0	0	0	0	0	0	0	0	0	0	0	0	0	0	0	0	0	0	0
133716	TLYAVATTI	NS4b	2284	2292	HLA-A*02:01	0%	Dengue virus 1	ELISPOT	IFNg release	Positive	0	0	0	0	0	0	0	0	0	0	0	0	0	0	0	0	0	0	0	0	0	0	0	0	0	1	1	1	0	0	0
1068332	SSWEDVPYL	NS5	3323	3331	HLA-A*02:01	0%	Dengue virus 1	ELISPOT	IFNg release	Positive	0	0	0	0	0	0	0	0	0	0	0	0	0	0	0	0	0	0	0	0	0	0	0	0	0	1	1	1	0	0	0
150674	TVMDIISRR	NS5	3080	3088	HLA-A*11:01	0%	Dengue virus 1	multimer/tetramer	qualitative binding	Positive	0	0	0	0	0	0	0	0	0	0	0	0	0	0	0	0	0	0	0	0	0	0	0	0	0	0	0	0	0	0	0
184787	MEVQLVRQM	NS5	3107	3115	HLA-B*40:01	0%	Dengue virus 1	ELISPOT	IFNg release	Positive	0	0	0	0	0	0	0	0	0	0	0	0	0	0	0	0	0	0	0	0	0	0	0	0	0	0	0	0	0	0	0
150255	GPMKLVMAFI	C	43	52	HLA-B*07:02	100%	Dengue virus 1	ELISPOT	IFNg release	Positive	1	1	1	1	1	1	1	1	1	1	1	1	1	1	1	1	1	1	1	1	1	1	1	1	1	1	1	1	0	0	0
180637	MAFIAFLRF	C	48	56	HLA-B*35:01	100%	Dengue virus 1	ELISPOT	IFNg release	Positive	1	1	1	1	1	1	1	1	1	1	1	1	1	1	1	1	1	1	1	1	1	1	1	1	1	1	1	1	0	0	0
183175	FIAFLRFLA	C	50	58	HLA-A*68:02	100%	Dengue virus 1	ELISPOT	IFNg release	Positive	1	1	1	1	1	1	1	1	1	1	1	1	1	1	1	1	1	1	1	1	1	1	1	1	1	1	1	1	0	0	0
183220	FNMLKRARNR	C	14	23	HLA-A*33:01	100%	Dengue virus 1	ELISPOT	IFNg release	Positive	1	1	1	1	1	1	1	1	1	1	1	1	1	1	1	1	1	1	1	1	1	1	1	1	1	1	1	1	0	0	0
184458	LLMLLPTAL	C	83	91	HLA-B*08:01	100%	Dengue virus 1	ELISPOT	IFNg release	Positive	1	1	1	1	1	1	1	1	1	1	1	1	1	1	1	1	1	1	1	1	1	1	1	1	1	1	1	1	0	0	0
184835	MKLVMAFIAF	C	44	53	HLA-B*35:01	100%	Dengue virus 1	ELISPOT	IFNg release	Positive	1	1	1	1	1	1	1	1	1	1	1	1	1	1	1	1	1	1	1	1	1	1	1	1	1	1	1	1	0	0	0
185102	NRRKRSVTM	C	96	104	HLA-B*08:01	100%	Dengue virus 1	ELISPOT	IFNg release	Positive	1	1	1	1	1	1	1	1	1	1	1	1	1	1	1	1	1	1	1	1	1	1	1	1	1	1	1	1	0	0	0
185492	RRKRSVTMLL	C	97	106	HLA-B*08:01	100%	Dengue virus 1	ELISPOT	IFNg release	Positive	1	1	1	1	1	1	1	1	1	1	1	1	1	1	1	1	1	1	1	1	1	1	1	1	1	1	1	1	0	0	0
150378	LLMLVTPSM	PreM	271	279	HLA-B*08:01	100%	Dengue virus 1	ELISPOT	IFNg release	Positive	1	1	1	1	1	1	1	1	1	1	1	1	1	1	1	1	1	1	1	1	1	1	1	1	1	1	1	1	0	1	1
16554	FLDLPLPWT	E	213	221	HLA-A2	100%	Dengue virus 1	ICS	IFNg release	Positive	1	1	1	1	1	1	1	1	1	1	1	1	1	1	1	1	1	1	1	1	1	1	1	1	1	1	1	1	1	1	1
183421	GLNSRSTSL	E	747	755	HLA-B*08:01	100%	Dengue virus 1	ELISPOT	IFNg release	Positive	1	1	1	1	1	1	1	1	1	1	1	1	1	1	1	1	1	1	1	1	1	1	1	1	1	1	1	1	1	0	1
184377	LGLNSRSTSL	E	746	755	HLA-B*08:01	100%	Dengue virus 1	ELISPOT	IFNg release	Positive	1	1	1	1	1	1	1	1	1	1	1	1	1	1	1	1	1	1	1	1	1	1	1	1	1	1	1	1	1	0	1
184626	LTDYGALTL	E	456	464	HLA-A*01:01	100%	Dengue virus 1	ELISPOT	IFNg release	Positive	1	1	1	1	1	1	1	1	1	1	1	1	1	1	1	1	1	1	1	1	1	1	1	1	1	1	1	1	1	0	1
185212	PTSEIQLTDY	E	449	458	HLA-A*01:01	100%	Dengue virus 1	ELISPOT	IFNg release	Positive	1	1	1	1	1	1	1	1	1	1	1	1	1	1	1	1	1	1	1	1	1	1	1	1	1	1	1	1	1	1	1
186524	WTMKIGIGIL	E	733	742	HLA-B*08:01	100%	Dengue virus 1	ELISPOT	IFNg release	Positive	1	1	1	1	1	1	1	1	1	1	1	1	1	1	1	1	1	1	1	1	1	1	1	1	1	1	1	1	0	1	1
186551	YENLKYSVIV	E	412	421	HLA-B*40:01	100%	Dengue virus 1	ELISPOT	IFNg release	Positive	1	1	1	1	1	1	1	1	1	1	1	1	1	1	1	1	1	1	1	1	1	1	1	1	1	1	1	1	1	1	1
419306	PTLDIELLK	E	319	327	HLA-A*11:01	100%	Dengue virus 1	ELISPOT	IFNg release	Positive	1	1	1	1	1	1	1	1	1	1	1	1	1	1	1	1	1	1	1	1	1	1	1	1	1	1	1	1	1	1	1
50596	QEGAMHTAL	E	536	544	HLA-A*02:01	100%	Dengue virus 1	ELISPOT	IFNg release	Positive	1	1	1	1	1	1	1	1	1	1	1	1	1	1	1	1	1	1	1	1	1	1	1	1	1	1	1	1	1	1	1
588005	RDFVEGLSGATWVDVVL	E	289	305	HLA class I	100%	Dengue virus 1	ICS	IFNg release	Positive	1	1	1	1	1	1	1	1	1	1	1	1	1	1	1	1	1	1	1	1	1	1	1	1	1	1	1	1	1	1	1
590597	VEGLSGATWVDVVLEHG	E	292	308	HLA class I	100%	Dengue virus 1	ICS	IFNg release	Positive-Intermediate	1	1	1	1	1	1	1	1	1	1	1	1	1	1	1	1	1	1	1	1	1	1	1	1	1	1	1	1	1	1	1
150258	GPWHLGKLEL	NS1	1041	1050	HLA-B*07:02	100%	Dengue virus 1	ELISPOT	IFNg release	Positive	1	1	1	1	1	1	1	1	1	1	1	1	1	1	1	1	1	1	1	1	1	1	1	1	1	1	1	1	0	0	0
183619	HTWTEQYKF	NS1	801	809	HLA-B57	100%	Dengue virus 1	51 chromium	cytotoxicity	Positive	1	1	1	1	1	1	1	1	1	1	1	1	1	1	1	1	1	1	1	1	1	1	1	1	1	1	1	1	0	0	0
60104	SPKRLSAAI	NS1	813	821	HLA-B*08:01	100%	Dengue virus 1	ELISPOT	IFNg release	Positive	1	1	1	1	1	1	1	1	1	1	1	1	1	1	1	1	1	1	1	1	1	1	1	1	1	1	1	1	0	0	0
185468	RPMFAVGLLF	NS2a	1211	1220	HLA-B*35:01	100%	Dengue virus 1	ELISPOT	IFNg release	Positive	1	1	1	1	1	1	1	1	1	1	1	1	1	1	1	1	1	1	1	1	1	1	1	1	1	1	1	1	0	0	0
185651	SILLSSLLK	NS2b	1360	1368	HLA-A*11:01	100%	Dengue virus 1	ELISPOT	IFNg release	Positive	1	1	1	1	1	1	1	1	1	1	1	1	1	1	1	1	1	1	1	1	1	1	1	1	1	1	1	1	1	0	0
419005	LLAVSGVYPM	NS2b	1448	1457	HLA-B*15:01	100%	Dengue virus 1	ELISPOT	IFNg release	Positive	1	1	1	1	1	1	1	1	1	1	1	1	1	1	1	1	1	1	1	1	1	1	1	1	1	0	0	0	0	0	0
13346	EMAEALKGMPIRYQT	NS3	231	245	HLA class I	100%	Dengue virus 1	ELISPOT	IFNg release	Positive	1	1	1	1	1	1	1	1	1	1	1	1	1	1	1	1	1	1	1	1	1	1	1	1	1	1	1	1	0	0	0
150169	DPRRCLKPV	NS3	1900	1908	HLA-B*07:02	100%	Dengue virus 1	ELISPOT	IFNg release	Positive	1	1	1	1	1	1	1	1	1	1	1	1	1	1	1	1	1	1	1	1	1	1	1	1	1	1	1	1	0	0	0
150341	KPGTSGSPI	NS3	1606	1614	HLA-B*07:02	100%	Dengue virus 1	ELISPOT	IFNg release	Positive	1	1	1	1	1	1	1	1	1	1	1	1	1	1	1	1	1	1	1	1	1	1	1	1	1	1	1	1	0	0	0
150538	RPRWLDART	NS3	2068	2076	HLA-B*07:02	100%	Dengue virus 1	ELISPOT	IFNg release	Positive	1	1	1	1	1	1	1	1	1	1	1	1	1	1	1	1	1	1	1	1	1	1	1	1	1	1	1	1	0	0	0
150556	RVIDPRRCLK	NS3	1897	1906	HLA-A*03:01	100%	Dengue virus 1	ELISPOT	IFNg release	Positive	1	1	1	1	1	1	1	1	1	1	1	1	1	1	1	1	1	1	1	1	1	1	1	1	1	1	1	1	0	0	0
150643	TPEGIIPALF	NS3	1976	1985	HLA-B*35:01	100%	Dengue virus 1	ELISPOT	IFNg release	Positive	1	1	1	1	1	1	1	1	1	1	1	1	1	1	1	1	1	1	1	1	1	1	1	1	1	1	1	1	0	0	0
180373	APTRVVASEM	NS3	1698	1707	HLA class I	100%	Dengue virus 1	ELISPOT	IFNg release	Positive	1	1	1	1	1	1	1	1	1	1	1	1	1	1	1	1	1	1	1	1	1	1	1	1	1	1	1	1	0	0	0
180408	DPASIAARGY	NS3	1766	1775	HLA-B*35:01	100%	Dengue virus 1	ELISPOT	IFNg release	Positive	1	1	1	1	1	1	1	1	1	1	1	1	1	1	1	1	1	1	1	1	1	1	1	1	1	1	1	1	0	0	0
180423	EERDIPERSW	NS3	1811	1820	HLA-B*44:02	100%	Dengue virus 1	ELISPOT	IFNg release	Positive	1	1	1	1	1	1	1	1	1	1	1	1	1	1	1	1	1	1	1	1	1	1	1	1	1	1	1	1	0	0	0
180472	GEARKTFVEL	NS3	2003	2012	HLA-B*40:01	100%	Dengue virus 1	ELISPOT	IFNg release	Positive	1	1	1	1	1	1	1	1	1	1	1	1	1	1	1	1	1	1	1	1	1	1	1	1	1	1	1	1	0	0	0
180670	MPVTVASAAQ	NS3	1923	1932	HLA-B*35:01	100%	Dengue virus 1	ELISPOT	IFNg release	Positive	1	1	1	1	1	1	1	1	1	1	1	1	1	1	1	1	1	1	1	1	1	1	1	1	1	1	1	1	0	0	0
180804	VPNYNMIIM	NS3	1751	1759	HLA-B*51:01	100%	Dengue virus 1	ELISPOT	IFNg release	Positive	1	1	1	1	1	1	1	1	1	1	1	1	1	1	1	1	1	1	1	1	1	1	1	1	1	1	1	1	0	0	0
182519	AIKRKLRTL	NS3	1687	1695	HLA-B*08:01	100%	Dengue virus 1	ELISPOT	IFNg release	Positive	1	1	1	1	1	1	1	1	1	1	1	1	1	1	1	1	1	1	1	1	1	1	1	1	1	1	1	1	0	0	0
182772	DISEMGANF	NS3	1885	1893	HLA-A*26:01	100%	Dengue virus 1	ELISPOT	IFNg release	Positive	1	1	1	1	1	1	1	1	1	1	1	1	1	1	1	1	1	1	1	1	1	1	1	1	1	1	1	1	0	0	0
183223	FPGKTVWFVP	NS3	1830	1839	HLA-B*35:01	100%	Dengue virus 1	ELISPOT	IFNg release	Positive	1	1	1	1	1	1	1	1	1	1	1	1	1	1	1	1	1	1	1	1	1	1	1	1	1	1	1	1	0	0	0
183598	HPGSGKTRRY	NS3	1670	1679	HLA-B*35:01	100%	Dengue virus 1	ELISPOT	IFNg release	Positive	1	1	1	1	1	1	1	1	1	1	1	1	1	1	1	1	1	1	1	1	1	1	1	1	1	1	1	1	0	0	0
183929	IYRILQRGLL	NS3	1497	1506	HLA-B*08:01	100%	Dengue virus 1	ELISPOT	IFNg release	Positive	1	1	1	1	1	1	1	1	1	1	1	1	1	1	1	1	1	1	1	1	1	1	1	1	1	1	1	1	0	0	0
184316	LEENMDVEIW	NS3	2049	2058	HLA-B*44:03	100%	Dengue virus 1	ELISPOT	IFNg release	Positive	1	1	1	1	1	1	1	1	1	1	1	1	1	1	1	1	1	1	1	1	1	1	1	1	1	1	1	1	0	0	0
184884	MPIRYQTTAV	NS3	1714	1723	HLA-B*35:01	100%	Dengue virus 1	ELISPOT	IFNg release	Positive	1	1	1	1	1	1	1	1	1	1	1	1	1	1	1	1	1	1	1	1	1	1	1	1	1	1	1	1	0	0	0
184896	MPVTVASAA	NS3	1923	1931	HLA-B*35:01	100%	Dengue virus 1	ELISPOT	IFNg release	Positive	1	1	1	1	1	1	1	1	1	1	1	1	1	1	1	1	1	1	1	1	1	1	1	1	1	1	1	1	0	0	0
185124	NTPEGIIPA	NS3	1975	1983	HLA-A*68:02	100%	Dengue virus 1	ELISPOT	IFNg release	Positive	1	1	1	1	1	1	1	1	1	1	1	1	1	1	1	1	1	1	1	1	1	1	1	1	1	1	1	1	0	0	0
185125	NTPEGIIPAL	NS3	1975	1984	HLA-A*68:02	100%	Dengue virus 1	ELISPOT	IFNg release	Positive	1	1	1	1	1	1	1	1	1	1	1	1	1	1	1	1	1	1	1	1	1	1	1	1	1	1	1	1	0	0	0
185741	SPVRVPNYNM	NS3	1747	1756	HLA-B*35:01	100%	Dengue virus 1	ELISPOT	IFNg release	Positive	1	1	1	1	1	1	1	1	1	1	1	1	1	1	1	1	1	1	1	1	1	1	1	1	1	1	1	1	0	0	0
185827	SYKVASEGF	NS3	2023	2031	HLA-A*24:02	100%	Dengue virus 1	ELISPOT	IFNg release	Positive	1	1	1	1	1	1	1	1	1	1	1	1	1	1	1	1	1	1	1	1	1	1	1	1	1	1	1	1	0	0	0
186284	VLDDGIYRI	NS3	1492	1500	HLA-A*02:01	100%	Dengue virus 1	ELISPOT	IFNg release	Positive	1	1	1	1	1	1	1	1	1	1	1	1	1	1	1	1	1	1	1	1	1	1	1	1	1	1	1	1	0	0	0
186559	YKVASEGFQY	NS3	2024	2033	HLA-B*35:01	100%	Dengue virus 1	ELISPOT	IFNg release	Positive	1	1	1	1	1	1	1	1	1	1	1	1	1	1	1	1	1	1	1	1	1	1	1	1	1	1	1	1	0	0	0
186603	YSDPLALREF	NS3	2077	2086	HLA-B*35:01	100%	Dengue virus 1	ELISPOT	IFNg release	Positive	1	1	1	1	1	1	1	1	1	1	1	1	1	1	1	1	1	1	1	1	1	1	1	1	1	1	1	1	0	0	0
22825	GTSGSPIVNR	NS3	133	142	HLA-B*57:01	100%	Dengue virus 1	51 chromium	cytotoxicity	Positive	1	1	1	1	1	1	1	1	1	1	1	1	1	1	1	1	1	1	1	1	1	1	1	1	1	1	1	1	0	0	0
35237	LDNINTPEGIIPALFEPERE	NS3	496	515	HLA class I	100%	Dengue virus 1	ELISPOT	IFNg release	Positive	1	1	1	1	1	1	1	1	1	1	1	1	1	1	1	1	1	1	1	1	1	1	1	1	1	1	1	1	0	0	0
38766	LPVWLSYKV	NS3	2018	2026	HLA-B*51:01	100%	Dengue virus 1	ELISPOT	IFNg release	Positive	1	1	1	1	1	1	1	1	1	1	1	1	1	1	1	1	1	1	1	1	1	1	1	1	1	1	1	1	0	0	0
39095	LRGEARKTFVELMRR	NS3	526	540	HLA class I	100%	Dengue virus 1	ELISPOT	IFNg release	Positive	1	1	1	1	1	1	1	1	1	1	1	1	1	1	1	1	1	1	1	1	1	1	1	1	1	1	1	1	0	0	0
419024	NMIIMDEAHF	NS3	1755	1764	HLA-B*15:01	100%	Dengue virus 1	ELISPOT	IFNg release	Positive	1	1	1	1	1	1	1	1	1	1	1	1	1	1	1	1	1	1	1	1	1	1	1	1	1	1	1	1	0	0	0
419124	AVEPGKNPK	NS3	1574	1582	HLA-A*11:01	100%	Dengue virus 1	ELISPOT	IFNg release	Positive	1	1	1	1	1	1	1	1	1	1	1	1	1	1	1	1	1	1	1	1	1	1	1	1	1	1	1	1	0	0	0
419229	IYRILQRGL	NS3	1497	1505	HLA-A*24:02	100%	Dengue virus 1	ELISPOT	IFNg release	Positive	1	1	1	1	1	1	1	1	1	1	1	1	1	1	1	1	1	1	1	1	1	1	1	1	1	1	1	1	0	0	0
50894	QGKRLEPSWASVKKDLISYG	NS3	1536	1555	HLA class I	100%	Dengue virus 1	ELISPOT	IFNg release	Positive	1	1	1	1	1	1	1	1	1	1	1	1	1	1	1	1	1	1	1	1	1	1	1	1	1	1	1	1	0	0	0
539753	NYNMIIMDEAHFTDPA	NS3	1753	1768	HLA class I	100%	Dengue virus 1	ICS	IFNg release	Positive	1	1	1	1	1	1	1	1	1	1	1	1	1	1	1	1	1	1	1	1	1	1	1	1	1	1	1	1	0	0	0
56309	RVIDPRRCL	NS3	1897	1905	HLA-B*07:02	100%	Dengue virus 1	ELISPOT	IFNg release	Positive	1	1	1	1	1	1	1	1	1	1	1	1	1	1	1	1	1	1	1	1	1	1	1	1	1	1	1	1	0	0	0
585710	LAPTRVVASEM	NS3	83	93	HLA class I	100%	Dengue virus 1	ICS	IFNg release	Positive-High	1	1	1	1	1	1	1	1	1	1	1	1	1	1	1	1	1	1	1	1	1	1	1	1	1	1	1	1	0	0	0
65546	TPEGIIPAL	NS3	1976	1984	HLA class I	100%	Dengue virus 1	51 chromium	cytotoxicity	Positive	1	1	1	1	1	1	1	1	1	1	1	1	1	1	1	1	1	1	1	1	1	1	1	1	1	1	1	1	0	0	0
150649	TPQDNQLAYV	NS4a	2222	2231	HLA-B*07:02	100%	Dengue virus 1	ELISPOT	IFNg release	Positive	1	1	1	1	1	1	1	1	1	1	1	1	1	1	1	1	1	1	1	1	1	1	1	1	1	1	1	1	0	0	0
180780	TPQDNQLAY	NS4a	2222	2230	HLA-B*35:01	100%	Dengue virus 1	ELISPOT	IFNg release	Positive	1	1	1	1	1	1	1	1	1	1	1	1	1	1	1	1	1	1	1	1	1	1	1	1	1	1	1	1	0	0	0
182912	EFFLMVLLI	NS4a	2206	2214	HLA-A*24:02	100%	Dengue virus 1	ELISPOT	IFNg release	Positive	1	1	1	1	1	1	1	1	1	1	1	1	1	1	1	1	1	1	1	1	1	1	1	1	1	1	1	1	0	0	0
183162	FFLMVLLIPE	NS4a	2207	2216	HLA-A*33:01	100%	Dengue virus 1	ELISPOT	IFNg release	Positive	1	1	1	1	1	1	1	1	1	1	1	1	1	1	1	1	1	1	1	1	1	1	1	1	1	1	1	1	0	0	0
183813	IPEPDRQRTP	NS4a	2214	2223	HLA-B*35:01	100%	Dengue virus 1	ELISPOT	IFNg release	Positive	1	1	1	1	1	1	1	1	1	1	1	1	1	1	1	1	1	1	1	1	1	1	1	1	1	1	1	1	0	0	0
184522	LPDTIETLM	NS4a	2142	2150	HLA-B*35:01	100%	Dengue virus 1	ELISPOT	IFNg release	Positive	1	1	1	1	1	1	1	1	1	1	1	1	1	1	1	1	1	1	1	1	1	1	1	1	1	1	1	1	0	0	0
184523	LPDTIETLML	NS4a	2142	2151	HLA-B*35:01	100%	Dengue virus 1	ELISPOT	IFNg release	Positive	1	1	1	1	1	1	1	1	1	1	1	1	1	1	1	1	1	1	1	1	1	1	1	1	1	1	1	1	0	0	0
41986	MLLALIAVL	NS4a	2150	2158	HLA-A2	100%	Dengue virus 1	ICS	IFNg release	Positive	1	1	1	1	1	1	1	1	1	1	1	1	1	1	1	1	1	1	1	1	1	1	1	1	1	1	1	1	0	0	0
150579	SPGKFWNTTI	NS4b	2453	2462	HLA-B*07:02	100%	Dengue virus 1	ELISPOT	IFNg release	Positive	1	1	1	1	1	1	1	1	1	1	1	1	1	1	1	1	1	1	1	1	1	1	1	1	1	1	1	1	0	0	0
180382	ATGPLTTLW	NS4b	2442	2450	HLA-B*58:01	100%	Dengue virus 1	ELISPOT	IFNg release	Positive	1	1	1	1	1	1	1	1	1	1	1	1	1	1	1	1	1	1	1	1	1	1	1	1	1	1	1	1	0	0	0
186427	VVYDAKFEK	NS4b	2403	2411	HLA-A*11:01	100%	Dengue virus 1	ELISPOT	IFNg release	Positive	1	1	1	1	1	1	1	1	1	1	1	1	1	1	1	1	1	1	1	1	1	1	1	1	1	1	1	1	0	0	0
27180	ILLMRTTWA	NS4b	2426	2434	HLA-A2	100%	Dengue virus 1	ICS	IFNg release	Positive	1	1	1	1	1	1	1	1	1	1	1	1	1	1	1	1	1	1	1	1	1	1	1	1	1	1	1	1	0	0	0
41093	MANIFRGSY	NS4b	2466	2474	HLA-B*35:01	100%	Dengue virus 1	ELISPOT	IFNg release	Positive	1	1	1	1	1	1	1	1	1	1	1	1	1	1	1	1	1	1	1	1	1	1	1	1	1	1	1	1	0	0	0
64286	TIENTTANISLTAIA	NS4b	2300	2314	HLA class I	100%	Dengue virus 1	ELISPOT	IFNg release	Positive	1	1	1	1	1	1	1	1	1	1	1	1	1	1	1	1	1	1	1	1	1	1	1	1	1	1	1	1	0	0	0
69652	VLMLVAHYA	NS4b	2356	2364	HLA-A2	100%	Dengue virus 1	ICS	IFNg release	Positive	1	1	1	1	1	1	1	1	1	1	1	1	1	1	1	1	1	1	1	1	1	1	1	1	1	1	1	1	0	0	0
74558	YLAGAGLAF	NS4b	2474	2482	HLA-B*07:02	100%	Dengue virus 1	ELISPOT	IFNg release	Positive	1	1	1	1	1	1	1	1	1	1	1	1	1	1	1	1	1	1	1	1	1	1	1	1	1	1	1	1	0	0	0
133702	SRNSTHEMY	NS5	2703	2711	HLA-A*02:01	100%	Dengue virus 1	ELISPOT	IFNg release	Positive	1	1	1	1	1	1	1	1	1	1	1	1	1	1	1	1	1	1	1	1	1	1	1	1	1	1	1	1	0	0	0
14325	ETACLGKSY	NS5	3242	3250	HLA-A*26:01	100%	Dengue virus 1	ELISPOT	IFNg release	Positive	1	1	1	1	1	1	1	1	1	1	1	1	1	1	1	1	1	1	1	1	1	1	1	1	1	1	1	1	0	0	0
150308	IPMATYGWNL	NS5	2607	2616	HLA-B*07:02	100%	Dengue virus 1	ELISPOT	IFNg release	Positive	1	1	1	1	1	1	1	1	1	1	1	1	1	1	1	1	1	1	1	1	1	1	1	1	1	1	1	1	0	0	0
180376	ASSMVNGVVR	NS5	2809	2818	HLA-B*57:01	100%	Dengue virus 1	ELISPOT	IFNg release	Positive	1	1	1	1	1	1	1	1	1	1	1	1	1	1	1	1	1	1	1	1	1	1	1	1	1	1	1	1	0	0	0
180411	DTTPFGQQR	NS5	2837	2845	HLA-A*68:01	100%	Dengue virus 1	ELISPOT	IFNg release	Positive	1	1	1	1	1	1	1	1	1	1	1	1	1	1	1	1	1	1	1	1	1	1	1	1	1	1	1	1	0	0	0
180548	KAKGSRAIW	NS5	2959	2967	HLA-B*57:01	100%	Dengue virus 1	ELISPOT	IFNg release	Positive	1	1	1	1	1	1	1	1	1	1	1	1	1	1	1	1	1	1	1	1	1	1	1	1	1	1	1	1	0	0	0
180571	KPRICTREEF	NS5	2882	2891	HLA-B*07:02	100%	Dengue virus 1	ELISPOT	IFNg release	Positive	1	1	1	1	1	1	1	1	1	1	1	1	1	1	1	1	1	1	1	1	1	1	1	1	1	1	1	1	0	0	0
180723	RFLEFEALGF	NS5	2974	2983	HLA-A*23:01	100%	Dengue virus 1	ELISPOT	IFNg release	Positive	1	1	1	1	1	1	1	1	1	1	1	1	1	1	1	1	1	1	1	1	1	1	1	1	1	1	1	1	0	0	0
182554	ALLATSIFK	NS5	3054	3062	HLA-A*11:01	100%	Dengue virus 1	ELISPOT	IFNg release	Positive	1	1	1	1	1	1	1	1	1	1	1	1	1	1	1	1	1	1	1	1	1	1	1	1	1	1	1	1	0	0	0
182599	AQMWQLMYF	NS5	3251	3259	HLA-B*15:01	100%	Dengue virus 1	ELISPOT	IFNg release	Positive	1	1	1	1	1	1	1	1	1	1	1	1	1	1	1	1	1	1	1	1	1	1	1	1	1	1	1	1	0	0	0
182836	DYMTSMKRF	NS5	3373	3381	HLA-A*24:02	100%	Dengue virus 1	ELISPOT	IFNg release	Positive	1	1	1	1	1	1	1	1	1	1	1	1	1	1	1	1	1	1	1	1	1	1	1	1	1	0	0	0	0	0	0
183147	FCSHHFHQL	NS5	3200	3208	HLA-B*08:01	100%	Dengue virus 1	ELISPOT	IFNg release	Positive	1	1	1	1	1	1	1	1	1	1	1	1	1	1	1	1	1	1	1	1	1	1	1	1	1	1	1	1	0	0	0
183275	FTNMEVQLIR	NS5	3104	3113	HLA-B*35:01	100%	Dengue virus 1	ELISPOT	IFNg release	Positive	1	1	1	1	1	1	1	1	1	1	1	1	1	1	1	1	1	1	1	1	1	1	1	1	1	0	0	0	0	0	0
183828	IPMVTQIAM	NS5	2827	2835	HLA-B*07:02	100%	Dengue virus 1	ELISPOT	IFNg release	Positive	1	1	1	1	1	1	1	1	1	1	1	1	1	1	1	1	1	1	1	1	1	1	1	1	1	1	1	1	0	0	0
184849	MLLNRFTMA	NS5	24	32	HLA-B*08:01	100%	Dengue virus 1	ELISPOT	IFNg release	Positive	1	1	1	1	1	1	1	1	1	1	1	1	1	1	1	1	1	1	1	1	1	1	1	1	1	1	1	1	0	0	0
184898	MQRKHGGML	NS5	2689	2697	HLA-B*08:01	100%	Dengue virus 1	ELISPOT	IFNg release	Positive	1	1	1	1	1	1	1	1	1	1	1	1	1	1	1	1	1	1	1	1	1	1	1	1	1	1	1	1	0	0	0
184956	MTTEDMLSVW	NS5	3296	3305	HLA-B*58:01	100%	Dengue virus 1	ICS	IFNg release	Positive	1	1	1	1	1	1	1	1	1	1	1	1	1	1	1	1	1	1	1	1	1	1	1	1	1	1	1	1	0	0	0
186150	TVMDVISRR	NS5	3080	3088	HLA-A*68:01	100%	Dengue virus 1	ELISPOT	IFNg release	Positive	1	1	1	1	1	1	1	1	1	1	1	1	1	1	1	1	1	1	1	1	1	1	1	1	1	1	1	1	0	0	0
186176	TWSIHAHHQW	NS5	3286	3295	HLA-A*23:01	100%	Dengue virus 1	ELISPOT	IFNg release	Positive	1	1	1	1	1	1	1	1	1	1	1	1	1	1	1	1	1	1	1	1	1	1	1	1	1	1	1	1	0	0	0
186217	VEDERFWDL	NS5	2918	2926	HLA-B*40:01	100%	Dengue virus 1	ELISPOT	IFNg release	Positive	1	1	1	1	1	1	1	1	1	1	1	1	1	1	1	1	1	1	1	1	1	1	1	1	1	1	1	1	0	0	0
419208	GVEGEGLHK	NS5	2998	3006	HLA-A*11:01	100%	Dengue virus 1	ELISPOT	IFNg release	Positive-High	1	1	1	1	1	1	1	1	1	1	1	1	1	1	1	1	1	1	1	1	1	1	1	1	1	1	1	1	0	0	0
5223	ATYGWNLVK	NS5	2610	2618	HLA-A*03:01	100%	Dengue virus 1	ELISPOT	IFNg release	Positive	1	1	1	1	1	1	1	1	1	1	1	1	1	1	1	1	1	1	1	1	1	1	1	1	1	1	1	1	0	0	0
539435	ISGDDCVVKPIDDRFAT	NS5	3152	3168	HLA class I	100%	Dengue virus 1	ICS	IFNg release	Positive	1	1	1	1	1	1	1	1	1	1	1	1	1	1	1	1	1	1	1	1	1	1	1	1	1	1	1	1	0	0	0
539702	MVTQIAMTDTTPFGQQR	NS5	2829	2845	HLA class I	100%	Dengue virus 1	ICS	IFNg release	Positive	1	1	1	1	1	1	1	1	1	1	1	1	1	1	1	1	1	1	1	1	1	1	1	1	1	1	1	1	0	0	0
65567	TPFGQQRVF	NS5	2839	2847	HLA class I	100%	Dengue virus 1	ELISPOT	IFNg release	Positive	1	1	1	1	1	1	1	1	1	1	1	1	1	1	1	1	1	1	1	1	1	1	1	1	1	1	1	1	0	0	0
67362	TYGWNLVKL	NS5	2611	2619	HLA-A*24:02	100%	Dengue virus 1	ELISPOT	IFNg release	Positive	1	1	1	1	1	1	1	1	1	1	1	1	1	1	1	1	1	1	1	1	1	1	1	1	1	1	1	1	0	0	0
73076	WSIHAHHQW	NS5	3287	3295	HLA-B*57:01	100%	Dengue virus 1	ELISPOT	IFNg release	Positive	1	1	1	1	1	1	1	1	1	1	1	1	1	1	1	1	1	1	1	1	1	1	1	1	1	1	1	1	0	0	0
183654	IAVGMVTLY	E	760	768	HLA-B*35:01	81% - <100%	Dengue virus 1	ELISPOT	IFNg release	Positive	1	1	1	1	1	1	1	1	1	1	1	1	1	1	1	1	1	1	0	0	1	1	1	1	1	1	1	1	0	1	1
185893	TEVTNPAVL	E	329	337	HLA-B*40:01	81% - <100%	Dengue virus 1	ELISPOT	IFNg release	Positive	1	1	1	1	1	0	1	1	1	1	1	1	1	0	0	1	1	1	0	1	1	1	1	1	1	1	1	1	1	1	1
183246	FRRLTSREVL	NS2a	1220	1229	HLA-B*08:01	81% - <100%	Dengue virus 1	ELISPOT	IFNg release	Positive	1	1	0	1	1	1	1	1	1	1	1	1	1	1	1	1	0	1	1	1	0	1	1	1	1	1	1	1	0	0	0

184274	LALMATFKM	NS2a	1202	1210	HLA-B*35:01	81% - <100%	Dengue virus 1	ELISPOT	IFNg release	Positive	1	1	1	0	1	1	1	1	1	1	0	1	1	1	1	1	1	1	1	1	1	1	0	1	1	0	0	0	0	0	0
184856	MLMTGTLAVF	NS2a	1158	1167	HLA-B*35:01	81% - <100%	Dengue virus 1	ELISPOT	IFNg release	Positive	0	1	1	1	1	1	1	1	1	1	1	1	1	1	1	1	1	1	1	1	1	1	1	1	1	1	1	1	0	0	0
133501	AIVREAIKR	NS3	1682	1690	HLA-A*02:01	81% - <100%	Dengue virus 1	ELISPOT	IFNg release	Positive	1	1	1	1	1	1	1	1	1	1	1	1	0	1	1	1	1	1	1	1	1	1	1	1	1	1	1	1	0	0	0
183229	FPQSNAVIQD	NS3	1801	1810	HLA-B*35:01	81% - <100%	Dengue virus 1	ELISPOT	IFNg release	Positive	1	1	1	1	1	1	1	1	1	1	1	1	0	1	1	0	1	1	1	1	1	1	1	1	1	1	1	1	0	0	0
38400	LPAIVREAI	NS3	1680	1688	HLA-B*53:01	81% - <100%	Dengue virus 1	ELISPOT	IFNg release	Positive	1	1	1	1	1	1	1	1	1	1	1	1	0	1	1	1	1	1	1	1	1	1	1	1	1	1	1	1	0	0	0
419125	AVKSEHTGR	NS3	1722	1730	HLA-A*11:01	81% - <100%	Dengue virus 1	ELISPOT	IFNg release	Positive	1	1	1	1	1	1	1	1	0	1	1	1	1	1	1	1	1	1	1	1	1	1	1	1	1	0	0	0	0	0	0
539170	DLMCHATF	NS3	1734	1741	HLA-B*08:01	81% - <100%	Dengue virus 1	ICS	IFNg release	Positive	1	1	1	1	1	1	1	1	1	1	1	1	1	1	1	1	1	0	1	1	1	1	1	1	1	1	1	1	0	0	0
539663	MGEAAAIFMTATPPGSV	NS3	1782	1798	HLA class I	81% - <100%	Dengue virus 1	ICS	IFNg release	Positive	1	0	1	1	1	1	1	1	1	1	1	1	1	1	1	1	1	1	0	1	1	1	1	1	1	1	1	1	0	0	0
62184	SVKKDLISYGGGWRF	NS3	71	85	HLA class I	81% - <100%	Dengue virus 1	ELISPOT	IFNg release	Positive	1	1	1	1	1	1	1	1	1	1	1	1	1	1	0	1	1	1	1	1	1	1	1	1	1	1	1	1	0	0	0
419128	AYRHAMEEL	NS4a	2134	2142	HLA-A*24:02	81% - <100%	Dengue virus 1	ELISPOT	IFNg release	Positive-High	1	1	1	1	1	1	0	1	1	1	1	1	1	1	0	1	1	1	0	1	1	1	1	1	1	1	1	1	0	0	0
150474	NPLTLTAAV	NS4b	2348	2356	HLA-B*07:02	81% - <100%	Dengue virus 1	ELISPOT	IFNg release	Positive	1	1	0	1	1	1	1	1	1	1	1	1	1	1	1	1	1	1	1	1	0	1	1	1	1	1	1	1	0	0	0
184212	KVRKDIPQW	NS5	3178	3186	HLA-B*57:01	81% - <100%	Dengue virus 1	ELISPOT	IFNg release	Positive	1	1	1	1	0	1	1	1	1	1	1	1	1	1	1	1	1	1	1	1	1	1	1	1	1	1	1	1	0	0	0
185279	QMQRKHGGML	NS5	2688	2697	HLA-B*08:01	81% - <100%	Dengue virus 1	ELISPOT	IFNg release	Positive	1	1	1	1	1	1	1	1	1	1	1	1	1	1	1	1	1	1	1	0	1	1	1	1	1	1	1	1	0	0	0
185354	REIVVPCRNQ	NS5	3214	3223	HLA-B*40:01	81% - <100%	Dengue virus 1	ELISPOT	IFNg release	Positive	1	1	0	1	1	1	1	1	1	1	1	1	1	1	1	1	1	1	1	1	0	1	1	1	1	1	1	1	0	0	0
540100	VVKPIDDRFATALTALN	NS5	3157	3173	HLA class I	81% - <100%	Dengue virus 1	ICS	IFNg release	Positive	1	1	1	1	1	1	1	1	1	1	1	1	1	1	0	1	1	1	0	1	1	1	1	1	1	1	1	1	0	0	0
62903	TAKWLWGFLSRNKKPRICTR	NS5	2869	2888	HLA class I	81% - <100%	Dengue virus 1	ELISPOT	IFNg release	Positive	1	1	0	1	1	1	1	1	1	1	1	1	1	1	1	1	1	1	1	1	1	1	1	1	0	0	0	0	0	0	0
72563	WHYDEDNPYKTWAYHGSYEV	NS5	2784	2803	HLA class I	81% - <100%	Dengue virus 1	ELISPOT	IFNg release	Positive	1	1	1	1	1	1	1	1	1	1	1	1	1	1	1	1	0	1	1	1	1	1	1	1	1	1	1	1	0	0	0
183596	HPGFTVIALF	PreM	244	253	HLA-B*35:01	51%-80%	Dengue virus 1	ELISPOT	IFNg release	Positive	1	1	1	0	1	1	0	0	1	1	1	0	1	1	1	1	1	0	1	1	1	1	0	1	1	1	1	1	0	1	1
180836	YGGPISQHNY	NS1	1022	1031	HLA-B*35:01	51%-80%	Dengue virus 1	ELISPOT	IFNg release	Positive	1	1	0	0	1	1	0	0	1	1	1	0	1	1	1	1	1	0	1	0	0	1	0	1	1	1	1	1	0	0	0
180641	MALSIVSLF	NS2a	1297	1305	HLA-B*51:01	51%-80%	Dengue virus 1	ELISPOT	IFNg release	Positive	1	1	1	0	1	1	0	0	0	1	1	0	1	1	1	1	1	0	1	0	1	1	0	1	1	0	0	0	0	0	0
185327	QYIYMGQPL	NS3	1947	1955	HLA-A*24:02	51%-80%	Dengue virus 1	ELISPOT	IFNg release	Positive	1	1	1	1	1	1	0	0	0	1	1	0	1	1	1	1	1	0	1	0	1	1	0	1	1	1	1	1	0	0	0
183800	IMNRRKRSV	C	94	102	HLA-B*08:01	>0% - 50%	Dengue virus 1	ELISPOT	IFNg release	Positive	0	0	1	1	0	0	1	1	1	0	0	1	0	0	0	0	0	1	0	1	1	0	1	0	0	1	1	1	0	0	0
184003	KEISSMLNIM	C	86	95	HLA-B*40:01	>0% - 50%	Dengue virus 1	ELISPOT	IFNg release	Positive	0	0	1	1	0	0	1	1	1	0	0	1	0	0	0	0	0	1	0	1	1	0	1	0	0	0	0	0	0	0	0
184880	MNRRKRSVT	C	95	103	HLA-B*08:01	>0% - 50%	Dengue virus 1	ELISPOT	IFNg release	Positive	0	0	1	1	0	0	1	1	1	0	0	1	0	0	0	0	0	1	0	1	1	0	1	0	0	1	1	1	0	0	0
167826	TSGTYVSAIAQAKASQE	NS3	157	173	HLA class I	>0% - 50%	Dengue virus 1	ELISPOT	IFNg release	Positive	0	1	1	1	0	0	1	1	1	0	0	1	0	0	0	0	0	1	0	1	1	0	1	0	0	1	1	1	0	0	0
183594	HPASAWTLYA	NS4b	2278	2287	HLA-B*35:01	>0% - 50%	Dengue virus 1	ELISPOT	IFNg release	Positive	0	0	0	1	0	0	1	1	0	0	0	1	0	0	0	0	0	1	0	1	0	0	1	0	0	1	1	1	0	0	0
185485	RQMESEGIFL	NS5	3113	3122	HLA-B*15:01	>0% - 50%	Dengue virus 1	ELISPOT	IFNg release	Positive	0	0	0	1	0	0	1	1	1	1	0	1	0	0	0	0	0	1	0	1	0	0	1	0	0	0	0	0	0	0	0

CD8 DENV-2 Epitopes

											DENV-2 constructs of vaccines					
Epitope_ID	AA_Seq	Protein_Name	Start	End	Allele	step	Serotype	Method/Technique	Assay Group	Qualitative Measure	TV003	TDEN	DPIV	Dengvaxia	DENVax	TVDV
150399	LQMENKAWLV	E	231	240	HLA-B*08:01	0%	Dengue virus 2	ELISPOT	IFNg release	Positive	1	1	1	1	1	1
183748	ILIGVVITW	E	737	745	HLA-B*58:01	0%	Dengue virus 2	ELISPOT	IFNg release	Positive	0	0	0	0	0	0
184300	LATLRKYCI	E	53	61	HLA-B*08:01	0%	Dengue virus 2	ELISPOT	IFNg release	Positive	0	0	0	0	0	0
41407	MENKAWLVHRQWFLD	E	201	215	HLA-B*37:01	0%	Dengue virus 2	ICS	IFNg release	Positive	1	1	1	1	1	1
180496	GVFTTNIWLKLKEKQ	NS1	936	950	HLA class I	0%	Dengue virus 2	ICS	IFNg release	Positive	0	1	0	0	1	0
150391	LMMTTIGIVL	NS2a	1228	1237	HLA-B*08:01	0%	Dengue virus 2	ELISPOT	IFNg release	Positive	0	1	1	0	1	0
183741	ILCVPNAVIL	NS2a	1279	1288	HLA-B*08:01	0%	Dengue virus 2	ELISPOT	IFNg release	Positive	0	1	1	0	1	0
150381	LLVISGLFPV	NS2b	1448	1457	HLA-A*02:01	0%	Dengue virus 2	ELISPOT	IFNg release	Positive	0	1	1	0	1	0
150425	LVISGLFPV	NS2b	1449	1457	HLA-A*02:01	0%	Dengue virus 2	ELISPOT	IFNg release	Positive	0	1	1	0	1	0
180538	ISGLFPVSIPITAAA	NS2b	106	120	HLA class I	0%	Dengue virus 2	ICS	IFNg release	Positive	0	1	1	0	1	0
17802	FSPGTSGSPIIDKKG	NS3	130	144	HLA class I	0%	Dengue virus 2	ELISPOT	IFNg release	Positive	0	1	1	0	1	0
180750	SPGTSGSPIIDKKGK	NS3	131	145	HLA-A*11:01	0%	Dengue virus 2	ELISPOT	IFNg release	Positive	0	1	1	0	1	0
182892	EENMEVEIW	NS3	2049	2057	HLA-B*44:02	0%	Dengue virus 2	ELISPOT	IFNg release	Positive	0	0	0	0	0	0
184318	LEENMEVEIW	NS3	2048	2057	HLA-B*44:02	0%	Dengue virus 2	ELISPOT	IFNg release	Positive	0	0	0	0	0	0
185388	RIKQKGILGY	NS3	1499	1508	HLA-B*15:01	0%	Dengue virus 2	ELISPOT	IFNg release	Positive	0	1	1	0	1	0
22816	GTSGSPIADK	NS3	1608	1617	HLA-A*11:01	0%	Dengue virus 2	multimer/tetramer	qualitative binding	Positive	0	0	0	0	0	0
22817	GTSGSPIADKK	NS3	1608	1618	HLA-A11	0%	Dengue virus 2	ELISPOT	IFNg release	Positive	0	0	0	0	0	0
22818	GTSGSPIIDK	NS3	133	142	HLA-A*11:01	0%	Dengue virus 2	ELISPOT	IFNg release	Positive	0	1	1	0	1	0
22819	GTSGSPIIDKK	NS3	133	143	HLA-A11	0%	Dengue virus 2	51 chromium	cytotoxicity	Positive	0	1	1	0	1	0
22823	GTSGSPIVDK	NS3	133	142	HLA-A11	0%	Dengue virus 2	51 chromium	cytotoxicity	Positive	0	0	0	0	0	0
54176	RIKQKGIL	NS3	1499	1506	HLA-B8	0%	Dengue virus 2	ELISPOT	IFNg release	Positive	0	1	1	0	1	0
65548	TPEGIIPSL	NS3	1975	1983	HLA-B35	0%	Dengue virus 2	51 chromium	cytotoxicity	Positive	0	0	0	0	0	0
8910	DKKGKVVGL	NS3	1616	1624	HLA-B8	0%	Dengue virus 2	ELISPOT	IFNg release	Positive	0	1	1	0	1	0
150612	TAEAGGRAY	NS4a	2126	2134	HLA-B*35:01	0%	Dengue virus 2	ELISPOT	IFNg release	Positive	0	1	1	0	1	0
150679	VIDLDPIPY	NS4b	2395	2403	HLA-A*01:01	0%	Dengue virus 2	ELISPOT	IFNg release	Positive	0	1	1	0	1	0
16757	FLLVAHYAI	NS4b	112	120	HLA-A2	0%	Dengue virus 2	ICS	TNF release	Positive	0	0	1	0	1	0
180789	TVIDLDPIPYDPKFE	NS4b	2394	2408	HLA class I	0%	Dengue virus 2	ELISPOT	IFNg release	Positive	0	1	1	0	1	0
150227	FTMRHKKATY	NS5	2734	2743	HLA-B*35:01	0%	Dengue virus 2	ELISPOT	IFNg release	Positive	0	1	1	0	0	0
163620	TITEEIAVQ	NS5	3127	3135	HLA-A*02:01	0%	Dengue virus 2	ELISPOT	IFNg release	Positive	0	0	0	0	1	0
180579	KSYAQMWSLMYFHRR	NS5	3247	3261	HLA class I	0%	Dengue virus 2	ELISPOT	IFNg release	Positive	0	1	1	0	1	0
180828	YAQMWSLMYF	NS5	3249	3258	HLA-B*35:01	0%	Dengue virus 2	ELISPOT	IFNg release	Positive	0	1	1	0	1	0
184840	MLINRFTMR	NS5	2729	2737	HLA-B*08:01	0%	Dengue virus 2	ELISPOT	IFNg release	Positive	0	1	1	0	1	0
32939	KPWDIIPMV	NS5	2821	2829	HLA-B*55:02	0%	Dengue virus 2	51 chromium	cytotoxicity	Positive	0	0	0	0	0	0
32941	KPWDVLPMV	NS5	2821	2829	HLA class I	0%	Dengue virus 2	51 chromium	cytotoxicity	Positive	0	0	0	0	0	0
32942	KPWDVLPTV	NS5	2821	2829	HLA class I	0%	Dengue virus 2	51 chromium	cytotoxicity	Positive	0	0	0	0	0	0
539144	CVVKPLDDRFASALTAL	NS5	3156	3172	HLA class I	0%	Dengue virus 2	ICS	IFNg release	Positive	0	1	1	0	1	0
539436	ISGDDCVVKPLDDRFA	NS5	3151	3166	HLA class I	0%	Dengue virus 2	ICS	IFNg release	Positive	0	1	1	0	1	0
73428	YAQMWSLMY	NS5	3249	3257	HLA-B*15:01	0%	Dengue virus 2	ELISPOT	IFNg release	Positive	0	1	1	0	1	0
150252	GPLKLFMAL	C	42	50	HLA-B*08:01	100%	Dengue virus 2	ELISPOT	IFNg release	Positive	0	1	1	0	1	0
185399	RKKARSTPF	C	5	13	HLA-B*08:01	100%	Dengue virus 2	ELISPOT	IFNg release	Positive	0	0	0	0	0	0
150299	ILLTAVAPSM	PreM	270	279	HLA-B*08:01	100%	Dengue virus 2	ELISPOT	IFNg release	Positive	1	1	1	1	0	1
180588	LAYTIGTTHF	PreM	254	263	HLA-B*35:01	100%	Dengue virus 2	ELISPOT	IFNg release	Positive	1	1	1	1	1	1
183577	HFQRALIFIL	PreM	262	271	HLA-B*08:01	100%	Dengue virus 2	ELISPOT	IFNg release	Positive	1	1	1	1	1	1
186264	VGRQEKGKSL	PreM	128	137	HLA-B*08:01	100%	Dengue virus 2	ELISPOT	IFNg release	Positive	0	0	0	0	0	0
150456	MSYSMCTGKF	E	577	586	HLA-B*35:01	100%	Dengue virus 2	ELISPOT	IFNg release	Positive	1	1	1	1	1	1
150529	RLITVNPIV	E	630	638	HLA-A*02:01	100%	Dengue virus 2	ELISPOT	IFNg release	Positive	1	1	1	1	1	1
150531	RLRMDKLQL	E	566	574	HLA-B*08:01	100%	Dengue virus 2	ELISPOT	IFNg release	Positive	1	1	1	1	1	1
150725	WLVHRQWFL	E	486	494	HLA-B*08:01	100%	Dengue virus 2	ELISPOT	IFNg release	Positive	1	1	1	1	1	1
16553	FLDLPLPWL	E	213	221	HLA-A2	100%	Dengue virus 2	ICS	TNF release	Positive	1	1	1	1	1	1
180563	KILIGVIITWIGMNS	E	456	470	HLA class I	100%	Dengue virus 2	ICS	IFNg release	Positive	1	1	1	1	1	0
180618	LPLPWLPGAD	E	496	505	HLA-B*35:01	100%	Dengue virus 2	ELISPOT	IFNg release	Positive	1	1	1	1	1	1
180816	WGNGCGLFGKGGIVT	E	381	395	HLA class I	100%	Dengue virus 2	ICS	IFNg release	Positive	1	1	1	1	1	1
183437	GMNSRSTSL	E	747	755	HLA-B*08:01	100%	Dengue virus 2	ELISPOT	IFNg release	Positive	1	1	1	1	1	0
183708	IGMNSRSTSL	E	746	755	HLA-B*08:01	100%	Dengue virus 2	ELISPOT	IFNg release	Positive	1	1	1	1	1	0
183747	ILIGVIITW	E	737	745	HLA-B*58:01	100%	Dengue virus 2	ELISPOT	IFNg release	Positive	1	1	1	1	1	0
183840	IQKETLVTF	E	512	520	HLA-B*15:01	100%	Dengue virus 2	ELISPOT	IFNg release	Positive	1	1	1	1	1	1
183841	IQMSSGNLLF	E	550	559	HLA-A*23:01	100%	Dengue virus 2	ELISPOT	IFNg release	Positive	1	1	1	1	1	1
184337	LEKRHVLGRL	E	622	631	HLA-B*08:01	100%	Dengue virus 2	ELISPOT	IFNg release	Positive	1	1	1	1	1	1
184467	LLQMEDKAWL	E	478	487	HLA-B*08:01	100%	Dengue virus 2	ELISPOT	IFNg release	Positive	0	0	0	0	0	0
184571	LQLKGMSYSM	E	572	581	HLA-B*08:01	100%	Dengue virus 2	ELISPOT	IFNg release	Positive	1	1	1	1	1	1
184920	MSSGNLLFTG	E	552	561	HLA-B*58:01	100%	Dengue virus 2	ELISPOT	IFNg release	Positive	1	1	1	1	1	1
185758	SRSTSLSVSL	E	750	759	HLA-B*08:01	100%	Dengue virus 2	ELISPOT	IFNg release	Positive	1	1	1	1	0	0
185831	SYSMCTGKF	E	578	586	HLA-A*23:01	100%	Dengue virus 2	ELISPOT	IFNg release	Positive	1	1	1	1	1	1
51388	QLKGMSYSM	E	573	581	HLA-B*08:01	100%	Dengue virus 2	ELISPOT	IFNg release	Positive	1	1	1	1	1	1
180450	FAGPVSQHNY	NS1	1022	1031	HLA-B*35:01	100%	Dengue virus 2	ELISPOT	IFNg release	Positive	0	0	1	0	0	0
180689	NRAVHADMGYWIESA	NS1	966	980	HLA class I	100%	Dengue virus 2	ICS	IFNg release	Positive	0	1	1	0	1	0
182938	EKEENLVNSL	NS1	1115	1124	HLA-B*08:01	100%	Dengue virus 2	ELISPOT	IFNg release	Positive	0	1	1	0	1	0
184512	LMWKQITPEL	NS1	841	850	HLA-B*08:01	100%	Dengue virus 2	ELISPOT	IFNg release	Positive	0	1	1	0	1	0
185342	RAVHADMGY	NS1	192	200	HLA-B*58:01	100%	Dengue virus 2	ELISPOT	IFNg release	Positive	0	1	1	0	1	0
185465	RPLKEKEENL	NS1	1111	1120	HLA-B*08:01	100%	Dengue virus 2	ELISPOT	IFNg release	Positive	0	1	1	0	1	0
19225	GEDGCWYGM	NS1	1100	1108	HLA-B*40:01	100%	Dengue virus 2	ELISPOT	IFNg release	Positive	0	1	1	0	1	0
150206	FLEEMLRTRV	NS2a	1131	1140	HLA-A*02:01	100%	Dengue virus 2	ELISPOT	IFNg release	Positive	0	1	1	0	1	0
150415	LTDALALGM	NS2a	1250	1258	HLA-A*01:01	100%	Dengue virus 2	ELISPOT	IFNg release	Positive	0	1	1	0	1	0
150457	MTDDIGMGV	NS2a	1191	1199	HLA-A*01:01	100%	Dengue virus 2	ELISPOT	IFNg release	Positive	0	1	1	0	1	0
150540	RPTFAAGLLL	NS2a	1211	1220	HLA-B*07:02	100%	Dengue virus 2	ELISPOT	IFNg release	Positive	0	1	1	0	1	0
150640	TMTDDIGMGV	NS2a	1176	1185	HLA-A*02:01	100%	Dengue virus 2	ELISPOT	IFNg release	Positive	0	1	1	0	1	0
150741	YQLAVTIMA	NS2a	1157	1165	HLA-A*02:01	100%	Dengue virus 2	ELISPOT	IFNg release	Positive	0	1	1	0	1	0
183825	IPLALTIKGL	NS2a	1319	1328	HLA-B*08:01	100%	Dengue virus 2	ELISPOT	IFNg release	Positive	0	1	1	0	1	0
184864	MLRTRVGTK	NS2a	1149	1157	HLA-B*08:01	100%	Dengue virus 2	ELISPOT	IFNg release	Positive	0	1	1	0	1	0
186041	TRVGTKHAIL	NS2a	1152	1161	HLA-B*08:01	100%	Dengue virus 2	ELISPOT	IFNg release	Positive	0	1	1	0	1	0
150101	AAAWYLWEV	NS2b	1463	1471	HLA-A*02:01	100%	Dengue virus 2	ELISPOT	IFNg release	Positive	0	1	1	0	1	0
150584	SPILSITISE	NS2b	1416	1425	HLA-B*35:01	100%	Dengue virus 2	ELISPOT	IFNg release	Positive	0	1	1	0	1	0
150610	TAAAWYLWEV	NS2b	117	126	HLA-A*02:01	100%	Dengue virus 2	ELISPOT	IFNg release	Positive	0	1	1	0	1	0
180529	IPITAAAWY	NS2b	1459	1467	HLA-B*35:01	100%	Dengue virus 2	ELISPOT	IFNg release	Positive	0	1	1	0	1	0
180531	IPMTGPLVAG	NS2b	1371	1380	HLA-B*35:01	100%	Dengue virus 2	ELISPOT	IFNg release	Positive	0	1	1	0	1	0
180646	MAVGMVSIL	NS2b	1354	1362	HLA-B*35:01	100%	Dengue virus 2	ELISPOT	IFNg release	Positive	0	1	1	0	1	0
183819	IPITAAAWYL	NS2b	1459	1468	HLA-B*08:01	100%	Dengue virus 2	ELISPOT	IFNg release	Positive	0	1	1	0	1	0
11063	EAHFTDPASIAARGY	NS3	285	299	HLA class I	100%	Dengue virus 2	ELISPOT	IFNg release	Positive	0	1	1	0	1	0
11125	EALRGLPIR	NS3	233	241	HLA class I	100%	Dengue virus 2	ELISPOT	IFNg release	Positive	0	1	1	0	1	0
124391	KTFVDLMRR	NS3	2006	2014	HLA-A*11:01	100%	Dengue virus 2	ELISPOT	IFNg release	Positive	0	1	1	0	1	0
13358	EMEEALRGLPIRYQT	NS3	230	244	HLA class I	100%	Dengue virus 2	ELISPOT	IFNg release	Positive	0	1	1	0	1	0
136890	TPPGSRDPF	NS3	1792	1800	HLA-B*35:01	100%	Dengue virus 2	ELISPOT	IFNg release	Positive	0	1	1	0	1	0
150137	APTRVVAAEM	NS3	222	231	HLA-B7	100%	Dengue virus 2	multimer/tetramer	qualitative binding	Positive	0	1	1	0	1	0
150167	DLMRRGDLPV	NS3	2010	2019	HLA-A*02:01	100%	Dengue virus 2	ELISPOT	IFNg release	Positive	0	1	1	0	1	0
150217	FPQSNAPIM	NS3	1800	1808	HLA-B*35:01	100%	Dengue virus 2	ELISPOT	IFNg release	Positive	0	0	1	0	0	0
150450	MPVTHSSAA	NS3	1922	1930	HLA-B*08:01	100%	Dengue virus 2	ELISPOT	IFNg release	Positive	0	1	1	0	1	0
150451	MPVTHSSAAQ	NS3	1922	1931	HLA-B*35:01	100%	Dengue virus 2	ELISPOT	IFNg release	Positive	0	1	1	0	1	0
150526	RIYSDPLALK	NS3	2074	2083	HLA-A*11:01	100%	Dengue virus 2	ELISPOT	IFNg release	Positive	0	1	1	0	1	0
150595	SQIGAGVYK	NS3	1509	1517	HLA-A*11:01	100%	Dengue virus 2	ELISPOT	IFNg release	Positive	0	1	1	0	1	0
150693	VPNYNLIIM	NS3	1750	1758	HLA-B*07:02	100%	Dengue virus 2	ELISPOT	IFNg release	Positive	0	1	1	0	1	0
150740	YLPAIVREA	NS3	1678	1686	HLA-A*02:01	100%	Dengue virus 2	ELISPOT	IFNg release	Positive	0	1	1	0	1	0
180428	EGEWKEGEEVQVLAL	NS3	1561	1575	HLA class I	100%	Dengue virus 2	ICS	IFNg release	Positive	0	1	1	0	1	0
180440	EREIPERSWNSGHEW	NS3	336	350	HLA-B*57:01	100%	Dengue virus 2	ELISPOT	IFNg release	Positive	0	1	0	0	1	0
180460	FPQSNAPIMD	NS3	1800	1809	HLA-B*35:01	100%	Dengue virus 2	ELISPOT	IFNg release	Positive	0	0	1	0	0	0
180471	GEARKTFVDL	NS3	2002	2011	HLA-B*40:01	100%	Dengue virus 2	ELISPOT	IFNg release	Positive	0	1	1	0	1	0
180503	HPGAGKTKRY	NS3	1669	1678	HLA-B*35:01	100%	Dengue virus 2	ELISPOT	IFNg release	Positive	0	1	1	0	1	0
180507	HTGREIVDLMCHATF	NS3	1726	1740	HLA class I	100%	Dengue virus 2	ELISPOT	IFNg release	Positive	0	1	1	0	1	0
180575	KRYLPAIVREAIKRG	NS3	201	215	HLA-A*31:01	100%	Dengue virus 2	ELISPOT	IFNg release	Positive	0	1	1	0	1	0
180605	LMCHATFTM	NS3	1734	1742	HLA-B*15:01	100%	Dengue virus 2	ELISPOT	IFNg release	Positive	0	1	1	0	1	0
180617	LPIRYQTPAI	NS3	1713	1722	HLA-B*35:01	100%	Dengue virus 2	ELISPOT	IFNg release	Positive	0	1	1	0	1	0
180647	MEEALRGLPIRYQTP	NS3	1706	1720	HLA class I	100%	Dengue virus 2	ELISPOT	IFNg release	Positive	0	1	1	0	1	0
180724	RGEARKTFVDLMRRG	NS3	2001	2015	HLA class I	100%	Dengue virus 2	ELISPOT	IFNg release	Positive	0	1	1	0	1	0
180734	RSWNSGHEW	NS3	342	350	HLA-B*58:01	100%	Dengue virus 2	ICS	IFNg release	Positive	0	1	0	0	1	0
180756	STRVEMGEAAGIFMT	NS3	301	315	HLA-B*40:06	100%	Dengue virus 2	ELISPOT	IFNg release	Positive-Low	0	1	1	0	1	0
182848	EARKTFVDL	NS3	2003	2011	HLA-B*08:01	100%	Dengue virus 2	ELISPOT	IFNg release	Positive	0	1	1	0	1	0
182898	EEREIPERSW	NS3	1810	1819	HLA-B*44:03	100%	Dengue virus 2	ELISPOT	IFNg release	Positive	0	1	1	0	1	0
183347	GERKKLKPRW	NS3	2061	2070	HLA-B*44:03	100%	Dengue virus 2	ELISPOT	IFNg release	Positive	0	1	1	0	1	0
183724	IKRGLRTLIL	NS3	1687	1696	HLA-B*08:01	100%	Dengue virus 2	ELISPOT	IFNg release	Positive	0	1	1	0	1	0
185200	PRRCMKPVIL	NS3	1900	1909	HLA-B*08:01	100%	Dengue virus 2	ELISPOT	IFNg release	Positive	0	1	1	0	1	0
186211	VDRKGKVVGL	NS3	1615	1624	HLA-B*08:01	100%	Dengue virus 2	ELISPOT	IFNg release	Positive	0	0	0	0	0	0
186242	VEMGEAAGIF	NS3	1779	1788	HLA-B*44:03	100%	Dengue virus 2	ELISPOT	IFNg release	Positive	0	1	1	0	1	0
186301	VLMHRGKRI	NS3	1532	1540	HLA-B*08:01	100%	Dengue virus 2	ELISPOT	IFNg release	Positive	0	0	0	0	0	0
22824	GTSGSPIVDR	NS3	1608	1617	HLA-A*11:01	100%	Dengue virus 2	51 chromium	cytotoxicity	Positive	0	0	0	0	0	0
26965	ILAPTRVVAAEMEEA	NS3	220	234	HLA class I	100%	Dengue virus 2	ELISPOT	IFNg release	Positive	0	1	1	0	1	0
2859	ALRGLPIRY	NS3	234	242	HLA-A*03:01	100%	Dengue virus 2	51 chromium	cytotoxicity	Positive	0	1	1	0	1	0
539620	LRTLILAPTRVVAAEM	NS3	1691	1706	HLA class I	100%	Dengue virus 2	ICS	IFNg release	Positive	0	1	1	0	1	0
539751	NYNLIIMDEAHFTDPA	NS3	1752	1767	HLA class I	100%	Dengue virus 2	ICS	IFNg release	Positive	0	1	1	0	1	0
539752	NYNLIIMDEAHFTDPASI	NS3	1752	1769	HLA class I	100%	Dengue virus 2	ICS	IFNg release	Positive	0	1	1	0	1	0
539985	TLILAPTRVVAAEMEEA	NS3	1693	1709	HLA class I	100%	Dengue virus 2	ICS	IFNg release	Positive	0	1	1	0	1	0
5542	AVSLDFSPGTSGSPI	NS3	125	139	HLA class I	100%	Dengue virus 2	ELISPOT	IFNg release	Positive	0	1	1	0	1	0
56310	RVIDPRRCMK	NS3	1896	1905	HLA-A*31:01	100%	Dengue virus 2	ELISPOT	IFNg release	Positive	0	1	1	0	1	0
57554	SEMGANFKA	NS3	1872	1880	HLA-B*44:03	100%	Dengue virus 2	ELISPOT	IFNg release	Positive	0	1	1	0	1	0
67210	TVWFVPSIK	NS3	358	366	HLA class I	100%	Dengue virus 2	ELISPOT	IFNg release	Positive	0	1	1	0	1	0
150289	IILEFFLIV	NS4a	2202	2210	HLA-A*02:01	100%	Dengue virus 2	ELISPOT	IFNg release	Positive	0	1	1	0	1	0
150752	YVVIAILTV	NS4a	2229	2237	HLA-A*02:01	100%	Dengue virus 2	ELISPOT	IFNg release	Positive	0	1	1	0	1	0
150753	YVVIAILTVV	NS4a	2229	2238	HLA-A*02:01	100%	Dengue virus 2	ELISPOT	IFNg release	Positive	0	1	1	0	1	0
180620	LPTFMTQKAR	NS4a	2106	2115	HLA-B*35:01	100%	Dengue virus 2	ELISPOT	IFNg release	Positive	0	1	1	0	1	0
180781	TPQDNQLTY	NS4a	2221	2229	HLA-B*35:01	100%	Dengue virus 2	ELISPOT	IFNg release	Positive	0	1	1	0	1	0
2877	ALSELPETL	NS4a	2137	2145	HLA-A*02:01	100%	Dengue virus 2	ELISPOT	IFNg release	Positive	0	1	1	0	1	0
51562	QLTYVVIAIL	NS4a	2226	2235	HLA-B*08:01	100%	Dengue virus 2	ELISPOT	IFNg release	Positive	0	1	1	0	1	0
150147	ATVLMGLGK	NS4b	2315	2323	HLA-A*11:01	100%	Dengue virus 2	multimer/tetramer	qualitative binding	Positive	0	1	1	0	1	0
150373	LLILCVTQV	NS4b	2416	2424	HLA-A*02:01	100%	Dengue virus 2	ELISPOT	IFNg release	Positive	0	0	0	0	0	0
150377	LLLVAHYAI	NS4b	2355	2363	HLA-A*02:01	100%	Dengue virus 2	ELISPOT	IFNg release	Positive	0	1	0	0	0	0
150387	LMGLGKGWPL	NS4b	2318	2327	HLA-A*02:01	100%	Dengue virus 2	ELISPOT	IFNg release	Positive	0	1	1	0	1	0
150389	LMMRTTWAL	NS4b	2425	2433	HLA-B*08:01	100%	Dengue virus 2	ELISPOT	IFNg release	Positive	0	1	1	0	1	0
150445	MLLILCVTQV	NS4b	2415	2424	HLA-A*02:01	100%	Dengue virus 2	ELISPOT	IFNg release	Positive	0	0	0	0	0	0
150534	RPASAWTLY	NS4b	2276	2284	HLA-B*07:02	100%	Dengue virus 2	ELISPOT	IFNg release	Positive	0	1	1	0	1	0
150639	TLYAVATTFV	NS4b	2282	2291	HLA-A*02:01	100%	Dengue virus 2	ELISPOT	IFNg release	Positive	0	1	1	0	1	0
150686	VLMMRTTWA	NS4b	2424	2432	HLA-B*08:01	100%	Dengue virus 2	ELISPOT	IFNg release	Positive	0	1	1	0	1	0
180380	ATGPISTLW	NS4b	2440	2448	HLA-B*58:01	100%	Dengue virus 2	ELISPOT	IFNg release	Positive	0	1	1	0	1	0
180387	CEALTLATGPISTLW	NS4b	191	205	HLA class I	100%	Dengue virus 2	ELISPOT	IFNg release	Positive	0	1	1	0	1	0
180512	IANQATVLM	NS4b	2311	2319	HLA-B*35:01	100%	Dengue virus 2	ELISPOT	IFNg release	Positive	0	1	1	0	1	0
180514	IAVSMANIF	NS4b	2460	2468	HLA-B*35:01	100%	Dengue virus 2	ELISPOT	IFNg release	Positive	0	1	1	0	1	0
180601	LILCVTQVLM	NS4b	2417	2426	HLA-B*35:01	100%	Dengue virus 2	ELISPOT	IFNg release	Positive	0	0	0	0	0	0
180794	VATTFVTPM	NS4b	2286	2294	HLA-B*35:01	100%	Dengue virus 2	ELISPOT	IFNg release	Positive	0	1	1	0	1	0
180802	VPLLAIGCY	NS4b	2334	2342	HLA-B*35:01	100%	Dengue virus 2	ELISPOT	IFNg release	Positive	0	1	1	0	1	0
182747	CYSQVNPITL	NS4b	2341	2350	HLA-A*24:02	100%	Dengue virus 2	ELISPOT	IFNg release	Positive	0	1	1	0	1	0
183670	IENSSVNVSL	NS4b	2299	2308	HLA-B*08:01	100%	Dengue virus 2	ELISPOT	IFNg release	Positive	0	1	1	0	1	0
184439	LLILCVTQVL	NS4b	2416	2425	HLA-B*08:01	100%	Dengue virus 2	ELISPOT	IFNg release	Positive	0	0	0	0	0	0
184814	MGLGKGWPL	NS4b	2319	2327	HLA-B*08:01	100%	Dengue virus 2	ELISPOT	IFNg release	Positive	0	1	1	0	1	0
186002	TPMLRHSIE	NS4b	2292	2300	HLA-B*08:01	100%	Dengue virus 2	ELISPOT	IFNg release	Positive	0	1	1	0	1	0

NCBI IDs of DENV-2 circulating strains from Brazil, (100% identity=1,green ; else 0, red)

Epitope_ID	ACY70783.1	ACW82875.1	ACO06162.1	ACS32031.1	ACO06171.1	ACY70780.1	ADI80655.1	AGK36290.1	AGK36292.1	AGK36297.1	AKQ00025.1	AKQ00022.1	AKQ00021.1	QDZ58871.1	QOW96380.1	QOW96384.1	ACO06165.1	AGK36291.1	AGK36294.1	AGN94882.1	AKQ00027.1	AKQ00040.1	QOW96379.1	QOW96388.1	ACO06168.1	AGK36293.1	AGN94880.1	AGN94881.1	AKQ00024.1	AKQ00020.1	QBL56211.1	AGK36298.1	AGN94883.1	AKQ00026.1	QTX93212.1
150399	0	0	0	0	0	0	0	0	0	0	0	0	0	0	0	0	0	0	0	0	0	0	0	0	0	0	0	0	0	0	0	0	0	0	0
183748	0	0	0	0	0	0	0	0	0	0	0	0	0	0	0	0	0	0	0	0	0	0	0	0	0	0	0	0	0	0	0	0	0	0	0
184300	0	0	0	0	0	0	0	0	0	0	0	0	0	0	0	0	0	0	0	0	0	0	0	0	0	0	0	0	0	0	0	0	0	0	0
41407	0	0	0	0	0	0	0	0	0	0	0	0	0	0	0	0	0	0	0	0	0	0	0	0	0	0	0	0	0	0	0	0	0	0	0
180496	0	0	0	0	0	0	0	0	0	0	0	0	0	0	0	0	0	0	0	0	0	0	0	0	0	0	0	0	0	0	0	0	0	0	0
150391	0	0	0	0	0	0	0	0	0	0	0	0	0	0	0	0	0	0	0	0	0	0	0	0	0	0	0	0	0	0	0	0	0	0	0
183741	0	0	0	0	0	0	0	0	0	0	0	0	0	0	0	0	0	0	0	0	0	0	0	0	0	0	0	0	0	0	0	0	0	0	0
150381	0	0	0	0	0	0	0	0	0	0	0	0	0	0	0	0	0	0	0	0	0	0	0	0	0	0	0	0	0	0	0	0	0	0	0
150425	0	0	0	0	0	0	0	0	0	0	0	0	0	0	0	0	0	0	0	0	0	0	0	0	0	0	0	0	0	0	0	0	0	0	0
180538	0	0	0	0	0	0	0	0	0	0	0	0	0	0	0	0	0	0	0	0	0	0	0	0	0	0	0	0	0	0	0	0	0	0	0
17802	0	0	0	0	0	0	0	0	0	0	0	0	0	0	0	0	0	0	0	0	0	0	0	0	0	0	0	0	0	0	0	0	0	0	0
180750	0	0	0	0	0	0	0	0	0	0	0	0	0	0	0	0	0	0	0	0	0	0	0	0	0	0	0	0	0	0	0	0	0	0	0
182892	0	0	0	0	0	0	0	0	0	0	0	0	0	0	0	0	0	0	0	0	0	0	0	0	0	0	0	0	0	0	0	0	0	0	0
184318	0	0	0	0	0	0	0	0	0	0	0	0	0	0	0	0	0	0	0	0	0	0	0	0	0	0	0	0	0	0	0	0	0	0	0
185388	0	0	0	0	0	0	0	0	0	0	0	0	0	0	0	0	0	0	0	0	0	0	0	0	0	0	0	0	0	0	0	0	0	0	0
22816	0	0	0	0	0	0	0	0	0	0	0	0	0	0	0	0	0	0	0	0	0	0	0	0	0	0	0	0	0	0	0	0	0	0	0
22817	0	0	0	0	0	0	0	0	0	0	0	0	0	0	0	0	0	0	0	0	0	0	0	0	0	0	0	0	0	0	0	0	0	0	0
22818	0	0	0	0	0	0	0	0	0	0	0	0	0	0	0	0	0	0	0	0	0	0	0	0	0	0	0	0	0	0	0	0	0	0	0
22819	0	0	0	0	0	0	0	0	0	0	0	0	0	0	0	0	0	0	0	0	0	0	0	0	0	0	0	0	0	0	0	0	0	0	0
22823	0	0	0	0	0	0	0	0	0	0	0	0	0	0	0	0	0	0	0	0	0	0	0	0	0	0	0	0	0	0	0	0	0	0	0
54176	0	0	0	0	0	0	0	0	0	0	0	0	0	0	0	0	0	0	0	0	0	0	0	0	0	0	0	0	0	0	0	0	0	0	0
65548	0	0	0	0	0	0	0	0	0	0	0	0	0	0	0	0	0	0	0	0	0	0	0	0	0	0	0	0	0	0	0	0	0	0	0
8910	0	0	0	0	0	0	0	0	0	0	0	0	0	0	0	0	0	0	0	0	0	0	0	0	0	0	0	0	0	0	0	0	0	0	0
150612	0	0	0	0	0	0	0	0	0	0	0	0	0	0	0	0	0	0	0	0	0	0	0	0	0	0	0	0	0	0	0	0	0	0	0
150679	0	0	0	0	0	0	0	0	0	0	0	0	0	0	0	0	0	0	0	0	0	0	0	0	0	0	0	0	0	0	0	0	0	0	0
16757	0	0	0	0	0	0	0	0	0	0	0	0	0	0	0	0	0	0	0	0	0	0	0	0	0	0	0	0	0	0	0	0	0	0	0
180789	0	0	0	0	0	0	0	0	0	0	0	0	0	0	0	0	0	0	0	0	0	0	0	0	0	0	0	0	0	0	0	0	0	0	0
150227	0	0	0	0	0	0	0	0	0	0	0	0	0	0	0	0	0	0	0	0	0	0	0	0	0	0	0	0	0	0	0	0	0	0	0
163620	0	0	0	0	0	0	0	0	0	0	0	0	0	0	0	0	0	0	0	0	0	0	0	0	0	0	0	0	0	0	0	0	0	0	0
180579	0	0	0	0	0	0	0	0	0	0	0	0	0	0	0	0	0	0	0	0	0	0	0	0	0	0	0	0	0	0	0	0	0	0	0
180828	0	0	0	0	0	0	0	0	0	0	0	0	0	0	0	0	0	0	0	0	0	0	0	0	0	0	0	0	0	0	0	0	0	0	0
184840	0	0	0	0	0	0	0	0	0	0	0	0	0	0	0	0	0	0	0	0	0	0	0	0	0	0	0	0	0	0	0	0	0	0	0
32939	0	0	0	0	0	0	0	0	0	0	0	0	0	0	0	0	0	0	0	0	0	0	0	0	0	0	0	0	0	0	0	0	0	0	0
32941	0	0	0	0	0	0	0	0	0	0	0	0	0	0	0	0	0	0	0	0	0	0	0	0	0	0	0	0	0	0	0	0	0	0	0
32942	0	0	0	0	0	0	0	0	0	0	0	0	0	0	0	0	0	0	0	0	0	0	0	0	0	0	0	0	0	0	0	0	0	0	0
539144	0	0	0	0	0	0	0	0	0	0	0	0	0	0	0	0	0	0	0	0	0	0	0	0	0	0	0	0	0	0	0	0	0	0	0
539436	0	0	0	0	0	0	0	0	0	0	0	0	0	0	0	0	0	0	0	0	0	0	0	0	0	0	0	0	0	0	0	0	0	0	0
73428	0	0	0	0	0	0	0	0	0	0	0	0	0	0	0	0	0	0	0	0	0	0	0	0	0	0	0	0	0	0	0	0	0	0	0
150252	1	1	1	1	1	1	1	1	1	1	1	1	1	1	1	1	1	1	1	1	1	1	1	1	1	1	1	1	1	1	1	1	1	1	1
185399	1	1	1	1	1	1	1	1	1	1	1	1	1	1	1	1	1	1	1	1	1	1	1	1	1	1	1	1	1	1	1	1	1	1	1
150299	1	1	1	1	1	1	1	1	1	1	1	1	1	1	1	1	1	1	1	1	1	1	1	1	1	1	1	1	1	1	1	1	1	1	1
180588	1	1	1	1	1	1	1	1	1	1	1	1	1	1	1	1	1	1	1	1	1	1	1	1	1	1	1	1	1	1	1	1	1	1	1
183577	1	1	1	1	1	1	1	1	1	1	1	1	1	1	1	1	1	1	1	1	1	1	1	1	1	1	1	1	1	1	1	1	1	1	1
186264	1	1	1	1	1	1	1	1	1	1	1	1	1	1	1	1	1	1	1	1	1	1	1	1	1	1	1	1	1	1	1	1	1	1	1
150456	1	1	1	1	1	1	1	1	1	1	1	1	1	1	1	1	1	1	1	1	1	1	1	1	1	1	1	1	1	1	1	1	1	1	1
150529	1	1	1	1	1	1	1	1	1	1	1	1	1	1	1	1	1	1	1	1	1	1	1	1	1	1	1	1	1	1	1	1	1	1	1
150531	1	1	1	1	1	1	1	1	1	1	1	1	1	1	1	1	1	1	1	1	1	1	1	1	1	1	1	1	1	1	1	1	1	1	1
150725	1	1	1	1	1	1	1	1	1	1	1	1	1	1	1	1	1	1	1	1	1	1	1	1	1	1	1	1	1	1	1	1	1	1	1
16553	1	1	1	1	1	1	1	1	1	1	1	1	1	1	1	1	1	1	1	1	1	1	1	1	1	1	1	1	1	1	1	1	1	1	1
180563	1	1	1	1	1	1	1	1	1	1	1	1	1	1	1	1	1	1	1	1	1	1	1	1	1	1	1	1	1	1	1	1	1	1	1
180618	1	1	1	1	1	1	1	1	1	1	1	1	1	1	1	1	1	1	1	1	1	1	1	1	1	1	1	1	1	1	1	1	1	1	1
180816	1	1	1	1	1	1	1	1	1	1	1	1	1	1	1	1	1	1	1	1	1	1	1	1	1	1	1	1	1	1	1	1	1	1	1
183437	1	1	1	1	1	1	1	1	1	1	1	1	1	1	1	1	1	1	1	1	1	1	1	1	1	1	1	1	1	1	1	1	1	1	1
183708	1	1	1	1	1	1	1	1	1	1	1	1	1	1	1	1	1	1	1	1	1	1	1	1	1	1	1	1	1	1	1	1	1	1	1
183747	1	1	1	1	1	1	1	1	1	1	1	1	1	1	1	1	1	1	1	1	1	1	1	1	1	1	1	1	1	1	1	1	1	1	1
183840	1	1	1	1	1	1	1	1	1	1	1	1	1	1	1	1	1	1	1	1	1	1	1	1	1	1	1	1	1	1	1	1	1	1	1
183841	1	1	1	1	1	1	1	1	1	1	1	1	1	1	1	1	1	1	1	1	1	1	1	1	1	1	1	1	1	1	1	1	1	1	1
184337	1	1	1	1	1	1	1	1	1	1	1	1	1	1	1	1	1	1	1	1	1	1	1	1	1	1	1	1	1	1	1	1	1	1	1
184467	1	1	1	1	1	1	1	1	1	1	1	1	1	1	1	1	1	1	1	1	1	1	1	1	1	1	1	1	1	1	1	1	1	1	1
184571	1	1	1	1	1	1	1	1	1	1	1	1	1	1	1	1	1	1	1	1	1	1	1	1	1	1	1	1	1	1	1	1	1	1	1
184920	1	1	1	1	1	1	1	1	1	1	1	1	1	1	1	1	1	1	1	1	1	1	1	1	1	1	1	1	1	1	1	1	1	1	1
185758	1	1	1	1	1	1	1	1	1	1	1	1	1	1	1	1	1	1	1	1	1	1	1	1	1	1	1	1	1	1	1	1	1	1	1
185831	1	1	1	1	1	1	1	1	1	1	1	1	1	1	1	1	1	1	1	1	1	1	1	1	1	1	1	1	1	1	1	1	1	1	1
51388	1	1	1	1	1	1	1	1	1	1	1	1	1	1	1	1	1	1	1	1	1	1	1	1	1	1	1	1	1	1	1	1	1	1	1
180450	1	1	1	1	1	1	1	1	1	1	1	1	1	1	1	1	1	1	1	1	1	1	1	1	1	1	1	1	1	1	1	1	1	1	1
180689	1	1	1	1	1	1	1	1	1	1	1	1	1	1	1	1	1	1	1	1	1	1	1	1	1	1	1	1	1	1	1	1	1	1	1
182938	1	1	1	1	1	1	1	1	1	1	1	1	1	1	1	1	1	1	1	1	1	1	1	1	1	1	1	1	1	1	1	1	1	1	1
184512	1	1	1	1	1	1	1	1	1	1	1	1	1	1	1	1	1	1	1	1	1	1	1	1	1	1	1	1	1	1	1	1	1	1	1
185342	1	1	1	1	1	1	1	1	1	1	1	1	1	1	1	1	1	1	1	1	1	1	1	1	1	1	1	1	1	1	1	1	1	1	1
185465	1	1	1	1	1	1	1	1	1	1	1	1	1	1	1	1	1	1	1	1	1	1	1	1	1	1	1	1	1	1	1	1	1	1	1
19225	1	1	1	1	1	1	1	1	1	1	1	1	1	1	1	1	1	1	1	1	1	1	1	1	1	1	1	1	1	1	1	1	1	1	1
150206	1	1	1	1	1	1	1	1	1	1	1	1	1	1	1	1	1	1	1	1	1	1	1	1	1	1	1	1	1	1	1	1	1	1	1
150415	1	1	1	1	1	1	1	1	1	1	1	1	1	1	1	1	1	1	1	1	1	1	1	1	1	1	1	1	1	1	1	1	1	1	1
150457	1	1	1	1	1	1	1	1	1	1	1	1	1	1	1	1	1	1	1	1	1	1	1	1	1	1	1	1	1	1	1	1	1	1	1
150540	1	1	1	1	1	1	1	1	1	1	1	1	1	1	1	1	1	1	1	1	1	1	1	1	1	1	1	1	1	1	1	1	1	1	1
150640	1	1	1	1	1	1	1	1	1	1	1	1	1	1	1	1	1	1	1	1	1	1	1	1	1	1	1	1	1	1	1	1	1	1	1
150741	1	1	1	1	1	1	1	1	1	1	1	1	1	1	1	1	1	1	1	1	1	1	1	1	1	1	1	1	1	1	1	1	1	1	1
183825	1	1	1	1	1	1	1	1	1	1	1	1	1	1	1	1	1	1	1	1	1	1	1	1	1	1	1	1	1	1	1	1	1	1	1
184864	1	1	1	1	1	1	1	1	1	1	1	1	1	1	1	1	1	1	1	1	1	1	1	1	1	1	1	1	1	1	1	1	1	1	1
186041	1	1	1	1	1	1	1	1	1	1	1	1	1	1	1	1	1	1	1	1	1	1	1	1	1	1	1	1	1	1	1	1	1	1	1
150101	1	1	1	1	1	1	1	1	1	1	1	1	1	1	1	1	1	1	1	1	1	1	1	1	1	1	1	1	1	1	1	1	1	1	1
150584	1	1	1	1	1	1	1	1	1	1	1	1	1	1	1	1	1	1	1	1	1	1	1	1	1	1	1	1	1	1	1	1	1	1	1
150610	1	1	1	1	1	1	1	1	1	1	1	1	1	1	1	1	1	1	1	1	1	1	1	1	1	1	1	1	1	1	1	1	1	1	1
180529	1	1	1	1	1	1	1	1	1	1	1	1	1	1	1	1	1	1	1	1	1	1	1	1	1	1	1	1	1	1	1	1	1	1	1
180531	1	1	1	1	1	1	1	1	1	1	1	1	1	1	1	1	1	1	1	1	1	1	1	1	1	1	1	1	1	1	1	1	1	1	1
180646	1	1	1	1	1	1	1	1	1	1	1	1	1	1	1	1	1	1	1	1	1	1	1	1	1	1	1	1	1	1	1	1	1	1	1
183819	1	1	1	1	1	1	1	1	1	1	1	1	1	1	1	1	1	1	1	1	1	1	1	1	1	1	1	1	1	1	1	1	1	1	1
11063	1	1	1	1	1	1	1	1	1	1	1	1	1	1	1	1	1	1	1	1	1	1	1	1	1	1	1	1	1	1	1	1	1	1	1
11125	1	1	1	1	1	1	1	1	1	1	1	1	1	1	1	1	1	1	1	1	1	1	1	1	1	1	1	1	1	1	1	1	1	1	1
124391	1	1	1	1	1	1	1	1	1	1	1	1	1	1	1	1	1	1	1	1	1	1	1	1	1	1	1	1	1	1	1	1	1	1	1
13358	1	1	1	1	1	1	1	1	1	1	1	1	1	1	1	1	1	1	1	1	1	1	1	1	1	1	1	1	1	1	1	1	1	1	1
136890	1	1	1	1	1	1	1	1	1	1	1	1	1	1	1	1	1	1	1	1	1	1	1	1	1	1	1	1	1	1	1	1	1	1	1
150137	1	1	1	1	1	1	1	1	1	1	1	1	1	1	1	1	1	1	1	1	1	1	1	1	1	1	1	1	1	1	1	1	1	1	1
150167	1	1	1	1	1	1	1	1	1	1	1	1	1	1	1	1	1	1	1	1	1	1	1	1	1	1	1	1	1	1	1	1	1	1	1
150217	1	1	1	1	1	1	1	1	1	1	1	1	1	1	1	1	1	1	1	1	1	1	1	1	1	1	1	1	1	1	1	1	1	1	1
150450	1	1	1	1	1	1	1	1	1	1	1	1	1	1	1	1	1	1	1	1	1	1	1	1	1	1	1	1	1	1	1	1	1	1	1
150451	1	1	1	1	1	1	1	1	1	1	1	1	1	1	1	1	1	1	1	1	1	1	1	1	1	1	1	1	1	1	1	1	1	1	1
150526	1	1	1	1	1	1	1	1	1	1	1	1	1	1	1	1	1	1	1	1	1	1	1	1	1	1	1	1	1	1	1	1	1	1	1
150595	1	1	1	1	1	1	1	1	1	1	1	1	1	1	1	1	1	1	1	1	1	1	1	1	1	1	1	1	1	1	1	1	1	1	1
150693	1	1	1	1	1	1	1	1	1	1	1	1	1	1	1	1	1	1	1	1	1	1	1	1	1	1	1	1	1	1	1	1	1	1	1
150740	1	1	1	1	1	1	1	1	1	1	1	1	1	1	1	1	1	1	1	1	1	1	1	1	1	1	1	1	1	1	1	1	1	1	1
180428	1	1	1	1	1	1	1	1	1	1	1	1	1	1	1	1	1	1	1	1	1	1	1	1	1	1	1	1	1	1	1	1	1	1	1
180440	1	1	1	1	1	1	1	1	1	1	1	1	1	1	1	1	1	1	1	1	1	1	1	1	1	1	1	1	1	1	1	1	1	1	1
180460	1	1	1	1	1	1	1	1	1	1	1	1	1	1	1	1	1	1	1	1	1	1	1	1	1	1	1	1	1	1	1	1	1	1	1
180471	1	1	1	1	1	1	1	1	1	1	1	1	1	1	1	1	1	1	1	1	1	1	1	1	1	1	1	1	1	1	1	1	1	1	1
180503	1	1	1	1	1	1	1	1	1	1	1	1	1	1	1	1	1	1	1	1	1	1	1	1	1	1	1	1	1	1	1	1	1	1	1
180507	1	1	1	1	1	1	1	1	1	1	1	1	1	1	1	1	1	1	1	1	1	1	1	1	1	1	1	1	1	1	1	1	1	1	1
180575	1	1	1	1	1	1	1	1	1	1	1	1	1	1	1	1	1	1	1	1	1	1	1	1	1	1	1	1	1	1	1	1	1	1	1
180605	1	1	1	1	1	1	1	1	1	1	1	1	1	1	1	1	1	1	1	1	1	1	1	1	1	1	1	1	1	1	1	1	1	1	1
180617	1	1	1	1	1	1	1	1	1	1	1	1	1	1	1	1	1	1	1	1	1	1	1	1	1	1	1	1	1	1	1	1	1	1	1
180647	1	1	1	1	1	1	1	1	1	1	1	1	1	1	1	1	1	1	1	1	1	1	1	1	1	1	1	1	1	1	1	1	1	1	1
180724	1	1	1	1	1	1	1	1	1	1	1	1	1	1	1	1	1	1	1	1	1	1	1	1	1	1	1	1	1	1	1	1	1	1	1
180734	1	1	1	1	1	1	1	1	1	1	1	1	1	1	1	1	1	1	1	1	1	1	1	1	1	1	1	1	1	1	1	1	1	1	1
180756	1	1	1	1	1	1	1	1	1	1	1	1	1	1	1	1	1	1	1	1	1	1	1	1	1	1	1	1	1	1	1	1	1	1	1
182848	1	1	1	1	1	1	1	1	1	1	1	1	1	1	1	1	1	1	1	1	1	1	1	1	1	1	1	1	1	1	1	1	1	1	1
182898	1	1	1	1	1	1	1	1	1	1	1	1	1	1	1	1	1	1	1	1	1	1	1	1	1	1	1	1	1	1	1	1	1	1	1
183347	1	1	1	1	1	1	1	1	1	1	1	1	1	1	1	1	1	1	1	1	1	1	1	1	1	1	1	1	1	1	1	1	1	1	1
183724	1	1	1	1	1	1	1	1	1	1	1	1	1	1	1	1	1	1	1	1	1	1	1	1	1	1	1	1	1	1	1	1	1	1	1
185200	1	1	1	1	1	1	1	1	1	1	1	1	1	1	1	1	1	1	1	1	1	1	1	1	1	1	1	1	1	1	1	1	1	1	1
186211	1	1	1	1	1	1	1	1	1	1	1	1	1	1	1	1	1	1	1	1	1	1	1	1	1	1	1	1	1	1	1	1	1	1	1
186242	1	1	1	1	1	1	1	1	1	1	1	1	1	1	1	1	1	1	1	1	1	1	1	1	1	1	1	1	1	1	1	1	1	1	1
186301	1	1	1	1	1	1	1	1	1	1	1	1	1	1	1	1	1	1	1	1	1	1	1	1	1	1	1	1	1	1	1	1	1	1	1
22824	1	1	1	1	1	1	1	1	1	1	1	1	1	1	1	1	1	1	1	1	1	1	1	1	1	1	1	1	1	1	1	1	1	1	1
26965	1	1	1	1	1	1	1	1	1	1	1	1	1	1	1	1	1	1	1	1	1	1	1	1	1	1	1	1	1	1	1	1	1	1	1
2859	1	1	1	1	1	1	1	1	1	1	1	1	1	1	1	1	1	1	1	1	1	1	1	1	1	1	1	1	1	1	1	1	1	1	1
539620	1	1	1	1	1	1	1	1	1	1	1	1	1	1	1	1	1	1	1	1	1	1	1	1	1	1	1	1	1	1	1	1	1	1	1
539751	1	1	1	1	1	1	1	1	1	1	1	1	1	1	1	1	1	1	1	1	1	1	1	1	1	1	1	1	1	1	1	1	1	1	1
539752	1	1	1	1	1	1	1	1	1	1	1	1	1	1	1	1	1	1	1	1	1	1	1	1	1	1	1	1	1	1	1	1	1	1	1
539985	1	1	1	1	1	1	1	1	1	1	1	1	1	1	1	1	1	1	1	1	1	1	1	1	1	1	1	1	1	1	1	1	1	1	1
5542	1	1	1	1	1	1	1	1	1	1	1	1	1	1	1	1	1	1	1	1	1	1	1	1	1	1	1	1	1	1	1	1	1	1	1
56310	1	1	1	1	1	1	1	1	1	1	1	1	1	1	1	1	1	1	1	1	1	1	1	1	1	1	1	1	1	1	1	1	1	1	1
57554	1	1	1	1	1	1	1	1	1	1	1	1	1	1	1	1	1	1	1	1	1	1	1	1	1	1	1	1	1	1	1	1	1	1	1
67210	1	1	1	1	1	1	1	1	1	1	1	1	1	1	1	1	1	1	1	1	1	1	1	1	1	1	1	1	1	1	1	1	1	1	1
150289	1	1	1	1	1	1	1	1	1	1	1	1	1	1	1	1	1	1	1	1	1	1	1	1	1	1	1	1	1	1	1	1	1	1	1
150752	1	1	1	1	1	1	1	1	1	1	1	1	1	1	1	1	1	1	1	1	1	1	1	1	1	1	1	1	1	1	1	1	1	1	1
150753	1	1	1	1	1	1	1	1	1	1	1	1	1	1	1	1	1	1	1	1	1	1	1	1	1	1	1	1	1	1	1	1	1	1	1
180620	1	1	1	1	1	1	1	1	1	1	1	1	1	1	1	1	1	1	1	1	1	1	1	1	1	1	1	1	1	1	1	1	1	1	1
180781	1	1	1	1	1	1	1	1	1	1	1	1	1	1	1	1	1	1	1	1	1	1	1	1	1	1	1	1	1	1	1	1	1	1	1
2877	1	1	1	1	1	1	1	1	1	1	1	1	1	1	1	1	1	1	1	1	1	1	1	1	1	1	1	1	1	1	1	1	1	1	1
51562	1	1	1	1	1	1	1	1	1	1	1	1	1	1	1	1	1	1	1	1	1	1	1	1	1	1	1	1	1	1	1	1	1	1	1
150147	1	1	1	1	1	1	1	1	1	1	1	1	1	1	1	1	1	1	1	1	1	1	1	1	1	1	1	1	1	1	1	1	1	1	1
150373	1	1	1	1	1	1	1	1	1	1	1	1	1	1	1	1	1	1	1	1	1	1	1	1	1	1	1	1	1	1	1	1	1	1	1
150377	1	1	1	1	1	1	1	1	1	1	1	1	1	1	1	1	1	1	1	1	1	1	1	1	1	1	1	1	1	1	1	1	1	1	1
150387	1	1	1	1	1	1	1	1	1	1	1	1	1	1	1	1	1	1	1	1	1	1	1	1	1	1	1	1	1	1	1	1	1	1	1
150389	1	1	1	1	1	1	1	1	1	1	1	1	1	1	1	1	1	1	1	1	1	1	1	1	1	1	1	1	1	1	1	1	1	1	1
150445	1	1	1	1	1	1	1	1	1	1	1	1	1	1	1	1	1	1	1	1	1	1	1	1	1	1	1	1	1	1	1	1	1	1	1
150534	1	1	1	1	1	1	1	1	1	1	1	1	1	1	1	1	1	1	1	1	1	1	1	1	1	1	1	1	1	1	1	1	1	1	1
150639	1	1	1	1	1	1	1	1	1	1	1	1	1	1	1	1	1	1	1	1	1	1	1	1	1	1	1	1	1	1	1	1	1	1	1
150686	1	1	1	1	1	1	1	1	1	1	1	1	1	1	1	1	1	1	1	1	1	1	1	1	1	1	1	1	1	1	1	1	1	1	1
180380	1	1	1	1	1	1	1	1	1	1	1	1	1	1	1	1	1	1	1	1	1	1	1	1	1	1	1	1	1	1	1	1	1	1	1
180387	1	1	1	1	1	1	1	1	1	1	1	1	1	1	1	1	1	1	1	1	1	1	1	1	1	1	1	1	1	1	1	1	1	1	1
180512	1	1	1	1	1	1	1	1	1	1	1	1	1	1	1	1	1	1	1	1	1	1	1	1	1	1	1	1	1	1	1	1	1	1	1
180514	1	1	1	1	1	1	1	1	1	1	1	1	1	1	1	1	1	1	1	1	1	1	1	1	1	1	1	1	1	1	1	1	1	1	1
180601	1	1	1	1	1	1	1	1	1	1	1	1	1	1	1	1	1	1	1	1	1	1	1	1	1	1	1	1	1	1	1	1	1	1	1
180794	1	1	1	1	1	1	1	1	1	1	1	1	1	1	1	1	1	1	1	1	1	1	1	1	1	1	1	1	1	1	1	1	1	1	1
180802	1	1	1	1	1	1	1	1	1	1	1	1	1	1	1	1	1	1	1	1	1	1	1	1	1	1	1	1	1	1	1	1	1	1	1
182747	1	1	1	1	1	1	1	1	1	1	1	1	1	1	1	1	1	1	1	1	1	1	1	1	1	1	1	1	1	1	1	1	1	1	1
183670	1	1	1	1	1	1	1	1	1	1	1	1	1	1	1	1	1	1	1	1	1	1	1	1	1	1	1	1	1	1	1	1	1	1	1
184439	1	1	1	1	1	1	1	1	1	1	1	1	1	1	1	1	1	1	1	1	1	1	1	1	1	1	1	1	1	1	1	1	1	1	1
184814	1	1	1	1	1	1	1	1	1	1	1	1	1	1	1	1	1	1	1	1	1	1	1	1	1	1	1	1	1	1	1	1	1	1	1
186002	1	1	1	1	1	1	1	1	1	1	1	1	1	1	1	1	1	1	1	1	1	1	1	1	1	1	1	1	1	1	1	1	1	1	1

240933	TPMLRHSI	NS4b	2292	2299	HLA-B*07:02	100%	Dengue virus 2	ELISPOT	IFNg release	Positive	1	1	1	1	1	1	1	1	1	1	1	1	1	1	1	1	1	1	1	1	1	1	1	1	1	1	1	1	1	1	1	1	1	1	1	0	1	1	0	1	0
10587	DVFFTPPEK	NS5	2622	2630	HLA class I	100%	Dengue virus 2	ELISPOT	IFNg release	Positive	1	1	1	1	1	1	1	1	1	1	1	1	1	1	1	1	1	1	1	1	1	1	1	1	1	1	1	1	1	1	1	1	1	1	1	0	0	1	0	0	0
150309	IPMSTYGWNL	NS5	2605	2614	HLA-B*07:02	100%	Dengue virus 2	ELISPOT	IFNg release	Positive	1	1	1	1	1	1	1	1	1	1	1	1	1	1	1	1	1	1	1	1	1	1	1	1	1	1	1	1	1	1	1	1	1	1	1	0	1	1	0	1	0
150541	RPTPRGTVM	NS5	3073	3081	HLA-B*07:02	100%	Dengue virus 2	ELISPOT	IFNg release	Positive	1	1	1	1	1	1	1	1	1	1	1	1	1	1	1	1	1	1	1	1	1	1	1	1	1	1	1	1	1	1	1	1	1	1	1	0	1	1	0	1	0
150554	RTTWSIHAK	NS5	3283	3291	HLA-A*11:01	100%	Dengue virus 2	ELISPOT	IFNg release	Positive	1	1	1	1	1	1	1	1	1	1	1	1	1	1	1	1	1	1	1	1	1	1	1	1	1	1	1	1	1	1	1	1	1	1	1	0	1	1	0	1	0
150698	VPYLGKREDQ	NS5	3327	3336	HLA-B*07:02	100%	Dengue virus 2	ELISPOT	IFNg release	Positive	1	1	1	1	1	1	1	1	1	1	1	1	1	1	1	1	1	1	1	1	1	1	1	1	1	1	1	1	1	1	1	1	1	1	1	0	0	0	0	0	0
163027	NIQTAINQV	NS5	3353	3361	HLA-A*02:01	100%	Dengue virus 2	ELISPOT	IFNg release	Positive-Low	1	1	1	1	1	1	1	1	1	1	1	1	1	1	1	1	1	1	1	1	1	1	1	1	1	1	1	1	1	1	1	1	1	1	1	0	0	1	0	0	0
180437	EPKEGTKKLM	NS5	2856	2865	HLA-B*35:01	100%	Dengue virus 2	ELISPOT	IFNg release	Positive	1	1	1	1	1	1	1	1	1	1	1	1	1	1	1	1	1	1	1	1	1	1	1	1	1	1	1	1	1	1	1	1	1	1	1	0	1	1	0	1	0
180443	ETACLGKSYA	NS5	3241	3250	HLA-A*26:01	100%	Dengue virus 2	ELISPOT	IFNg release	Positive	1	1	1	1	1	1	1	1	1	1	1	1	1	1	1	1	1	1	1	1	1	1	1	1	1	1	1	1	1	1	1	1	1	1	1	0	1	1	0	1	0
180488	GPGHEEPIPM	NS5	2598	2607	HLA-B*35:01	100%	Dengue virus 2	ELISPOT	IFNg release	Positive	1	1	1	1	1	1	1	1	1	1	1	1	1	1	1	1	1	1	1	1	1	1	1	1	1	1	1	1	1	1	1	1	1	1	1	0	1	1	0	1	0
180500	HKKLAEAIFKLTYQN	NS5	3052	3066	HLA class I	100%	Dengue virus 2	ELISPOT	IFNg release	Positive	1	1	1	1	1	1	1	1	1	1	1	1	1	1	1	1	1	1	1	1	1	1	1	1	1	1	1	1	1	1	1	1	1	1	1	0	1	1	0	1	0
180582	KTWAYHGSYETKQTG	NS5	2792	2806	HLA class I	100%	Dengue virus 2	ICS	IFNg release	Positive	1	1	1	1	1	1	1	1	1	1	1	1	1	1	1	1	1	1	1	1	1	1	1	1	1	1	1	1	1	1	1	1	1	1	1	0	1	1	0	1	0
180584	KVRKDIQQW	NS5	3177	3185	HLA-B*57:01	100%	Dengue virus 2	ELISPOT	IFNg release	Positive	1	1	1	1	1	1	1	1	1	1	1	1	1	1	1	1	1	1	1	1	1	1	1	1	1	1	1	1	1	1	1	1	1	1	1	0	0	1	0	0	0
180643	MAMTDTTPFGQQRVF	NS5	2832	2846	HLA class I	100%	Dengue virus 2	ELISPOT	IFNg release	Positive	1	1	1	1	1	1	1	1	1	1	1	1	1	1	1	1	1	1	1	1	1	1	1	1	1	1	1	1	1	1	1	1	1	1	1	0	1	1	0	1	0
180668	MPSVIEKMET	NS5	2678	2687	HLA-B*53:01	100%	Dengue virus 2	ELISPOT	IFNg release	Positive	1	1	1	1	1	1	1	1	1	1	1	1	1	1	1	1	1	1	1	1	1	1	1	1	1	1	1	1	1	1	1	1	1	1	1	0	1	0	0	0	0
180675	MSTYGWNLVRLQSGV	NS5	2607	2621	HLA class I	100%	Dengue virus 2	ELISPOT	IFNg release	Positive	1	1	1	1	1	1	1	1	1	1	1	1	1	1	1	1	1	1	1	1	1	1	1	1	1	1	1	1	1	1	1	1	1	1	1	0	1	1	0	1	0
180758	SWHYDQDHPYKTWAY	NS5	291	305	HLA class I	100%	Dengue virus 2	ELISPOT	IFNg release	Positive	1	1	1	1	1	1	1	1	1	1	1	1	1	1	1	1	1	1	1	1	1	1	1	1	1	1	1	1	1	1	1	1	1	1	1	0	1	1	0	1	0
180774	TLRVLNLVENWLNNN	NS5	2652	2666	HLA class I	100%	Dengue virus 2	ELISPOT	IFNg release	Positive	1	1	1	1	1	1	1	1	1	1	1	1	1	1	1	1	1	1	1	1	1	1	1	1	1	1	1	1	1	1	1	1	1	1	1	0	1	1	0	1	0
180814	WAYHGSYET	NS5	2794	2802	HLA-B*35:01	100%	Dengue virus 2	ELISPOT	IFNg release	Positive	1	1	1	1	1	1	1	1	1	1	1	1	1	1	1	1	1	1	1	1	1	1	1	1	1	1	1	1	1	1	1	1	1	1	1	0	1	1	0	1	0
180817	WHYDQDHPY	NS5	2783	2791	HLA-B*35:01	100%	Dengue virus 2	ELISPOT	IFNg release	Positive	1	1	1	1	1	1	1	1	1	1	1	1	1	1	1	1	1	1	1	1	1	1	1	1	1	1	1	1	1	1	1	1	1	1	1	0	1	1	0	1	0
184131	KREDQWCGSL	NS5	3332	3341	HLA-B*08:01	100%	Dengue virus 2	ELISPOT	IFNg release	Positive	1	1	1	1	1	1	1	1	1	1	1	1	1	1	1	1	1	1	1	1	1	1	1	1	1	1	1	1	1	1	1	1	1	1	1	0	1	1	0	1	0
185931	TKQTGSASSM	NS5	2802	2811	HLA-B*08:01	100%	Dengue virus 2	ELISPOT	IFNg release	Positive	1	1	1	1	1	1	1	1	1	1	1	1	1	1	1	1	1	1	1	1	1	1	1	1	1	1	1	1	1	1	1	1	1	1	1	0	1	1	0	1	0
190862	MVTQMAMTDTTPFGQQR	NS5	2828	2844	HLA class I	100%	Dengue virus 2	ICS	IFNg release	Positive	1	1	1	1	1	1	1	1	1	1	1	1	1	1	1	1	1	1	1	1	1	1	1	1	1	1	1	1	1	1	1	1	1	1	1	0	1	1	0	1	0
31747	KLAEAIFKL	NS5	3054	3062	HLA-A*02:01	100%	Dengue virus 2	ELISPOT	IFNg release	Positive-Low	1	1	1	1	1	1	1	1	1	1	1	1	1	1	1	1	1	1	1	1	1	1	1	1	1	1	1	1	1	1	1	1	1	1	1	0	1	1	0	1	0
53476	REDQWCGSL	NS5	3333	3341	HLA-B*40:01	100%	Dengue virus 2	ELISPOT	IFNg release	Positive	1	1	1	1	1	1	1	1	1	1	1	1	1	1	1	1	1	1	1	1	1	1	1	1	1	1	1	1	1	1	1	1	1	1	1	0	1	1	0	1	0
69671	VLNPYMPSV	NS5	2673	2681	HLA-A*02:01	100%	Dengue virus 2	ELISPOT	IFNg release	Positive	1	1	1	1	1	1	1	1	1	1	1	1	1	1	1	1	1	1	1	1	1	1	1	1	1	1	1	1	1	1	1	1	1	1	1	0	1	1	0	1	0
110018	VAFLRFLTI	C	51	59	HLA-B*08:01	81% - <100%	Dengue virus 2	ELISPOT	IFNg release	Positive	1	1	1	1	1	1	1	1	1	1	1	1	1	1	1	1	1	1	1	1	1	1	1	0	1	1	0	1	1	1	1	1	1	1	1	0	1	1	0	1	0
183766	ILNRRRRTA	C	94	102	HLA-B*08:01	81% - <100%	Dengue virus 2	ELISPOT	IFNg release	Positive	1	1	0	1	1	1	1	1	1	1	1	1	1	1	1	1	0	1	1	1	1	1	1	1	1	1	1	1	1	1	1	1	1	1	1	0	0	1	0	0	0
184378	LGMLQGRGPL	C	35	44	HLA-B*08:01	81% - <100%	Dengue virus 2	ELISPOT	IFNg release	Positive	1	1	1	1	1	1	1	1	1	1	1	1	1	1	0	1	1	1	1	1	1	1	1	1	1	1	1	1	1	1	1	1	1	1	1	0	1	1	0	1	0
184518	LNRRRRTAG	C	95	103	HLA-B*08:01	81% - <100%	Dengue virus 2	ELISPOT	IFNg release	Positive	1	1	0	1	1	1	1	1	1	1	1	1	1	1	1	1	0	1	1	1	1	1	1	1	1	1	1	1	1	1	1	1	1	1	1	0	0	1	0	0	0
184643	LTIPPTAGIL	C	21	30	HLA-B*08:01	81% - <100%	Dengue virus 2	ELISPOT	IFNg release	Positive	1	1	1	1	1	1	1	1	1	1	1	1	1	1	1	1	1	1	1	1	1	1	1	0	1	1	0	1	1	1	1	1	1	1	1	0	1	1	0	1	0
185104	NRRRRTAGV	C	96	104	HLA-B*08:01	81% - <100%	Dengue virus 2	ELISPOT	IFNg release	Positive	1	1	0	1	1	1	1	1	1	1	1	1	1	1	1	1	0	1	1	1	1	1	1	1	1	1	1	1	1	1	1	1	1	1	1	0	0	0	0	0	0
186354	VQQLTKRFSL	C	26	35	HLA-B*08:01	81% - <100%	Dengue virus 2	ELISPOT	IFNg release	Positive	1	1	1	1	1	1	1	1	1	1	1	1	1	1	0	1	1	1	1	1	1	1	1	1	1	1	1	1	1	1	1	1	1	1	1	0	1	1	0	1	0
150224	FTIMAAILAY	PreM	247	256	HLA-B*35:01	81% - <100%	Dengue virus 2	ELISPOT	IFNg release	Positive	1	1	1	1	1	1	1	1	1	1	1	1	1	1	1	1	1	1	1	1	1	0	1	1	0	1	1	1	1	1	1	1	1	1	1	1	1	1	1	0	1
150571	SLLFKTEDGV	PreM	136	145	HLA-A*02:01	81% - <100%	Dengue virus 2	ELISPOT	IFNg release	Positive	1	1	0	1	1	1	1	1	0	1	1	1	1	1	1	1	0	1	1	1	1	1	1	1	1	1	1	1	1	1	1	1	1	1	1	1	1	1	1	1	1
183621	HVQRIETWIL	PreM	233	242	HLA-B*08:01	81% - <100%	Dengue virus 2	ELISPOT	IFNg release	Positive	1	1	1	1	1	1	1	1	1	1	1	1	1	1	1	1	1	1	1	1	1	0	1	1	1	1	1	1	1	1	1	1	1	1	1	0	0	0	0	1	0
150115	AIYGAAFSGV	E	722	731	HLA-A*02:01	81% - <100%	Dengue virus 2	ELISPOT	IFNg release	Positive	1	1	1	1	1	1	1	1	1	1	1	1	1	0	0	1	1	1	1	1	1	1	0	0	0	1	1	1	1	1	1	1	1	1	1	1	1	1	1	1	0
180433	EPGQLKLNWF	E	663	672	HLA-B*35:01	81% - <100%	Dengue virus 2	ELISPOT	IFNg release	Positive	1	1	1	1	1	1	1	1	1	1	1	1	1	1	0	1	1	1	1	1	1	1	1	1	1	1	1	1	1	1	1	1	1	1	1	1	0	1	1	1	1
180453	FGAIYGAAF	E	720	728	HLA-B*35:01	81% - <100%	Dengue virus 2	ELISPOT	IFNg release	Positive	1	1	1	1	1	1	1	1	1	1	1	1	1	0	0	1	1	1	1	1	1	1	0	0	1	1	1	1	1	1	1	1	1	1	1	1	1	1	1	1	0
180726	RMAILGDTAWDFGSL	E	691	705	HLA-B7	81% - <100%	Dengue virus 2	ICS	IFNg release	Positive	1	1	1	1	1	1	1	1	1	1	1	1	1	1	0	1	1	1	1	1	1	1	1	1	1	1	1	1	1	1	1	1	1	1	1	1	1	1	1	1	0
182846	EAKQPATLR	E	329	337	HLA-A*68:01	81% - <100%	Dengue virus 2	ELISPOT	IFNg release	Positive	1	1	1	1	1	1	1	1	1	1	1	1	1	1	0	1	1	1	1	1	1	1	1	1	1	1	1	1	1	1	1	1	1	1	1	1	1	1	1	1	1
183310	GAAFSGVSW	E	725	733	HLA-B*58:01	81% - <100%	Dengue virus 2	ELISPOT	IFNg release	Positive	1	1	1	1	1	1	1	1	1	1	1	1	1	0	0	1	1	1	1	1	1	1	0	0	0	1	1	1	1	1	1	1	1	1	1	1	1	1	1	1	0
184899	MRGAKRMAI	E	686	694	HLA-B*08:01	81% - <100%	Dengue virus 2	ELISPOT	IFNg release	Positive	1	1	1	1	1	1	1	1	1	1	1	1	1	1	0	1	1	1	1	1	1	1	1	1	1	1	1	1	1	1	1	1	1	1	1	1	1	1	1	1	0
184900	MRGAKRMAIL	E	686	695	HLA-B*08:01	81% - <100%	Dengue virus 2	ELISPOT	IFNg release	Positive	1	1	1	1	1	1	1	1	1	1	1	1	1	1	0	1	1	1	1	1	1	1	1	1	1	1	1	1	1	1	1	1	1	1	1	1	1	1	1	1	0
27061	ILGDTAWDFG	E	96	105	HLA class I	81% - <100%	Dengue virus 2	ELISPOT	IFNg release	Positive	1	1	1	1	1	1	1	1	1	1	1	1	1	1	0	1	1	1	1	1	1	1	1	1	1	1	1	1	1	1	1	1	1	1	1	1	1	1	1	1	0
583702	GVSGGSWVDIVLEHGSCV	E	14	31	HLA class I	81% - <100%	Dengue virus 2	ICS	IFNg release	Positive-Low	1	1	1	1	1	1	1	1	1	1	1	1	1	1	1	1	1	1	1	1	1	1	1	1	1	1	1	1	1	1	1	1	1	1	0	1	1	1	1	1	1
150259	GPWHLGKLEM	NS1	1041	1050	HLA-B*35:01	81% - <100%	Dengue virus 2	ELISPOT	IFNg release	Positive	1	1	1	1	1	1	0	1	1	1	1	1	1	1	1	1	1	1	1	1	1	1	1	1	1	1	1	1	1	1	1	1	1	1	1	0	1	1	0	1	0
186474	WKTWGKAKML	NS1	890	899	HLA-B*08:01	81% - <100%	Dengue virus 2	ELISPOT	IFNg release	Positive	1	0	1	1	1	1	1	1	1	1	1	1	1	1	1	1	1	1	1	1	1	1	1	1	1	1	1	1	1	1	1	1	1	1	1	0	1	1	0	1	0
150448	MMATIGIAL	NS2a	1229	1237	HLA-B*35:01	81% - <100%	Dengue virus 2	ELISPOT	IFNg release	Positive	1	1	0	1	1	1	1	1	1	1	1	1	1	1	1	1	0	1	1	1	1	1	1	1	0	1	1	1	1	1	1	1	1	1	1	0	0	0	0	0	0
150449	MMATIGIALL	NS2a	1229	1238	HLA-A*02:01	81% - <100%	Dengue virus 2	ELISPOT	IFNg release	Positive	1	1	0	1	1	1	1	1	1	1	1	1	1	1	1	1	0	1	1	1	1	1	1	1	0	1	1	1	1	1	1	1	1	1	1	0	0	0	0	0	0
180671	MSFRDLGRVM	NS2a	1175	1184	HLA-B*35:01	81% - <100%	Dengue virus 2	ELISPOT	IFNg release	Positive	1	1	1	1	1	1	1	1	1	1	1	1	1	0	0	0	1	1	1	1	1	1	0	0	1	1	1	1	1	1	0	1	1	1	1	0	0	1	0	1	0
180717	RDLGRVMVMVGATMT	NS2a	51	65	HLA class I	81% - <100%	Dengue virus 2	ICS	IFNg release	Positive	1	1	1	1	1	1	1	1	1	1	1	1	1	0	0	0	1	1	1	1	1	1	0	0	1	1	1	1	1	1	0	1	1	1	1	0	0	1	0	1	0
150243	GLLTVCYVL	NS2b	1381	1389	HLA-A*02:01	81% - <100%	Dengue virus 2	ELISPOT	IFNg release	Positive	1	1	1	1	1	1	1	1	1	1	1	1	1	1	0	1	1	1	1	1	1	1	1	1	1	1	1	1	1	1	1	1	1	1	1	0	1	1	0	1	0
150304	IMAVGMVSI	NS2b	1353	1361	HLA-A*02:01	81% - <100%	Dengue virus 2	ELISPOT	IFNg release	Positive	1	1	1	1	1	1	1	1	1	1	1	1	1	1	1	1	1	1	1	1	1	1	1	1	0	1	1	1	1	1	1	1	1	1	1	0	1	1	0	1	0
183779	IMAVGMVSIL	NS2b	1353	1362	HLA-B*08:01	81% - <100%	Dengue virus 2	ELISPOT	IFNg release	Positive	1	1	1	1	1	1	1	1	1	1	1	1	1	1	1	1	1	1	1	1	1	1	1	1	0	1	1	1	1	1	1	1	1	1	1	0	1	1	0	1	0
10643	DVKKDLISY	NS3	71	79	HLA-B62	81% - <100%	Dengue virus 2	51 chromium	cytotoxicity	Positive	1	1	0	1	1	1	1	1	1	1	1	1	1	1	1	1	0	1	1	1	1	1	1	1	0	1	1	1	1	1	1	1	1	1	1	0	1	1	0	1	0
150346	KTFDSEYVK	NS3	1863	1871	HLA-A*11:01	81% - <100%	Dengue virus 2	multimer/tetramer	qualitative binding	Positive	1	1	1	1	0	1	1	1	1	1	1	1	1	1	1	1	1	1	1	1	1	1	1	1	1	1	1	1	1	1	1	1	1	1	1	0	1	1	0	1	0
150644	TPEGIIPSMF	NS3	1975	1984	HLA-B*53:01	81% - <100%	Dengue virus 2	ELISPOT	IFNg release	Positive	1	1	1	1	1	1	1	1	0	1	1	1	1	1	1	1	1	1	1	1	1	1	1	1	1	1	1	1	1	1	1	1	1	1	1	0	1	1	0	1	0
180407	DNINTPEGIIPSMFE	NS3	496	510	HLA-B35	81% - <100%	Dengue virus 2	ELISPOT	IFNg release	Positive	1	1	1	1	1	1	1	1	0	1	1	1	1	1	1	1	1	1	1	1	1	1	1	1	1	1	1	1	1	1	1	1	1	1	1	0	1	1	0	1	0
180589	LDARIYSDPLALKEF	NS3	596	610	HLA class I	81% - <100%	Dengue virus 2	ELISPOT	IFNg release	Positive	1	1	1	1	1	1	1	0	1	1	1	1	1	1	1	1	1	1	1	1	1	1	1	1	1	1	1	1	1	1	1	1	1	1	1	0	1	1	0	1	0
180623	LPVWLAYKV	NS3	2017	2025	HLA-B*51:01	81% - <100%	Dengue virus 2	ELISPOT	IFNg release	Positive	1	1	1	1	1	1	1	1	1	1	1	1	1	1	1	1	1	0	1	1	1	1	1	1	1	1	1	1	1	1	1	1	1	1	1	0	1	0	0	0	0
180624	LPVWLAYKVA	NS3	2017	2026	HLA-B*35:01	81% - <100%	Dengue virus 2	ELISPOT	IFNg release	Positive	1	1	1	1	1	1	1	1	1	1	1	1	1	1	1	1	1	0	1	1	1	1	1	1	1	1	1	1	1	1	1	1	1	1	1	0	1	0	0	0	0
182687	AYRIKQRGIL	NS3	1497	1506	HLA-B*08:01	81% - <100%	Dengue virus 2	ELISPOT	IFNg release	Positive	1	1	1	1	1	1	1	1	1	1	1	1	1	1	1	1	1	1	1	0	1	1	1	1	1	1	1	1	1	1	1	1	1	1	0	0	0	0	0	0	0
25929	IEPSWADVKKDLISY	NS3	65	79	HLA class I	81% - <100%	Dengue virus 2	ELISPOT	IFNg release	Positive	1	1	0	1	1	1	1	1	1	1	1	1	1	1	1	1	0	1	1	1	1	1	1	1	0	1	1	1	1	1	1	1	1	1	1	0	1	1	0	1	0
35238	LDNINTPEGIIPSMF	NS3	495	509	HLA class I	81% - <100%	Dengue virus 2	ELISPOT	IFNg release	Positive	1	1	1	1	1	1	1	1	0	1	1	1	1	1	1	1	1	1	1	1	1	1	1	1	1	1	1	1	1	1	1	1	1	1	1	0	1	1	0	1	0
46575	NYADRKWCF	NS3	2031	2039	HLA-A24	81% - <100%	Dengue virus 2	51 chromium	cytotoxicity	Positive	1	1	0	1	1	1	1	1	1	1	1	1	1	1	1	1	0	1	1	1	1	0	1	1	0	1	1	1	1	1	1	1	1	1	1	0	0	0	0	0	0
65550	TPEGIIPSMFEPERE	NS3	500	514	HLA class I	81% - <100%	Dengue virus 2	ELISPOT	IFNg release	Positive	1	1	1	1	1	1	1	1	0	1	1	1	1	1	1	1	1	1	1	1	1	1	1	1	1	1	1	1	1	1	1	1	1	1	1	0	1	1	0	1	0
790	ADVKKDLISYGGGWK	NS3	70	84	HLA class I	81% - <100%	Dengue virus 2	ELISPOT	IFNg release	Positive	1	1	0	1	1	1	1	1	1	1	1	1	1	1	1	1	0	1	1	1	1	1	1	1	0	1	1	1	1	1	1	1	1	1	1	0	1	1	0	1	0
150461	MTQKARNAL	NS4a	2110	2118	HLA-B*07:02	81% - <100%	Dengue virus 2	ELISPOT	IFNg release	Positive	1	1	1	1	1	1	1	1	0	1	1	1	1	1	1	1	1	1	1	1	1	1	1	1	1	1	1	1	1	1	1	1	1	1	1	0	0	0	0	0	0
185447	RNALDNLAVL	NS4a	2115	2124	HLA-B*08:01	81% - <100%	Dengue virus 2	ELISPOT	IFNg release	Positive	1	1	1	1	1	1	1	1	0	1	1	1	1	1	1	1	1	1	1	1	1	1	1	1	1	1	1	1	1	1	1	1	1	1	1	0	0	0	0	0	0
35568	LEKTKKDL	NS4b	2249	2256	HLA-B8	81% - <100%	Dengue virus 2	ELISPOT	IFNg release	Positive	1	1	1	1	1	1	1	1	1	1	1	1	1	1	1	1	1	1	1	1	1	1	1	1	1	0	1	1	1	1	1	1	1	1	1	0	1	1	0	1	0
150655	TPRMCTREEF	NS5	2881	2890	HLA-B*35:01	81% - <100%	Dengue virus 2	ELISPOT	IFNg release	Positive	1	1	0	1	1	1	1	1	1	1	1	1	1	1	1	1	0	1	1	1	1	1	1	1	1	1	1	1	1	1	1	1	1	1	1	0	1	1	0	1	0
150750	YTDYMPSMK	NS5	3370	3378	HLA-A*01:01	81% - <100%	Dengue virus 2	ELISPOT	IFNg release	Positive	1	1	1	1	1	1	1	1	1	1	1	1	1	1	0	1	1	1	1	0	1	1	1	1	1	1	1	1	1	1	1	1	1	1	1	0	1	1	0	1	0
161186	RLLTKPWDVVPMVTQ	NS5	326	340	HLA-B*55:02	81% - <100%	Dengue virus 2	ICS	IFNg release	Positive	1	1	1	1	1	1	1	1	1	1	1	1	1	1	1	1	1	1	1	0	1	1	1	1	1	1	1	1	1	1	1	1	1	1	1	0	1	1	0	1	0
174066	CGGLKNVREVKGLTKGG	NS5	2582	2598	HLA-A2	81% - <100%	Dengue virus 2	ICS	IFNg release	Positive	1	1	1	1	1	1	1	1	1	1	1	1	1	1	0	1	1	1	1	1	1	1	1	1	0	1	1	1	1	1	1	1	1	1	1	0	1	1	0	1	0
180606	LMKITAEWLW	NS5	2864	2873	HLA-B*53:01	81% - <100%	Dengue virus 2	ELISPOT	IFNg release	Positive	1	1	1	1	1	1	1	1	1	1	1	1	0	1	1	1	1	1	1	1	1	1	1	1	1	1	1	1	1	1	1	1	1	1	1	0	1	1	0	1	0
180667	MPSMKRFRRE	NS5	3374	3383	HLA-B*53:01	81% - <100%	Dengue virus 2	ELISPOT	IFNg release	Positive	1	1	1	0	1	1	1	1	1	1	1	1	1	1	1	1	1	1	1	1	1	1	1	1	1	1	1	1	1	1	0	1	1	1	1	0	1	1	0	1	0
182855	EAVEDGRFWE	NS5	2915	2924	HLA-B*58:01	81% - <100%	Dengue virus 2	ELISPOT	IFNg release	Positive	1	1	1	1	1	1	1	1	1	1	1	0	1	1	1	1	1	1	1	1	1	1	1	1	1	1	0	1	1	1	1	1	1	1	1	0	0	0	0	0	0
184234	KWKSRLNAL	NS5	2503	2511	HLA-B*08:01	81% - <100%	Dengue virus 2	ELISPOT	IFNg release	Positive	1	1	1	1	1	1	1	1	1	1	1	1	1	1	1	1	1	1	1	1	1	1	1	1	1	1	1	1	1	1	1	1	0	1	1	0	1	1	0	1	0
186219	VEDGRFWEL	NS5	2917	2925	HLA-B*40:01	81% - <100%	Dengue virus 2	ELISPOT	IFNg release	Positive	1	1	1	1	1	1	1	1	1	1	1	1	1	1	1	1	1	1	1	1	1	1	1	1	1	1	0	1	1	1	1	1	1	1	1	0	0	0	0	0	0
32943	KPWDVVPMV	NS5	2821	2829	HLA-B*51:01	81% - <100%	Dengue virus 2	51 chromium	cytotoxicity	Positive	1	1	1	1	1	1	1	1	1	1	1	1	1	1	1	1	1	1	1	0	1	1	1	1	1	1	1	1	1	1	1	1	1	1	1	0	1	1	0	1	0
185491	RREEEEAGVL	NS5	3381	3390	HLA-B*08:01	51%-80%	Dengue virus 2	ELISPOT	IFNg release	Positive	1	1	1	0	1	1	1	1	1	1	1	1	1	0	0	0	1	1	1	1	1	1	0	0	1	1	1	1	1	1	0	1	1	1	1	0	1	1	0	1	0
150634	TLMAMDLGEL	PreM	149	158	HLA-A*02:01	>0% - 50%	Dengue virus 2	ELISPOT	IFNg release	Positive	0	0	0	0	1	0	0	0	0	0	0	0	0	0	0	0	0	0	0	0	0	0	0	0	0	0	0	0	0	0	0	0	0	0	0	1	1	1	1	1	1
180818	WILRHPGFTMMAAIL	PreM	126	140	HLA class I	>0% - 50%	Dengue virus 2	ICS	IFNg release	Positive	0	0	0	0	0	0	0	0	0	0	0	0	0	0	0	0	0	0	0	0	0	0	0	0	1	0	0	0	0	0	0	0	0	0	0	0	0	0	0	1	0
150319	ITEAELTGY	E	450	458	HLA-A*01:01	>0% - 50%	Dengue virus 2	ELISPOT	IFNg release	Positive	0	0	1	0	0	0	0	0	0	0	0	0	0	0	0	0	1	0	0	0	0	0	0	0	1	0	0	0	0	0	0	0	0	0	0	1	1	1	1	1	1
150578	SPCKIPFEIM	E	611	620	HLA-B*35:01	>0% - 50%	Dengue virus 2	ELISPOT	IFNg release	Positive	0	0	1	0	0	0	0	0	0	0	0	0	0	0	0	0	1	0	0	0	0	0	0	0	1	0	0	0	0	0	0	0	0	0	0	1	1	1	1	1	1
180528	IPFEIMDLEK	E	615	624	HLA-B*35:01	>0% - 50%	Dengue virus 2	ELISPOT	IFNg release	Positive	0	0	1	0	0	0	0	0	0	0	0	0	0	0	0	0	1	0	0	0	0	0	0	0	1	0	0	0	0	0	0	0	0	0	0	1	1	1	1	1	1
183781	IMDLEKRHVL	E	619	628	HLA-B*08:01	>0% - 50%	Dengue virus 2	ELISPOT	IFNg release	Positive	0	0	1	0	0	0	0	0	0	0	0	0	0	0	0	0	1	0	0	0	0	0	0	0	1	0	0	0	0	0	0	0	0	0	0	1	1	1	1	1	1
184755	MDLEKRHVL	E	620	628	HLA-B*08:01	>0% - 50%	Dengue virus 2	ELISPOT	IFNg release	Positive	0	0	1	0	0	0	0	0	0	0	0	0	0	0	0	0	1	0	0	0	0	0	0	0	1	0	0	0	0	0	0	0	0	0	0	1	1	1	1	1	1
150300	ILLVAVSFV	NS2a	1146	1154	HLA-A*02:01	>0% - 50%	Dengue virus 2	ELISPOT	IFNg release	Positive	0	0	1	0	0	0	0	0	0	0	0	0	0	0	0	0	1	0	0	0	0	0	0	0	0	0	0	0	0	0	0	0	0	0	0	0	1	1	0	1	0
180735	RVGTKHAILLVAVSF	NS2a	1153	1167	HLA class I	>0% - 50%	Dengue virus 2	ICS	IFNg release	Positive	0	0	1	0	0	0	0	0	0	0	0	0	0	0	0	0	1	0	0	0	0	0	0	0	0	0	0	0	0	0	0	0	0	0	0	0	1	1	0	1	0
180360	AAEGINYADRRWCFD	NS3	2026	2040	HLA-A24	>0% - 50%	Dengue virus 2	ELISPOT	IFNg release	Positive	0	0	1	0	0	0	0	0	0	0	0	0	0	0	0	0	1	0	0	0	0	0	0	0	1	0	0	0	0	0	0	0	0	0	0	0	1	1	0	1	0
180611	LMRRGDLPVWLAYRV	NS3	2011	2025	HLA-B*55:02	>0% - 50%	Dengue virus 2	ICS	IFNg release	Positive	0	0	0	0	0	0	0	0	0	0	0	0	0	0	0	0	0	1	0	0	0	0	0	0	0	0	0	0	0	0	0	0	0	0	0	0	0	1	0	1	0
180625	LPVWLAYRV	NS3	2017	2025	HLA-B*51:01	>0% - 50%	Dengue virus 2	ELISPOT	IFNg release	Positive	0	0	0	0	0	0	0	0	0	0	0	0	0	0	0	0	0	1	0	0	0	0	0	0	0	0	0	0	0	0	0	0	0	0	0	0	0	1	0	1	0
180626	LPVWLAYRVA	NS3	2017	2026	HLA-B*51:01	>0% - 50%	Dengue virus 2	ELISPOT	IFNg release	Positive	0	0	0	0	0	0	0	0	0	0	0	0	0	0	0	0	0	1	0	0	0	0	0	0	0	0	0	0	0	0	0	0	0	0	0	0	0	1	0	1	0
27770	INYADRRWCF	NS3	555	564	HLA-A24	>0% - 50%	Dengue virus 2	ELISPOT	IFNg release	Positive	0	0	1	0	0	0	0	0	0	0	0	0	0	0	0	0	1	0	0	0	0	0	0	0	1	0	0	0	0	0	0	0	0	0	0	0	1	1	0	1	0
46576	NYADRRWCF	NS3	556	564	HLA-A24	>0% - 50%	Dengue virus 2	ELISPOT	IFNg release	Positive	0	0	1	0	0	0	0	0	0	0	0	0	0	0	0	0	1	0	0	0	0	0	0	0	1	0	0	0	0	0	0	0	0	0	0	0	1	1	0	1	0
37557	LLLTLLATV	NS4a	56	64	HLA-A2	>0% - 50%	Dengue virus 2	ICS	IFNg release	Positive	0	0	1	0	0	0	0	0	0	0	0	0	1	1	1	1	1	0	0	0	0	0	1	1	0	0	0	0	0	0	1	0	0	0	0	0	1	1	0	1	0
180483	GKKKTPRMCTREEFT	NS5	2877	2891	HLA class I	>0% - 50%	Dengue virus 2	ELISPOT	IFNg release	Positive	0	0	0	0	0	0	0	0	0	0	0	0	0	0	0	0	0	0	0	0	0	0	0	0	1	0	0	0	0	0	0	0	0	0	0	0	1	1	0	1	0
180681	NEEYTDYMPSMKRFR	NS5	3367	3381	HLA class I	>0% - 50%	Dengue virus 2	ELISPOT	IFNg release	Positive	0	0	0	0	0	0	0	0	0	0	0	0	0	1	0	1	0	0	0	0	0	0	1	1	1	0	0	0	0	0	1	0	0	0	0	0	1	1	0	1	0
184373	LGKKKTPRM	NS5	2876	2884	HLA-B*08:01	>0% - 50%	Dengue virus 2	ELISPOT	IFNg release	Positive	0	0	0	0	0	0	0	0	0	0	0	0	0	0	0	0	0	0	0	0	0	0	0	0	1	0	0	0	0	0	0	0	0	0	0	0	1	1	0	1	0
184957	MTTEDMLTVW	NS5	3295	3304	HLA-B*58:01	>0% - 50%	Dengue virus 2	ICS	IFNg release	Positive	0	0	0	0	0	0	0	0	0	0	0	0	0	0	0	0	0	0	0	0	0	0	0	0	0	0	1	0	0	0	0	0	0	0	0	0	1	1	0	1	0
74305	YILRDVSKK	NS5	517	525	HLA class I	>0% - 50%	Dengue virus 2	ELISPOT	IFNg release	Positive	0	0	1	0	0	0	0	0	0	0	0	0	0	0	0	0	1	0	0	0	0	0	0	0	0	0	0	1	0	0	0	0	0	0	0	0	1	1	0	1	0

CD8 DENV-3 Epitopes

											NCBI IDs of DENV-3 circulating strains from Brazil, (100% identity=1,green ; else 0, red)																																														DENV-3 constructs of vaccines					
Epitope_ID	AA_Seq	Protein_Name	Start	End	Allele	step	Serotype	Method/Technique	Assay Group	Qualitative Measure	ACY70744.1	ACY70765.1	ACY70760.1	ACY70761.1	ACY70767.1	ACY70771.1	ACY70777.1	ACY70764.1	ACY70772.1	ACY70769.1	ACY70768.1	ACY70748.1	ACY70755.1	ACO06166.1	AGN94904.1	AGN94907.1	AGN94896.1	AGN94897.1	AFK83756.1	ACY70773.1	ACO06174.1	ACY70750.1	ACY70774.1	AGN94902.1	AGN94899.1	AGN94908.1	AGN94910.1	AFK83755.1	ACY70770.1	ACY70776.1	ACW82871.1	ACY70757.1	ACO06163.1	ACW82872.1	ACY70756.1	ACO06172.1	AGN94898.1	AGN94900.1	AGN94903.1	ACO06169.1	ACY70749.1	AEV42062.1	AGN94905.1	AGN94901.1	AFK83754.1	TV003	TDEN	DPIV	Dengvaxia	DENVax	TVDV
3124	AMKGLPIRY	NS3	1708	1716	HLA-B62	0%	Dengue virus 3	51 chromium	cytotoxicity	Positive	0	0	0	0	0	0	0	0	0	0	0	0	0	0	0	0	0	0	0	0	0	0	0	0	0	0	0	0	0	0	0	0	0	0	0	0	0	0	0	0	0	0	0	0	0	0	0	0	0	0	0
590435	TRVVAAEMEEAMKGLPI	NS3	1698	1714	HLA class I	0%	Dengue virus 3	ICS	IFNg release	Positive-Low	0	0	0	0	0	0	0	0	0	0	0	0	0	0	0	0	0	0	0	0	0	0	0	0	0	0	0	0	0	0	0	0	0	0	0	0	0	0	0	0	0	0	0	0	0	0	0	0	0	0	0
32944	KPWDVVPTV	NS5	2819	2827	HLA class I	0%	Dengue virus 3	51 chromium	cytotoxicity	Positive	0	0	0	0	0	0	0	0	0	0	0	0	0	0	0	0	0	0	0	0	0	0	0	0	0	0	0	0	0	0	0	0	0	0	0	0	0	0	0	0	0	0	0	0	0	0	0	0	0	0	0
180577	KSGAIKVLK	C	74	82	HLA-A*11:01	100%	Dengue virus 3	ELISPOT	IFNg release	Positive	1	1	1	1	1	1	1	1	1	1	1	1	1	1	1	1	1	1	1	1	1	1	1	1	1	1	1	1	1	1	1	1	1	1	1	1	1	1	1	1	1	1	1	1	1	0	1	0	0	0	0
184260	LAIPPTAGVL	C	57	66	HLA-B*08:01	100%	Dengue virus 3	ELISPOT	IFNg release	Positive	1	1	1	1	1	1	1	1	1	1	1	1	1	1	1	1	1	1	1	1	1	1	1	1	1	1	1	1	1	1	1	1	1	1	1	1	1	1	1	1	1	1	1	1	1	1	1	1	0	0	0
184845	MLKRVRNRV	C	15	23	HLA-B*08:01	100%	Dengue virus 3	ELISPOT	IFNg release	Positive	1	1	1	1	1	1	1	1	1	1	1	1	1	1	1	1	1	1	1	1	1	1	1	1	1	1	1	1	1	1	1	1	1	1	1	1	1	1	1	1	1	1	1	1	1	1	1	1	0	0	0
180464	FTILALFLAH	PreM	247	256	HLA-B*35:01	100%	Dengue virus 3	ELISPOT	IFNg release	Positive	1	1	1	1	1	1	1	1	1	1	1	1	1	1	1	1	1	1	1	1	1	1	1	1	1	1	1	1	1	1	1	1	1	1	1	1	1	1	1	1	1	1	1	1	1	1	1	1	1	1	1
180504	HPGFTILALF	PreM	244	253	HLA-B*35:01	100%	Dengue virus 3	ELISPOT	IFNg release	Positive	1	1	1	1	1	1	1	1	1	1	1	1	1	1	1	1	1	1	1	1	1	1	1	1	1	1	1	1	1	1	1	1	1	1	1	1	1	1	1	1	1	1	1	1	1	1	1	1	1	1	1
184658	LTQKVVIFIL	PreM	262	271	HLA-B*08:01	100%	Dengue virus 3	ELISPOT	IFNg release	Positive	1	1	1	1	1	1	1	1	1	1	1	1	1	1	1	1	1	1	1	1	1	1	1	1	1	1	1	1	1	1	1	1	1	1	1	1	1	1	1	1	1	1	1	1	1	1	1	1	1	1	1
185685	SLLFKTASG	PreM	136	144	HLA-B*08:01	100%	Dengue virus 3	ELISPOT	IFNg release	Positive	1	1	1	1	1	1	1	1	1	1	1	1	1	1	1	1	1	1	1	1	1	1	1	1	1	1	1	1	1	1	1	1	1	1	1	1	1	1	1	1	1	1	1	1	1	1	1	1	1	1	1
180613	LPEEQDQNY	E	82	90	HLA-B*35:01	100%	Dengue virus 3	ELISPOT	IFNg release	Positive	1	1	1	1	1	1	1	1	1	1	1	1	1	1	1	1	1	1	1	1	1	1	1	1	1	1	1	1	1	1	1	1	1	1	1	1	1	1	1	1	1	1	1	1	1	0	1	0	1	1	1
180657	MLVTPSMTM	E	273	281	HLA-B*35:01	100%	Dengue virus 3	ELISPOT	IFNg release	Positive	1	1	1	1	1	1	1	1	1	1	1	1	1	1	1	1	1	1	1	1	1	1	1	1	1	1	1	1	1	1	1	1	1	1	1	1	1	1	1	1	1	1	1	1	1	1	0	1	0	0	0
184299	LATLRKLCI	E	53	61	HLA-B*08:01	100%	Dengue virus 3	ELISPOT	IFNg release	Positive	1	1	1	1	1	1	1	1	1	1	1	1	1	1	1	1	1	1	1	1	1	1	1	1	1	1	1	1	1	1	1	1	1	1	1	1	1	1	1	1	1	1	1	1	1	1	1	1	1	1	1
184536	LPEYGTLGLE	E	453	462	HLA-B*35:01	100%	Dengue virus 3	ELISPOT	IFNg release	Positive	1	1	1	1	1	1	1	1	1	1	1	1	1	1	1	1	1	1	1	1	1	1	1	1	1	1	1	1	1	1	1	1	1	1	1	1	1	1	1	1	1	1	1	1	1	1	1	1	1	1	1
186029	TPTWNRKEL	E	226	234	HLA-B*07:02	100%	Dengue virus 3	ELISPOT	IFNg release	Positive-Low	1	1	1	1	1	1	1	1	1	1	1	1	1	1	1	1	1	1	1	1	1	1	1	1	1	1	1	1	1	1	1	1	1	1	1	1	1	1	1	1	1	1	1	1	1	0	1	0	1	1	1
589817	SGATWVDVVLEHGGCV	E	268	283	HLA class I	100%	Dengue virus 3	ICS	IFNg release	Positive-High	1	1	1	1	1	1	1	1	1	1	1	1	1	1	1	1	1	1	1	1	1	1	1	1	1	1	1	1	1	1	1	1	1	1	1	1	1	1	1	1	1	1	1	1	1	1	1	1	1	1	1
150537	RPGYHTQTA	NS1	1030	1038	HLA-B*07:02	100%	Dengue virus 3	ELISPOT	IFNg release	Positive	1	1	1	1	1	1	1	1	1	1	1	1	1	1	1	1	1	1	1	1	1	1	1	1	1	1	1	1	1	1	1	1	1	1	1	1	1	1	1	1	1	1	1	1	1	1	1	1	0	0	0
150546	RSCTLPPLRY	NS1	1087	1096	HLA-A*01:01	100%	Dengue virus 3	ELISPOT	IFNg release	Positive	1	1	1	1	1	1	1	1	1	1	1	1	1	1	1	1	1	1	1	1	1	1	1	1	1	1	1	1	1	1	1	1	1	1	1	1	1	1	1	1	1	1	1	1	1	1	1	1	0	0	0
182939	EKEENMVKSL	NS1	1113	1122	HLA-B*08:01	100%	Dengue virus 3	ELISPOT	IFNg release	Positive	1	1	1	1	1	1	1	1	1	1	1	1	1	1	1	1	1	1	1	1	1	1	1	1	1	1	1	1	1	1	1	1	1	1	1	1	1	1	1	1	1	1	1	1	1	1	1	1	0	0	0
185734	SPKRLATAI	NS1	811	819	HLA-B*08:01	100%	Dengue virus 3	ELISPOT	IFNg release	Positive	1	1	1	1	1	1	1	1	1	1	1	1	1	1	1	1	1	1	1	1	1	1	1	1	1	1	1	1	1	1	1	1	1	1	1	1	1	1	1	1	1	1	1	1	1	1	1	1	0	0	0
180622	LPVWLAHKVA	NS3	226	235	HLA-B*35:01	100%	Dengue virus 3	ELISPOT	IFNg release	Positive	1	1	1	1	1	1	1	1	1	1	1	1	1	1	1	1	1	1	1	1	1	1	1	1	1	1	1	1	1	1	1	1	1	1	1	1	1	1	1	1	1	1	1	1	1	1	1	1	0	0	0
182917	EFKDFAAGR	NS3	2083	2091	HLA-A*33:01	100%	Dengue virus 3	ELISPOT	IFNg release	Positive	1	1	1	1	1	1	1	1	1	1	1	1	1	1	1	1	1	1	1	1	1	1	1	1	1	1	1	1	1	1	1	1	1	1	1	1	1	1	1	1	1	1	1	1	1	1	1	1	0	0	0
184065	KLNDWDFVV	NS3	399	407	HLA-A*02:01	100%	Dengue virus 3	ELISPOT	IFNg release	Positive-High	1	1	1	1	1	1	1	1	1	1	1	1	1	1	1	1	1	1	1	1	1	1	1	1	1	1	1	1	1	1	1	1	1	1	1	1	1	1	1	1	1	1	1	1	1	1	1	1	0	0	0
186234	VEIWTKEGEK	NS3	2053	2062	HLA-B*40:01	100%	Dengue virus 3	ELISPOT	IFNg release	Positive	1	1	1	1	1	1	1	1	1	1	1	1	1	1	1	1	1	1	1	1	1	1	1	1	1	1	1	1	1	1	1	1	1	1	1	1	1	1	1	1	1	1	1	1	1	1	1	1	0	0	0
19434	GESRKTFVE	NS3	2001	2009	HLA-B7	100%	Dengue virus 3	51 chromium	cytotoxicity	Positive	1	1	1	1	1	1	1	1	1	1	1	1	1	1	1	1	1	1	1	1	1	1	1	1	1	1	1	1	1	1	1	1	1	1	1	1	1	1	1	1	1	1	1	1	1	1	1	1	0	0	0
19435	GESRKTFVEL	NS3	211	220	HLA-B*40:01	100%	Dengue virus 3	ICS	IFNg release	Positive	1	1	1	1	1	1	1	1	1	1	1	1	1	1	1	1	1	1	1	1	1	1	1	1	1	1	1	1	1	1	1	1	1	1	1	1	1	1	1	1	1	1	1	1	1	1	1	1	0	0	0
22820	GTSGSPIINR	NS3	133	142	HLA-A*11:01	100%	Dengue virus 3	51 chromium	cytotoxicity	Positive	1	1	1	1	1	1	1	1	1	1	1	1	1	1	1	1	1	1	1	1	1	1	1	1	1	1	1	1	1	1	1	1	1	1	1	1	1	1	1	1	1	1	1	1	1	1	1	1	0	0	0
22821	GTSGSPIINRE	NS3	133	143	HLA-A11	100%	Dengue virus 3	ICS	IFNg release	Positive	1	1	1	1	1	1	1	1	1	1	1	1	1	1	1	1	1	1	1	1	1	1	1	1	1	1	1	1	1	1	1	1	1	1	1	1	1	1	1	1	1	1	1	1	1	1	1	1	0	0	0
34605	KYTDRKWCF	NS3	240	248	HLA-A24	100%	Dengue virus 3	ELISPOT	IFNg release	Positive	1	1	1	1	1	1	1	1	1	1	1	1	1	1	1	1	1	1	1	1	1	1	1	1	1	1	1	1	1	1	1	1	1	1	1	1	1	1	1	1	1	1	1	1	1	1	1	1	0	0	0
62183	SVKKDLISY	NS3	71	79	HLA-B62	100%	Dengue virus 3	51 chromium	cytotoxicity	Positive	1	1	1	1	1	1	1	1	1	1	1	1	1	1	1	1	1	1	1	1	1	1	1	1	1	1	1	1	1	1	1	1	1	1	1	1	1	1	1	1	1	1	1	1	1	1	1	1	0	0	0
183165	FFMMVLLIPE	NS4a	2205	2214	HLA-A*33:01	100%	Dengue virus 3	ELISPOT	IFNg release	Positive	1	1	1	1	1	1	1	1	1	1	1	1	1	1	1	1	1	1	1	1	1	1	1	1	1	1	1	1	1	1	1	1	1	1	1	1	1	1	1	1	1	1	1	1	1	1	1	1	0	0	0
37494	LLLGLMILL	NS4a	2148	2156	HLA-A2	100%	Dengue virus 3	ICS	IFNg release	Positive	1	1	1	1	1	1	1	1	1	1	1	1	1	1	1	1	1	1	1	1	1	1	1	1	1	1	1	1	1	1	1	1	1	1	1	1	1	1	1	1	1	1	1	1	1	1	1	1	0	0	0
180381	ATGPITTLW	NS4b	2439	2447	HLA-B*58:01	100%	Dengue virus 3	ELISPOT	IFNg release	Positive	1	1	1	1	1	1	1	1	1	1	1	1	1	1	1	1	1	1	1	1	1	1	1	1	1	1	1	1	1	1	1	1	1	1	1	1	1	1	1	1	1	1	1	1	1	1	1	1	0	0	0
180788	TTKRDLGMSK	NS4b	2250	2259	HLA-A*11:01	100%	Dengue virus 3	ELISPOT	IFNg release	Positive	1	1	1	1	1	1	1	1	1	1	1	1	1	1	1	1	1	1	1	1	1	1	1	1	1	1	1	1	1	1	1	1	1	1	1	1	1	1	1	1	1	1	1	1	1	1	1	1	0	0	0
183796	IMKSVGTGK	NS4b	2481	2489	HLA-A*03:01	100%	Dengue virus 3	ELISPOT	IFNg release	Positive	1	1	1	1	1	1	1	1	1	1	1	1	1	1	1	1	1	1	1	1	1	1	1	1	1	1	1	1	1	1	1	1	1	1	1	1	1	1	1	1	1	1	1	1	1	1	1	1	0	0	0
37534	LLLMRTSWA	NS4b	2423	2431	HLA-A2	100%	Dengue virus 3	biological activity	proliferation	Positive	1	1	1	1	1	1	1	1	1	1	1	1	1	1	1	1	1	1	1	1	1	1	1	1	1	1	1	1	1	1	1	1	1	1	1	1	1	1	1	1	1	1	1	1	1	1	1	1	0	0	0
180737	RYLEFEALGF	NS5	2971	2980	HLA-A*24:02	100%	Dengue virus 3	ELISPOT	IFNg release	Positive	1	1	1	1	1	1	1	1	1	1	1	1	1	1	1	1	1	1	1	1	1	1	1	1	1	1	1	1	1	1	1	1	1	1	1	1	1	1	1	1	1	1	1	1	1	1	1	1	0	0	0
185710	SMINGVVKL	NS5	318	326	HLA-A*02:01	100%	Dengue virus 3	ELISPOT	IFNg release	Positive	1	1	1	1	1	1	1	1	1	1	1	1	1	1	1	1	1	1	1	1	1	1	1	1	1	1	1	1	1	1	1	1	1	1	1	1	1	1	1	1	1	1	1	1	1	1	1	1	0	0	0
13119	ELKGMSYAM	E	155	163	HLA-B*08:01	81% - <100%	Dengue virus 3	ELISPOT	IFNg release	Positive	1	1	1	1	1	1	1	1	1	1	1	1	1	1	1	1	1	1	1	1	1	1	1	1	1	1	1	1	1	1	1	1	1	1	1	1	1	1	1	1	1	0	1	1	1	1	1	1	1	1	0
15796	FFDLPLPWT	E	211	219	HLA-A2	81% - <100%	Dengue virus 3	ICS	IFNg release	Positive	1	1	1	1	1	1	1	1	1	1	1	1	1	1	1	1	1	1	1	1	1	0	1	1	1	1	1	1	1	1	1	1	1	1	1	1	1	1	1	1	1	1	1	1	1	1	1	1	1	1	1
183843	IQNSGGTSIF	E	131	140	HLA-B*15:01	81% - <100%	Dengue virus 3	ELISPOT	IFNg release	Positive	1	1	1	1	1	1	1	1	1	1	1	1	1	1	1	1	1	1	1	1	1	1	1	1	1	1	1	1	1	1	1	1	1	1	1	1	1	1	1	1	1	0	1	1	1	0	1	0	1	0	0
184568	LPWTSGATT	E	496	504	HLA-B*35:01	81% - <100%	Dengue virus 3	ELISPOT	IFNg release	Positive	1	1	1	1	1	1	1	1	1	1	1	1	1	1	1	1	1	1	1	1	1	1	1	1	1	1	1	1	0	1	1	1	1	1	1	0	1	1	1	1	1	1	1	1	1	1	1	1	1	0	1
184924	MSYAMCTNTF	E	575	584	HLA-B*35:01	81% - <100%	Dengue virus 3	ELISPOT	IFNg release	Positive	1	1	1	1	1	1	1	1	1	1	1	1	1	1	1	1	1	1	1	1	1	1	1	1	1	1	1	1	1	1	1	1	1	1	1	1	1	0	1	1	1	0	1	1	1	0	0	0	0	0	0
184965	MVHQIFGSAY	E	713	722	HLA-B*15:01	81% - <100%	Dengue virus 3	ELISPOT	IFNg release	Positive	1	1	1	1	1	1	1	1	1	1	1	1	1	0	1	1	1	1	1	1	1	1	1	1	1	1	1	1	1	1	1	1	1	1	1	1	1	1	1	1	1	1	1	1	1	1	1	1	1	1	1
185419	RLKMDKLEL	E	148	156	HLA-B*08:01	81% - <100%	Dengue virus 3	ELISPOT	IFNg release	Positive	1	1	1	1	1	1	1	1	1	1	1	1	1	1	1	1	1	1	1	1	1	1	1	1	1	1	1	1	1	1	1	1	1	1	1	1	1	1	1	1	1	0	1	1	1	1	1	1	1	1	0
180738	RYMGEDGCWY	NS1	1095	1104	HLA-A*24:02	81% - <100%	Dengue virus 3	ELISPOT	IFNg release	Positive	1	1	1	1	1	1	1	1	1	1	1	1	1	1	1	1	1	0	1	1	1	1	1	1	1	1	1	1	1	1	1	1	1	1	1	1	1	1	1	1	1	1	1	1	1	1	1	1	0	0	0
183723	IKLTVVVGDI	NS1	857	866	HLA-B*08:01	81% - <100%	Dengue virus 3	ELISPOT	IFNg release	Positive	1	1	1	1	1	1	1	1	1	1	1	1	0	1	1	1	1	1	1	1	0	1	1	1	1	1	1	1	1	1	1	1	1	1	1	1	1	1	1	1	1	1	1	1	1	1	1	1	0	0	0
184585	LRTTTVSGKL	NS1	64	73	HLA-B*08:01	81% - <100%	Dengue virus 3	ELISPOT	IFNg release	Positive	1	1	1	1	1	1	1	1	1	1	1	1	1	1	1	1	1	1	1	1	1	1	1	1	1	1	1	1	1	1	1	0	1	1	1	1	1	1	1	1	1	1	1	1	1	1	1	1	0	0	0
185573	RYMGEDGCW	NS1	1095	1103	HLA-A*24:02	81% - <100%	Dengue virus 3	ELISPOT	IFNg release	Positive	1	1	1	1	1	1	1	1	1	1	1	1	1	1	1	1	1	0	1	1	1	1	1	1	1	1	1	1	1	1	1	1	1	1	1	1	1	1	1	1	1	1	1	1	1	1	1	1	0	0	0
183587	HMIAGVFFTF	NS2a	368	377	HLA-A*24:02	81% - <100%	Dengue virus 3	ELISPOT	IFNg release	Positive	1	1	1	1	1	1	1	1	1	1	1	1	1	0	1	1	1	1	1	1	1	1	1	1	1	0	1	1	1	1	1	1	1	1	1	1	0	0	1	1	1	0	1	1	0	0	0	0	0	0	0
184254	LAILFEEVM	NS2a	1140	1148	HLA-B*35:01	81% - <100%	Dengue virus 3	ELISPOT	IFNg release	Positive	1	1	1	1	1	1	1	1	1	1	1	1	1	1	1	1	1	1	1	1	1	1	1	1	1	1	1	1	1	0	1	1	1	1	1	1	1	1	1	1	1	1	1	1	1	1	1	1	0	0	0
184819	MIAGVFFTF	NS2a	369	377	HLA-B*35:01	81% - <100%	Dengue virus 3	ELISPOT	IFNg release	Positive	1	1	1	1	1	1	1	1	1	1	1	1	1	0	1	1	1	1	1	1	1	1	1	1	1	0	1	1	1	1	1	1	1	1	1	1	0	0	1	1	1	0	1	1	0	0	0	0	0	0	0
185978	TMGVLCLAIL	NS2a	1134	1143	HLA-B*08:01	81% - <100%	Dengue virus 3	ELISPOT	IFNg release	Positive	1	1	1	1	1	1	1	1	1	1	1	1	1	1	1	1	1	1	1	1	1	1	1	1	1	1	1	1	1	0	1	1	1	1	1	1	1	1	1	1	1	1	1	1	1	1	0	1	0	0	0
1067501	KPIDDRFAN	NS5	3158	3166	HLA-B*07:02	81% - <100%	Dengue virus 3	ELISPOT	IFNg release	Positive	1	1	1	1	1	1	1	1	1	1	1	1	1	1	1	1	1	1	1	1	1	1	1	0	1	1	1	1	1	1	1	1	1	1	1	1	1	1	1	1	1	1	1	1	0	1	1	1	0	0	0
180572	KPRLCTREEF	NS5	2879	2888	HLA-B*07:02	81% - <100%	Dengue virus 3	ELISPOT	IFNg release	Positive	1	1	1	1	1	1	1	1	1	1	1	1	1	1	1	1	1	1	0	0	1	1	1	1	1	1	1	1	1	1	1	1	1	1	1	1	1	1	1	1	1	0	1	1	1	0	0	0	0	0	0
180678	MVSRLLLNR	NS5	2723	2731	HLA-A*11:01	81% - <100%	Dengue virus 3	ELISPOT	IFNg release	Positive	1	1	1	1	1	1	1	1	1	1	1	1	1	1	1	1	1	1	1	1	1	1	1	1	1	0	1	1	1	1	1	1	1	1	1	1	0	0	1	1	1	1	1	1	1	1	1	1	0	0	0
182553	ALLALNDMGK	NS5	3167	3176	HLA-A*03:01	81% - <100%	Dengue virus 3	ELISPOT	IFNg release	Positive	1	1	1	1	1	1	1	1	1	0	1	1	1	1	1	1	1	1	1	1	1	1	1	1	1	1	1	1	1	1	1	1	1	1	1	1	1	1	1	1	1	1	1	1	1	1	1	1	0	0	0
186215	VEDEDFWKL	NS5	2915	2923	HLA-B*40:01	81% - <100%	Dengue virus 3	ELISPOT	IFNg release	Positive	1	1	1	1	1	1	1	1	1	1	1	1	1	1	1	1	1	1	1	1	1	1	1	1	1	1	1	1	1	1	1	1	1	1	1	1	1	1	1	1	1	0	1	1	1	0	0	0	0	0	0
186175	TWRDMAHTLI	NS2a	1174	1183	HLA-A*24:02	51%-80%	Dengue virus 3	ELISPOT	IFNg release	Positive	0	1	0	0	1	0	1	0	1	0	0	0	1	1	1	1	1	1	0	1	1	0	0	1	1	1	1	1	0	1	0	0	1	0	0	1	1	1	1	1	0	1	0	1	1	1	1	1	0	0	0
180801	VMGITAEWLW	NS5	2862	2871	HLA-B*53:01	51%-80%	Dengue virus 3	ELISPOT	IFNg release	Positive	0	1	0	1	1	0	1	0	1	1	0	0	1	1	1	1	1	1	1	1	1	0	1	1	1	1	1	1	0	1	0	1	1	0	0	1	1	1	1	1	1	0	1	1	1	0	0	0	0	0	0
180390	CLMMMLPATL	C	104	113	HLA-A*02:01	>0% - 50%	Dengue virus 3	ELISPOT	IFNg release	Positive	0	0	0	0	0	0	0	0	0	0	0	0	0	0	0	0	0	0	0	0	0	0	0	0	0	0	0	0	0	0	0	0	0	0	0	0	0	0	0	0	0	1	0	0	0	1	1	1	0	0	0
180609	LMMMLPATL	C	105	113	HLA-A*02:01	>0% - 50%	Dengue virus 3	ELISPOT	IFNg release	Positive	0	0	0	0	0	0	0	0	0	0	0	0	0	0	0	0	0	0	0	0	0	0	0	0	0	0	0	0	0	0	0	0	0	0	0	0	0	0	0	0	0	1	0	0	0	1	1	1	0	0	0
184264	LAKRFSRGL	C	29	37	HLA-B*08:01	>0% - 50%	Dengue virus 3	ELISPOT	IFNg release	Positive	0	0	0	0	0	0	0	0	0	0	0	0	0	0	0	0	0	0	0	0	0	0	0	0	0	0	0	0	0	0	0	0	0	0	0	0	0	0	0	0	0	1	0	0	0	1	1	1	0	0	0
184265	LAKRFSRGLL	C	29	38	HLA-B*08:01	>0% - 50%	Dengue virus 3	ELISPOT	IFNg release	Positive	0	0	0	0	0	0	0	0	0	0	0	0	0	0	0	0	0	0	0	0	0	0	0	0	0	0	0	0	0	0	0	0	0	0	0	0	0	0	0	0	0	1	0	0	0	1	1	1	0	0	0
185260	QLAKRFSRG	C	28	36	HLA-B*08:01	>0% - 50%	Dengue virus 3	ELISPOT	IFNg release	Positive	0	0	0	0	0	0	0	0	0	0	0	0	0	0	0	0	0	0	0	0	0	0	0	0	0	0	0	0	0	0	0	0	0	0	0	0	0	0	0	0	0	1	0	0	0	1	1	1	0	0	0
183793	IMKIGIGVLL	E	452	461	HLA-B*08:01	>0% - 50%	Dengue virus 3	ELISPOT	IFNg release	Positive	0	0	0	0	0	0	0	0	0	0	0	0	0	0	0	0	0	0	0	0	0	0	0	0	0	0	0	0	0	0	0	0	0	0	0	0	0	0	0	0	0	1	0	0	0	1	1	1	1	0	1
184412	LKGMSYAMCL	E	156	165	HLA-B*08:01	>0% - 50%	Dengue virus 3	ELISPOT	IFNg release	Positive	0	0	0	0	0	0	0	0	0	0	0	0	0	0	0	0	0	0	0	0	0	0	0	0	0	0	0	0	0	0	0	0	0	0	0	0	0	0	0	0	0	1	0	0	0	1	1	1	1	1	1
186470	WIMKIGIGVL	E	451	460	HLA-B*08:01	>0% - 50%	Dengue virus 3	ELISPOT	IFNg release	Positive	0	0	0	0	0	0	0	0	0	0	0	0	0	0	0	0	0	0	0	0	0	0	0	0	0	0	0	0	0	0	0	0	0	0	0	0	0	0	0	0	0	1	0	0	0	1	1	1	1	0	1
185464	RPINEKEENM	NS1	1109	1118	HLA-B*35:01	>0% - 50%	Dengue virus 3	ELISPOT	IFNg release	Positive	0	0	0	0	0	0	0	0	0	0	0	0	0	0	0	0	0	0	0	0	0	0	0	0	0	0	0	0	0	0	0	0	0	0	0	0	0	0	0	0	0	1	0	0	0	1	1	1	0	0	0
183395	GKKHMIAGVL	NS2a	1153	1162	HLA-B*08:01	>0% - 50%	Dengue virus 3	ELISPOT	IFNg release	Positive	0	0	0	0	0	0	0	0	0	0	0	0	0	0	0	0	0	0	0	0	0	0	0	0	0	0	0	0	0	0	0	0	0	0	0	0	1	1	0	0	0	1	0	0	0	1	0	1	0	0	0
69612	VLLLVTHYA	NS4b	2353	2361	HLA-A2	>0% - 50%	Dengue virus 3	ICS	IFNg release	Positive	0	0	0	0	0	0	0	0	0	0	0	0	0	0	0	0	0	0	0	0	0	0	0	0	0	0	0	0	0	0	0	0	0	0	0	0	0	0	0	0	0	1	0	0	0	1	0	1	0	0	0
180650	MEITAEWLW	NS5	2863	2871	HLA-B*58:01	>0% - 50%	Dengue virus 3	ELISPOT	IFNg release	Positive	1	0	1	0	0	1	0	1	0	0	1	1	0	0	0	0	0	0	0	0	0	1	0	0	0	0	0	0	1	0	1	0	0	1	1	0	0	0	0	0	0	1	0	0	0	1	1	1	0	0	0
180728	RPRLCTREEF	NS5	2879	2888	HLA-B*07:02	>0% - 50%	Dengue virus 3	ELISPOT	IFNg release	Positive	0	0	0	0	0	0	0	0	0	0	0	0	0	0	0	0	0	0	0	1	0	0	0	0	0	0	0	0	0	0	0	0	0	0	0	0	0	0	0	0	0	1	0	0	0	1	1	1	0	0	0
186127	TTWEDVPYL	NS5	3321	3329	HLA-A*02:01	>0% - 50%	Dengue virus 3	ELISPOT	IFNg release	Positive	0	0	0	0	0	0	0	0	0	0	0	0	0	1	0	0	0	0	0	0	1	0	0	0	0	0	0	0	0	0	0	0	1	0	0	1	0	0	0	1	0	1	0	0	1	0	1	0	0	0	0

CD8 DENV-4 Epitopes

Epitope_ID	AA_Seq	Protein_Name	Start	End	Allele	step	Serotype	Method/Technique	Assay Group	Qualitative Measure
180810	VTYECPLLV	PreM	162	170	HLA-A*02:01	0%	Dengue virus 4	ELISPOT	IFNg release	Positive
239411	ALWYVWQVK	NS2b	1463	1471	HLA-A*03:01	0%	Dengue virus 4	ELISPOT	IFNg release	Positive
418991	IPVTMALWY	NS2b	1458	1466	HLA-B*35:01	0%	Dengue virus 4	ELISPOT	IFNg release	Positive
186602	YSDPLALRE	NS3	2077	2085	HLA-A*01:01	0%	Dengue virus 4	ELISPOT	IFNg release	Positive
239768	GTSGSPIINK	NS3	1607	1616	HLA-A*03:01	0%	Dengue virus 4	ELISPOT	IFNg release	Positive
180383	ATGPVLTLW	NS4b	2436	2444	HLA-B*58:01	0%	Dengue virus 4	ELISPOT	IFNg release	Positive
180587	LATGPVLTLW	NS4b	2435	2444	HLA-B*53:01	0%	Dengue virus 4	ELISPOT	IFNg release	Positive
184375	LGKSYAQMW	NS5	3246	3254	HLA-B*57:01	0%	Dengue virus 4	ELISPOT	IFNg release	Positive
419017	MPAMKRYSAP	NS5	3371	3380	HLA-B*35:01	0%	Dengue virus 4	ELISPOT	IFNg release	Positive
419048	SPRLCTREEF	NS5	2877	2886	HLA-B*07:02	0%	Dengue virus 4	ELISPOT	IFNg release	Positive
182971	EMCEDTVTY	PreM	156	164	HLA-B*35:01	100%	Dengue virus 4	ELISPOT	IFNg release	Positive
583701	GVSGGAWVDLVLEHGGCV	E	14	31	HLA class I	100%	Dengue virus 4	ICS	IFNg release	Positive-Low
182901	EETNMITLL	NS2b	1434	1442	HLA-B*40:01	100%	Dengue virus 4	ELISPOT	IFNg release	Positive
156711	LAPTRVVAAEMEEAL	NS3	1695	1709	HLA-B7	100%	Dengue virus 4	51 chromium	cytotoxicity	Positive
180473	GEFRLRGEQR	NS3	1995	2004	HLA-B*40:01	100%	Dengue virus 4	ELISPOT	IFNg release	Positive
180475	GEQRKTFVEL	NS3	2001	2010	HLA-B*40:01	100%	Dengue virus 4	ELISPOT	IFNg release	Positive
180640	MALKDFKEF	NS3	2079	2087	HLA-B*35:01	100%	Dengue virus 4	ELISPOT	IFNg release	Positive
180778	TPEGIIPTLF	NS3	1974	1983	HLA-B*07:02	100%	Dengue virus 4	ELISPOT	IFNg release	Positive
180843	YPKTKLTDWD	NS3	1868	1877	HLA-B*35:01	100%	Dengue virus 4	ELISPOT	IFNg release	Positive
180850	YTPEGIIPTL	NS3	1973	1982	HLA-A*02:06	100%	Dengue virus 4	ELISPOT	IFNg release	Positive
182919	EFRLRGEQR	NS3	1996	2004	HLA-A*33:01	100%	Dengue virus 4	ELISPOT	IFNg release	Positive
183231	FPQSNSPIED	NS3	1799	1808	HLA-B*35:01	100%	Dengue virus 4	ELISPOT	IFNg release	Positive
183338	GEIGAVTLDF	NS3	1595	1604	HLA-B*44:03	100%	Dengue virus 4	ELISPOT	IFNg release	Positive
183427	GLYGNGVVTK	NS3	1622	1631	HLA-A*03:01	100%	Dengue virus 4	ELISPOT	IFNg release	Positive
184079	KLTDWDFVV	NS3	398	406	HLA-A*02:01	100%	Dengue virus 4	ELISPOT	IFNg release	Positive
186347	VPNYNLIVM	NS3	1749	1757	HLA-B*35:01	100%	Dengue virus 4	ELISPOT	IFNg release	Positive
195838	PNYNLIVMDEAHFTD	NS3	1750	1764	HLA class I	100%	Dengue virus 4	ICS	IFNg release	Positive
22822	GTSGSPIINRK	NS3	133	143	HLA-A11	100%	Dengue virus 4	51 chromium	cytotoxicity	Positive
34918	LAPTRVVAAEME	NS3	221	232	HLA-B7	100%	Dengue virus 4	51 chromium	cytotoxicity	Positive
540029	VEMGEAAAIFMTATPPG	NS3	1778	1794	HLA class I	100%	Dengue virus 4	ICS	IFNg release	Positive
581940	APTRVVAAEMEEALRGL	NS3	1558	1574	HLA class I	100%	Dengue virus 4	ICS	IFNg release	Positive-Low
62616	SYKDREWCF	NS3	389	397	HLA-A24	100%	Dengue virus 4	ELISPOT	IFNg release	Positive
65552	TPEGIIPTL	NS3	500	508	HLA-B*35:01	100%	Dengue virus 4	51 chromium	cytotoxicity	Positive
180596	LETLMLVALL	NS4a	2144	2153	HLA-B*40:01	100%	Dengue virus 4	ELISPOT	IFNg release	Positive
182926	EIASLPTYL	NS4a	2101	2109	HLA-A*68:02	100%	Dengue virus 4	ELISPOT	IFNg release	Positive
183650	IAVASGLLW	NS4a	2180	2188	HLA-B*57:01	100%	Dengue virus 4	ELISPOT	IFNg release	Positive
184531	LPESLETLML	NS4a	2140	2149	HLA-B*35:01	100%	Dengue virus 4	ELISPOT	IFNg release	Positive
185881	TEIASLPTYL	NS4a	2100	2109	HLA-B*40:01	100%	Dengue virus 4	ELISPOT	IFNg release	Positive
42083	MLVALLGAM	NS4a	56	64	HLA-A2	100%	Dengue virus 4	ICS	IFNg release	Positive
136826	CYSQVNPTTL	NS4b	2337	2346	HLA-A*24:02	100%	Dengue virus 4	ELISPOT	IFNg release	Positive
150647	TPMLRHTIEN	NS4b	2288	2297	HLA-B*07:02	100%	Dengue virus 4	ELISPOT	IFNg release	Positive
180764	TETTILDVDL	NS4b	2262	2271	HLA-B*40:01	100%	Dengue virus 4	ELISPOT	IFNg release	Positive
180803	VPLLAMGCY	NS4b	2330	2338	HLA-B*35:01	100%	Dengue virus 4	ELISPOT	IFNg release	Positive
183672	IENTSANLSL	NS4b	2295	2304	HLA-B*40:01	100%	Dengue virus 4	ELISPOT	IFNg release	Positive
184453	LLLMRTTWAF	NS4b	2420	2429	HLA-A*24:02	100%	Dengue virus 4	ELISPOT	IFNg release	Positive
184713	LYAVATTIL	NS4b	2279	2287	HLA-A*24:02	100%	Dengue virus 4	ELISPOT	IFNg release	Positive
186330	VMLLVHYAI	NS4b	2351	2359	HLA-A*24:02	100%	Dengue virus 4	ELISPOT	IFNg release	Positive
37535	LLLMRTTWA	NS4b	76	84	HLA-A2	100%	Dengue virus 4	ICS	TNF release	Positive
40472	LVMLLVHYA	NS4b	6	14	HLA-A2	100%	Dengue virus 4	ICS	IFNg release	Positive
150548	RSNAAIGAVF	NS5	2891	2900	HLA-B*15:01	100%	Dengue virus 4	ELISPOT	IFNg release	Positive
180684	NPRLCTREEF	NS5	2877	2886	HLA-B*07:02	100%	Dengue virus 4	ELISPOT	IFNg release	Positive
180718	REDLWCGSL	NS5	3330	3338	HLA-B*40:01	100%	Dengue virus 4	ELISPOT	IFNg release	Positive
183353	GESSSNPTI	NS5	2635	2643	HLA-B*44:02	100%	Dengue virus 4	ELISPOT	IFNg release	Positive
184051	KLGEFGRAK	NS5	2948	2956	HLA-A*03:01	100%	Dengue virus 4	ELISPOT	IFNg release	Positive
184130	KREDLWCGSL	NS5	3329	3338	HLA-B*08:01	100%	Dengue virus 4	ELISPOT	IFNg release	Positive
185232	QEEHKETWHY	NS5	2772	2781	HLA-B*44:02	100%	Dengue virus 4	ELISPOT	IFNg release	Positive
186453	WEPSKGWKNW	NS5	3182	3191	HLA-B*44:02	100%	Dengue virus 4	ELISPOT	IFNg release	Positive
539656	MAISGDDCVVKPLDERF	NS5	3146	3162	HLA class I	100%	Dengue virus 4	ICS	IFNg release	Positive
539703	MVTQLAMTDTTPFGQQR	NS5	2824	2840	HLA class I	100%	Dengue virus 4	ICS	IFNg release	Positive
180541	ITLLCLIPTV	C	102	111	HLA-A*02:01	81% - <100%	Dengue virus 4	ELISPOT	IFNg release	Positive
182707	CNLTSTWVMY	PreM	181	190	HLA-A*30:02	81% - <100%	Dengue virus 4	ELISPOT	IFNg release	Positive
186303	VLMMLVAPSY	PreM	17	26	HLA-B*15:01	81% - <100%	Dengue virus 4	ELISPOT	IFNg release	Positive
185147	NYKERMVTF	E	511	519	HLA-A*24:02	81% - <100%	Dengue virus 4	ELISPOT	IFNg release	Positive
185817	SWMIRILIGF	E	161	170	HLA-A*24:02	81% - <100%	Dengue virus 4	ELISPOT	IFNg release	Positive
240130	PPASDLKYSW	NS1	880	889	HLA-B*53:01	81% - <100%	Dengue virus 4	ELISPOT	IFNg release	Positive
167725	AMTTTLSIPHDLMELIDGIS	NS2a	50	69	HLA class I	81% - <100%	Dengue virus 4	ELISPOT	IFNg release	Positive
182562	ALPVYLMTL	NS2a	1329	1337	HLA-A*02:01	81% - <100%	Dengue virus 4	ELISPOT	IFNg release	Positive
183818	IPHDLMELI	NS2a	1241	1249	HLA-B*53:01	81% - <100%	Dengue virus 4	ELISPOT	IFNg release	Positive
184390	LIDGISLGL	NS2a	1248	1256	HLA-A*01:01	81% - <100%	Dengue virus 4	ELISPOT	IFNg release	Positive
184566	LPVYLMTLMK	NS2a	1330	1339	HLA-B*35:01	81% - <100%	Dengue virus 4	ELISPOT	IFNg release	Positive
180648	MEGVFHTMW	NS3	1516	1524	HLA-B*44:03	81% - <100%	Dengue virus 4	ELISPOT	IFNg release	Positive
182563	ALSEGVYRI	NS3	1491	1499	HLA-A*02:01	81% - <100%	Dengue virus 4	ELISPOT	IFNg release	Positive
183407	GLFGKTQVGV	NS3	1503	1512	HLA-A*02:01	81% - <100%	Dengue virus 4	ELISPOT	IFNg release	Positive
186008	TPPGATDPF	NS3	1791	1799	HLA-B*35:01	81% - <100%	Dengue virus 4	ELISPOT	IFNg release	Positive
539058	AHFTDPSSVAARGYIST	NS3	1760	1776	HLA class I	81% - <100%	Dengue virus 4	ICS	IFNg release	Positive
539576	LIVMDEAHFTDPSSVAA	NS3	1754	1770	HLA class I	81% - <100%	Dengue virus 4	ICS	IFNg release	Positive
186620	YVILTILTI	NS4a	2228	2236	HLA-A*24:02	81% - <100%	Dengue virus 4	ELISPOT	IFNg release	Positive
180379	ATGPILTLW	NS4b	2436	2444	HLA-B*58:01	81% - <100%	Dengue virus 4	ELISPOT	IFNg release	Positive
1067215	HSWEDIPYL	NS5	3319	3327	HLA-A*02:01	81% - <100%	Dengue virus 4	ELISPOT	IFNg release	Positive
184954	MTTEDMLKVW	NS5	3292	3301	HLA class I	81% - <100%	Dengue virus 4	ICS	IFNg release	Positive
186550	YDQENPYRTW	NS5	2781	2790	HLA-B*44:02	81% - <100%	Dengue virus 4	ELISPOT	IFNg release	Positive
186640	YYMATLKNV	NS5	2576	2584	HLA-A*24:02	81% - <100%	Dengue virus 4	ELISPOT	IFNg release	Positive
186641	YYMATLKNVT	NS5	2576	2585	HLA-A*24:02	81% - <100%	Dengue virus 4	ELISPOT	IFNg release	Positive
183667	IEKASLIEV	NS1	986	994	HLA-B*40:01	>0% - 50%	Dengue virus 4	ELISPOT	IFNg release	Positive
185050	NFLEVEDYGF	NS1	925	934	HLA-A*24:02	>0% - 50%	Dengue virus 4	ELISPOT	IFNg release	Positive
184591	LSIPHDLMEF	NS2a	1239	1248	HLA-A*01:01	>0% - 50%	Dengue virus 4	ELISPOT	IFNg release	Positive
186584	YPLAIPVTM	NS2b	1454	1462	HLA-B*53:01	>0% - 50%	Dengue virus 4	ELISPOT	IFNg release	Positive
182508	AIAVASGLLW	NS4a	2179	2188	HLA-B*44:02	>0% - 50%	Dengue virus 4	ELISPOT	IFNg release	Positive
183570	HEMYWVSGV	NS5	2703	2711	HLA-B*44:02	>0% - 50%	Dengue virus 4	ELISPOT	IFNg release	Positive
539149	DCVVKPLDERFSTSLLF	NS5	3152	3168	HLA class I	>0% - 50%	Dengue virus 4	ICS	IFNg release	Positive

NCBI IDs of DENV-4 circulating strains from Brazil, (100% identity=1,green ; else 0, red)

Epitope_ID	AEW50183.1	AFX65867.1	AFX65875.1	AFX65870.1	AFX65878.1	AFX65880.1	AFX65879.1	AKQ00033.1	AKQ00035.1	AIQ84228.1	AIQ84240.1	AIQ84244.1	AIQ84242.1	AIQ84233.1	AIQ84223.1	AFX65872.1	AMP43484.1	AKQ00031.1
180810	0	0	0	0	0	0	0	0	0	0	0	0	0	0	0	0	0	0
239411	0	0	0	0	0	0	0	0	0	0	0	0	0	0	0	0	0	0
418991	0	0	0	0	0	0	0	0	0	0	0	0	0	0	0	0	0	0
186602	0	0	0	0	0	0	0	0	0	0	0	0	0	0	0	0	0	0
239768	0	0	0	0	0	0	0	0	0	0	0	0	0	0	0	0	0	0
180383	0	0	0	0	0	0	0	0	0	0	0	0	0	0	0	0	0	0
180587	0	0	0	0	0	0	0	0	0	0	0	0	0	0	0	0	0	0
184375	0	0	0	0	0	0	0	0	0	0	0	0	0	0	0	0	0	0
419017	0	0	0	0	0	0	0	0	0	0	0	0	0	0	0	0	0	0
419048	0	0	0	0	0	0	0	0	0	0	0	0	0	0	0	0	0	0
182971	1	1	1	1	1	1	1	1	1	1	1	1	1	1	1	1	1	1
583701	1	1	1	1	1	1	1	1	1	1	1	1	1	1	1	1	1	1
182901	1	1	1	1	1	1	1	1	1	1	1	1	1	1	1	1	1	1
156711	1	1	1	1	1	1	1	1	1	1	1	1	1	1	1	1	1	1
180473	1	1	1	1	1	1	1	1	1	1	1	1	1	1	1	1	1	1
180475	1	1	1	1	1	1	1	1	1	1	1	1	1	1	1	1	1	1
180640	1	1	1	1	1	1	1	1	1	1	1	1	1	1	1	1	1	1
180778	1	1	1	1	1	1	1	1	1	1	1	1	1	1	1	1	1	1
180843	1	1	1	1	1	1	1	1	1	1	1	1	1	1	1	1	1	1
180850	1	1	1	1	1	1	1	1	1	1	1	1	1	1	1	1	1	1
182919	1	1	1	1	1	1	1	1	1	1	1	1	1	1	1	1	1	1
183231	1	1	1	1	1	1	1	1	1	1	1	1	1	1	1	1	1	1
183338	1	1	1	1	1	1	1	1	1	1	1	1	1	1	1	1	1	1
183427	1	1	1	1	1	1	1	1	1	1	1	1	1	1	1	1	1	1
184079	1	1	1	1	1	1	1	1	1	1	1	1	1	1	1	1	1	1
186347	1	1	1	1	1	1	1	1	1	1	1	1	1	1	1	1	1	1
195838	1	1	1	1	1	1	1	1	1	1	1	1	1	1	1	1	1	1
22822	1	1	1	1	1	1	1	1	1	1	1	1	1	1	1	1	1	1
34918	1	1	1	1	1	1	1	1	1	1	1	1	1	1	1	1	1	1
540029	1	1	1	1	1	1	1	1	1	1	1	1	1	1	1	1	1	1
581940	1	1	1	1	1	1	1	1	1	1	1	1	1	1	1	1	1	1
62616	1	1	1	1	1	1	1	1	1	1	1	1	1	1	1	1	1	1
65552	1	1	1	1	1	1	1	1	1	1	1	1	1	1	1	1	1	1
180596	1	1	1	1	1	1	1	1	1	1	1	1	1	1	1	1	1	1
182926	1	1	1	1	1	1	1	1	1	1	1	1	1	1	1	1	1	1
183650	1	1	1	1	1	1	1	1	1	1	1	1	1	1	1	1	1	1
184531	1	1	1	1	1	1	1	1	1	1	1	1	1	1	1	1	1	1
185881	1	1	1	1	1	1	1	1	1	1	1	1	1	1	1	1	1	1
42083	1	1	1	1	1	1	1	1	1	1	1	1	1	1	1	1	1	1
136826	1	1	1	1	1	1	1	1	1	1	1	1	1	1	1	1	1	1
150647	1	1	1	1	1	1	1	1	1	1	1	1	1	1	1	1	1	1
180764	1	1	1	1	1	1	1	1	1	1	1	1	1	1	1	1	1	1
180803	1	1	1	1	1	1	1	1	1	1	1	1	1	1	1	1	1	1
183672	1	1	1	1	1	1	1	1	1	1	1	1	1	1	1	1	1	1
184453	1	1	1	1	1	1	1	1	1	1	1	1	1	1	1	1	1	1
184713	1	1	1	1	1	1	1	1	1	1	1	1	1	1	1	1	1	1
186330	1	1	1	1	1	1	1	1	1	1	1	1	1	1	1	1	1	1
37535	1	1	1	1	1	1	1	1	1	1	1	1	1	1	1	1	1	1
40472	1	1	1	1	1	1	1	1	1	1	1	1	1	1	1	1	1	1
150548	1	1	1	1	1	1	1	1	1	1	1	1	1	1	1	1	1	1
180684	1	1	1	1	1	1	1	1	1	1	1	1	1	1	1	1	1	1
180718	1	1	1	1	1	1	1	1	1	1	1	1	1	1	1	1	1	1
183353	1	1	1	1	1	1	1	1	1	1	1	1	1	1	1	1	1	1
184051	1	1	1	1	1	1	1	1	1	1	1	1	1	1	1	1	1	1
184130	1	1	1	1	1	1	1	1	1	1	1	1	1	1	1	1	1	1
185232	1	1	1	1	1	1	1	1	1	1	1	1	1	1	1	1	1	1
186453	1	1	1	1	1	1	1	1	1	1	1	1	1	1	1	1	1	1
539656	1	1	1	1	1	1	1	1	1	1	1	1	1	1	1	1	1	1
539703	1	1	1	1	1	1	1	1	1	1	1	1	1	1	1	1	1	1
180541	1	1	1	1	1	1	1	1	1	1	1	1	1	1	1	1	1	1
182707	1	1	1	1	1	1	1	1	1	1	1	1	1	1	1	1	1	1
186303	1	1	1	1	1	1	1	1	1	1	1	1	1	1	1	1	1	1
185147	1	1	0	1	0	1	0	1	1	1	1	0	1	1	1	1	1	1
185817	1	1	1	0	1	1	1	1	1	1	1	1	1	1	0	0	1	1
240130	1	1	1	0	1	1	1	1	1	1	1	1	1	1	0	0	1	1
167725	1	1	1	1	1	1	1	1	1	1	1	1	1	1	1	1	1	1
182562	1	1	1	1	1	1	1	1	1	1	1	1	1	1	1	1	1	1
183818	1	1	1	1	1	1	1	1	1	1	1	1	1	1	1	1	1	1
184390	1	1	1	1	1	1	1	1	1	1	1	1	1	1	1	1	1	1
184566	1	1	1	1	1	1	1	1	1	1	1	1	1	1	1	1	1	1
180648	1	1	1	1	1	1	1	1	1	1	1	1	0	1	1	1	1	1
182563	1	1	1	1	1	1	1	1	1	1	1	1	1	1	1	1	1	1
183407	1	1	1	1	1	1	1	1	1	1	1	1	1	0	1	1	1	1
186008	1	1	1	1	1	1	1	1	1	1	1	1	1	1	1	1	1	1
539058	1	1	1	1	1	1	1	1	1	1	1	1	1	1	1	1	1	1
539576	1	1	1	1	1	1	1	1	1	1	1	1	1	1	1	1	1	1
186620	1	1	1	0	1	1	1	1	1	1	1	1	1	1	1	0	1	1
180379	1	1	1	1	1	1	1	1	0	1	1	1	0	1	1	1	1	1
1067215	1	1	1	1	1	1	1	1	1	1	1	1	1	1	1	1	0	1
184954	1	1	1	1	1	1	1	1	1	1	1	1	1	1	1	1	1	1
186550	1	1	1	1	1	1	1	1	1	1	1	1	1	1	1	1	1	1
186640	1	1	1	1	1	1	1	1	1	1	1	1	1	1	1	1	1	1
186641	1	1	1	1	1	1	1	1	1	1	1	1	1	1	1	1	1	1
183667	0	0	0	1	0	0	0	0	0	0	0	0	0	0	0	1	0	0
185050	0	0	0	0	0	0	0	0	0	0	0	0	0	0	0	0	0	0
184591	0	0	0	0	0	0	0	0	0	0	0	0	0	0	0	0	0	0
186584	0	0	0	1	0	0	0	0	0	0	0	0	0	0	0	1	0	0
182508	0	0	0	0	0	0	0	0	0	0	0	0	0	0	0	0	0	0
183570	0	0	0	0	0	0	0	0	0	0	0	0	0	0	0	0	0	0
539149	0	0	0	0	0	0	0	0	0	0	0	0	0	0	0	0	0	0

	NCBI IDs of DENV-4 circulating strains from Brazil, (100% identity=1,green ; else 0, red)																		DENV-4 constructs of vaccines					
Epitope_ID	AIQ84245.1	AIQ84235.1	AIQ84238.1	AIQ84220.1	AIQ84226.1	AEX91754.1	AFX65881.1	AKQ00036.1	AKQ00030.1	ANK35834.1	AIQ84231.1	AIQ84221.1	AIQ84236.1	AFX65874.1	AKQ00032.1	AKQ00034.1	AKQ00028.1	AIQ84222.1	TV003	TDEN	DPIV	Dengvaxia	DENVax	TVDV
180810	0	0	0	0	0	0	0	0	0	0	0	0	0	0	0	0	0	0	0	0	0	0	0	1
239411	0	0	0	0	0	0	0	0	0	0	0	0	0	0	0	0	0	0	0	0	0	0	0	0
418991	0	0	0	0	0	0	0	0	0	0	0	0	0	0	0	0	0	0	0	0	0	0	0	0
186602	0	0	0	0	0	0	0	0	0	0	0	0	0	0	0	0	0	0	0	0	0	0	0	0
239768	0	0	0	0	0	0	0	0	0	0	0	0	0	0	0	0	0	0	0	0	0	0	0	0
180383	0	0	0	0	0	0	0	0	0	0	0	0	0	0	0	0	0	0	0	0	0	0	0	0
180587	0	0	0	0	0	0	0	0	0	0	0	0	0	0	0	0	0	0	0	0	0	0	0	0
184375	0	0	0	0	0	0	0	0	0	0	0	0	0	0	0	0	0	0	0	0	0	0	0	0
419017	0	0	0	0	0	0	0	0	0	0	0	0	0	0	0	0	0	0	0	0	0	0	0	0
419048	0	0	0	0	0	0	0	0	0	0	0	0	0	0	0	0	0	0	0	0	0	0	0	0
182971	1	1	1	1	1	1	1	1	1	1	1	1	1	1	1	1	1	1	1	1	1	0	1	1
583701	1	1	1	1	1	1	1	1	1	1	1	1	1	1	1	1	1	1	1	1	1	1	1	1
182901	1	1	1	1	1	1	1	1	1	1	1	1	1	1	1	1	1	1	1	1	1	0	0	0
156711	1	1	1	1	1	1	1	1	1	1	1	1	1	1	1	1	1	1	1	1	1	0	0	0
180473	1	1	1	1	1	1	1	1	1	1	1	1	1	1	1	1	1	1	1	1	1	0	0	0
180475	1	1	1	1	1	1	1	1	1	1	1	1	1	1	1	1	1	1	1	1	1	0	0	0
180640	1	1	1	1	1	1	1	1	1	1	1	1	1	1	1	1	1	1	1	1	1	0	0	0
180778	1	1	1	1	1	1	1	1	1	1	1	1	1	1	1	1	1	1	1	1	1	0	0	0
180843	1	1	1	1	1	1	1	1	1	1	1	1	1	1	1	1	1	1	1	1	1	0	0	0
180850	1	1	1	1	1	1	1	1	1	1	1	1	1	1	1	1	1	1	1	1	1	0	0	0
182919	1	1	1	1	1	1	1	1	1	1	1	1	1	1	1	1	1	1	1	1	1	0	0	0
183231	1	1	1	1	1	1	1	1	1	1	1	1	1	1	1	1	1	1	1	1	1	0	0	0
183338	1	1	1	1	1	1	1	1	1	1	1	1	1	1	1	1	1	1	1	1	1	0	0	0
183427	1	1	1	1	1	1	1	1	1	1	1	1	1	1	1	1	1	1	1	1	1	0	0	0
184079	1	1	1	1	1	1	1	1	1	1	1	1	1	1	1	1	1	1	1	1	1	0	0	0
186347	1	1	1	1	1	1	1	1	1	1	1	1	1	1	1	1	1	1	1	1	1	0	0	0
195838	1	1	1	1	1	1	1	1	1	1	1	1	1	1	1	1	1	1	1	1	1	0	0	0
22822	1	1	1	1	1	1	1	1	1	1	1	1	1	1	1	1	1	1	1	1	1	0	0	0
34918	1	1	1	1	1	1	1	1	1	1	1	1	1	1	1	1	1	1	1	1	1	0	0	0
540029	1	1	1	1	1	1	1	1	1	1	1	1	1	1	1	1	1	1	1	1	1	0	0	0
581940	1	1	1	1	1	1	1	1	1	1	1	1	1	1	1	1	1	1	1	1	1	0	0	0
62616	1	1	1	1	1	1	1	1	1	1	1	1	1	1	1	1	1	1	1	1	1	0	0	0
65552	1	1	1	1	1	1	1	1	1	1	1	1	1	1	1	1	1	1	1	1	1	0	0	0
180596	1	1	1	1	1	1	1	1	1	1	1	1	1	1	1	1	1	1	1	1	1	0	0	0
182926	1	1	1	1	1	1	1	1	1	1	1	1	1	1	1	1	1	1	1	1	1	0	0	0
183650	1	1	1	1	1	1	1	1	1	1	1	1	1	1	1	1	1	1	1	1	1	0	0	0
184531	1	1	1	1	1	1	1	1	1	1	1	1	1	1	1	1	1	1	1	1	1	0	0	0
185881	1	1	1	1	1	1	1	1	1	1	1	1	1	1	1	1	1	1	1	1	1	0	0	0
42083	1	1	1	1	1	1	1	1	1	1	1	1	1	1	1	1	1	1	1	1	1	0	0	0
136826	1	1	1	1	1	1	1	1	1	1	1	1	1	1	1	1	1	1	1	1	1	0	0	0
150647	1	1	1	1	1	1	1	1	1	1	1	1	1	1	1	1	1	1	1	1	1	0	0	0
180764	1	1	1	1	1	1	1	1	1	1	1	1	1	1	1	1	1	1	1	1	1	0	0	0
180803	1	1	1	1	1	1	1	1	1	1	1	1	1	1	1	1	1	1	1	1	1	0	0	0
183672	1	1	1	1	1	1	1	1	1	1	1	1	1	1	1	1	1	1	1	1	1	0	0	0
184453	1	1	1	1	1	1	1	1	1	1	1	1	1	1	1	1	1	1	1	1	1	0	0	0
184713	1	1	1	1	1	1	1	1	1	1	1	1	1	1	1	1	1	1	1	1	1	0	0	0
186330	1	1	1	1	1	1	1	1	1	1	1	1	1	1	1	1	1	1	1	0	1	0	0	0
37535	1	1	1	1	1	1	1	1	1	1	1	1	1	1	1	1	1	1	1	1	1	0	0	0
40472	1	1	1	1	1	1	1	1	1	1	1	1	1	1	1	1	1	1	1	0	1	0	0	0
150548	1	1	1	1	1	1	1	1	1	1	1	1	1	1	1	1	1	1	1	1	1	0	0	0
180684	1	1	1	1	1	1	1	1	1	1	1	1	1	1	1	1	1	1	1	1	1	0	0	0
180718	1	1	1	1	1	1	1	1	1	1	1	1	1	1	1	1	1	1	1	1	1	0	0	0
183353	1	1	1	1	1	1	1	1	1	1	1	1	1	1	1	1	1	1	1	1	1	0	0	0
184051	1	1	1	1	1	1	1	1	1	1	1	1	1	1	1	1	1	1	1	1	1	0	0	0
184130	1	1	1	1	1	1	1	1	1	1	1	1	1	1	1	1	1	1	1	1	1	0	0	0
185232	1	1	1	1	1	1	1	1	1	1	1	1	1	1	1	1	1	1	1	1	1	0	0	0
186453	1	1	1	1	1	1	1	1	1	1	1	1	1	1	1	1	1	1	1	1	1	0	0	0
539656	1	1	1	1	1	1	1	1	1	1	1	1	1	1	1	1	1	1	1	1	1	0	0	0
539703	1	1	1	1	1	1	1	1	1	1	1	1	1	1	1	1	1	1	1	1	1	0	0	0
180541	1	1	1	1	1	1	1	0	1	1	1	1	1	1	1	1	1	1	1	1	1	0	0	0
182707	1	1	1	1	1	1	0	1	1	1	1	1	1	1	1	1	1	1	1	1	1	0	1	0
186303	1	1	1	1	1	1	0	1	1	1	1	1	1	1	1	1	1	1	1	1	1	0	1	1
185147	1	1	1	1	1	1	0	1	1	1	1	1	1	1	1	1	1	1	1	1	1	1	1	1
185817	1	1	1	1	1	1	0	1	1	1	1	1	1	1	1	1	1	1	1	1	1	0	1	0
240130	1	1	0	1	1	1	0	1	1	1	1	1	1	1	1	1	1	1	0	0	0	0	0	0
167725	1	1	1	1	1	1	0	1	1	1	1	1	1	1	1	1	1	1	0	0	0	0	0	0
182562	1	1	1	1	1	1	1	0	1	1	1	1	1	1	1	1	1	1	1	1	1	0	0	0
183818	1	1	1	1	1	1	0	1	1	1	1	1	1	1	1	1	1	1	1	1	1	0	0	0
184390	1	1	1	1	1	1	0	1	1	1	1	1	1	1	1	1	1	1	1	1	1	0	0	0
184566	1	1	1	1	1	1	1	0	1	1	1	1	1	1	1	1	1	1	1	1	1	0	0	0
180648	1	1	1	1	1	1	1	1	1	1	1	1	1	1	1	1	1	1	1	1	1	0	0	0
182563	1	1	1	1	1	1	0	1	1	1	1	1	1	1	1	1	1	1	1	1	1	0	0	0
183407	1	1	1	1	1	1	1	1	1	1	1	1	1	1	1	1	1	1	1	1	1	0	0	0
186008	1	1	1	1	1	1	0	1	1	1	1	1	1	1	1	1	1	1	1	1	1	0	0	0
539058	1	1	1	1	1	1	1	1	1	1	0	1	1	1	1	1	1	1	1	1	1	0	0	0
539576	1	1	1	1	1	1	1	1	1	1	0	1	1	1	1	1	1	1	1	1	1	0	0	0
186620	1	1	1	1	1	1	1	1	1	1	1	1	1	1	1	1	1	1	1	1	1	0	0	0
180379	1	1	1	1	1	1	1	1	1	1	1	1	1	1	1	1	1	1	1	1	1	0	0	0
1067215	1	1	1	1	1	1	1	1	1	1	1	1	1	1	1	1	1	1	1	1	1	0	0	0
184954	1	1	0	1	1	1	1	1	1	1	1	1	1	1	1	1	1	1	1	1	1	0	0	0
186550	1	1	1	1	1	1	0	1	1	1	1	1	1	1	1	1	1	1	1	1	1	0	0	0
186640	1	1	1	1	1	1	1	0	1	1	1	1	1	1	1	1	1	1	1	1	1	0	0	0
186641	1	1	1	1	1	1	1	0	1	1	1	1	1	1	1	1	1	1	1	1	1	0	0	0
183667	0	0	0	0	0	0	1	0	0	0	0	0	0	0	0	0	0	0	1	1	1	0	0	0
185050	0	0	0	0	0	0	1	0	0	0	0	0	0	0	0	0	0	0	0	0	0	0	0	0
184591	0	0	0	0	0	0	1	0	0	0	0	0	0	0	0	0	0	0	0	0	0	0	0	0
186584	0	1	0	0	0	0	1	0	0	0	0	0	0	0	0	0	0	0	1	1	1	0	0	0
182508	0	0	0	0	0	0	1	0	0	0	0	0	0	0	0	0	0	0	0	0	0	0	0	0
183570	0	0	0	0	0	0	1	0	0	0	0	0	0	0	0	0	0	0	0	0	0	0	0	0
539149	0	0	0	0	0	0	1	0	0	0	0	0	0	0	0	0	0	0	0	0	0	0	0	0

Supplementary Table S8. Alignment of CD4 and CD8 epitopes in circulating dengue viruses from Mexico and six vaccine candidates. *Detailed information, for Mexico, on each of the vaccine carried CD4 and CD8 T cell epitopes that are not present in any of the viral isolates, present in all of the viral isolates, present in only 81% – <100% viral isolates, present in only 51% – 80% viral isolates, and present in only >0% – 50% of viral isolates for each of the epitope within each of the six vaccine candidates and for all the four dengue serotypes. The first table summarizes information retrieved on circulating viral isolate sequences available from Mexico. Next, the globally reported CD4/CD8 T cell epitopes are organized into: DENV-1 specific CD4 epitopes, DENV-2 specific CD4 epitopes, DENV-3 specific CD4 epitopes, DENV-1 specific CD8 epitopes, DENV-2 specific CD8 epitopes, DENV-3 specific CD8 epitopes. There are no DENV-4 sequences reported from Mexico. Also, the data for DENV-3 is not included in the analysis because of only 2 sequences, hence uninterpretable. In each of these tables, identity of the epitope to each of the corresponding viral isolate serotype and each of the vaccine constructs are indicated. Green color shade and number 1 indicates 100% identity in the given virus isolate/vaccine construct. Pink color shade and number zero indicates not identical in the given virus isolate/vaccine construct.*

NCBI ID	Strain Name	Host	Date	Continent	Country	Prot_Name	VirusType
ACW82947.1	DENV-1/MX/BID-V3696/2007	Human	2007-01-01	North America	Mexico	polyprotein	Dengue virus 1
ACW82962.1	DENV-1/MX/BID-V3728/2007	Human	2007-01-01	North America	Mexico	polyprotein	Dengue virus 1
ACW82963.1	DENV-1/MX/BID-V3730/2007	Human	2007-01-01	North America	Mexico	polyprotein	Dengue virus 1
ACW82941.1	DENV-1/MX/BID-V3682/2007	Human	2007-01-01	North America	Mexico	polyprotein	Dengue virus 1
ACW82975.1	DENV-1/MX/BID-V3754/2008	Human	2008-01-01	North America	Mexico	polyprotein	Dengue virus 1
ACW82944.1	DENV-1/MX/BID-V3687/2007	Human	2007-01-01	North America	Mexico	polyprotein	Dengue virus 1
ACW82940.1	DENV-1/MX/BID-V3665/2007	Human	2007-01-01	North America	Mexico	polyprotein	Dengue virus 1
ACW82978.1	DENV-1/MX/BID-V3759/2008	Mosquito	2008-01-01	North America	Mexico	polyprotein	Dengue virus 1
ACW82946.1	DENV-1/MX/BID-V3694/2007	Human	2007-01-01	North America	Mexico	polyprotein	Dengue virus 1
ACW82974.1	DENV-1/MX/BID-V3753/2008	Human	2008-01-01	North America	Mexico	polyprotein	Dengue virus 1
ACW82960.1	DENV-1/MX/BID-V3726/2007	Human	2007-01-01	North America	Mexico	polyprotein	Dengue virus 1
ACW82971.1	DENV-1/MX/BID-V3747/2008	Human	2008-01-01	North America	Mexico	polyprotein	Dengue virus 1
ACW82950.1	DENV-1/MX/BID-V3699/2007	Human	2007-01-01	North America	Mexico	polyprotein	Dengue virus 1
ACW82964.1	DENV-1/MX/BID-V3731/2007	Human	2007-01-01	North America	Mexico	polyprotein	Dengue virus 1
ACW82979.1	DENV-1/MX/BID-V3760/2008	Mosquito	2008-01-01	North America	Mexico	polyprotein	Dengue virus 1
ACW82977.1	DENV-1/MX/BID-V3758/2008	Mosquito	2008-01-01	North America	Mexico	polyprotein	Dengue virus 1
ACW82943.1	DENV-1/MX/BID-V3686/2007	Human	2007-01-01	North America	Mexico	polyprotein	Dengue virus 1
ACW82945.1	DENV-1/MX/BID-V3689/2007	Human	2007-01-01	North America	Mexico	polyprotein	Dengue virus 1
ACW82976.1	DENV-1/MX/BID-V3756/2008	Mosquito	2008-01-01	North America	Mexico	polyprotein	Dengue virus 1
ACW82951.1	DENV-1/MX/BID-V3700/2007	Human	2007-01-01	North America	Mexico	polyprotein	Dengue virus 1
ACW82970.1	DENV-1/MX/BID-V3746/2008	Human	2008-01-01	North America	Mexico	polyprotein	Dengue virus 1
ADA60779.1	DENV-1/MX/BID-V3690/2007	Human	2007-01-01	North America	Mexico	polyprotein	Dengue virus 1
ADA60794.1	DENV-1/MX/BID-V3748/2008	Human	2008-01-01	North America	Mexico	polyprotein	Dengue virus 1
ADA60781.1	DENV-1/MX/BID-V3703/2007	Human	2007-01-01	North America	Mexico	polyprotein	Dengue virus 1
ADA60782.1	DENV-1/MX/BID-V3707/2007	Human	2007-01-01	North America	Mexico	polyprotein	Dengue virus 1
ADA60787.1	DENV-1/MX/BID-V3722/2007	Human	2007-01-01	North America	Mexico	polyprotein	Dengue virus 1
ADA60773.1	DENV-1/MX/BID-V3669/2007	Human	2007-01-01	North America	Mexico	polyprotein	Dengue virus 1
ADA60795.1	DENV-1/MX/BID-V3761/2008	Mosquito	2008-01-01	North America	Mexico	polyprotein	Dengue virus 1
ADA60778.1	DENV-1/MX/BID-V3688/2007	Human	2007-01-01	North America	Mexico	polyprotein	Dengue virus 1
ADA60772.1	DENV-1/MX/BID-V3668/2007	Human	2007-01-01	North America	Mexico	polyprotein	Dengue virus 1
ADO97104.1	DENV-1/MX/BID-V3733/2007	Human	2007-01-01	North America	Mexico	polyprotein	Dengue virus 1
AHI43703.1	DENV-1/MX/BID-V7586/2007	Human	2007-01-01	North America	Mexico	polyprotein	Dengue virus 1
AHI43728.1	DENV-1/MX/BID-V7614/2009	Human	2009-01-01	North America	Mexico	polyprotein	Dengue virus 1

AHG23208.1	DENV-1/MX/BID-V3676/2007	Human	2007-01-01	North America	Mexico	polyprotein	Dengue virus 1
AHI43700.1	DENV-1/MX/BID-V7572/2007	Human	2007-01-01	North America	Mexico	polyprotein	Dengue virus 1
AHI43723.1	DENV-1/MX/BID-V7609/2008	Human	2008-01-01	North America	Mexico	polyprotein	Dengue virus 1
AHI43730.1	DENV-1/MX/BID-V7619/2009	Human	2009-01-01	North America	Mexico	polyprotein	Dengue virus 1
AHI43727.1	DENV-1/MX/BID-V7613/2009	Human	2009-01-01	North America	Mexico	polyprotein	Dengue virus 1
AHI43731.1	DENV-1/MX/BID-V7624/2011	Human	2011-01-01	North America	Mexico	polyprotein	Dengue virus 1
AHI43729.1	DENV-1/MX/BID-V7615/2009	Human	2009-01-01	North America	Mexico	polyprotein	Dengue virus 1
AHI43720.1	DENV-1/MX/BID-V7606/2008	Human	2008-01-01	North America	Mexico	polyprotein	Dengue virus 1
AHI43710.1	DENV-1/MX/BID-V7593/2007	Human	2007-01-01	North America	Mexico	polyprotein	Dengue virus 1
AHI43712.1	DENV-1/MX/BID-V7595/2007	Human	2007-01-01	North America	Mexico	polyprotein	Dengue virus 1
AHI43751.1	DENV-1/MX/BID-V8195/2012	Human	2012-01-01	North America	Mexico	polyprotein	Dengue virus 1
AHI43708.1	DENV-1/MX/BID-V7591/2007	Human	2007-01-01	North America	Mexico	polyprotein	Dengue virus 1
AHI43699.1	DENV-1/MX/BID-V7569/2009	Mosquito	2009-01-01	North America	Mexico	polyprotein	Dengue virus 1
AHI43725.1	DENV-1/MX/BID-V7611/2009	Human	2009-01-01	North America	Mexico	polyprotein	Dengue virus 1
AHI43717.1	DENV-1/MX/BID-V7601/2008	Human	2008-01-01	North America	Mexico	polyprotein	Dengue virus 1
AHI43709.1	DENV-1/MX/BID-V7592/2007	Human	2007-01-01	North America	Mexico	polyprotein	Dengue virus 1
AHG23209.1	DENV-1/MX/BID-V3725/2007	Human	2007-01-01	North America	Mexico	polyprotein	Dengue virus 1
AHI43690.1	DENV-1/MX/BID-V7302/2011	Human	2011-01-01	North America	Mexico	polyprotein	Dengue virus 1
QNL13176.1	Vero	Human	2019-10-07	North America	Mexico	polyprotein	Dengue virus 1
ACW82949.1	DENV-1/MX/BID-V3698/2007	Human	2007-01-01	North America	Mexico	polyprotein	Dengue virus 1
ACW82969.1	DENV-1/MX/BID-V3744/2008	Human	2008-01-01	North America	Mexico	polyprotein	Dengue virus 1
ACW82939.1	DENV-1/MX/BID-V3664/2006	Human	2006-01-01	North America	Mexico	polyprotein	Dengue virus 1
ACW82953.1	DENV-1/MX/BID-V3709/2007	Human	2007-01-01	North America	Mexico	polyprotein	Dengue virus 1
ACW82942.1	DENV-1/MX/BID-V3685/2007	Human	2007-01-01	North America	Mexico	polyprotein	Dengue virus 1
ACW82958.1	DENV-1/MX/BID-V3723/2007	Human	2007-01-01	North America	Mexico	polyprotein	Dengue virus 1
ACW82973.1	DENV-1/MX/BID-V3752/2008	Human	2008-01-01	North America	Mexico	polyprotein	Dengue virus 1
ACW82972.1	DENV-1/MX/BID-V3749/2008	Human	2008-01-01	North America	Mexico	polyprotein	Dengue virus 1
ADA60769.1	DENV-1/MX/BID-V3658/2006	Human	2006-01-01	North America	Mexico	polyprotein	Dengue virus 1
ADA60777.1	DENV-1/MX/BID-V3679/2007	Human	2007-01-01	North America	Mexico	polyprotein	Dengue virus 1
ADA60792.1	DENV-1/MX/BID-V3742/2007	Human	2007-01-01	North America	Mexico	polyprotein	Dengue virus 1
ADA60776.1	DENV-1/MX/BID-V3677/2007	Human	2007-01-01	North America	Mexico	polyprotein	Dengue virus 1
ADA60784.1	DENV-1/MX/BID-V3712/2007	Human	2007-01-01	North America	Mexico	polyprotein	Dengue virus 1
ADO97103.1	DENV-1/MX/BID-V3683/2007	Human	2007-01-01	North America	Mexico	polyprotein	Dengue virus 1
ADO97105.1	DENV-1/MX/BID-V3757/2008	Mosquito	2008-01-01	North America	Mexico	polyprotein	Dengue virus 1

AHI43716.1	DENV-1/MX/BID-V7600/2008	Human	2008-01-01	North America	Mexico	polyprotein	Dengue virus 1
AHI43719.1	DENV-1/MX/BID-V7605/2008	Human	2008-01-01	North America	Mexico	polyprotein	Dengue virus 1
AHI43711.1	DENV-1/MX/BID-V7594/2007	Human	2007-01-01	North America	Mexico	polyprotein	Dengue virus 1
AHI43689.1	DENV-1/MX/BID-V7298/2011	Human	2011-01-01	North America	Mexico	polyprotein	Dengue virus 1
AHI43701.1	DENV-1/MX/BID-V7576/2007	Human	2007-01-01	North America	Mexico	polyprotein	Dengue virus 1
AHI43707.1	DENV-1/MX/BID-V7590/2007	Human	2007-01-01	North America	Mexico	polyprotein	Dengue virus 1
AHI43752.1	DENV-1/MX/BID-V8196/2011	Human	2011-01-01	North America	Mexico	polyprotein	Dengue virus 1
AHI43695.1	DENV-1/MX/BID-V7563/2008	Mosquito	2008-01-01	North America	Mexico	polyprotein	Dengue virus 1
AHI43696.1	DENV-1/MX/BID-V7565/2008	Mosquito	2008-01-01	North America	Mexico	polyprotein	Dengue virus 1
AHI43726.1	DENV-1/MX/BID-V7612/2009	Human	2009-01-01	North America	Mexico	polyprotein	Dengue virus 1
AHG23193.1	DENV-1/MX/BID-V3705/2007	Human	2007-01-01	North America	Mexico	polyprotein	Dengue virus 1
AHI43704.1	DENV-1/MX/BID-V7587/2007	Human	2007-01-01	North America	Mexico	polyprotein	Dengue virus 1
AHI43718.1	DENV-1/MX/BID-V7604/2008	Human	2008-01-01	North America	Mexico	polyprotein	Dengue virus 1
AHI43702.1	DENV-1/MX/BID-V7580/2007	Human	2007-01-01	North America	Mexico	polyprotein	Dengue virus 1
ACW82956.1	DENV-2/MX/BID-V3714/2007	Human	2007-01-01	North America	Mexico	polyprotein	Dengue virus 2
ACQ44478.1	DENV-2/MX/BID-V2964/2008	Human	2008-01-01	North America	Mexico	polyprotein	Dengue virus 2
ACY70846.1	DENV-2/MX/BID-V3768/2004	Human	2004-01-01	North America	Mexico	polyprotein	Dengue virus 2
ADA60786.1	DENV-2/MX/BID-V3717/2007	Human	2007-01-01	North America	Mexico	polyprotein	Dengue virus 2
ADA60770.1	DENV-2/MX/BID-V3661/2006	Human	2006-01-01	North America	Mexico	polyprotein	Dengue virus 2
AET43253.1	DENV-2/MX/BID-V3763/2008	Human	2008-01-01	North America	Mexico	polyprotein	Dengue virus 2
AHI43753.1	DENV-2/MX/BID-V8199/2011	Human	2011-01-01	North America	Mexico	polyprotein	Dengue virus 2
AHI43694.1	DENV-2/MX/BID-V7542/2007	Human	2007-01-01	North America	Mexico	polyprotein	Dengue virus 2
AHI43691.1	DENV-2/MX/BID-V7535/2009	Mosquito	2009-01-01	North America	Mexico	polyprotein	Dengue virus 2
AHI43692.1	DENV-2/MX/BID-V7537/2010	Mosquito	2010-01-01	North America	Mexico	polyprotein	Dengue virus 2
QBQ87236.1	G2AE	Mosquito	2012-01-01	North America	Mexico	polyprotein	Dengue virus 2
QBK56834.1	C6L_48h	Unknown	2017-01-12	North America	Mexico	polyprotein	Dengue virus 2
QBQ87238.1	G3AE	Mosquito	2012-01-01	North America	Mexico	polyprotein	Dengue virus 2
ACW82937.1	DENV-2/MX/BID-V3654/2006	Human	2006-01-01	North America	Mexico	polyprotein	Dengue virus 2
ACW82955.1	DENV-2/MX/BID-V3713/2007	Human	2007-01-01	North America	Mexico	polyprotein	Dengue virus 2
ACS32035.1	DENV-2/MX/BID-V2959/2005	Human	2005-01-01	North America	Mexico	polyprotein	Dengue virus 2
QBK56835.1	C6L_P30	Unknown	2017-01-12	North America	Mexico	polyprotein	Dengue virus 2
QBK56836.1	C6L_P56	Unknown	2017-01-12	North America	Mexico	polyprotein	Dengue virus 2
QBQ87237.1	G1AE	Mosquito	2012-01-01	North America	Mexico	polyprotein	Dengue virus 2
QNG98968.1	Vero	Human	2018-08-16	North America	Mexico	polyprotein	Dengue virus 2

ACQ44481.1	DENV-3/MX/BID-V2989/2007	Human	2007-01-01	North America	Mexico	polyprotein	Dengue virus 3
ACQ44480.1	DENV-3/MX/BID-V2987/2006	Human	2006-01-01	North America	Mexico	polyprotein	Dengue virus 3

CD4 DENV-1 Epitopes

Epitope_ID	AA_Seq	Protein_Name	Start	End	Allele	step	Serotype	Method/Technique	Assay Group	Qualitative Measure	ACW82947.1	ACW82962.1	[illegible]
1067569	LARWGSFKKNGAIKV	C	66	80	HLA-DRB1*07:01	0%	Dengue virus 1	ELISPOT	IFNg release	Positive-Low	0	0	[illegible]
195246	EISNMLNIMNRRKRT	C	87	101	HLA-DRB1*13:01	0%	Dengue virus 1	ELISPOT	IFNg release	Positive	0	0	[illegible]
195315	FNMLKRARNRVSTVS	C	13	27	HLA-DRB1*08:02	0%	Dengue virus 1	ELISPOT	IFNg release	Positive	0	0	[illegible]
195498	IKVLRGFKKEISNML	C	78	92	HLA-DRB1*04:01	0%	Dengue virus 1	ELISPOT	IFNg release	Positive	0	0	[illegible]
195535	ISNMLNIMNRRKRSV	C	88	102	HLA-DRB1*08:02	0%	Dengue virus 1	ELISPOT	IFNg release	Positive	0	0	[illegible]
195557	KEISNMLNIMNRRKR	C	86	100	HLA-DRB1*13:01	0%	Dengue virus 1	ELISPOT	IFNg release	Positive	0	0	[illegible]
195560	KEVSNMLNIMNRRKR	C	86	100	HLA-DRB1*13:01	0%	Dengue virus 1	ELISPOT	IFNg release	Positive	0	0	[illegible]
195805	NMLNIMNRRKRSVTM	C	90	104	HLA-DRB1*11:01	0%	Dengue virus 1	ELISPOT	IFNg release	Positive	0	0	[illegible]
195883	RGFKKEISNMLNIMN	C	82	96	HLA-DRB1*04:01	0%	Dengue virus 1	ELISPOT	IFNg release	Positive	0	0	[illegible]
195884	RGFKKEISNMLSIMN	C	82	96	HLA-DRB1*04:01	0%	Dengue virus 1	ELISPOT	IFNg release	Positive	0	0	[illegible]
196146	VLRGFKKEISNMLNI	C	80	94	HLA-DRB1*04:01	0%	Dengue virus 1	ELISPOT	IFNg release	Positive	0	0	[illegible]
196188	VSVLFMLLPTALAFH	C	102	116	HLA-DRB1*01:01	0%	Dengue virus 1	ELISPOT	IFNg release	Positive	0	0	[illegible]
196195	VTMLFMLLPTALAFH	C	102	116	HLA-DRB1*04:01	0%	Dengue virus 1	ELISPOT	IFNg release	Positive	0	0	[illegible]
39096	LRGFKKEISNML	C	81	92	HLA-DPw4	0%	Dengue virus 1	ICS	TNF release	Positive	0	0	[illegible]
539048	AFMAFLRFLAIPPTA	C	27	41	HLA-DRB1*15:02	0%	Dengue virus 1	ELISPOT	IFNg release	Positive	0	0	[illegible]
539257	FKKEISNMLNIMNRR	C	84	98	HLA-DRB1*04:03	0%	Dengue virus 1	ELISPOT	IFNg release	Positive	0	0	[illegible]
539278	GAIKVLRGFKKEISN	C	76	90	HLA-DRB1*12:02	0%	Dengue virus 1	ELISPOT	IFNg release	Positive	0	0	[illegible]
539616	LRGFKKEISNMLNIM	C	81	95	HLA-DRB1*10:01	0%	Dengue virus 1	ELISPOT	IFNg release	Positive	0	0	[illegible]
539681	MLNIRNRRKRSVTML	C	91	105	HLA-DRB1*13:01	0%	Dengue virus 1	ELISPOT	IFNg release	Positive	0	0	[illegible]
539866	RPSFNMLKRVRNRVS	C	10	24	HLA-DRB1*08:03	0%	Dengue virus 1	ELISPOT	IFNg release	Positive	0	0	[illegible]
540117	WGSFKKNGAIKVLRG	C	47	61	HLA-DRB1*09:01	0%	Dengue virus 1	ELISPOT	IFNg release	Positive	0	0	[illegible]
740331	SNMLNIMNRRKRSVT	C	89	103	HLA-DRB1*14:02	0%	Dengue virus 1	ELISPOT	IFNg release	Positive	0	0	[illegible]
867523	KVLRGFKKEISNMLN	C	79	93	HLA-DRB3*02:02	0%	Dengue virus 1	ELISPOT	IFNg release	Positive	0	0	[illegible]
195589	KSLLFKTSVGVNMCT	PreM	135	149	HLA-DRB1*07:01	0%	Dengue virus 1	ELISPOT	IFNg release	Positive	0	0	[illegible]
869062	SVGVNMCTLIAMDLG	PreM	142	156	HLA-DQA1*01:02/DQB1*06:02	0%	Dengue virus 1	ELISPOT	IFNg release	Positive	0	0	[illegible]
195359	GGLFTSVGKLIHQVF	E	706	720	HLA-DRB1*11:01	0%	Dengue virus 1	ELISPOT	IFNg release	Positive	0	0	[illegible]
195361	GGVFTSVGKLVHQIF	E	426	440	HLA-DRB1*11:01	0%	Dengue virus 1	ELISPOT	IFNg release	Positive	0	0	[illegible]
195484	IGGLFTSVGKLIHQV	E	705	719	HLA-DRB1*07:01	0%	Dengue virus 1	ELISPOT	IFNg release	Positive	0	0	[illegible]
539840	REDLLVTFKTAHAKK	E	513	527	HLA-DRB1*04:03	0%	Dengue virus 1	ELISPOT	IFNg release	Positive	0	0	[illegible]
539854	RKDLLVTFKTAHAKK	E	513	527	HLA-DRB1*04:03	0%	Dengue virus 1	ELISPOT	IFNg release	Positive	0	0	[illegible]
867045	HAKKQEVAVLGSQEG	E	244	258	HLA-DQA1*03:01/DQB1*03:02	0%	Dengue virus 1	ELISPOT	IFNg release	Positive	0	0	[illegible]
867378	KCRLKMDKLTLKGMS	E	564	578	HLA-DRB3*02:02	0%	Dengue virus 1	ELISPOT	IFNg release	Positive	0	0	[illegible]
869085	SYIVIGAGKKALKLS	E	376	390	HLA-DRB5*01:01	0%	Dengue virus 1	ELISPOT	IFNg release	Positive	0	0	[illegible]
869690	WLAHKQWFLDLPLPW	E	206	220	HLA-DQA1*01:01/DQB1*05:01	0%	Dengue virus 1	ELISPOT	IFNg release	Positive	0	0	[illegible]
195684	LNHILLENDIKFTVV	NS1	850	864	HLA-DRB1*03:01	0%	Dengue virus 1	ELISPOT	IFNg release	Positive	0	0	[illegible]
195690	LNYILLENDMKFTVV	NS1	850	864	HLA-DRB1*03:01	0%	Dengue virus 1	ELISPOT	IFNg release	Positive	0	0	[illegible]
195785	NETWKLTRASFIEVK	NS1	982	996	HLA-DRB1*07:01	0%	Dengue virus 1	ELISPOT	IFNg release	Positive	0	0	[illegible]
591686	KFTVVVGDVSGILAQ	NS1	860	874	HLA-DRB1*03:01	0%	Dengue virus 1	ELISPOT	IFNg release	Positive	0	0	[illegible]
868492	QISNELNYILLENDM	NS1	845	859	HLA-DPB1*01:01	0%	Dengue virus 1	ELISPOT	IFNg release	Positive	0	0	[illegible]
868630	QYKFQADSPKKLSAA	NS1	806	820	HLA-DRB3*02:02	0%	Dengue virus 1	ELISPOT	IFNg release	Positive	0	0	[illegible]
167816	SLVASVELPNSLEELGDGLAMGIMI	NS2a	109	133	HLA class II	0%	Dengue virus 1	ELISPOT	IFNg release	Positive	0	0	[illegible]
195196	AWKTMAMALSIVSLF	NS2a	1291	1305	HLA-DRB1*08:02	0%	Dengue virus 1	ELISPOT	IFNg release	Positive	0	0	[illegible]
195285	FAVGLLFRRLTSREI	NS2a	1214	1228	HLA-DRB1*11:01	0%	Dengue virus 1	ELISPOT	IFNg release	Positive	0	0	[illegible]
195323	FQSHQLWATLLSLTF	NS2a	1267	1281	HLA-DPB1*04:02	0%	Dengue virus 1	ELISPOT	IFNg release	Positive	0	0	[illegible]
195389	GLLFRKLTSREVLLL	NS2a	1217	1231	HLA-DRB1*11:01	0%	Dengue virus 1	ELISPOT	IFNg release	Positive	0	0	[illegible]
195390	GLLFRRLTSREILLL	NS2a	1217	1231	HLA-DRB1*07:01	0%	Dengue virus 1	ELISPOT	IFNg release	Positive	0	0	[illegible]
195392	GLLFRRLTSREVPLL	NS2a	1217	1231	HLA-DRB1*10:01	0%	Dengue virus 1	ELISPOT	IFNg release	Positive	0	0	[illegible]
195423	GTLVVFFLLIMGQLT	NS2a	1162	1176	HLA-DRB1*15:01	0%	Dengue virus 1	ELISPOT	IFNg release	Positive	0	0	[illegible]
195595	KTMAMVLSIVSLFPL	NS2a	1293	1307	HLA-DRB1*03:01	0%	Dengue virus 1	ELISPOT	IFNg release	Positive	0	0	[illegible]
195614	LAVGLLFRRLTSREV	NS2a	1214	1228	HLA-DRB1*11:01	0%	Dengue virus 1	ELISPOT	IFNg release	Positive	0	0	[illegible]
195670	LLSLTFIKTTFSLHY	NS2a	1276	1290	HLA-DRB1*07:01	0%	Dengue virus 1	ELISPOT	IFNg release	Positive	0	0	[illegible]
195713	LSLTFIRTSLSLDYA	NS2a	1277	1291	HLA-DRB1*03:01	0%	Dengue virus 1	ELISPOT	IFNg release	Positive	0	0	[illegible]
195745	MAMVLSIVSLFPLCL	NS2a	1295	1309	HLA-DRB1*07:01	0%	Dengue virus 1	ELISPOT	IFNg release	Positive	0	0	[illegible]
195756	MGLLFRRLTSREVLL	NS2a	1216	1230	HLA-DRB1*11:01	0%	Dengue virus 1	ELISPOT	IFNg release	Positive	0	0	[illegible]
195881	REVLLLTVGLSLVAC	NS2a	1226	1240	HLA-DRB1*07:01	0%	Dengue virus 1	ELISPOT	IFNg release	Positive	0	0	[illegible]
195959	SILIEEVMRSRWSRK	NS2a	1143	1157	HLA-DRB1*11:04	0%	Dengue virus 1	ELISPOT	IFNg release	Positive	0	0	[illegible]
196216	WKTMAMVLSIVSLFP	NS2a	1292	1306	HLA-DRB1*08:02	0%	Dengue virus 1	ELISPOT	IFNg release	Positive	0	0	[illegible]
539086	AMGIMILKLLTDFQS	NS2a	1255	1269	HLA-DRB1*04:03	0%	Dengue virus 1	ELISPOT	IFNg release	Positive	0	0	[illegible]
539272	FSLHYAWKTMAMVLS	NS2a	1286	1300	HLA-DRB1*09:01	0%	Dengue virus 1	ELISPOT	IFNg release	Positive	0	0	[illegible]
539517	KTTFSLHYAWKTMAM	NS2a	1283	1297	HLA-DRB1*09:01	0%	Dengue virus 1	ELISPOT	IFNg release	Positive	0	0	[illegible]
539624	LSLTFIKTTLSLHYA	NS2a	1277	1291	HLA-DRB1*14:04	0%	Dengue virus 1	ELISPOT	IFNg release	Positive	0	0	[illegible]
539625	LSLTFIKTTYSLHYA	NS2a	1277	1291	HLA-DRB1*14:04	0%	Dengue virus 1	ELISPOT	IFNg release	Positive	0	0	[illegible]
539626	LSLTFIRSTIPLVMA	NS2a	1275	1289	HLA-DRB1*14:04	0%	Dengue virus 1	ELISPOT	IFNg release	Positive	0	0	[illegible]
539651	LVVFLLLIMGQLTWS	NS2a	1164	1178	HLA-DRB1*15:06	0%	Dengue virus 1	ELISPOT	IFNg release	Positive	0	0	[illegible]
539680	MLMTGTLVVFFLLIM	NS2a	1158	1172	HLA-DRB1*15:06	0%	Dengue virus 1	ELISPOT	IFNg release	Positive	0	0	[illegible]
539912	SLDYAWKTMAMALSI	NS2a	1287	1301	HLA-DRB1*09:01	0%	Dengue virus 1	ELISPOT	IFNg release	Positive	0	0	[illegible]
539913	SLHYAWKTMAMVLSI	NS2a	1287	1301	HLA-DRB1*09:01	0%	Dengue virus 1	ELISPOT	IFNg release	Positive	0	0	[illegible]
539918	SLTFIKTTLSLHHAW	NS2a	1278	1292	HLA-DRB1*14:04	0%	Dengue virus 1	ELISPOT	IFNg release	Positive	0	0	[illegible]
539970	TFSLHYAWKTMAMVL	NS2a	1285	1299	HLA-DRB1*14:06	0%	Dengue virus 1	ELISPOT	IFNg release	Positive	0	0	[illegible]
539974	TGTLVVFLLLTLGQL	NS2a	1161	1175	HLA-DRB1*15:06	0%	Dengue virus 1	ELISPOT	IFNg release	Positive	0	0	[illegible]
540006	TTLLSLTFIRTSLSL	NS2a	1274	1288	HLA-DRB1*12:02	0%	Dengue virus 1	ELISPOT	IFNg release	Positive	0	0	[illegible]
591779	TLAVFLLLTMGQLTW	NS2a	36	50	HLA-DRB1*11:01	0%	Dengue virus 1	ELISPOT	IFNg release	Positive	0	0	[illegible]
739508	LLCISIMIEEVMRSR	NS2a	1139	1153	HLA-DRB1*11:04	0%	Dengue virus 1	ELISPOT	IFNg release	Positive	0	0	[illegible]
739578	LSLTFIKTTLSLHHA	NS2a	1277	1291	HLA-DRB1*04:07	0%	Dengue virus 1	ELISPOT	IFNg release	Positive	0	0	[illegible]
866767	FQSYQLWATLLSLTF	NS2a	1267	1281	HLA-DPB1*04:02	0%	Dengue virus 1	ELISPOT	IFNg release	Positive	0	0	[illegible]
866873	GILAVFFLLIMGQLT	NS2a	1162	1176	HLA-DPB1*04:02	0%	Dengue virus 1	ELISPOT	IFNg release	Positive	0	0	[illegible]
867232	ILKLLTDFQSHQLWA	NS2a	1260	1274	HLA-DPB1*01:01	0%	Dengue virus 1	ELISPOT	IFNg release	Positive	0	0	[illegible]
867997	LTSREILLLTVGLSL	NS2a	1223	1237	HLA-DPB1*04:02	0%	Dengue virus 1	ELISPOT	IFNg release	Positive	0	0	[illegible]
868553	QPHQLWATLLSLTFV	NS2a	1268	1282	HLA-DPB1*04:02	0%	Dengue virus 1	ELISPOT	IFNg release	Positive	0	0	[illegible]
869103	TDFQPHQLWTTLLSL	NS2a	1265	1279	HLA-DPB1*04:02	0%	Dengue virus 1	ELISPOT	IFNg release	Positive	0	0	[illegible]
195191	AVGIVSILLSSLLKN	NS2b	1355	1369	HLA-DRB1*08:02	0%	Dengue virus 1	ELISPOT	IFNg release	Positive	0	0	[illegible]
195510	IMAVGIVSILLSSLL	NS2b	1353	1367	HLA-DRB1*07:01	0%	Dengue virus 1	ELISPOT	IFNg release	Positive	0	0	[illegible]
1392180	GKEIVDLMCHATFTM	NS3	1730	1744		0%	Dengue virus 1	biological activity	activation	Positive	0	0	[illegible]
195622	LDDGIYRIMQRGLLG	NS3	1493	1507	HLA-DRB1*11:01	0%	Dengue virus 1	ELISPOT	IFNg release	Positive	0	0	[illegible]
24915	HTGKEIVDLMCHATF	NS3	251	265	HLA-DPw2	0%	Dengue virus 1	ICS	IFNg release	Positive	0	0	[illegible]
539156	DGIYRIMQRGLLGRS	NS3	1495	1509	HLA-DRB1*12:02	0%	Dengue virus 1	ELISPOT	IFNg release	Positive	0	0	[illegible]
539309	GIYRIMQRGLLGRSQ	NS3	1496	1510	HLA-DRB1*14:04	0%	Dengue virus 1	ELISPOT	IFNg release	Positive	0	0	[illegible]
539839	REAIRRKLRTLILAP	NS3	1685	1699	HLA-DRB1*14:04	0%	Dengue virus 1	ELISPOT	IFNg release	Positive	0	0	[illegible]
539993	TNCLRKNGKRVIQLS	NS3	1848	1862	HLA-DRB1*13:01	0%	Dengue virus 1	ELISPOT	IFNg release	Positive	0	0	[illegible]
540075	VREAIKRKLRTLVLA	NS3	1684	1698	HLA-DRB1*12:02	0%	Dengue virus 1	ELISPOT	IFNg release	Positive	0	0	[illegible]
54391	RKLTIMDLHPGSGKT	NS3	1662	1676	HLA class II	0%	Dengue virus 1	ICS	IFNg release	Positive	0	0	[illegible]
738417	AAAIFMTATPPGSAE	NS3	1785	1799	HLA-DRB1*04:07	0%	Dengue virus 1	ELISPOT	IFNg release	Positive	0	0	[illegible]
738418	AAAIFMTATPPGSME	NS3	1785	1799	HLA-DRB1*04:07	0%	Dengue virus 1	ELISPOT	IFNg release	Positive	0	0	[illegible]
867025	GVFQENVFHTMWHVT	NS3	1514	1528	HLA-DPB1*04:01	0%	Dengue virus 1	ELISPOT	IFNg release	Positive	0	0	[illegible]
195485	IGLLCVMASSALLWM	NS4a	2177	2191	HLA-DRB1*07:01	0%	Dengue virus 1	ELISPOT	IFNg release	Positive	0	0	[illegible]
195680	LMLLALTAVLTGGVT	NS4a	2149	2163	HLA-DRB1*01:01	0%	Dengue virus 1	ELISPOT	IFNg release	Positive	0	0	[illegible]
196046	TIETLMLLALIAALT	NS4a	2145	2159	HLA-DRB1*01:01	0%	Dengue virus 1	ELISPOT	IFNg release	Positive	0	0	[illegible]
739034	GLGKTSIGLLCVMAS	NS4a	2171	2185	HLA-DRB1*11:04	0%	Dengue virus 1	ELISPOT	IFNg release	Positive	0	0	[illegible]
739085	GVTLFFLSGRGLGKT	NS4a	2161	2175	HLA-DRB1*01:02	0%	Dengue virus 1	ELISPOT	IFNg release	Positive	0	0	[illegible]
867567	LCVMASSALLWMANV	NS4a	2180	2194	HLA-DQA1*01:02/DQB1*06:02	0%	Dengue virus 1	ELISPOT	IFNg release	Positive	0	0	[illegible]
867760	LLCVMASSVLLWMAS	NS4a	2179	2193	HLA-DQA1*01:02/DQB1*06:02	0%	Dengue virus 1	ELISPOT	IFNg release	Positive	0	0	[illegible]
195308	FMLVAHYAIIGPGLQ	NS4b	2357	2371	HLA-DRB1*07:01	0%	Dengue virus 1	ELISPOT	IFNg release	Positive	0	0	[illegible]
195455	HHHATMLDVDLRPAS	NS4b	2267	2281	HLA-DRB1*03:01	0%	Dengue virus 1	ELISPOT	IFNg release	Positive	0	0	[illegible]
738560	AWTLYAVATTIITPM	NS4b	2282	2296	HLA-DRB1*04:07	0%	Dengue virus 1	ELISPOT	IFNg release	Positive	0	0	[illegible]
868336	PLTLTAAVLMLAAHY	NS4b	2349	2363	HLA-DQA1*01:02/DQB1*06:02	0%	Dengue virus 1	ELISPOT	IFNg release	Positive	0	0	[illegible]
195781	NEDHWFSRGNSLSGV	NS5	2985	2999	HLA-DRB1*04:01	0%	Dengue virus 1	ELISPOT	IFNg release	Positive	0	0	[illegible]
195923	RWLWGFLSRNKKPRI	NS5	2871	2885	HLA-DRB1*15:01	0%	Dengue virus 1	ELISPOT	IFNg release	Positive	0	0	[illegible]
196191	VTARWLWGFLSRNKK	NS5	2868	2882	HLA-DRB1*15:01	0%	Dengue virus 1	ELISPOT	IFNg release	Positive	0	0	[illegible]
196242	YQNKVVRVQRPAKSG	NS5	3065	3079	HLA-DRB1*08:02	0%	Dengue virus 1	ELISPOT	IFNg release	Positive	0	0	[illegible]
539537	LAKAIFKLTYQNKVV	NS5	3056	3070	HLA-DRB1*14:04	0%	Dengue virus 1	ELISPOT	IFNg release	Positive	0	0	[illegible]
540018	TYGLNTFTNMEAQLI	NS5	3098	3112	HLA-DRB1*15:06	0%	Dengue virus 1	ELISPOT	IFNg release	Positive	0	0	[illegible]
540083	VRVQRPAKSGTVMDV	NS5	96	110	HLA-DRB1*13:01	0%	Dengue virus 1	ELISPOT	IFNg release	Positive	0	0	[illegible]
195113	AFLRFLAIPPTAGIL	C	30	44	HLA-DRB1*01:01	100%	Dengue virus 1	ELISPOT	IFNg release	Positive	1	1	[illegible]
195299	FIAFLRFLAIPPTAG	C	28	42	HLA-DRB1*01:01	100%	Dengue virus 1	ELISPOT	IFNg release	Positive	1	1	[illegible]
195314	FNMLKRARNRVSTGS	C	1	15	HLA-DRB1*08:02	100%	Dengue virus 1	ELISPOT	IFNg release	Positive	1	1	[illegible]
195468	IAFLRFLAIPPTAGI	C	29	43	HLA-DRB1*01:01	100%	Dengue virus 1	ELISPOT	IFNg release	Positive	1	1	[illegible]
195540	ISSMLNIMNRRKRSV	C	76	90	HLA-DRB1*14:02	100%	Dengue virus 1	ELISPOT	IFNg release	Positive	1	1	[illegible]
195681	LMLLPTALAFHLTTR	C	94	108	HLA-DRB1*04:01	100%	Dengue virus 1	ELISPOT	IFNg release	Positive	1	1	[illegible]
195944	SFNMLKRARNRVSTG	C	12	26	HLA-DRB1*11:01	100%	Dengue virus 1	ELISPOT	IFNg release	Positive	1	1	[illegible]
196151	VMAFIAFLRFLAIPP	C	25	39	HLA-DRB1*11:01	100%	Dengue virus 1	ELISPOT	IFNg release	Positive	1	1	[illegible]
539043	AFIAFLRFLAIPPTA	C	27	41	HLA-DRB1*12:02	100%	Dengue virus 1	ELISPOT	IFNg release	Positive	1	1	[illegible]
539785	PSFNMLKRARNRVST	C	11	25	HLA-DRB1*10:01	100%	Dengue virus 1	ELISPOT	IFNg release	Positive	1	1	[illegible]
195260	ENLKYSVIVTVHTGD	E	128	142	HLA-DRB1*07:01	100%	Dengue virus 1	ELISPOT	IFNg release	Positive	1	1	[illegible]
195362	GGVFTSVGKLVHQVF	E	426	440	HLA-DRB1*11:01	100%	Dengue virus 1	ELISPOT	IFNg release	Positive	1	1	[illegible]
195646	LITANPIVTDKEKPV	E	346	360	HLA-DRB1*03:01	100%	Dengue virus 1	ELISPOT	IFNg release	Positive	1	1	[illegible]
195814	NRQDLLVTFKTAHAK	E	227	241	HLA-DRB1*04:01	100%	Dengue virus 1	ELISPOT	IFNg release	Positive	1	1	[illegible]
195874	QYENLKYSVIVTVHT	E	126	140	HLA-DRB1*07:01	100%	Dengue virus 1	ELISPOT	IFNg release	Positive	1	1	[illegible]
539169	DLLVTFKTAHAKKQE	E	230	244	HLA-DRB1*14:04	100%	Dengue virus 1	ELISPOT	IFNg release	Positive	1	1	[illegible]
539369	GVSWTMKIGIGILLT	E	445	459	HLA-DRB1*15:02	100%	Dengue virus 1	ELISPOT	IFNg release	Positive	1	1	[illegible]
539593	LLVTFKTAHAKKQEV	E	236	250	HLA-DRB1*14:02	100%	Dengue virus 1	ELISPOT	IFNg release	Positive	1	1	[illegible]
539670	MKIGIGILLTWLGLN	E	450	464	HLA-DRB1*15:06	100%	Dengue virus 1	ELISPOT	IFNg release	Positive	1	1	[illegible]
539800	QDLLVTFKTAHAKKQ	E	229	243	HLA-DRB1*09:01	100%	Dengue virus 1	ELISPOT	IFNg release	Positive	1	1	[illegible]
539869	RQDLLVTFKTAHAKK	E	228	242	HLA-DRB1*08:03	100%	Dengue virus 1	ELISPOT	IFNg release	Positive	1	1	[illegible]
741745	MRCVGIGNRDFVEGLSGATW	E	281	300	HLA class II	100%	Dengue virus 1	ELISPOT	IFNg release	Positive	1	1	[illegible]
868013	LVHKQWFLDLPLPWT	E	487	501	HLA-DQA1*01:01/DQB1*05:01	100%	Dengue virus 1	ELISPOT	IFNg release	Positive	1	1	[illegible]
869691	WLVHKQWFLDLPLPW	E	486	500	HLA-DQA1*01:01/DQB1*05:01	100%	Dengue virus 1	ELISPOT	IFNg release	Positive	1	1	[illegible]
95154	SSIGKMFEATARG	E	396	408	HLA-DQ8	100%	Dengue virus 1	ELISPOT	IFNg release	Positive	1	1	[illegible]
132583	QYKFQADSPKRLSAA	NS1	31	45	HLA-DRB1*03:01	100%	Dengue virus 1	ELISPOT	IFNg release	Positive	1	1	[illegible]
195685	LNHILLENDMKFTVV	NS1	75	89	HLA-DRB1*03:01	100%	Dengue virus 1	ELISPOT	IFNg release	Positive	1	1	[illegible]
740562	TVVVGDANGILAQGK	NS1	862	876	HLA-DRB1*01:02	100%	Dengue virus 1	ELISPOT	IFNg release	Positive	1	1	[illegible]
866693	FGIFTTNIWLKLRDS	NS1	196	210	HLA-DPB1*04:01	100%	Dengue virus 1	ELISPOT	IFNg release	Positive	1	1	[illegible]
867041	HADMGYWIESEKNET	NS1	231	245	HLA-DQA1*01:01/DQB1*05:01	100%	Dengue virus 1	ELISPOT	IFNg release	Positive	1	1	[illegible]
868574	QRAWNIWEVEDYGFG	NS1	183	197	HLA-DQA1*01:01/DQB1*05:01	100%	Dengue virus 1	ELISPOT	IFNg release	Positive	1	1	[illegible]
869135	TEQYKFQADSPKRLS	NS1	65	79	HLA-DRB5*01:01	100%	Dengue virus 1	ELISPOT	IFNg release	Positive	1	1	[illegible]
869721	YGFGIFTTNIWLKLR	NS1	194	208	HLA-DPB1*02:01	100%	Dengue virus 1	ELISPOT	IFNg release	Positive	1	1	[illegible]
195708	LSIVSLFPLCLSTTS	NS2a	1299	1313	HLA-DRB1*01:01	100%	Dengue virus 1	ELISPOT	IFNg release	Positive	1	1	[illegible]
539398	IEEVMRSRWSRKMLM	NS2a	1146	1160	HLA-DRB1*13:01	100%	Dengue virus 1	ELISPOT	IFNg release	Positive	1	1	[illegible]
540155	YLALMATFKMRPMFA	NS2a	1201	1215	HLA-DRB1*13:01	100%	Dengue virus 1	ELISPOT	IFNg release	Positive	1	1	[illegible]
738679	DLIRLCIMVGANASD	NS2a	1179	1193	HLA-DRB1*11:04	100%	Dengue virus 1	ELISPOT	IFNg release	Positive	1	1	[illegible]
868098	MGMGTTYLALMATFK	NS2a	456	470	HLA-DRB5*01:01	100%	Dengue virus 1	ELISPOT	IFNg release	Positive	1	1	[illegible]
867359	IVSILLSSLLKNDVP	NS2b	1358	1372	HLA-DPB1*04:02	100%	Dengue virus 1	ELISPOT	IFNg release	Positive	1	1	[illegible]
195163	ANCLRKNGKRVIQLS	NS3	1848	1862	HLA-DRB1*13:01	100%	Dengue virus 1	ELISPOT	IFNg release	Positive	1	1	[illegible]
195165	ANFRADRVIDPRRCL	NS3	1891	1905	HLA-DRB1*03:01	100%	Dengue virus 1	ELISPOT	IFNg release	Positive	1	1	[illegible]
195296	FHTMWHVTRGAVLMY	NS3	1521	1535	HLA-DRB1*07:01	100%	Dengue virus 1	ELISPOT	IFNg release	Positive	1	1	[illegible]
195377	GIYRILQRGLLGRSQ	NS3	1496	1510	HLA-DRB1*11:01	100%	Dengue virus 1	ELISPOT	IFNg release	Positive	1	1	[illegible]
195549	IYRILQRGLLGRSQV	NS3	1497	1511	HLA-DRB1*15:01	100%	Dengue virus 1	ELISPOT	IFNg release	Positive	1	1	[illegible]
195777	NCLRKNGKRVIQLSR	NS3	1849	1863	HLA-DRB1*11:01	100%	Dengue virus 1	ELISPOT	IFNg release	Positive	1	1	[illegible]
195778	NDIANCLRKNGKRVI	NS3	1845	1859	HLA-DRB1*11:01	100%	Dengue virus 1	ELISPOT	IFNg release	Positive	1	1	[illegible]
195914	RTLILAPTRVVASEM	NS3	1693	1707	HLA-DRB1*10:01	100%	Dengue virus 1	ELISPOT	IFNg release	Positive	1	1	[illegible]
196130	VILKDGPERVILAGP	NS3	1908	1922	HLA-DRB1*03:01	100%	Dengue virus 1	ELISPOT	IFNg release	Positive	1	1	[illegible]
196138	VLDDGIYRILQRGLL	NS3	1492	1506	HLA-DRB1*11:01	100%	Dengue virus 1	ELISPOT	IFNg release	Positive	1	1	[illegible]
196197	VTRGAVLMYQGKRLE	NS3	1527	1541	HLA-DRB1*15:01	100%	Dengue virus 1	ELISPOT	IFNg release	Positive	1	1	[illegible]
30397	KEGERKKLRPRWLDA	NS3	585	599	HLA class II	100%	Dengue virus 1	ICS	IFNg release	Positive	1	1	[illegible]
49768	PTRVVASEMAEALKG	NS3	224	238	HLA-DRB1*15:01	100%	Dengue virus 1	ICS	TNF release	Positive	1	1	[illegible]
539022	AAAIFMTATPPGSVE	NS3	1785	1799	HLA-DRB1*04:03	100%	Dengue virus 1	ELISPOT	IFNg release	Positive	1	1	[illegible]
539027	AAIFMTATPPGSVEA	NS3	1786	1800	HLA-DRB1*10:01	100%	Dengue virus 1	ELISPOT	IFNg release	Positive	1	1	[illegible]
539155	DGIYRILQRGLLGRS	NS3	1495	1509	HLA-DRB1*12:02	100%	Dengue virus 1	ELISPOT	IFNg release	Positive	1	1	[illegible]
539171	DLMCHATFTMRLLSP	NS3	1734	1748	HLA-DRB1*13:01	100%	Dengue virus 1	ELISPOT	IFNg release	Positive	1	1	[illegible]
539288	GEAAAIFMTATPPGS	NS3	1783	1797	HLA-DRB1*04:03	100%	Dengue virus 1	ELISPOT	IFNg release	Positive	1	1	[illegible]
539543	LDDGIYRILQRGLLG	NS3	1493	1507	HLA-DRB1*12:02	100%	Dengue virus 1	ELISPOT	IFNg release	Positive	1	1	[illegible]
539715	NGKRVIQLSRKTFDT	NS3	1854	1868	HLA-DRB1*12:02	100%	Dengue virus 1	ELISPOT	IFNg release	Positive	1	1	[illegible]
539986	TLILAPTRVVASEMA	NS3	1694	1708	HLA-DRB1*13:01	100%	Dengue virus 1	ELISPOT	IFNg release	Positive	1	1	[illegible]
866721	FKTPEGEVGAIALDF	NS3	1591	1605	HLA-DQA1*03:01/DQB1*03:02	100%	Dengue virus 1	ELISPOT	IFNg release	Positive	1	1	[illegible]
868113	MIIMDEAHFTDPASI	NS3	1756	1770	HLA-DQA1*01:01/DQB1*05:01	100%	Dengue virus 1	ELISPOT	IFNg release	Positive	1	1	[illegible]
868343	PNYNMIIMDEAHFTD	NS3	1752	1766	HLA-DQA1*05:01/DQB1*02:01	100%	Dengue virus 1	ELISPOT	IFNg release	Positive	1	1	[illegible]
869688	WHVTRGAVLMYQGKR	NS3	1525	1539	HLA-DQA1*01:02/DQB1*06:02	100%	Dengue virus 1	ELISPOT	IFNg release	Positive	1	1	[illegible]
95169	YRILQRGLLGRSQ	NS3	23	35	HLA-DR1	100%	Dengue virus 1	ELISPOT	IFNg release	Positive	1	1	[illegible]
195596	KTSIGLLCVTASSAL	NS4a	2174	2188	HLA-DRB1*07:01	100%	Dengue virus 1	ELISPOT	IFNg release	Positive	1	1	[illegible]
196214	WIAASIILEFFLMVL	NS4a	2198	2212	HLA-DRB1*15:01	100%	Dengue virus 1	ELISPOT	IFNg release	Positive	1	1	[illegible]
867109	HWIAASIILEFFLMV	NS4a	2197	2211	HLA-DPB1*04:01	100%	Dengue virus 1	ELISPOT	IFNg release	Positive	1	1	[illegible]
867112	IAASIILEFFLMVLL	NS4a	2199	2213	HLA-DQA1*01:01/DQB1*05:01	100%	Dengue virus 1	ELISPOT	IFNg release	Positive	1	1	[illegible]
195114	AGAGLAFSLMKSLGG	NS4b	2476	2490	HLA-DRB1*04:01	100%	Dengue virus 1	ELISPOT	IFNg release	Positive	1	1	[illegible]
195193	AVLMLVAHYAIIGPG	NS4b	2355	2369	HLA-DRB1*01:01	100%	Dengue virus 1	ELISPOT	IFNg release	Positive	1	1	[illegible]
195441	HATMLDVDLRPASAW	NS4b	2269	2283	HLA-DRB1*03:01	100%	Dengue virus 1	ELISPOT	IFNg release	Positive	1	1	[illegible]
195476	IDLDPVVYDAKFEKQ	NS4b	2398	2412	HLA-DRB1*03:01	100%	Dengue virus 1	ELISPOT	IFNg release	Positive	1	1	[illegible]
195561	KFWNTTIAVSMANIF	NS4b	2456	2470	HLA-DRB1*07:01	100%	Dengue virus 1	ELISPOT	IFNg release	Positive	1	1	[illegible]
195620	LCTSQILLMRTTWAL	NS4b	2421	2435	HLA-DRB1*04:01	100%	Dengue virus 1	ELISPOT	IFNg release	Positive	1	1	[illegible]
195637	LGQIMLLILCTSQIL	NS4b	2413	2427	HLA-DRB1*08:02	100%	Dengue virus 1	ELISPOT	IFNg release	Positive	1	1	[illegible]
195682	LMLVAHYAIIGPGLQ	NS4b	2357	2371	HLA-DRB1*07:01	100%	Dengue virus 1	ELISPOT	IFNg release	Positive	1	1	[illegible]
195760	MLLILCTSQILLMRT	NS4b	2417	2431	HLA-DRB1*07:01	100%	Dengue virus 1	ELISPOT	IFNg release	Positive	1	1	[illegible]
195848	QIMLLILCTSQILLM	NS4b	2415	2429	HLA-DRB1*07:01	100%	Dengue virus 1	ELISPOT	IFNg release	Positive	1	1	[illegible]
196143	VLMLVAHYAIIGPGL	NS4b	2356	2370	HLA-DRB1*01:01	100%	Dengue virus 1	ELISPOT	IFNg release	Positive	1	1	[illegible]
539322	GLAFSLMKSLGGGRR	NS4b	2479	2493	HLA-DRB1*10:01	100%	Dengue virus 1	ELISPOT	IFNg release	Positive	1	1	[illegible]
539725	NISLTAIANQAAILM	NS4b	2307	2321	HLA-DRB1*04:03	100%	Dengue virus 1	ELISPOT	IFNg release	Positive	1	1	[illegible]
738686	DLRPASAWTLYAVAT	NS4b	2276	2290	HLA-DRB1*01:02	100%	Dengue virus 1	ELISPOT	IFNg release	Positive	1	1	[illegible]
740732	WTLYAVATTVITPMM	NS4b	2283	2297	HLA-DRB1*04:07	100%	Dengue virus 1	ELISPOT	IFNg release	Positive	1	1	[illegible]
866002	AIANQAAILMGLDKG	NS4b	2312	2326	HLA-DQA1*01:02/DQB1*06:02	100%	Dengue virus 1	ELISPOT	IFNg release	Positive	1	1	[illegible]
869401	VDGIVAIDLDPVVYD	NS4b	2392	2406	HLA-DQA1*01:01/DQB1*05:01	100%	Dengue virus 1	ELISPOT	IFNg release	Positive	1	1	[illegible]
1066544	ACLGKSYAQMWQLMY	NS5	3244	3258	HLA-DRB1*04:01	100%	Dengue virus 1	ELISPOT	IFNg release	Positive-Low	1	1	[illegible]
195147	AKNGTVMDVISRRDQ	NS5	3076	3090	HLA-DRB1*03:01	100%	Dengue virus 1	ELISPOT	IFNg release	Positive	1	1	[illegible]
195150	AKWLWGFLSRNKKPR	NS5	2870	2884	HLA-DRB1*15:01	100%	Dengue virus 1	ELISPOT	IFNg release	Positive	1	1	[illegible]
195168	AQMWQLMYFHRRDLR	NS5	3251	3265	HLA-DRB1*13:01	100%	Dengue virus 1	ELISPOT	IFNg release	Positive	1	1	[illegible]
195208	CVYNMMGKREKKLGE	NS5	2942	2956	HLA-DRB1*11:01	100%	Dengue virus 1	ELISPOT	IFNg release	Positive	1	1	[illegible]
195360	GGMLVRNPLSRNSTH	NS5	2694	2708	HLA-DRB1*08:02	100%	Dengue virus 1	ELISPOT	IFNg release	Positive	1	1	[illegible]
195385	GLHKLGYILRDISKI	NS5	3003	3017	HLA-DRB1*08:02	100%	Dengue virus 1	ELISPOT	IFNg release	Positive	1	1	[illegible]
195421	GTGNIVSAVNMTSRM	NS5	2716	2730	HLA-DRB1*08:02	100%	Dengue virus 1	ELISPOT	IFNg release	Positive	1	1	[illegible]
195438	GYILRDISKIPGGNM	NS5	3008	3022	HLA-DRB1*08:02	100%	Dengue virus 1	ELISPOT	IFNg release	Positive	1	1	[illegible]
195464	HWFSRENSLSGVEGE	NS5	2988	3002	HLA-DRB1*04:01	100%	Dengue virus 1	ELISPOT	IFNg release	Positive	1	1	[illegible]
195655	LLATSIFKLTYQNKV	NS5	3055	3069	HLA-DRB1*07:01	100%	Dengue virus 1	ELISPOT	IFNg release	Positive	1	1	[illegible]
195783	NENYLDYMTSMKRFK	NS5	3368	3382	HLA-DRB1*11:01	100%	Dengue virus 1	ELISPOT	IFNg release	Positive	1	1	[illegible]
195786	NGTVMDVISRRDQRG	NS5	3078	3092	HLA-DRB1*03:01	100%	Dengue virus 1	ELISPOT	IFNg release	Positive	1	1	[illegible]
195824	NYLDYMTSMKRFKNE	NS5	3370	3384	HLA-DRB1*14:02	100%	Dengue virus 1	ELISPOT	IFNg release	Positive	1	1	[illegible]
195852	QLIMKDGREIVVPCR	NS5	3207	3221	HLA-DRB1*03:01	100%	Dengue virus 1	ELISPOT	IFNg release	Positive	1	1	[illegible]
195903	RPAKNGTVMDVISRR	NS5	3074	3088	HLA-DRB1*03:01	100%	Dengue virus 1	ELISPOT	IFNg release	Positive	1	1	[illegible]
195956	SIFKLTYQNKVVRVQ	NS5	3059	3073	HLA-DRB1*09:01	100%	Dengue virus 1	ELISPOT	IFNg release	Positive	1	1	[illegible]
196031	TCVYNMMGKREKKLG	NS5	2941	2955	HLA-DRB1*11:01	100%	Dengue virus 1	ELISPOT	IFNg release	Positive	1	1	[illegible]
196039	TGNIVSAVNMTSRML	NS5	2717	2731	HLA-DRB1*04:01	100%	Dengue virus 1	ELISPOT	IFNg release	Positive	1	1	[illegible]
196220	WLWGFLSRNKKPRIC	NS5	2872	2886	HLA-DRB1*11:01	100%	Dengue virus 1	ELISPOT	IFNg release	Positive	1	1	[illegible]
196241	YQNKVVRVQRPAKNG	NS5	3065	3079	HLA-DRB1*08:02	100%	Dengue virus 1	ELISPOT	IFNg release	Positive	1	1	[illegible]
539096	ATSIFKLTYQNKVVR	NS5	3057	3071	HLA-DRB1*14:04	100%	Dengue virus 1	ELISPOT	IFNg release	Positive	1	1	[illegible]
539350	GTAKLRWFVERNLVK	NS5	2551	2565	HLA-DRB1*09:01	100%	Dengue virus 1	ELISPOT	IFNg release	Positive	1	1	[illegible]
539905	SHHFHQLIMKDGREI	NS5	3202	3216	HLA-DRB1*13:01	100%	Dengue virus 1	ELISPOT	IFNg release	Positive	1	1	[illegible]
540001	TSIFKLTYQNKVVRV	NS5	3058	3072	HLA-DRB1*09:01	100%	Dengue virus 1	ELISPOT	IFNg release	Positive	1	1	[illegible]
540138	WQLMYFHRRDLRLAA	NS5	3254	3268	HLA-DRB1*14:04	100%	Dengue virus 1	ELISPOT	IFNg release	Positive	1	1	[illegible]
738814	ENYLDYMTSMKRFKN	NS5	3369	3383	HLA-DRB1*14:02	100%	Dengue virus 1	ELISPOT	IFNg release	Positive	1	1	[illegible]
740024	QNKVVRVQRPAKNGT	NS5	3066	3080	HLA-DRB1*14:06	100%	Dengue virus 1	ELISPOT	IFNg release	Positive	1	1	[illegible]
740786	YLDYMTSMKRFKNES	NS5	3371	3385	HLA-DRB1*14:02	100%	Dengue virus 1	ELISPOT	IFNg release	Positive	1	1	[illegible]
867505	KSYAQMWQLMYFHRR	NS5	3248	3262	HLA-DPB1*01:01	100%	Dengue virus 1	ELISPOT	IFNg release	Positive	1	1	[illegible]
868240	NKVVRVQRPAKNGTV	NS5	3067	3081	HLA-DRB4*01:01	100%	Dengue virus 1	ELISPOT	IFNg release	Positive	1	1	[illegible]
868296	PEHALLATSIFKLTY	NS5	3051	3065	HLA-DPB1*02:01	100%	Dengue virus 1	ELISPOT	IFNg release	Positive	1	1	[illegible]
868641	RFLEFEALGFMNEDH	NS5	2974	2988	HLA-DPB1*04:01	100%	Dengue virus 1	ELISPOT	IFNg release	Positive	1	1	[illegible]
869091	TAKLRWFVERNLVKP	NS5	2552	2566	HLA-DQA1*01:01/DQB1*05:01	100%	Dengue virus 1	ELISPOT	IFNg release	Positive	1	1	[illegible]
195117	AGILARWSSFKKNGA	C	51	65	HLA-DRB1*15:01	81% - <100%	Dengue virus 1	ELISPOT	IFNg release	Positive	1	1	[illegible]
195586	KRSVTMLLMLLPTAL	C	87	101	HLA-DRB1*01:01	81% - <100%	Dengue virus 1	ELISPOT	IFNg release	Positive	1	1	[illegible]
195761	MLNIMNRRKRSVTML	C	69	83	HLA-DRB1*08:02	81% - <100%	Dengue virus 1	ELISPOT	IFNg release	Positive	1	1	[illegible]
195885	RGFKKEISSMLNIMN	C	60	74	HLA-DRB1*04:01	81% - <100%	Dengue virus 1	ELISPOT	IFNg release	Positive	1	1	[illegible]
196196	VTMLLMLLPTALAFH	C	90	104	HLA-DRB1*04:01	81% - <100%	Dengue virus 1	ELISPOT	IFNg release	Positive	1	1	[illegible]
539258	FKKEISSMLNIMNRR	C	62	76	HLA-DRB1*10:01	81% - <100%	Dengue virus 1	ELISPOT	IFNg release	Positive	1	1	[illegible]
539259	FKKNGAIKVLRGFKK	C	50	64	HLA-DRB1*08:03	81% - <100%	Dengue virus 1	ELISPOT	IFNg release	Positive	1	1	[illegible]
539459	KEISSMLNIMNRRKR	C	64	78	HLA-DRB1*14:04	81% - <100%	Dengue virus 1	ELISPOT	IFNg release	Positive	1	1	[illegible]
539475	KKEISSMLNIMNRRK	C	63	77	HLA-DRB1*14:04	81% - <100%	Dengue virus 1	ELISPOT	IFNg release	Positive	1	1	[illegible]
539495	KNGAIKVLRGFKKEI	C	52	66	HLA-DRB1*12:02	81% - <100%	Dengue virus 1	ELISPOT	IFNg release	Positive	1	1	[illegible]
539613	LRFLAIPPTAGILAR	C	32	46	HLA-DRB1*04:03	81% - <100%	Dengue virus 1	ELISPOT	IFNg release	Positive	1	1	[illegible]
539617	LRGFKKEISSMLNIM	C	59	73	HLA-DRB1*13:01	81% - <100%	Dengue virus 1	ELISPOT	IFNg release	Positive	1	1	[illegible]
539711	NGAIKVLRGFKKEIS	C	53	67	HLA-DRB1*13:01	81% - <100%	Dengue virus 1	ELISPOT	IFNg release	Positive	1	1	[illegible]
539780	PMKLVMAFIAFLRFL	C	21	35	HLA-DRB1*13:01	81% - <100%	Dengue virus 1	ELISPOT	IFNg release	Positive	1	1	[illegible]
540059	VLRGFKKEISSMLNI	C	68	82	HLA-DRB1*04:07	81% - <100%	Dengue virus 1	ELISPOT	IFNg release	Positive	1	1	[illegible]
738927	FLRFLAIPPTAGILA	C	41	55	HLA-DRB1*14:02	81% - <100%	Dengue virus 1	ELISPOT	IFNg release	Positive	1	1	[illegible]
95107	KLVMAFIAFLRFL	C	45	57	HLA-DPw4	81% - <100%	Dengue virus 1	ELISPOT	IFNg release	Positive	1	1	[illegible]
195368	GIIFILLMLVTPSMA	PreM	266	280	HLA-DRB1*01:01	81% - <100%	Dengue virus 1	ELISPOT	IFNg release	Positive	1	1	[illegible]
195562	KGIIFILLMLVTPSM	PreM	265	279	HLA-DRB1*08:02	81% - <100%	Dengue virus 1	ELISPOT	IFNg release	Positive	1	1	[illegible]
195831	PGFTVIALFLAHAIG	PreM	245	259	HLA-DRB1*15:01	81% - <100%	Dengue virus 1	ELISPOT	IFNg release	Positive	1	1	[illegible]
195887	RGKSLLFKTSAGVNM	PreM	111	125	HLA-DRB1*07:01	81% - <100%	Dengue virus 1	ELISPOT	IFNg release	Positive	1	1	[illegible]
196085	TVIALFLAHAIGTSI	PreM	248	262	HLA-DRB1*07:01	81% - <100%	Dengue virus 1	ELISPOT	IFNg release	Positive	1	1	[illegible]
539516	KSLLFKTSAGVNMCT	PreM	123	137	HLA-DRB1*09:01	81% - <100%	Dengue virus 1	ELISPOT	IFNg release	Positive	1	1	[illegible]
867083	HPGFTVIALFLAHAI	PreM	244	258	HLA-DPB1*02:01	81% - <100%	Dengue virus 1	ELISPOT	IFNg release	Positive	1	1	[illegible]
867912	LRHPGFTVIALFLAH	PreM	242	256	HLA-DPB1*02:01	81% - <100%	Dengue virus 1	ELISPOT	IFNg release	Positive	1	1	[illegible]
195145	AKFKCVTKLEGKIVQ	E	117	131	HLA-DRB1*11:01	81% - <100%	Dengue virus 1	ELISPOT	IFNg release	Positive	1	1	[illegible]
195547	IVQYENLKYSVIVTV	E	124	138	HLA-DRB1*15:02	81% - <100%	Dengue virus 1	ELISPOT	IFNg release	Positive	1	1	[illegible]
196053	TLDIELLKTEVTNPA	E	35	49	HLA-DRB1*04:01	81% - <100%	Dengue virus 1	ELISPOT	IFNg release	Positive	1	1	[illegible]
539378	HAKKQEVVVLGSQEGAMH	E	239	256	HLA class II	81% - <100%	Dengue virus 1	ICS	IFNg release	Positive	1	1	[illegible]
540052	VLGSQEGAMHTALTGA	E	247	262	HLA class II	81% - <100%	Dengue virus 1	ICS	IFNg release	Positive	1	1	[illegible]
540104	VVVLGSQEGAMHTALTG	E	245	261	HLA class II	81% - <100%	Dengue virus 1	ICS	IFNg release	Positive	1	1	[illegible]
867101	HTALTGATEIQTSGT	E	541	555	HLA-DQA1*01:02/DQB1*06:02	81% - <100%	Dengue virus 1	ELISPOT	IFNg release	Positive	1	1	[illegible]
195579	KNETWKLARASFIEV	NS1	206	220	HLA-DRB1*07:01	81% - <100%	Dengue virus 1	ELISPOT	IFNg release	Positive	1	1	[illegible]
195784	NETWKLARASFIEVK	NS1	207	221	HLA-DRB1*07:01	81% - <100%	Dengue virus 1	ELISPOT	IFNg release	Positive	1	1	[illegible]
539401	IESEKNETWKLARASFI	NS1	202	218	HLA class II	81% - <100%	Dengue virus 1	ICS	IFNg release	Positive	1	1	[illegible]
738503	ANGILAQGKKMIRPQ	NS1	868	882	HLA-DRB1*14:06	81% - <100%	Dengue virus 1	ELISPOT	IFNg release	Positive	1	1	[illegible]
867405	KIIGADIQNTTFIID	NS1	897	911	HLA-DPB1*04:02	81% - <100%	Dengue virus 1	ELISPOT	IFNg release	Positive	1	1	[illegible]
195286	FAVGLLFRRLTSREV	NS2a	1214	1228	HLA-DRB1*08:03	81% - <100%	Dengue virus 1	ELISPOT	IFNg release	Positive	1	1	[illegible]
195391	GLLFRRLTSREVLLL	NS2a	1217	1231	HLA-DRB1*08:03	81% - <100%	Dengue virus 1	ELISPOT	IFNg release	Positive	1	1	[illegible]
195665	LLLTIGLSLVASVEL	NS2a	1229	1243	HLA-DRB1*07:01	81% - <100%	Dengue virus 1	ELISPOT	IFNg release	Positive	1	1	[illegible]
195674	LLTIGLSLVASVELP	NS2a	1230	1244	HLA-DRB1*07:01	81% - <100%	Dengue virus 1	ELISPOT	IFNg release	Positive	1	1	[illegible]
195983	SREVLLLTIGLSLVA	NS2a	1225	1239	HLA-DRB1*15:01	81% - <100%	Dengue virus 1	ELISPOT	IFNg release	Positive	1	1	[illegible]
539094	ATFKMRPMFAVGLLF	NS2a	1206	1220	HLA-DRB1*01:02	81% - <100%	Dengue virus 1	ELISPOT	IFNg release	Positive	1	1	[illegible]
539205	EEVMRSRWSRKMLMT	NS2a	1147	1161	HLA-DRB1*13:01	81% - <100%	Dengue virus 1	ELISPOT	IFNg release	Positive	1	1	[illegible]
539657	MALSIVSLFPLCLST	NS2a	1297	1311	HLA-DRB1*15:06	81% - <100%	Dengue virus 1	ELISPOT	IFNg release	Positive	1	1	[illegible]
539888	RWSRKMLMTGTLAVF	NS2a	1153	1167	HLA-DRB1*04:03	81% - <100%	Dengue virus 1	ELISPOT	IFNg release	Positive	1	1	[illegible]
540038	VGLLFRRLTSREVLL	NS2a	1216	1230	HLA-DRB1*08:03	81% - <100%	Dengue virus 1	ELISPOT	IFNg release	Positive	1	1	[illegible]
738553	AVGLLFRRLTSREVL	NS2a	1215	1229	HLA-DRB1*01:02	81% - <100%	Dengue virus 1	ELISPOT	IFNg release	Positive	1	1	[illegible]
866118	AMALSIVSLFPLCLS	NS2a	1296	1310	HLA-DPB1*04:01	81% - <100%	Dengue virus 1	ELISPOT	IFNg release	Positive	1	1	[illegible]
868100	MGQLTWNDLIRLCIM	NS2a	433	447	HLA-DPB1*04:02	81% - <100%	Dengue virus 1	ELISPOT	IFNg release	Positive	1	1	[illegible]
195228	DTLTILLKATLLAVS	NS2b	1438	1452	HLA-DRB1*08:02	81% - <100%	Dengue virus 1	ELISPOT	IFNg release	Positive	1	1	[illegible]
195508	ILVEVQDDGTMKIKD	NS2b	68	82	HLA-DRB1*03:01	81% - <100%	Dengue virus 1	ELISPOT	IFNg release	Positive	1	1	[illegible]
195715	LSSLLKNDVPLAGPL	NS2b	1363	1377	HLA-DRB1*03:01	81% - <100%	Dengue virus 1	ELISPOT	IFNg release	Positive	1	1	[illegible]
195724	LTILLKATLLAVSGV	NS2b	1440	1454	HLA-DRB1*08:02	81% - <100%	Dengue virus 1	ELISPOT	IFNg release	Positive	1	1	[illegible]
195878	RDDTLTILLKATLLA	NS2b	1436	1450	HLA-DRB1*12:02	81% - <100%	Dengue virus 1	ELISPOT	IFNg release	Positive	1	1	[illegible]
195993	SSLLKNDVPLAGPLI	NS2b	1364	1378	HLA-DRB1*03:01	81% - <100%	Dengue virus 1	ELISPOT	IFNg release	Positive	1	1	[illegible]
196111	VEVQDDGTMKIKDEE	NS2b	1421	1435	HLA-DRB1*03:01	81% - <100%	Dengue virus 1	ELISPOT	IFNg release	Positive	1	1	[illegible]
866201	ATLFVWYFWQKKKQR	NS2b	1461	1475	HLA-DPB1*01:01	81% - <100%	Dengue virus 1	ELISPOT	IFNg release	Positive	1	1	[illegible]
867544	LAGPLIAGGMLIACY	NS2b	1373	1387	HLA-DQA1*01:02/DQB1*06:02	81% - <100%	Dengue virus 1	ELISPOT	IFNg release	Positive	1	1	[illegible]
167826	TSGTYVSAIAQAKASQE	NS3	157	173	HLA class II	81% - <100%	Dengue virus 1	ELISPOT	IFNg release	Positive	1	1	[illegible]
195201	CHATFTMRLLSPVRV	NS3	1737	1751	HLA-DRB1*01:01	81% - <100%	Dengue virus 1	ELISPOT	IFNg release	Positive	1	1	[illegible]
195242	EGKIVGLYGNGVVTT	NS3	1618	1632	HLA-DRB1*15:01	81% - <100%	Dengue virus 1	ELISPOT	IFNg release	Positive	1	1	[illegible]
195338	FTMRLLSPVRVPNYN	NS3	1741	1755	HLA-DRB1*01:01	81% - <100%	Dengue virus 1	ELISPOT	IFNg release	Positive	1	1	[illegible]
195379	GKIVGLYGNGVVTTS	NS3	1619	1633	HLA-DRB1*15:01	81% - <100%	Dengue virus 1	ELISPOT	IFNg release	Positive	1	1	[illegible]
195585	KRKLRTLILAPTRVV	NS3	1689	1703	HLA-DRB1*10:01	81% - <100%	Dengue virus 1	ELISPOT	IFNg release	Positive	1	1	[illegible]
195705	LRTLILAPTRVVASE	NS3	1692	1706	HLA-DRB1*08:02	81% - <100%	Dengue virus 1	ELISPOT	IFNg release	Positive	1	1	[illegible]
195896	RKLRTLILAPTRVVA	NS3	1690	1704	HLA-DRB1*01:01	81% - <100%	Dengue virus 1	ELISPOT	IFNg release	Positive	1	1	[illegible]
45669	NREGKIVGLYGNGVV	NS3	141	155	HLA-DRB1*15:01	81% - <100%	Dengue virus 1	ICS	IFNg release	Positive	1	1	[illegible]
539485	KLRTLILAPTRVVAS	NS3	1691	1705	HLA-DRB1*04:03	81% - <100%	Dengue virus 1	ELISPOT	IFNg release	Positive	1	1	[illegible]
539579	LKGMPIRYQTTAVKS	NS3	1711	1725	HLA-DRB1*04:03	81% - <100%	Dengue virus 1	ELISPOT	IFNg release	Positive	1	1	[illegible]
539902	SGTYVSAIAQAKASQ	NS3	1633	1647	HLA-DRB1*10:01	81% - <100%	Dengue virus 1	ELISPOT	IFNg release	Positive	1	1	[illegible]
540040	VGLYGNGVVTTSGTY	NS3	1622	1636	HLA-DRB1*15:06	81% - <100%	Dengue virus 1	ELISPOT	IFNg release	Positive	1	1	[illegible]
540074	VREAIKRKLRTLILA	NS3	1684	1698	HLA-DRB1*12:02	81% - <100%	Dengue virus 1	ELISPOT	IFNg release	Positive	1	1	[illegible]
739269	IVGLYGNGVVTTSGT	NS3	132	146	HLA-DRB1*01:02	81% - <100%	Dengue virus 1	ELISPOT	IFNg release	Positive	1	1	[illegible]
866181	ASQEGPLPEIEDEVF	NS3	1645	1659	HLA-DQA1*05:01/DQB1*02:01	81% - <100%	Dengue virus 1	ELISPOT	IFNg release	Positive	1	1	[illegible]
866469	EAIKRKLRTLILAPT	NS3	1686	1700	HLA-DPB1*01:01	81% - <100%	Dengue virus 1	ELISPOT	IFNg release	Positive	1	1	[illegible]
867396	KGMPIRYQTTAVKSE	NS3	1712	1726	HLA-DRB3*02:02	81% - <100%	Dengue virus 1	ELISPOT	IFNg release	Positive	1	1	[illegible]
869341	TTSGTYVSAIAQAKA	NS3	1631	1645	HLA-DQA1*01:02/DQB1*06:02	81% - <100%	Dengue virus 1	ELISPOT	IFNg release	Positive	1	1	[illegible]
869696	WNTGEEVQVIAVEPG	NS3	1564	1578	HLA-DQA1*03:01/DQB1*03:02	81% - <100%	Dengue virus 1	ELISPOT	IFNg release	Positive	1	1	[illegible]

195478	IETLMLLALIAVLTG	NS4a	2146	2160	HLA-DRB1*15:01	81% - <100%	Denguevirus 1	ELISPOT	IFNg release	Positive	1	1	1	1	1	1	1	1	1	1	1	1	1	1	1	1	1	1	1	1	1	1	1	1	1	1	1	1	1	1	1	1	1	1	1	1	1	1	1	1	1	1	1	1	1	1	1	1	1	1	1	1	1	1	1	1	1	1	1	0	1	1	1	1	1	1	1	1	1	1	1	1	1	1	1	1	1	1	1	1	1	1	1	1	0	0	0
195679	LMLLALIAVLTGGVT	NS4a	2149	2163	HLA-DRB1*08:02	81% - <100%	Denguevirus 1	ELISPOT	IFNg release	Positive	1	1	1	1	1	1	1	1	1	1	1	1	1	1	1	1	1	1	1	1	1	1	1	1	1	1	1	1	1	1	1	1	1	1	1	1	1	1	1	1	1	1	1	1	1	1	1	1	1	0	1	1	1	1	1	1	1	1	1	1	1	1	1	1	1	1	1	1	1	1	1	1	1	1	0	0	0
196255	YVVIGLLFMILTVAA	NS4a	2230	2244	HLA-DRB1*11:01	81% - <100%	Denguevirus 1	ELISPOT	IFNg release	Positive	1	1	1	1	1	1	0	1	1	1	1	1	1	1	1	1	1	1	1	1	1	1	1	1	1	1	1	1	1	1	1	1	1	1	1	1	1	1	1	1	1	1	1	1	1	1	1	1	1	1	1	1	1	1	1	1	1	1	1	1	1	1	1	1	1	1	1	1	1	1	1	1	1	1	0	0	0
539383	HLTLRAQNALDNLVM	NS4a	2110	2124	HLA-DRB3*02:02	81% - <100%	Denguevirus 1	ELISPOT	IFNg release	Positive	1	1	1	1	1	1	1	1	1	1	1	1	1	1	1	1	1	1	1	1	1	1	1	1	1	0	1	1	1	1	0	1	0	1	1	1	1	1	1	0	1	1	0	1	1	0	1	1	1	1	1	1	1	1	1	1	1	0	0	1	1	1	1	1	1	1	0	0	1	1	1	1	0	1	0	1	1	1	1	0	0	0	0	0	0
539744	NQLAYVVIGLLFMIL	NS4a	2226	2240	HLA-DRB1*15:02	81% - <100%	Denguevirus 1	ELISPOT	IFNg release	Positive	1	1	1	1	1	1	0	1	1	1	1	1	1	1	1	1	1	1	1	1	1	1	1	1	1	1	1	1	1	1	1	1	1	1	1	1	1	1	1	1	1	1	1	1	1	1	1	1	1	1	1	1	1	1	1	1	1	1	1	1	1	1	1	1	1	1	1	1	1	1	1	1	1	1	0	0	0
738677	DLILEIGKLPQHLTL	NS4a	2099	2113	HLA-DRB1*11:04	81% - <100%	Denguevirus 1	ELISPOT	IFNg release	Positive	1	1	1	1	1	1	1	1	1	1	1	1	1	1	1	1	1	1	1	1	1	1	1	1	1	1	1	1	1	1	0	1	1	1	1	1	1	1	1	0	1	1	0	1	1	0	1	1	1	1	1	1	1	0	1	1	1	0	0	1	1	1	1	1	1	1	0	1	1	1	1	1	1	1	0	1	1	1	1	1	1	0	0	0	0	0	0
868132	MLLALIAVLTGGVTL	NS4a	2150	2164	HLA-DPB1*04:02	81% - <100%	Denguevirus 1	ELISPOT	IFNg release	Positive	1	1	1	1	1	1	1	1	1	1	1	1	1	1	1	1	1	1	1	1	1	1	1	1	1	1	1	1	1	1	1	1	1	1	1	1	1	1	1	1	1	1	1	1	1	1	1	1	1	0	1	1	1	1	1	1	1	1	1	1	1	1	1	1	1	1	1	1	1	1	1	1	1	1	0	0	0
195186	ATNIQVAINQVRRLI	NS5	3352	3366	HLA-DRB1*03:01	81% - <100%	Denguevirus 1	ELISPOT	IFNg release	Positive	1	1	1	1	1	1	1	1	1	1	1	1	1	1	1	1	1	1	0	1	1	1	1	1	1	1	1	1	1	1	1	1	1	1	1	1	1	1	1	1	1	1	1	1	1	1	1	1	1	1	1	1	1	1	1	1	1	1	1	1	1	1	1	1	1	1	1	1	1	1	1	1	1	1	0	0	0
195212	DDRFATALTALNDMG	NS5	3163	3177	HLA-DRB1*04:01	81% - <100%	Denguevirus 1	ELISPOT	IFNg release	Positive	1	1	1	1	1	1	1	1	1	1	1	1	1	1	1	1	1	1	1	1	1	1	1	1	1	1	1	1	1	1	1	1	1	1	1	1	1	1	1	1	1	1	1	1	1	1	1	1	1	1	1	1	1	1	1	1	0	1	1	1	1	1	1	1	0	1	1	1	1	1	1	1	1	1	0	0	0
195223	DMGKVRKDIPQWEPS	NS5	3175	3189	HLA-DRB1*03:01	81% - <100%	Denguevirus 1	ELISPOT	IFNg release	Positive	1	1	1	1	1	1	1	1	1	1	1	1	1	1	1	1	1	1	1	1	1	1	1	1	1	1	1	1	1	1	1	1	1	1	1	1	1	1	1	0	1	1	1	1	1	1	1	1	1	1	1	1	1	1	1	1	1	1	1	1	1	1	1	1	1	1	1	1	1	1	1	1	1	1	0	0	0
195236	EEFTRKVRSNAAIGA	NS5	2889	2903	HLA-DRB1*08:02	81% - <100%	Denguevirus 1	ELISPOT	IFNg release	Positive	1	1	1	1	1	1	1	1	1	1	1	1	1	1	1	1	1	1	1	1	1	1	1	1	1	1	1	1	1	1	1	1	1	1	1	1	1	1	1	1	1	1	1	1	1	1	1	1	1	1	1	1	1	1	1	1	1	1	1	1	0	1	1	1	1	1	1	1	1	1	1	1	1	1	0	0	0
195354	GEKWKRQLNQLSKSE	NS5	2503	2517	HLA-DRB1*04:01	81% - <100%	Denguevirus 1	ELISPOT	IFNg release	Positive	1	1	1	1	1	1	1	1	1	1	1	1	1	1	1	1	1	1	1	1	1	1	1	1	1	1	1	1	1	1	1	1	1	1	1	1	1	1	0	1	1	1	1	1	1	1	1	1	1	1	1	0	1	1	1	1	1	1	1	1	1	1	1	1	1	1	0	1	1	1	1	1	1	1	0	1	1	1	1	1	1	1	1	1	0	0	0
195582	KPIDDRFATALTALN	NS5	3160	3174	HLA-DRB1*04:01	81% - <100%	Denguevirus 1	ELISPOT	IFNg release	Positive	1	1	1	1	1	1	1	1	1	1	1	1	1	1	1	1	1	1	1	1	1	1	1	1	1	1	1	1	1	1	1	1	1	1	1	1	1	1	1	1	1	1	1	1	1	1	1	1	1	1	1	1	1	1	1	1	1	1	1	1	1	1	1	1	1	1	1	1	1	1	1	1	1	1	0	0	0
195755	MGKVRKDIPQWEPSK	NS5	3176	3190	HLA-DRB1*03:01	81% - <100%	Denguevirus 1	ELISPOT	IFNg release	Positive	1	1	1	1	1	1	1	1	1	1	1	1	1	1	1	1	1	1	1	1	1	1	1	1	1	1	1	1	1	1	1	1	1	1	1	1	1	1	1	0	1	1	1	1	1	1	1	1	1	1	1	1	1	1	1	1	1	1	1	1	1	1	1	1	1	1	1	1	1	1	1	1	1	1	0	0	0
196211	WATNIQVAINQVRRL	NS5	3351	3365	HLA-DRB1*03:01	81% - <100%	Denguevirus 1	ELISPOT	IFNg release	Positive	1	1	1	1	1	1	1	1	1	1	1	1	1	1	1	1	1	1	0	1	1	1	1	1	1	1	1	1	1	1	1	1	1	1	1	1	1	1	1	1	1	1	1	1	1	1	1	1	1	1	1	1	1	1	1	1	1	1	1	1	1	1	1	1	1	1	1	1	1	1	1	1	1	1	0	0	0
539107	AVNMTSRMLLNRFTM	NS5	2723	2737	HLA-DRB1*15:02	81% - <100%	Denguevirus 1	ELISPOT	IFNg release	Positive	1	1	1	1	1	1	1	1	1	1	1	1	1	1	1	1	1	1	1	1	1	1	1	1	1	1	1	1	1	1	1	1	1	1	1	1	1	1	1	0	1	1	1	1	1	1	1	1	1	1	1	1	1	1	1	1	1	1	1	1	1	1	1	1	1	1	0	1	1	1	1	1	1	1	0	1	1	1	1	1	1	1	1	1	0	0	0
539396	IDDRFATALTALNDM	NS5	3162	3176	HLA-DPB1*01:01	81% - <100%	Denguevirus 1	ELISPOT	IFNg release	Positive	1	1	1	1	1	1	1	1	1	1	1	1	1	1	1	1	1	1	1	1	1	1	1	1	1	1	1	1	1	1	1	1	1	1	1	1	1	1	1	1	1	1	1	1	1	1	1	1	1	1	1	1	1	1	1	1	0	1	1	1	1	1	1	1	0	1	1	1	1	1	1	1	1	1	0	0	0
539435	ISGDDCVVKPIDDRFAT	NS5	3152	3168	HLA class II	81% - <100%	Denguevirus 1	ICS	IFNg release	Positive	1	1	1	1	1	1	1	1	1	1	1	1	1	1	1	1	1	1	1	1	1	1	1	1	1	1	1	1	1	1	1	1	1	1	1	1	1	1	1	1	1	1	1	1	1	1	1	1	1	1	1	1	1	1	1	1	1	1	1	1	1	1	1	1	1	1	1	1	1	1	1	1	1	1	0	0	0
539691	MTSRMLLNRFTMAHR	NS5	17	31	HLA-DRB1*14:06	81% - <100%	Denguevirus 1	ELISPOT	IFNg release	Positive	1	1	1	1	1	1	1	1	1	1	1	1	1	1	1	1	1	1	1	1	1	1	1	1	1	1	1	1	1	1	1	1	1	1	1	1	1	1	1	0	1	1	1	1	1	1	1	1	1	1	1	1	1	1	1	1	1	1	1	1	1	1	1	1	1	1	1	1	1	1	1	1	1	1	0	0	0
539700	MVNGVVRLLTKPWDV	NS5	103	117	HLA-DRB1*12:02	81% - <100%	Denguevirus 1	ELISPOT	IFNg release	Positive	1	1	1	1	1	1	1	1	1	1	1	1	1	1	1	1	1	1	1	1	1	1	1	1	1	1	1	1	1	1	1	1	1	1	1	1	1	1	1	1	1	1	1	1	1	1	1	1	1	0	1	1	1	1	1	1	1	1	1	1	1	1	1	1	1	1	1	1	1	1	1	1	1	1	0	0	0
539702	MVTQIAMTDTTPFGQQR	NS5	2829	2845	HLA class II	81% - <100%	Denguevirus 1	ICS	IFNg release	Positive	1	1	1	1	1	1	0	1	1	1	1	1	1	1	1	1	1	1	1	1	1	1	1	1	1	1	1	1	1	1	1	1	1	1	1	1	1	1	1	1	1	1	1	1	1	1	1	1	1	1	1	1	1	1	1	1	1	1	1	1	1	1	1	1	1	1	1	1	1	1	1	1	1	1	0	0	0
539862	RMLLNRFTMAHRKPT	NS5	20	34	HLA-DRB1*14:02	81% - <100%	Denguevirus 1	ELISPOT	IFNg release	Positive	1	1	1	1	1	1	1	1	1	1	1	1	1	1	1	1	1	1	1	1	1	1	1	1	1	1	1	1	1	1	1	1	1	1	1	1	1	1	1	0	1	1	1	1	1	1	1	1	1	1	1	1	1	1	1	1	1	1	1	1	1	1	1	1	1	1	1	1	1	1	1	1	1	1	1	1	1	1	1	1	1	0	0	0	0	0	0
540020	TYGLNTFTNMEVQLI	NS5	3098	3112	HLA-DRB1*04:03	81% - <100%	Denguevirus 1	ELISPOT	IFNg release	Positive	1	1	1	1	1	1	1	1	1	1	1	1	1	1	1	1	1	1	1	0	1	1	1	1	1	1	1	1	1	1	1	1	1	1	1	1	1	1	1	1	1	1	1	1	1	1	1	1	1	1	1	1	1	1	1	1	1	1	1	1	1	1	1	1	1	1	0	1	1	1	1	1	1	1	0	1	1	1	1	1	1	1	1	1	0	0	0
540100	VVKPIDDRFATALTALN	NS5	3157	3173	HLA class II	81% - <100%	Denguevirus 1	ICS	IFNg release	Positive	1	1	1	1	1	1	1	1	1	1	1	1	1	1	1	1	1	1	1	1	1	1	1	1	1	1	1	1	1	1	1	1	1	1	1	1	1	1	1	1	1	1	1	1	1	1	1	1	1	1	1	1	1	1	1	1	1	1	1	1	1	1	1	1	1	1	1	1	1	1	1	1	0	1	1	1	1	1	1	1	1	0	0	0	0	0	0
740668	VQLIRQMESEGIFLP	NS5	3109	3123	HLA-DRB1*11:04	81% - <100%	Denguevirus 1	ELISPOT	IFNg release	Positive	1	1	1	1	1	1	1	1	1	1	1	1	1	1	1	1	1	1	1	1	1	1	0	1	1	1	1	1	1	1	1	1	1	1	1	1	1	1	1	1	1	1	1	1	1	1	1	1	1	1	1	1	1	1	1	1	1	1	1	1	1	1	1	1	1	1	1	1	1	1	1	1	1	1	1	1	1	1	1	1	1	1	1	1	0	0	0
866481	EAVEDERFWDLVHRE	NS5	2916	2930	HLA-DQA1*01:01/DQB1*05:01	81% - <100%	Denguevirus 1	ELISPOT	IFNg release	Positive	1	1	1	1	1	1	0	1	1	1	1	1	1	1	1	1	1	1	1	0	1	1	1	1	1	1	1	1	1	1	1	1	1	1	1	1	1	1	1	1	1	1	1	1	1	1	1	1	1	1	1	1	1	1	1	1	1	1	1	1	1	1	1	1	1	1	1	1	1	1	1	1	1	1	1	1	1	1	1	1	1	1	1	1	0	0	0
195904	RPSFNMLKRARNRVS	C	10	24	HLA-DRB1*11:01	51%-80%	Denguevirus 1	ELISPOT	IFNg release	Positive	1	1	1	0	0	1	1	1	1	1	1	1	1	0	0	1	0	1	0	1	1	0	1	1	1	0	1	1	1	0	1	1	0	0	1	1	1	1	1	1	0	1	1	1	1	1	1	1	1	0	1	1	0	1	1	1	1	1	1	1	0	0	1	1	1	1	1	0	1	1	1	0	1	1	1	0	1	1	0	1	0	1	1	1	0	0	0
196043	TGRPSFNMLKRARNR	C	8	22	HLA-DRB1*11:01	51%-80%	Denguevirus 1	ELISPOT	IFNg release	Positive	1	1	1	0	0	1	1	1	1	1	1	1	1	0	0	1	0	1	0	1	1	0	1	1	1	0	1	1	1	0	1	1	0	0	1	1	1	1	1	1	0	1	1	1	1	1	1	1	1	0	1	1	0	1	1	1	1	1	1	1	0	0	1	1	1	1	1	0	1	1	1	0	1	1	1	0	1	1	0	1	0	1	1	1	0	0	0
739424	LAVFLLLVMGQLTWN	NS2a	1164	1178	HLA-DRB1*11:04	51%-80%	Denguevirus 1	ELISPOT	IFNg release	Positive	1	1	1	0	0	1	1	1	1	1	1	1	0	0	0	1	0	1	0	1	1	0	1	0	1	0	1	1	1	0	1	0	0	0	1	1	1	1	0	1	0	0	1	1	1	1	0	1	1	0	1	0	0	1	0	1	1	1	1	1	0	0	1	1	1	1	1	0	1	0	1	0	1	1	1	0	0	1	0	1	0	0	0	0	0	0	0
195143	AIVREAIKRKLRTLI	NS3	1682	1696	HLA-DRB1*11:01	51%-80%	Denguevirus 1	ELISPOT	IFNg release	Positive	0	0	1	1	1	1	0	0	1	1	0	1	1	1	1	1	1	0	1	0	1	1	1	1	1	1	1	0	1	1	1	1	1	1	1	1	1	1	1	1	1	1	1	1	0	0	1	1	0	1	1	1	1	0	1	0	0	1	1	0	1	1	0	1	0	1	1	1	1	1	1	1	1	1	1	1	1	1	1	1	1	0	0	0	0	0	0
739540	LPAIVREAIKRKLRT	NS3	1680	1694	HLA-DRB1*14:02	51%-80%	Denguevirus 1	ELISPOT	IFNg release	Positive	0	0	1	1	1	1	0	0	1	1	0	1	1	1	1	1	1	0	1	0	1	1	1	1	1	1	1	0	1	1	1	1	1	1	1	1	1	1	1	1	1	1	1	1	0	0	1	1	0	1	1	1	1	0	1	0	0	1	1	0	1	1	0	1	0	1	1	1	1	1	1	1	1	1	1	1	1	1	1	1	1	1	1	1	0	0	0
539816	QPSFNMLKRARNRVS	C	10	24	HLA-DRB1*08:03	>0% - 50%	Denguevirus 1	ELISPOT	IFNg release	Positive	0	0	0	1	1	0	0	0	0	0	0	0	0	1	1	0	1	0	1	0	0	1	0	0	0	1	0	0	0	1	0	0	1	1	0	0	0	0	0	0	1	0	0	0	0	0	0	0	0	1	0	0	1	0	0	0	0	0	0	0	1	1	0	0	0	0	0	1	0	0	0	1	0	0	0	1	0	0	1	0	1	0	0	0	0	0	0
195216	DIELLKTEVTNPAVL	E	37	51	HLA-DRB1*04:01	>0% - 50%	Denguevirus 1	ELISPOT	IFNg release	Positive	0	0	0	0	0	0	0	0	0	0	0	0	0	0	0	0	0	0	0	0	0	0	0	0	0	0	0	0	0	0	0	0	0	0	0	0	0	0	1	0	0	0	0	0	0	1	0	0	0	0	0	1	0	0	1	0	0	0	0	0	0	0	0	0	0	0	0	0	0	0	0	0	0	0	0	0	0	0	0	0	0	1	1	1	1	1	1
195477	IELLKTEVTNPAVLR	E	38	52	HLA-DRB1*04:01	>0% - 50%	Denguevirus 1	ELISPOT	IFNg release	Positive	0	0	0	0	0	0	0	0	0	0	0	0	0	0	0	0	0	0	0	0	0	0	0	0	0	0	0	0	0	0	0	0	0	0	0	0	0	0	1	0	0	0	0	0	0	1	0	0	0	0	0	1	0	0	1	0	0	0	0	0	0	0	0	0	0	0	0	0	0	0	0	0	0	0	0	0	0	0	0	0	0	1	1	1	1	1	1
195623	LDIELLKTEVTNPAV	E	36	50	HLA-DRB1*04:01	>0% - 50%	Denguevirus 1	ELISPOT	IFNg release	Positive	0	0	0	0	0	0	0	0	0	0	0	0	0	0	0	0	0	0	0	0	0	0	0	0	0	0	0	0	0	0	0	0	0	0	0	0	0	0	1	0	0	0	0	0	0	1	0	0	0	0	0	1	0	0	1	0	0	0	0	0	0	0	0	0	0	0	0	0	0	0	0	0	0	0	0	0	0	0	0	0	0	1	1	1	1	1	1
866284	DDQRAWNIWEVEDYG	NS1	181	195	HLA-DQA1*01:01/DQB1*05:01	>0% - 50%	Denguevirus 1	ELISPOT	IFNg release	Positive	0	0	0	0	0	0	0	0	0	0	0	0	0	0	0	0	0	0	0	0	0	0	0	0	0	0	0	0	0	0	0	0	0	0	0	0	0	0	1	0	0	0	0	0	0	0	0	0	0	0	0	1	0	0	0	0	0	0	0	0	0	0	0	0	0	0	0	0	0	0	0	0	0	0	0	0	0	0	0	0	0	0	0	0	0	0	0
195162	AMVLSIVSLFPLCLS	NS2a	1296	1310	HLA-DRB1*15:01	>0% - 50%	Denguevirus 1	ELISPOT	IFNg release	Positive	0	0	0	0	0	0	0	0	0	0	0	0	0	1	0	0	0	0	0	0	0	0	0	0	0	0	0	0	0	0	0	0	0	0	0	0	0	0	0	0	0	0	0	0	0	0	0	0	0	0	0	0	0	0	0	0	0	0	0	0	0	0	0	0	0	0	0	0	0	0	0	0	0	0	0	0	0	0	0	0	1	0	0
195422	GTLAVFLLLIMGQLT	NS2a	1162	1176	HLA-DRB1*01:01	>0% - 50%	Denguevirus 1	ELISPOT	IFNg release	Positive	0	0	0	1	1	0	0	0	0	0	0	0	1	1	1	0	1	0	1	0	0	1	0	1	0	1	0	0	0	1	0	1	1	1	0	0	0	0	1	0	1	1	0	0	0	0	1	0	0	1	0	1	1	0	1	0	0	0	0	0	1	1	0	0	0	0	0	1	0	1	0	1	0	0	0	1	1	0	1	0	1	0	0	0	0	0	0
195613	LAVFLLLIMGQLTWN	NS2a	1164	1178	HLA-DRB1*15:06	>0% - 50%	Denguevirus 1	ELISPOT	IFNg release	Positive	0	0	0	1	1	0	0	0	0	0	0	0	0	1	1	0	1	0	1	0	0	1	0	0	0	1	0	0	0	1	0	1	1	1	0	0	0	0	1	0	1	1	0	0	0	0	0	0	0	1	0	1	1	0	0	0	0	0	0	0	1	1	0	0	0	0	0	1	0	0	0	1	0	0	0	1	1	0	1	0	1	0	0	0	0	0	0
539679	MLMTGTLAVFLLLIM	NS2a	1158	1172	HLA-DRB1*15:06	>0% - 50%	Denguevirus 1	ELISPOT	IFNg release	Positive	0	0	0	1	1	0	0	0	0	0	0	0	1	1	1	0	1	0	1	0	0	1	0	1	0	1	0	0	0	1	0	1	0	1	0	0	0	0	1	0	1	1	0	0	0	0	1	0	0	1	0	1	1	0	1	0	0	0	0	0	1	1	0	0	0	0	0	1	0	1	0	1	0	0	0	1	0	0	1	0	1	0	0	0	0	0	0
866200	ATLFLWYFWQKKKQR	NS2b	1461	1475	HLA-DPB1*01:01	>0% - 50%	Denguevirus 1	ELISPOT	IFNg release	Positive	0	0	0	0	0	0	0	0	0	0	0	0	0	0	0	0	0	0	0	0	0	0	0	0	0	0	0	0	0	0	0	0	0	0	0	0	0	0	0	1	0	0	0	0	0	0	0	0	0	1	0	0	1	0	0	0	0	0	1	0	0	0	0	0	0	0	0	0	0	0	0	0	0	0	0	0	0	0	0	0	0	0	0	0	0	0	0
540071	VREAIKKKLRTLILA	NS3	1684	1698	HLA-DRB1*12:02	>0% - 50%	Denguevirus 1	ELISPOT	IFNg release	Positive	0	0	0	0	0	0	0	0	0	0	0	0	0	0	0	0	0	0	0	1	0	0	0	0	0	0	0	0	0	0	0	0	0	0	0	0	0	0	0	0	0	0	0	0	0	0	0	0	0	0	0	0	0	0	0	0	0	0	0	0	0	0	0	0	0	0	0	0	0	0	0	0	0	0	0	0	0	0	0	0	0	0	0	0	0	0	0
539509	KSEFNIYKRSGIMEV	NS5	2515	2529	HLA-DRB1*15:02	>0% - 50%	Denguevirus 1	ELISPOT	IFNg release	Positive	0	0	0	0	0	0	0	0	0	0	0	0	0	0	0	0	0	0	0	0	0	0	0	0	0	0	0	0	0	0	0	0	0	0	0	0	1	0	0	0	0	0	0	0	0	0	0	0	0	0	0	0	0	0	0	0	0	0	0	0	0	0	0	0	0	0	0	0	0	0	0	0	0	0	0	0	0	0	0	0	0	0	0

CD4 DENV-2 Epitopes

											NCBI IDs of DENV-2 circulating strains from Mexico, (100% identity=1,green ; else 0, red)																				DENV-2 constructs of vaccines					
Epitope_ID	AA_Seq	Protein_Name	Start	End	Allele	step	Serotype	Method/Technique	Assay Group	Qualitative Measure	ACW82956.1	ACQ44478.1	ACY70846.1	ADA60786.1	ADA60770.1	AET43253.1	AHI43753.1	AHI43694.1	AHI43691.1	AHI43692.1	QBQ87236.1	QBK56834.1	QBQ87238.1	ACW82937.1	ACW82955.1	ACS32035.1	QBK56835.1	QBK56836.1	QBQ87237.1	QNG98968.1	TV003	TDEN	DPIV	Dengvaxia	DENVax	TVDV
150404	LRLFMALVAFLRFLT	C	44	58	HLA-DRB1*11:01	0%	Dengue virus 2	ELISPOT	IFNg release	Positive	0	0	0	0	0	0	0	0	0	0	0	0	0	0	0	0	0	0	0	0	0	0	0	0	0	0
195121	AGMIIMLIPTVVAFH	C	102	116	HLA-DRB1*08:02	0%	Dengue virus 2	ELISPOT	IFNg release	Positive	0	0	0	0	0	0	0	0	0	0	0	0	0	0	0	0	0	0	0	0	0	0	0	0	0	0
195369	GIIIMIIPTVMAFHL	C	103	117	HLA-DRB1*08:02	0%	Dengue virus 2	ELISPOT	IFNg release	Positive	0	0	0	0	0	0	0	0	0	0	0	0	0	0	0	0	0	0	0	0	0	0	0	0	0	0
195413	GPLRLFMALVAFLRF	C	42	56	HLA-DRB1*04:01	0%	Dengue virus 2	ELISPOT	IFNg release	Positive	0	0	0	0	0	0	0	0	0	0	0	0	0	0	0	0	0	0	0	0	0	0	0	0	0	0
195432	GVIIMMIPTVMAFHL	C	103	117	HLA-DRB1*08:02	0%	Dengue virus 2	ELISPOT	IFNg release	Positive	0	0	0	0	0	0	0	0	0	0	0	0	0	0	0	0	0	0	0	0	0	0	0	0	0	0
195490	IGRMLNILSRRRRTA	C	88	102	HLA-DRB1*11:01	0%	Dengue virus 2	ELISPOT	IFNg release	Positive	0	0	0	0	0	0	0	0	0	0	0	0	0	0	0	0	0	0	0	0	0	0	0	0	0	0
195654	LKLYMALVAFLRFLT	C	44	58	HLA-DRB1*15:01	0%	Dengue virus 2	ELISPOT	IFNg release	Positive	0	0	0	0	0	0	0	0	0	0	0	0	0	0	0	0	0	0	0	0	0	0	0	0	0	0
195910	RTAGVIIMMIPTVVA	C	100	114	HLA-DRB1*08:02	0%	Dengue virus 2	ELISPOT	IFNg release	Positive	0	0	0	0	0	0	0	0	0	0	0	0	0	0	0	0	0	0	0	0	0	0	0	0	0	0
196084	TVGVIIMLIPTAMAF	C	101	115	HLA-DRB1*08:02	0%	Dengue virus 2	ELISPOT	IFNg release	Positive	0	0	0	0	0	0	0	0	0	0	0	0	0	0	0	0	0	0	0	0	0	0	0	0	0	0
53867	RGFRKEIGRMLNILNRRRS	C	17	35	HLA class II	0%	Dengue virus 2	ELISPOT	IFNg release	Positive	0	0	0	0	0	0	0	0	0	0	0	0	0	0	0	0	0	0	0	0	0	0	0	0	0	0
539046	AFLRFLTIPPTVGIL	C	52	66	HLA-DRB1*10:01	0%	Dengue virus 2	ELISPOT	IFNg release	Positive	0	0	0	0	0	0	0	0	0	0	0	0	0	0	0	0	0	0	0	0	0	0	0	0	0	0
539213	EIGRMLNILNKRRRT	C	87	101	HLA-DRB1*13:01	0%	Dengue virus 2	ELISPOT	IFNg release	Positive	0	0	0	0	0	0	0	0	0	0	0	0	0	0	0	0	0	0	0	0	0	0	0	0	0	0
539343	GRMLNIMNRRRRTAG	C	89	103	HLA-DRB1*13:01	0%	Dengue virus 2	ELISPOT	IFNg release	Positive	0	0	0	0	0	0	0	0	0	0	0	0	0	0	0	0	0	0	0	0	0	0	0	0	0	0
539549	LFTALVAFLRFLTIP	C	46	60	HLA-DRB1*15:06	0%	Dengue virus 2	ELISPOT	IFNg release	Positive	0	0	0	0	0	0	0	0	0	0	0	0	0	0	0	0	0	0	0	0	0	0	0	0	0	0
62853	TAGILKRWGTIKKSKANVL	C	62	81	HLA class II	0%	Dengue virus 2	ELISPOT	IFNg release	Positive	0	0	0	0	0	0	0	0	0	0	0	0	0	0	0	0	0	0	0	0	0	0	0	0	0	0
739176	IGRMLNILNKRRRTA	C	88	102	HLA-DRB1*14:02	0%	Dengue virus 2	ELISPOT	IFNg release	Positive	0	0	0	0	0	0	0	0	0	0	0	0	0	0	0	0	0	0	0	0	0	0	0	0	0	0
739177	IGRMLNILNRRRRSA	C	88	102	HLA-DRB1*14:02	0%	Dengue virus 2	ELISPOT	IFNg release	Positive	0	0	0	0	0	0	0	0	0	0	0	0	0	0	0	0	0	0	0	0	0	1	0	0	1	0
739179	IGRMLNILNRRRRSV	C	88	102	HLA-DRB1*14:02	0%	Dengue virus 2	ELISPOT	IFNg release	Positive	0	0	0	0	0	0	0	0	0	0	0	0	0	0	0	0	0	0	0	0	0	0	0	0	0	0
739180	IGRMLNILNRRRRTT	C	88	102	HLA-DRB1*14:02	0%	Dengue virus 2	ELISPOT	IFNg release	Positive	0	0	0	0	0	0	0	0	0	0	0	0	0	0	0	0	0	0	0	0	0	0	0	0	0	0
739181	IGRMLNILNRRRRTV	C	42	56	HLA-DRB1*14:02	0%	Dengue virus 2	ELISPOT	IFNg release	Positive	0	0	0	0	0	0	0	0	0	0	0	0	0	0	0	0	0	0	0	0	0	0	0	0	0	0
739182	IGRMLNIMNRRRRTA	C	88	102	HLA-DRB1*14:02	0%	Dengue virus 2	ELISPOT	IFNg release	Positive	0	0	0	0	0	0	0	0	0	0	0	0	0	0	0	0	0	0	0	0	0	0	0	0	0	0
739183	IGRMMNILNRRRRTA	C	88	102	HLA-DRB1*14:02	0%	Dengue virus 2	ELISPOT	IFNg release	Positive	0	0	0	0	0	0	0	0	0	0	0	0	0	0	0	0	0	0	0	0	0	0	0	0	0	0
740579	VAFLRFLTISPTAGI	C	51	65	HLA-DRB1*04:07	0%	Dengue virus 2	ELISPOT	IFNg release	Positive	0	0	0	0	0	0	0	0	0	0	0	0	0	0	0	0	0	0	0	0	0	0	0	0	0	0
866992	GPLKLYMALVAFLRF	C	42	56	HLA-DRB5*01:01	0%	Dengue virus 2	ELISPOT	IFNg release	Positive	0	0	0	0	0	0	0	0	0	0	0	0	0	0	0	0	0	0	0	0	0	0	0	0	0	0
867734	LKGFRKEIGRMLNIL	C	81	95	HLA-DRB3*02:02	0%	Dengue virus 2	ELISPOT	IFNg release	Positive	0	0	0	0	0	0	0	0	0	0	0	0	0	0	0	0	0	0	0	0	0	0	0	0	0	0
867910	LRGFRKEIGRMLNIM	C	81	95	HLA-DRB3*02:02	0%	Dengue virus 2	ELISPOT	IFNg release	Positive	0	0	0	0	0	0	0	0	0	0	0	0	0	0	0	0	0	0	0	0	0	0	0	0	0	0
867911	LRGFRREIGRMLNIL	C	81	95	HLA-DRB3*02:02	0%	Dengue virus 2	ELISPOT	IFNg release	Positive	0	0	0	0	0	0	0	0	0	0	0	0	0	0	0	0	0	0	0	0	0	0	0	0	0	0
868674	RLFMALVAFLRFLTI	C	45	59	HLA-DPB1*04:02	0%	Dengue virus 2	ELISPOT	IFNg release	Positive	0	0	0	0	0	0	0	0	0	0	0	0	0	0	0	0	0	0	0	0	0	0	0	0	0	0
150280	HPGFTLMAAILAYTI	PreM	244	258	HLA-DRB1*01:01	0%	Dengue virus 2	ELISPOT	IFNg release	Positive	0	0	0	0	0	0	0	0	0	0	0	0	0	0	0	0	0	0	0	0	0	0	0	0	0	0
180708	QRIETWILRHPGFTM	PreM	121	135	HLA class II	0%	Dengue virus 2	ELISPOT	IFNg release	Positive-Low	0	0	0	0	0	0	0	0	0	0	0	0	0	0	0	0	0	0	0	0	0	0	0	0	1	0
195318	FQKILIFILLTAVAP	PreM	263	277	HLA-DRB1*07:01	0%	Dengue virus 2	ELISPOT	IFNg release	Positive	0	0	0	0	0	0	0	0	0	0	0	0	0	0	0	0	0	0	0	0	0	0	0	0	0	0
195320	FQRTLIFILLTAVAP	PreM	263	277	HLA-DRB1*07:01	0%	Dengue virus 2	ELISPOT	IFNg release	Positive	0	0	0	0	0	0	0	0	0	0	0	0	0	0	0	0	0	0	0	0	0	0	0	0	0	0
195322	FQRVLIFILLTAVAP	PreM	263	277	HLA-DRB1*07:01	0%	Dengue virus 2	ELISPOT	IFNg release	Positive	0	0	0	0	0	0	0	0	0	0	0	0	0	0	0	0	0	0	0	0	0	0	0	0	0	0
195504	ILRHPGFTLMAAILA	PreM	241	255	HLA-DRB1*15:01	0%	Dengue virus 2	ELISPOT	IFNg release	Positive	0	0	0	0	0	0	0	0	0	0	0	0	0	0	0	0	0	0	0	0	0	0	0	0	0	0
195863	QRVLIFILLTAVAPS	PreM	264	278	HLA-DRB1*08:02	0%	Dengue virus 2	ELISPOT	IFNg release	Positive	0	0	0	0	0	0	0	0	0	0	0	0	0	0	0	0	0	0	0	0	0	0	0	0	0	0
196141	VLIFILLTAIAPSMT	PreM	266	280	HLA-DRB1*08:02	0%	Dengue virus 2	ELISPOT	IFNg release	Positive	0	0	0	0	0	0	0	0	0	0	0	0	0	0	0	0	0	0	0	0	0	0	0	0	0	0
196232	YFQRVLIFILLTAVT	PreM	262	276	HLA-DRB1*07:01	0%	Dengue virus 2	ELISPOT	IFNg release	Positive	0	0	0	0	0	0	0	0	0	0	0	0	0	0	0	0	0	0	0	0	0	0	0	0	0	0
539881	RVLIFILLTAIAPSM	PreM	265	279	HLA-DRB1*15:06	0%	Dengue virus 2	ELISPOT	IFNg release	Positive	0	0	0	0	0	0	0	0	0	0	0	0	0	0	0	0	0	0	0	0	0	0	0	0	0	0
868650	RHPGFTLMAAILAYT	PreM	243	257	HLA-DQA1*01:02/DQB1*06:02	0%	Dengue virus 2	ELISPOT	IFNg release	Positive	0	0	0	0	0	0	0	0	0	0	0	0	0	0	0	0	0	0	0	0	0	0	0	0	0	0
195793	NLEYTIMITPHSGEE	E	134	148	HLA-DRB1*04:01	0%	Dengue virus 2	ELISPOT	IFNg release	Positive	0	0	0	0	0	0	0	0	0	0	0	0	0	0	0	0	0	0	0	0	0	0	0	0	0	0
539427	IQKKTLVTFKNPHAK	E	512	526	HLA-DRB1*04:03	0%	Dengue virus 2	ELISPOT	IFNg release	Positive	0	0	0	0	0	0	0	0	0	0	0	0	0	0	0	0	0	0	0	0	0	0	0	0	0	0
539462	KEMLVTFKNPHAKRQ	E	514	528	HLA-DRB1*04:03	0%	Dengue virus 2	ELISPOT	IFNg release	Positive	0	0	0	0	0	0	0	0	0	0	0	0	0	0	0	0	0	0	0	0	0	0	0	0	0	0
539479	KKTLVTFKNPHAKKQ	E	514	528	HLA-DRB1*14:04	0%	Dengue virus 2	ELISPOT	IFNg release	Positive	0	0	0	0	0	0	0	0	0	0	0	0	0	0	0	0	0	0	0	0	0	0	0	0	0	0
539629	LSVTLVLVGFVTLYL	E	475	489	HLA-DRB1*15:06	0%	Dengue virus 2	ELISPOT	IFNg release	Positive	0	0	0	0	0	0	0	0	0	0	0	0	0	0	0	0	0	0	0	0	0	0	0	0	0	0
866243	AWMVHRQWFLDLPLP	E	205	219	HLA-DQA1*01:01/DQB1*05:01	0%	Dengue virus 2	ELISPOT	IFNg release	Positive	0	0	0	0	0	0	0	0	0	0	0	0	0	0	0	0	0	0	0	0	0	0	0	0	0	0
867375	KAWLVHRQWFLDLPF	E	204	218	HLA-DQA1*01:01/DQB1*05:01	0%	Dengue virus 2	ELISPOT	IFNg release	Positive	0	0	0	0	0	0	0	0	0	0	0	0	0	0	0	0	0	0	0	0	0	0	0	0	0	0
868347	PPFGDSYVIIGVEPG	E	371	385	HLA-DQA1*03:01/DQB1*03:02	0%	Dengue virus 2	ELISPOT	IFNg release	Positive	0	0	0	0	0	0	0	0	0	0	0	0	0	0	0	0	0	0	0	0	0	0	0	0	0	0
739191	IKGIMHAGKRSLRPQ	NS1	868	882	HLA-DRB1*14:02	0%	Dengue virus 2	ELISPOT	IFNg release	Positive	0	0	0	0	0	0	0	0	0	0	0	0	0	0	0	0	0	0	0	0	0	0	0	0	0	0
866829	GFGFFTTNIWLKLRE	NS1	934	948	HLA-DPB1*01:01	0%	Dengue virus 2	ELISPOT	IFNg release	Positive	0	0	0	0	0	0	0	0	0	0	0	0	0	0	0	0	0	0	0	0	0	0	0	0	0	0
150245	GMGVTYLALLAAYKV	NS2a	1196	1210	HLA-DRB1*01:01	0%	Dengue virus 2	ELISPOT	IFNg release	Positive	0	0	0	0	0	0	0	0	0	0	0	0	0	0	0	0	0	0	0	0	0	0	0	0	0	0
167819	SSQQKTDWIPLALTIKGLNP	NS2a	184	203	HLA-DP	0%	Dengue virus 2	ELISPOT	IFNg release	Positive	0	0	0	0	0	0	0	0	0	0	0	0	0	0	0	0	0	0	0	0	0	1	0	0	1	0
195207	CTILAVVSVSPLLLT	NS2a	1144	1158	HLA-DRB1*07:01	0%	Dengue virus 2	ELISPOT	IFNg release	Positive	0	0	0	0	0	0	0	0	0	0	0	0	0	0	0	0	0	0	0	0	0	1	0	0	1	0
195269	ERYQLAVTITAILCV	NS2a	1268	1282	HLA-DRB1*07:01	0%	Dengue virus 2	ELISPOT	IFNg release	Positive	0	0	0	0	0	0	0	0	0	0	0	0	0	0	0	0	0	0	0	0	0	0	0	0	0	0
195523	IPLVLIIKGLNPTAI	NS2a	1319	1333	HLA-DRB1*08:02	0%	Dengue virus 2	ELISPOT	IFNg release	Positive	0	0	0	0	0	0	0	0	0	0	0	0	0	0	0	0	0	0	0	0	0	0	0	0	0	0
195615	LAVVSVSPLLLTSSQ	NS2a	1186	1200	HLA-DRB1*07:01	0%	Dengue virus 2	ELISPOT	IFNg release	Positive	0	0	0	0	0	0	0	0	0	0	0	0	0	0	0	0	0	0	0	0	0	1	0	0	1	0
195712	LSLTFIRSTTPLIMA	NS2a	1275	1289	HLA-DRB1*07:01	0%	Dengue virus 2	ELISPOT	IFNg release	Positive	0	0	0	0	0	0	0	0	0	0	0	0	0	0	0	0	0	0	0	0	0	0	0	0	0	0
195763	MMVLKIVRKMERYQL	NS2a	1258	1272	HLA-DRB1*03:01	0%	Dengue virus 2	ELISPOT	IFNg release	Positive	0	0	0	0	0	0	0	0	0	0	0	0	0	0	0	0	0	0	0	0	0	0	0	0	0	0
195798	NMEKYQLAVTIMAIL	NS2a	1266	1280	HLA-DRB1*04:01	0%	Dengue virus 2	ELISPOT	IFNg release	Positive	0	0	0	0	0	0	0	0	0	0	0	0	0	0	0	0	0	0	0	0	0	1	0	0	1	0
539077	ALGIMVLKMVRSMEK	NS2a	1255	1269	HLA-DRB1*12:02	0%	Dengue virus 2	ELISPOT	IFNg release	Positive	0	0	0	0	0	0	0	0	0	0	0	0	0	0	0	0	0	0	0	0	0	0	0	0	0	0
539082	ALTIKGLNPTAILLT	NS2a	1322	1336	HLA-DRB1*09:01	0%	Dengue virus 2	ELISPOT	IFNg release	Positive	0	0	0	0	0	0	0	0	0	0	0	0	0	0	0	0	0	0	0	0	0	0	0	0	0	0
539110	AWALGMMVLKIVRNM	NS2a	1253	1267	HLA-DRB1*13:01	0%	Dengue virus 2	ELISPOT	IFNg release	Positive	0	0	0	0	0	0	0	0	0	0	0	0	0	0	0	0	0	0	0	0	0	0	0	0	0	0
539392	IALGIMVLKMVRSME	NS2a	1254	1268	HLA-DRB1*13:01	0%	Dengue virus 2	ELISPOT	IFNg release	Positive	0	0	0	0	0	0	0	0	0	0	0	0	0	0	0	0	0	0	0	0	0	0	0	0	0	0
539556	LGIMILKLVRKMEKY	NS2a	1256	1270	HLA-DRB1*12:02	0%	Dengue virus 2	ELISPOT	IFNg release	Positive	0	0	0	0	0	0	0	0	0	0	0	0	0	0	0	0	0	0	0	0	0	0	0	0	0	0
539557	LGIMVLKIVRSMEKY	NS2a	1256	1270	HLA-DRB1*12:02	0%	Dengue virus 2	ELISPOT	IFNg release	Positive	0	0	0	0	0	0	0	0	0	0	0	0	0	0	0	0	0	0	0	0	0	0	0	0	0	0
539561	LGMMILKIVRNMEKY	NS2a	1256	1270	HLA-DRB1*13:01	0%	Dengue virus 2	ELISPOT	IFNg release	Positive	0	0	0	0	0	0	0	0	0	0	0	0	0	0	0	0	0	0	0	0	0	0	0	0	0	0
539699	MVLKMVRNMEKYQLA	NS2a	1259	1273	HLA-DRB1*13:01	0%	Dengue virus 2	ELISPOT	IFNg release	Positive	0	0	0	0	0	0	0	0	0	0	0	0	0	0	0	0	0	0	0	0	0	1	0	0	1	0
740700	VVSVSPLLLTSSQQK	NS2a	1188	1202	HLA-DRB1*01:02	0%	Dengue virus 2	ELISPOT	IFNg release	Positive	0	0	0	0	0	0	0	0	0	0	0	0	0	0	0	0	0	0	0	0	0	1	0	0	1	0
24916	HTGREIVDLMCHATE	NS3	251	265	HLA-DPw2	0%	Dengue virus 2	ICS	IFNg release	Positive	0	0	0	0	0	0	0	0	0	0	0	0	0	0	0	0	0	0	0	0	0	0	0	0	0	0
150192	ETLLLLTLLAAVTGG	NS4a	2146	2160	HLA-DRB1*01:01	0%	Dengue virus 2	ELISPOT	IFNg release	Positive	0	0	0	0	0	0	0	0	0	0	0	0	0	0	0	0	0	0	0	0	0	0	0	0	0	0
195810	NQLTYVVIAILTLVA	NS4a	2225	2239	HLA-DRB1*07:01	0%	Dengue virus 2	ELISPOT	IFNg release	Positive	0	0	0	0	0	0	0	0	0	0	0	0	0	0	0	0	0	0	0	0	0	0	0	0	0	0
195118	AGLLFSIMRNTTSAR	NS4b	2476	2490	HLA-DRB1*04:01	0%	Dengue virus 2	ELISPOT	IFNg release	Positive	0	0	0	0	0	0	0	0	0	0	0	0	0	0	0	0	0	0	0	0	0	0	0	0	0	0
195393	GLLFSIMKNTANTRR	NS4b	2463	2477	HLA-DRB1*04:01	0%	Dengue virus 2	ELISPOT	IFNg release	Positive	0	0	0	0	0	0	0	0	0	0	0	0	0	0	0	0	0	0	0	0	0	0	0	0	0	0
195394	GLLFSIMKNTTNARR	NS4b	2477	2491	HLA-DRB1*04:01	0%	Dengue virus 2	ELISPOT	IFNg release	Positive	0	0	0	0	0	0	0	0	0	0	0	0	0	0	0	0	0	0	0	0	0	0	0	0	0	0
195395	GLLFSIMKNTTNSRR	NS4b	2477	2491	HLA-DRB1*04:01	0%	Dengue virus 2	ELISPOT	IFNg release	Positive	0	0	0	0	0	0	0	0	0	0	0	0	0	0	0	0	0	0	0	0	0	0	0	0	0	0
195396	GLLFSIMKNTTSARR	NS4b	2477	2491	HLA-DRB1*04:01	0%	Dengue virus 2	ELISPOT	IFNg release	Positive	0	0	0	0	0	0	0	0	0	0	0	0	0	0	0	0	0	0	0	0	0	0	0	0	0	0
195398	GLLFSVMKNTTNTRR	NS4b	2477	2491	HLA-DRB1*04:01	0%	Dengue virus 2	ELISPOT	IFNg release	Positive	0	0	0	0	0	0	0	0	0	0	0	0	0	0	0	0	0	0	0	0	0	0	0	0	0	0
195659	LLFSIMRNTTTARRG	NS4b	2478	2492	HLA-DRB1*04:01	0%	Dengue virus 2	ELISPOT	IFNg release	Positive	0	0	0	0	0	0	0	0	0	0	0	0	0	0	0	0	0	0	0	0	0	0	0	0	0	0
867335	ITLTAALLLLIAHYA	NS4b	2348	2362	HLA-DPB1*04:02	0%	Dengue virus 2	ELISPOT	IFNg release	Positive	0	0	0	0	0	0	0	0	0	0	0	0	0	0	0	0	0	0	0	0	0	0	0	0	0	0
869090	TAIANQATVLMGLGR	NS4b	2309	2323	HLA-DQA1*01:02/DQB1*06:02	0%	Dengue virus 2	ELISPOT	IFNg release	Positive	0	0	0	0	0	0	0	0	0	0	0	0	0	0	0	0	0	0	0	0	0	0	0	0	0	0
195250	ELIMKDGRKLVVPCR	NS5	3206	3220	HLA-DRB1*03:01	0%	Dengue virus 2	ELISPOT	IFNg release	Positive	0	0	0	0	0	0	0	0	0	0	0	0	0	0	0	0	0	0	0	0	0	0	0	0	0	0
195384	GLFKSIQHLTSQEEI	NS5	3118	3132	HLA-DRB1*11:01	0%	Dengue virus 2	ELISPOT	IFNg release	Positive	0	0	0	0	0	0	0	0	0	0	0	0	0	0	0	0	0	0	0	0	0	0	0	0	0	0
195445	HFHELIMKDGRILVV	NS5	3203	3217	HLA-DRB1*03:01	0%	Dengue virus 2	ELISPOT	IFNg release	Positive	0	0	0	0	0	0	0	0	0	0	0	0	0	0	0	0	0	0	0	0	0	0	0	0	0	0
195460	HRLGYILRDVSRKEG	NS5	3004	3018	HLA-DRB1*08:02	0%	Dengue virus 2	ELISPOT	IFNg release	Positive	0	0	0	0	0	0	0	0	0	0	0	0	0	0	0	0	0	0	0	0	0	0	0	0	0	0
195513	IMKDGRKLVVPCRNQ	NS5	3207	3221	HLA-DRB1*03:01	0%	Dengue virus 2	ELISPOT	IFNg release	Positive	0	0	0	0	0	0	0	0	0	0	0	0	0	0	0	0	0	0	0	0	0	0	0	0	0	0
32939	KPWDIIPMV	NS5	2821	2829	HLA class II	0%	Dengue virus 2	ELISA	IL-2 release	Positive	0	0	0	0	0	0	0	0	0	0	0	0	0	0	0	0	0	0	0	0	0	0	0	0	0	0
32941	KPWDVLPMV	NS5	2821	2829	HLA class II	0%	Dengue virus 2	ELISA	IL-2 release	Positive	0	0	0	0	0	0	0	0	0	0	0	0	0	0	0	0	0	0	0	0	0	0	0	0	0	0
32942	KPWDVLPTV	NS5	2821	2829	HLA class II	0%	Dengue virus 2	ELISA	IL-2 release	Positive	0	0	0	0	0	0	0	0	0	0	0	0	0	0	0	0	0	0	0	0	0	0	0	0	0	0
539560	LGKNEFQIYKKSGIQ	NS5	2511	2525	HLA-DRB1*15:02	0%	Dengue virus 2	ELISPOT	IFNg release	Positive	0	0	0	0	0	0	0	0	0	0	0	0	0	0	0	0	0	0	0	0	0	0	0	0	0	0
540064	VNGVVRLLTKPWDVI	NS5	2812	2826	HLA-DRB1*12:02	0%	Dengue virus 2	ELISPOT	IFNg release	Positive	0	0	0	0	0	0	0	0	0	0	0	0	0	0	0	0	0	0	0	0	0	0	0	0	0	0
869148	TFVESWEDIPYLGKR	NS5	3319	3333	HLA-DQA1*01:01/DQB1*05:01	0%	Dengue virus 2	ELISPOT	IFNg release	Positive	0	0	0	0	0	0	0	0	0	0	0	0	0	0	0	0	0	0	0	0	0	0	0	0	0	0
150105	AFLRFLTIPPTAGIL	C	52	66	HLA-DRB1*01:01	100%	Dengue virus 2	ELISPOT	IFNg release	Positive	1	1	1	1	1	1	1	1	1	1	1	1	1	1	1	1	1	1	1	1	0	1	1	0	1	0
180768	TIKKSKAINVLRGFR	C	71	85	HLA class II	100%	Dengue virus 2	ELISPOT	IFNg release	Positive	1	1	1	1	1	1	1	1	1	1	1	1	1	1	1	1	1	1	1	1	0	1	1	0	1	0
195326	FRKEIGRMLNILNRR	C	84	98	HLA-DRB1*04:01	100%	Dengue virus 2	ELISPOT	IFNg release	Positive	1	1	1	1	1	1	1	1	1	1	1	1	1	1	1	1	1	1	1	1	0	1	1	0	1	0
195489	IGRMLNILNRRRRTA	C	88	102	HLA-DRB1*08:02	100%	Dengue virus 2	ELISPOT	IFNg release	Positive	1	1	1	1	1	1	1	1	1	1	1	1	1	1	1	1	1	1	1	1	0	0	1	0	0	0
195556	KEIGRMLNILNRRRR	C	86	100	HLA-DRB1*08:02	100%	Dengue virus 2	ELISPOT	IFNg release	Positive	1	1	1	1	1	1	1	1	1	1	1	1	1	1	1	1	1	1	1	1	0	1	1	0	1	0
195886	RGFRKEIGRMLNILN	C	82	96	HLA-DRB1*04:01	100%	Dengue virus 2	ELISPOT	IFNg release	Positive	1	1	1	1	1	1	1	1	1	1	1	1	1	1	1	1	1	1	1	1	0	1	1	0	1	0
196064	TPFNMLKRERNRVST	C	11	25	HLA-DRB1*08:02	100%	Dengue virus 2	ELISPOT	IFNg release	Positive	1	1	1	1	1	1	1	1	1	1	1	1	1	1	1	1	1	1	1	1	0	1	1	0	1	0
196213	WGTIKKSKAINVLRG	C	69	83	HLA-DRB1*07:01	100%	Dengue virus 2	ELISPOT	IFNg release	Positive	1	1	1	1	1	1	1	1	1	1	1	1	1	1	1	1	1	1	1	1	0	1	1	0	1	0
26836	IKKSKAINVLRGFRKEIGRM	C	72	91	HLA class II	100%	Dengue virus 2	ELISPOT	IFNg release	Positive	1	1	1	1	1	1	1	1	1	1	1	1	1	1	1	1	1	1	1	1	0	1	1	0	1	0
39098	LRGFRKEIGRML	C	81	92	HLA-DPw4	100%	Dengue virus 2	ICS	IFNg release	Positive	1	1	1	1	1	1	1	1	1	1	1	1	1	1	1	1	1	1	1	1	0	1	1	0	1	0
539214	EIGRMLNILNRRRRT	C	87	101	HLA-DRB1*12:02	100%	Dengue virus 2	ELISPOT	IFNg release	Positive	1	1	1	1	1	1	1	1	1	1	1	1	1	1	1	1	1	1	1	1	0	0	1	0	0	0
539264	FLRFLTIPPTAGILK	C	53	67	HLA-DRB1*14:02	100%	Dengue virus 2	ELISPOT	IFNg release	Positive	1	1	1	1	1	1	1	1	1	1	1	1	1	1	1	1	1	1	1	1	0	1	1	0	1	0
539410	IKKSKAINVLRGFRK	C	72	86	HLA-DRB1*14:04	100%	Dengue virus 2	ELISPOT	IFNg release	Positive	1	1	1	1	1	1	1	1	1	1	1	1	1	1	1	1	1	1	1	1	0	1	1	0	1	0
539514	KSKAINVLRGFRKEI	C	74	88	HLA-DRB1*12:02	100%	Dengue virus 2	ELISPOT	IFNg release	Positive	1	1	1	1	1	1	1	1	1	1	1	1	1	1	1	1	1	1	1	1	0	1	1	0	1	0
539618	LRGFRKEIGRMLNIL	C	81	95	HLA-DRB1*09:01	100%	Dengue virus 2	ELISPOT	IFNg release	Positive	1	1	1	1	1	1	1	1	1	1	1	1	1	1	1	1	1	1	1	1	0	1	1	0	1	0
539887	RWGTIKKSKAINVLR	C	68	82	HLA-DRB1*08:03	100%	Dengue virus 2	ELISPOT	IFNg release	Positive	1	1	1	1	1	1	1	1	1	1	1	1	1	1	1	1	1	1	1	1	0	1	1	0	1	0
540060	VLRGFRKEIGRMLNI	C	80	94	HLA-DRB1*09:01	100%	Dengue virus 2	ELISPOT	IFNg release	Positive	1	1	1	1	1	1	1	1	1	1	1	1	1	1	1	1	1	1	1	1	0	1	1	0	1	0
540069	VQQLTKRFSLGMLQG	C	26	40	HLA-DRB1*13:01	100%	Dengue virus 2	ELISPOT	IFNg release	Positive	1	1	1	1	1	1	1	1	1	1	1	1	1	1	1	1	1	1	1	1	0	1	1	0	1	0
739879	PFNMLKRERNRVSTV	C	12	26	HLA-DRB1*14:02	100%	Dengue virus 2	ELISPOT	IFNg release	Positive	1	1	1	1	1	1	1	1	1	1	1	1	1	1	1	1	1	1	1	1	0	1	1	0	1	0
857784	LKRWGTIKKSKAINV	C	66	80	HLA-DRB1*07:01	100%	Dengue virus 2	ELISPOT	IFNg release	Positive-Low	1	1	1	1	1	1	1	1	1	1	1	1	1	1	1	1	1	1	1	1	0	1	1	0	1	0
195503	ILRHPGFTIMAAILA	PreM	127	141	HLA-DRB1*07:01	100%	Dengue virus 2	ELISPOT	IFNg release	Positive	1	1	1	1	1	1	1	1	1	1	1	1	1	1	1	1	1	1	1	1	1	1	1	1	0	1
195702	LRHPGFTIMAAILAY	PreM	242	256	HLA-DRB1*11:01	100%	Dengue virus 2	ELISPOT	IFNg release	Positive	1	1	1	1	1	1	1	1	1	1	1	1	1	1	1	1	1	1	1	1	1	1	1	1	0	1
195892	RIETWILRHPGFTIM	PreM	122	136	HLA-DRB1*15:01	100%	Dengue virus 2	ELISPOT	IFNg release	Positive	1	1	1	1	1	1	1	1	1	1	1	1	1	1	1	1	1	1	1	1	1	1	1	0	0	1
539943	SVALVPHVGMGLETR	PreM	206	220	HLA-DRB1*15:02	100%	Dengue virus 2	ELISPOT	IFNg release	Positive	1	1	1	1	1	1	1	1	1	1	1	1	1	1	1	1	1	1	1	1	1	1	1	1	1	1
540130	WILRHPGFTIMAAIL	PreM	126	140	HLA-DRB1*15:06	100%	Dengue virus 2	ELISPOT	IFNg release	Positive	1	1	1	1	1	1	1	1	1	1	1	1	1	1	1	1	1	1	1	1	1	1	1	1	0	1

180564	KKQDVVVLGSQEGAM	E	526	540	HLA class II	100%	Dengue virus 2	ELISPOT	IFNg release	Positive	1	1	1	1	1	1	1	1	1	1	1	1	1	1	1	1	1	1	1	1	1	1	1	1	1	1
180568	KNPHAKKQDVVVLGS	E	521	535	HLA class II	100%	Dengue virus 2	ELISPOT	IFNg release	Positive	1	1	1	1	1	1	1	1	1	1	1	1	1	1	1	1	1	1	1	1	1	1	1	1	1	1
180590	LDFELIKTEAKQPAT	E	41	55	HLA class II	100%	Dengue virus 2	ELISPOT	IFNg release	Positive	1	1	1	1	1	1	1	1	1	1	1	1	1	1	1	1	1	1	1	1	1	1	1	1	1	1
180820	WLVHRQWFLDLPLPW	E	196	210	HLA class II	100%	Dengue virus 2	ELISPOT	IFNg release	Positive	1	1	1	1	1	1	1	1	1	1	1	1	1	1	1	1	1	1	1	1	1	1	1	1	1	1
195351	GATEIQMSSGNLLFT	E	266	280	HLA-DRB1*03:01	100%	Dengue virus 2	ELISPOT	IFNg release	Positive	1	1	1	1	1	1	1	1	1	1	1	1	1	1	1	1	1	1	1	1	1	1	1	1	1	1
195366	GHLKCRLRMDKLQLK	E	281	295	HLA-DRB1*03:01	100%	Dengue virus 2	ELISPOT	IFNg release	Positive	1	1	1	1	1	1	1	1	1	1	1	1	1	1	1	1	1	1	1	1	1	1	1	1	1	1
195952	SGVSWTMKILIGVII	E	449	463	HLA-DRB1*15:01	100%	Dengue virus 2	ELISPOT	IFNg release	Positive	1	1	1	1	1	1	1	1	1	1	1	1	1	1	1	1	1	1	1	1	0	1	0	1	1	0
196022	SWTMKILIGVIITWI	E	452	466	HLA-DRB1*08:02	100%	Dengue virus 2	ELISPOT	IFNg release	Positive	1	1	1	1	1	1	1	1	1	1	1	1	1	1	1	1	1	1	1	1	0	1	0	1	1	0
196215	WIQKETLVTFKNPHA	E	231	245	HLA-DRB1*08:02	100%	Dengue virus 2	ELISPOT	IFNg release	Positive	1	1	1	1	1	1	1	1	1	1	1	1	1	1	1	1	1	1	1	1	1	1	1	1	1	1
539236	ENLEYTIVITPHSGE	E	133	147	HLA-DRB1*04:07	100%	Dengue virus 2	ELISPOT	IFNg release	Positive	1	1	1	1	1	1	1	1	1	1	1	1	1	1	1	1	1	1	1	1	1	0	1	0	1	1
539377	HAKKQDVVVLGSQEGAMH	E	244	261	HLA class II	100%	Dengue virus 2	ICS	IFNg release	Positive	1	1	1	1	1	1	1	1	1	1	1	1	1	1	1	1	1	1	1	1	1	1	1	1	1	1
539464	KETLVTFKNPHAKKQ	E	234	248	HLA-DRB1*10:01	100%	Dengue virus 2	ELISPOT	IFNg release	Positive	1	1	1	1	1	1	1	1	1	1	1	1	1	1	1	1	1	1	1	1	1	1	1	1	1	1
539648	LVTFKNPHAKKQDVV	E	237	251	HLA-DRB1*15:02	100%	Dengue virus 2	ELISPOT	IFNg release	Positive	1	1	1	1	1	1	1	1	1	1	1	1	1	1	1	1	1	1	1	1	1	1	1	1	1	1
539689	MRGAKRMAILGDTAW	E	406	420	HLA-DRB1*15:06	100%	Dengue virus 2	ELISPOT	IFNg release	Positive	1	1	1	1	1	1	1	1	1	1	1	1	1	1	1	1	1	1	1	1	1	1	1	1	1	0
539733	NLEYTIVITPHSGEE	E	134	148	HLA-DRB1*04:07	100%	Dengue virus 2	ELISPOT	IFNg release	Positive	1	1	1	1	1	1	1	1	1	1	1	1	1	1	1	1	1	1	1	1	1	0	1	0	1	1
539806	QKETLVTFKNPHAKK	E	513	527	HLA-DRB1*04:03	100%	Dengue virus 2	ELISPOT	IFNg release	Positive	1	1	1	1	1	1	1	1	1	1	1	1	1	1	1	1	1	1	1	1	1	1	1	1	1	1
65140	TLVTFKNPHAKKQDV	E	236	250	HLA class II	100%	Dengue virus 2	ELISPOT	IFNg release	Positive	1	1	1	1	1	1	1	1	1	1	1	1	1	1	1	1	1	1	1	1	1	1	1	1	1	1
738866	EYTIVITPHSGEEHA	E	136	150	HLA-DRB1*04:07	100%	Dengue virus 2	ELISPOT	IFNg release	Positive	1	1	1	1	1	1	1	1	1	1	1	1	1	1	1	1	1	1	1	1	1	0	1	0	1	1
739010	GGVFTSIGKALHQVF	E	706	720	HLA-DRB1*14:02	100%	Dengue virus 2	ELISPOT	IFNg release	Positive	1	1	1	1	1	1	1	1	1	1	1	1	1	1	1	1	1	1	1	1	1	1	1	1	1	0
741744	MRCIGISNRDFVEGVSGGSW	E	281	300	HLA class II	100%	Dengue virus 2	ELISPOT	IFNg release	Positive	1	1	1	1	1	1	1	1	1	1	1	1	1	1	1	1	1	1	1	1	1	1	1	1	0	1
866193	ATEIQMSSGNLLFTG	E	257	271	HLA-DRB3*02:02	100%	Dengue virus 2	ELISPOT	IFNg release	Positive	1	1	1	1	1	1	1	1	1	1	1	1	1	1	1	1	1	1	1	1	1	1	1	1	1	1
180396	DGCWYGMEIRPLKEKEENLVNS	NS1	1102	1123	HLA class II	100%	Dengue virus 2	ICS	IFNg release	Positive	1	1	1	1	1	1	1	1	1	1	1	1	1	1	1	1	1	1	1	1	0	1	1	0	1	0
180603	LKYSWKTWGKAKMLS	NS1	111	125	HLA class II	100%	Dengue virus 2	ELISPOT	IFNg release	Positive	1	1	1	1	1	1	1	1	1	1	1	1	1	1	1	1	1	1	1	1	0	1	1	0	1	0
195845	QDVFCDSKLMSAAIK	NS1	175	189	HLA-DRB1*03:01	100%	Dengue virus 2	ELISPOT	IFNg release	Positive	1	1	1	1	1	1	1	1	1	1	1	1	1	1	1	1	1	1	1	1	0	0	1	0	1	0
539828	QYKFQPESPSKLASA	NS1	806	820	HLA-DRB3*02:02	100%	Dengue virus 2	ELISPOT	IFNg release	Positive	1	1	1	1	1	1	1	1	1	1	1	1	1	1	1	1	1	1	1	1	0	1	1	0	1	0
738766	EGICGIRSVTRLENL	NS1	52	66	HLA-DRB1*11:04	100%	Dengue virus 2	ELISPOT	IFNg release	Positive	1	1	1	1	1	1	1	1	1	1	1	1	1	1	1	1	1	1	1	1	0	1	1	0	1	0
866460	DYGFGVFTTNIWLKL	NS1	932	946	HLA-DPB1*02:01	100%	Dengue virus 2	ELISPOT	IFNg release	Positive	1	1	1	1	1	1	1	1	1	1	1	1	1	1	1	1	1	1	1	1	0	1	1	0	1	0
150131	ALTIKGLNPTAIFLT	NS2a	1308	1322	HLA-DRB1*09:01	100%	Dengue virus 2	ELISPOT	IFNg release	Positive	1	1	1	1	1	1	1	1	1	1	1	1	1	1	1	1	1	1	1	1	0	1	1	0	1	0
150273	GVTYLALLAAFKVRP	NS2a	1198	1212	HLA-DRB1*01:01	100%	Dengue virus 2	ELISPOT	IFNg release	Positive	1	1	1	1	1	1	1	1	1	1	1	1	1	1	1	1	1	1	1	1	0	1	1	0	1	0
195153	ALLAAFKVRPTFAAG	NS2a	1203	1217	HLA-DRB1*08:02	100%	Dengue virus 2	ELISPOT	IFNg release	Positive	1	1	1	1	1	1	1	1	1	1	1	1	1	1	1	1	1	1	1	1	0	1	1	0	1	0
539540	LALLAAFKVRPTFAA	NS2a	1202	1216	HLA-DRB1*14:04	100%	Dengue virus 2	ELISPOT	IFNg release	Positive	1	1	1	1	1	1	1	1	1	1	1	1	1	1	1	1	1	1	1	1	0	1	1	0	1	0
540094	VTYLALLAAFKVRPT	NS2a	1199	1213	HLA-DRB1*12:02	100%	Dengue virus 2	ELISPOT	IFNg release	Positive	1	1	1	1	1	1	1	1	1	1	1	1	1	1	1	1	1	1	1	1	0	1	1	0	1	0
540154	YLALLAAFKVRPTFA	NS2a	1201	1215	HLA-DRB1*14:04	100%	Dengue virus 2	ELISPOT	IFNg release	Positive	1	1	1	1	1	1	1	1	1	1	1	1	1	1	1	1	1	1	1	1	0	1	1	0	1	0
868918	SLGVLGMALFLEEML	NS2a	1136	1150	HLA-DQA1*05:01/DQB1*02:01	100%	Dengue virus 2	ELISPOT	IFNg release	Positive	1	1	1	1	1	1	1	1	1	1	1	1	1	1	1	1	1	1	1	1	0	1	1	0	1	0
167732	DQAEISGSSPILSITISEDG	NS2b	63	82	HLA-DP	100%	Dengue virus 2	ELISPOT	IFNg release	Positive	1	1	1	1	1	1	1	1	1	1	1	1	1	1	1	1	1	1	1	1	0	1	1	0	1	0
195992	SSLLKNDIPMTGPLV	NS2b	19	33	HLA-DRB1*03:01	100%	Dengue virus 2	ELISPOT	IFNg release	Positive	1	1	1	1	1	1	1	1	1	1	1	1	1	1	1	1	1	1	1	1	0	1	1	0	1	0
1067121	GKVVGLYGNGVVTRS	NS3	1619	1633	HLA-DRB1*15:01	100%	Dengue virus 2	ELISPOT	IFNg release	Positive	1	1	1	1	1	1	1	1	1	1	1	1	1	1	1	1	1	1	1	1	0	1	1	0	1	0
12606	EIVDLMCHATFTMRL	NS3	255	269	HLA class II	100%	Dengue virus 2	ELISPOT	IFNg release	Positive	1	1	1	1	1	1	1	1	1	1	1	1	1	1	1	1	1	1	1	1	0	1	1	0	1	0
1392191	GREIVDLMCHATFTM	NS3	1728	1742		100%	Dengue virus 2	biological activity	activation	Positive	1	1	1	1	1	1	1	1	1	1	1	1	1	1	1	1	1	1	1	1	0	1	1	0	1	0
139758	GKTKRYLPAIVREAI	NS3	1673	1687	HLA-DRB1*09:01	100%	Dengue virus 2	ELISPOT	IFNg release	Positive	1	1	1	1	1	1	1	1	1	1	1	1	1	1	1	1	1	1	1	1	0	1	1	0	1	0
180698	PNYNLIIMDEAHFTD	NS3	1751	1765	HLA class II	100%	Dengue virus 2	ELISPOT	IFNg release	Positive	1	1	1	1	1	1	1	1	1	1	1	1	1	1	1	1	1	1	1	1	0	1	1	0	1	0
180807	VTDFKGKTVWFVPSI	NS3	1826	1840	HLA class II	100%	Dengue virus 2	ELISPOT	IFNg release	Positive-Low	1	1	1	1	1	1	1	1	1	1	1	1	1	1	1	1	1	1	1	1	0	1	1	0	1	0
195295	FHTMWHVTRGAVLMH	NS3	1521	1535	HLA-DRB1*07:01	100%	Dengue virus 2	ELISPOT	IFNg release	Positive	1	1	1	1	1	1	1	1	1	1	1	1	1	1	1	1	1	1	1	1	0	1	1	0	1	0
195420	GTFHTMWHVTRGAVL	NS3	1519	1533	HLA-DRB1*07:01	100%	Dengue virus 2	ELISPOT	IFNg release	Positive	1	1	1	1	1	1	1	1	1	1	1	1	1	1	1	1	1	1	1	1	0	1	1	0	1	0
195564	KGKVVGLYGNGVVTR	NS3	1618	1632	HLA-DRB1*15:01	100%	Dengue virus 2	ELISPOT	IFNg release	Positive	1	1	1	1	1	1	1	1	1	1	1	1	1	1	1	1	1	1	1	1	0	1	1	0	1	0
195765	MRLLSPVRVPNYNLI	NS3	1742	1756	HLA-DRB1*13:01	100%	Dengue virus 2	ELISPOT	IFNg release	Positive	1	1	1	1	1	1	1	1	1	1	1	1	1	1	1	1	1	1	1	1	0	1	1	0	1	0
195888	RGLRTLILAPTRVVA	NS3	1689	1703	HLA-DRB1*08:02	100%	Dengue virus 2	ELISPOT	IFNg release	Positive	1	1	1	1	1	1	1	1	1	1	1	1	1	1	1	1	1	1	1	1	0	1	1	0	1	0
21115	GLRTLILAPTRVVAA	NS3	1690	1704	HLA-DRB1*04:03	100%	Dengue virus 2	ELISPOT	IFNg release	Positive	1	1	1	1	1	1	1	1	1	1	1	1	1	1	1	1	1	1	1	1	0	1	1	0	1	0
26965	ILAPTRVVAAEMEEA	NS3	1695	1709	HLA-DQA1*03:01/DQB1*03:02	100%	Dengue virus 2	ELISPOT	IFNg release	Positive	1	1	1	1	1	1	1	1	1	1	1	1	1	1	1	1	1	1	1	1	0	1	1	0	1	0
30396	KEGERKKLKPRWLDA	NS3	584	598	HLA class II	100%	Dengue virus 2	ICS	IFNg release	Positive	1	1	1	1	1	1	1	1	1	1	1	1	1	1	1	1	1	1	1	1	0	1	1	0	1	0
31726	KKVIQLSRKTFDSEY	NS3	1855	1869	HLA-DRB1*12:02	100%	Dengue virus 2	ELISPOT	IFNg release	Positive	1	1	1	1	1	1	1	1	1	1	1	1	1	1	1	1	1	1	1	1	0	1	1	0	1	0
34231	KVVGLYGNGVVTRSG	NS3	145	159	HLA class II	100%	Dengue virus 2	ELISPOT	IFNg release	Positive	1	1	1	1	1	1	1	1	1	1	1	1	1	1	1	1	1	1	1	1	0	1	1	0	1	0
36560	LIIMDEAHFTDPASI	NS3	1755	1769	HLA-DQA1*01:01/DQB1*05:01	100%	Dengue virus 2	ELISPOT	IFNg release	Positive	1	1	1	1	1	1	1	1	1	1	1	1	1	1	1	1	1	1	1	1	0	1	1	0	1	0
39225	LRTLILAPTRVVAAE	NS3	1691	1705	HLA-DRB1*12:02	100%	Dengue virus 2	ELISPOT	IFNg release	Positive	1	1	1	1	1	1	1	1	1	1	1	1	1	1	1	1	1	1	1	1	0	1	1	0	1	0
4152	ARGYISTRVEMGEAA	NS3	1757	1771	HLA-DRB1*04:07	100%	Dengue virus 2	ELISPOT	IFNg release	Positive	1	1	1	1	1	1	1	1	1	1	1	1	1	1	1	1	1	1	1	1	0	1	1	0	1	0
49766	PTRVVAAEMEEALRG	NS3	223	237	HLA-DRB1*15:01	100%	Dengue virus 2	ICS	IFNg release	Positive	1	1	1	1	1	1	1	1	1	1	1	1	1	1	1	1	1	1	1	1	0	1	1	0	1	0
539025	AAGIFMTATPPGSRD	NS3	1784	1798	HLA-DRB1*04:03	100%	Dengue virus 2	ELISPOT	IFNg release	Positive	1	1	1	1	1	1	1	1	1	1	1	1	1	1	1	1	1	1	1	1	0	1	1	0	1	0
539041	AEMEEALRGLPIRYQ	NS3	1704	1718	HLA-DRB1*13:01	100%	Dengue virus 2	ELISPOT	IFNg release	Positive	1	1	1	1	1	1	1	1	1	1	1	1	1	1	1	1	1	1	1	1	0	1	1	0	1	0
539289	GEAAGIFMTATPPGS	NS3	1782	1796	HLA-DRB1*04:03	100%	Dengue virus 2	ELISPOT	IFNg release	Positive	1	1	1	1	1	1	1	1	1	1	1	1	1	1	1	1	1	1	1	1	0	1	1	0	1	0
539448	IVREAIKRGLRTLIL	NS3	1682	1696	HLA-DRB1*12:02	100%	Dengue virus 2	ELISPOT	IFNg release	Positive	1	1	1	1	1	1	1	1	1	1	1	1	1	1	1	1	1	1	1	1	0	1	1	0	1	0
539467	KGKTVWFVPSIKAGN	NS3	1830	1844	HLA-DRB1*08:03	100%	Dengue virus 2	ELISPOT	IFNg release	Positive	1	1	1	1	1	1	1	1	1	1	1	1	1	1	1	1	1	1	1	1	0	1	1	0	1	0
539573	LILAPTRVVAAEMEE	NS3	1694	1708	HLA-DRB1*13:01	100%	Dengue virus 2	ELISPOT	IFNg release	Positive	1	1	1	1	1	1	1	1	1	1	1	1	1	1	1	1	1	1	1	1	0	1	1	0	1	0
539713	NGKKVIQLSRKTFDS	NS3	1853	1867	HLA-DRB1*12:02	100%	Dengue virus 2	ELISPOT	IFNg release	Positive	1	1	1	1	1	1	1	1	1	1	1	1	1	1	1	1	1	1	1	1	0	1	1	0	1	0
539857	RKNGKKVIQLSRKTF	NS3	1851	1865	HLA-DRB1*08:03	100%	Dengue virus 2	ELISPOT	IFNg release	Positive	1	1	1	1	1	1	1	1	1	1	1	1	1	1	1	1	1	1	1	1	0	1	1	0	1	0
63672	TFHTMWHVTRGAVLM	NS3	45	59	HLA class II	100%	Dengue virus 2	ELISPOT	IFNg release	Positive	1	1	1	1	1	1	1	1	1	1	1	1	1	1	1	1	1	1	1	1	0	1	1	0	1	0
64666	TKRYLPAIVREAIKR	NS3	200	214	HLA class II	100%	Dengue virus 2	ELISPOT	IFNg release	Positive	1	1	1	1	1	1	1	1	1	1	1	1	1	1	1	1	1	1	1	1	0	1	1	0	1	0
740081	REAIKRGLRTLILAP	NS3	1670	1684	HLA-DRB1*14:06	100%	Dengue virus 2	ELISPOT	IFNg release	Positive	1	1	1	1	1	1	1	1	1	1	1	1	1	1	1	1	1	1	1	1	0	1	1	0	1	0
740193	RTLILAPTRVVAAEM	NS3	1678	1692	HLA-DRB1*14:06	100%	Dengue virus 2	ELISPOT	IFNg release	Positive	1	1	1	1	1	1	1	1	1	1	1	1	1	1	1	1	1	1	1	1	0	1	1	0	1	0
866477	EALRGLPIRYQTPAI	NS3	1708	1722	HLA-DRB4*01:01	100%	Dengue virus 2	ELISPOT	IFNg release	Positive	1	1	1	1	1	1	1	1	1	1	1	1	1	1	1	1	1	1	1	1	0	1	1	0	1	0
868734	RYLPAIVREAIKRGL	NS3	1677	1691	HLA-DRB4*01:01	100%	Dengue virus 2	ELISPOT	IFNg release	Positive	1	1	1	1	1	1	1	1	1	1	1	1	1	1	1	1	1	1	1	1	0	1	1	0	1	0
869224	TLILAPTRVVAAEME	NS3	1693	1707	HLA-DQA1*01:02/DQB1*06:02	100%	Dengue virus 2	ELISPOT	IFNg release	Positive	1	1	1	1	1	1	1	1	1	1	1	1	1	1	1	1	1	1	1	1	0	1	1	0	1	0
150362	LETLLLLTLLATVTG	NS4a	2145	2159	HLA-DRB1*01:01	100%	Dengue virus 2	ELISPOT	IFNg release	Positive	1	1	1	1	1	1	1	1	1	1	1	1	1	1	1	1	1	1	1	1	0	1	1	0	1	0
167780	LTLNLITEMGRLPTFMTQKA	NS4a	2	21	HLA class II	100%	Dengue virus 2	ELISPOT	IFNg release	Positive	1	1	1	1	1	1	1	1	1	1	1	1	1	1	1	1	1	1	1	1	0	1	1	0	1	0
195107	AASIILEFFLIVLLI	NS4a	2185	2199	HLA-DRB1*15:01	100%	Dengue virus 2	ELISPOT	IFNg release	Positive	1	1	1	1	1	1	1	1	1	1	1	1	1	1	1	1	1	1	1	1	0	1	1	0	1	0
195635	LGMCCIITASILLWY	NS4a	2176	2190	HLA-DRB1*07:01	100%	Dengue virus 2	ELISPOT	IFNg release	Positive	1	1	1	1	1	1	1	1	1	1	1	1	1	1	1	1	1	1	1	1	0	1	1	0	1	0
195767	MTLGMCCIITASILL	NS4a	2160	2174	HLA-DRB1*07:01	100%	Dengue virus 2	ELISPOT	IFNg release	Positive	1	1	1	1	1	1	1	1	1	1	1	1	1	1	1	1	1	1	1	1	0	1	1	0	1	0
196054	TLETLLLLTLLATVT	NS4a	2144	2158	HLA-DRB1*15:01	100%	Dengue virus 2	ELISPOT	IFNg release	Positive	1	1	1	1	1	1	1	1	1	1	1	1	1	1	1	1	1	1	1	1	0	1	1	0	1	0
539804	QIQPHWIAASIILEF	NS4a	2192	2206	HLA-DRB1*13:01	100%	Dengue virus 2	ELISPOT	IFNg release	Positive	1	1	1	1	1	1	1	1	1	1	1	1	1	1	1	1	1	1	1	1	0	1	1	0	1	0
540148	YAQIQPHWIAASIIL	NS4a	2176	2190	HLA-DRB1*13:01	100%	Dengue virus 2	ELISPOT	IFNg release	Positive	1	1	1	1	1	1	1	1	1	1	1	1	1	1	1	1	1	1	1	1	0	1	1	0	1	0
738567	AYNHALSELPETLET	NS4a	2119	2133	HLA-DRB1*04:07	100%	Dengue virus 2	ELISPOT	IFNg release	Positive	1	1	1	1	1	1	1	1	1	1	1	1	1	1	1	1	1	1	1	1	0	1	1	0	1	0
195621	LCVTQVLMMRTTWAL	NS4b	2405	2419	HLA-DRB1*07:01	100%	Dengue virus 2	ELISPOT	IFNg release	Positive	1	1	1	1	1	1	1	1	1	1	1	1	1	1	1	1	1	1	1	1	0	1	1	0	1	0
195974	SNILDIDLRPASAWT	NS4b	2155	2169	HLA-DRB1*03:01	100%	Dengue virus 2	ELISPOT	IFNg release	Positive	1	1	1	1	1	1	1	1	1	1	1	1	1	1	1	1	1	1	1	1	0	1	1	0	1	0
539724	NIFRGSYLAGAGLLF	NS4b	2452	2466	HLA-DRB1*09:01	100%	Dengue virus 2	ELISPOT	IFNg release	Positive	1	1	1	1	1	1	1	1	1	1	1	1	1	1	1	1	1	1	1	1	0	1	1	0	1	0
739437	LDIDLRPASAWTLYA	NS4b	2257	2271	HLA-DRB1*01:02	100%	Dengue virus 2	ELISPOT	IFNg release	Positive	1	1	1	1	1	1	1	1	1	1	1	1	1	1	1	1	1	1	1	1	0	1	1	0	1	0
869089	TAIANQATVLMGLGK	NS4b	2309	2323	HLA-DQA1*01:02/DQB1*06:02	100%	Dengue virus 2	ELISPOT	IFNg release	Positive	1	1	1	1	1	1	1	1	1	1	1	1	1	1	1	1	1	1	1	1	0	1	1	0	1	0
150597	SRAIWYMWLGARFLE	NS5	2962	2976	HLA-DRB1*01:01	100%	Dengue virus 2	ELISPOT	IFNg release	Positive	1	1	1	1	1	1	1	1	1	1	1	1	1	1	1	1	1	1	1	1	0	1	1	0	1	0
180582	KTWAYHGSYETKQTG	NS5	2792	2806	HLA class II	100%	Dengue virus 2	ICS	IFNg release	Positive	1	1	1	1	1	1	1	1	1	1	1	1	1	1	1	1	1	1	1	1	0	1	1	0	1	0
190862	MVTQMAMTDTTPFGQQR	NS5	2828	2844	HLA class II	100%	Dengue virus 2	ICS	IFNg release	Positive	1	1	1	1	1	1	1	1	1	1	1	1	1	1	1	1	1	1	1	1	0	1	1	0	1	0
195200	CETCVYNMMGKREKK	NS5	2924	2938	HLA-DRB1*11:01	100%	Dengue virus 2	ELISPOT	IFNg release	Positive	1	1	1	1	1	1	1	1	1	1	1	1	1	1	1	1	1	1	1	1	0	1	1	0	1	0
195213	DELIGRARISQGAGW	NS5	3223	3237	HLA-DRB1*08:02	100%	Dengue virus 2	ELISPOT	IFNg release	Positive	1	1	1	1	1	1	1	1	1	1	1	1	1	1	1	1	1	1	1	1	0	1	1	0	1	0
195409	GNIVSSVNMISRMLI	NS5	2717	2731	HLA-DRB1*07:01	100%	Dengue virus 2	ELISPOT	IFNg release	Positive	1	1	1	1	1	1	1	1	1	1	1	1	1	1	1	1	1	1	1	1	0	1	1	0	1	0
195428	GTVMDIISRRDQRGS	NS5	3078	3092	HLA-DRB1*03:01	100%	Dengue virus 2	ELISPOT	IFNg release	Positive	1	1	1	1	1	1	1	1	1	1	1	1	1	1	1	1	1	1	1	1	0	1	1	0	1	0
196187	VSSVNMISRMLINRF	NS5	2720	2734	HLA-DRB1*15:01	100%	Dengue virus 2	ELISPOT	IFNg release	Positive	1	1	1	1	1	1	1	1	1	1	1	1	1	1	1	1	1	1	1	1	0	1	1	0	1	0
196210	WAKNIQTAINQVRSL	NS5	3336	3350	HLA-DRB1*03:01	100%	Dengue virus 2	ELISPOT	IFNg release	Positive	1	1	1	1	1	1	1	1	1	1	1	1	1	1	1	1	1	1	1	1	0	0	1	0	0	0
196243	YQNKVVRVQRPTPRG	NS5	3064	3078	HLA-DRB1*08:02	100%	Dengue virus 2	ELISPOT	IFNg release	Positive	1	1	1	1	1	1	1	1	1	1	1	1	1	1	1	1	1	1	1	1	0	1	1	0	1	0
32943	KPWDVVPMV	NS5	2821	2829	HLA class II	100%	Dengue virus 2	ELISA	IL-2 release	Positive	1	1	1	1	1	1	1	1	1	1	1	1	1	1	1	1	1	1	1	1	0	1	1	0	1	0
539150	DDRFASALTALNDMG	NS5	3162	3176	HLA-DRB1*04:03	100%	Dengue virus 2	ELISPOT	IFNg release	Positive	1	1	1	1	1	1	1	1	1	1	1	1	1	1	1	1	1	1	1	1	0	1	1	0	1	0
539195	EAIFKLTYQNKVVRV	NS5	3057	3071	HLA-DRB1*09:01	100%	Dengue virus 2	ELISPOT	IFNg release	Positive	1	1	1	1	1	1	1	1	1	1	1	1	1	1	1	1	1	1	1	1	0	1	1	0	1	0
539348	GSAKLRWFVERNMVT	NS5	2549	2563	HLA-DRB1*09:01	100%	Dengue virus 2	ELISPOT	IFNg release	Positive	1	1	1	1	1	1	1	1	1	1	1	1	1	1	1	1	1	1	1	1	0	1	1	0	1	0
539599	LMYFHRRDLRLAANA	NS5	3255	3269	HLA-DRB1*13:01	100%	Dengue virus 2	ELISPOT	IFNg release	Positive	1	1	1	1	1	1	1	1	1	1	1	1	1	1	1	1	1	1	1	1	0	1	1	0	1	0
539603	LNTFTNMEAQLIRQM	NS5	3100	3114	HLA-DRB1*10:01	100%	Dengue virus 2	ELISPOT	IFNg release	Positive	1	1	1	1	1	1	1	1	1	1	1	1	1	1	1	1	1	1	1	1	0	1	1	0	1	0
539871	RRDLRLAANAICSAV	NS5	3260	3274	HLA-DRB3*02:02	100%	Dengue virus 2	ELISPOT	IFNg release	Positive	1	1	1	1	1	1	1	1	1	1	1	1	1	1	1	1	1	1	1	1	0	1	1	0	1	0
540065	VNGVVRLLTKPWDVV	NS5	2812	2826	HLA-DRB1*12:02	100%	Dengue virus 2	ELISPOT	IFNg release	Positive	1	1	1	1	1	1	1	1	1	1	1	1	1	1	1	1	1	1	1	1	0	1	1	0	1	0
739166	IFKLTYQNKVVRVQR	NS5	3045	3059	HLA-DRB1*14:06	100%	Dengue virus 2	ELISPOT	IFNg release	Positive	1	1	1	1	1	1	1	1	1	1	1	1	1	1	1	1	1	1	1	1	0	1	1	0	1	0
740675	VRSNAALGAIFTDEN	NS5	2880	2894	HLA-DRB1*11:04	100%	Dengue virus 2	ELISPOT	IFNg release	Positive	1	1	1	1	1	1	1	1	1	1	1	1	1	1	1	1	1	1	1	1	0	1	1	0	1	0
866015	AIWYMWLGARFLEFE	NS5	2964	2978	HLA-DPB1*01:01	100%	Dengue virus 2	ELISPOT	IFNg release	Positive	1	1	1	1	1	1	1	1	1	1	1	1	1	1	1	1	1	1	1	1	0	1	1	0	1	0
866566	EHKKLAEAIFKLTYQ	NS5	3051	3065	HLA-DPB1*01:01	100%	Dengue virus 2	ELISPOT	IFNg release	Positive	1	1	1	1	1	1	1	1	1	1	1	1	1	1	1	1	1	1	1	1	0	1	1	0	1	0
866648	ETCVYNMMGKREKKL	NS5	2939	2953	HLA-DRB5*01:01	100%	Dengue virus 2	ELISPOT	IFNg release	Positive	1	1	1	1	1	1	1	1	1	1	1	1	1	1	1	1	1	1	1	1	0	1	1	0	1	0
867419	KKLAEAIFKLTYQNK	NS5	3053	3067	HLA-DPB1*02:01	100%	Dengue virus 2	ELISPOT	IFNg release	Positive	1	1	1	1	1	1	1	1	1	1	1	1	1	1	1	1	1	1	1	1	0	1	1	0	1	0
867433	KLAEAIFKLTYQNKV	NS5	3054	3068	HLA-DPB1*02:01	100%	Dengue virus 2	ELISPOT	IFNg release	Positive	1	1	1	1	1	1	1	1	1	1	1	1	1	1	1	1	1	1	1	1	0	1	1	0	1	0
867600	LEFEALGFLNEDHWF	NS5	2975	2989	HLA-DPB1*04:01	100%	Dengue virus 2	ELISPOT	IFNg release	Positive	1	1	1	1	1	1	1	1	1	1	1	1	1	1	1	1	1	1	1	1	0	1	1	0	1	0
868637	RDLRLAANAICSAVP	NS5	3261	3275	HLA-DQA1*05:01/DQB1*02:01	100%	Dengue virus 2	ELISPOT	IFNg release	Positive	1	1	1	1	1	1	1	1	1	1	1	1	1	1	1	1	1	1	1	1	0	1	1	0	1	0
868640	RFLEFEALGFLNEDH	NS5	2973	2987	HLA-DPB1*01:01	100%	Dengue virus 2	ELISPOT	IFNg release	Positive	1	1	1	1	1	1	1	1	1	1	1	1	1	1	1	1	1	1	1	1	0	1	1	0	1	0
868668	RKVRSNAALGAIFTD	NS5	2892	2906	HLA-DRB3*02:02	100%	Dengue virus 2	ELISPOT	IFNg release	Positive	1	1	1	1	1	1	1	1	1	1	1	1	1	1	1	1	1	1	1	1	0	1	1	0	1	0
869307	TRKVRSNAALGAIFT	NS5	2891	2905	HLA-DRB3*02:02	100%	Dengue virus 2	ELISPOT	IFNg release	Positive	1	1	1	1	1	1	1	1	1	1	1	1	1	1	1	1	1	1	1	1	0	1	1	0	1	0
195417	GRMLNILNRRRRTAG	C	89	103	HLA-DRB1*11:01	81% - <100%	Dengue virus 2	ELISPOT	IFNg release	Positive	1	1	1	1	1	1	1	1	1	1	1	1	0	0	1	1	1	1	1	1	0	0	1	0	0	0
196093	VAFLRFLTIPPTAGI	C	51	65	HLA-DRB1*04:01	81% - <100%	Dengue virus 2	ELISPOT	IFNg release	Positive	1	1	1	1	1	1	1	1	1	1	0	1	1	1	1	1	1	1	1	1	0	1	1	0	1	0
740399	STPFNMLKRERNRVS	C	10	24	HLA-DRB1*14:02	81% - <100%	Dengue virus 2	ELISPOT	IFNg release	Positive	1	1	1	1	1	1	1	1	1	1	1	0	1	1	1	1	0	0	1	1	0	0	0	0	0	0
150490	PGFTIMAAILAYTIG	PreM	245	259	HLA-DRB1*01:01	81% - <100%	Dengue virus 2	ELISPOT	IFNg release	Positive	1	1	1	1	1	1	1	1	1	1	0	1	1	1	1	1	1	1	1	1	1	1	1	1	0	1

866485	EDGVNMCTLMAIDLG	PreM	142	156	HLA-DQA1*01:02/DQB1*06:02	81% - <100%	Dengue virus 2	ELISPOT	IFNg release	Positive	1	1	1	1	1	1	1	1	1	1	1	0	1	1	1	1	0	0	1	1	0	0	0	0	0	0
144940	RHVLGRLITVNPIVT	E	345	359	HLA-DRB1*08:02	81% - <100%	Dengue virus 2	ELISPOT	IFNg release	Positive	1	1	1	1	1	1	1	1	1	1	0	1	1	1	1	1	1	1	1	1	1	1	1	1	1	1
195407	GMNSRSTSLSVSLVL	E	467	481	HLA-DRB1*07:01	81% - <100%	Dengue virus 2	ELISPOT	IFNg release	Positive	1	1	1	1	0	1	1	1	1	1	1	1	1	1	1	1	1	1	1	1	1	1	1	1	0	0
195638	LGRLITVNPIVTEKD	E	628	642	HLA-DRB1*08:02	81% - <100%	Dengue virus 2	ELISPOT	IFNg release	Positive	1	0	1	1	1	1	1	1	1	1	0	1	1	1	1	1	1	1	1	1	1	1	1	1	1	1
196140	VLGRLITVNPIVTEK	E	627	641	HLA-DRB1*08:02	81% - <100%	Dengue virus 2	ELISPOT	IFNg release	Positive	1	0	1	1	1	1	1	1	1	1	0	1	1	1	1	1	1	1	1	1	1	1	1	1	1	1
539252	FETTMRGAKRMAILG	E	402	416	HLA-DRB1*15:02	81% - <100%	Dengue virus 2	ELISPOT	IFNg release	Positive	1	1	1	1	1	1	1	1	1	1	1	1	1	1	1	1	0	0	1	1	0	1	0	1	1	0
868713	RTGLDFNEMVLLQME	E	178	192	HLA-DQA1*01:02/DQB1*06:02	81% - <100%	Dengue virus 2	ELISPOT	IFNg release	Positive	1	1	1	1	1	1	1	1	1	1	1	1	1	1	1	1	0	0	1	1	1	1	1	1	1	1
869123	TEKDSPVNIEAEPPF	E	349	363	HLA-DQA1*03:01/DQB1*03:02	81% - <100%	Dengue virus 2	ELISPOT	IFNg release	Positive	1	0	1	1	1	1	1	1	1	1	0	1	1	1	1	1	1	1	1	1	1	1	1	1	1	1
869152	TGLDFNEMVLLQMED	E	189	203	HLA-DQA1*03:01/DQB1*03:02	81% - <100%	Dengue virus 2	ELISPOT	IFNg release	Positive	1	1	1	1	1	1	1	1	1	1	1	0	1	1	1	1	0	0	1	1	0	0	0	0	0	0
167759	IMTGDIKGIMQA	NS1	88	99	HLA-DQ6	81% - <100%	Dengue virus 2	ELISPOT	IFNg release	Positive	1	1	1	1	1	1	1	1	1	1	1	1	1	1	1	1	1	1	1	0	0	1	1	0	1	0
195458	HNQTFLIDGPETAEC	NS1	904	918	HLA-DRB1*04:01	81% - <100%	Dengue virus 2	ELISPOT	IFNg release	Positive	1	1	1	1	1	1	1	1	0	1	1	1	1	1	1	1	1	1	1	0	0	1	1	0	1	0
195576	KLTIMTGDIKGIMQA	NS1	85	99	HLA-DRB1*03:01	81% - <100%	Dengue virus 2	ELISPOT	IFNg release	Positive	1	1	1	1	1	1	1	1	1	1	1	1	1	1	1	1	1	1	1	0	0	1	1	0	1	0
195583	KQDVFCDSKLMSAAI	NS1	949	963	HLA-DRB1*03:01	81% - <100%	Dengue virus 2	ELISPOT	IFNg release	Positive	1	1	1	1	1	1	1	1	1	1	1	0	1	1	1	1	1	1	1	1	0	0	1	0	1	0
195880	REKQDVFCDSKLMSA	NS1	947	961	HLA-DRB1*03:01	81% - <100%	Dengue virus 2	ELISPOT	IFNg release	Positive	1	1	1	1	1	1	0	1	1	1	1	0	1	1	1	1	1	1	1	1	0	0	1	0	0	0
195954	SHNQTFLIDGPETAE	NS1	903	917	HLA-DRB1*04:01	81% - <100%	Dengue virus 2	ELISPOT	IFNg release	Positive	1	1	1	1	1	1	1	1	0	0	1	1	1	1	1	1	1	1	1	0	0	1	1	0	1	0
196133	VKLTIMTGDIKGIMQ	NS1	84	98	HLA-DRB1*03:01	81% - <100%	Dengue virus 2	ELISPOT	IFNg release	Positive	1	1	1	1	1	1	1	1	1	1	1	1	1	1	1	1	1	1	1	0	0	1	1	0	1	0
738586	CGIRSVTRLENLMWK	NS1	55	69	HLA-DRB1*11:04	81% - <100%	Dengue virus 2	ELISPOT	IFNg release	Positive	1	1	1	1	1	1	1	1	1	1	1	1	1	1	1	1	0	0	1	1	0	1	1	0	1	0
866699	FGVFTTNIWLKLREK	NS1	935	949	HLA-DPB1*04:01	81% - <100%	Dengue virus 2	ELISPOT	IFNg release	Positive	1	1	1	1	1	1	0	1	1	1	1	0	1	1	1	1	1	1	1	1	0	0	1	0	0	0
866831	GFGVFTTNIWLKLRE	NS1	934	948	HLA-DPB1*04:01	81% - <100%	Dengue virus 2	ELISPOT	IFNg release	Positive	1	1	1	1	1	1	0	1	1	1	1	1	1	1	1	1	1	1	1	1	0	0	1	0	0	0
867040	HADMGYWIESALNDT	NS1	970	984	HLA-DQA1*01:01/DQB1*05:01	81% - <100%	Dengue virus 2	ELISPOT	IFNg release	Positive	1	1	1	1	0	1	1	1	1	1	1	1	1	1	1	1	1	1	1	1	0	1	1	0	1	0
869722	YGFGVFTTNIWLKLR	NS1	933	947	HLA-DPB1*04:01	81% - <100%	Dengue virus 2	ELISPOT	IFNg release	Positive	1	1	1	1	1	1	0	1	1	1	1	1	1	1	1	1	1	1	1	1	0	0	1	0	0	0
195688	LNPTAIFLTTLSRTS	NS2a	1328	1342	HLA-DRB1*07:01	81% - <100%	Dengue virus 2	ELISPOT	IFNg release	Positive	1	1	1	1	1	1	1	1	1	1	1	0	1	1	1	1	0	0	1	1	0	1	0	0	1	0
196217	WKVSCTILAAVSVSP	NS2a	1292	1306	HLA-DRB1*04:01	81% - <100%	Dengue virus 2	ELISPOT	IFNg release	Positive	1	1	1	1	1	1	1	1	1	1	1	0	1	1	1	1	0	0	1	1	0	0	0	0	0	0
539706	NAWKVSCTILAAVSV	NS2a	1290	1304	HLA-DRB1*09:01	81% - <100%	Dengue virus 2	ELISPOT	IFNg release	Positive	1	1	1	1	1	1	1	1	1	1	1	0	1	1	1	1	0	0	1	1	0	0	0	0	0	0
539789	PTAIFLTTLSRTSKK	NS2a	1316	1330	HLA-DRB1*04:03	81% - <100%	Dengue virus 2	ELISPOT	IFNg release	Positive	1	1	1	1	1	1	1	1	1	1	1	0	1	1	1	1	0	0	1	1	0	1	0	0	1	0
539893	SCTILAAVSVSPLLL	NS2a	1295	1309	HLA-DRB1*10:01	81% - <100%	Dengue virus 2	ELISPOT	IFNg release	Positive	1	1	1	1	1	1	1	1	1	1	1	0	1	1	1	1	0	0	1	1	0	0	0	0	0	0
539959	TAIFLTTLSRTSKKR	NS2a	1317	1331	HLA-DRB1*14:02	81% - <100%	Dengue virus 2	ELISPOT	IFNg release	Positive	1	1	1	1	1	1	1	1	1	1	1	0	1	1	1	1	0	0	1	1	0	1	0	0	1	0
150433	MAVGMVSILASSLLK	NS2b	1354	1368	HLA-DRB1*01:01	81% - <100%	Dengue virus 2	ELISPOT	IFNg release	Positive	1	1	1	1	1	1	1	1	1	1	1	1	1	1	1	1	0	0	1	1	0	1	1	0	1	0
195239	EEQTLTILIRTGLLV	NS2b	91	105	HLA-DRB1*15:01	81% - <100%	Dengue virus 2	ELISPOT	IFNg release	Positive	1	1	1	1	1	1	1	1	1	1	1	0	1	1	1	1	0	0	1	1	0	1	1	0	1	0
195869	QTLTILIRTGLLVIS	NS2b	1438	1452	HLA-DRB1*15:01	81% - <100%	Dengue virus 2	ELISPOT	IFNg release	Positive	1	1	1	1	1	1	1	1	1	1	1	0	1	1	1	1	0	0	1	1	0	1	1	0	1	0
195913	RTGLLVISGVFPVSI	NS2b	1445	1459	HLA-DRB1*08:02	81% - <100%	Dengue virus 2	ELISPOT	IFNg release	Positive	1	1	1	1	1	1	1	1	1	1	1	0	1	1	1	1	0	0	1	1	0	0	0	0	0	0
539574	LIRTGLLVISGVFPV	NS2b	1443	1457	HLA-DRB1*13:01	81% - <100%	Dengue virus 2	ELISPOT	IFNg release	Positive	1	1	1	1	1	1	1	1	1	1	1	0	1	1	1	1	0	0	1	1	0	0	0	0	0	0
866986	GMVSILASSLLKNDI	NS2b	1357	1371	HLA-DPB1*01:01	81% - <100%	Dengue virus 2	ELISPOT	IFNg release	Positive	1	1	1	1	1	1	1	1	1	1	1	1	1	1	1	1	0	0	1	1	0	1	1	0	1	0
539200	EDDIFRKKRLTIMDL	NS3	1654	1668	HLA-DRB1*13:01	81% - <100%	Dengue virus 2	ELISPOT	IFNg release	Positive	1	1	1	1	1	1	1	1	1	1	1	0	1	1	1	1	0	0	1	1	0	0	0	0	0	0
539203	EERVILAGPMPVTHS	NS3	1899	1913	HLA-DRB1*09:01	81% - <100%	Dengue virus 2	ELISPOT	IFNg release	Positive	1	1	1	1	1	1	1	1	1	1	1	0	1	1	1	1	1	1	1	1	0	1	1	0	1	0
539283	GAYRIKQRGILGYSQ	NS3	1496	1510	HLA-DRB1*08:03	81% - <100%	Dengue virus 2	ELISPOT	IFNg release	Positive	1	1	1	1	1	1	1	1	1	1	1	0	1	1	1	1	0	0	1	1	0	0	0	0	0	0
868183	MWHVTRGAVLMHRGK	NS3	1524	1538	HLA-DRB3*02:02	81% - <100%	Dengue virus 2	ELISPOT	IFNg release	Positive	1	1	1	1	1	1	1	1	1	1	1	0	1	1	1	1	0	0	1	1	0	0	0	0	0	0
195414	GRAYNHALSELPETL	NS4a	2131	2145	HLA-DRB1*04:01	81% - <100%	Dengue virus 2	ELISPOT	IFNg release	Positive	1	0	1	1	1	1	1	1	1	1	1	1	1	1	1	1	1	1	1	1	0	1	1	0	1	0
196089	TVTGGIFLFLMSGKG	NS4a	2142	2156	HLA-DRB1*11:01	81% - <100%	Dengue virus 2	ELISPOT	IFNg release	Positive	1	1	1	1	1	1	1	1	1	1	1	0	1	1	1	1	0	0	1	1	0	0	0	0	0	0
539609	LPTFMTQKARNALDN	NS4a	2092	2106	HLA-DRB1*10:01	81% - <100%	Dengue virus 2	ELISPOT	IFNg release	Positive	1	1	1	1	1	1	1	1	1	1	1	0	1	1	1	1	0	0	1	1	0	0	0	0	0	0
539801	QDNQLTYVVIAILTV	NS4a	2223	2237	HLA-DRB1*15:02	81% - <100%	Dengue virus 2	ELISPOT	IFNg release	Positive	1	1	1	1	1	1	1	1	1	1	0	1	1	1	1	1	1	1	1	1	0	1	1	0	1	0
150611	TAALLLLVAHYAIIG	NS4b	2351	2365	HLA-DRB1*01:01	81% - <100%	Dengue virus 2	ELISPOT	IFNg release	Positive	1	0	1	1	1	1	1	1	1	1	1	0	1	1	1	1	1	0	1	1	0	1	0	0	0	0
150636	TLTAALLLLVAHYAI	NS4b	2335	2349	HLA-DRB1*01:01	81% - <100%	Dengue virus 2	ELISPOT	IFNg release	Positive	1	1	1	1	1	1	1	1	1	1	1	0	1	1	1	1	1	0	1	1	0	1	0	0	0	0
195882	RFWNTTIAVSMANIF	NS4b	2454	2468	HLA-DRB1*07:01	81% - <100%	Dengue virus 2	ELISPOT	IFNg release	Positive	1	1	1	1	1	1	1	1	1	1	1	1	1	1	1	1	1	0	1	1	0	1	1	0	1	0
195980	SQVNPITLTAALLLL	NS4b	2343	2357	HLA-DQA1*01:02/DQB1*06:02	81% - <100%	Dengue virus 2	ELISPOT	IFNg release	Positive	1	1	1	1	1	1	1	1	1	1	1	0	1	1	1	1	1	1	1	1	0	1	0	0	0	0
539755	PASAWTLYAVATTFV	NS4b	2277	2291	HLA-DRB1*09:01	81% - <100%	Dengue virus 2	ELISPOT	IFNg release	Positive	1	1	1	1	1	1	0	1	1	1	1	1	1	1	1	1	1	1	1	1	0	1	1	0	1	0
866802	FWNTTIAVSMANIFR	NS4b	2455	2469	HLA-DQA1*01:02/DQB1*06:02	81% - <100%	Dengue virus 2	ELISPOT	IFNg release	Positive	1	1	1	1	1	1	1	1	1	1	1	1	1	1	1	1	1	0	1	1	0	1	1	0	1	0
867709	LILCVTQVLMMRTTW	NS4b	2417	2431	HLA-DPB1*04:02	81% - <100%	Dengue virus 2	ELISPOT	IFNg release	Positive	1	1	1	1	1	1	1	1	1	1	1	0	1	1	1	1	0	0	1	1	0	0	0	0	0	0
195111	ACLGKSYAQMWTLMY	NS5	3243	3257	HLA-DRB1*04:01	81% - <100%	Dengue virus 2	ELISPOT	IFNg release	Positive	1	1	1	1	1	1	1	1	1	1	1	0	1	1	1	1	0	0	1	1	0	0	0	0	0	0
195446	HFHELVMKDGRVLVV	NS5	3203	3217	HLA-DRB1*13:01	81% - <100%	Dengue virus 2	ELISPOT	IFNg release	Positive	1	1	1	1	1	1	1	1	1	1	1	0	1	1	1	1	0	0	1	1	0	0	0	0	0	0
195759	MLINRFTMKHKKATY	NS5	2729	2743	HLA-DRB1*11:01	81% - <100%	Dengue virus 2	ELISPOT	IFNg release	Positive	1	1	1	1	1	1	1	1	1	1	1	0	1	1	1	1	0	0	1	1	0	0	0	0	0	0
195800	NMISRMLINRFTMKH	NS5	2724	2738	HLA-DRB1*08:02	81% - <100%	Dengue virus 2	ELISPOT	IFNg release	Positive	1	1	1	1	1	1	1	1	1	1	1	0	1	1	1	1	0	0	1	1	0	0	0	0	0	0
196040	TGNIVSSVNMISRML	NS5	2716	2730	HLA-DRB1*04:01	81% - <100%	Dengue virus 2	ELISPOT	IFNg release	Positive	1	1	1	1	1	1	1	1	1	1	1	0	1	1	1	1	0	0	1	1	0	0	0	0	0	0
196225	WTLMYFHRRDLRLAA	NS5	3253	3267	HLA-DRB1*03:01	81% - <100%	Dengue virus 2	ELISPOT	IFNg release	Positive	1	1	1	1	1	1	1	1	1	1	1	0	1	1	1	1	0	0	1	1	0	0	0	0	0	0
539161	DHWFSRGNSLSGVEG	NS5	2986	3000	HLA-DRB1*09:01	81% - <100%	Dengue virus 2	ELISPOT	IFNg release	Positive	1	1	1	1	1	1	1	1	1	1	1	0	1	1	1	1	0	0	1	1	0	0	0	0	0	0
539668	MISRMLINRFTMKHK	NS5	2725	2739	HLA-DRB1*14:04	81% - <100%	Dengue virus 2	ELISPOT	IFNg release	Positive	1	1	1	1	1	1	1	1	1	1	1	0	1	1	1	1	0	0	1	1	0	0	0	0	0	0
539932	SRMLINRFTMKHKKA	NS5	2727	2741	HLA-DRB1*08:03	81% - <100%	Dengue virus 2	ELISPOT	IFNg release	Positive	1	1	1	1	1	1	1	1	1	1	1	0	1	1	1	1	0	0	1	1	0	0	0	0	0	0
540066	VNMISRMLINRFTMK	NS5	2723	2737	HLA-DRB1*12:02	81% - <100%	Dengue virus 2	ELISPOT	IFNg release	Positive	1	1	1	1	1	1	1	1	1	1	1	0	1	1	1	1	0	0	1	1	0	0	0	0	0	0
738754	EEFTRKVRSNAALGA	NS5	2874	2888	HLA-DRB1*11:04	81% - <100%	Dengue virus 2	ELISPOT	IFNg release	Positive	1	1	1	1	1	1	1	1	0	1	1	1	1	1	1	1	1	1	1	1	0	1	1	0	1	0
869083	SYAQMWTLMYFHRRD	NS5	3248	3262	HLA-DPB1*04:01	81% - <100%	Dengue virus 2	ELISPOT	IFNg release	Positive	1	1	1	1	1	1	1	1	1	1	1	0	1	1	1	1	0	0	1	1	0	0	0	0	0	0
869292	TPVESWEEVPYLGKR	NS5	3319	3333	HLA-DQA1*01:01/DQB1*05:01	81% - <100%	Dengue virus 2	ELISPOT	IFNg release	Positive	1	1	1	1	1	1	1	1	1	1	1	0	1	1	1	1	0	0	1	1	0	0	0	0	0	0
150369	LKLFMALVAFLRFLT	C	44	58	HLA-DRB1*01:01	51%-80%	Dengue virus 2	ELISPOT	IFNg release	Positive	0	0	0	0	0	1	1	0	1	1	0	1	1	0	0	1	1	1	1	1	0	1	1	0	1	0
180597	LFMALVAFLRFLTIP	C	46	60	HLA class II	51%-80%	Dengue virus 2	ELISPOT	IFNg release	Positive	0	0	0	0	0	1	1	0	1	1	0	1	1	0	0	1	1	1	1	1	0	1	1	0	1	0
[truncated: 161,471 more chars]
